# Supplementary material for: pyCancerSig: subclassifying human cancer with comprehensive single nucleotide, structural and microsatellite mutational signature deconstruction from whole genome sequencing
Source: BMC Bioinformatics. 2020 Apr 3;21:128. doi: 10.1186/s12859-020-3451-8 (PMC7118897; doi:10.1186/s12859-020-3451-8)
Supplement: Supplementary file 3 — Additional file 3. [file 12859_2020_3451_MOESM3_ESM.pdf]

Cancer processes Weights for TCGA-E2-A14X

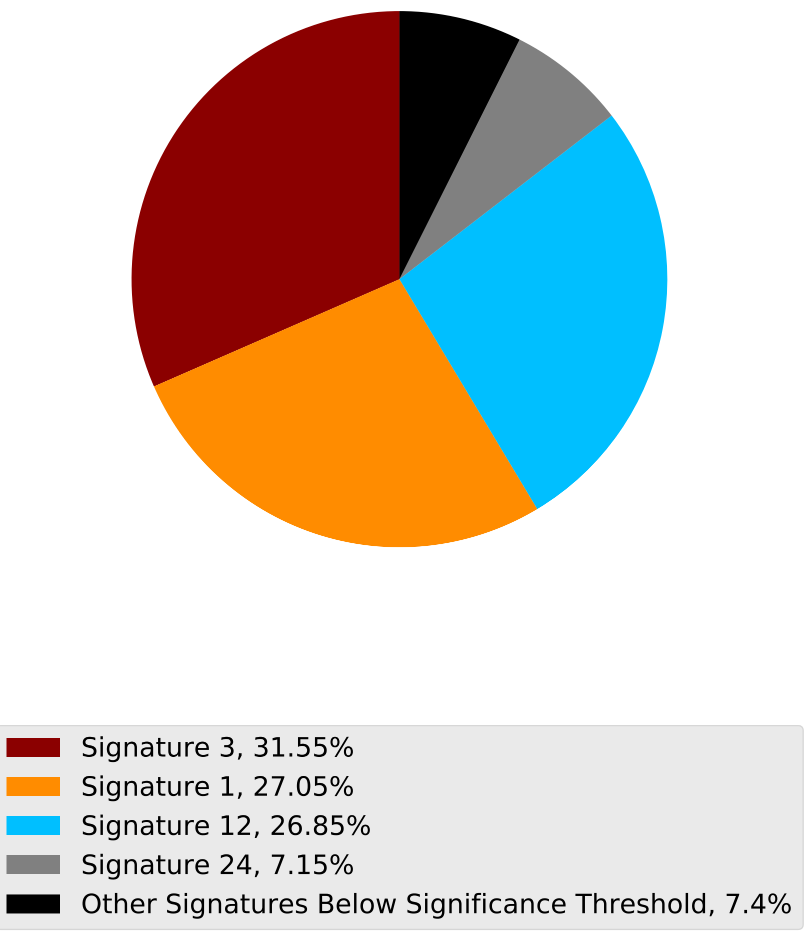

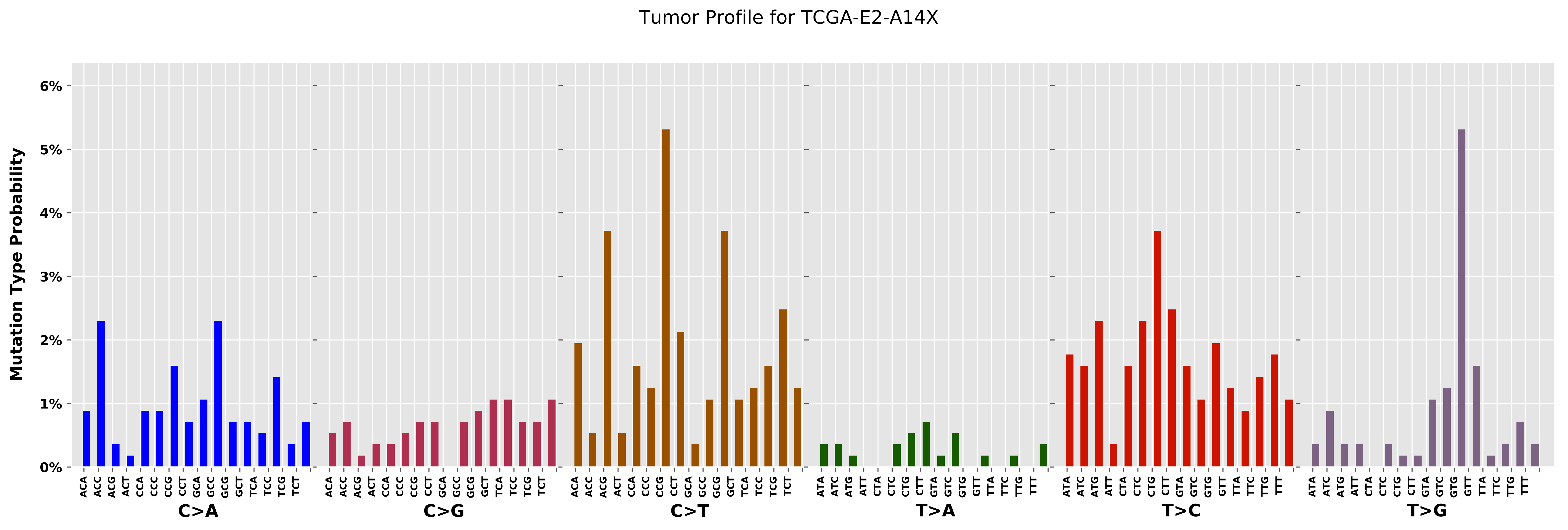

Tumor Profile for TCGA-E2-A14X
Mutation Type Probability
0%
1%
2%
3%
4%
5%
6%
C>A
C>G
C>T
T>A
T>C
T>G

Cancer processes Weights for TCGA-A8-A08L

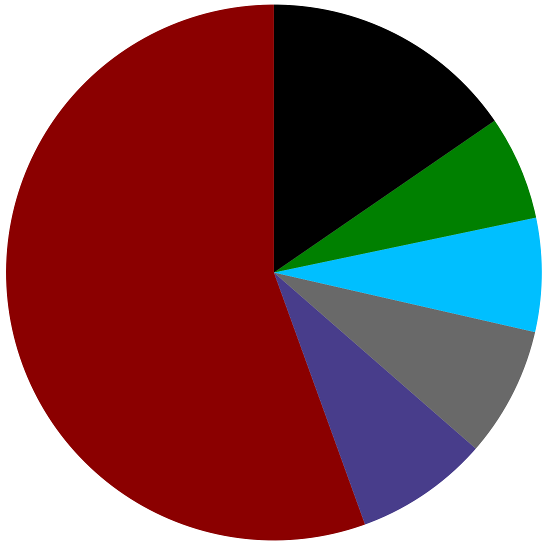

- Signature 3, 55.52%
- Signature 6, 8.06%
- Signature 13, 7.84%
- Signature 12, 6.86%
- Signature 25, 6.3%
- Other Signatures Below Significance Threshold, 15.41%

Tumor Profile for TCGA-A8-A08L

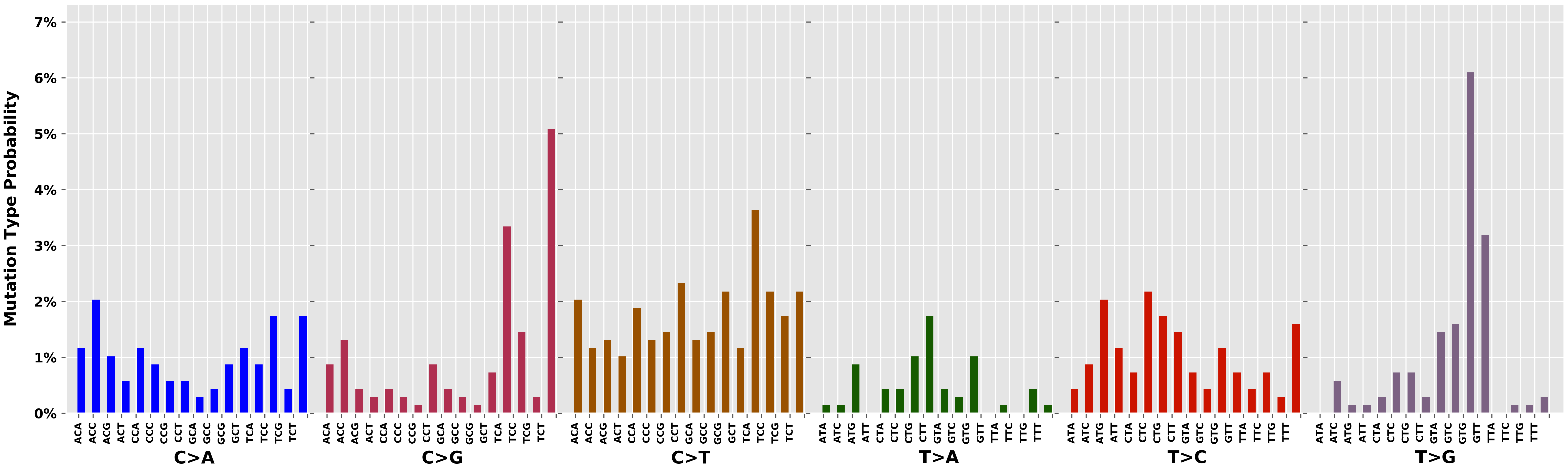

Cancer processes Weights for TCGA-AO-A12H

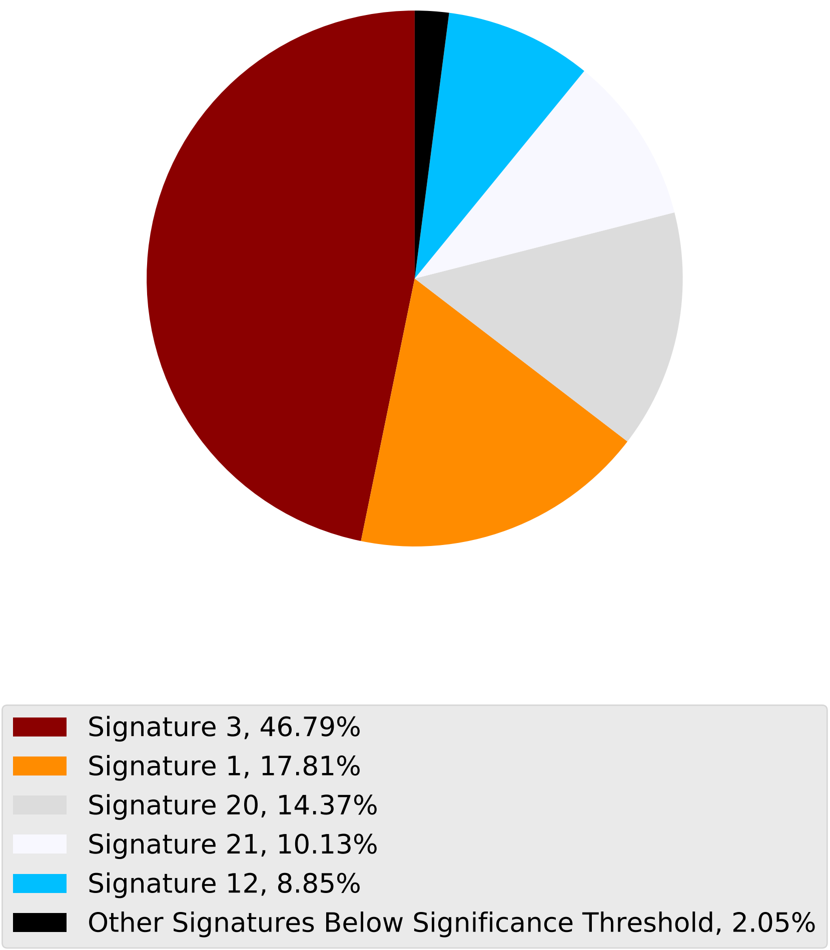

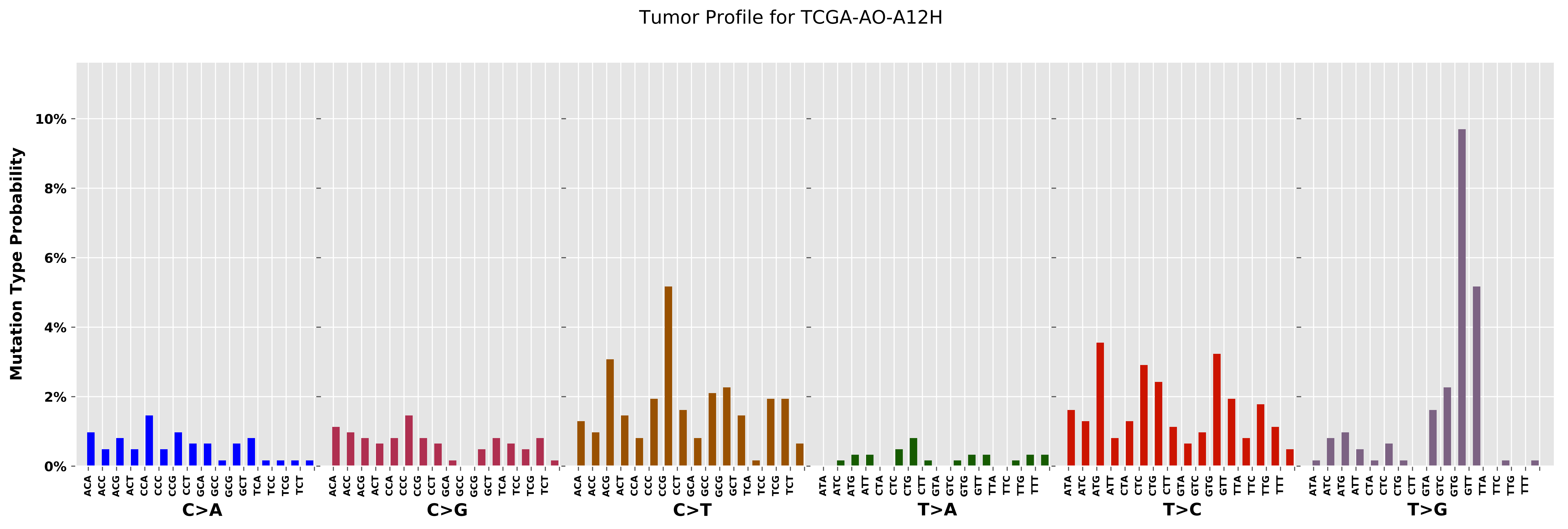

Tumor Profile for TCGA-AO-A12H
Mutation Type Probability
0%
2%
4%
6%
8%
10%
C>A
C>G
C>T
T>A
T>C
T>G

Cancer processes Weights for TCGA-EW-A3U0

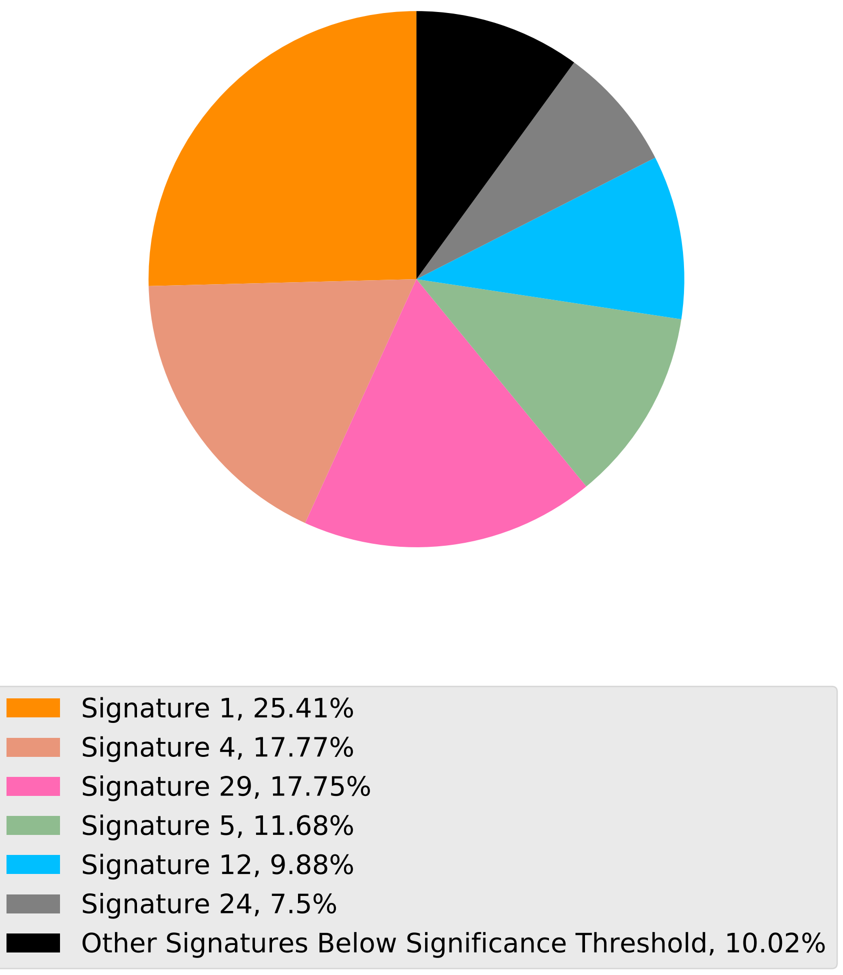

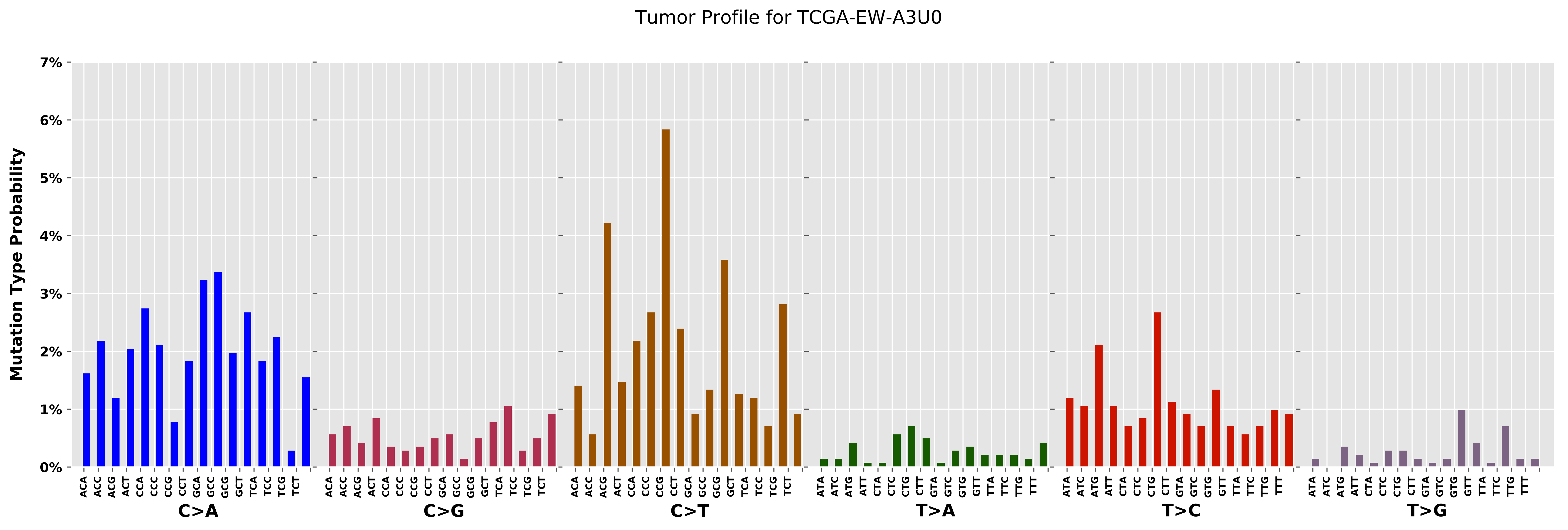
Tumor Profile for TCGA-EW-A3U0
Mutation Type Probability
0%
1%
2%
3%
4%
5%
6%
7%
C>A
C>G
C>T
T>A
T>C
T>G

Cancer processes Weights for TCGA-BH-A1FC

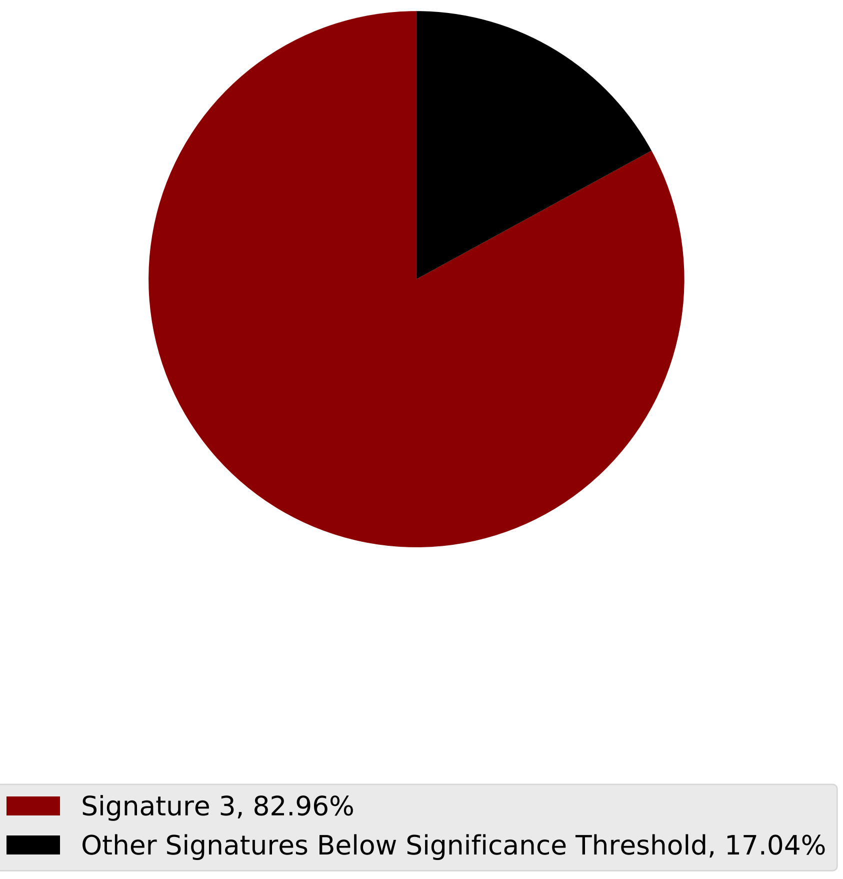

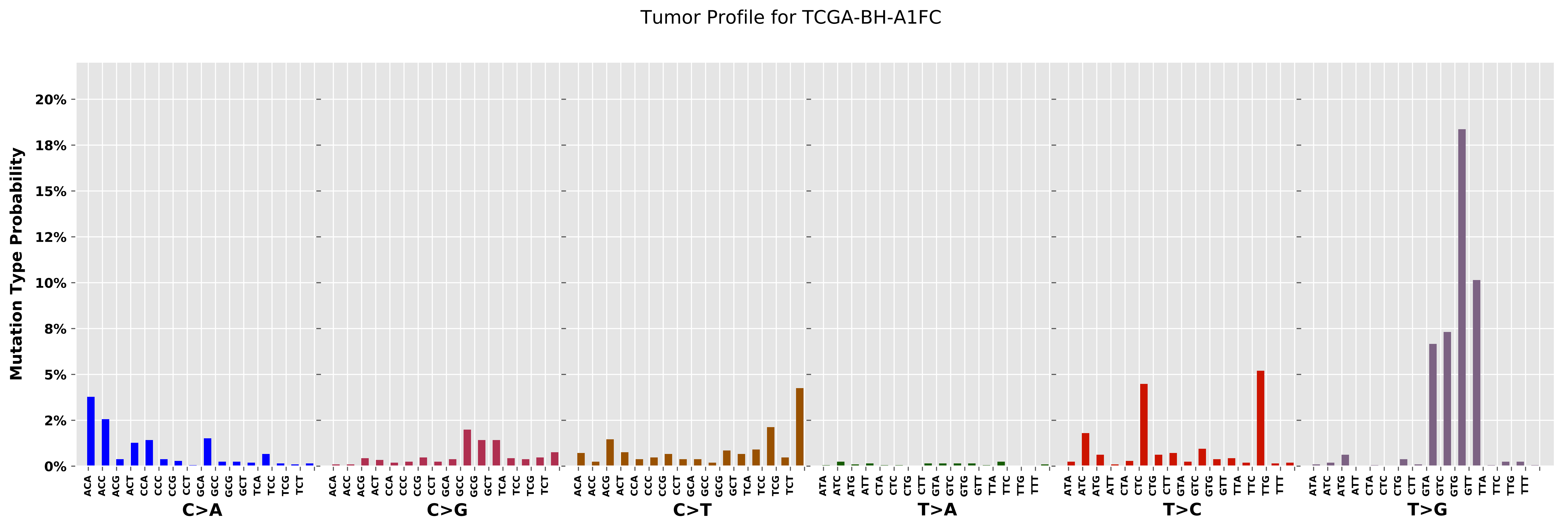

Tumor Profile for TCGA-BH-A1FC
Mutation Type Probability
0%
2%
5%
8%
10%
12%
15%
18%
20%
C>A
C>G
C>T
T>A
T>C
T>G

Cancer processes Weights for TCGA-AF-2691

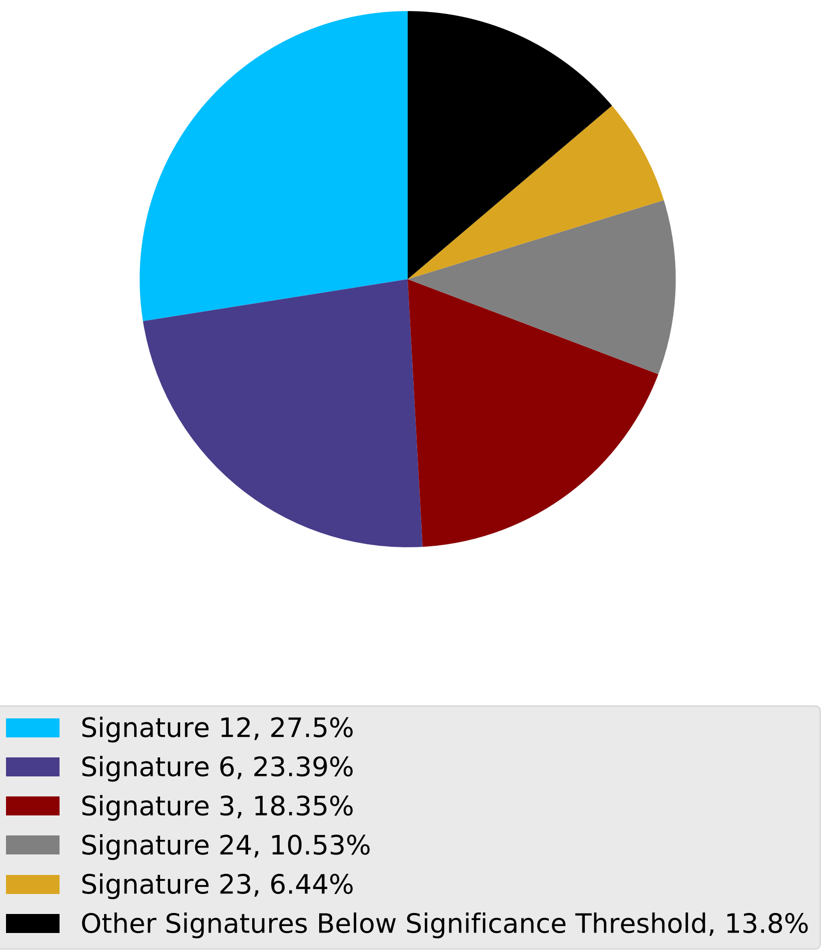

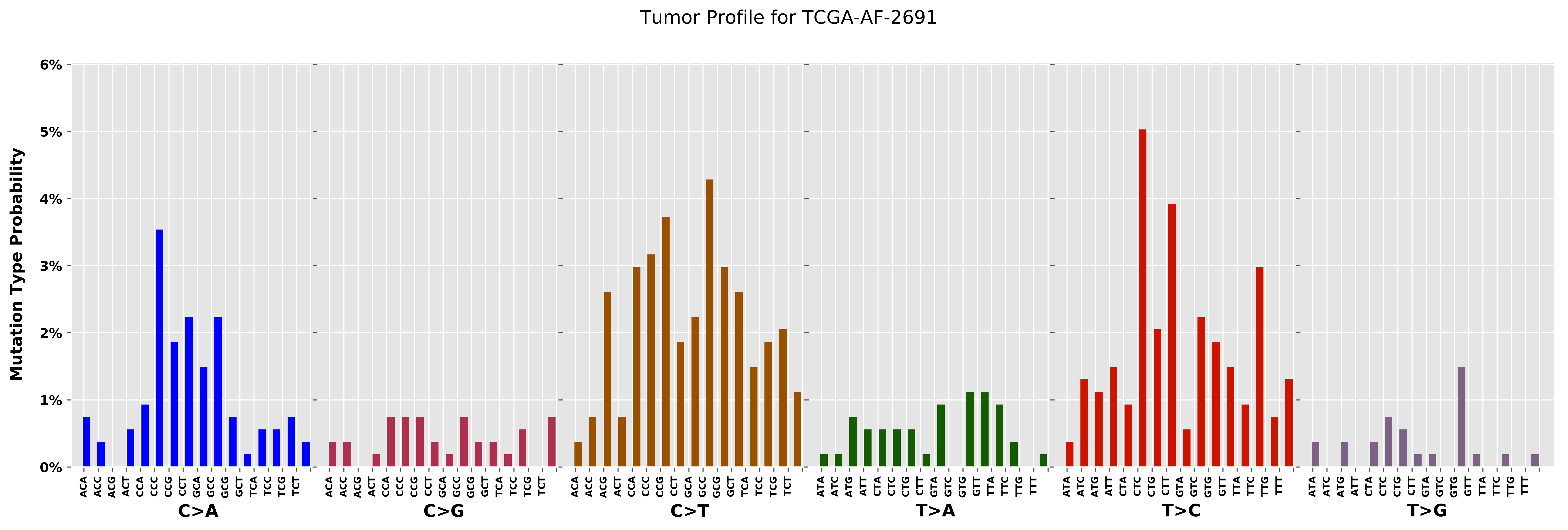

Tumor Profile for TCGA-AF-2691
Mutation Type Probability
6%
5%
4%
3%
2%
1%
0%
C>A
C>G
C>T
T>A
T>C
T>G
ACA ACC ACG ACT CCA CCC CCG CCT GCA GCC GCG GCT TCA TCC TCG TCT
ATA ATC ATG ATT CTA CTC CTG CTT GTA GTC GTG GTT TTA TTC TTG TTT

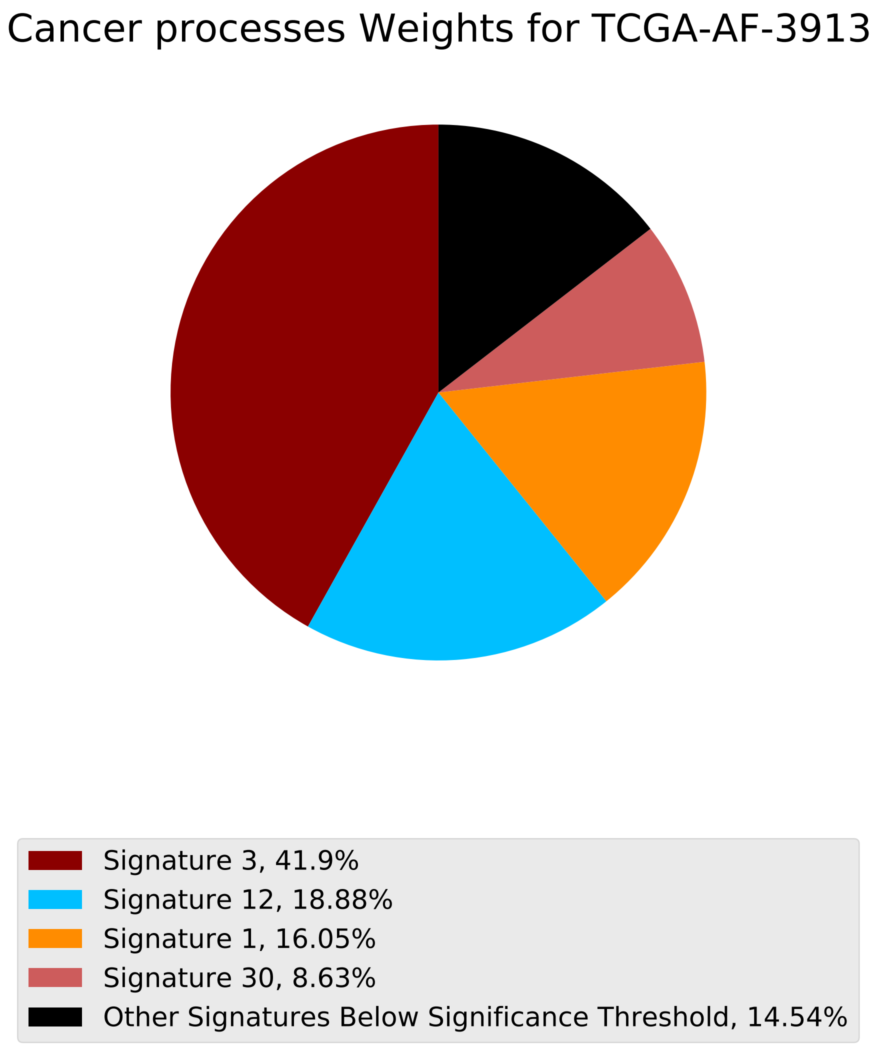

Cancer processes Weights for TCGA-AF-3913
Signature 3, 41.9%
Signature 12, 18.88%
Signature 1, 16.05%
Signature 30, 8.63%
Other Signatures Below Significance Threshold, 14.54%

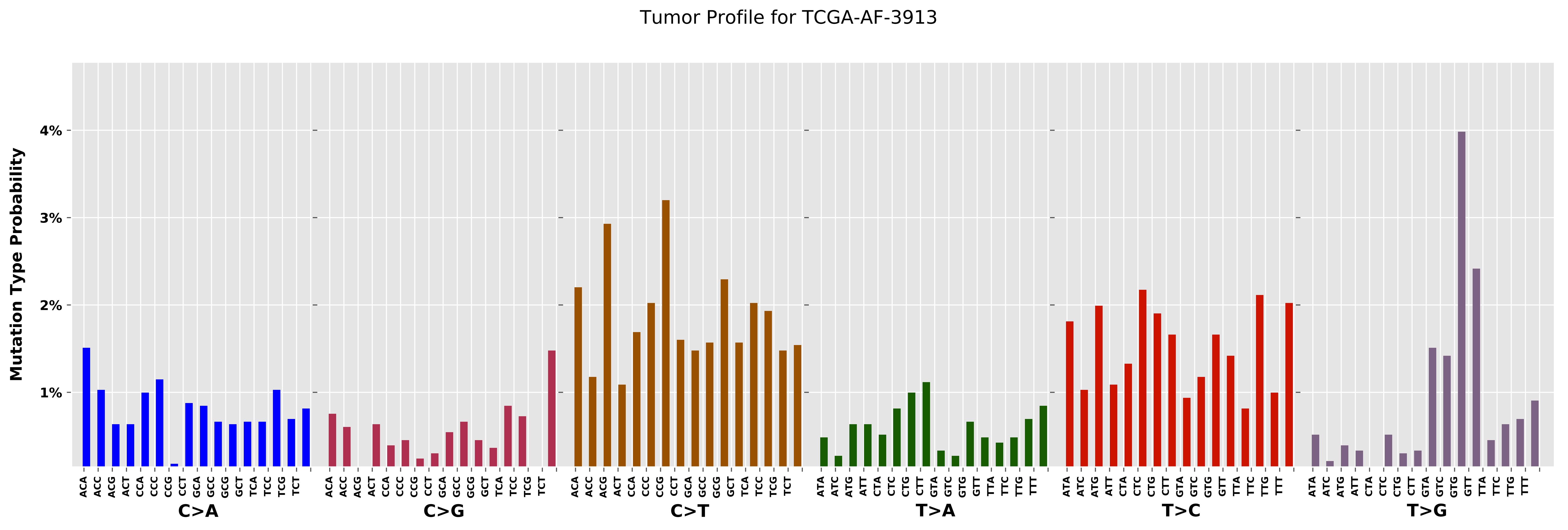

Tumor Profile for TCGA-AF-3913
Mutation Type Probability
4%
3%
2%
1%
ACA ACC ACG ACT CCA CCC CCG CCT GCA GCC GCG GCT TCA TCC TCG TCT
C>A
C>G
C>T
ATA ATC ATG ATT CTA CTC CTG CTT GTA GTC GTG GTT TTA TTC TTG TTT
T>A
T>C
T>G

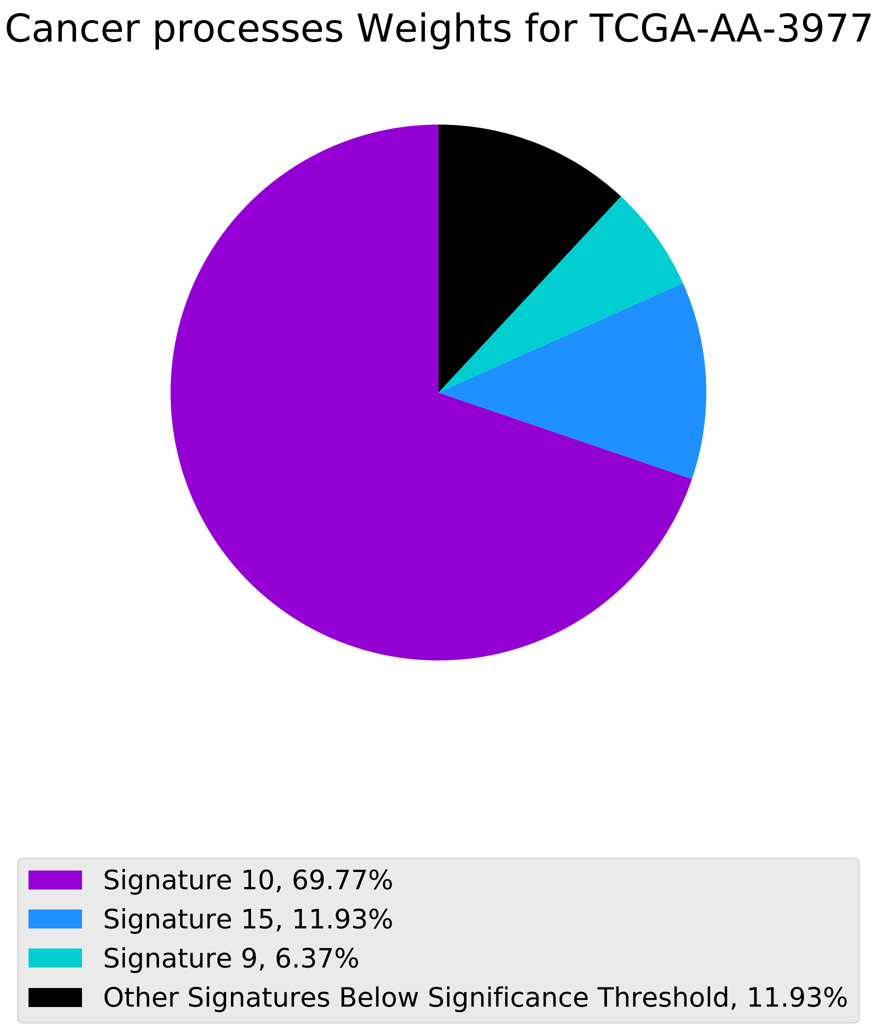
Cancer processes Weights for TCGA-AA-3977
Signature 10, 69.77%
Signature 15, 11.93%
Signature 9, 6.37%
Other Signatures Below Significance Threshold, 11.93%

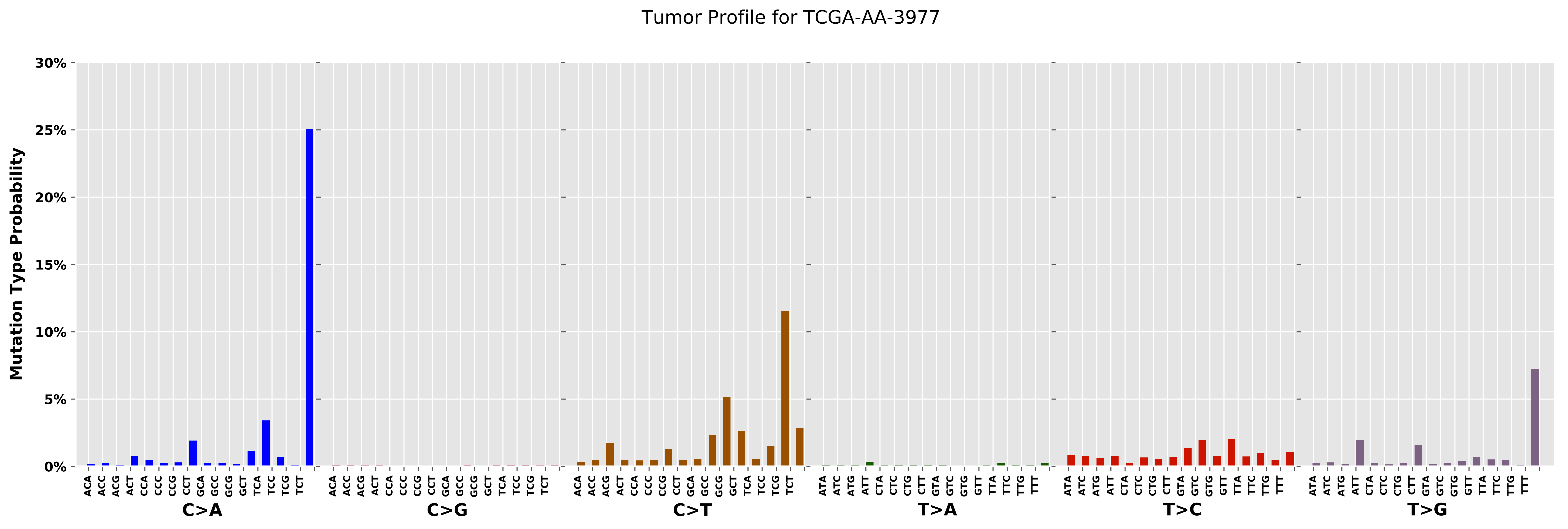
Tumor Profile for TCGA-AA-3977
Mutation Type Probability
0%
5%
10%
15%
20%
25%
30%
C>A
C>G
C>T
T>A
T>C
T>G

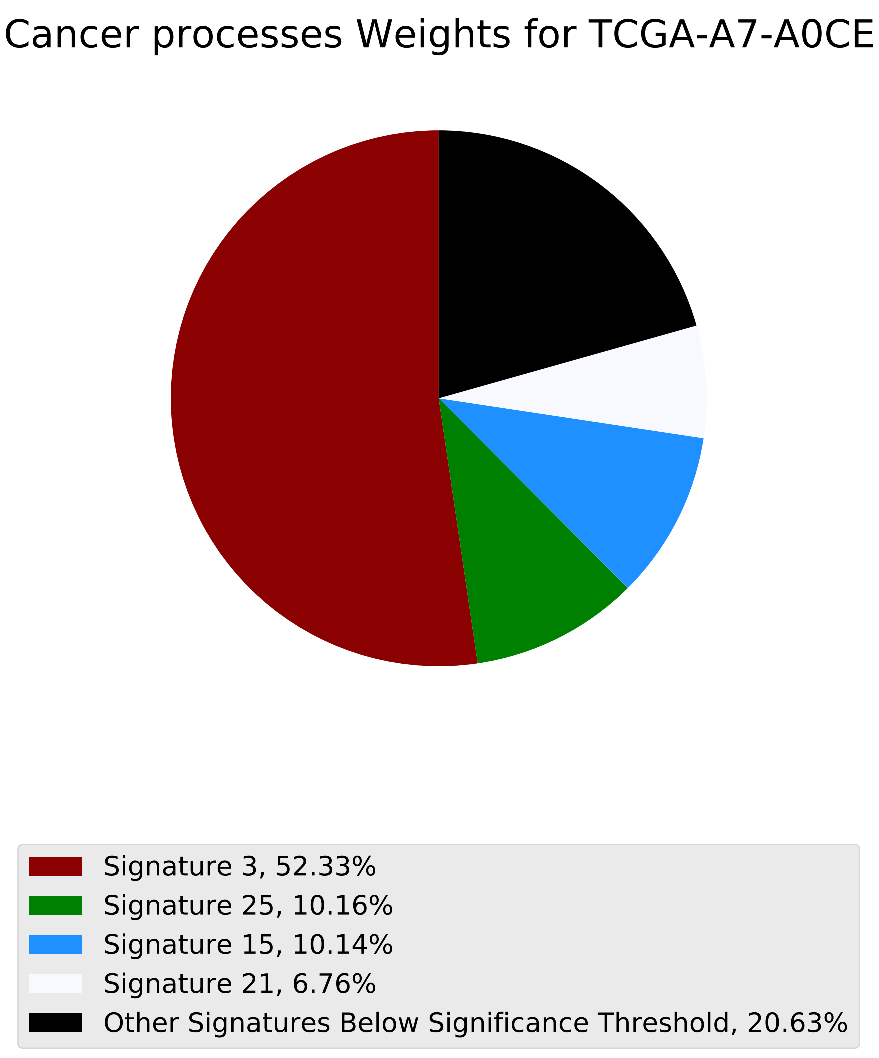
Cancer processes Weights for TCGA-A7-A0CE
Signature 3, 52.33%
Signature 25, 10.16%
Signature 15, 10.14%
Signature 21, 6.76%
Other Signatures Below Significance Threshold, 20.63%

Tumor Profile for TCGA-A7-A0CE

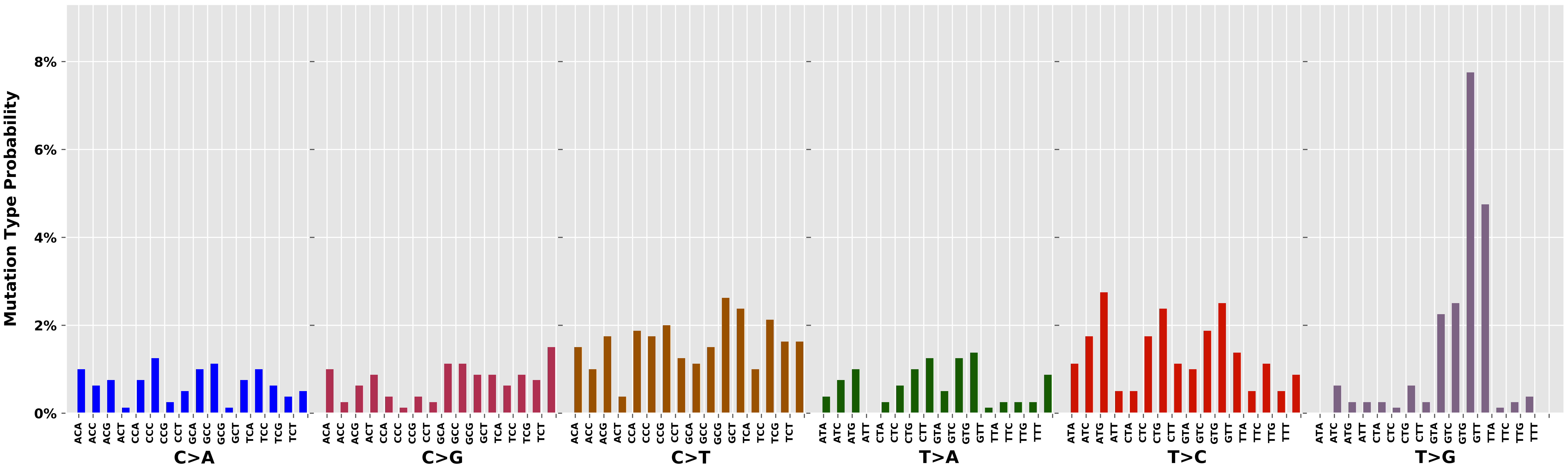

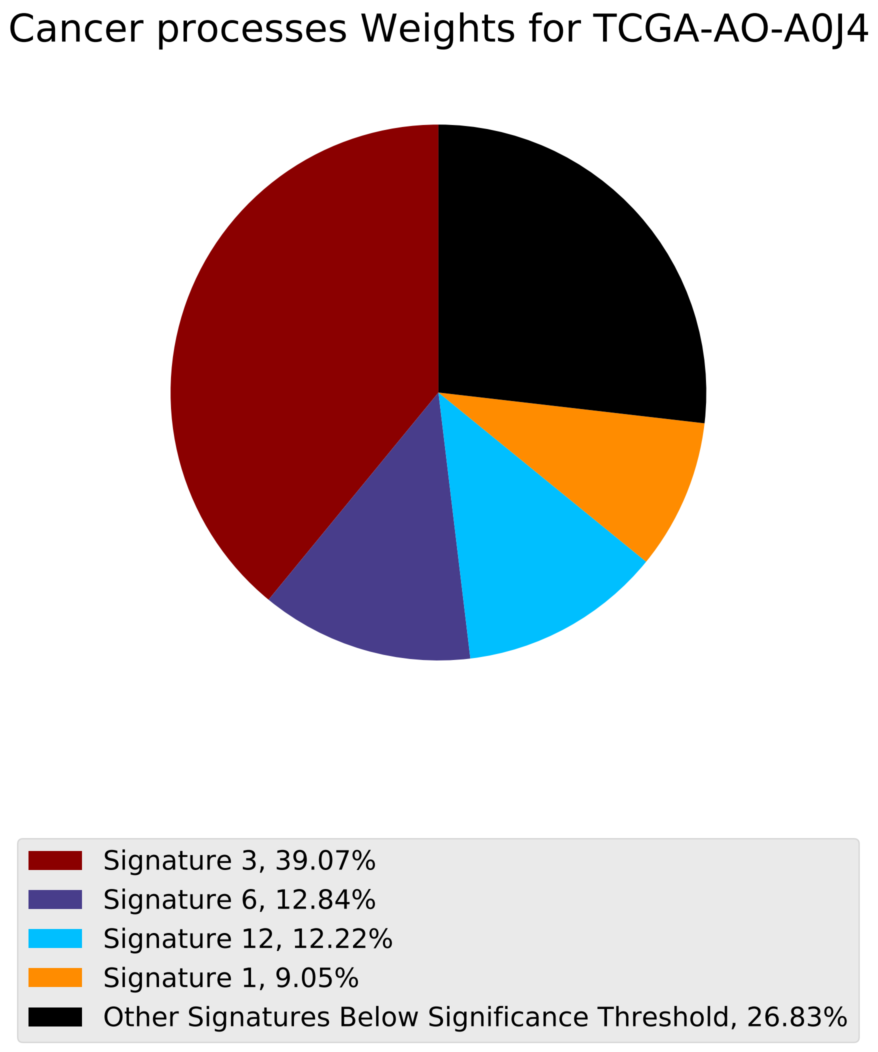
Cancer processes Weights for TCGA-AO-A0J4
Signature 3, 39.07%
Signature 6, 12.84%
Signature 12, 12.22%
Signature 1, 9.05%
Other Signatures Below Significance Threshold, 26.83%

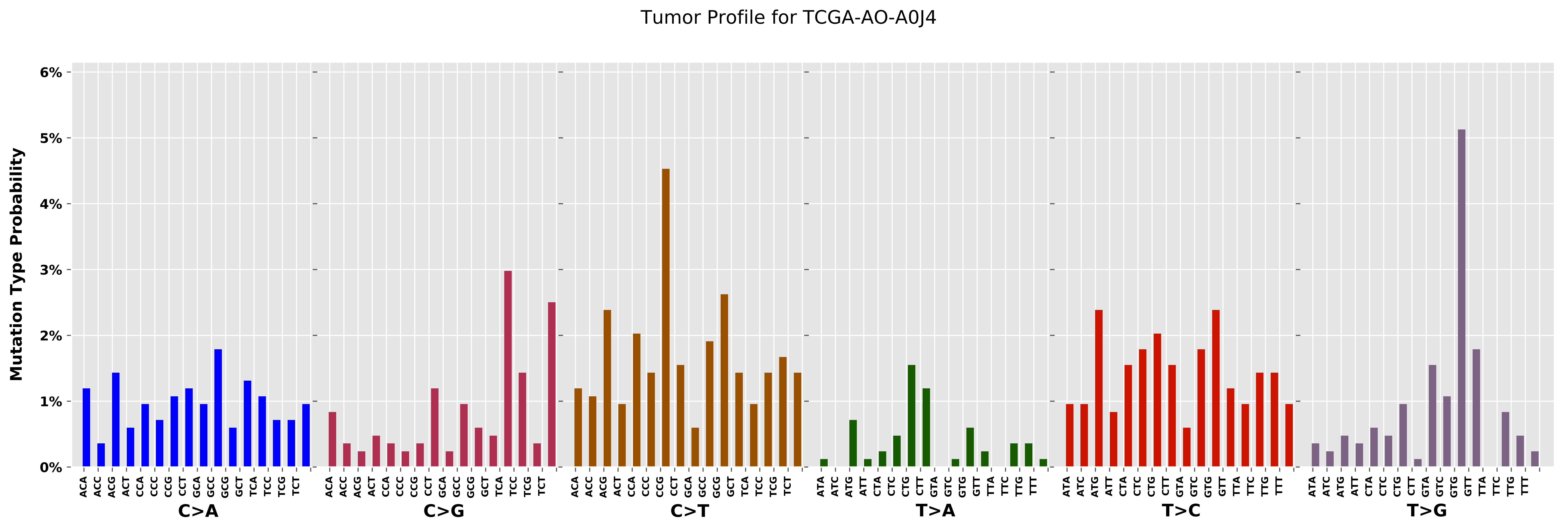
Tumor Profile for TCGA-AO-A0J4
Mutation Type Probability
0%
1%
2%
3%
4%
5%
6%
ACA ACC ACG ACT CCA CCC CCG CCT GCA GCC GCG GCT TCA TCC TCG TCT
C>A
C>G
C>T
ATA ATC ATG ATT CTA CTC CTG CTT GTA GTC GTG GTT TTA TTC TTG TTT
T>A
T>C
T>G

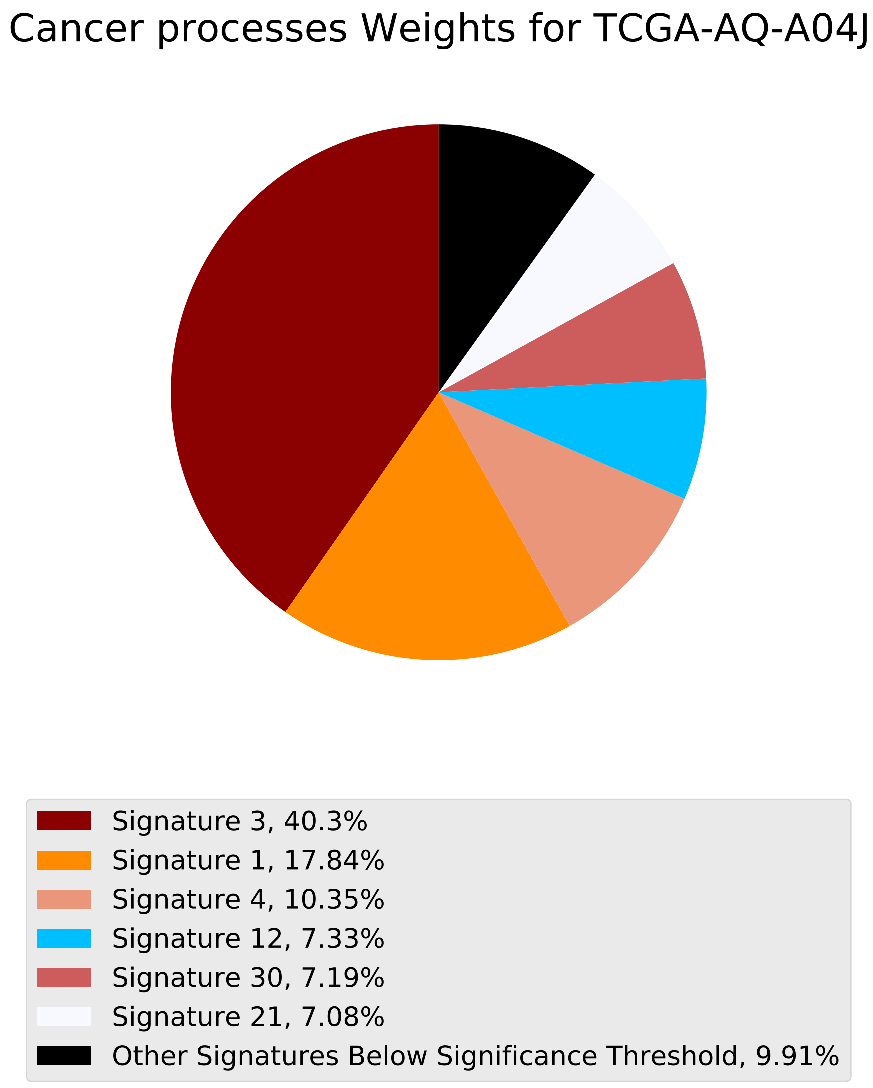
Cancer processes Weights for TCGA-AQ-A04J
Signature 3, 40.3%
Signature 1, 17.84%
Signature 4, 10.35%
Signature 12, 7.33%
Signature 30, 7.19%
Signature 21, 7.08%
Other Signatures Below Significance Threshold, 9.91%

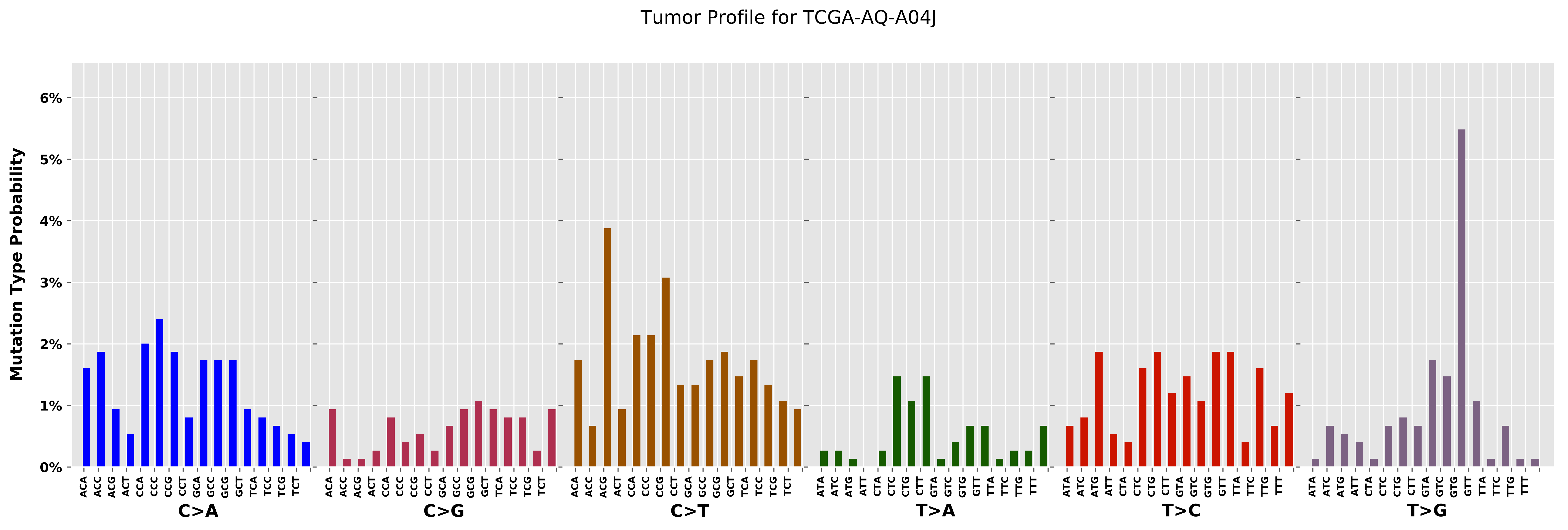
Tumor Profile for TCGA-AQ-A04J
Mutation Type Probability
0%
1%
2%
3%
4%
5%
6%
C>A
C>G
C>T
T>A
T>C
T>G

Cancer processes Weights for TCGA-AG-4007

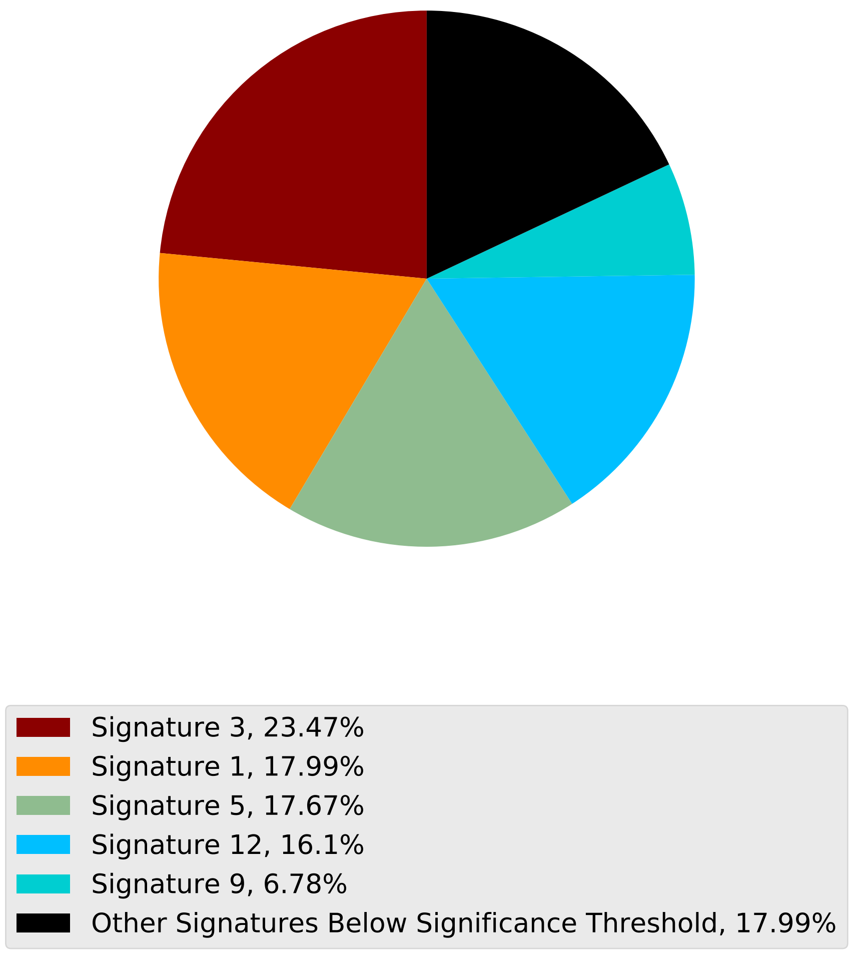

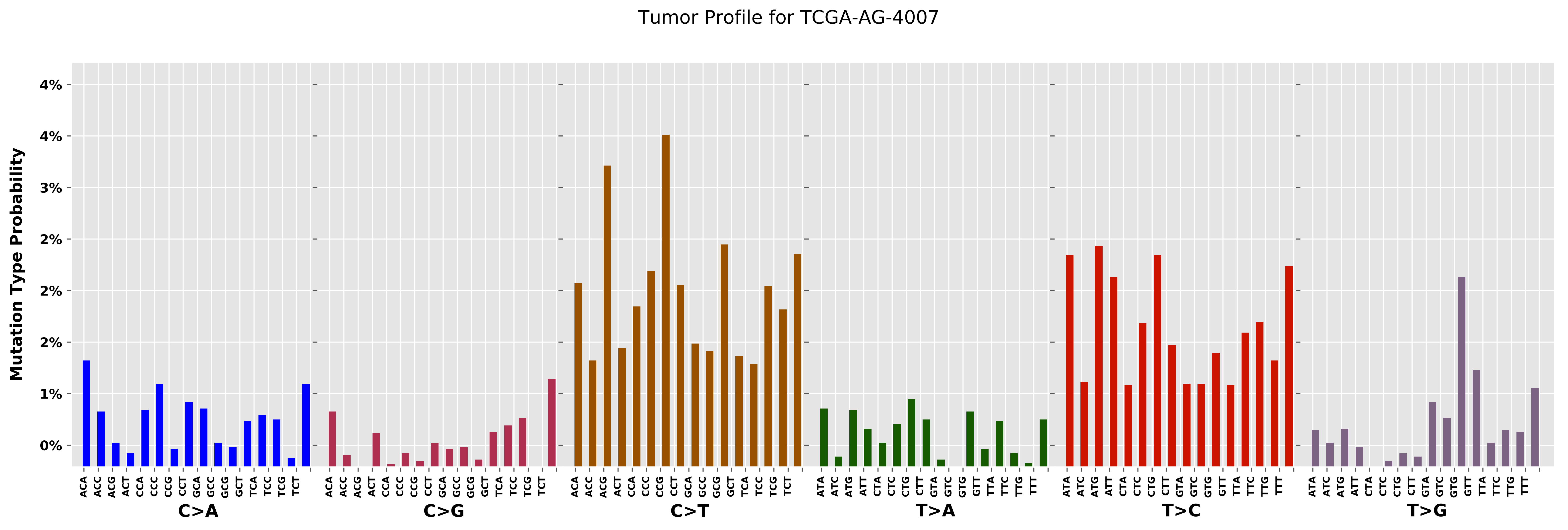
Tumor Profile for TCGA-AG-4007
Mutation Type Probability
4%
4%
3%
2%
2%
2%
1%
0%
C>A
C>G
C>T
T>A
T>C
T>G
ACA ACC ACG ACT CCA CCC CCG CCT GCA GCC GCG GCT TCA TCC TCG TCT
ATA ATC ATG ATT CTA CTC CTG CTT GTA GTC GTG GTT TTA TTC TTG TTT

Cancer processes Weights for TCGA-CA-6717

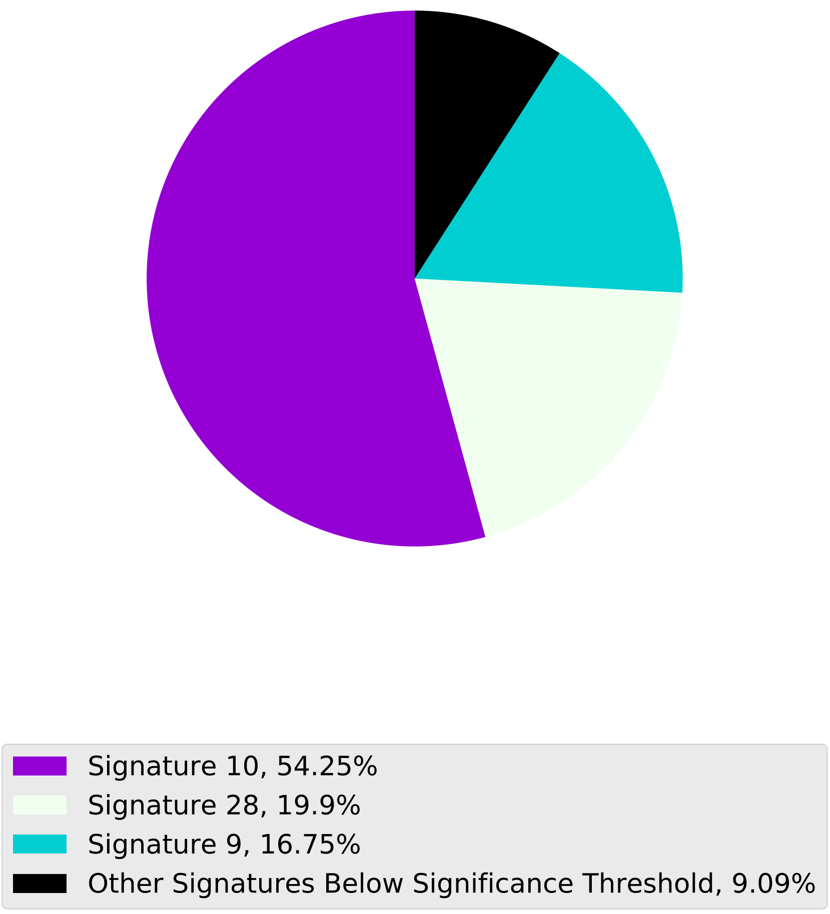

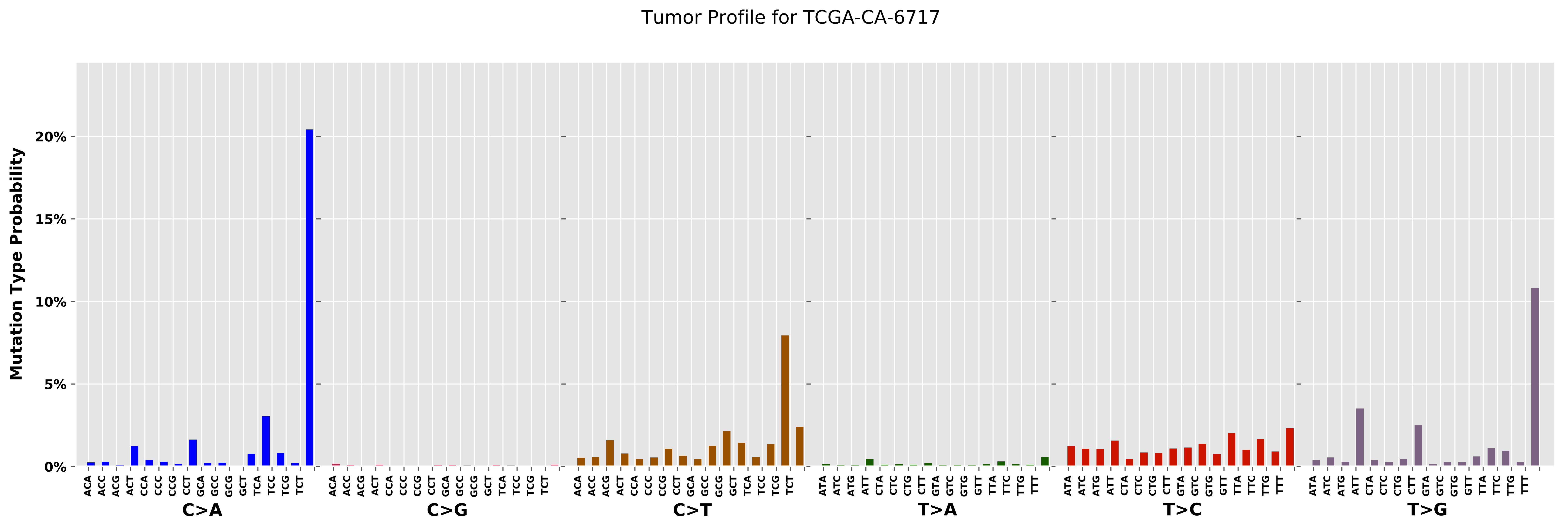

Tumor Profile for TCGA-CA-6717
Mutation Type Probability
0%
5%
10%
15%
20%
C>A
C>G
C>T
T>A
T>C
T>G

Cancer processes Weights for TCGA-E2-A14P

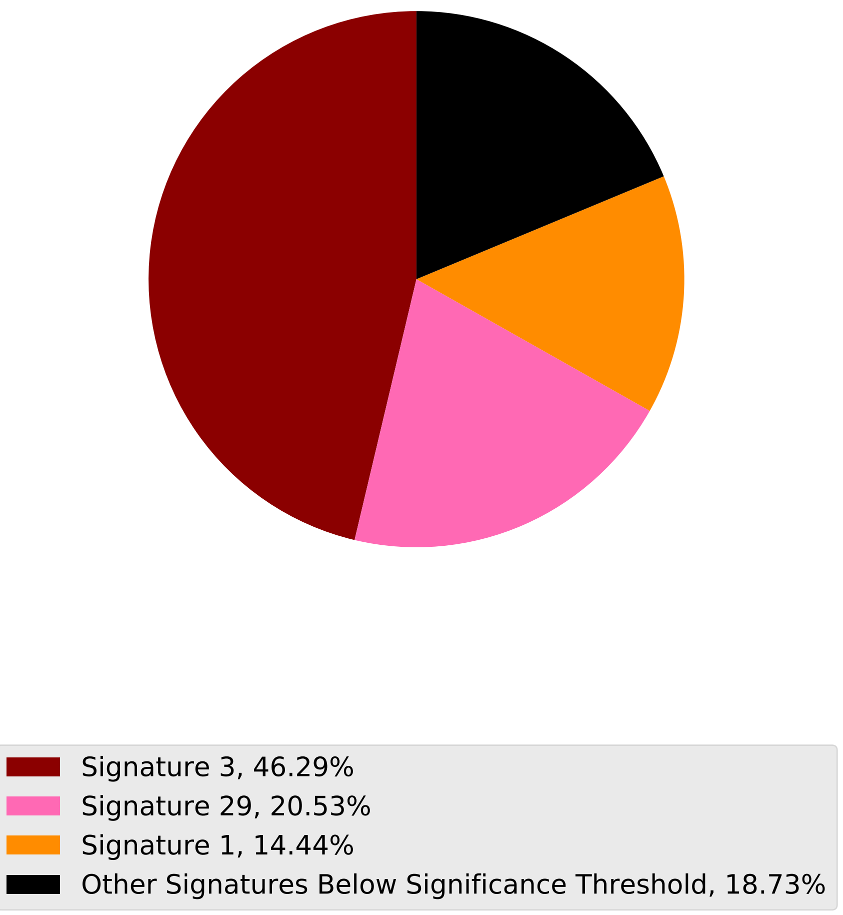

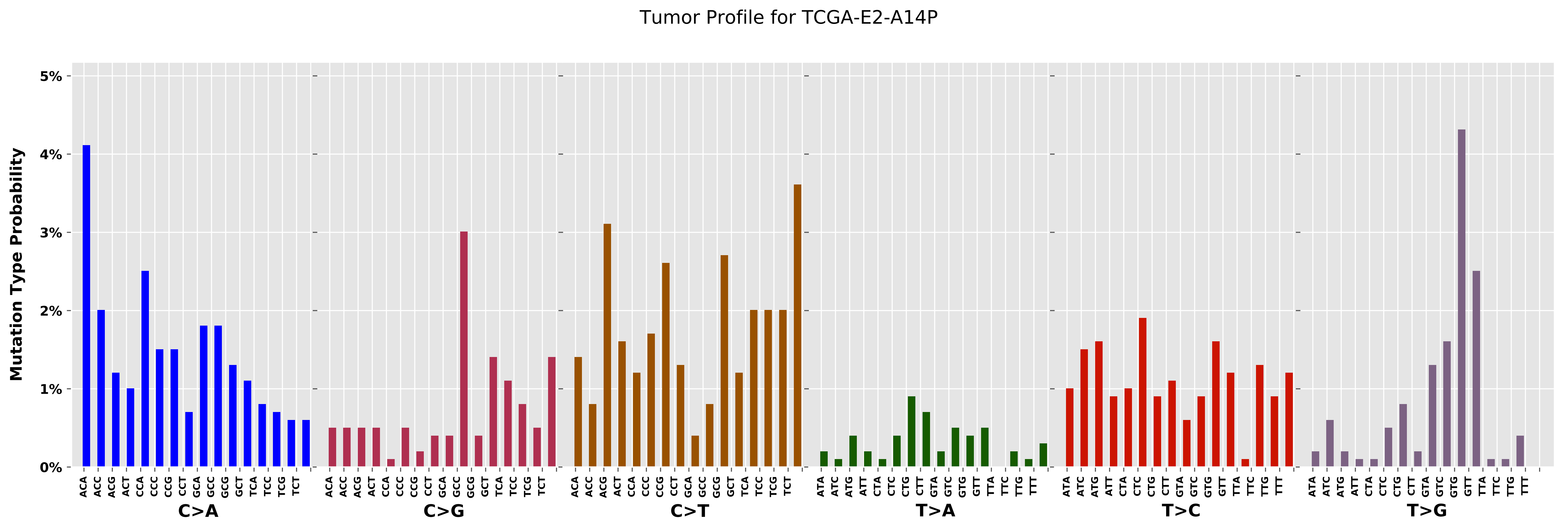
Tumor Profile for TCGA-E2-A14P
Mutation Type Probability
0%
1%
2%
3%
4%
5%
C>A
C>G
C>T
T>A
T>C
T>G

Cancer processes Weights for TCGA-AZ-6601

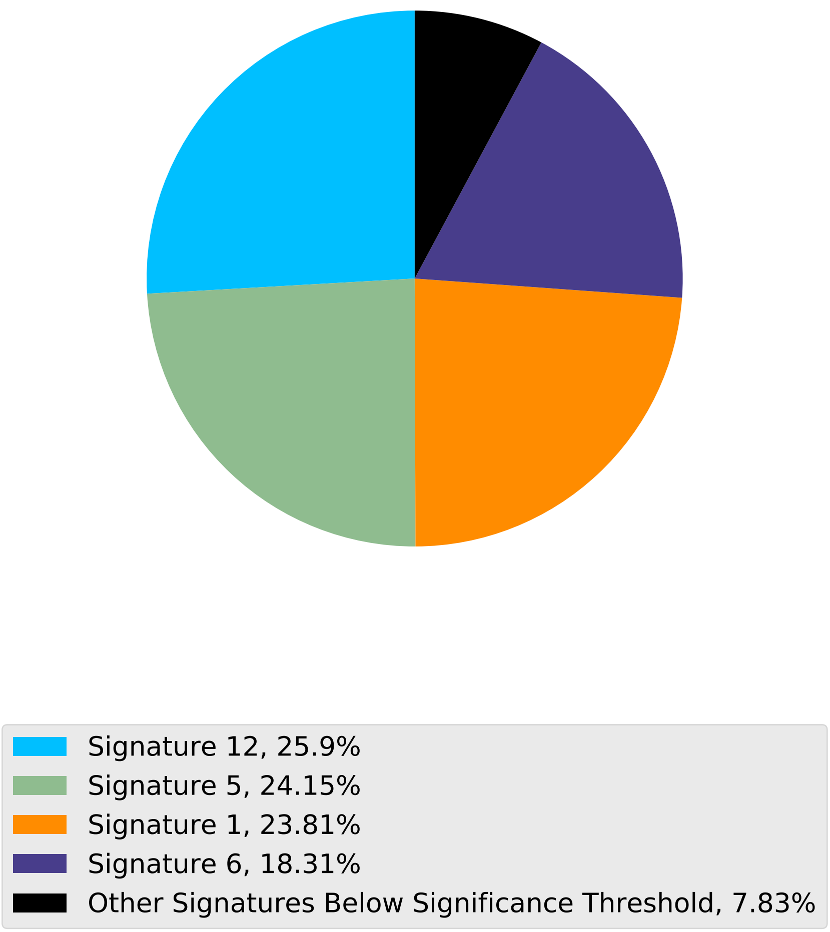

Tumor Profile for TCGA-AZ-6601

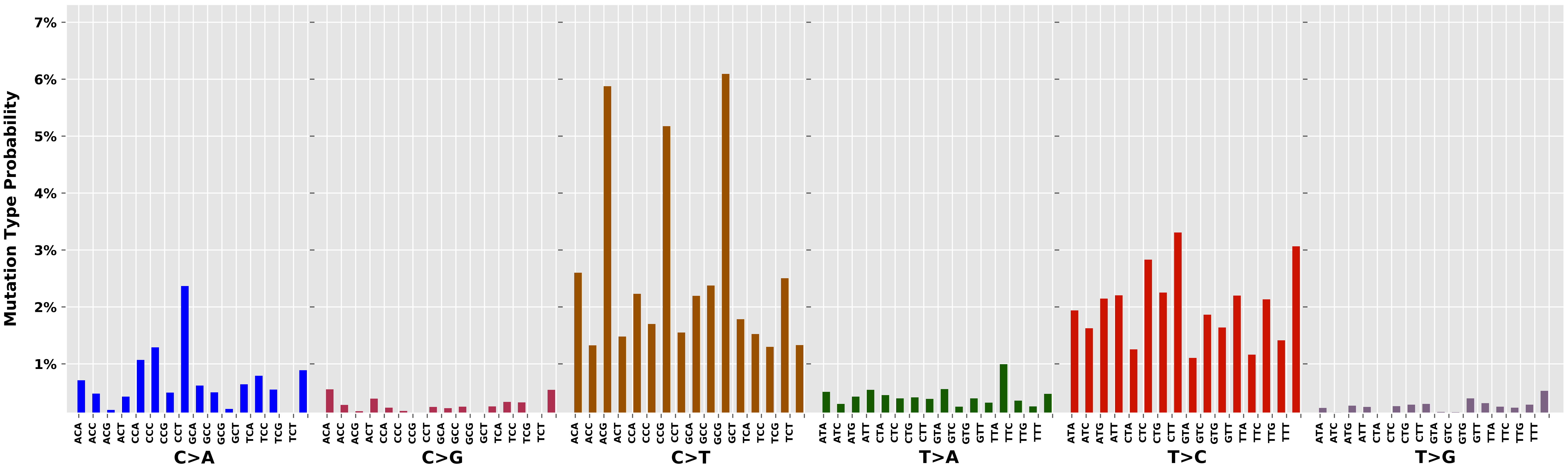

Cancer processes Weights for TCGA-A6-6781

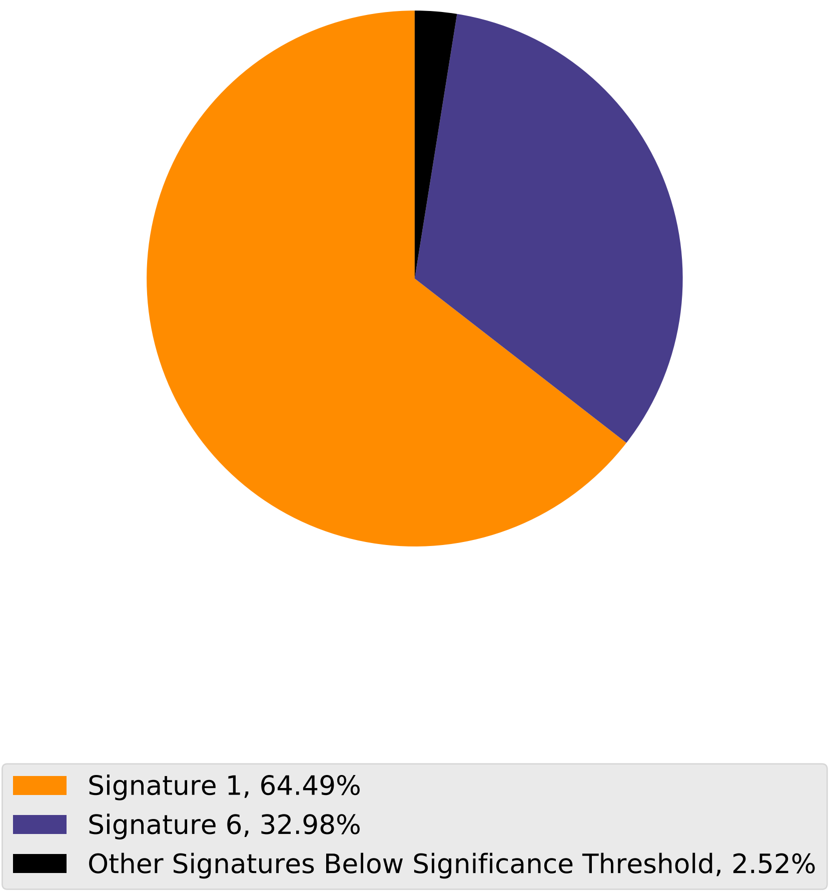

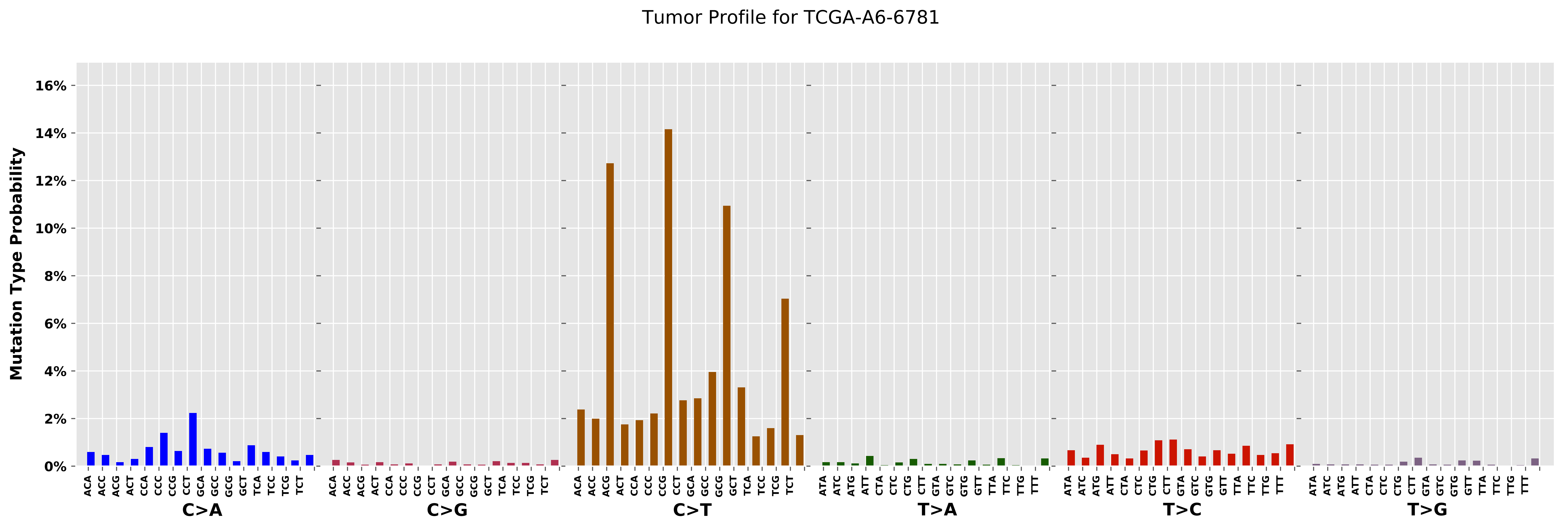

Tumor Profile for TCGA-A6-6781
Mutation Type Probability
0%
2%
4%
6%
8%
10%
12%
14%
16%
C>A
C>G
C>T
T>A
T>C
T>G

Cancer processes Weights for TCGA-GM-A2DF

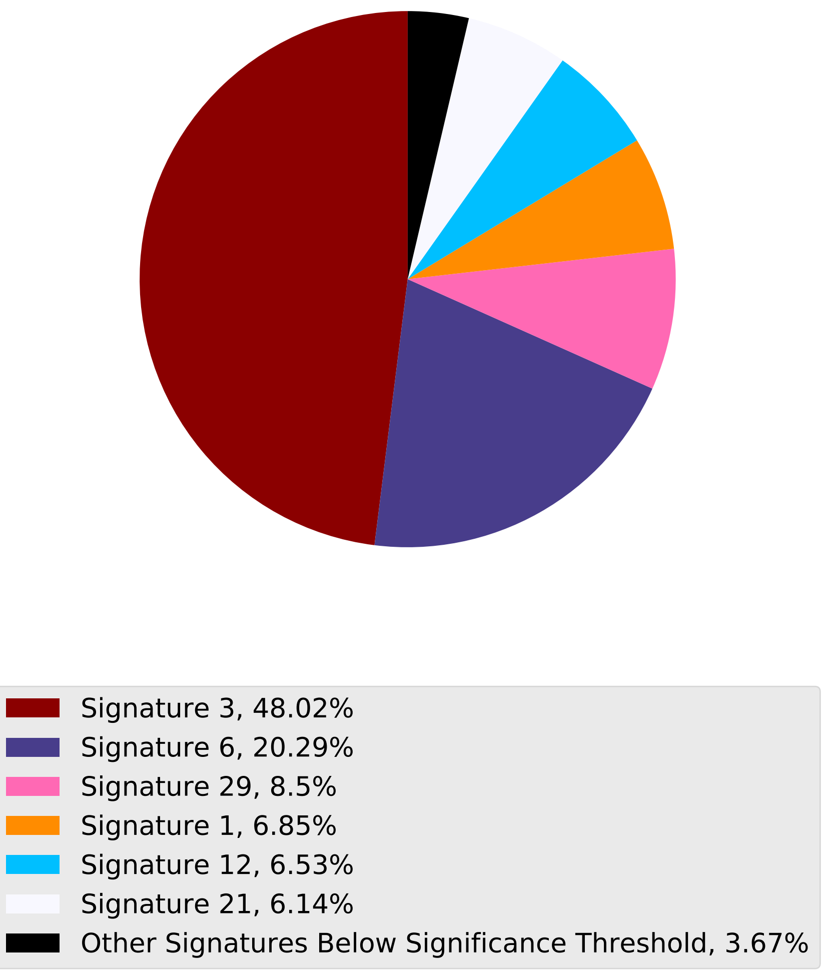

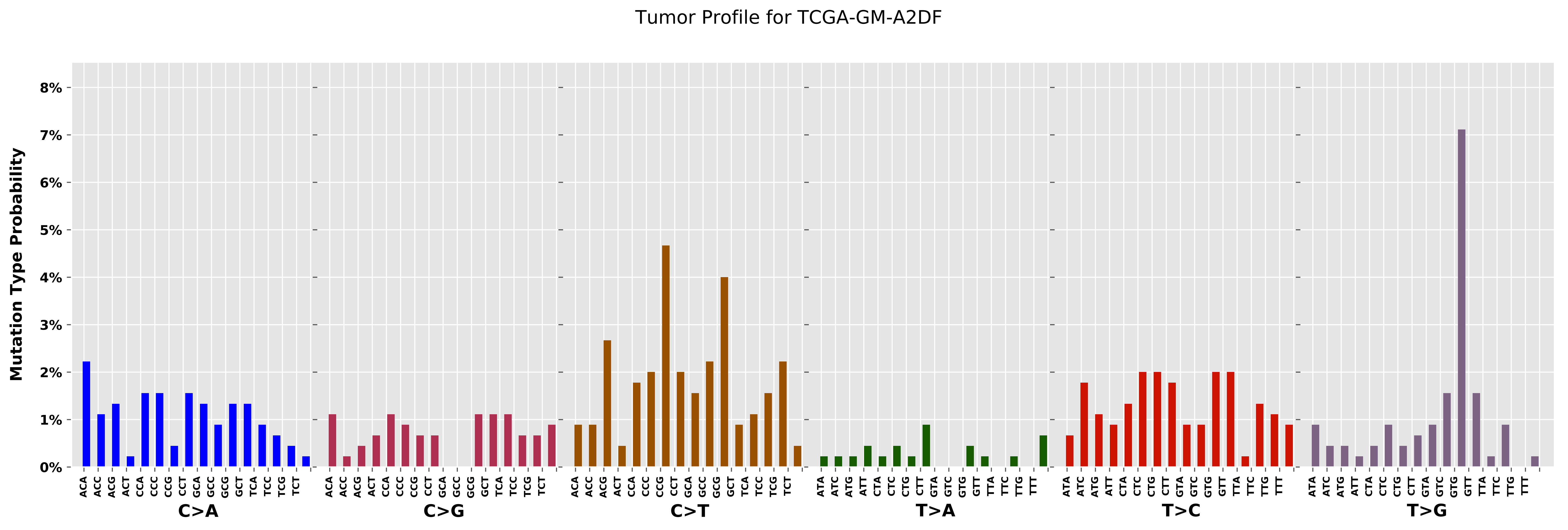

Tumor Profile for TCGA-GM-A2DF
Mutation Type Probability
0%
1%
2%
3%
4%
5%
6%
7%
8%
C>A
C>G
C>T
T>A
T>C
T>G

Cancer processes Weights for TCGA-C8-A12L

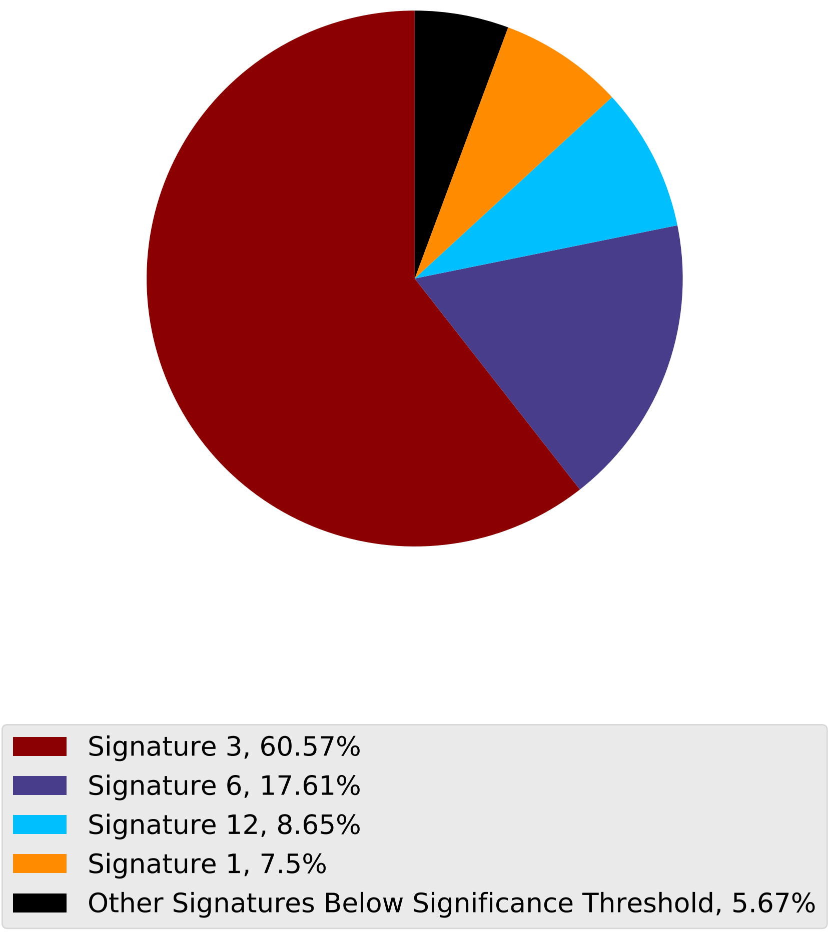

Tumor Profile for TCGA-C8-A12L

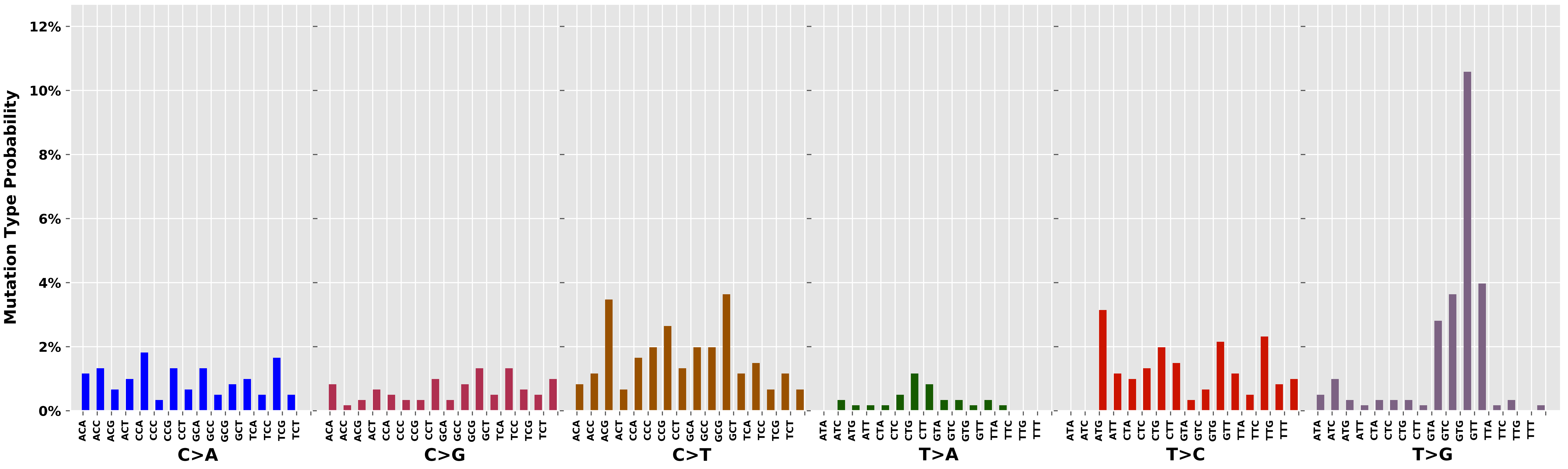

Cancer processes Weights for TCGA-AG-3890

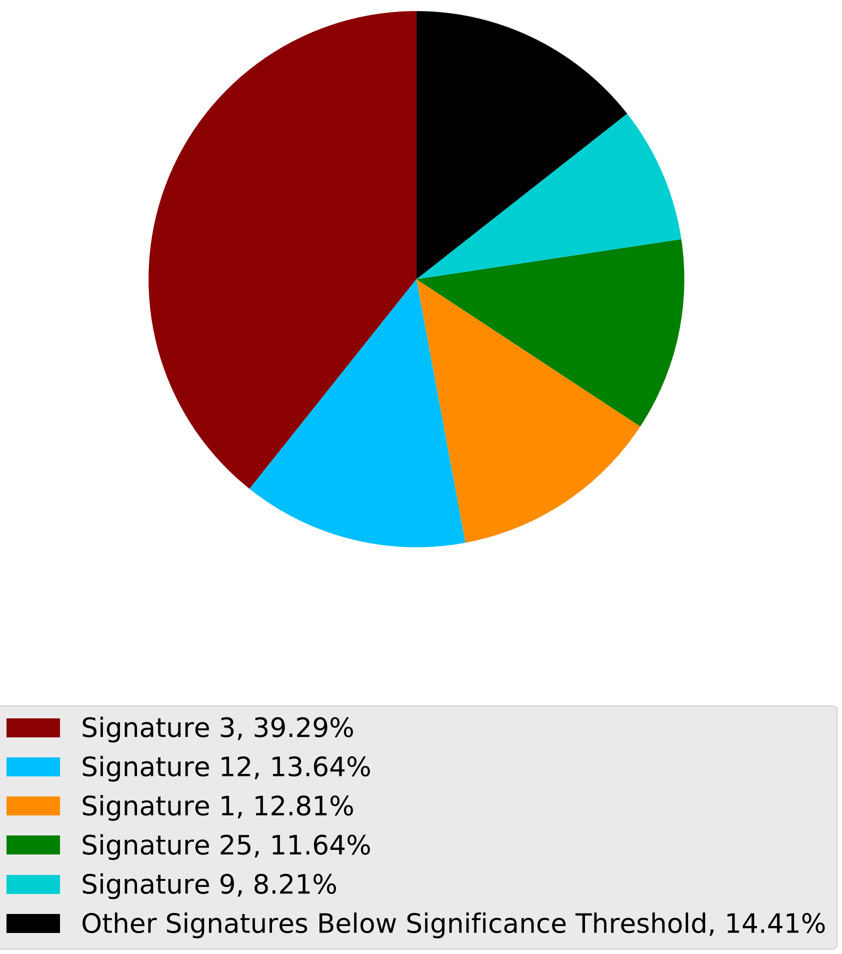

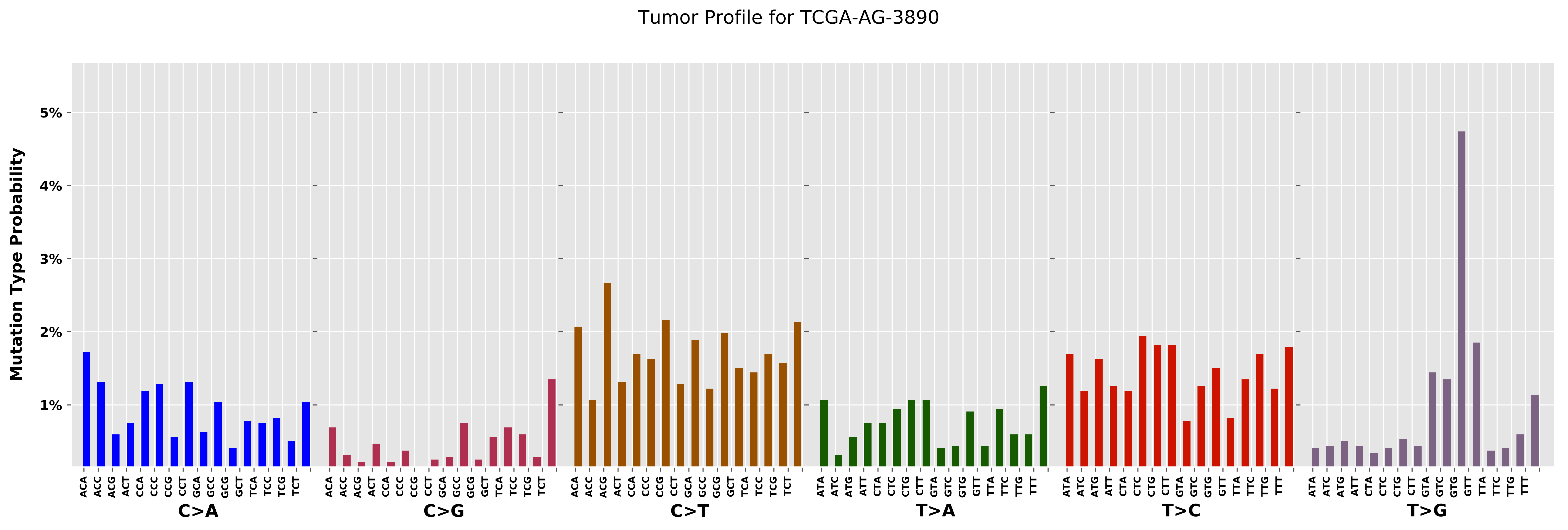
Tumor Profile for TCGA-AG-3890
Mutation Type Probability
5%
4%
3%
2%
1%
C>A
C>G
C>T
T>A
T>C
T>G

Cancer processes Weights for TCGA-E2-A1LK

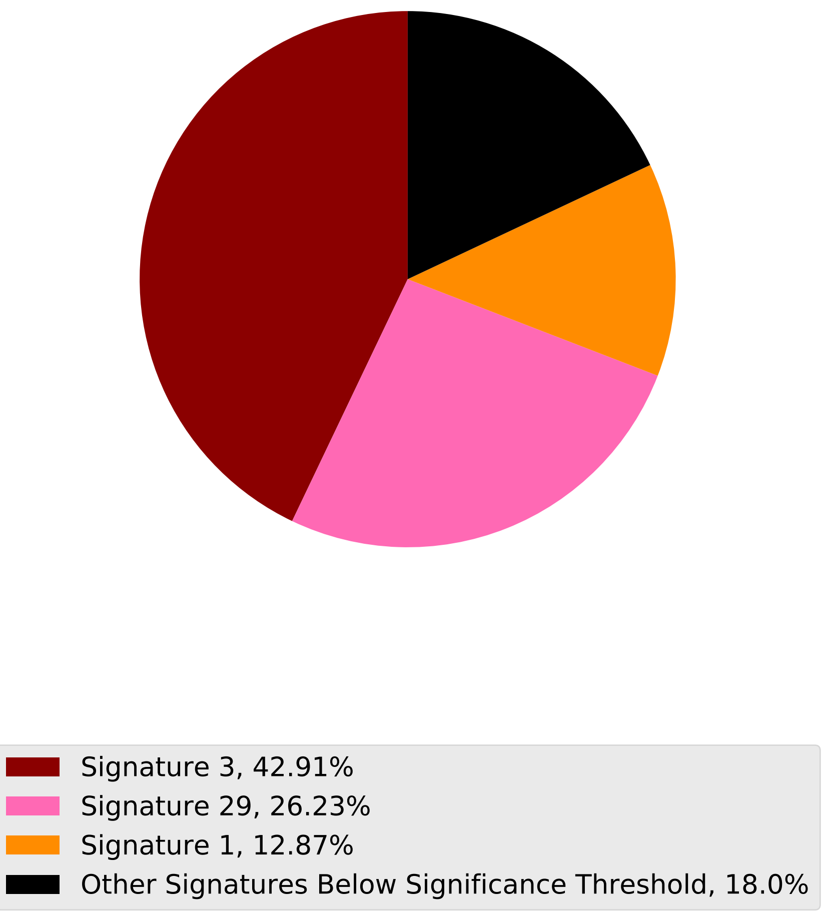

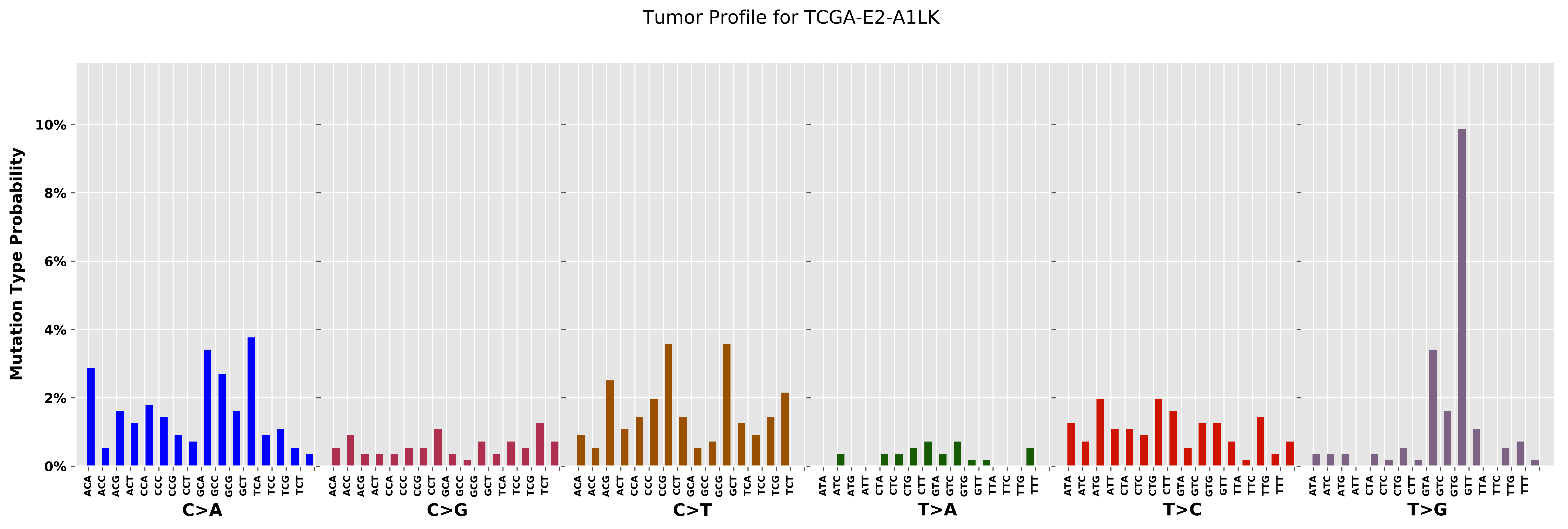

Tumor Profile for TCGA-E2-A1LK
Mutation Type Probability
0%
2%
4%
6%
8%
10%
C>A
C>G
C>T
T>A
T>C
T>G

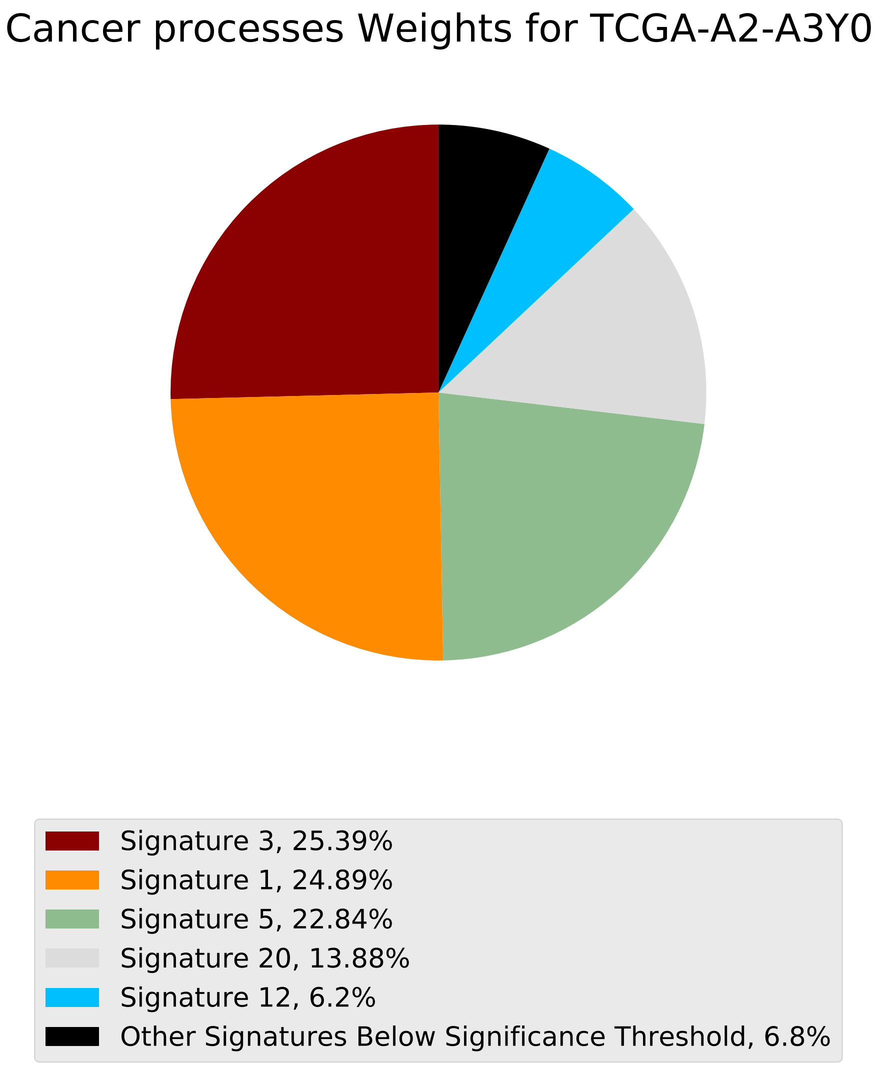
Cancer processes Weights for TCGA-A2-A3Y0
Signature 3, 25.39%
Signature 1, 24.89%
Signature 5, 22.84%
Signature 20, 13.88%
Signature 12, 6.2%
Other Signatures Below Significance Threshold, 6.8%

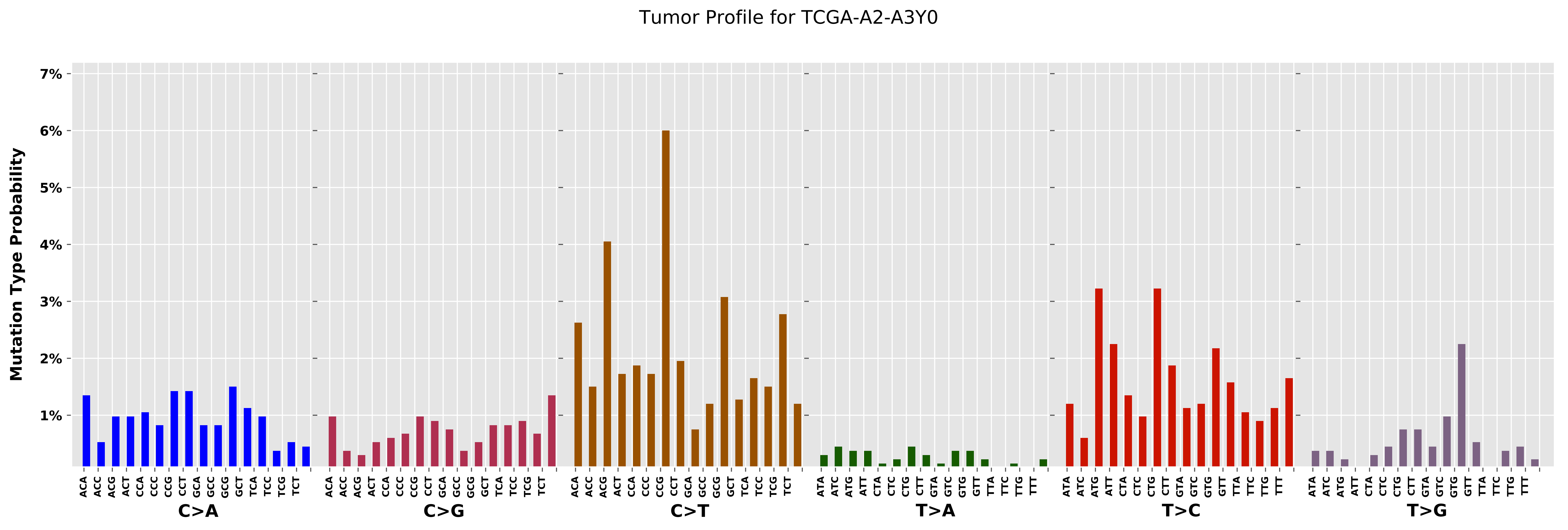
Tumor Profile for TCGA-A2-A3Y0
Mutation Type Probability
7%
6%
5%
4%
3%
2%
1%
C>A
C>G
C>T
T>A
T>C
T>G

Cancer processes Weights for TCGA-B6-A0I1

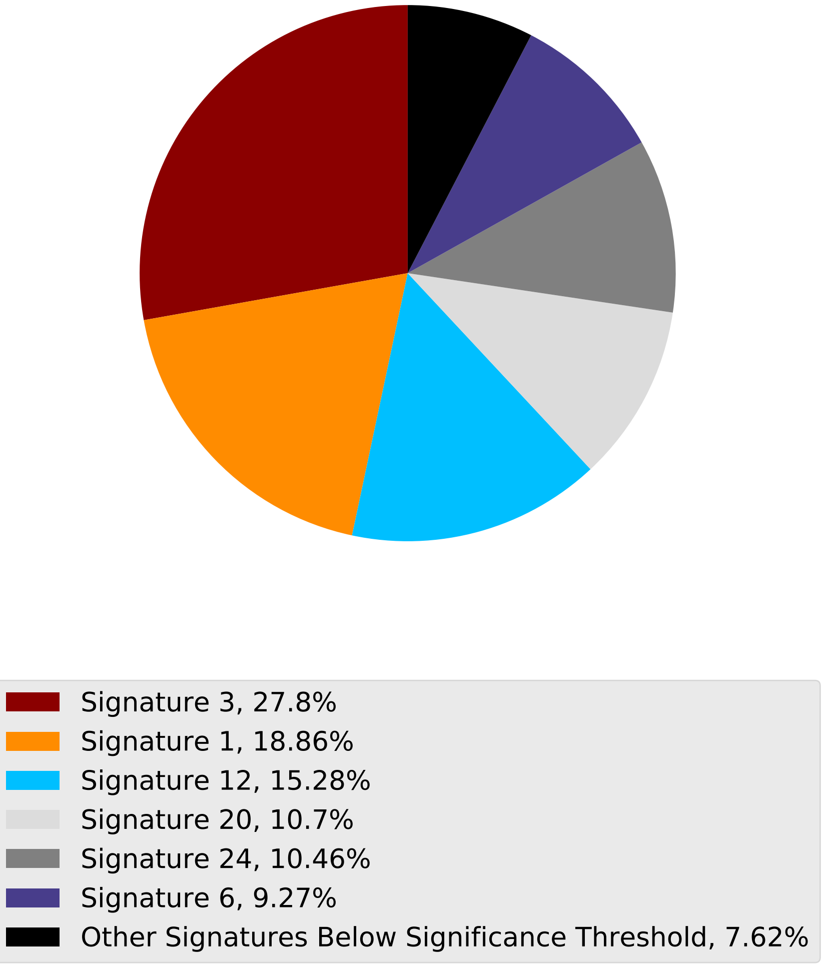

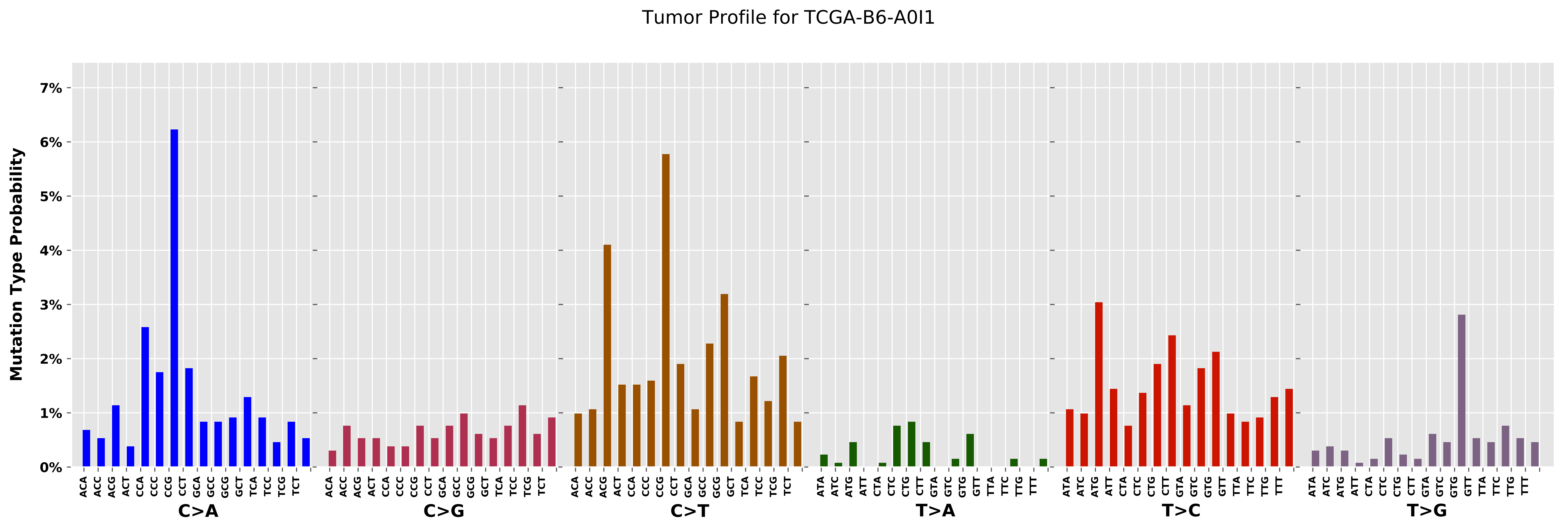

Tumor Profile for TCGA-B6-A0I1
Mutation Type Probability
0%
1%
2%
3%
4%
5%
6%
7%
C>A
C>G
C>T
T>A
T>C
T>G
ACA ACC ACG ACT CCA CCC CCG CCT GCA GCC GCG GCT TCA TCC TCG TCT
ATA ATC ATG ATT CTA CTC CTG CTT GTA GTC GTG GTT TTA TTC TTG TTT

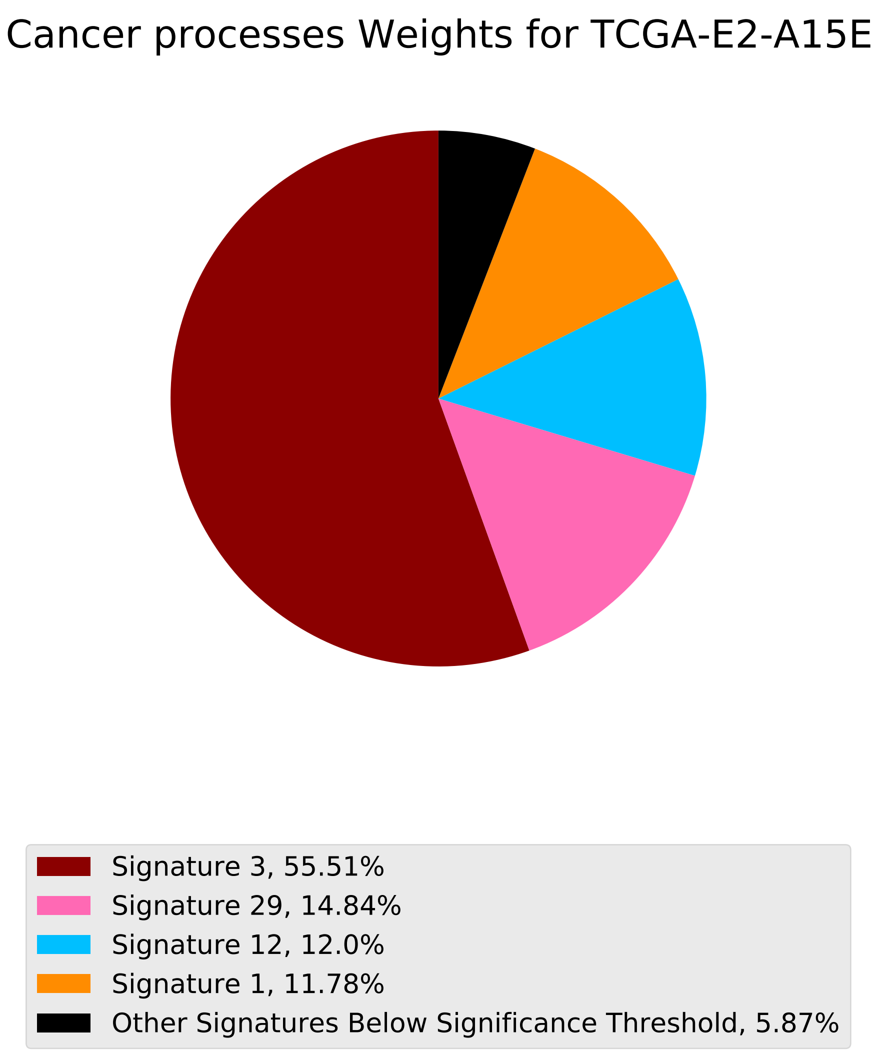
Cancer processes Weights for TCGA-E2-A15E
Signature 3, 55.51%
Signature 29, 14.84%
Signature 12, 12.0%
Signature 1, 11.78%
Other Signatures Below Significance Threshold, 5.87%

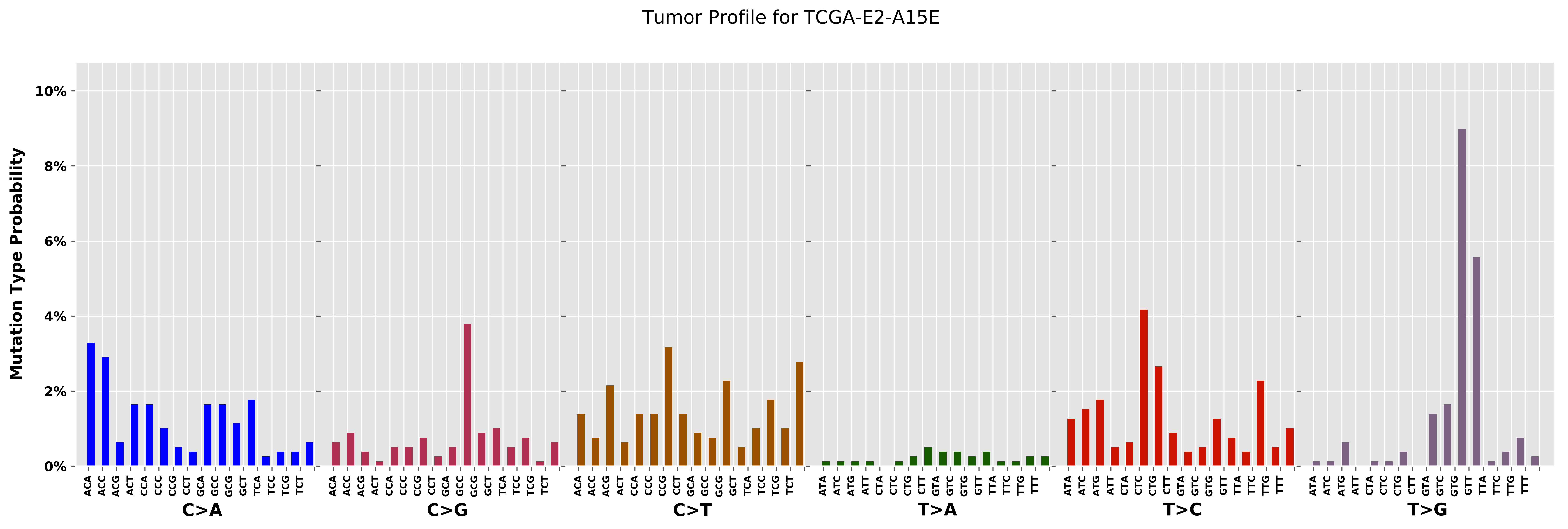
Tumor Profile for TCGA-E2-A15E
Mutation Type Probability
0%
2%
4%
6%
8%
10%
C>A
C>G
C>T
T>A
T>C
T>G

Cancer processes Weights for TCGA-A6-2680

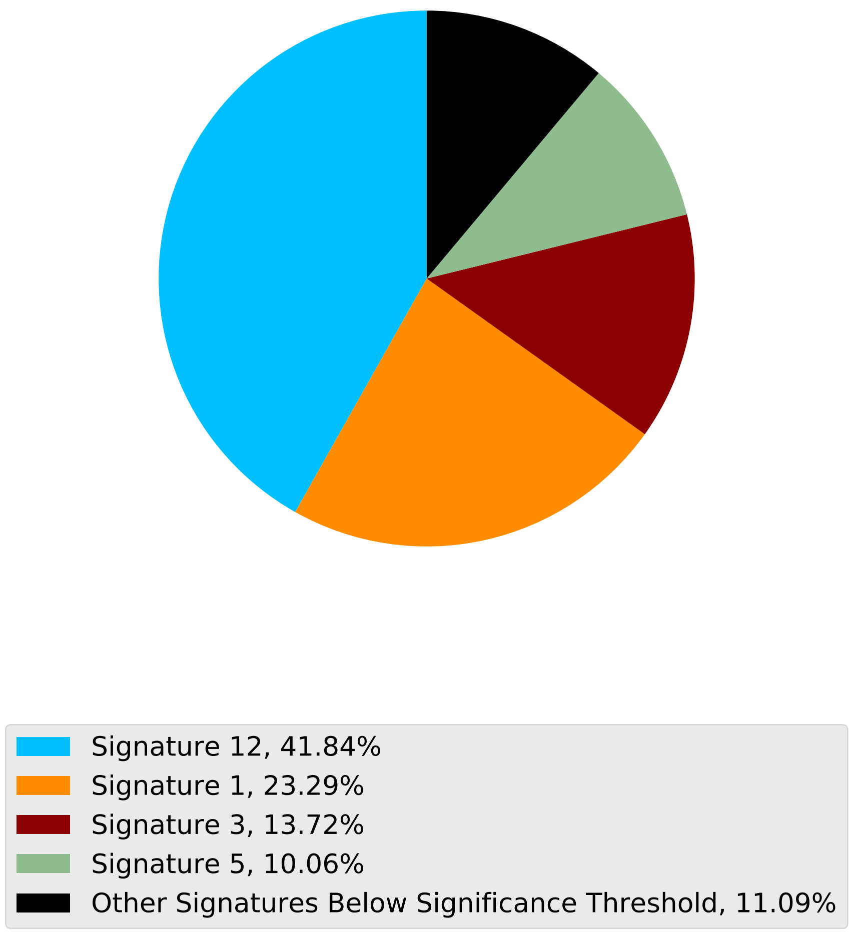

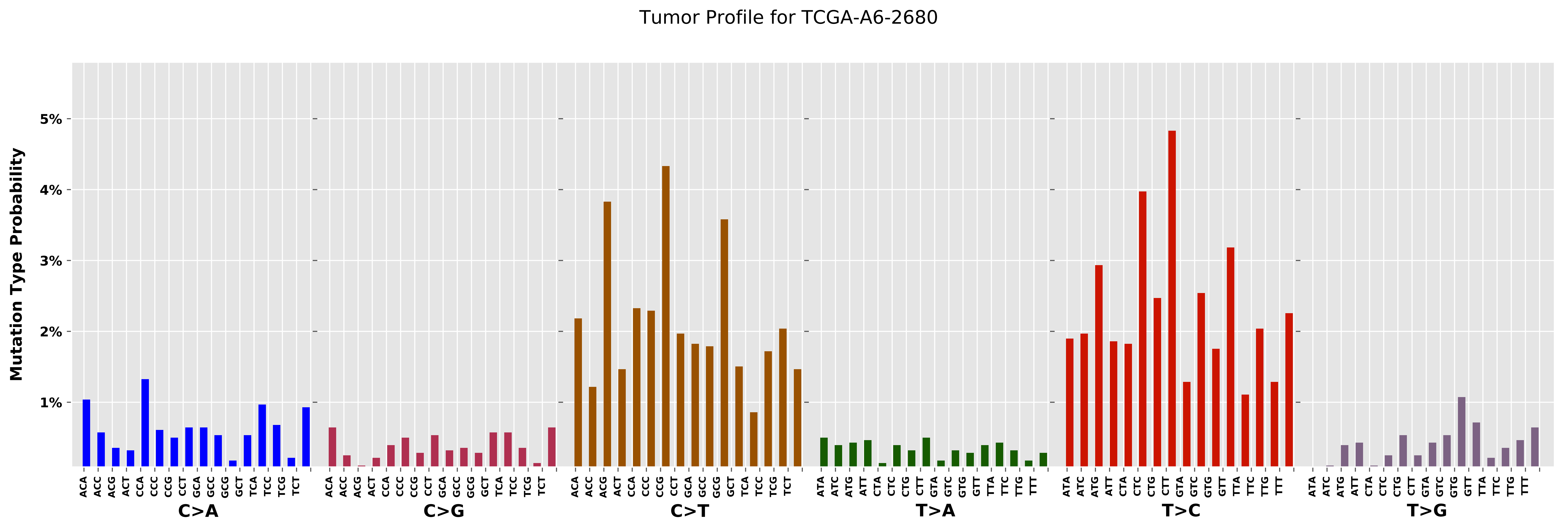

Tumor Profile for TCGA-A6-2680
Mutation Type Probability
5%
4%
3%
2%
1%
C>A
C>G
C>T
T>A
T>C
T>G

Cancer processes Weights for TCGA-A8-A07B

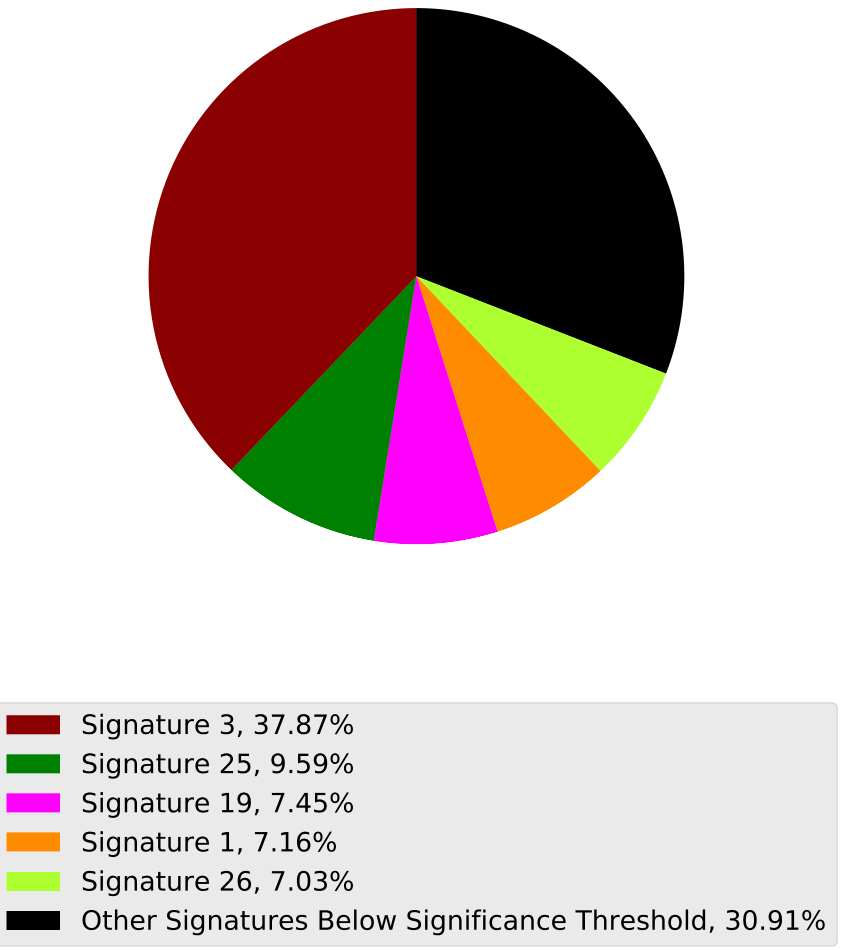

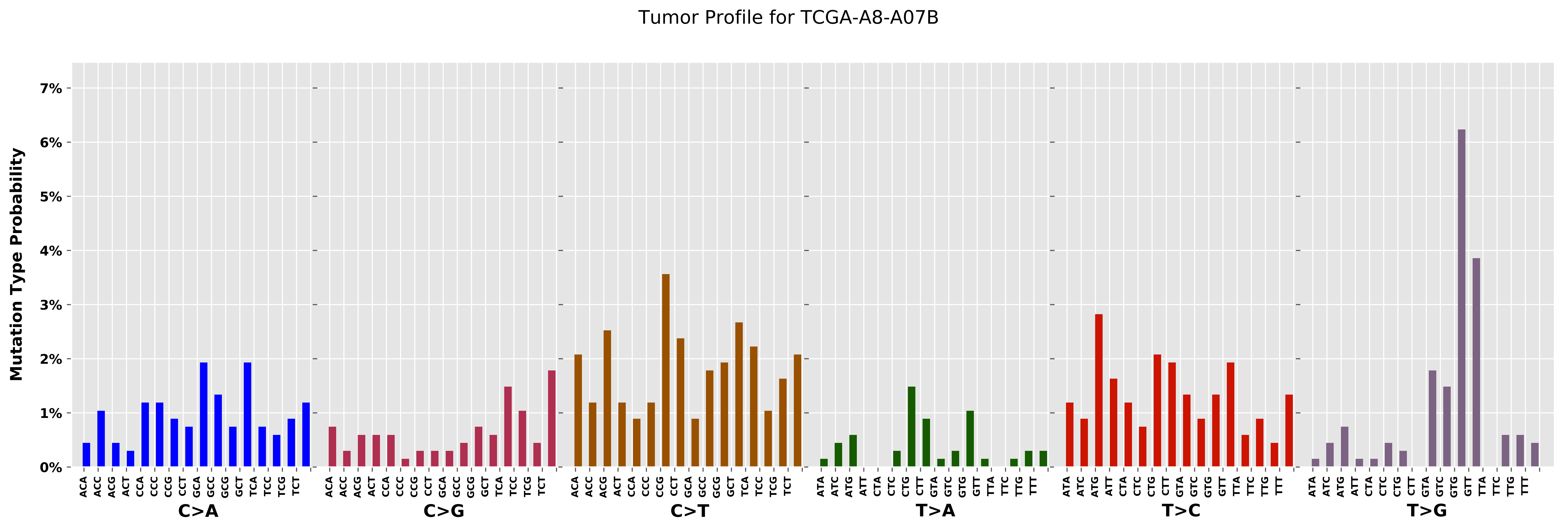

Tumor Profile for TCGA-A8-A07B
Mutation Type Probability
7%
6%
5%
4%
3%
2%
1%
0%
C>A
C>G
C>T
T>A
T>C
T>G

Cancer processes Weights for TCGA-AO-A0J6

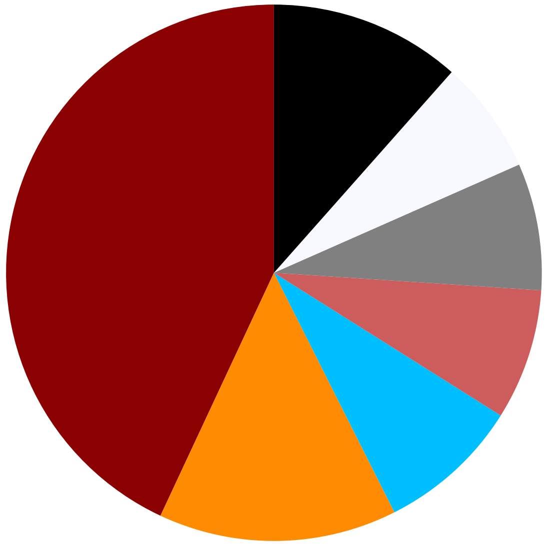

Signature 3, 43.04%
Signature 1, 14.39%
Signature 12, 8.64%
Signature 30, 7.88%
Signature 24, 7.66%
Signature 21, 6.84%
Other Signatures Below Significance Threshold, 11.55%

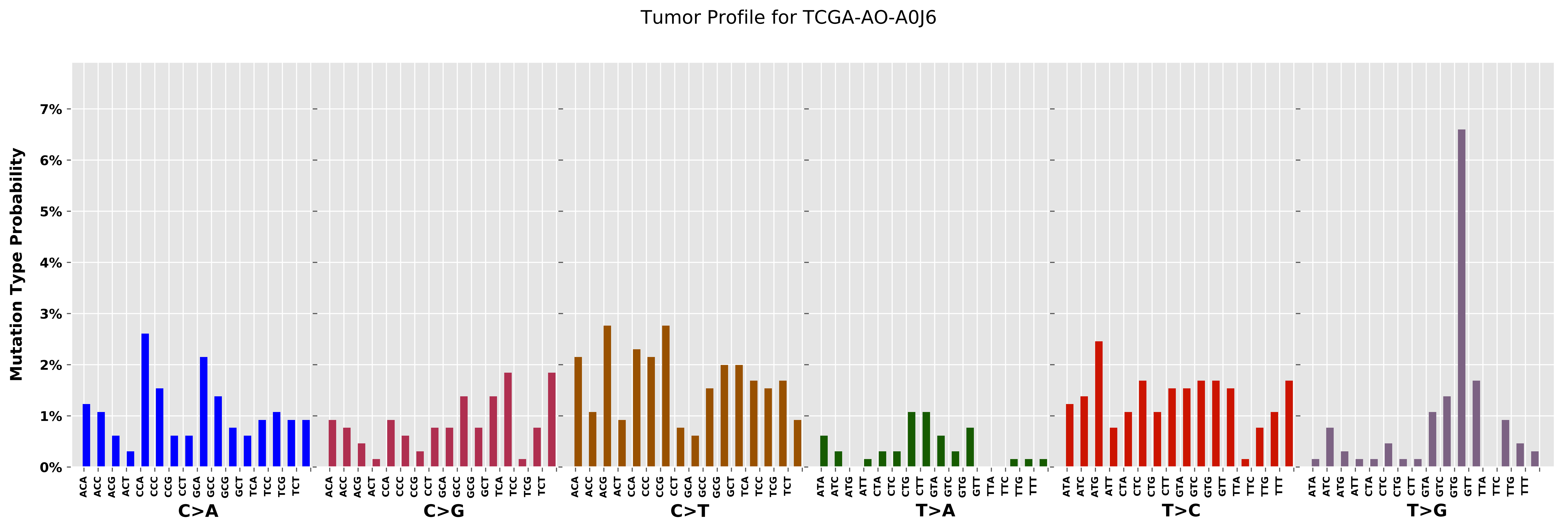
Tumor Profile for TCGA-AO-A0J6
Mutation Type Probability
0%
1%
2%
3%
4%
5%
6%
7%
C>A
C>G
C>T
T>A
T>C
T>G

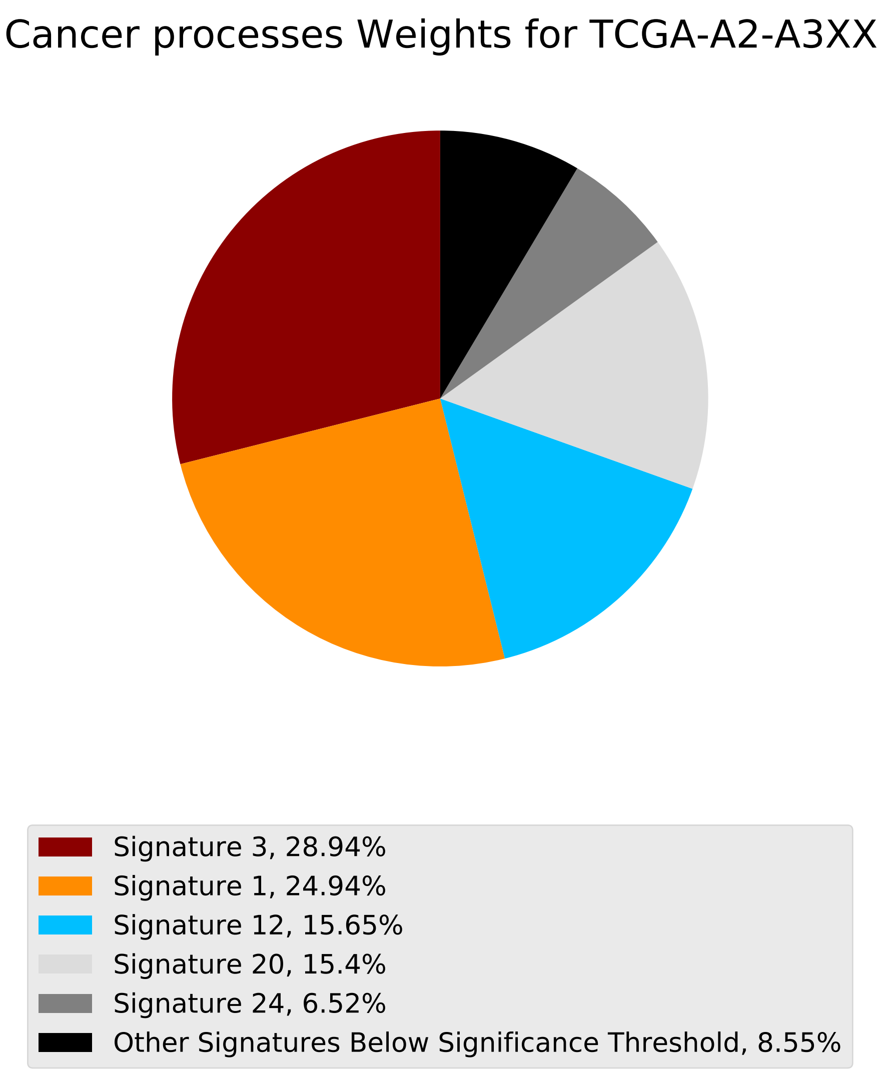
Cancer processes Weights for TCGA-A2-A3XX
Signature 3, 28.94%
Signature 1, 24.94%
Signature 12, 15.65%
Signature 20, 15.4%
Signature 24, 6.52%
Other Signatures Below Significance Threshold, 8.55%

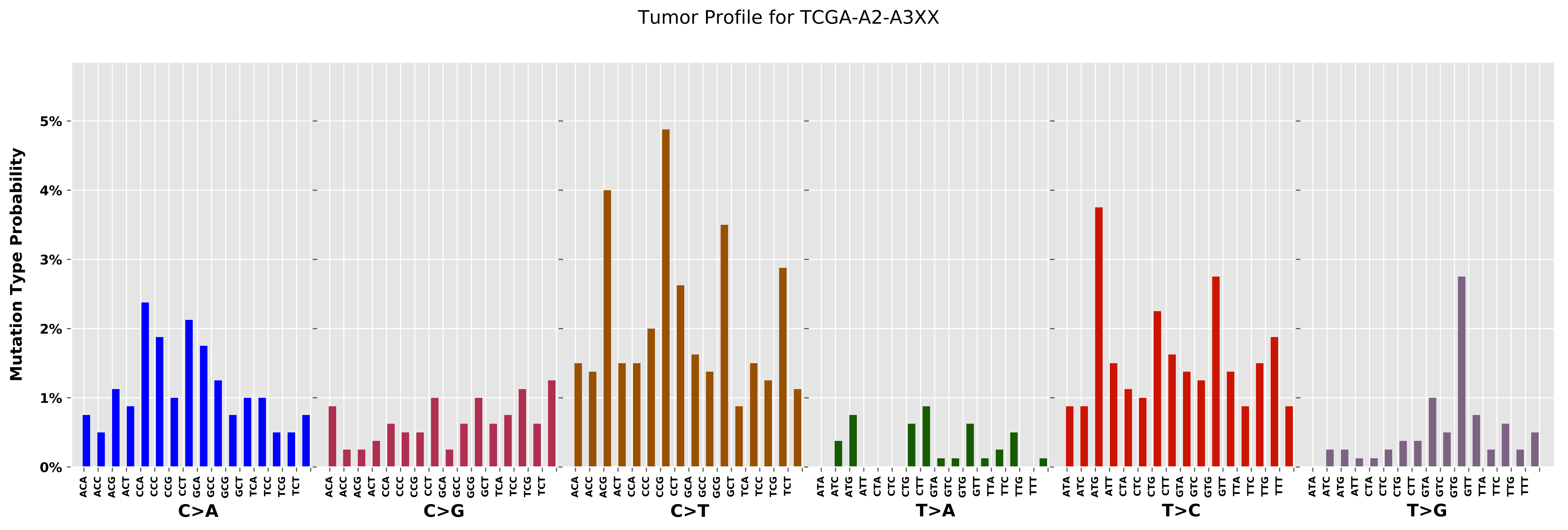

Tumor Profile for TCGA-A2-A3XX
Mutation Type Probability
0%
1%
2%
3%
4%
5%
C>A
C>G
C>T
T>A
T>C
T>G

Cancer processes Weights for TCGA-A8-A075

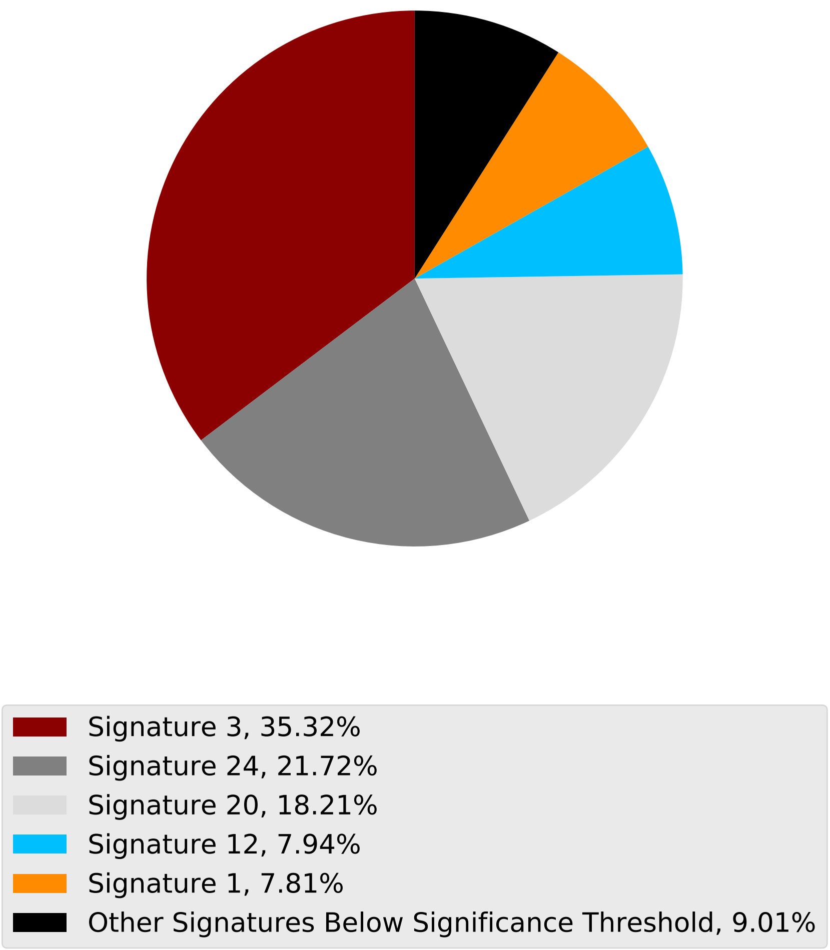

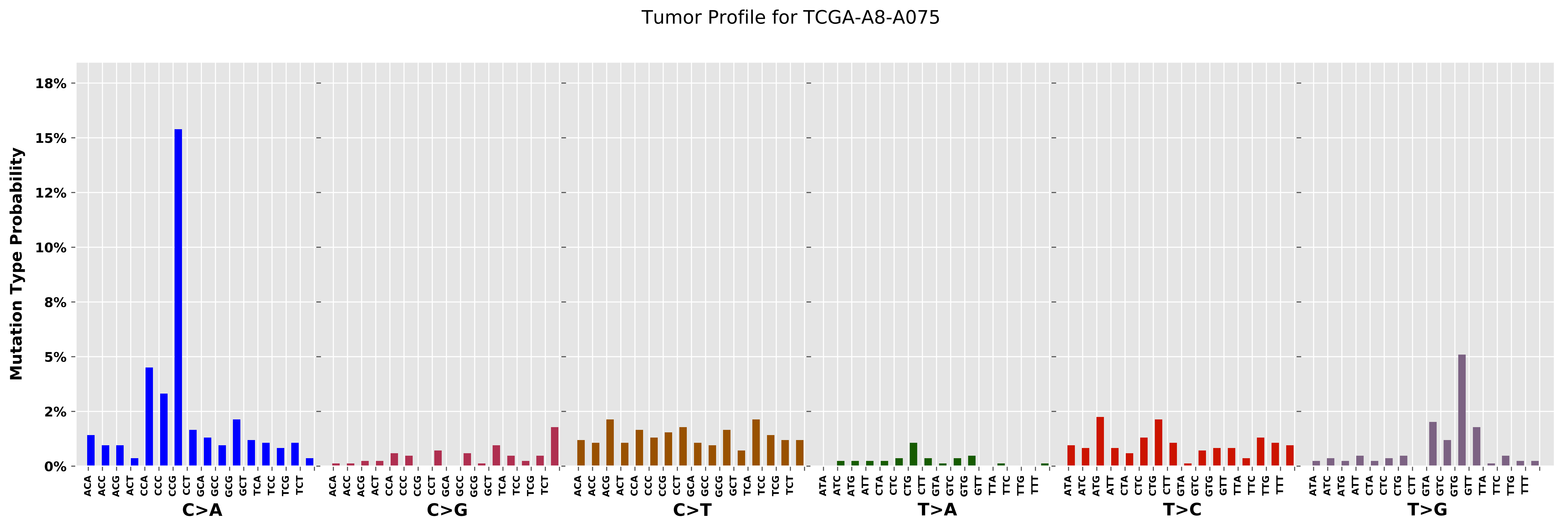

Tumor Profile for TCGA-A8-A075
Mutation Type Probability
18%
15%
12%
10%
8%
5%
2%
0%
C>A
C>G
C>T
T>A
T>C
T>G

Cancer processes Weights for TCGA-AR-A1AY

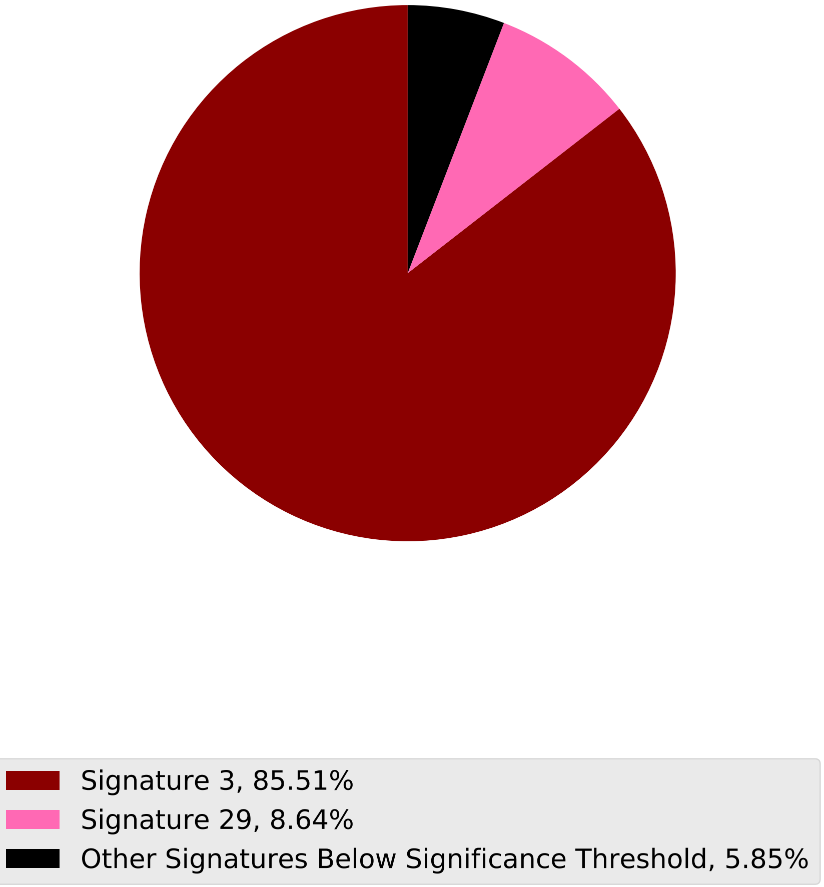

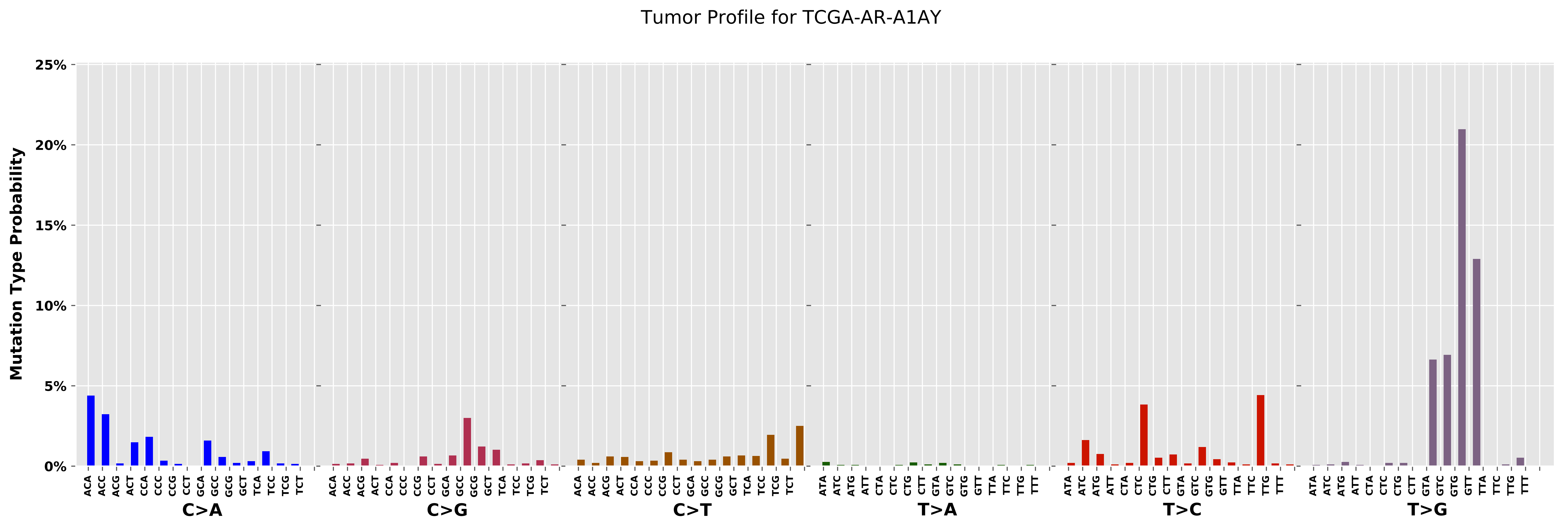

Tumor Profile for TCGA-AR-A1AY
Mutation Type Probability
25%
20%
15%
10%
5%
0%
C>A
C>G
C>T
T>A
T>C
T>G

Cancer processes Weights for TCGA-AG-3901

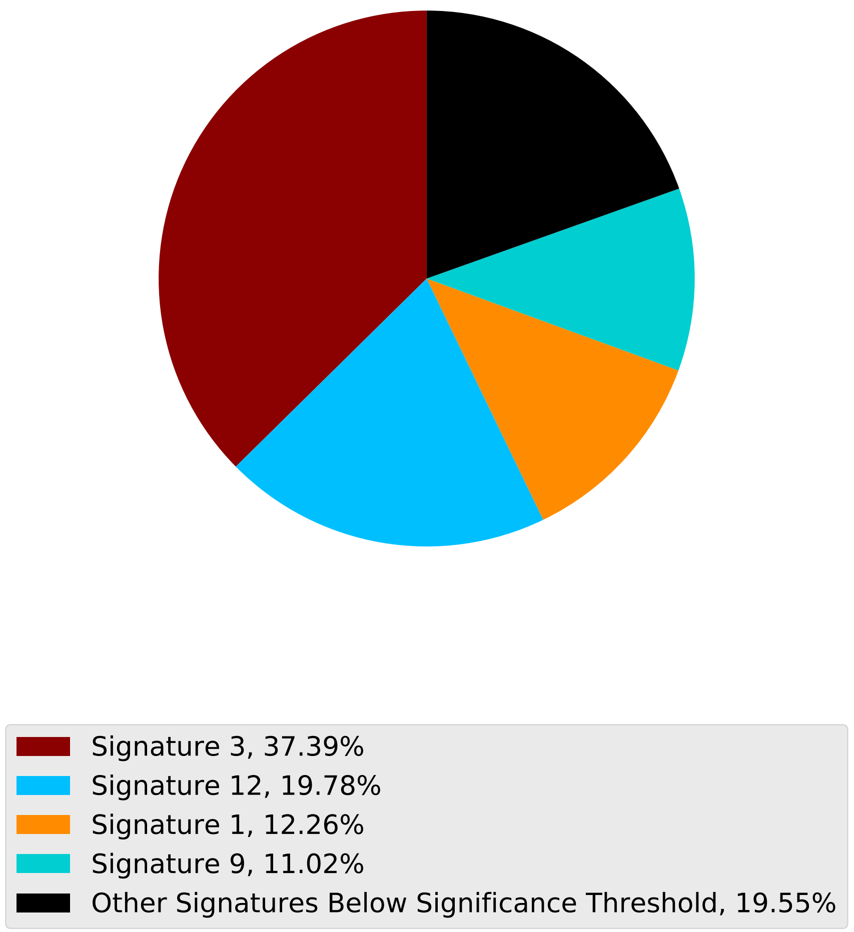

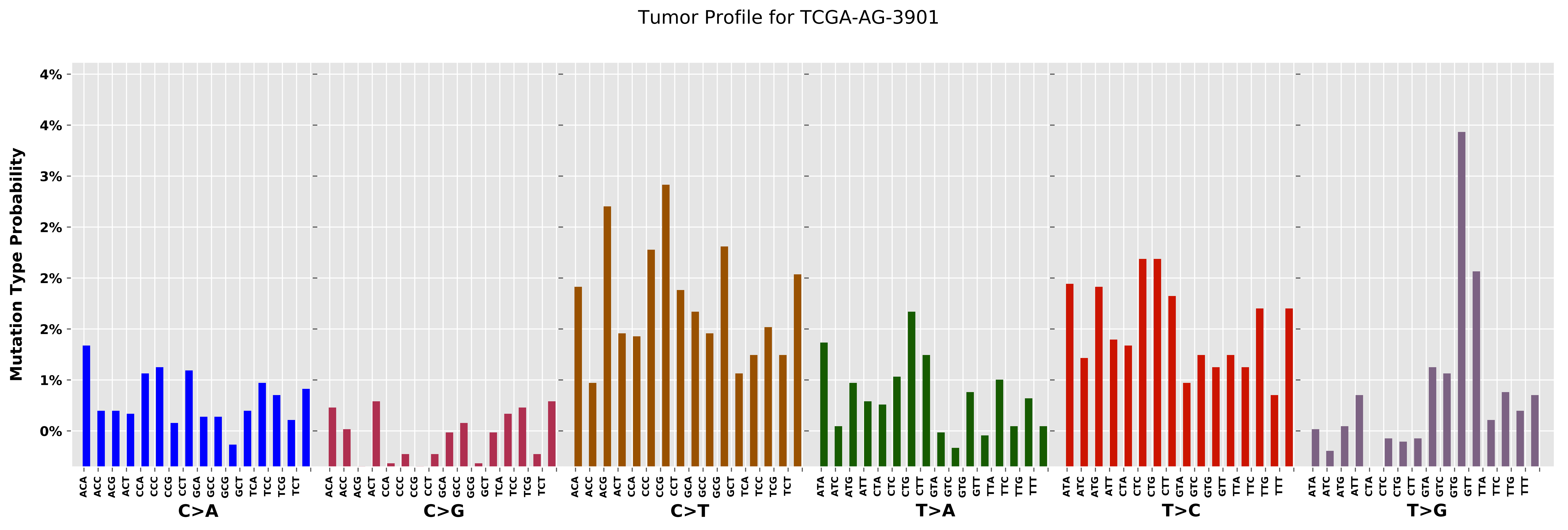

Tumor Profile for TCGA-AG-3901
Mutation Type Probability
4%
4%
3%
2%
2%
2%
1%
0%
C>A
C>G
C>T
T>A
T>C
T>G

Cancer processes Weights for TCGA-E2-A152

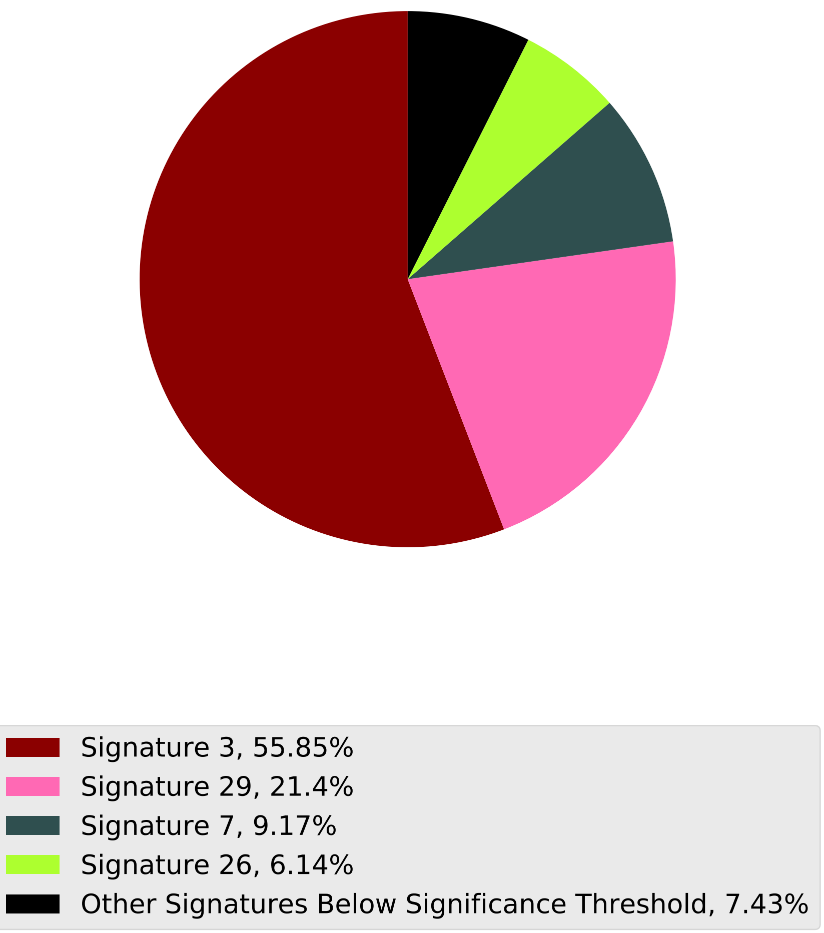

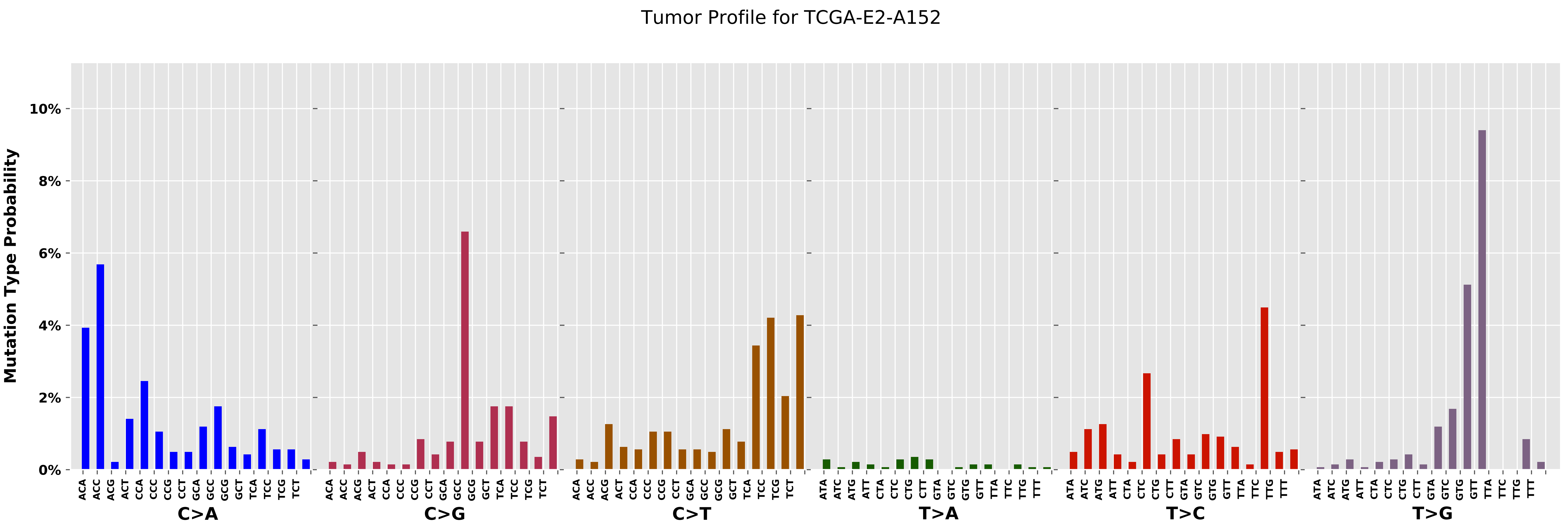

Tumor Profile for TCGA-E2-A152
Mutation Type Probability
0%
2%
4%
6%
8%
10%
ACA ACC ACG ACT CCA CCC CCG CCT GCA GCC GCG GCT TCA TCC TCG TCT
C>A
ACA ACC ACG ACT CCA CCC CCG CCT GCA GCC GCG GCT TCA TCC TCG TCT
C>G
ACA ACC ACG ACT CCA CCC CCG CCT GCA GCC GCG GCT TCA TCC TCG TCT
C>T
ATA ATC ATG ATT CTA CTC CTG CTT GTA GTC GTG GTT TTA TTC TTG TTT
T>A
ATA ATC ATG ATT CTA CTC CTG CTT GTA GTC GTG GTT TTA TTC TTG TTT
T>C
ATA ATC ATG ATT CTA CTC CTG CTT GTA GTC GTG GTT TTA TTC TTG TTT
T>G

Cancer processes Weights for TCGA-AG-4015

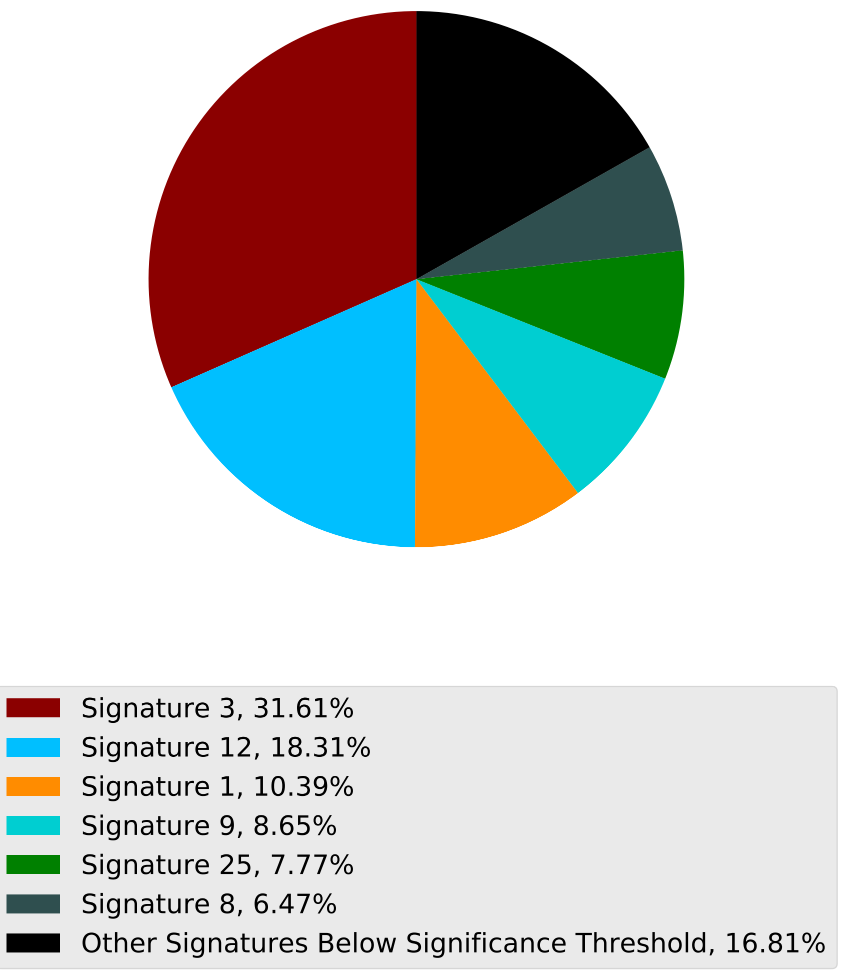

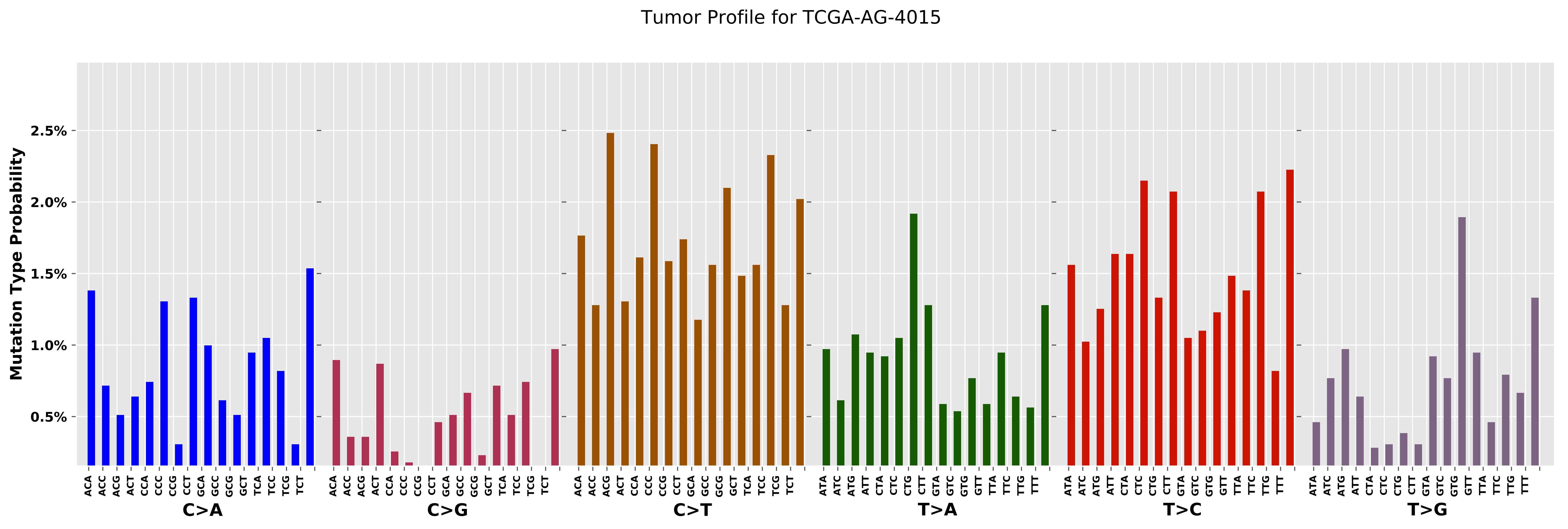

Tumor Profile for TCGA-AG-4015
Mutation Type Probability
2.5%
2.0%
1.5%
1.0%
0.5%
C>A
C>G
C>T
T>A
T>C
T>G

Cancer processes Weights for TCGA-BH-A0B9

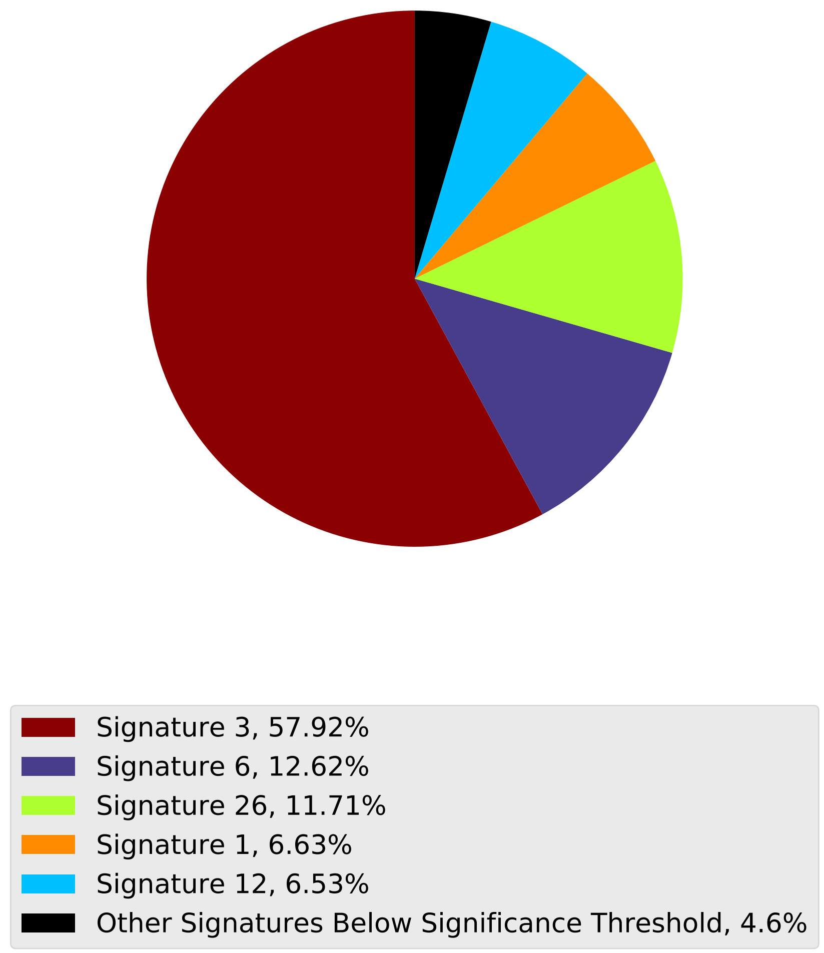

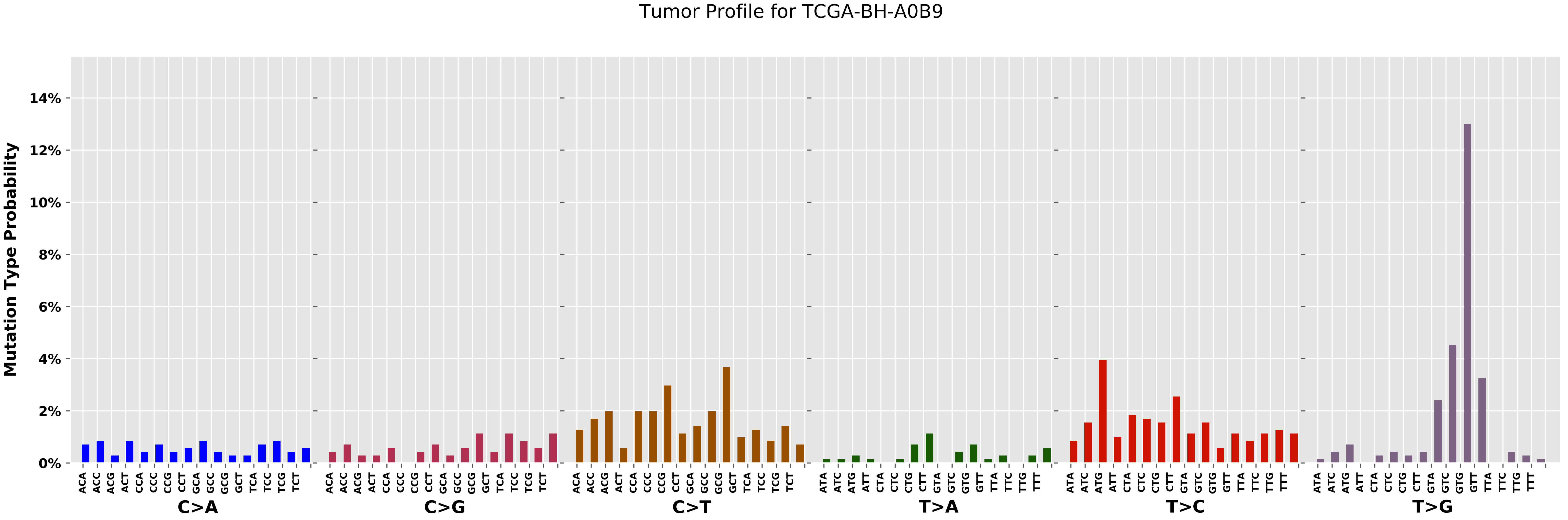
Tumor Profile for TCGA-BH-A0B9
Mutation Type Probability
0%
2%
4%
6%
8%
10%
12%
14%
C>A
C>G
C>T
T>A
T>C
T>G
ACA
ACC
ACG
ACT
CCA
CCC
CCG
CCT
GCA
GCC
GCG
GCT
TCA
TCC
TCG
TCT
ATA
ATC
ATG
ATT
CTA
CTC
CTG
CTT
GTA
GTC
GTG
GTT
TTA
TTC
TTG
TTT

Cancer processes Weights for TCGA-A2-A0D1

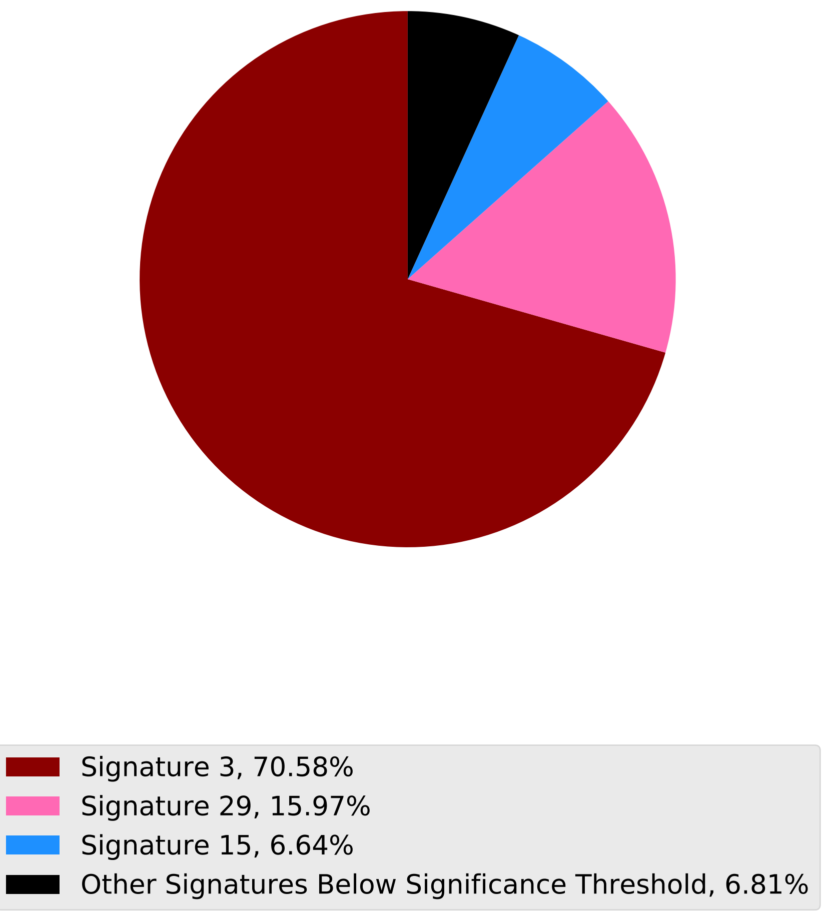

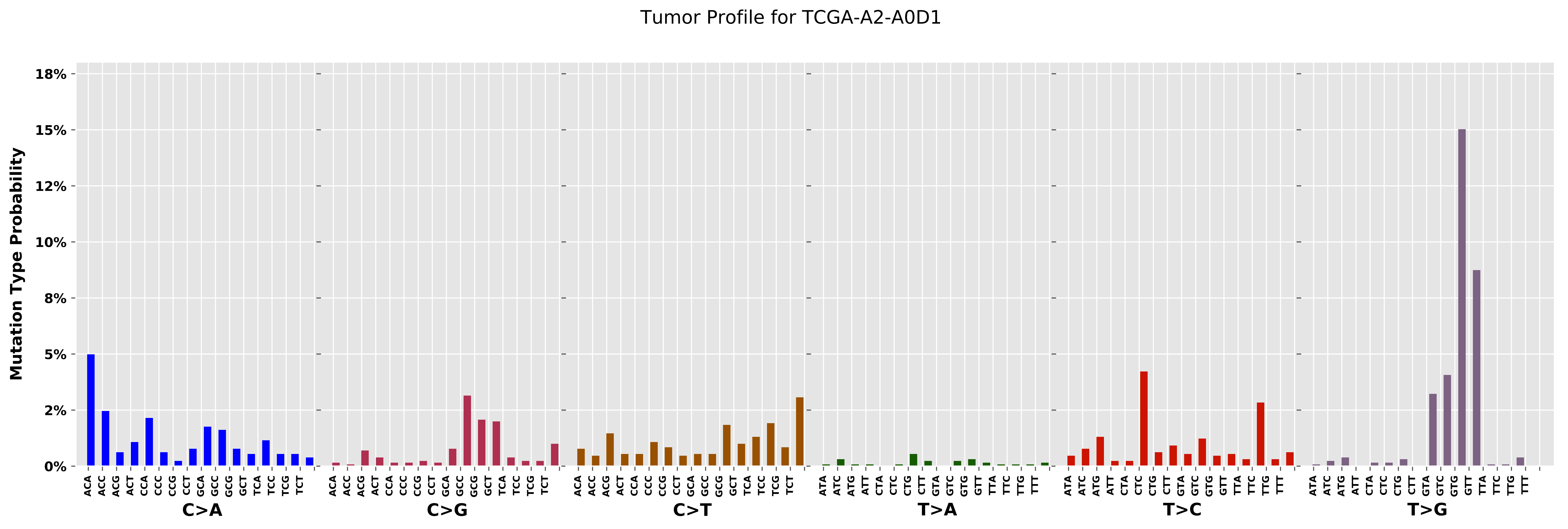
Tumor Profile for TCGA-A2-A0D1
Mutation Type Probability
18%
15%
12%
10%
8%
5%
2%
0%
C>A
C>G
C>T
T>A
T>C
T>G

Cancer processes Weights for TCGA-AA-3529

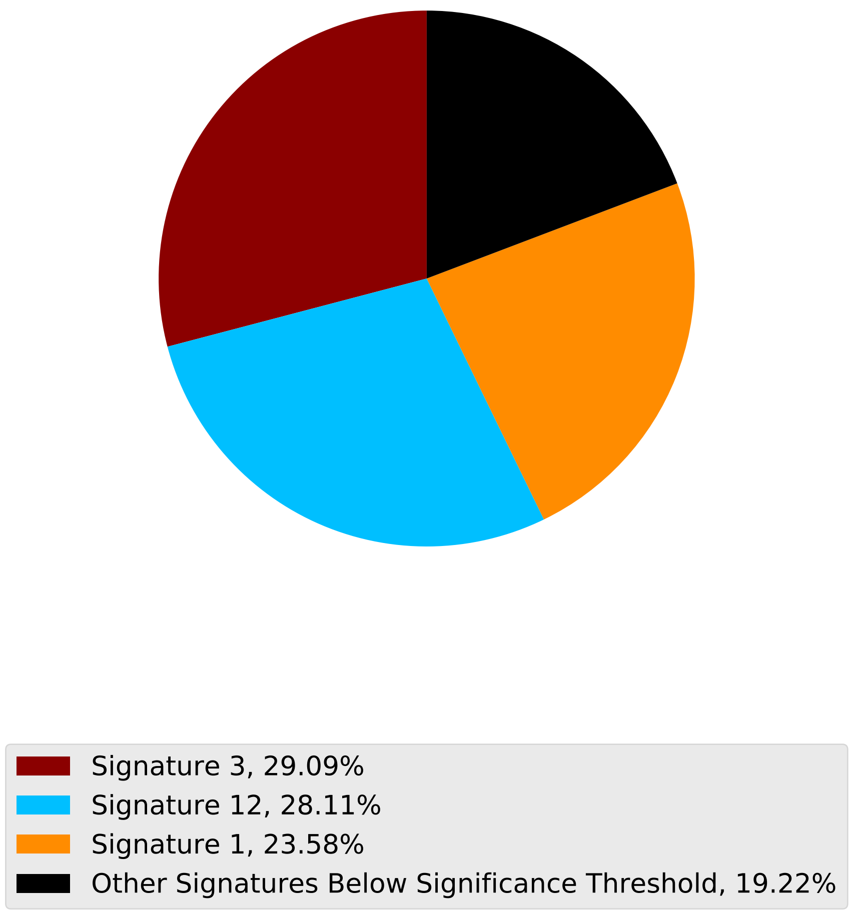

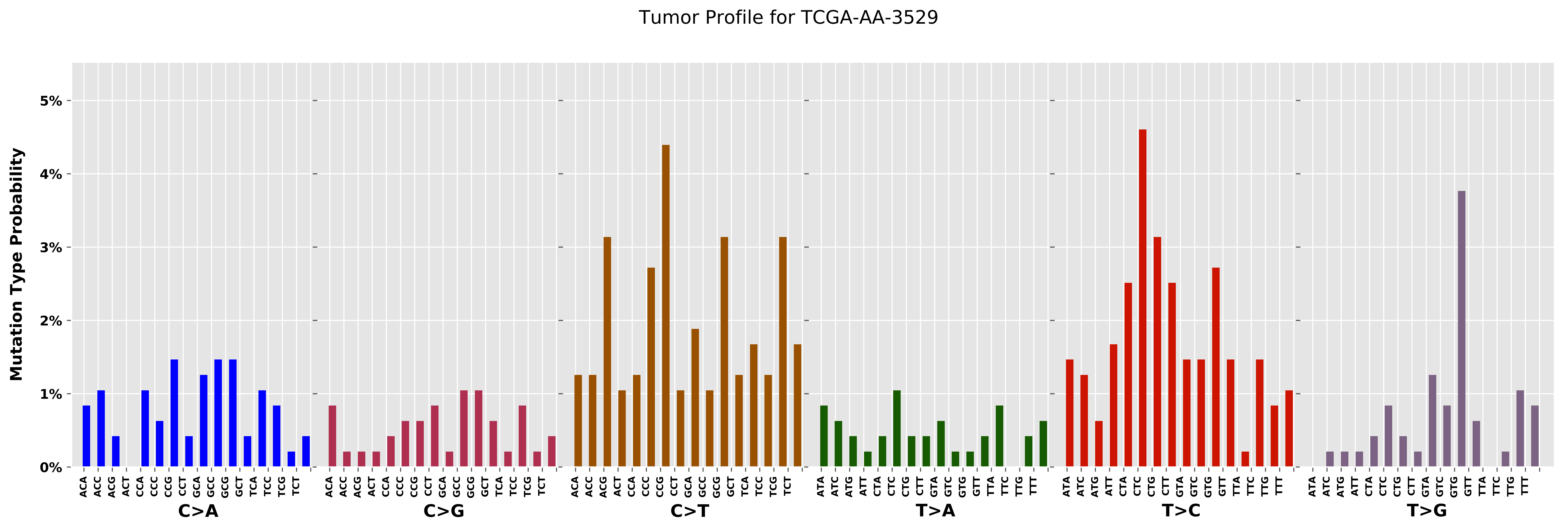
Tumor Profile for TCGA-AA-3529
Mutation Type Probability
0%
1%
2%
3%
4%
5%
ACA ACC ACG ACT CCA CCC CCG CCT GCA GCC GCG GCT TCA TCC TCG TCT
ATA ATC ATG ATT CTA CTC CTG CTT GTA GTC GTG GTT TTA TTC TTG TTT
C>A
C>G
C>T
T>A
T>C
T>G

Cancer processes Weights for TCGA-A6-3807

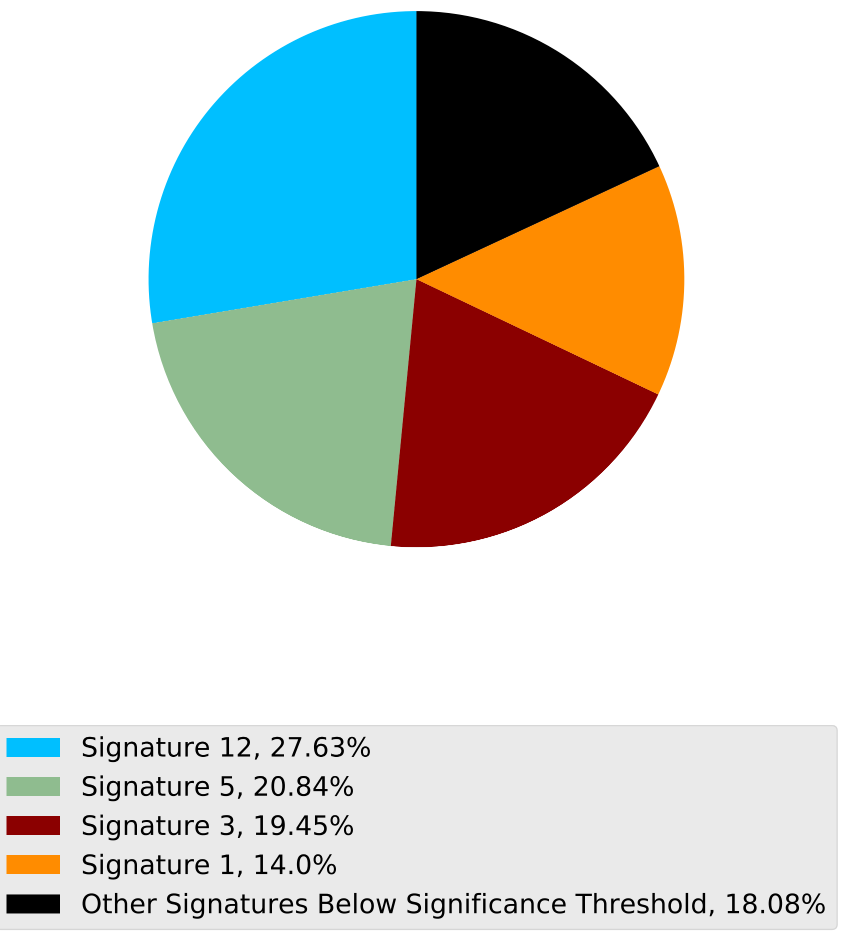

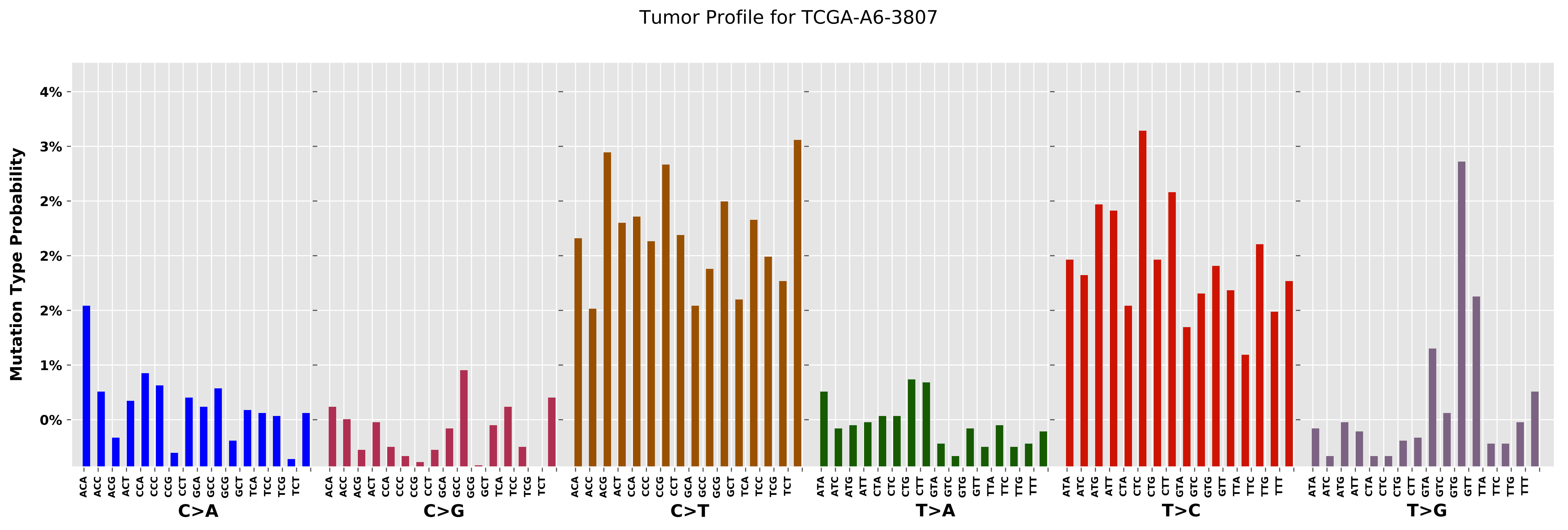
Tumor Profile for TCGA-A6-3807
Mutation Type Probability
0%
1%
2%
2%
2%
3%
4%
C>A
C>G
C>T
T>A
T>C
T>G
ACA ACC ACG ACT CCA CCC CCG CCT GCA GCC GCG GCT TCA TCC TCG TCT
ATA ATC ATG ATT CTA CTC CTG CTT GTA GTC GTG GTT TTA TTC TTG TTT

Cancer processes Weights for TCGA-A2-A0YG

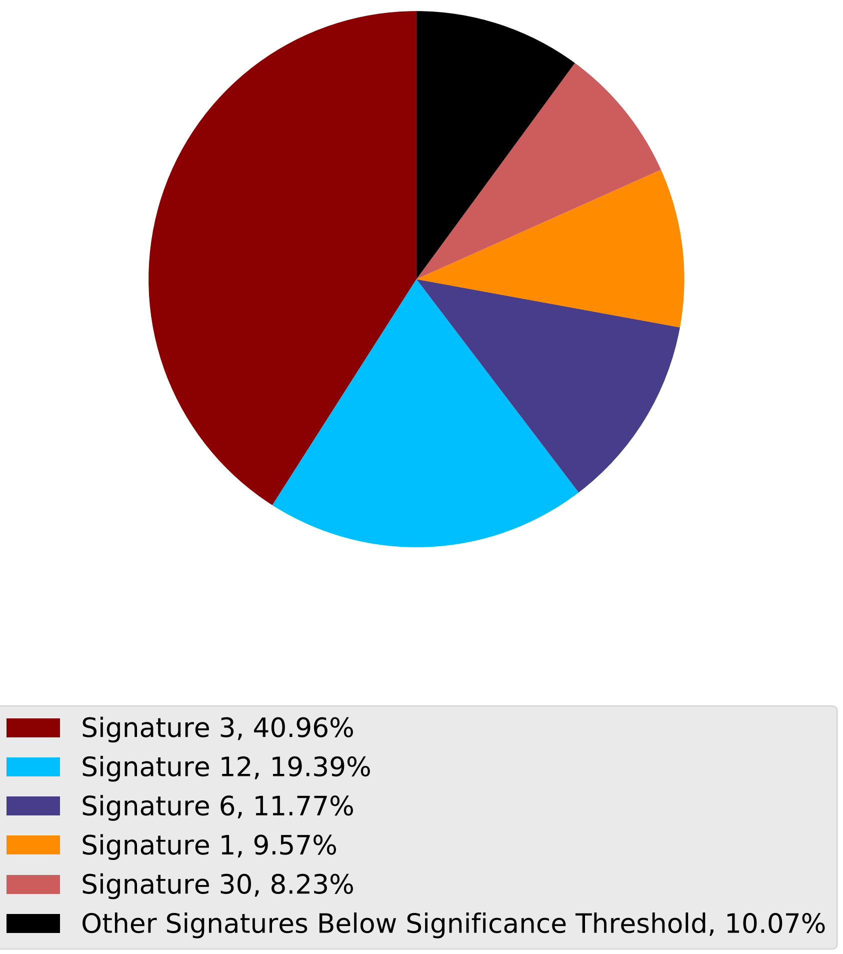

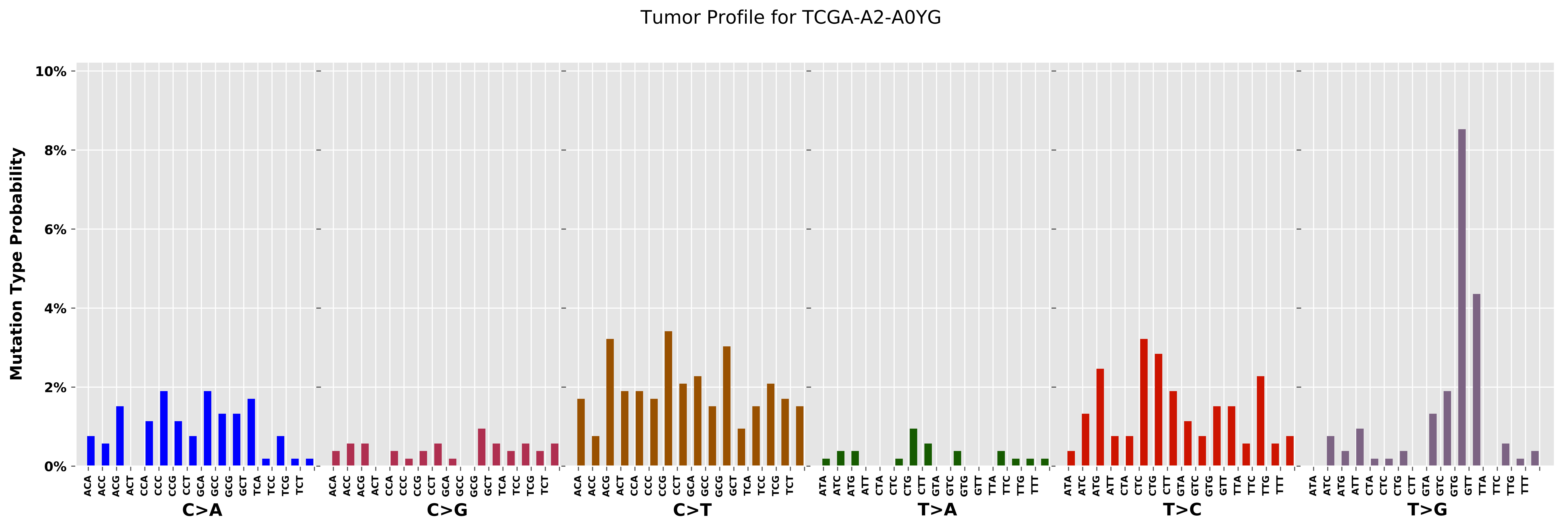

Tumor Profile for TCGA-A2-A0YG
Mutation Type Probability
10%
8%
6%
4%
2%
0%
ACA ACC ACG ACT CCA CCC CCG CCT GCA GCC GCG GCT TCA TCC TCG TCT
C>A
C>G
C>T
ATA ATC ATG ATT CTA CTC CTG CTT GTA GTC GTG GTT TTA TTC TTG TTT
T>A
T>C
T>G

Cancer processes Weights for TCGA-C8-A12Q

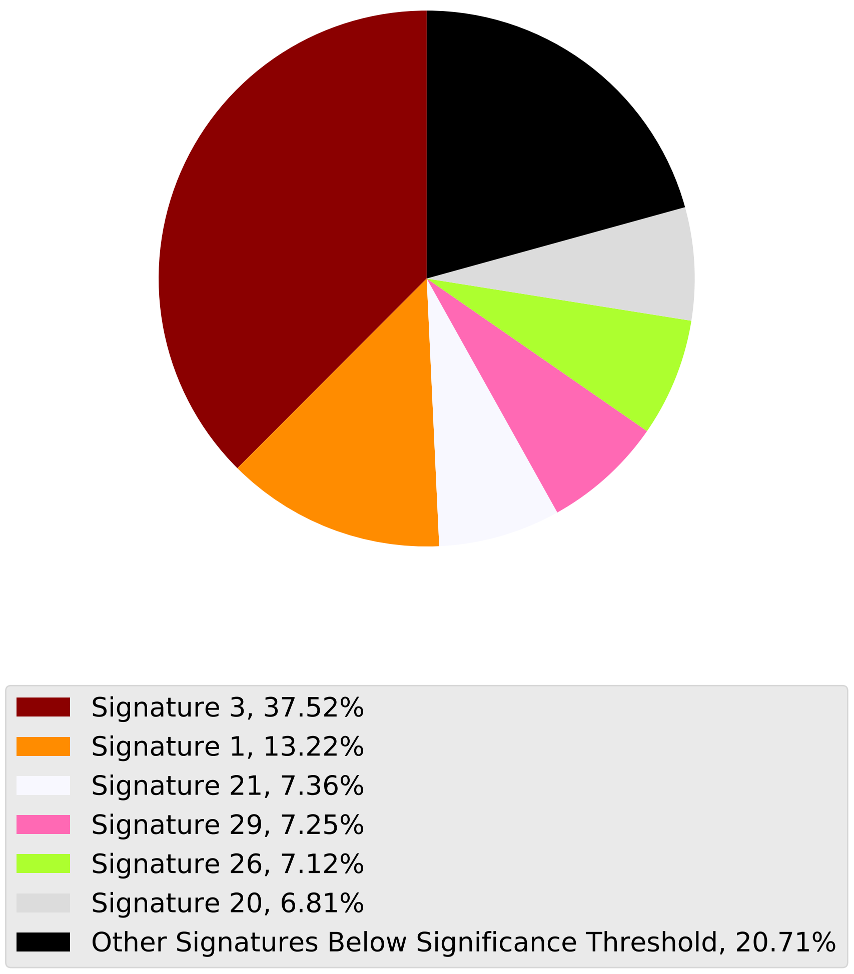

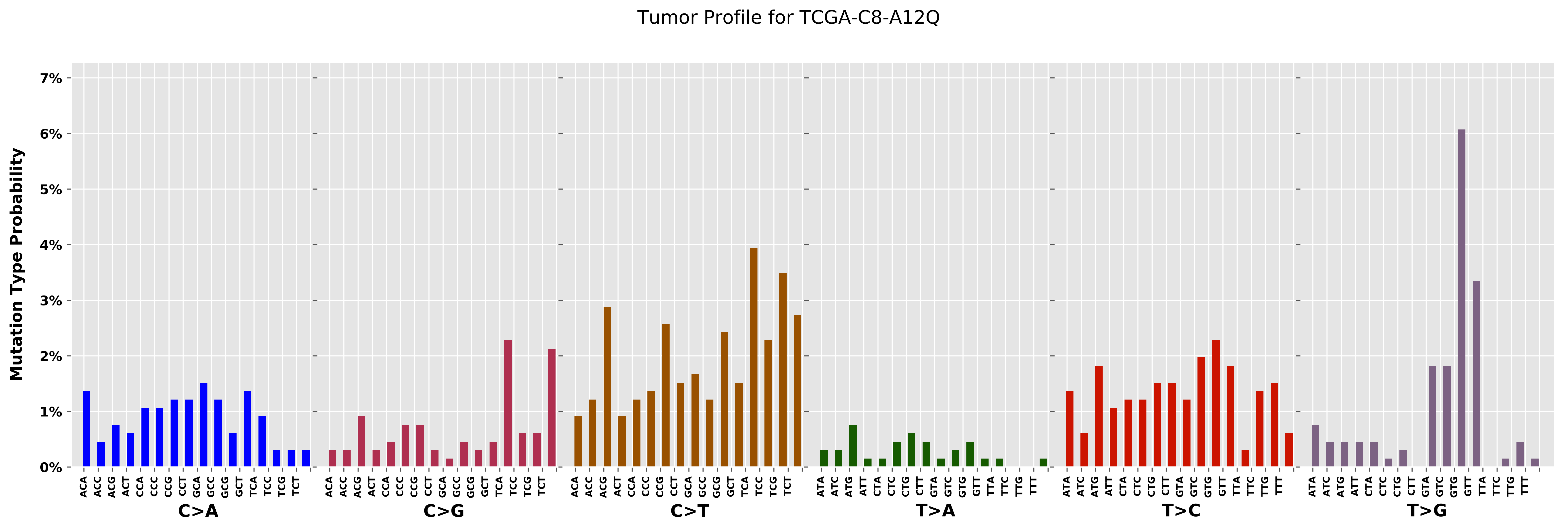

Tumor Profile for TCGA-C8-A12Q
Mutation Type Probability
0%
1%
2%
3%
4%
5%
6%
7%
C>A
C>G
C>T
T>A
T>C
T>G
ACA ACC ACG ACT CCA CCC CCG CCT GCA GCC GCG GCT TCA TCC TCG TCT
ATA ATC ATG ATT CTA CTC CTG CTT GTA GTC GTG GTT TTA TTC TTG TTT

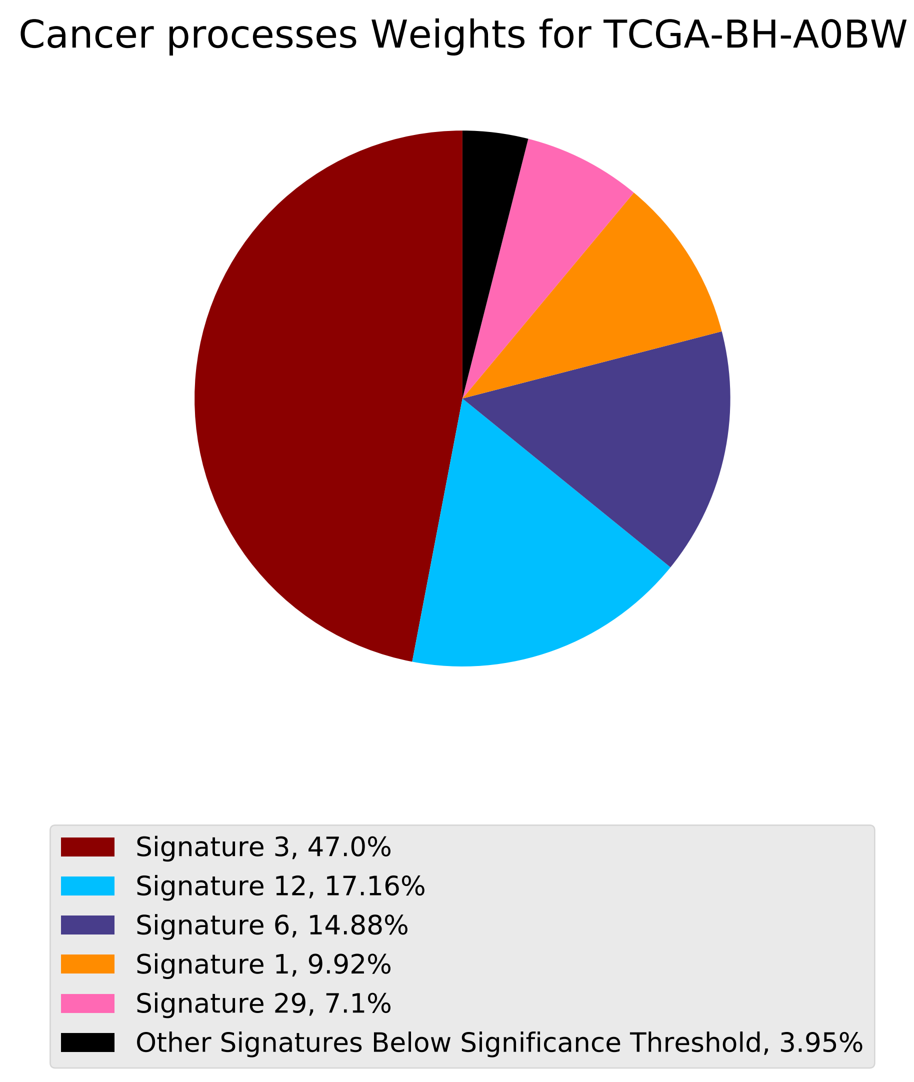
Cancer processes Weights for TCGA-BH-A0BW
Signature 3, 47.0%
Signature 12, 17.16%
Signature 6, 14.88%
Signature 1, 9.92%
Signature 29, 7.1%
Other Signatures Below Significance Threshold, 3.95%

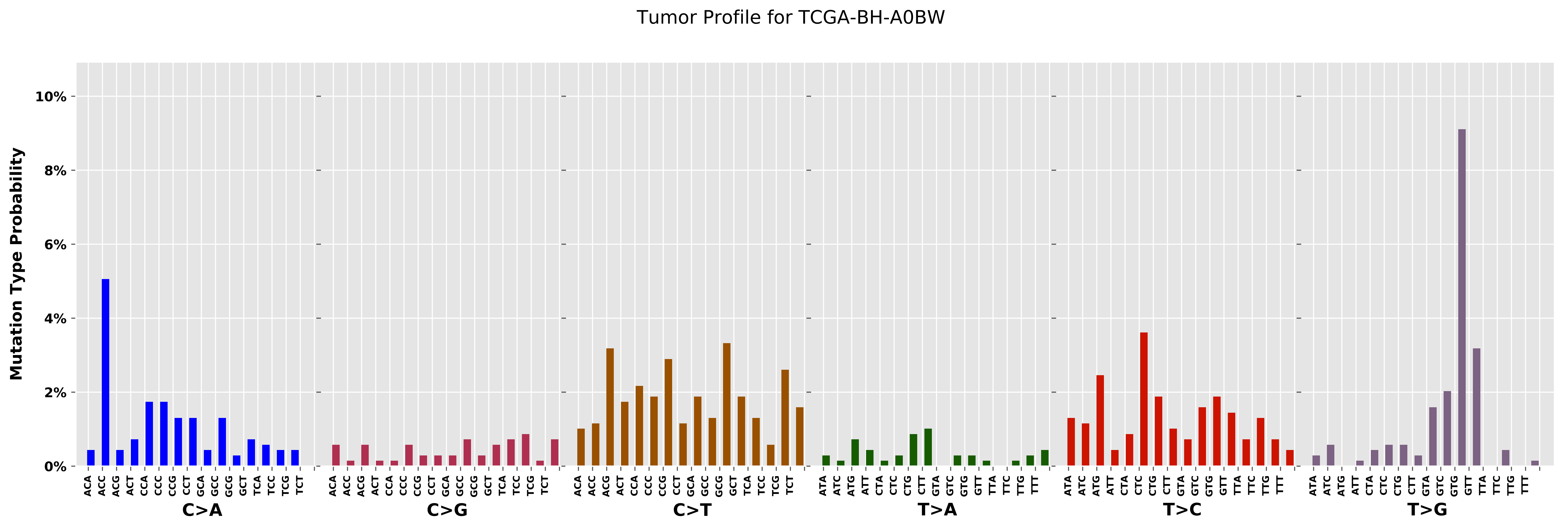

Tumor Profile for TCGA-BH-A0BW
Mutation Type Probability
10%
8%
6%
4%
2%
0%
C>A
C>G
C>T
T>A
T>C
T>G

Cancer processes Weights for TCGA-A2-A0EY

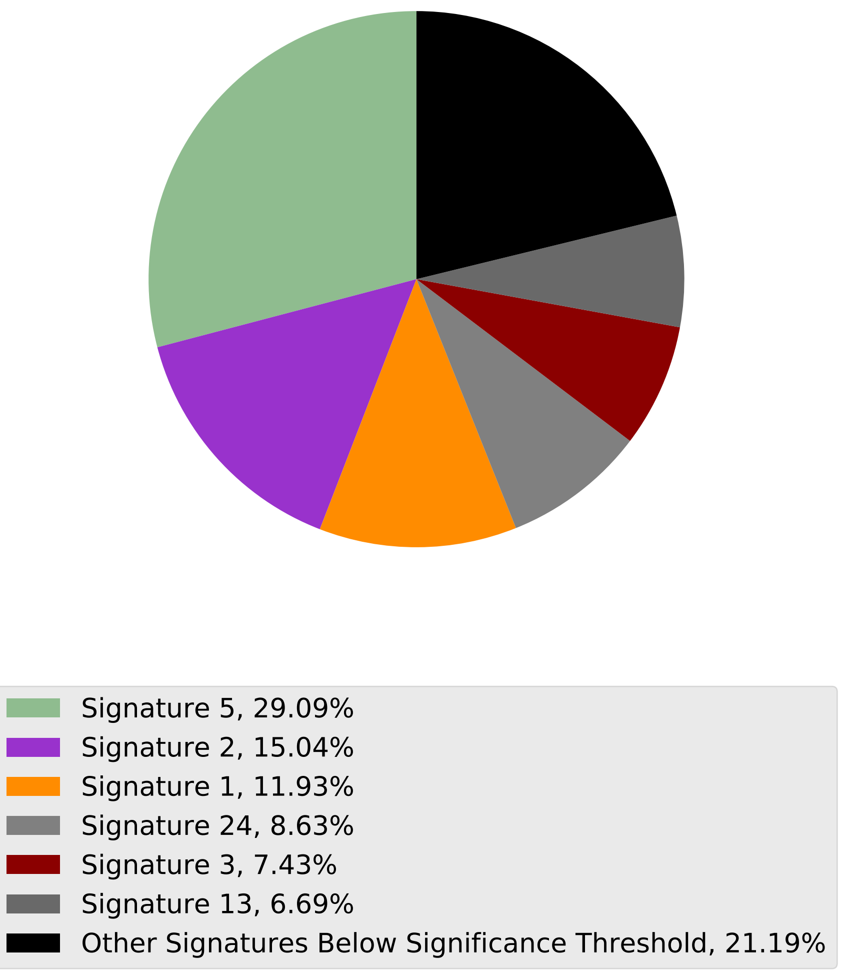

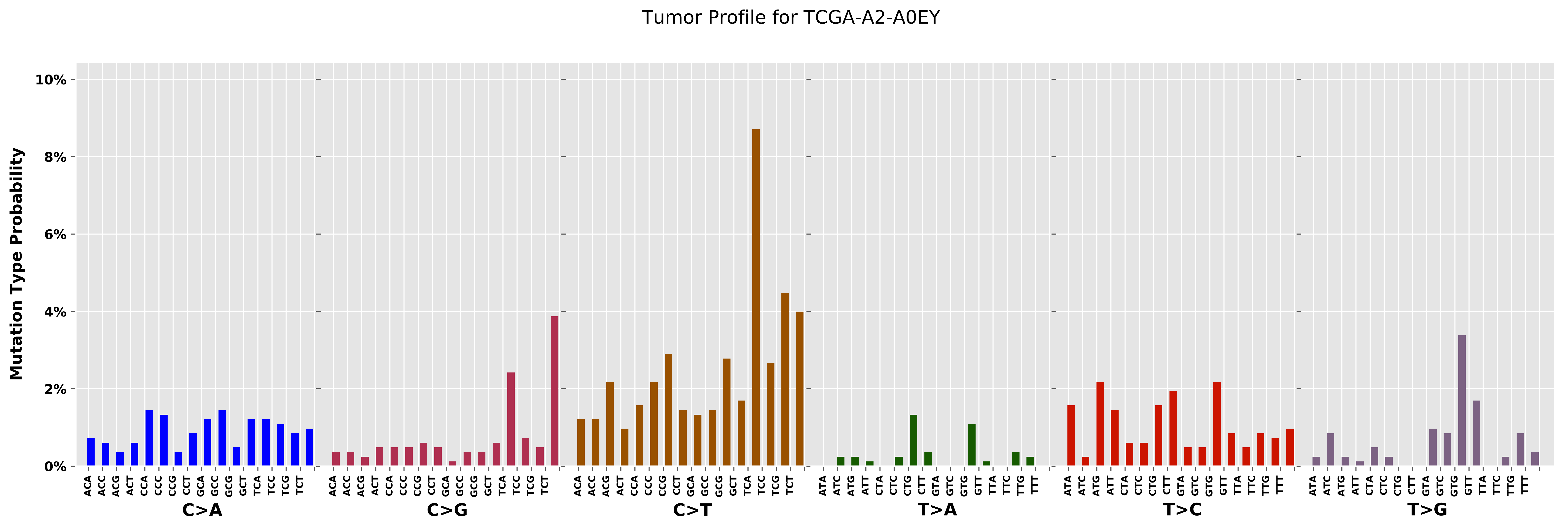

Tumor Profile for TCGA-A2-A0EY
Mutation Type Probability
0%
2%
4%
6%
8%
10%
C>A
C>G
C>T
T>A
T>C
T>G

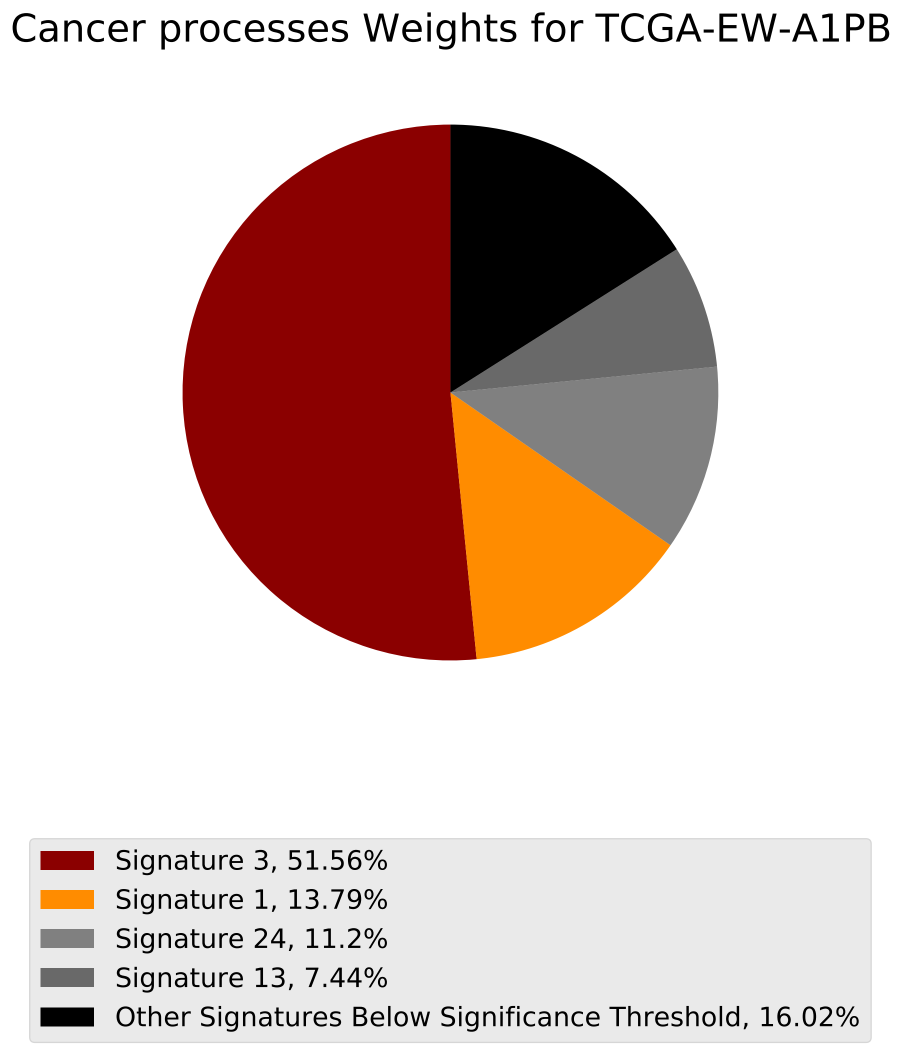
Cancer processes Weights for TCGA-EW-A1PB
Signature 3, 51.56%
Signature 1, 13.79%
Signature 24, 11.2%
Signature 13, 7.44%
Other Signatures Below Significance Threshold, 16.02%

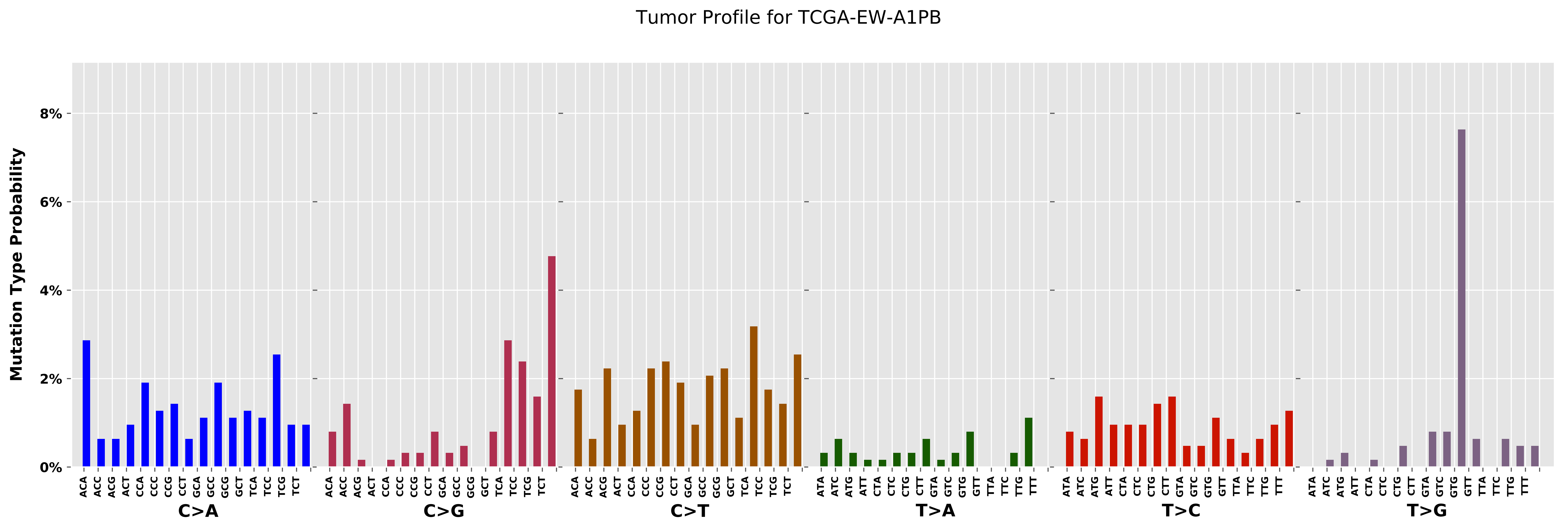

Tumor Profile for TCGA-EW-A1PB
Mutation Type Probability
0%
2%
4%
6%
8%
C>A
C>G
C>T
T>A
T>C
T>G

Cancer processes Weights for TCGA-AD-6964

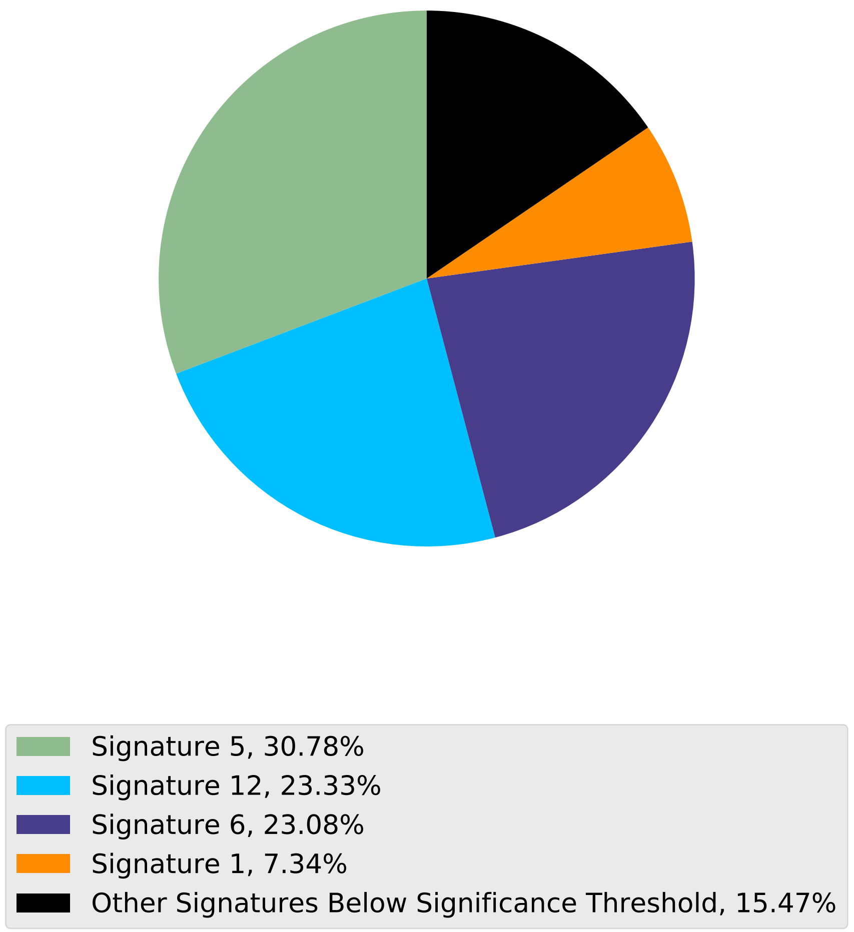

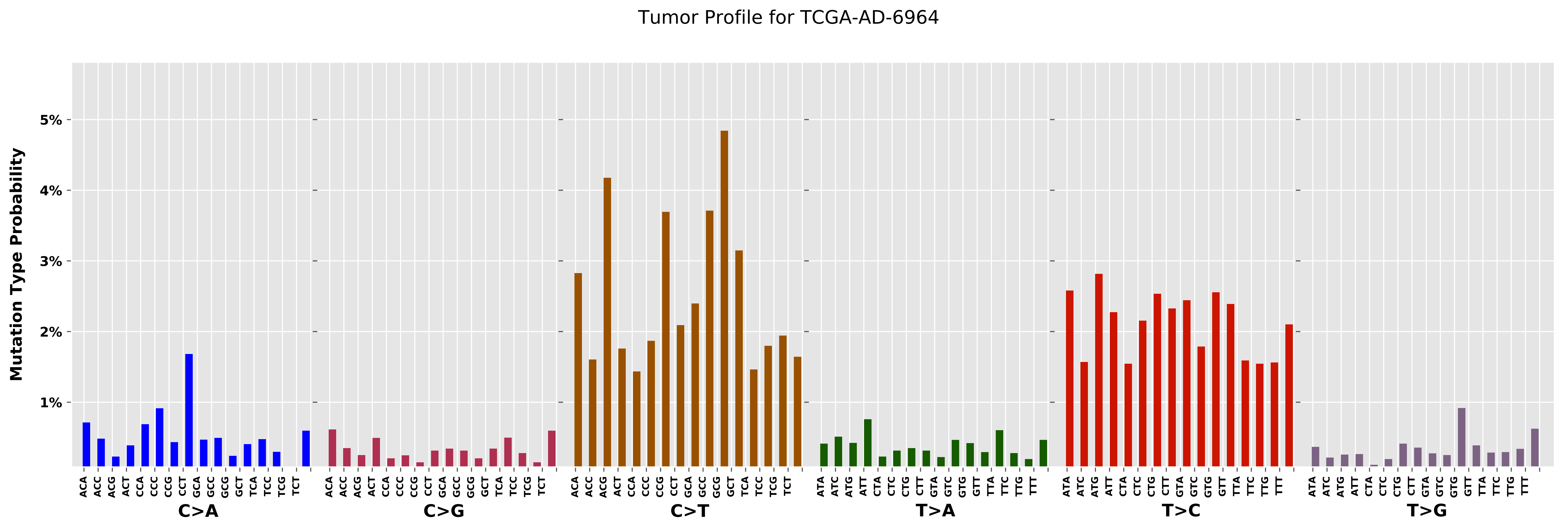
Tumor Profile for TCGA-AD-6964
Mutation Type Probability
5%
4%
3%
2%
1%
C>A
C>G
C>T
T>A
T>C
T>G
ACA
ACC
ACG
ACT
CCA
CCC
CCG
CCT
GCA
GCC
GCG
GCT
TCA
TCC
TCG
TCT
ATA
ATC
ATG
ATT
CTA
CTC
CTG
CTT
GTA
GTC
GTG
GTT
TTA
TTC
TTG
TTT

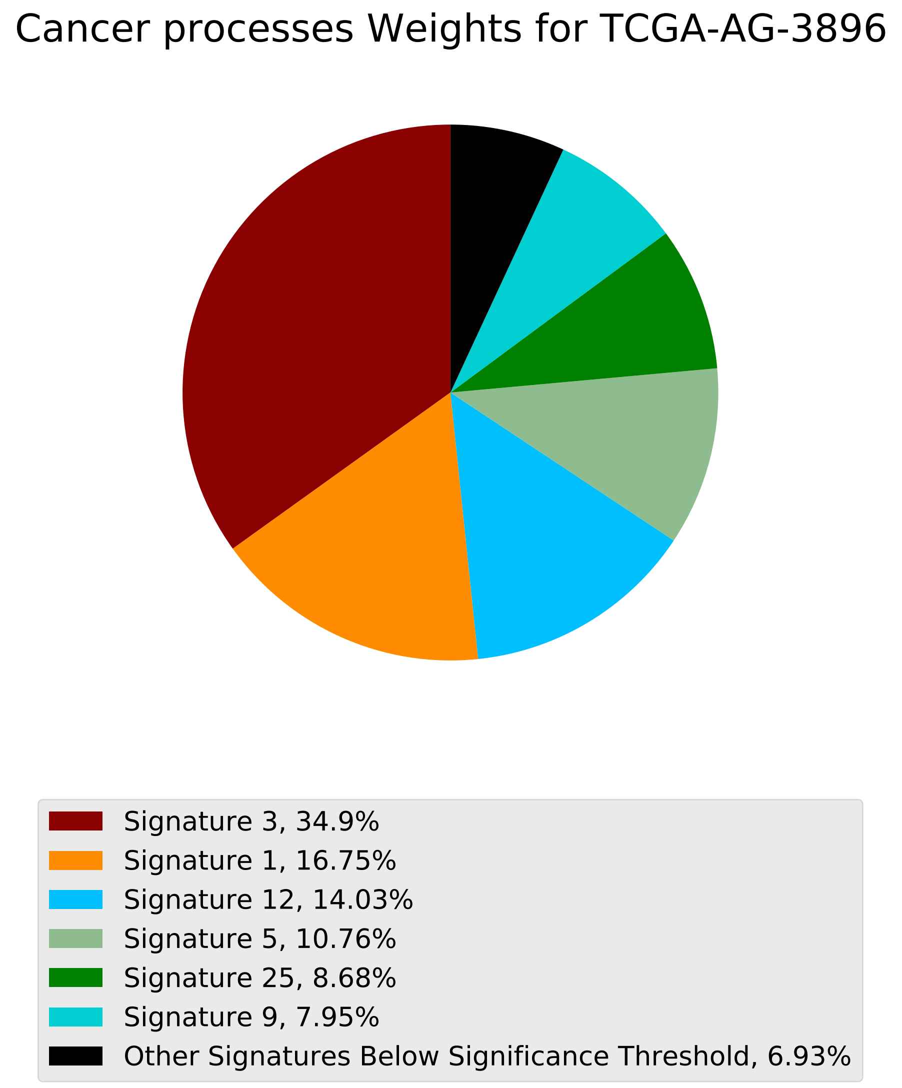
Cancer processes Weights for TCGA-AG-3896
Signature 3, 34.9%
Signature 1, 16.75%
Signature 12, 14.03%
Signature 5, 10.76%
Signature 25, 8.68%
Signature 9, 7.95%
Other Signatures Below Significance Threshold, 6.93%

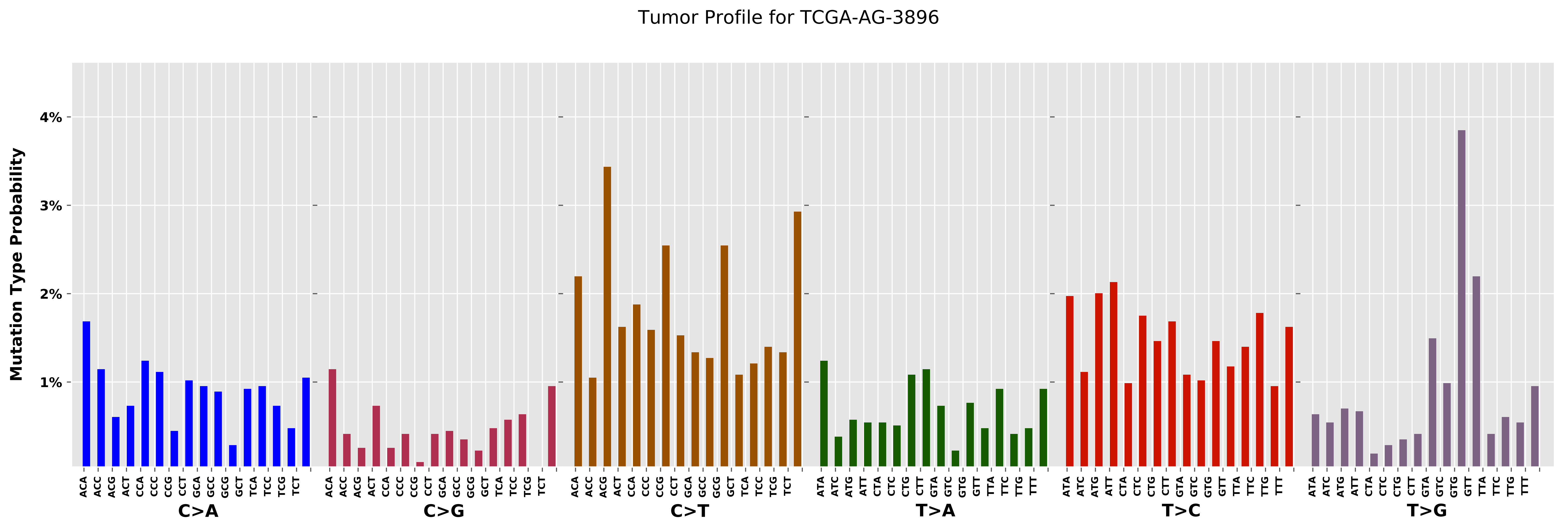

Tumor Profile for TCGA-AG-3896
Mutation Type Probability
4%
3%
2%
1%
C>A
C>G
C>T
T>A
T>C
T>G

Cancer processes Weights for TCGA-E2-A15K

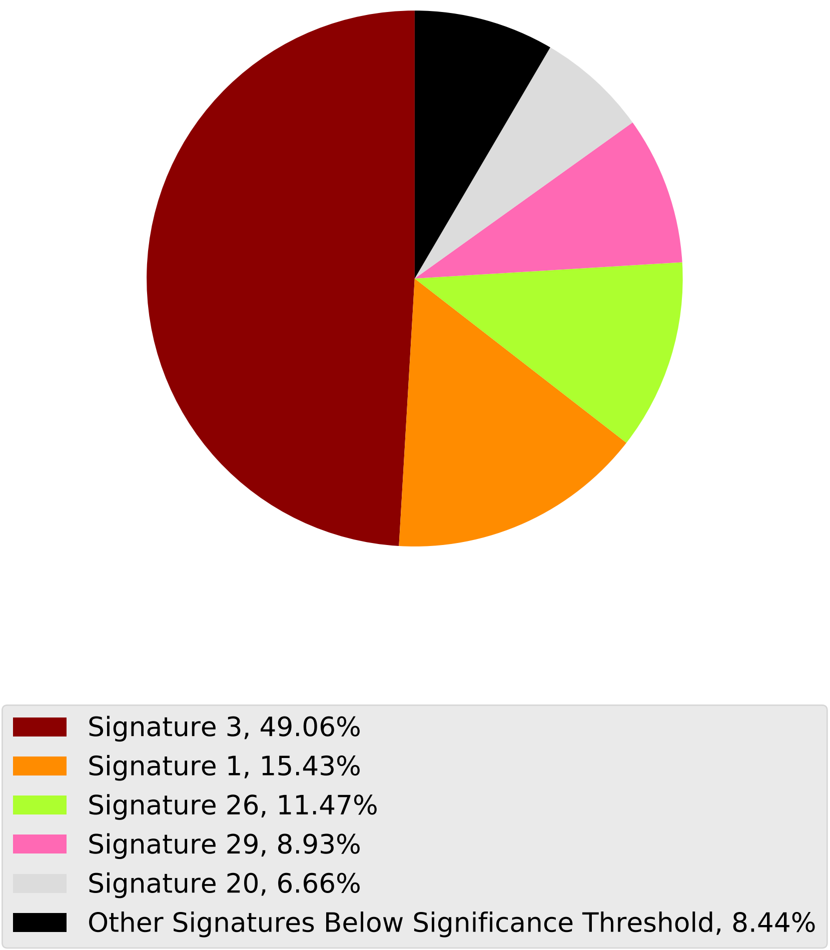

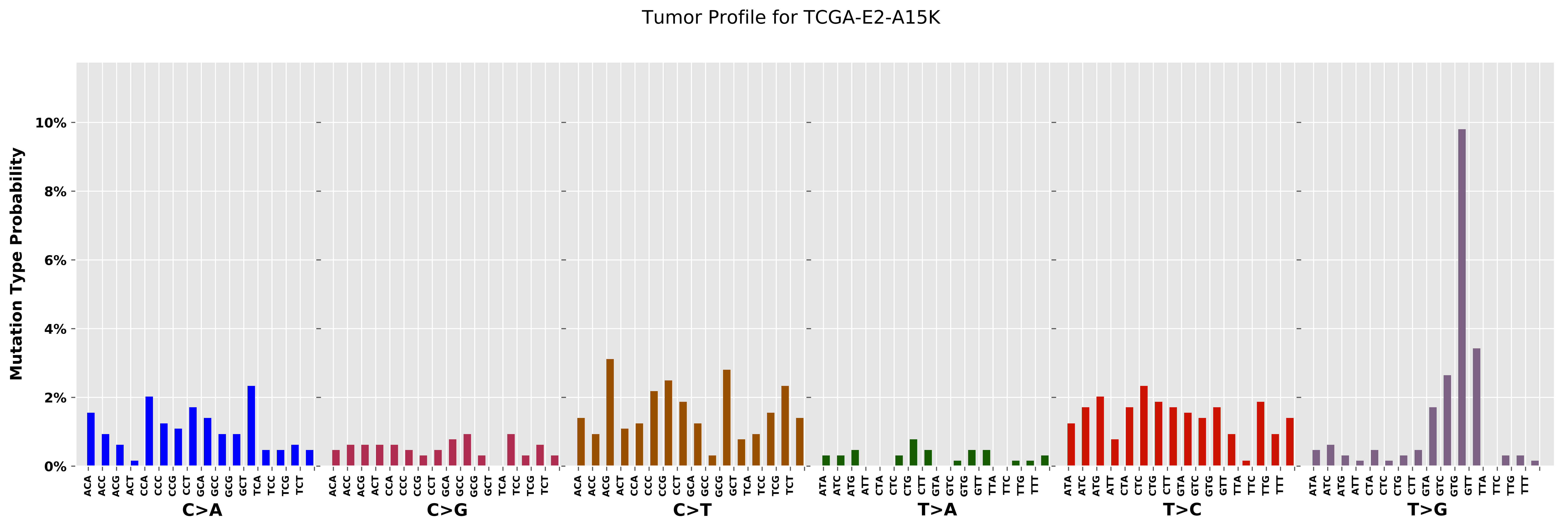

Tumor Profile for TCGA-E2-A15K
Mutation Type Probability
0%
2%
4%
6%
8%
10%
C>A
C>G
C>T
T>A
T>C
T>G

Cancer processes Weights for TCGA-B6-A0RE

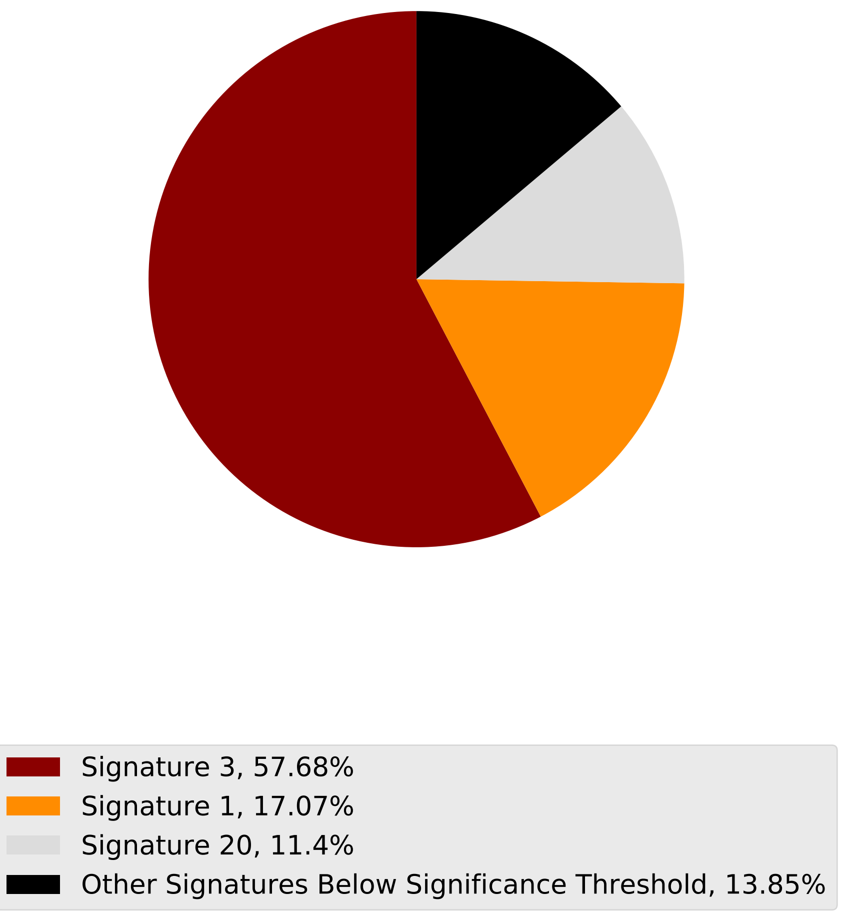

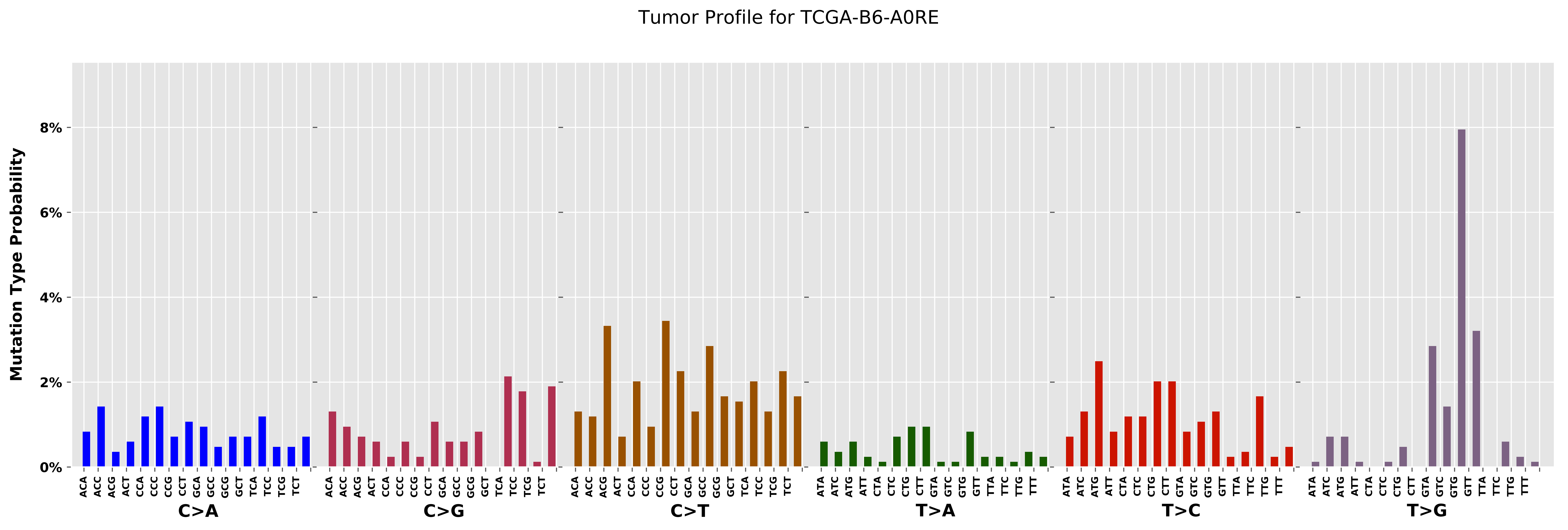

Tumor Profile for TCGA-B6-A0RE
Mutation Type Probability
8%
6%
4%
2%
0%
ACA ACC ACG ACT CCA CCC CCG CCT GCA GCC GCG GCT TCA TCC TCG TCT
C>A
ACA ACC ACG ACT CCA CCC CCG CCT GCA GCC GCG GCT TCA TCC TCG TCT
C>G
ACA ACC ACG ACT CCA CCC CCG CCT GCA GCC GCG GCT TCA TCC TCG TCT
C>T
ATA ATC ATG ATT CTA CTC CTG CTT GTA GTC GTG GTT TTA TTC TTG TTT
T>A
ATA ATC ATG ATT CTA CTC CTG CTT GTA GTC GTG GTT TTA TTC TTG TTT
T>C
ATA ATC ATG ATT CTA CTC CTG CTT GTA GTC GTG GTT TTA TTC TTG TTT
T>G

Cancer processes Weights for TCGA-A8-A08S

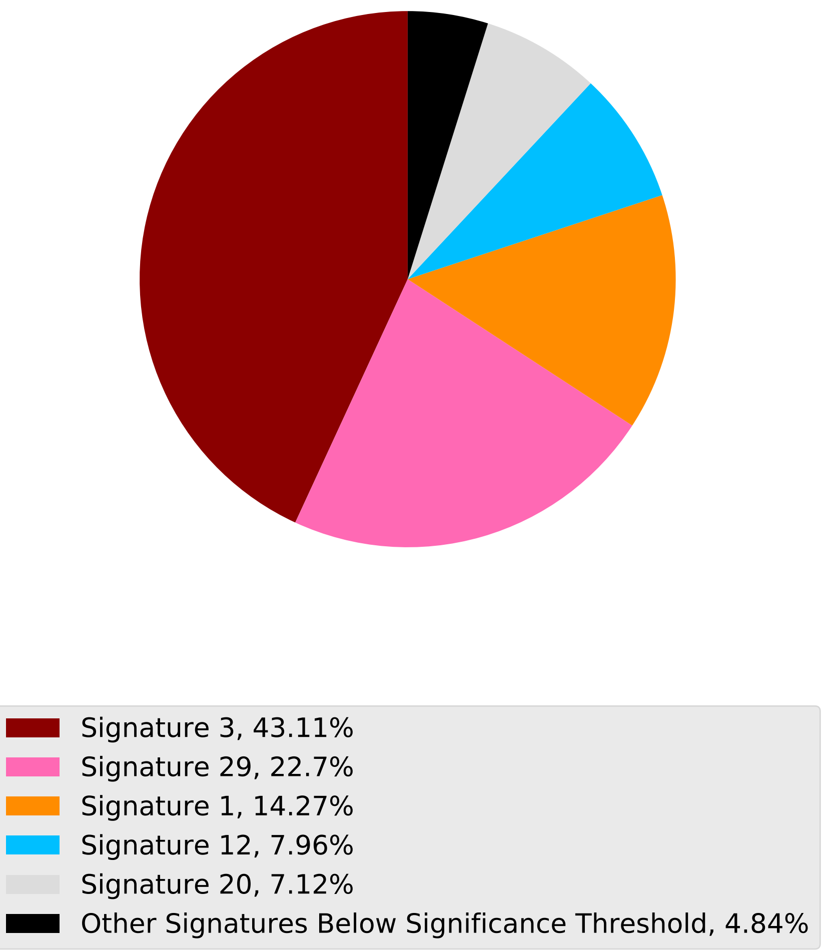

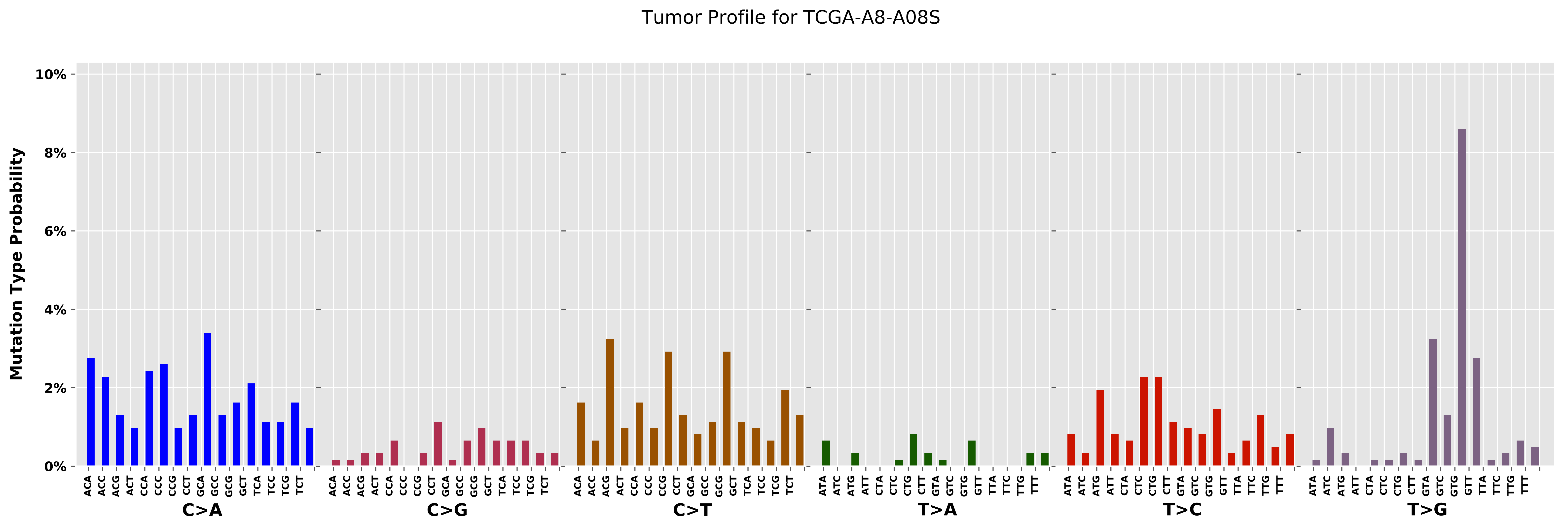
Tumor Profile for TCGA-A8-A08S
Mutation Type Probability
10%
8%
6%
4%
2%
0%
ACA ACC ACG ACT CCA CCC CCG CCT GCA GCC GCG GCT TCA TCC TCG TCT
C>A
C>G
C>T
ATA ATC ATG ATT CTA CTC CTG CTT GTA GTC GTG GTT TTA TTC TTG TTT
T>A
T>C
T>G

Cancer processes Weights for TCGA-AA-A03F

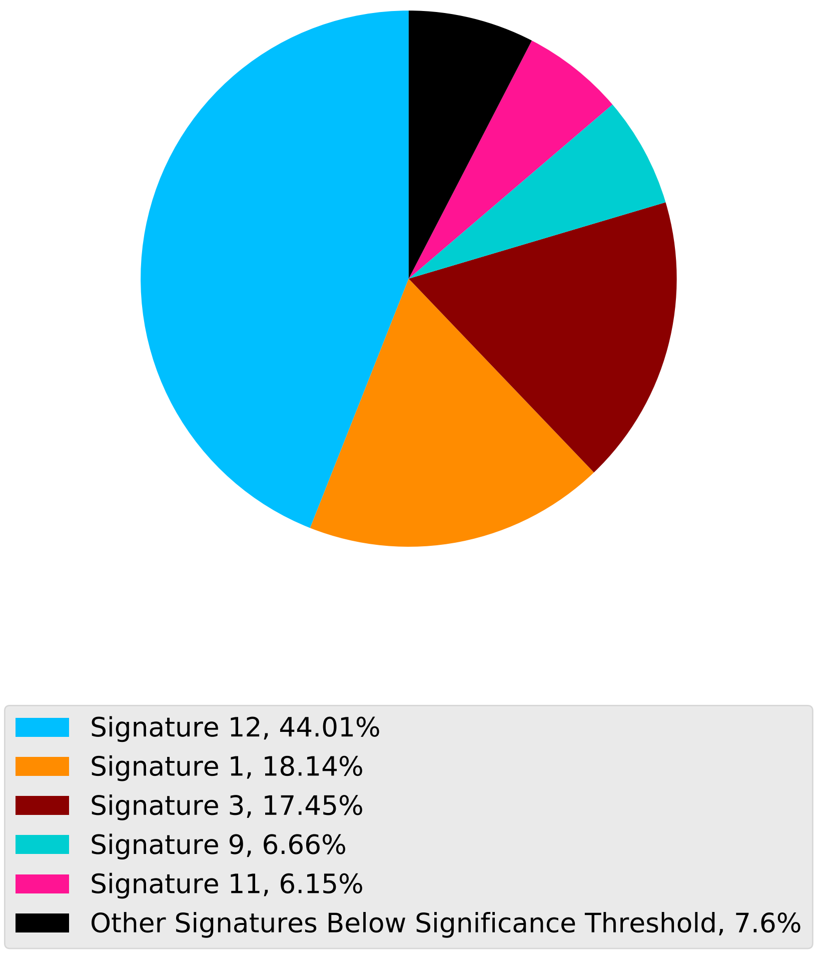

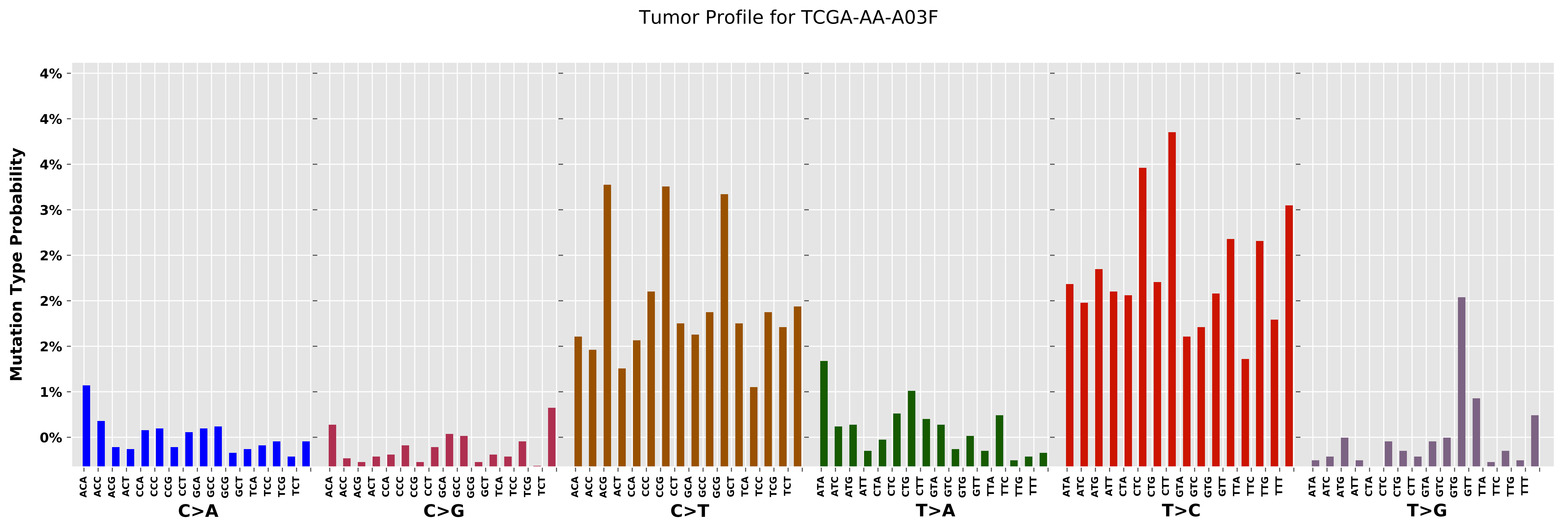
Tumor Profile for TCGA-AA-A03F
Mutation Type Probability
4%
4%
4%
3%
2%
2%
2%
1%
0%
C>A
C>G
C>T
T>A
T>C
T>G
ACA
ACC
ACG
ACT
CCA
CCC
CCG
CCT
GCA
GCC
GCG
GCT
TCA
TCC
TCG
TCT
ATA
ATC
ATG
ATT
CTA
CTC
CTG
CTT
GTA
GTC
GTG
GTT
TTA
TTC
TTG
TTT

Cancer processes Weights for TCGA-EW-A1PH

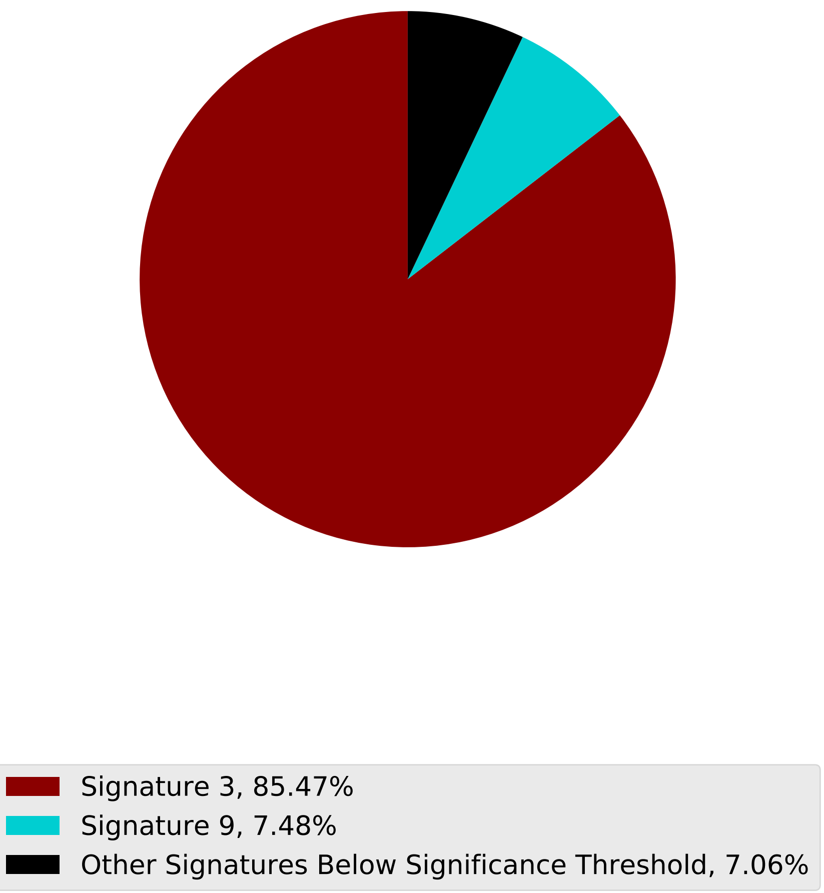

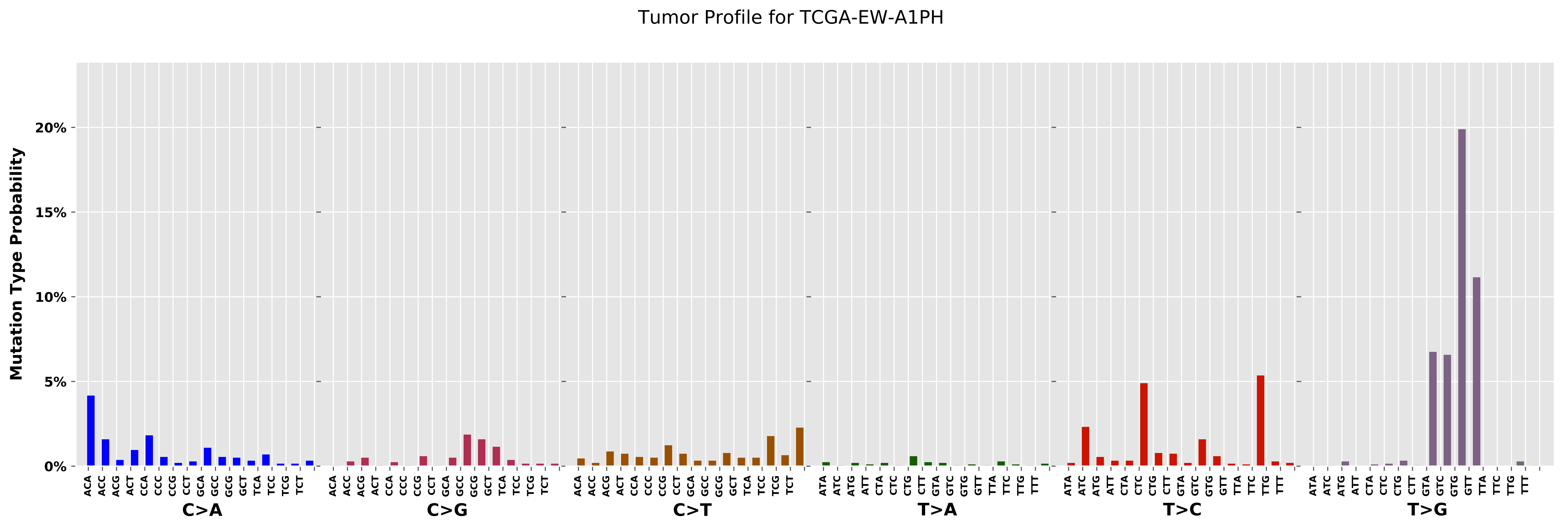

Tumor Profile for TCGA-EW-A1PH
Mutation Type Probability
20%
15%
10%
5%
0%
C>A
C>G
C>T
T>A
T>C
T>G

Cancer processes Weights for TCGA-E2-A1LG

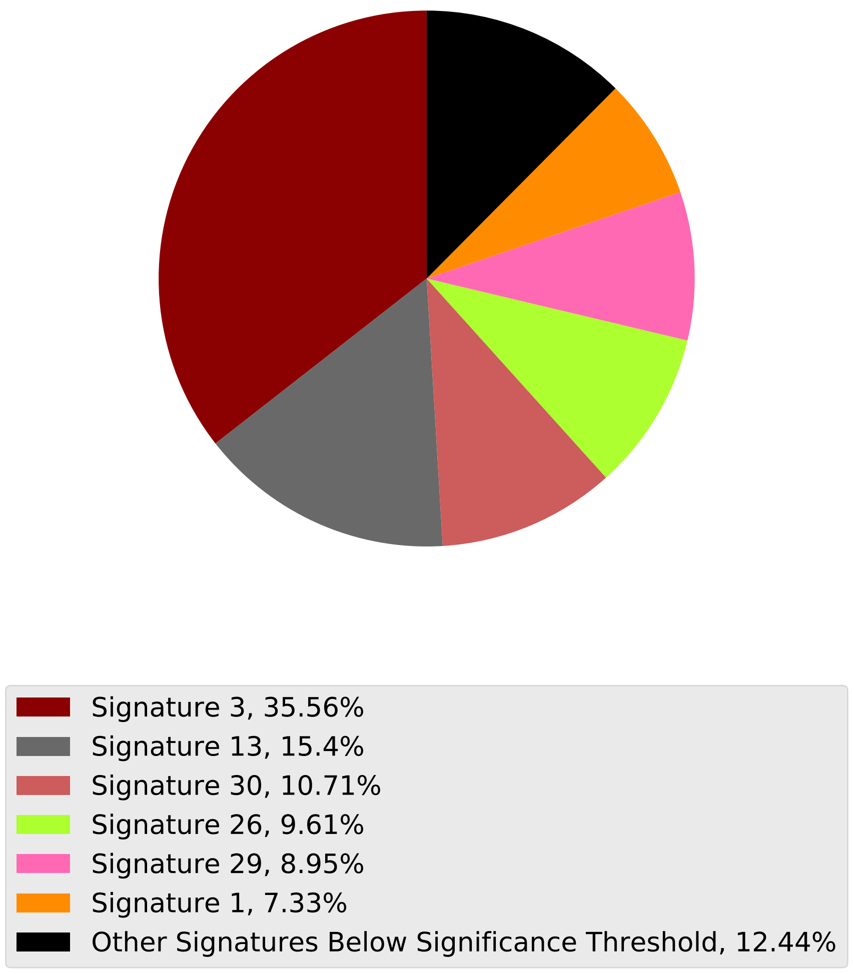

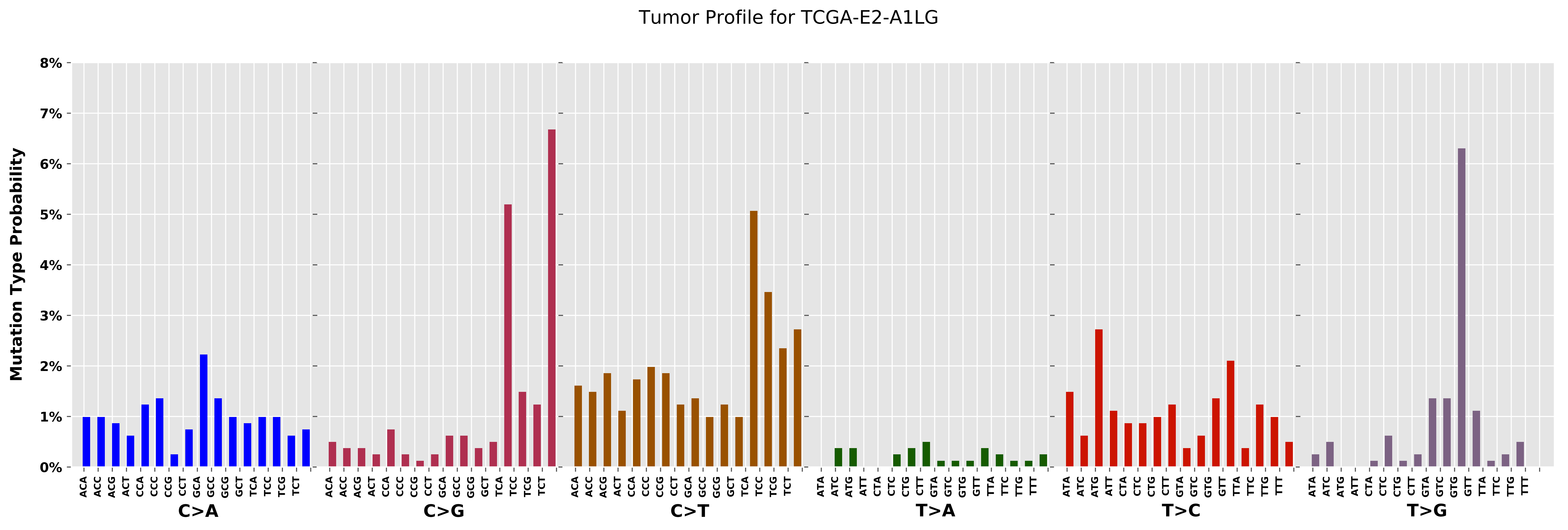
Tumor Profile for TCGA-E2-A1LG
Mutation Type Probability
0%
1%
2%
3%
4%
5%
6%
7%
8%
C>A
C>G
C>T
T>A
T>C
T>G

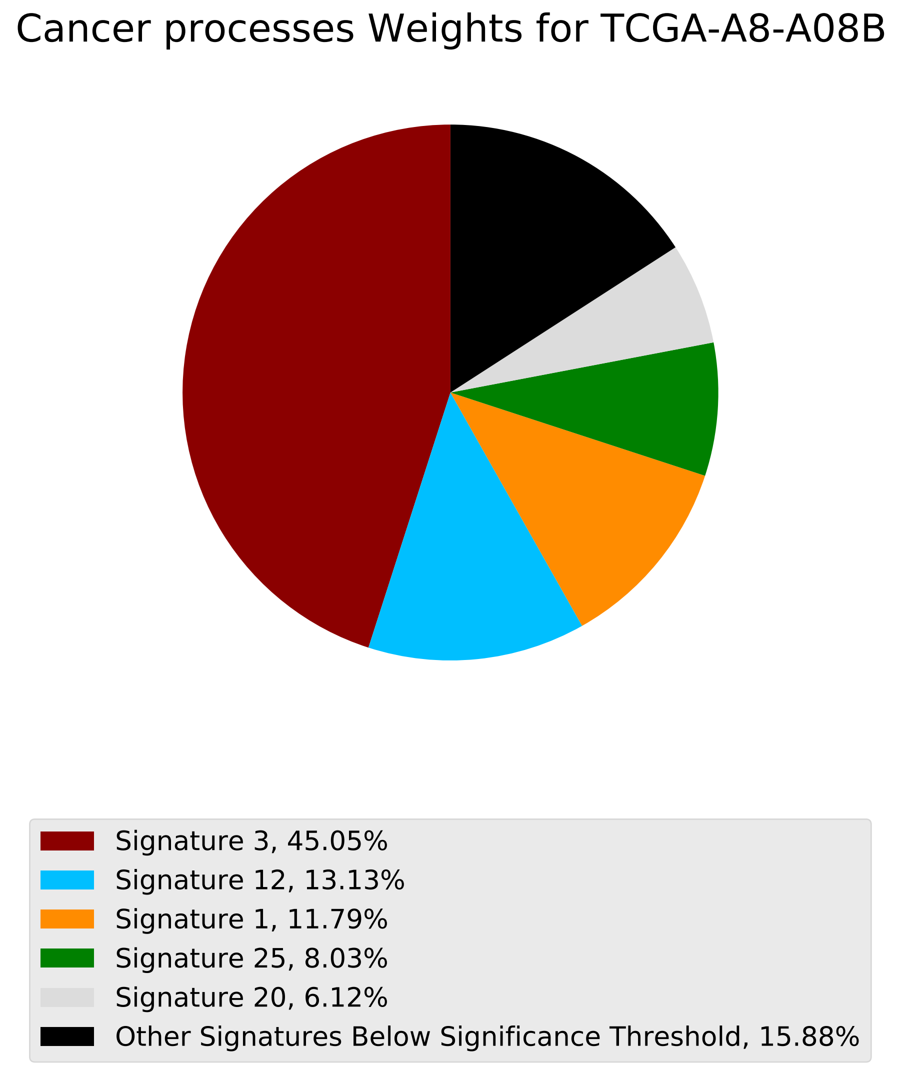
Cancer processes Weights for TCGA-A8-A08B
Signature 3, 45.05%
Signature 12, 13.13%
Signature 1, 11.79%
Signature 25, 8.03%
Signature 20, 6.12%
Other Signatures Below Significance Threshold, 15.88%

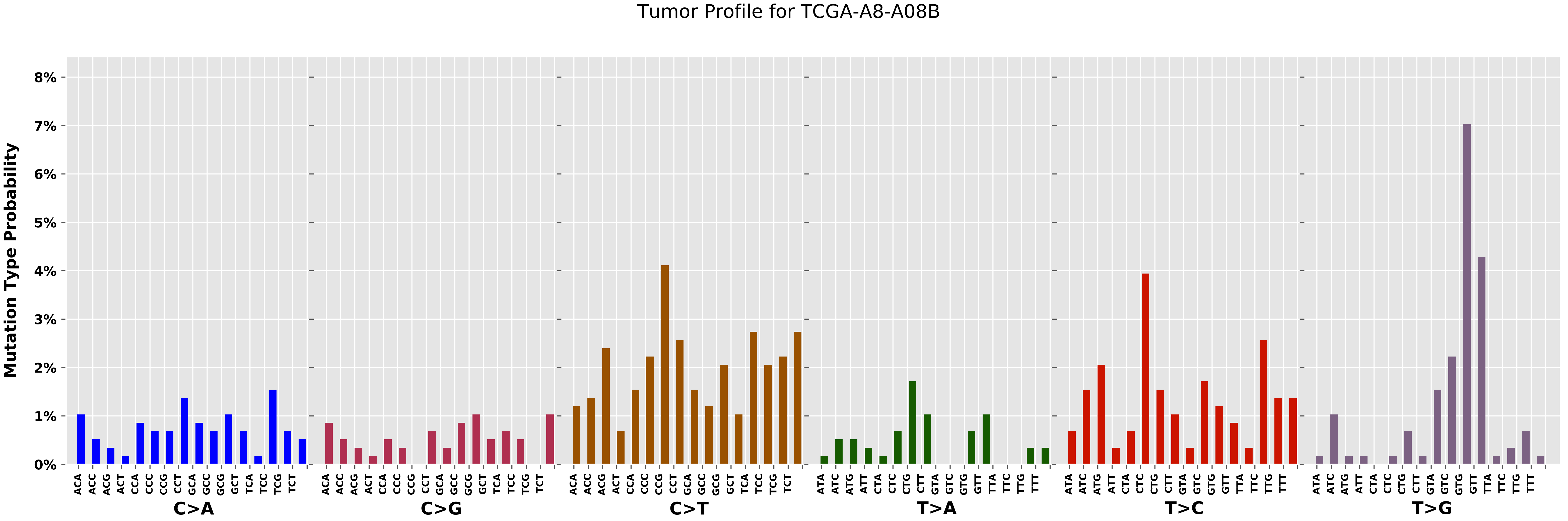
Tumor Profile for TCGA-A8-A08B
Mutation Type Probability
0%
1%
2%
3%
4%
5%
6%
7%
8%
C>A
C>G
C>T
T>A
T>C
T>G
ACA ACC ACG ACT CCA CCC CCG CCT GCA GCC GCG GCT TCA TCC TCG TCT
ATA ATC ATG ATT CTA CTC CTG CTT GTA GTC GTG GTT TTA TTC TTG TTT

Cancer processes Weights for TCGA-B6-A0RT

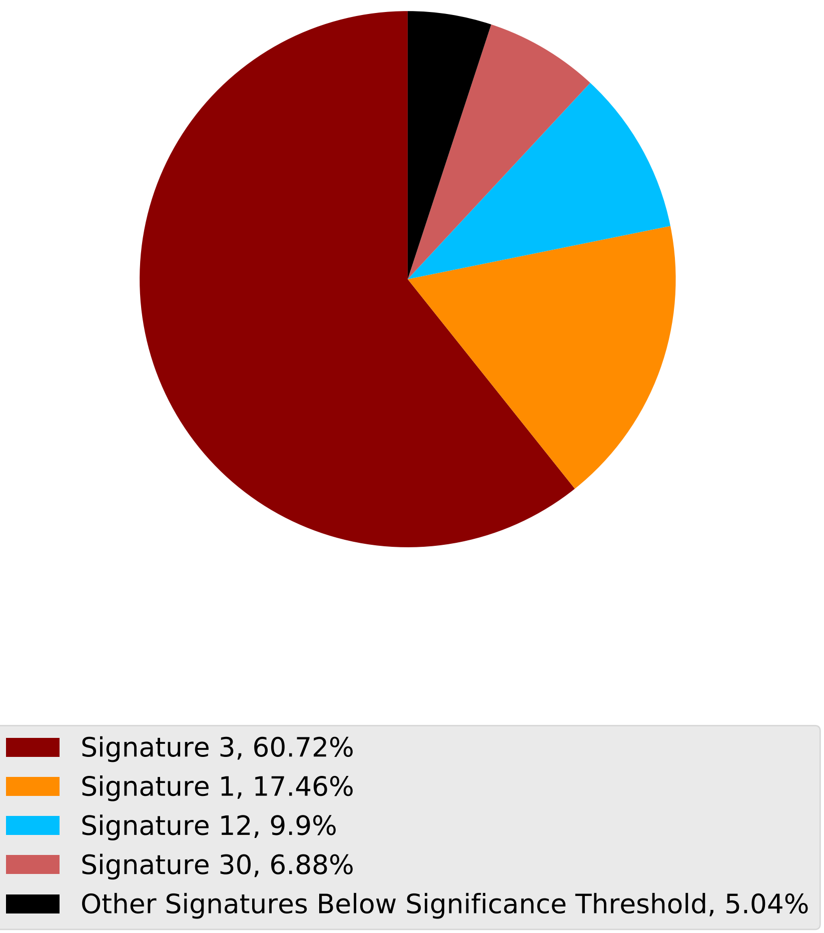

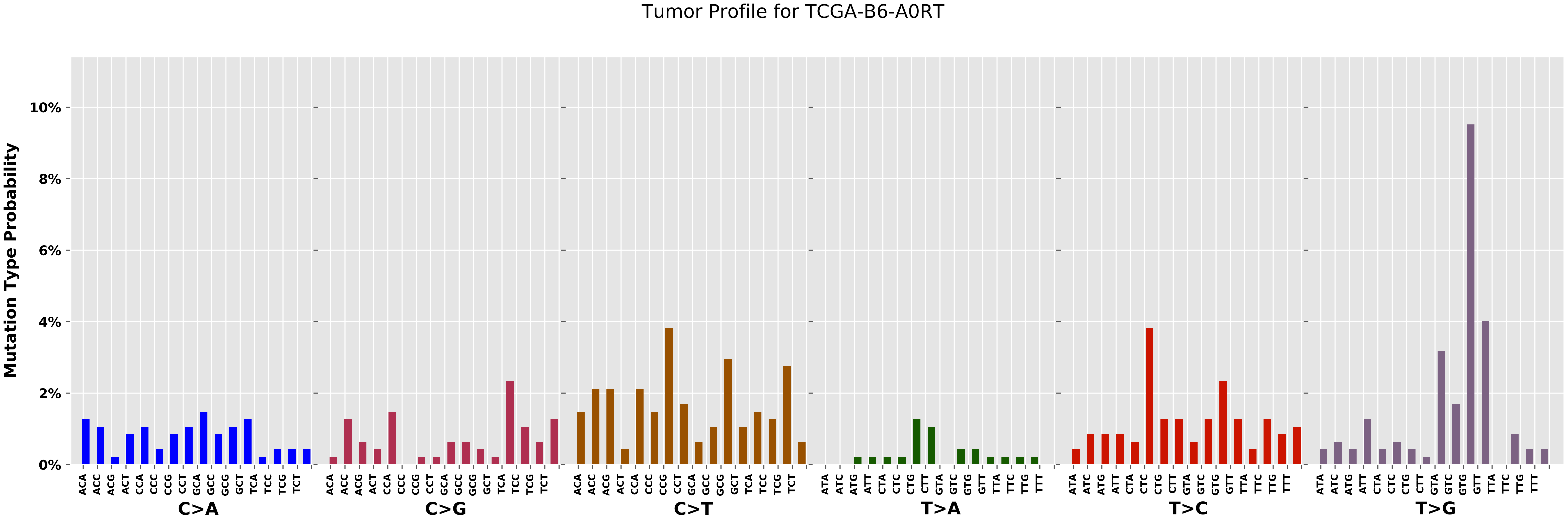

Tumor Profile for TCGA-B6-A0RT
Mutation Type Probability
10%
8%
6%
4%
2%
0%
C>A
C>G
C>T
T>A
T>C
T>G

Cancer processes Weights for TCGA-GM-A3XL

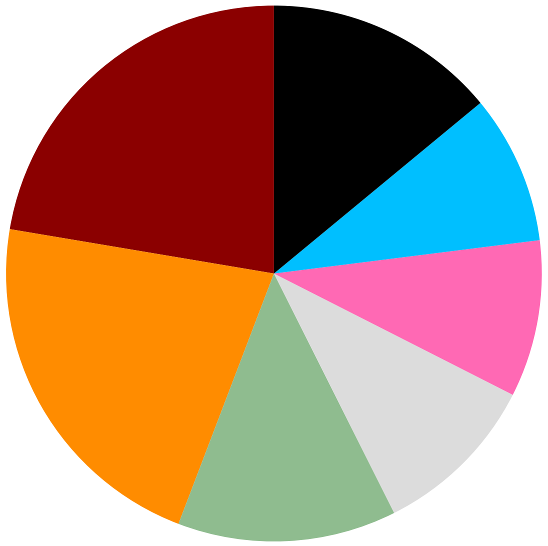

Signature 3, 22.35%
Signature 1, 21.85%
Signature 5, 13.21%
Signature 20, 10.11%
Signature 29, 9.47%
Signature 12, 9.0%
Other Signatures Below Significance Threshold, 14.01%

Tumor Profile for TCGA-GM-A3XL

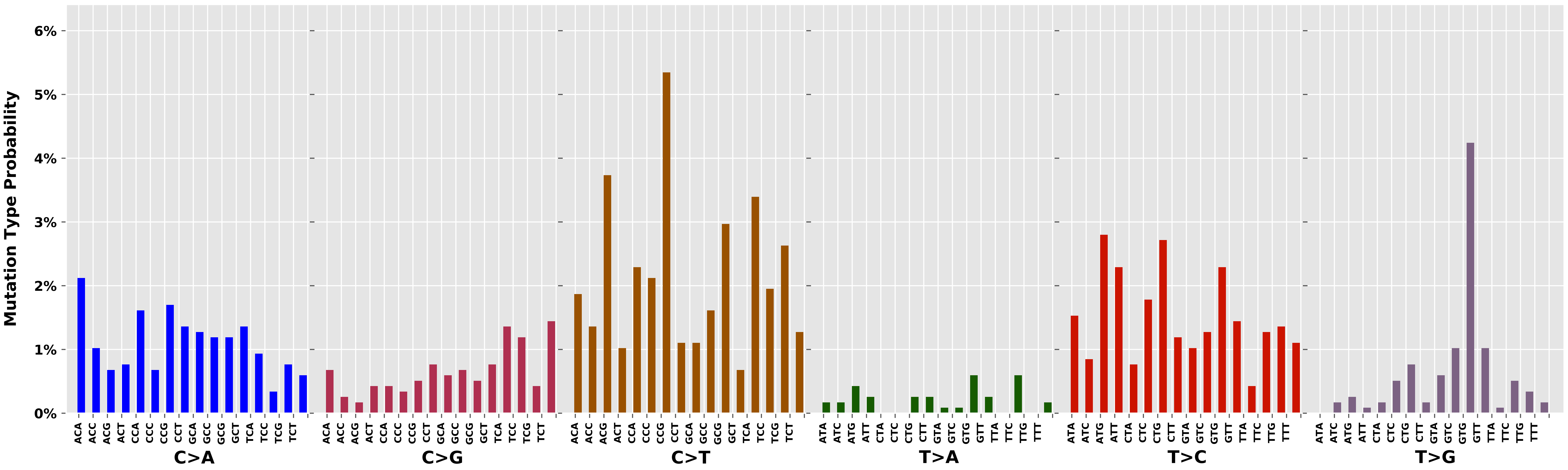

Cancer processes Weights for TCGA-AO-A0J2

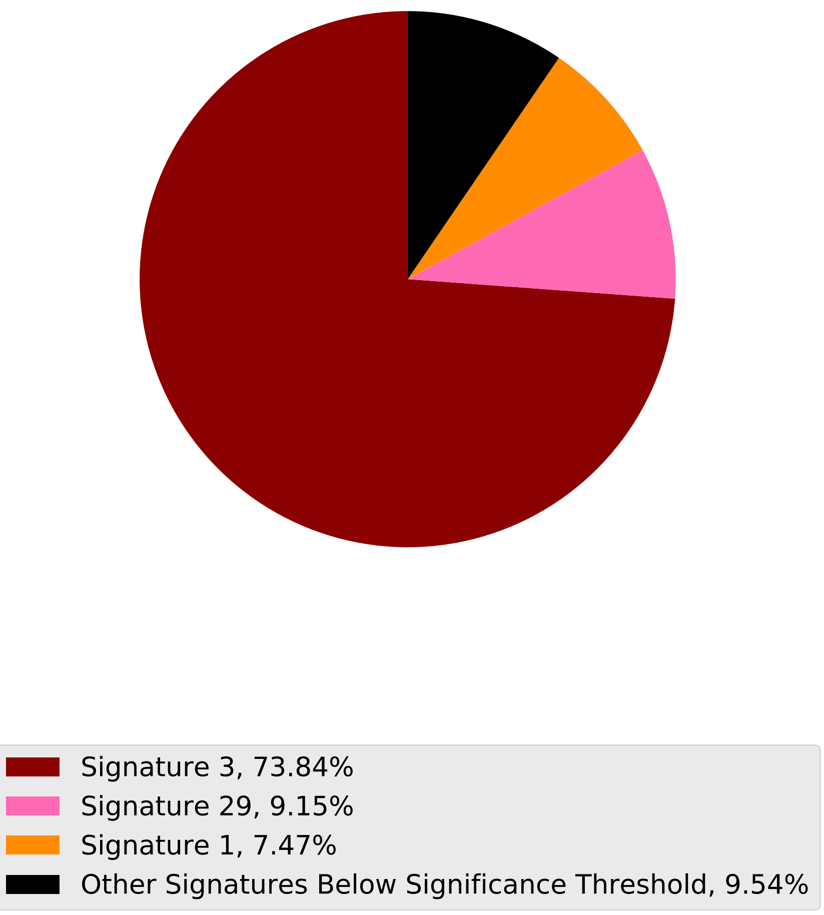

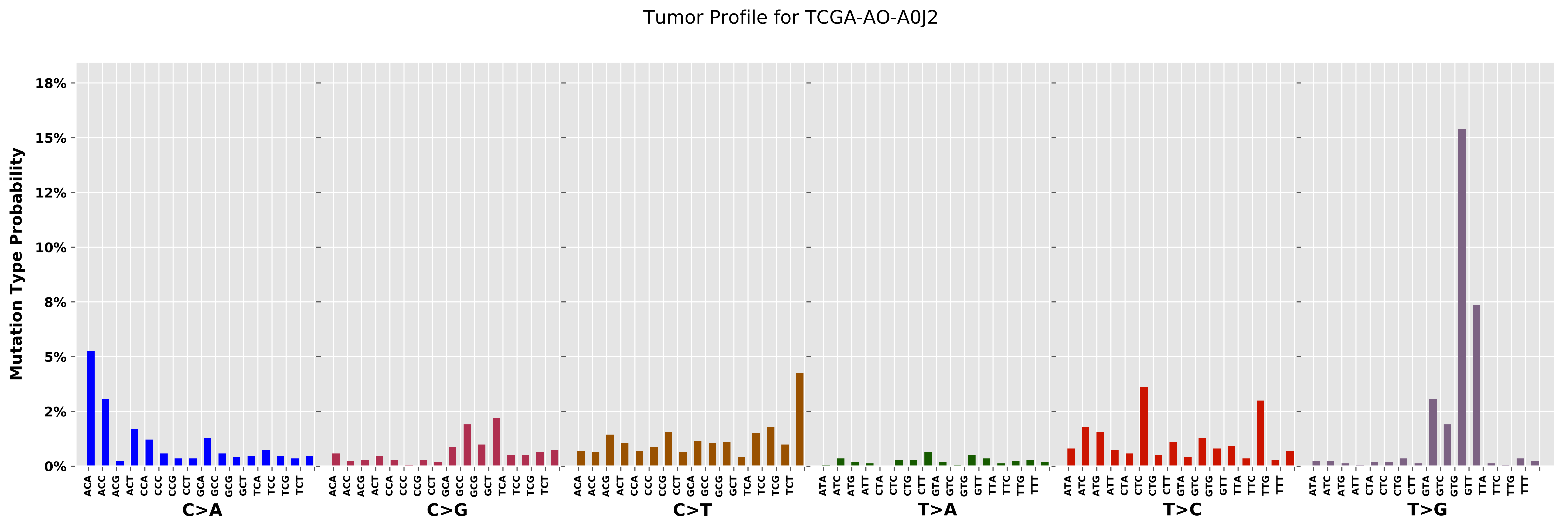
Tumor Profile for TCGA-AO-A0J2
Mutation Type Probability
18%
15%
12%
10%
8%
5%
2%
0%
ACA ACC ACG ACT CCA CCC CCG CCT GCA GCC GCG GCT TCA TCC TCG TCT
ATA ATC ATG ATT CTA CTC CTG CTT GTA GTC GTG GTT TTA TTC TTG TTT
C>A
C>G
C>T
T>A
T>C
T>G

Cancer processes Weights for TCGA-E2-A156

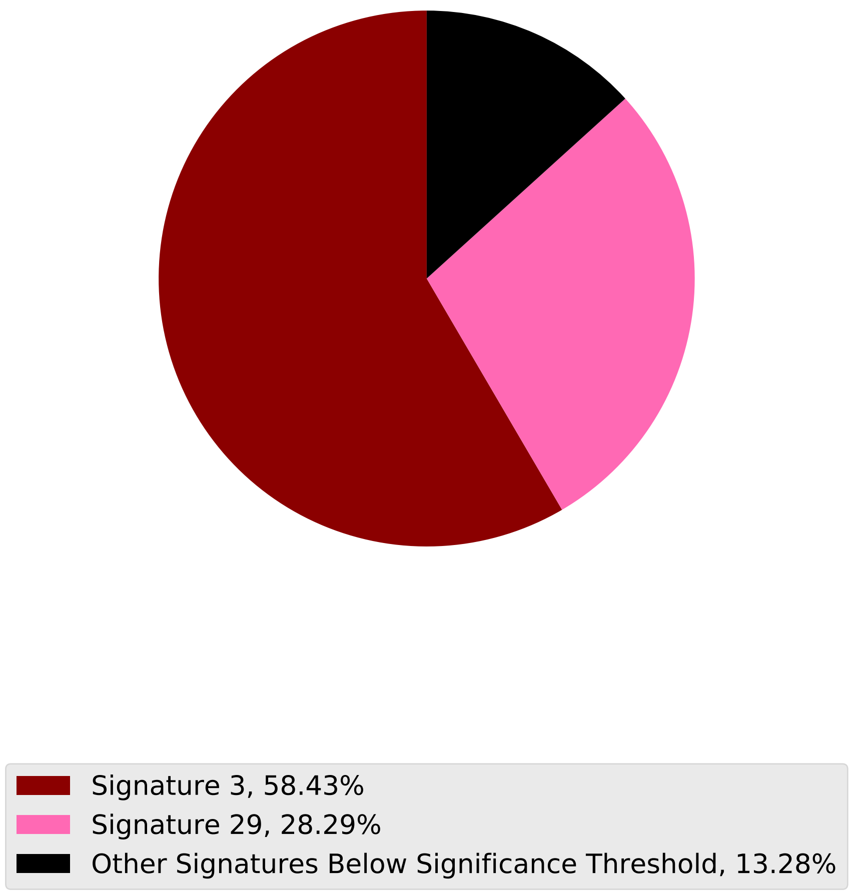

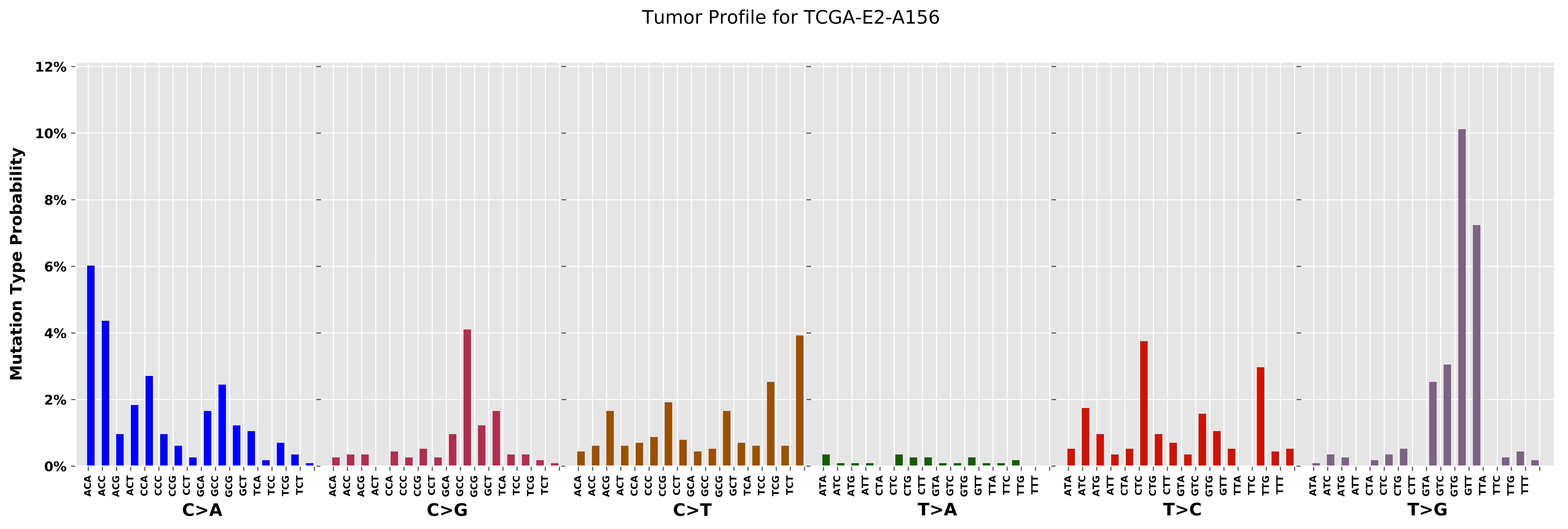
Tumor Profile for TCGA-E2-A156
Mutation Type Probability
0%
2%
4%
6%
8%
10%
12%
C>A
C>G
C>T
T>A
T>C
T>G
ACA ACC ACG ACT CCA CCC CCG CCT GCA GCC GCG GCT TCA TCC TCG TCT
ATA ATC ATG ATT CTA CTC CTG CTT GTA GTC GTG GTT TTA TTC TTG TTT

Cancer processes Weights for TCGA-BH-A18R

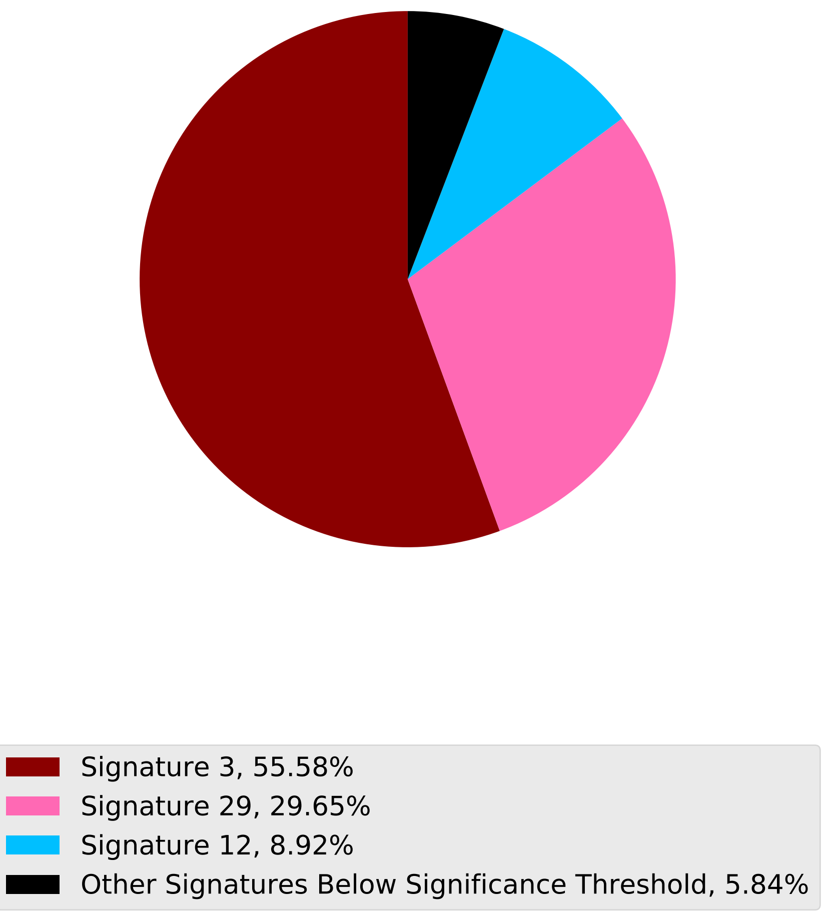

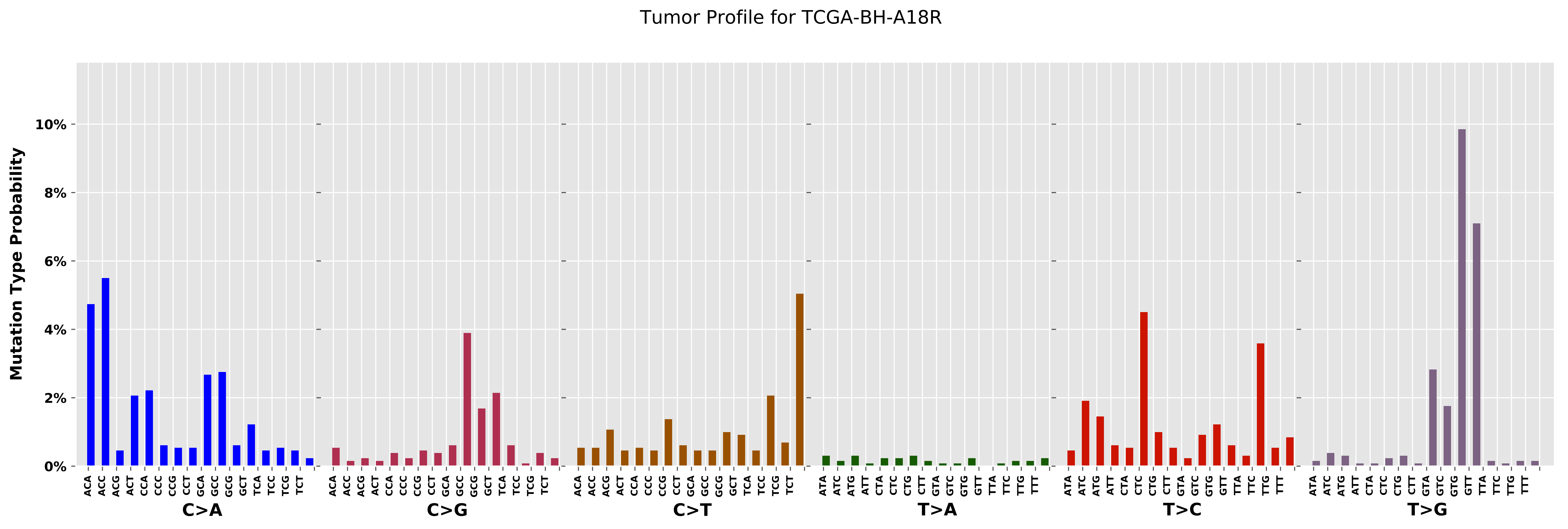
Tumor Profile for TCGA-BH-A18R
Mutation Type Probability
0%
2%
4%
6%
8%
10%
C>A
C>G
C>T
T>A
T>C
T>G
ACA
ACC
ACG
ACT
CCA
CCC
CCG
CCT
GCA
GCC
GCG
GCT
TCA
TCC
TCG
TCT
ATA
ATC
ATG
ATT
CTA
CTC
CTG
CTT
GTA
GTC
GTG
GTT
TTA
TTC
TTG
TTT

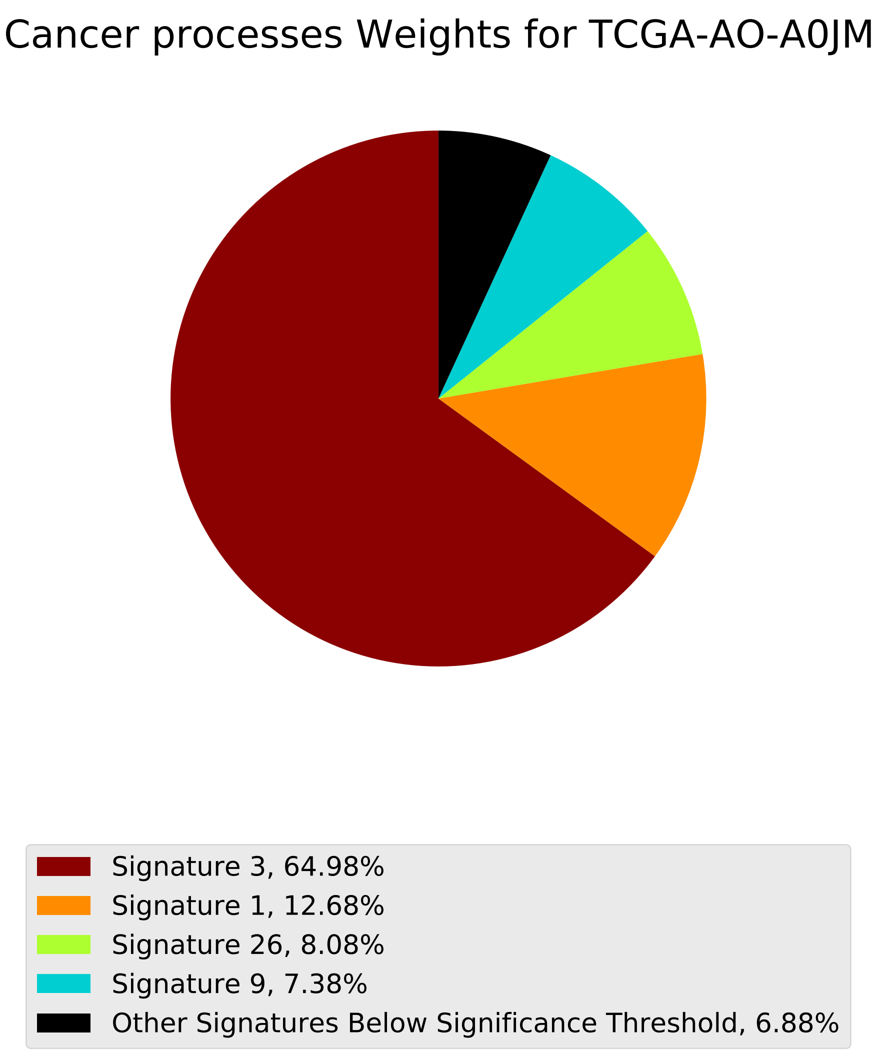
Cancer processes Weights for TCGA-AO-A0JM
Signature 3, 64.98%
Signature 1, 12.68%
Signature 26, 8.08%
Signature 9, 7.38%
Other Signatures Below Significance Threshold, 6.88%

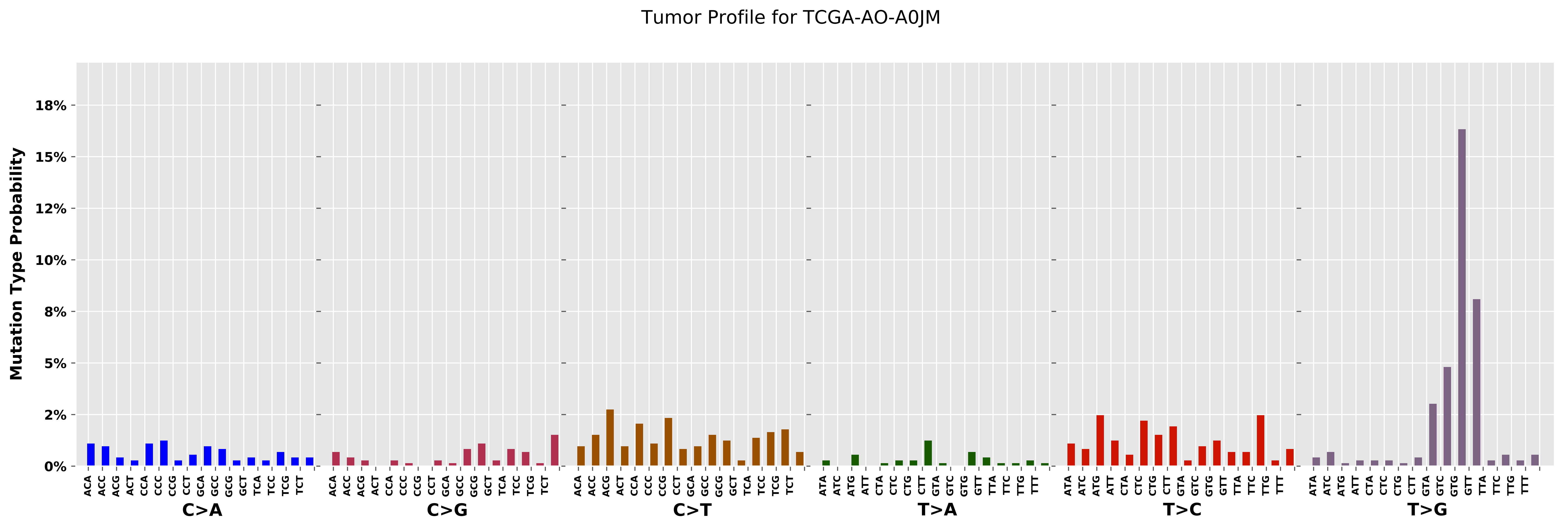

Tumor Profile for TCGA-AO-A0JM
Mutation Type Probability
18%
15%
12%
10%
8%
5%
2%
0%
C>A
C>G
C>T
T>A
T>C
T>G

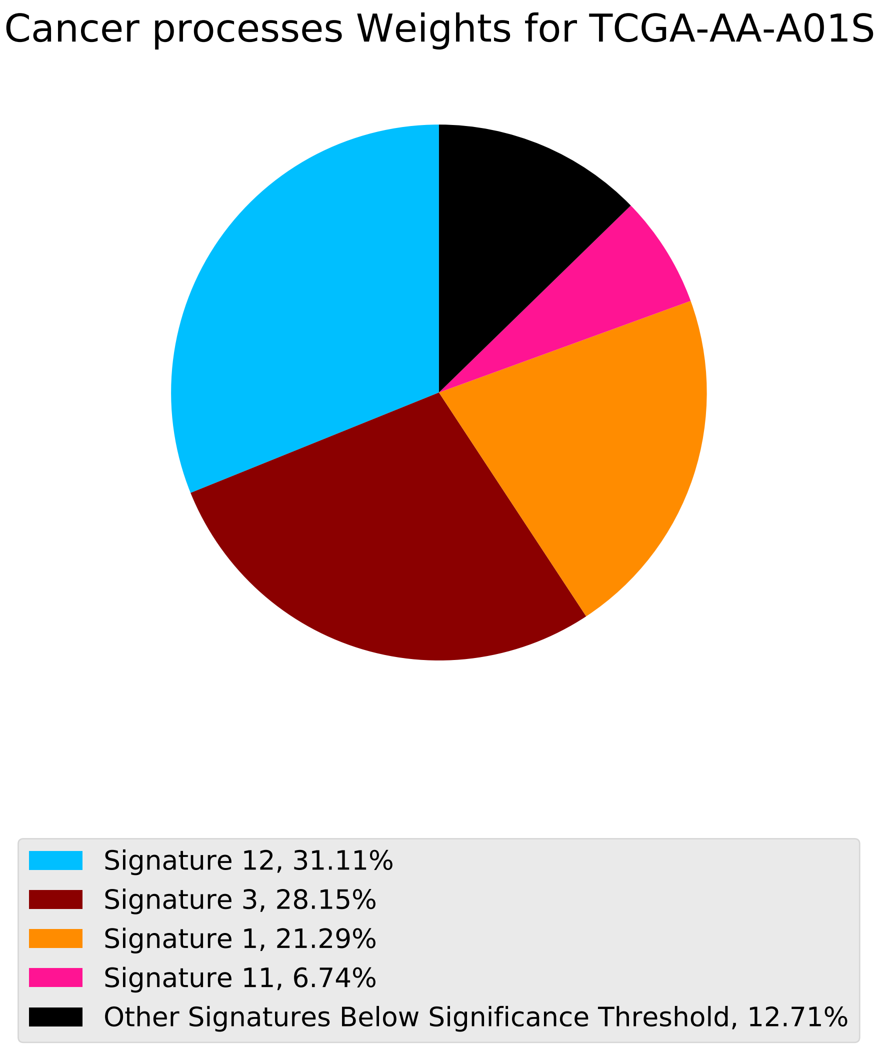
Cancer processes Weights for TCGA-AA-A01S
Signature 12, 31.11%
Signature 3, 28.15%
Signature 1, 21.29%
Signature 11, 6.74%
Other Signatures Below Significance Threshold, 12.71%

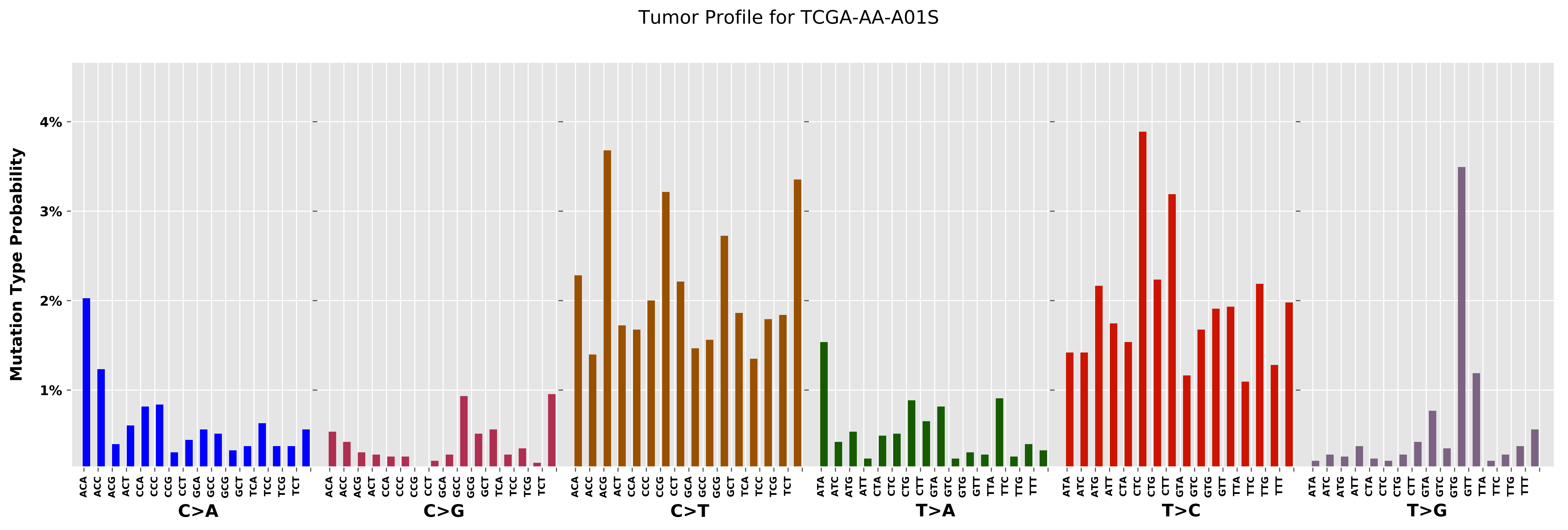
Tumor Profile for TCGA-AA-A01S
Mutation Type Probability
4%
3%
2%
1%
C>A
C>G
C>T
T>A
T>C
T>G
ACA ACC ACG ACT CCA CCC CCG CCT GCA GCC GCG GCT TCA TCC TCG TCT
ATA ATC ATG ATT CTA CTC CTG CTT GTA GTC GTG GTT TTA TTC TTG TTT

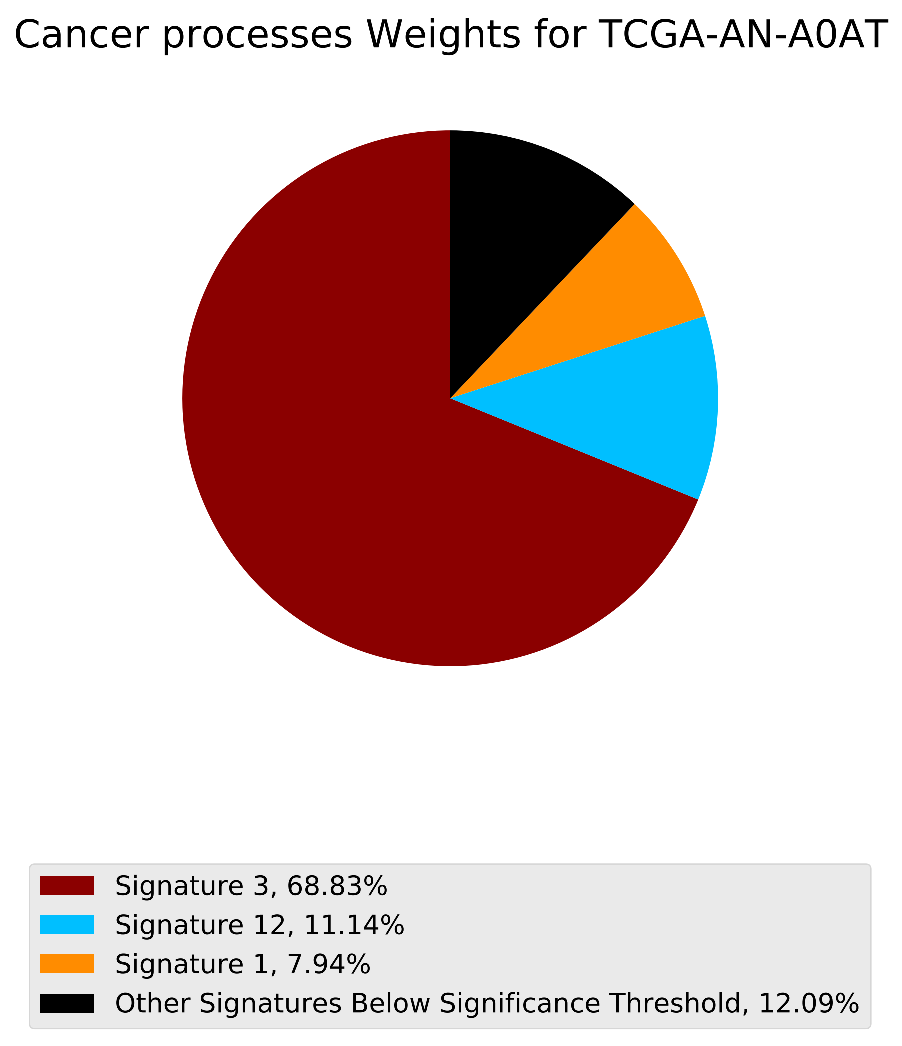
Cancer processes Weights for TCGA-AN-A0AT
Signature 3, 68.83%
Signature 12, 11.14%
Signature 1, 7.94%
Other Signatures Below Significance Threshold, 12.09%

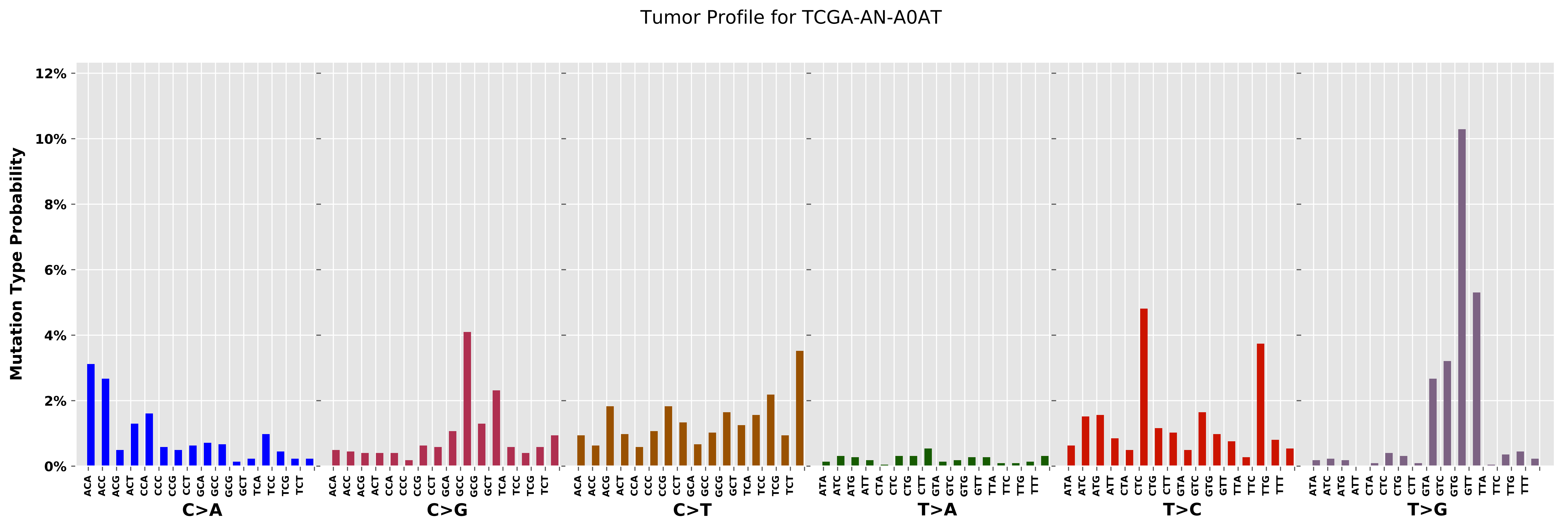

Tumor Profile for TCGA-AN-A0AT
Mutation Type Probability
12%
10%
8%
6%
4%
2%
0%
C>A
C>G
C>T
T>A
T>C
T>G

Cancer processes Weights for TCGA-B6-A0WX

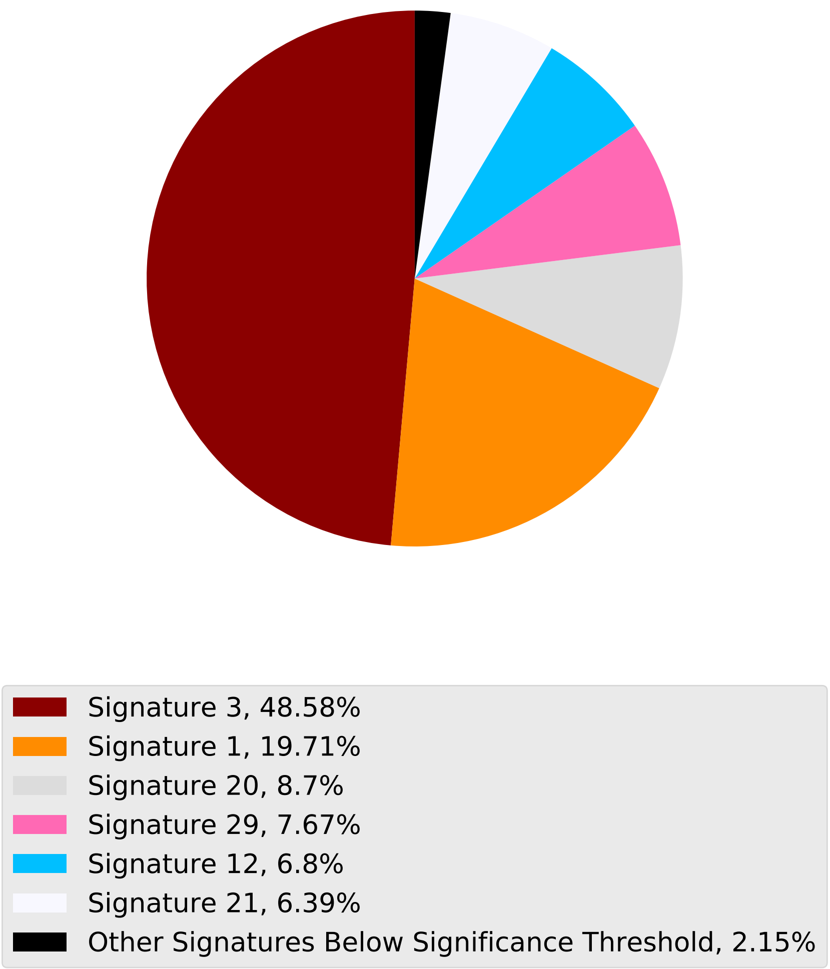

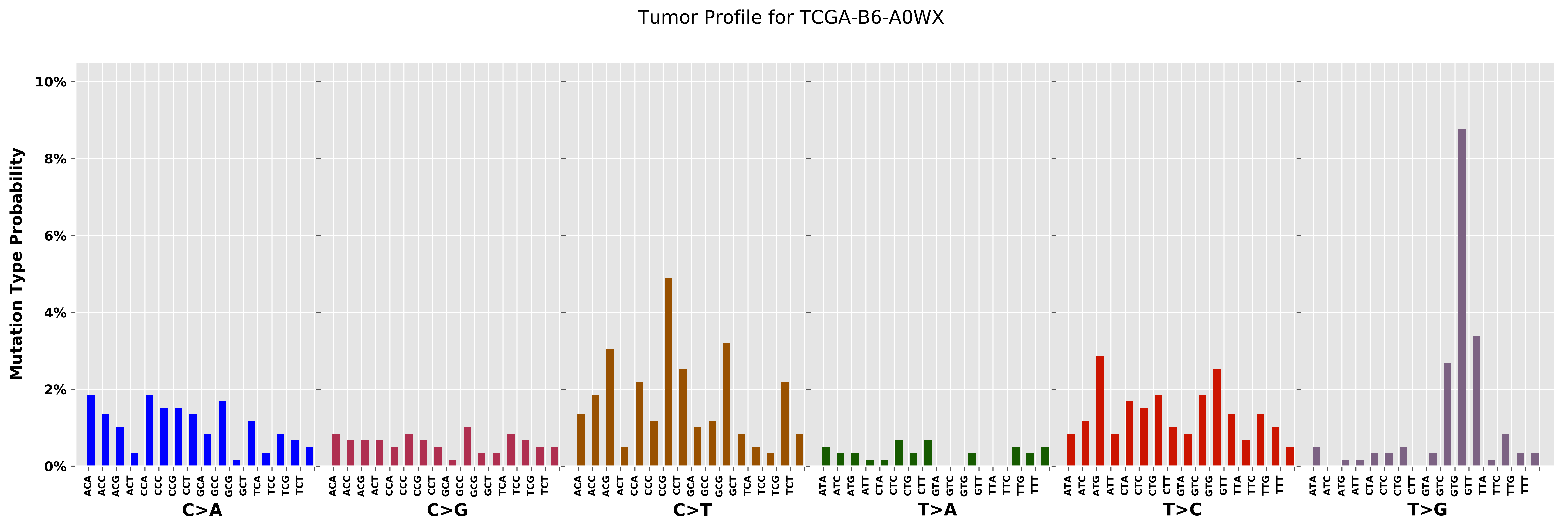

Tumor Profile for TCGA-B6-A0WX
Mutation Type Probability
0%
2%
4%
6%
8%
10%
C>A
C>G
C>T
T>A
T>C
T>G

Cancer processes Weights for TCGA-BH-A0WA

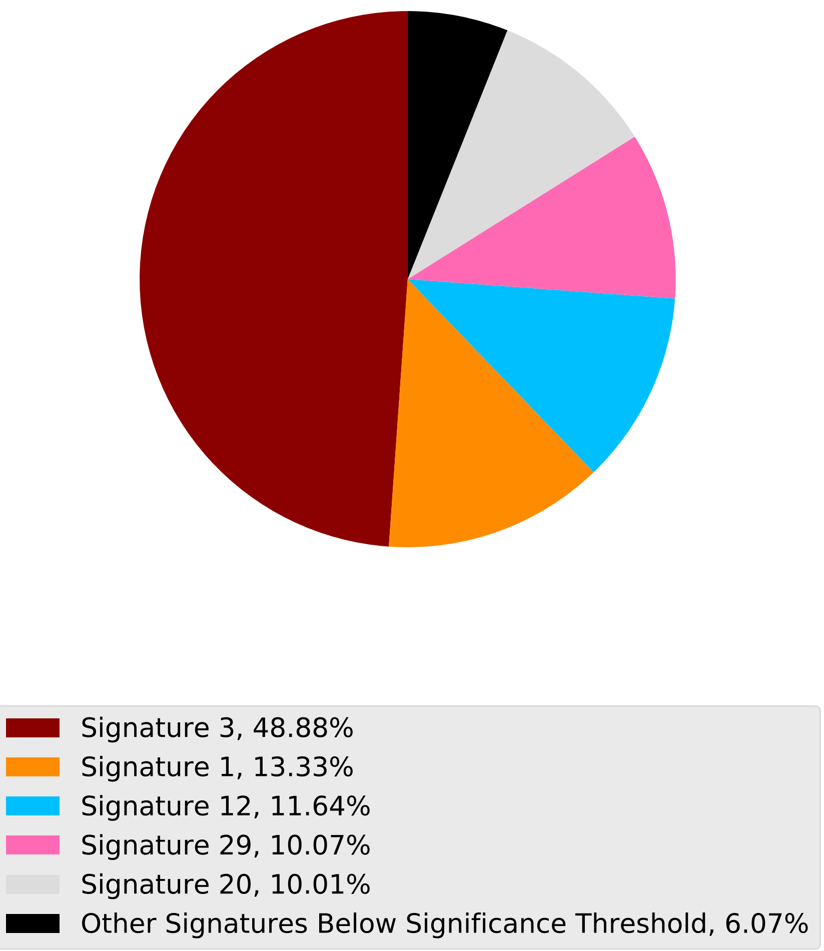

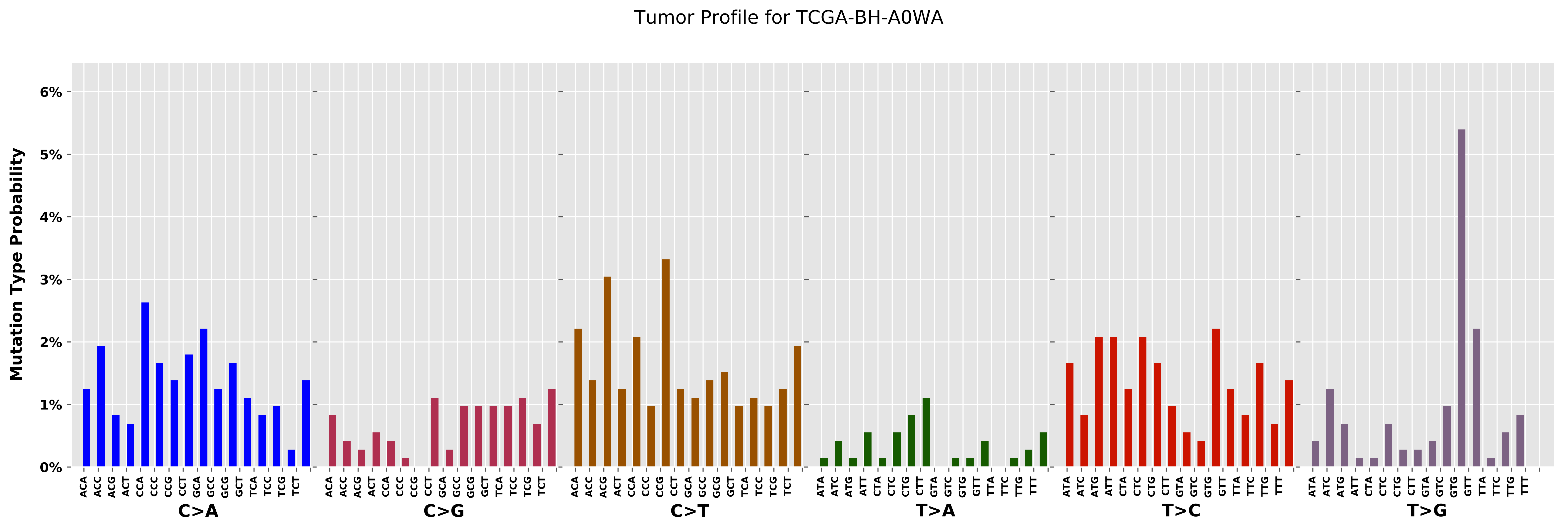

Tumor Profile for TCGA-BH-A0WA
Mutation Type Probability
0%
1%
2%
3%
4%
5%
6%
C>A
C>G
C>T
T>A
T>C
T>G
ACA ACC ACG ACT CCA CCC CCG CCT GCA GCC GCG GCT TCA TCC TCG TCT
ATA ATC ATG ATT CTA CTC CTG CTT GTA GTC GTG GTT TTA TTC TTG TTT

Cancer processes Weights for TCGA-A7-A13D

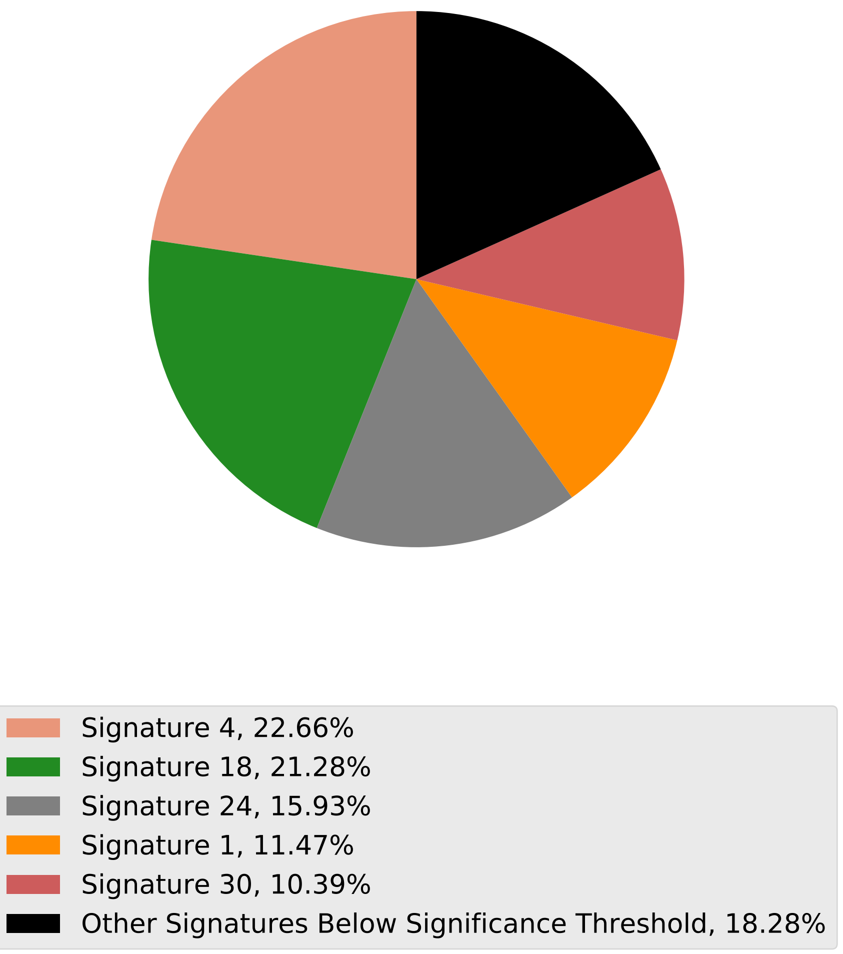

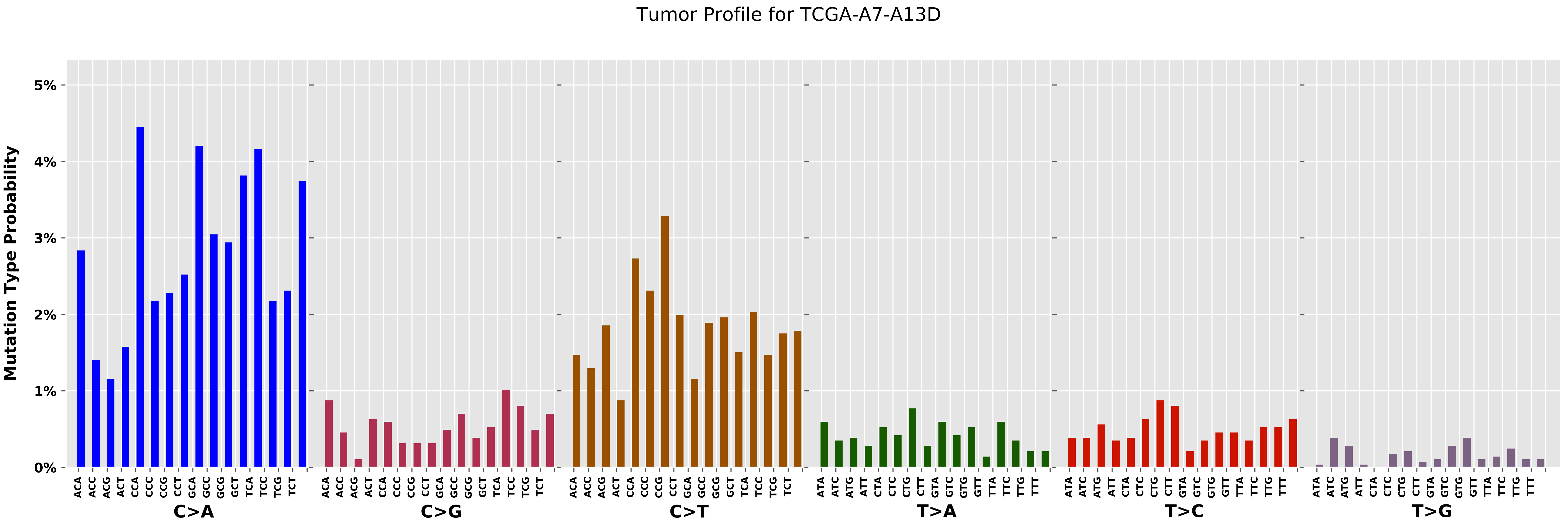
Tumor Profile for TCGA-A7-A13D
Mutation Type Probability
0%
1%
2%
3%
4%
5%
ACA ACC ACG ACT CCA CCC CCG CCT GCA GCC GCG GCT TCA TCC TCG TCT
C>A
ACA ACC ACG ACT CCA CCC CCG CCT GCA GCC GCG GCT TCA TCC TCG TCT
C>G
ACA ACC ACG ACT CCA CCC CCG CCT GCA GCC GCG GCT TCA TCC TCG TCT
C>T
ATA ATC ATG ATT CTA CTC CTG CTT GTA GTC GTG GTT TTA TTC TTG TTT
T>A
ATA ATC ATG ATT CTA CTC CTG CTT GTA GTC GTG GTT TTA TTC TTG TTT
T>C
ATA ATC ATG ATT CTA CTC CTG CTT GTA GTC GTG GTT TTA TTC TTG TTT
T>G

Cancer processes Weights for TCGA-B6-A0IJ

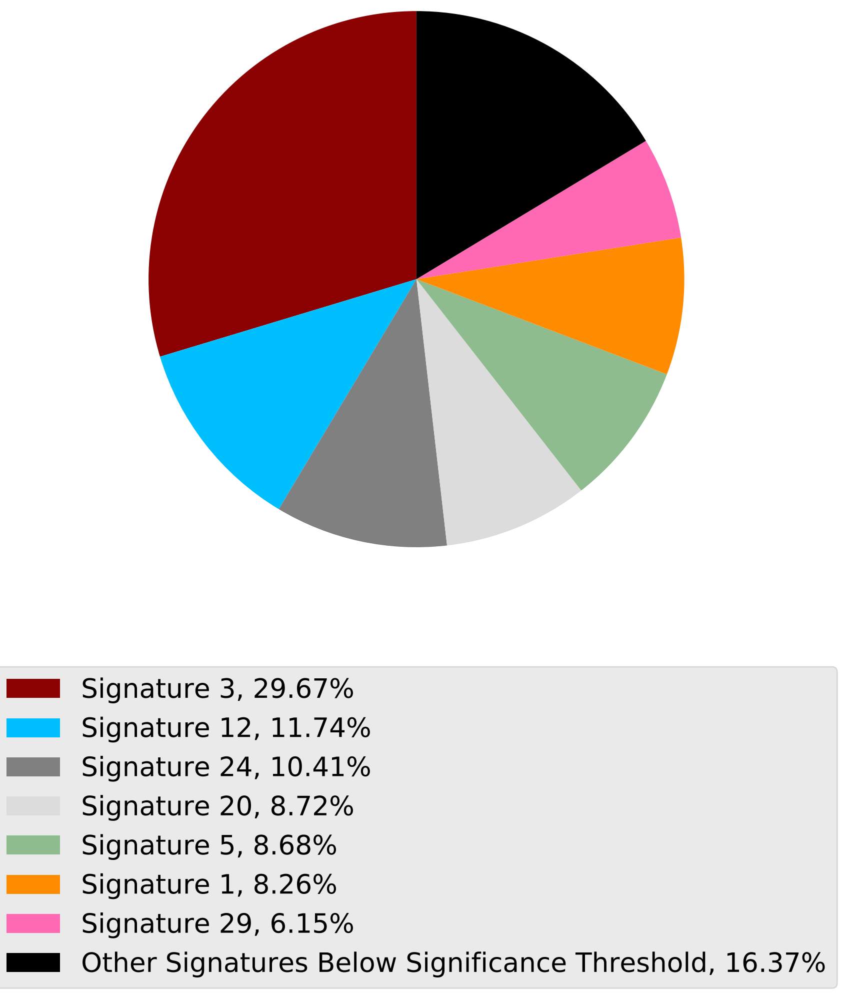

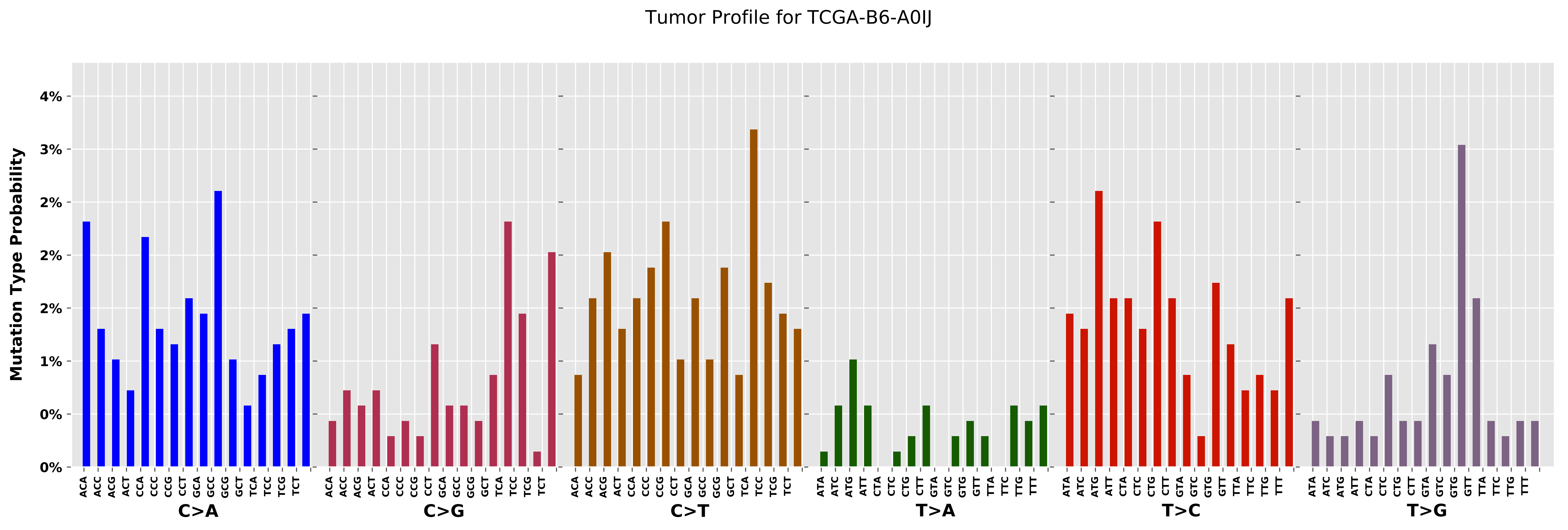
Tumor Profile for TCGA-B6-A0IJ
Mutation Type Probability
0%
1%
2%
3%
4%
C>A
C>G
C>T
T>A
T>C
T>G

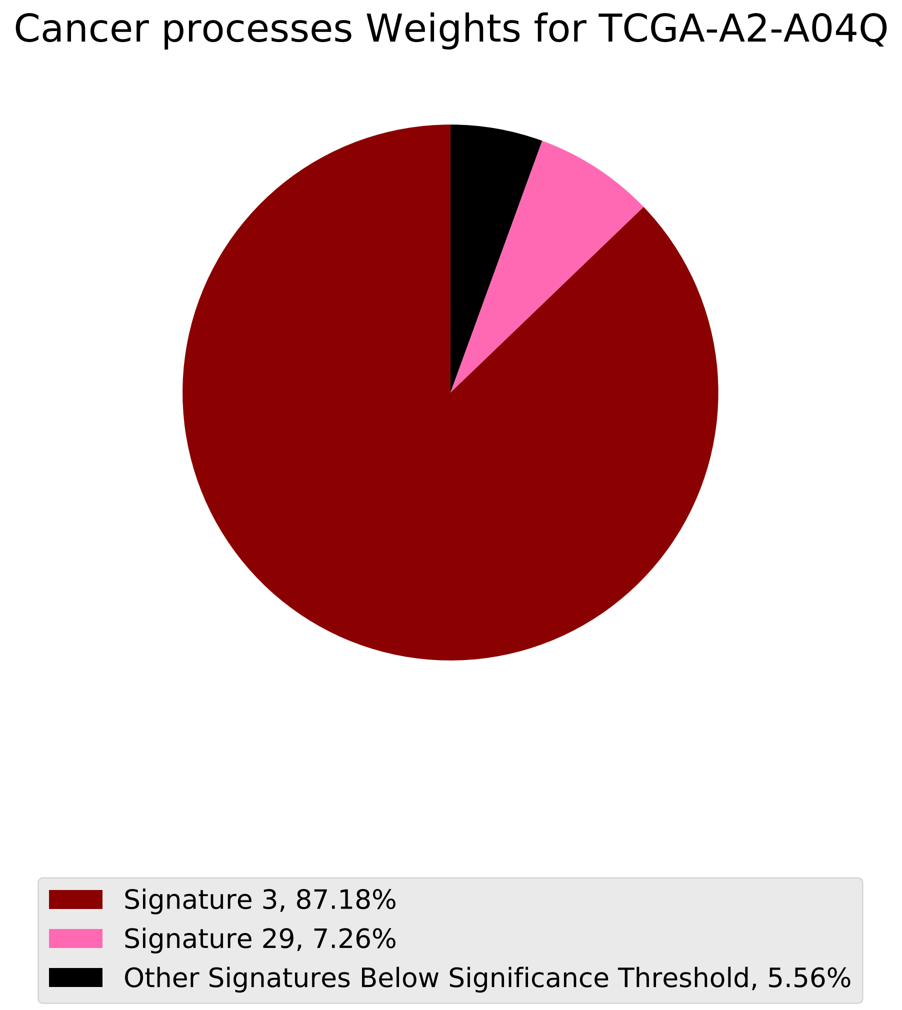
Cancer processes Weights for TCGA-A2-A04Q
Signature 3, 87.18%
Signature 29, 7.26%
Other Signatures Below Significance Threshold, 5.56%

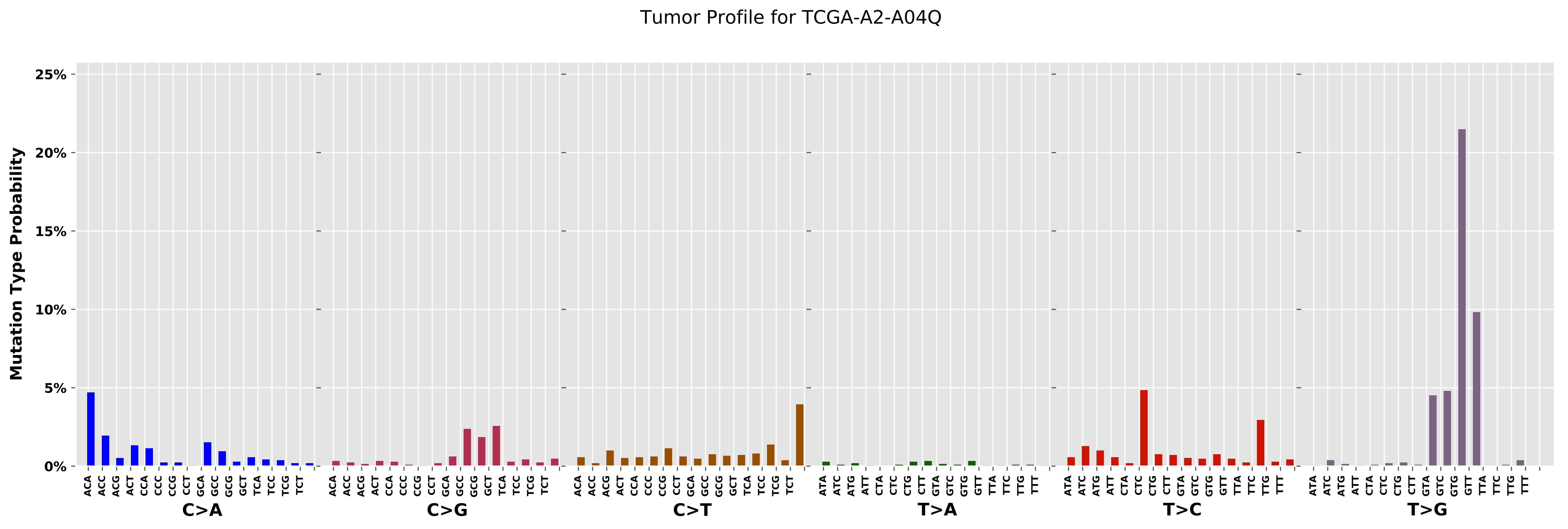

Tumor Profile for TCGA-A2-A04Q
Mutation Type Probability
25%
20%
15%
10%
5%
0%
C>A
C>G
C>T
T>A
T>C
T>G

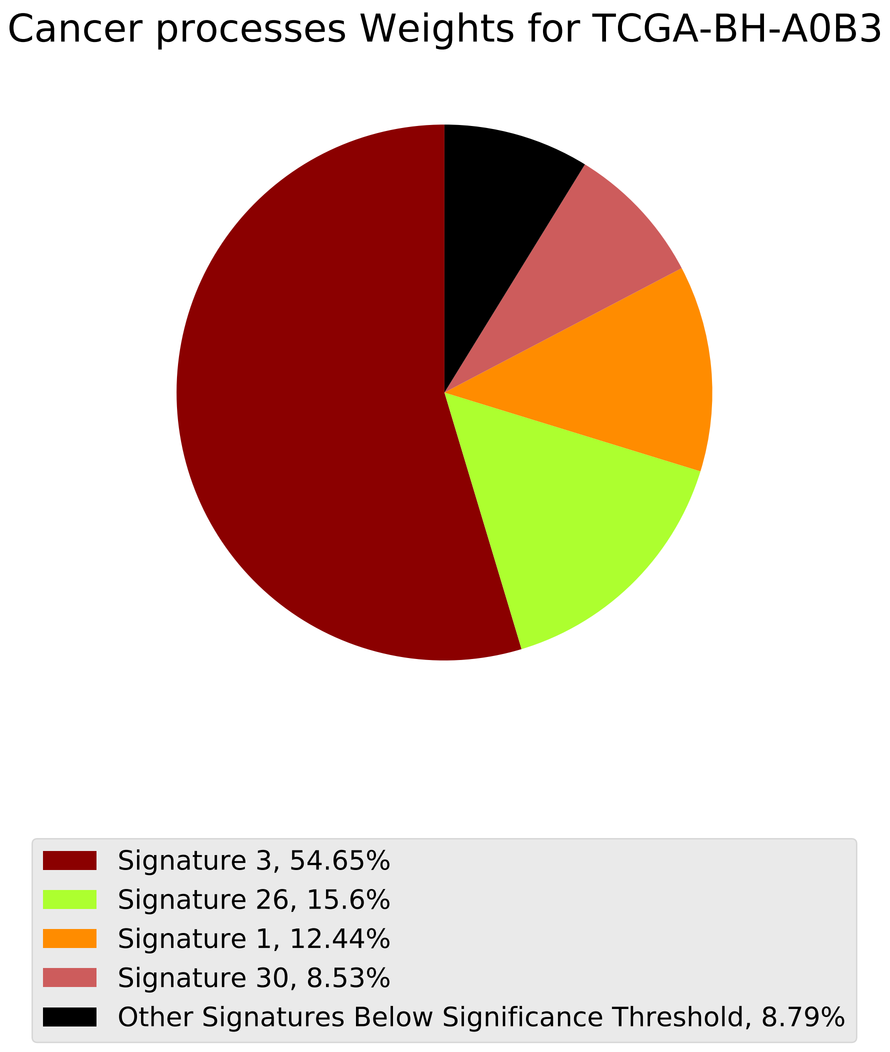
Cancer processes Weights for TCGA-BH-A0B3
Signature 3, 54.65%
Signature 26, 15.6%
Signature 1, 12.44%
Signature 30, 8.53%
Other Signatures Below Significance Threshold, 8.79%

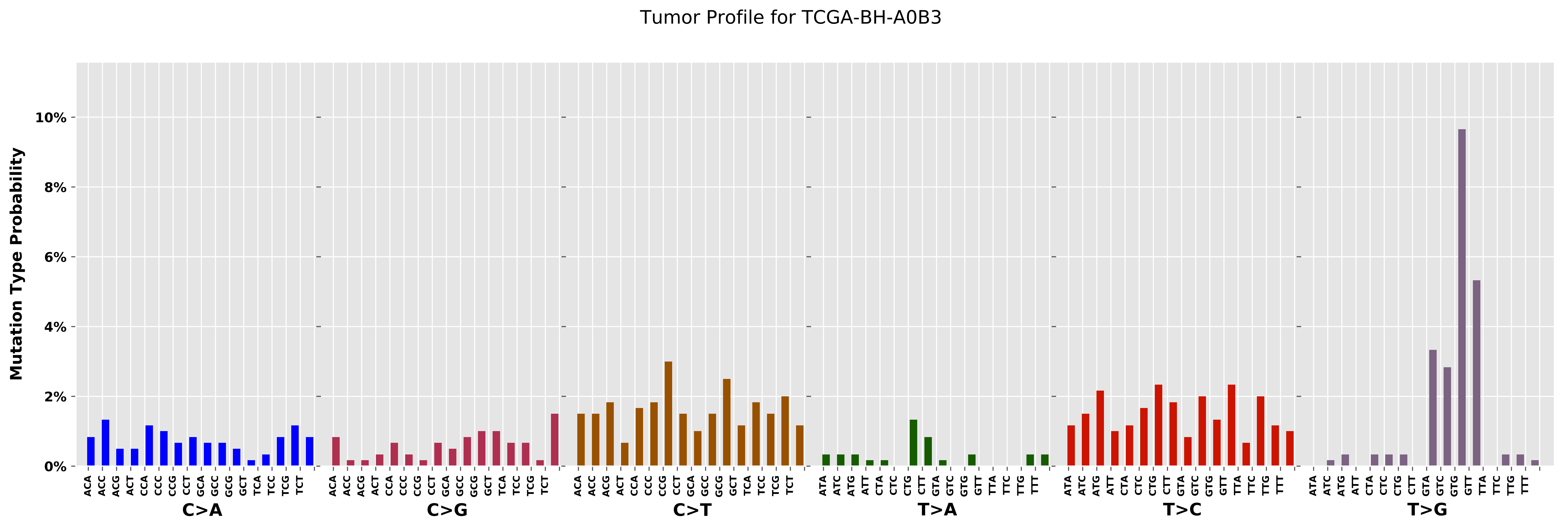

Tumor Profile for TCGA-BH-A0B3
Mutation Type Probability
0%
2%
4%
6%
8%
10%
C>A
C>G
C>T
T>A
T>C
T>G
ACA ACC ACG ACT CCA CCC CCG CCT GCA GCC GCG GCT TCA TCC TCG TCT
ATA ATC ATG ATT CTA CTC CTG CTT GTA GTC GTG GTT TTA TTC TTG TTT

Cancer processes Weights for TCGA-BH-A18U

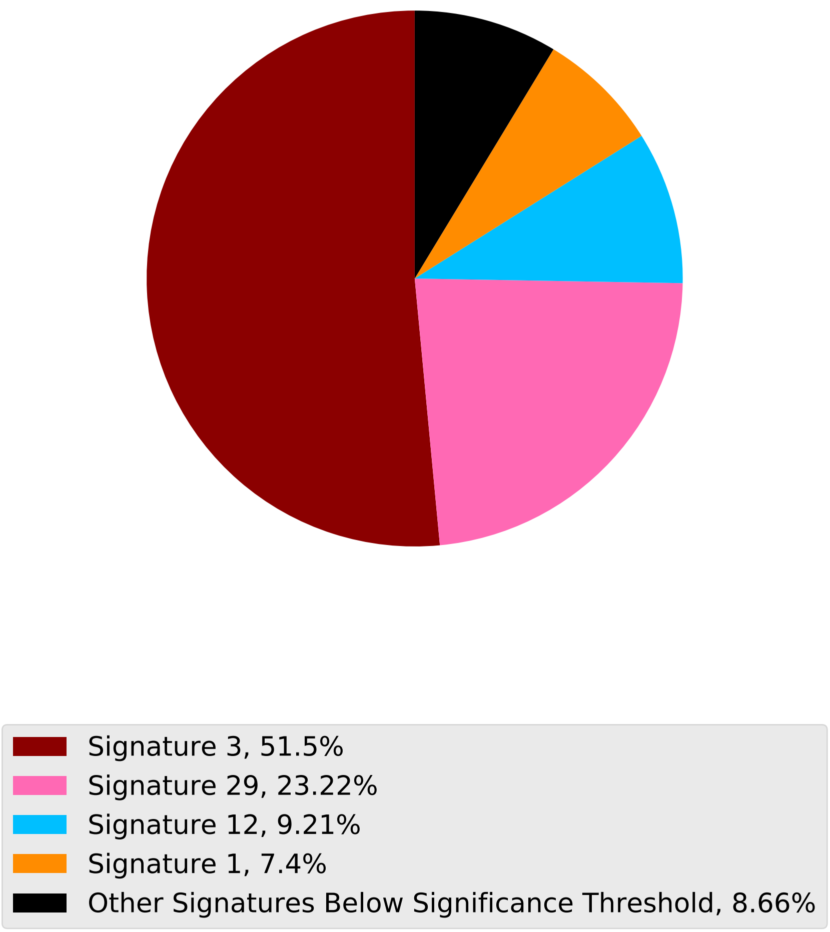

Tumor Profile for TCGA-BH-A18U

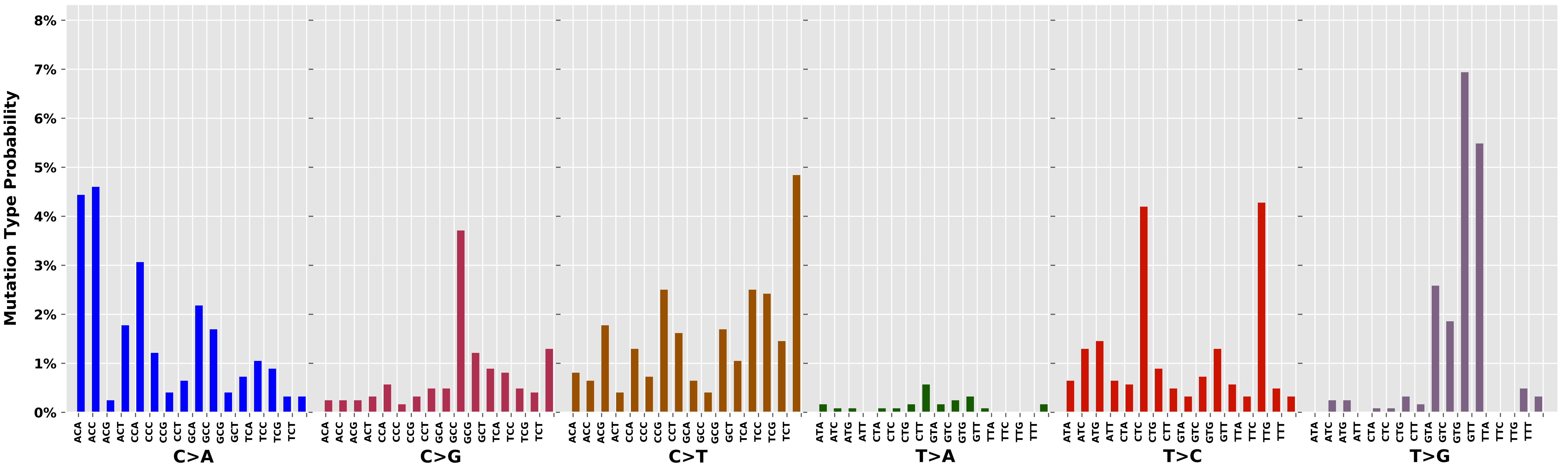

Cancer processes Weights for TCGA-GI-A2C9

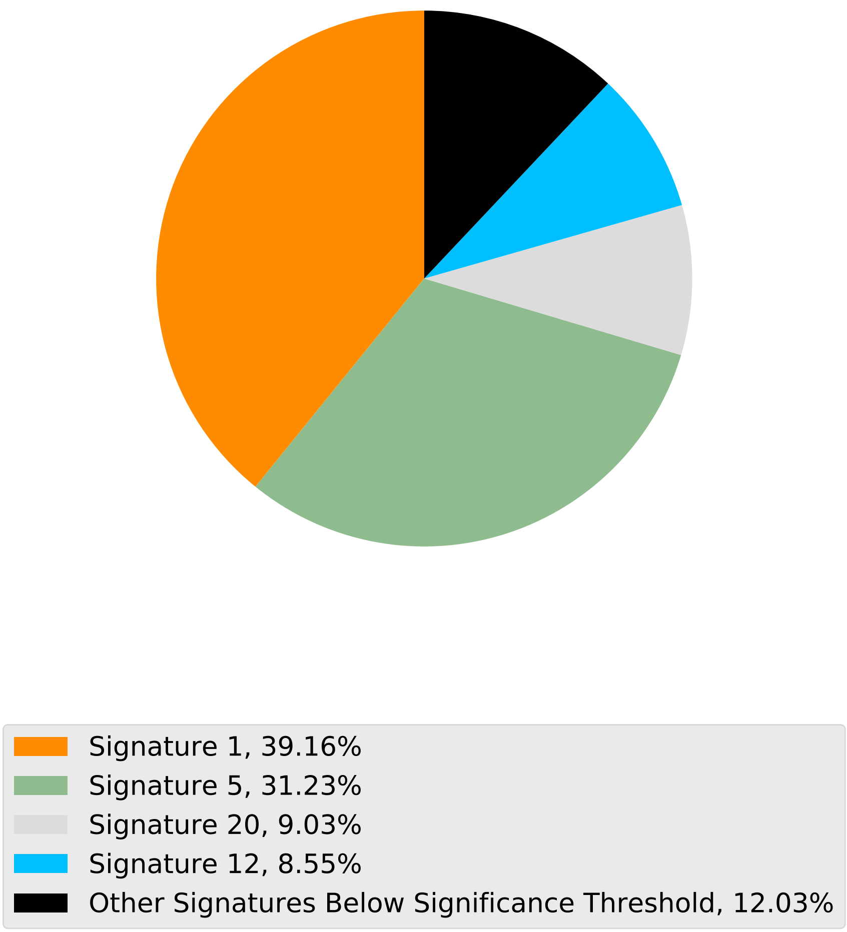

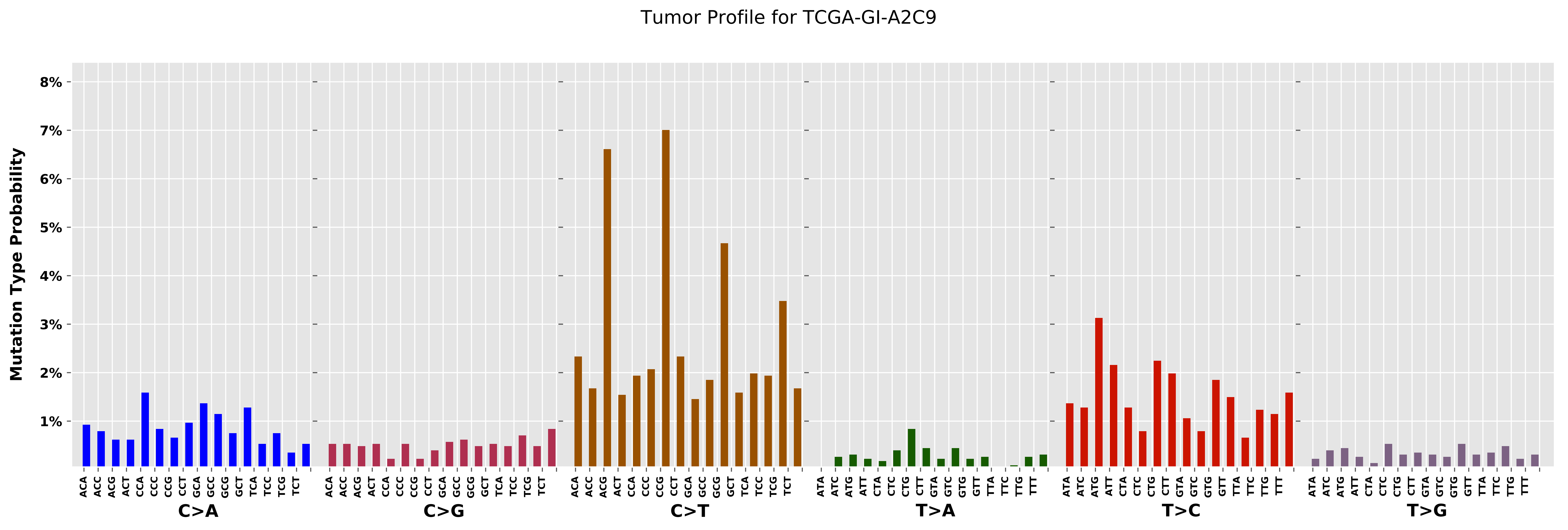

Tumor Profile for TCGA-GI-A2C9
Mutation Type Probability
8%
7%
6%
5%
4%
3%
2%
1%
C>A
C>G
C>T
T>A
T>C
T>G
ACA ACC ACG ACT CCA CCC CCG CCT GCA GCC GCG GCT TCA TCC TCG TCT
ATA ATC ATG ATT CTA CTC CTG CTT GTA GTC GTG GTT TTA TTC TTG TTT

Cancer processes Weights for TCGA-BH-A0DT

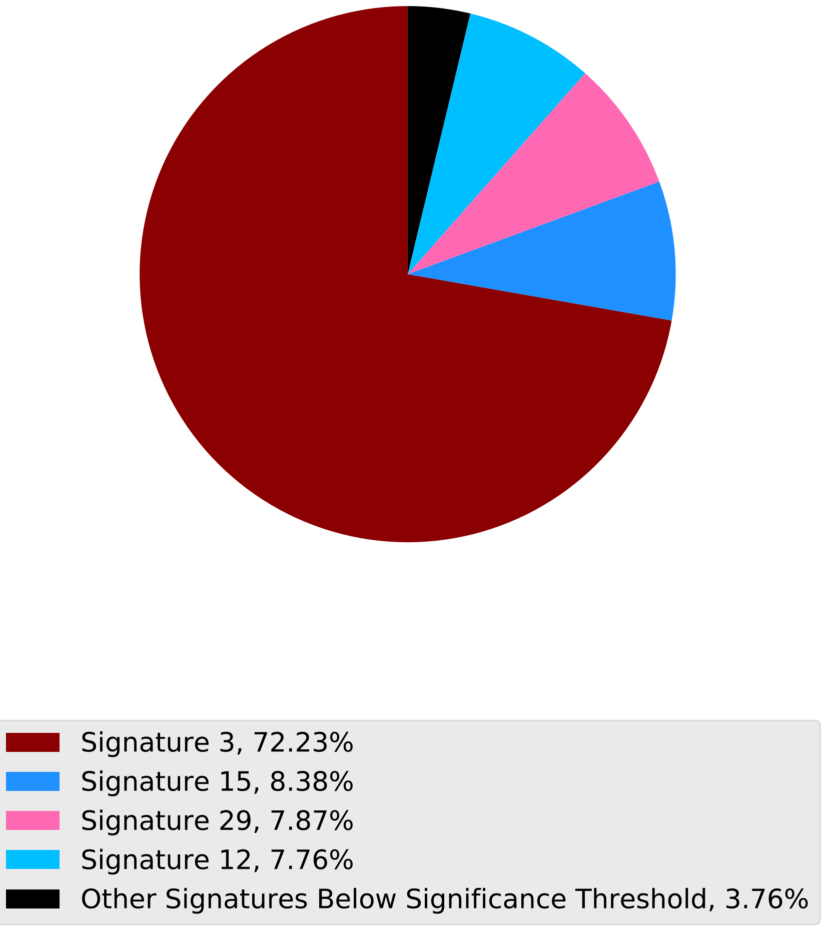

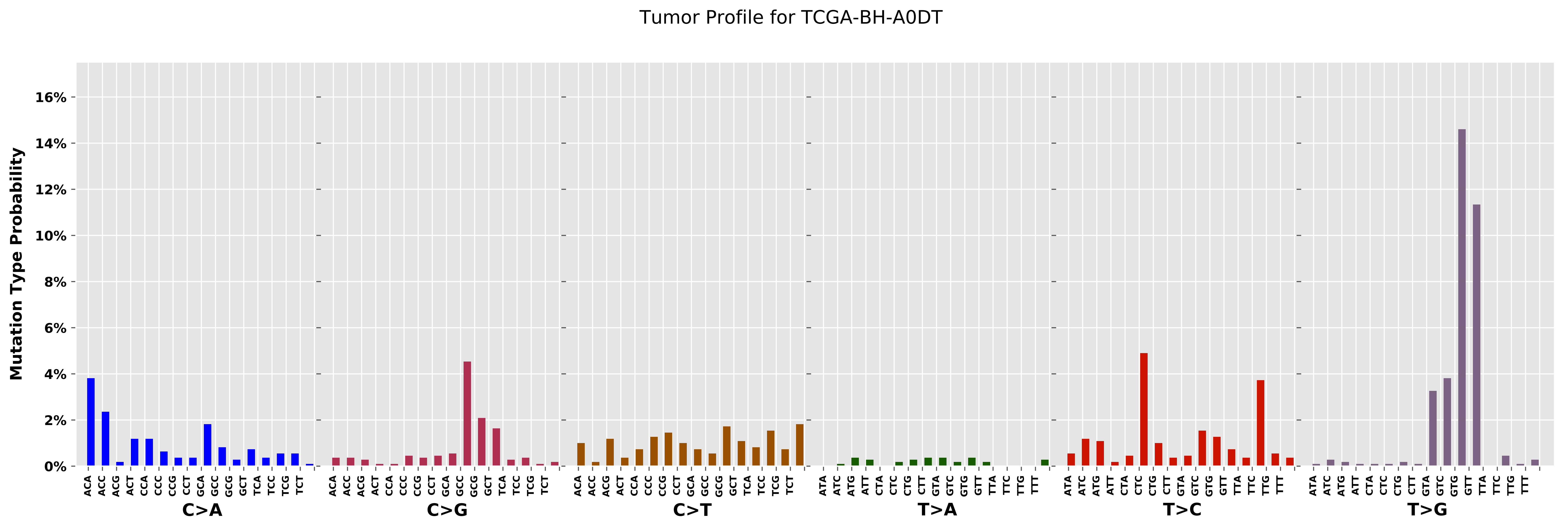

Tumor Profile for TCGA-BH-A0DT
Mutation Type Probability
16%
14%
12%
10%
8%
6%
4%
2%
0%
C>A
C>G
C>T
T>A
T>C
T>G
ACA ACC ACG ACT CCA CCC CCG CCT GCA GCC GCG GCT TCA TCC TCG TCT
ATA ATC ATG ATT CTA CTC CTG CTT GTA GTC GTG GTT TTA TTC TTG TTT

Cancer processes Weights for TCGA-A2-A0D4

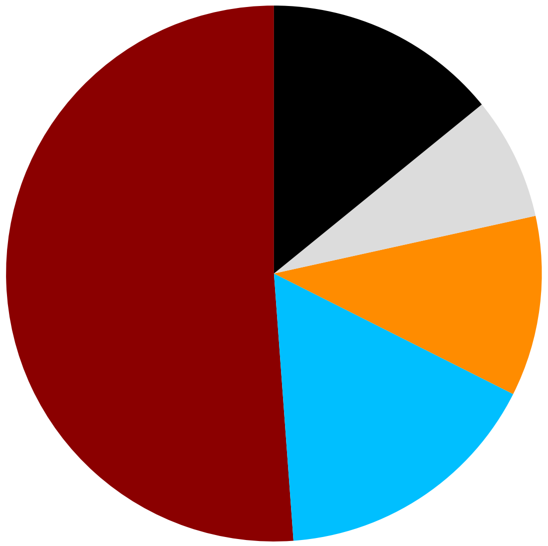

Tumor Profile for TCGA-A2-A0D4

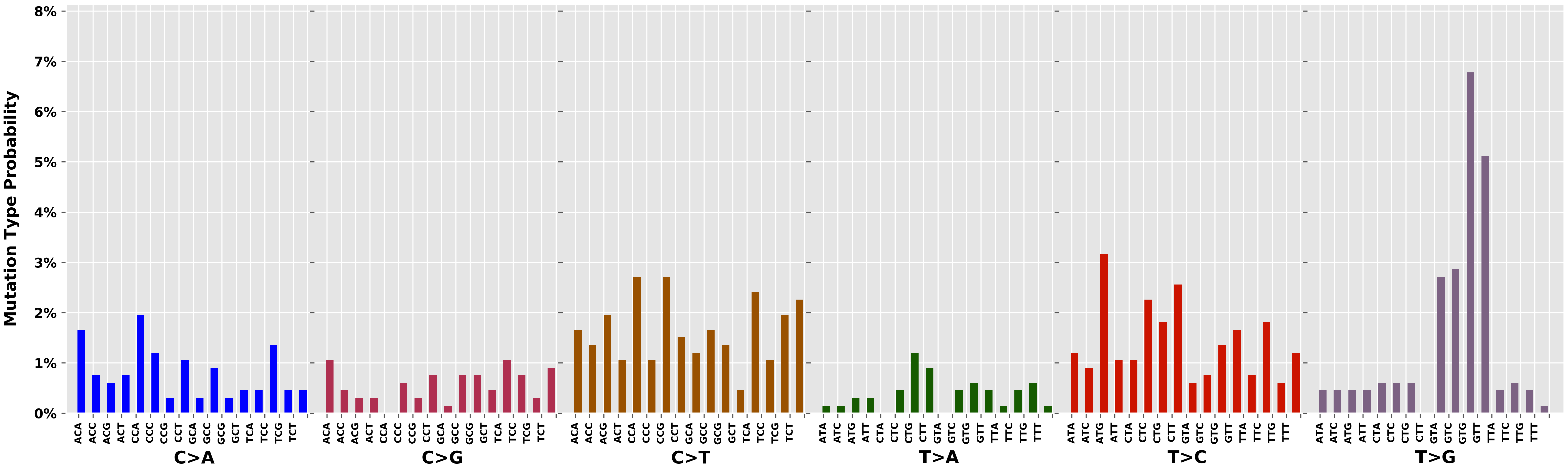

Cancer processes Weights for TCGA-EW-A1P8

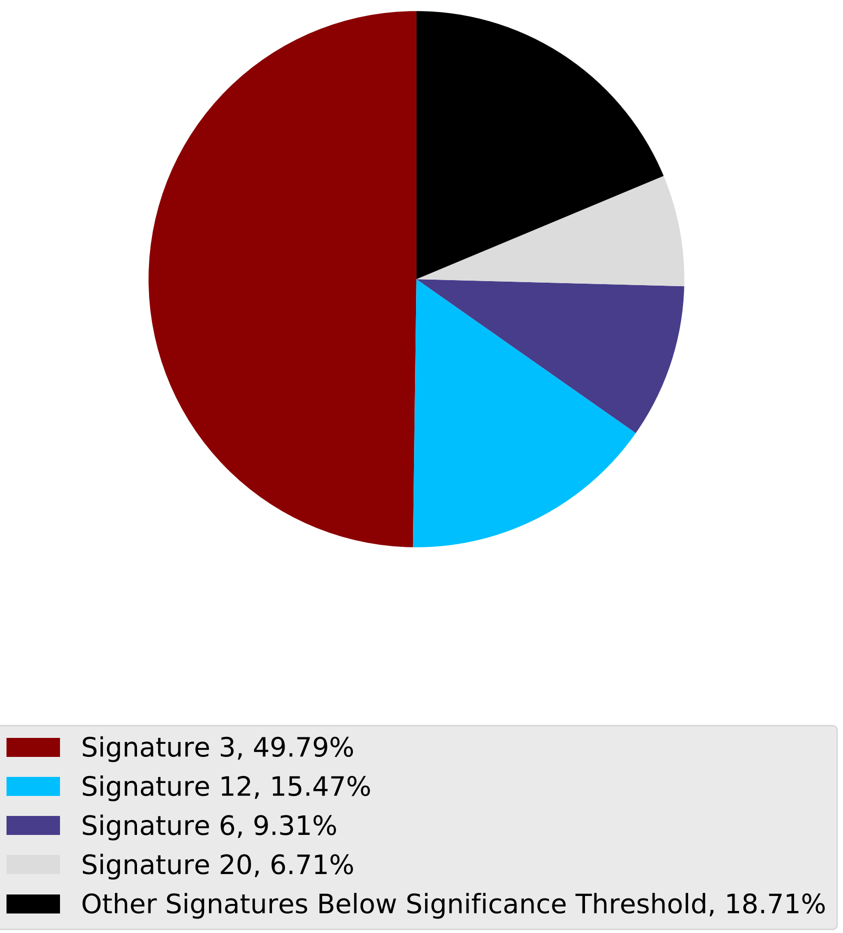

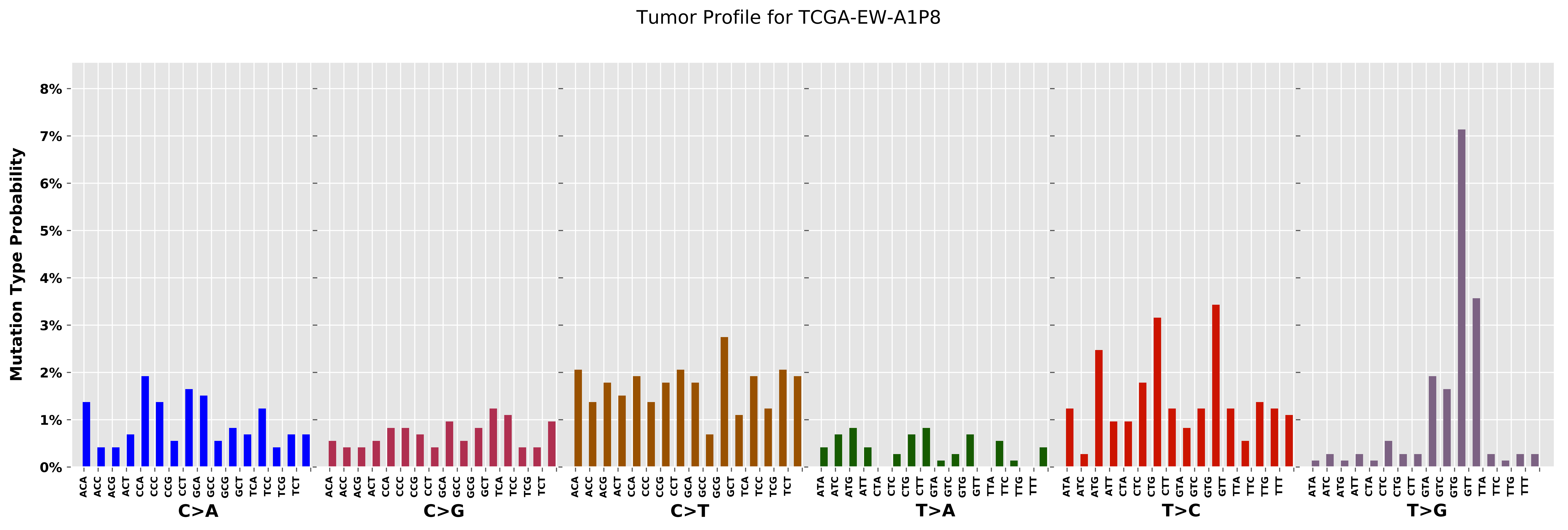
Tumor Profile for TCGA-EW-A1P8
Mutation Type Probability
0%
1%
2%
3%
4%
5%
6%
7%
8%
C>A
C>G
C>T
T>A
T>C
T>G
ACA ACC ACG ACT CCA CCC CCG CCT GCA GCC GCG GCT TCA TCC TCG TCT
ATA ATC ATG ATT CTA CTC CTG CTT GTA GTC GTG GTT TTA TTC TTG TTT

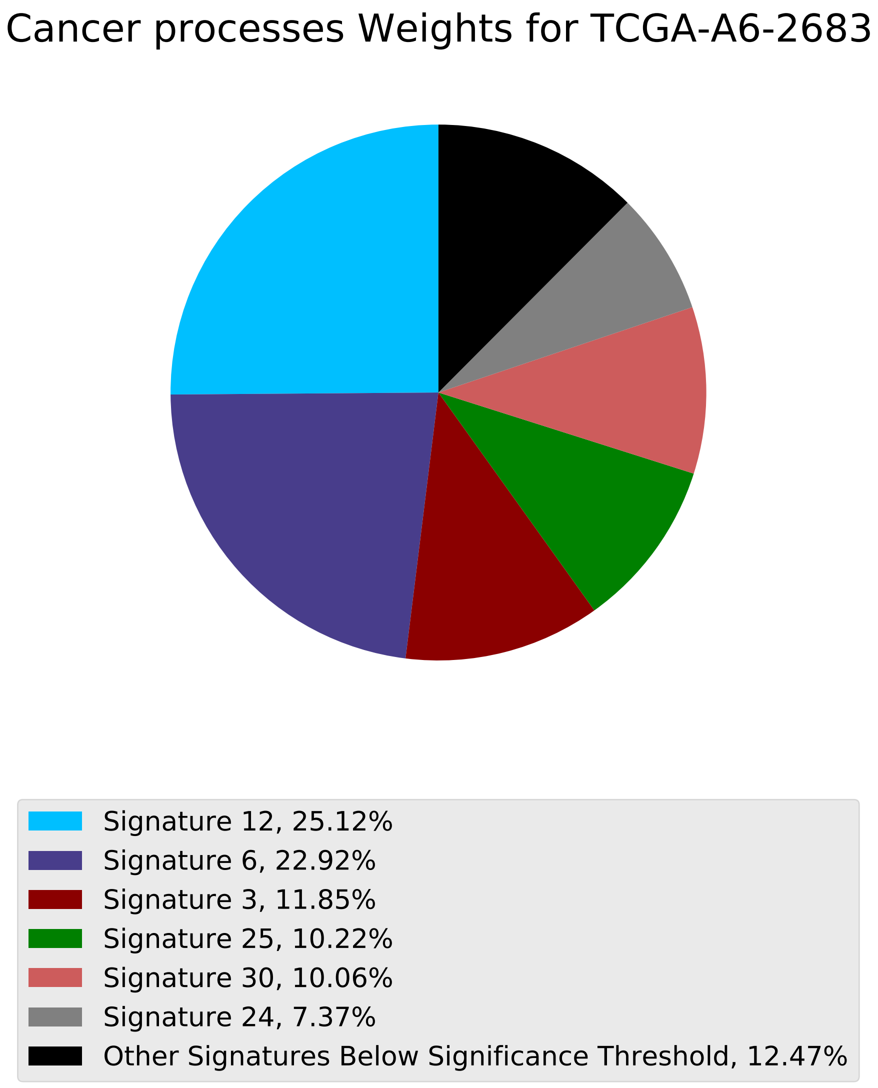
Cancer processes Weights for TCGA-A6-2683
Signature 12, 25.12%
Signature 6, 22.92%
Signature 3, 11.85%
Signature 25, 10.22%
Signature 30, 10.06%
Signature 24, 7.37%
Other Signatures Below Significance Threshold, 12.47%

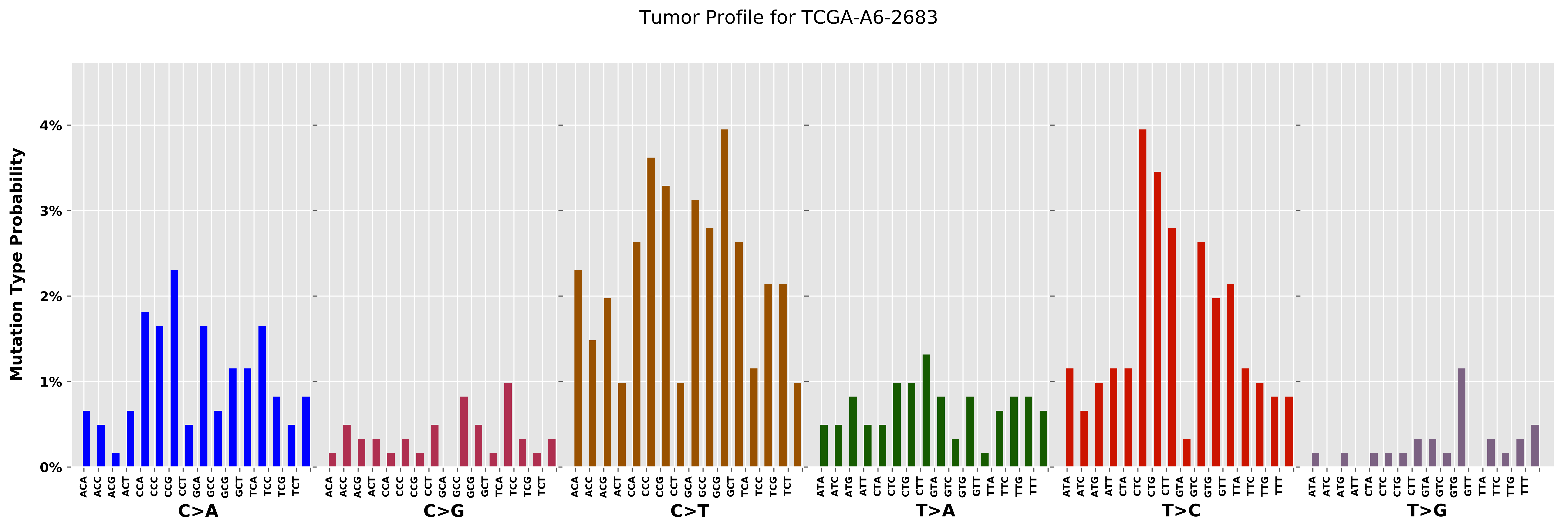
Tumor Profile for TCGA-A6-2683
Mutation Type Probability
0%
1%
2%
3%
4%
C>A
C>G
C>T
T>A
T>C
T>G
ACA ACC ACG ACT CCA CCC CCG CCT GCA GCC GCG GCT TCA TCC TCG TCT
ATA ATC ATG ATT CTA CTC CTG CTT GTA GTC GTG GTT TTA TTC TTG TTT

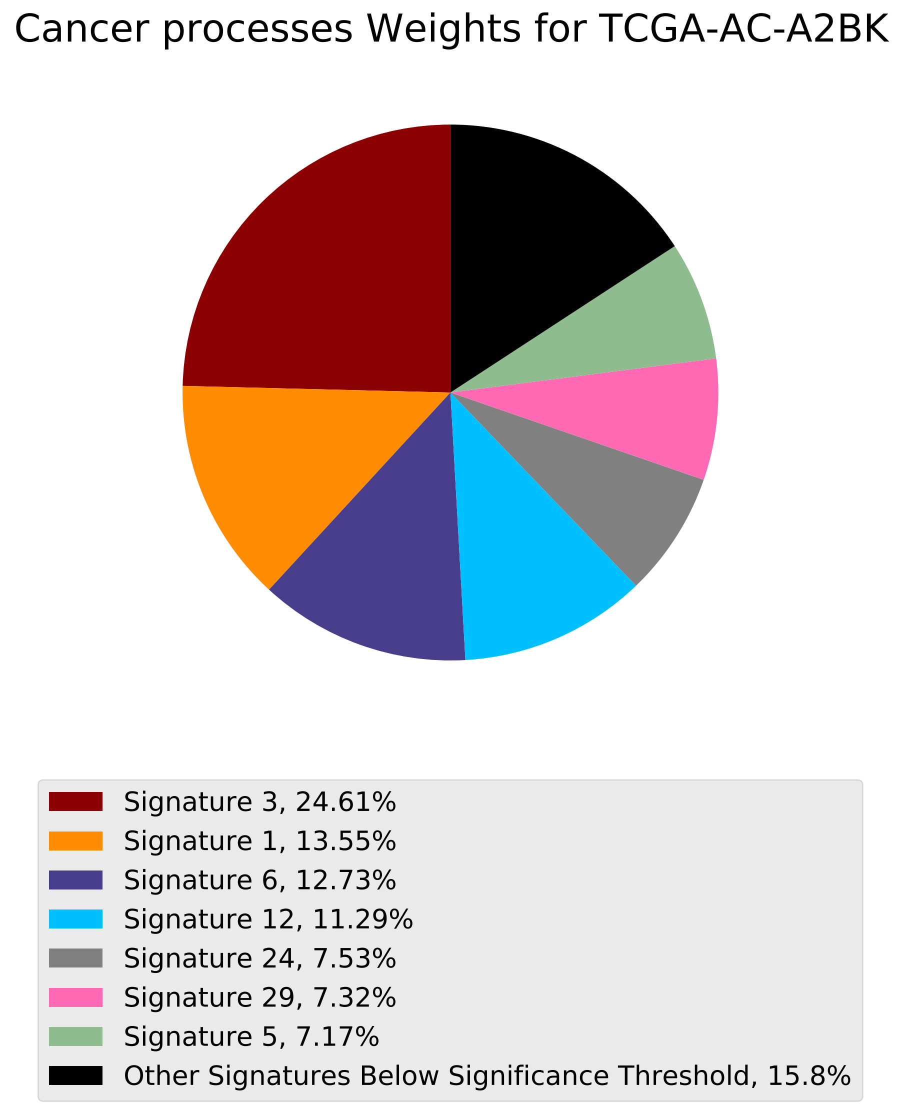
Cancer processes Weights for TCGA-AC-A2BK
Signature 3, 24.61%
Signature 1, 13.55%
Signature 6, 12.73%
Signature 12, 11.29%
Signature 24, 7.53%
Signature 29, 7.32%
Signature 5, 7.17%
Other Signatures Below Significance Threshold, 15.8%

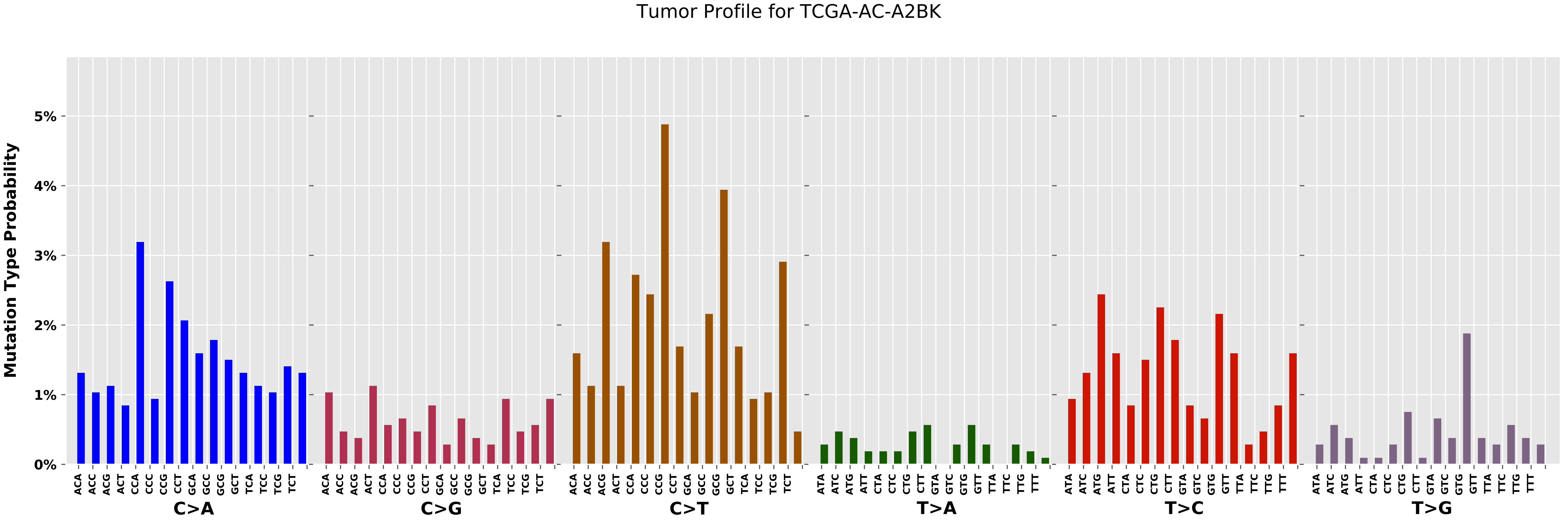

Tumor Profile for TCGA-AC-A2BK
Mutation Type Probability
0%
1%
2%
3%
4%
5%
ACA ACC ACG ACT CCA CCC CCG CCT GCA GCC GCG GCT TCA TCC TCG TCT
ATA ATC ATG ATT CTA CTC CTG CTT GTA GTC GTG GTT TTA TTC TTG TTT
C>A
C>G
C>T
T>A
T>C
T>G

Cancer processes Weights for TCGA-AG-A032

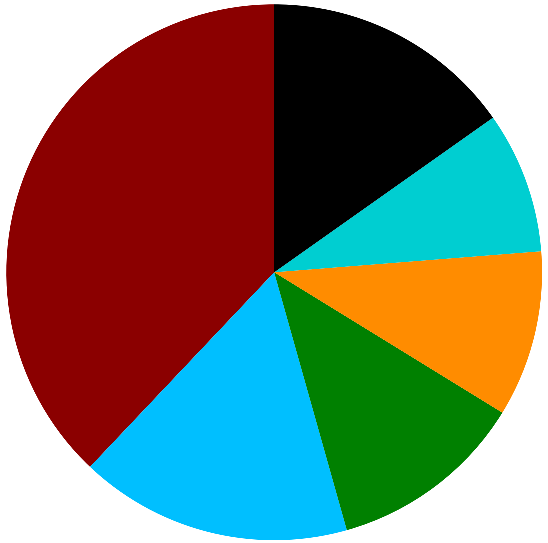

- Signature 3, 37.93%
- Signature 12, 16.44%
- Signature 25, 11.85%
- Signature 1, 10.03%
- Signature 9, 8.54%
- Other Signatures Below Significance Threshold, 15.21%

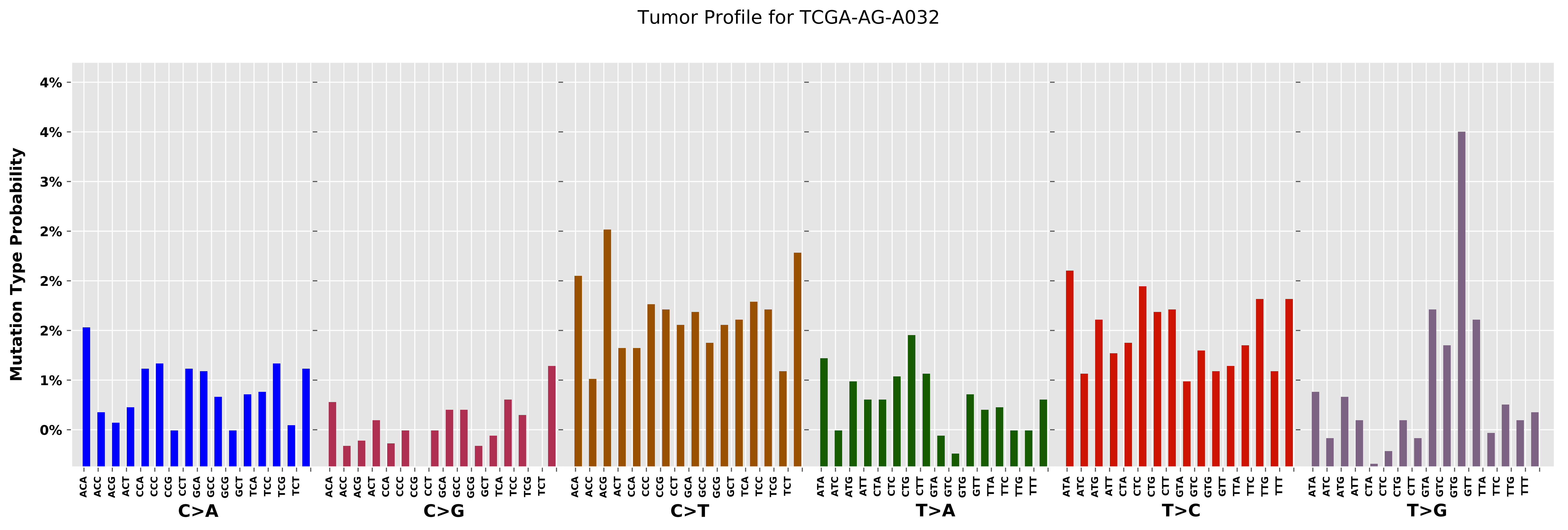

Tumor Profile for TCGA-AG-A032
Mutation Type Probability
4%
4%
3%
2%
2%
2%
1%
0%
C>A
C>G
C>T
T>A
T>C
T>G
ACA ACC ACG ACT CCA CCC CCG CCT GCA GCC GCG GCT TCA TCC TCG TCT
ATA ATC ATG ATT CTA CTC CTG CTT GTA GTC GTG GTT TTA TTC TTG TTT

Cancer processes Weights for TCGA-B6-A0I2

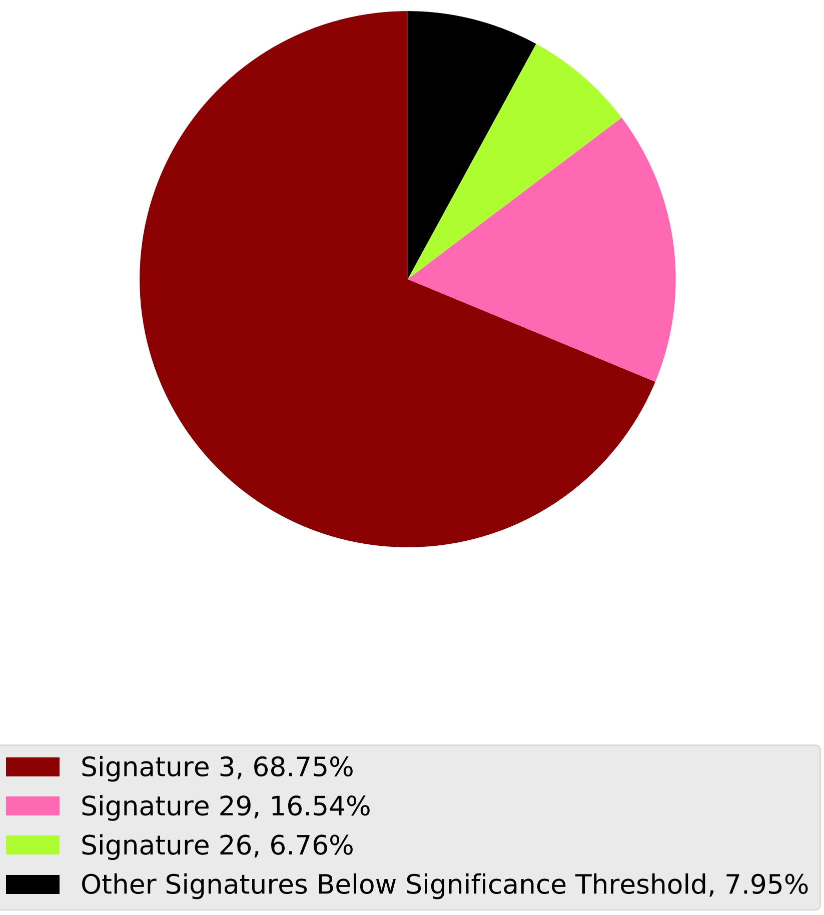

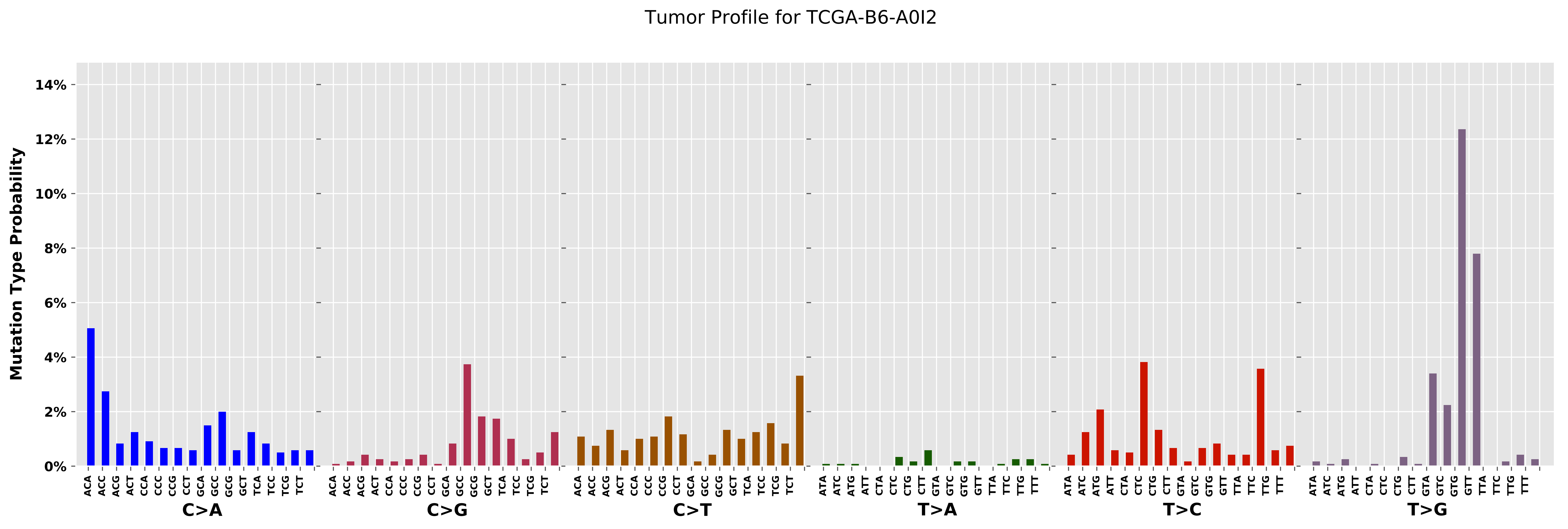
Tumor Profile for TCGA-B6-A0I2
Mutation Type Probability
14%
12%
10%
8%
6%
4%
2%
0%
C>A
C>G
C>T
T>A
T>C
T>G

Cancer processes Weights for TCGA-A8-A094

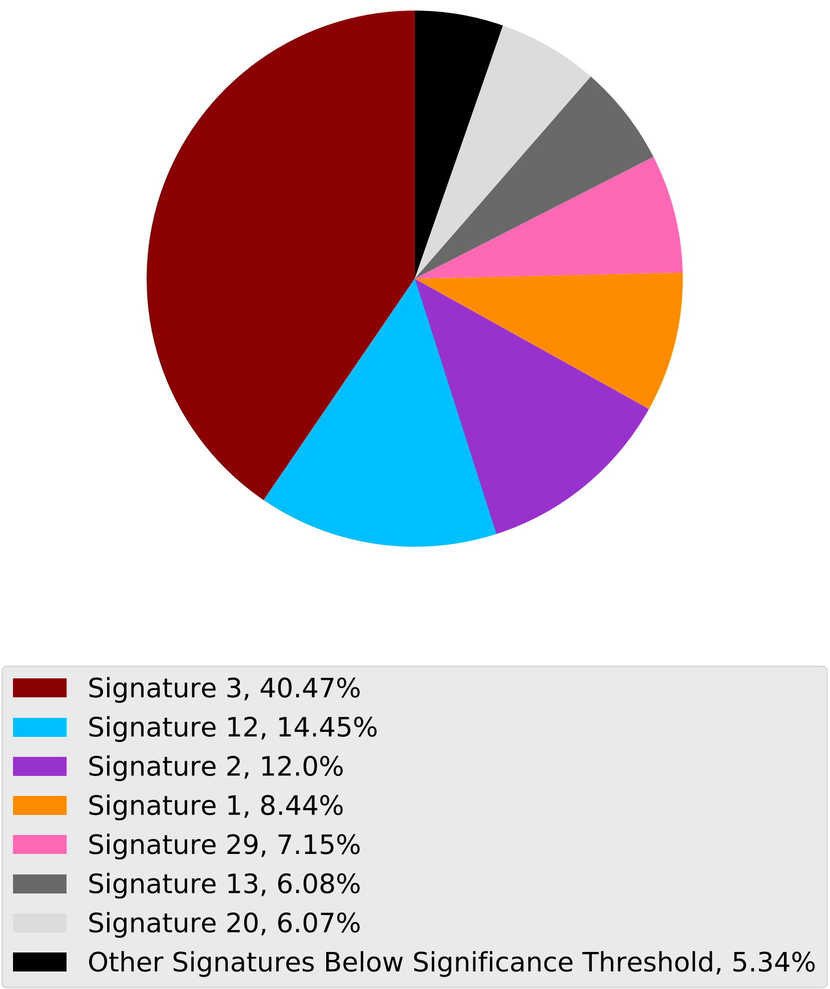

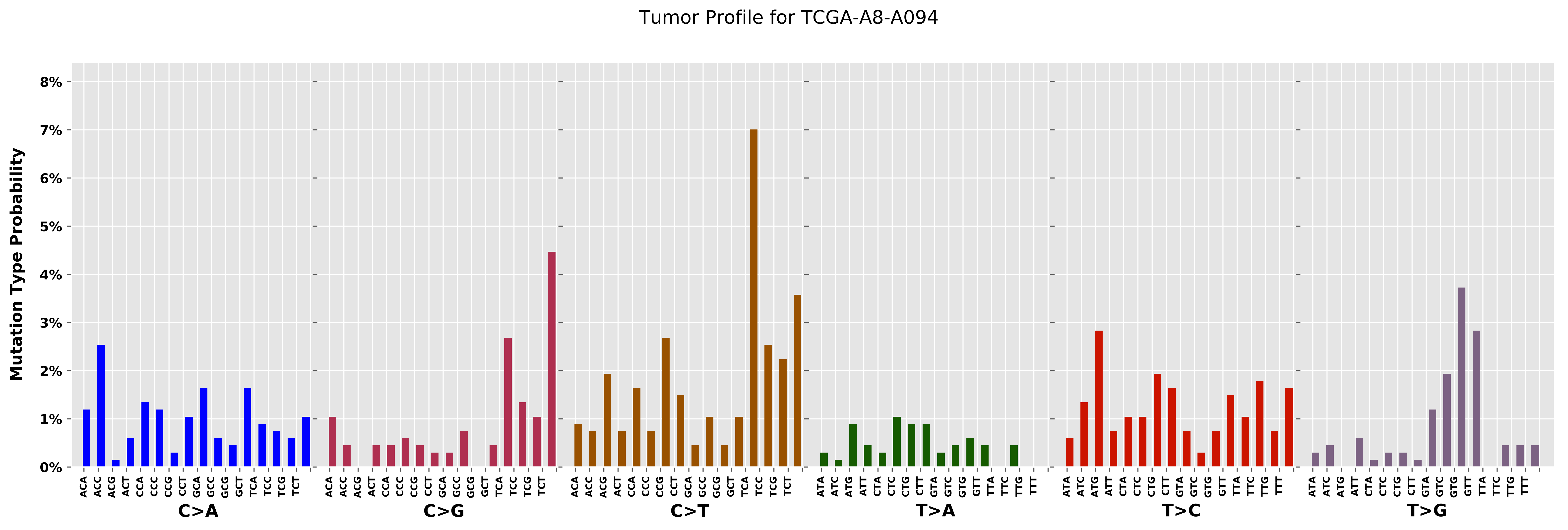

Tumor Profile for TCGA-A8-A094
Mutation Type Probability
8%
7%
6%
5%
4%
3%
2%
1%
0%
C>A
C>G
C>T
T>A
T>C
T>G

Cancer processes Weights for TCGA-AA-3994

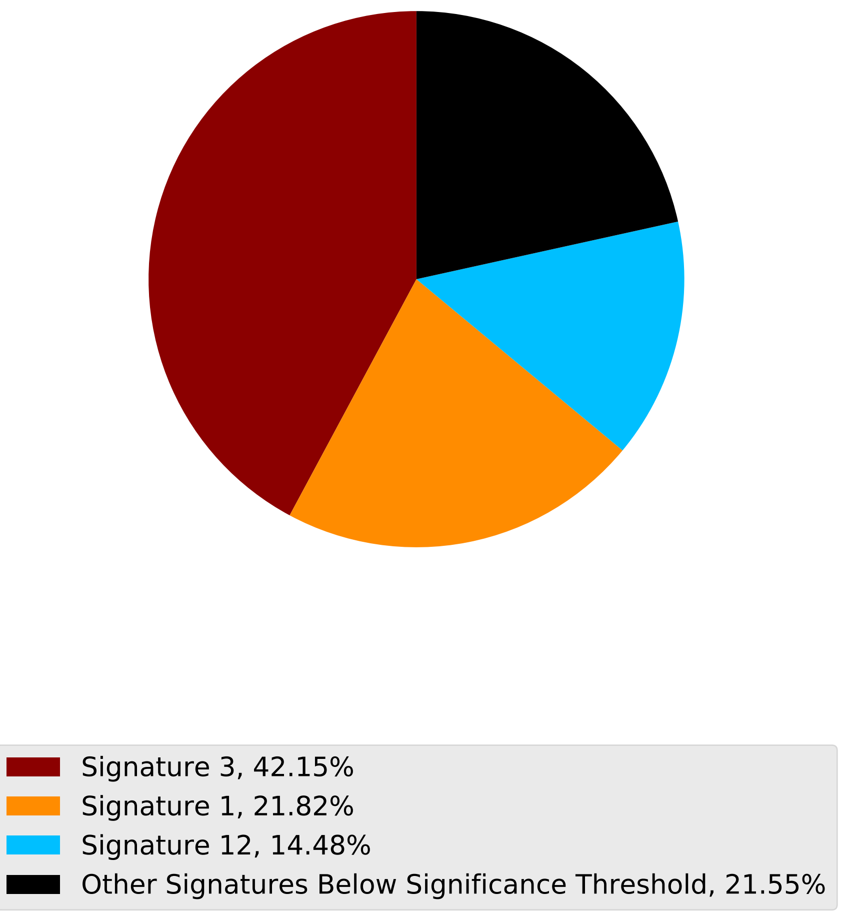

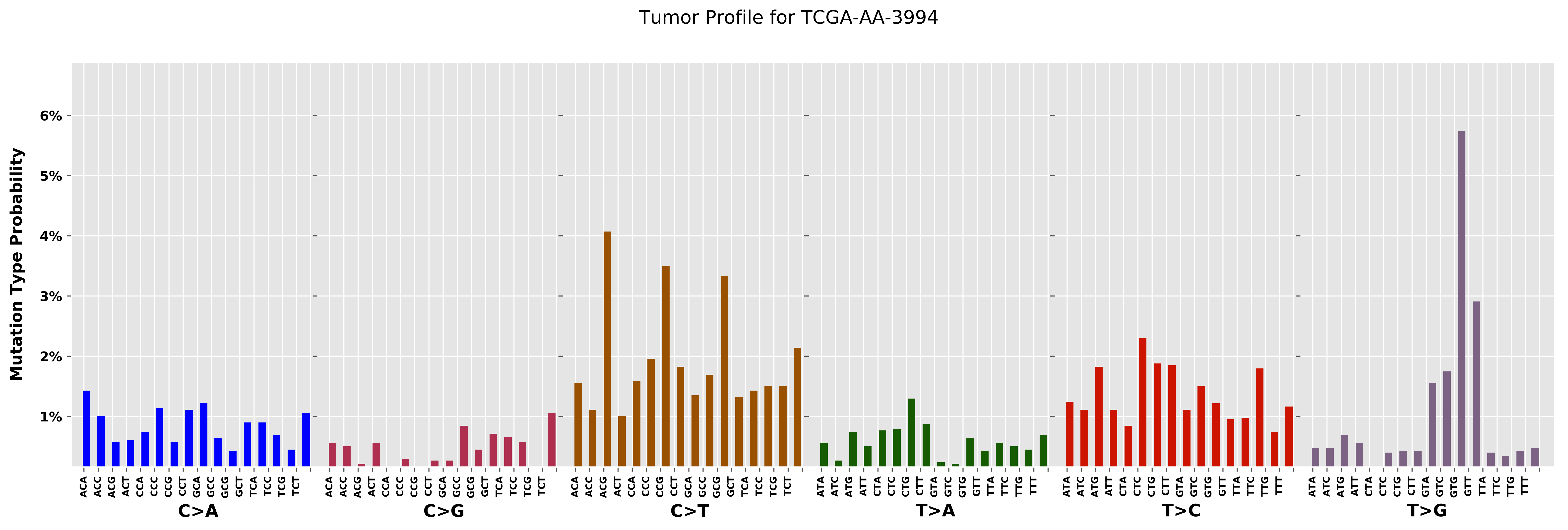

Tumor Profile for TCGA-AA-3994
Mutation Type Probability
6%
5%
4%
3%
2%
1%
C>A
C>G
C>T
T>A
T>C
T>G

Cancer processes Weights for TCGA-AG-3574

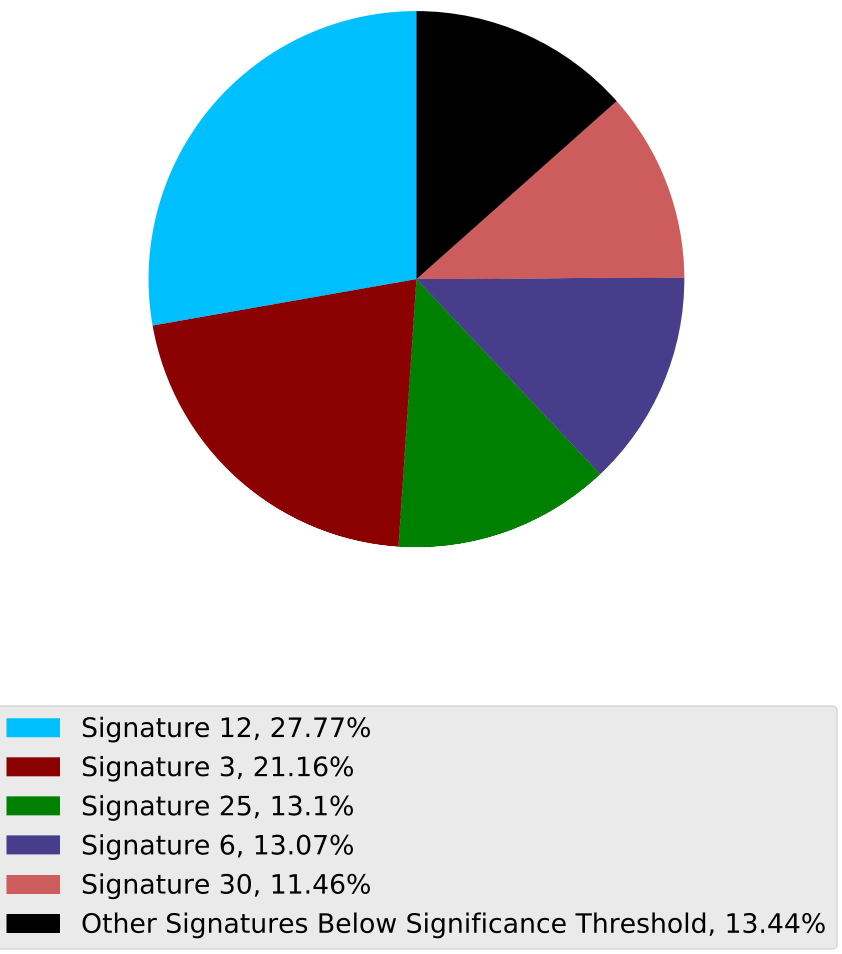

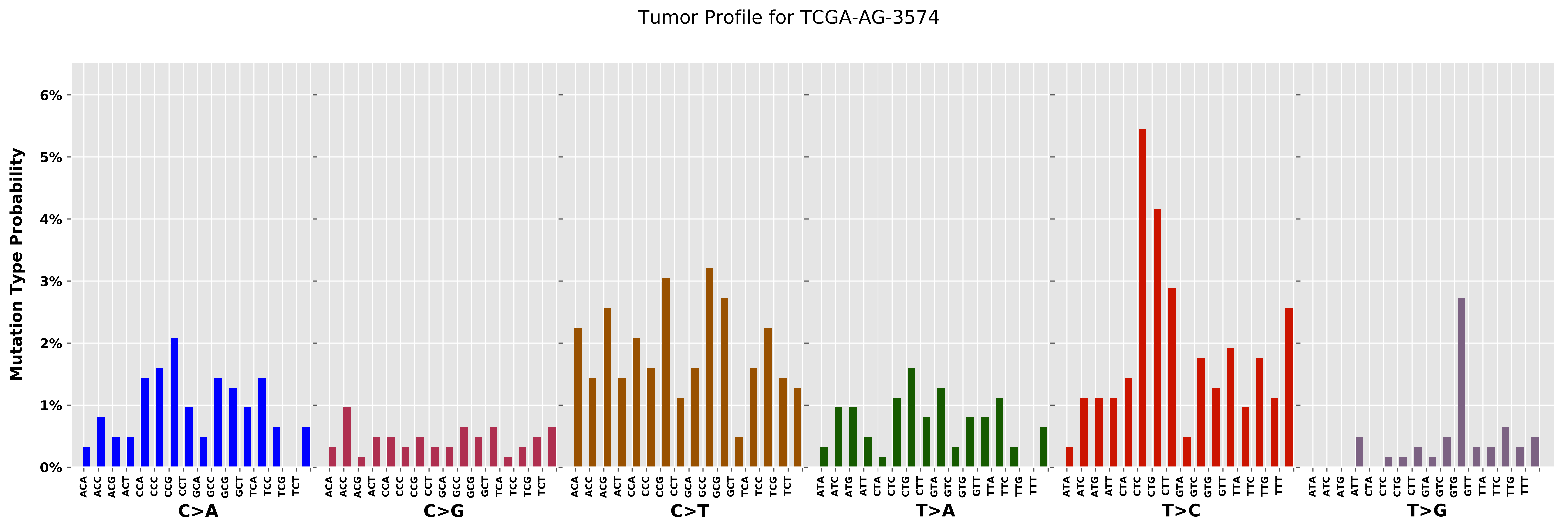

Tumor Profile for TCGA-AG-3574
Mutation Type Probability
0%
1%
2%
3%
4%
5%
6%
C>A
C>G
C>T
T>A
T>C
T>G
ACA ACC ACG ACT CCA CCC CCG CCT GCA GCC GCG GCT TCA TCC TCG TCT
ATA ATC ATG ATT CTA CTC CTG CTT GTA GTC GTG GTT TTA TTC TTG TTT

Cancer processes Weights for TCGA-AO-A03L

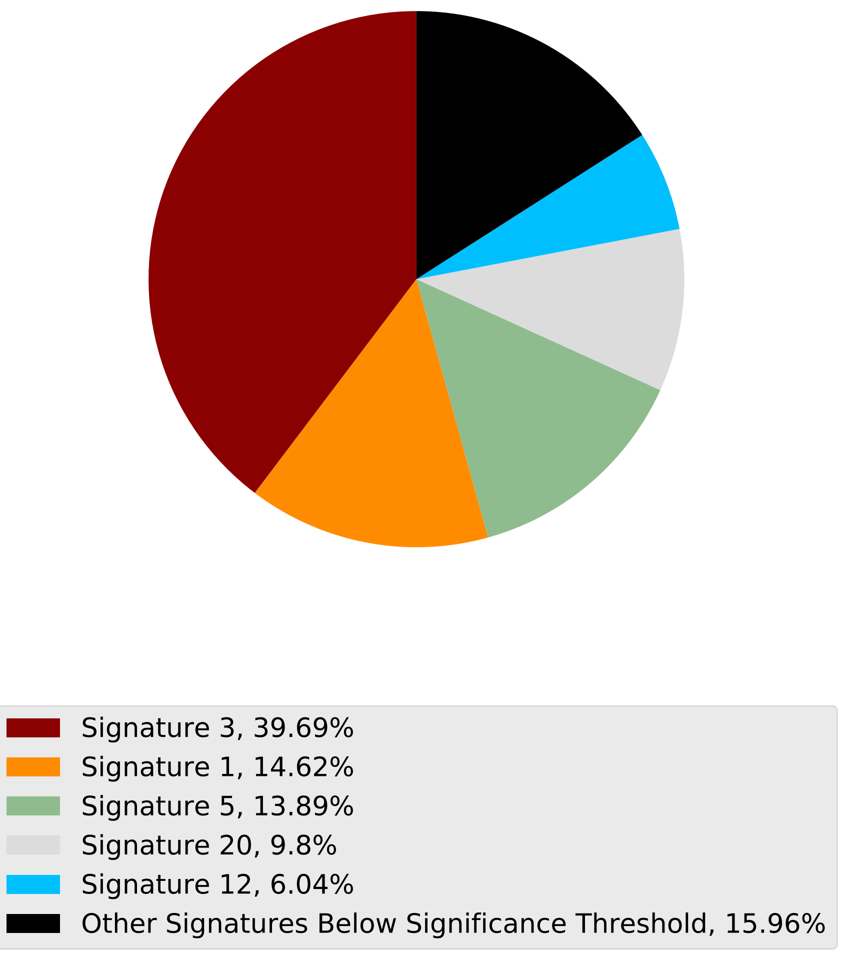

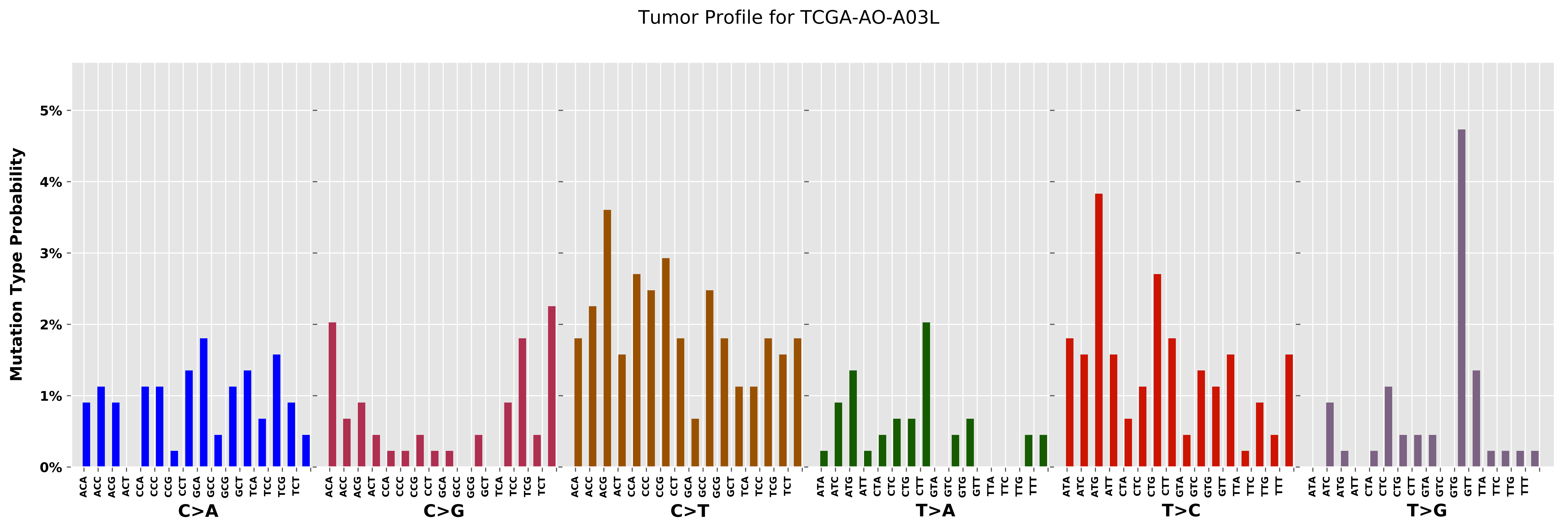

Tumor Profile for TCGA-AO-A03L
Mutation Type Probability
0%
1%
2%
3%
4%
5%
C>A
C>G
C>T
T>A
T>C
T>G
ACA ACC ACG ACT CCA CCC CCG CCT GCA GCC GCG GCT TCA TCC TCG TCT
ATA ATC ATG ATT CTA CTC CTG CTT GTA GTC GTG GTT TTA TTC TTG TTT

Cancer processes Weights for TCGA-A6-6141

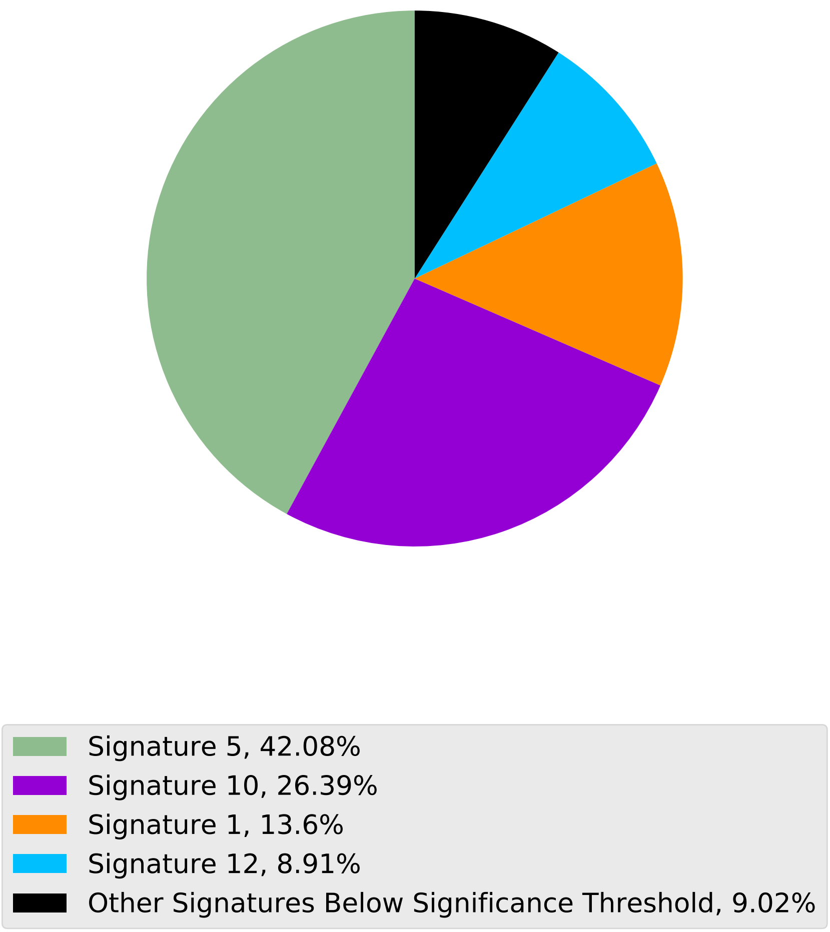

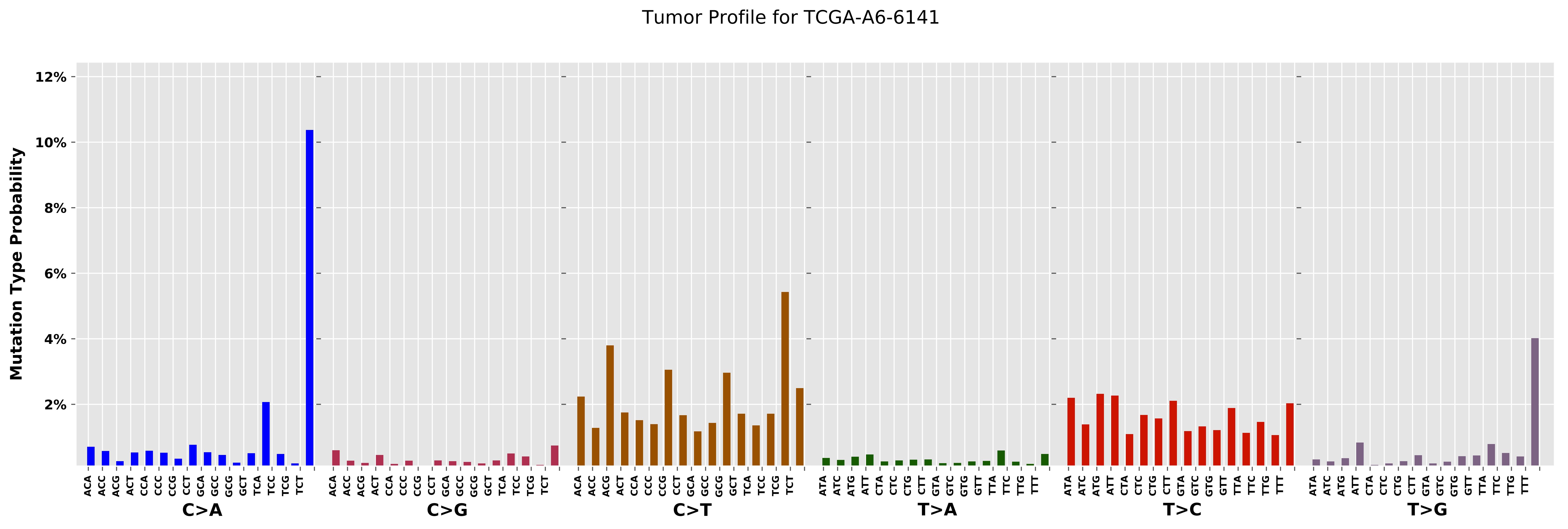

Tumor Profile for TCGA-A6-6141
Mutation Type Probability
12%
10%
8%
6%
4%
2%
C>A
C>G
C>T
T>A
T>C
T>G
ACA ACC ACG ACT CCA CCC CCG CCT GCA GCC GCG GCT TCA TCC TCG TCT
ATA ATC ATG ATT CTA CTC CTG CTT GTA GTC GTG GTT TTA TTC TTG TTT

Cancer processes Weights for TCGA-AA-A02O

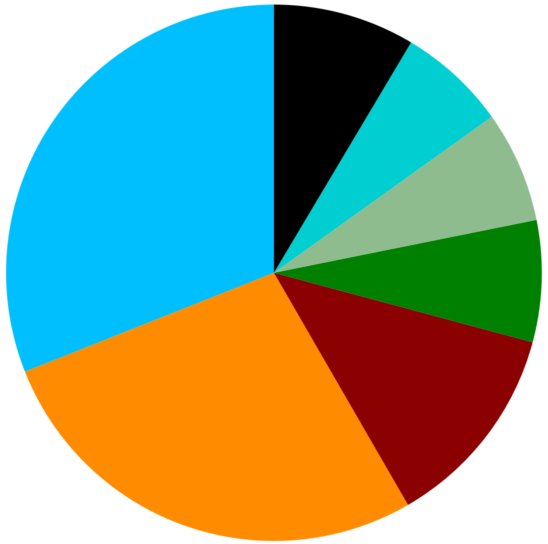

Tumor Profile for TCGA-AA-A02O

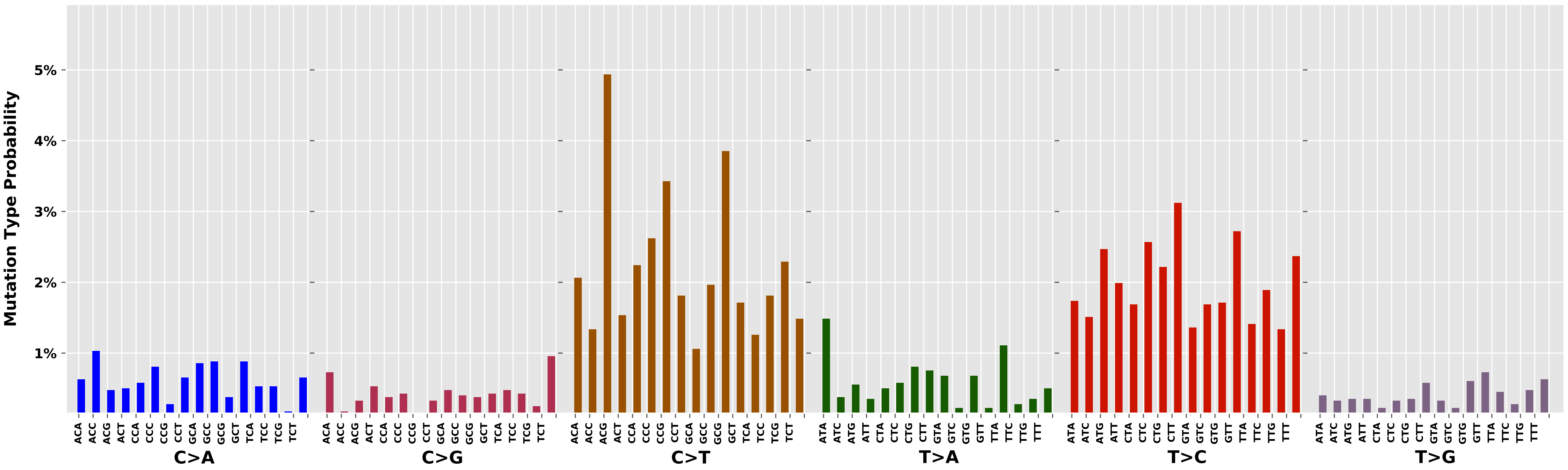

Cancer processes Weights for TCGA-AG-3885

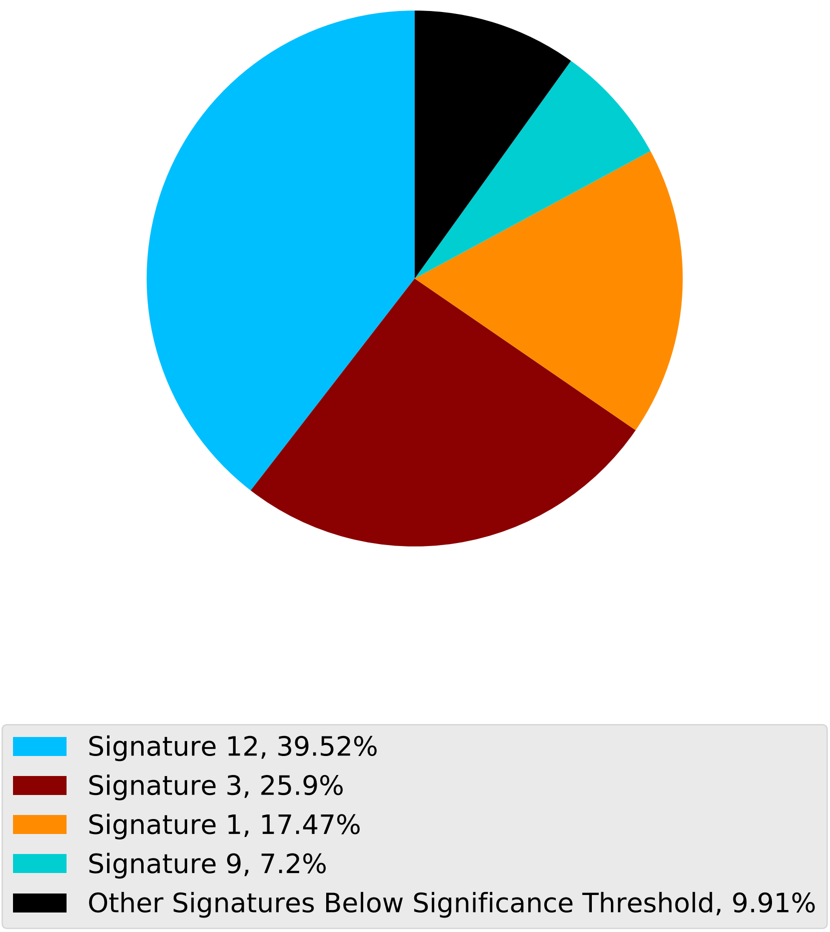

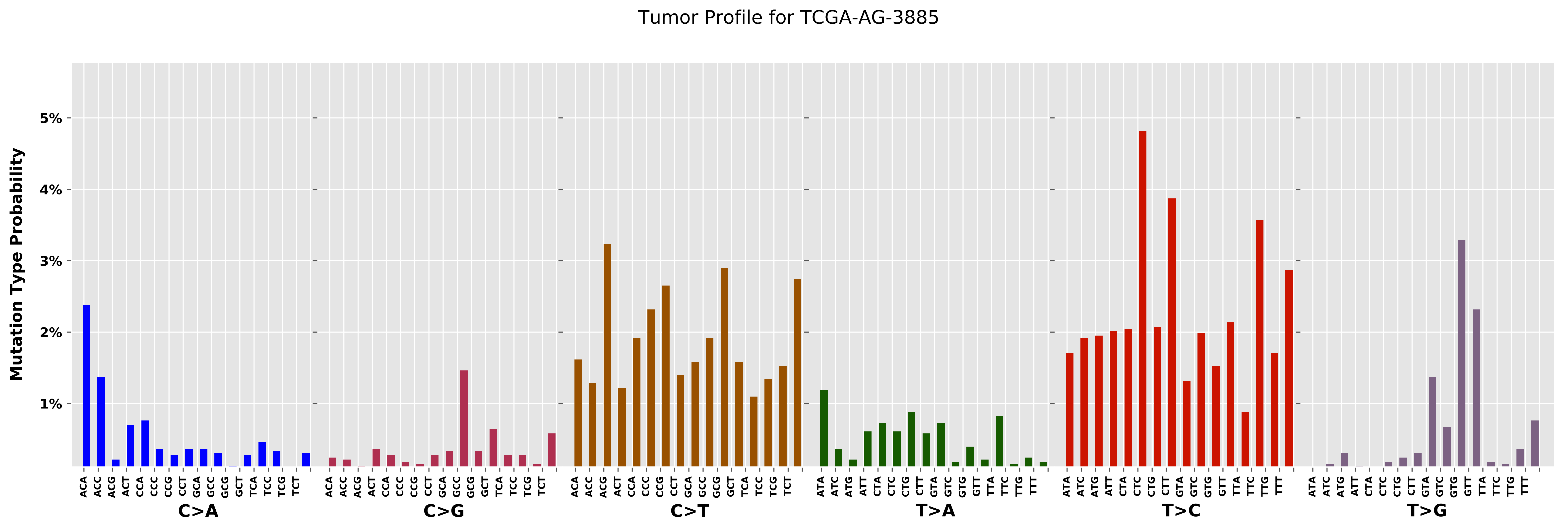
Tumor Profile for TCGA-AG-3885
Mutation Type Probability
5%
4%
3%
2%
1%
C>A
C>G
C>T
T>A
T>C
T>G

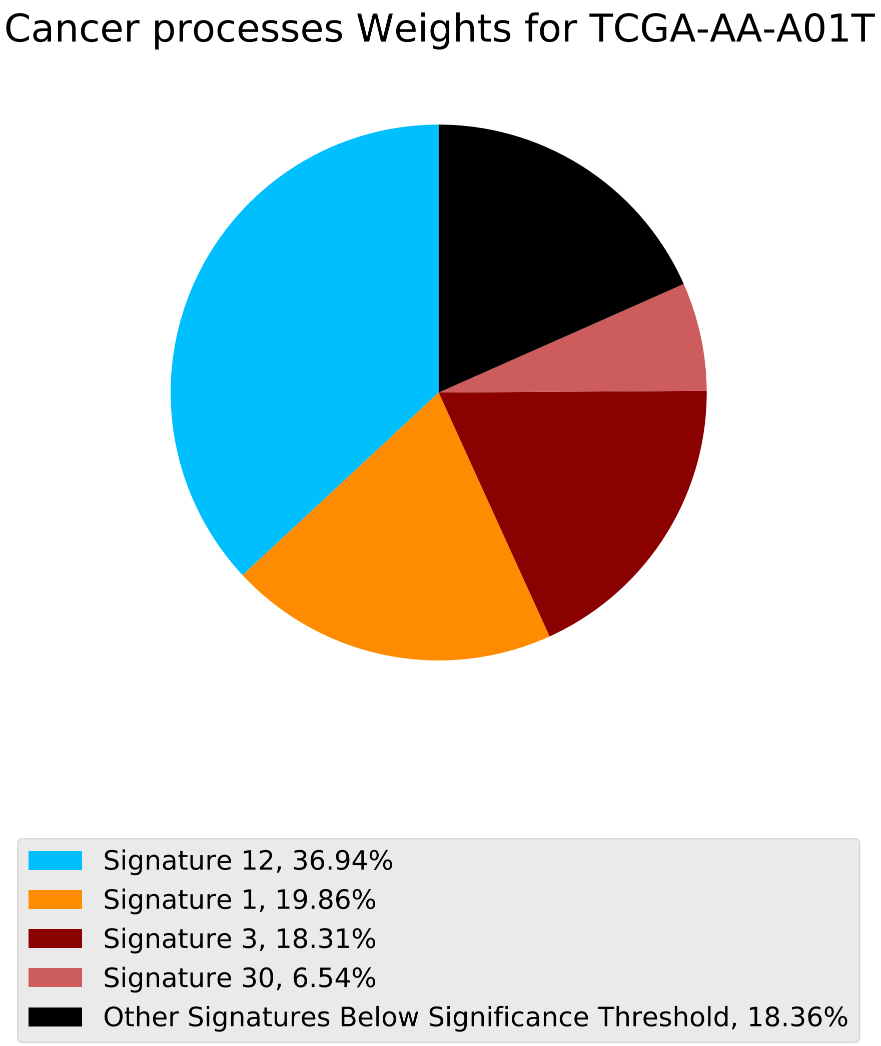
Cancer processes Weights for TCGA-AA-A01T
Signature 12, 36.94%
Signature 1, 19.86%
Signature 3, 18.31%
Signature 30, 6.54%
Other Signatures Below Significance Threshold, 18.36%

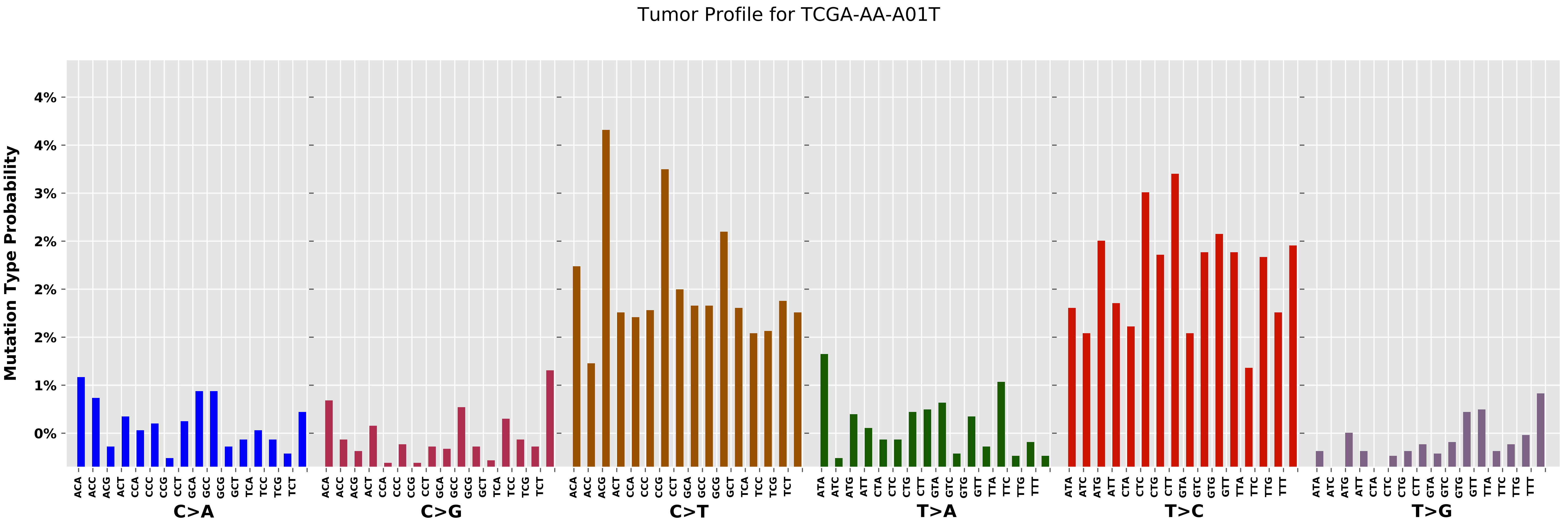

Tumor Profile for TCGA-AA-A01T
Mutation Type Probability
4%
4%
3%
2%
2%
2%
1%
0%
ACA ACC ACG ACT CCA CCC CCG CCT GCA GCC GCG GCT TCA TCC TCG TCT
C>A
ACA ACC ACG ACT CCA CCC CCG CCT GCA GCC GCG GCT TCA TCC TCG TCT
C>G
ACA ACC ACG ACT CCA CCC CCG CCT GCA GCC GCG GCT TCA TCC TCG TCT
C>T
ATA ATC ATG ATT CTA CTC CTG CTT GTA GTC GTG GTT TTA TTC TTG TTT
T>A
ATA ATC ATG ATT CTA CTC CTG CTT GTA GTC GTG GTT TTA TTC TTG TTT
T>C
ATA ATC ATG ATT CTA CTC CTG CTT GTA GTC GTG GTT TTA TTC TTG TTT
T>G

Cancer processes Weights for TCGA-A8-A07I

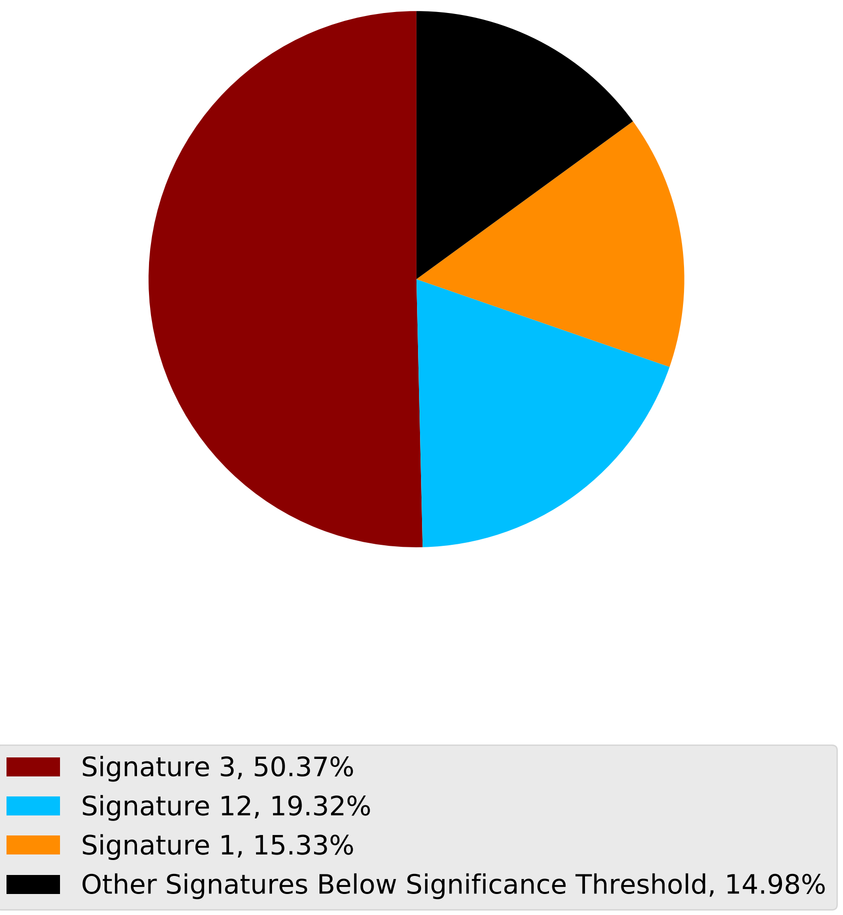

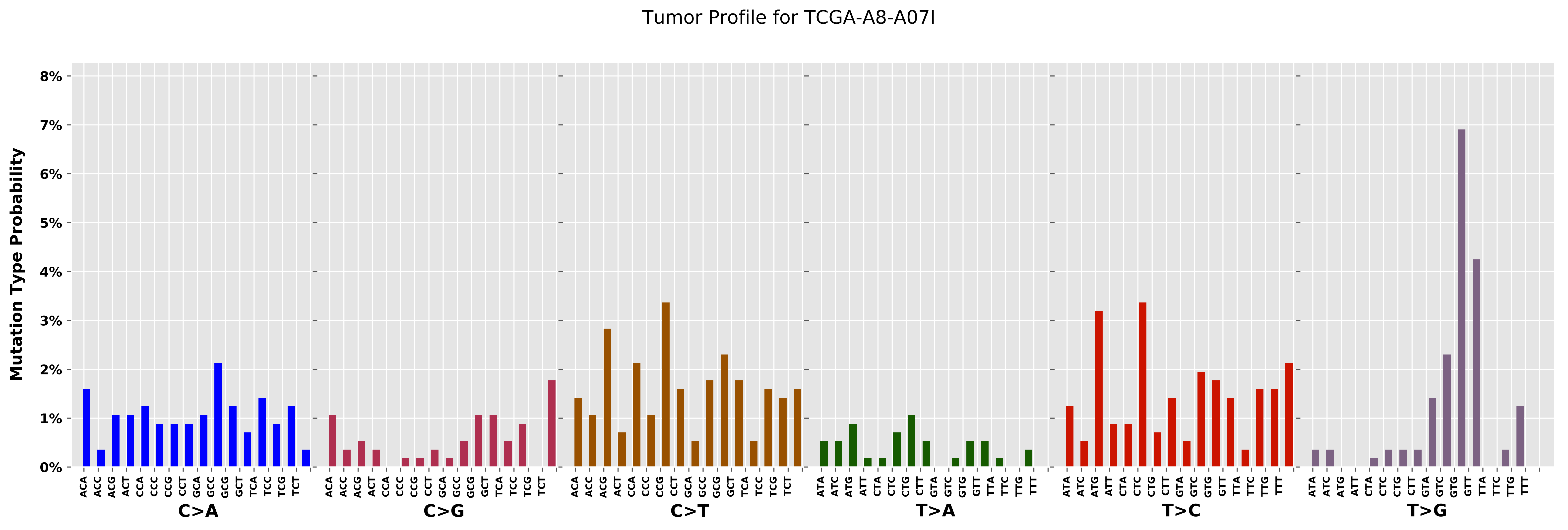

Tumor Profile for TCGA-A8-A07I
Mutation Type Probability
0%
1%
2%
3%
4%
5%
6%
7%
8%
C>A
C>G
C>T
T>A
T>C
T>G
ACA ACC ACG ACT CCA CCC CCG CCT GCA GCC GCG GCT TCA TCC TCG TCT
ATA ATC ATG ATT CTA CTC CTG CTT GTA GTC GTG GTT TTA TTC TTG TTT

Cancer processes Weights for TCGA-A6-2681

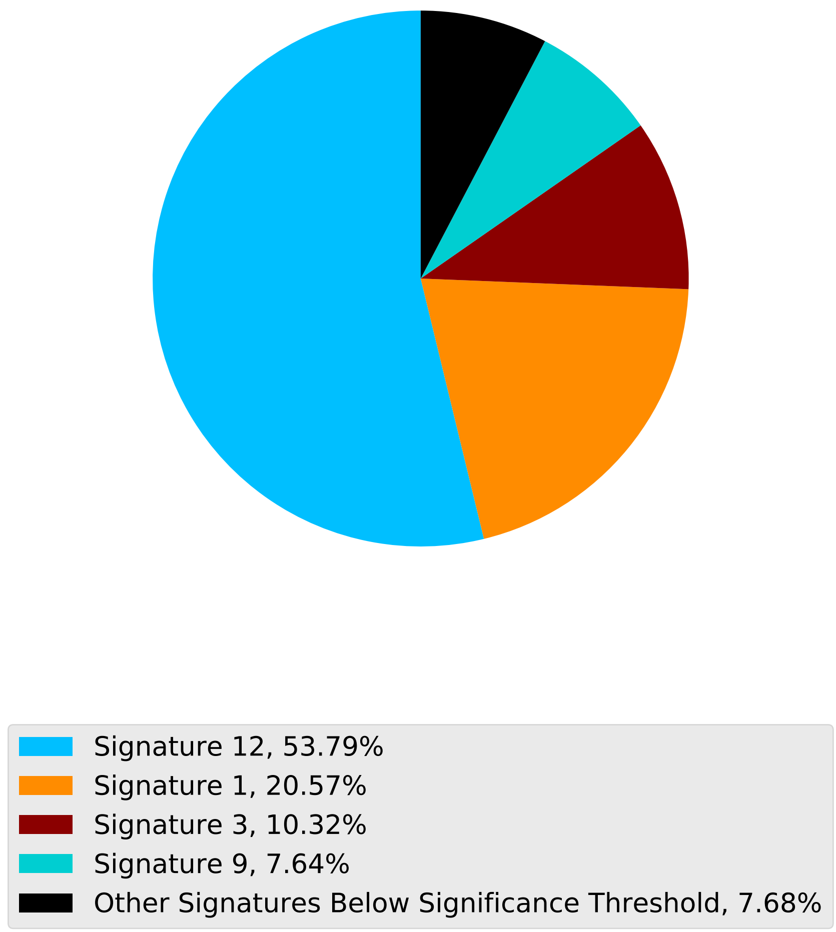

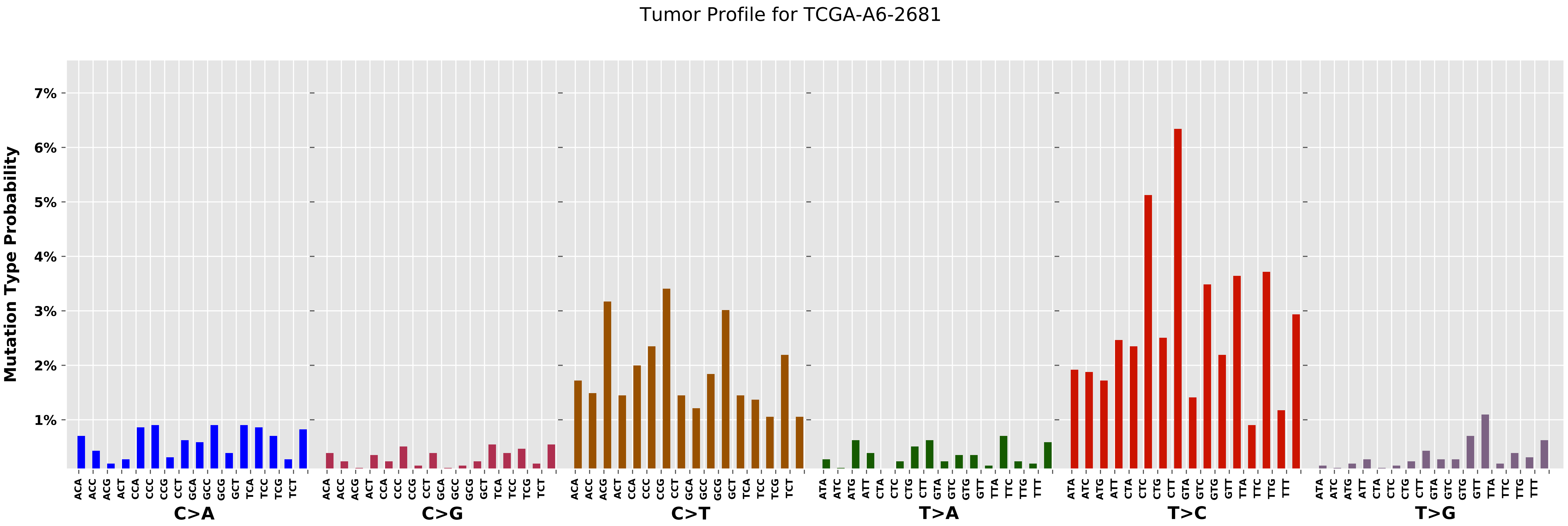
Tumor Profile for TCGA-A6-2681
Mutation Type Probability
1%
2%
3%
4%
5%
6%
7%
C>A
C>G
C>T
T>A
T>C
T>G
ACA ACC ACG ACT CCA CCC CCG CCT GCA GCC GCG GCT TCA TCC TCG TCT
ATA ATC ATG ATT CTA CTC CTG CTT GTA GTC GTG GTT TTA TTC TTG TTT

Cancer processes Weights for TCGA-AO-A124

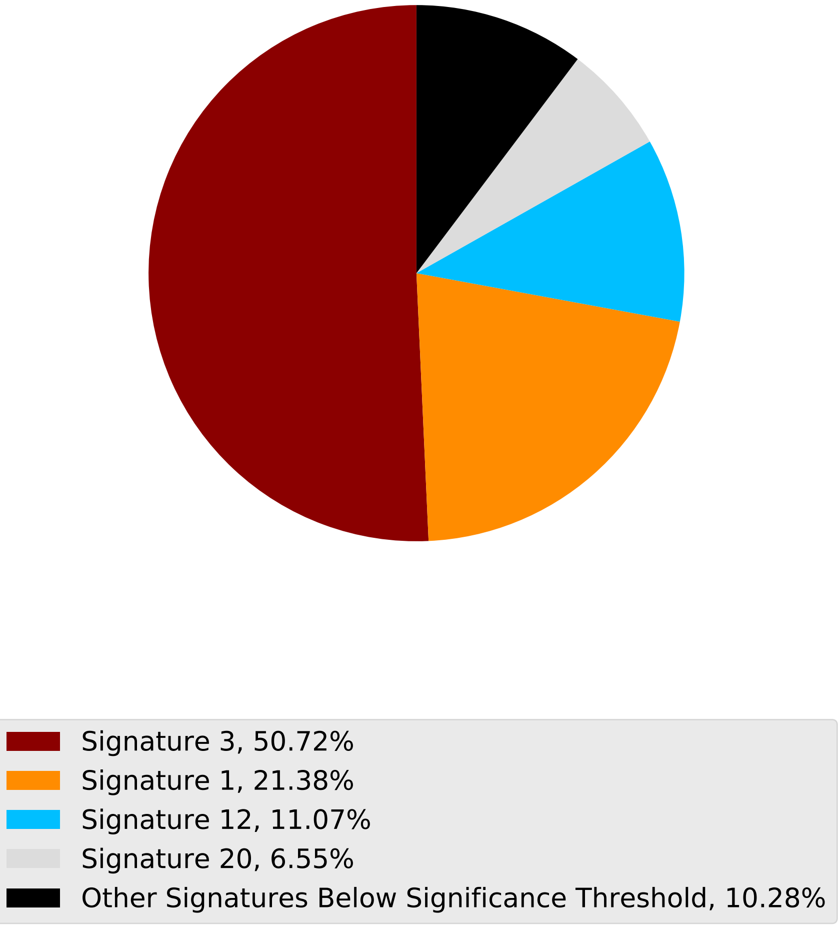

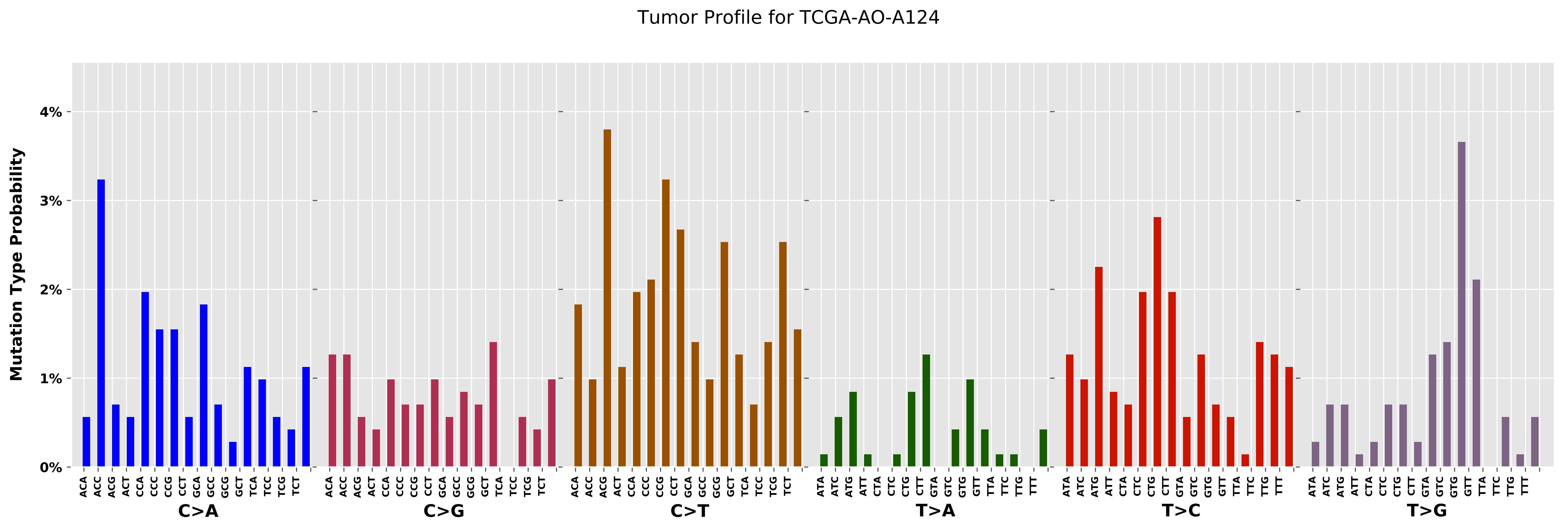

Tumor Profile for TCGA-AO-A124
Mutation Type Probability
0%
1%
2%
3%
4%
C>A
C>G
C>T
T>A
T>C
T>G

Cancer processes Weights for TCGA-A2-A259

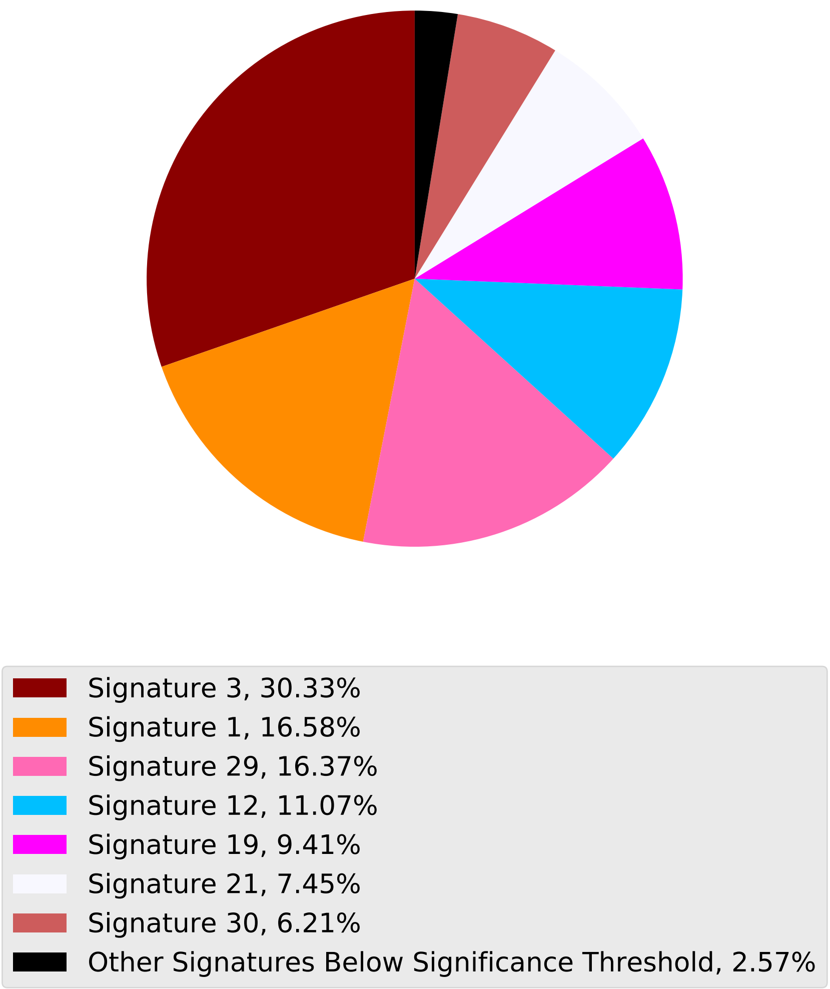

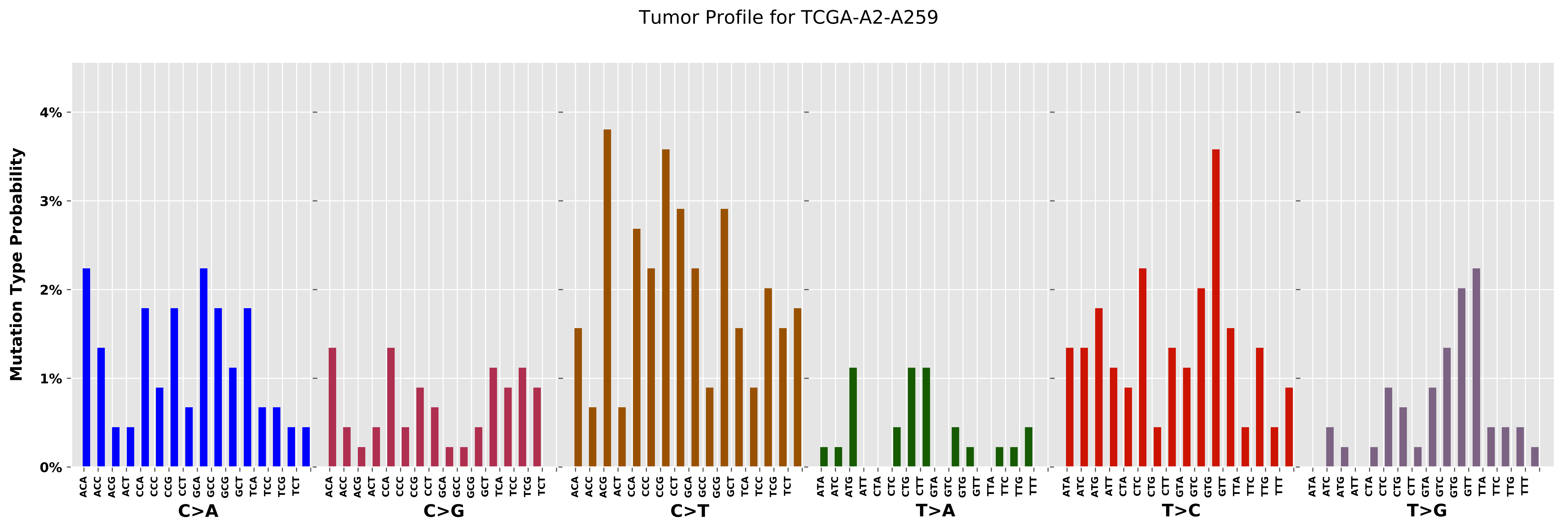
Tumor Profile for TCGA-A2-A259
Mutation Type Probability
0%
1%
2%
3%
4%
C>A
C>G
C>T
T>A
T>C
T>G
ACA ACC ACG ACT CCA CCC CCG CCT GCA GCC GCG GCT TCA TCC TCG TCT
ATA ATC ATG ATT CTA CTC CTG CTT GTA GTC GTG GTT TTA TTC TTG TTT

Cancer processes Weights for TCGA-BH-A0DG

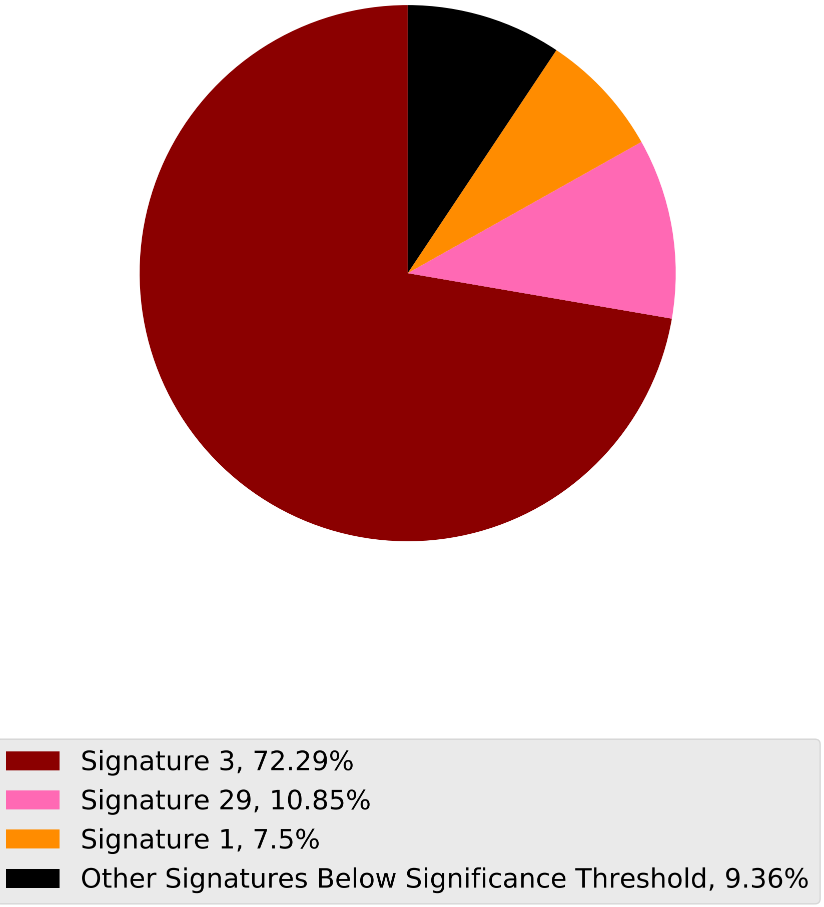

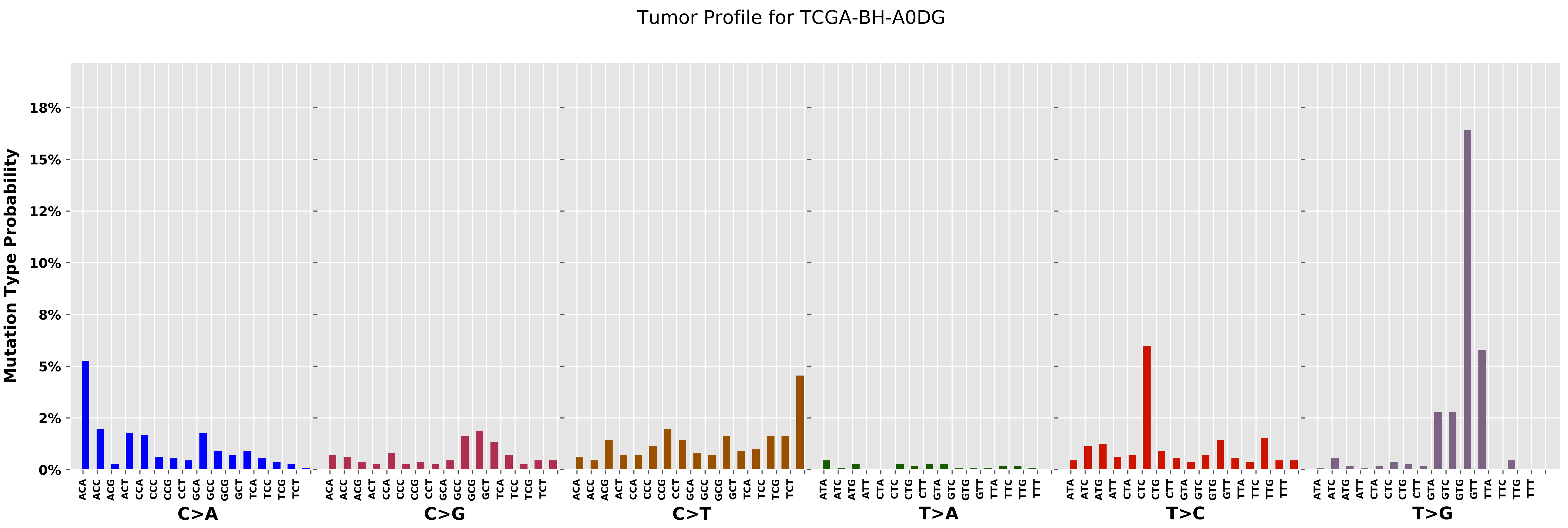

Tumor Profile for TCGA-BH-A0DG
Mutation Type Probability
18%
15%
12%
10%
8%
5%
2%
0%
ACA ACC ACG ACT CCA CCC CCG CCT GCA GCC GCG GCT TCA TCC TCG TCT
C>A
C>G
C>T
ATA ATC ATG ATT CTA CTC CTG CTT GTA GTC GTG GTT TTA TTC TTG TTT
T>A
T>C
T>G

Cancer processes Weights for TCGA-A2-A3KC

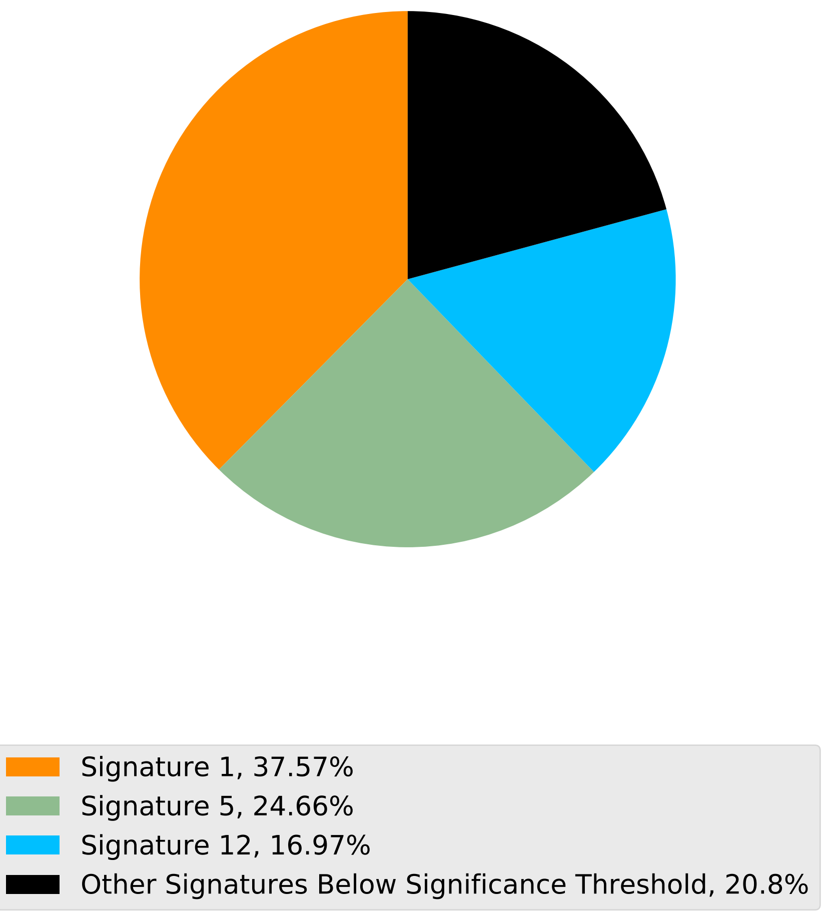

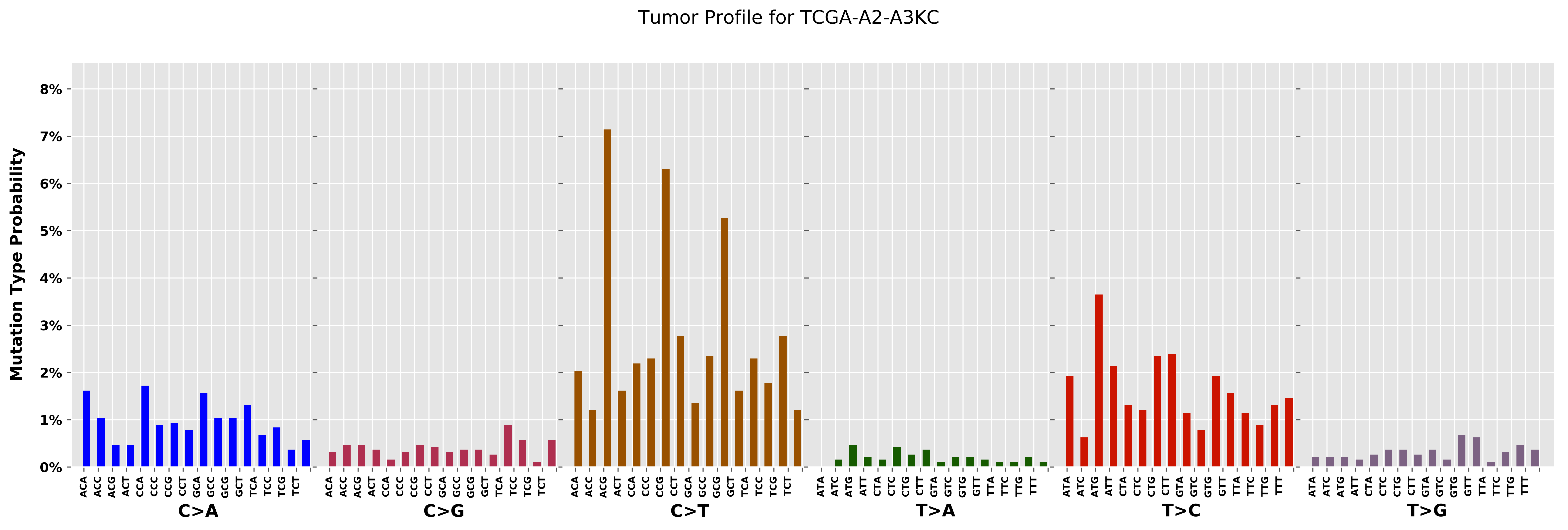

Tumor Profile for TCGA-A2-A3KC
Mutation Type Probability
0%
1%
2%
3%
4%
5%
6%
7%
8%
C>A
C>G
C>T
T>A
T>C
T>G
ACA ACC ACG ACT CCA CCC CCG CCT GCA GCC GCG GCT TCA TCC TCG TCT
ATA ATC ATG ATT CTA CTC CTG CTT GTA GTC GTG GTT TTA TTC TTG TTT

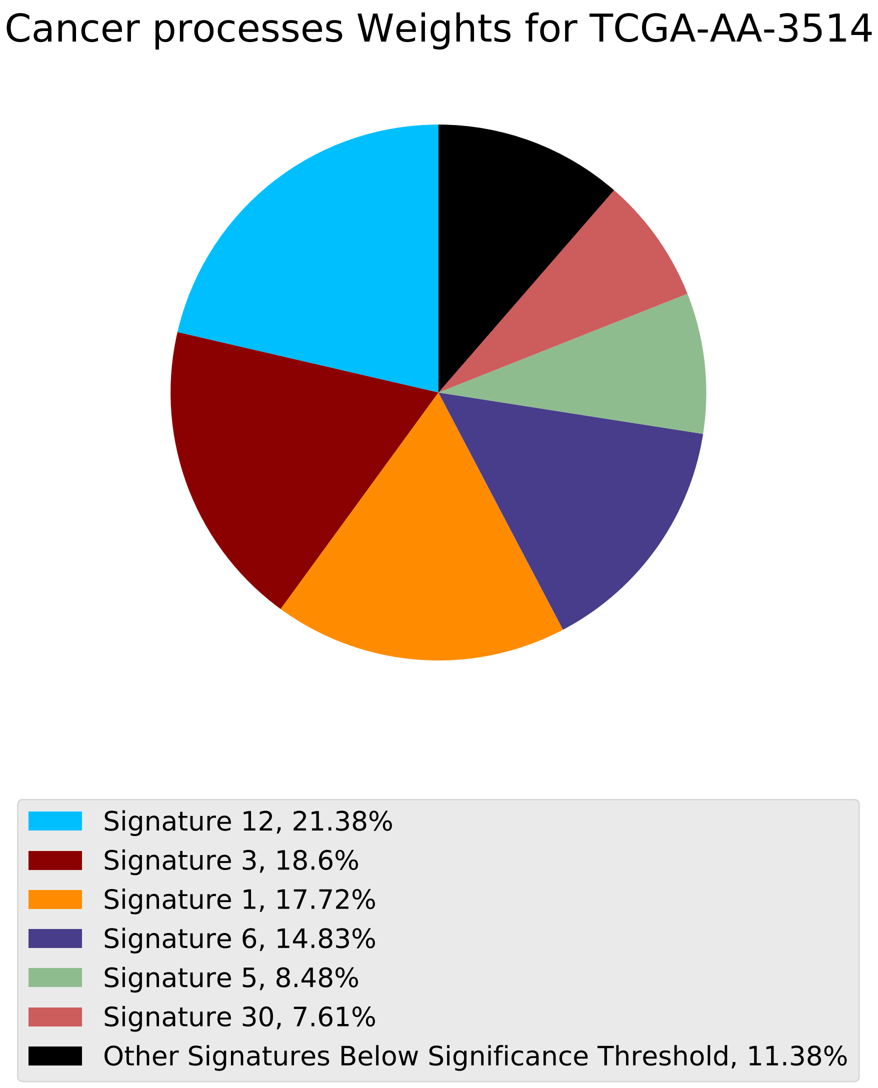
Cancer processes Weights for TCGA-AA-3514
Signature 12, 21.38%
Signature 3, 18.6%
Signature 1, 17.72%
Signature 6, 14.83%
Signature 5, 8.48%
Signature 30, 7.61%
Other Signatures Below Significance Threshold, 11.38%

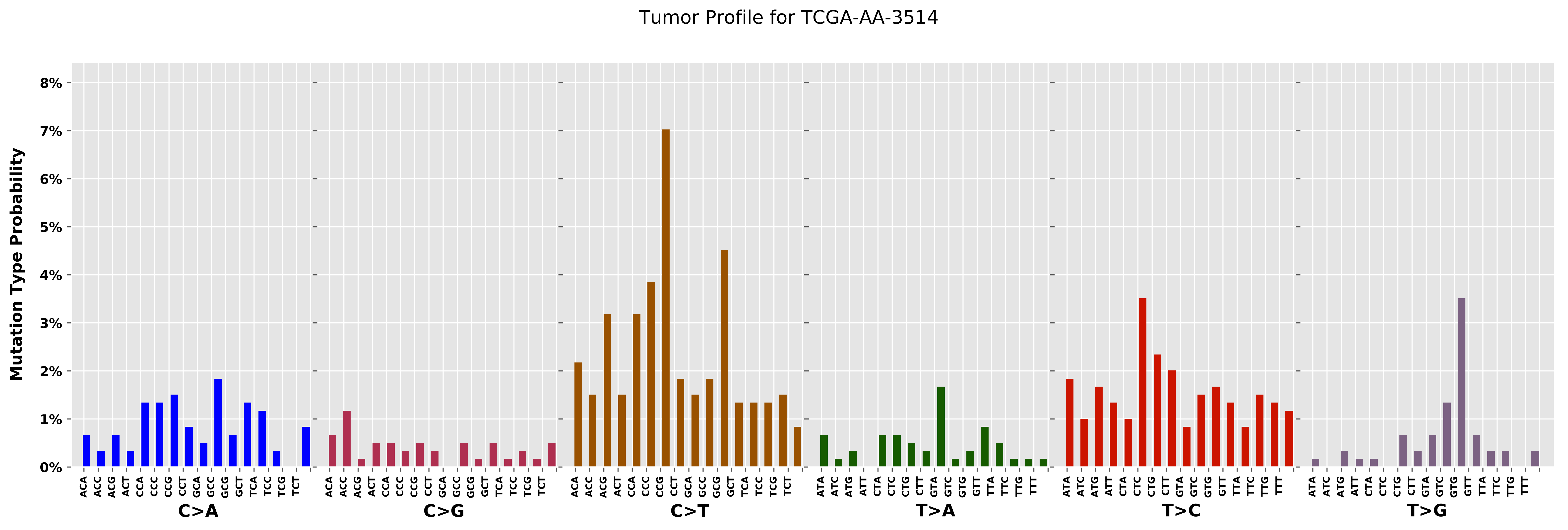
Tumor Profile for TCGA-AA-3514
Mutation Type Probability
0%
1%
2%
3%
4%
5%
6%
7%
8%
C>A
C>G
C>T
T>A
T>C
T>G
ACA ACC ACG ACT CCA CCC CCG CCT GCA GCC GCG GCT TCA TCC TCG TCT
ATA ATC ATG ATT CTA CTC CTG CTT GTA GTC GTG GTT TTA TTC TTG TTT

Cancer processes Weights for TCGA-AN-A0XR

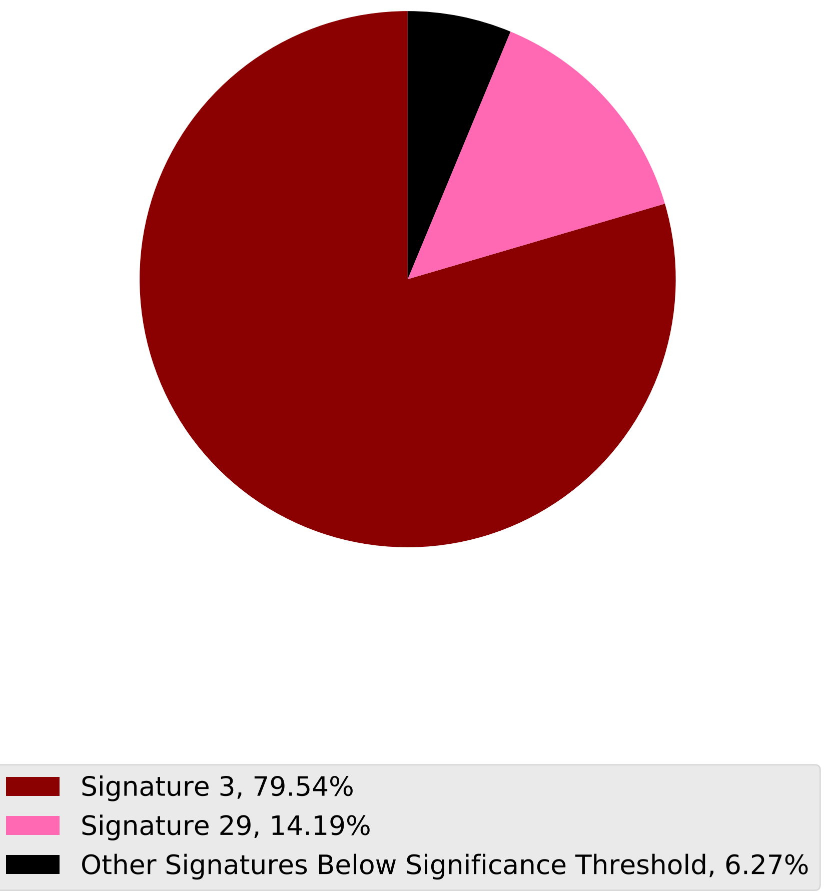

Tumor Profile for TCGA-AN-A0XR

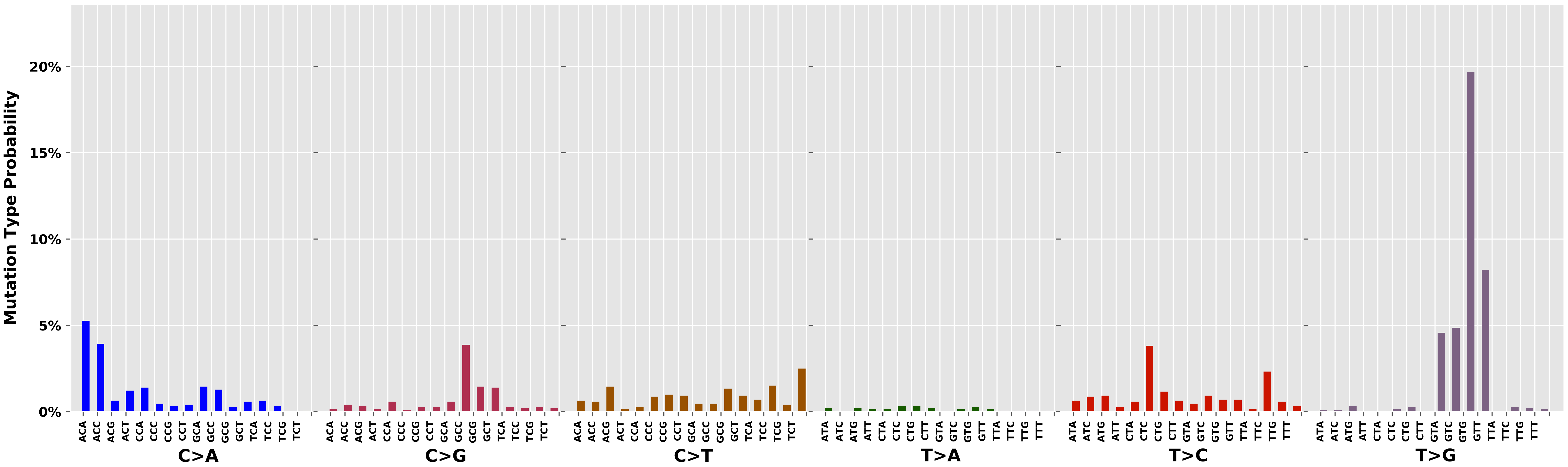

Cancer processes Weights for TCGA-BH-A0EA

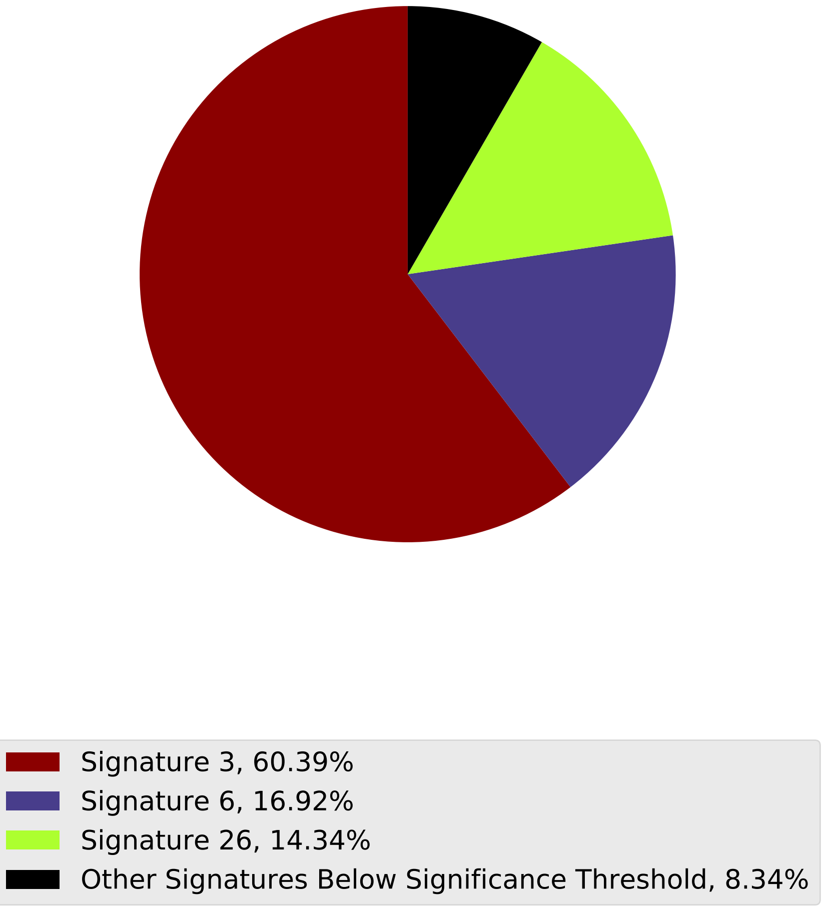

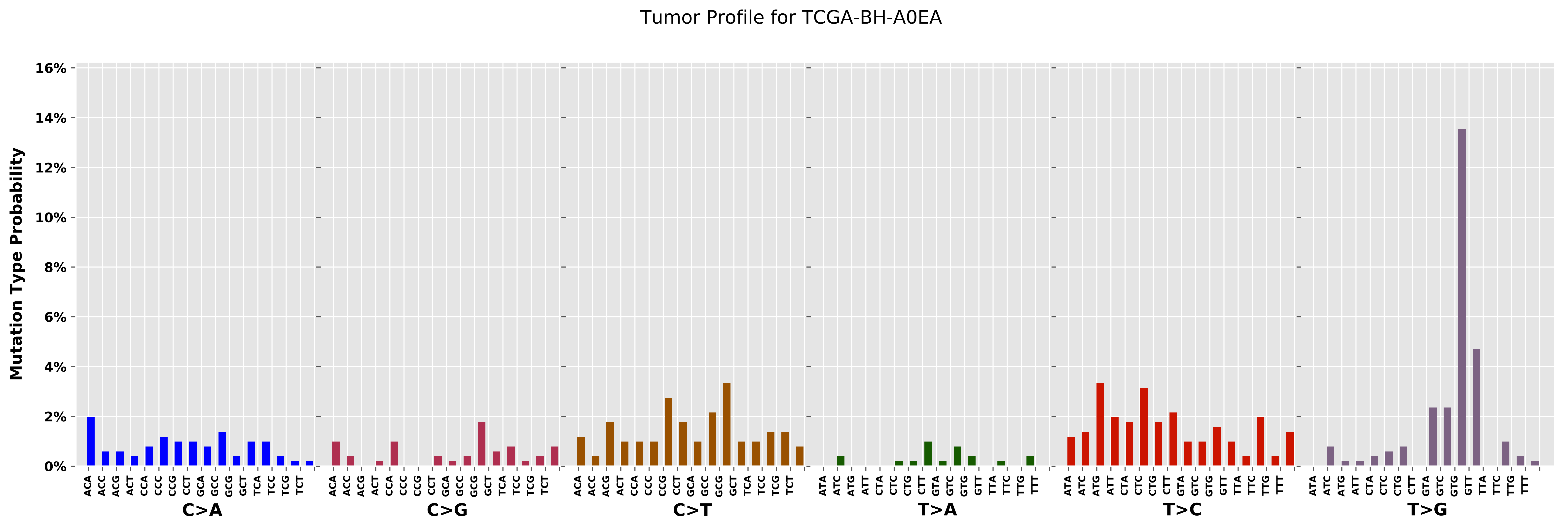

Tumor Profile for TCGA-BH-A0EA
Mutation Type Probability
16%
14%
12%
10%
8%
6%
4%
2%
0%
C>A
C>G
C>T
T>A
T>C
T>G
ACA ACC ACG ACT CCA CCC CCG CCT GCA GCC GCG GCT TCA TCC TCG TCT
ATA ATC ATG ATT CTA CTC CTG CTT GTA GTC GTG GTT TTA TTC TTG TTT

Cancer processes Weights for TCGA-AR-A256

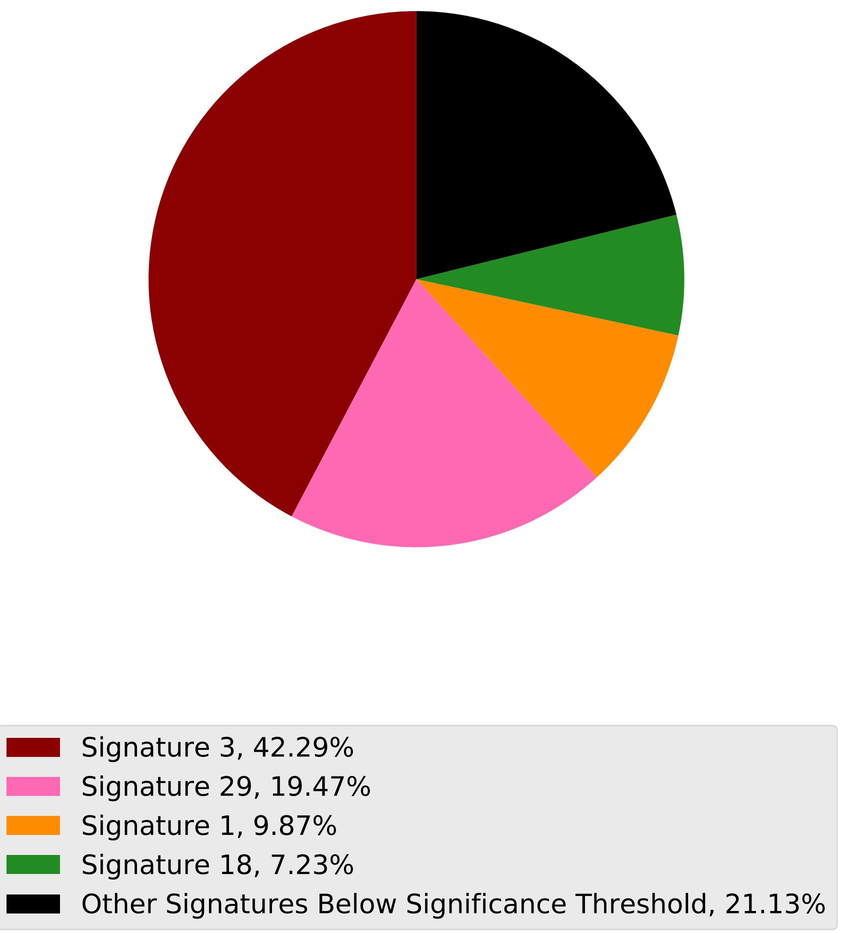

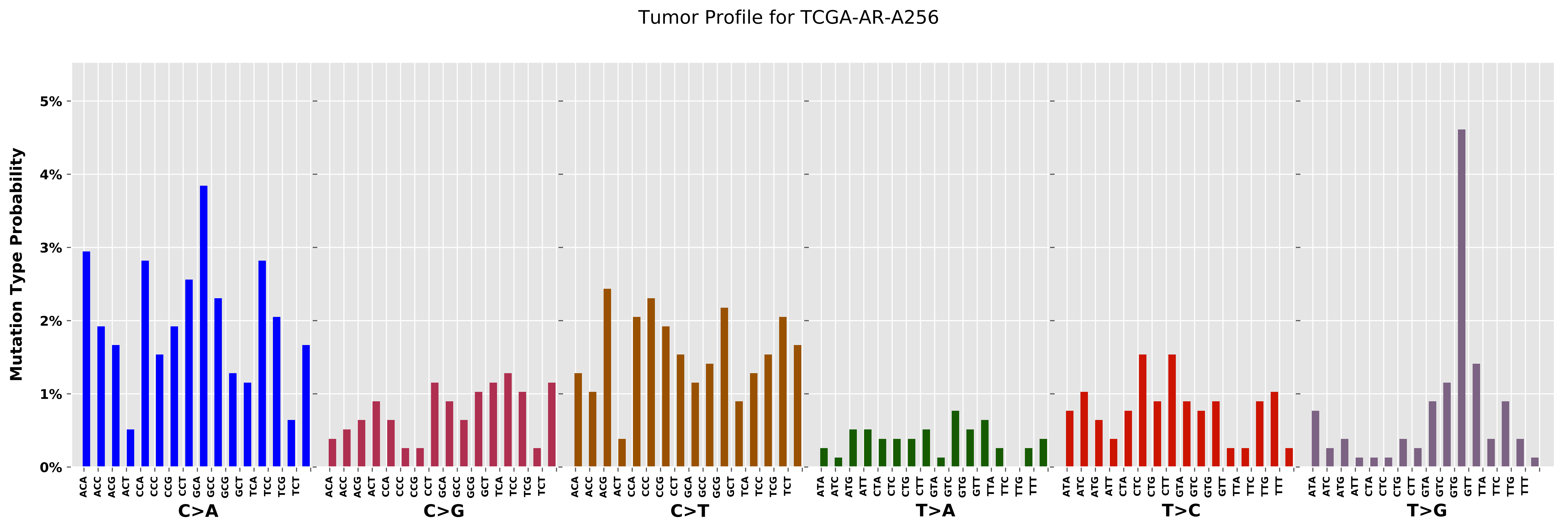

Tumor Profile for TCGA-AR-A256
Mutation Type Probability
0%
1%
2%
3%
4%
5%
C>A
C>G
C>T
T>A
T>C
T>G
ACA ACC ACG ACT CCA CCC CCG CCT GCA GCC GCG GCT TCA TCC TCG TCT
ATA ATC ATG ATT CTA CTC CTG CTT GTA GTC GTG GTT TTA TTC TTG TTT

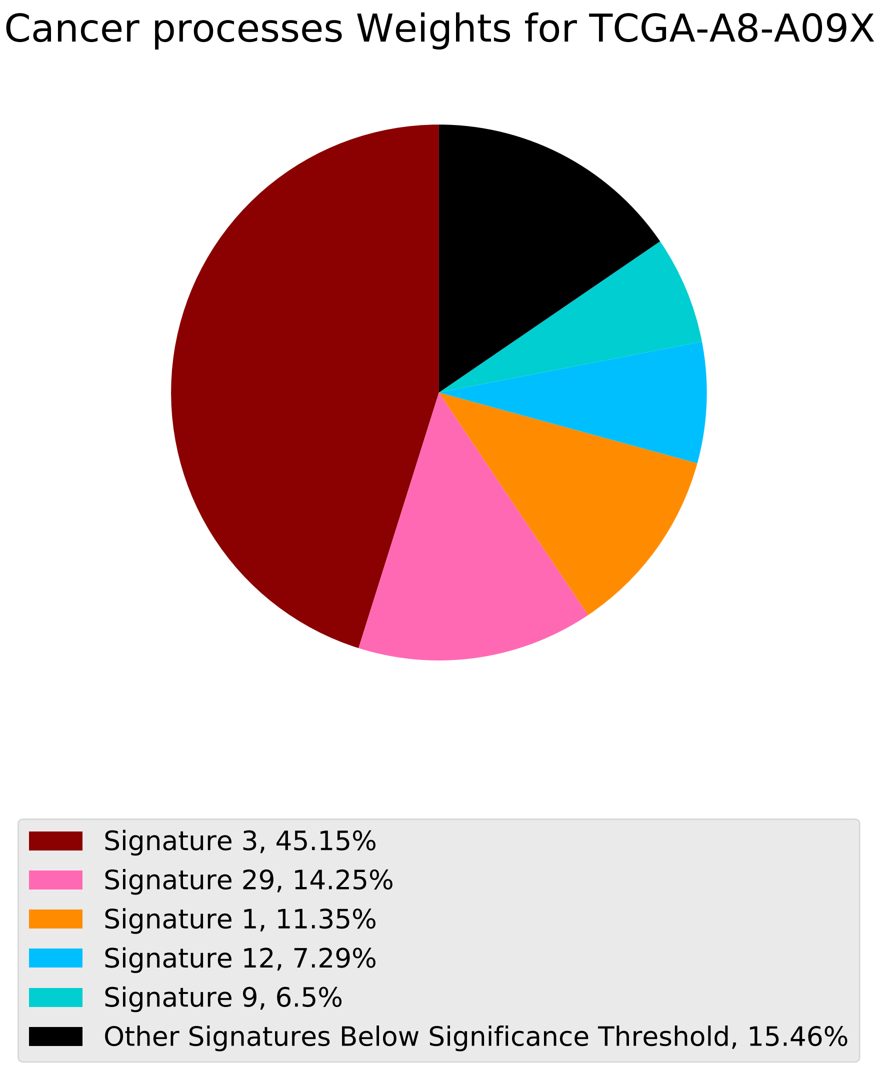
Cancer processes Weights for TCGA-A8-A09X
Signature 3, 45.15%
Signature 29, 14.25%
Signature 1, 11.35%
Signature 12, 7.29%
Signature 9, 6.5%
Other Signatures Below Significance Threshold, 15.46%

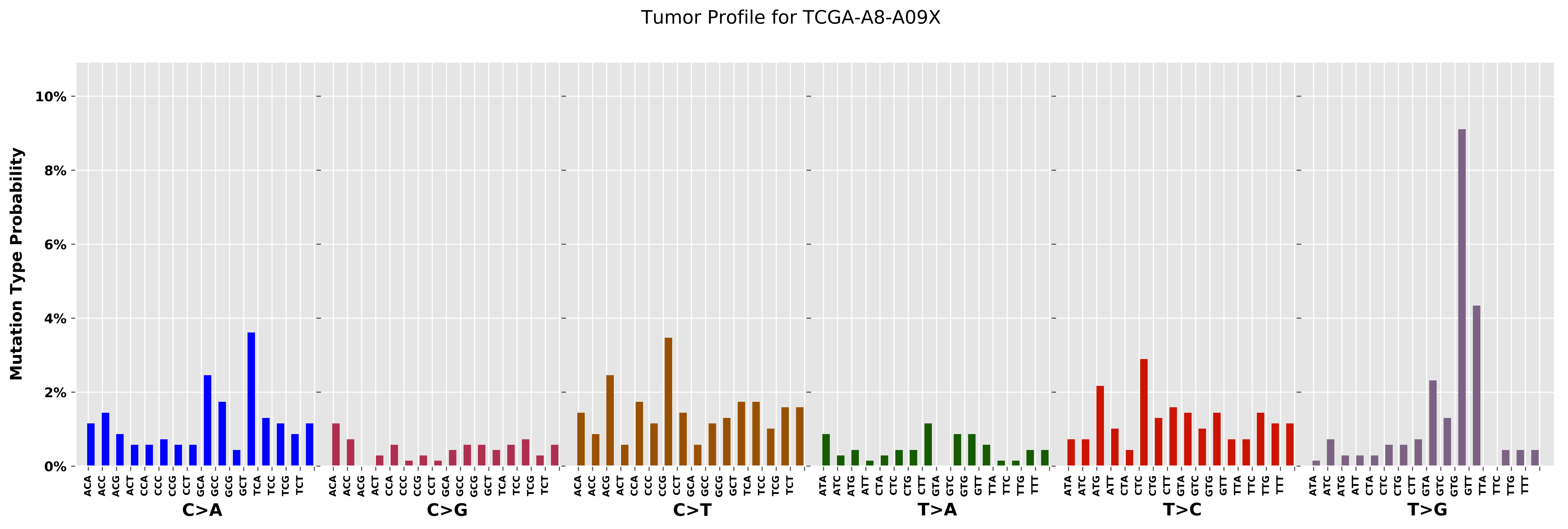

Tumor Profile for TCGA-A8-A09X
Mutation Type Probability
0%
2%
4%
6%
8%
10%
C>A
C>G
C>T
T>A
T>C
T>G
ACA ACC ACG ACT CCA CCC CCG CCT GCA GCC GCG GCT TCA TCC TCG TCT
ATA ATC ATG ATT CTA CTC CTG CTT GTA GTC GTG GTT TTA TTC TTG TTT

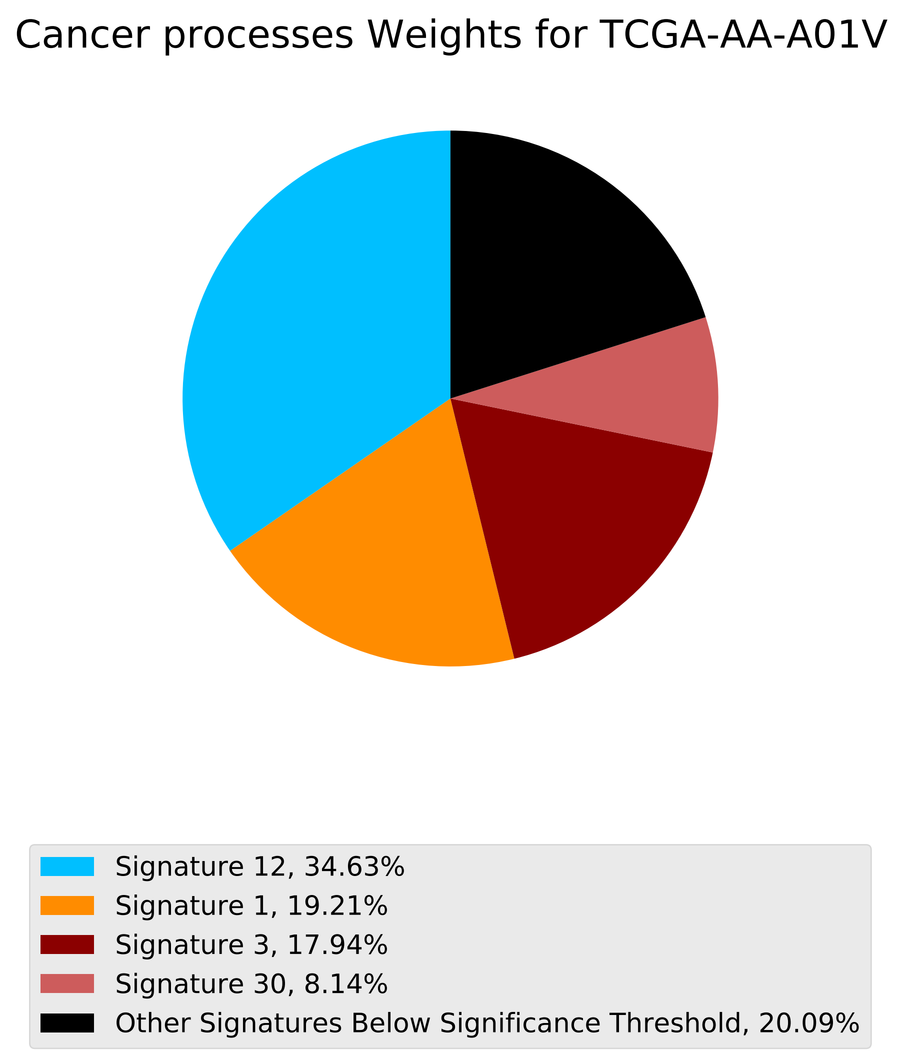
Cancer processes Weights for TCGA-AA-A01V
Signature 12, 34.63%
Signature 1, 19.21%
Signature 3, 17.94%
Signature 30, 8.14%
Other Signatures Below Significance Threshold, 20.09%

Tumor Profile for TCGA-AA-A01V

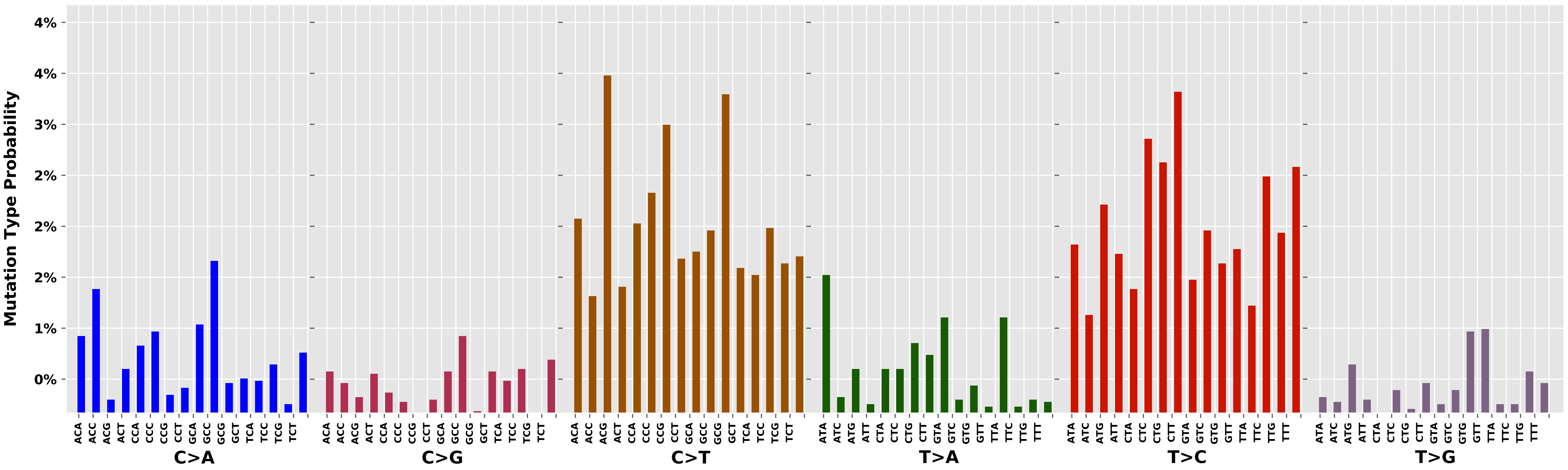

Cancer processes Weights for TCGA-A2-A04T

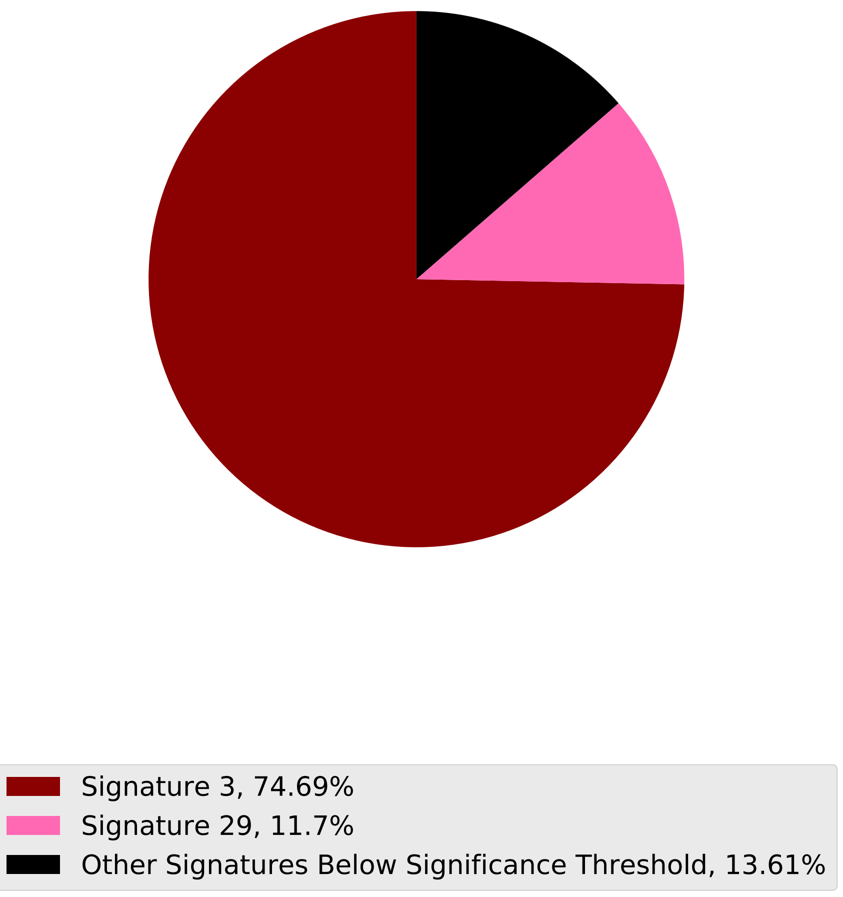

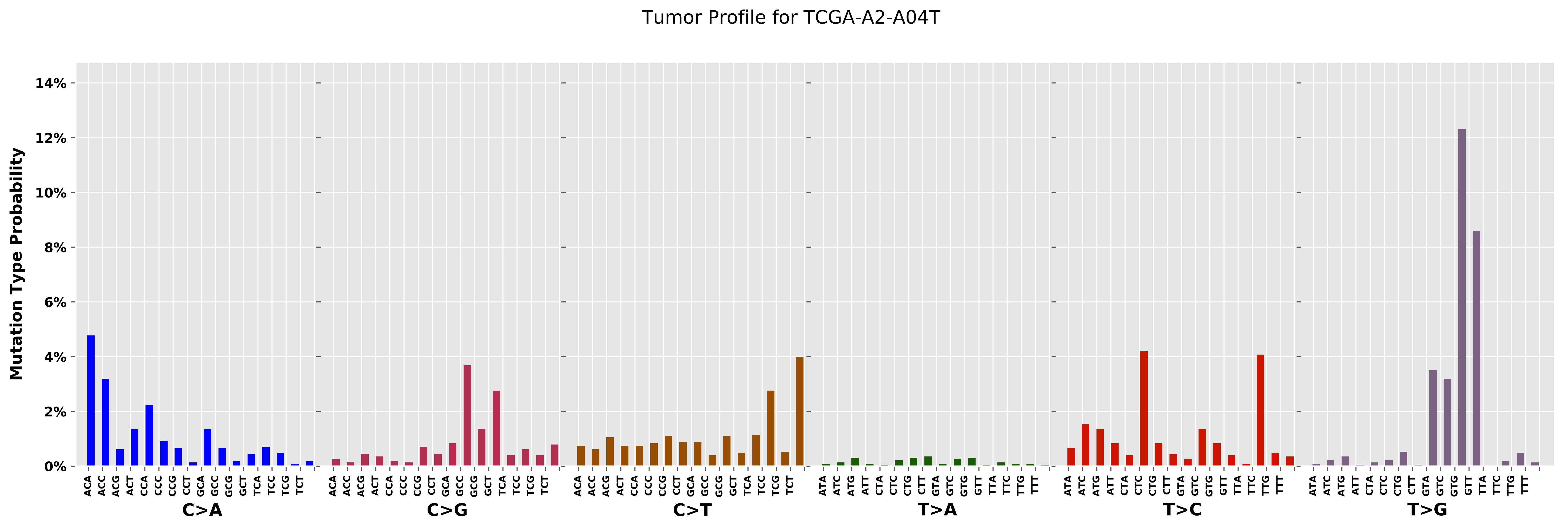

Tumor Profile for TCGA-A2-A04T
Mutation Type Probability
0%
2%
4%
6%
8%
10%
12%
14%
C>A
C>G
C>T
T>A
T>C
T>G
ACA ACC ACG ACT CCA CCC CCG CCT GCA GCC GCG GCT TCA TCC TCG TCT
ATA ATC ATG ATT CTA CTC CTG CTT GTA GTC GTG GTT TTA TTC TTG TTT

Cancer processes Weights for TCGA-BH-A0H6

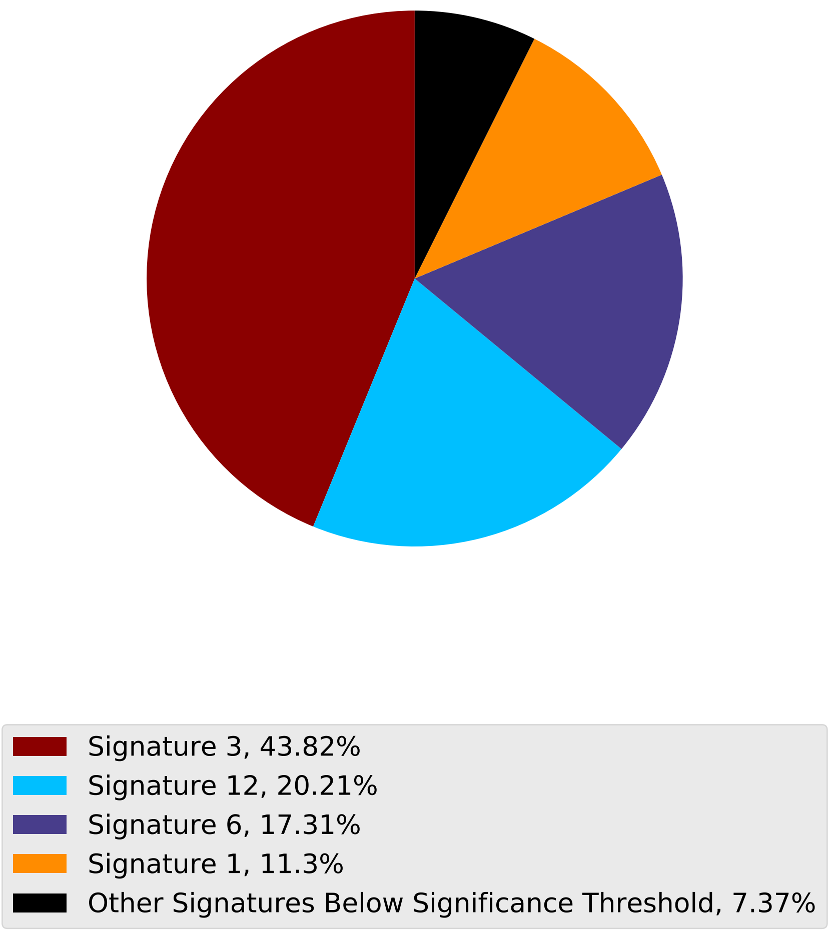

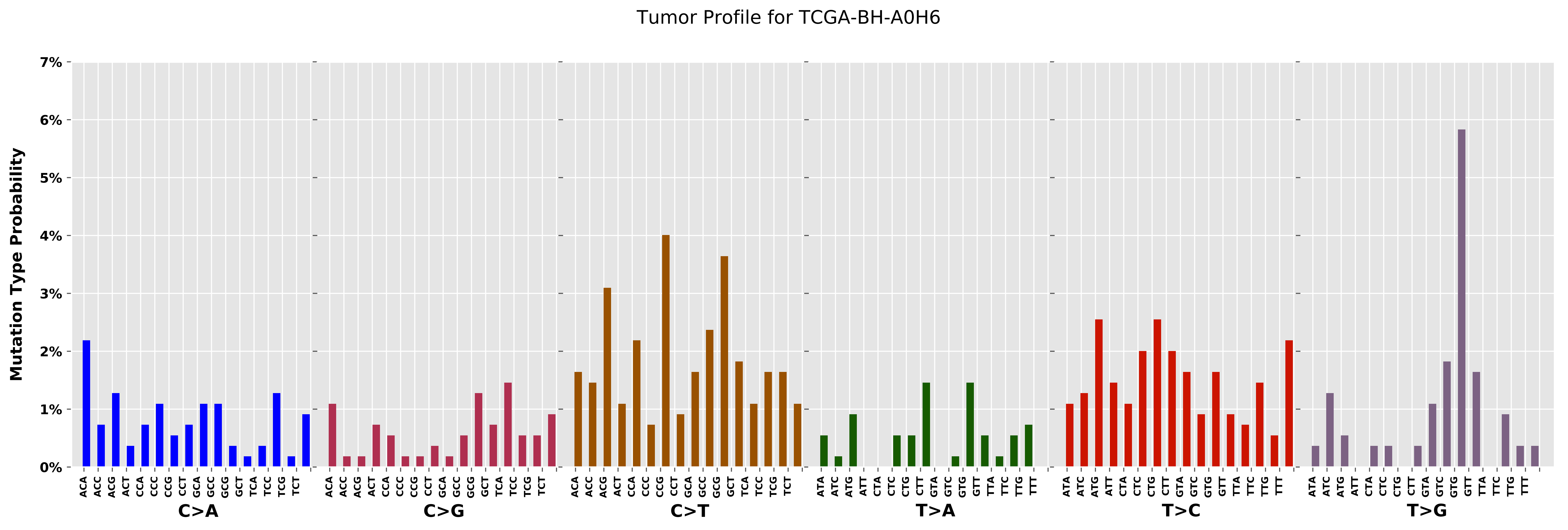
Tumor Profile for TCGA-BH-A0H6
Mutation Type Probability
0%
1%
2%
3%
4%
5%
6%
7%
C>A
C>G
C>T
T>A
T>C
T>G

Cancer processes Weights for TCGA-D8-A27F

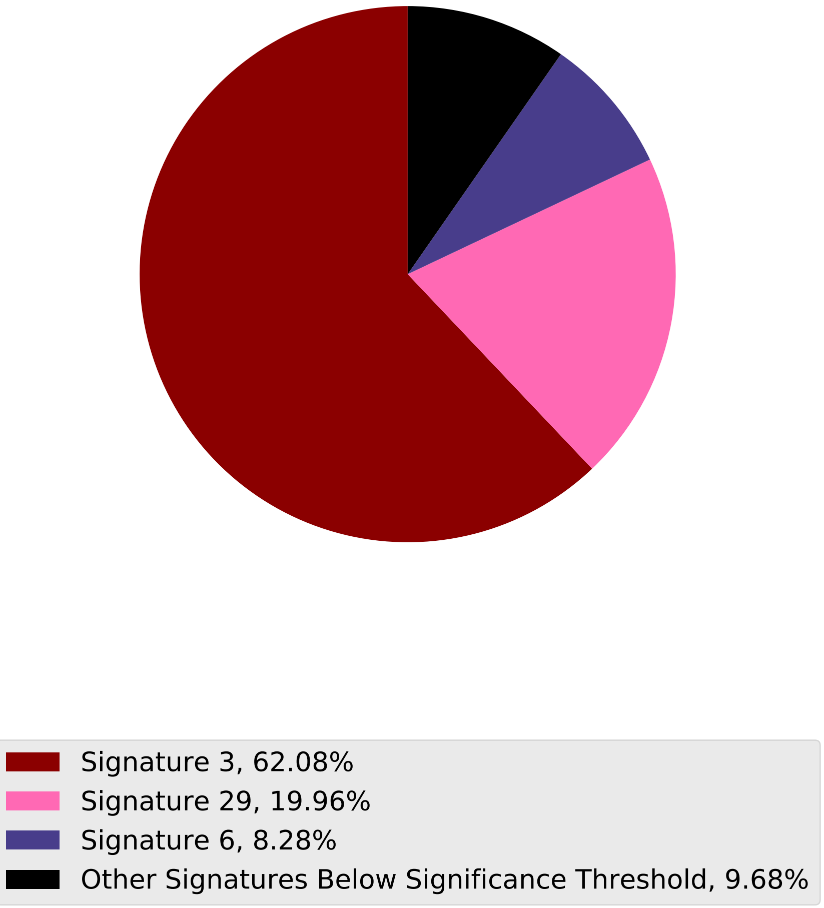

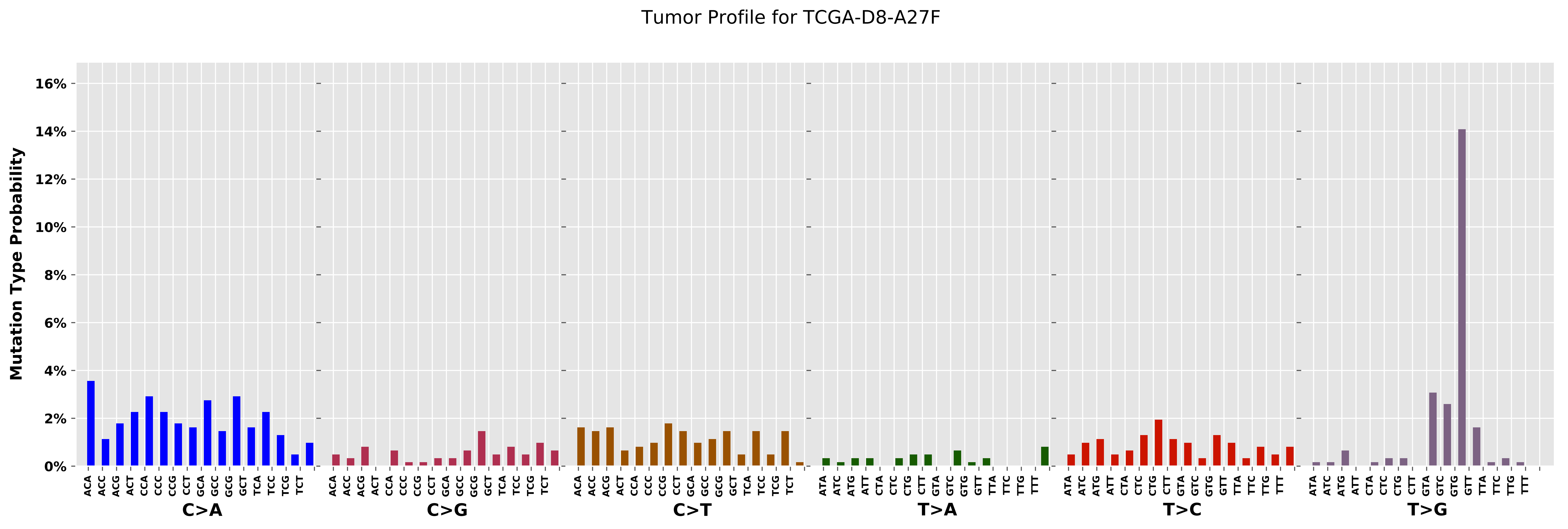

Tumor Profile for TCGA-D8-A27F
Mutation Type Probability
0%
2%
4%
6%
8%
10%
12%
14%
16%
C>A
C>G
C>T
T>A
T>C
T>G

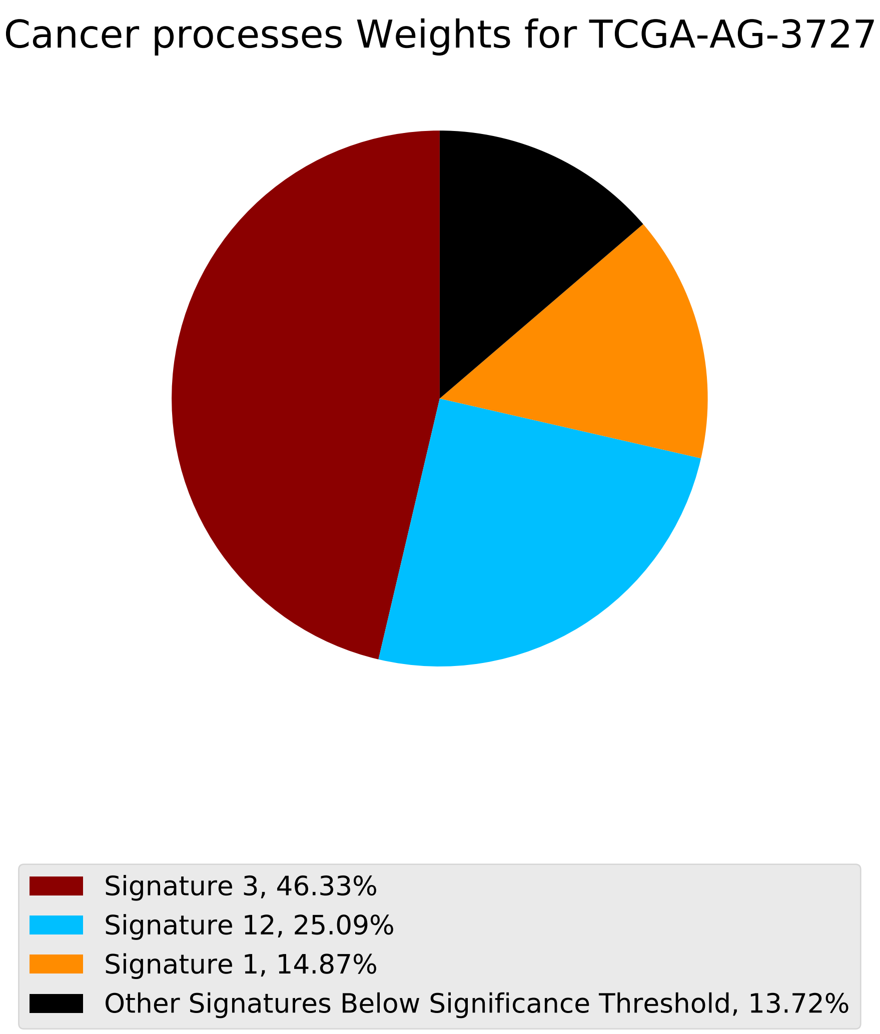
Cancer processes Weights for TCGA-AG-3727
Signature 3, 46.33%
Signature 12, 25.09%
Signature 1, 14.87%
Other Signatures Below Significance Threshold, 13.72%

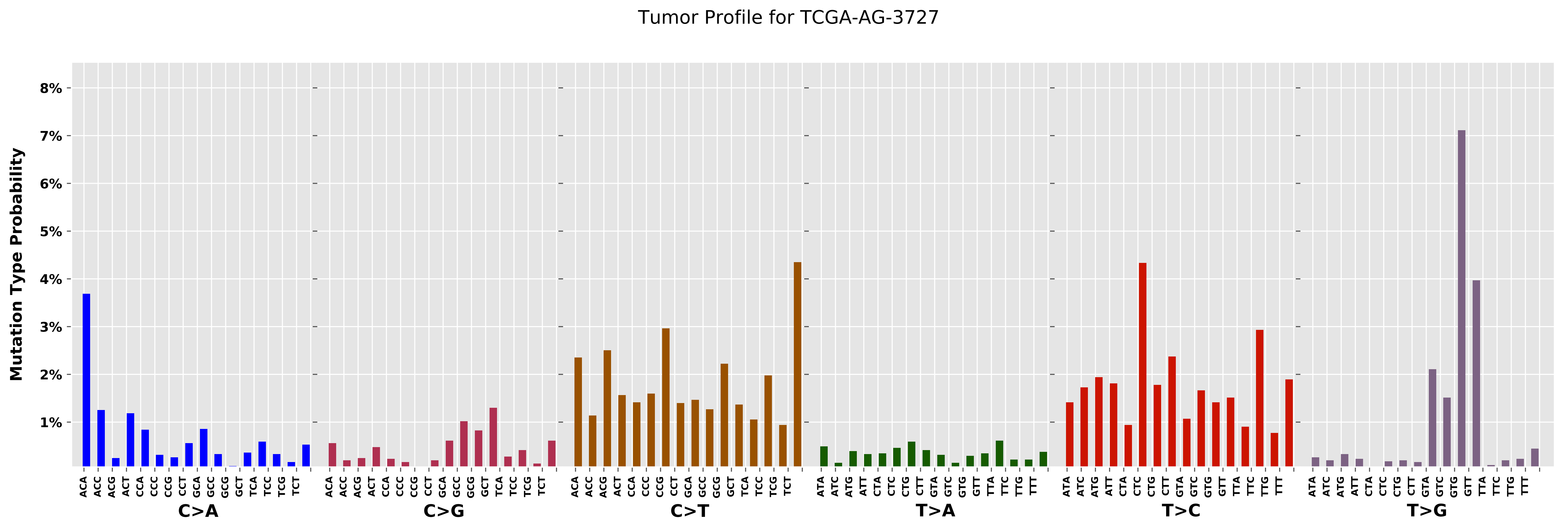

Tumor Profile for TCGA-AG-3727
Mutation Type Probability
8%
7%
6%
5%
4%
3%
2%
1%
ACA ACC ACG ACT CCA CCC CCG CCT GCA GCC GCG GCT TCA TCC TCG TCT
C>A
C>G
C>T
ATA ATC ATG ATT CTA CTC CTG CTT GTA GTC GTG GTT TTA TTC TTG TTT
T>A
T>C
T>G

Cancer processes Weights for TCGA-A8-A09I

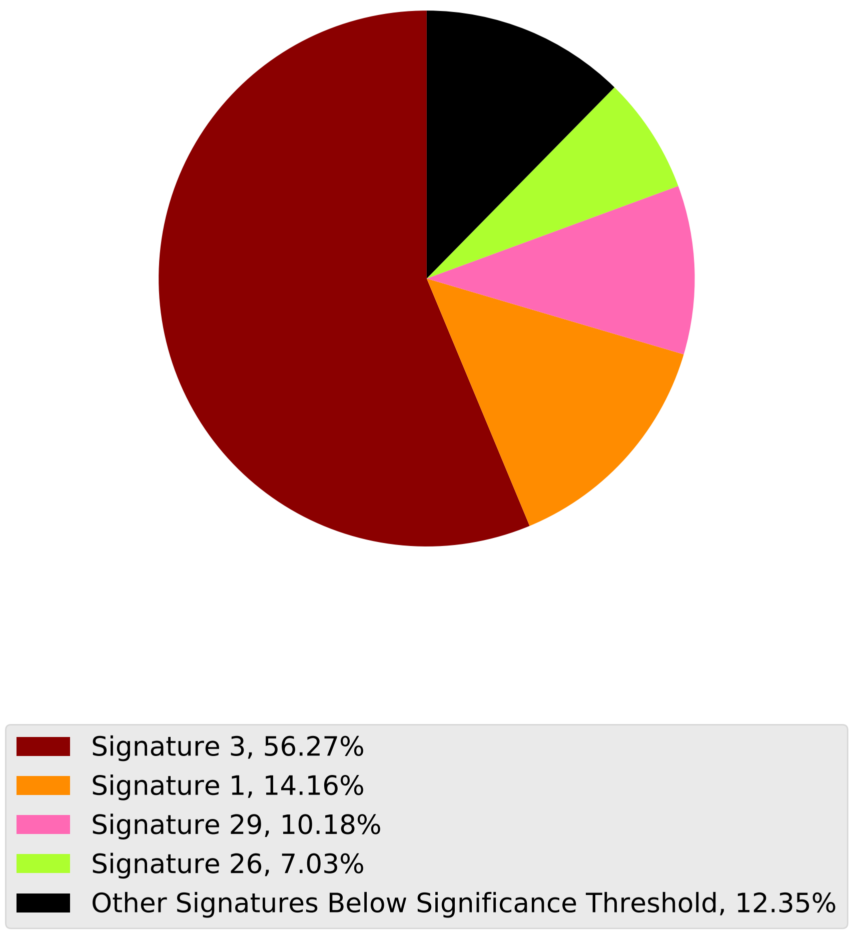

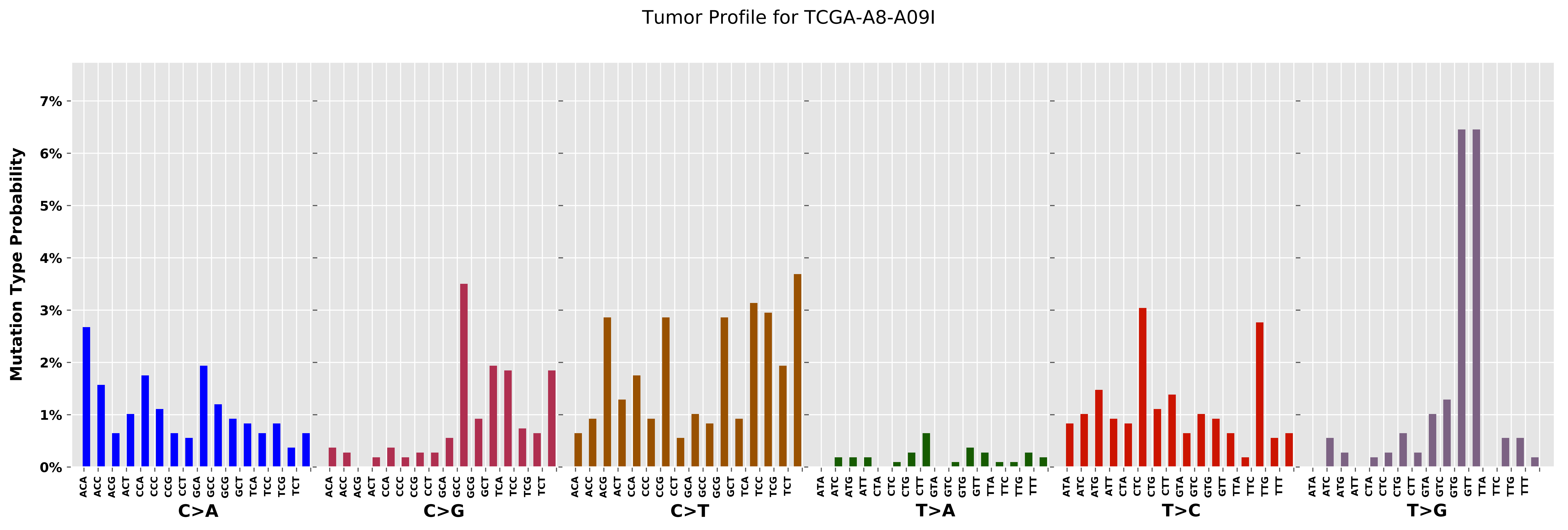
Tumor Profile for TCGA-A8-A09I
Mutation Type Probability
0%
1%
2%
3%
4%
5%
6%
7%
ACA ACC ACG ACT CCA CCC CCG CCT GCA GCC GCG GCT TCA TCC TCG TCT
C>A
C>G
C>T
ATA ATC ATG ATT CTA CTC CTG CTT GTA GTC GTG GTT TTA TTC TTG TTT
T>A
T>C
T>G

Cancer processes Weights for TCGA-AA-3664

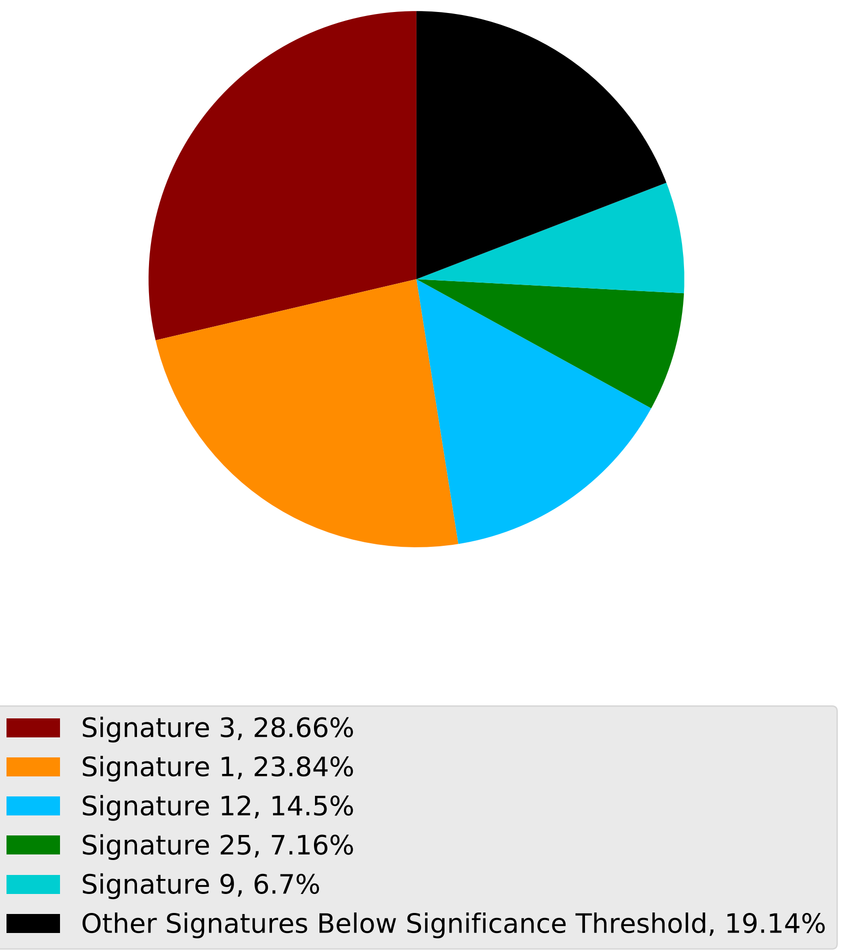

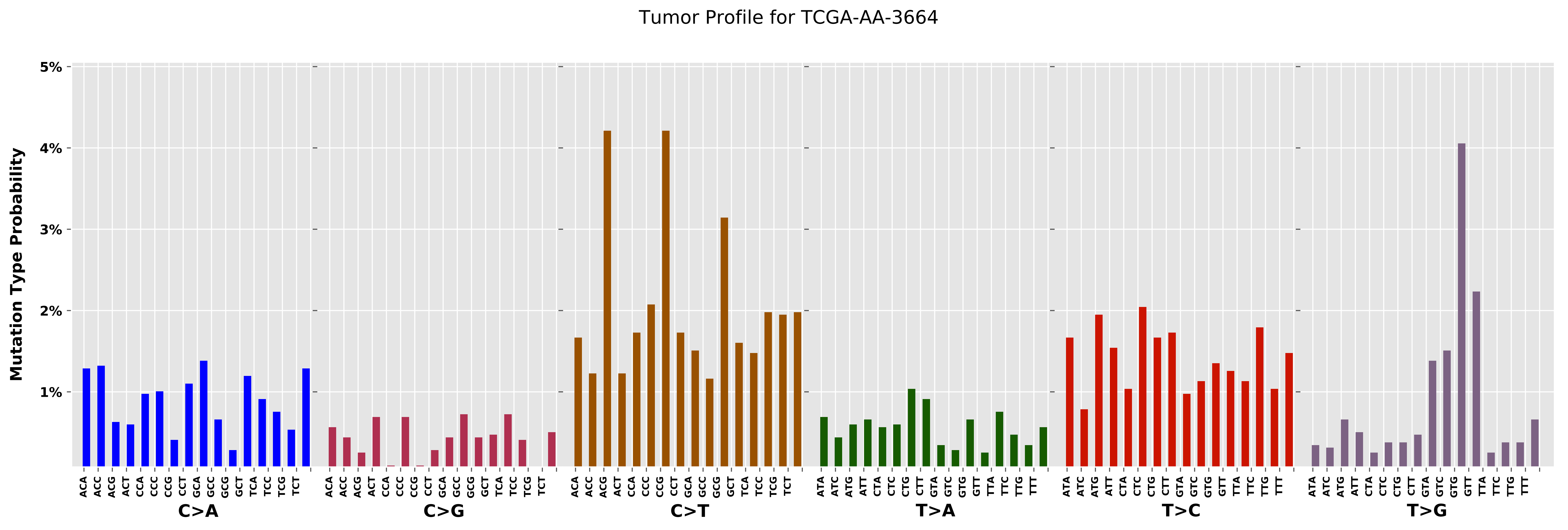

Tumor Profile for TCGA-AA-3664
Mutation Type Probability
5%
4%
3%
2%
1%
C>A
C>G
C>T
T>A
T>C
T>G

Cancer processes Weights for TCGA-E2-A1LL

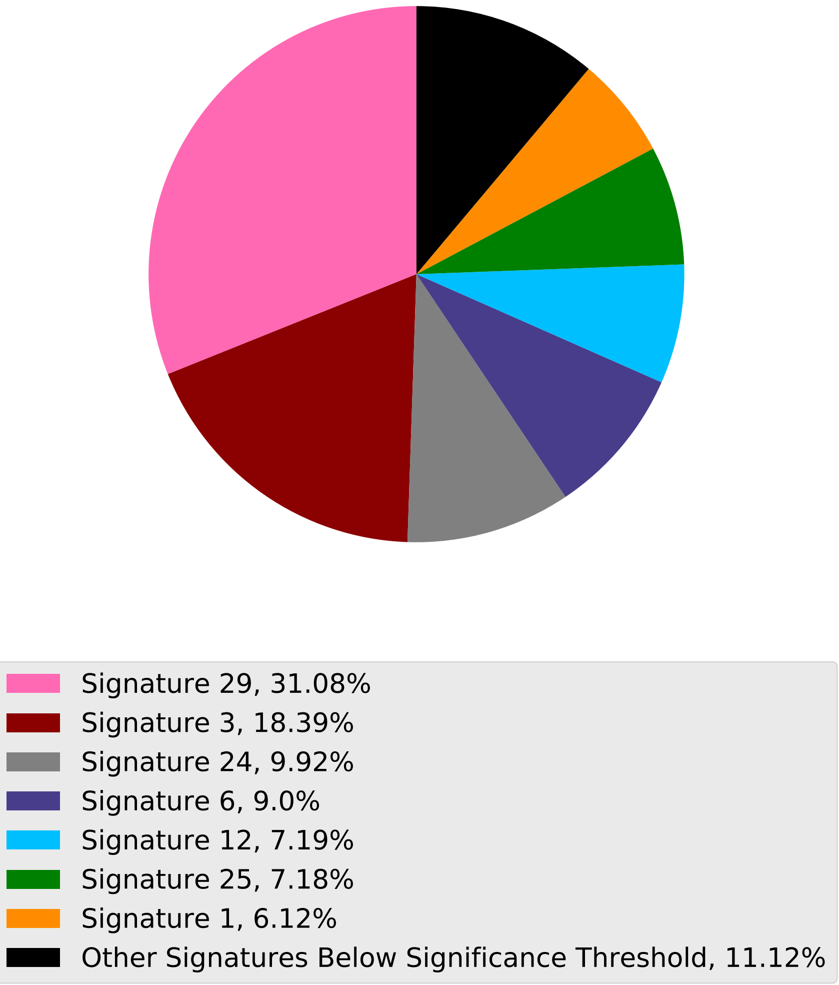

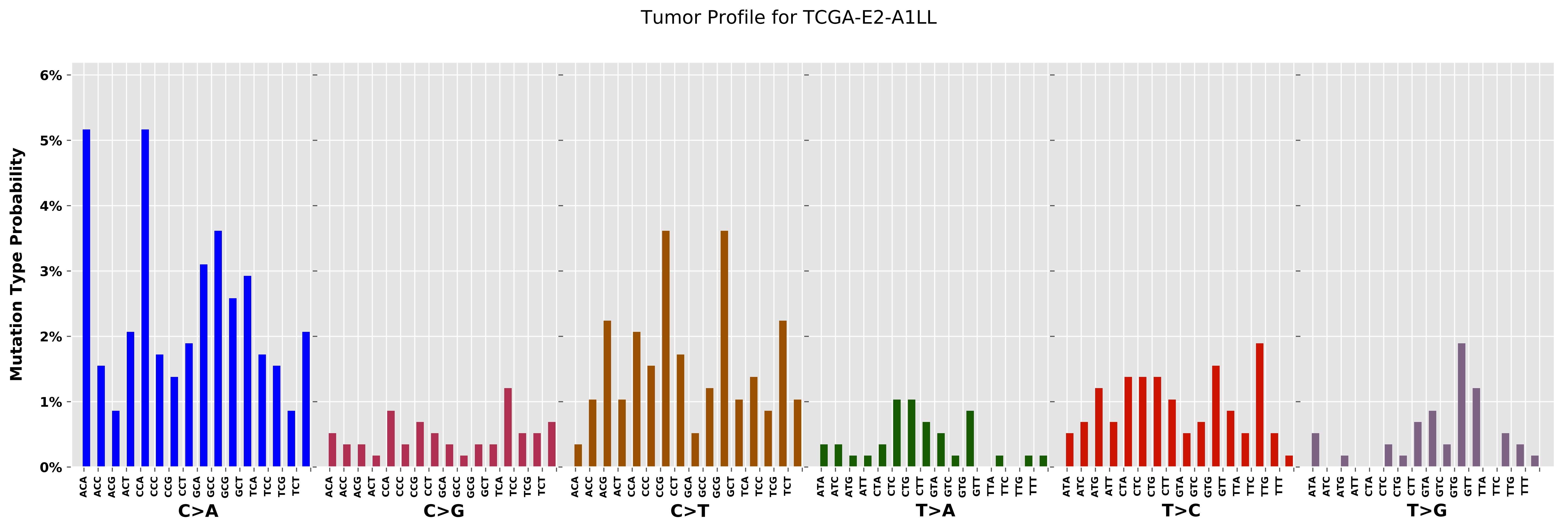
Tumor Profile for TCGA-E2-A1LL
Mutation Type Probability
0%
1%
2%
3%
4%
5%
6%
C>A
C>G
C>T
T>A
T>C
T>G
ACA ACC ACG ACT CCA CCC CCG CCT GCA GCC GCG GCT TCA TCC TCG TCT
ATA ATC ATG ATT CTA CTC CTG CTT GTA GTC GTG GTT TTA TTC TTG TTT

Cancer processes Weights for TCGA-A1-A0SM

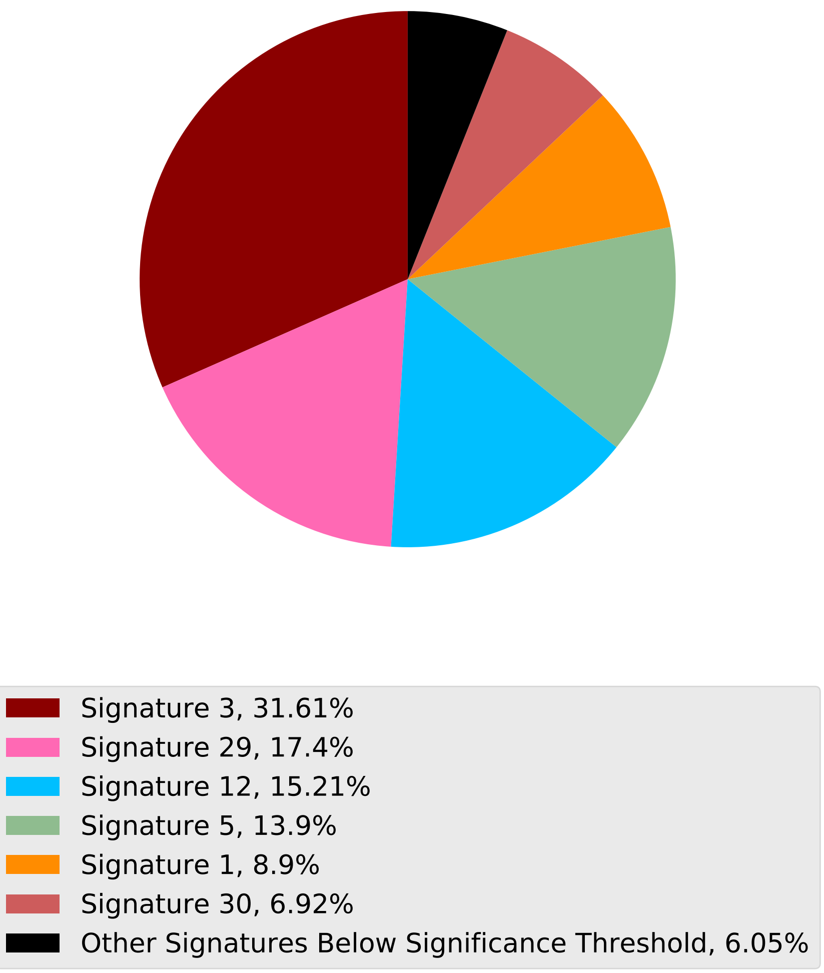

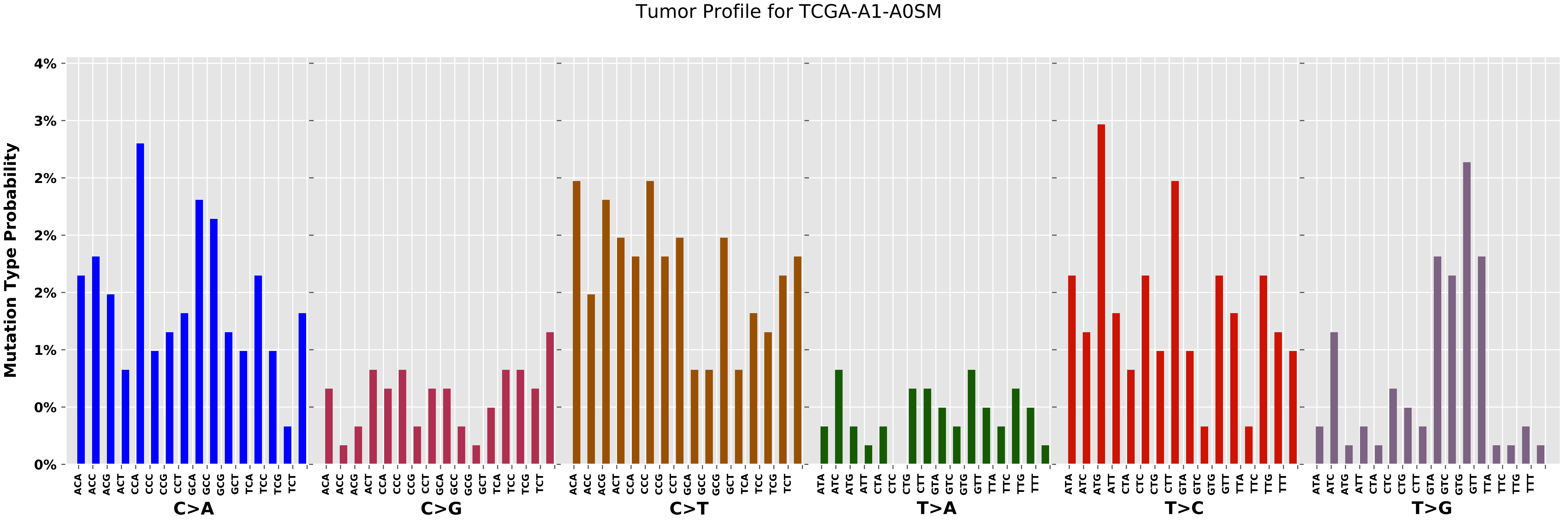

Tumor Profile for TCGA-A1-A0SM
Mutation Type Probability
4%
3%
2%
2%
2%
1%
0%
0%
0%
C>A
C>G
C>T
T>A
T>C
T>G
ACA ACC ACG ACT CCA CCC CCG CCT GCA GCC GCG GCT TCA TCC TCG TCT
ATA ATC ATG ATT CTA CTC CTG CTT GTA GTC GTG GTT TTA TTC TTG TTT

Cancer processes Weights for TCGA-C8-A130

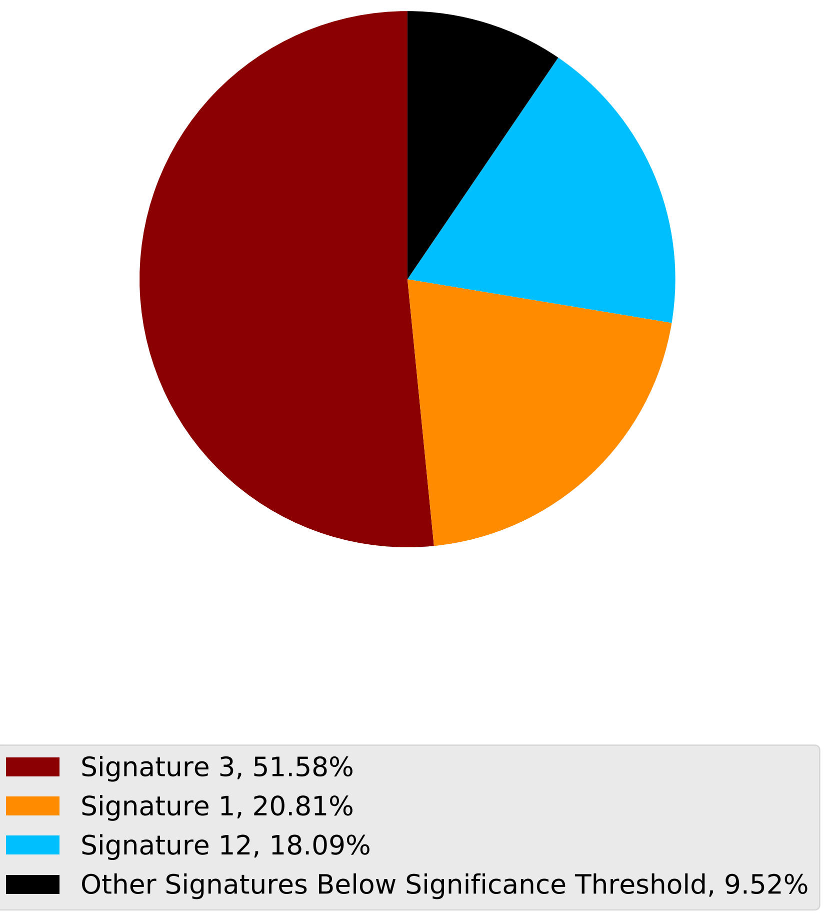

Tumor Profile for TCGA-C8-A130

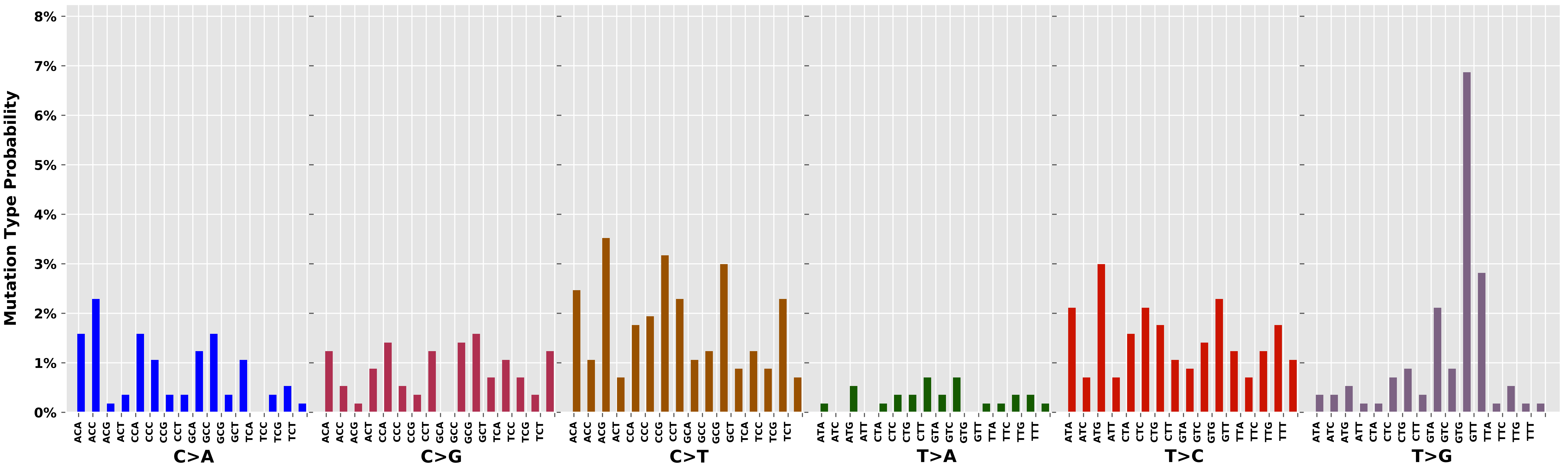

Cancer processes Weights for TCGA-BH-A0H0

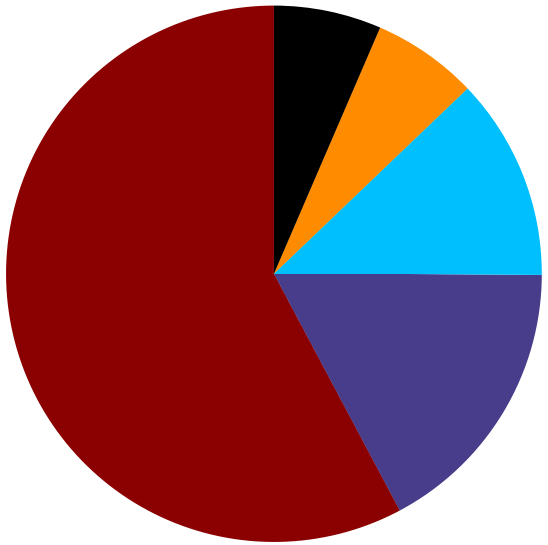

- Signature 3, 57.78%
- Signature 6, 17.16%
- Signature 12, 12.2%
- Signature 1, 6.37%
- Other Signatures Below Significance Threshold, 6.49%

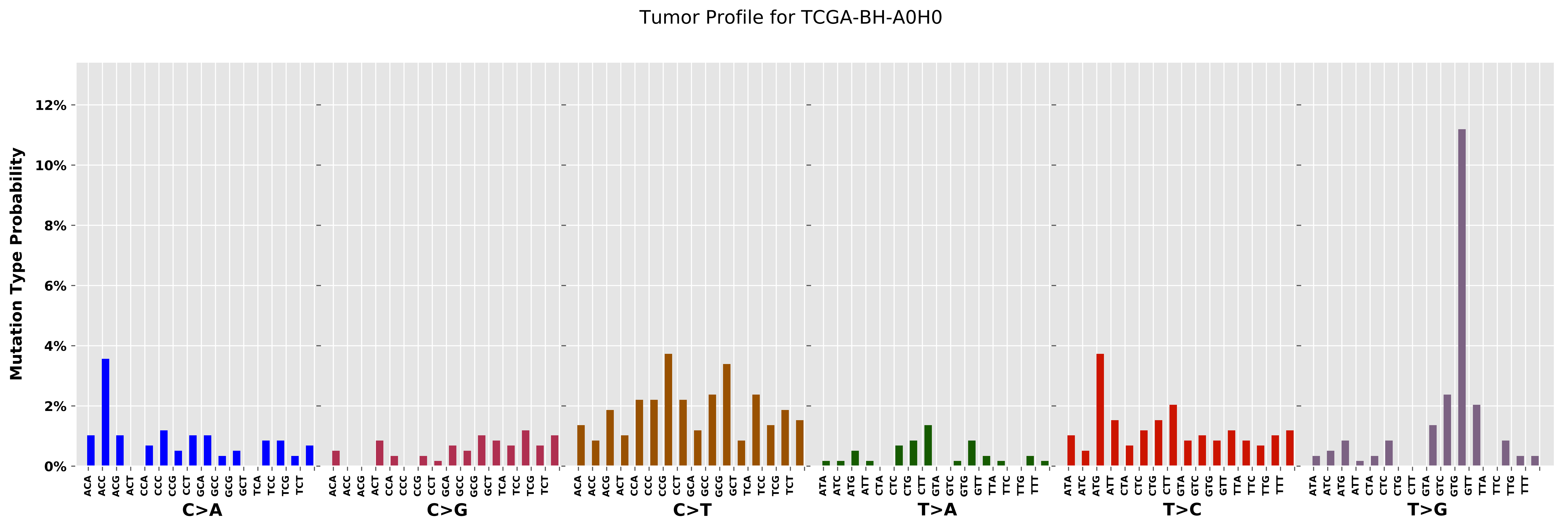

Tumor Profile for TCGA-BH-A0H0
Mutation Type Probability
0%
2%
4%
6%
8%
10%
12%
C>A
C>G
C>T
T>A
T>C
T>G

Cancer processes Weights for TCGA-EW-A1J5

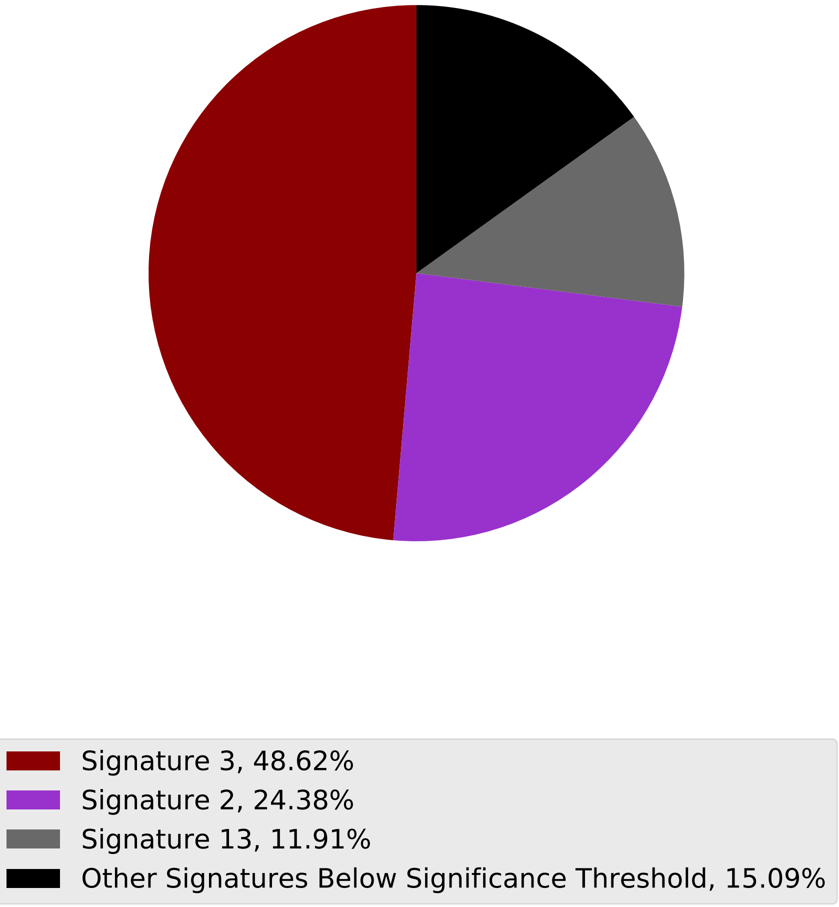

Tumor Profile for TCGA-EW-A1J5

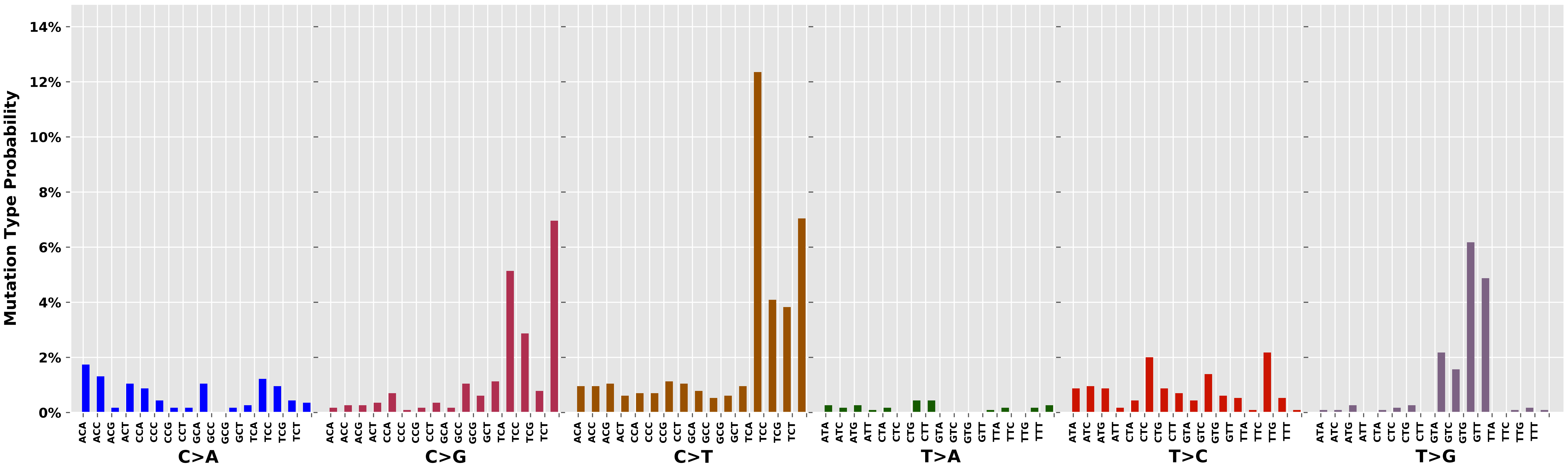

Cancer processes Weights for TCGA-EW-A1PC

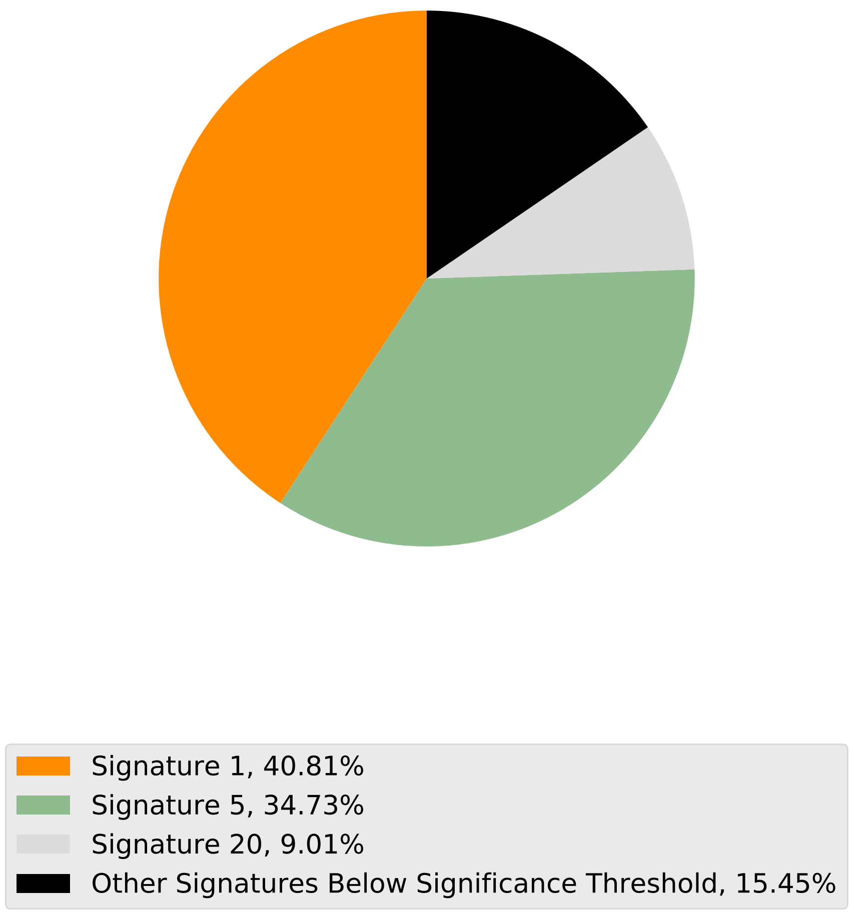

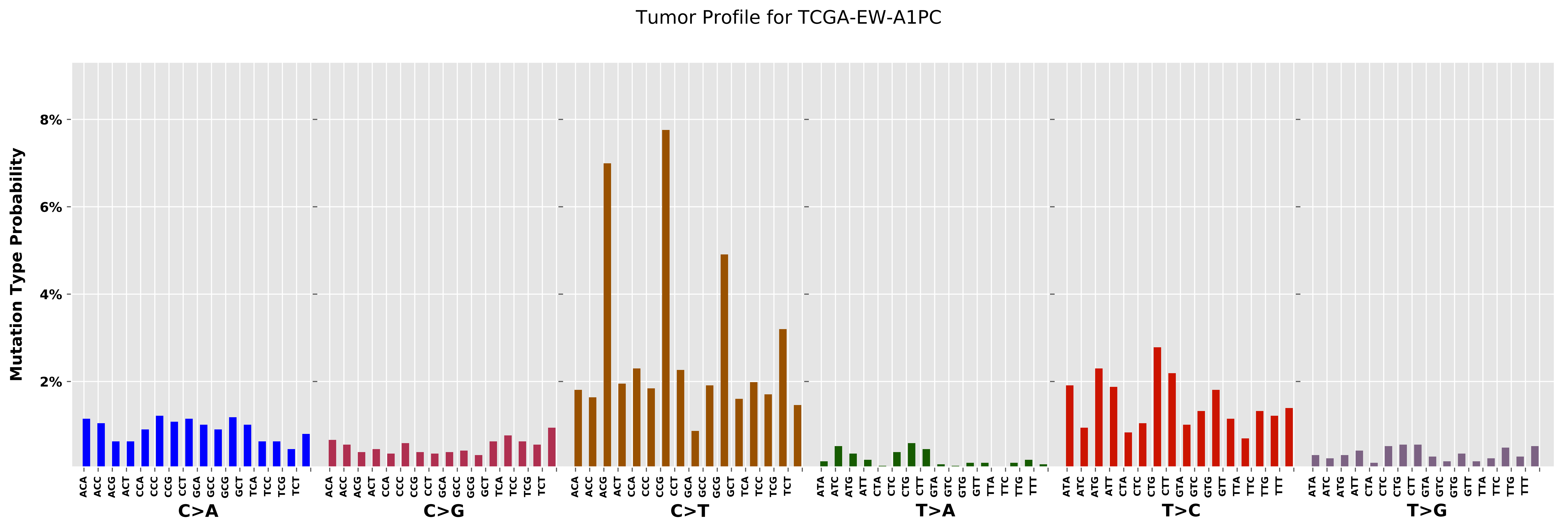

Tumor Profile for TCGA-EW-A1PC
Mutation Type Probability
8%
6%
4%
2%
ACA ACC ACG ACT CCA CCC CCG CCT GCA GCC GCG GCT TCA TCC TCG TCT
C>A
ACA ACC ACG ACT CCA CCC CCG CCT GCA GCC GCG GCT TCA TCC TCG TCT
C>G
ACA ACC ACG ACT CCA CCC CCG CCT GCA GCC GCG GCT TCA TCC TCG TCT
C>T
ATA ATC ATG ATT CTA CTC CTG CTT GTA GTC GTG GTT TTA TTC TTG TTT
T>A
ATA ATC ATG ATT CTA CTC CTG CTT GTA GTC GTG GTT TTA TTC TTG TTT
T>C
ATA ATC ATG ATT CTA CTC CTG CTT GTA GTC GTG GTT TTA TTC TTG TTT
T>G

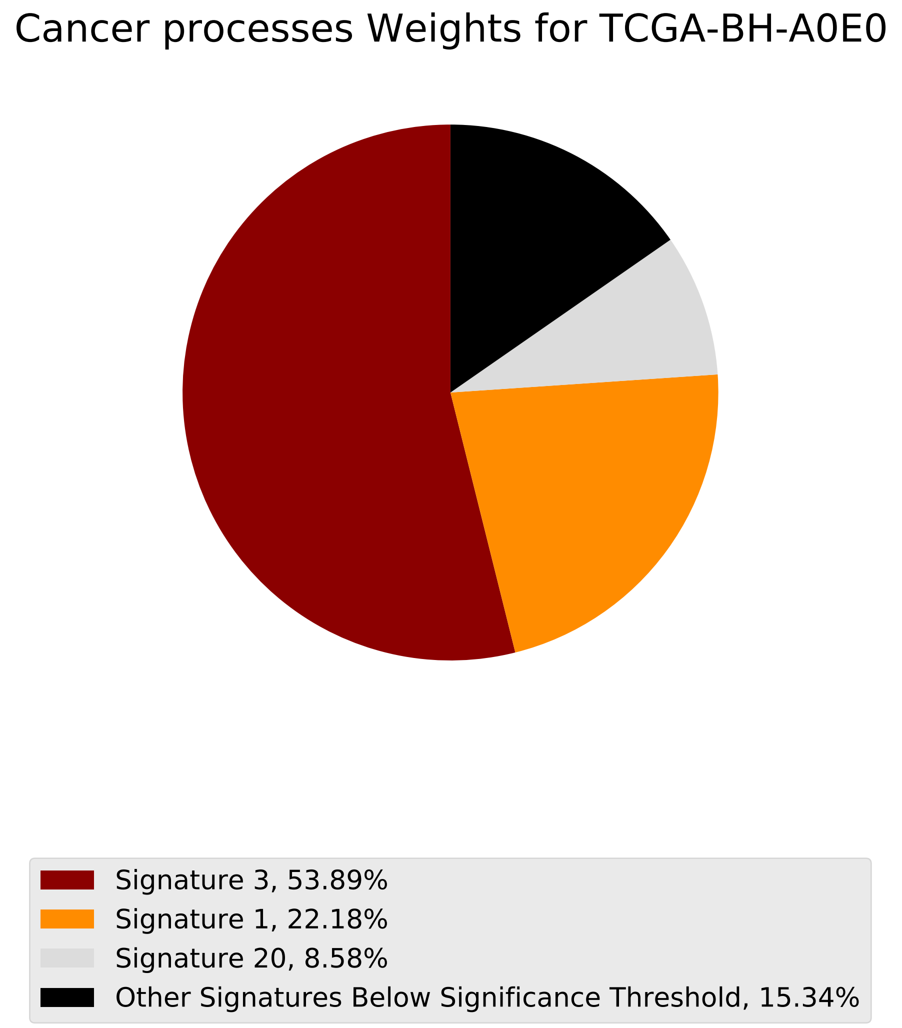

Cancer processes Weights for TCGA-BH-A0E0
Signature 3, 53.89%
Signature 1, 22.18%
Signature 20, 8.58%
Other Signatures Below Significance Threshold, 15.34%

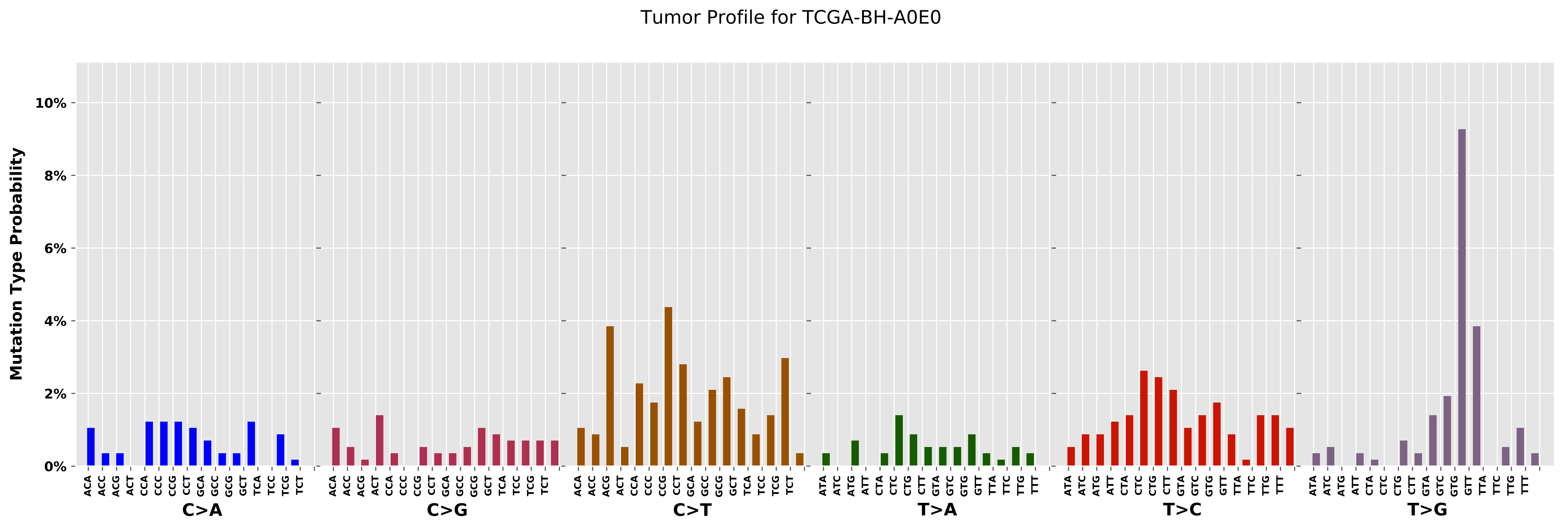
Tumor Profile for TCGA-BH-A0E0
Mutation Type Probability
10%
8%
6%
4%
2%
0%
C>A
C>G
C>T
T>A
T>C
T>G
ACA ACC ACG ACT CCA CCC CCG CCT GCA GCC GCG GCT TCA TCC TCG TCT
ATA ATC ATG ATT CTA CTC CTG CTT GTA GTC GTG GTT TTA TTC TTG TTT

Cancer processes Weights for TCGA-A2-A0D0

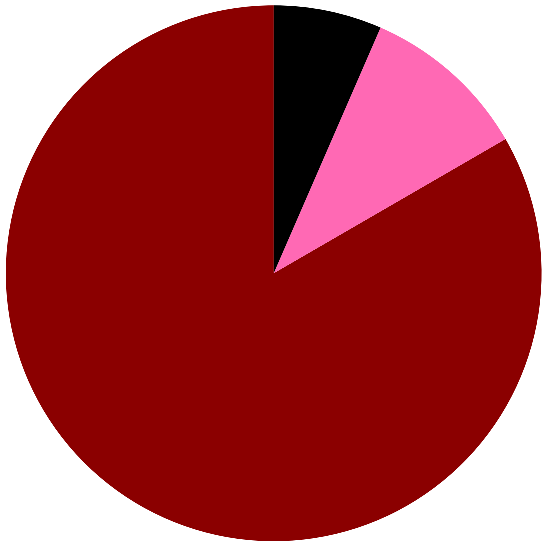

Signature 3, 83.35%
Signature 29, 10.12%
Other Signatures Below Significance Threshold, 6.53%

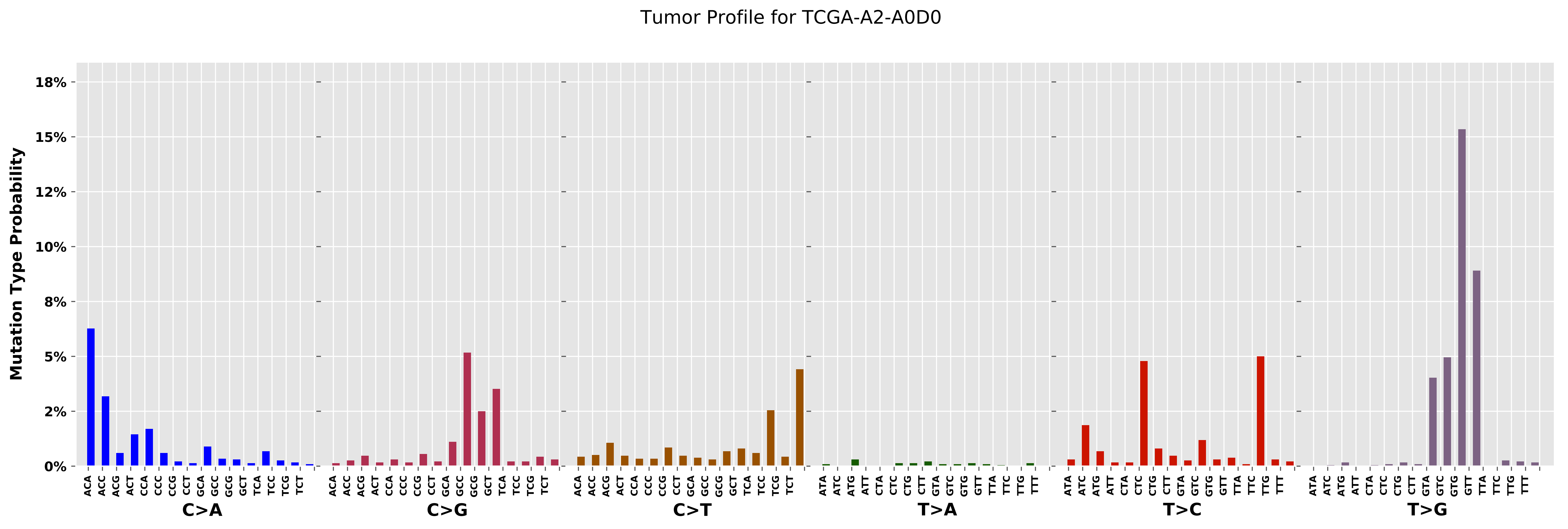

Tumor Profile for TCGA-A2-A0D0
Mutation Type Probability
18%
15%
12%
10%
8%
5%
2%
0%
C>A
C>G
C>T
T>A
T>C
T>G

Cancer processes Weights for TCGA-AA-3956

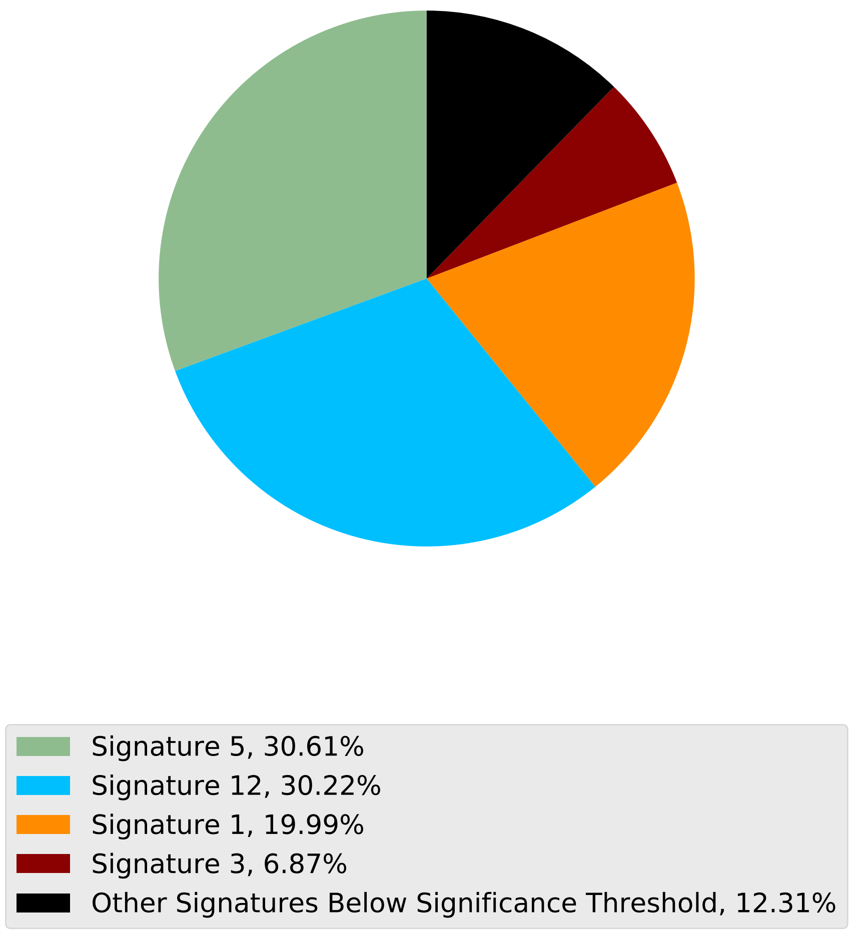

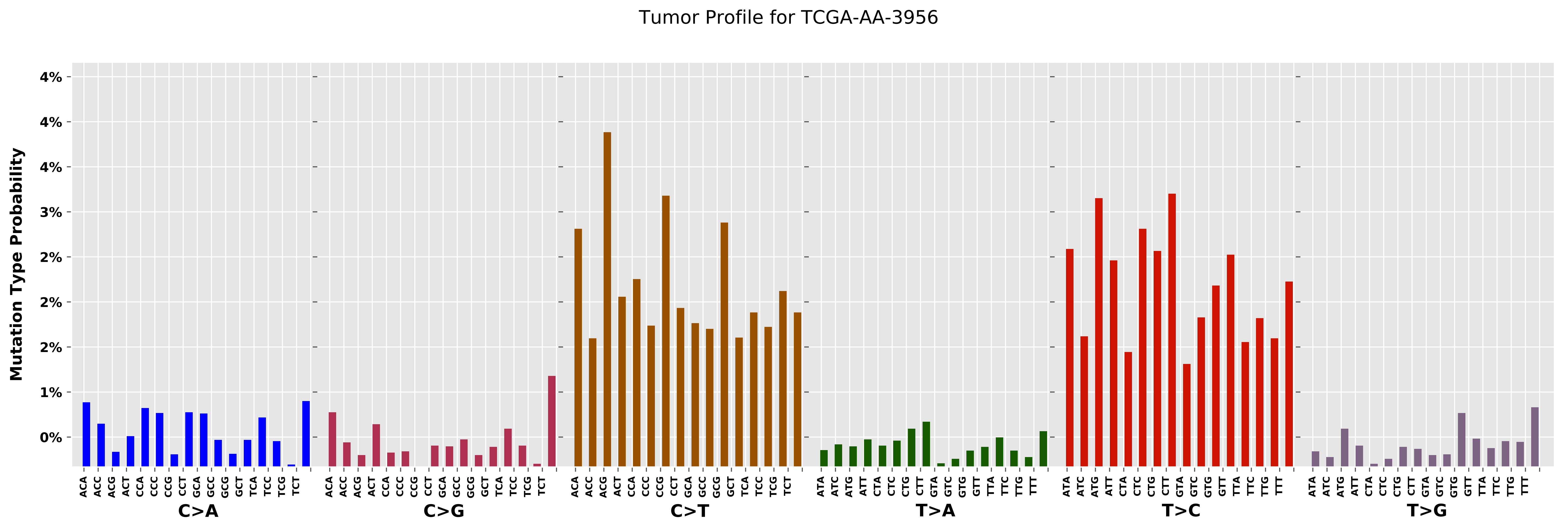
Tumor Profile for TCGA-AA-3956
Mutation Type Probability
4%
4%
4%
3%
2%
2%
2%
1%
0%
C>A
C>G
C>T
T>A
T>C
T>G
ACA ACC ACG ACT CCA CCC CCG CCT GCA GCC GCG GCT TCA TCC TCG TCT
ATA ATC ATG ATT CTA CTC CTG CTT GTA GTC GTG GTT TTA TTC TTG TTT

Cancer processes Weights for TCGA-AR-A0TX

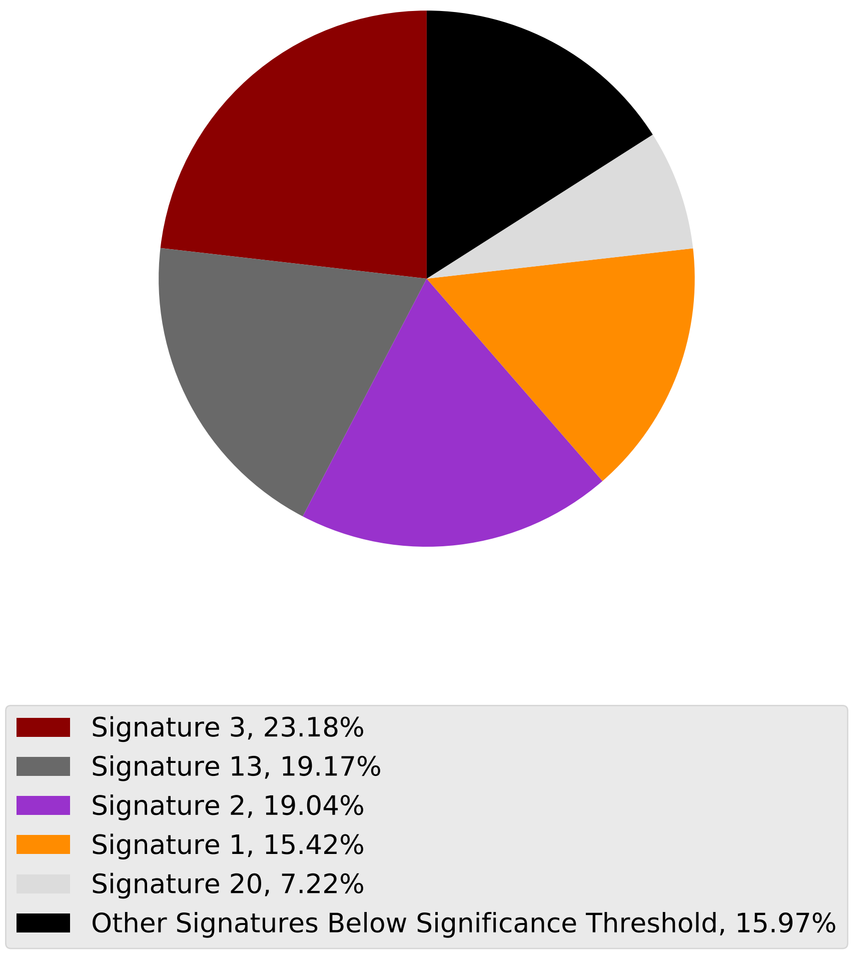

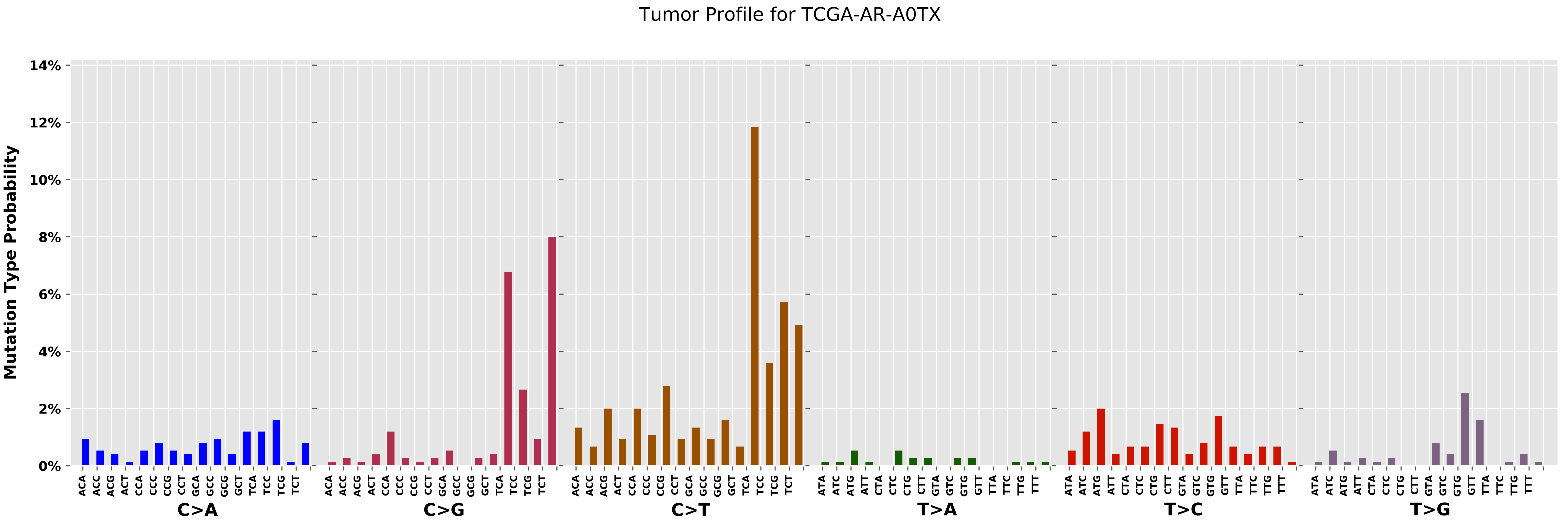
Tumor Profile for TCGA-AR-A0TX
Mutation Type Probability
14%
12%
10%
8%
6%
4%
2%
0%
ACA ACC ACG ACT CCA CCC CCG CCT GCA GCC GCG GCT TCA TCC TCG TCT
C>A
ACA ACC ACG ACT CCA CCC CCG CCT GCA GCC GCG GCT TCA TCC TCG TCT
C>G
ACA ACC ACG ACT CCA CCC CCG CCT GCA GCC GCG GCT TCA TCC TCG TCT
C>T
ATA ATC ATG ATT CTA CTC CTG CTT GTA GTC GTG GTT TTA TTC TTG TTT
T>A
ATA ATC ATG ATT CTA CTC CTG CTT GTA GTC GTG GTT TTA TTC TTG TTT
T>C
ATA ATC ATG ATT CTA CTC CTG CTT GTA GTC GTG GTT TTA TTC TTG TTT
T>G

Cancer processes Weights for TCGA-EI-6917

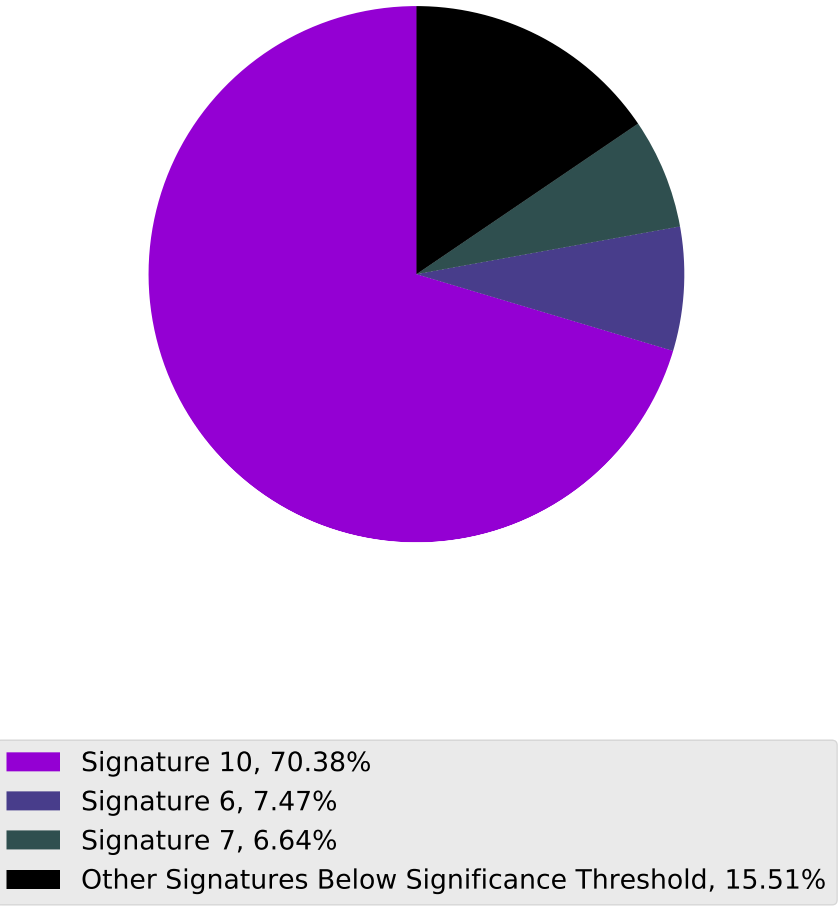

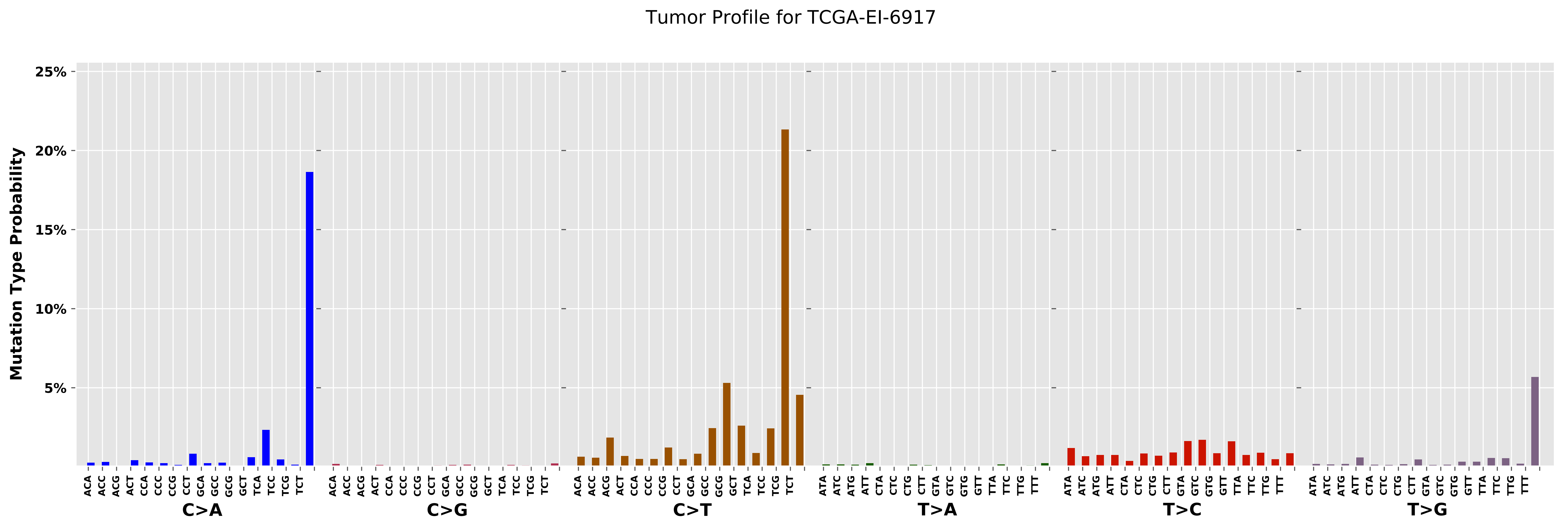
Tumor Profile for TCGA-EI-6917
Mutation Type Probability
25%
20%
15%
10%
5%
C>A
C>G
C>T
T>A
T>C
T>G
ACA ACC ACG ACT CCA CCC CCG CCT GCA GCC GCG GCT TCA TCC TCG TCT
ATA ATC ATG ATT CTA CTC CTG CTT GTA GTC GTG GTT TTA TTC TTG TTT

Cancer processes Weights for TCGA-AA-A01X

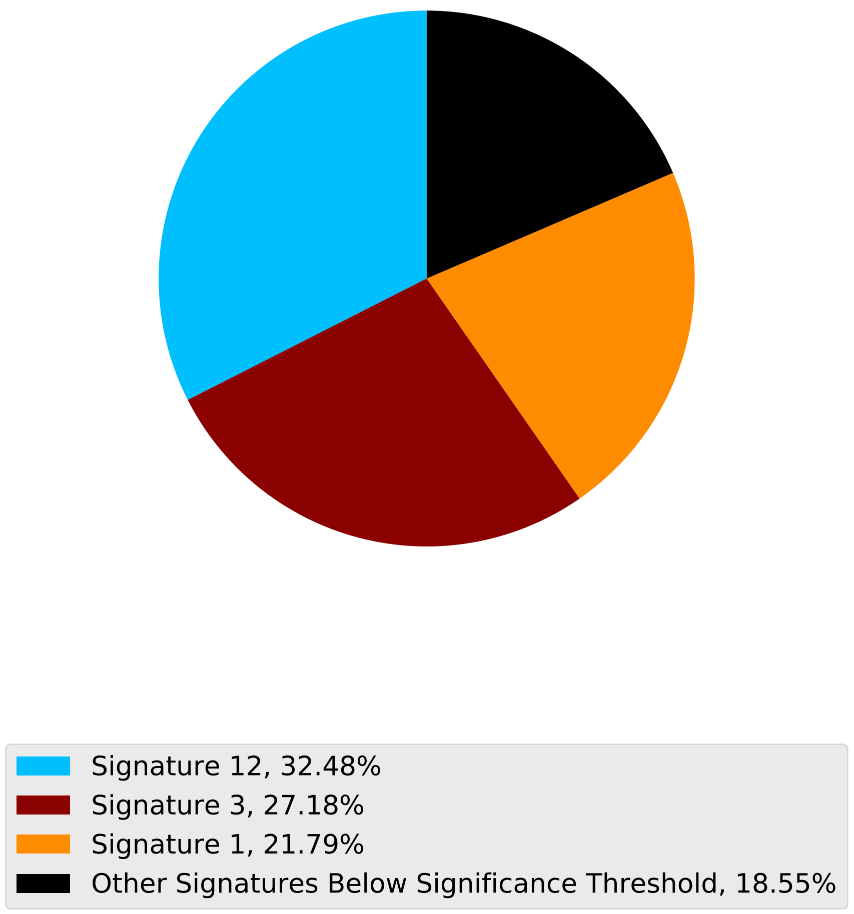

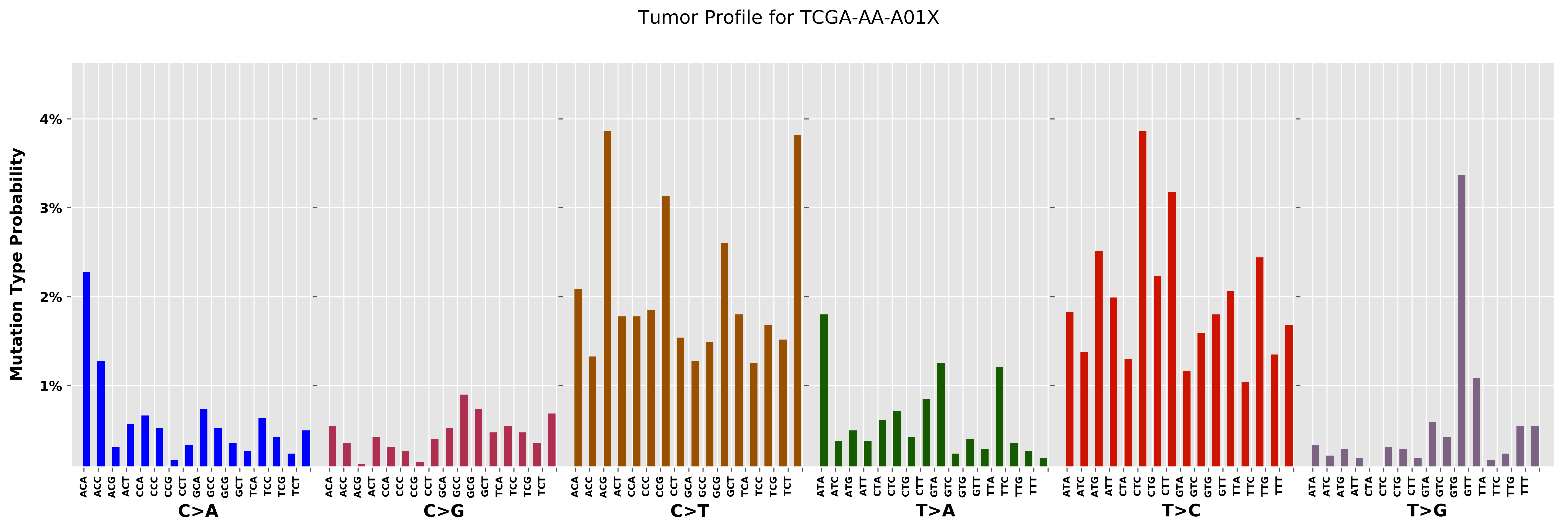

Tumor Profile for TCGA-AA-A01X
Mutation Type Probability
4%
3%
2%
1%
C>A
C>G
C>T
T>A
T>C
T>G
ACA ACC ACG ACT CCA CCC CCG CCT GCA GCC GCG GCT TCA TCC TCG TCT
ATA ATC ATG ATT CTA CTC CTG CTT GTA GTC GTG GTT TTA TTC TTG TTT

Cancer processes Weights for TCGA-E2-A15H

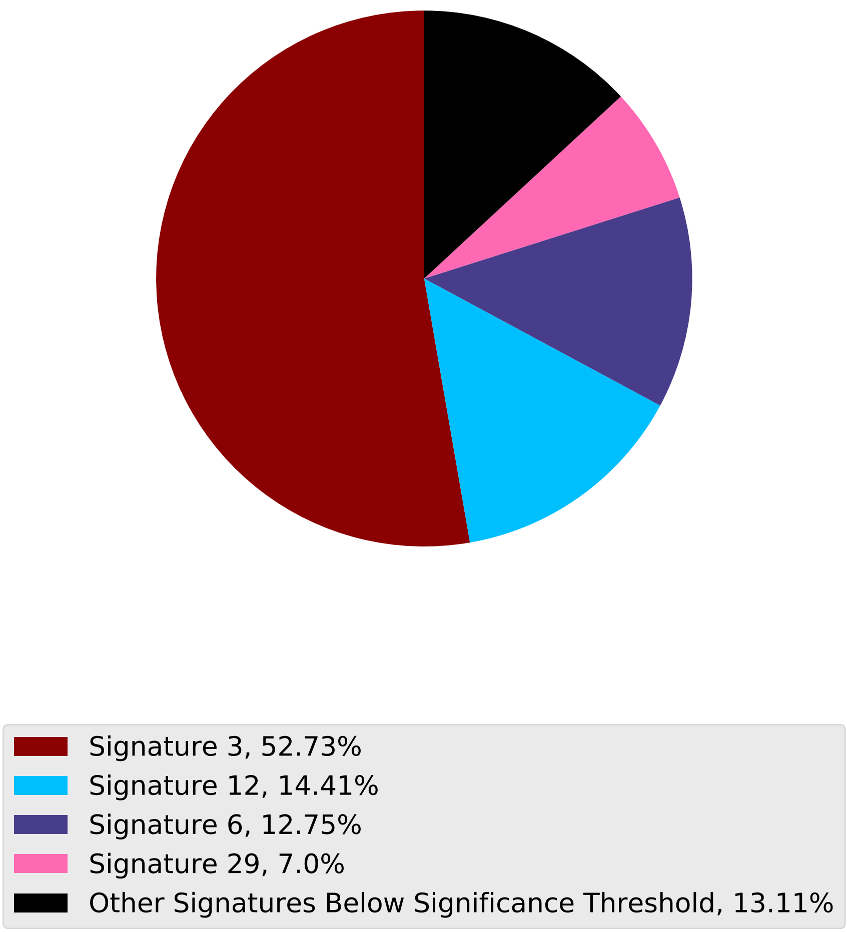

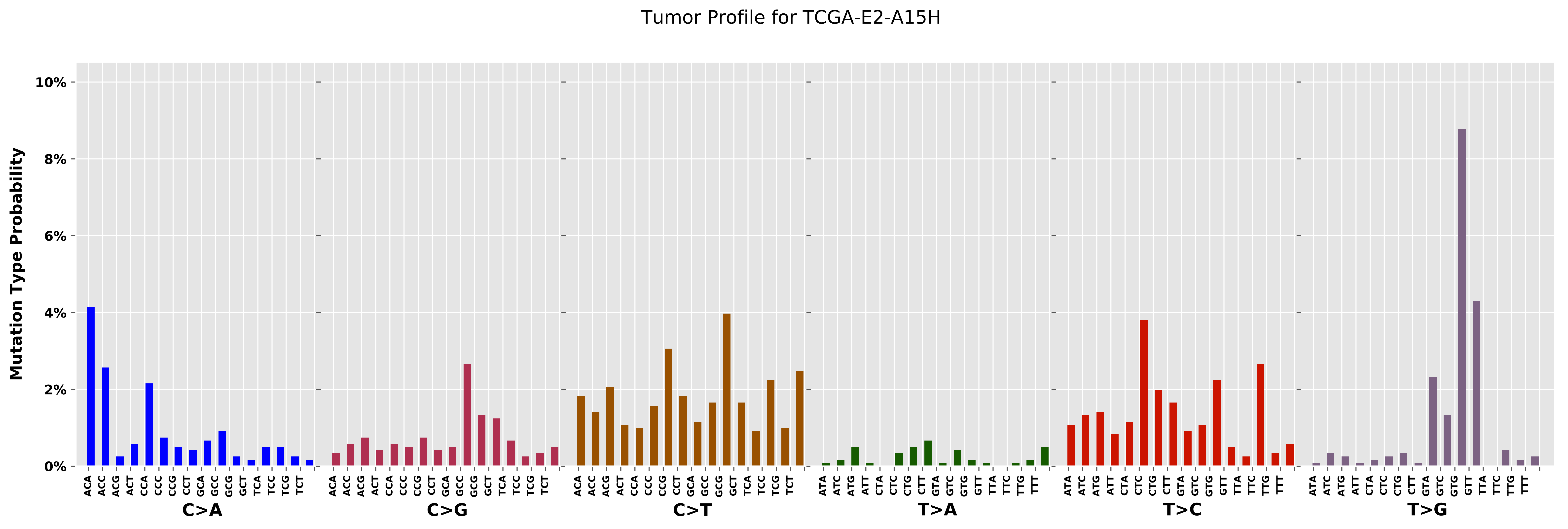
Tumor Profile for TCGA-E2-A15H
Mutation Type Probability
10%
8%
6%
4%
2%
0%
C>A
C>G
C>T
T>A
T>C
T>G
ACA ACC ACG ACT CCA CCC CCG CCT GCA GCC GCG GCT TCA TCC TCG TCT
ATA ATC ATG ATT CTA CTC CTG CTT GTA GTC GTG GTT TTA TTC TTG TTT

Cancer processes Weights for TCGA-E9-A1NH

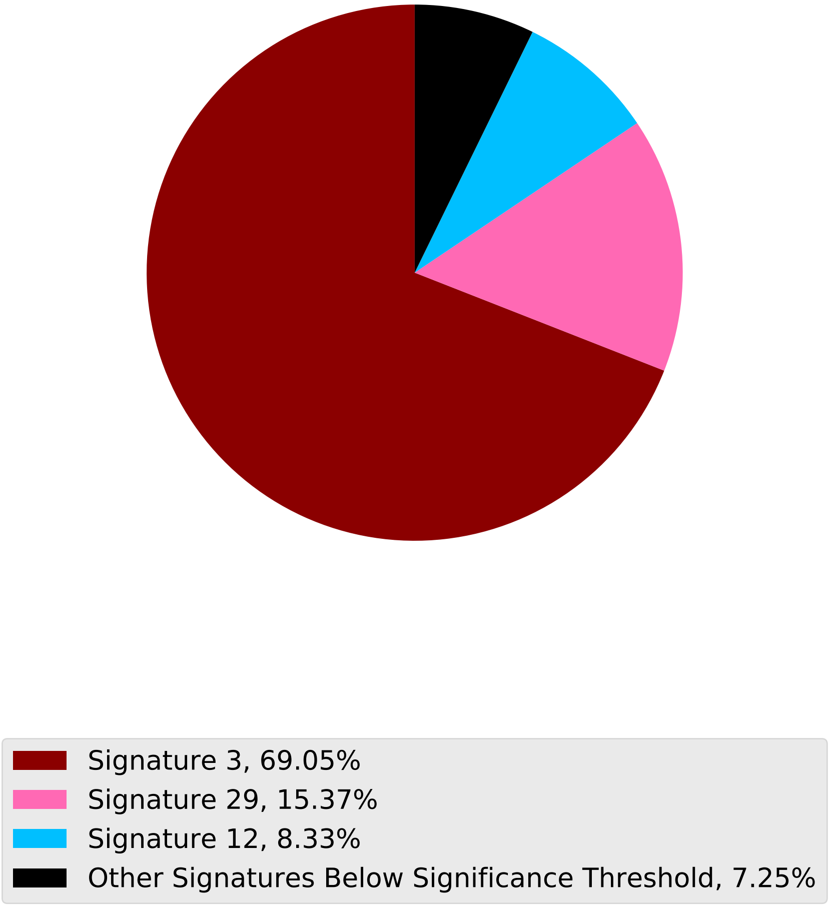

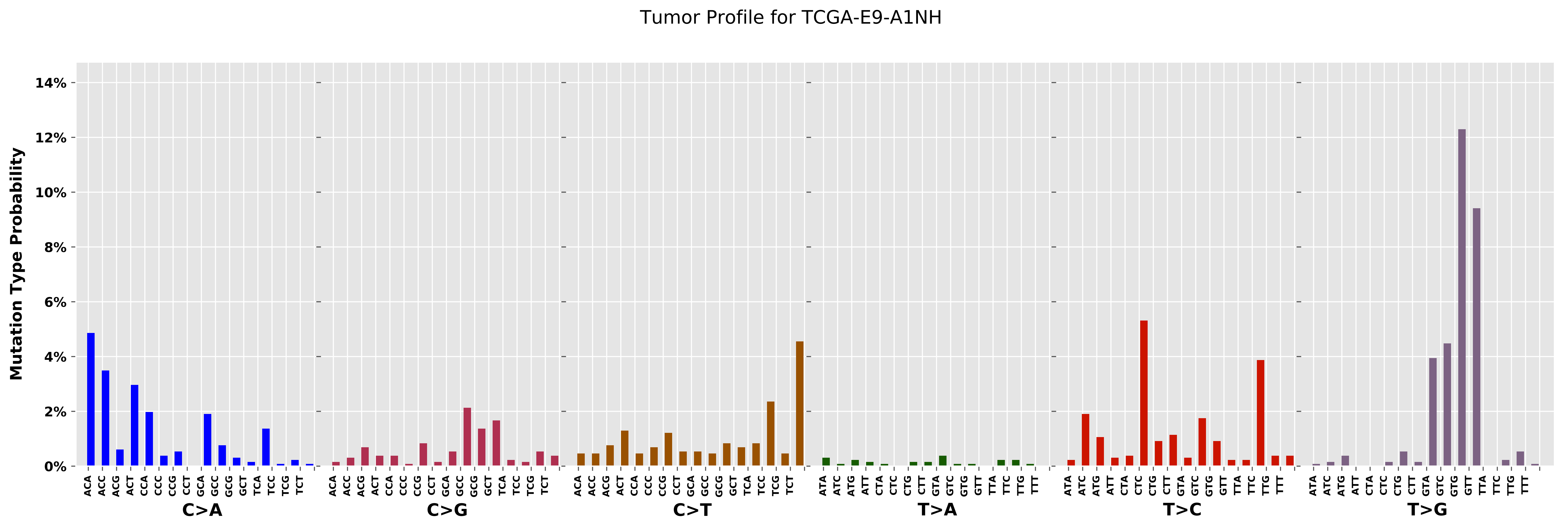

Tumor Profile for TCGA-E9-A1NH
Mutation Type Probability
0%
2%
4%
6%
8%
10%
12%
14%
C>A
C>G
C>T
T>A
T>C
T>G

Cancer processes Weights for TCGA-AA-A01R

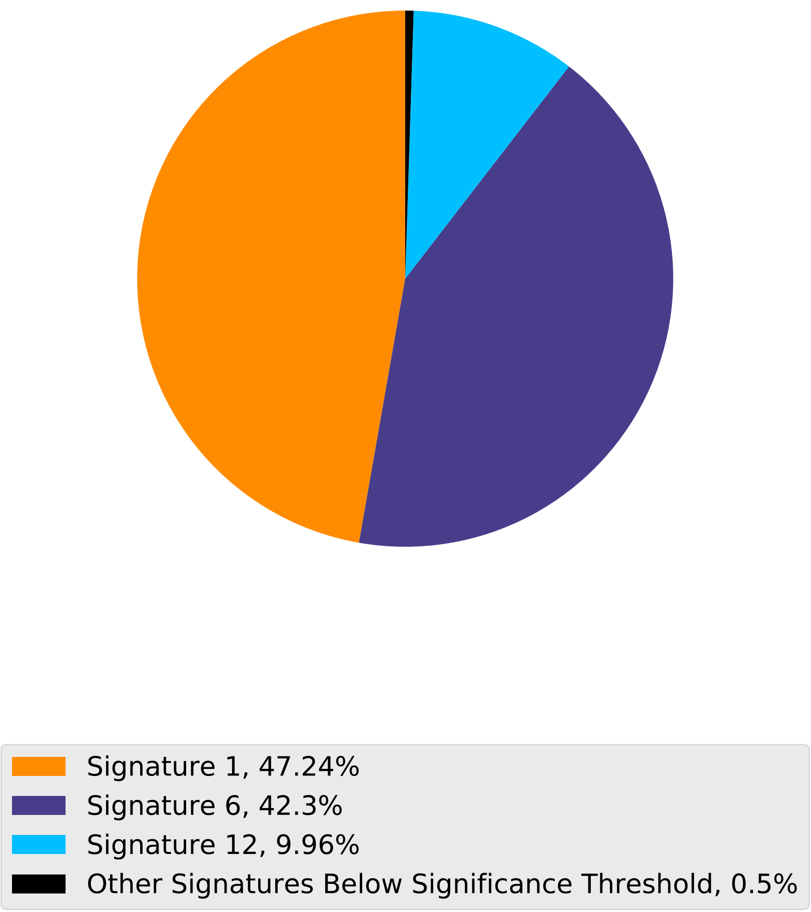

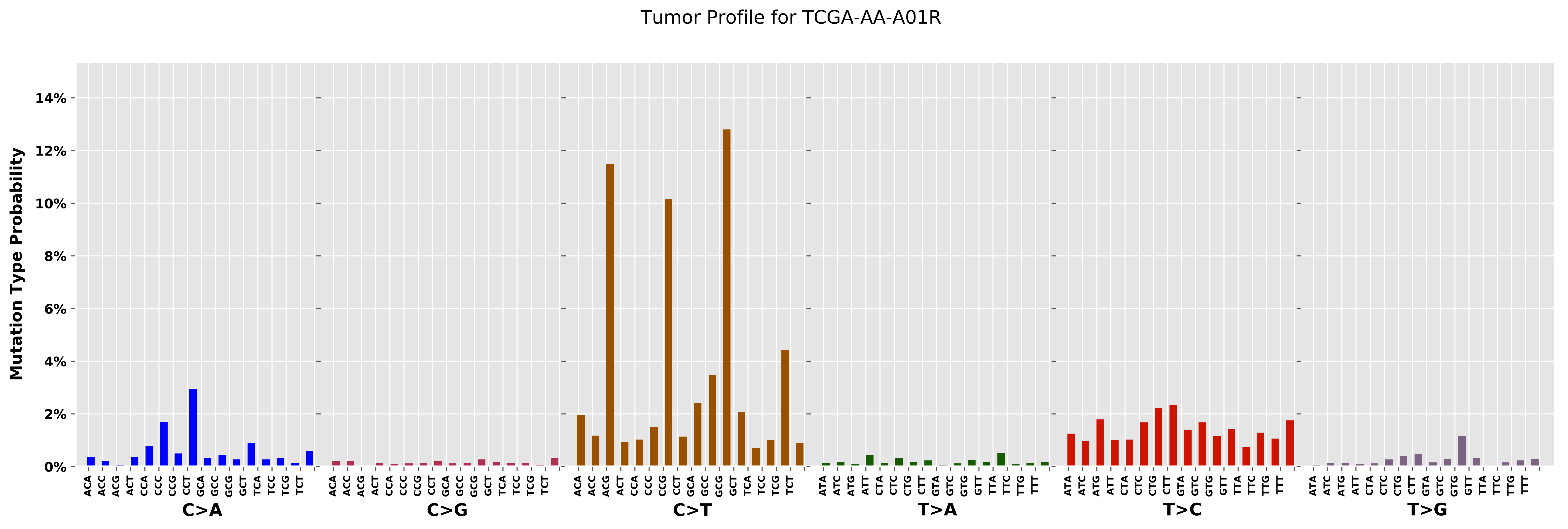
Tumor Profile for TCGA-AA-A01R
Mutation Type Probability
14%
12%
10%
8%
6%
4%
2%
0%
C>A
C>G
C>T
T>A
T>C
T>G
ACA ACC ACG ACT CCA CCC CCG CCT GCA GCC GCG GCT TCA TCC TCG TCT
ATA ATC ATG ATT CTA CTC CTG CTT GTA GTC GTG GTT TTA TTC TTG TTT

Cancer processes Weights for TCGA-AA-3666

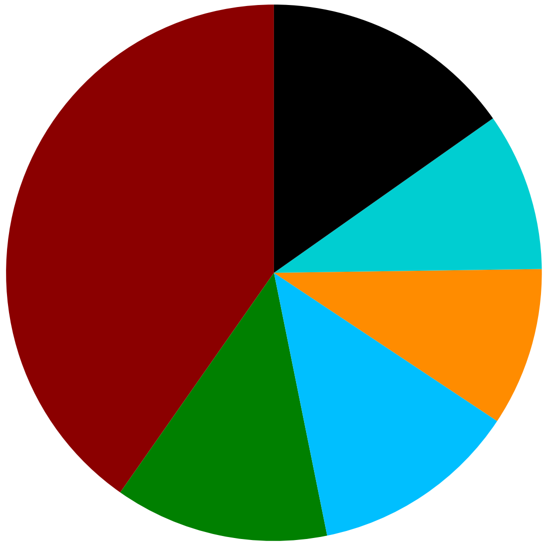

Signature 3, 40.27%
Signature 25, 12.91%
Signature 12, 12.49%
Signature 1, 9.55%
Signature 9, 9.54%
Other Signatures Below Significance Threshold, 15.23%

Tumor Profile for TCGA-AA-3666

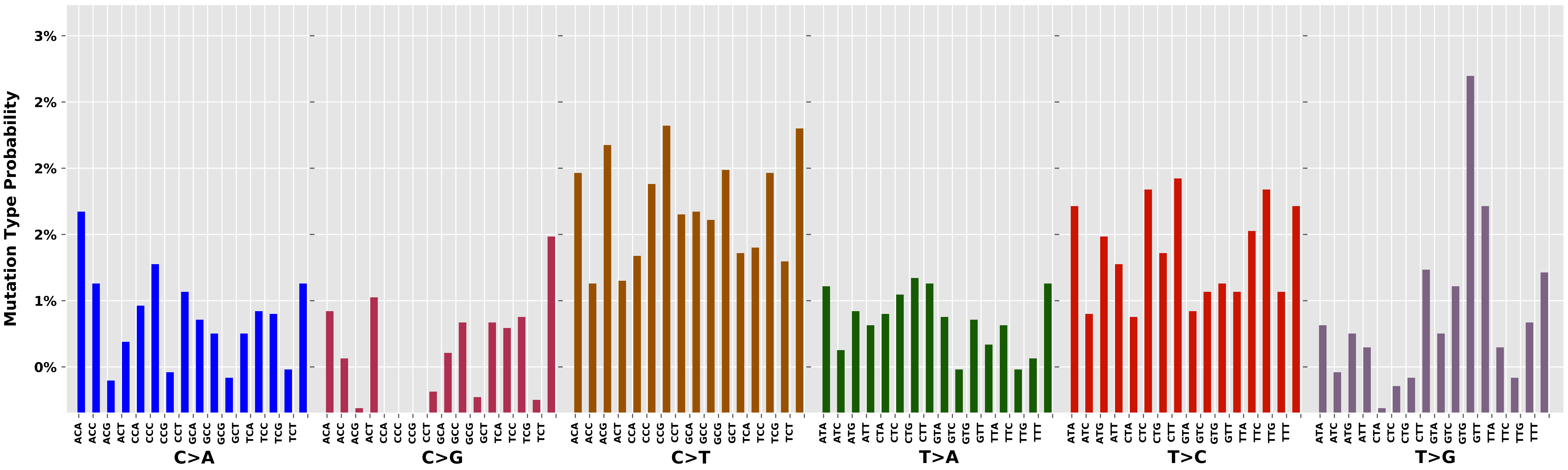

Cancer processes Weights for TCGA-AR-A2LK

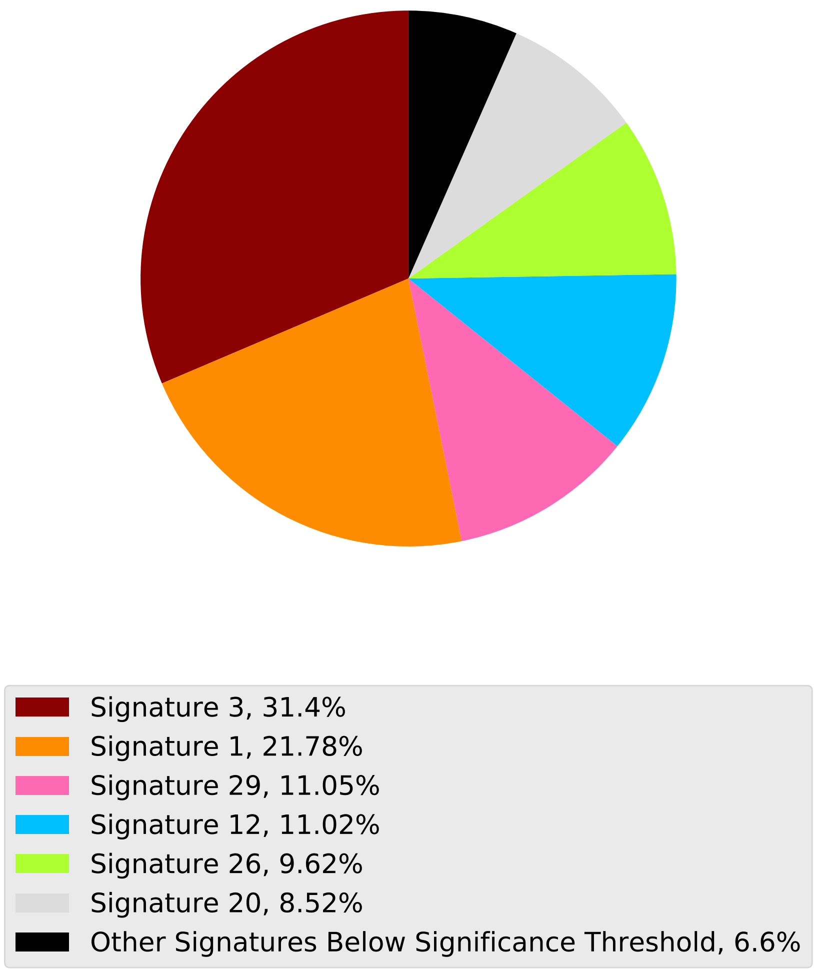

Tumor Profile for TCGA-AR-A2LK

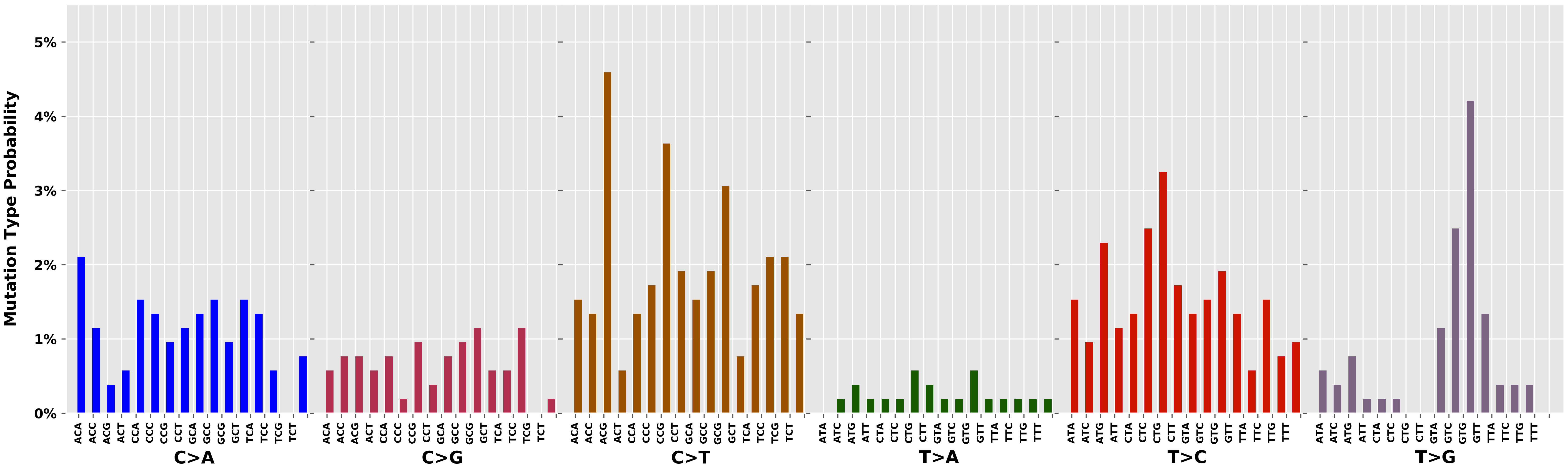

Cancer processes Weights for TCGA-AA-3534

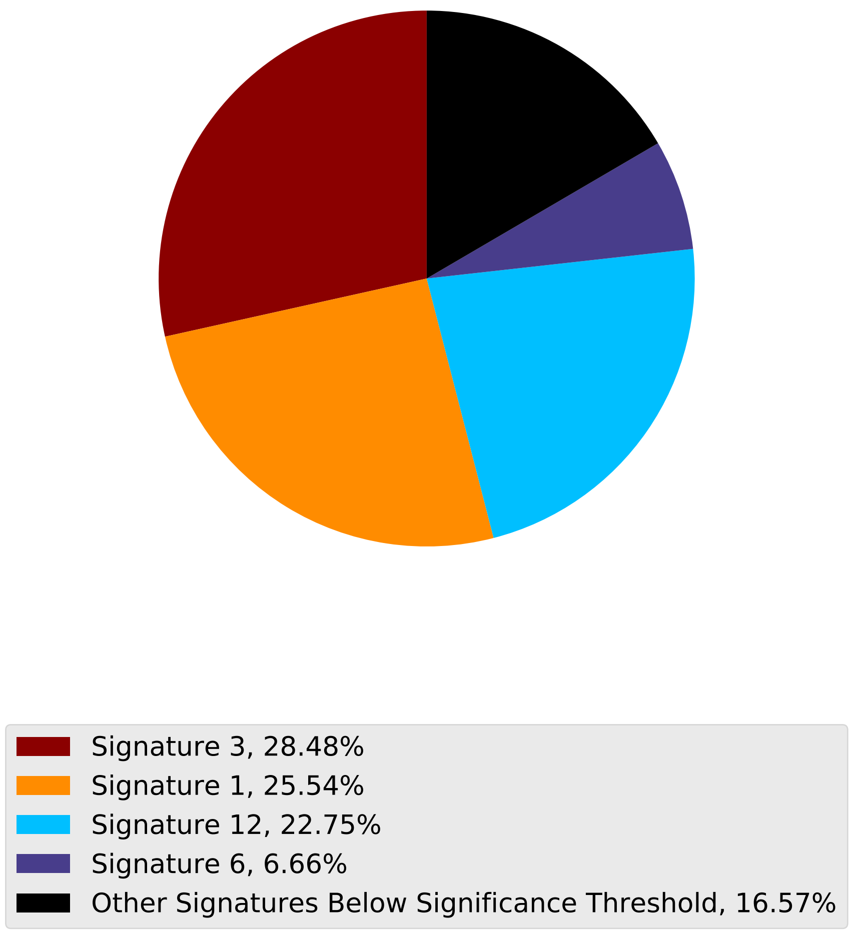

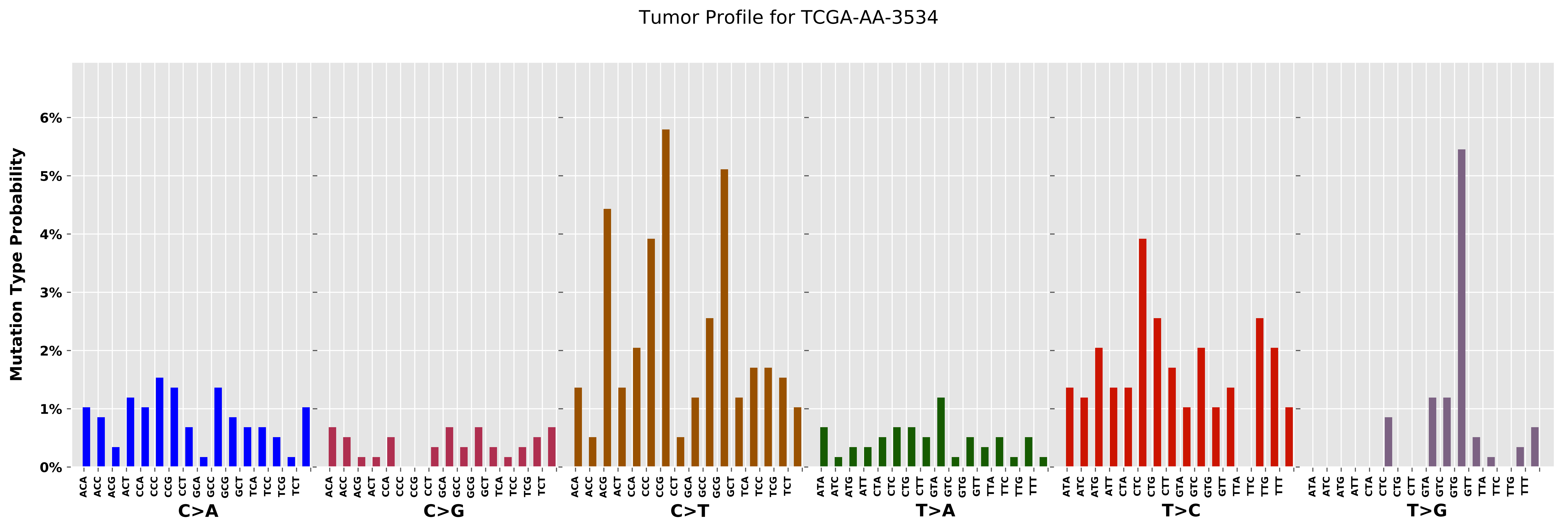

Tumor Profile for TCGA-AA-3534
Mutation Type Probability
0%
1%
2%
3%
4%
5%
6%
C>A
C>G
C>T
T>A
T>C
T>G

Cancer processes Weights for TCGA-A2-A25B

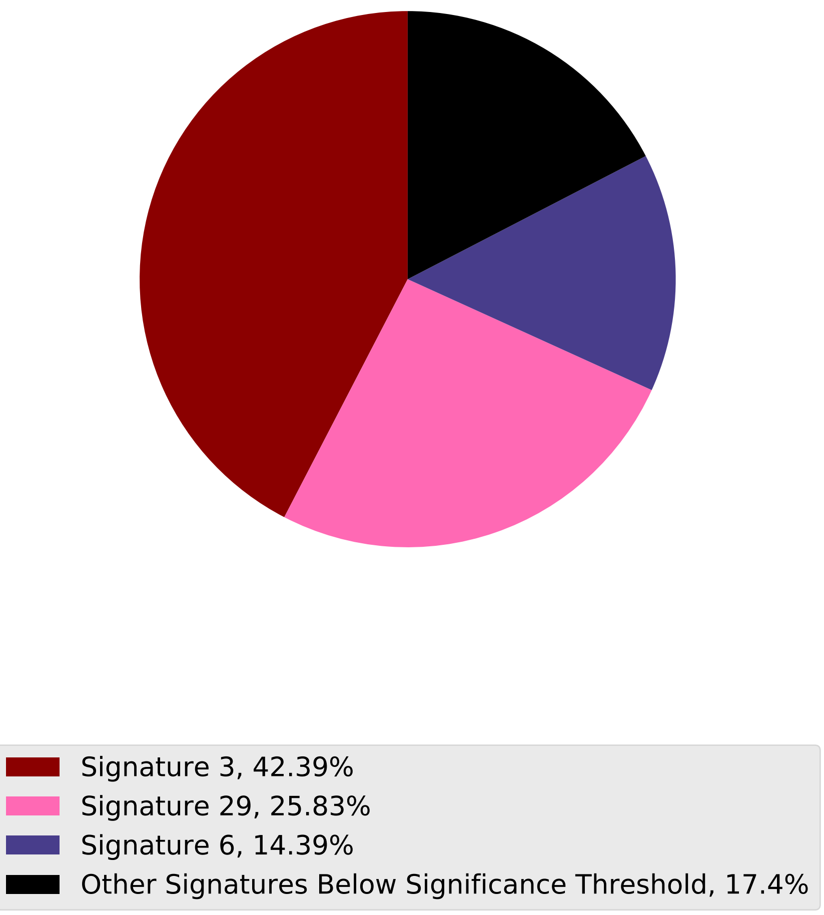

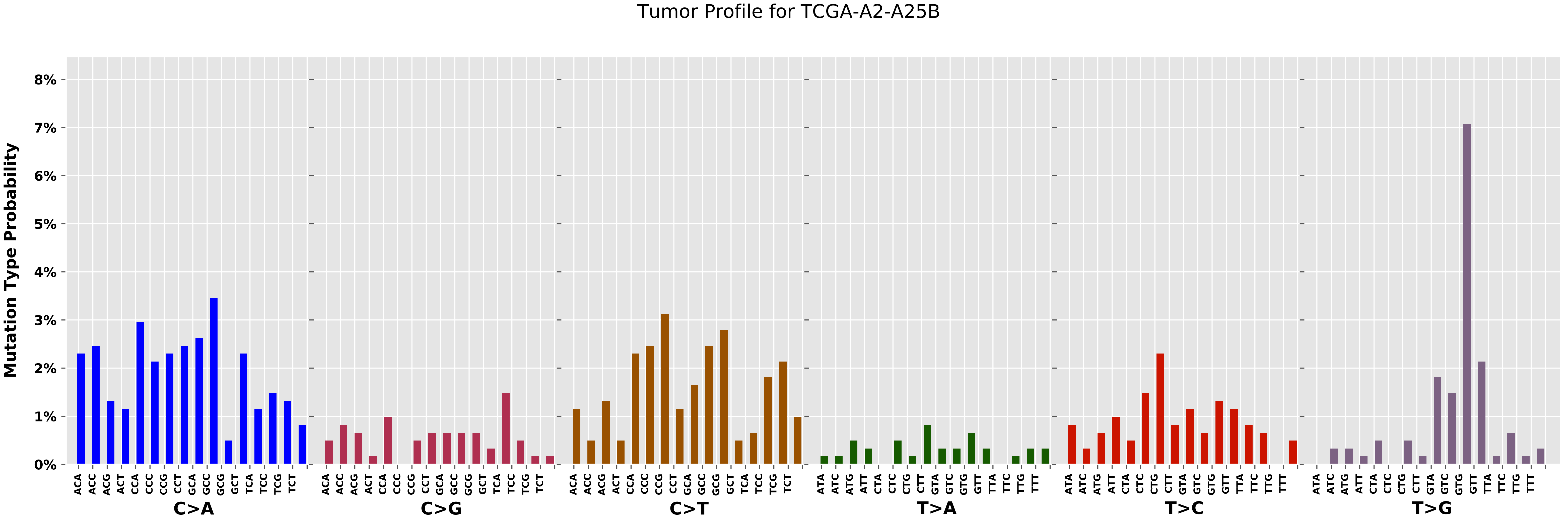

Tumor Profile for TCGA-A2-A25B
Mutation Type Probability
0%
1%
2%
3%
4%
5%
6%
7%
8%
C>A
C>G
C>T
T>A
T>C
T>G
ACA ACC ACG ACT CCA CCC CCG CCT GCA GCC GCG GCT TCA TCC TCG TCT
ATA ATC ATG ATT CTA CTC CTG CTT GTA GTC GTG GTT TTA TTC TTG TTT

Cancer processes Weights for TCGA-AG-4008

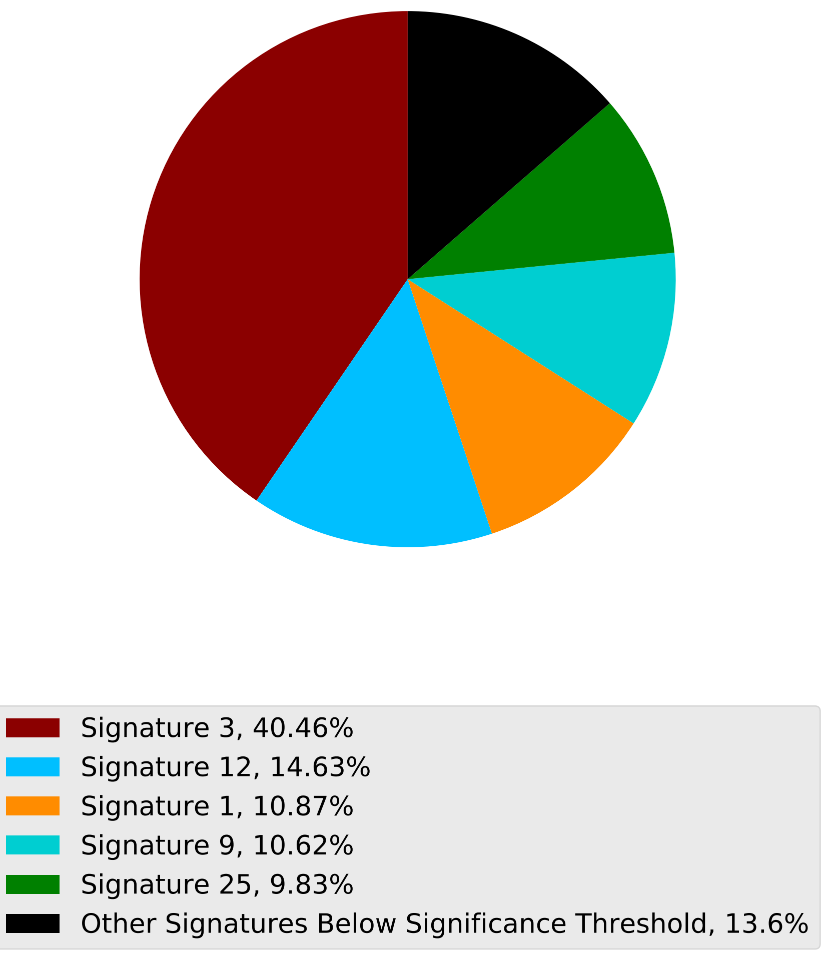

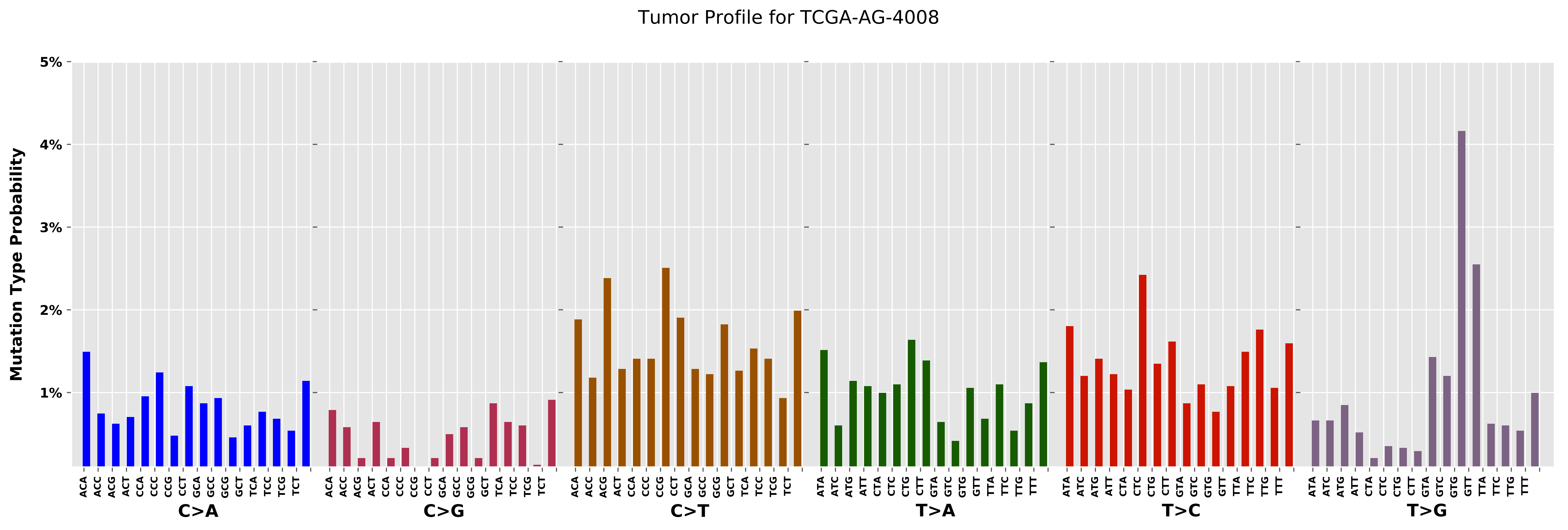

Tumor Profile for TCGA-AG-4008
Mutation Type Probability
5%
4%
3%
2%
1%
C>A
C>G
C>T
T>A
T>C
T>G

Cancer processes Weights for TCGA-A7-A26G

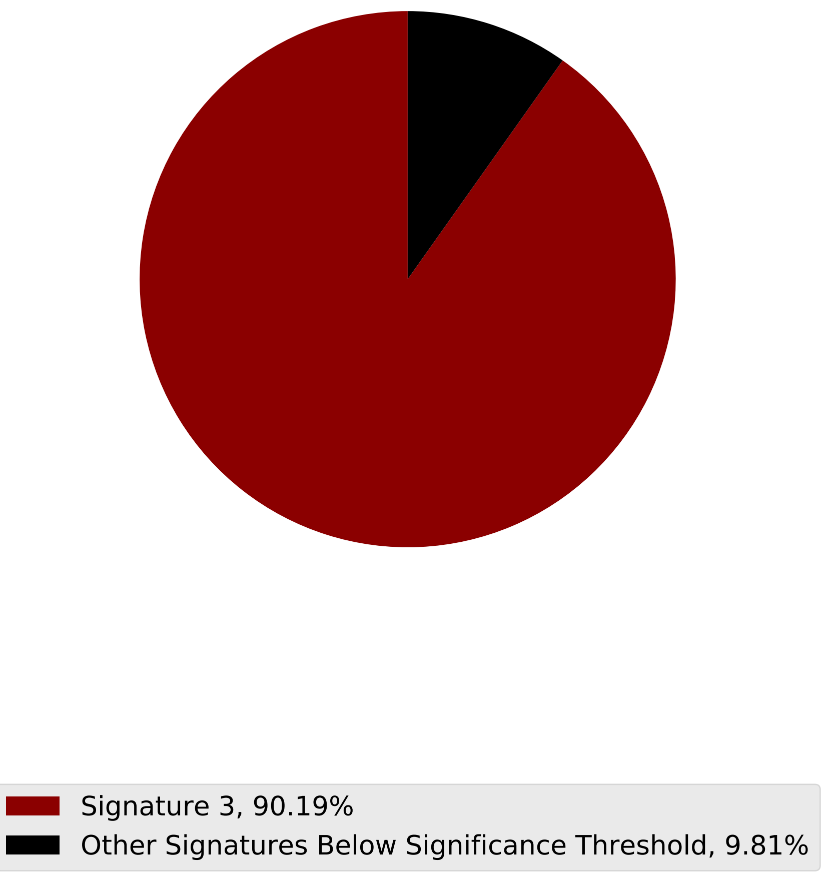

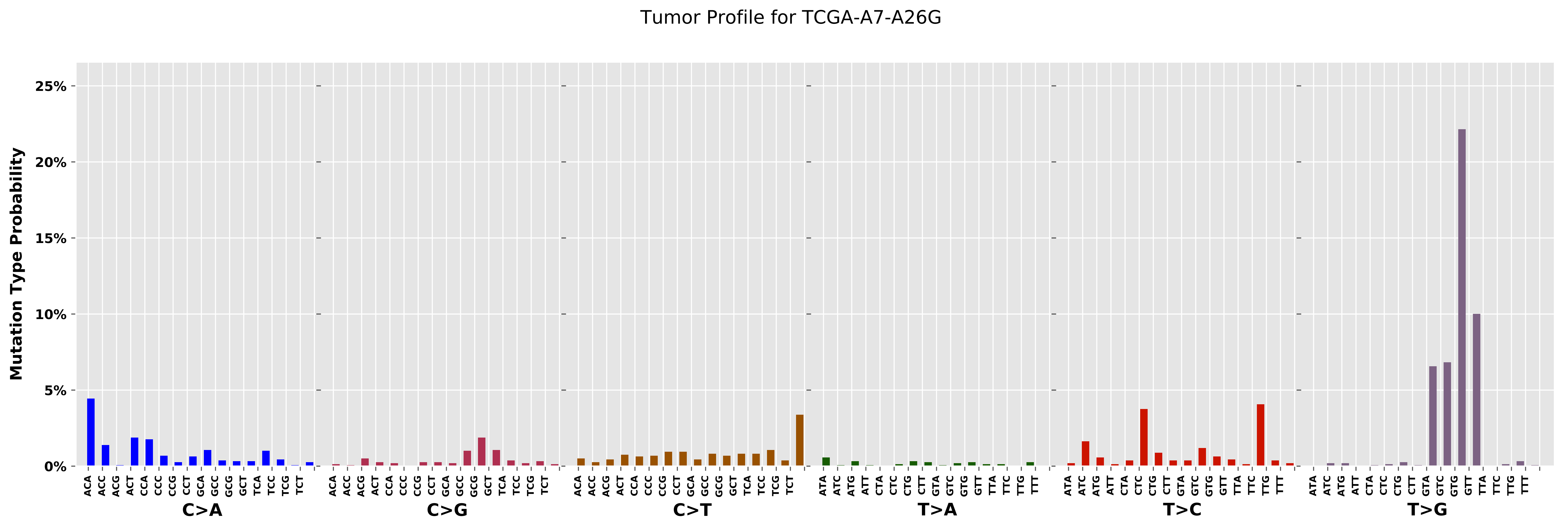
Tumor Profile for TCGA-A7-A26G
Mutation Type Probability
25%
20%
15%
10%
5%
0%
ACA ACC ACG ACT CCA CCC CCG CCT GCA GCC GCG GCT TCA TCC TCG TCT
C>A
ACA ACC ACG ACT CCA CCC CCG CCT GCA GCC GCG GCT TCA TCC TCG TCT
C>G
ACA ACC ACG ACT CCA CCC CCG CCT GCA GCC GCG GCT TCA TCC TCG TCT
C>T
ATA ATC ATG ATT CTA CTC CTG CTT GTA GTC GTG GTT TTA TTC TTG TTT
T>A
ATA ATC ATG ATT CTA CTC CTG CTT GTA GTC GTG GTT TTA TTC TTG TTT
T>C
ATA ATC ATG ATT CTA CTC CTG CTT GTA GTC GTG GTT TTA TTC TTG TTT
T>G

Cancer processes Weights for TCGA-AR-A24Z

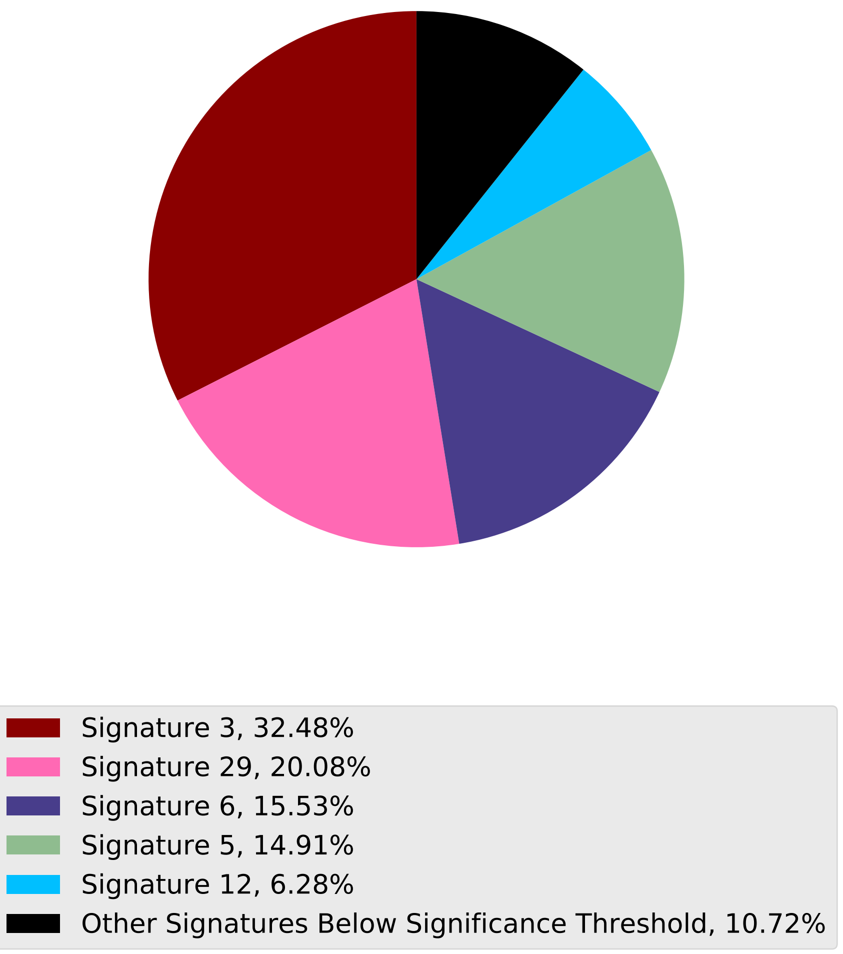

Tumor Profile for TCGA-AR-A24Z

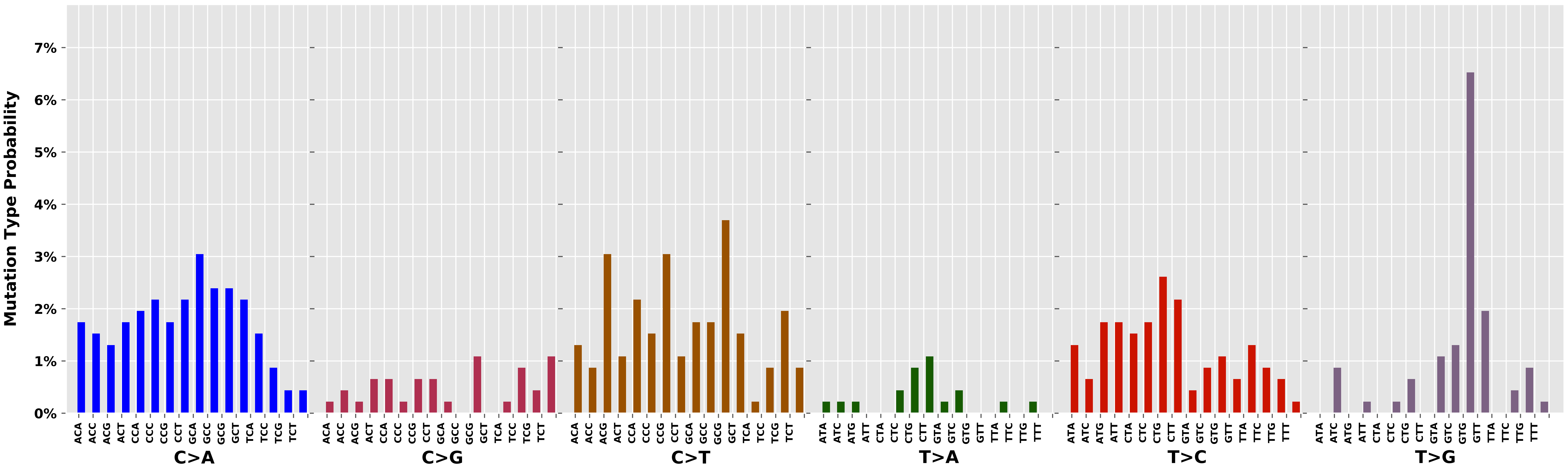

Cancer processes Weights for TCGA-A2-A04P

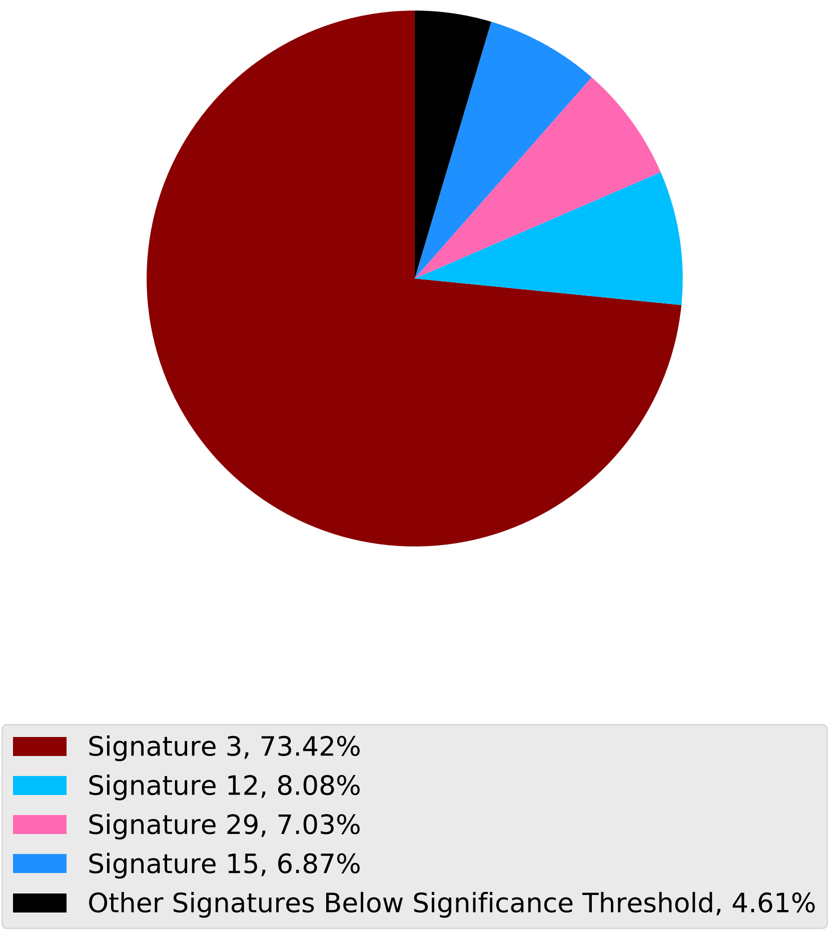

Tumor Profile for TCGA-A2-A04P

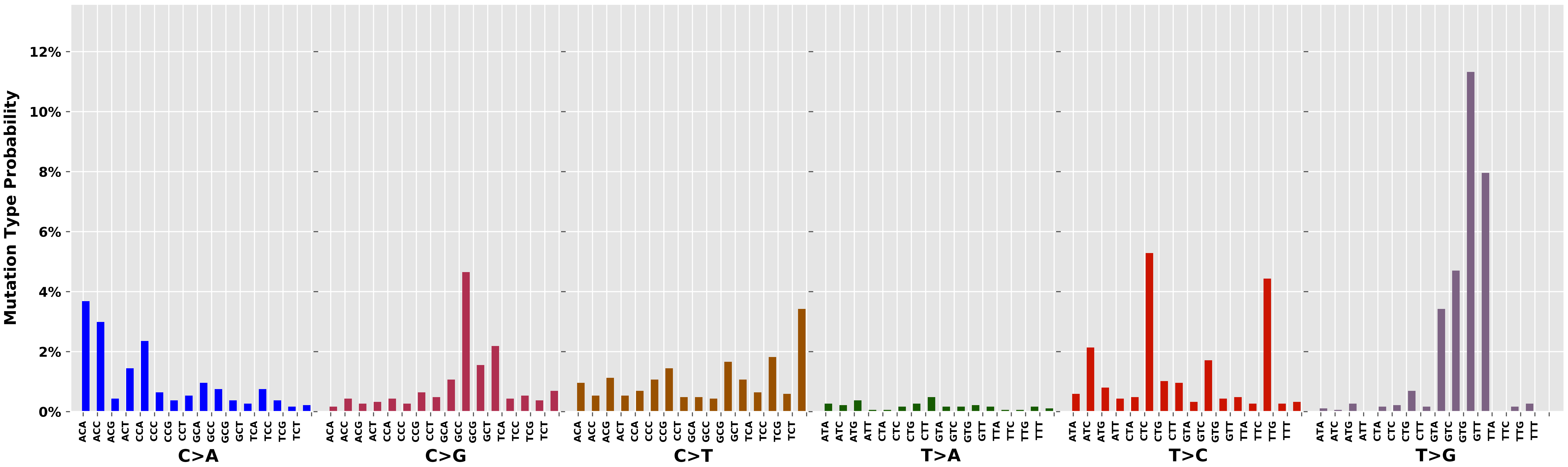

Cancer processes Weights for TCGA-CA-6718

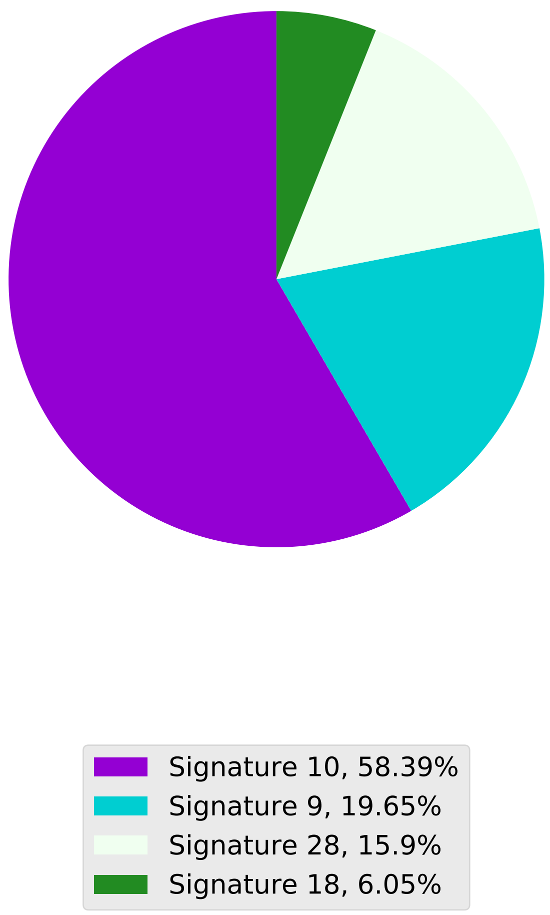

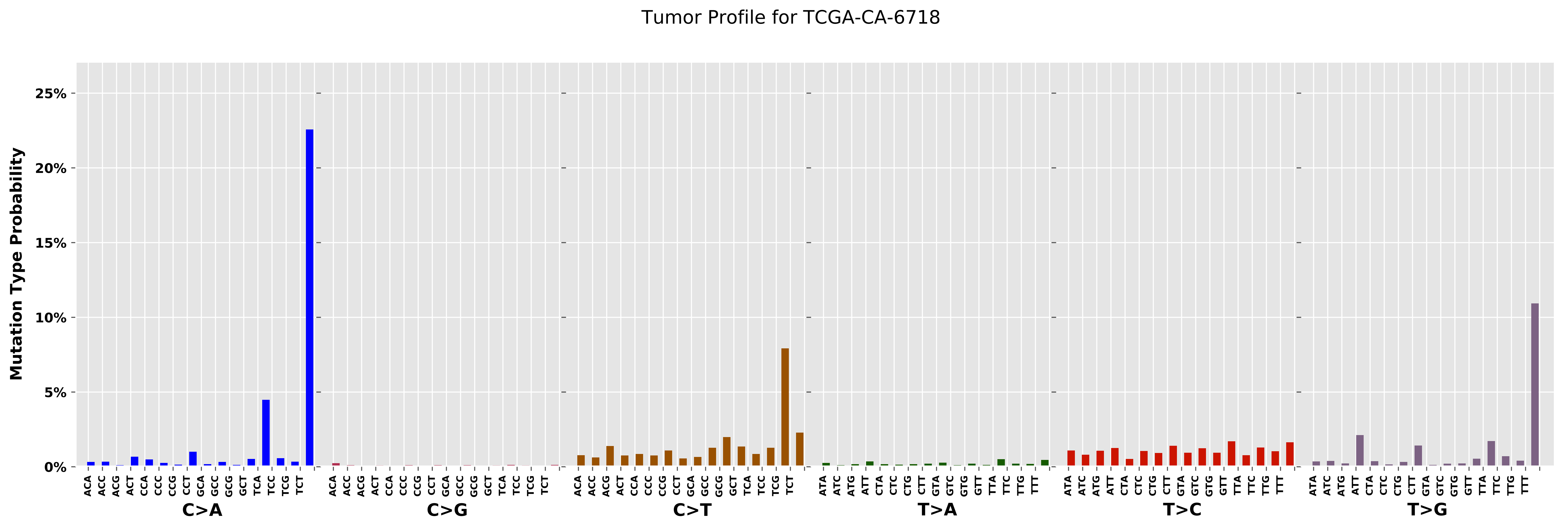

Tumor Profile for TCGA-CA-6718
Mutation Type Probability
25%
20%
15%
10%
5%
0%
C>A
C>G
C>T
T>A
T>C
T>G

Cancer processes Weights for TCGA-BH-A0AV

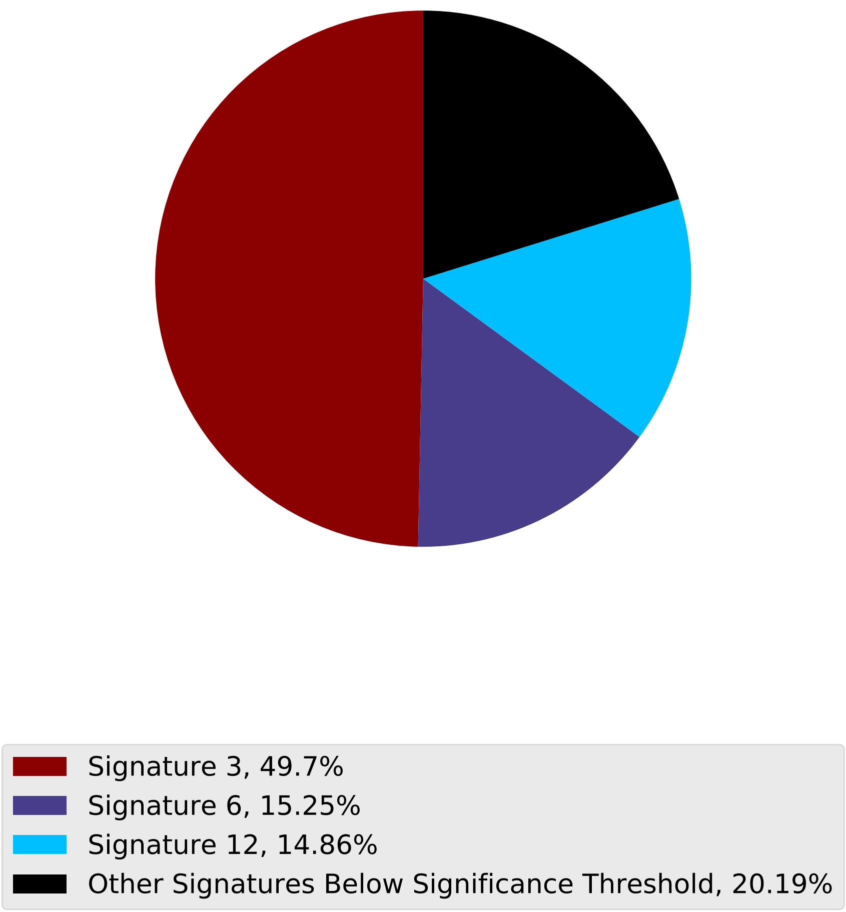

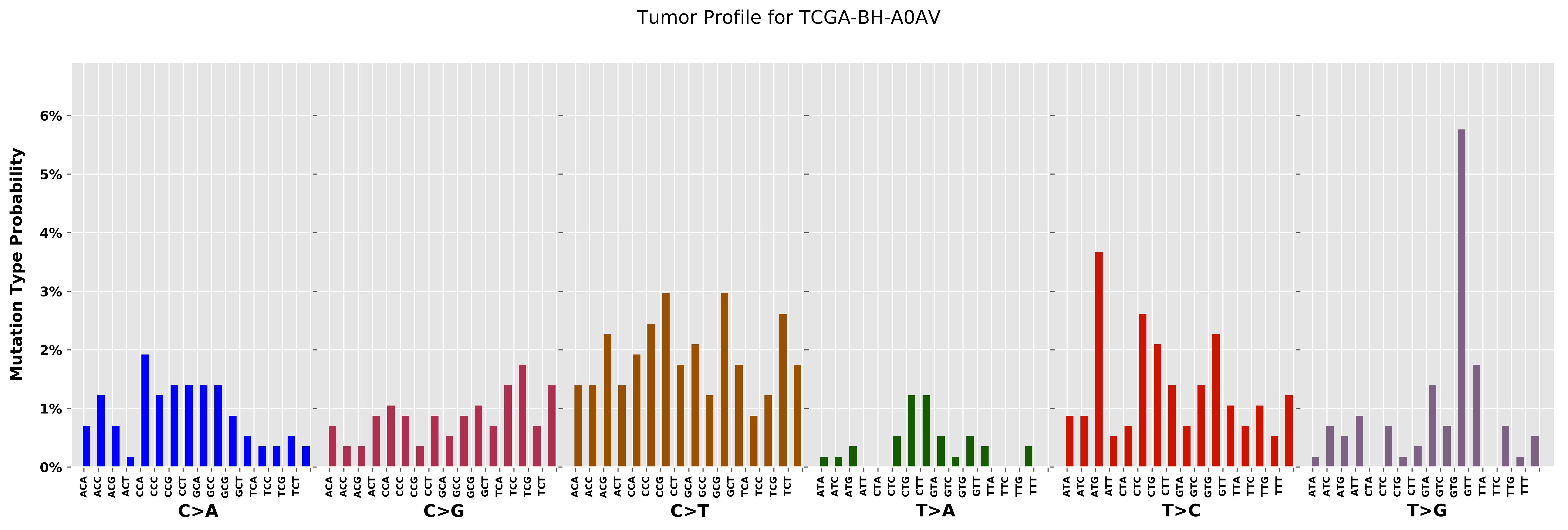
Tumor Profile for TCGA-BH-A0AV
Mutation Type Probability
0%
1%
2%
3%
4%
5%
6%
ACA ACC ACG ACT CCA CCC CCG CCT GCA GCC GCG GCT TCA TCC TCG TCT
C>A
C>G
C>T
ATA ATC ATG ATT CTA CTC CTG CTT GTA GTC GTG GTT TTA TTC TTG TTT
T>A
T>C
T>G

Cancer processes Weights for TCGA-A2-A0CM

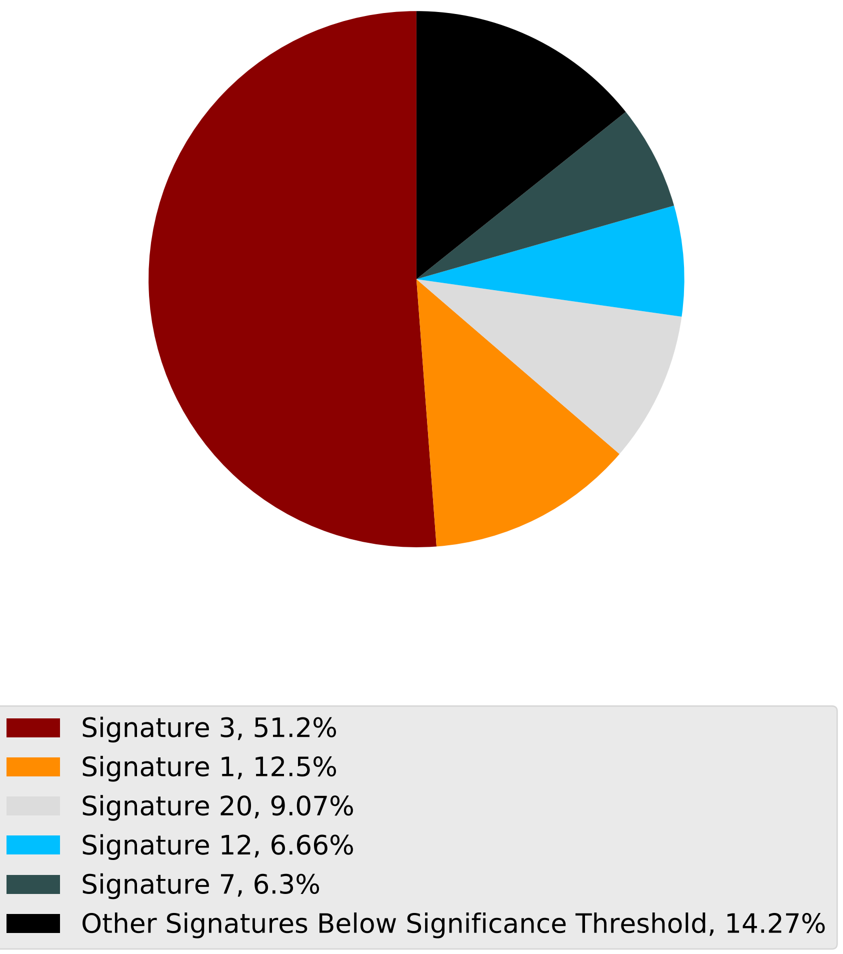

Tumor Profile for TCGA-A2-A0CM

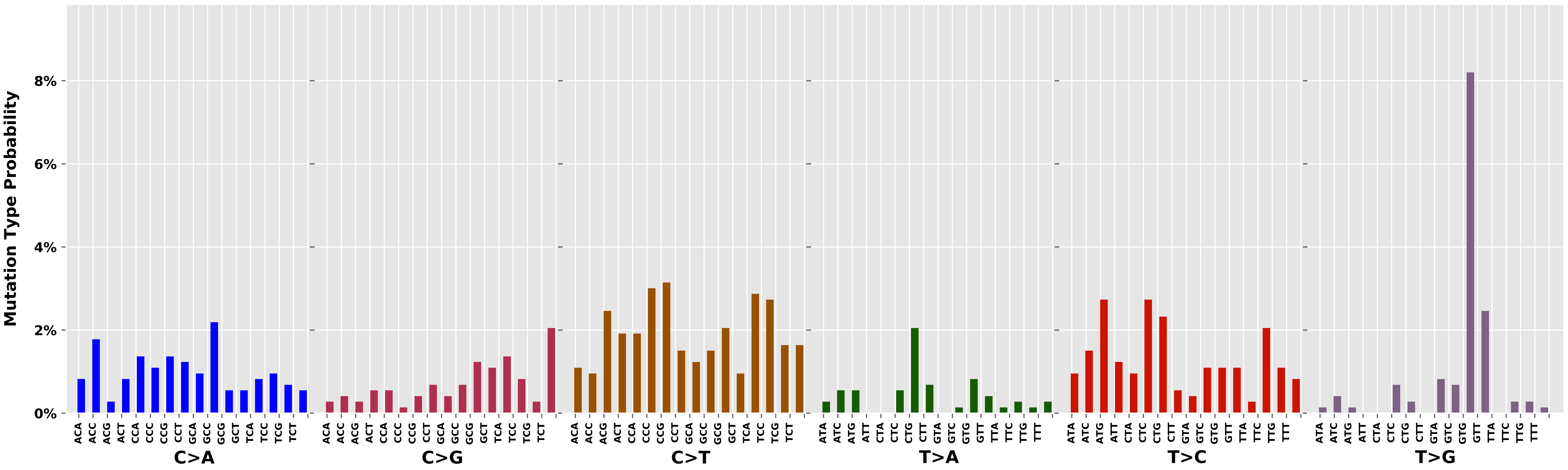

Cancer processes Weights for TCGA-AN-A04D

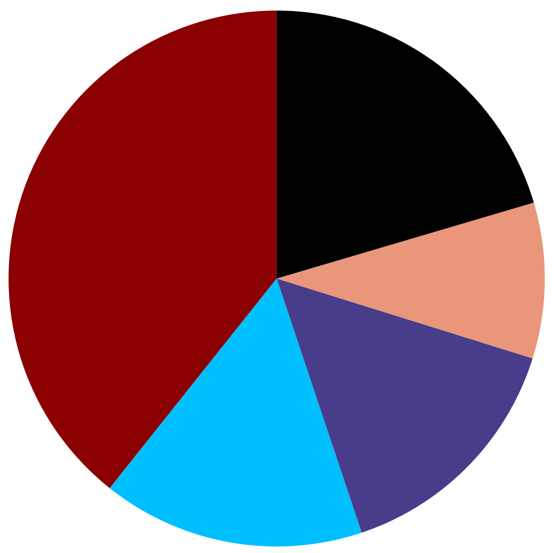

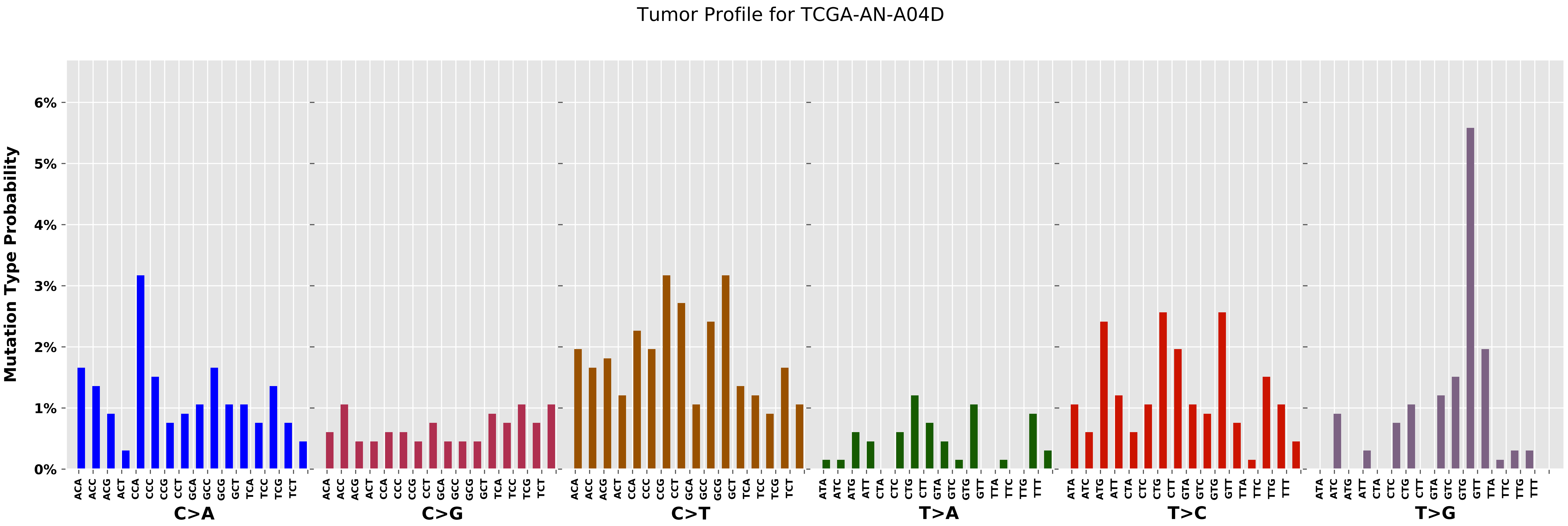

Tumor Profile for TCGA-AN-A04D
Mutation Type Probability
0%
1%
2%
3%
4%
5%
6%
C>A
C>G
C>T
T>A
T>C
T>G

Cancer processes Weights for TCGA-A8-A092

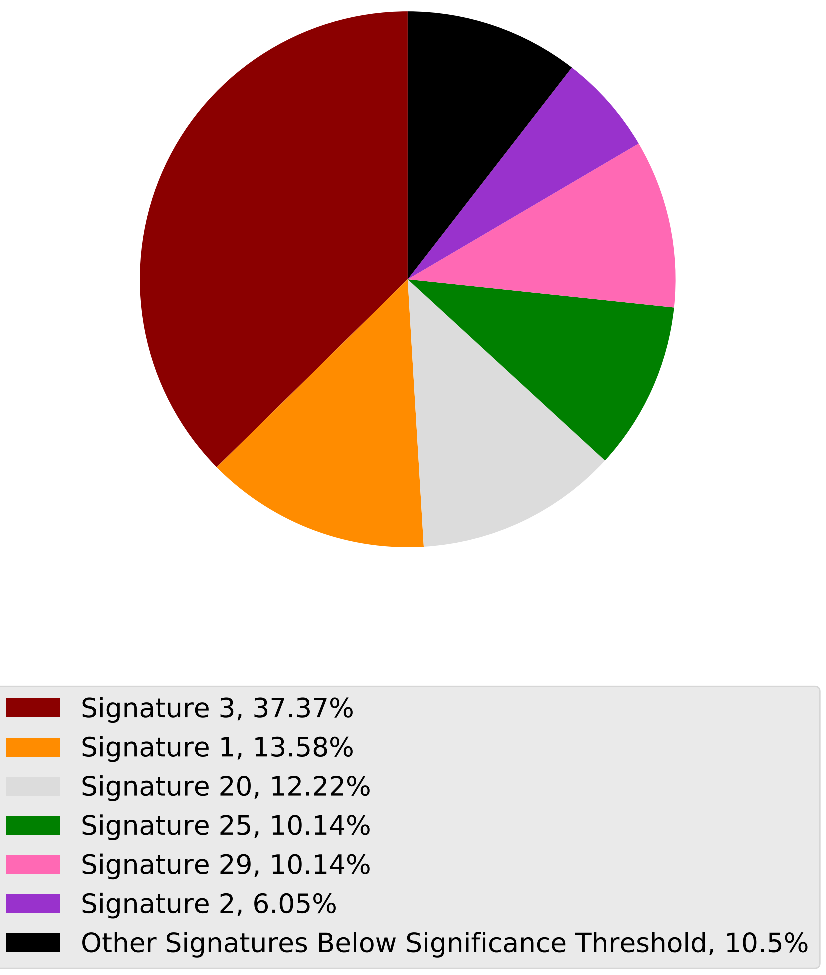

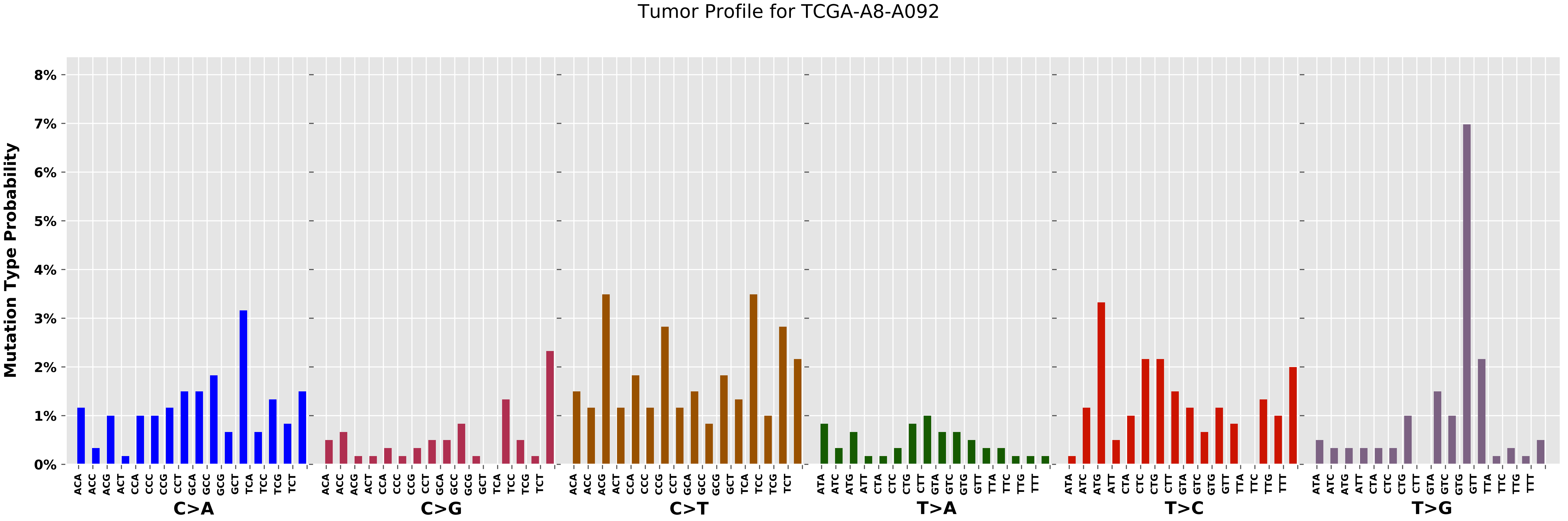
Tumor Profile for TCGA-A8-A092
Mutation Type Probability
0%
1%
2%
3%
4%
5%
6%
7%
8%
C>A
C>G
C>T
T>A
T>C
T>G

Cancer processes Weights for TCGA-A2-A04X

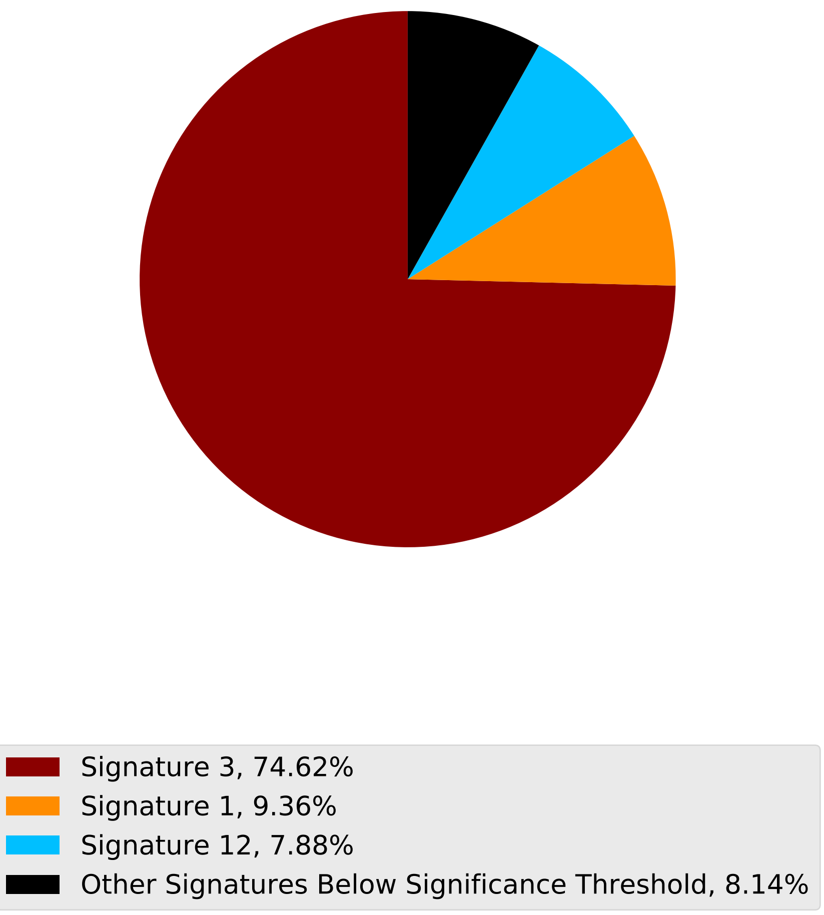

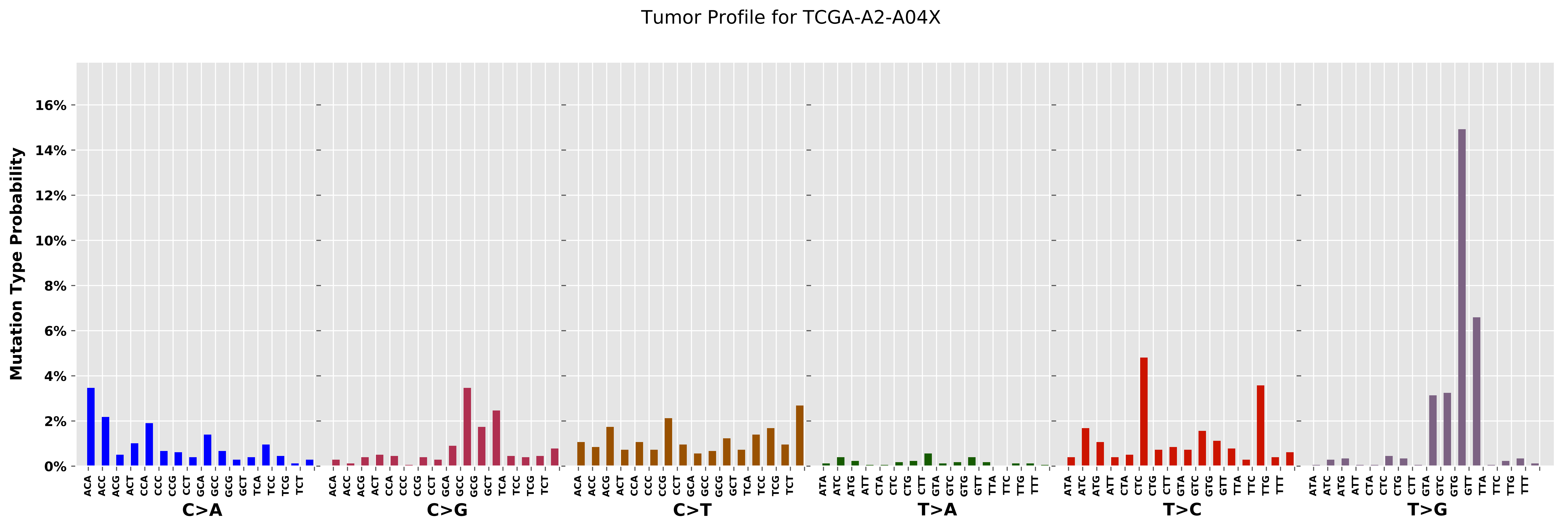
Tumor Profile for TCGA-A2-A04X
Mutation Type Probability
0%
2%
4%
6%
8%
10%
12%
14%
16%
C>A
C>G
C>T
T>A
T>C
T>G
ACA ACC ACG ACT CCA CCC CCG CCT GCA GCC GCG GCT TCA TCC TCG TCT
ATA ATC ATG ATT CTA CTC CTG CTT GTA GTC GTG GTT TTA TTC TTG TTT

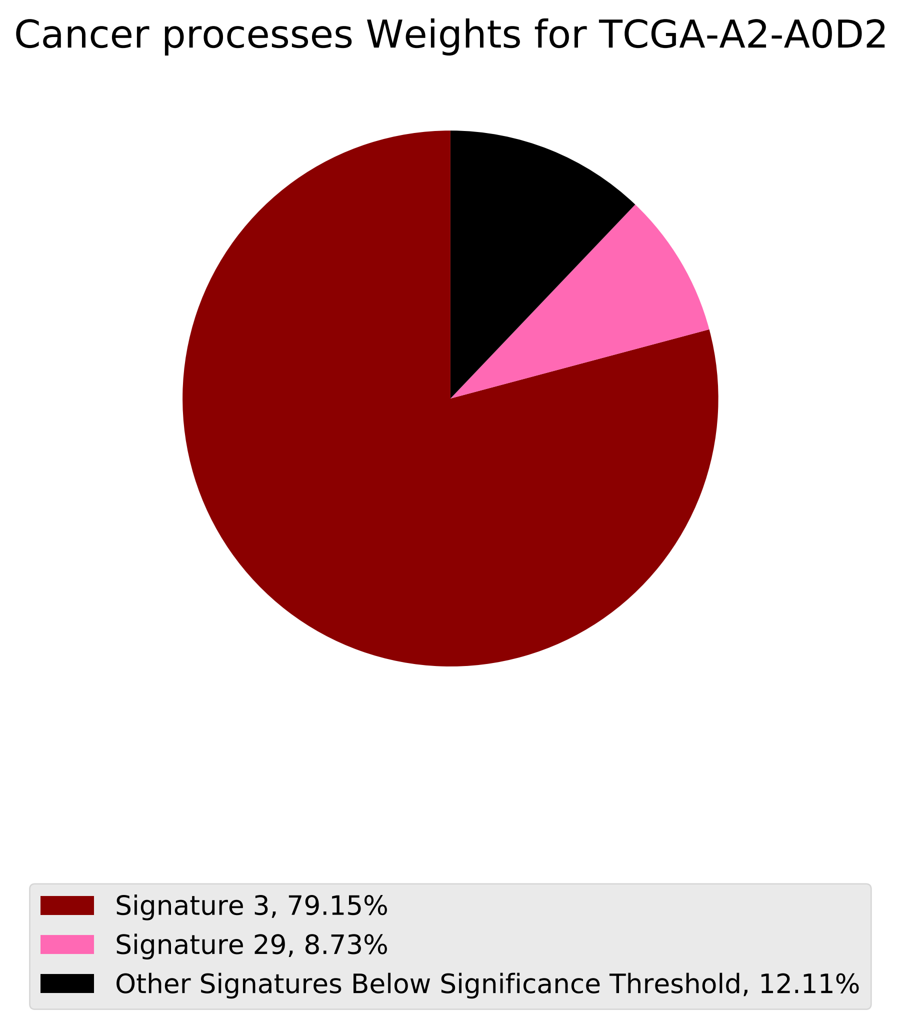
Cancer processes Weights for TCGA-A2-A0D2
Signature 3, 79.15%
Signature 29, 8.73%
Other Signatures Below Significance Threshold, 12.11%

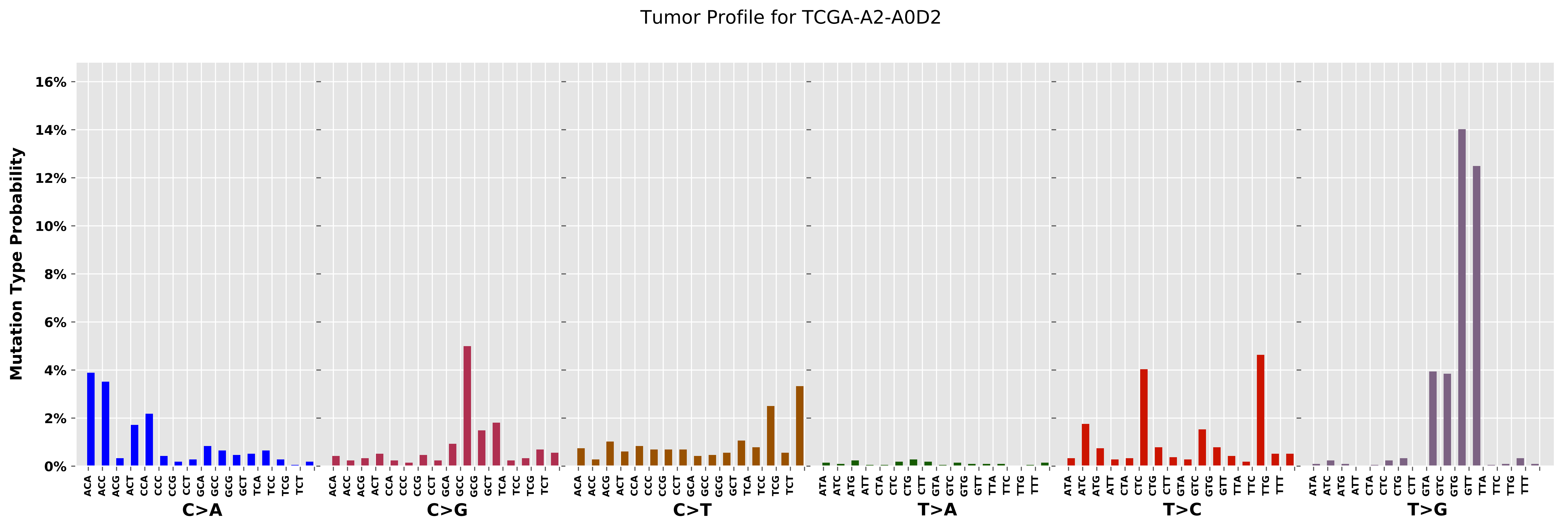

Tumor Profile for TCGA-A2-A0D2
Mutation Type Probability
0%
2%
4%
6%
8%
10%
12%
14%
16%
C>A
C>G
C>T
T>A
T>C
T>G

Cancer processes Weights for TCGA-B6-A0RU

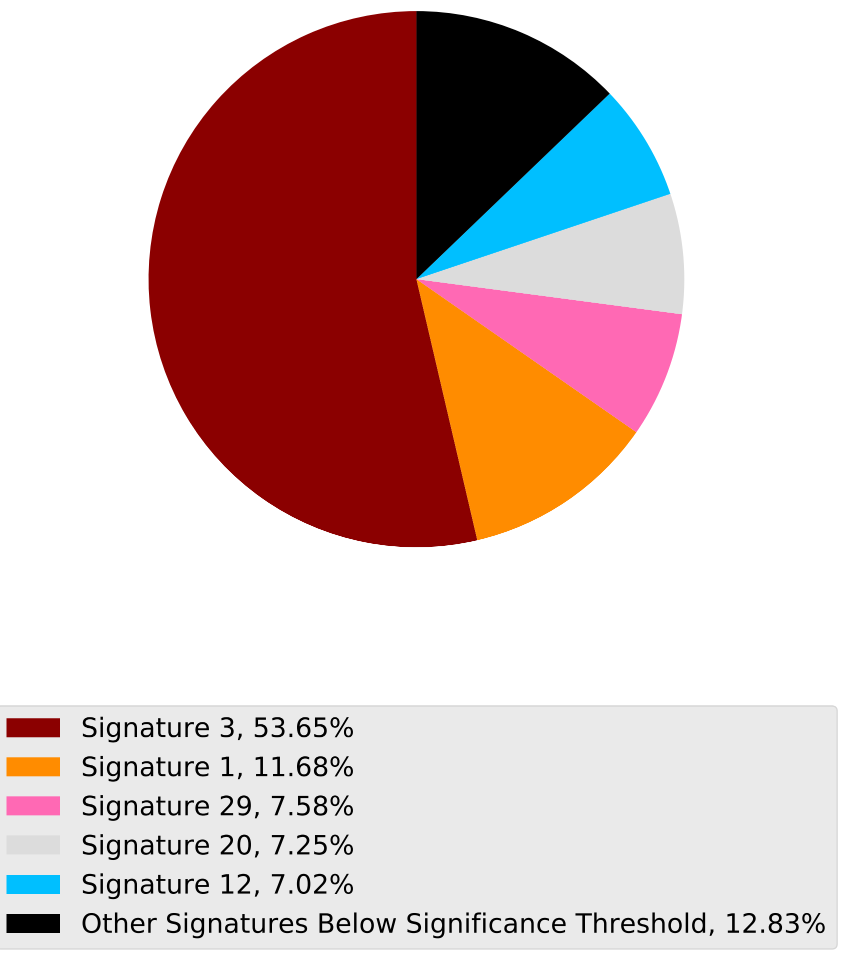

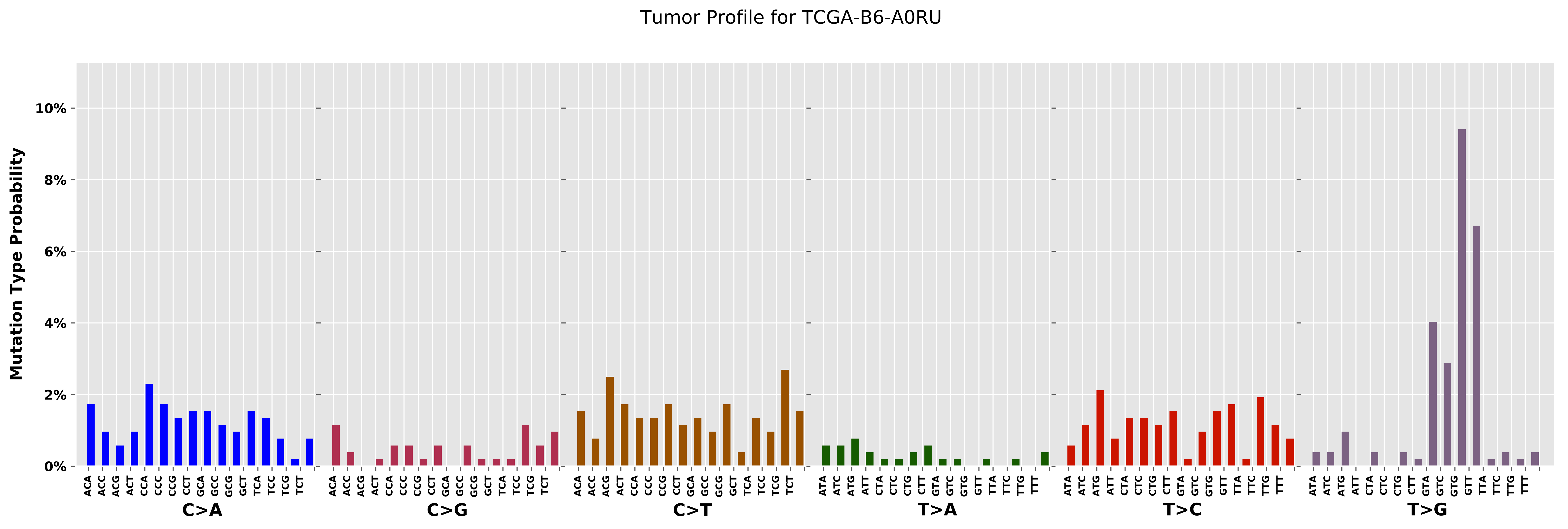

Tumor Profile for TCGA-B6-A0RU
Mutation Type Probability
0%
2%
4%
6%
8%
10%
C>A
C>G
C>T
T>A
T>C
T>G
