# Supplementary material for: pyCancerSig: subclassifying human cancer with comprehensive single nucleotide, structural and microsatellite mutational signature deconstruction from whole genome sequencing
Source: BMC Bioinformatics. 2020 Apr 3;21:128. doi: 10.1186/s12859-020-3451-8 (PMC7118897; doi:10.1186/s12859-020-3451-8)
Supplement: Supplementary file 4 — Additional file 4. [file 12859_2020_3451_MOESM4_ESM.pdf]

Cancer processes Weights for TCGA-E2-A14X

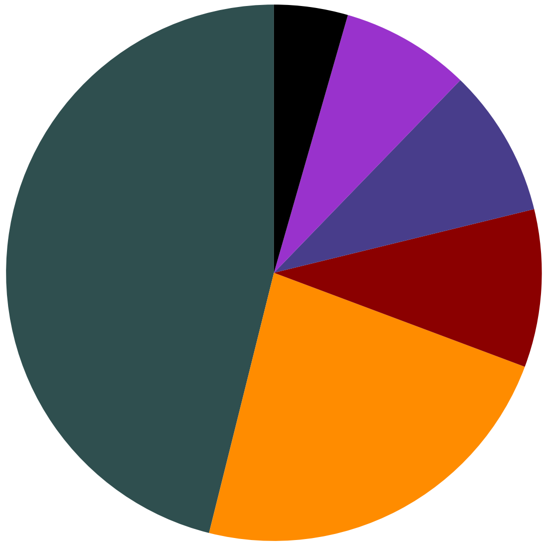

Signature H, 46.1%
Signature A, 23.2%
Signature C, 9.51%
Signature F, 8.94%
Signature B, 7.8%
Other Signatures Below Significance Threshold, 4.45%

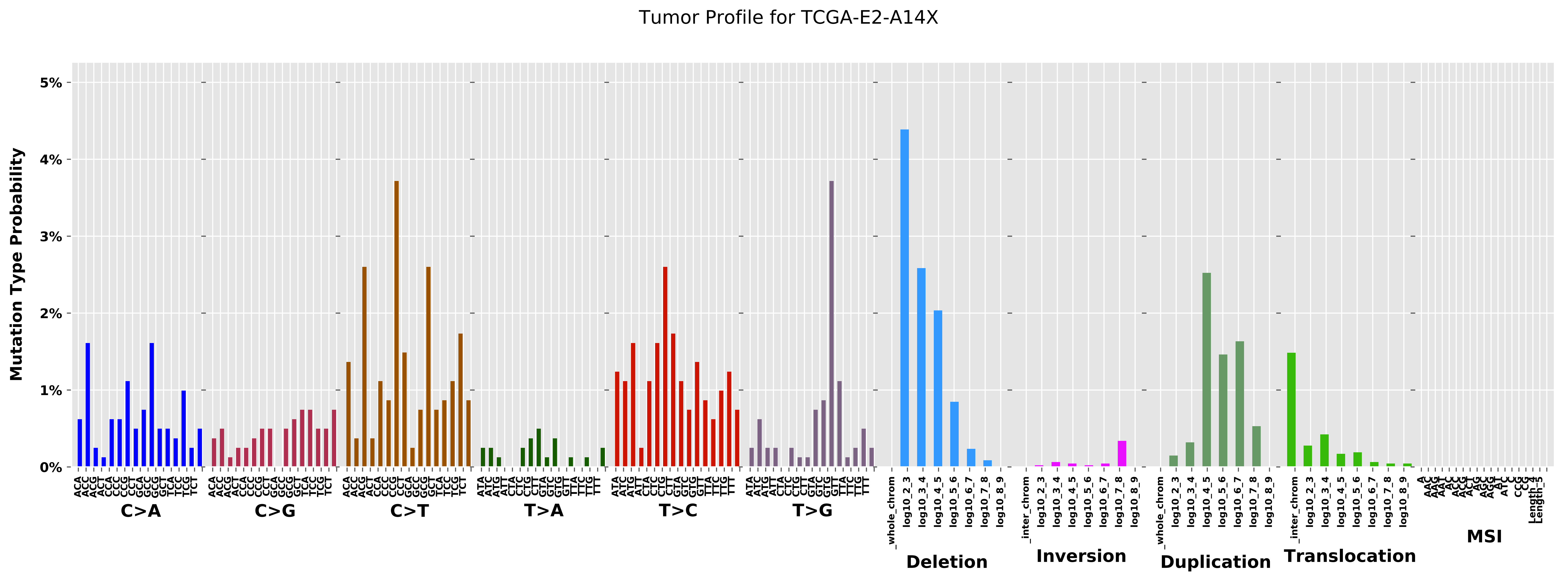
Tumor Profile for TCGA-E2-A14X
Mutation Type Probability
0%
1%
2%
3%
4%
5%
C>A
C>G
C>T
T>A
T>C
T>G
Deletion
Inversion
Duplication
Translocation
MSI

Cancer processes Weights for TCGA-A8-A08L

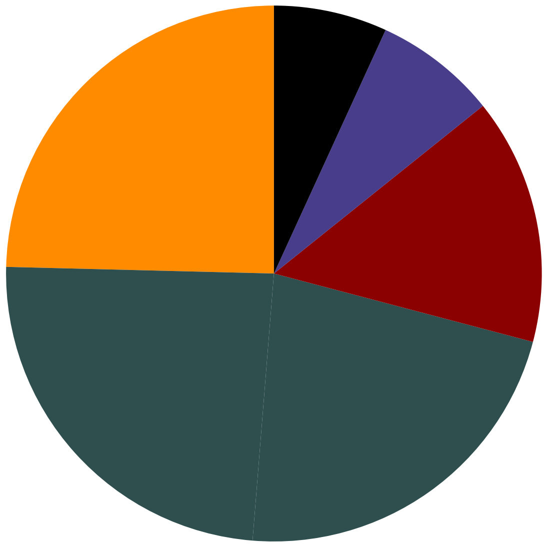

- Signature A, 24.61%
- Signature H, 24.12%
- Signature G, 22.16%
- Signature C, 14.87%
- Signature F, 7.4%
- Other Signatures Below Significance Threshold, 6.84%

Tumor Profile for TCGA-A8-A08L

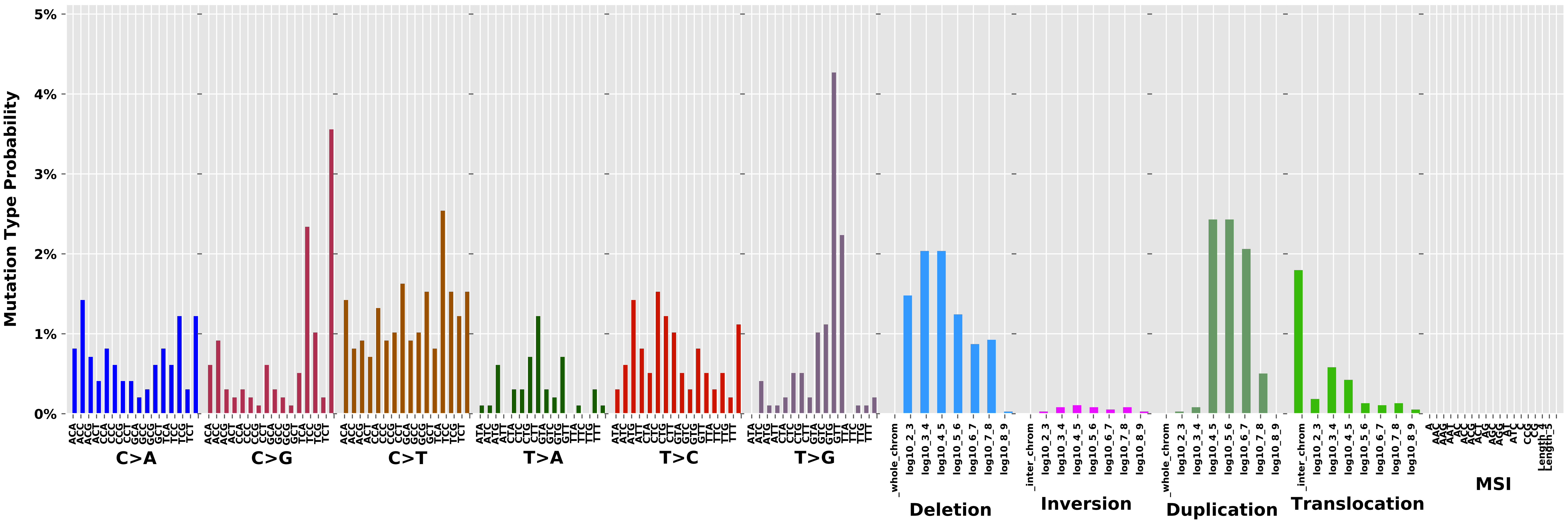

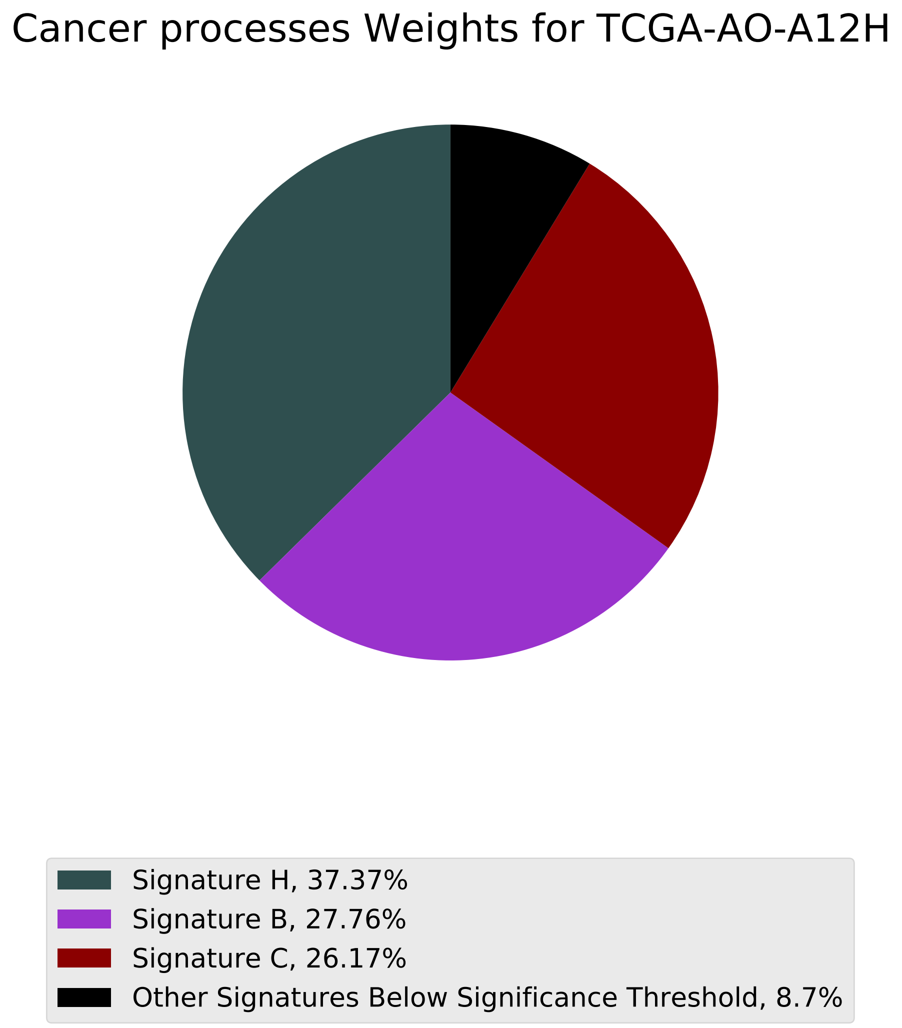
Cancer processes Weights for TCGA-AO-A12H
Signature H, 37.37%
Signature B, 27.76%
Signature C, 26.17%
Other Signatures Below Significance Threshold, 8.7%

Tumor Profile for TCGA-AO-A12H

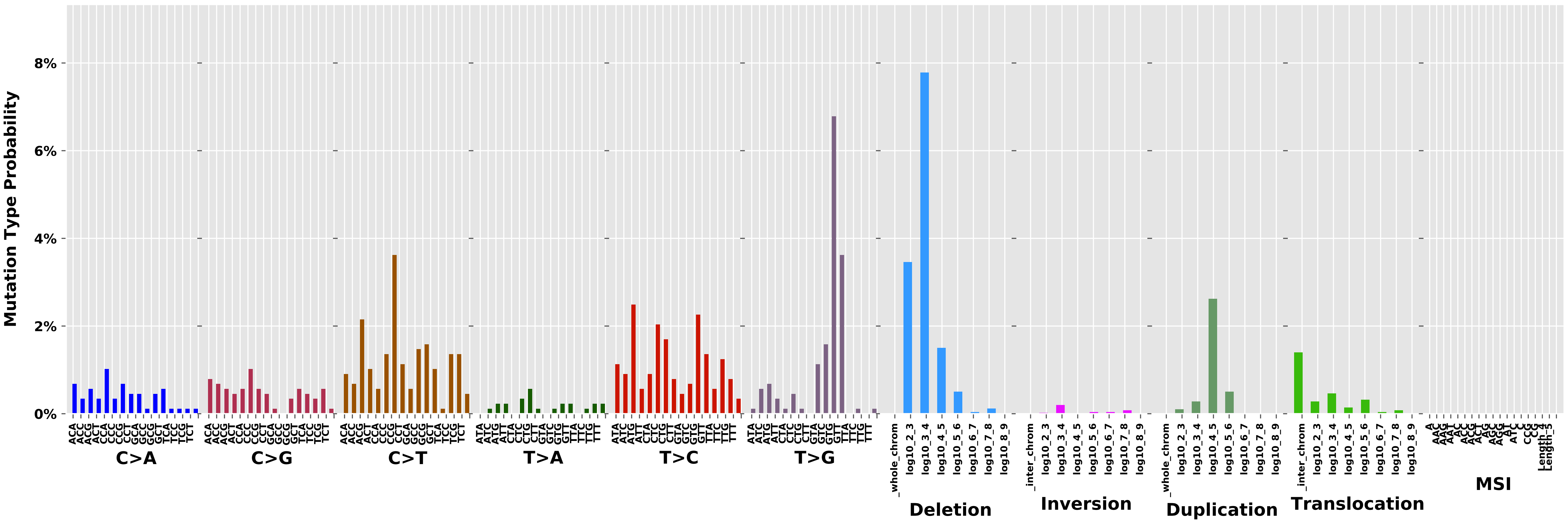

Cancer processes Weights for TCGA-EW-A3U0

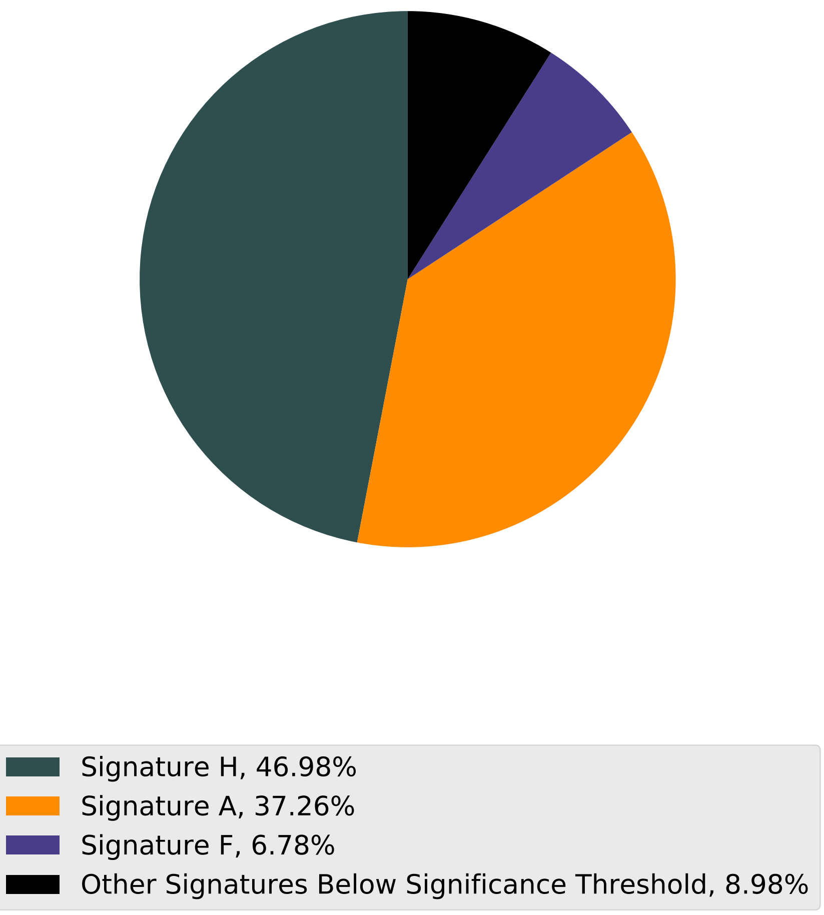

Tumor Profile for TCGA-EW-A3U0

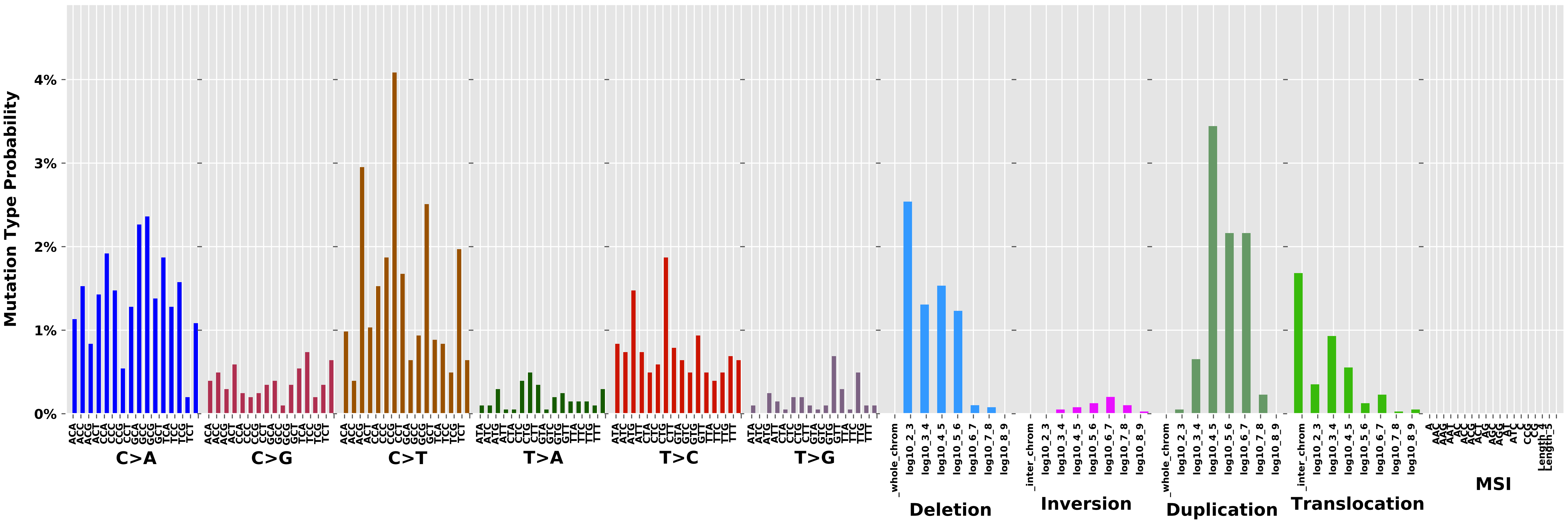

Cancer processes Weights for TCGA-BH-A1FC

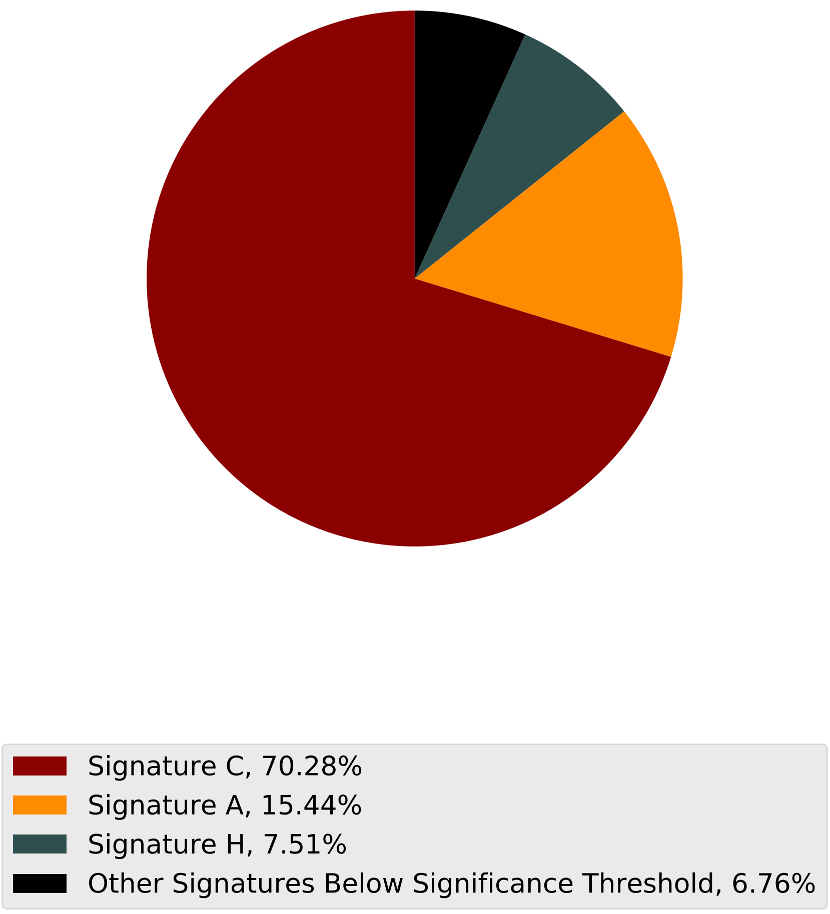

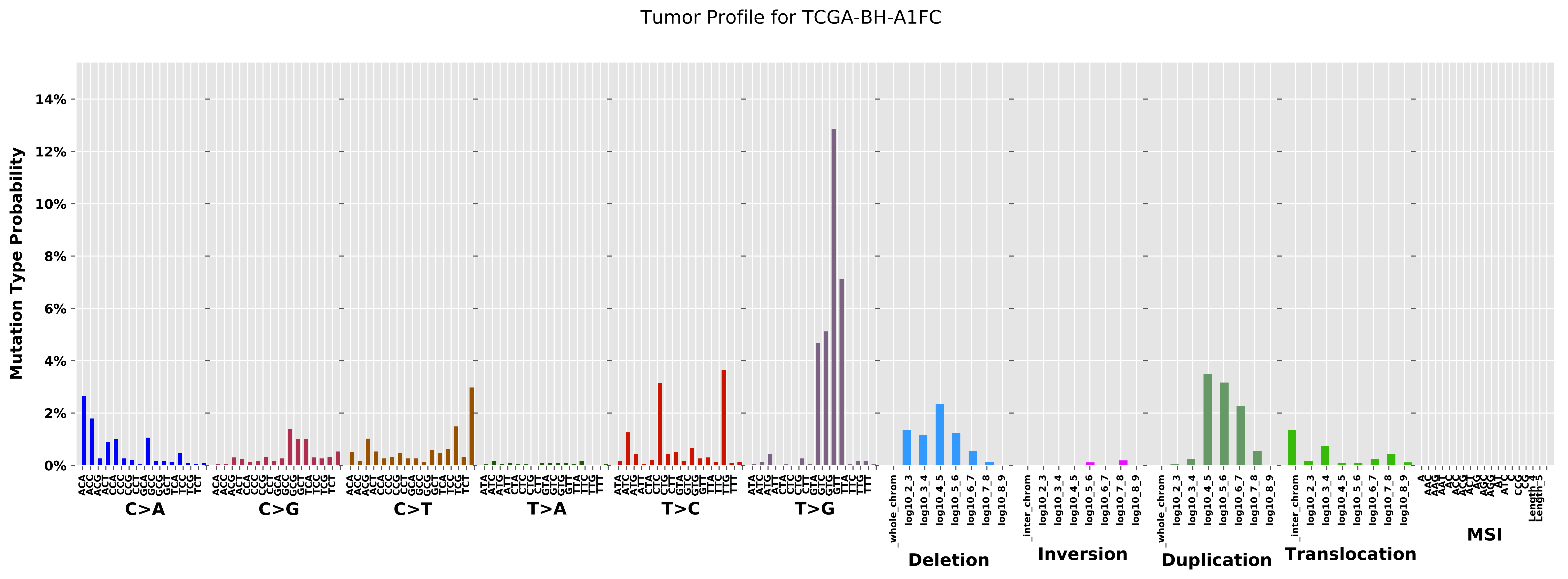

Tumor Profile for TCGA-BH-A1FC
Mutation Type Probability
14%
12%
10%
8%
6%
4%
2%
0%
C>A
C>G
C>T
T>A
T>C
T>G
Deletion
Inversion
Duplication
Translocation
MSI

Cancer processes Weights for TCGA-AF-2691

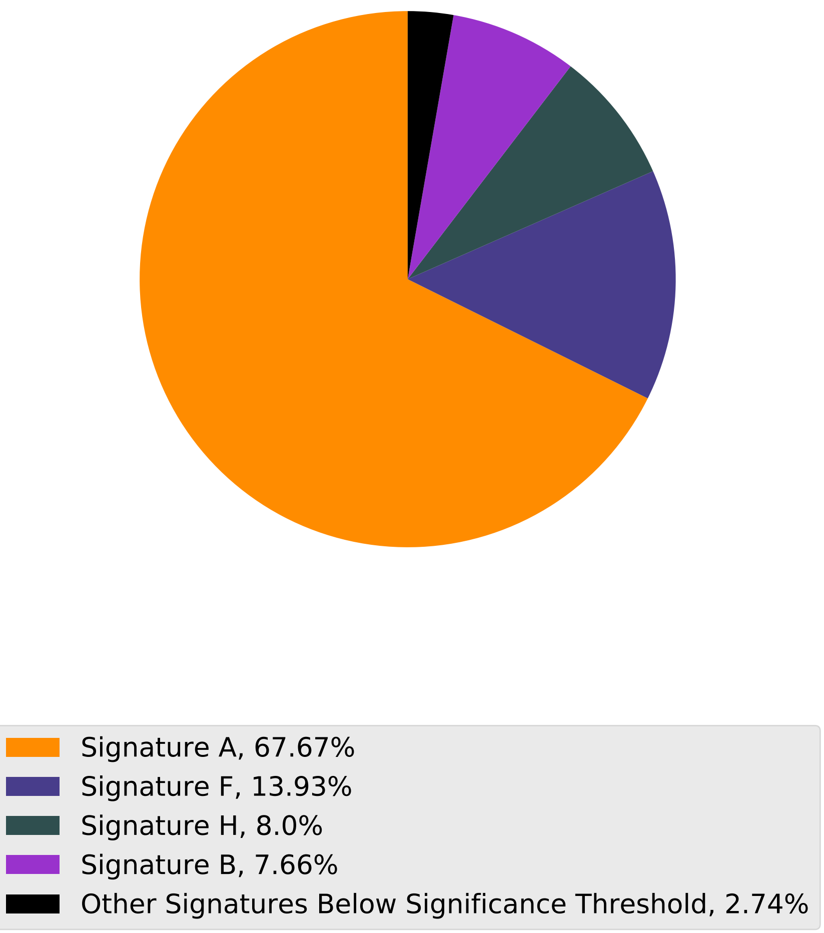

Tumor Profile for TCGA-AF-2691

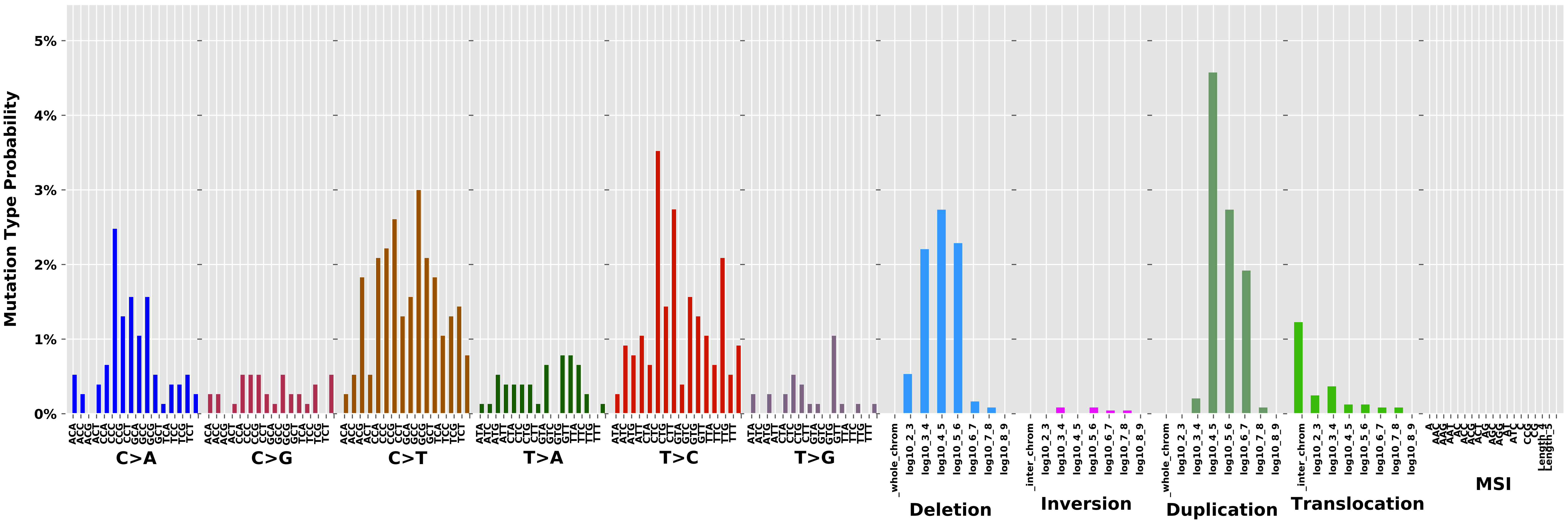

Cancer processes Weights for TCGA-AF-3913

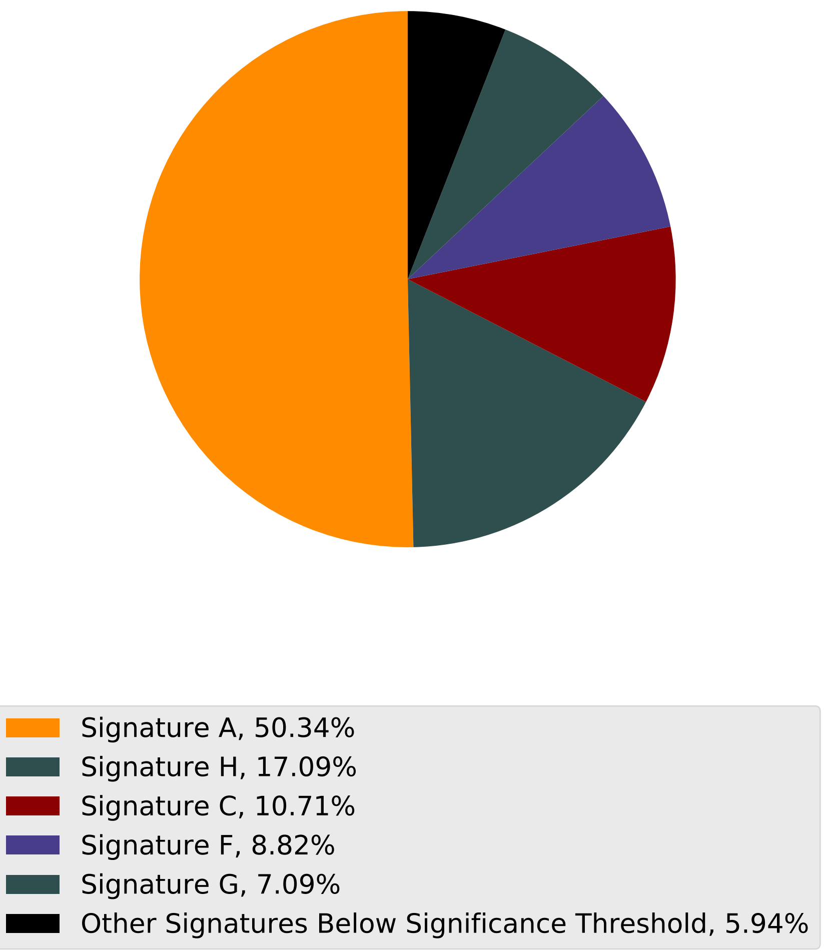

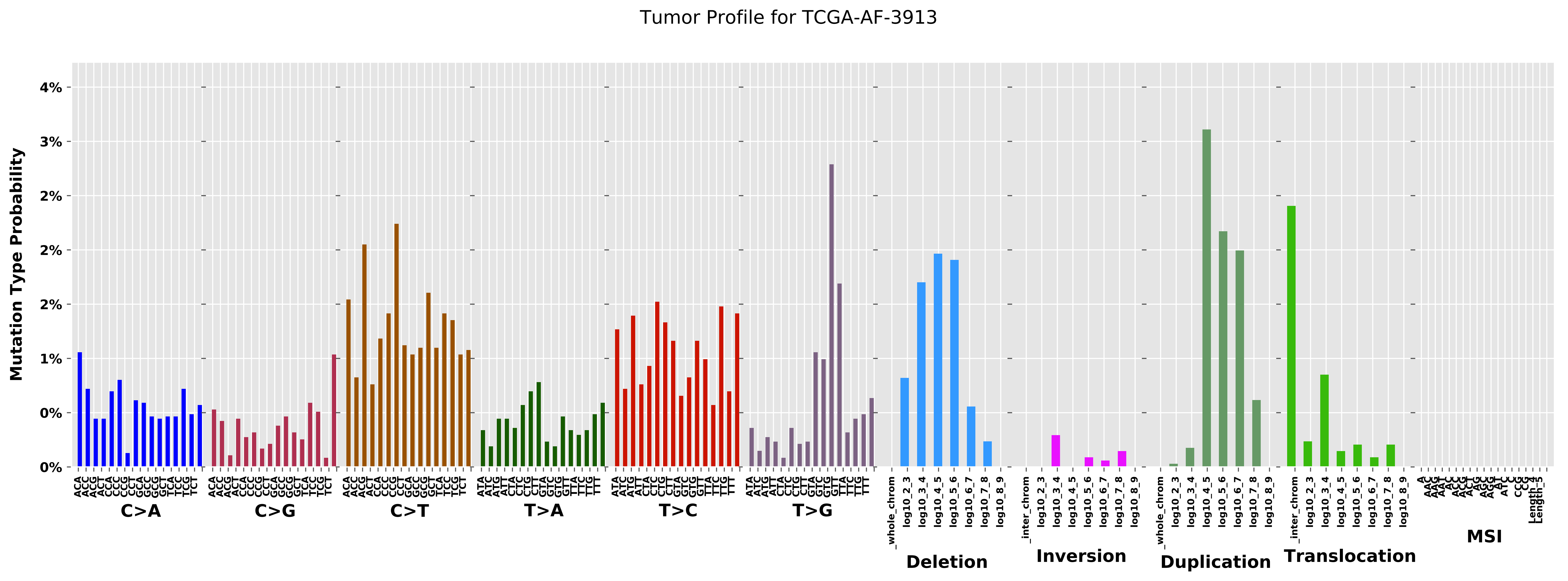
Tumor Profile for TCGA-AF-3913
Mutation Type Probability
4%
3%
2%
2%
2%
1%
0%
0%
C>A
C>G
C>T
T>A
T>C
T>G
Deletion
Inversion
Duplication
Translocation
MSI

Cancer processes Weights for TCGA-AA-3977

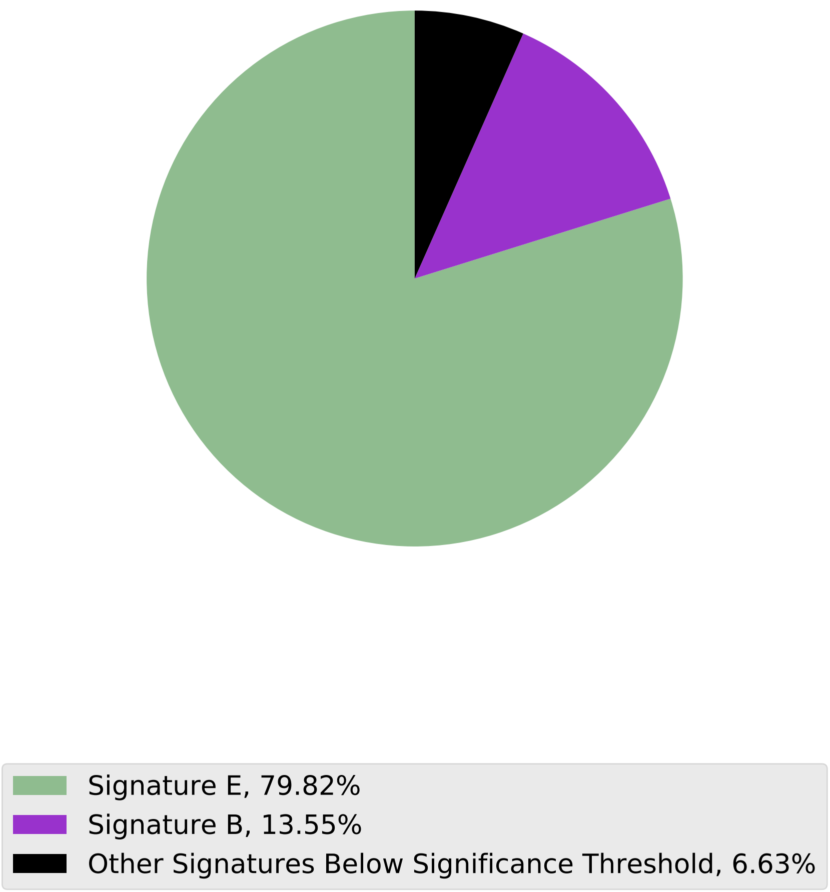

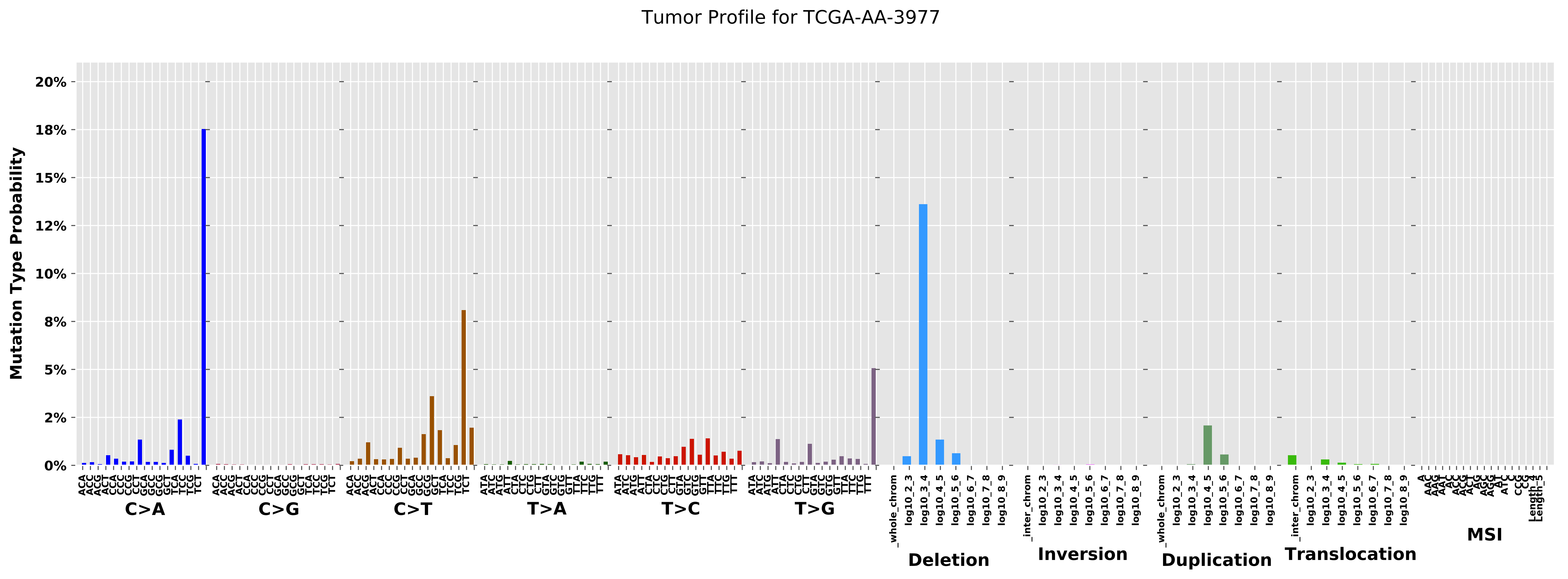

Tumor Profile for TCGA-AA-3977
Mutation Type Probability
20%
18%
15%
12%
10%
8%
5%
2%
0%
C>A
C>G
C>T
T>A
T>C
T>G
Deletion
Inversion
Duplication
Translocation
MSI

Cancer processes Weights for TCGA-A7-A0CE

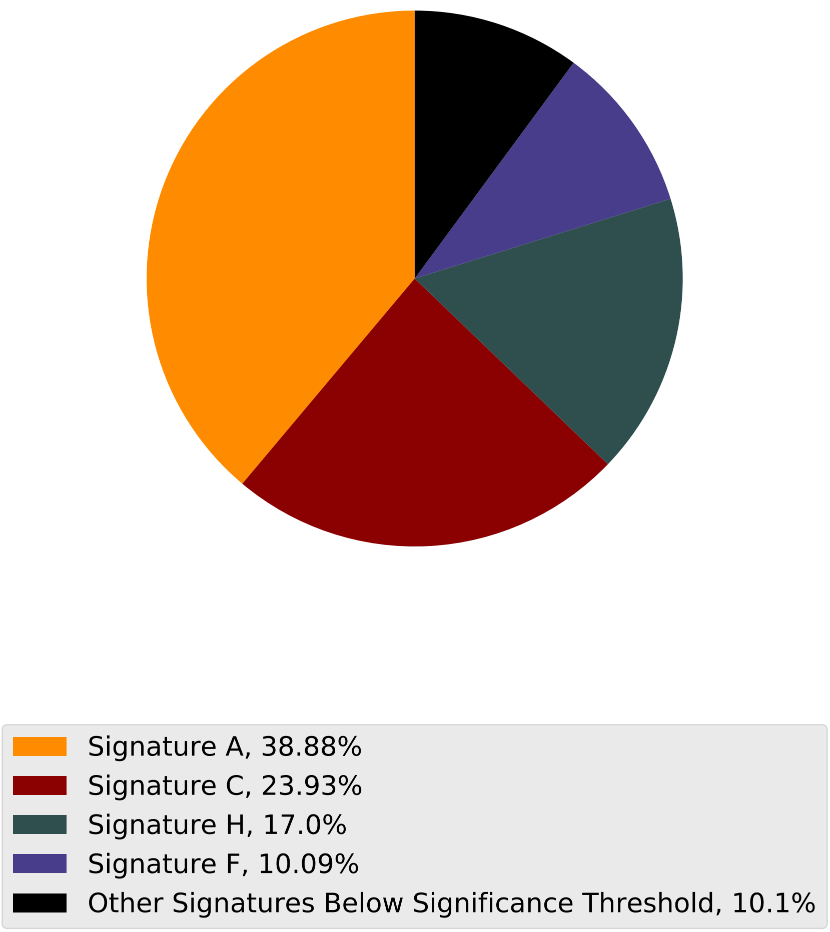

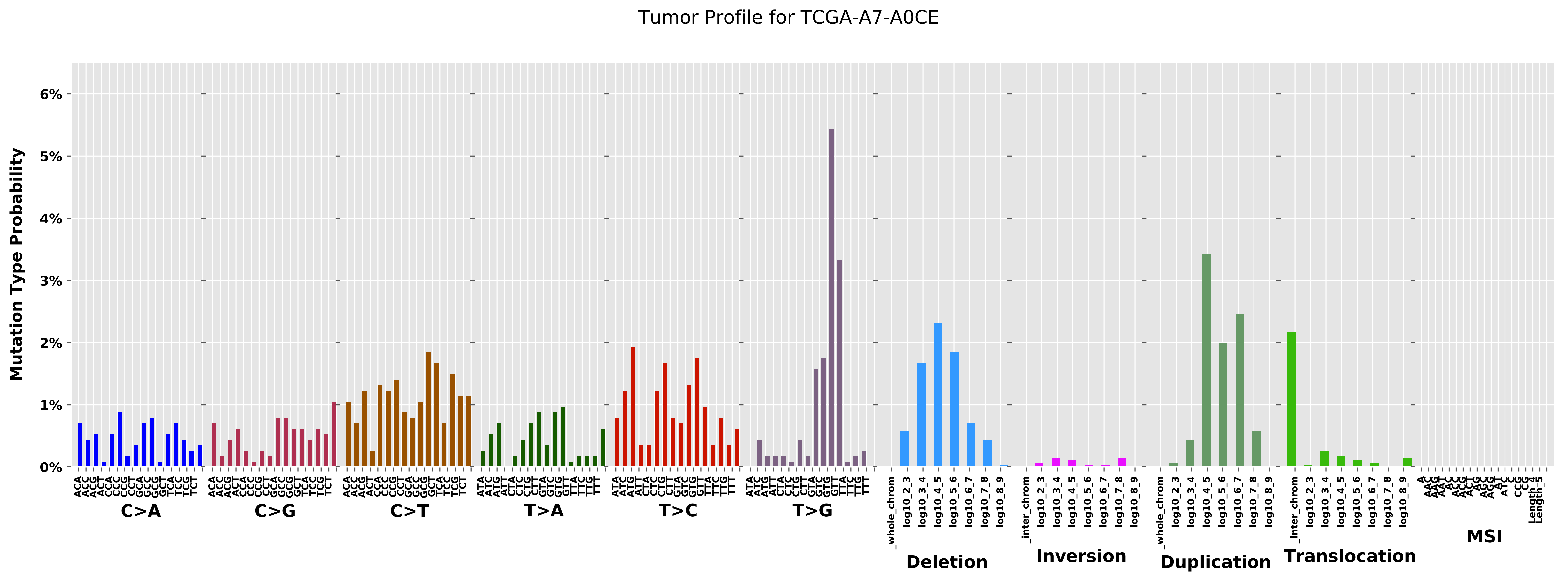
Tumor Profile for TCGA-A7-A0CE
Mutation Type Probability
0%
1%
2%
3%
4%
5%
6%
C>A
C>G
C>T
T>A
T>C
T>G
Deletion
Inversion
Duplication
Translocation
MSI

Cancer processes Weights for TCGA-AO-A0J4

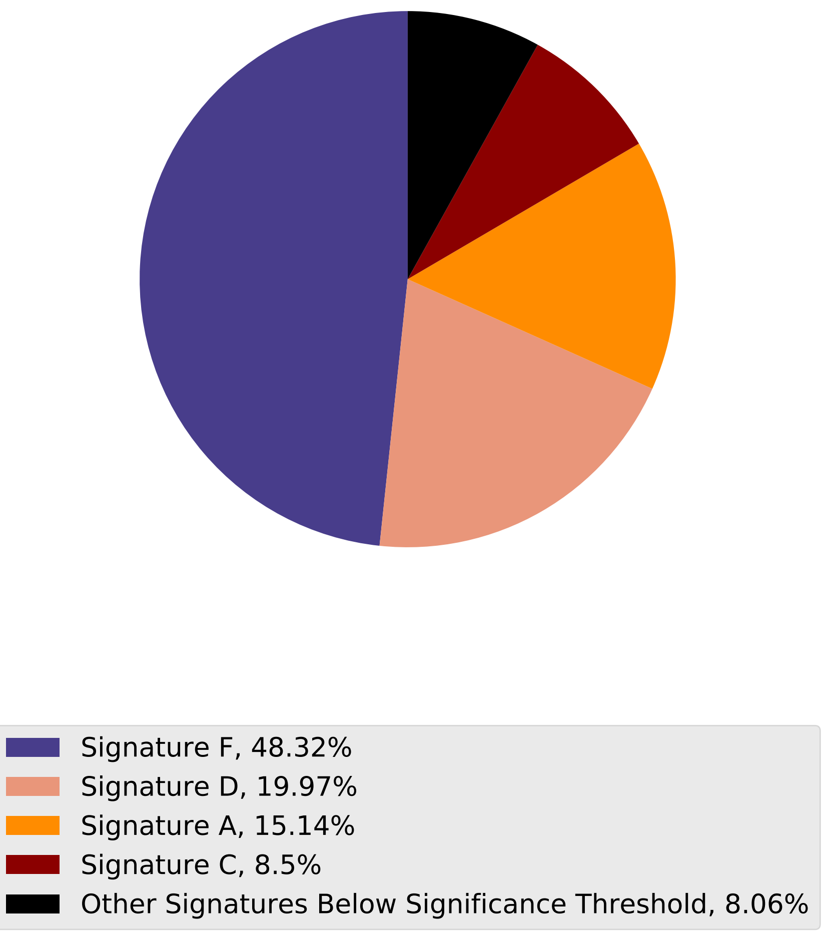

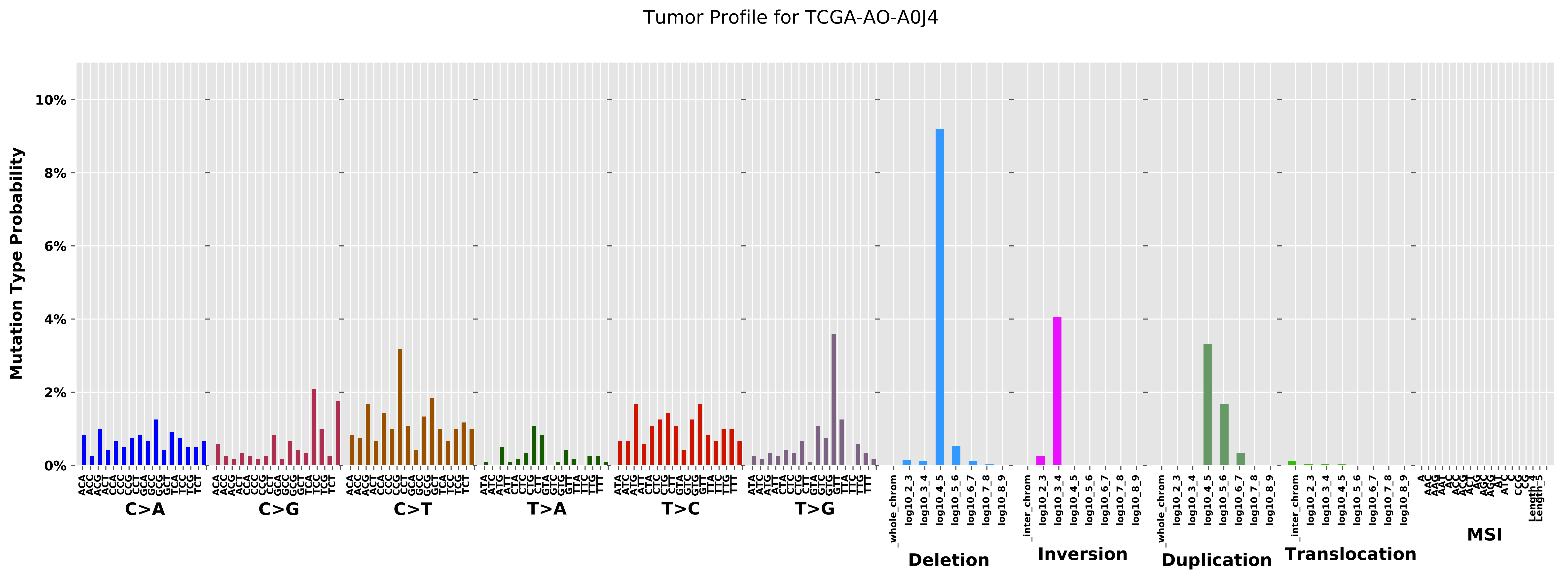

Tumor Profile for TCGA-AO-A0J4
Mutation Type Probability
10%
8%
6%
4%
2%
0%
C>A
C>G
C>T
T>A
T>C
T>G
Deletion
Inversion
Duplication
Translocation
MSI

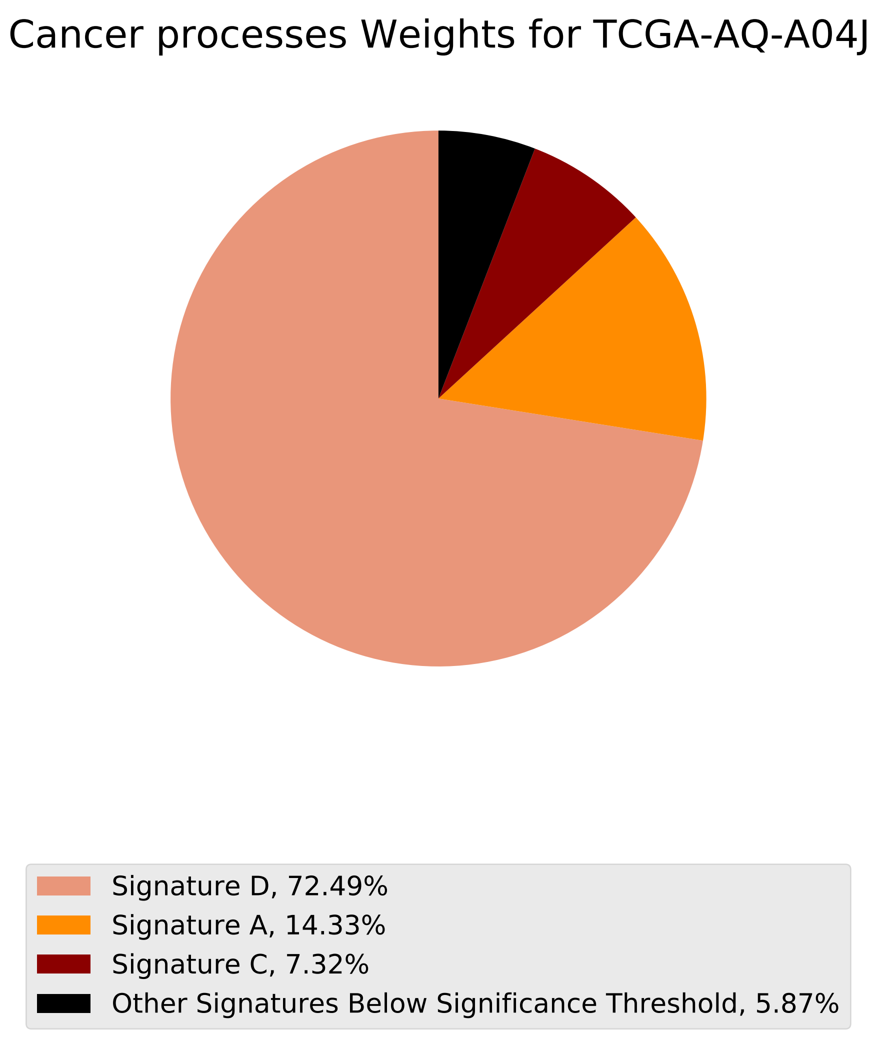
Cancer processes Weights for TCGA-AQ-A04J
Signature D, 72.49%
Signature A, 14.33%
Signature C, 7.32%
Other Signatures Below Significance Threshold, 5.87%

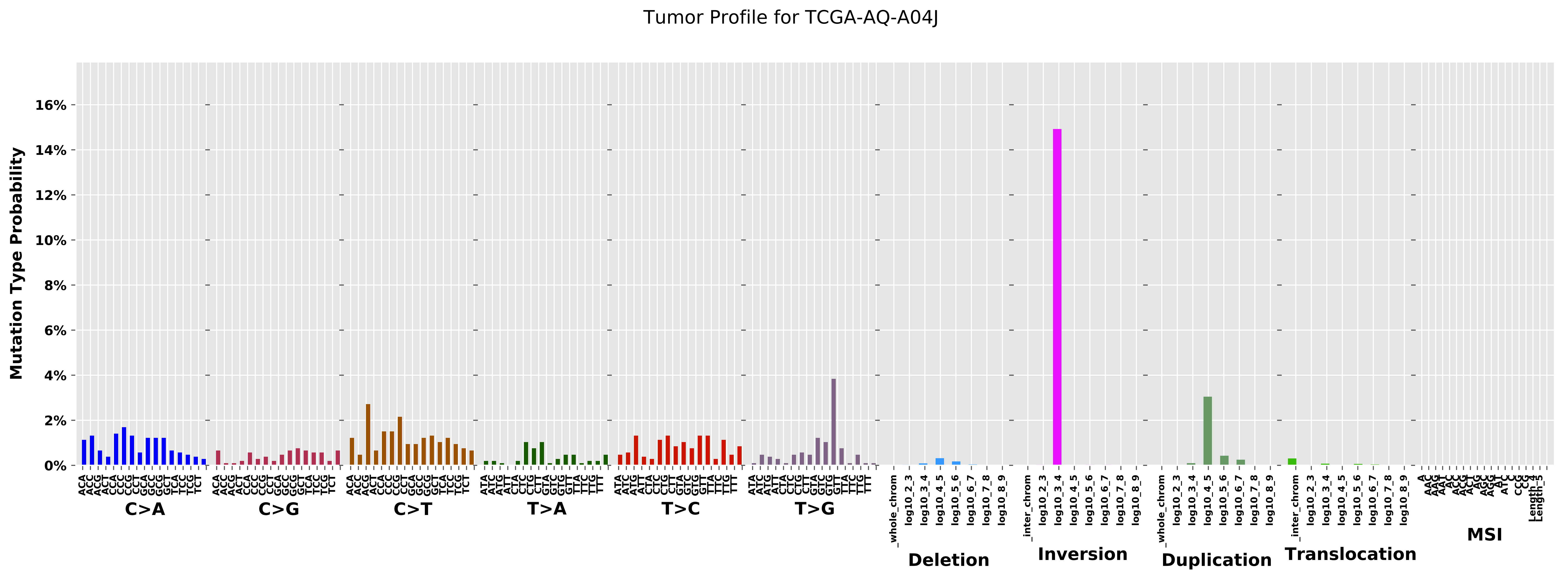

Tumor Profile for TCGA-AQ-A04J
Mutation Type Probability
0%
2%
4%
6%
8%
10%
12%
14%
16%
C>A
C>G
C>T
T>A
T>C
T>G
Deletion
Inversion
Duplication
Translocation
MSI

Cancer processes Weights for TCGA-AG-4007

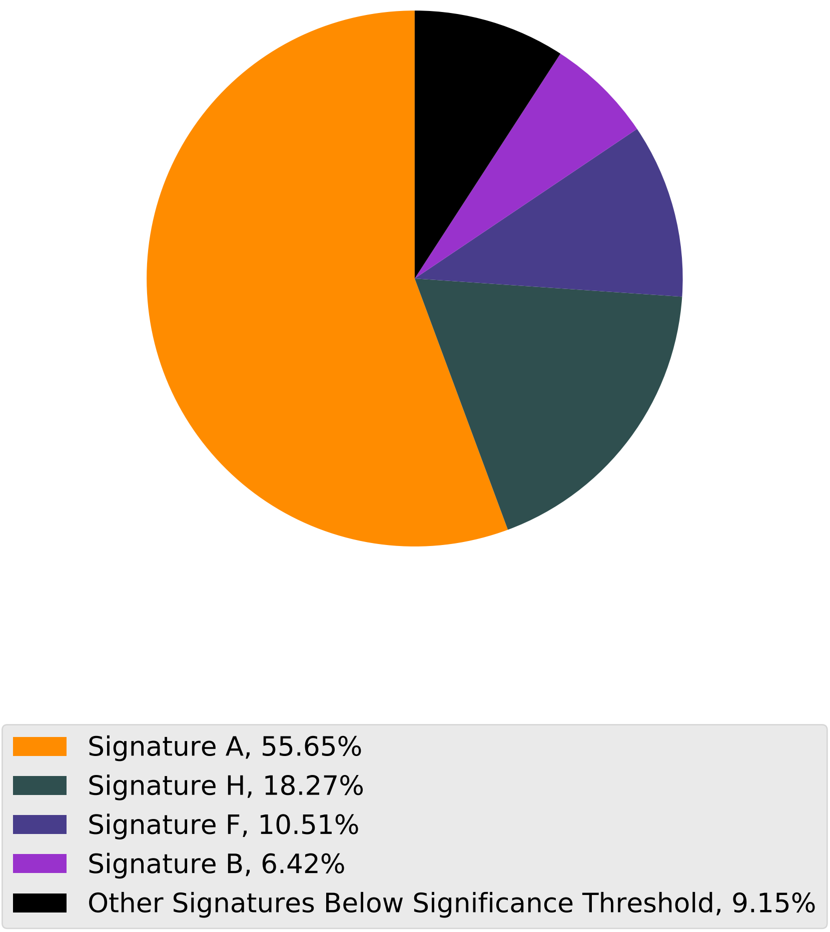

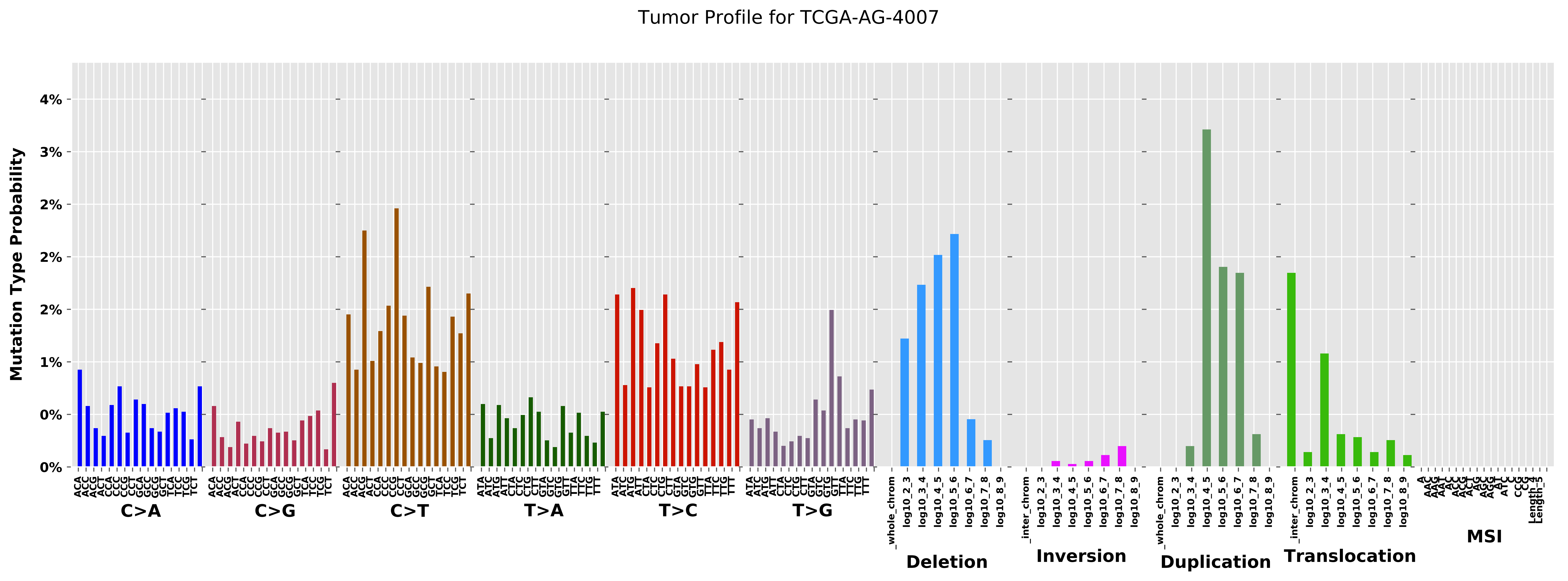
Tumor Profile for TCGA-AG-4007
Mutation Type Probability
4%
3%
2%
2%
2%
1%
0%
0%
C>A
C>G
C>T
T>A
T>C
T>G
Deletion
Inversion
Duplication
Translocation
MSI
_whole_chrom
_inter_chrom
log10_2_3
log10_3_4
log10_4_5
log10_5_6
log10_6_7
log10_7_8
log10_8_9

Cancer processes Weights for TCGA-CA-6717

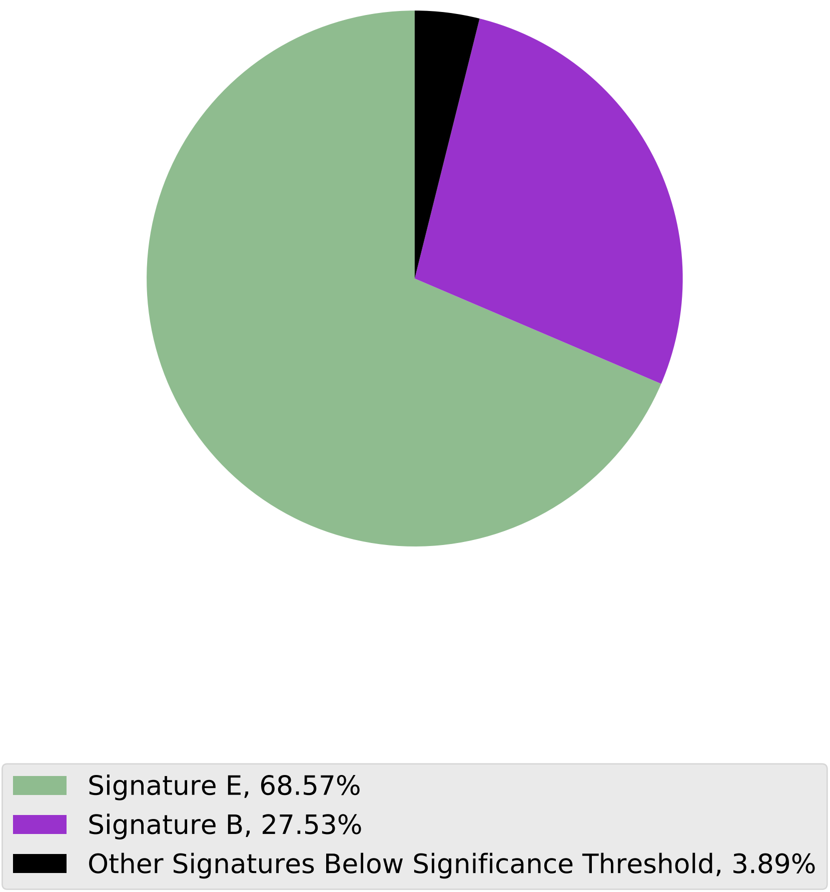

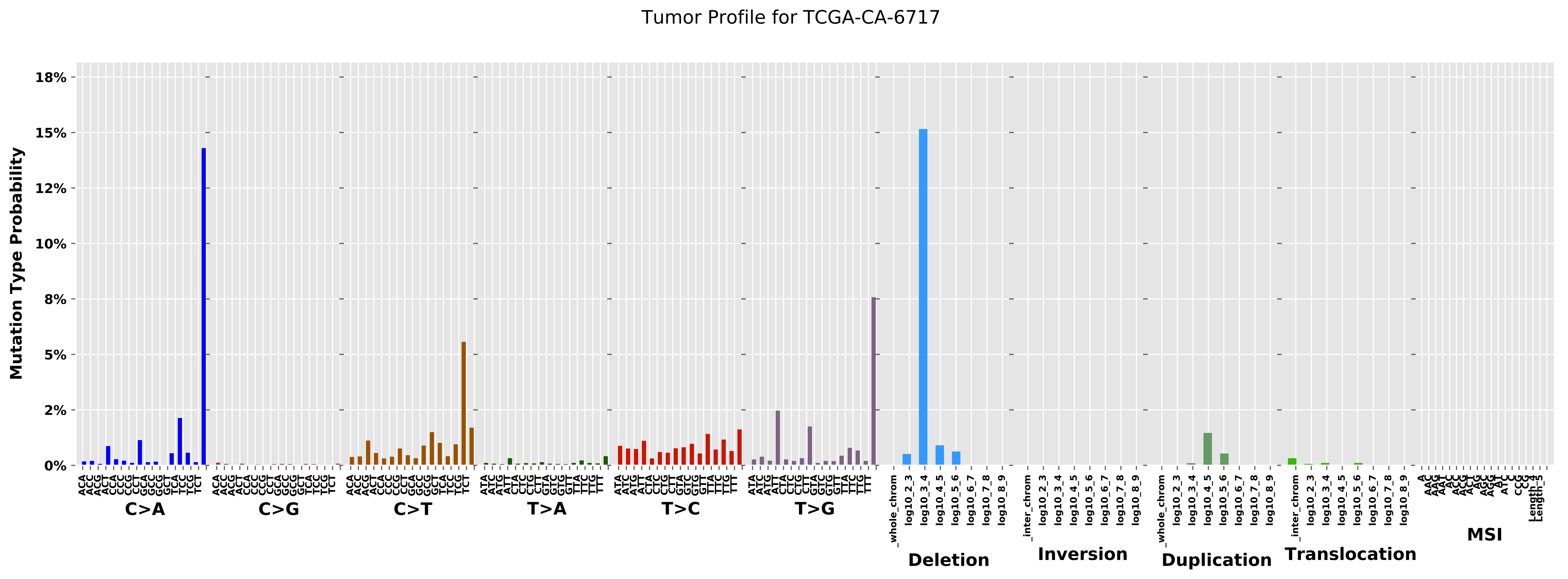

Tumor Profile for TCGA-CA-6717
Mutation Type Probability
18%
15%
12%
10%
8%
5%
2%
0%
C>A
C>G
C>T
T>A
T>C
T>G
Deletion
Inversion
Duplication
Translocation
MSI

Cancer processes Weights for TCGA-E2-A14P

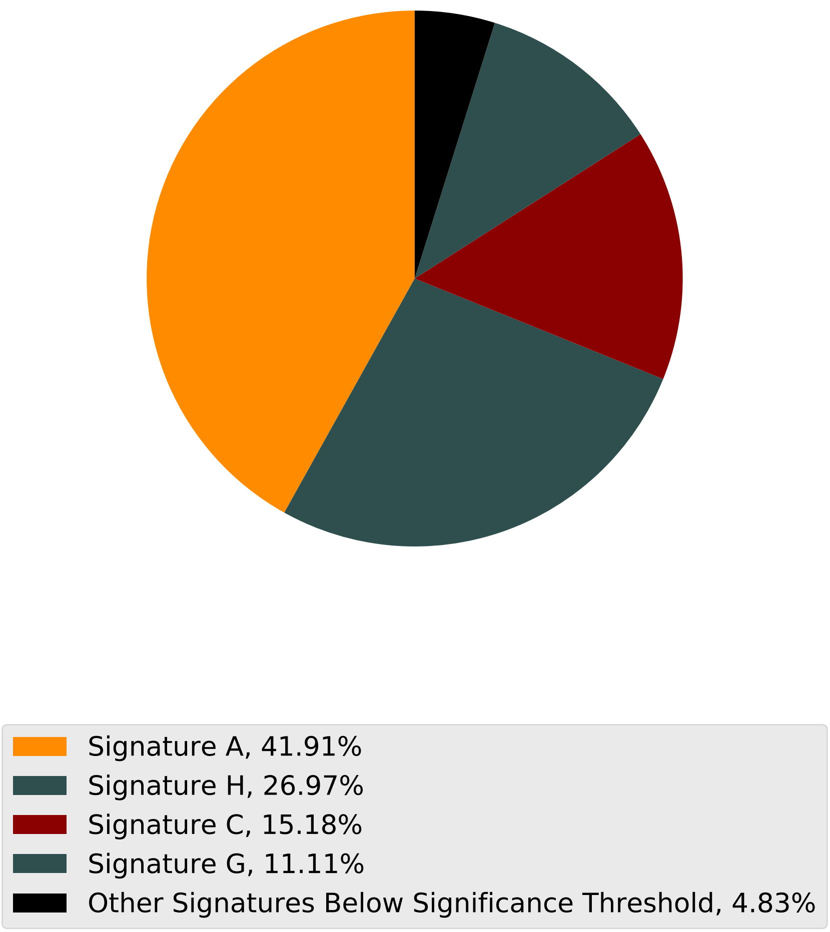

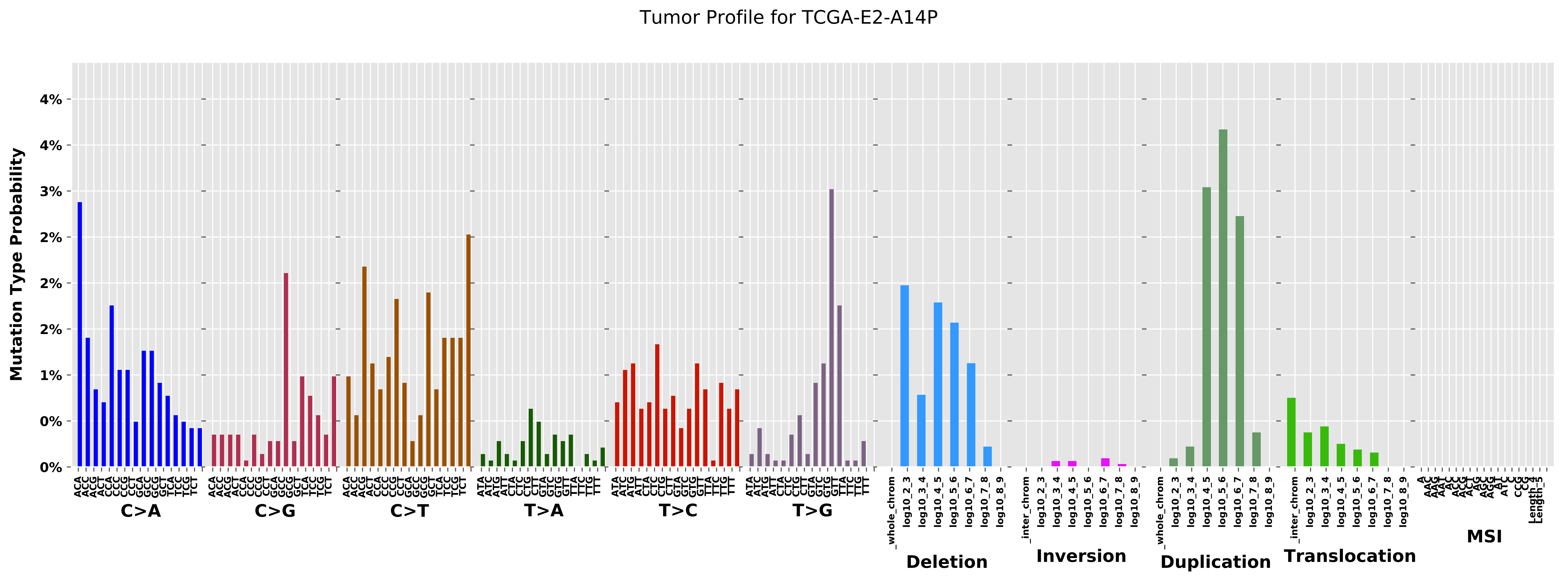

Tumor Profile for TCGA-E2-A14P
Mutation Type Probability
4%
4%
3%
2%
2%
2%
1%
0%
0%
C>A
C>G
C>T
T>A
T>C
T>G
Deletion
Inversion
Duplication
Translocation
MSI

Cancer processes Weights for TCGA-AZ-6601

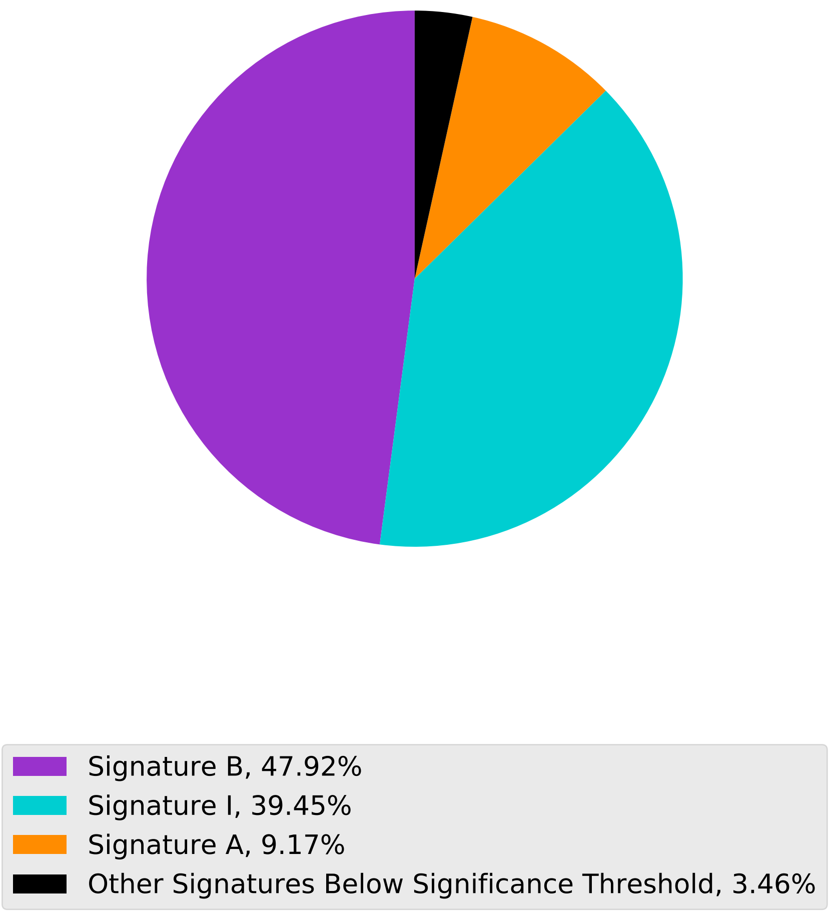

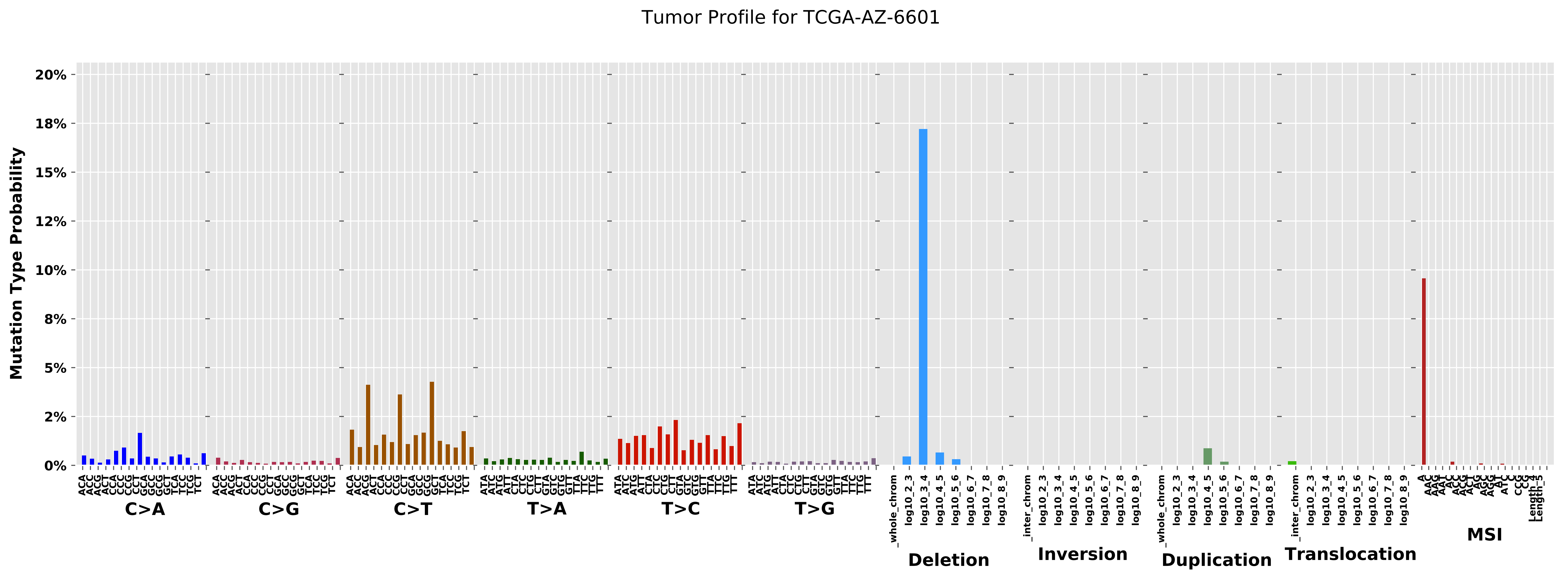

Tumor Profile for TCGA-AZ-6601
Mutation Type Probability
20%
18%
15%
12%
10%
8%
5%
2%
0%
C>A
C>G
C>T
T>A
T>C
T>G
Deletion
Inversion
Duplication
Translocation
MSI

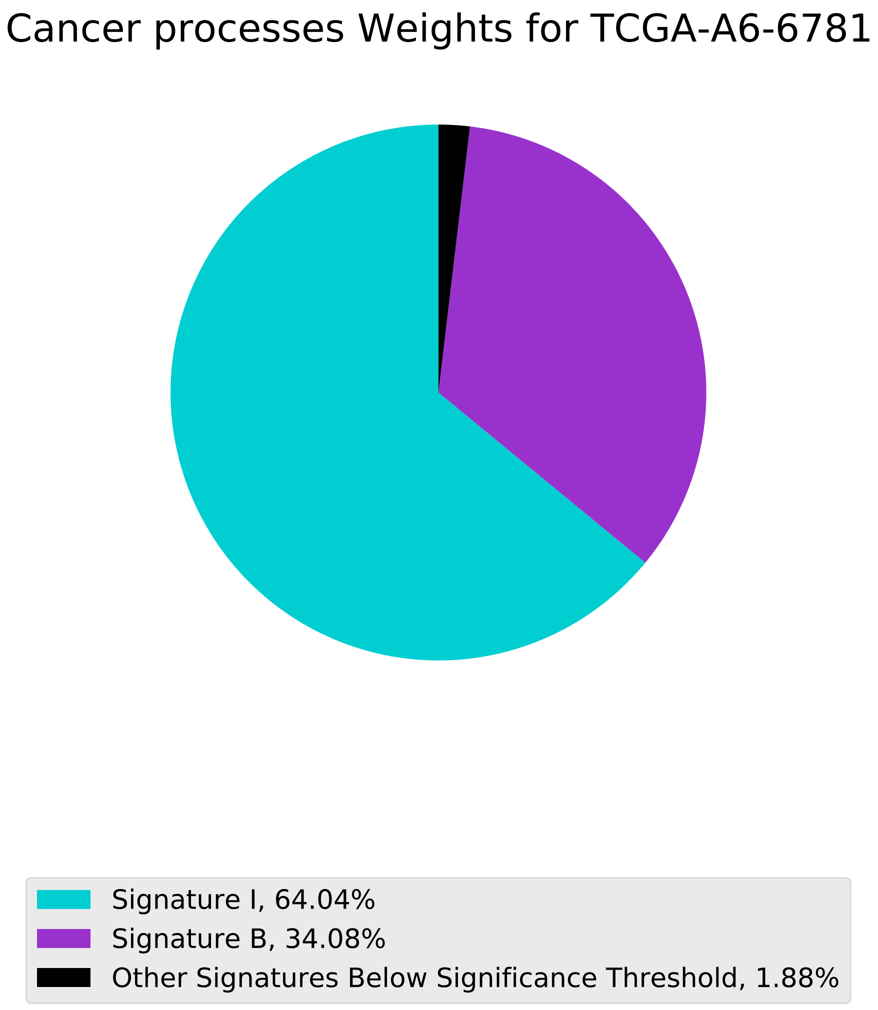
Cancer processes Weights for TCGA-A6-6781
Signature I, 64.04%
Signature B, 34.08%
Other Signatures Below Significance Threshold, 1.88%

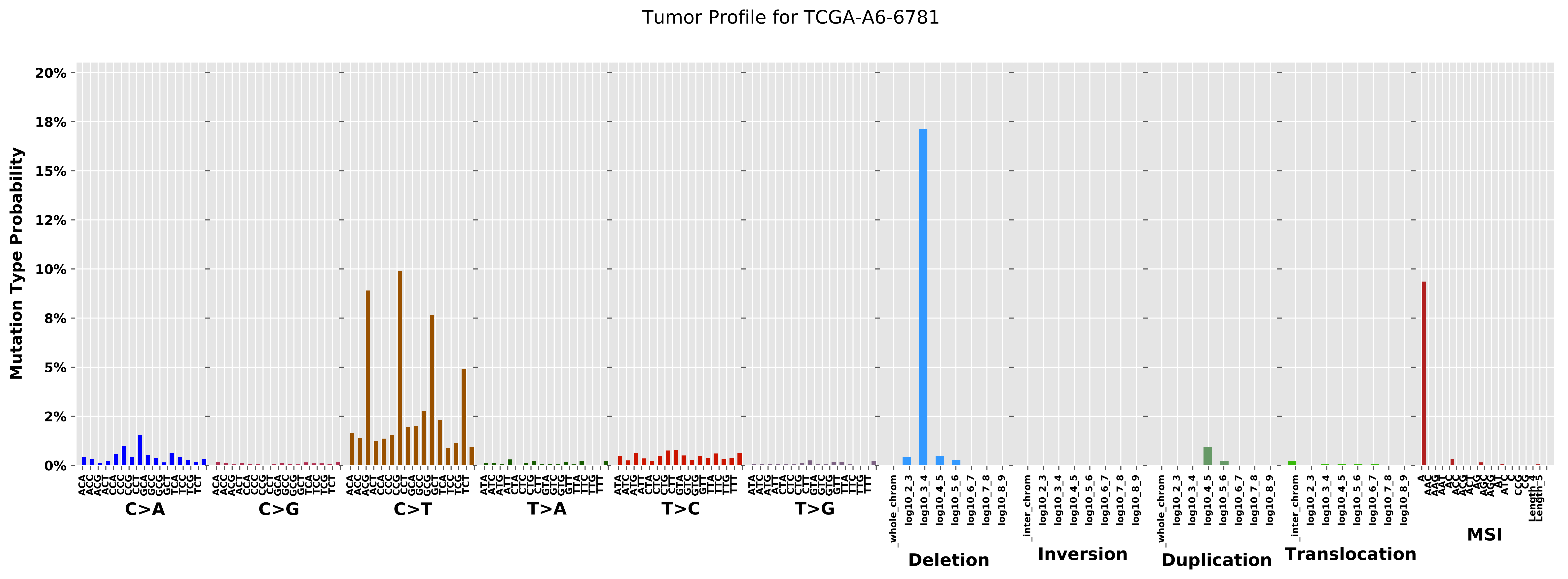

Tumor Profile for TCGA-A6-6781
Mutation Type Probability
C>A
C>G
C>T
T>A
T>C
T>G
Deletion
Inversion
Duplication
Translocation
MSI

Cancer processes Weights for TCGA-GM-A2DF

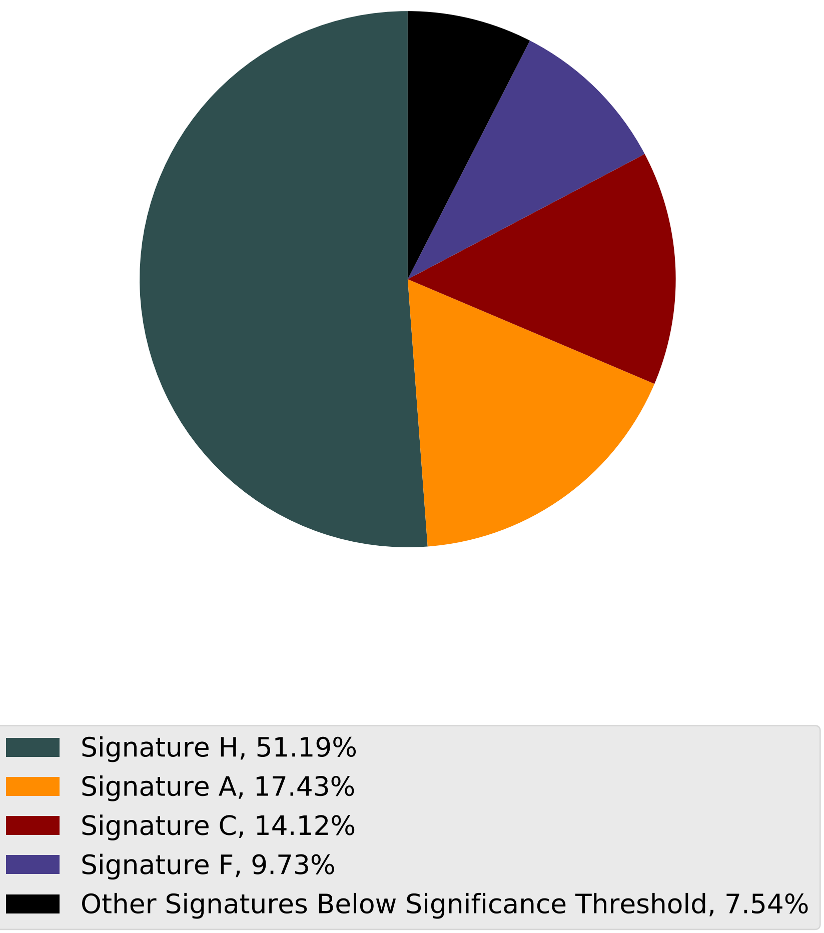

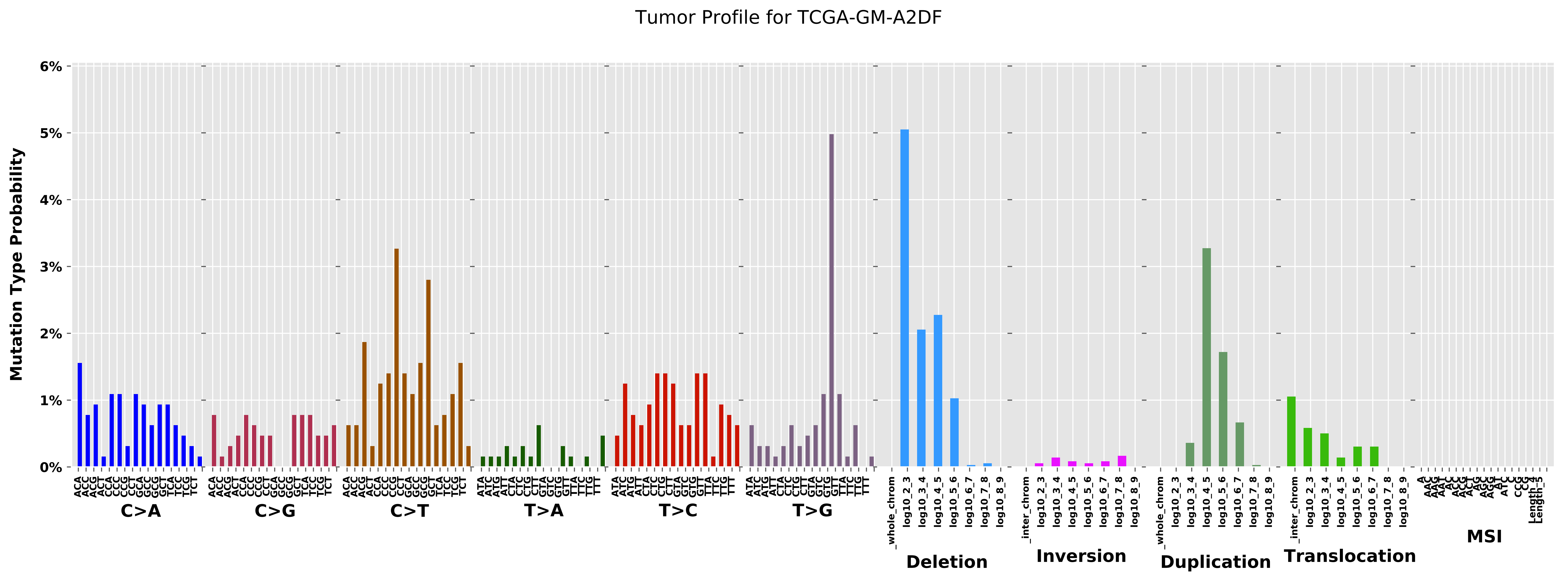

Tumor Profile for TCGA-GM-A2DF
Mutation Type Probability
6%
5%
4%
3%
2%
1%
0%
C>A
C>G
C>T
T>A
T>C
T>G
Deletion
Inversion
Duplication
Translocation
MSI

Cancer processes Weights for TCGA-C8-A12L

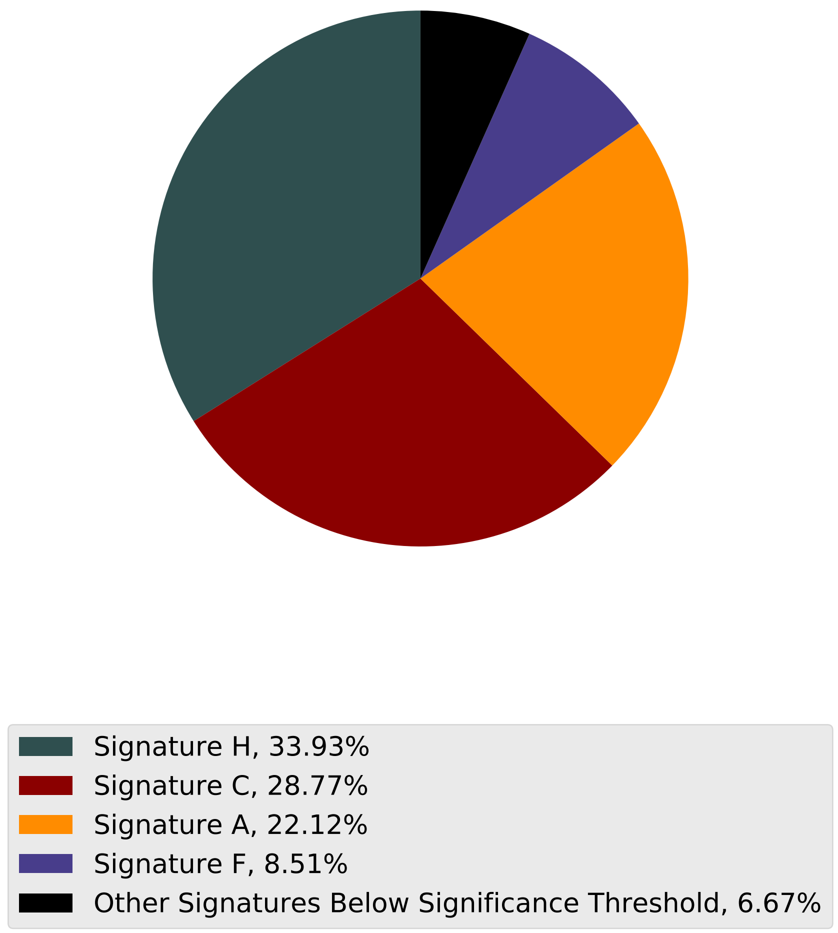

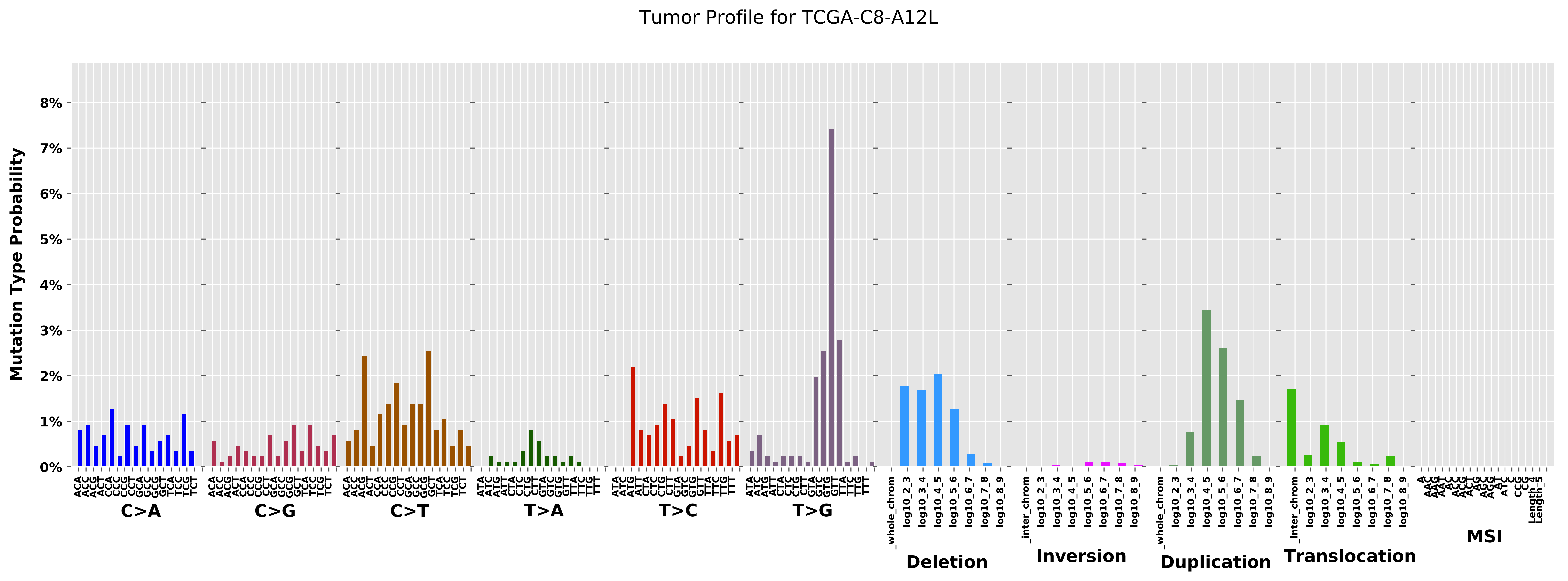
Tumor Profile for TCGA-C8-A12L
Mutation Type Probability
C>A
C>G
C>T
T>A
T>C
T>G
Deletion
Inversion
Duplication
Translocation
MSI

Cancer processes Weights for TCGA-AG-3890

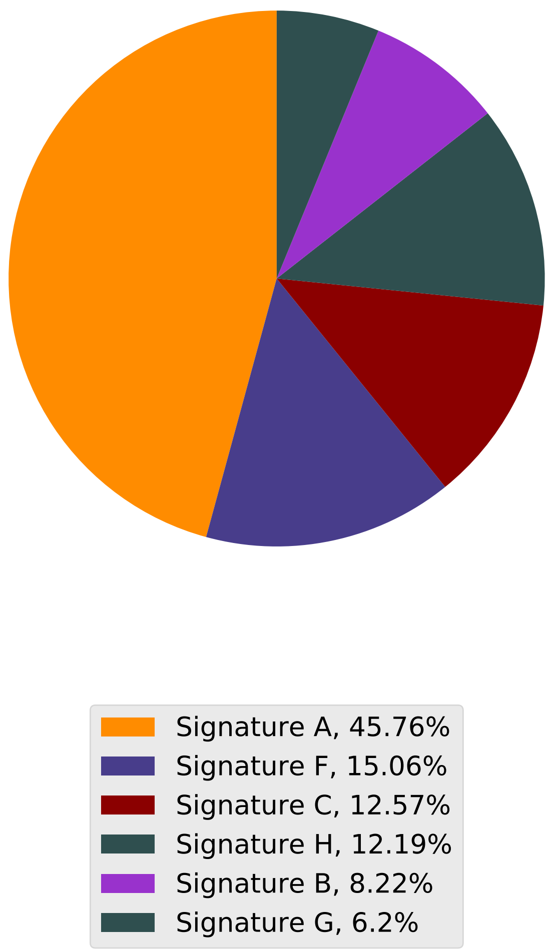

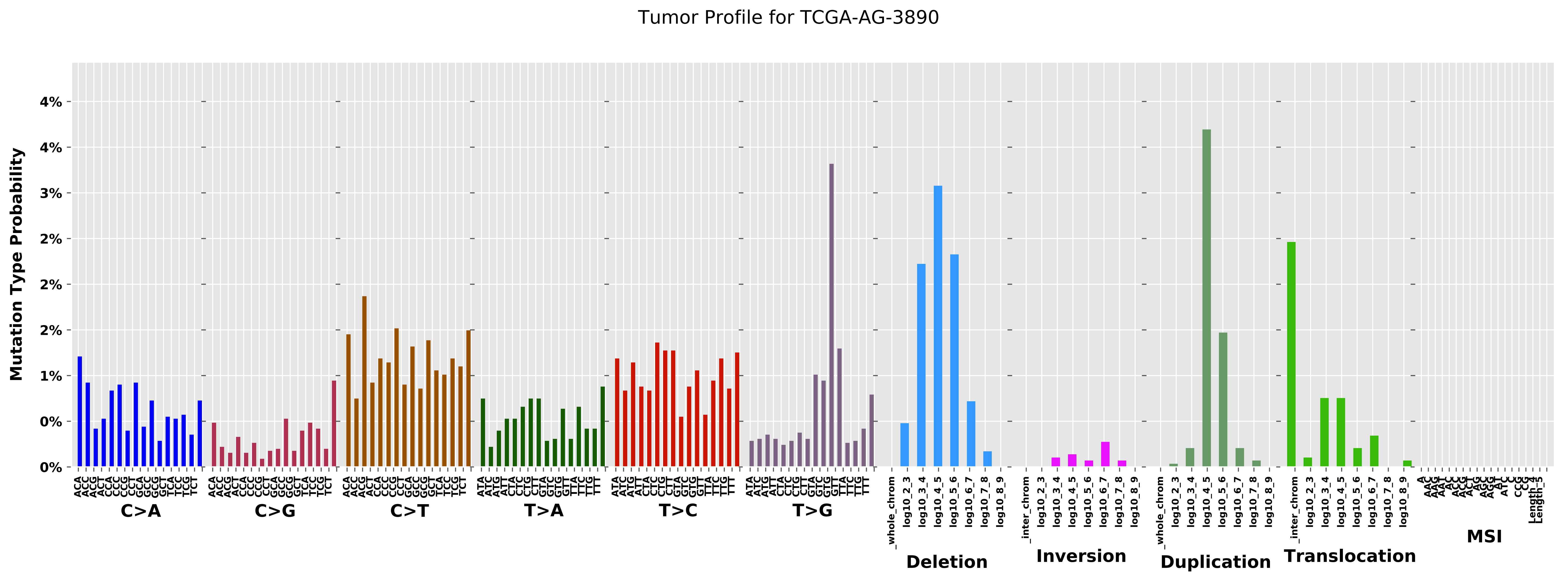
Tumor Profile for TCGA-AG-3890
Mutation Type Probability
4%
4%
3%
2%
2%
2%
1%
0%
0%
C>A
C>G
C>T
T>A
T>C
T>G
Deletion
Inversion
Duplication
Translocation
MSI

Cancer processes Weights for TCGA-E2-A1LK

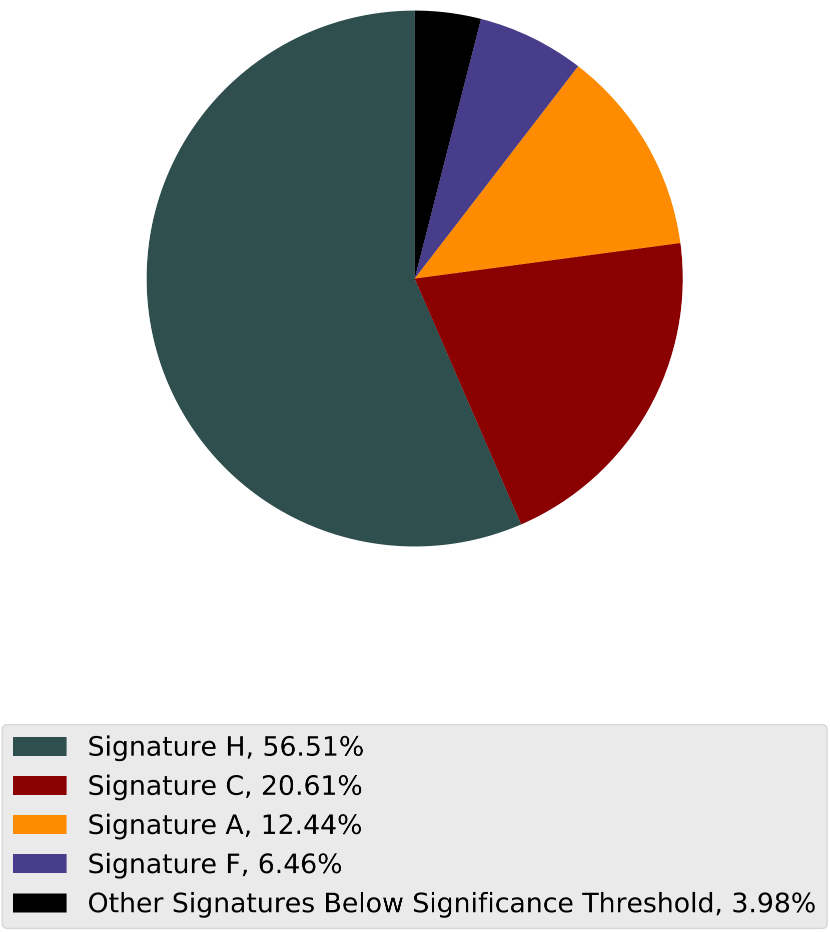

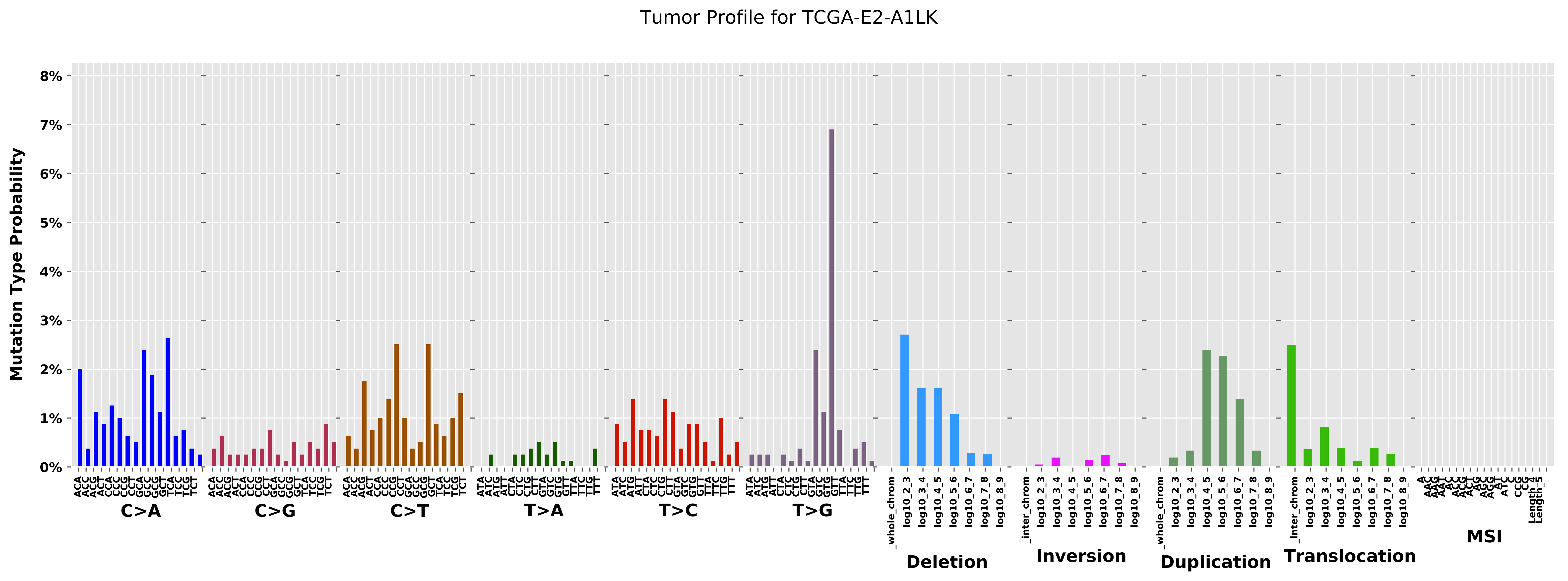

Tumor Profile for TCGA-E2-A1LK
Mutation Type Probability
0%
1%
2%
3%
4%
5%
6%
7%
8%
C>A
C>G
C>T
T>A
T>C
T>G
Deletion
Inversion
Duplication
Translocation
MSI
_whole_chrom
_inter_chrom
log10_2_3
log10_3_4
log10_4_5
log10_5_6
log10_6_7
log10_7_8
log10_8_9

Cancer processes Weights for TCGA-A2-A3Y0

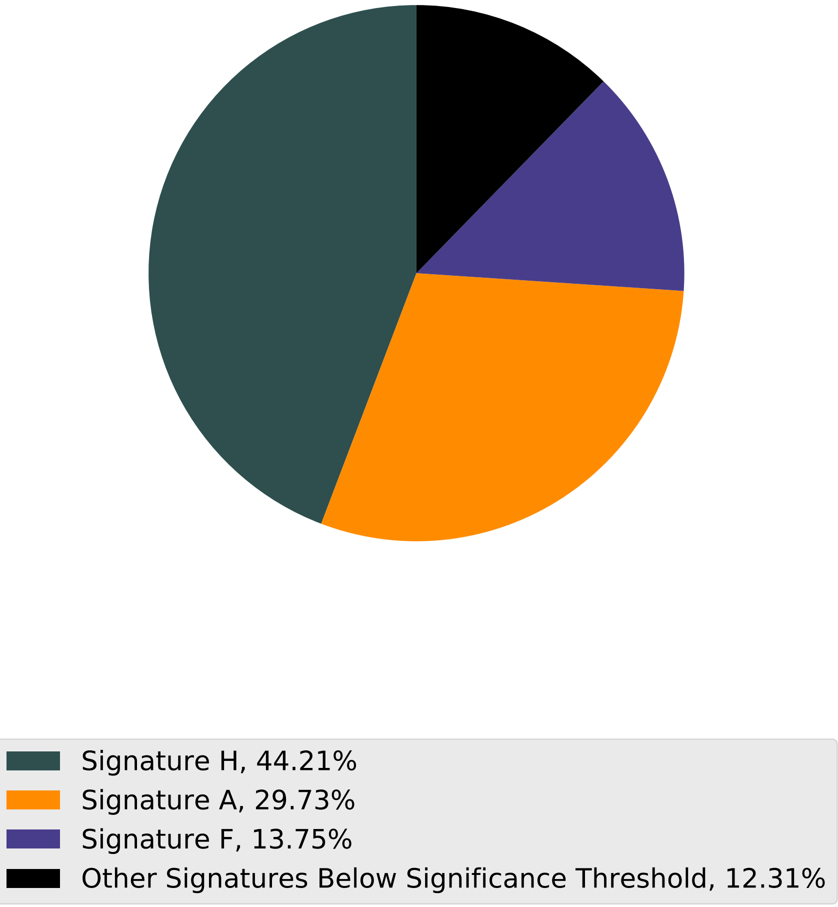

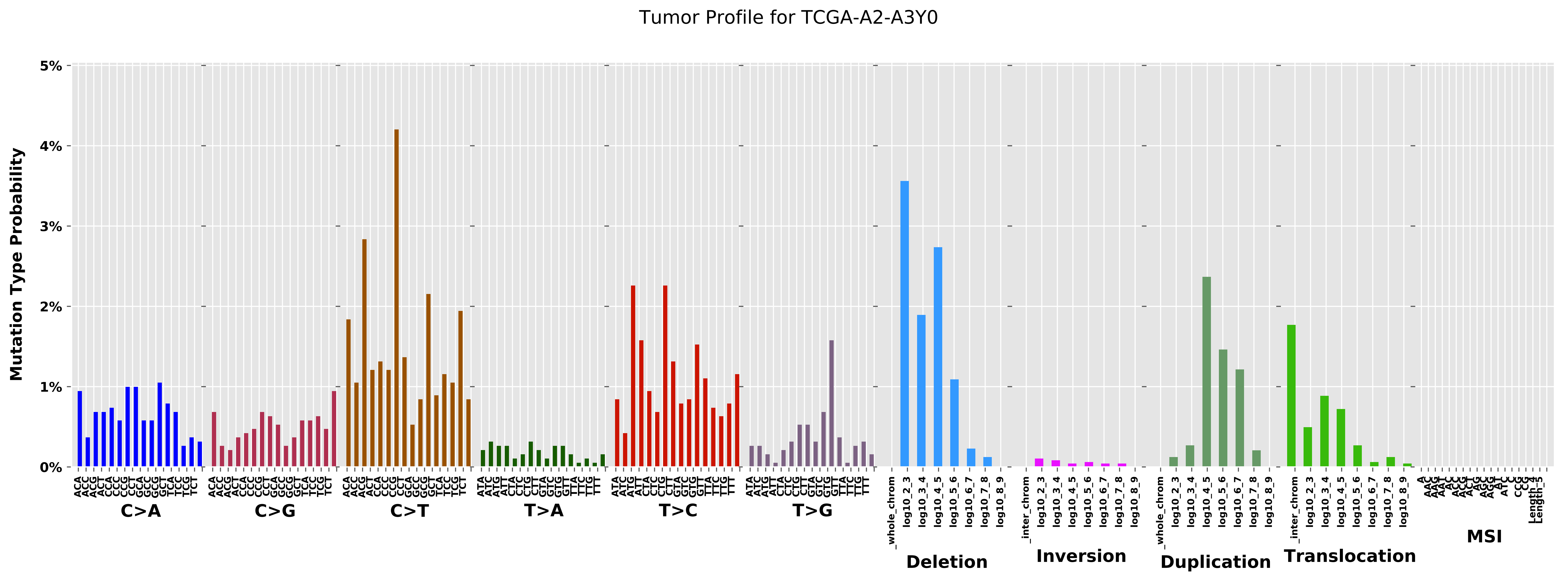
Tumor Profile for TCGA-A2-A3Y0
Mutation Type Probability
5%
4%
3%
2%
1%
0%
C>A
C>G
C>T
T>A
T>C
T>G
_whole_chrom
log10_2_3
log10_3_4
log10_4_5
log10_5_6
log10_6_7
log10_7_8
log10_8_9
Deletion
_inter_chrom
Inversion
Duplication
Translocation
MSI

Cancer processes Weights for TCGA-B6-A0I1

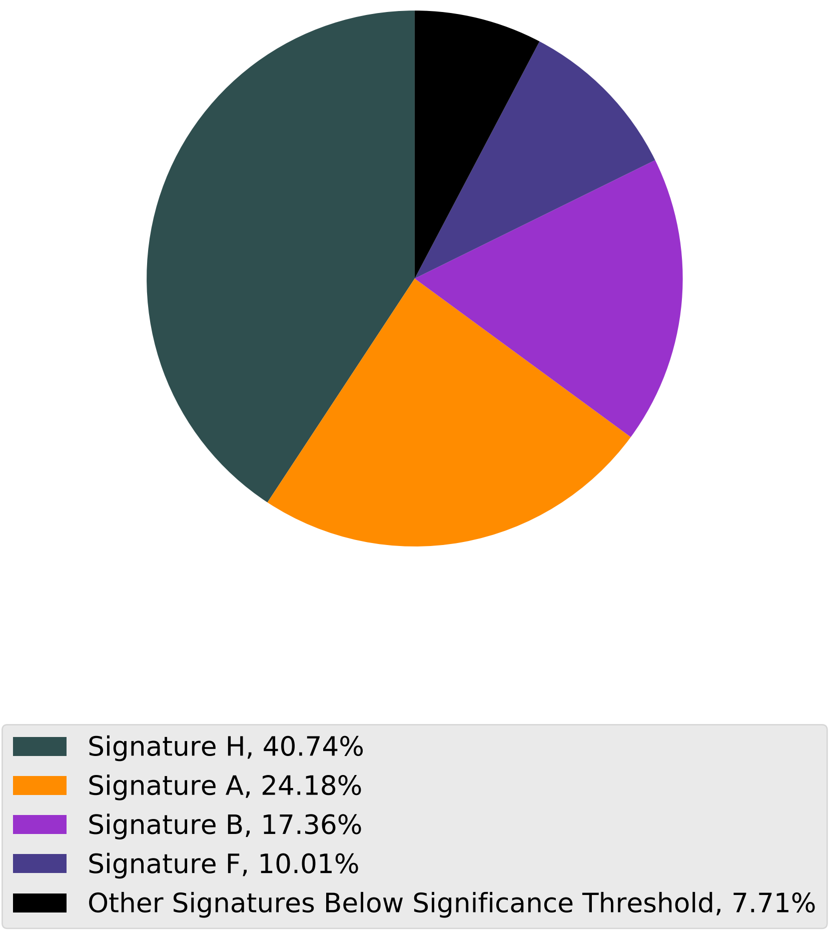

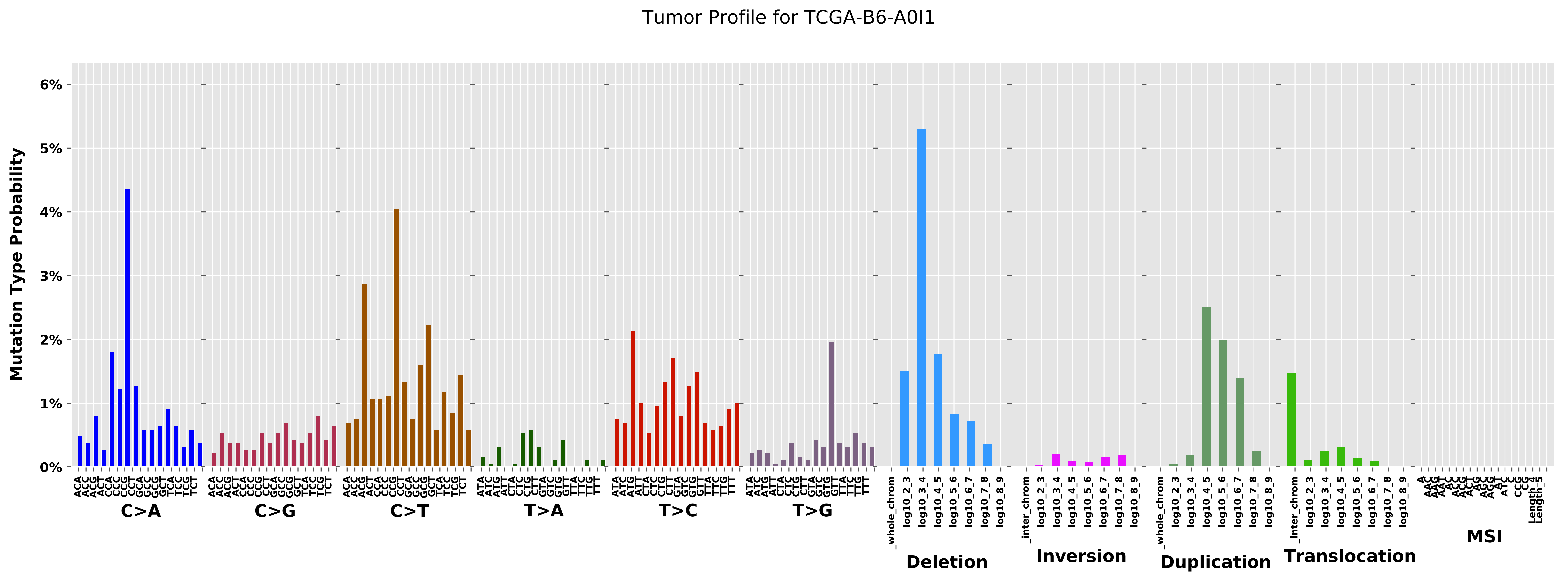
Tumor Profile for TCGA-B6-A0I1
Mutation Type Probability
6%
5%
4%
3%
2%
1%
0%
C>A
C>G
C>T
T>A
T>C
T>G
Deletion
Inversion
Duplication
Translocation
MSI
_whole_chrom
_inter_chrom
log10_2_3
log10_3_4
log10_4_5
log10_5_6
log10_6_7
log10_7_8
log10_8_9

Cancer processes Weights for TCGA-E2-A15E

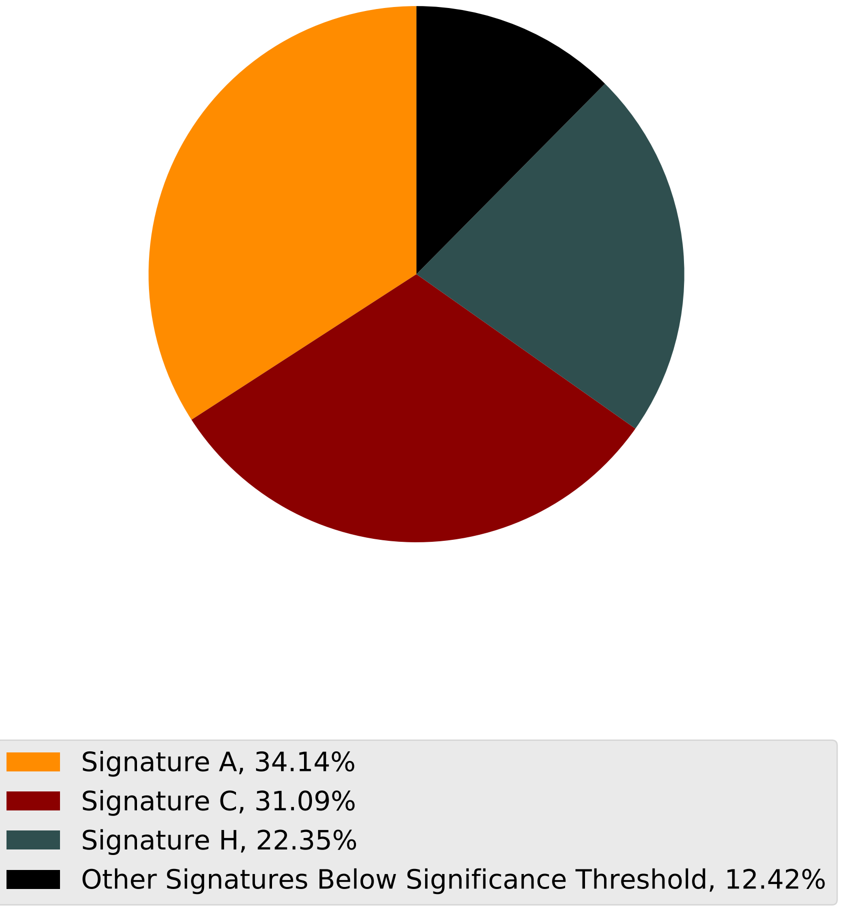

Tumor Profile for TCGA-E2-A15E

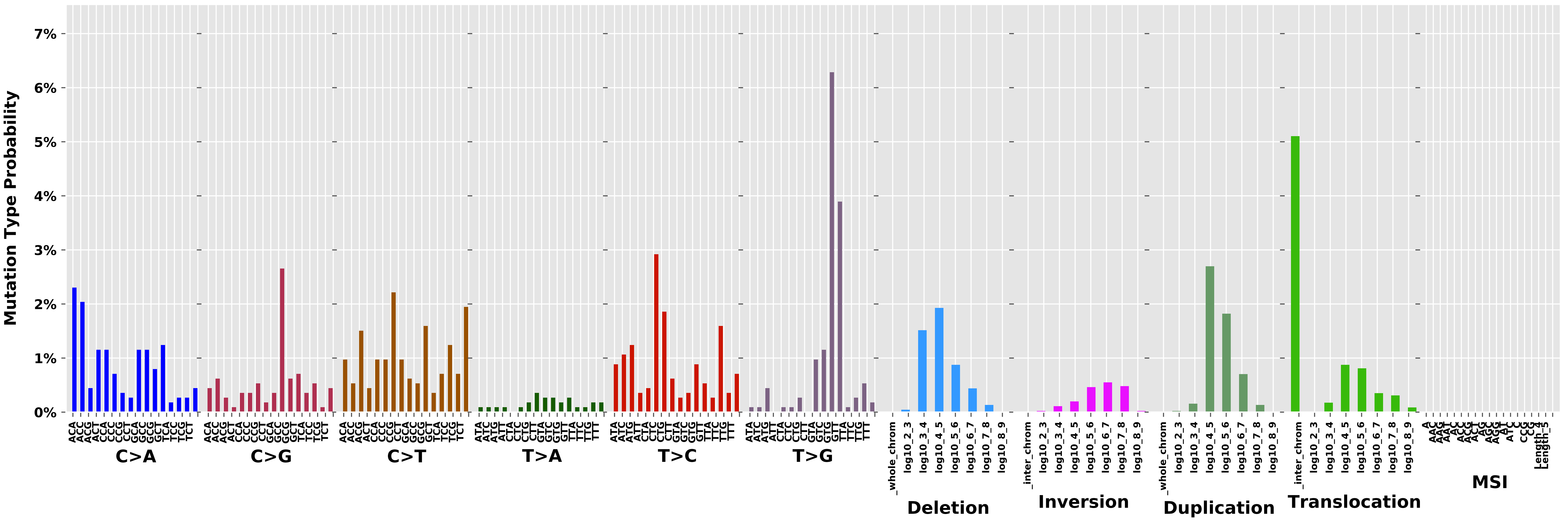

Cancer processes Weights for TCGA-A6-2680

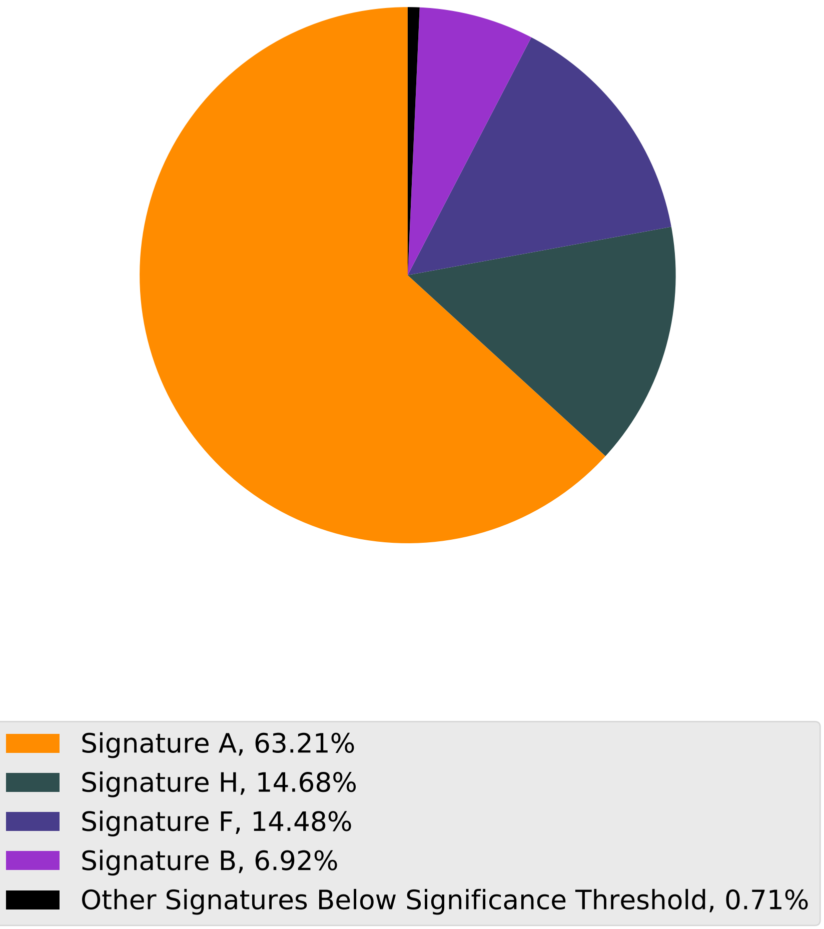

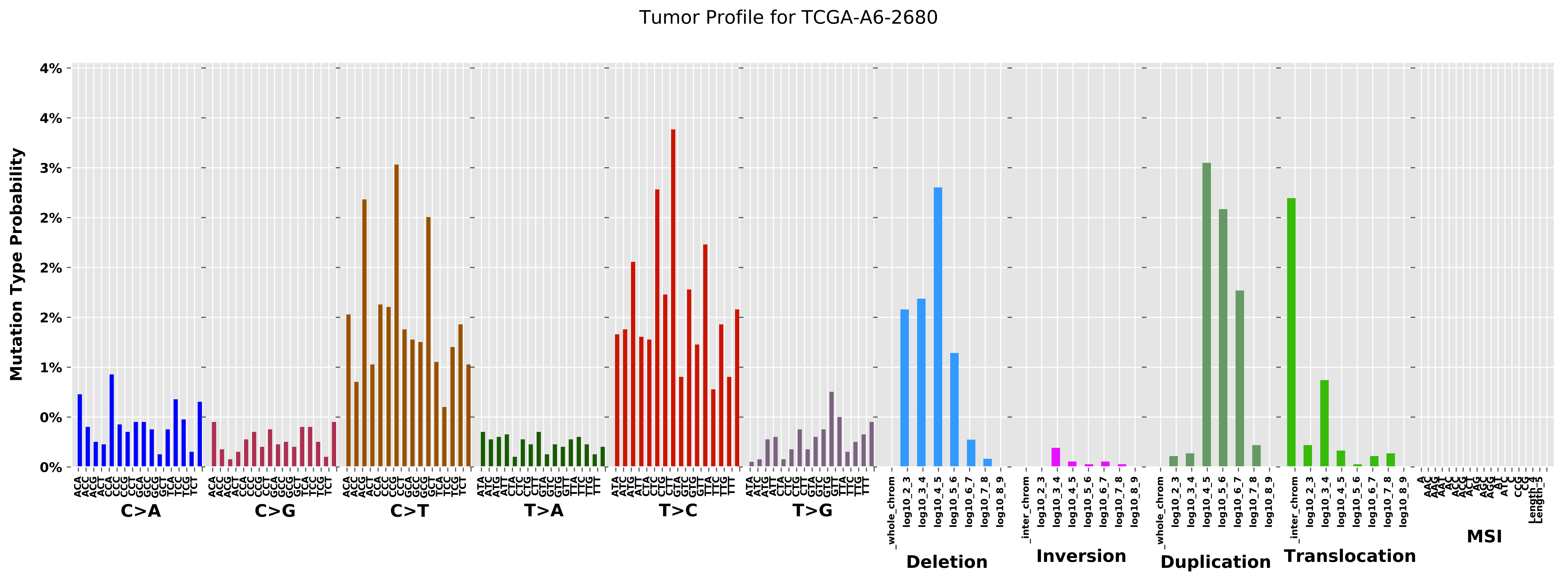

Tumor Profile for TCGA-A6-2680
Mutation Type Probability
C>A
C>G
C>T
T>A
T>C
T>G
Deletion
Inversion
Duplication
Translocation
MSI

Cancer processes Weights for TCGA-A8-A07B

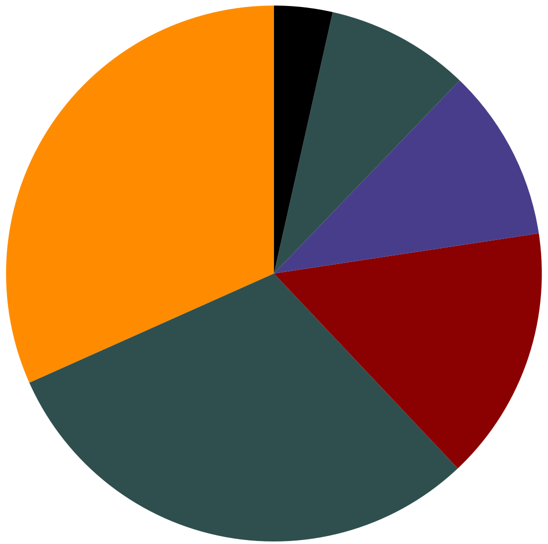

- Signature A, 31.68%
- Signature H, 30.36%
- Signature C, 15.35%
- Signature F, 10.41%
- Signature G, 8.67%
- Other Signatures Below Significance Threshold, 3.52%

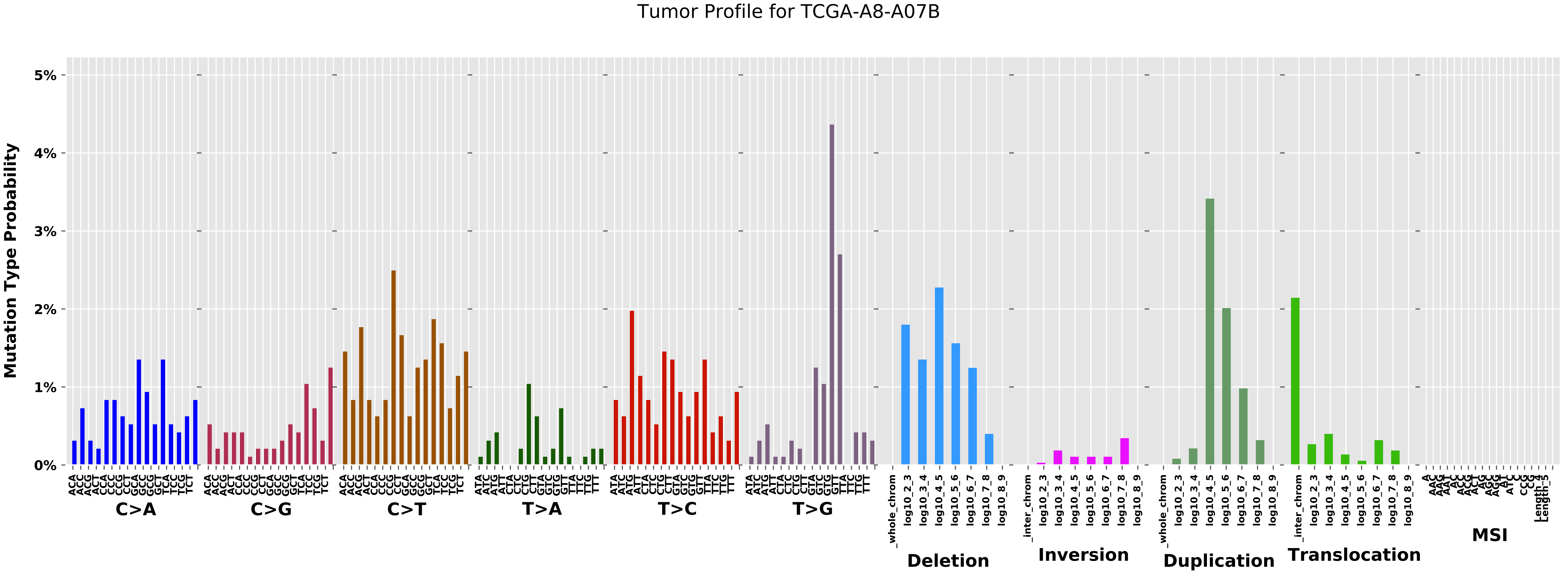

Tumor Profile for TCGA-A8-A07B
Mutation Type Probability
0%
1%
2%
3%
4%
5%
C>A
C>G
C>T
T>A
T>C
T>G
Deletion
Inversion
Duplication
Translocation
MSI

Cancer processes Weights for TCGA-AO-A0J6

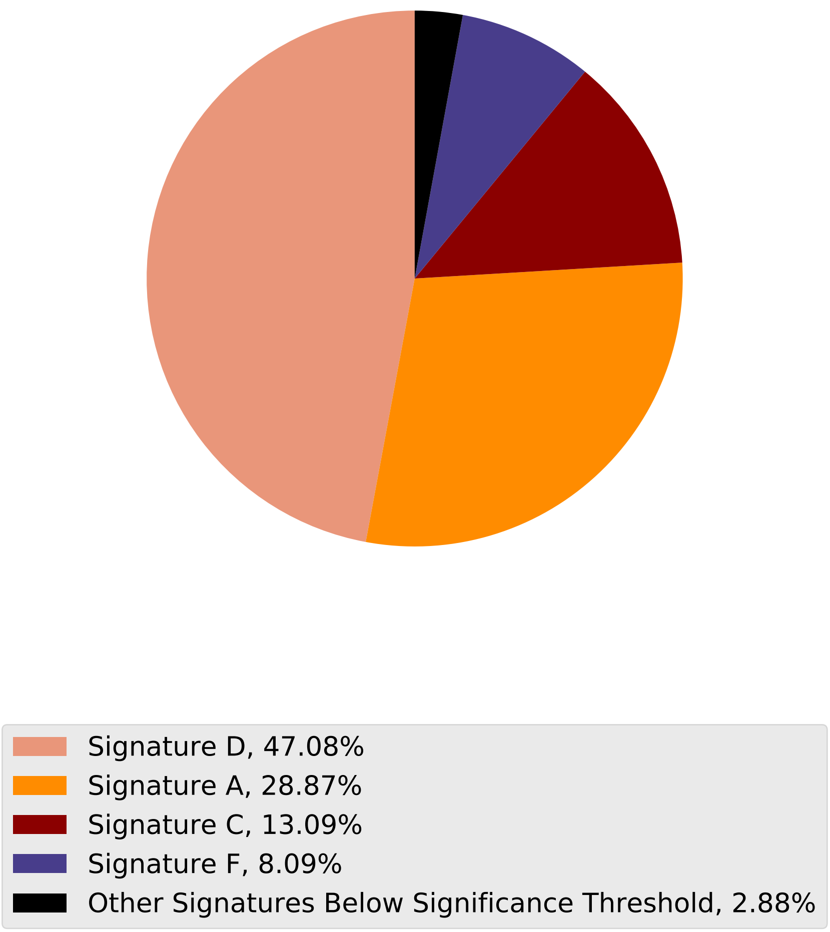

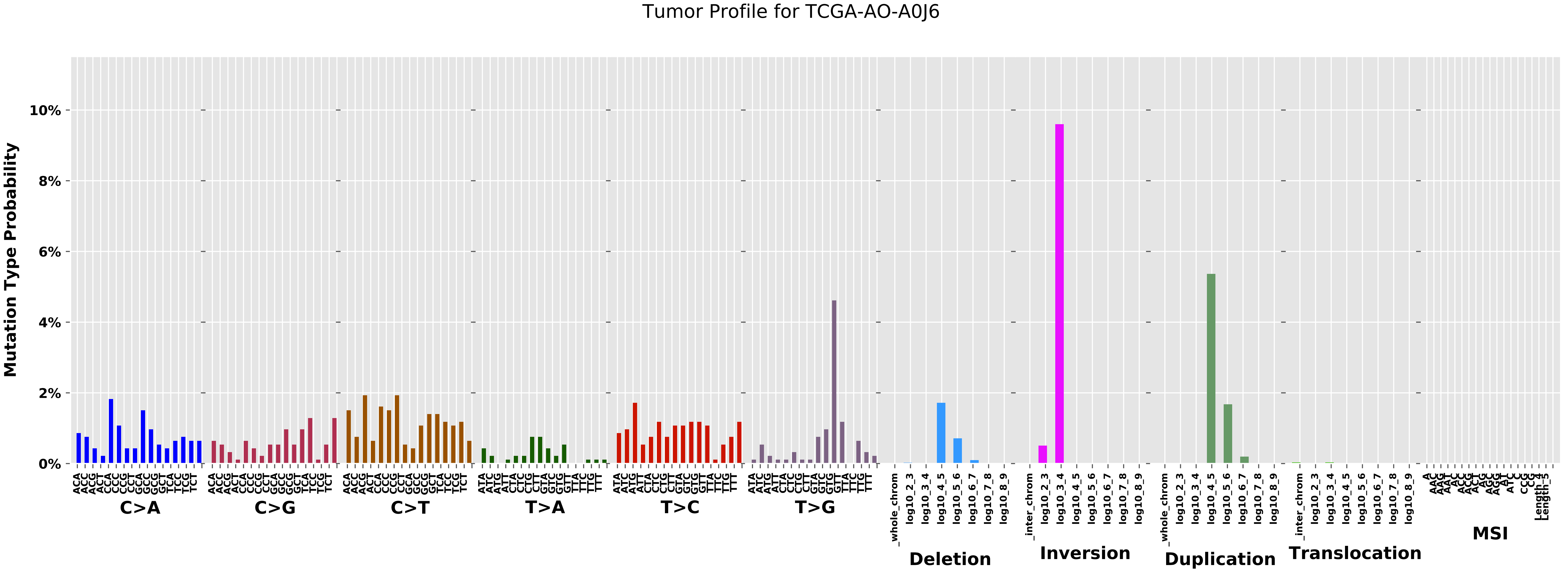
Tumor Profile for TCGA-AO-A0J6
Mutation Type Probability
0%
2%
4%
6%
8%
10%
C>A
C>G
C>T
T>A
T>C
T>G
Deletion
Inversion
Duplication
Translocation
MSI

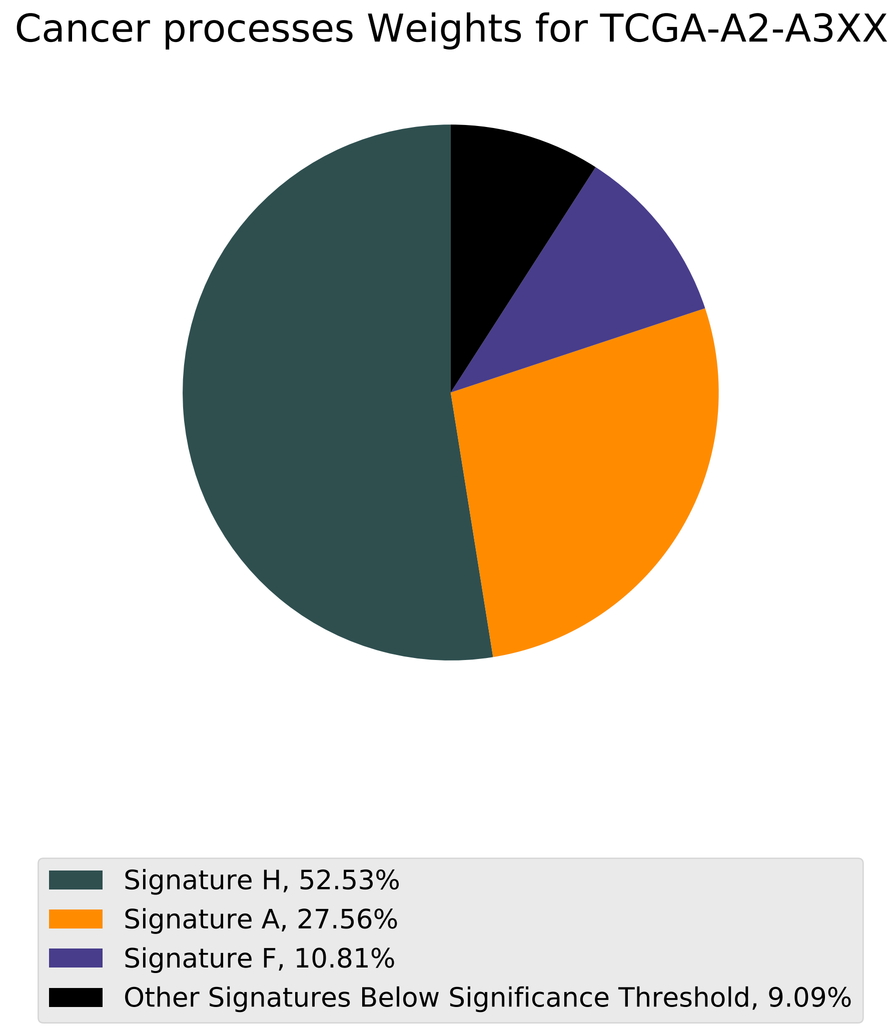

Cancer processes Weights for TCGA-A2-A3XX
Signature H, 52.53%
Signature A, 27.56%
Signature F, 10.81%
Other Signatures Below Significance Threshold, 9.09%

Tumor Profile for TCGA-A2-A3XX

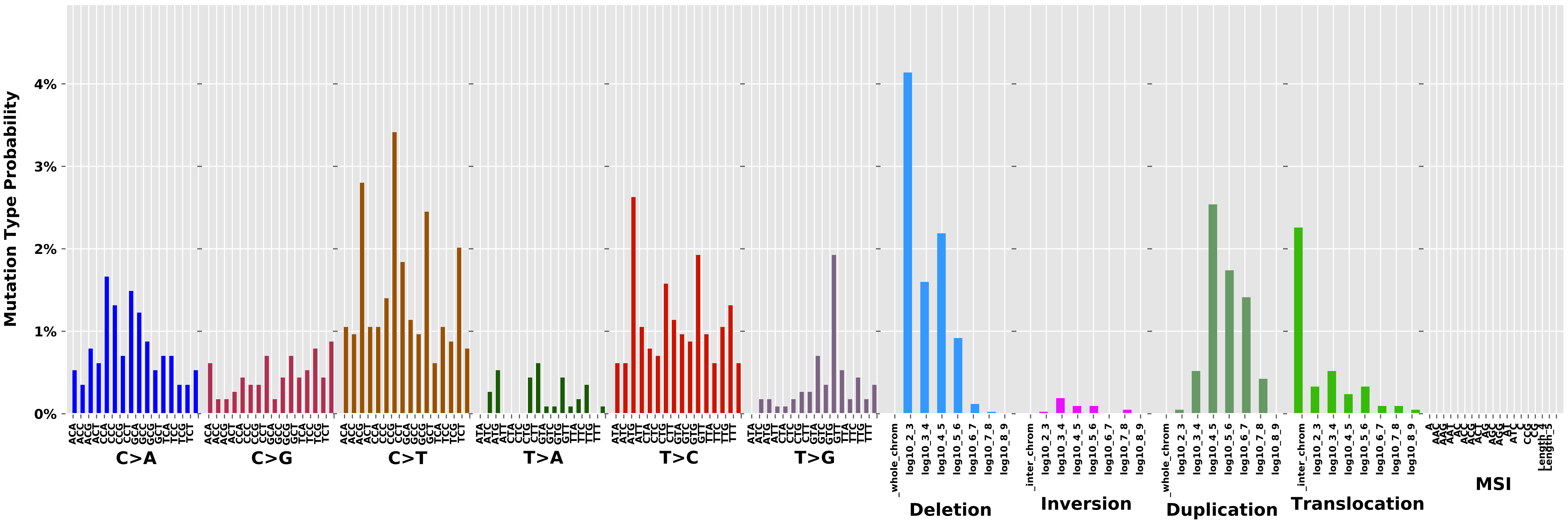

Cancer processes Weights for TCGA-A8-A075

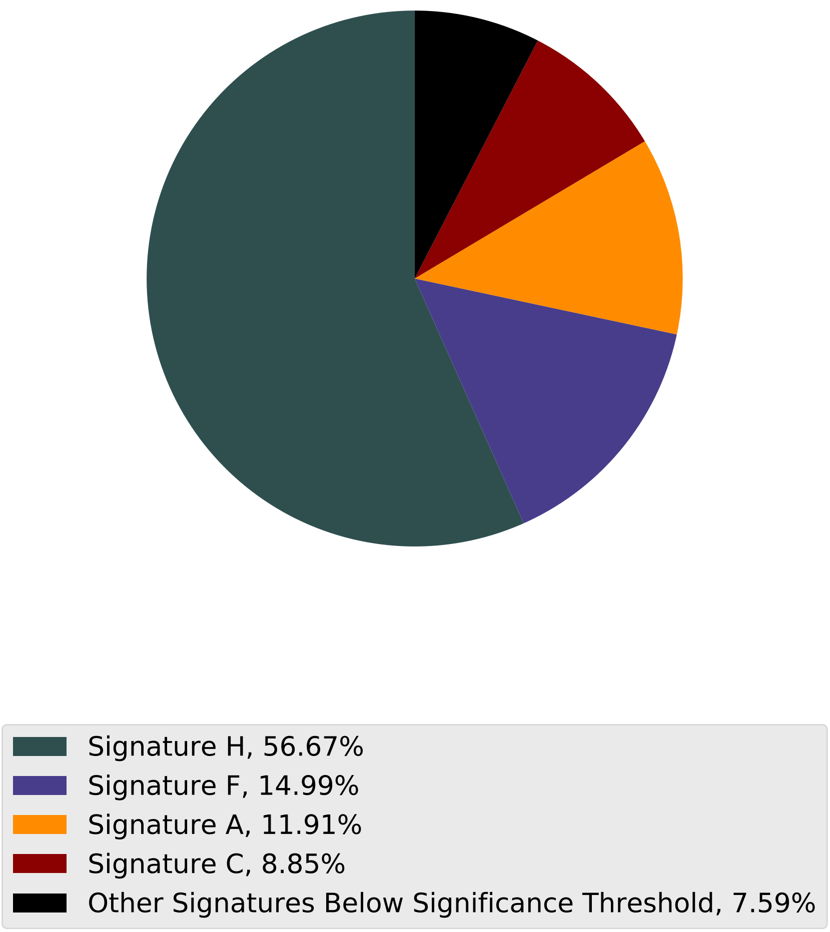

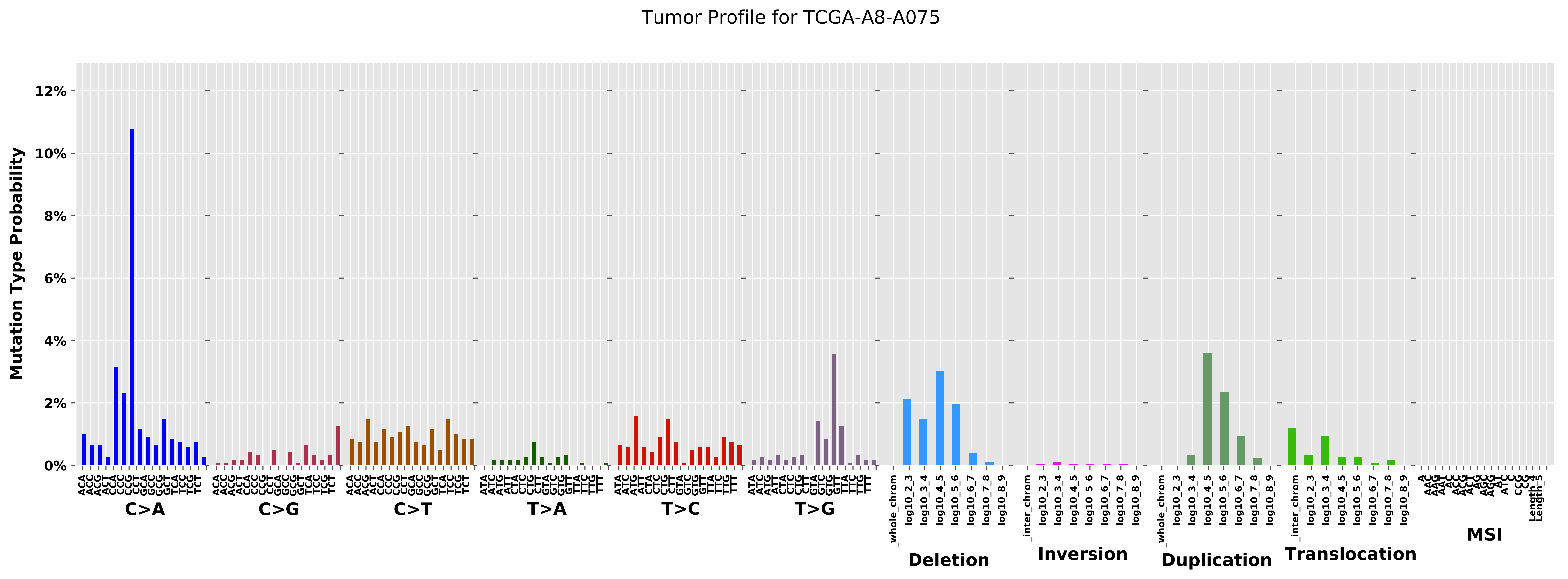
Tumor Profile for TCGA-A8-A075
Mutation Type Probability
0%
2%
4%
6%
8%
10%
12%
C>A
C>G
C>T
T>A
T>C
T>G
Deletion
Inversion
Duplication
Translocation
MSI
_whole_chrom
_inter_chrom
log10_2_3
log10_3_4
log10_4_5
log10_5_6
log10_6_7
log10_7_8
log10_8_9

Cancer processes Weights for TCGA-AR-A1AY

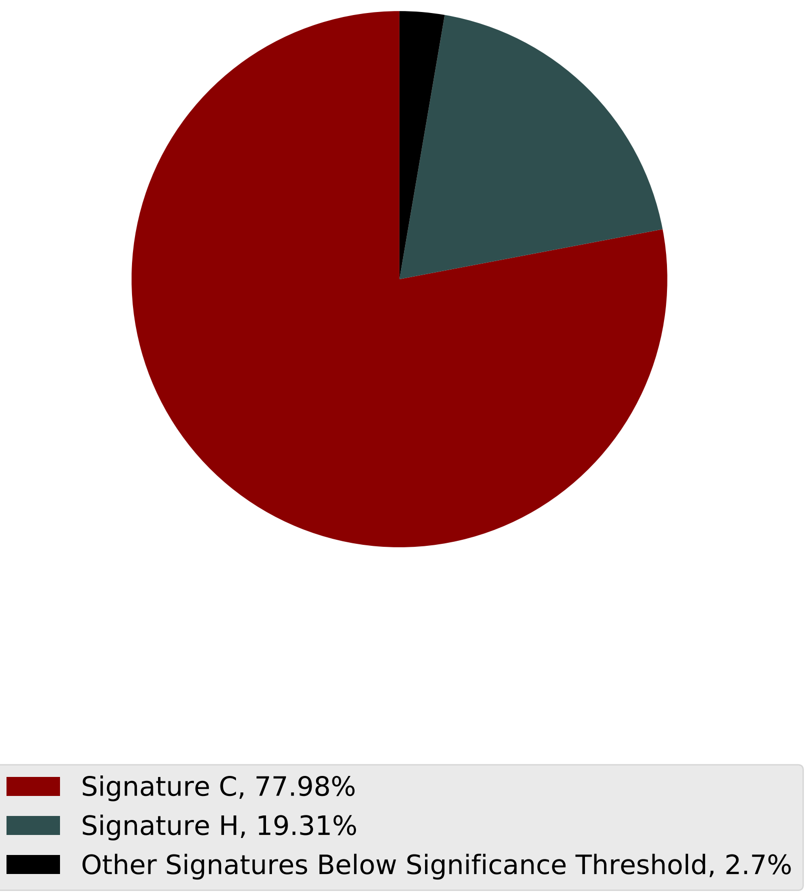

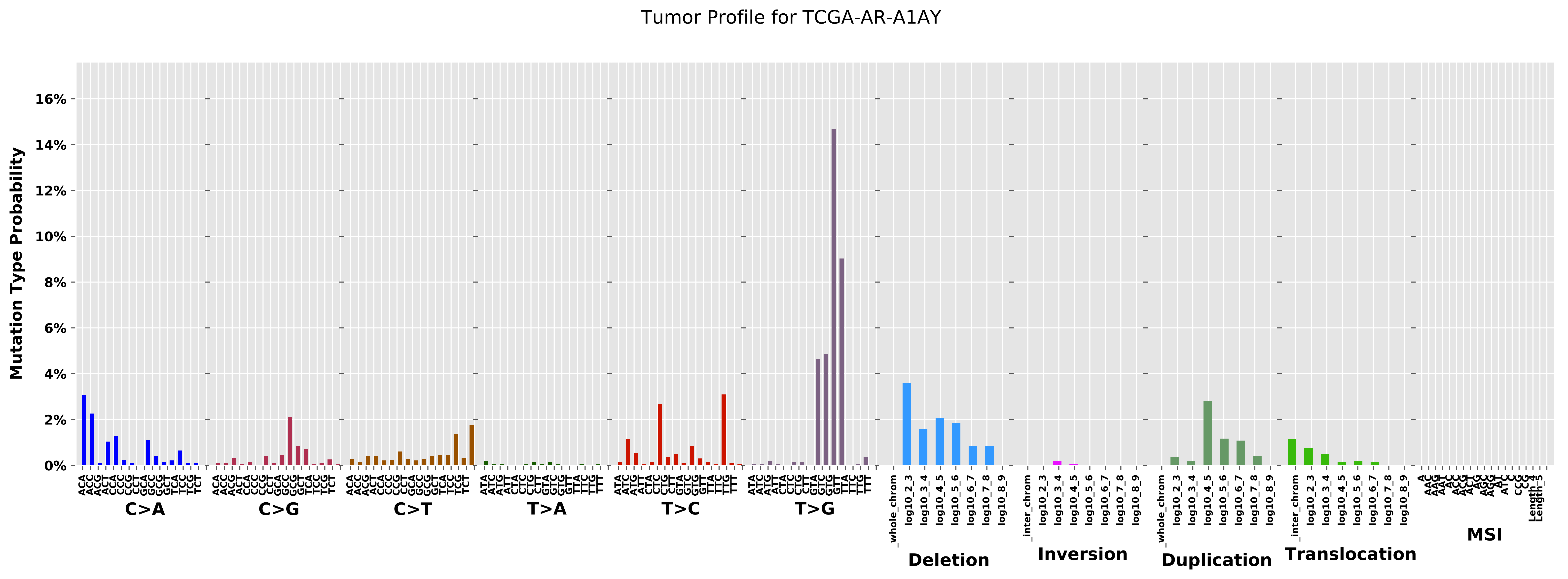
Tumor Profile for TCGA-AR-A1AY
Mutation Type Probability
16%
14%
12%
10%
8%
6%
4%
2%
0%
C>A
C>G
C>T
T>A
T>C
T>G
_whole_chrom
log10_2_3
log10_3_4
log10_4_5
log10_5_6
log10_6_7
log10_7_8
log10_8_9
Deletion
_inter_chrom
Inversion
Duplication
Translocation
MSI

Cancer processes Weights for TCGA-AG-3901

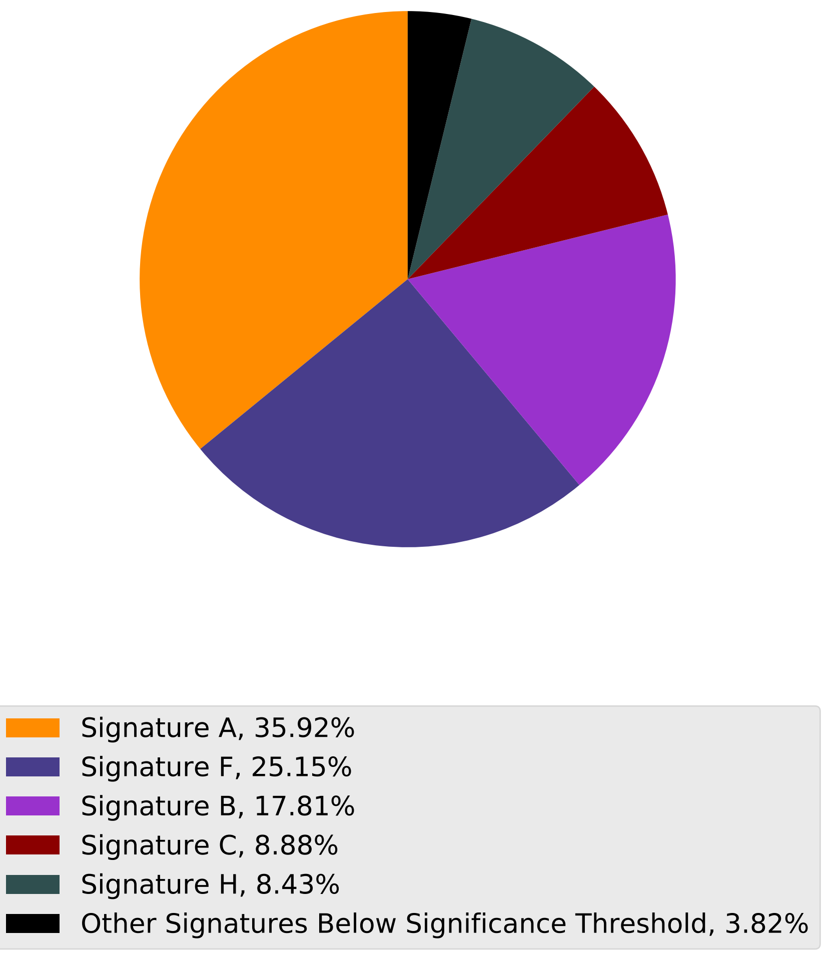

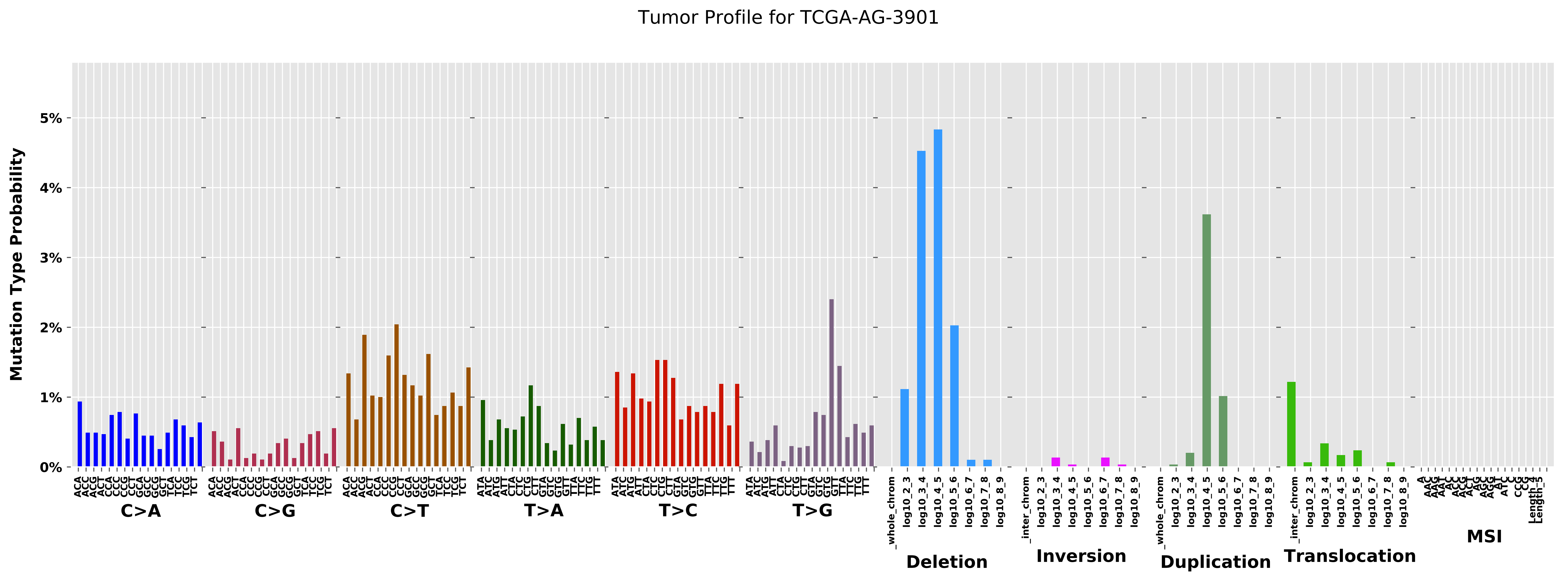
Tumor Profile for TCGA-AG-3901
Mutation Type Probability
0%
1%
2%
3%
4%
5%
C>A
C>G
C>T
T>A
T>C
T>G
Deletion
Inversion
Duplication
Translocation
MSI
_whole_chrom
_inter_chrom
log10_2_3
log10_3_4
log10_4_5
log10_5_6
log10_6_7
log10_7_8
log10_8_9

Cancer processes Weights for TCGA-E2-A152

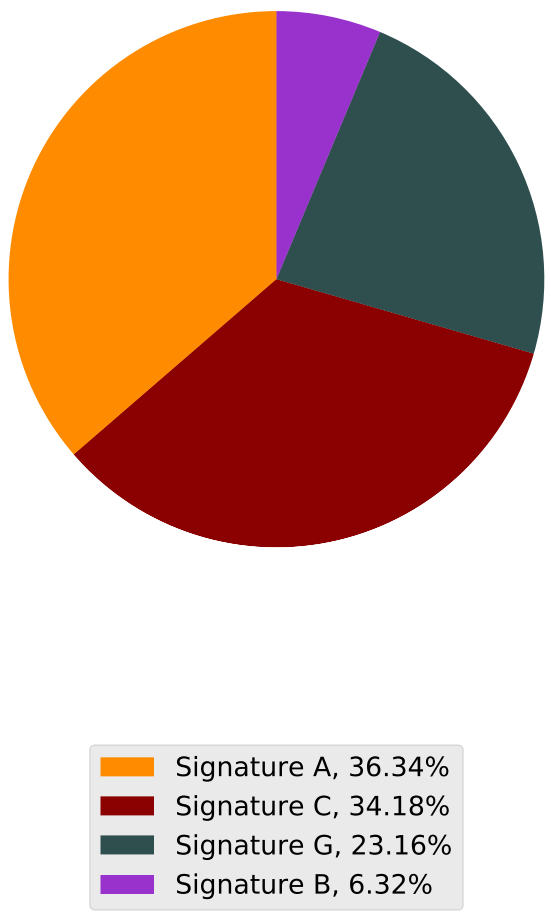

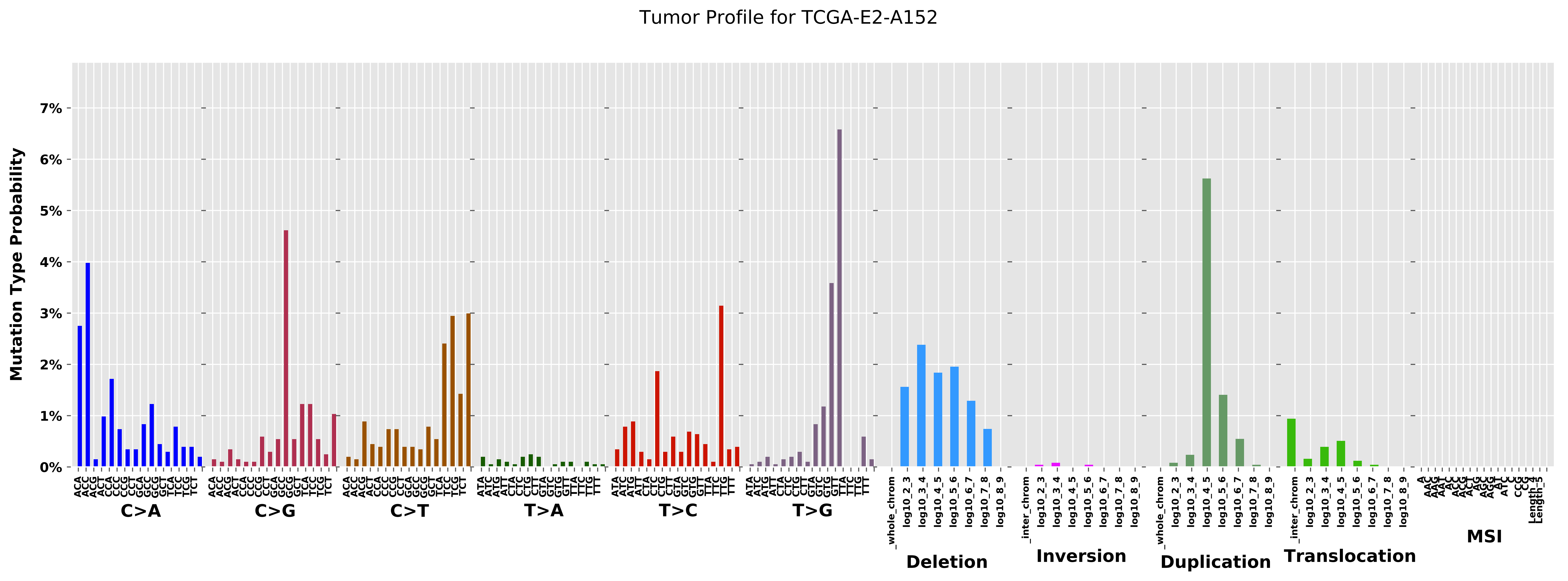

Tumor Profile for TCGA-E2-A152
Mutation Type Probability
0%
1%
2%
3%
4%
5%
6%
7%
C>A
C>G
C>T
T>A
T>C
T>G
Deletion
Inversion
Duplication
Translocation
MSI

Cancer processes Weights for TCGA-AG-4015

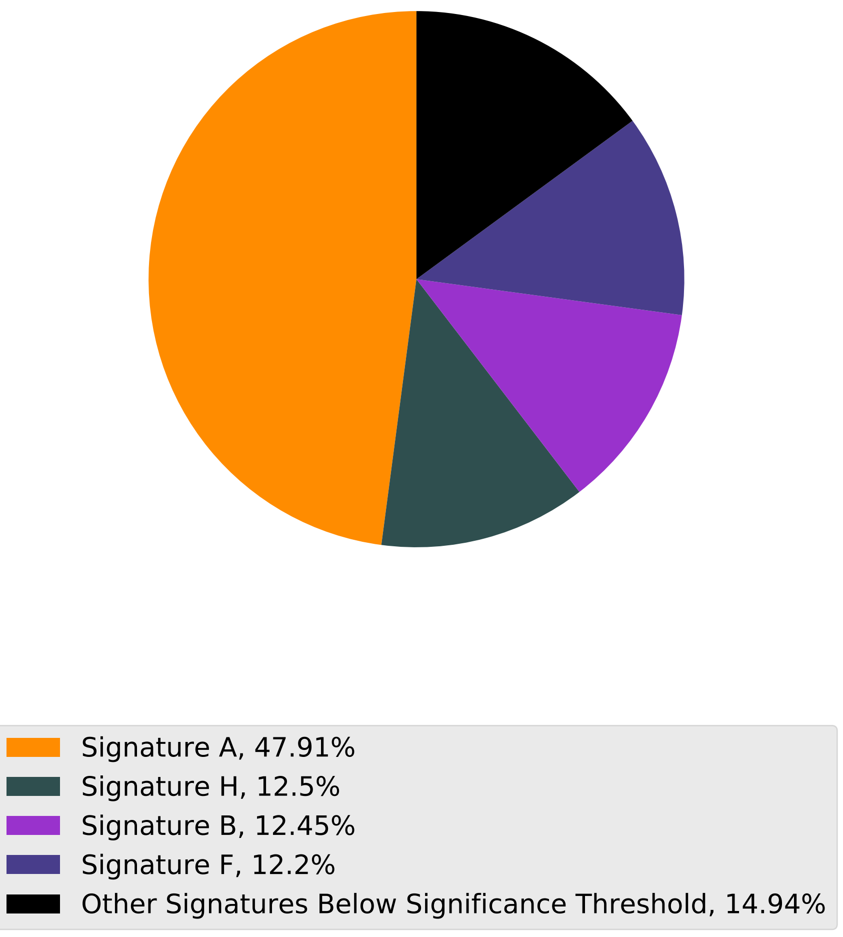

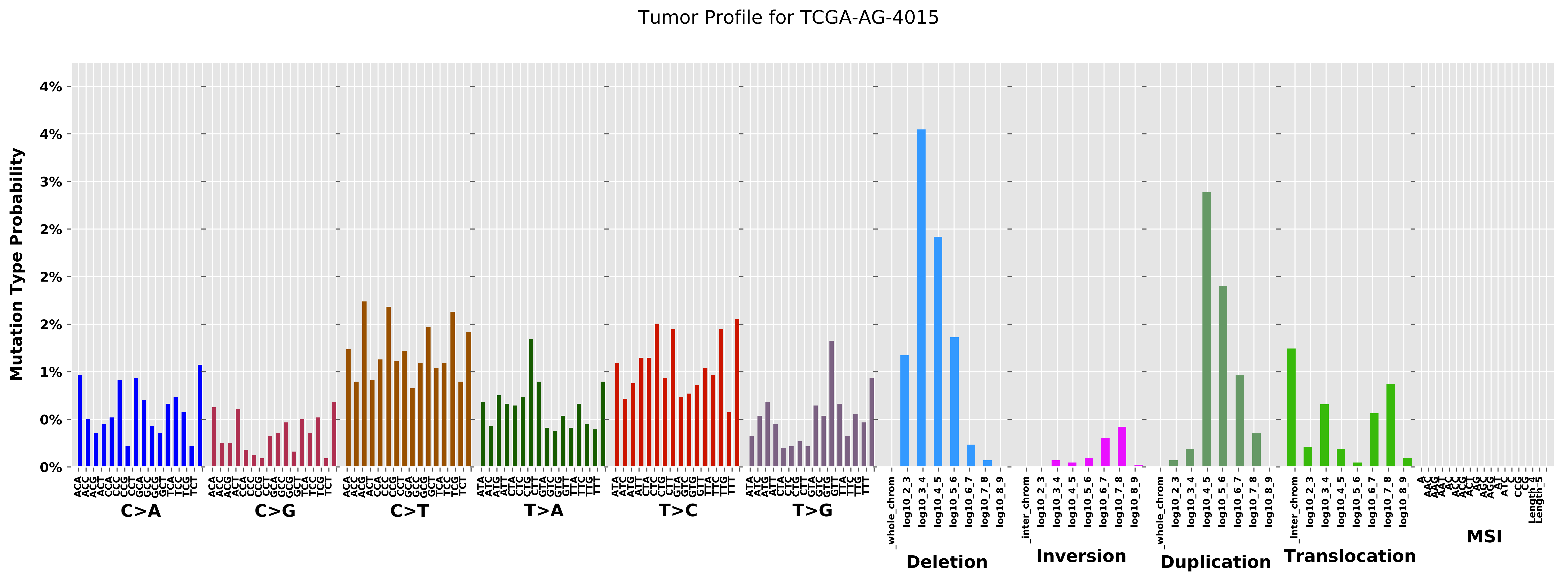
Tumor Profile for TCGA-AG-4015
Mutation Type Probability
C>A
C>G
C>T
T>A
T>C
T>G
Deletion
Inversion
Duplication
Translocation
MSI

Cancer processes Weights for TCGA-BH-A0B9

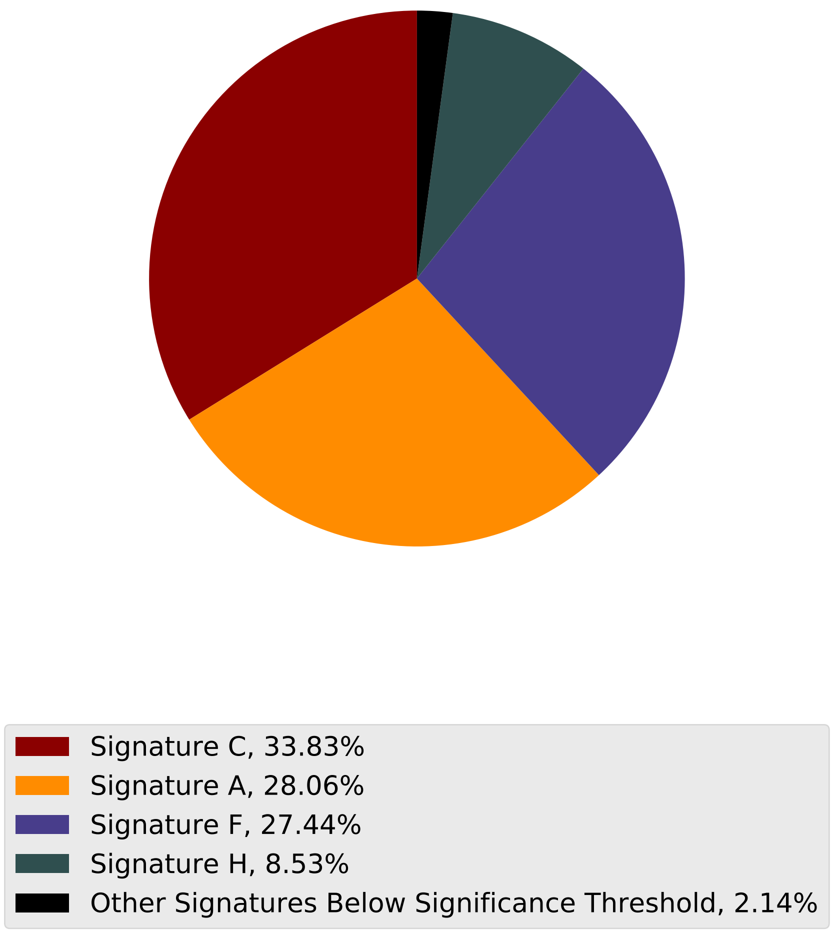

Tumor Profile for TCGA-BH-A0B9

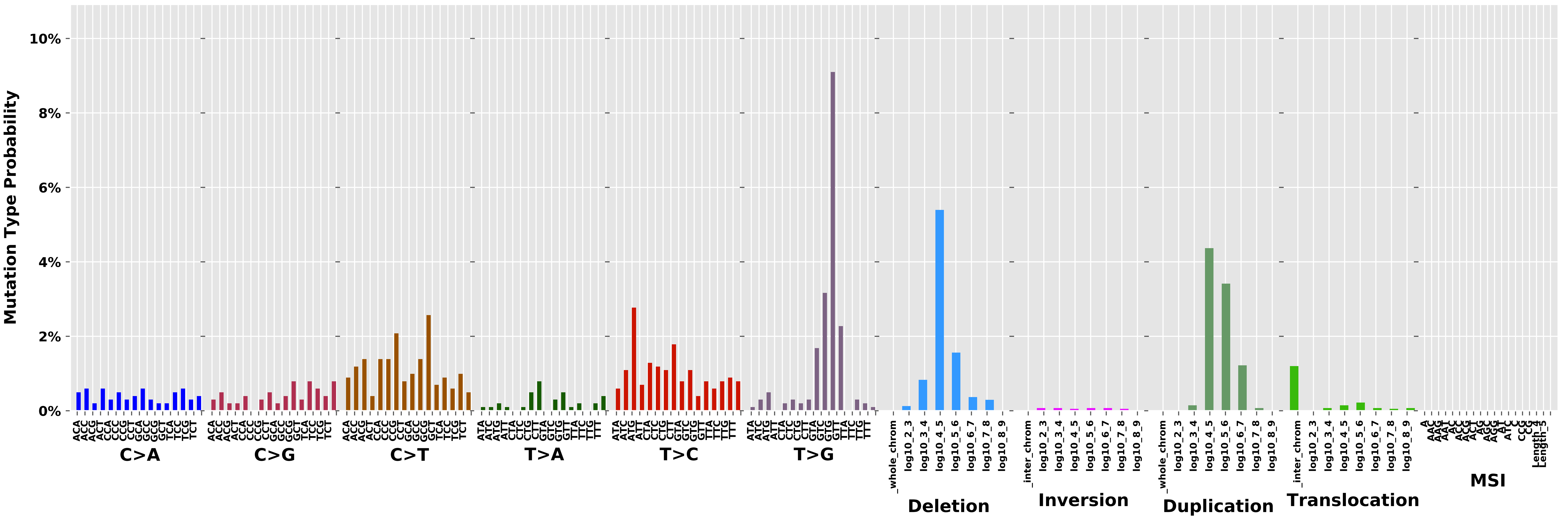

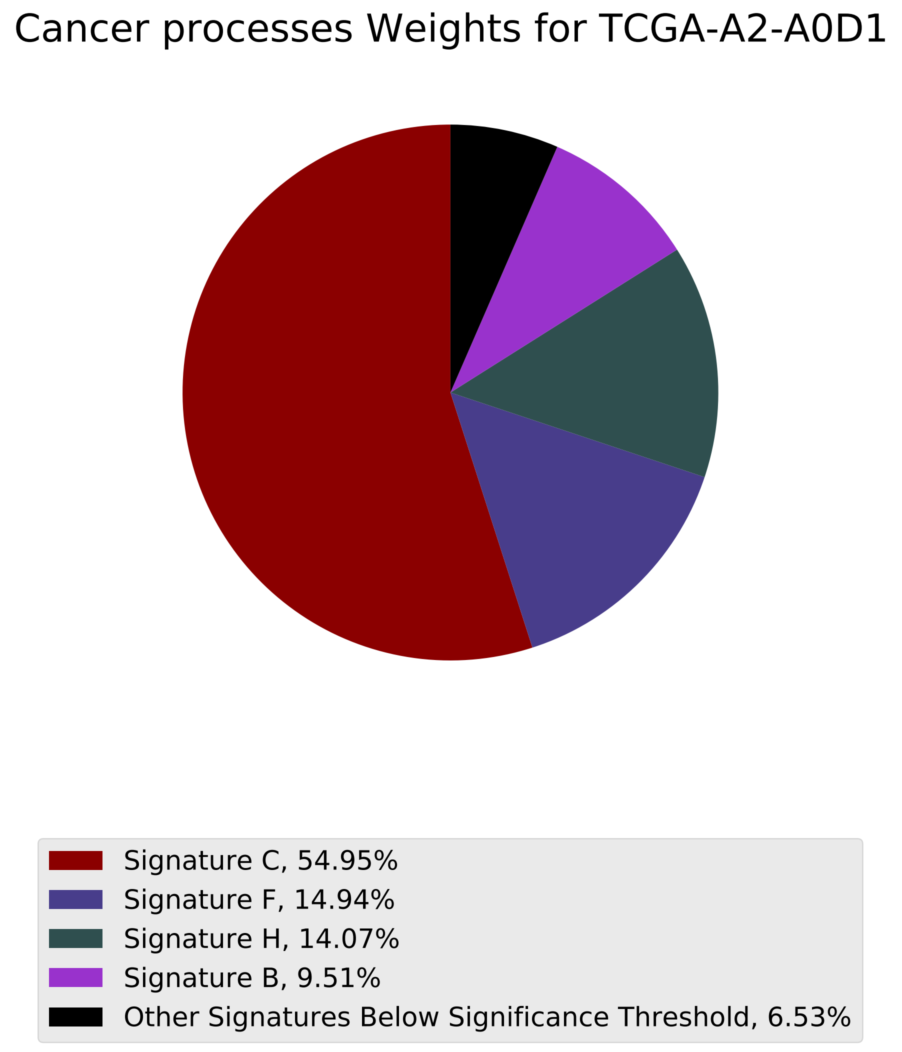
Cancer processes Weights for TCGA-A2-A0D1
Signature C, 54.95%
Signature F, 14.94%
Signature H, 14.07%
Signature B, 9.51%
Other Signatures Below Significance Threshold, 6.53%

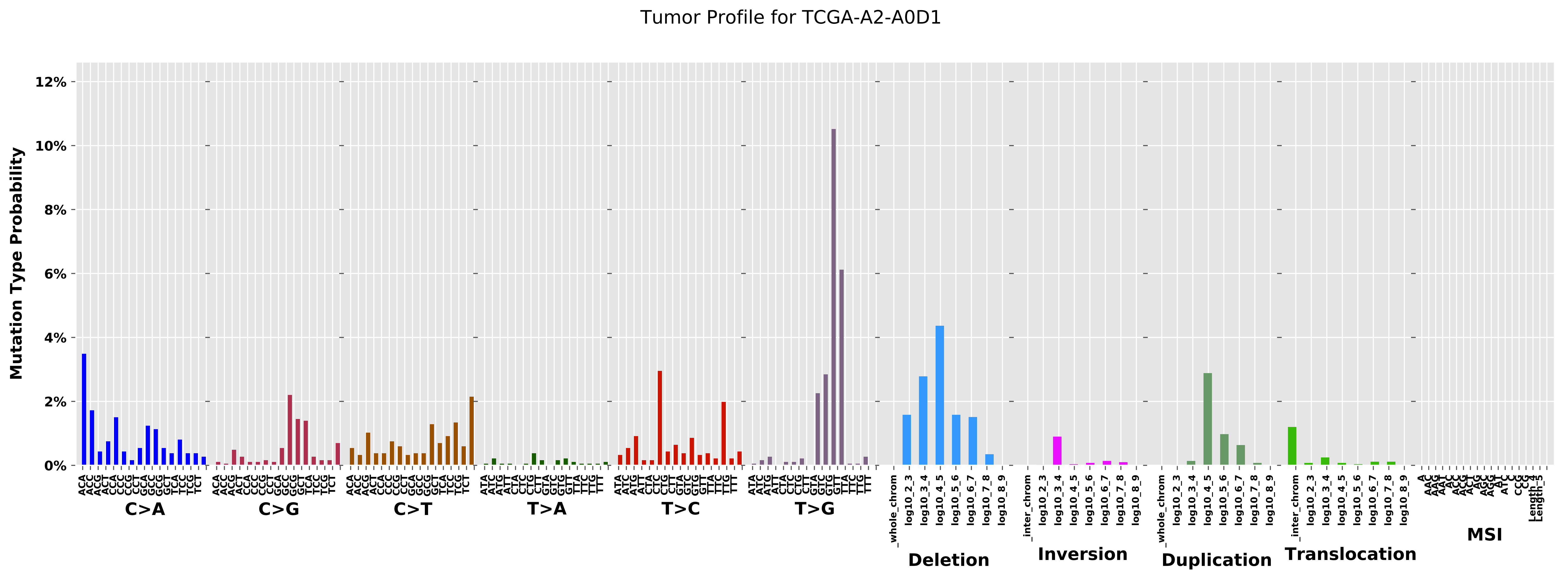

Tumor Profile for TCGA-A2-A0D1
Mutation Type Probability
0%
2%
4%
6%
8%
10%
12%
C>A
C>G
C>T
T>A
T>C
T>G
Deletion
Inversion
Duplication
Translocation
MSI
_whole_chrom
_inter_chrom
log10_2_3
log10_3_4
log10_4_5
log10_5_6
log10_6_7
log10_7_8
log10_8_9

Cancer processes Weights for TCGA-AA-3529

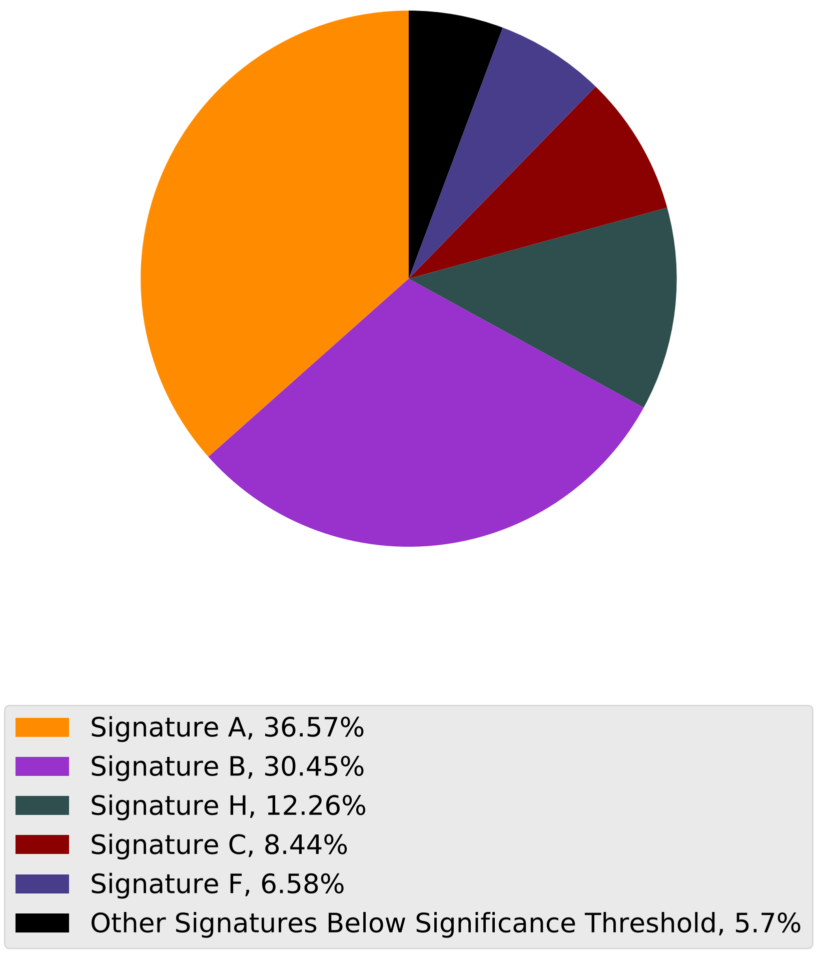

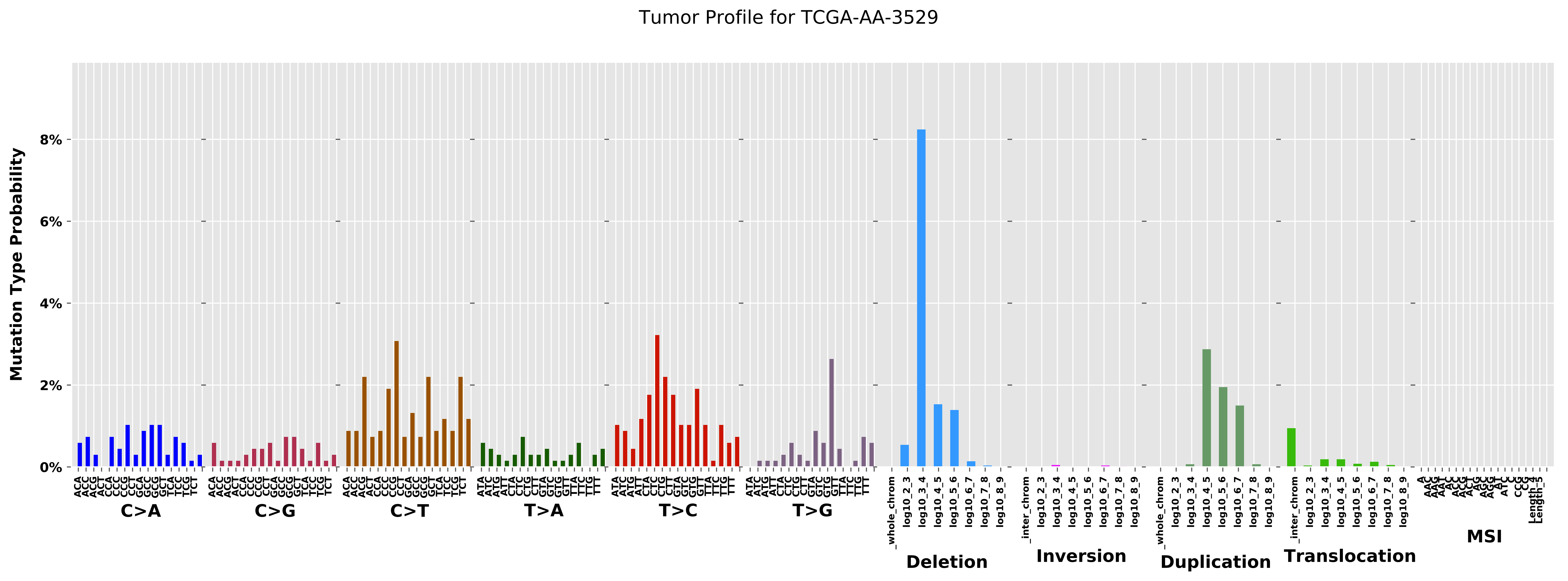
Tumor Profile for TCGA-AA-3529
Mutation Type Probability
8%
6%
4%
2%
0%
C>A
C>G
C>T
T>A
T>C
T>G
Deletion
Inversion
Duplication
Translocation
MSI

Cancer processes Weights for TCGA-A6-3807

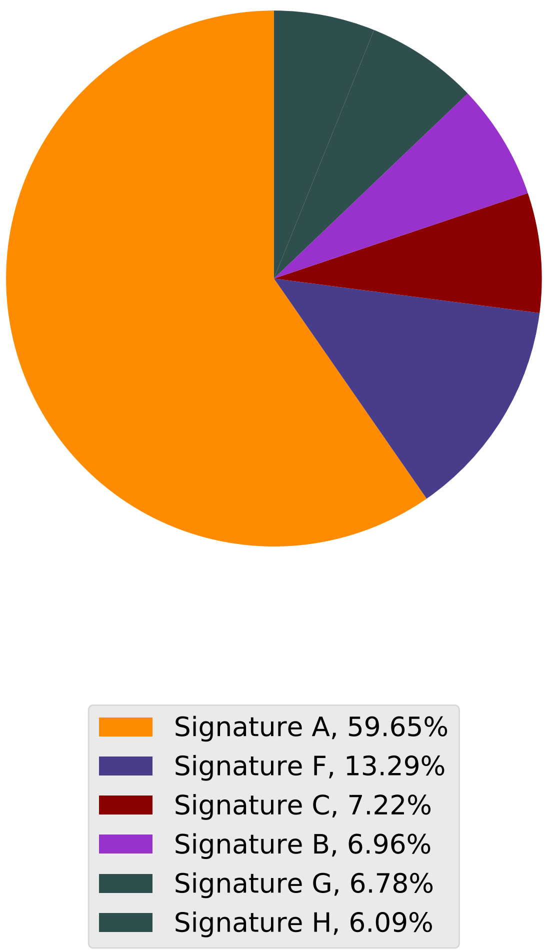

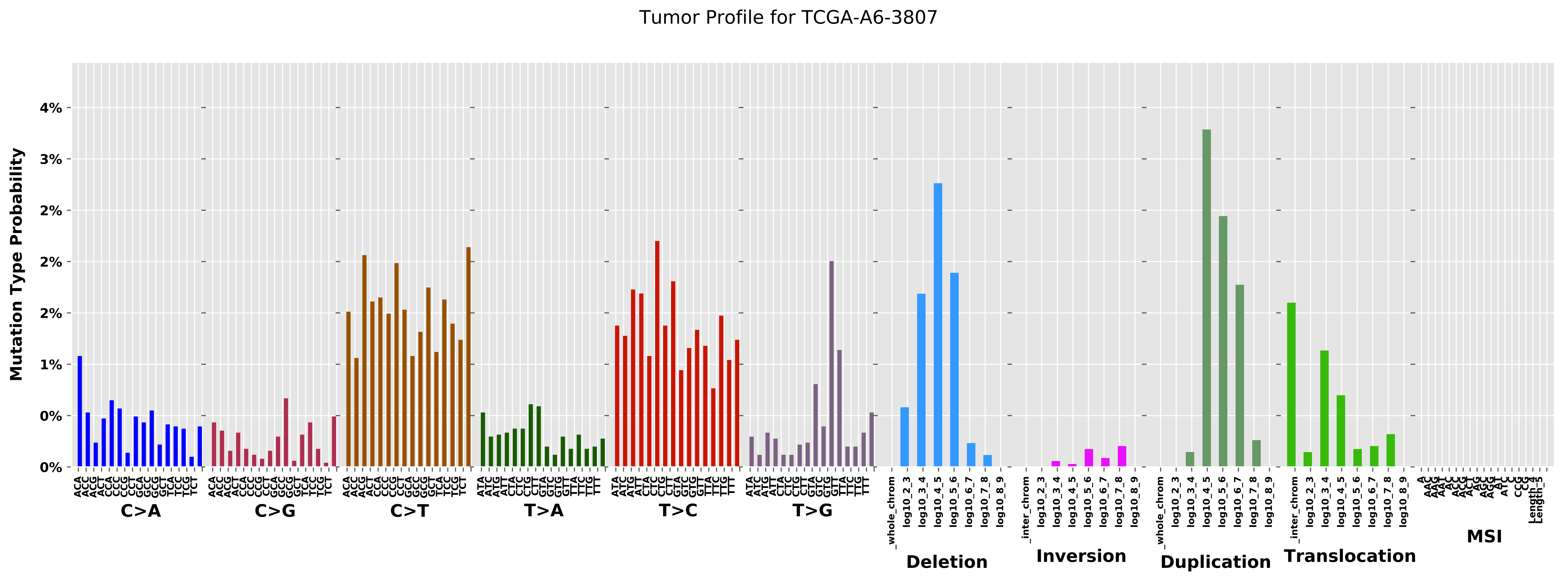

Tumor Profile for TCGA-A6-3807
Mutation Type Probability
4%
3%
2%
2%
2%
1%
0%
0%
C>A
C>G
C>T
T>A
T>C
T>G
Deletion
Inversion
Duplication
Translocation
MSI
_whole_chrom
_inter_chrom
log10_2_3
log10_3_4
log10_4_5
log10_5_6
log10_6_7
log10_7_8
log10_8_9

Cancer processes Weights for TCGA-A2-A0YG

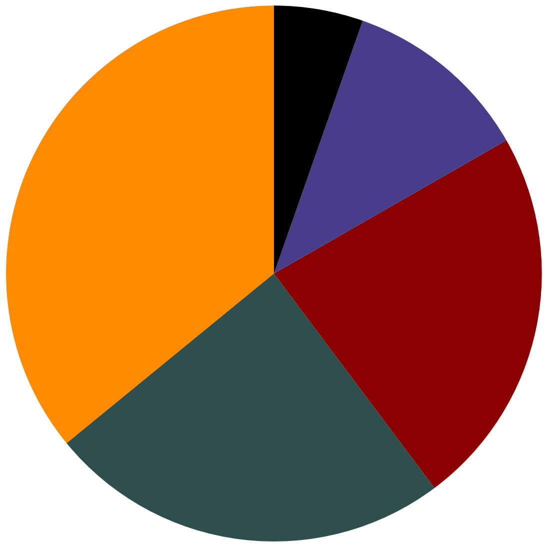

	Signature A, 35.91%
	Signature H, 24.32%
	Signature C, 23.05%
	Signature F, 11.34%
	Other Signatures Below Significance Threshold, 5.38%

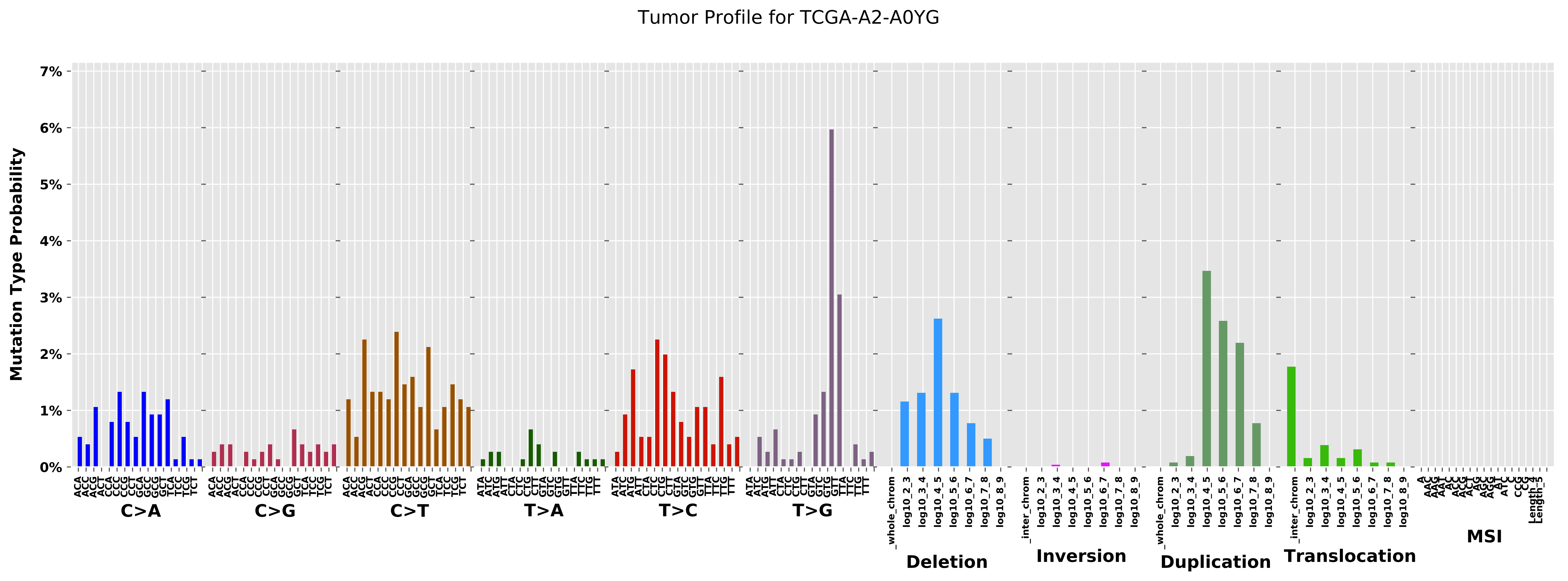

Tumor Profile for TCGA-A2-A0YG
Mutation Type Probability
7%
6%
5%
4%
3%
2%
1%
0%
C>A
C>G
C>T
T>A
T>C
T>G
Deletion
Inversion
Duplication
Translocation
MSI

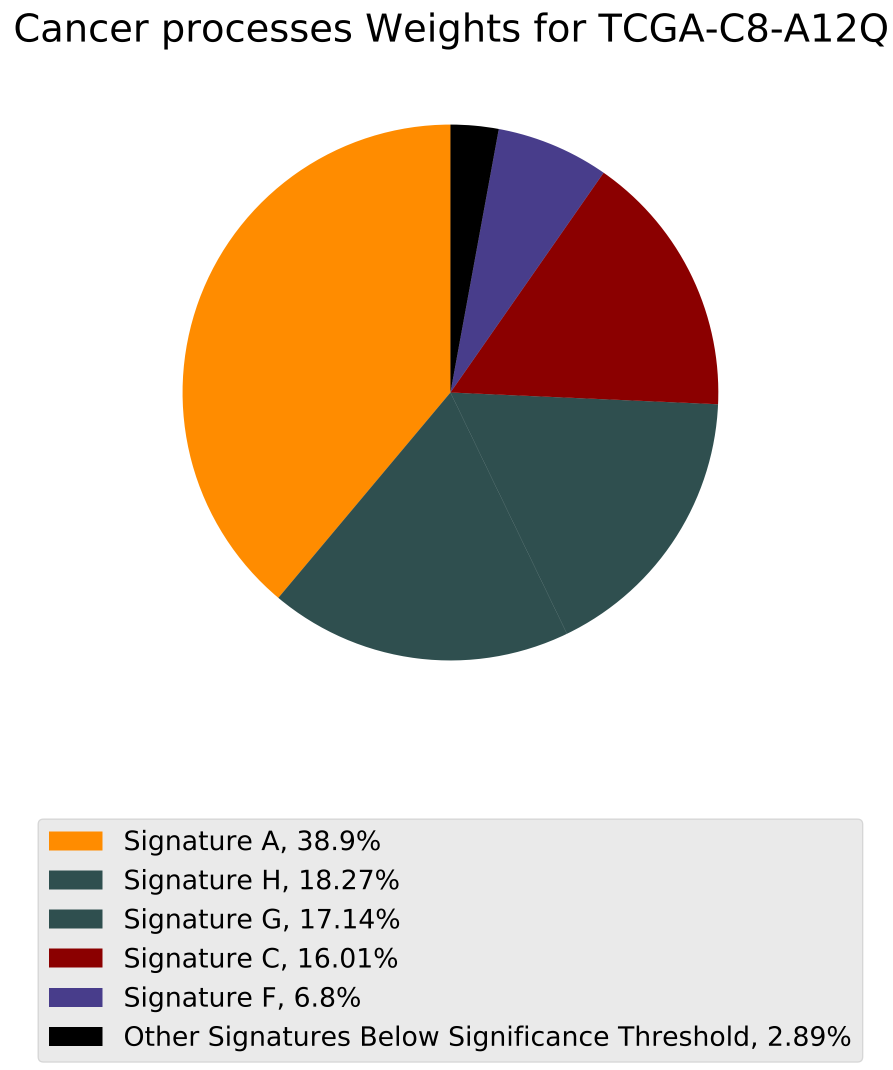

Cancer processes Weights for TCGA-C8-A12Q
Signature A, 38.9%
Signature H, 18.27%
Signature G, 17.14%
Signature C, 16.01%
Signature F, 6.8%
Other Signatures Below Significance Threshold, 2.89%

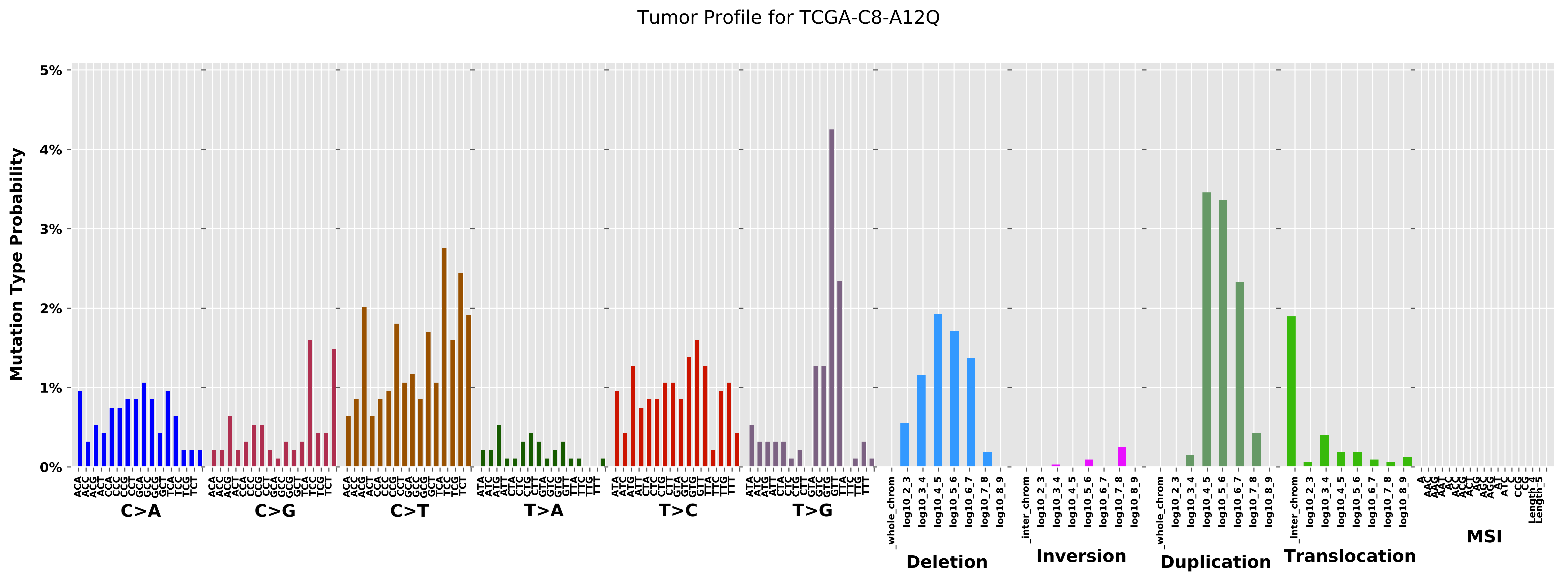
Tumor Profile for TCGA-C8-A12Q
Mutation Type Probability
0%
1%
2%
3%
4%
5%
C>A
C>G
C>T
T>A
T>C
T>G
Deletion
Inversion
Duplication
Translocation
MSI

Cancer processes Weights for TCGA-BH-A0BW

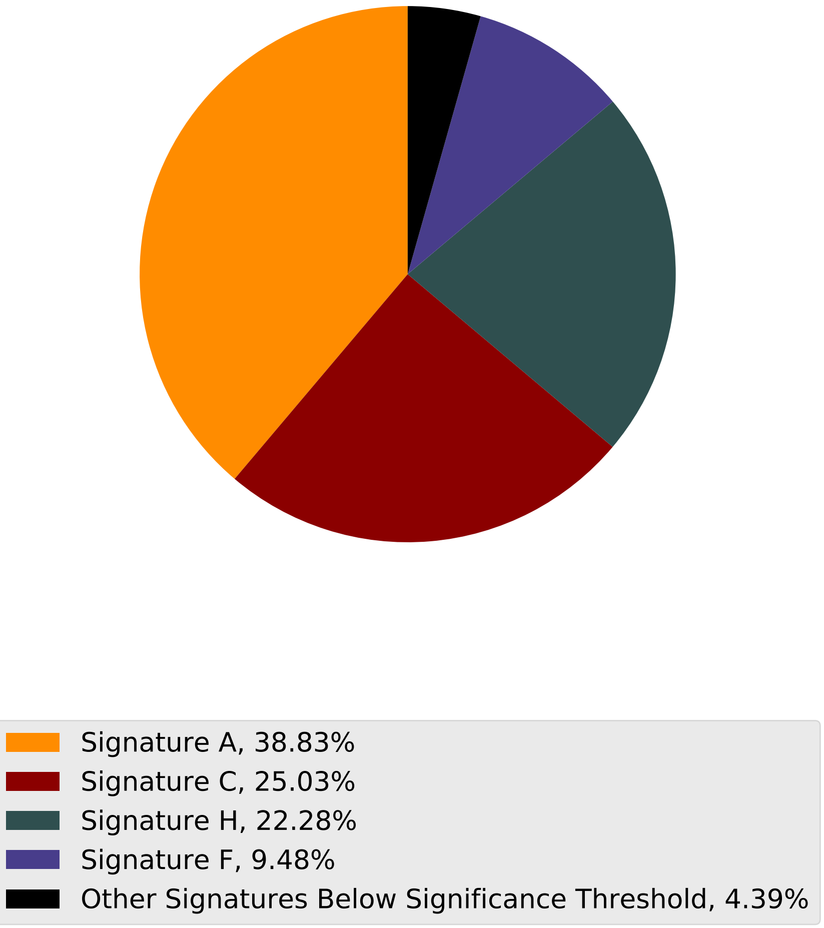

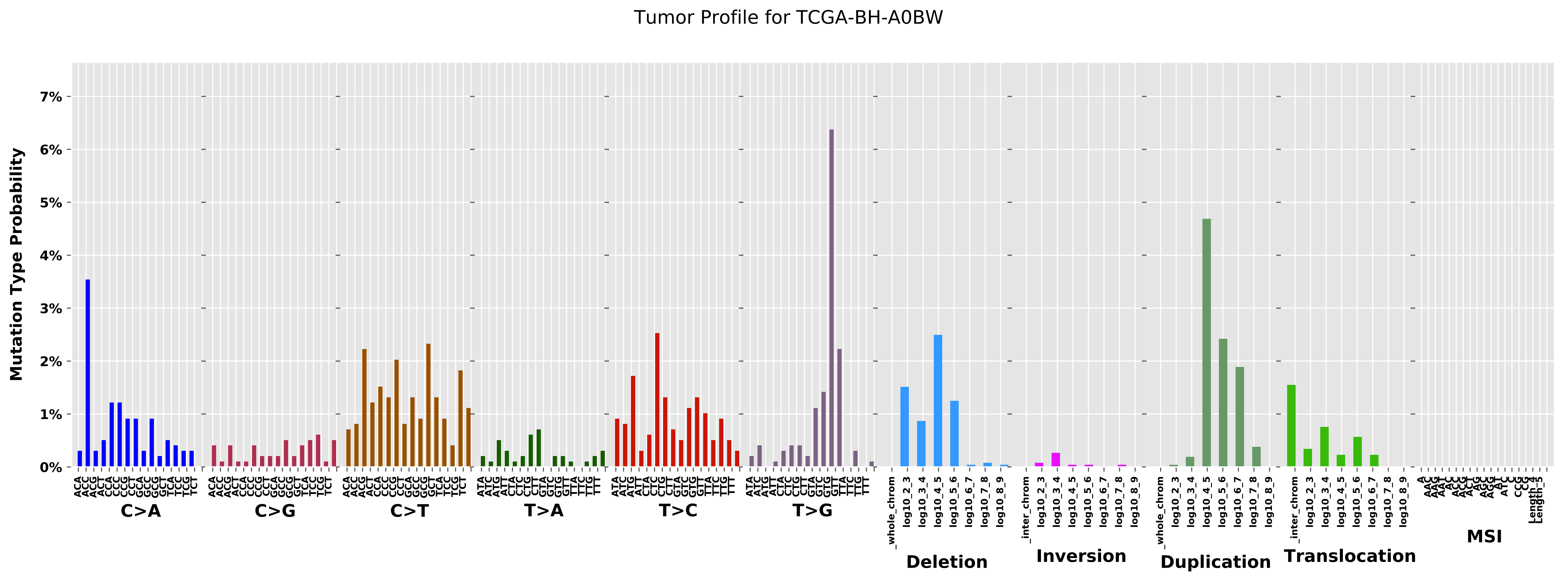

Tumor Profile for TCGA-BH-A0BW
Mutation Type Probability
7%
6%
5%
4%
3%
2%
1%
0%
C>A
C>G
C>T
T>A
T>C
T>G
Deletion
Inversion
Duplication
Translocation
MSI

Cancer processes Weights for TCGA-A2-A0EY

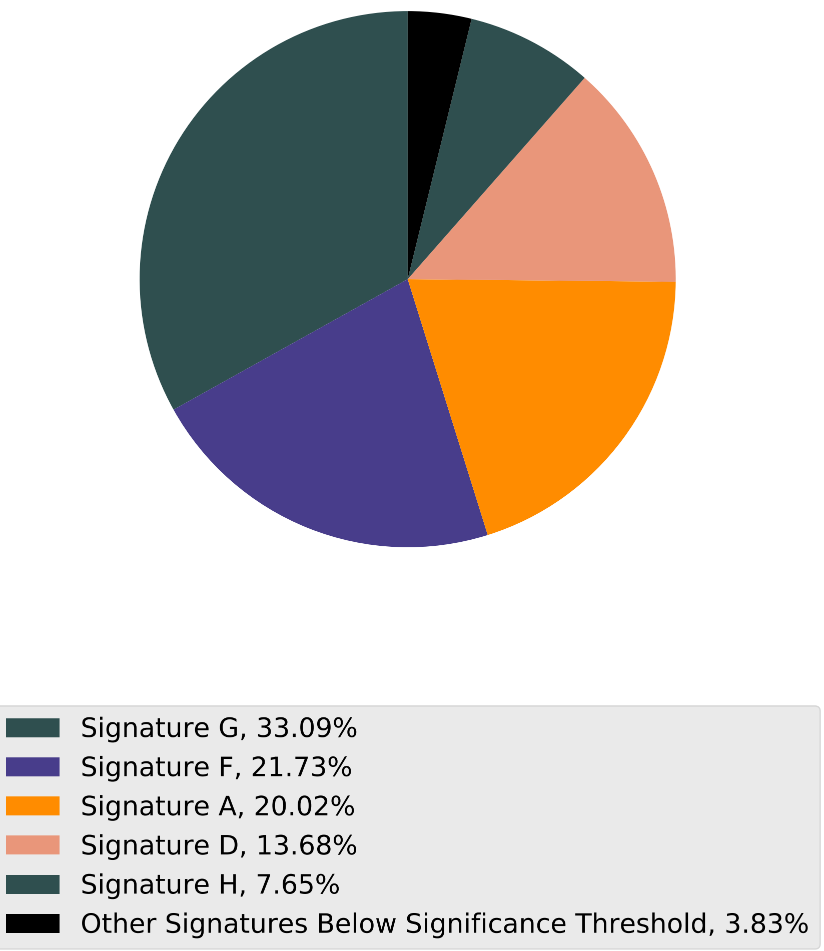

Tumor Profile for TCGA-A2-A0EY

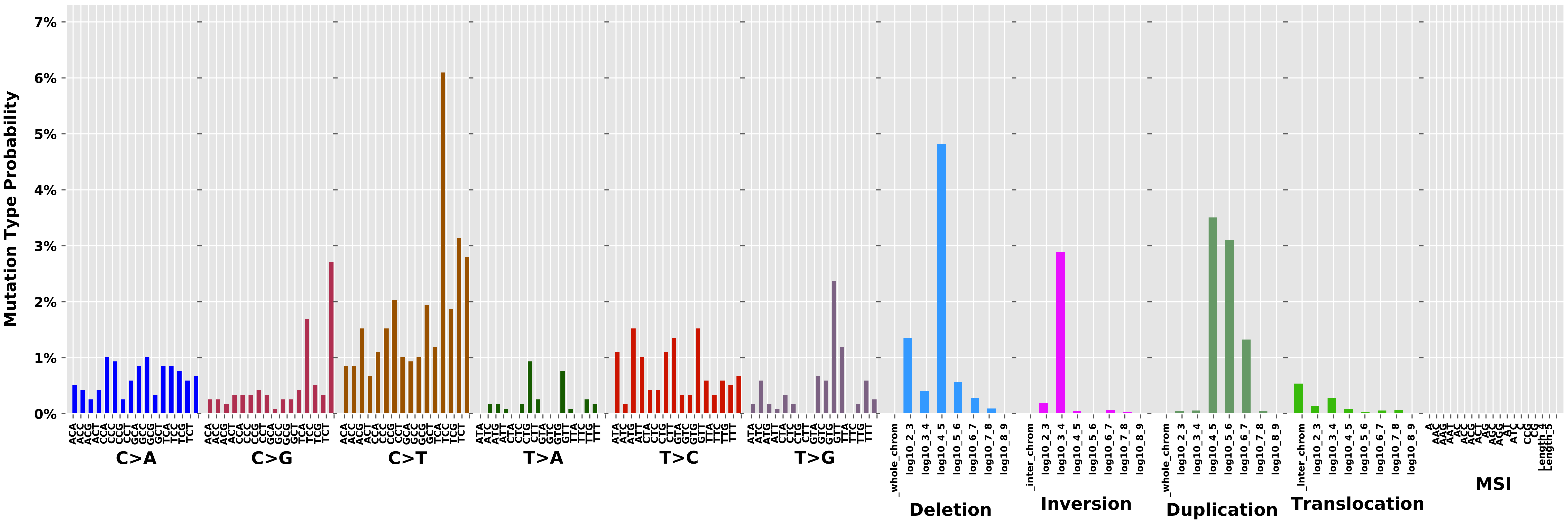

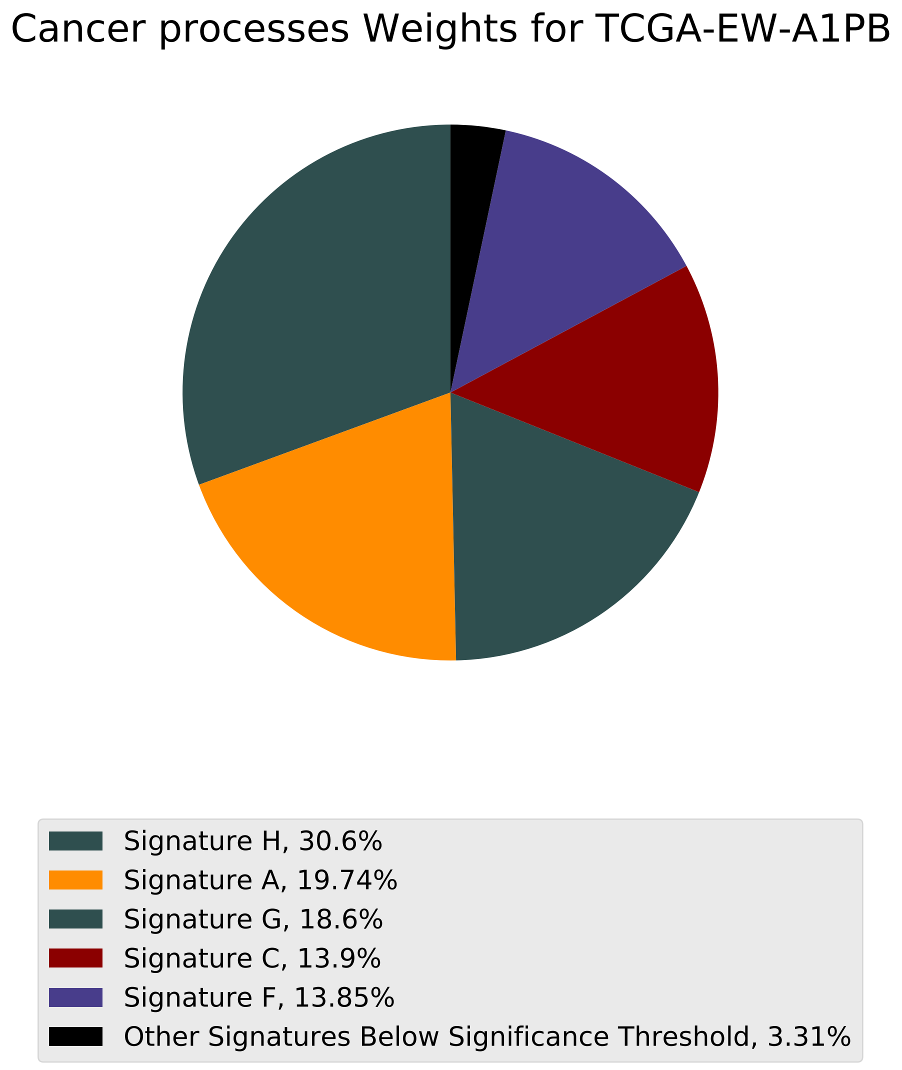
Cancer processes Weights for TCGA-EW-A1PB
Signature H, 30.6%
Signature A, 19.74%
Signature G, 18.6%
Signature C, 13.9%
Signature F, 13.85%
Other Signatures Below Significance Threshold, 3.31%

Tumor Profile for TCGA-EW-A1PB

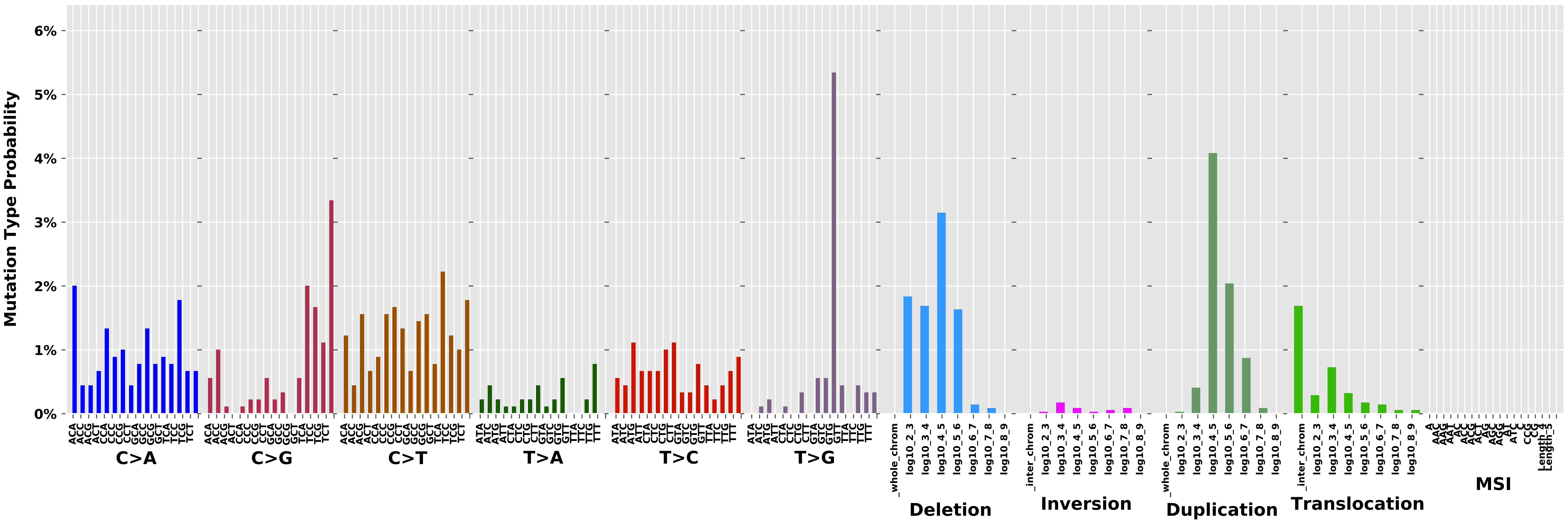

Cancer processes Weights for TCGA-AD-6964

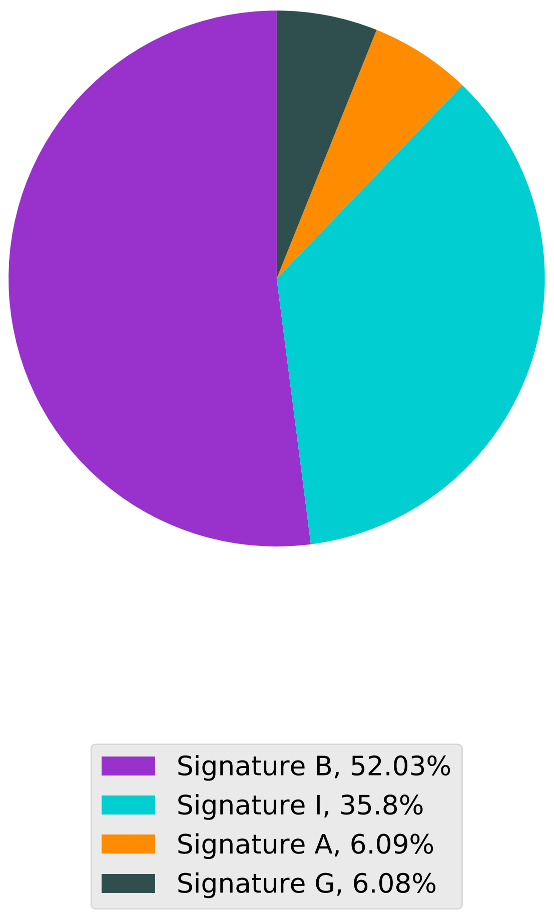

Tumor Profile for TCGA-AD-6964

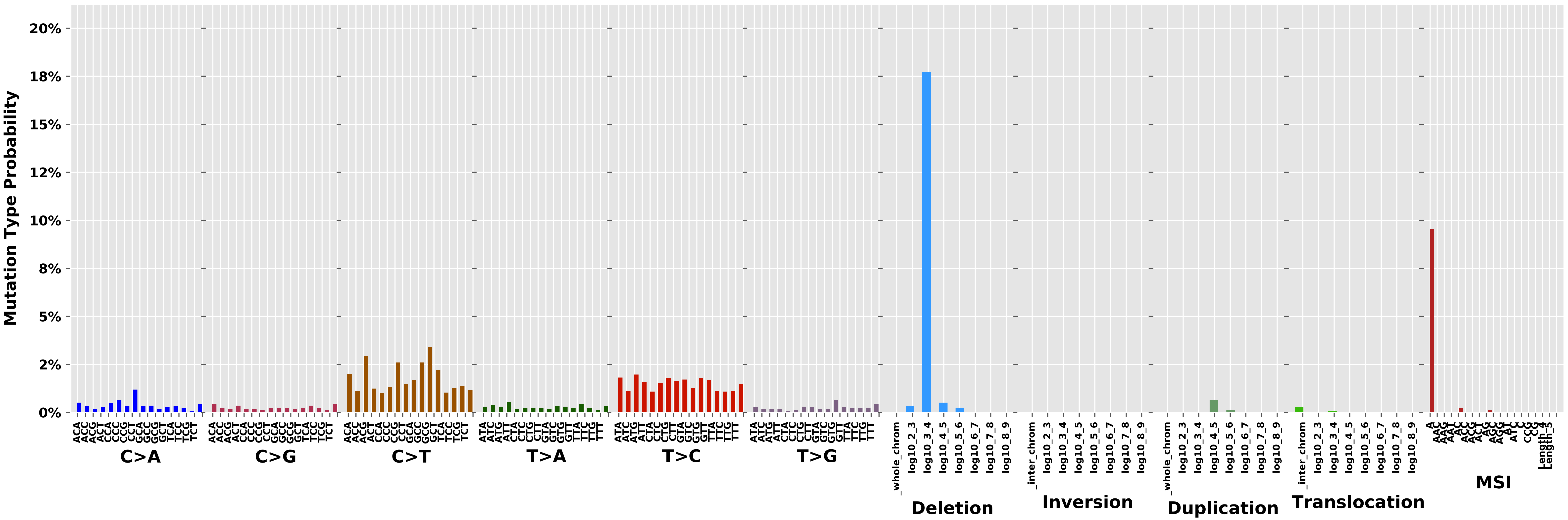

Cancer processes Weights for TCGA-AG-3896

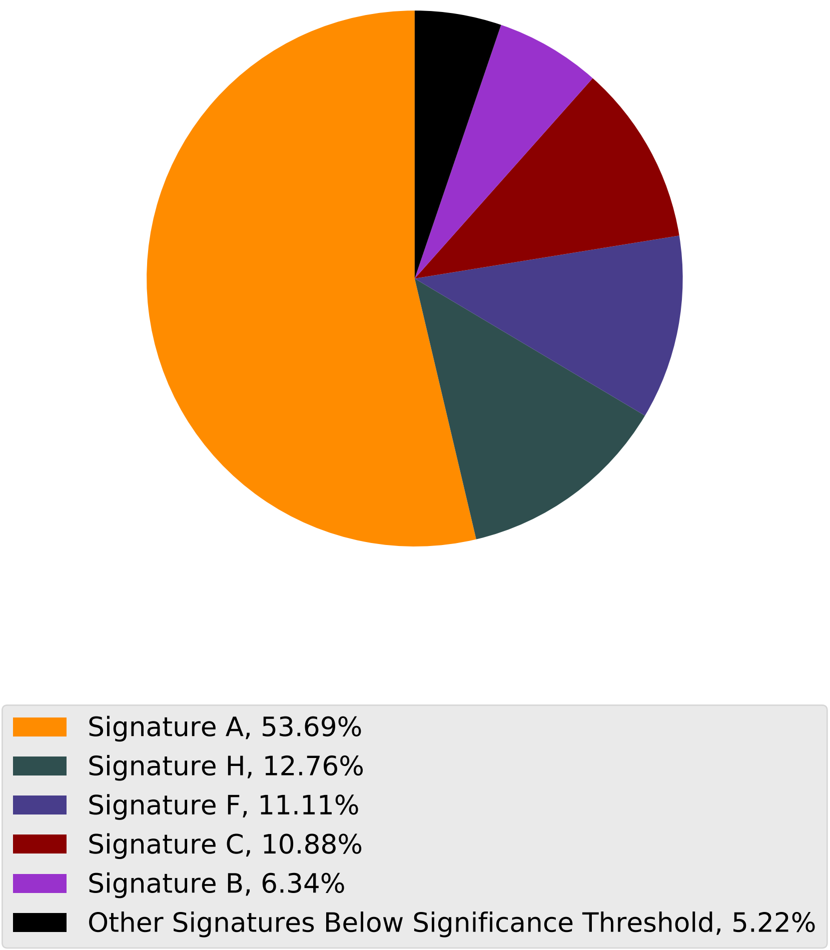

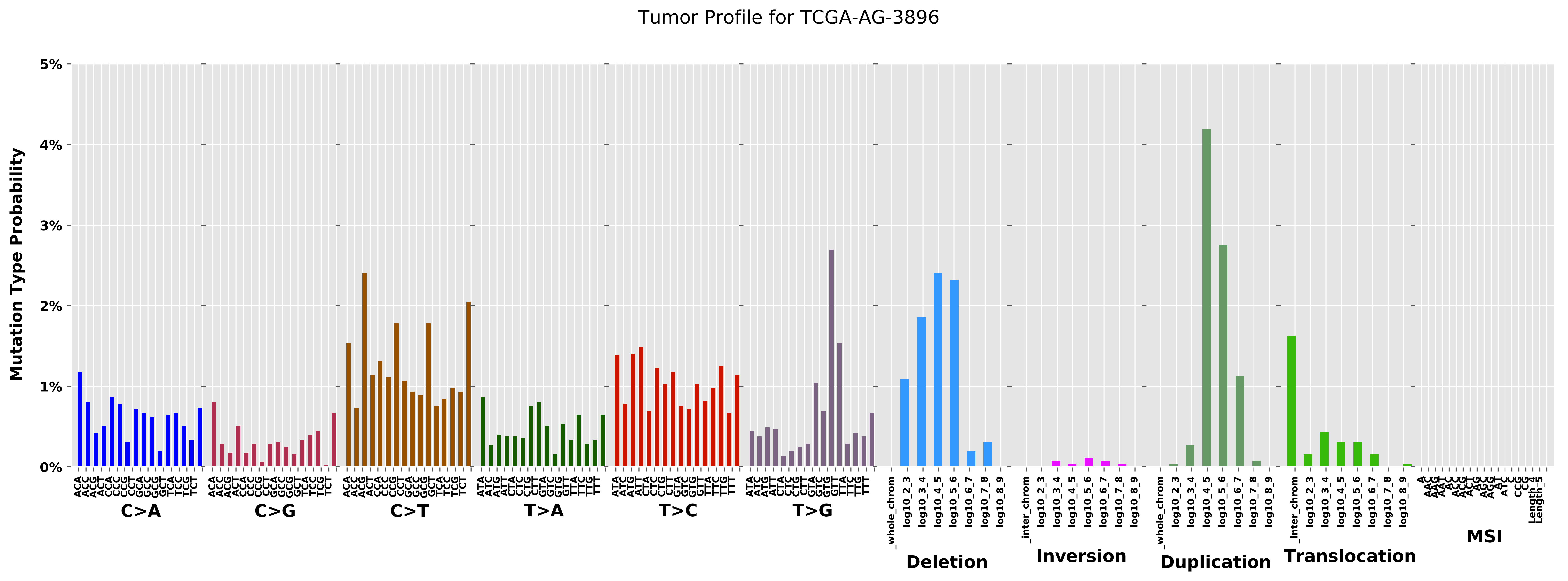

Tumor Profile for TCGA-AG-3896
Mutation Type Probability
0%
1%
2%
3%
4%
5%
C>A
C>G
C>T
T>A
T>C
T>G
Deletion
Inversion
Duplication
Translocation
MSI

Cancer processes Weights for TCGA-E2-A15K

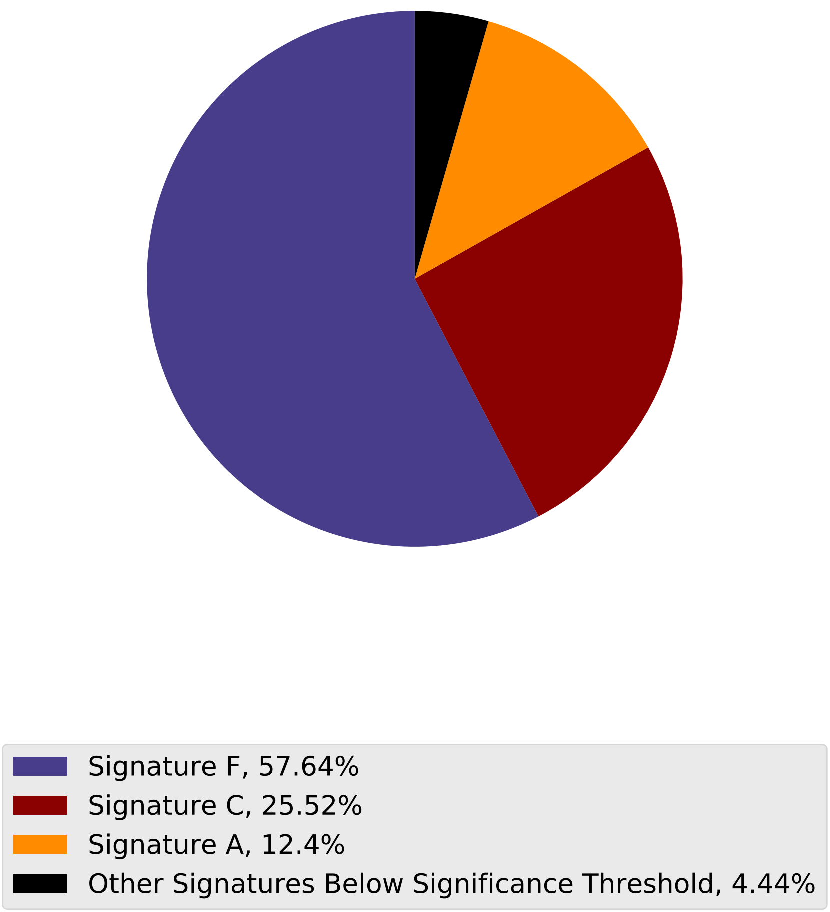

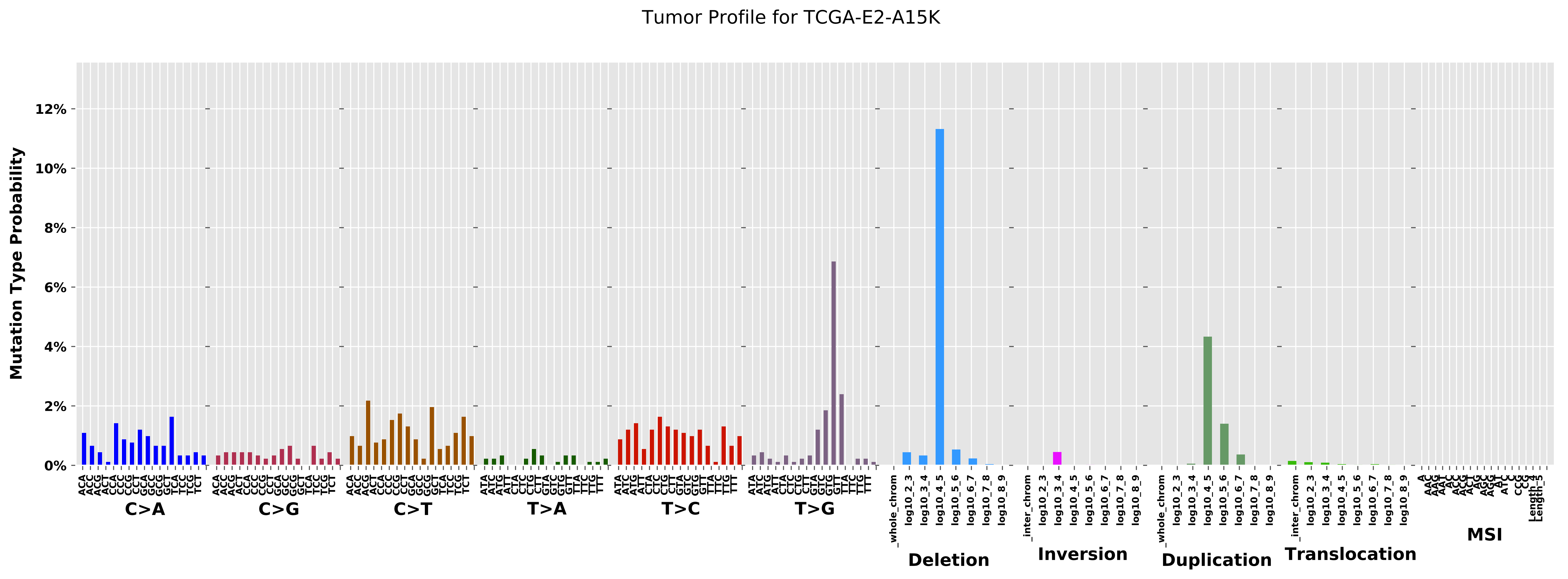

Tumor Profile for TCGA-E2-A15K
Mutation Type Probability
0%
2%
4%
6%
8%
10%
12%
C>A
C>G
C>T
T>A
T>C
T>G
Deletion
Inversion
Duplication
Translocation
MSI

Cancer processes Weights for TCGA-B6-A0RE

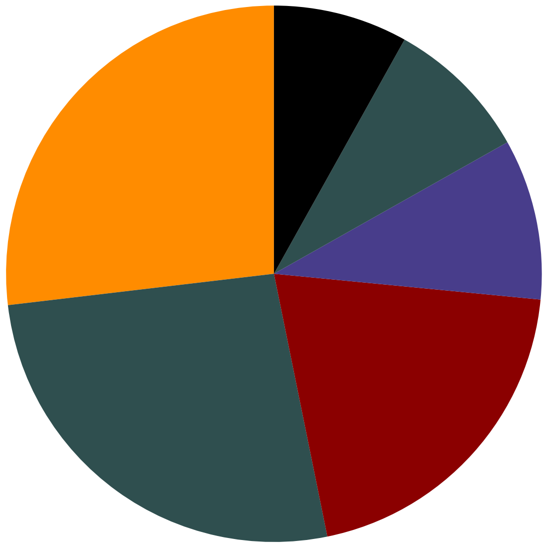

- Signature A, 26.87%
- Signature H, 26.33%
- Signature C, 20.27%
- Signature F, 9.69%
- Signature G, 8.74%
- Other Signatures Below Significance Threshold, 8.11%

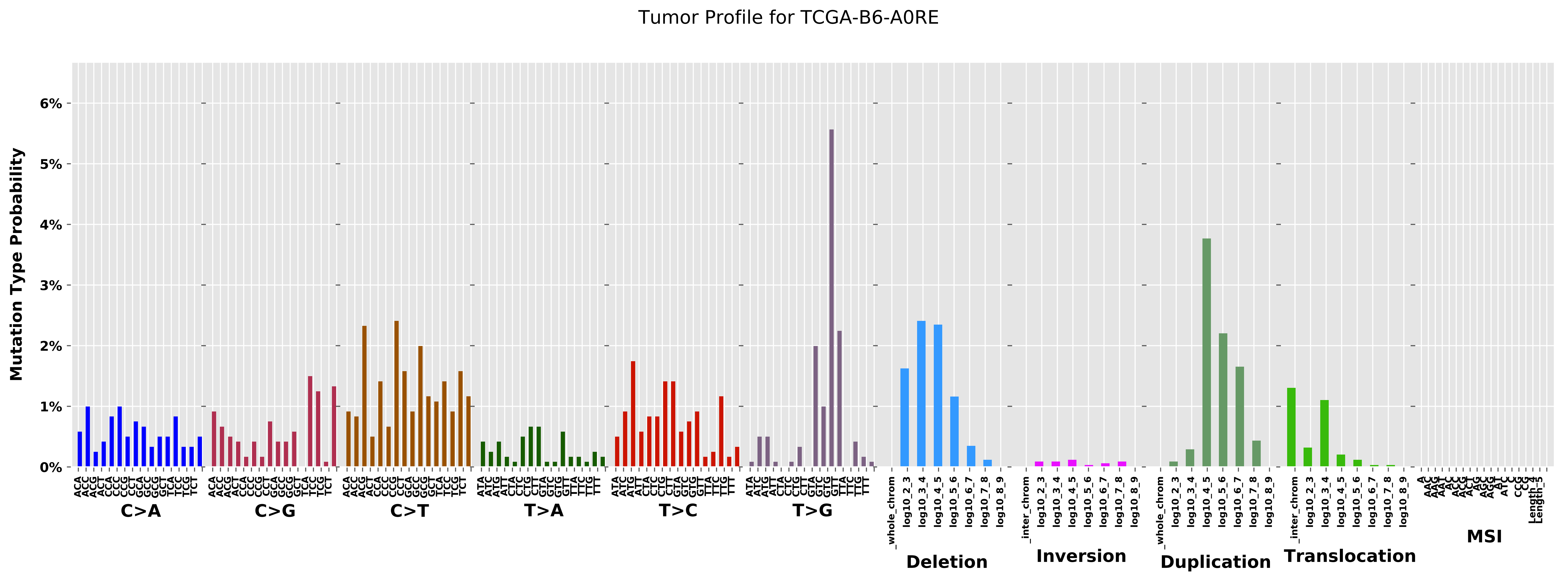

Tumor Profile for TCGA-B6-A0RE
Mutation Type Probability
0%
1%
2%
3%
4%
5%
6%
C>A
C>G
C>T
T>A
T>C
T>G
Deletion
Inversion
Duplication
Translocation
MSI

Cancer processes Weights for TCGA-A8-A08S

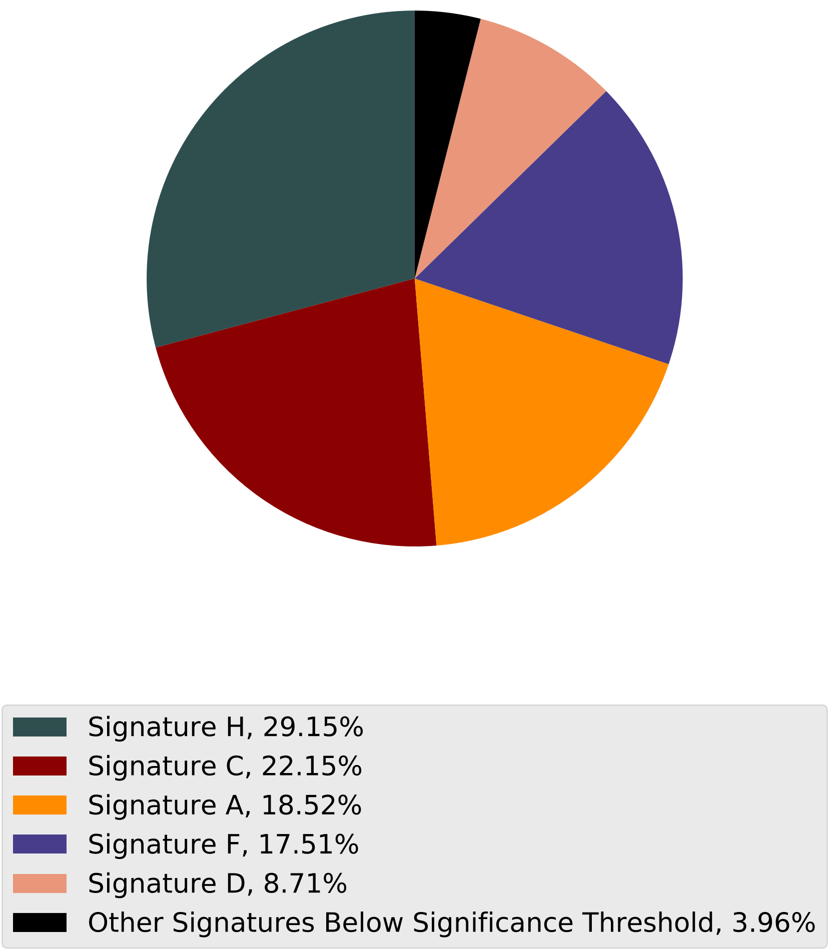

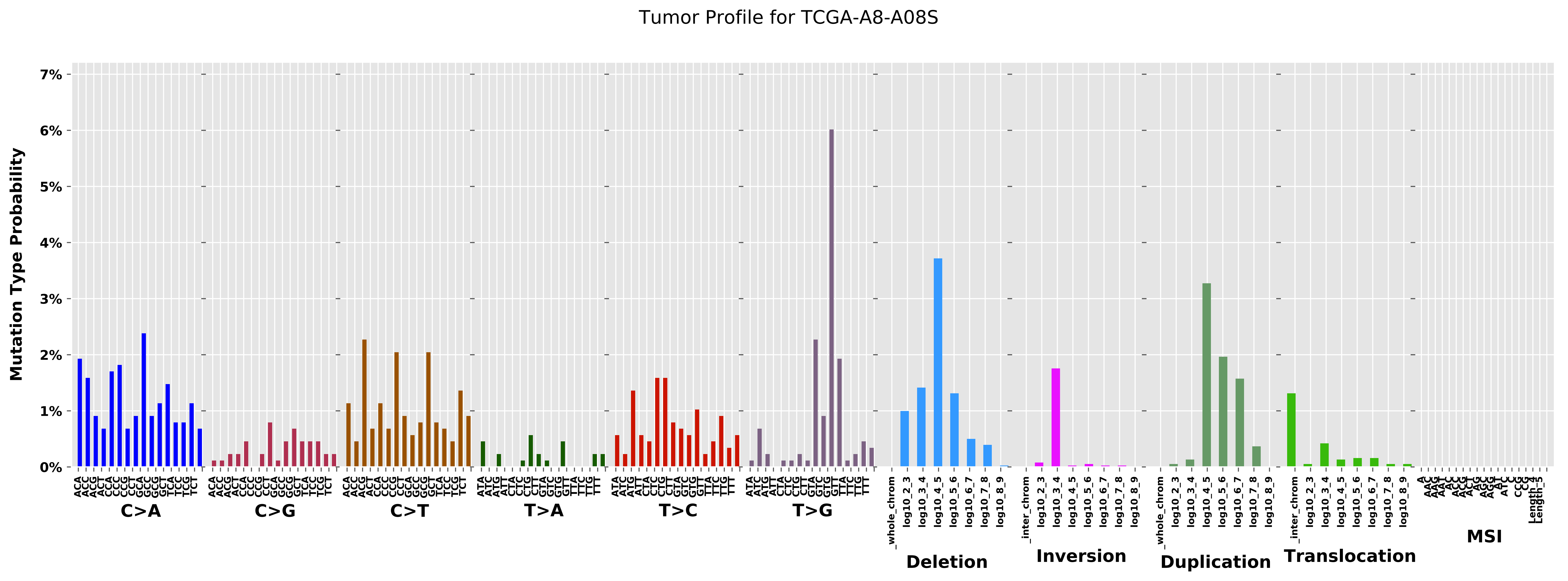
Tumor Profile for TCGA-A8-A08S
Mutation Type Probability
7%
6%
5%
4%
3%
2%
1%
0%
C>A
C>G
C>T
T>A
T>C
T>G
Deletion
Inversion
Duplication
Translocation
MSI

Cancer processes Weights for TCGA-AA-A03F

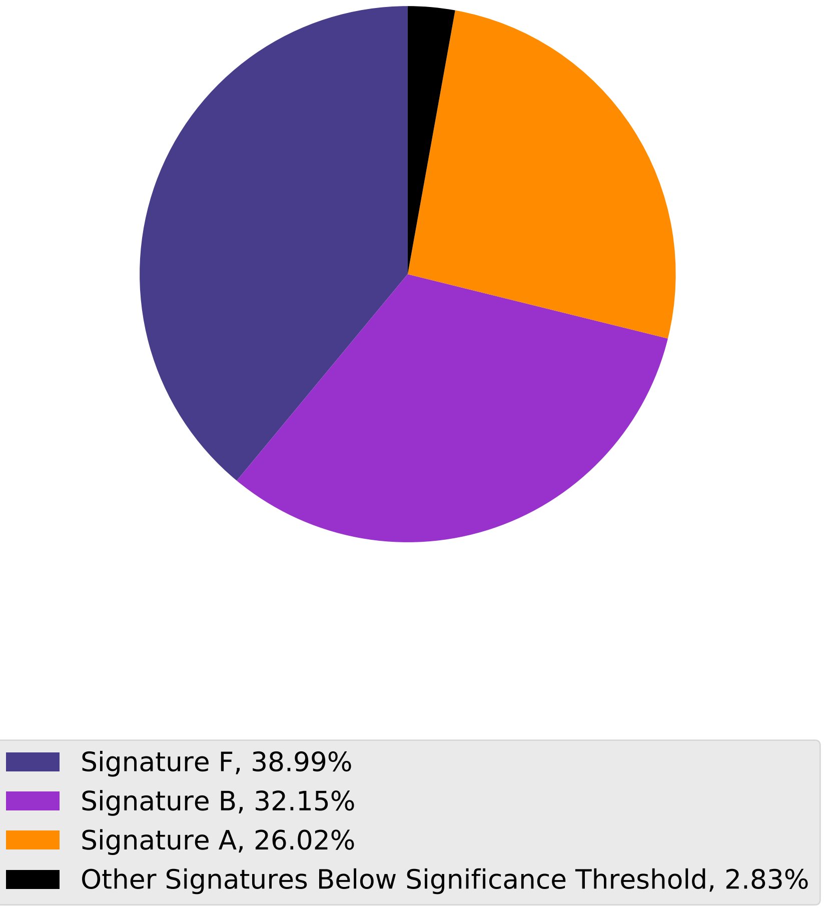

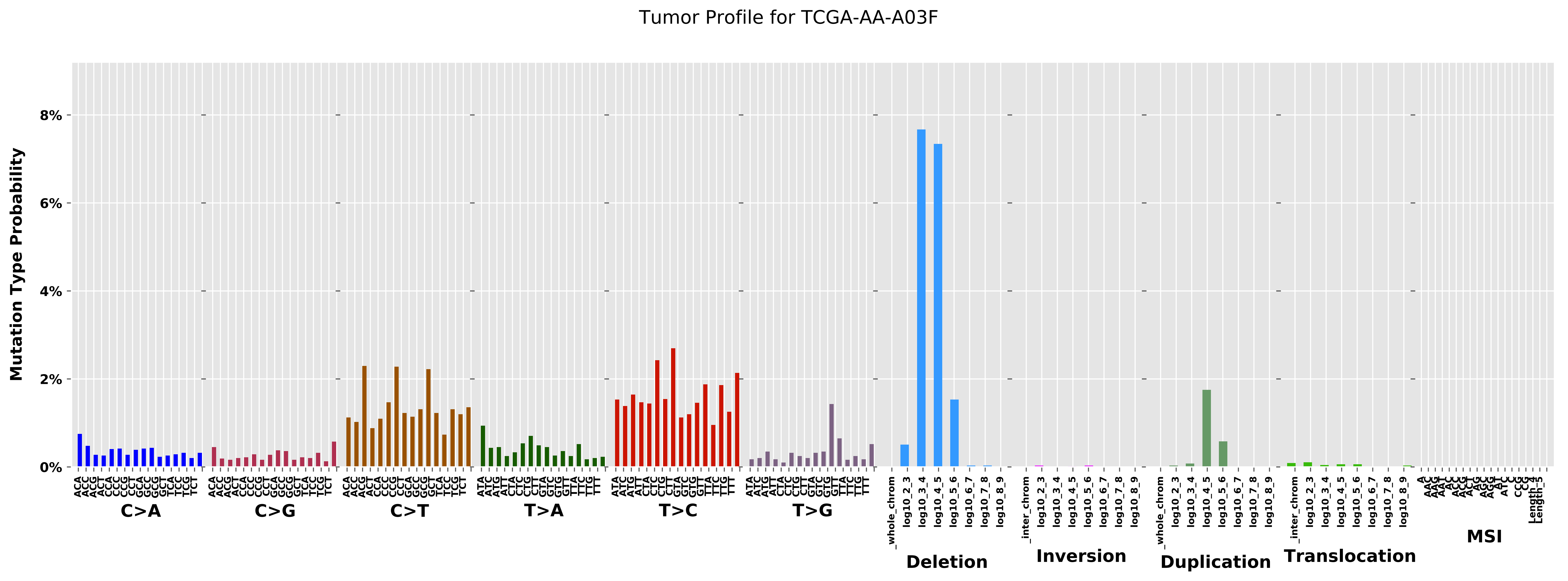

Tumor Profile for TCGA-AA-A03F
Mutation Type Probability
8%
6%
4%
2%
0%
C>A
C>G
C>T
T>A
T>C
T>G
Deletion
Inversion
Duplication
Translocation
MSI

Cancer processes Weights for TCGA-EW-A1PH

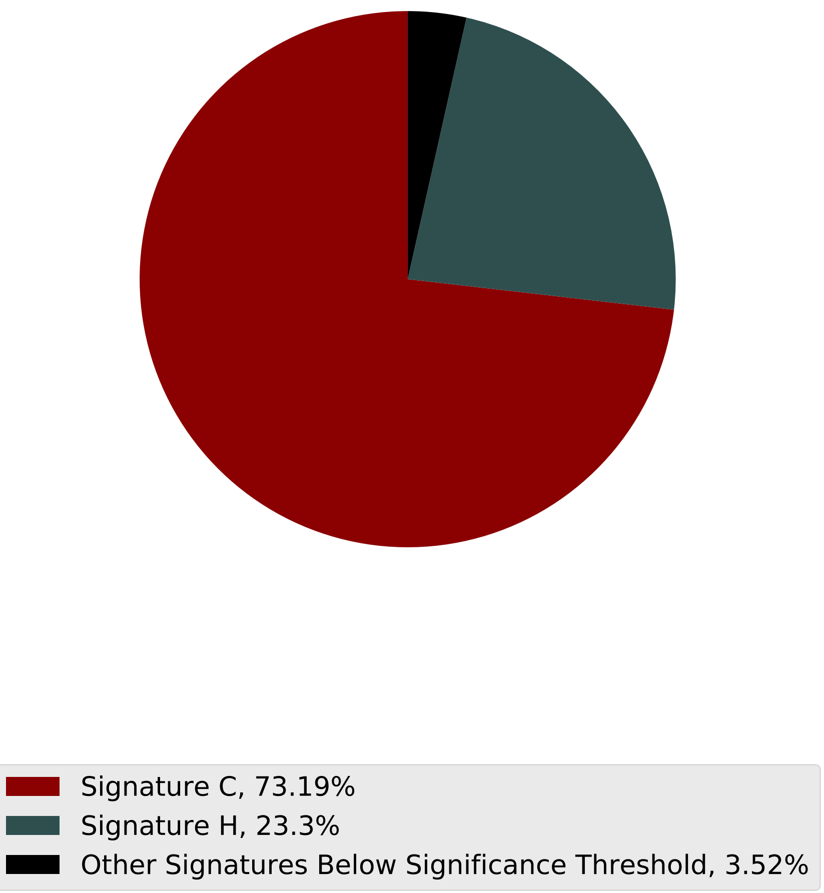

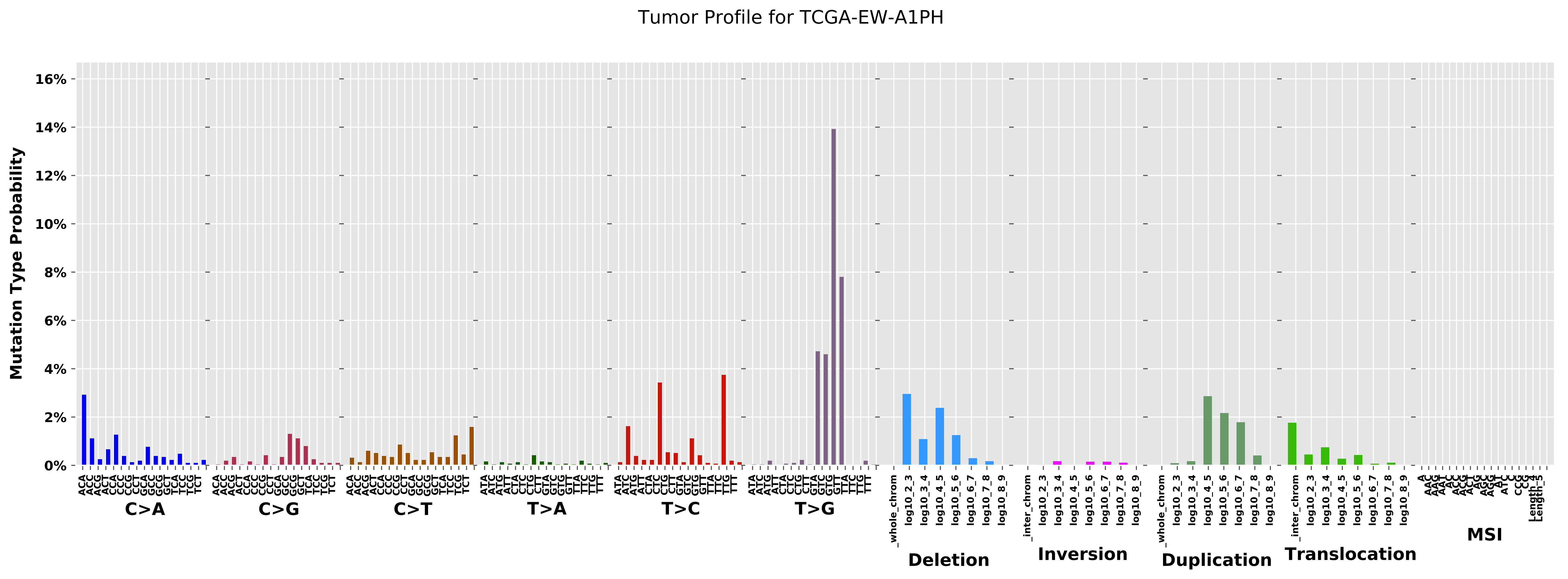
Tumor Profile for TCGA-EW-A1PH
Mutation Type Probability
0%
2%
4%
6%
8%
10%
12%
14%
16%
C>A
C>G
C>T
T>A
T>C
T>G
Deletion
Inversion
Duplication
Translocation
MSI

Cancer processes Weights for TCGA-E2-A1LG

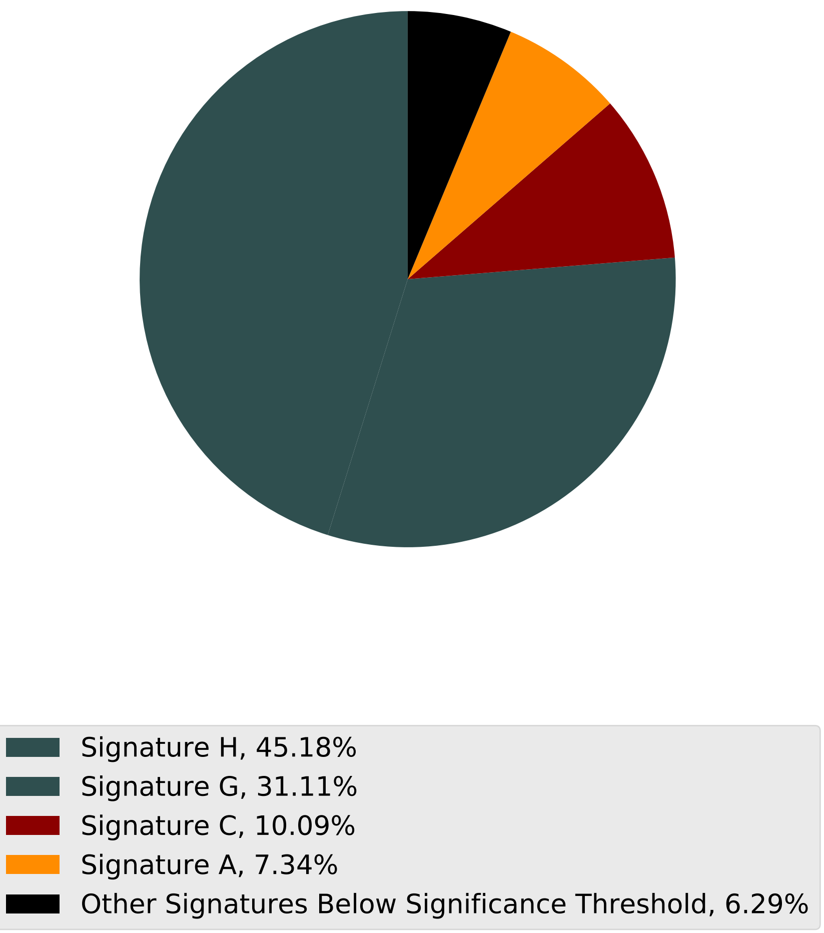

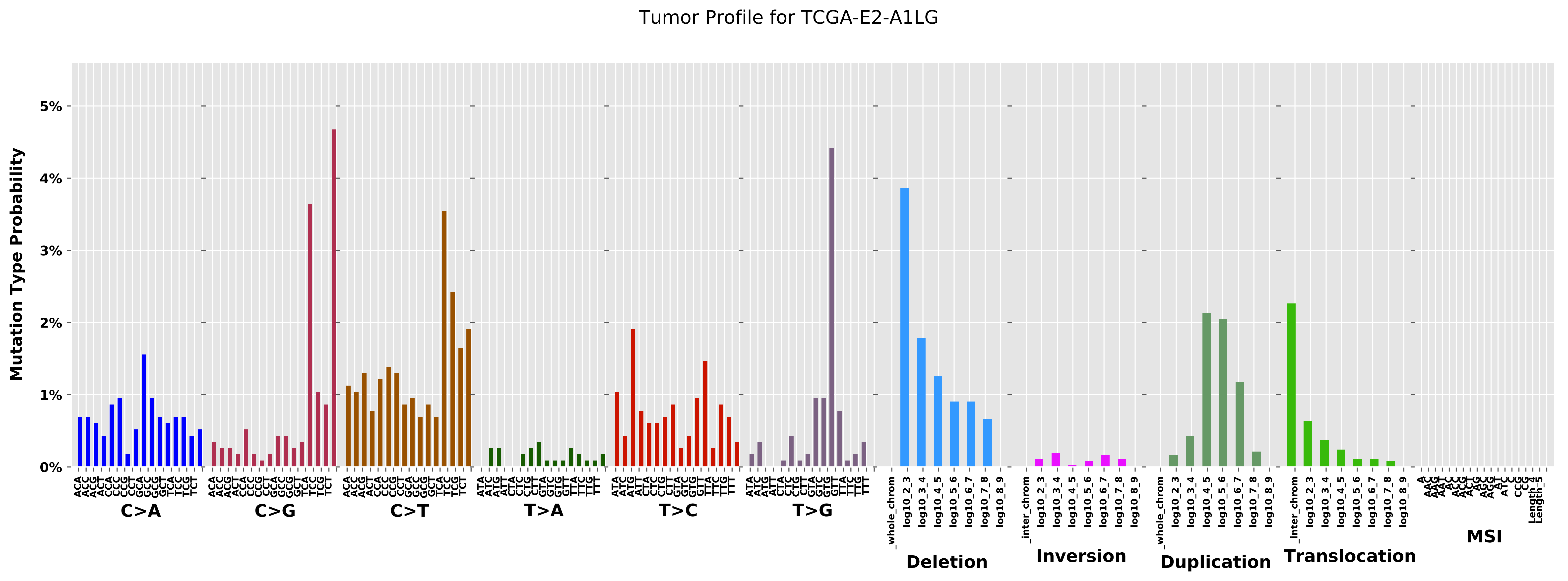
Tumor Profile for TCGA-E2-A1LG
Mutation Type Probability
0%
1%
2%
3%
4%
5%
C>A
C>G
C>T
T>A
T>C
T>G
Deletion
Inversion
Duplication
Translocation
MSI

Cancer processes Weights for TCGA-A8-A08B

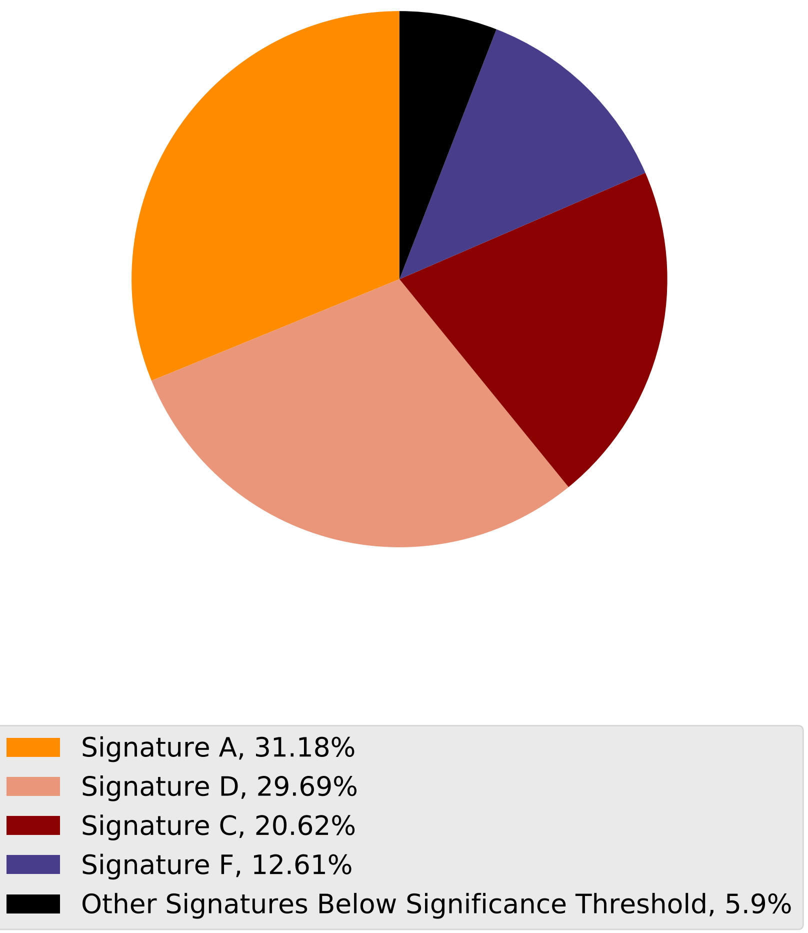

Tumor Profile for TCGA-A8-A08B

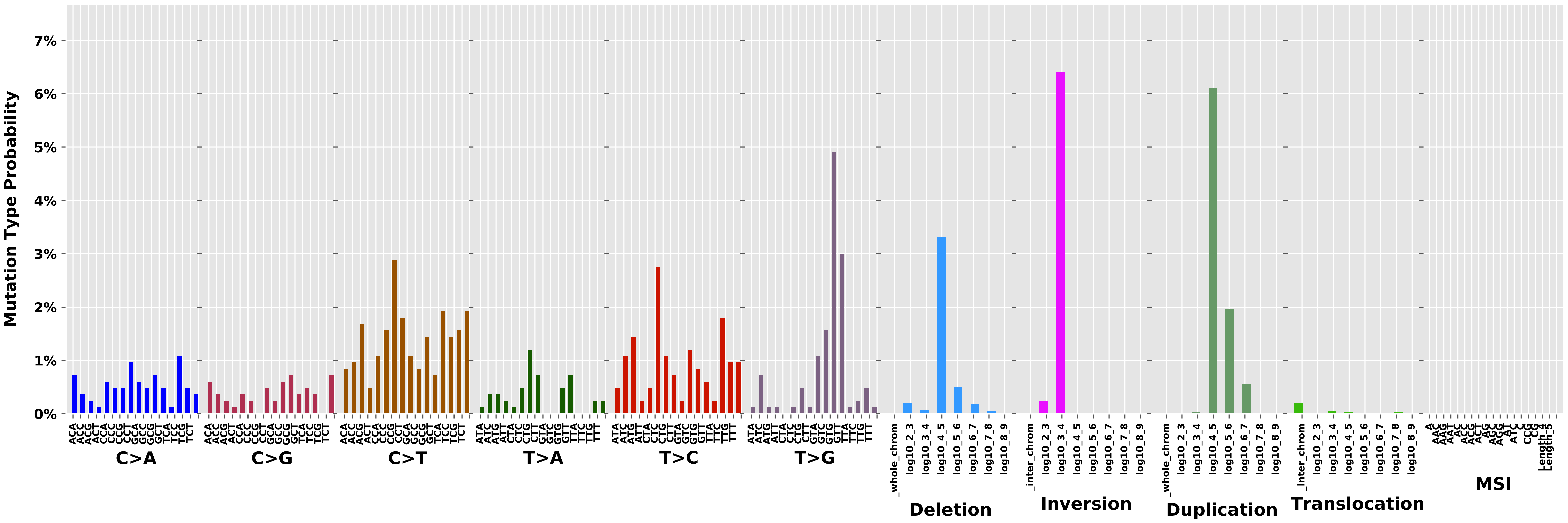

Cancer processes Weights for TCGA-B6-A0RT

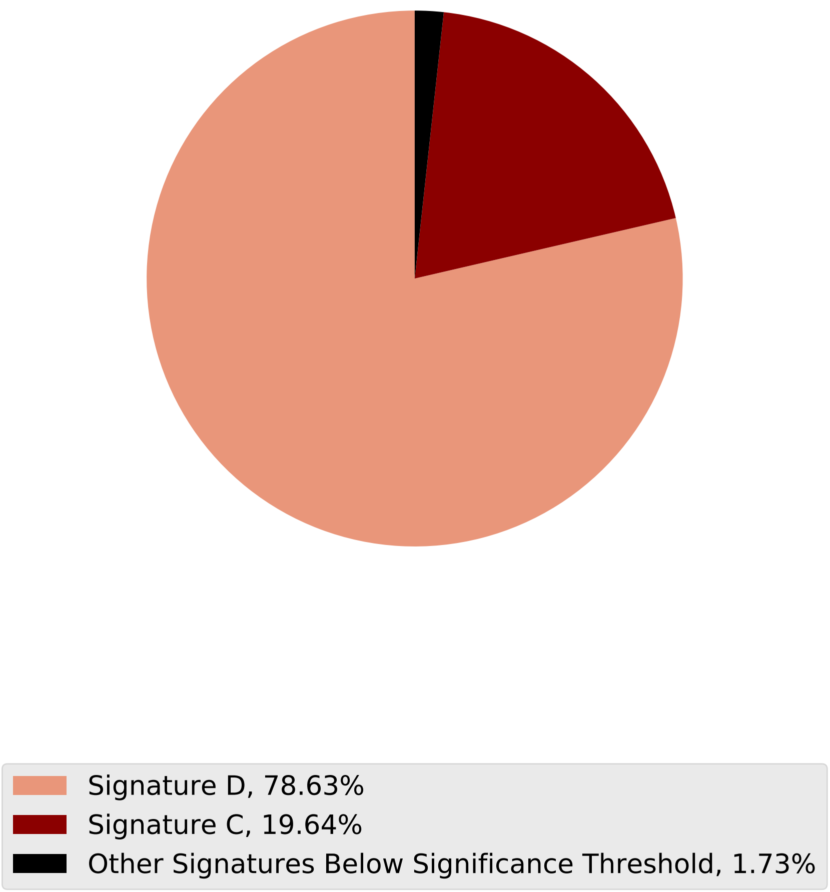

Tumor Profile for TCGA-B6-A0RT

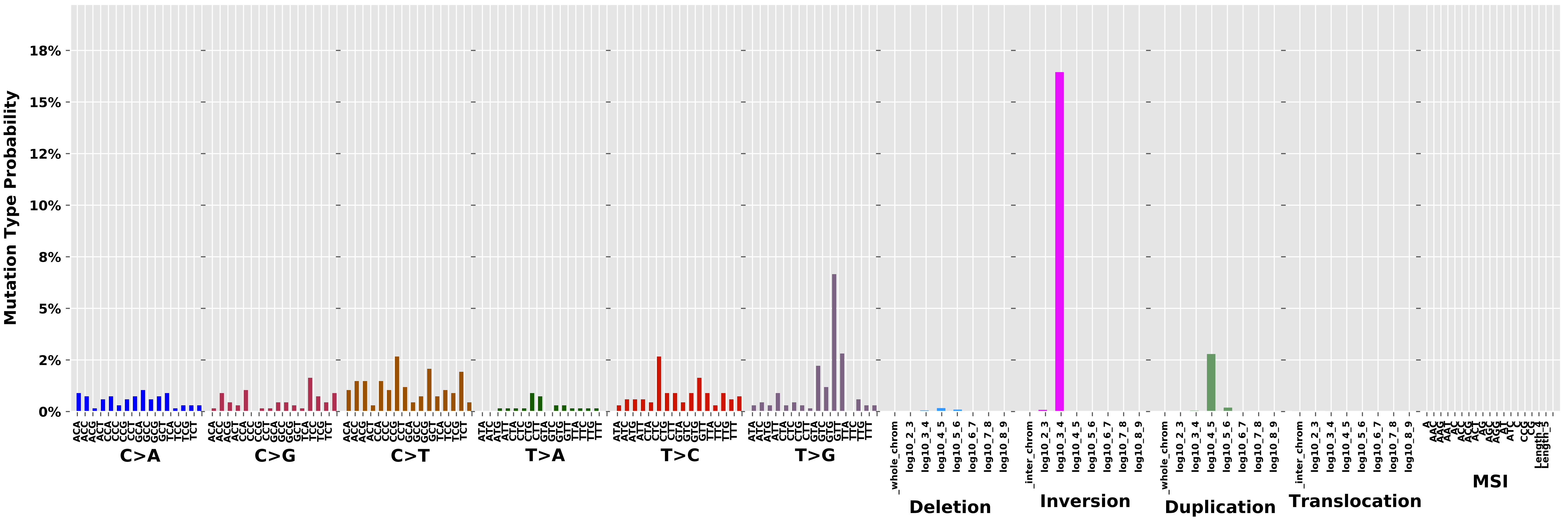

Cancer processes Weights for TCGA-GM-A3XL

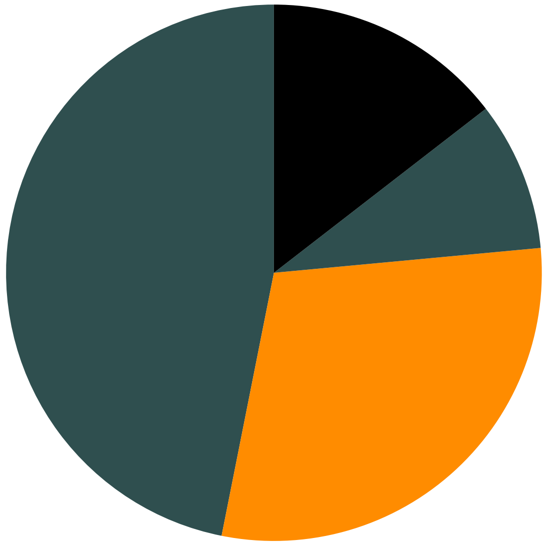

- Signature H, 46.87%
- Signature A, 29.61%
- Signature G, 9.0%
- Other Signatures Below Significance Threshold, 14.52%

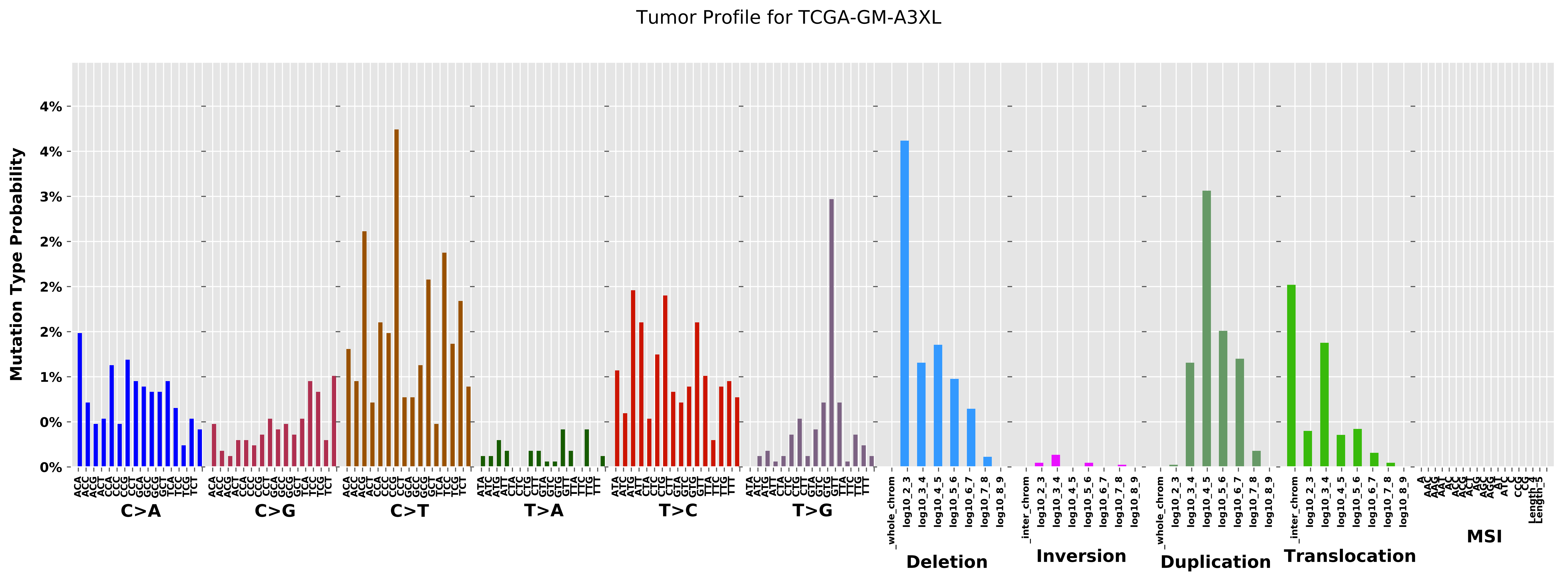
Tumor Profile for TCGA-GM-A3XL
Mutation Type Probability
4%
4%
3%
2%
2%
2%
1%
0%
0%
C>A
C>G
C>T
T>A
T>C
T>G
Deletion
Inversion
Duplication
Translocation
MSI
_whole_chrom
_inter_chrom
log10_2_3
log10_3_4
log10_4_5
log10_5_6
log10_6_7
log10_7_8
log10_8_9

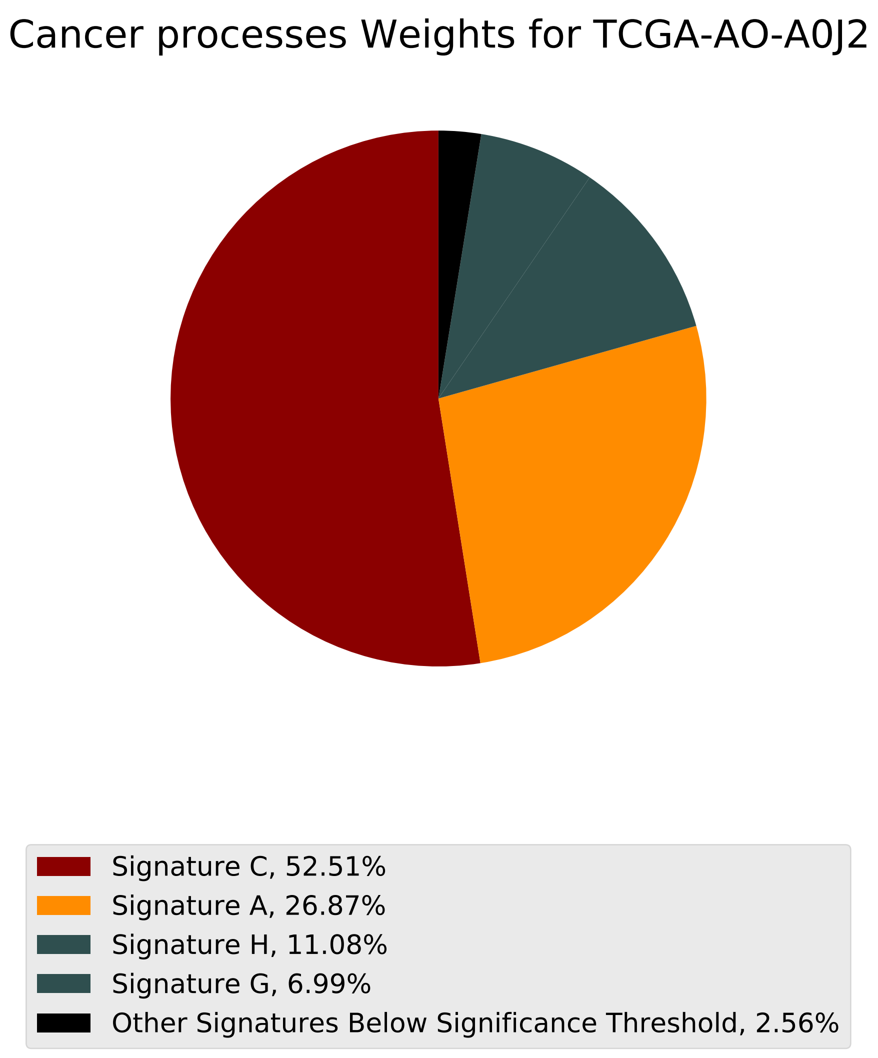
Cancer processes Weights for TCGA-AO-A0J2
Signature C, 52.51%
Signature A, 26.87%
Signature H, 11.08%
Signature G, 6.99%
Other Signatures Below Significance Threshold, 2.56%

Tumor Profile for TCGA-AO-A0J2

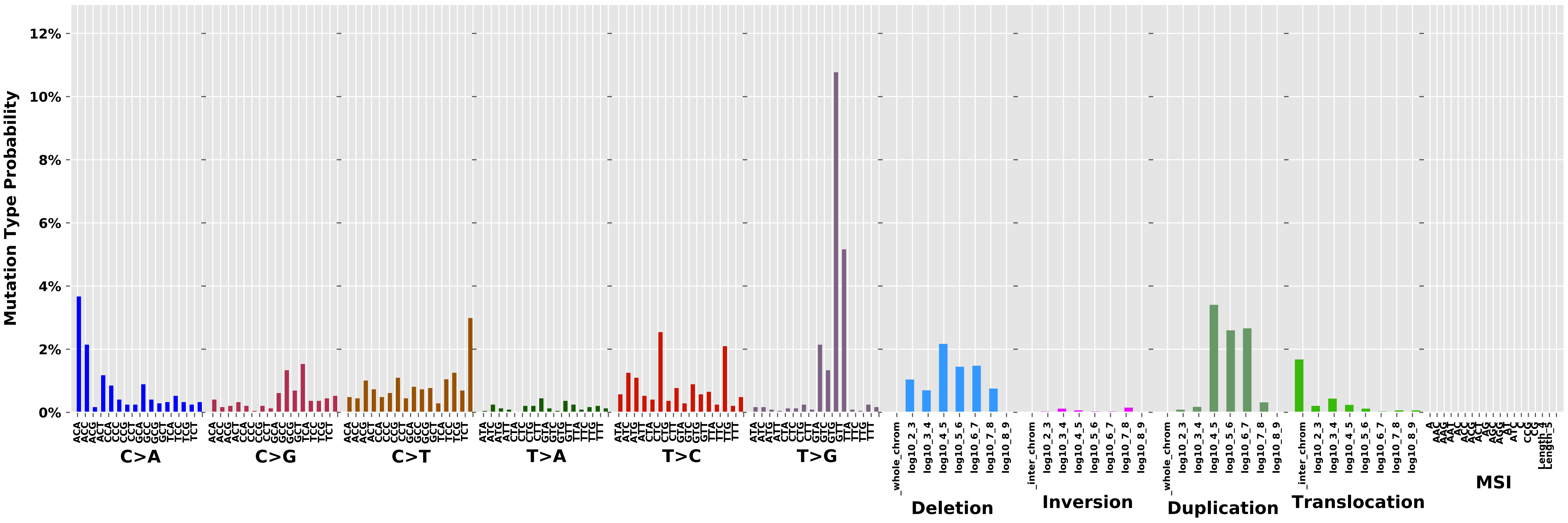

Cancer processes Weights for TCGA-E2-A156

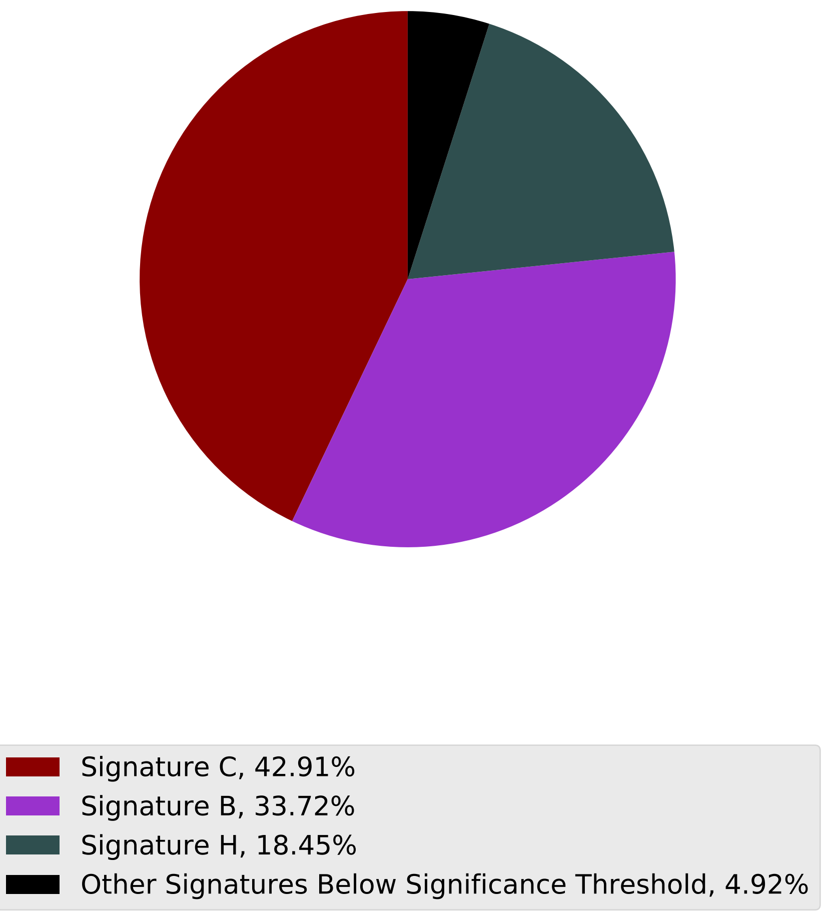

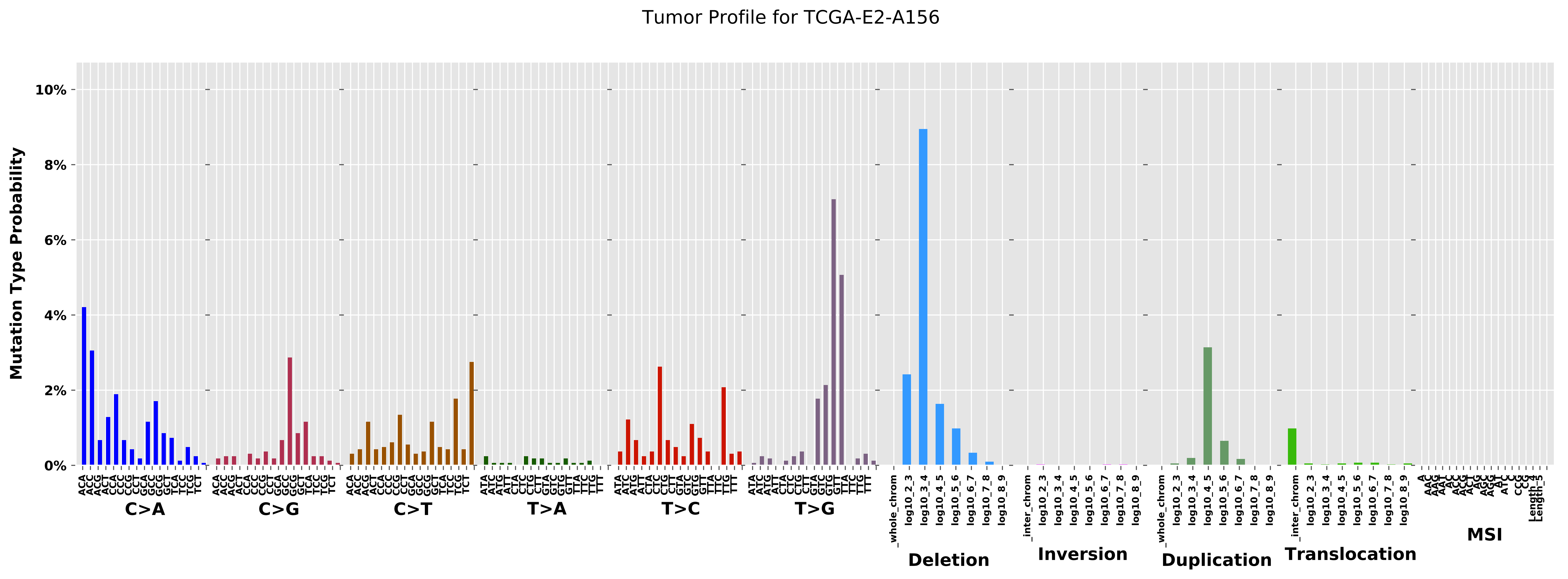

Tumor Profile for TCGA-E2-A156
Mutation Type Probability
10%
8%
6%
4%
2%
0%
C>A
C>G
C>T
T>A
T>C
T>G
Deletion
Inversion
Duplication
Translocation
MSI

Cancer processes Weights for TCGA-BH-A18R

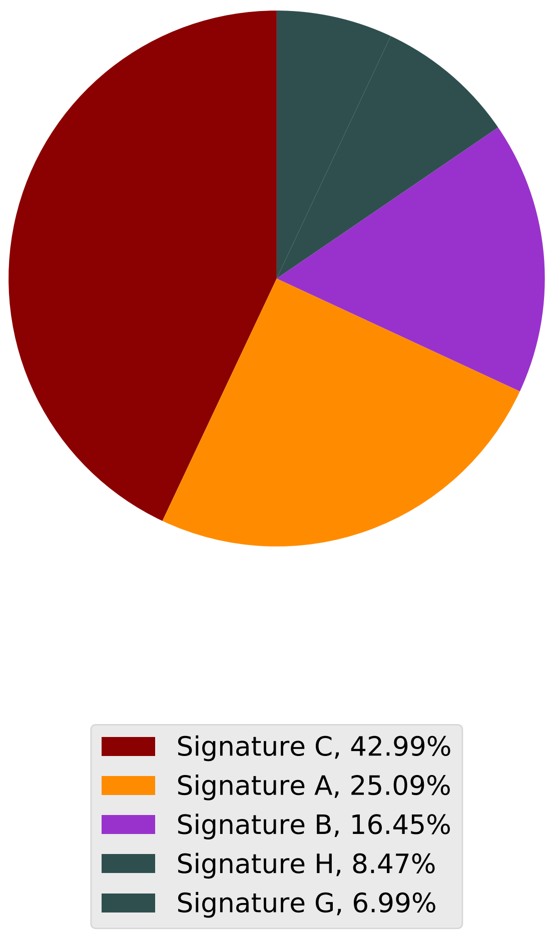

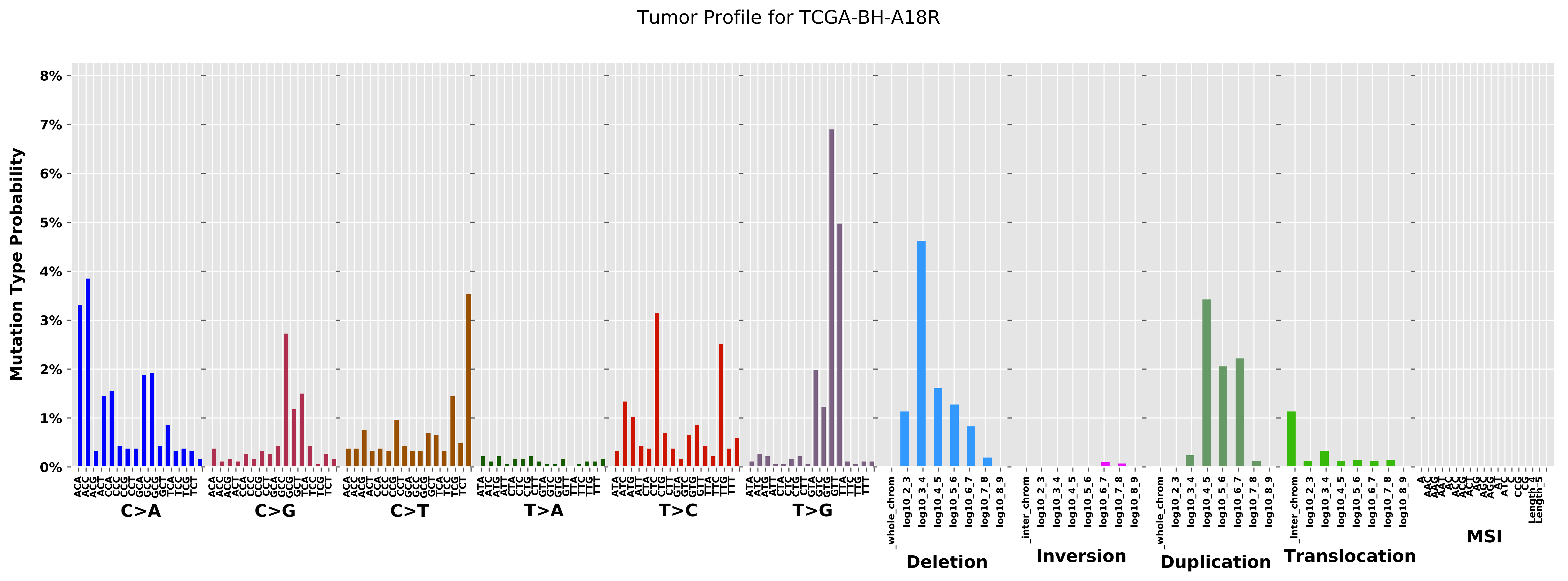

Tumor Profile for TCGA-BH-A18R
Mutation Type Probability
8%
7%
6%
5%
4%
3%
2%
1%
0%
C>A
C>G
C>T
T>A
T>C
T>G
Deletion
Inversion
Duplication
Translocation
MSI

Cancer processes Weights for TCGA-AO-A0JM

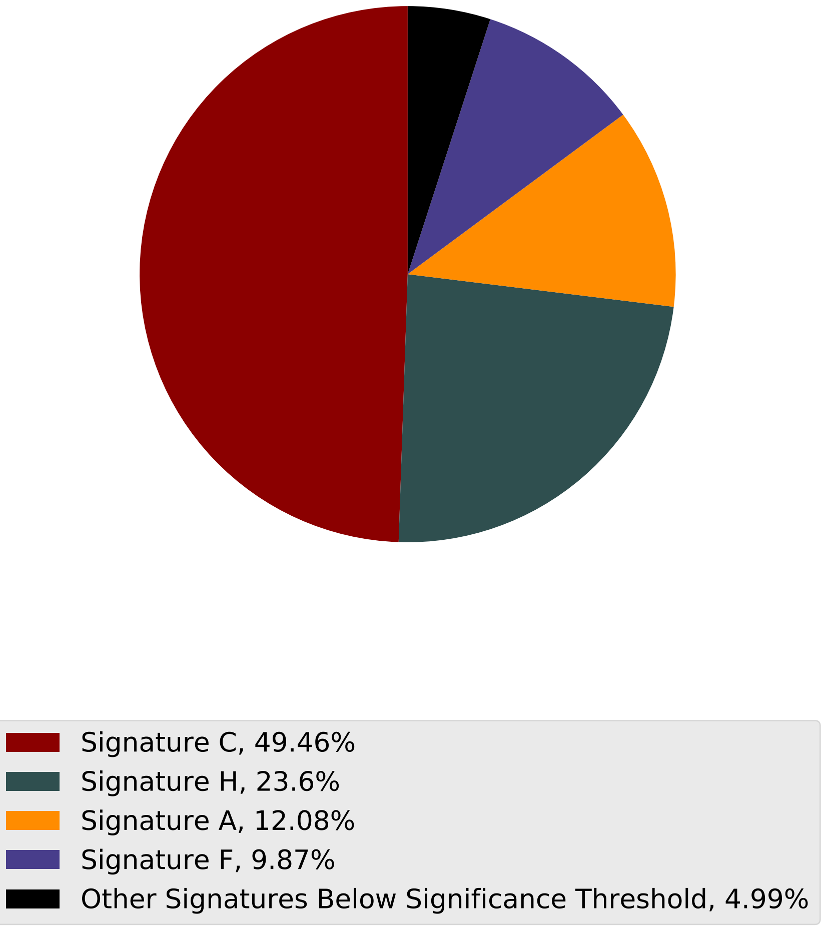

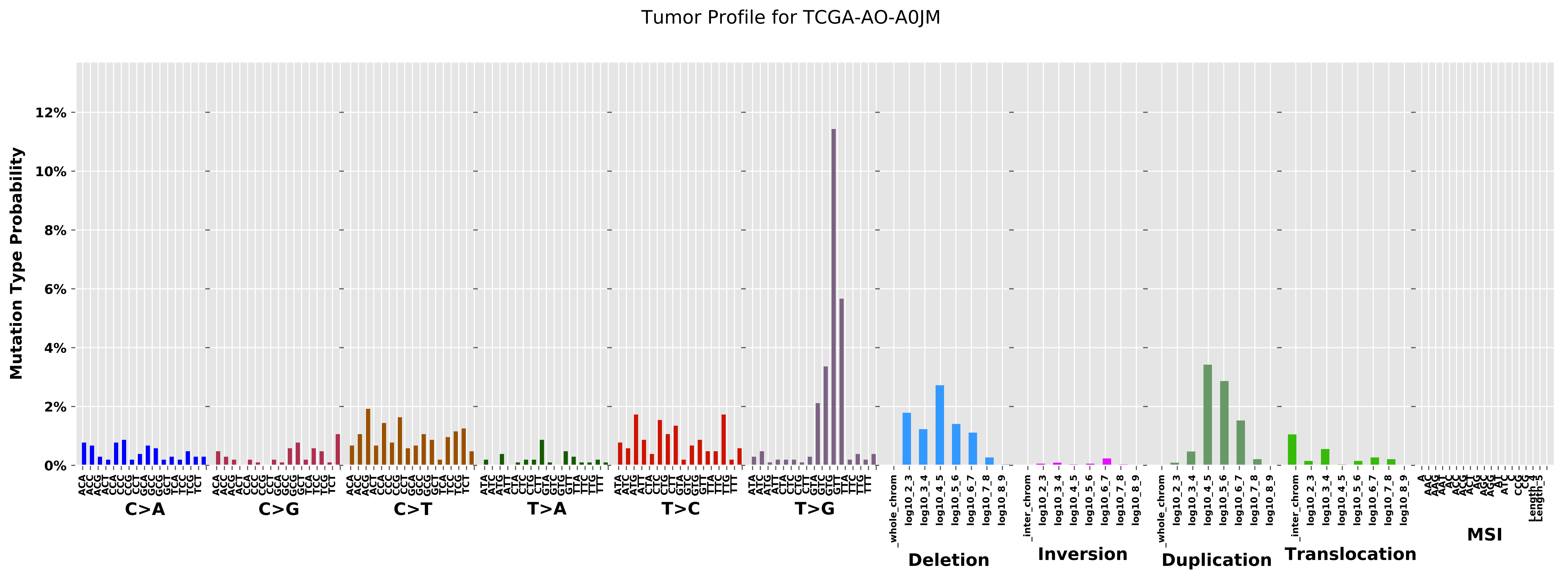
Tumor Profile for TCGA-AO-A0JM
Mutation Type Probability
12%
10%
8%
6%
4%
2%
0%
C>A
C>G
C>T
T>A
T>C
T>G
Deletion
Inversion
Duplication
Translocation
MSI

Cancer processes Weights for TCGA-AA-A01S

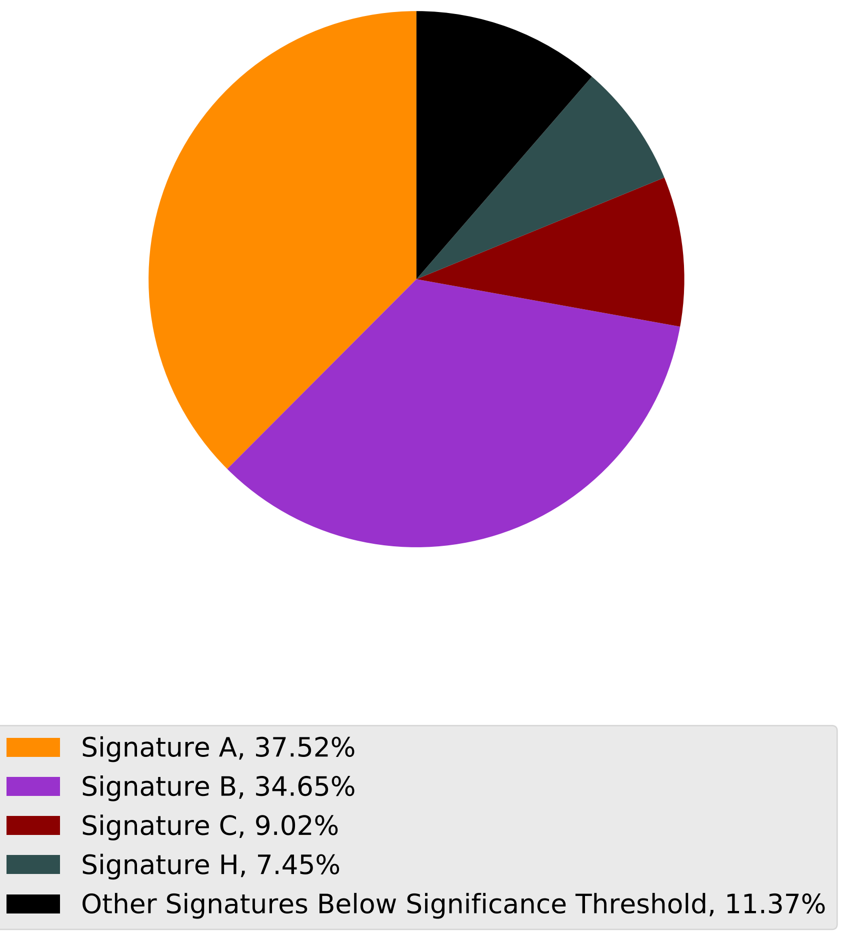

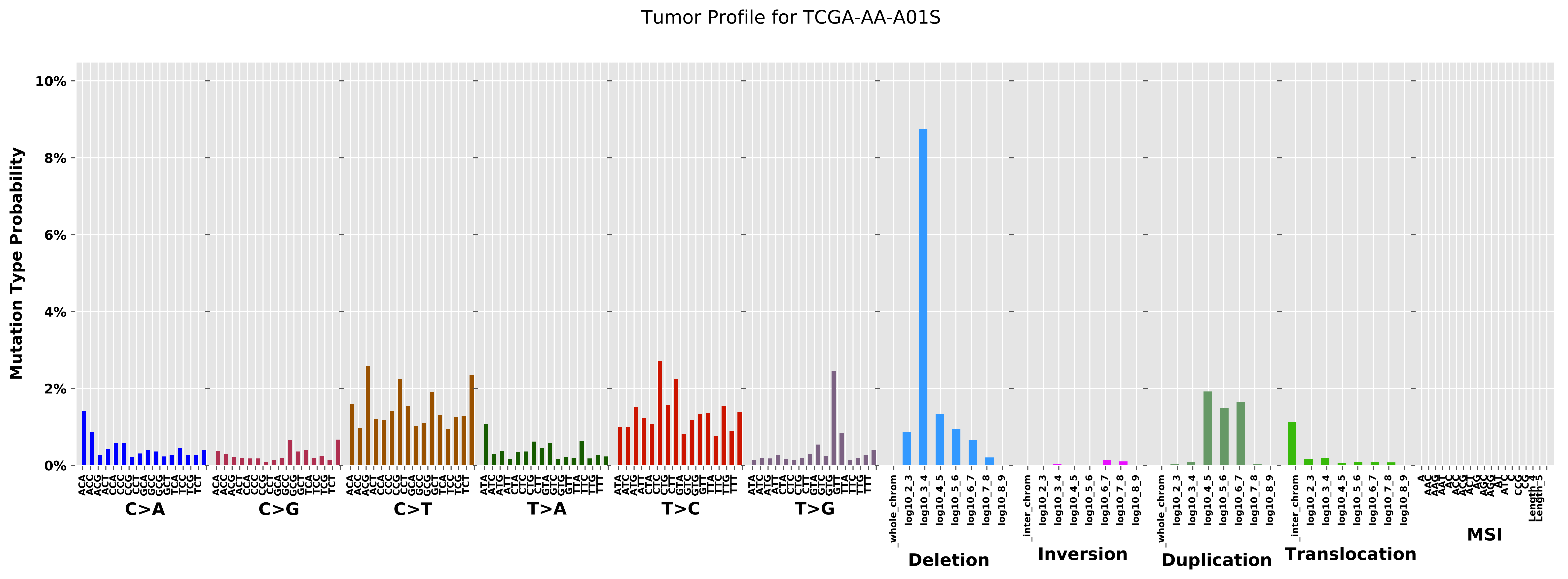

Tumor Profile for TCGA-AA-A01S
Mutation Type Probability
0%
2%
4%
6%
8%
10%
C>A
C>G
C>T
T>A
T>C
T>G
Deletion
Inversion
Duplication
Translocation
MSI
_whole_chrom
_inter_chrom
log10_2_3
log10_3_4
log10_4_5
log10_5_6
log10_6_7
log10_7_8
log10_8_9

Cancer processes Weights for TCGA-AN-A0AT

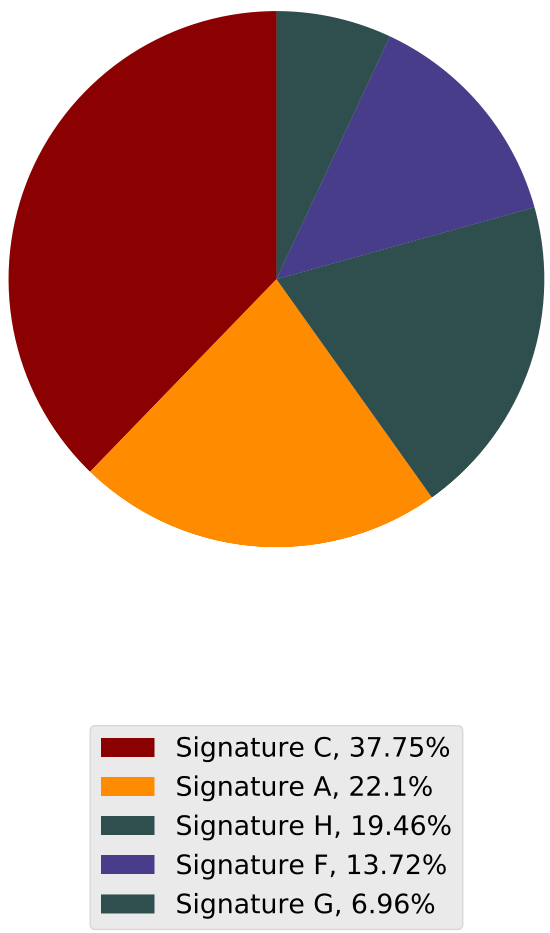

Tumor Profile for TCGA-AN-A0AT

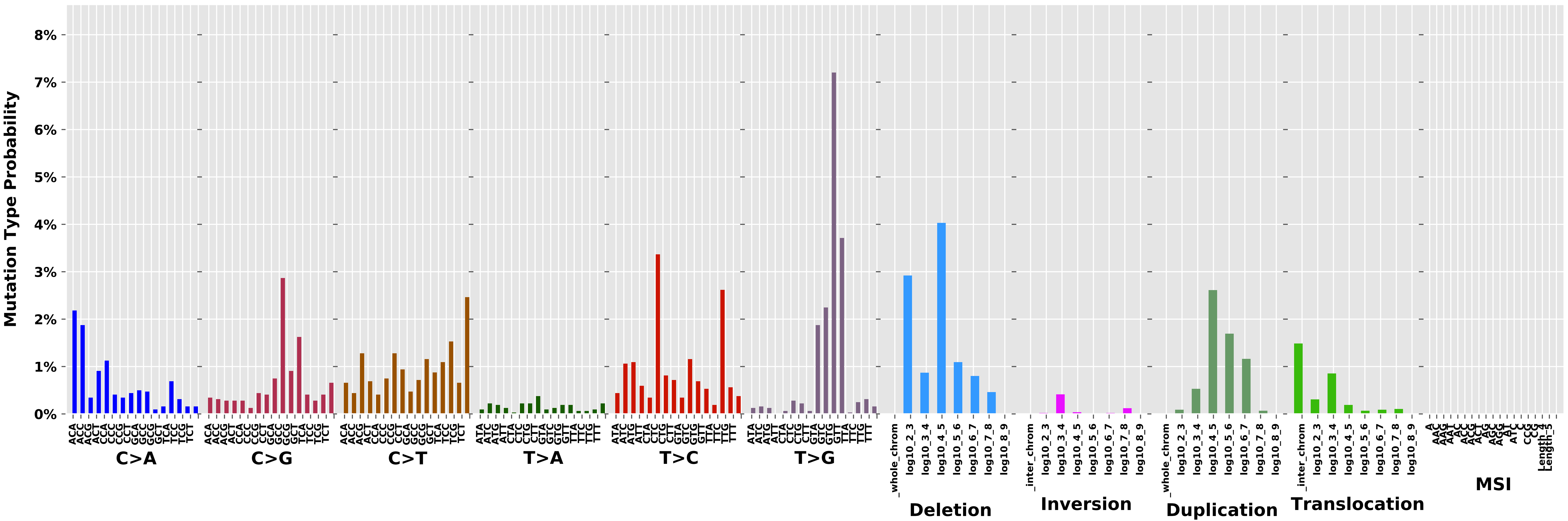

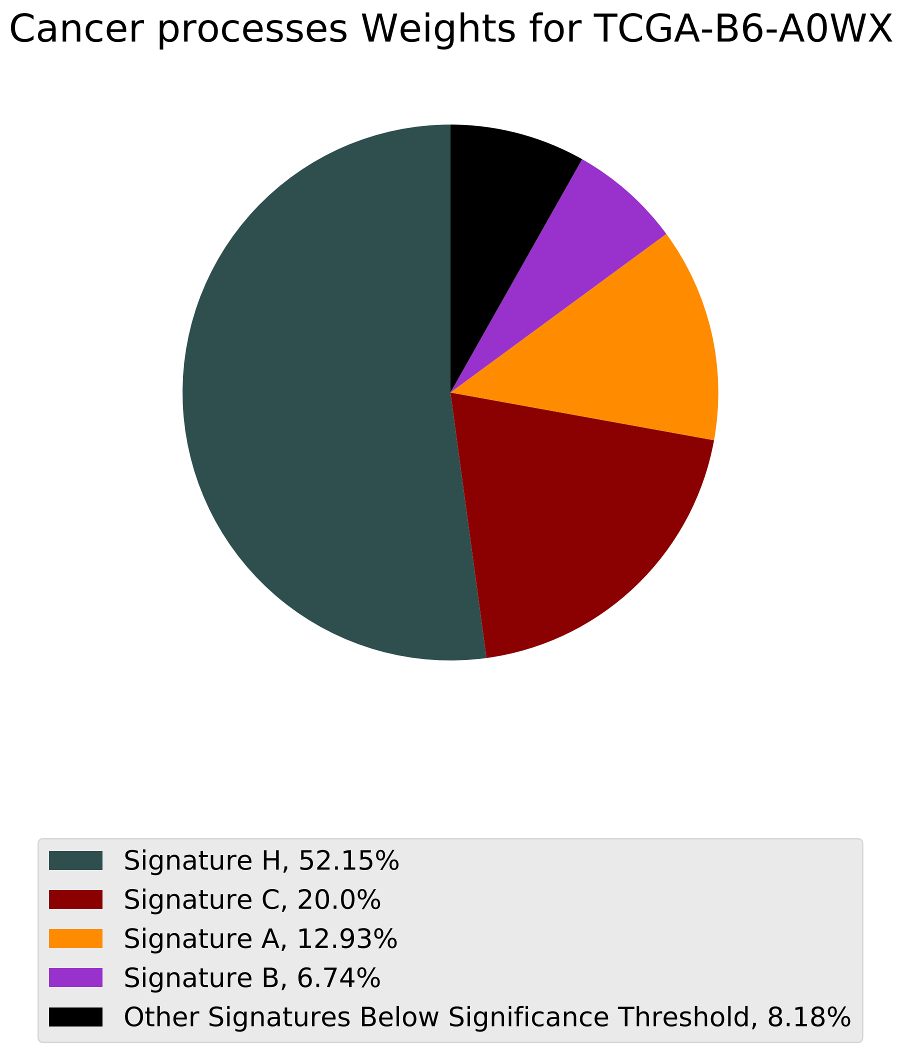
Cancer processes Weights for TCGA-B6-A0WX
Signature H, 52.15%
Signature C, 20.0%
Signature A, 12.93%
Signature B, 6.74%
Other Signatures Below Significance Threshold, 8.18%

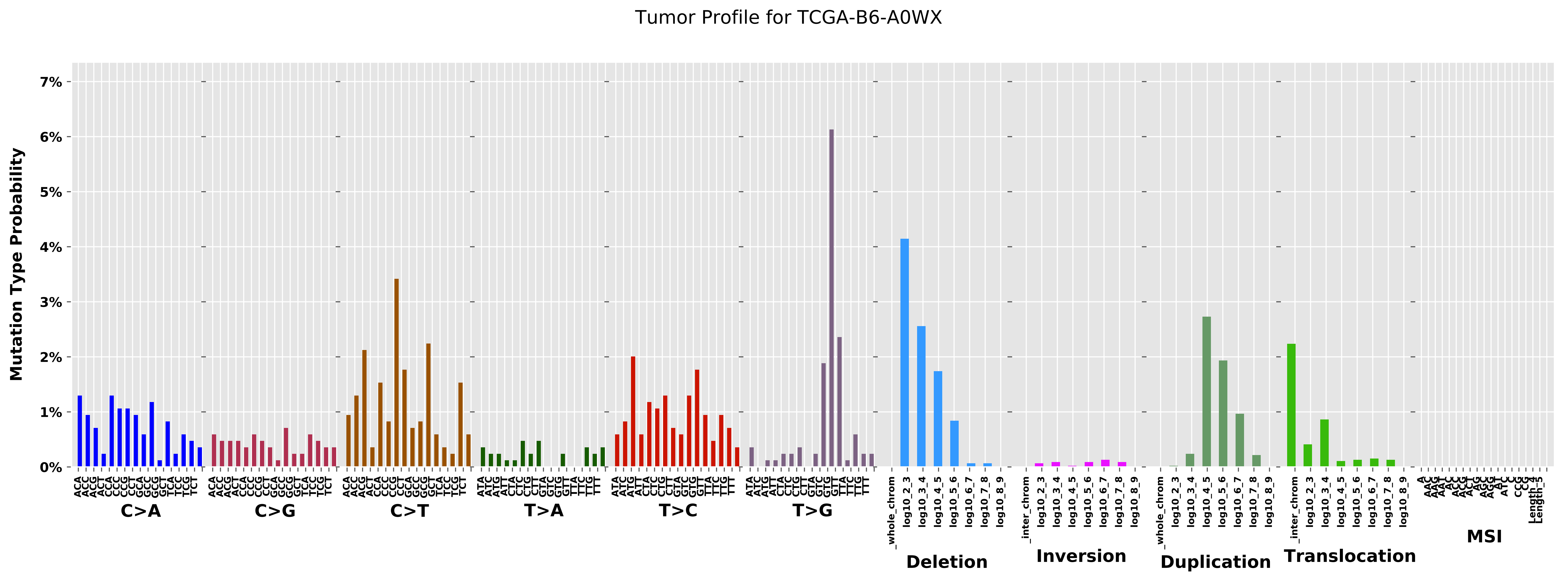
Tumor Profile for TCGA-B6-A0WX
Mutation Type Probability
7%
6%
5%
4%
3%
2%
1%
0%
ACA ACG ACT CCA CCC CCG CCT GCA GCC GCG GCT TCA TCC TCG TCT
C>A
ACA ACG ACT CCA CCC CCG CCT GCA GCC GCG GCT TCA TCC TCG TCT
C>G
ACA ACG ACT CCA CCC CCG CCT GCA GCC GCG GCT TCA TCC TCG TCT
C>T
ATA ATC ATG ATT CTA CTC CTG CTT GTA GTC GTG GTT TTA TTC TTG TTT
T>A
ATA ATC ATG ATT CTA CTC CTG CTT GTA GTC GTG GTT TTA TTC TTG TTT
T>C
ATA ATC ATG ATT CTA CTC CTG CTT GTA GTC GTG GTT TTA TTC TTG TTT
T>G
_whole_chrom log10_2_3 log10_3_4 log10_4_5 log10_5_6 log10_6_7 log10_7_8 log10_8_9
Deletion
_inter_chrom log10_2_3 log10_3_4 log10_4_5 log10_5_6 log10_6_7 log10_7_8 log10_8_9
Inversion
_whole_chrom log10_2_3 log10_3_4 log10_4_5 log10_5_6 log10_6_7 log10_7_8 log10_8_9
Duplication
_inter_chrom log10_2_3 log10_3_4 log10_4_5 log10_5_6 log10_6_7 log10_7_8 log10_8_9
Translocation
MSI

Cancer processes Weights for TCGA-BH-A0WA

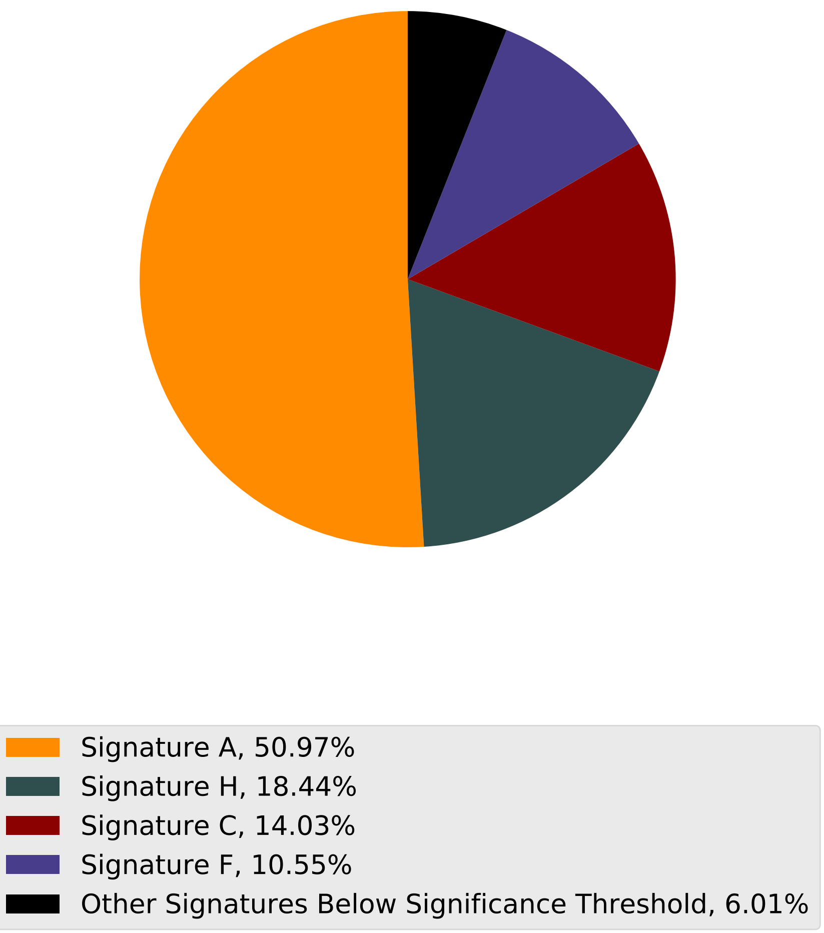

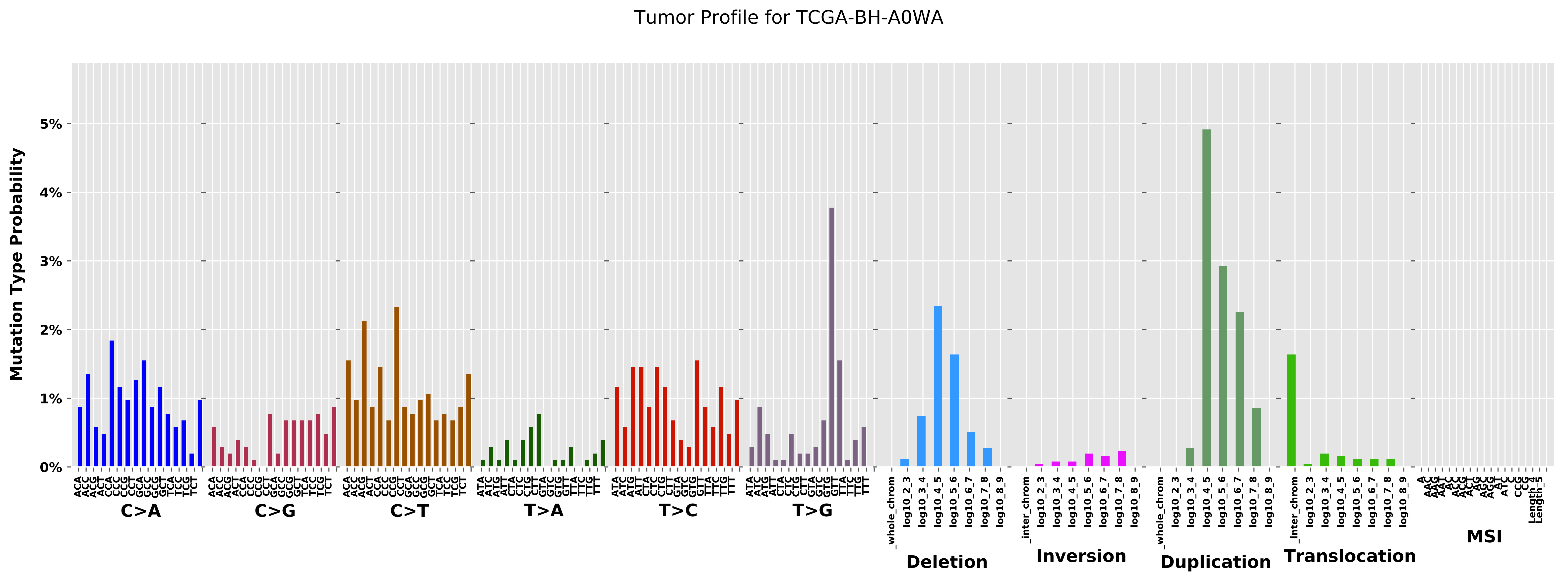

Tumor Profile for TCGA-BH-A0WA
Mutation Type Probability
0%
1%
2%
3%
4%
5%
C>A
C>G
C>T
T>A
T>C
T>G
Deletion
Inversion
Duplication
Translocation
MSI

Cancer processes Weights for TCGA-A7-A13D

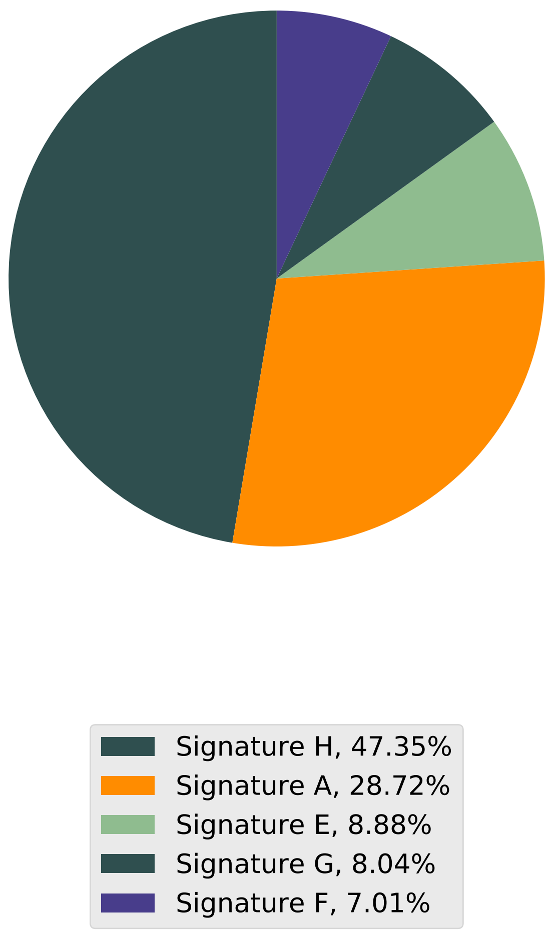

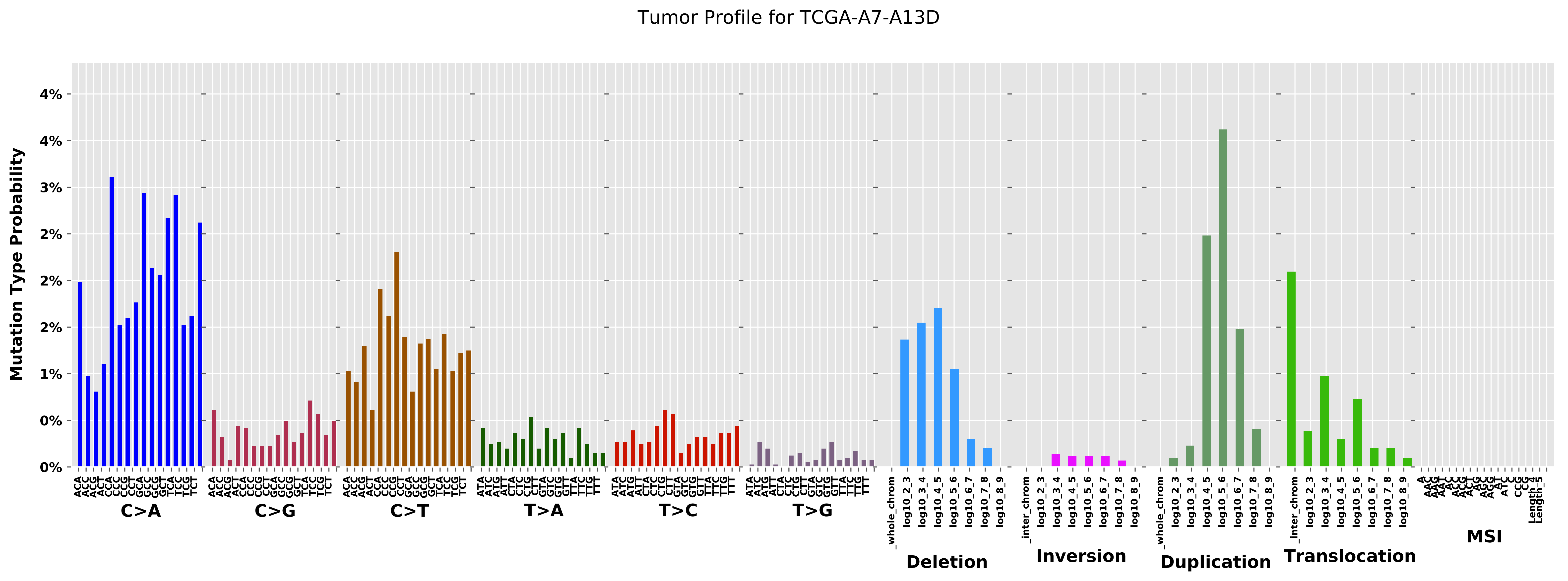

Tumor Profile for TCGA-A7-A13D
Mutation Type Probability
C>A
C>G
C>T
T>A
T>C
T>G
Deletion
Inversion
Duplication
Translocation
MSI

Cancer processes Weights for TCGA-B6-A0IJ

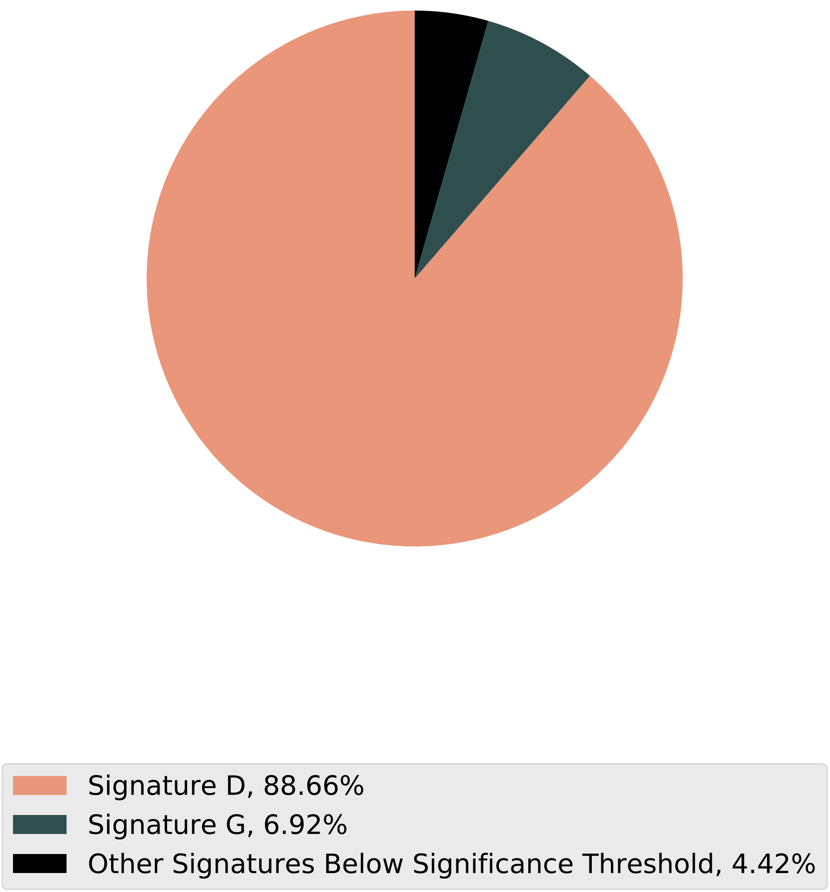

Tumor Profile for TCGA-B6-A0IJ

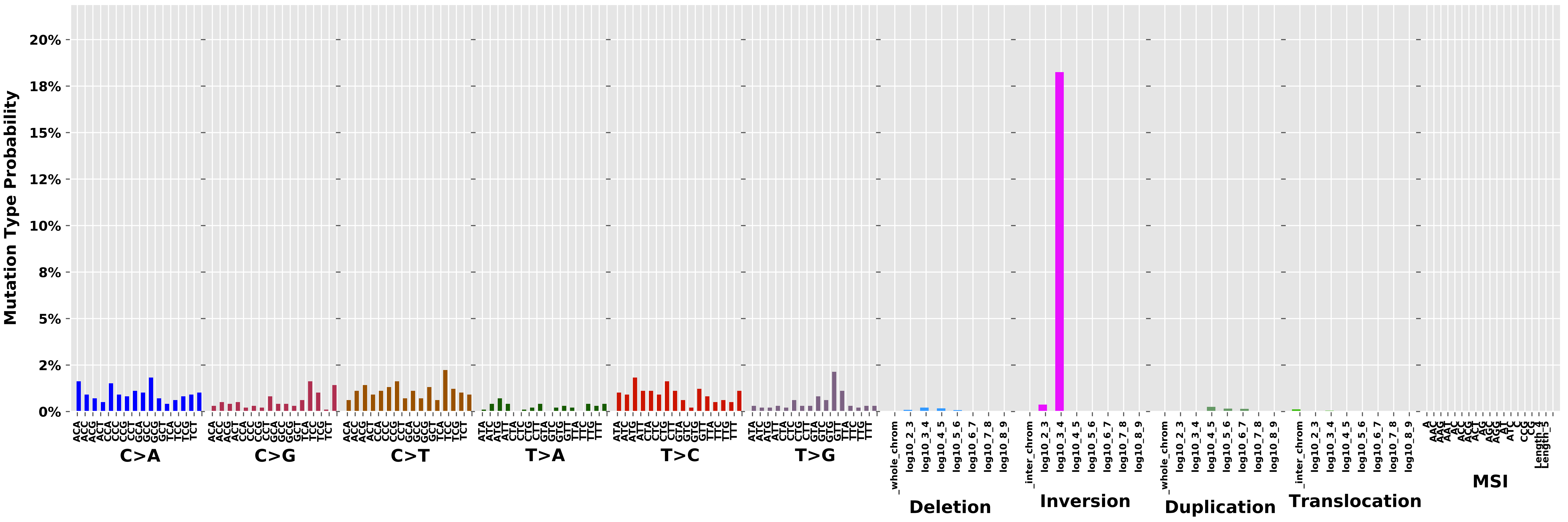

Cancer processes Weights for TCGA-A2-A04Q

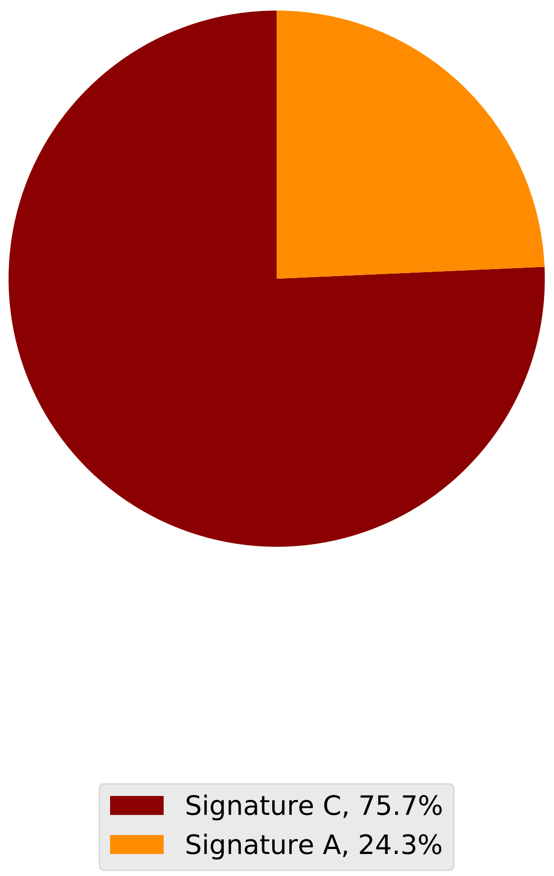

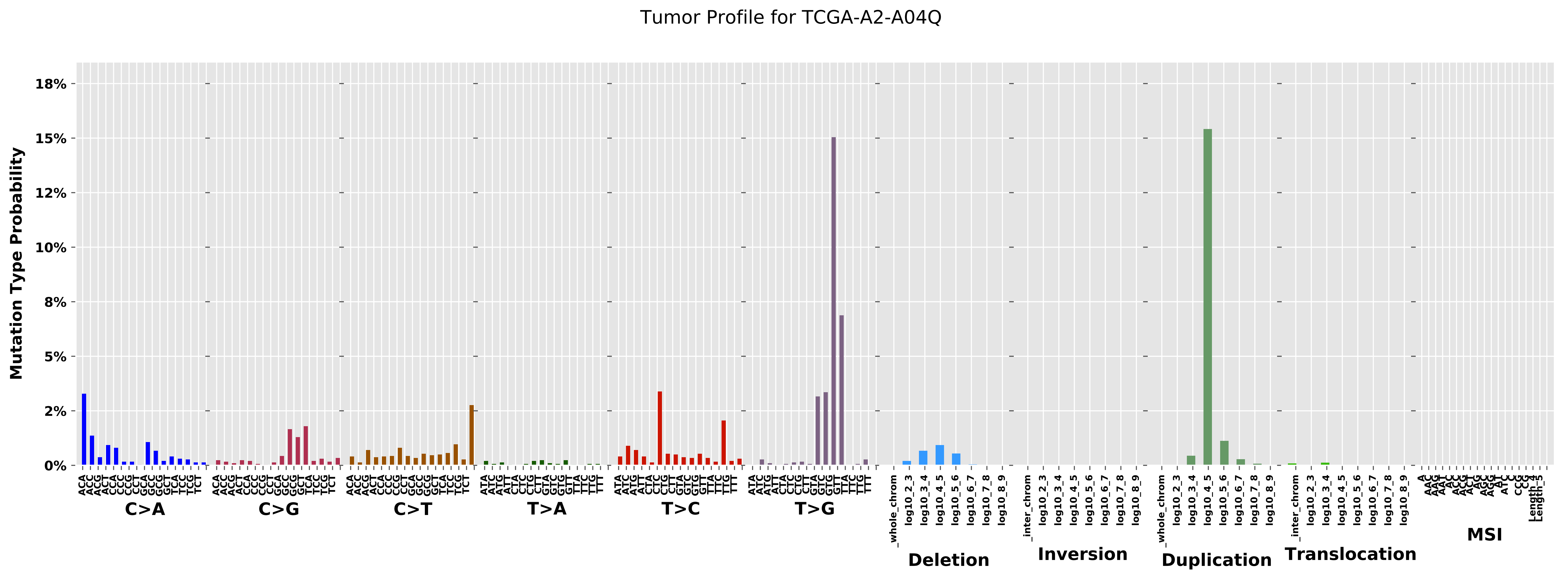
Tumor Profile for TCGA-A2-A04Q
Mutation Type Probability
18%
15%
12%
10%
8%
5%
2%
0%
C>A
C>G
C>T
T>A
T>C
T>G
Deletion
Inversion
Duplication
Translocation
MSI

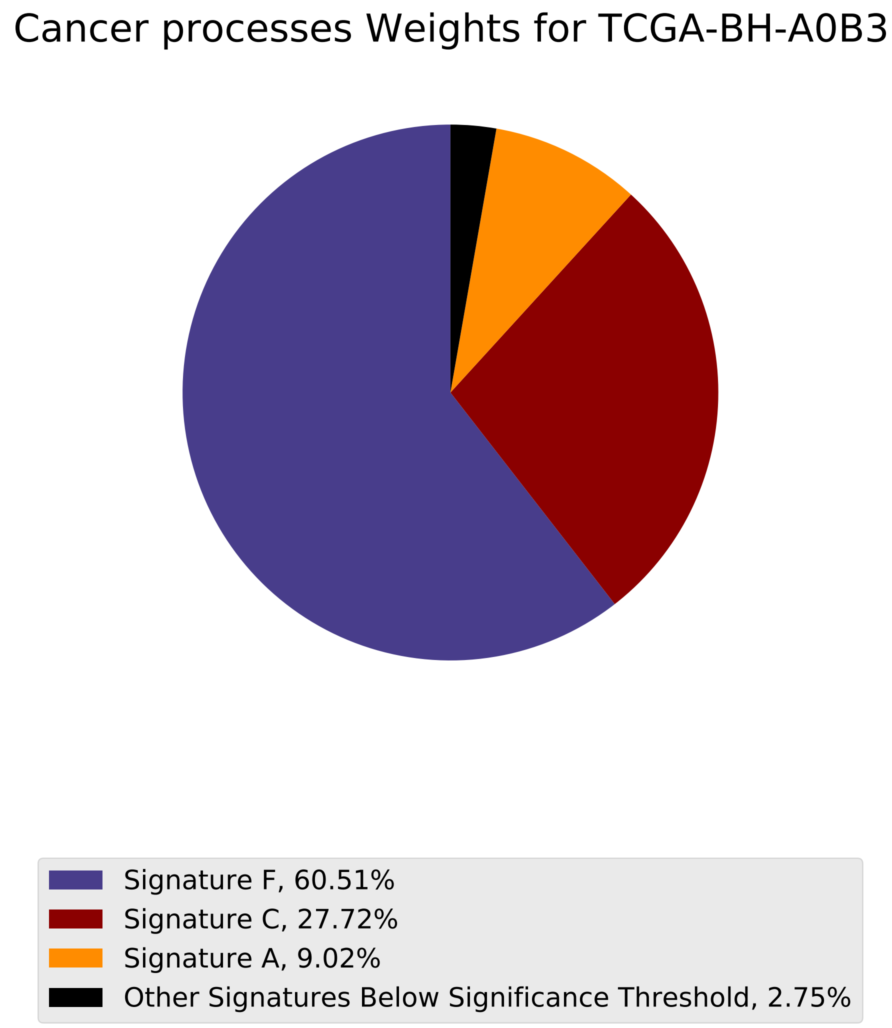
Cancer processes Weights for TCGA-BH-A0B3
Signature F, 60.51%
Signature C, 27.72%
Signature A, 9.02%
Other Signatures Below Significance Threshold, 2.75%

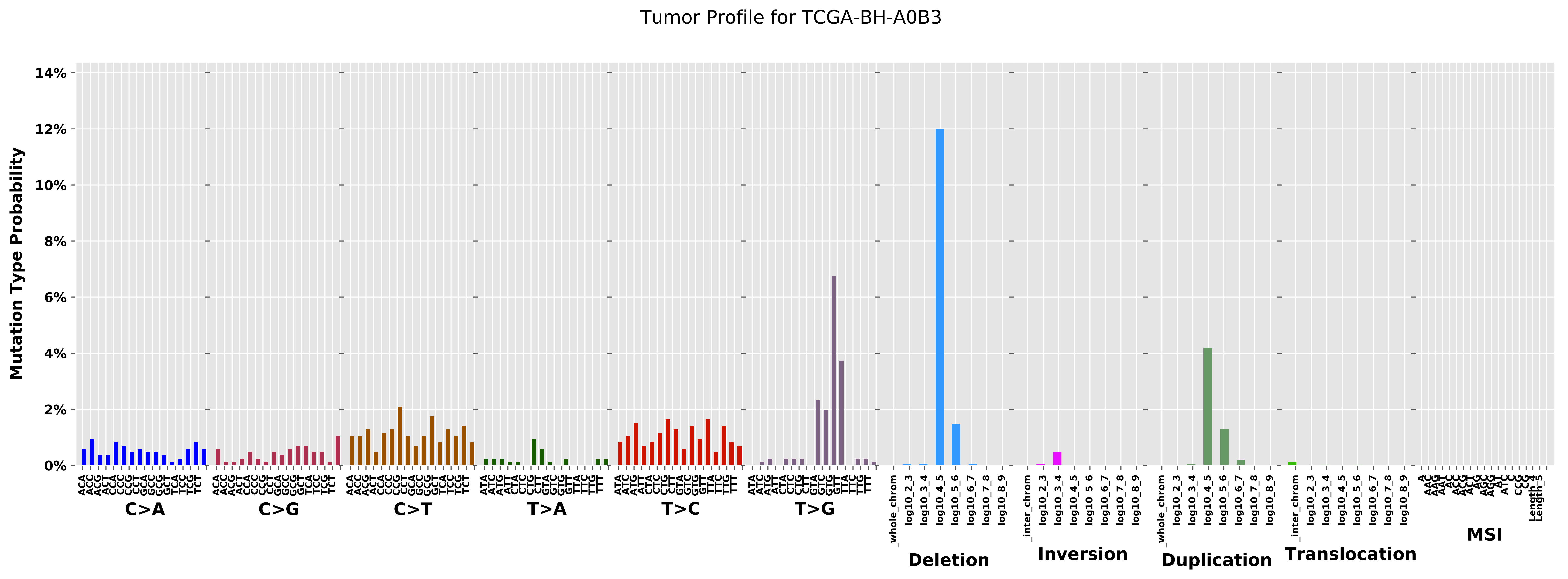

Tumor Profile for TCGA-BH-A0B3
Mutation Type Probability
0%
2%
4%
6%
8%
10%
12%
14%
C>A
C>G
C>T
T>A
T>C
T>G
Deletion
Inversion
Duplication
Translocation
MSI
_whole_chrom
_inter_chrom
log10_2_3
log10_3_4
log10_4_5
log10_5_6
log10_6_7
log10_7_8
log10_8_9

Cancer processes Weights for TCGA-BH-A18U

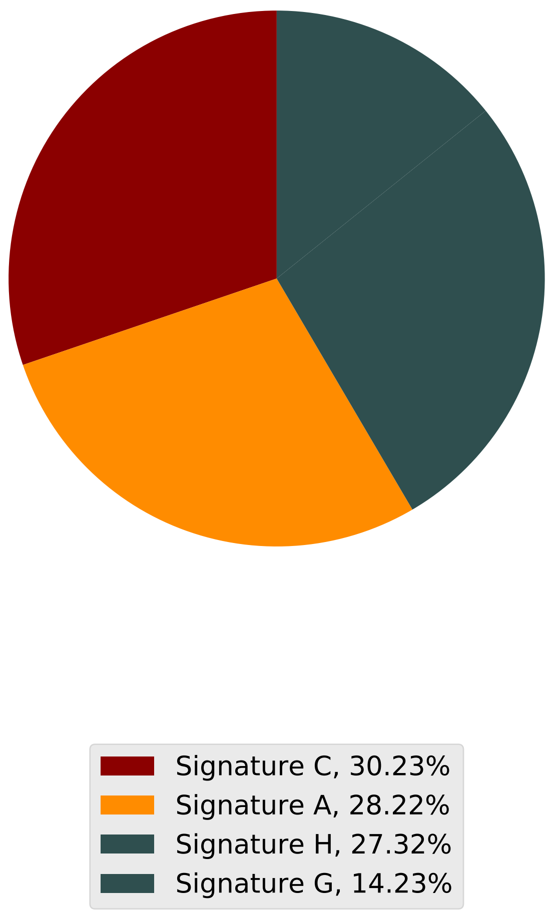

Tumor Profile for TCGA-BH-A18U

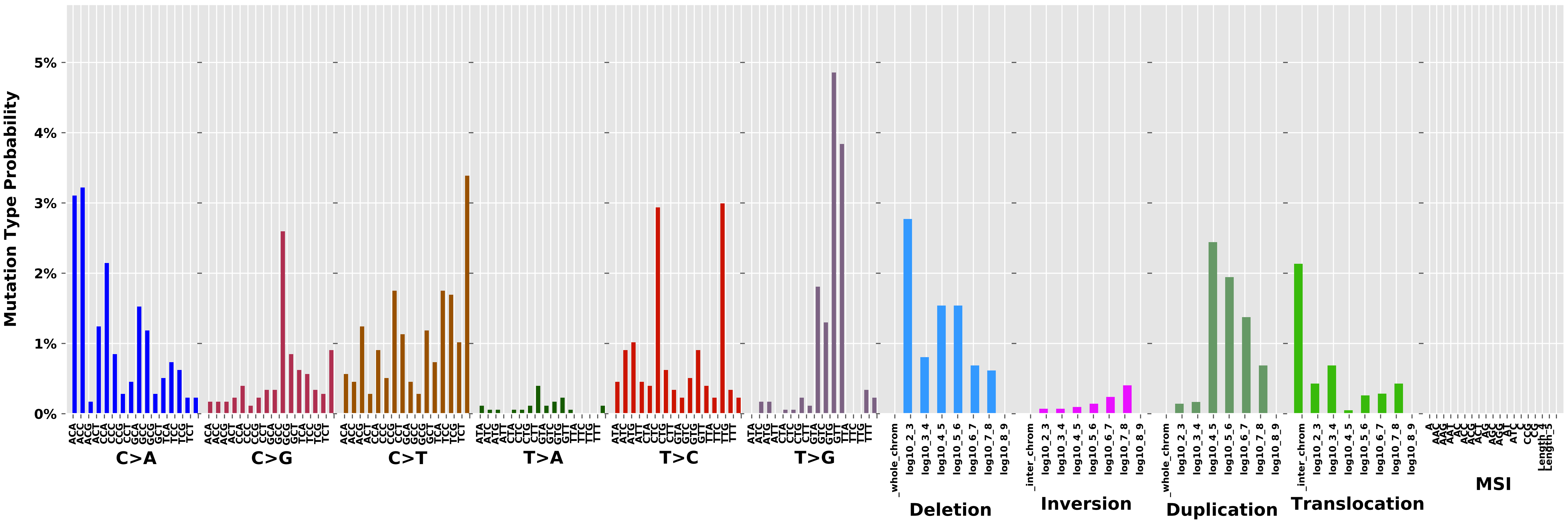

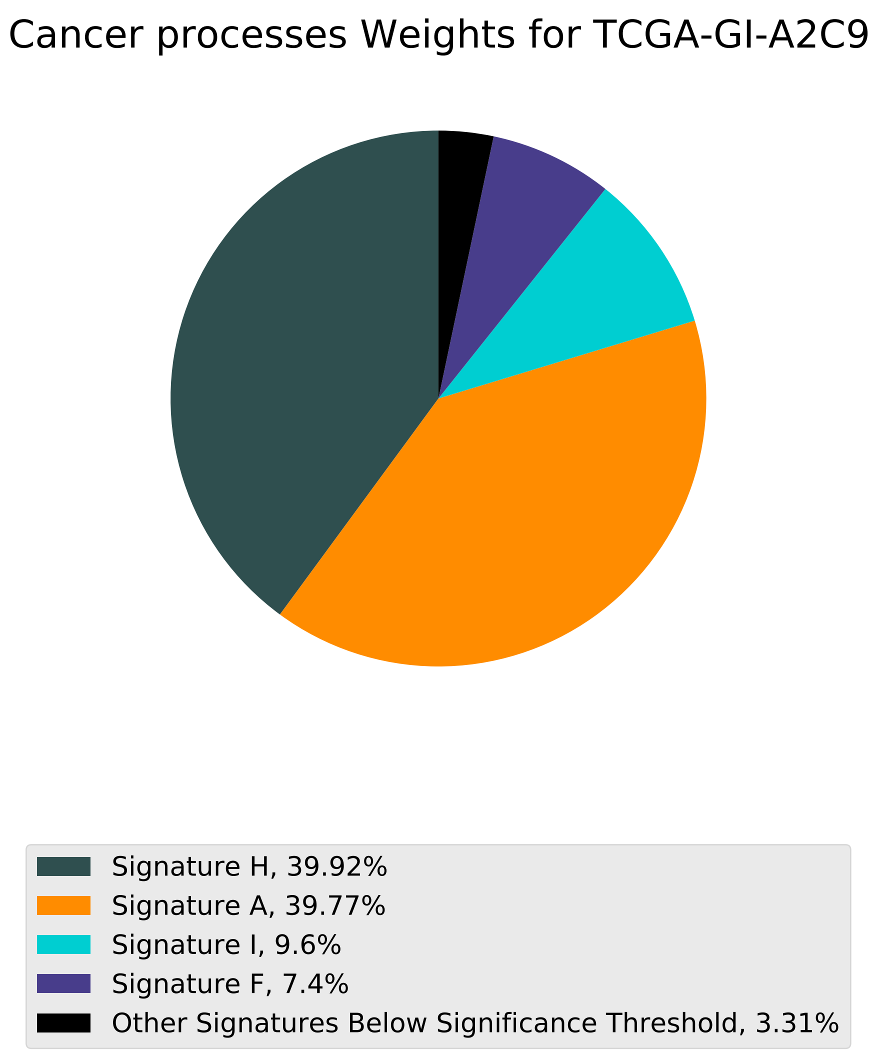
Cancer processes Weights for TCGA-GI-A2C9
Signature H, 39.92%
Signature A, 39.77%
Signature I, 9.6%
Signature F, 7.4%
Other Signatures Below Significance Threshold, 3.31%

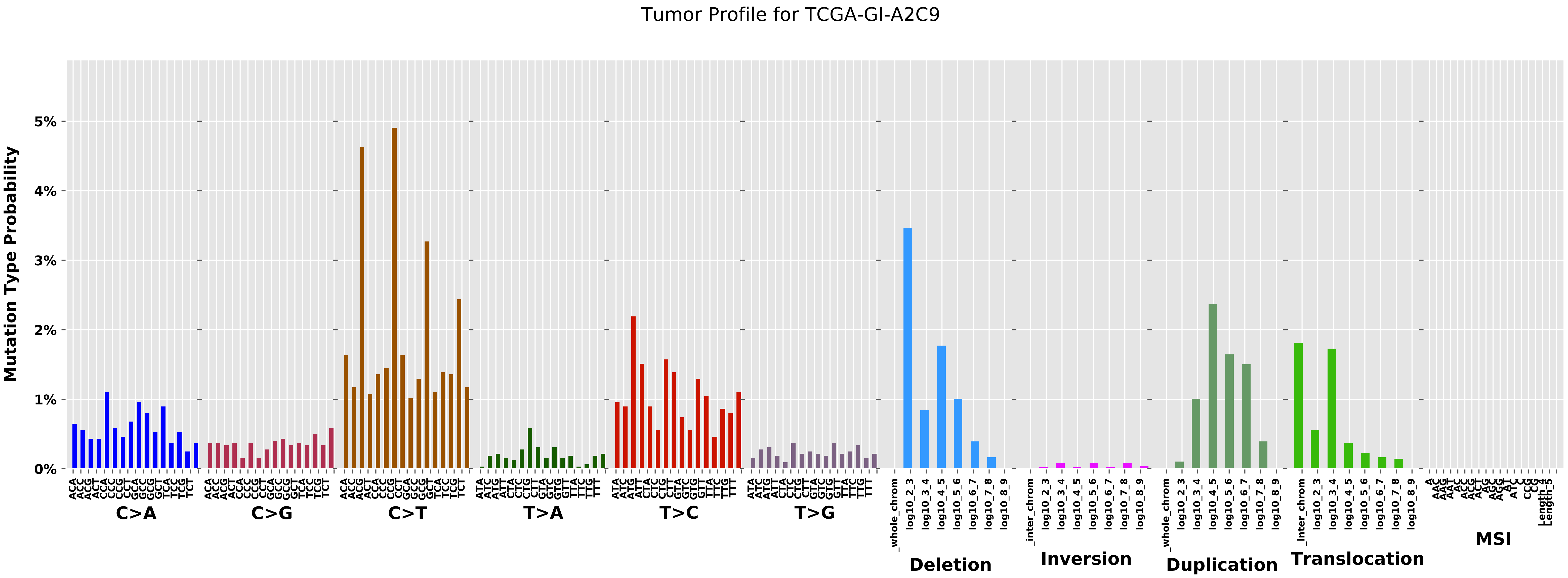

Tumor Profile for TCGA-GI-A2C9
Mutation Type Probability
0%
1%
2%
3%
4%
5%
C>A
C>G
C>T
T>A
T>C
T>G
Deletion
Inversion
Duplication
Translocation
MSI

Cancer processes Weights for TCGA-BH-A0DT

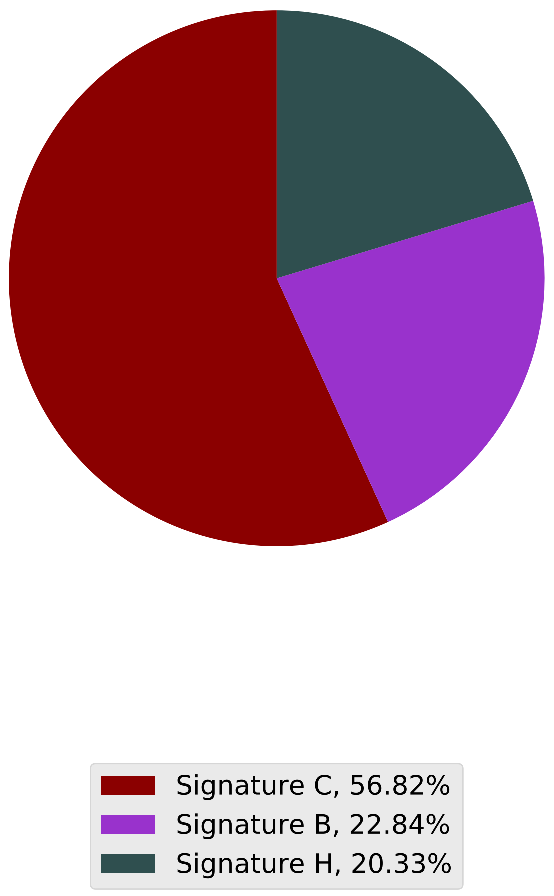

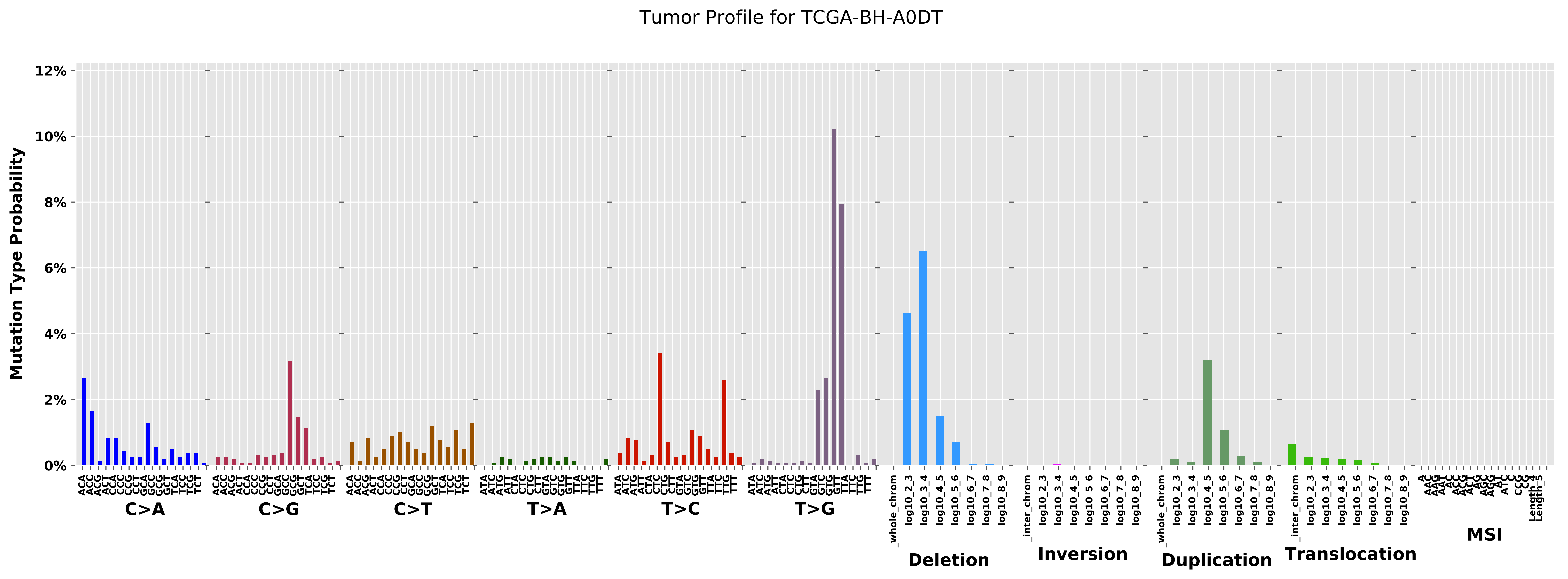

Tumor Profile for TCGA-BH-A0DT
Mutation Type Probability
12%
10%
8%
6%
4%
2%
0%
C>A
C>G
C>T
T>A
T>C
T>G
Deletion
Inversion
Duplication
Translocation
MSI

Cancer processes Weights for TCGA-A2-A0D4

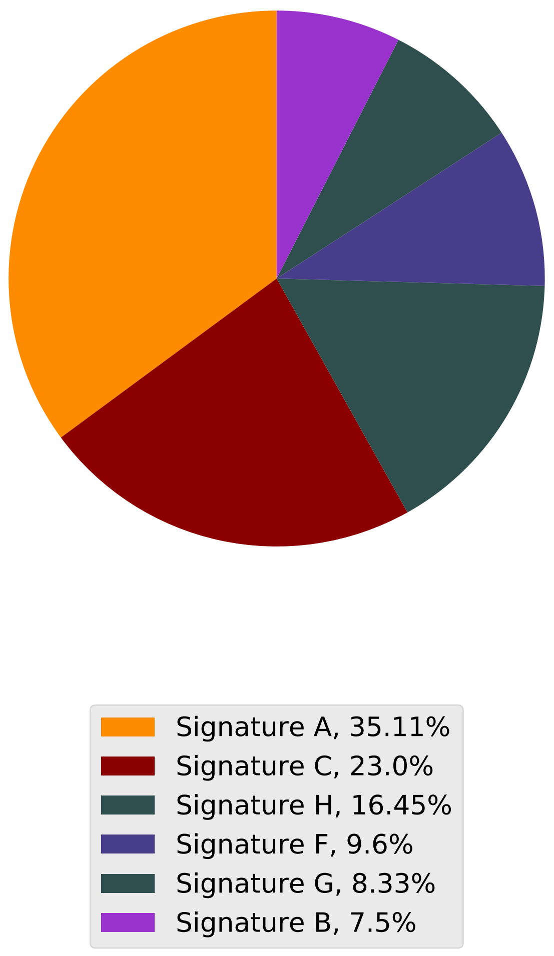

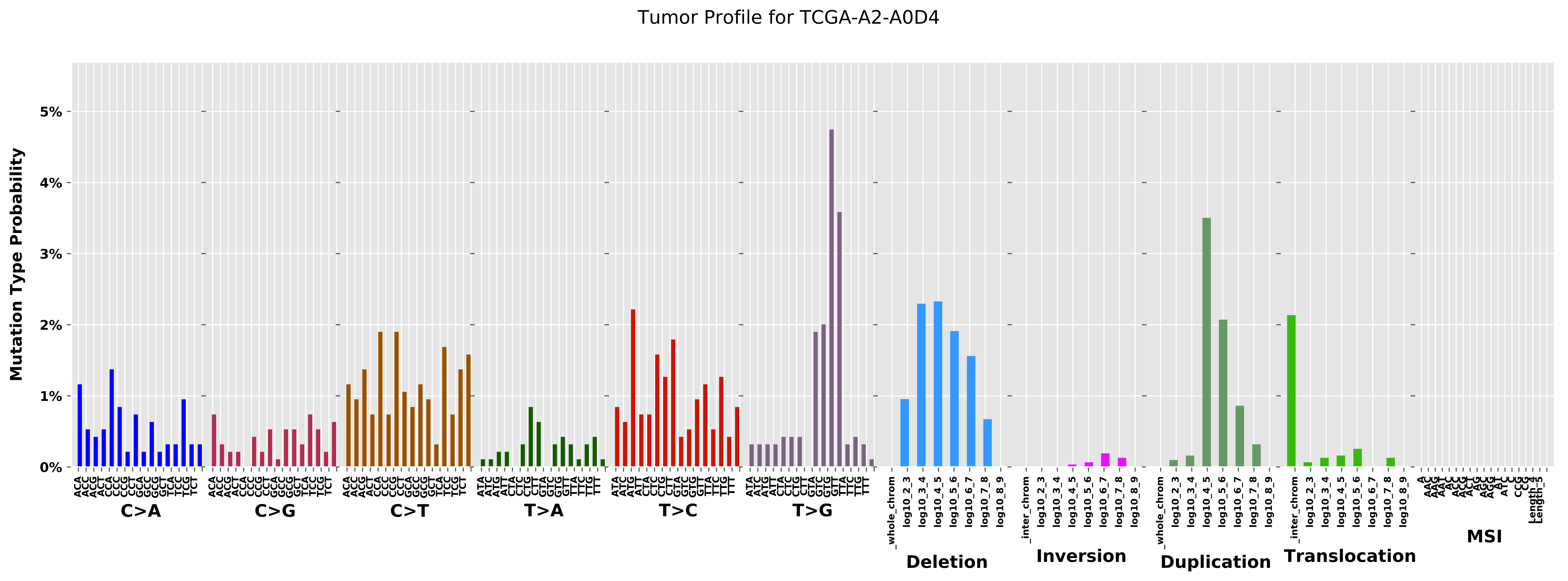
Tumor Profile for TCGA-A2-A0D4
Mutation Type Probability
0%
1%
2%
3%
4%
5%
C>A
C>G
C>T
T>A
T>C
T>G
Deletion
Inversion
Duplication
Translocation
MSI
_whole_chrom
_inter_chrom
log10_2_3
log10_3_4
log10_4_5
log10_5_6
log10_6_7
log10_7_8
log10_8_9

Cancer processes Weights for TCGA-EW-A1P8

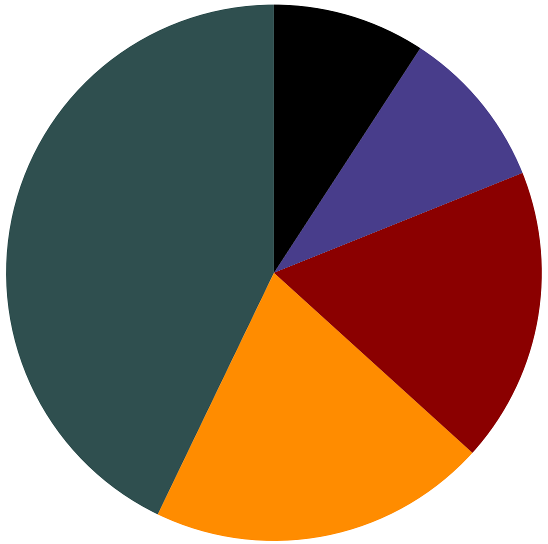

- Signature H, 42.86%
- Signature A, 20.43%
- Signature C, 17.79%
- Signature F, 9.73%
- Other Signatures Below Significance Threshold, 9.2%

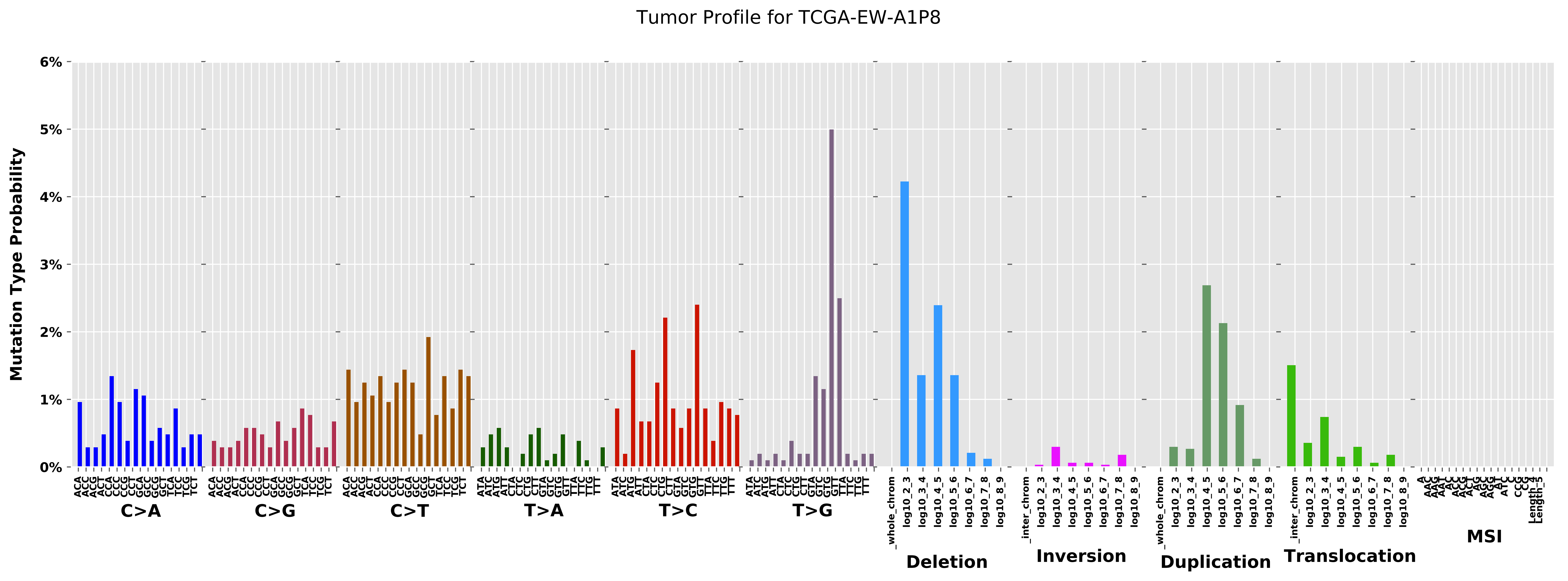

Tumor Profile for TCGA-EW-A1P8
Mutation Type Probability
6%
5%
4%
3%
2%
1%
0%
C>A
C>G
C>T
T>A
T>C
T>G
Deletion
Inversion
Duplication
Translocation
MSI

Cancer processes Weights for TCGA-A6-2683

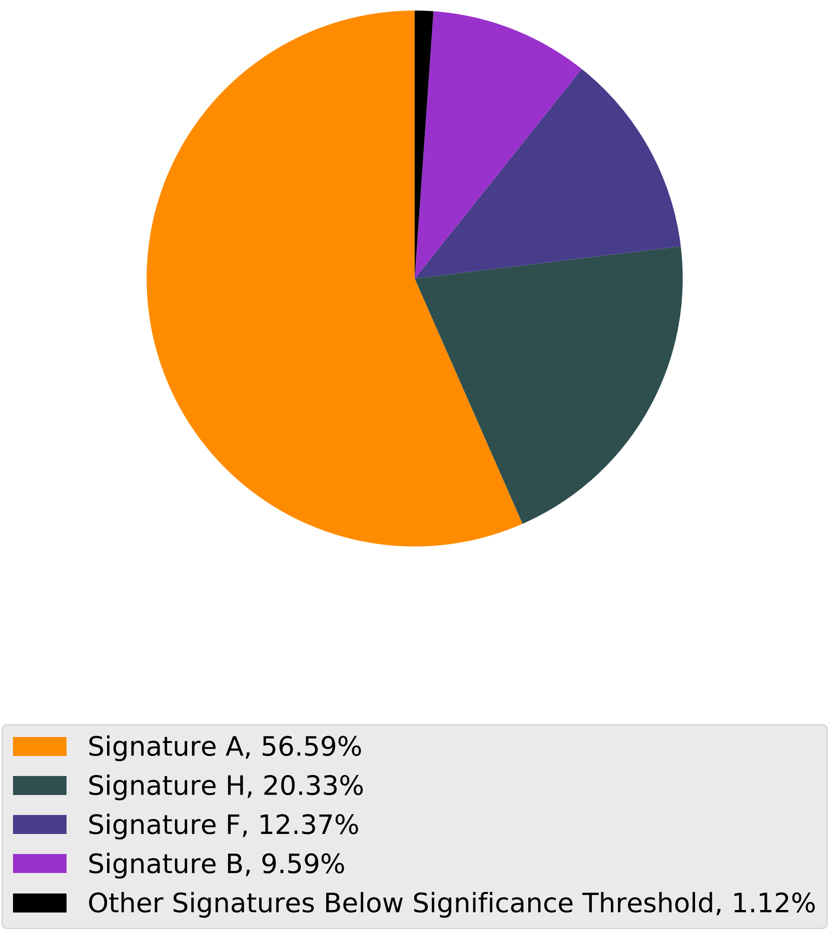

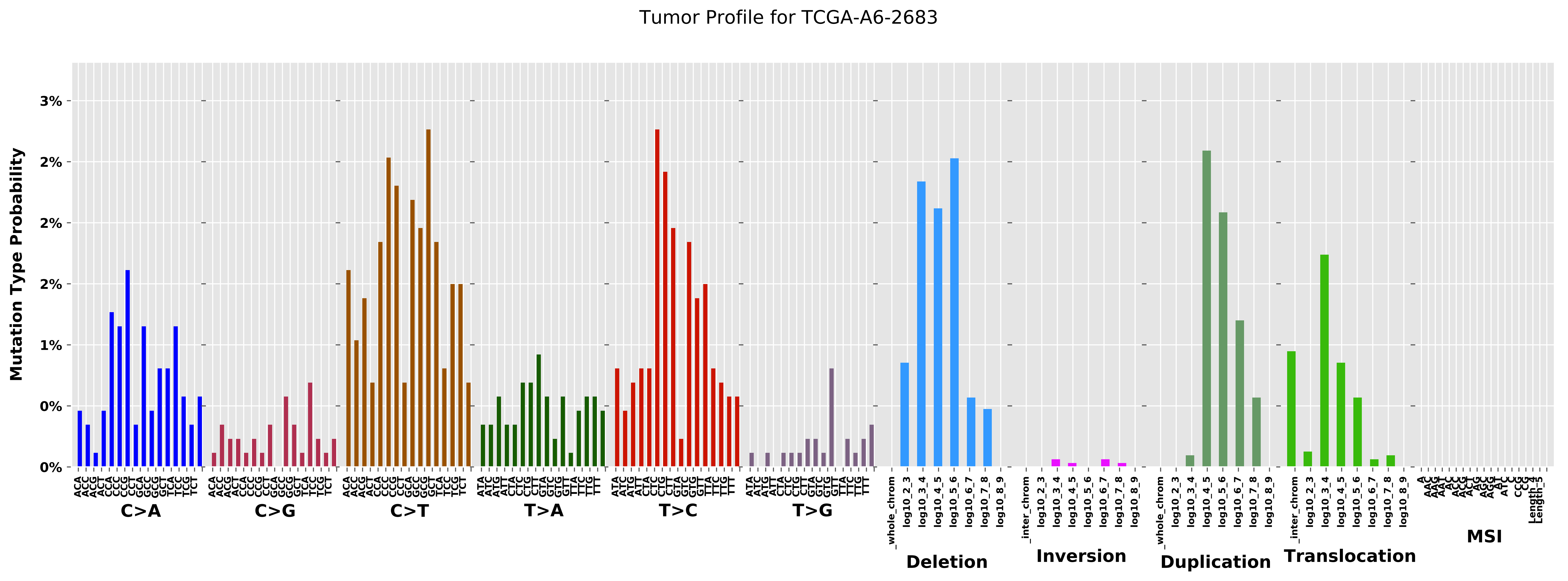
Tumor Profile for TCGA-A6-2683
Mutation Type Probability
3%
2%
2%
2%
1%
0%
0%
C>A
C>G
C>T
T>A
T>C
T>G
Deletion
Inversion
Duplication
Translocation
MSI

Cancer processes Weights for TCGA-AC-A2BK

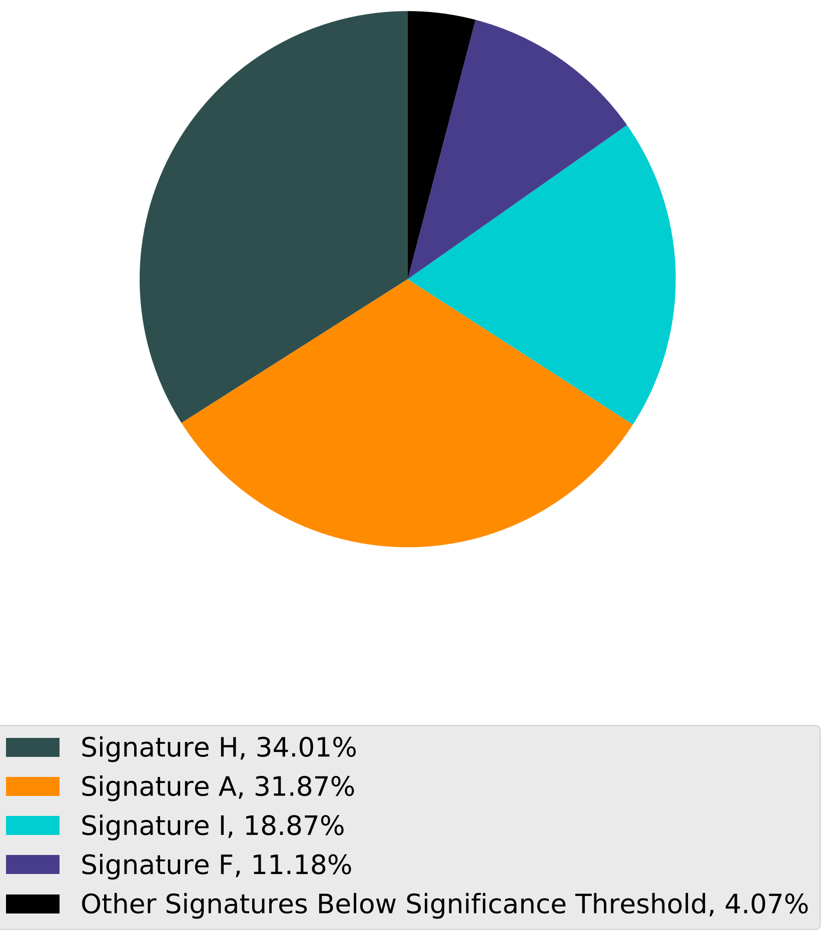

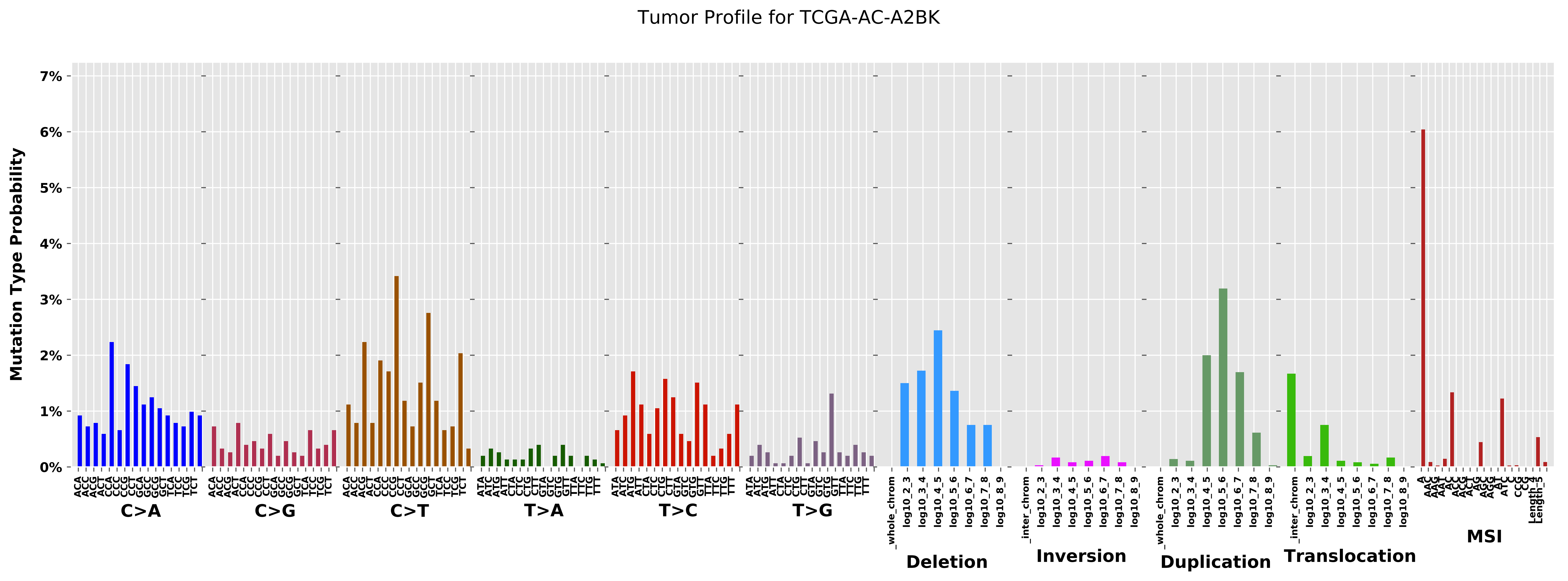
Tumor Profile for TCGA-AC-A2BK
Mutation Type Probability
7%
6%
5%
4%
3%
2%
1%
0%
C>A
C>G
C>T
T>A
T>C
T>G
Deletion
Inversion
Duplication
Translocation
MSI

Cancer processes Weights for TCGA-AG-A032

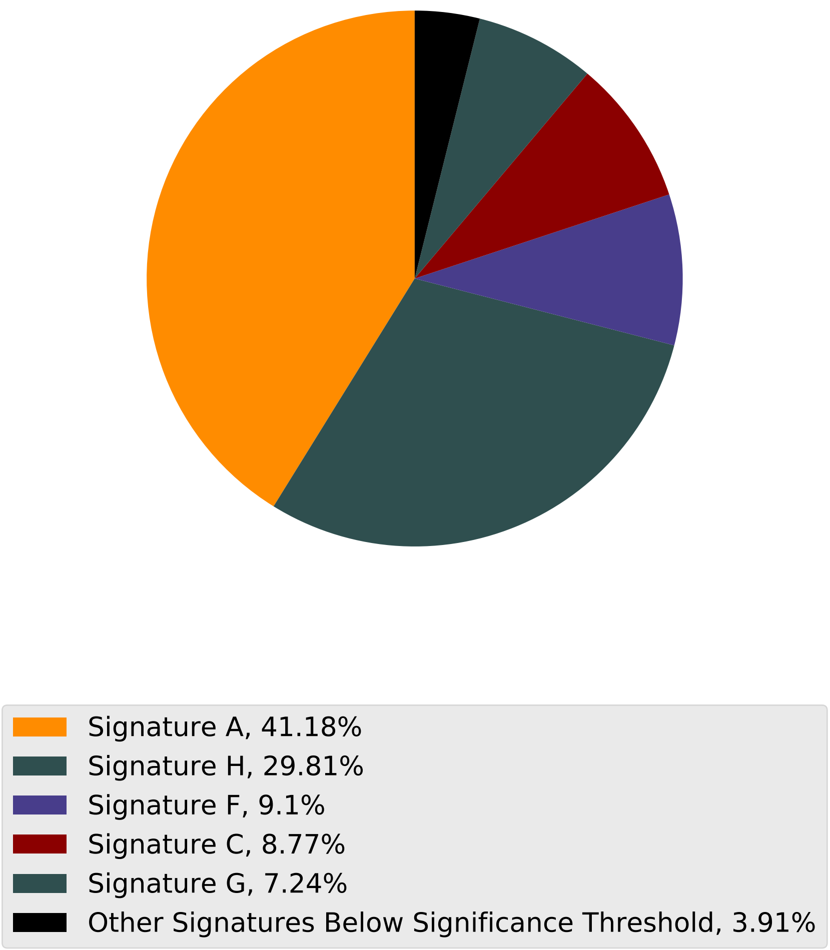

Tumor Profile for TCGA-AG-A032

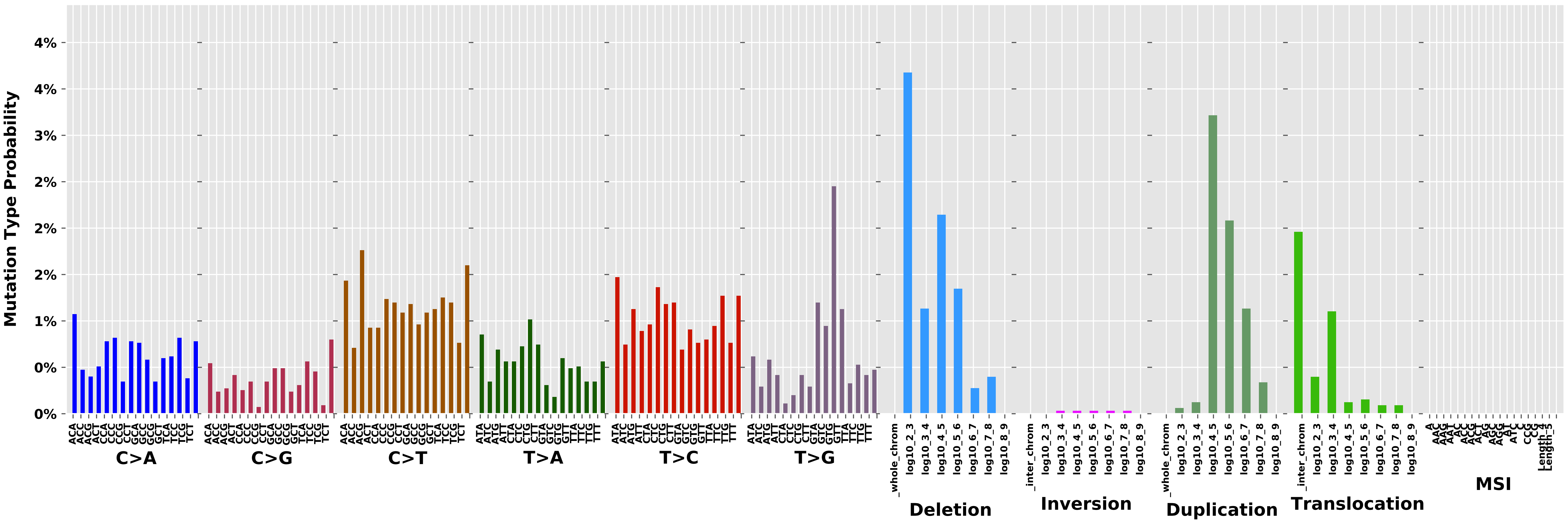

Cancer processes Weights for TCGA-B6-A0I2

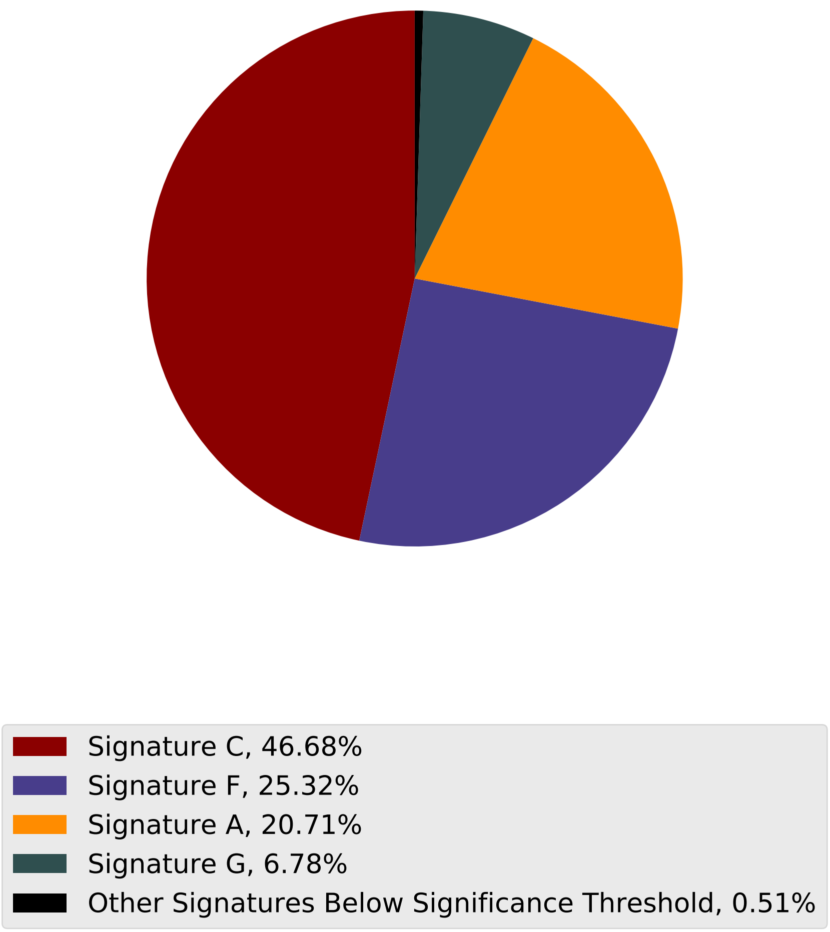

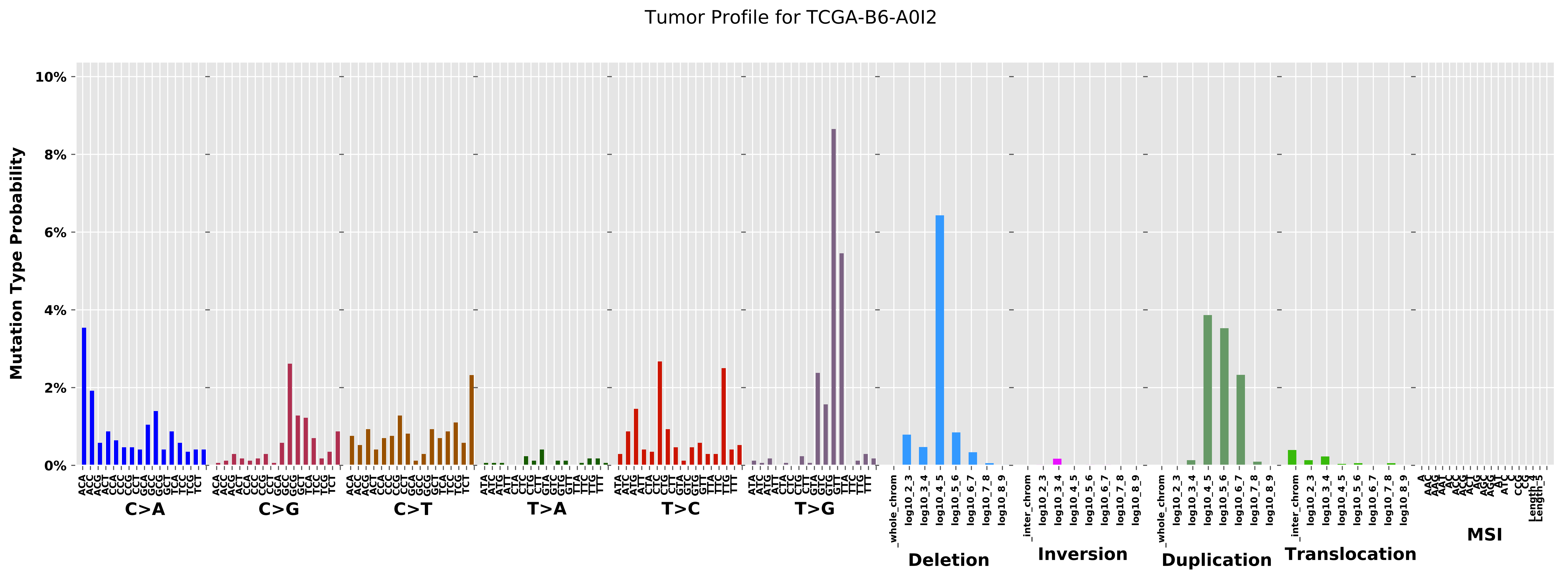

Tumor Profile for TCGA-B6-A0I2
Mutation Type Probability
10%
8%
6%
4%
2%
0%
C>A
C>G
C>T
T>A
T>C
T>G
Deletion
Inversion
Duplication
Translocation
MSI

Cancer processes Weights for TCGA-A8-A094

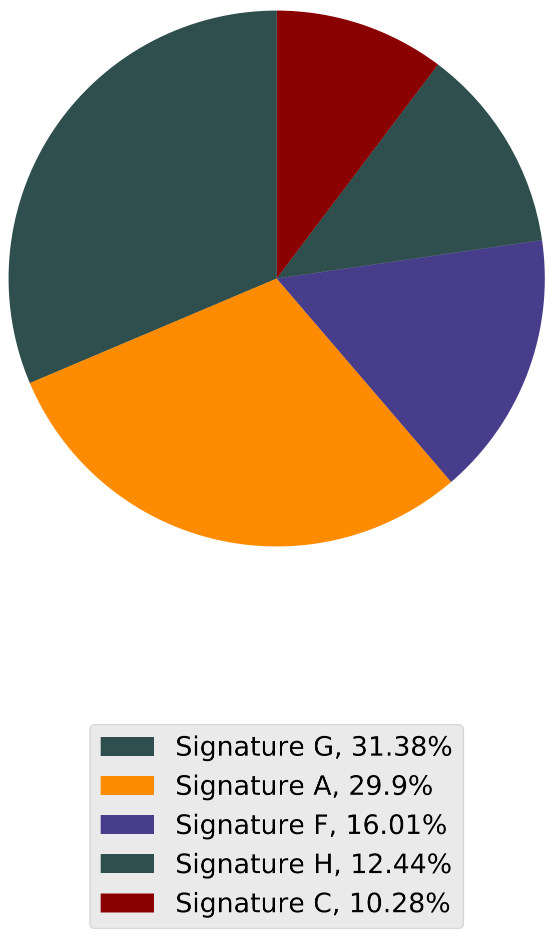

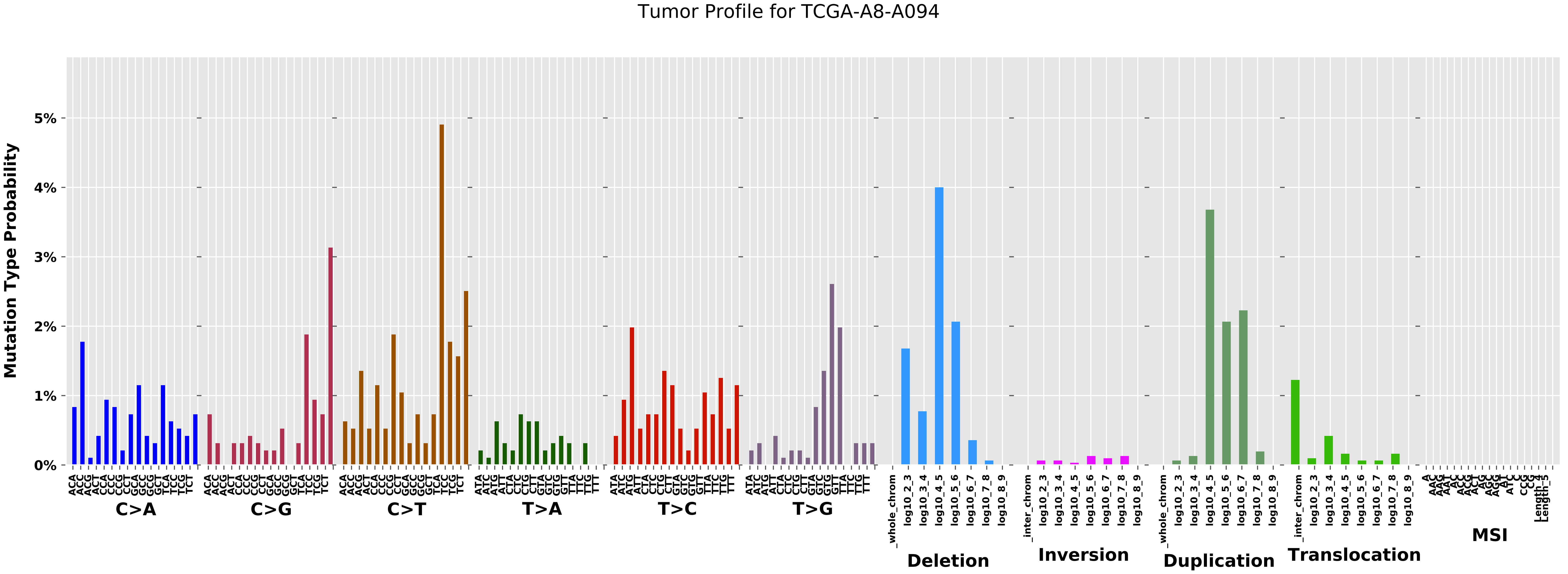

Tumor Profile for TCGA-A8-A094
Mutation Type Probability
0%
1%
2%
3%
4%
5%
C>A
C>G
C>T
T>A
T>C
T>G
Deletion
Inversion
Duplication
Translocation
MSI

Cancer processes Weights for TCGA-AA-3994

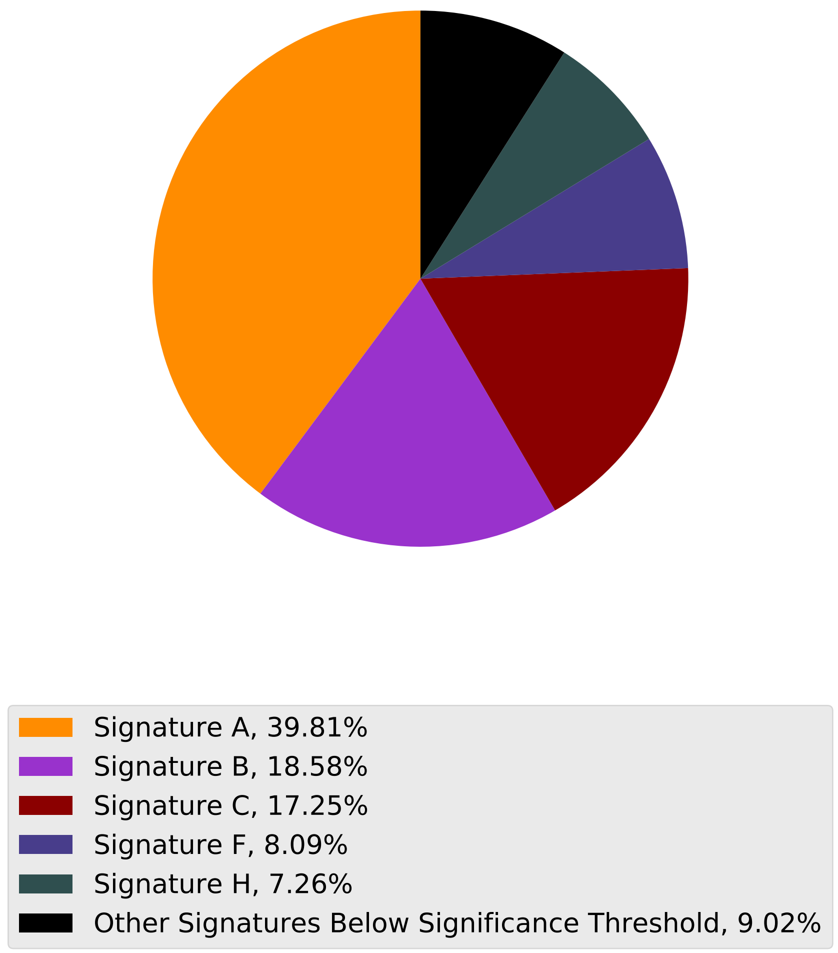

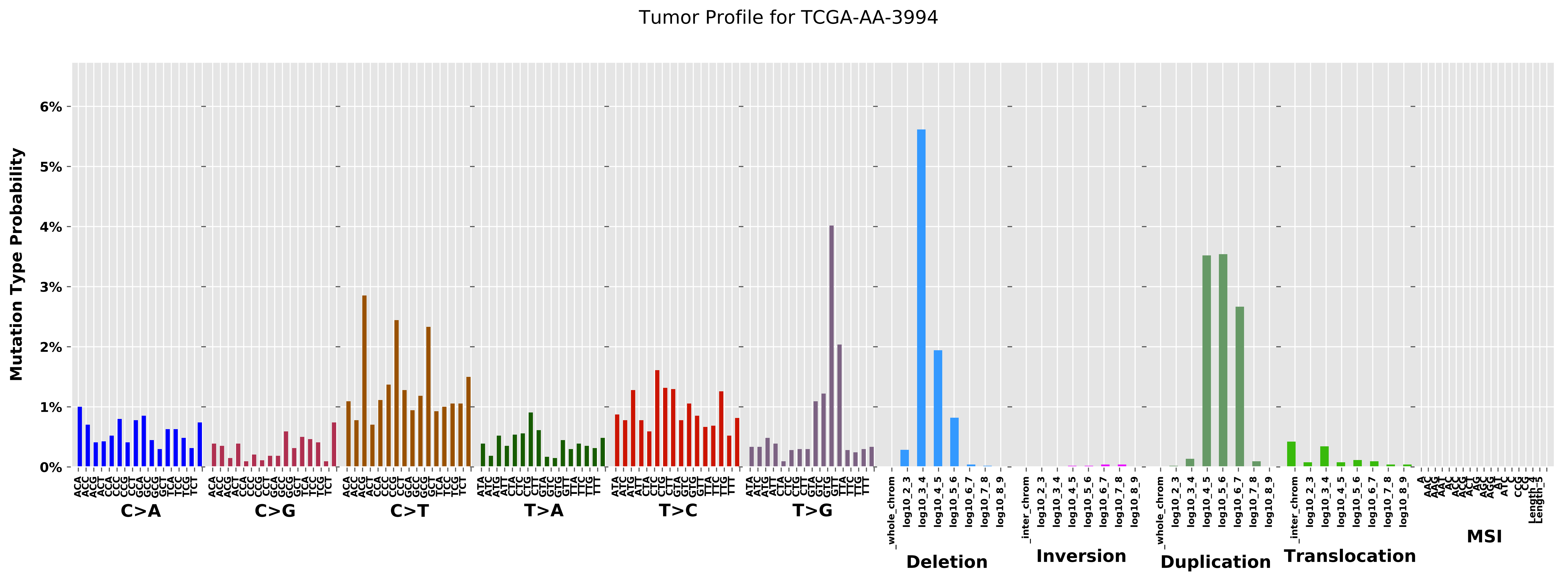
Tumor Profile for TCGA-AA-3994
Mutation Type Probability
6%
5%
4%
3%
2%
1%
0%
C>A
C>G
C>T
T>A
T>C
T>G
Deletion
Inversion
Duplication
Translocation
MSI

Cancer processes Weights for TCGA-AG-3574

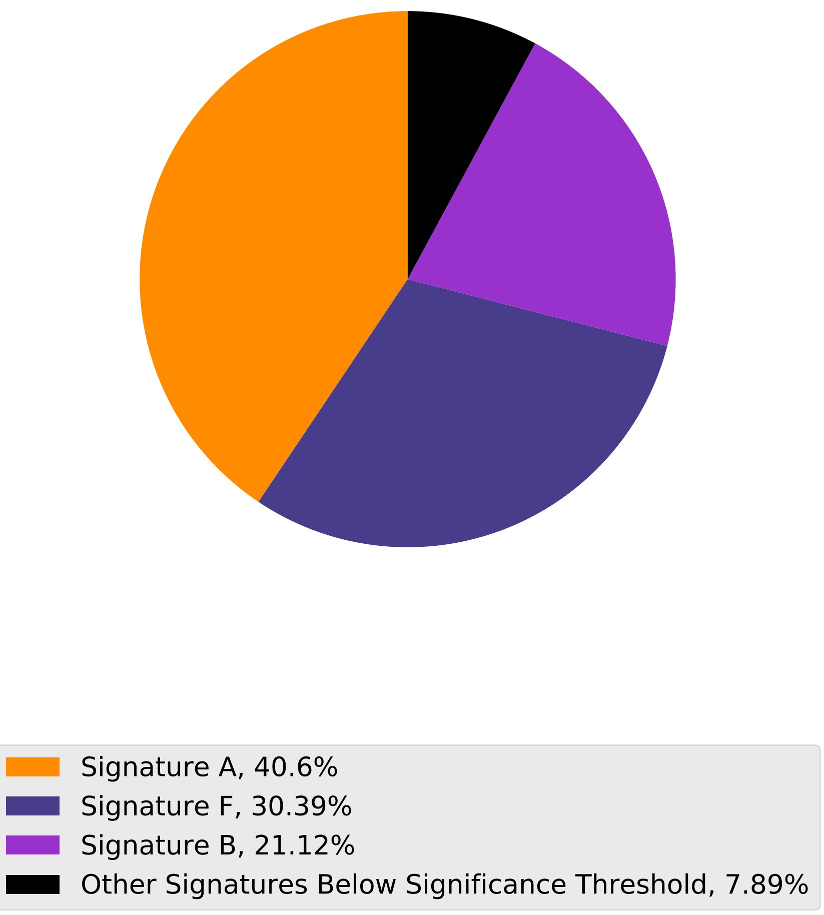

Tumor Profile for TCGA-AG-3574

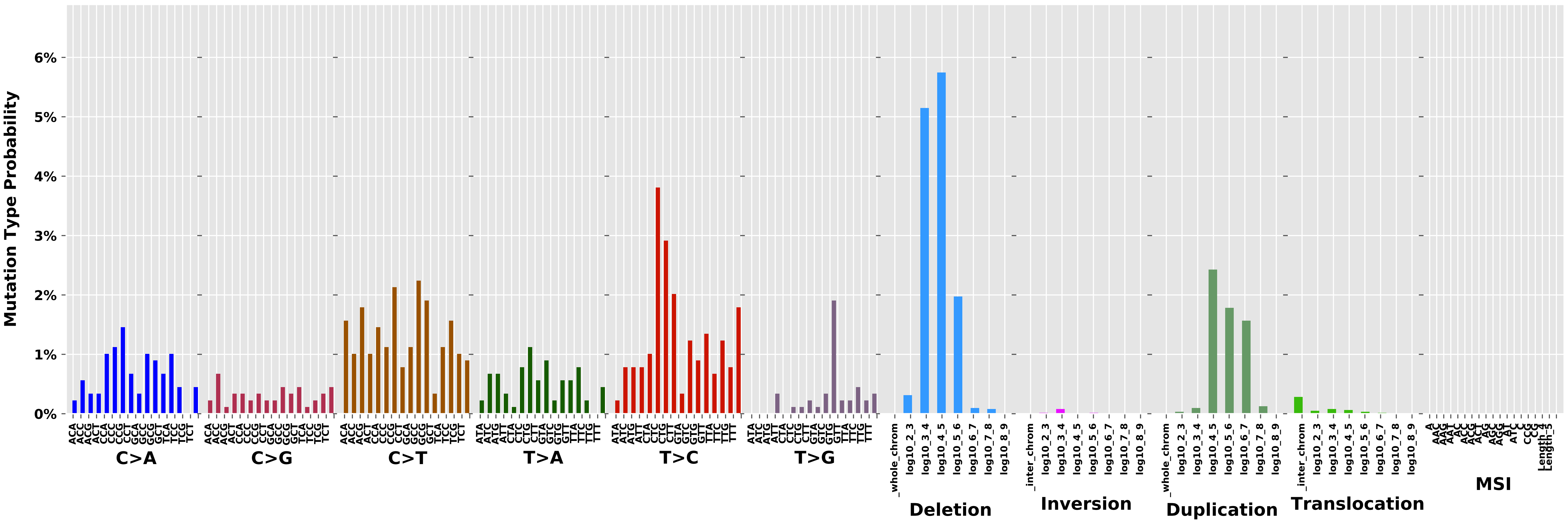

Cancer processes Weights for TCGA-AO-A03L

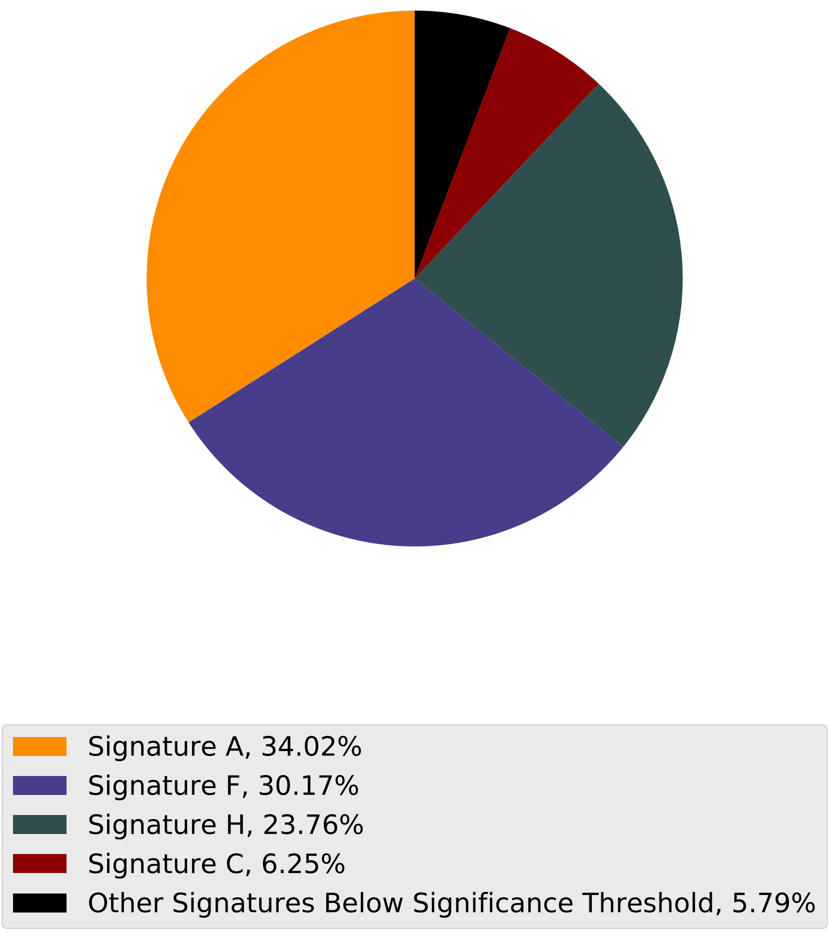

Tumor Profile for TCGA-AO-A03L

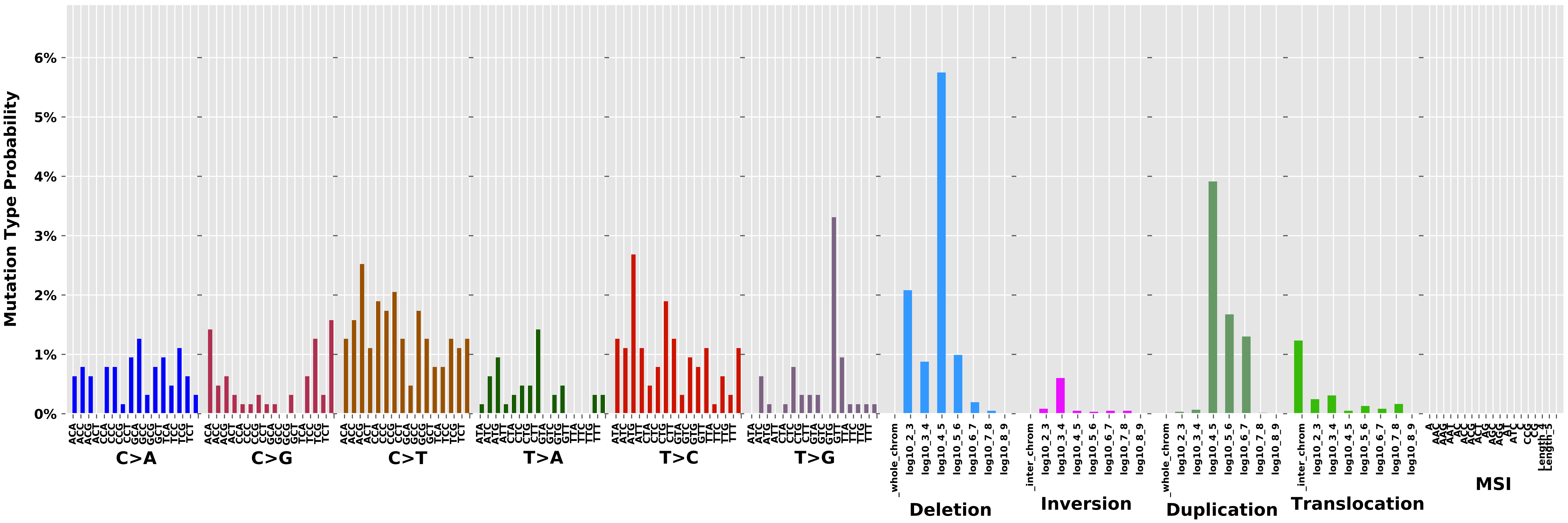

Cancer processes Weights for TCGA-A6-6141

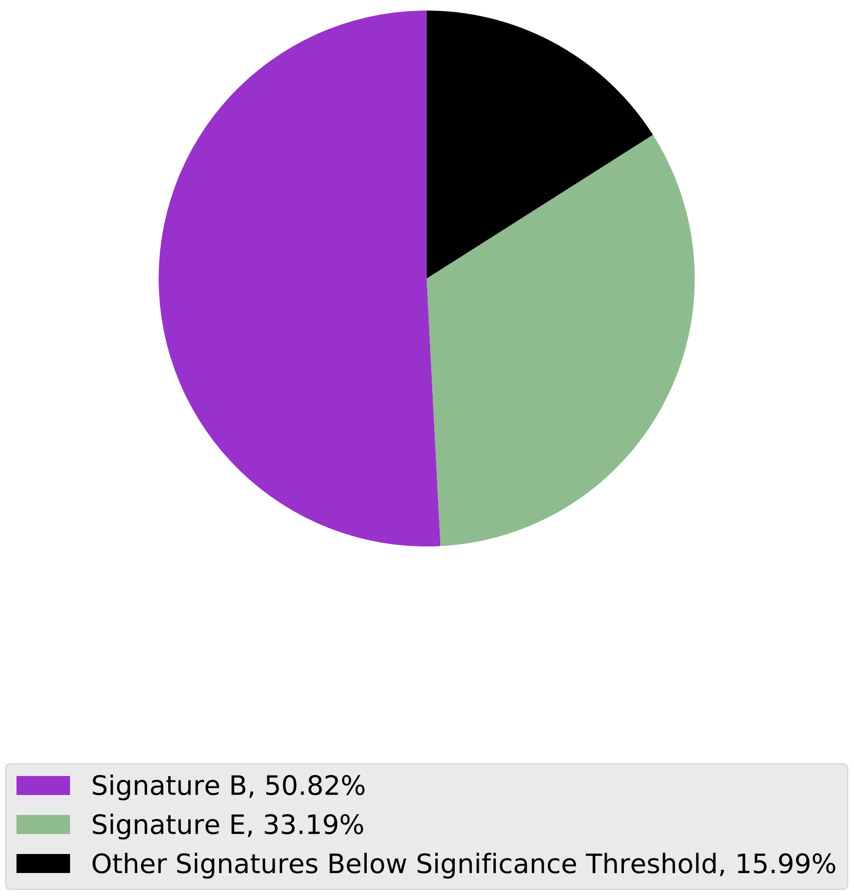

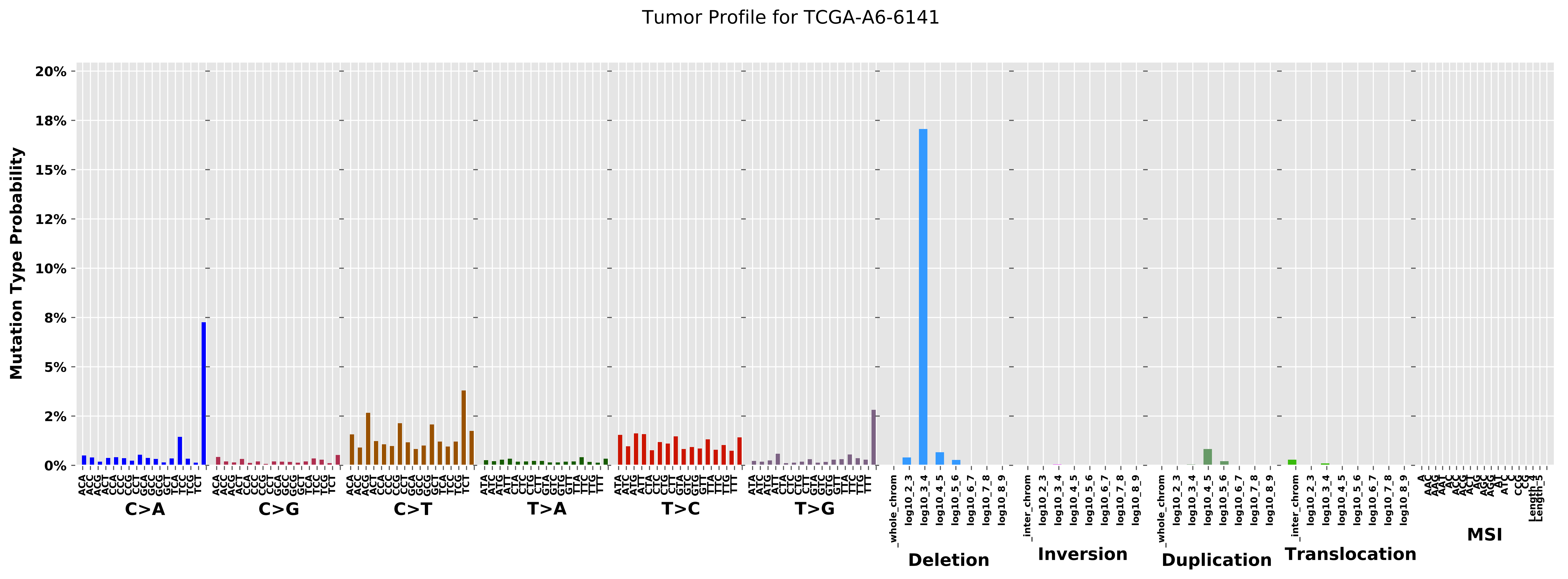

Tumor Profile for TCGA-A6-6141
Mutation Type Probability
20%
18%
15%
12%
10%
8%
5%
2%
0%
C>A
C>G
C>T
T>A
T>C
T>G
Deletion
Inversion
Duplication
Translocation
MSI

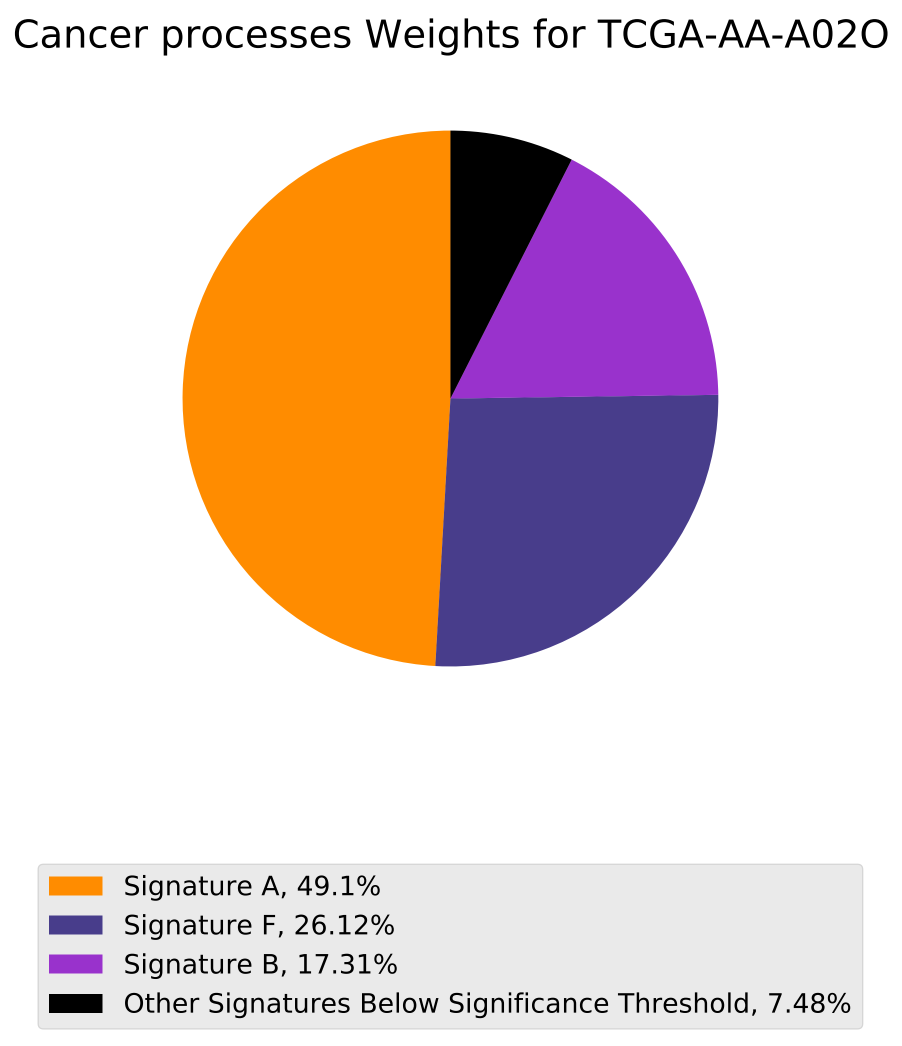
Cancer processes Weights for TCGA-AA-A02O
Signature A, 49.1%
Signature F, 26.12%
Signature B, 17.31%
Other Signatures Below Significance Threshold, 7.48%

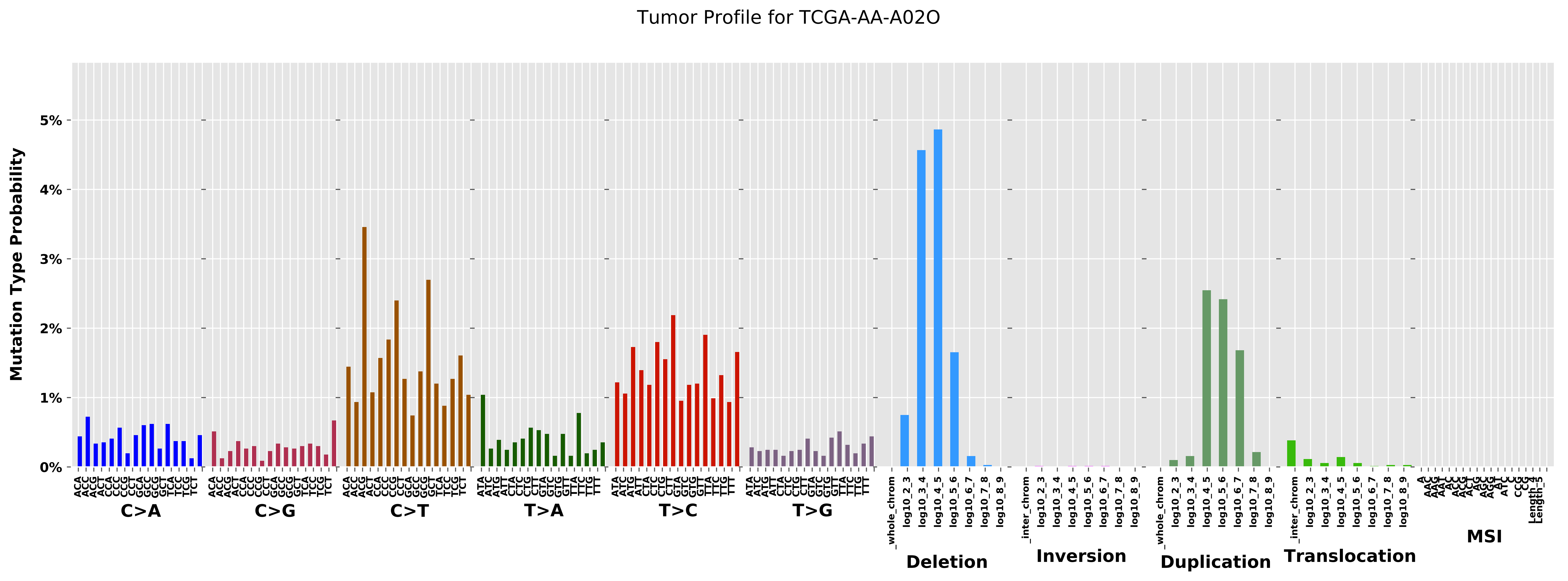

Tumor Profile for TCGA-AA-A02O
Mutation Type Probability
5%
4%
3%
2%
1%
0%
C>A
C>G
C>T
T>A
T>C
T>G
Deletion
Inversion
Duplication
Translocation
MSI

Cancer processes Weights for TCGA-AG-3885

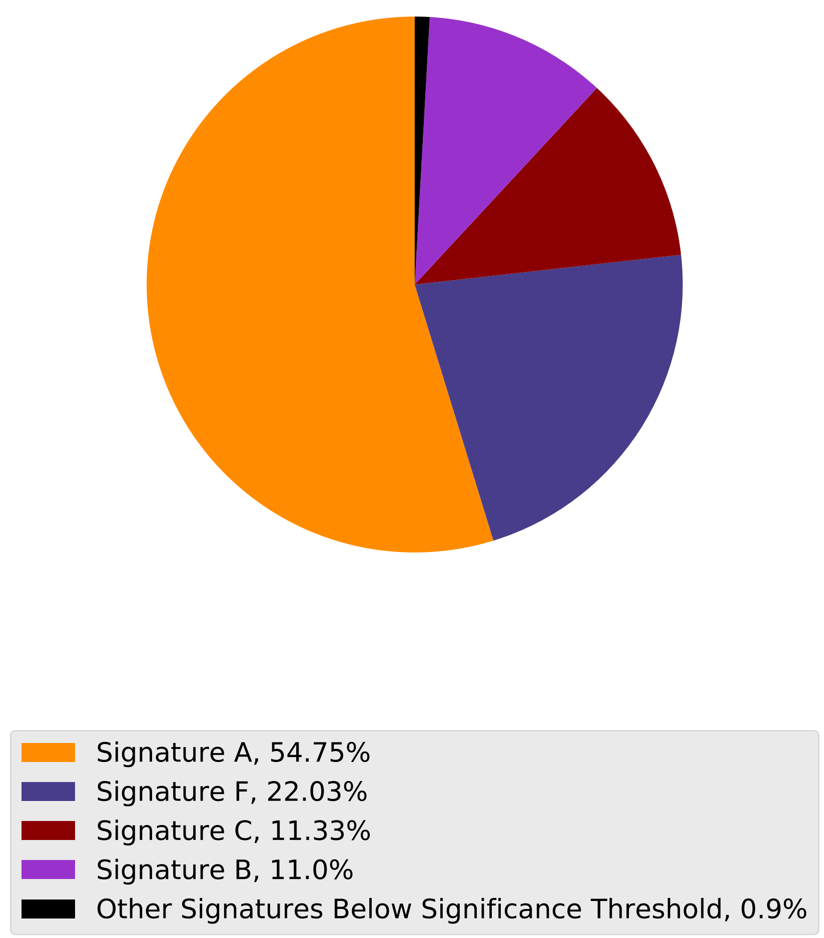

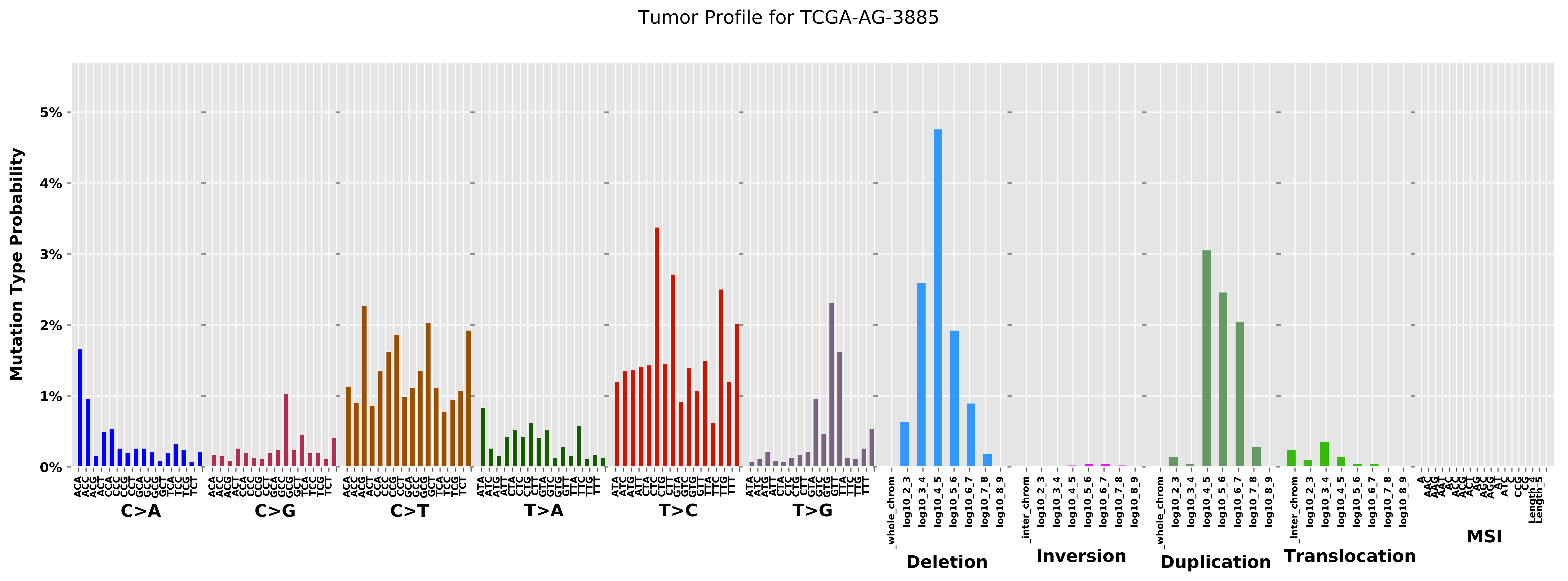
Tumor Profile for TCGA-AG-3885
Mutation Type Probability
0%
1%
2%
3%
4%
5%
C>A
C>G
C>T
T>A
T>C
T>G
Deletion
Inversion
Duplication
Translocation
MSI

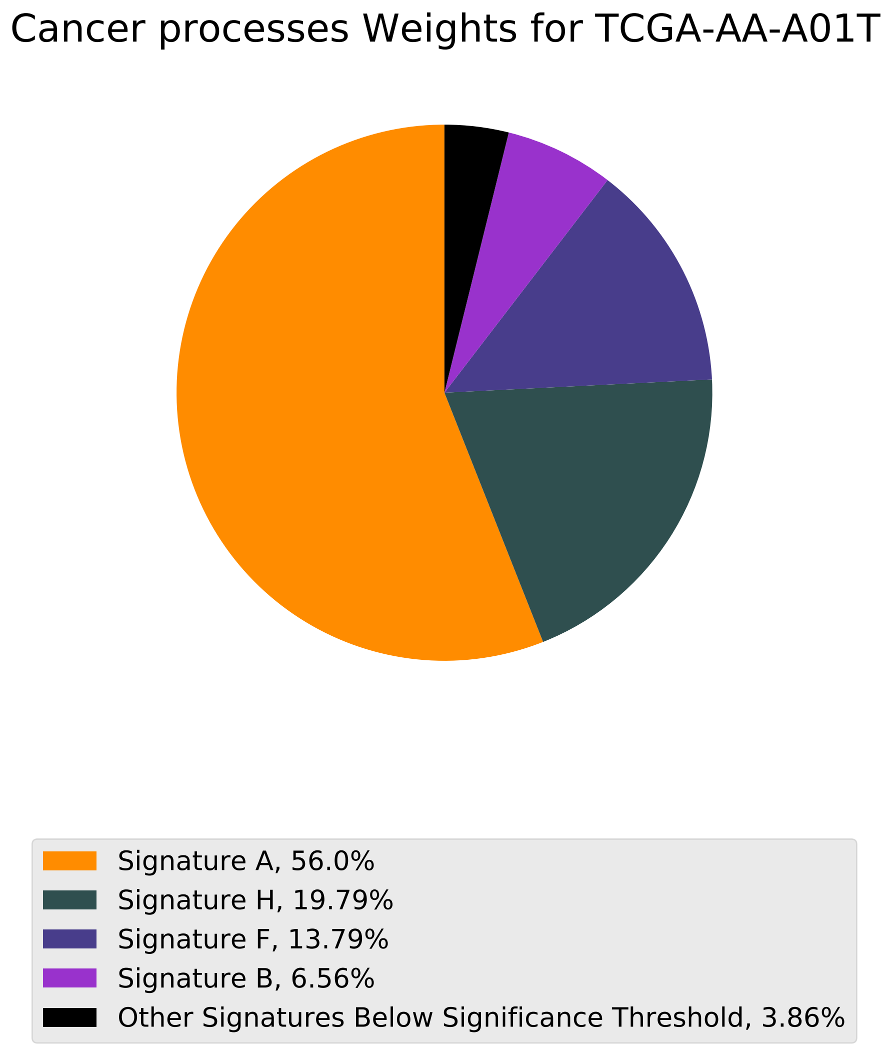
Cancer processes Weights for TCGA-AA-A01T
Signature A, 56.0%
Signature H, 19.79%
Signature F, 13.79%
Signature B, 6.56%
Other Signatures Below Significance Threshold, 3.86%

Tumor Profile for TCGA-AA-A01T

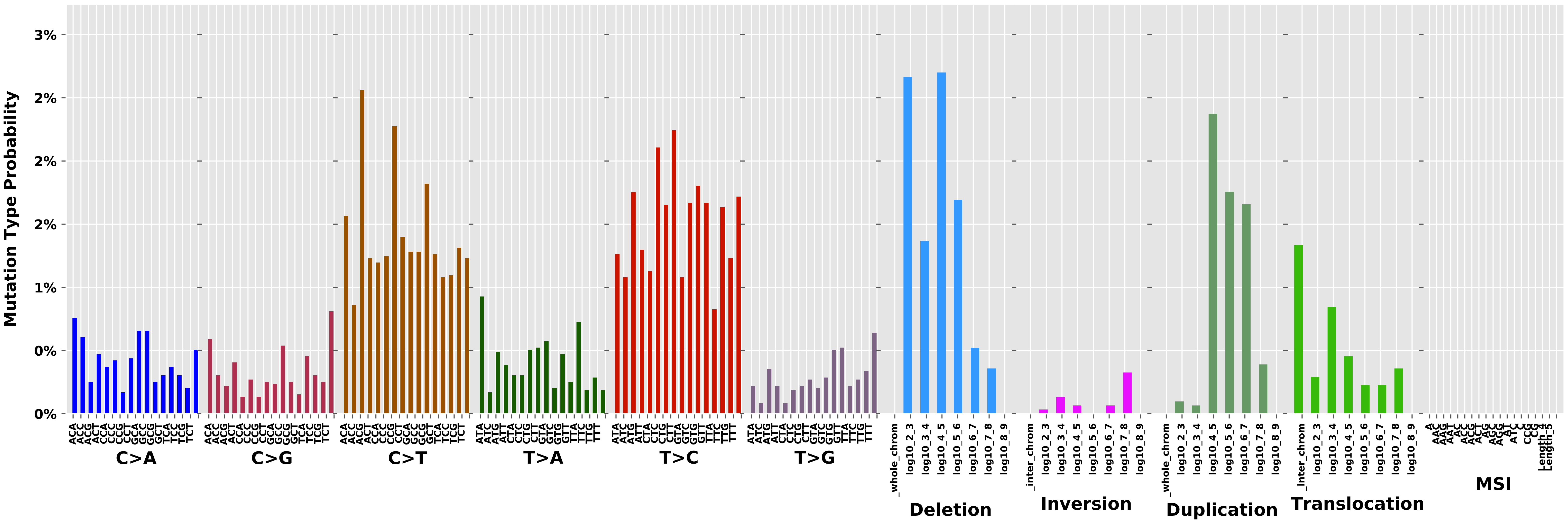

Cancer processes Weights for TCGA-A8-A07I

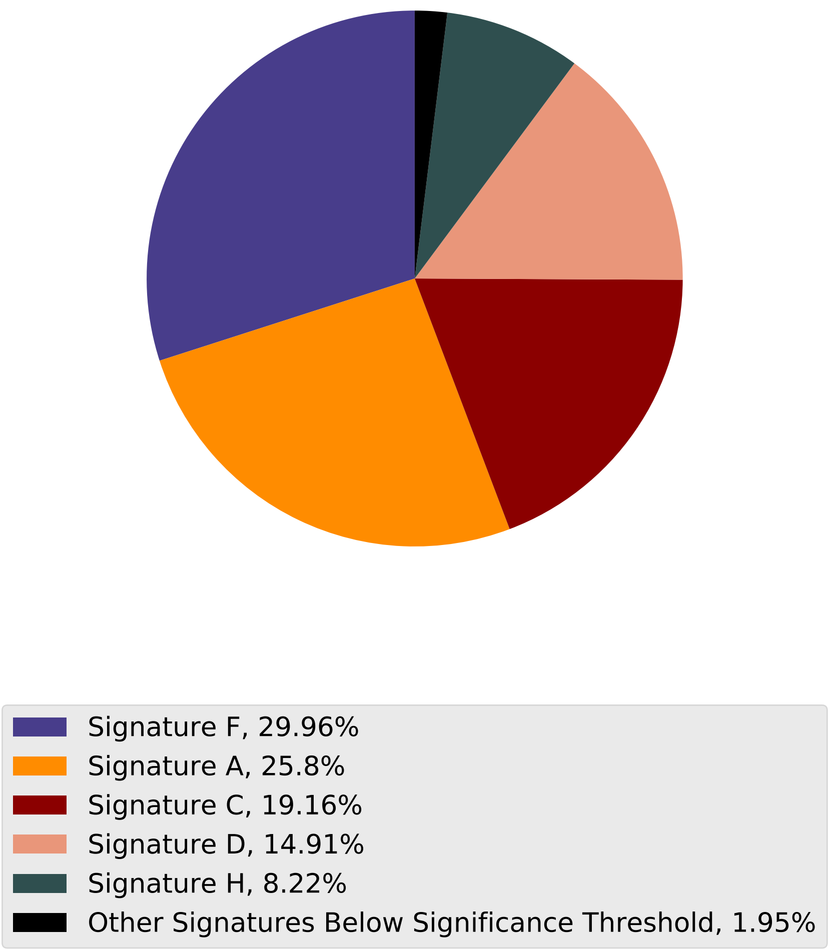

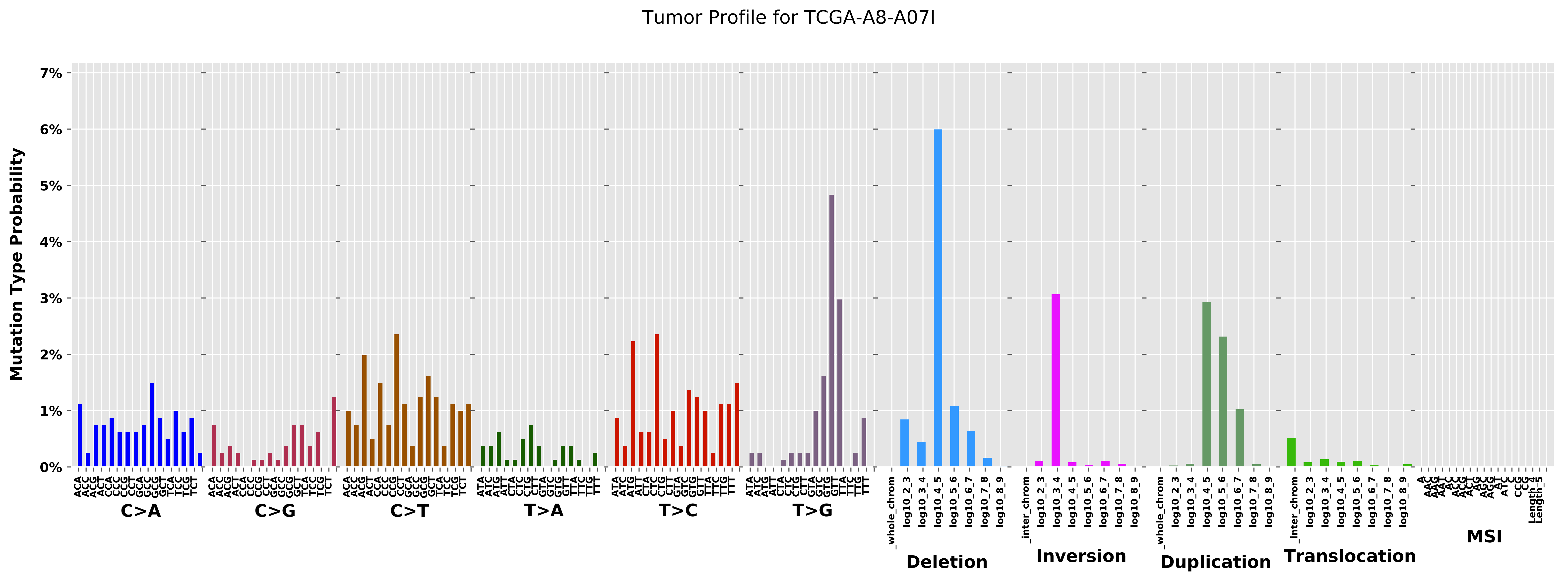
Tumor Profile for TCGA-A8-A07I
Mutation Type Probability
0%
1%
2%
3%
4%
5%
6%
7%
C>A
C>G
C>T
T>A
T>C
T>G
Deletion
Inversion
Duplication
Translocation
MSI
_whole_chrom
_inter_chrom
log10_2_3
log10_3_4
log10_4_5
log10_5_6
log10_6_7
log10_7_8
log10_8_9

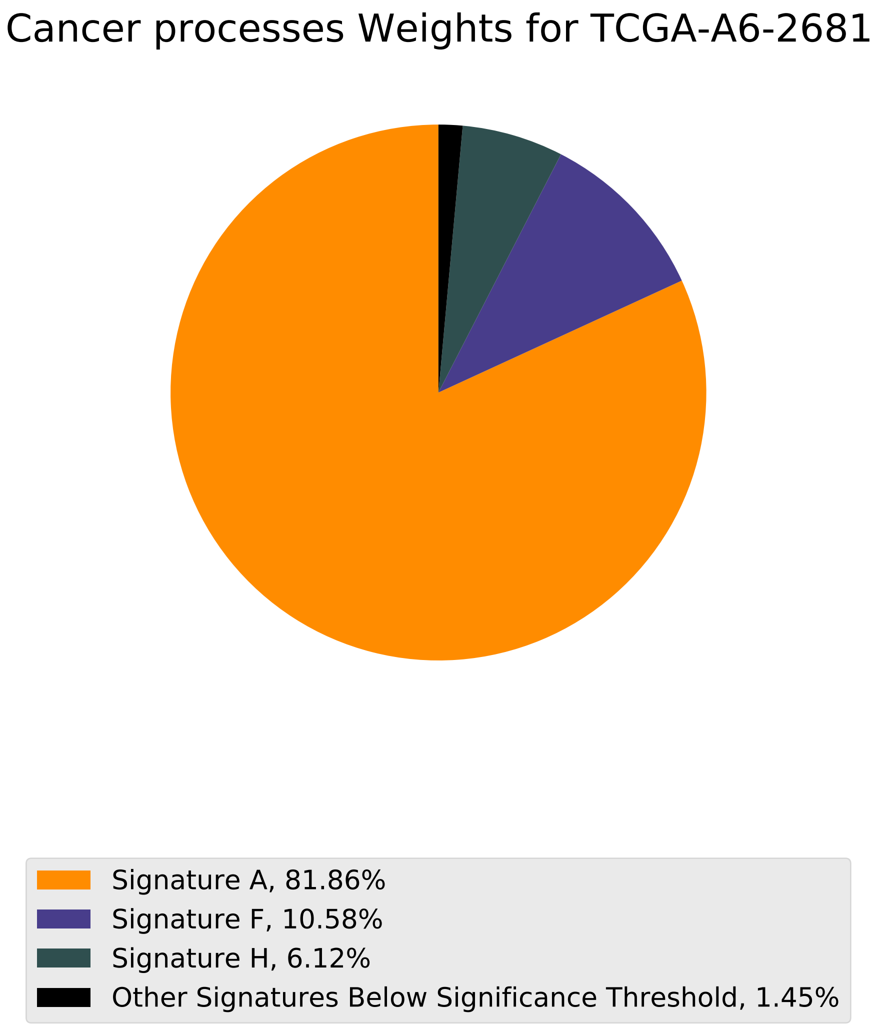
Cancer processes Weights for TCGA-A6-2681
Signature A, 81.86%
Signature F, 10.58%
Signature H, 6.12%
Other Signatures Below Significance Threshold, 1.45%

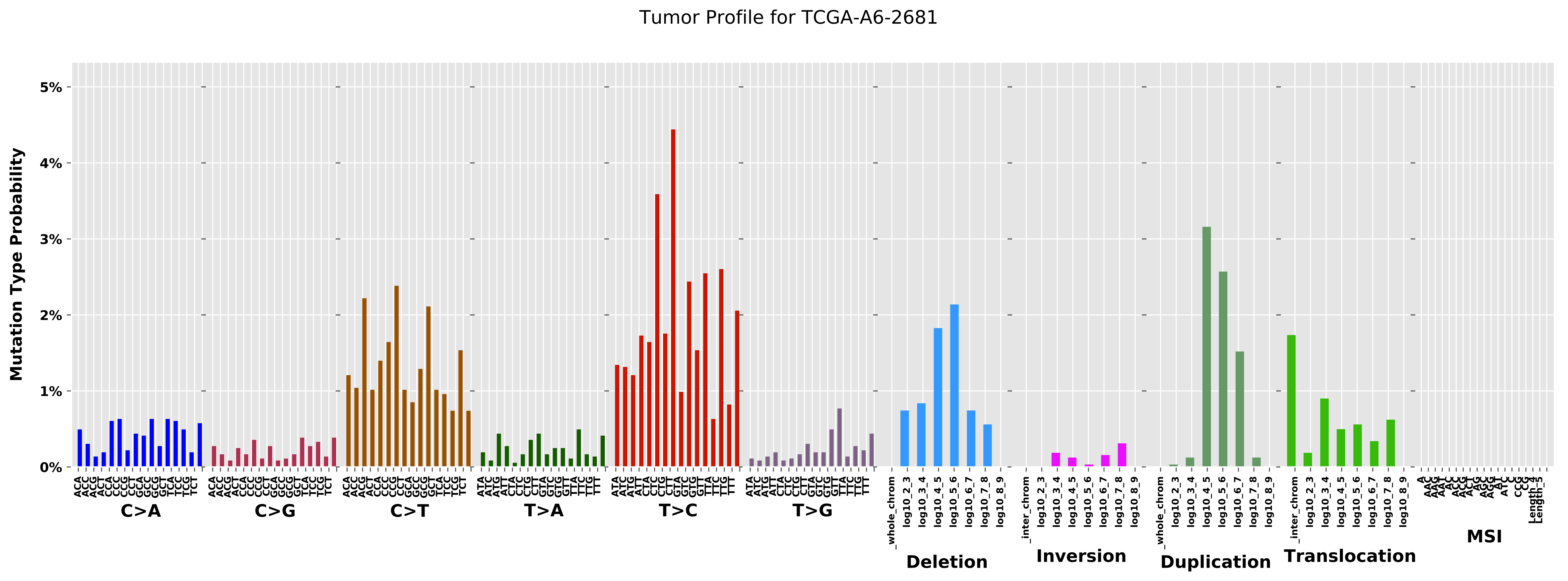

Tumor Profile for TCGA-A6-2681
Mutation Type Probability
0%
1%
2%
3%
4%
5%
C>A
C>G
C>T
T>A
T>C
T>G
Deletion
Inversion
Duplication
Translocation
MSI

Cancer processes Weights for TCGA-AO-A124

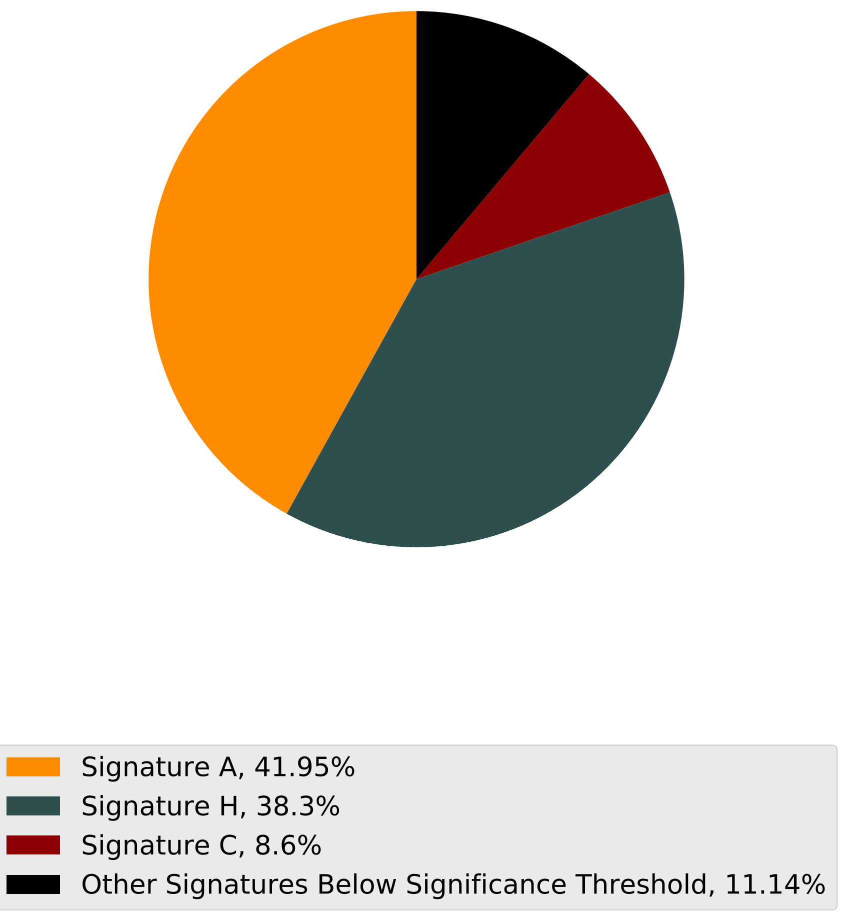

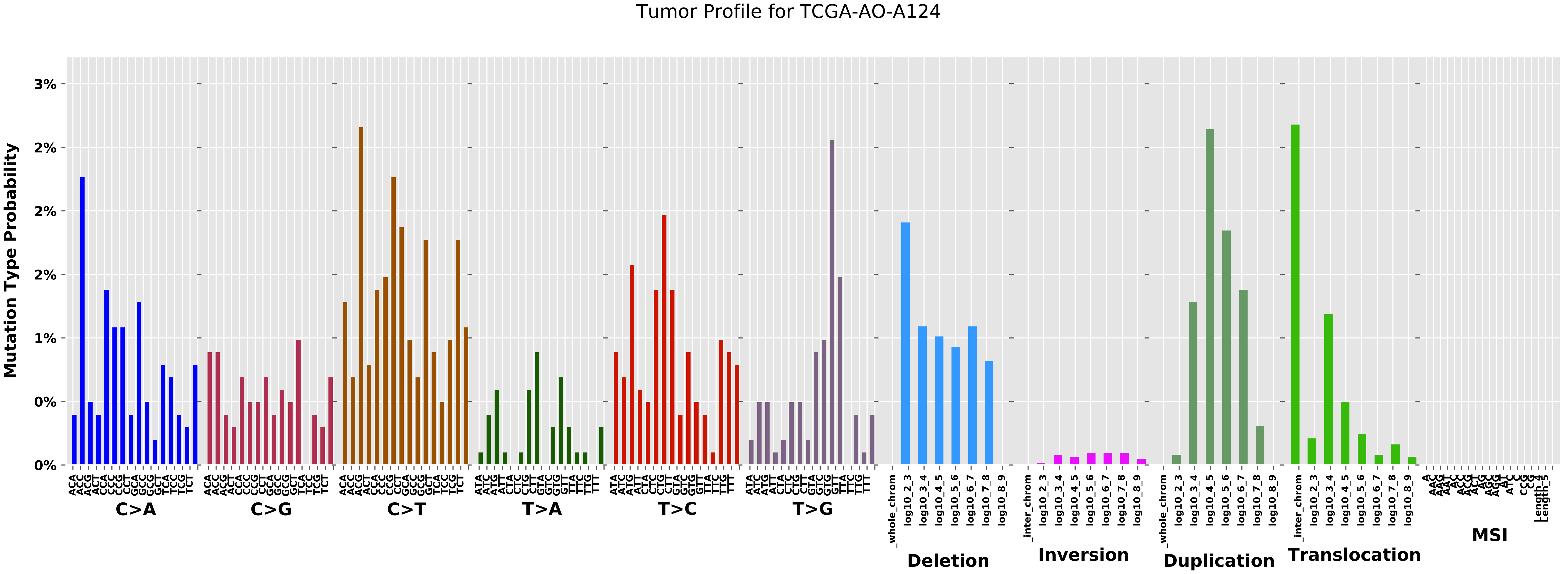
Tumor Profile for TCGA-AO-A124
Mutation Type Probability
3%
2%
2%
2%
1%
0%
0%
C>A
C>G
C>T
T>A
T>C
T>G
Deletion
Inversion
Duplication
Translocation
MSI
_whole_chrom
_inter_chrom
log10_2_3
log10_3_4
log10_4_5
log10_5_6
log10_6_7
log10_7_8
log10_8_9

Cancer processes Weights for TCGA-A2-A259

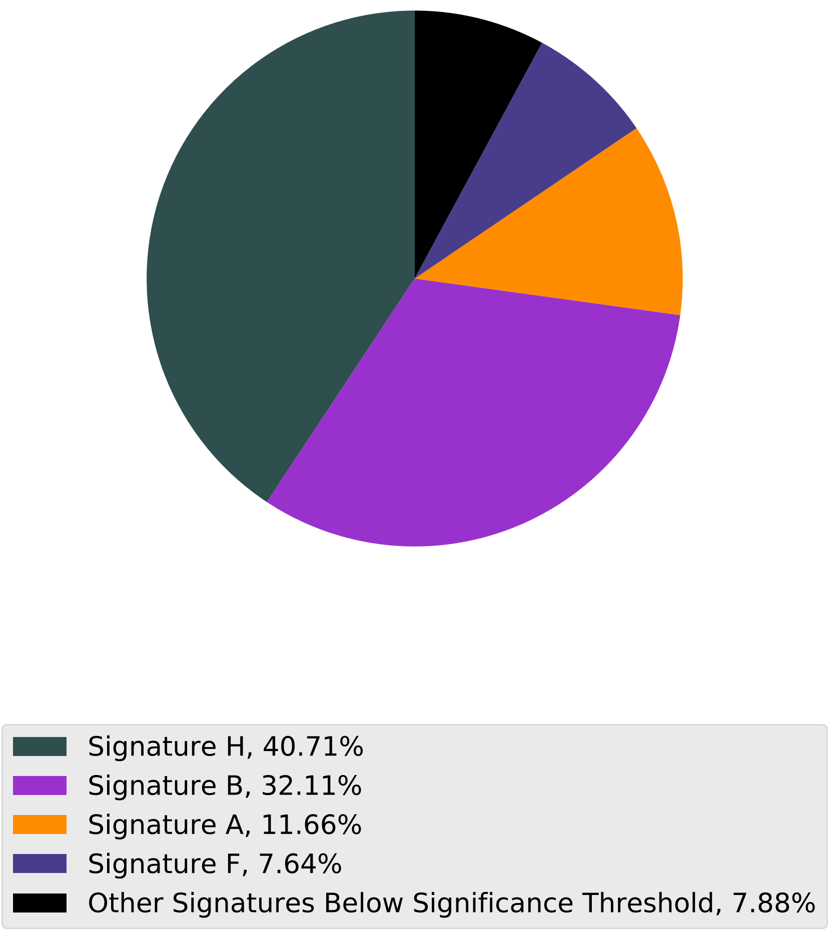

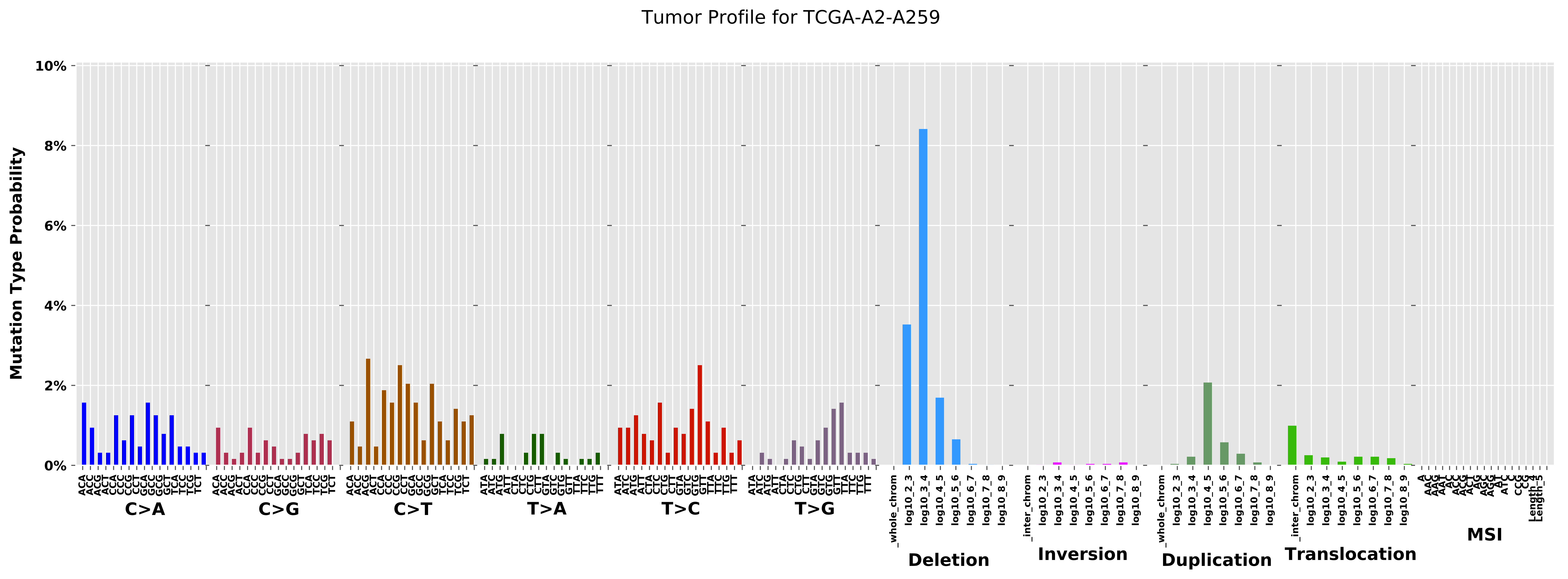
Tumor Profile for TCGA-A2-A259
Mutation Type Probability
0%
2%
4%
6%
8%
10%
C>A
C>G
C>T
T>A
T>C
T>G
Deletion
Inversion
Duplication
Translocation
MSI
_whole_chrom
_inter_chrom
log10_2_3
log10_3_4
log10_4_5
log10_5_6
log10_6_7
log10_7_8
log10_8_9

Cancer processes Weights for TCGA-BH-A0DG

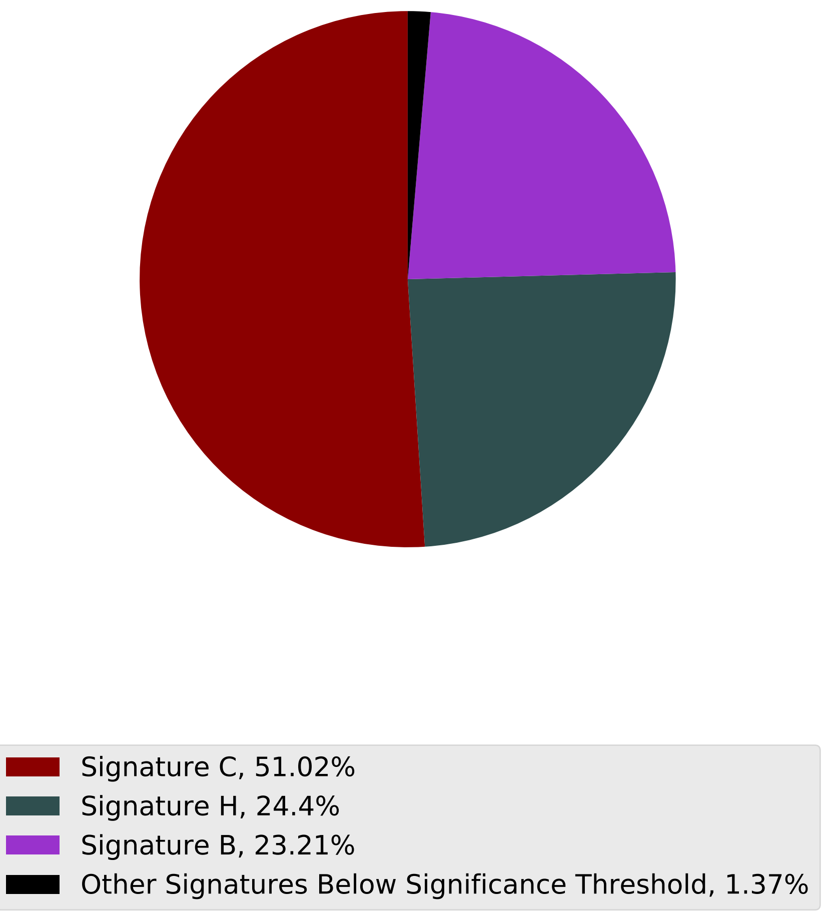

Tumor Profile for TCGA-BH-A0DG

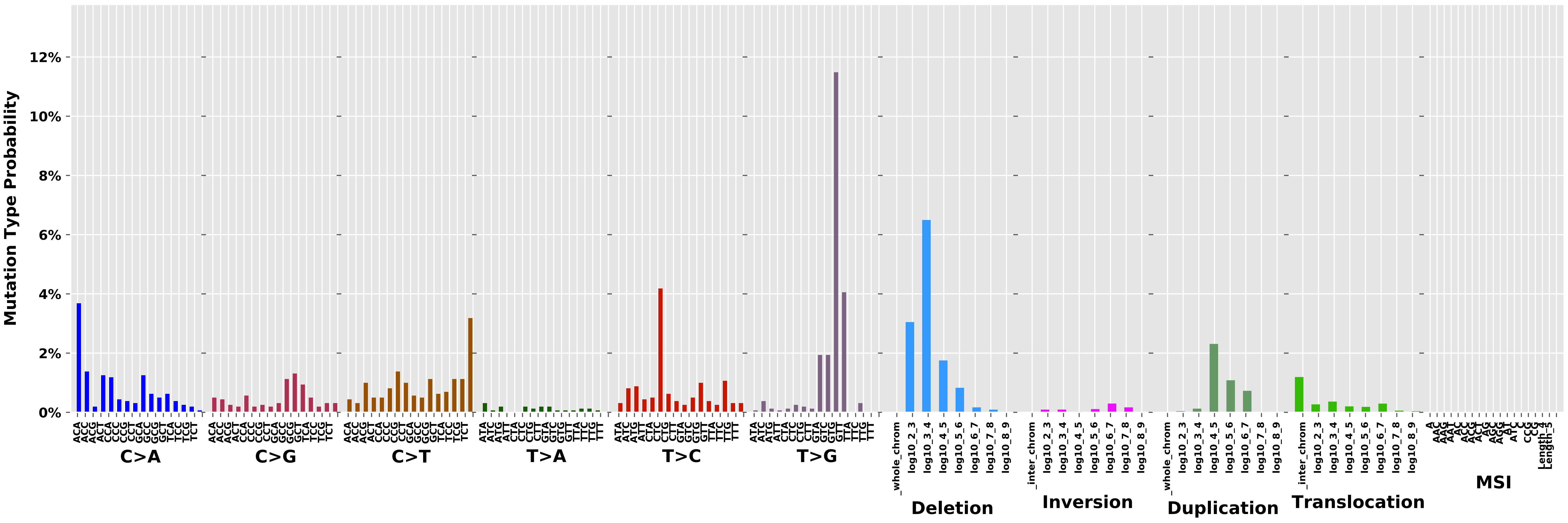

Cancer processes Weights for TCGA-A2-A3KC

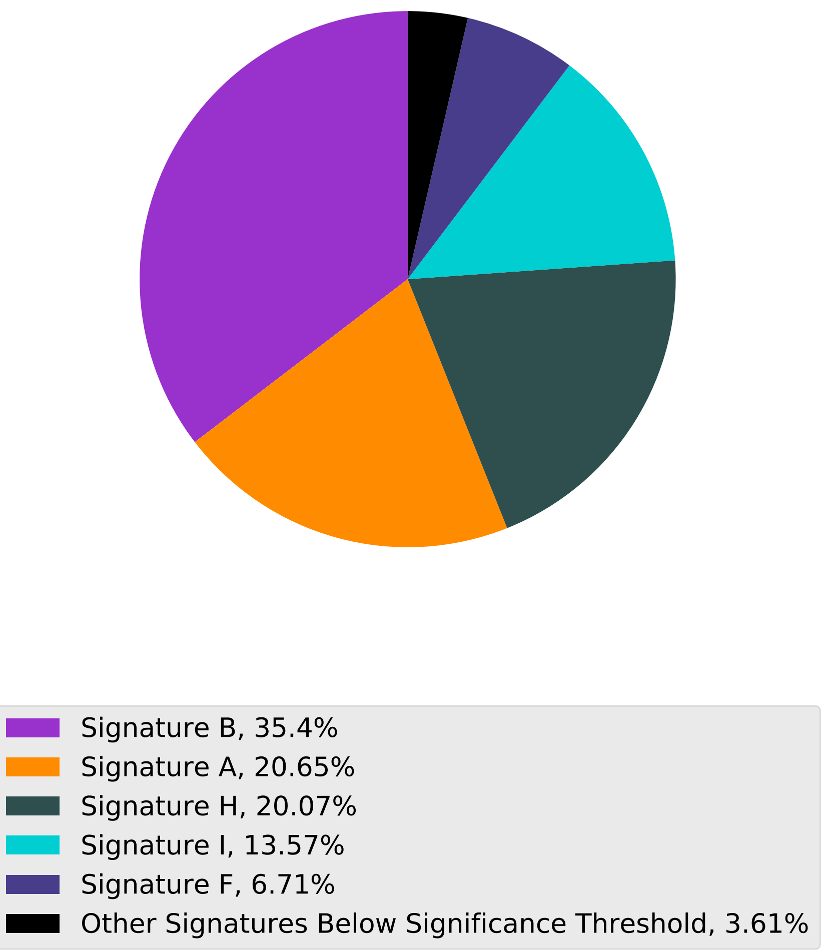

Tumor Profile for TCGA-A2-A3KC

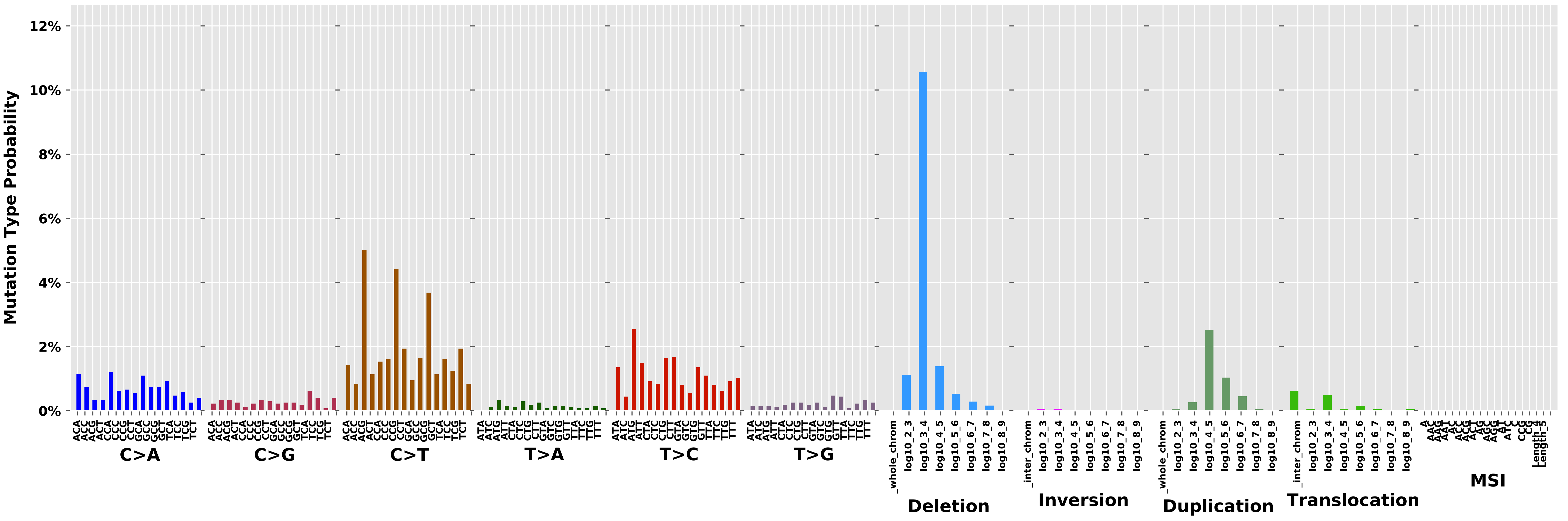

Cancer processes Weights for TCGA-AA-3514

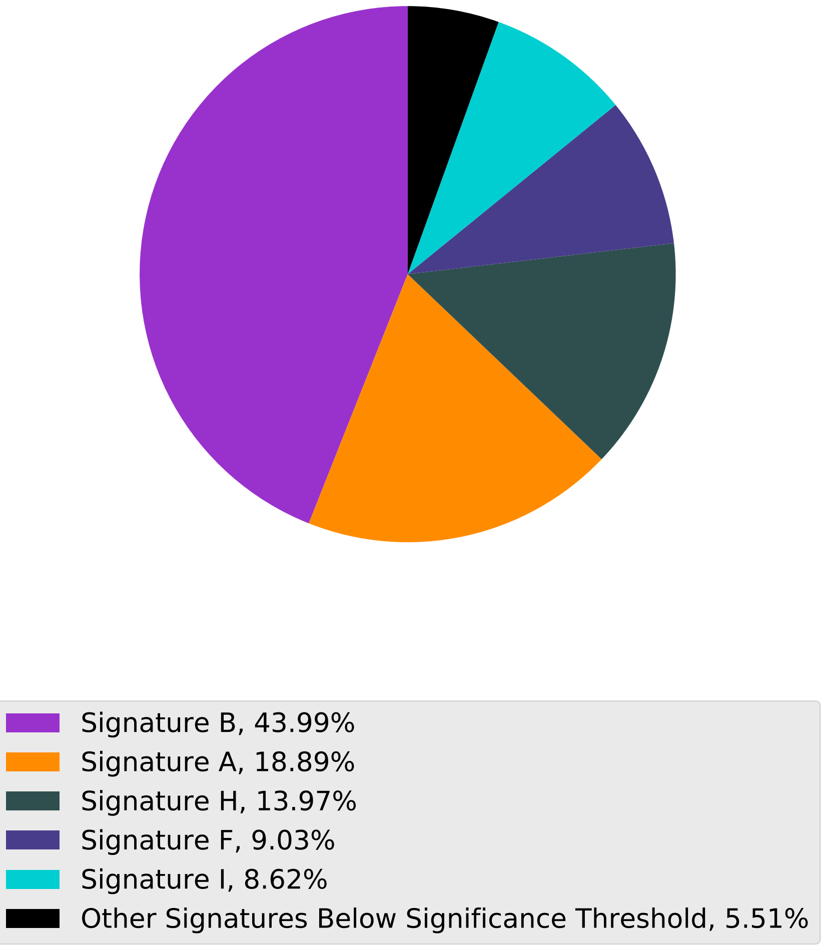

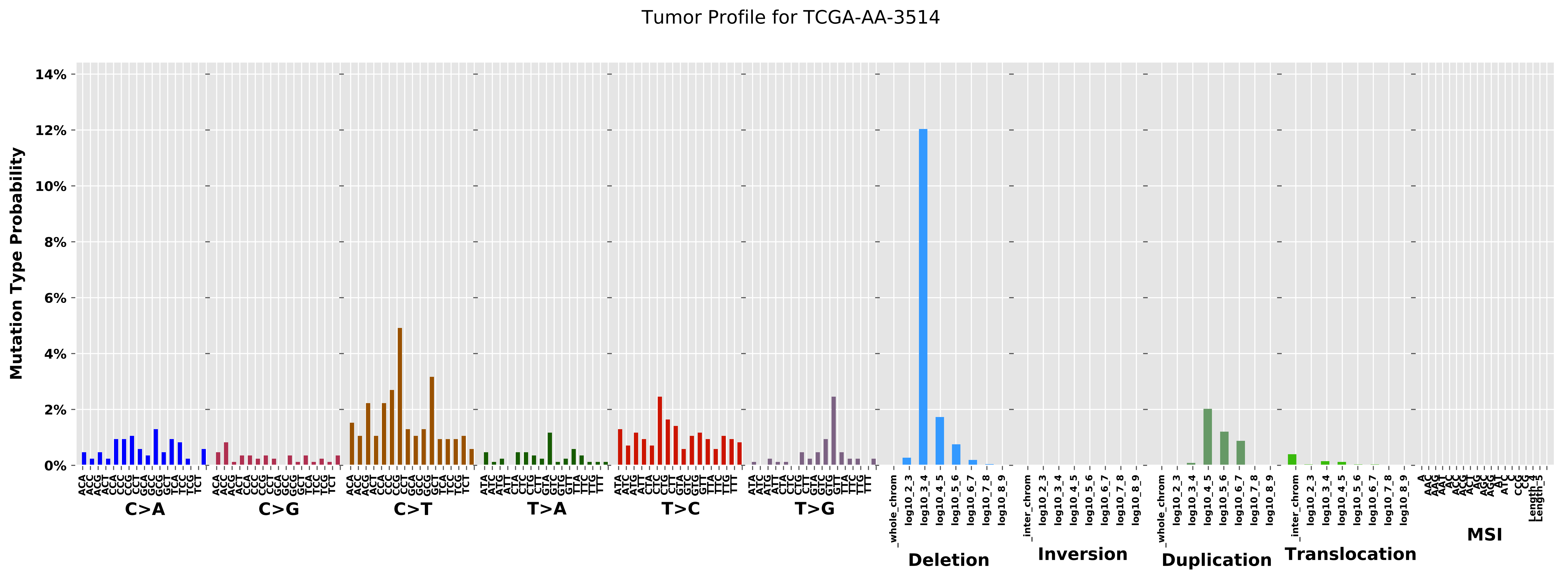

Tumor Profile for TCGA-AA-3514
Mutation Type Probability
14%
12%
10%
8%
6%
4%
2%
0%
C>A
C>G
C>T
T>A
T>C
T>G
Deletion
Inversion
Duplication
Translocation
MSI

Cancer processes Weights for TCGA-AN-A0XR

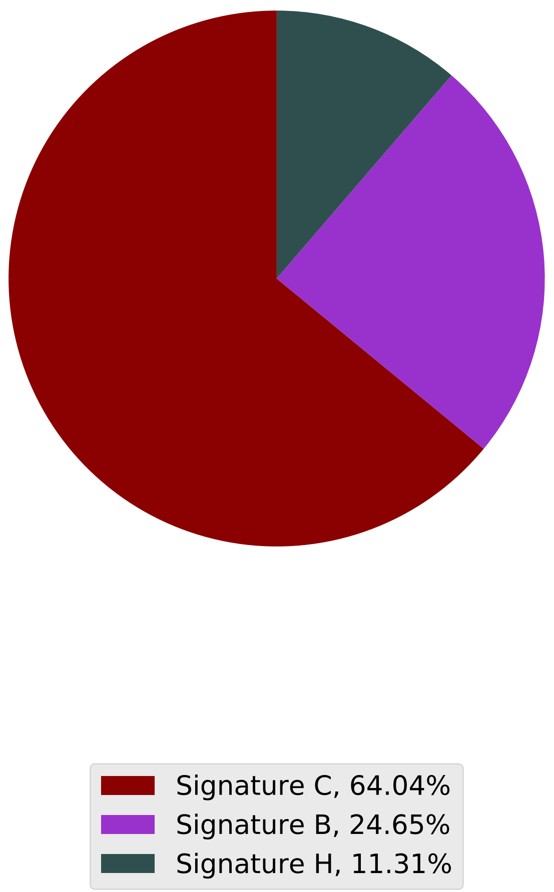

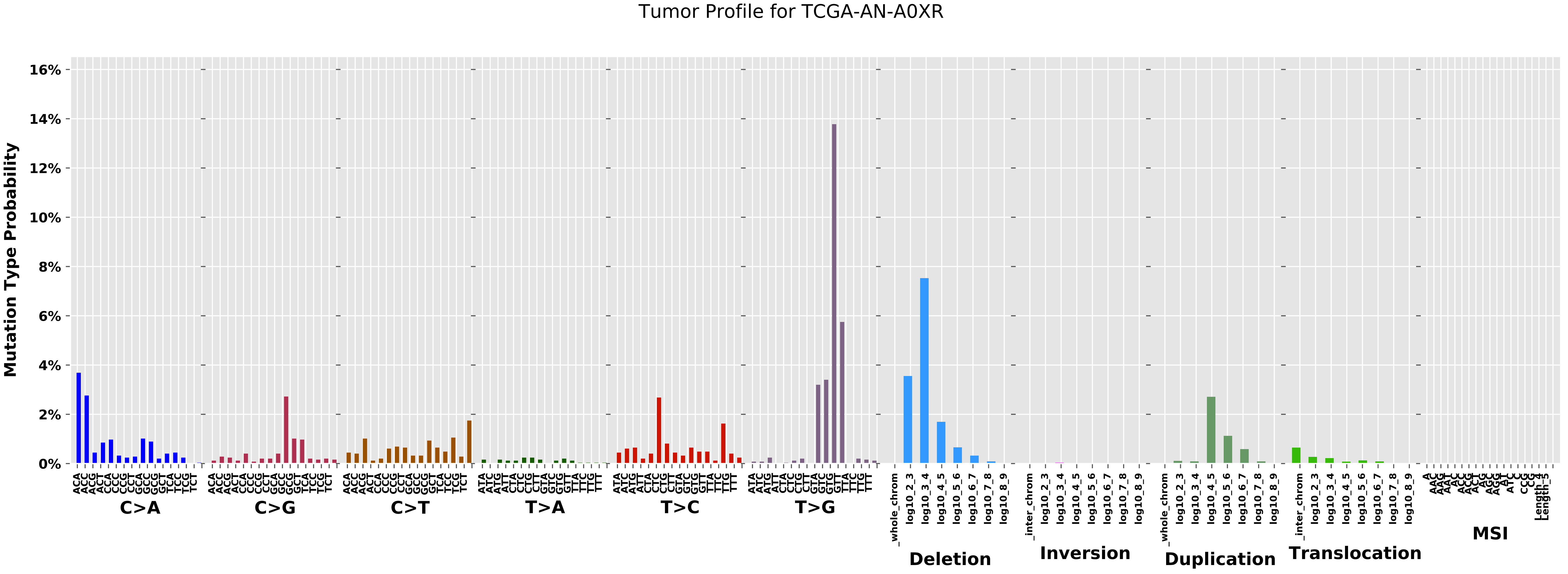

Tumor Profile for TCGA-AN-A0XR
Mutation Type Probability
16%
14%
12%
10%
8%
6%
4%
2%
0%
C>A
C>G
C>T
T>A
T>C
T>G
Deletion
Inversion
Duplication
Translocation
MSI

Cancer processes Weights for TCGA-BH-A0EA

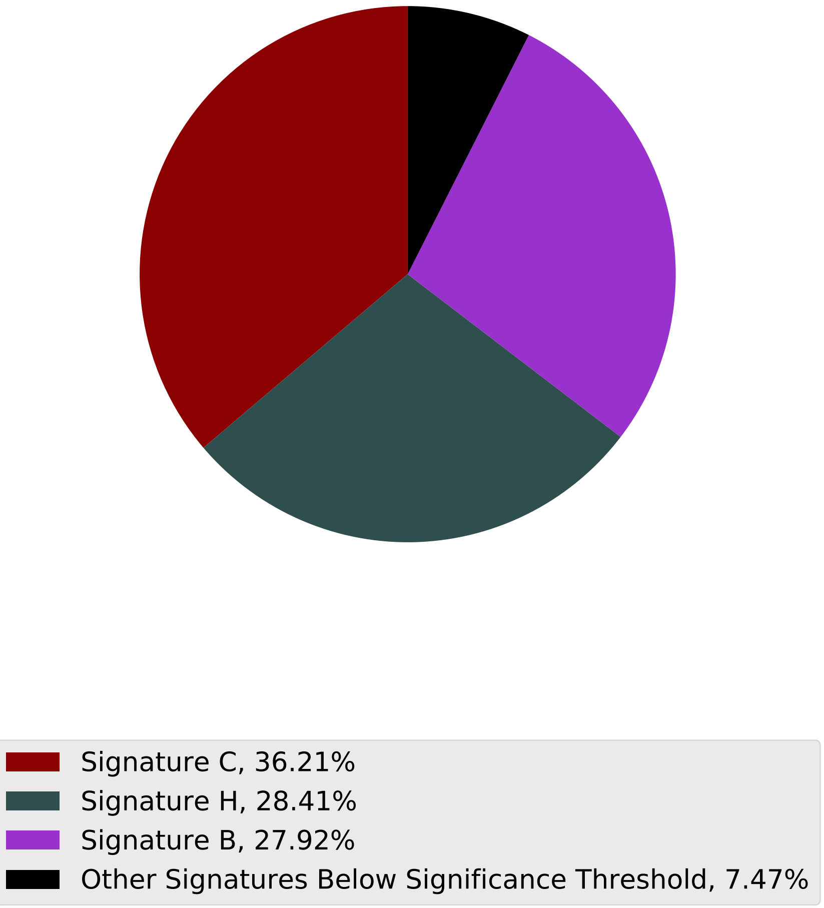

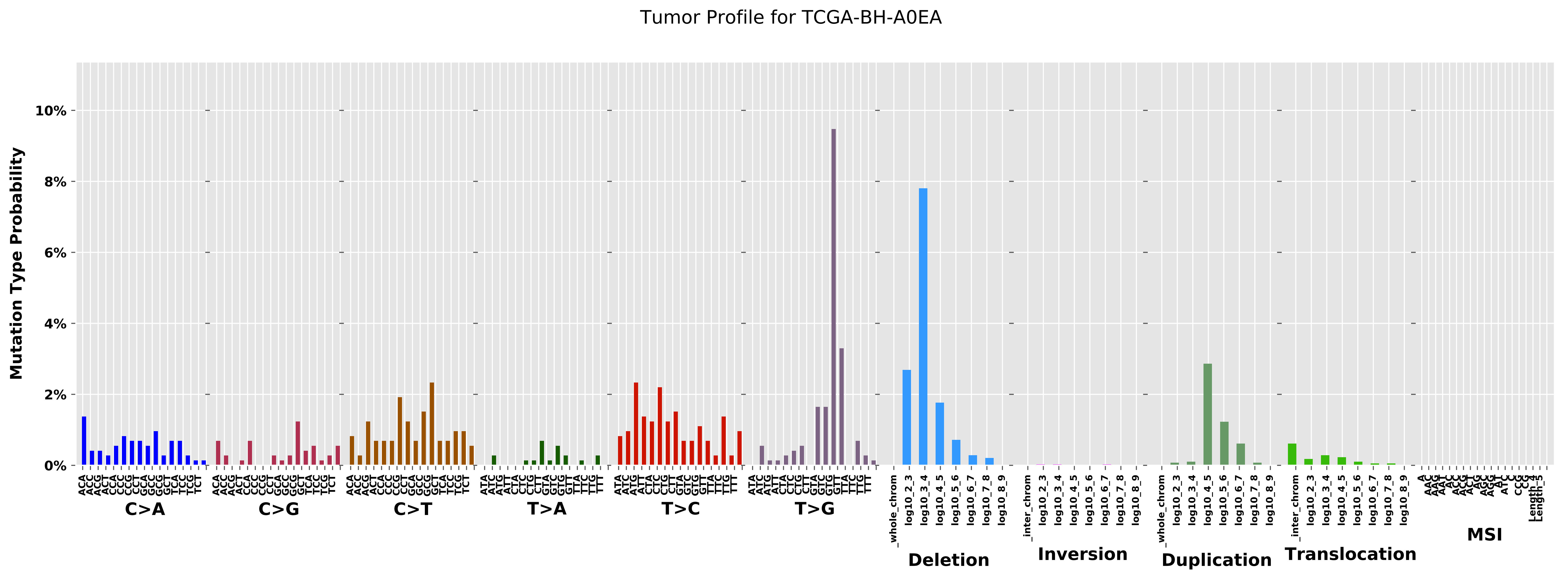

Tumor Profile for TCGA-BH-A0EA
Mutation Type Probability
10%
8%
6%
4%
2%
0%
C>A
C>G
C>T
T>A
T>C
T>G
Deletion
Inversion
Duplication
Translocation
MSI

Cancer processes Weights for TCGA-AR-A256

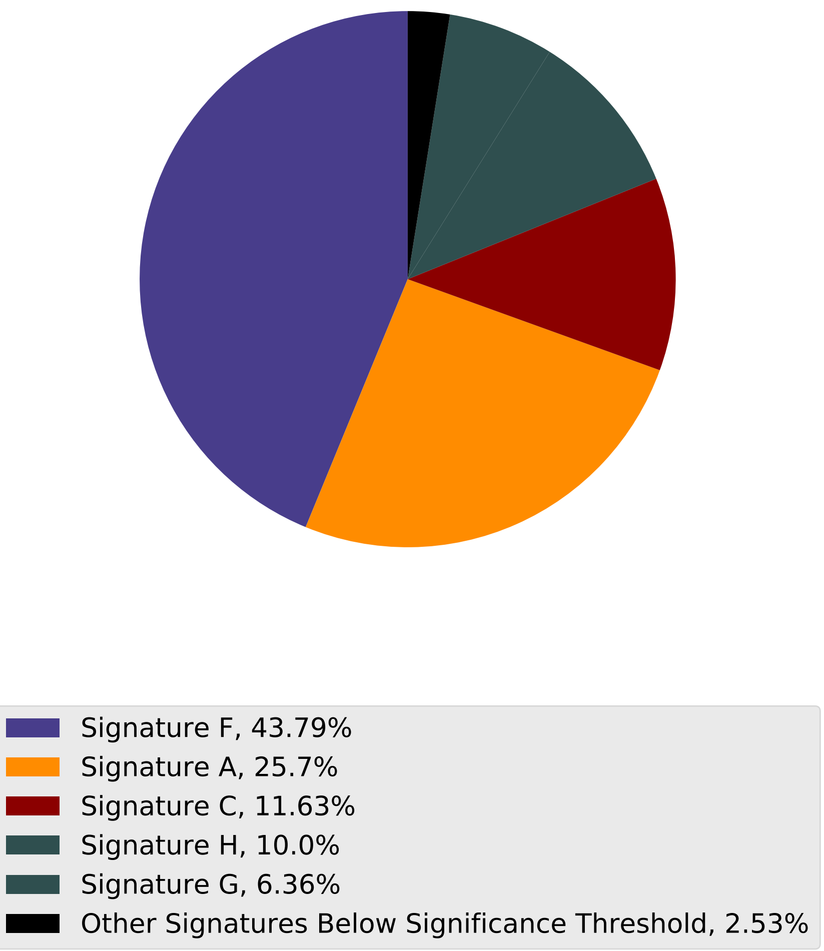

Tumor Profile for TCGA-AR-A256

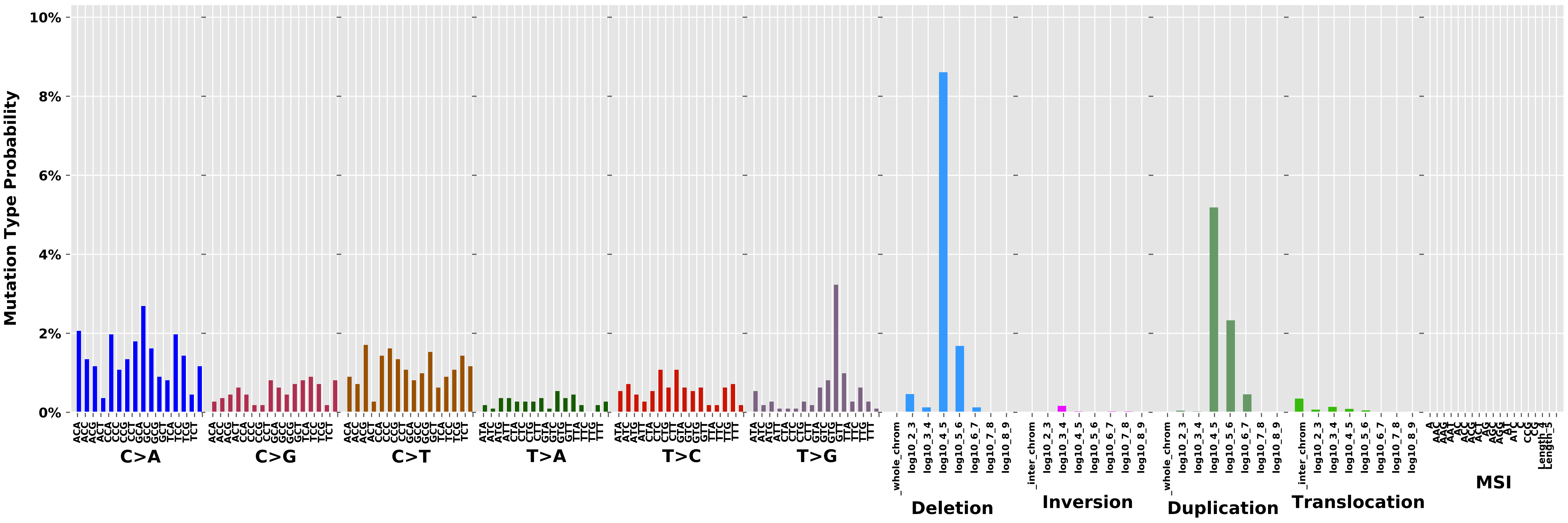

Cancer processes Weights for TCGA-A8-A09X

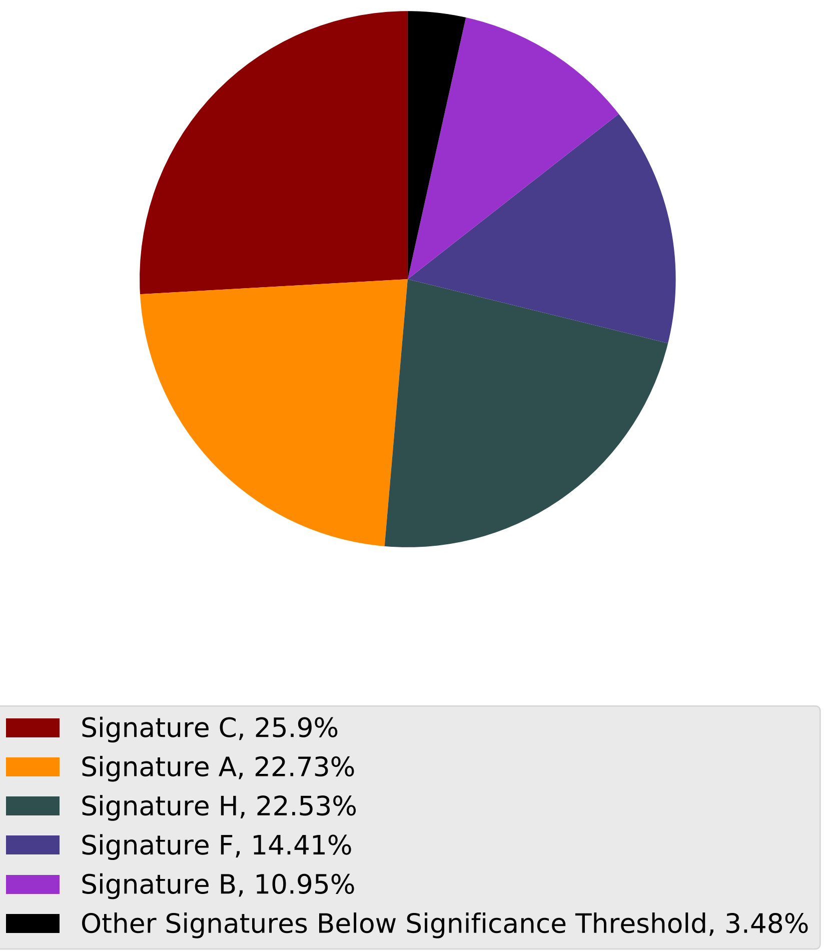

Tumor Profile for TCGA-A8-A09X

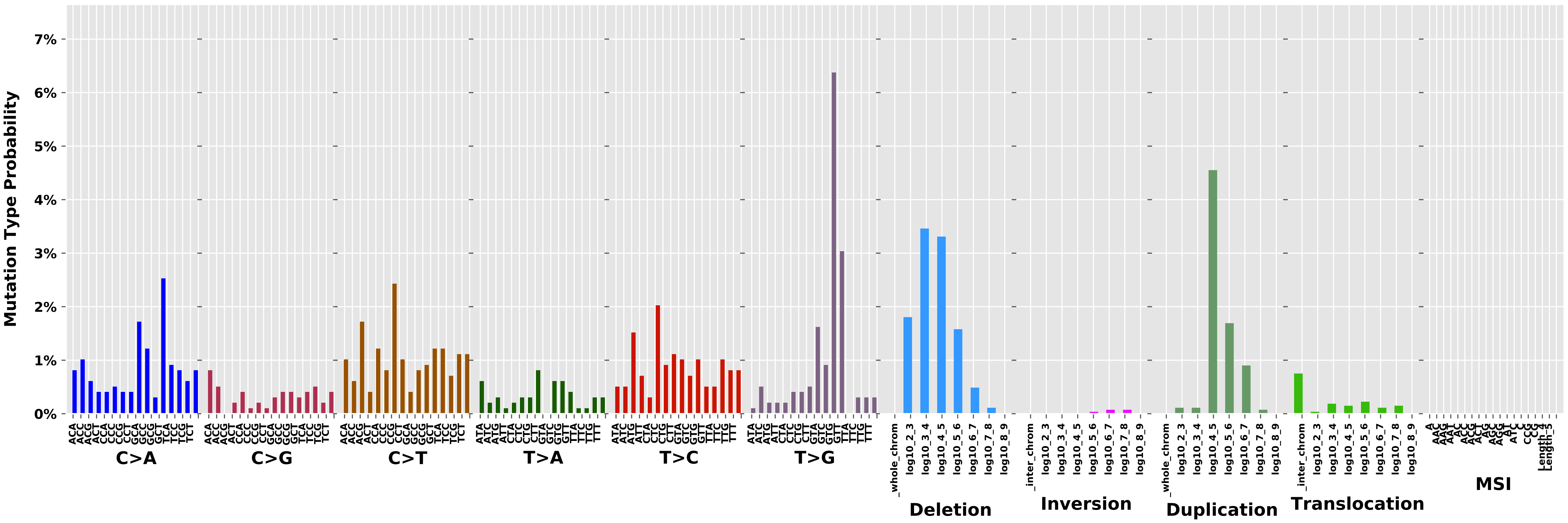

Cancer processes Weights for TCGA-AA-A01V

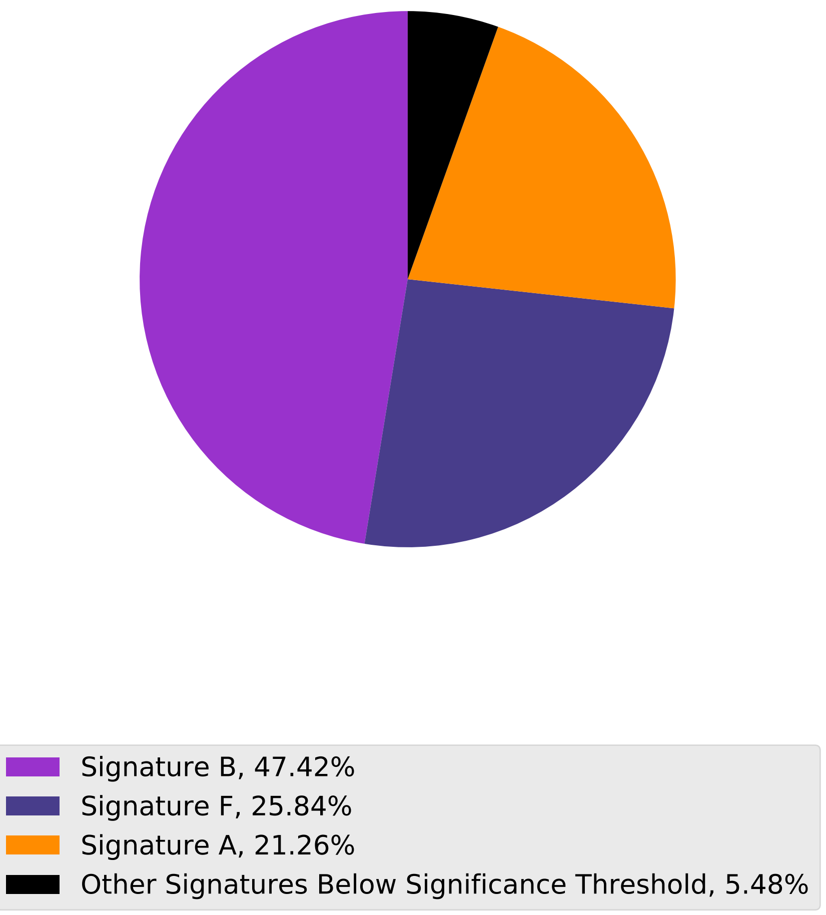

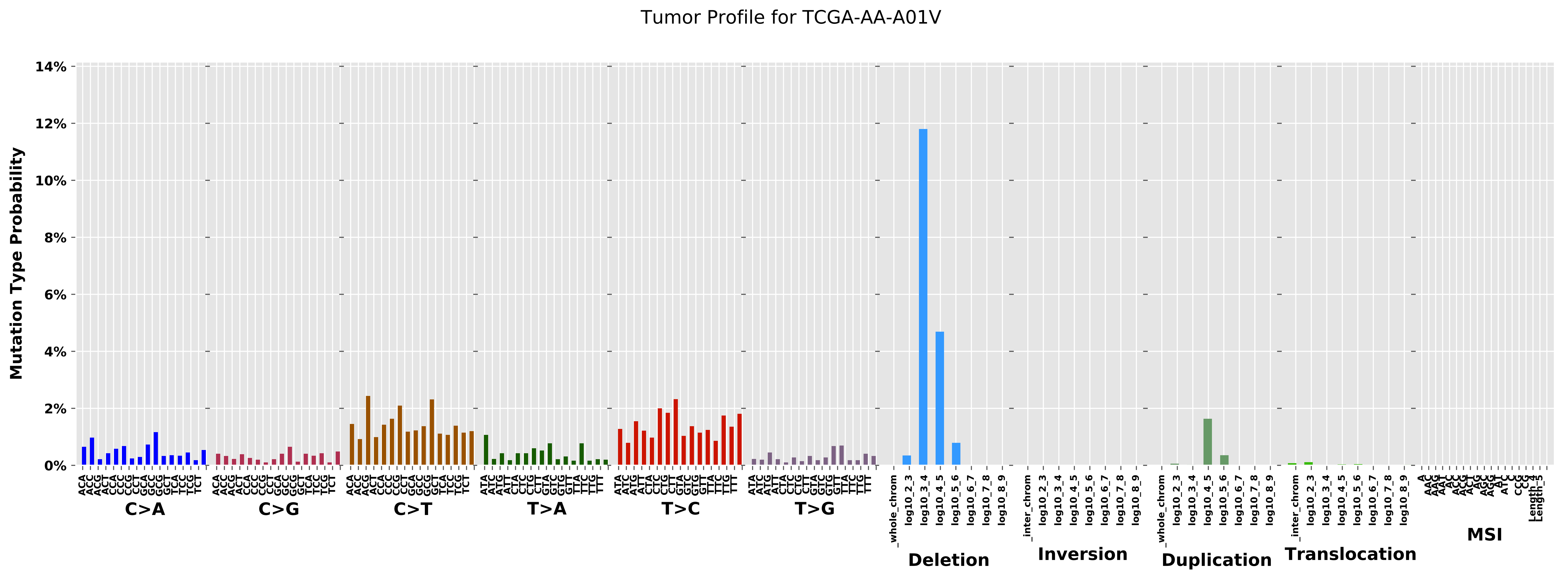

Tumor Profile for TCGA-AA-A01V
Mutation Type Probability
14%
12%
10%
8%
6%
4%
2%
0%
C>A
C>G
C>T
T>A
T>C
T>G
Deletion
Inversion
Duplication
Translocation
MSI

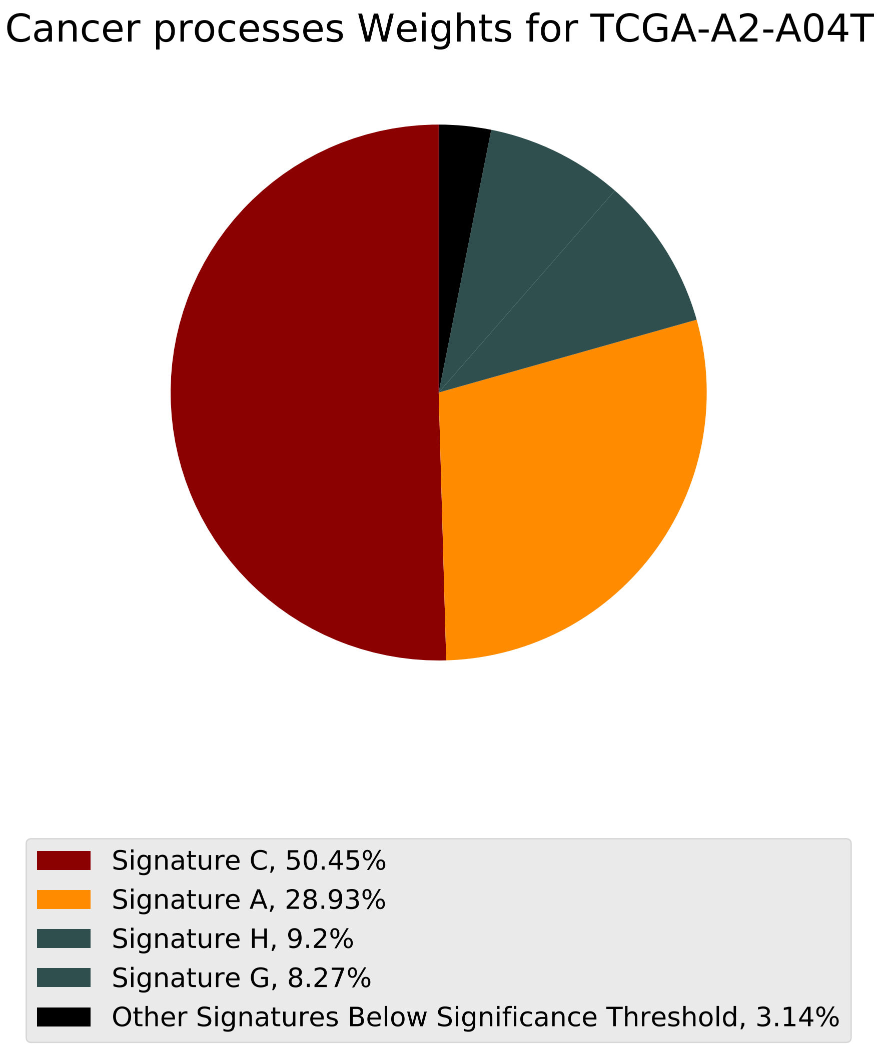
Cancer processes Weights for TCGA-A2-A04T
Signature C, 50.45%
Signature A, 28.93%
Signature H, 9.2%
Signature G, 8.27%
Other Signatures Below Significance Threshold, 3.14%

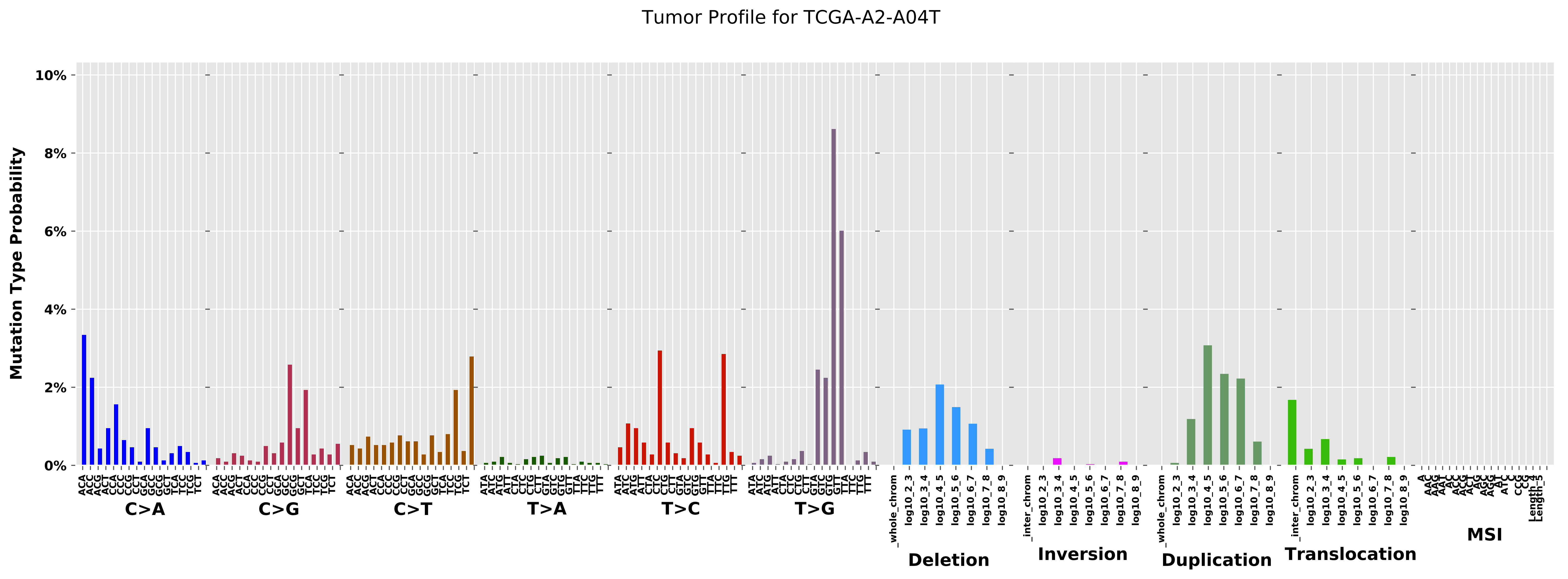
Tumor Profile for TCGA-A2-A04T
Mutation Type Probability
10%
8%
6%
4%
2%
0%
C>A
C>G
C>T
T>A
T>C
T>G
Deletion
Inversion
Duplication
Translocation
MSI

Cancer processes Weights for TCGA-BH-A0H6

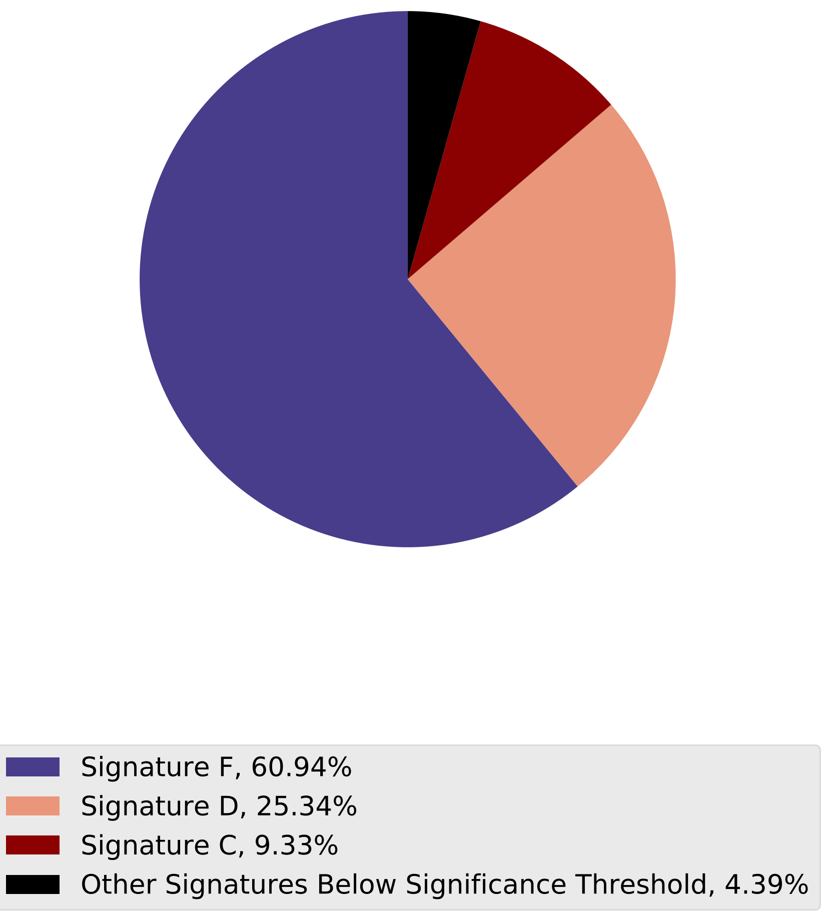

Tumor Profile for TCGA-BH-A0H6

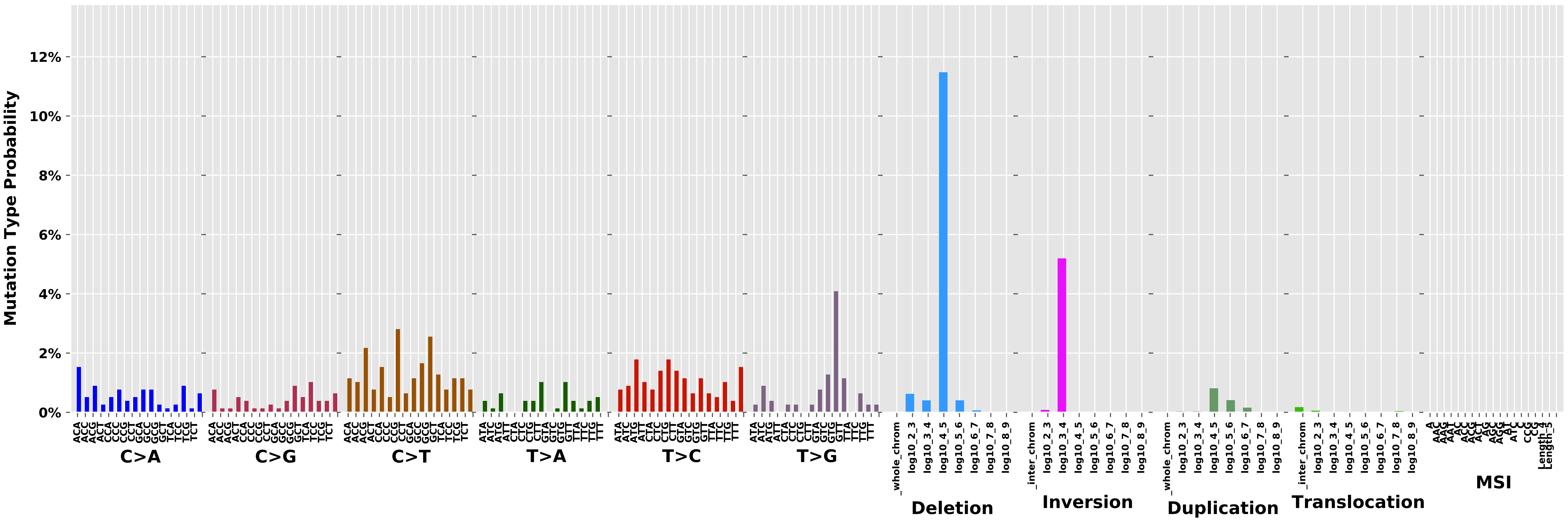

Cancer processes Weights for TCGA-D8-A27F

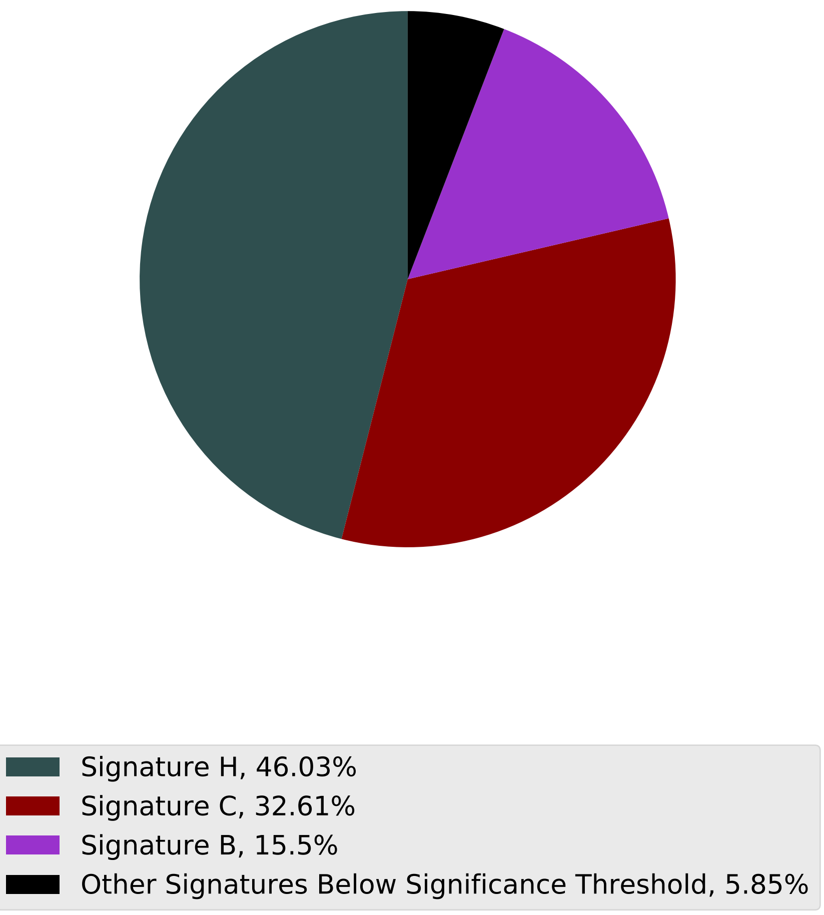

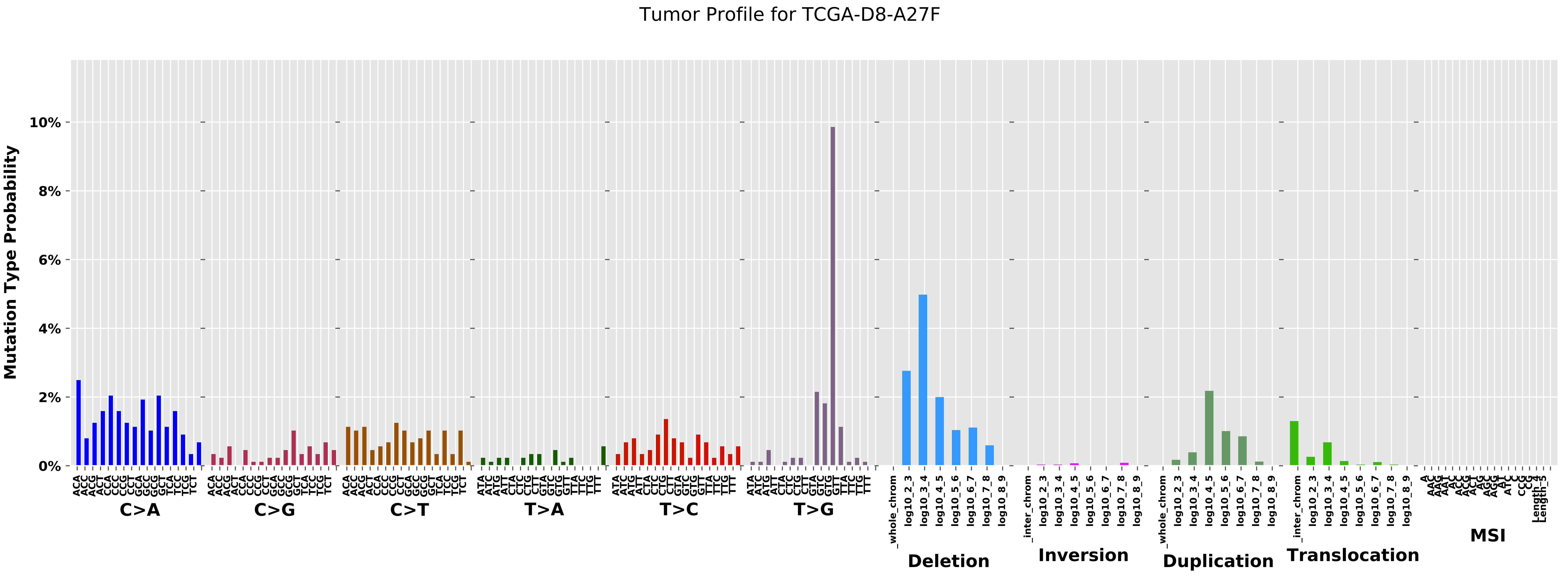
Tumor Profile for TCGA-D8-A27F
Mutation Type Probability
0%
2%
4%
6%
8%
10%
C>A
C>G
C>T
T>A
T>C
T>G
Deletion
Inversion
Duplication
Translocation
MSI

Cancer processes Weights for TCGA-AG-3727

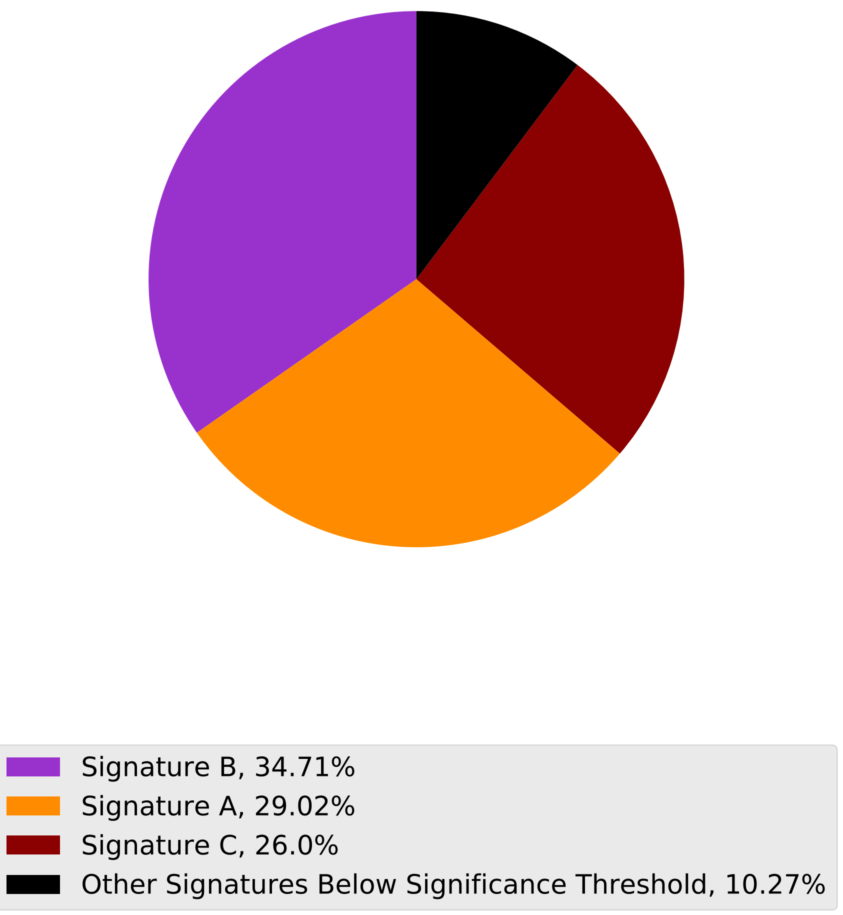

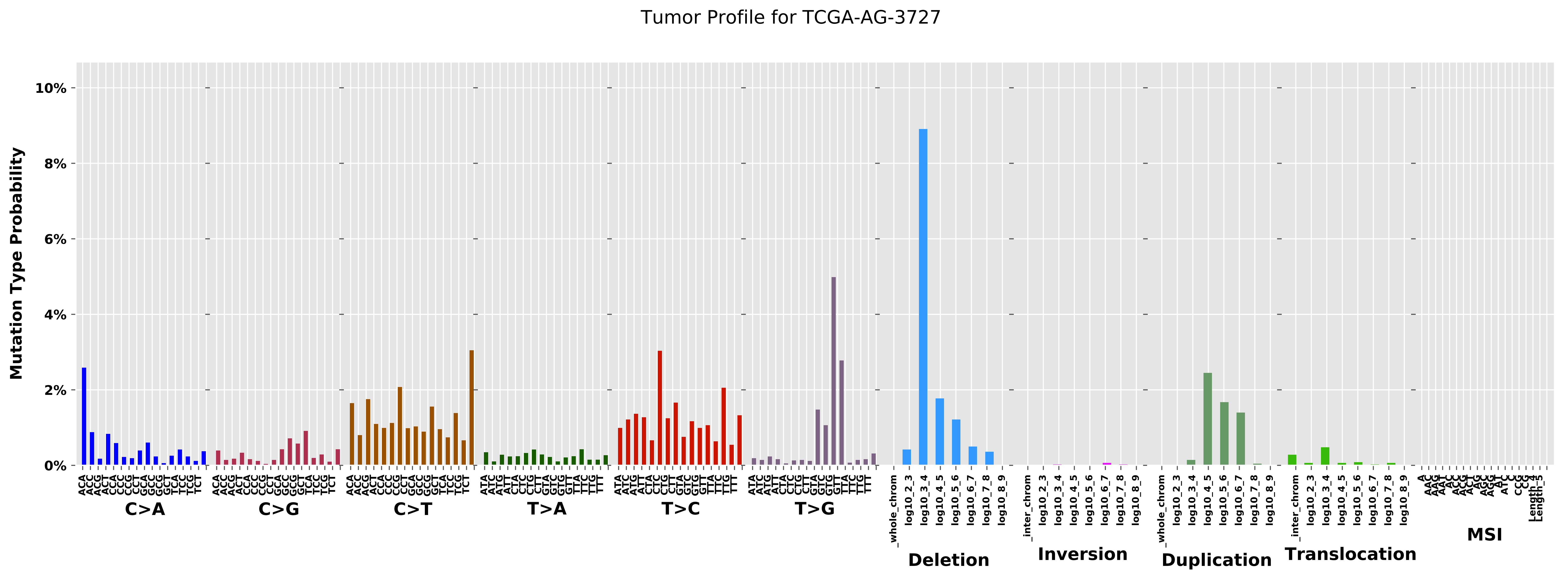
Tumor Profile for TCGA-AG-3727
Mutation Type Probability
10%
8%
6%
4%
2%
0%
C>A
C>G
C>T
T>A
T>C
T>G
Deletion
Inversion
Duplication
Translocation
MSI

Cancer processes Weights for TCGA-A8-A09I

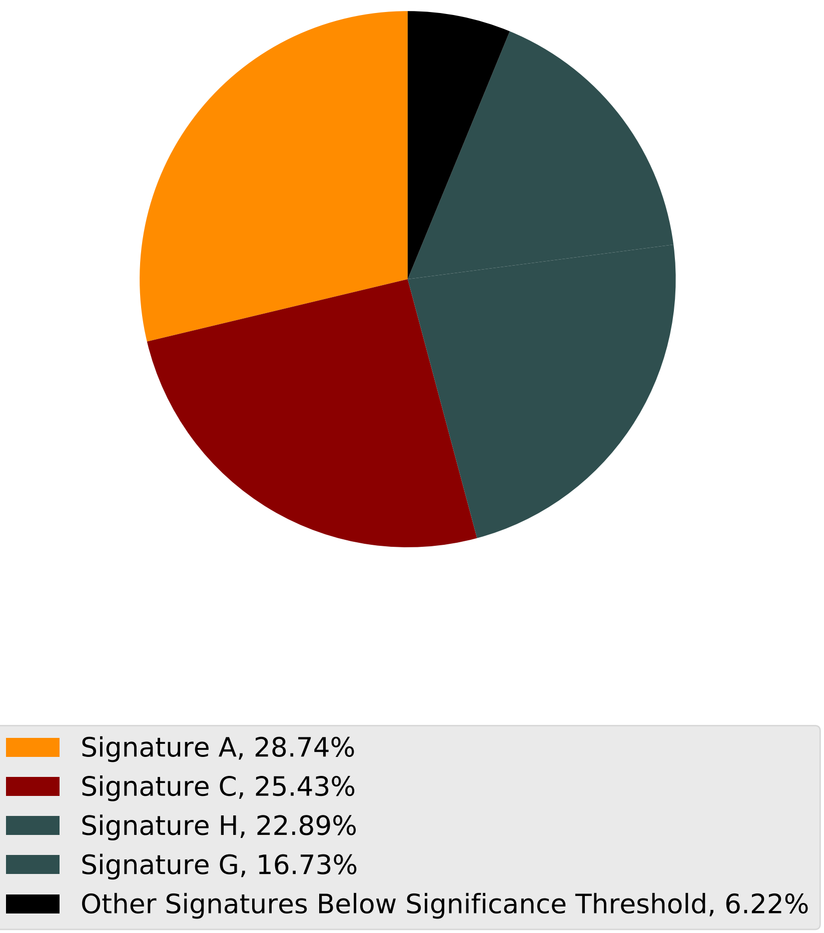

Tumor Profile for TCGA-A8-A09I

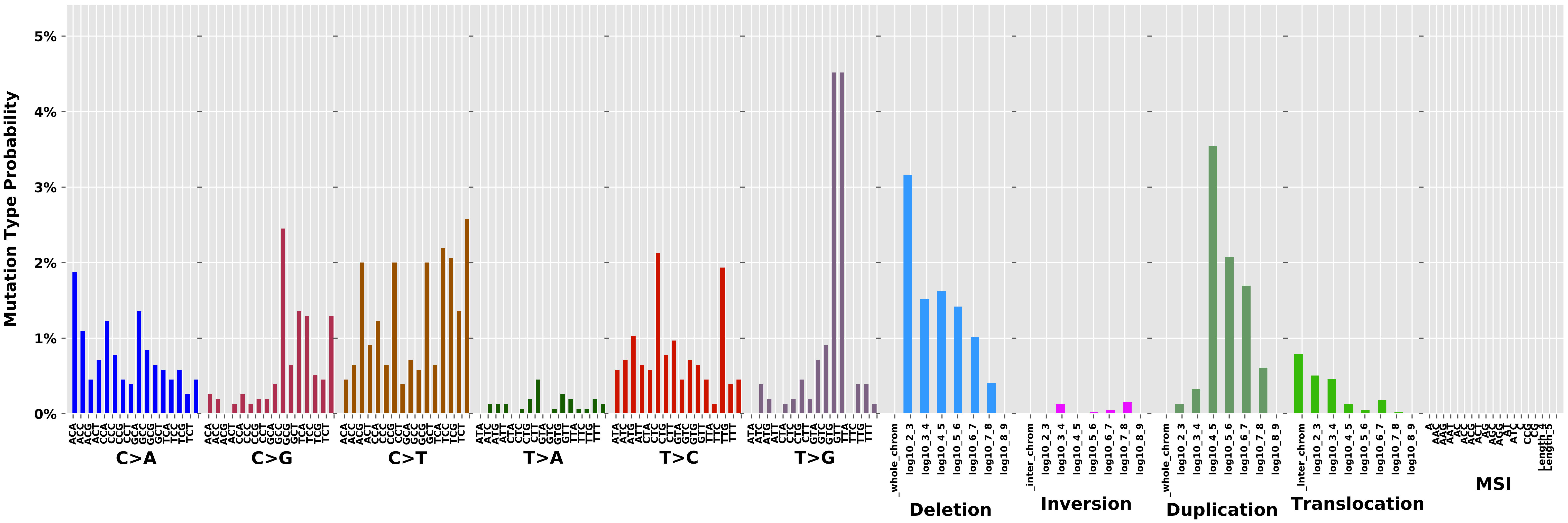

Cancer processes Weights for TCGA-AA-3664

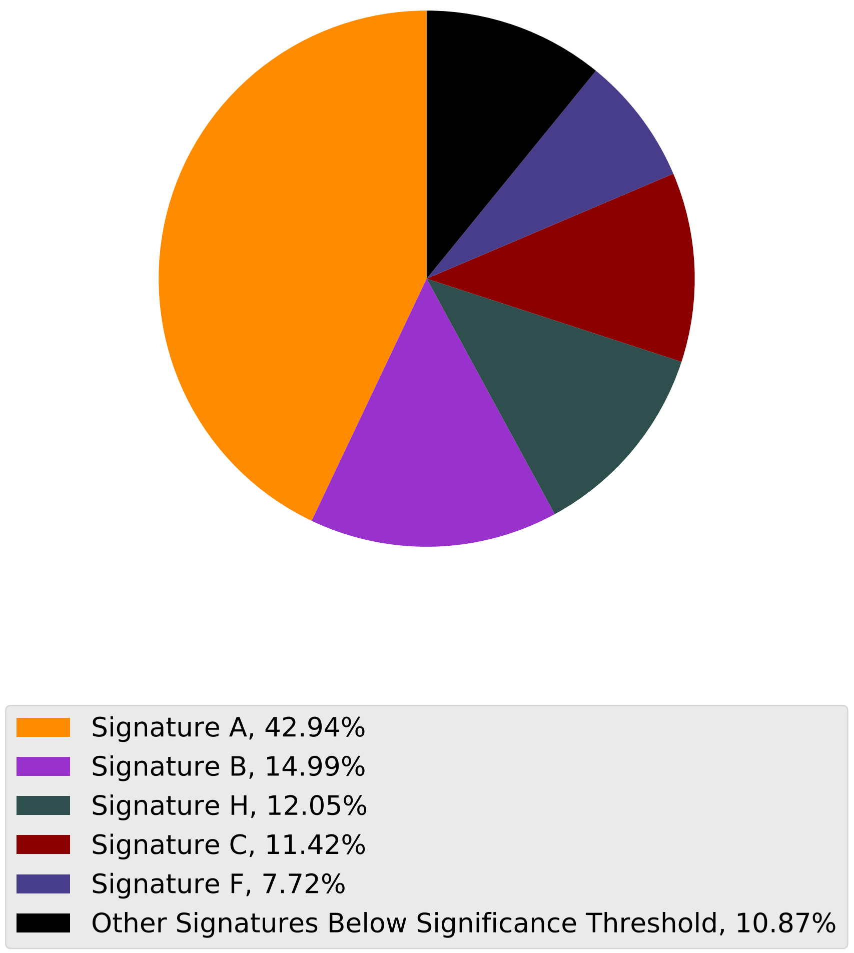

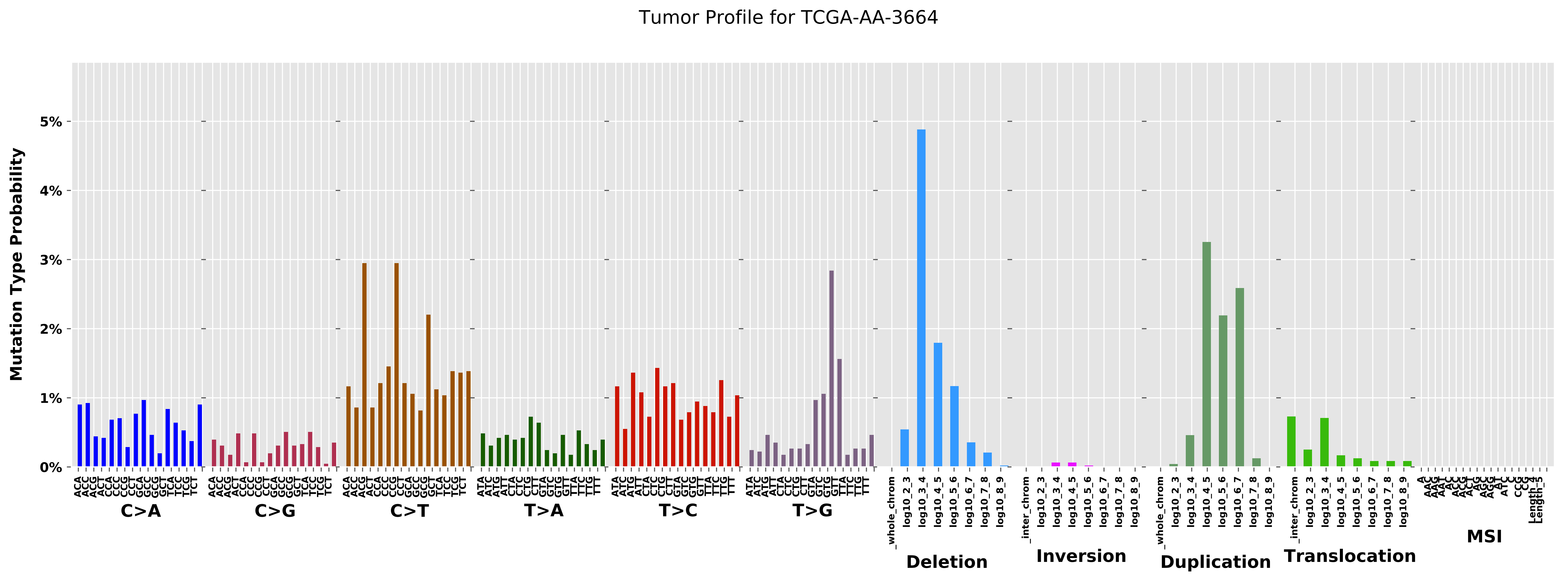
Tumor Profile for TCGA-AA-3664
Mutation Type Probability
0%
1%
2%
3%
4%
5%
C>A
C>G
C>T
T>A
T>C
T>G
Deletion
Inversion
Duplication
Translocation
MSI

Cancer processes Weights for TCGA-E2-A1LL

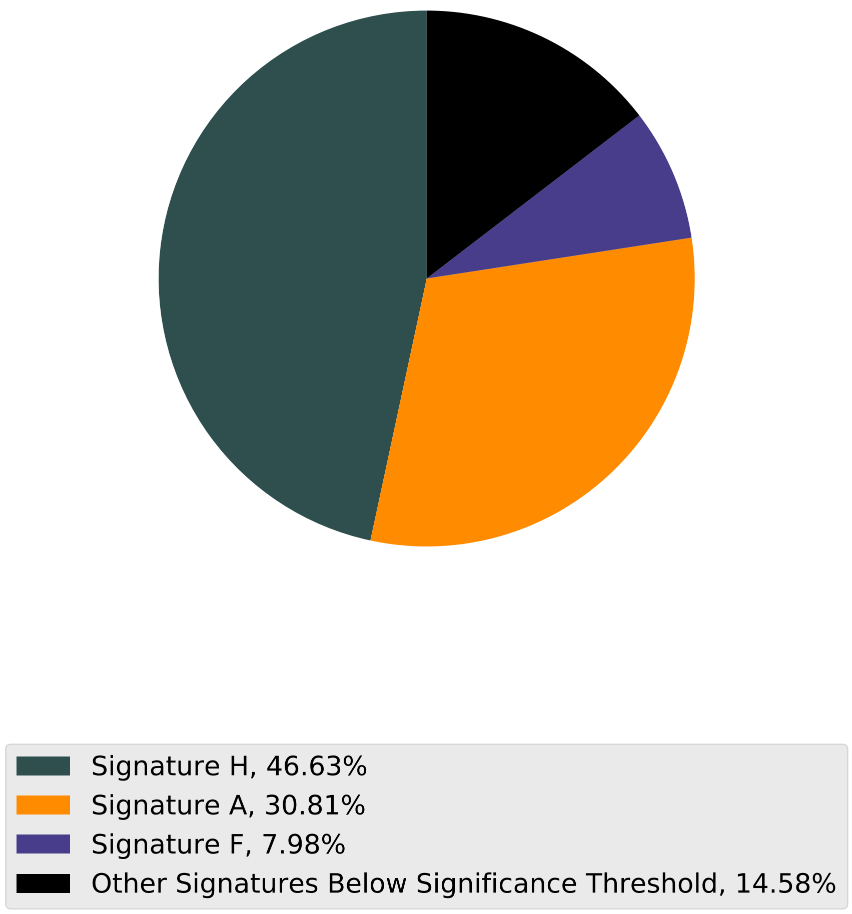

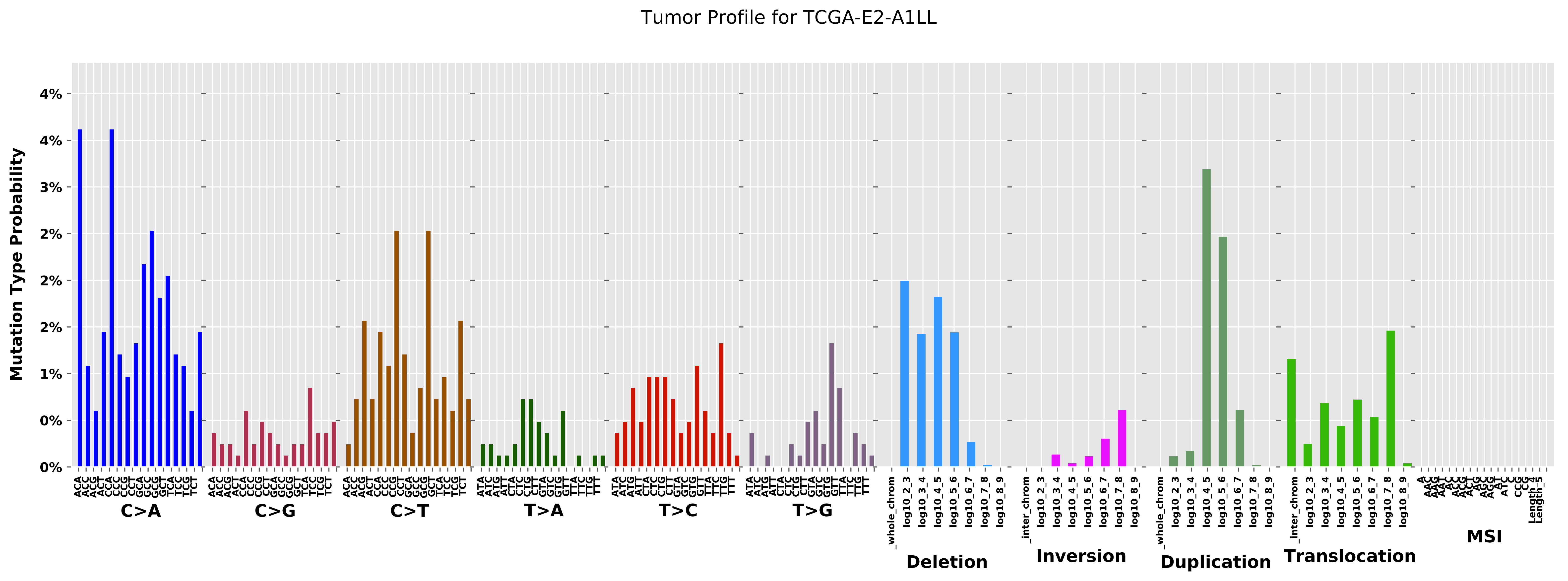
Tumor Profile for TCGA-E2-A1LL
Mutation Type Probability
4%
4%
3%
2%
2%
2%
1%
0%
0%
C>A
C>G
C>T
T>A
T>C
T>G
Deletion
Inversion
Duplication
Translocation
MSI

Cancer processes Weights for TCGA-A1-A0SM

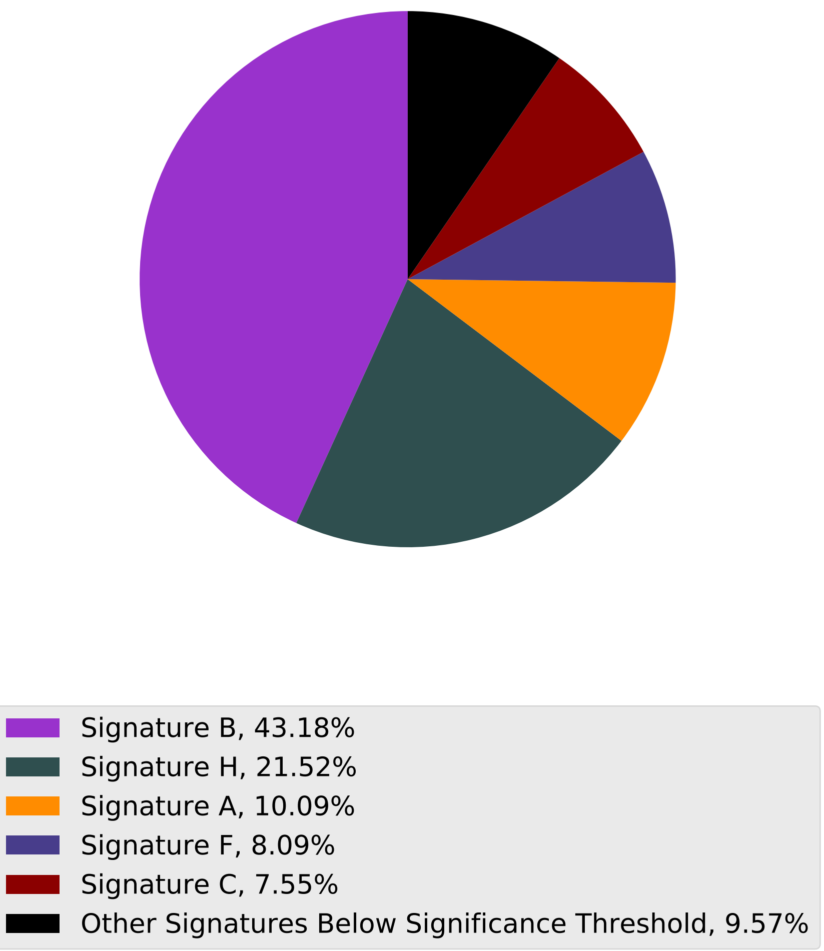

Tumor Profile for TCGA-A1-A0SM

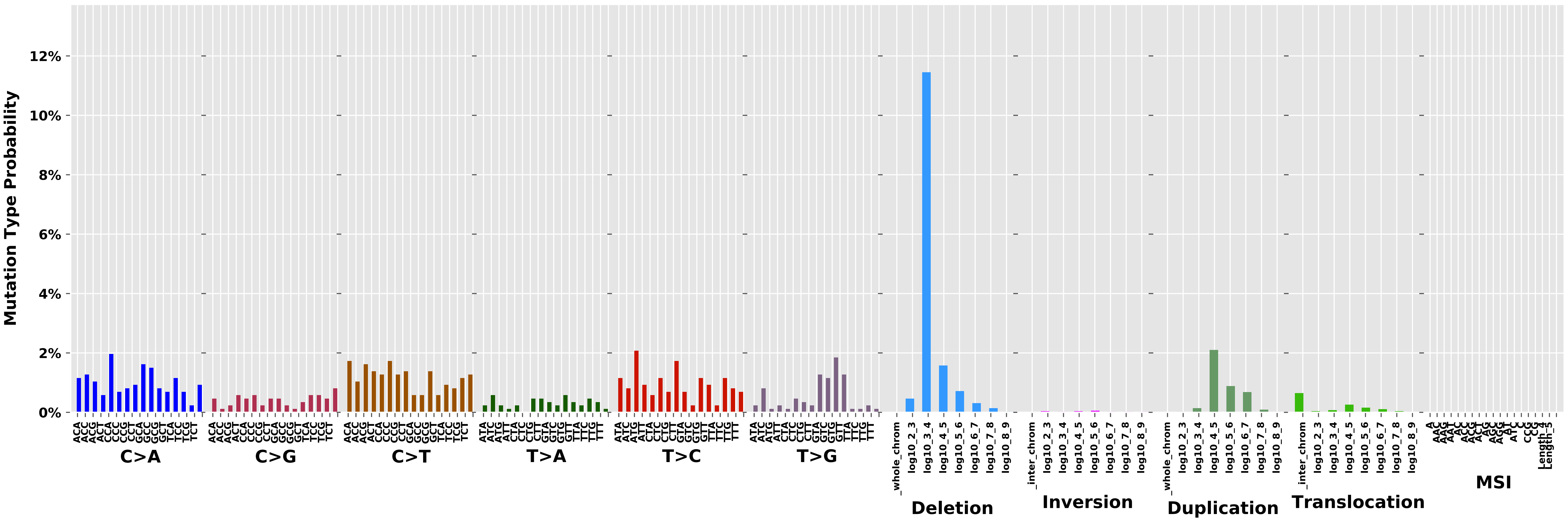

Cancer processes Weights for TCGA-C8-A130

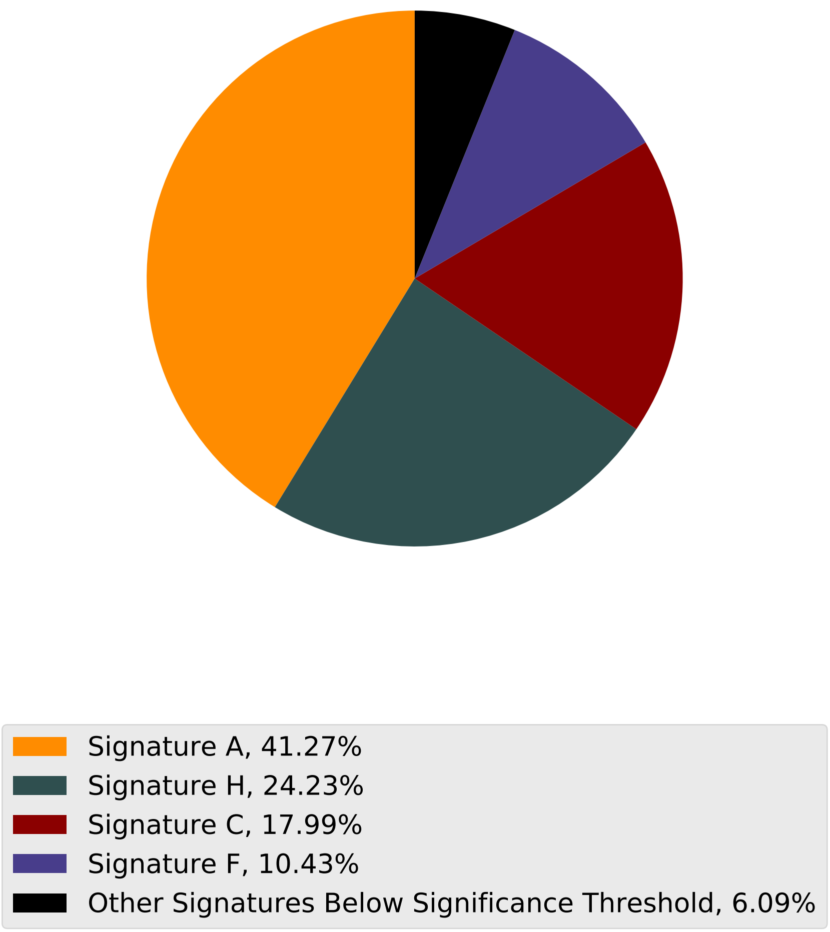

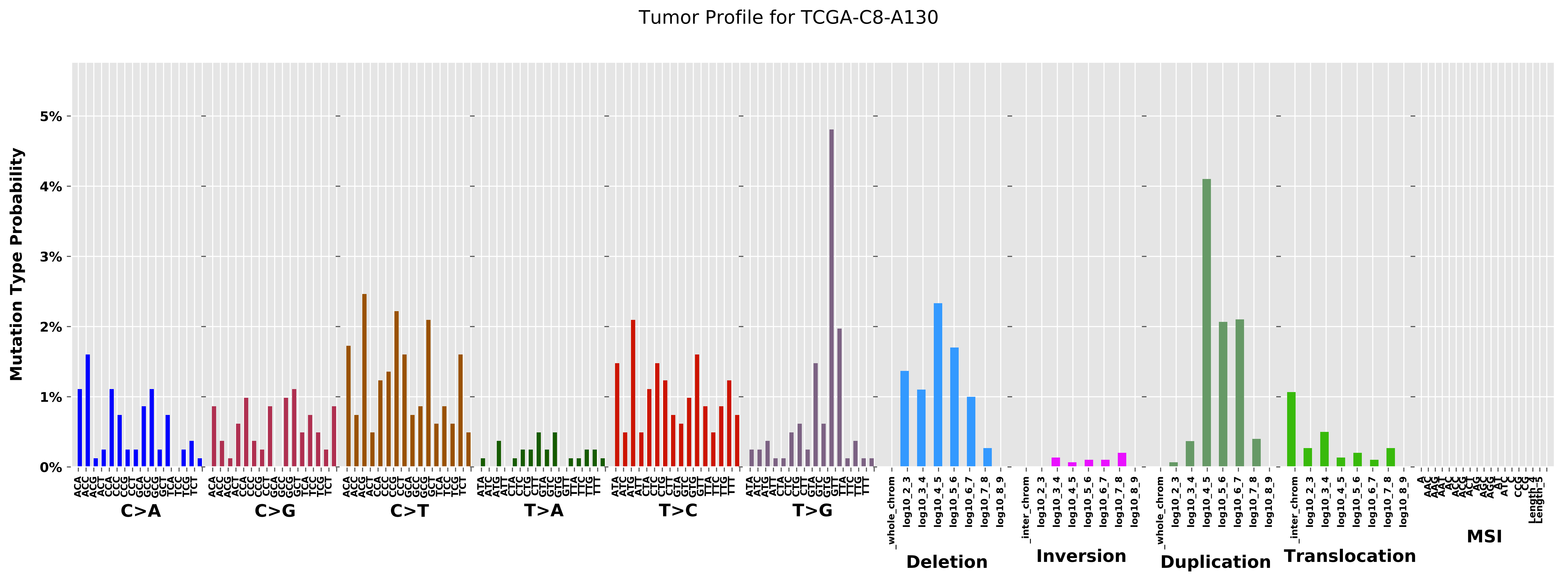
Tumor Profile for TCGA-C8-A130
Mutation Type Probability
0%
1%
2%
3%
4%
5%
C>A
C>G
C>T
T>A
T>C
T>G
Deletion
Inversion
Duplication
Translocation
MSI

Cancer processes Weights for TCGA-BH-A0H0

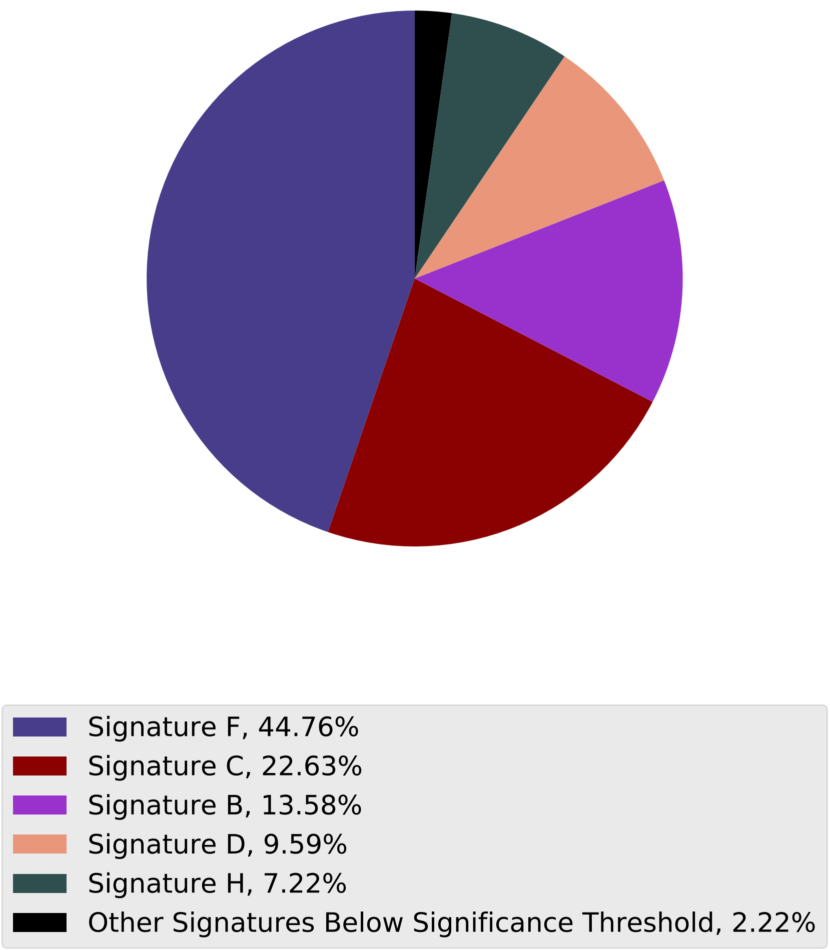

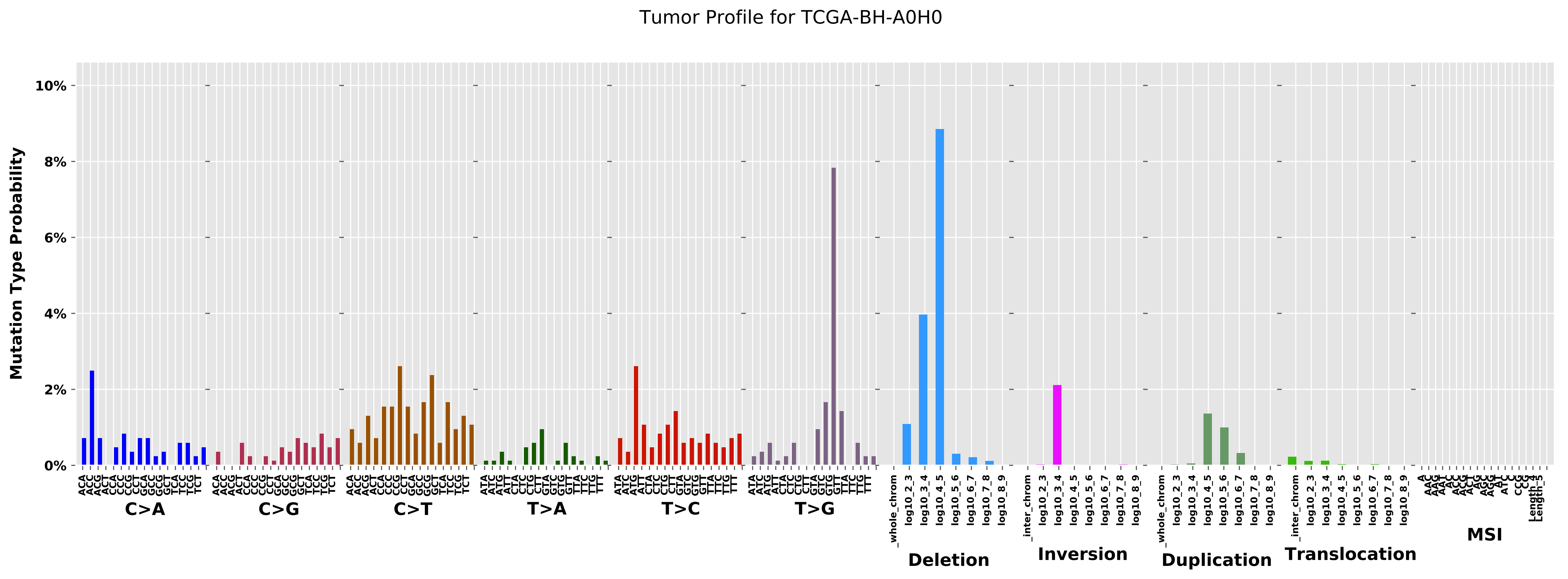

Tumor Profile for TCGA-BH-A0H0
Mutation Type Probability
10%
8%
6%
4%
2%
0%
C>A
C>G
C>T
T>A
T>C
T>G
Deletion
Inversion
Duplication
Translocation
MSI

Cancer processes Weights for TCGA-EW-A1J5

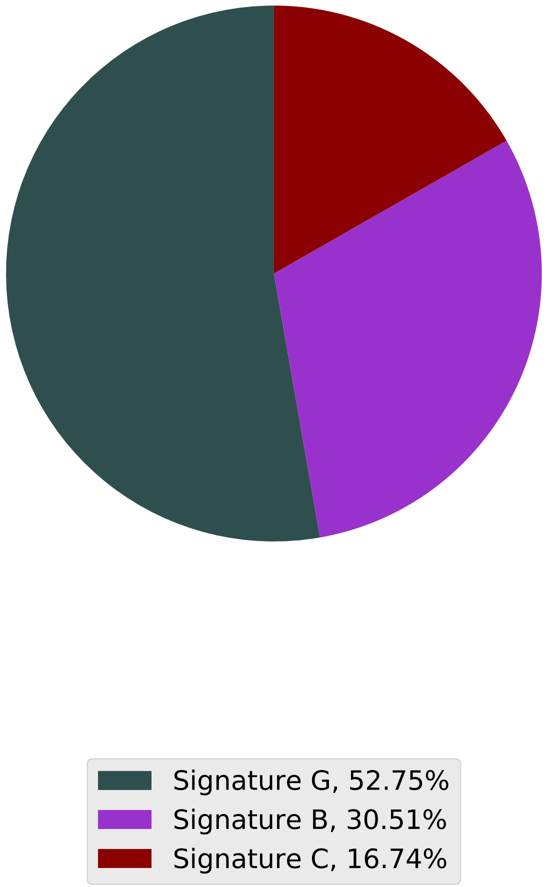

Tumor Profile for TCGA-EW-A1J5

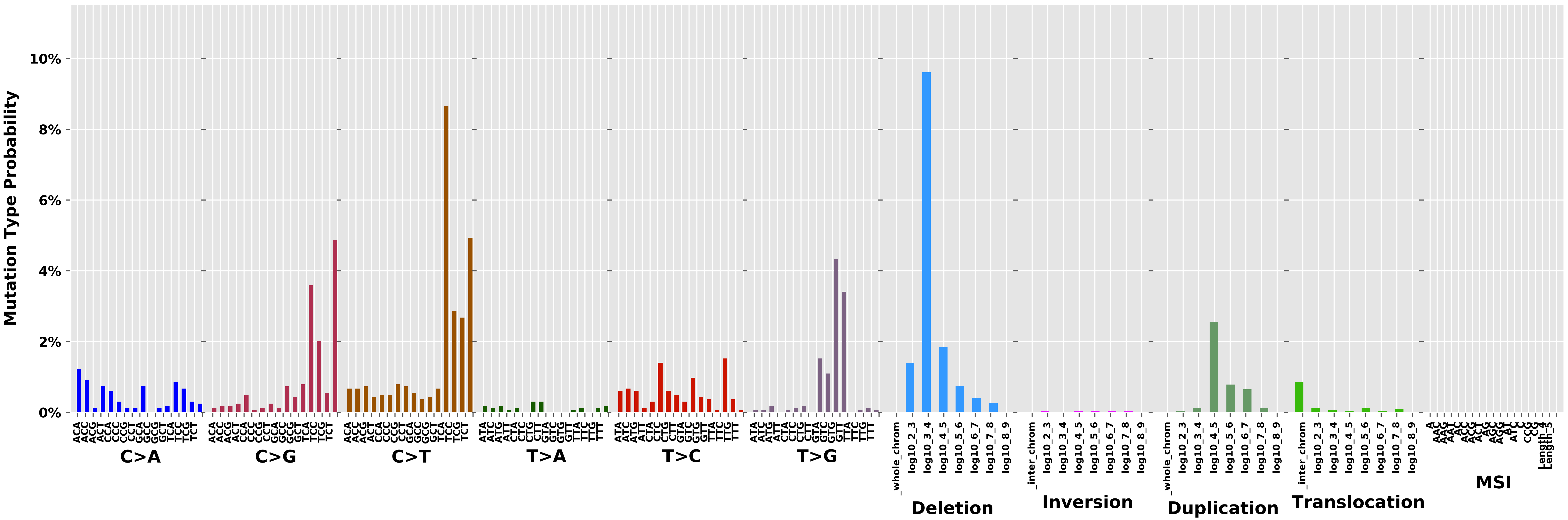

Cancer processes Weights for TCGA-EW-A1PC

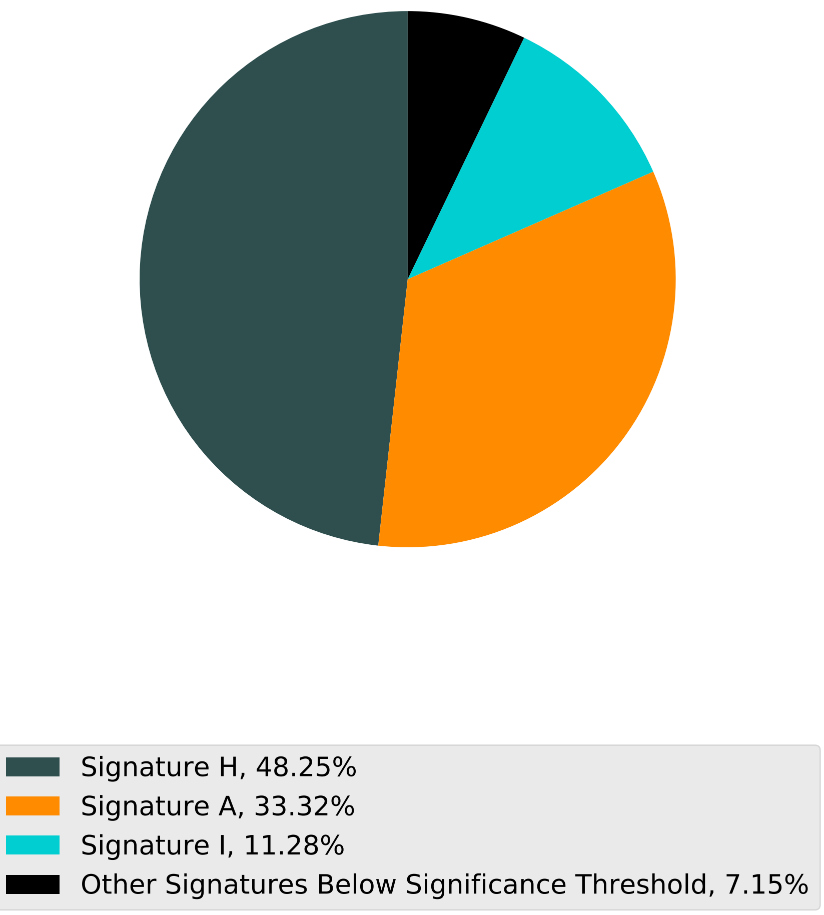

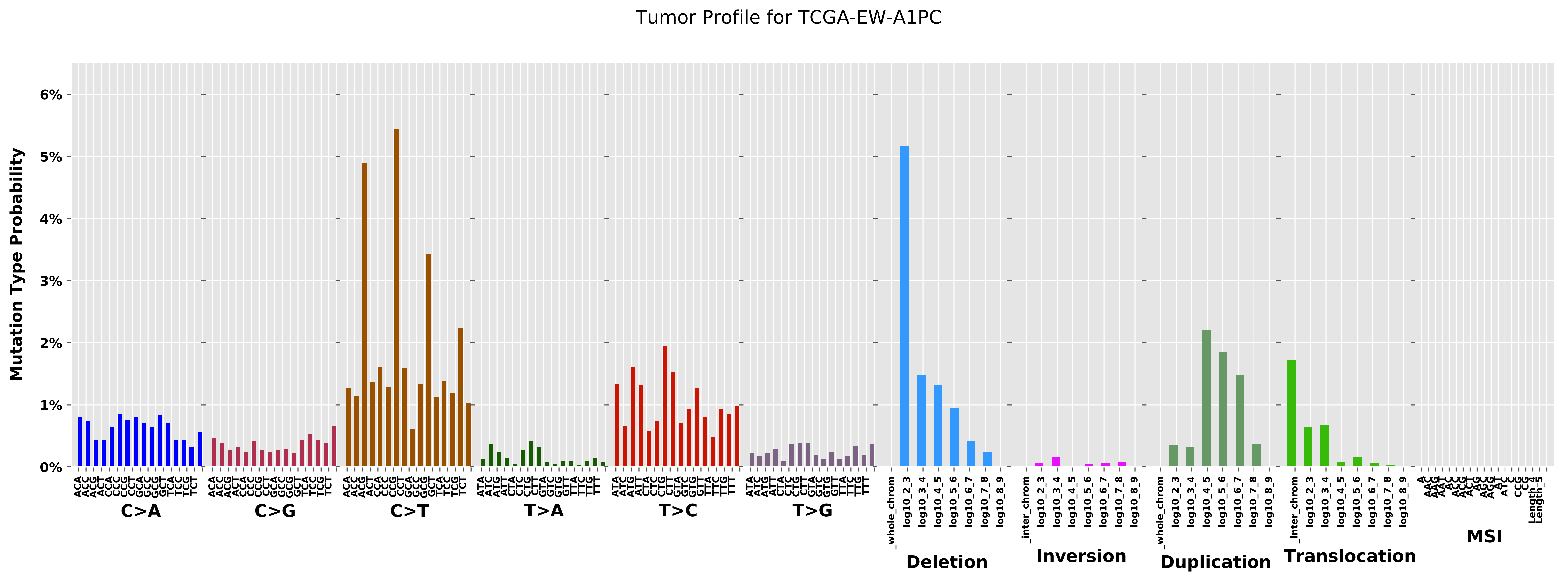

Tumor Profile for TCGA-EW-A1PC
Mutation Type Probability
0%
1%
2%
3%
4%
5%
6%
C>A
C>G
C>T
T>A
T>C
T>G
Deletion
Inversion
Duplication
Translocation
MSI

Cancer processes Weights for TCGA-BH-A0E0

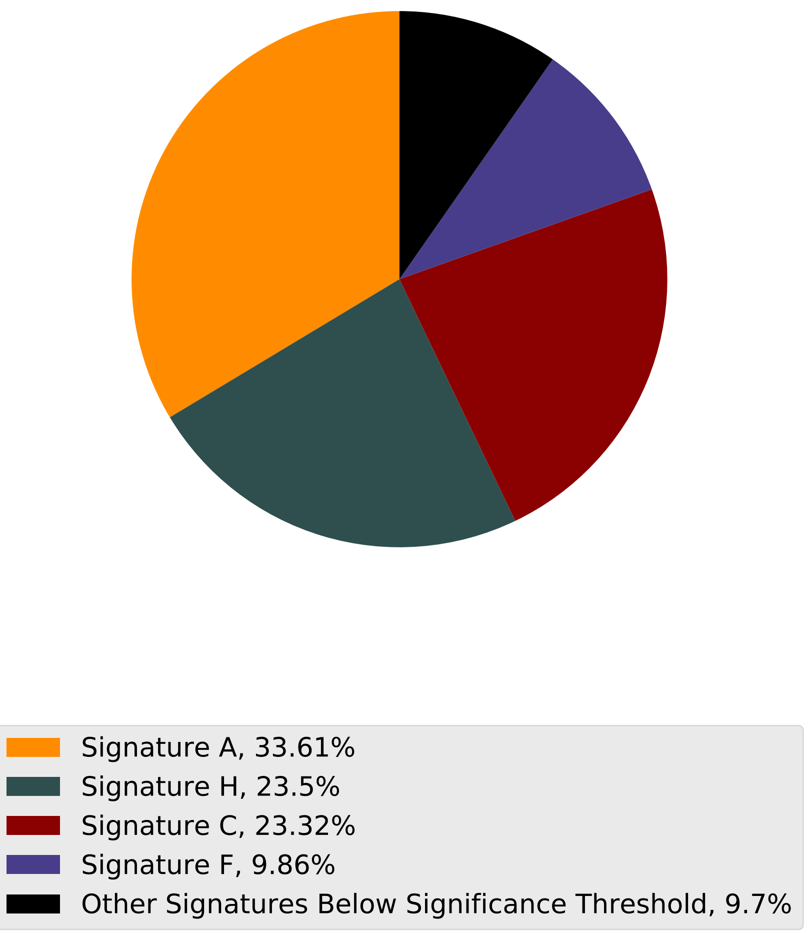

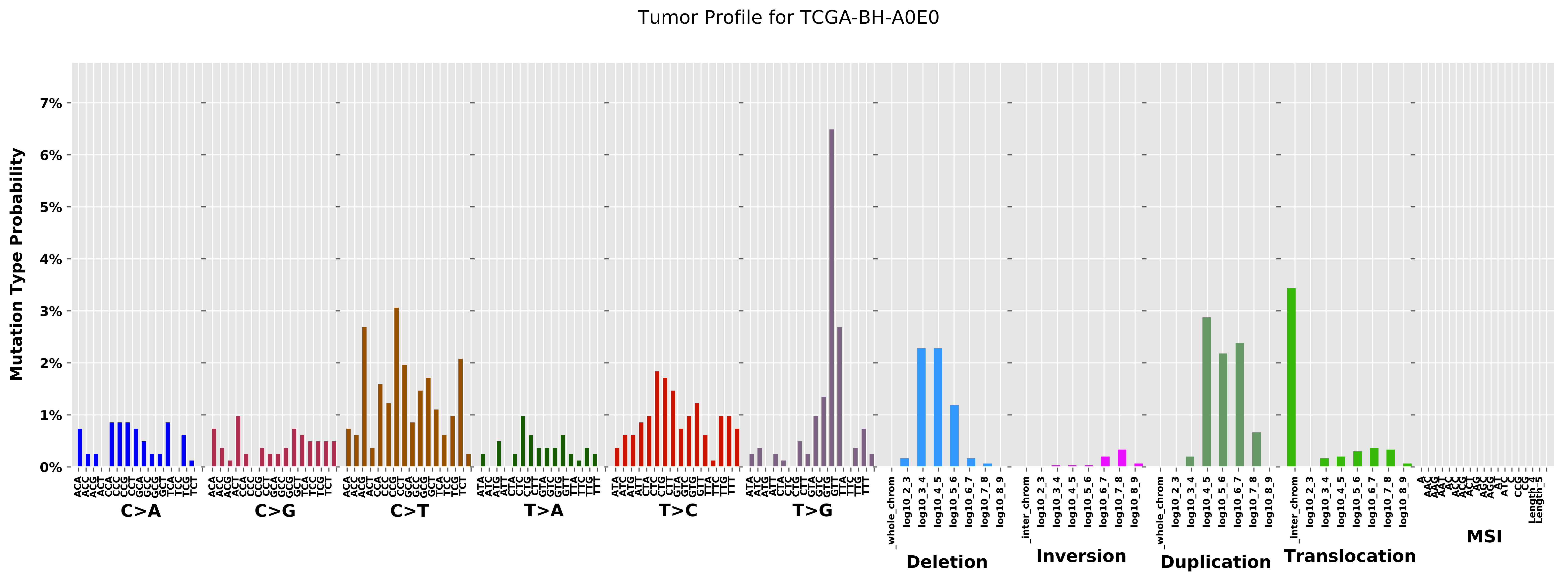

Tumor Profile for TCGA-BH-A0E0
Mutation Type Probability
0%
1%
2%
3%
4%
5%
6%
7%
C>A
C>G
C>T
T>A
T>C
T>G
Deletion
Inversion
Duplication
Translocation
MSI

Cancer processes Weights for TCGA-A2-A0D0

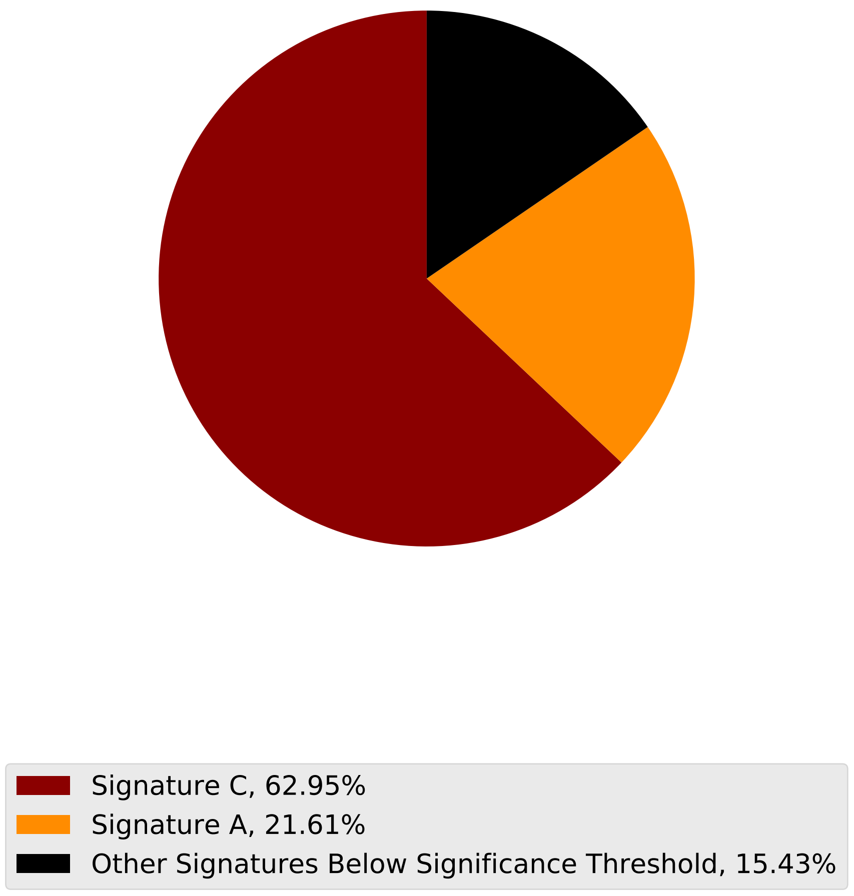

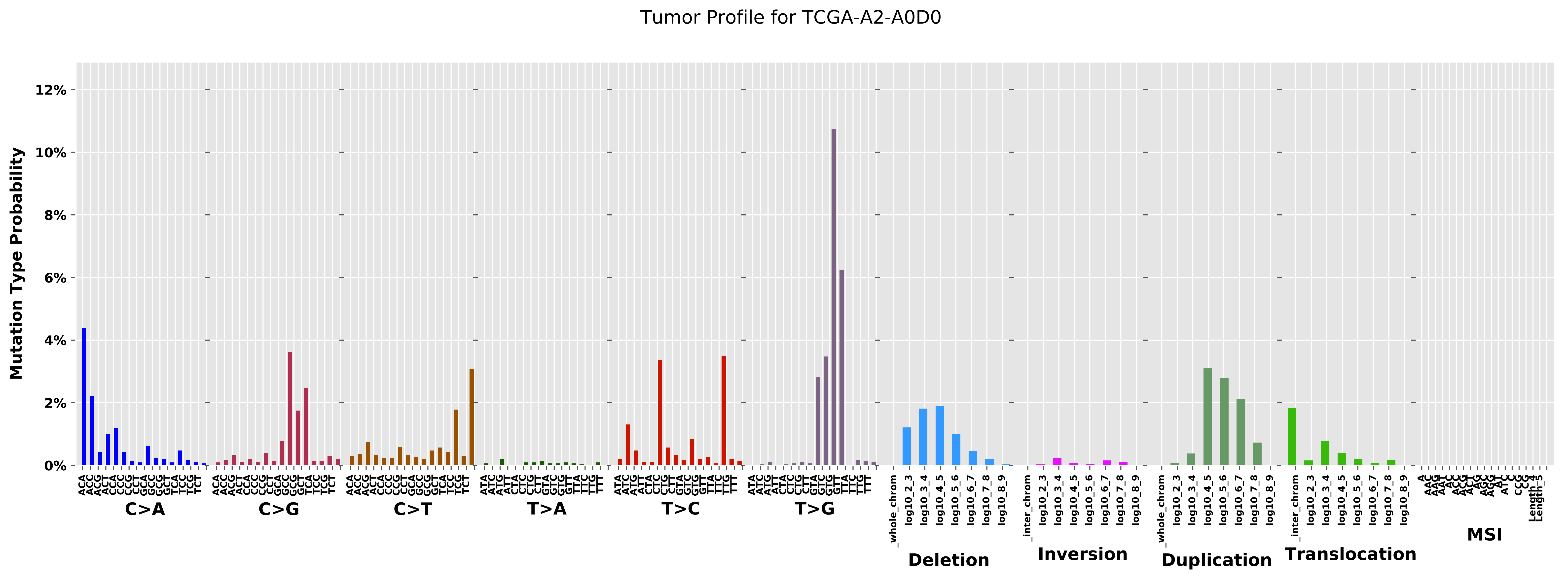
Tumor Profile for TCGA-A2-A0D0
Mutation Type Probability
12%
10%
8%
6%
4%
2%
0%
C>A
C>G
C>T
T>A
T>C
T>G
Deletion
Inversion
Duplication
Translocation
MSI

Cancer processes Weights for TCGA-AA-3956

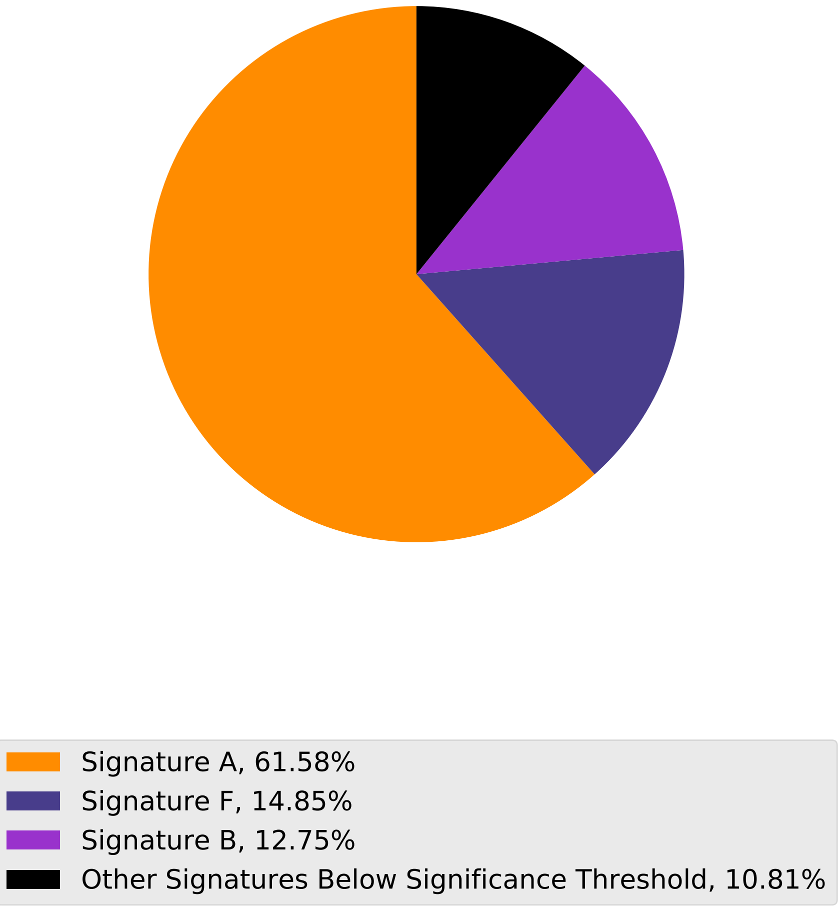

Tumor Profile for TCGA-AA-3956

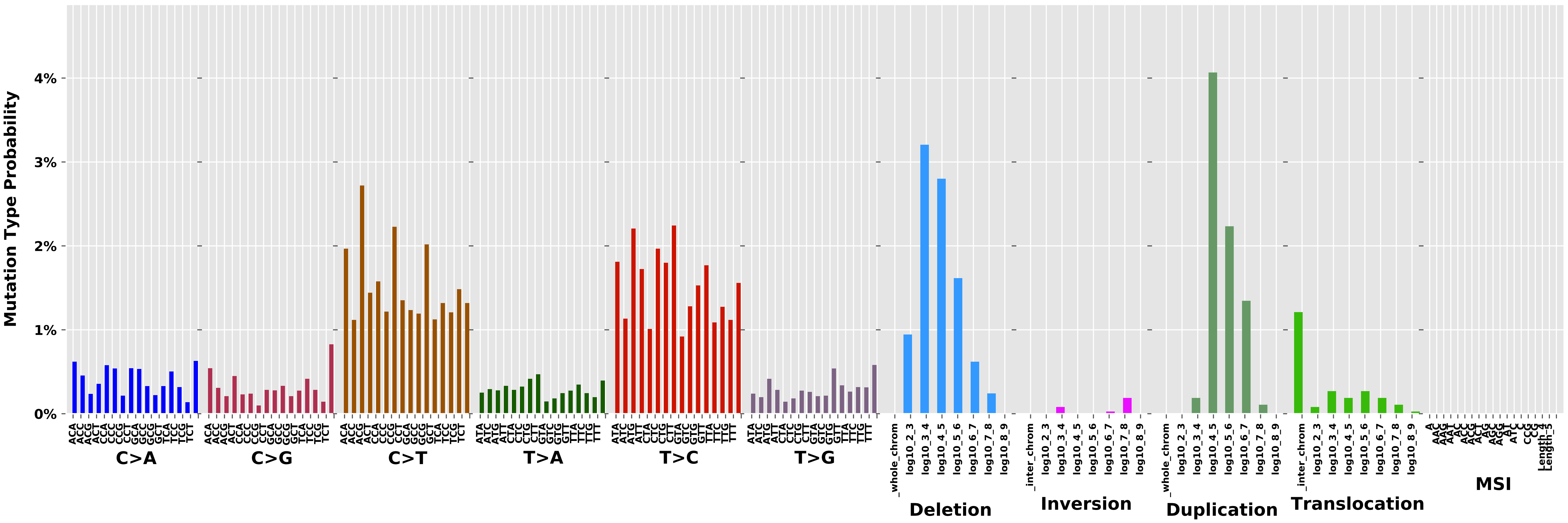

Cancer processes Weights for TCGA-AR-A0TX

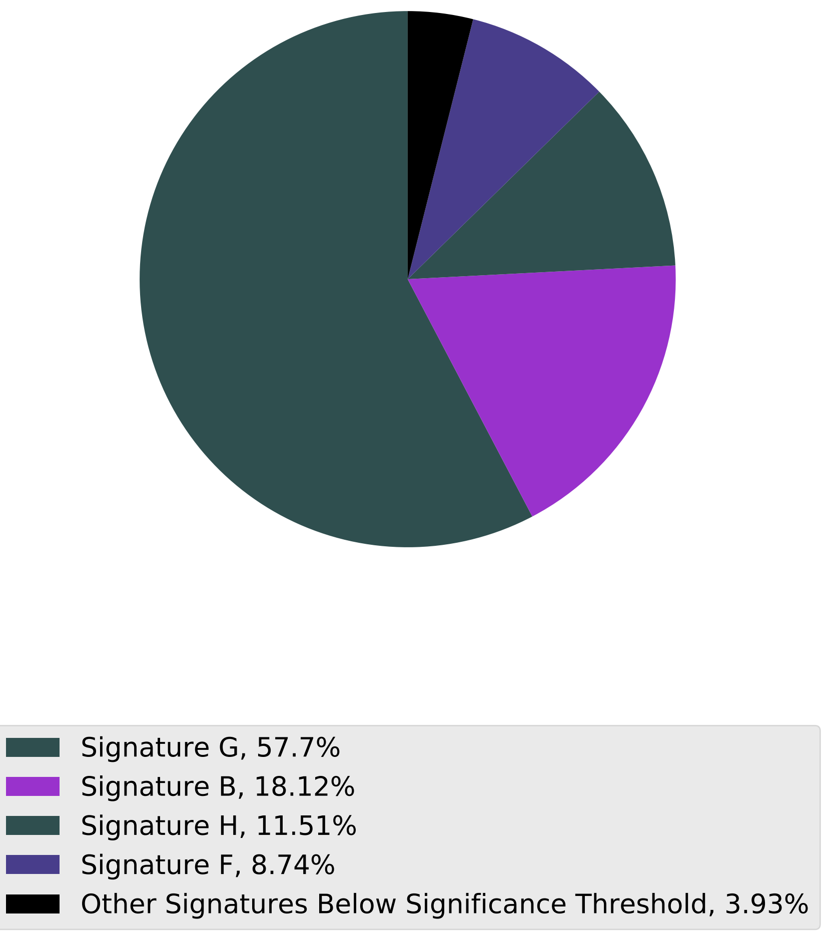

Tumor Profile for TCGA-AR-A0TX

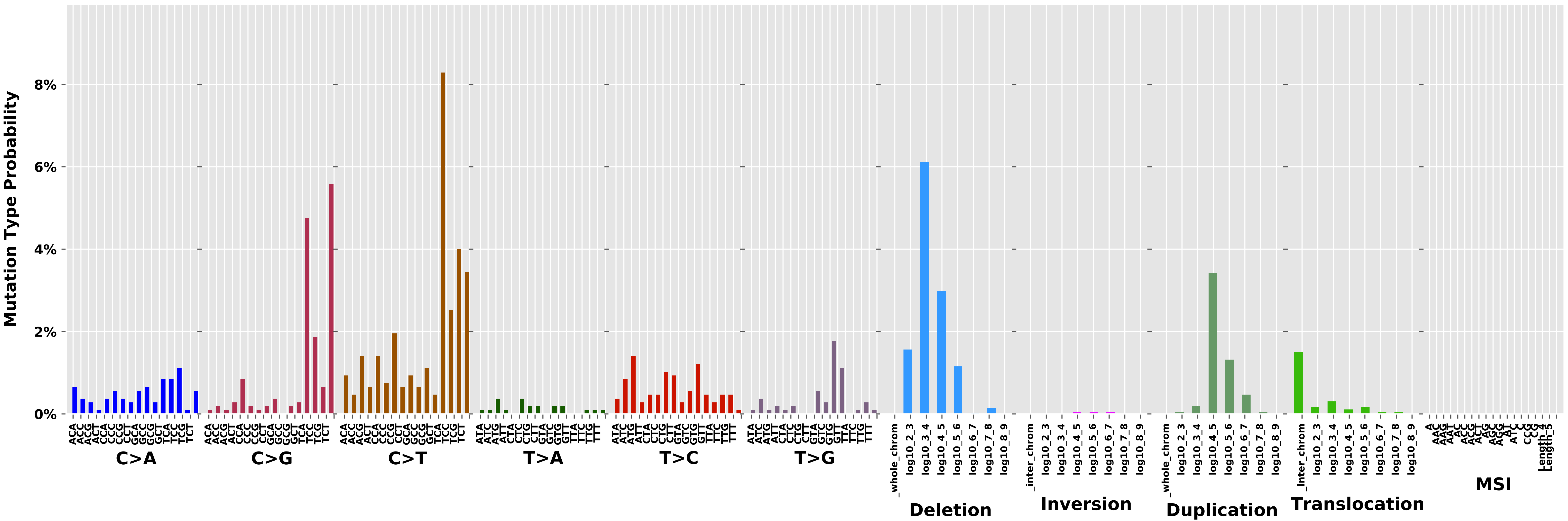

Cancer processes Weights for TCGA-EI-6917

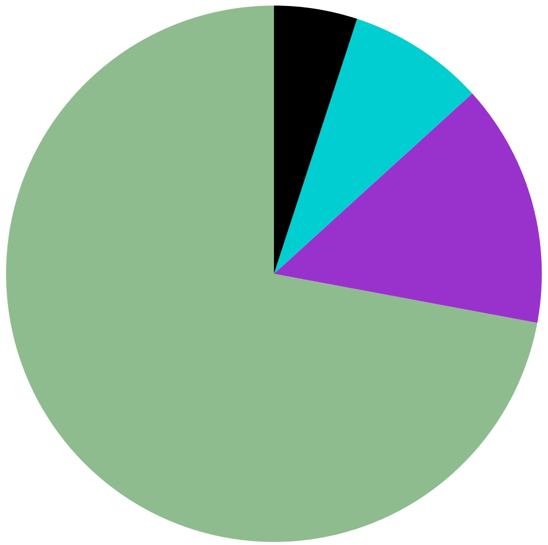

- Signature E, 72.07%
- Signature B, 14.66%
- Signature I, 8.24%
- Other Signatures Below Significance Threshold, 5.02%

Tumor Profile for TCGA-EI-6917

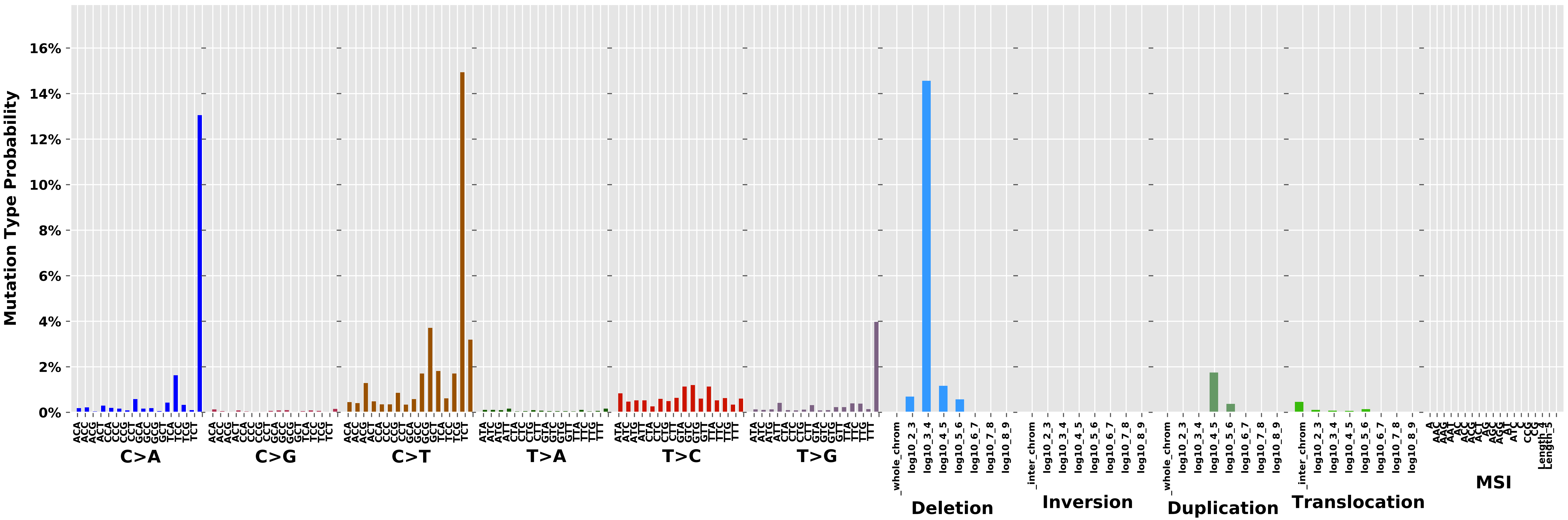

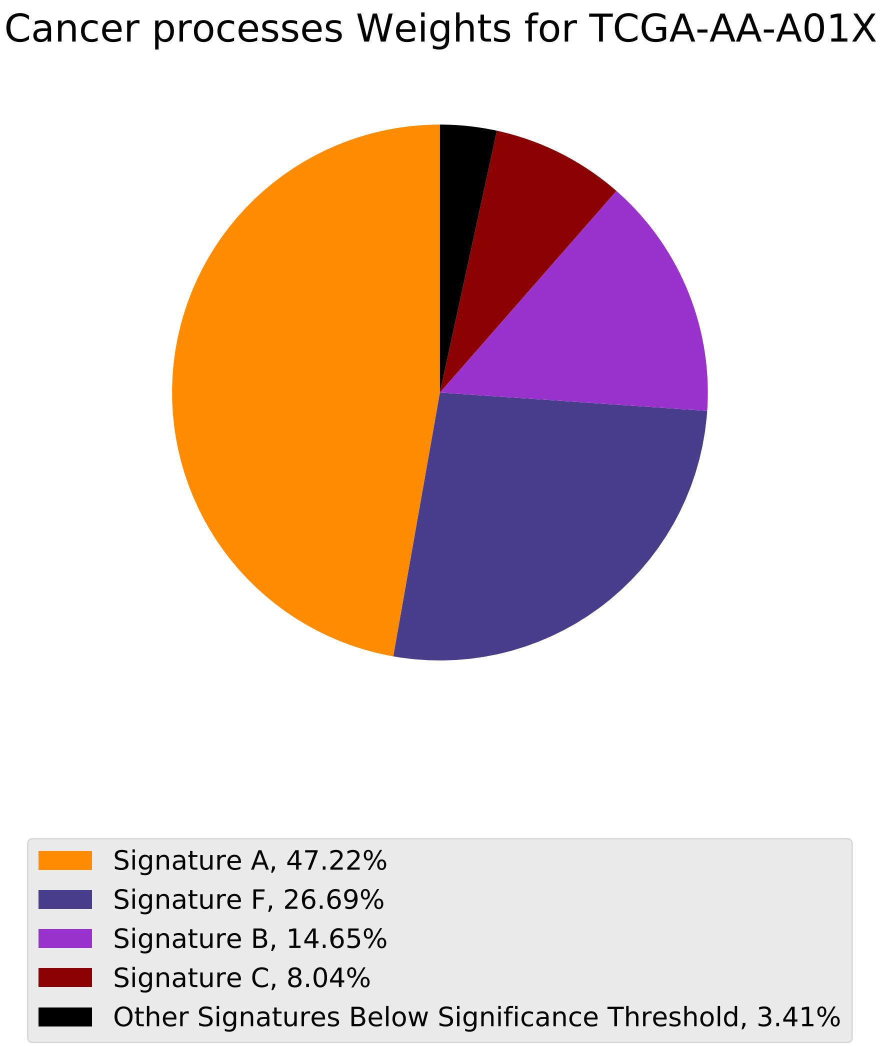

Cancer processes Weights for TCGA-AA-A01X
Signature A, 47.22%
Signature F, 26.69%
Signature B, 14.65%
Signature C, 8.04%
Other Signatures Below Significance Threshold, 3.41%

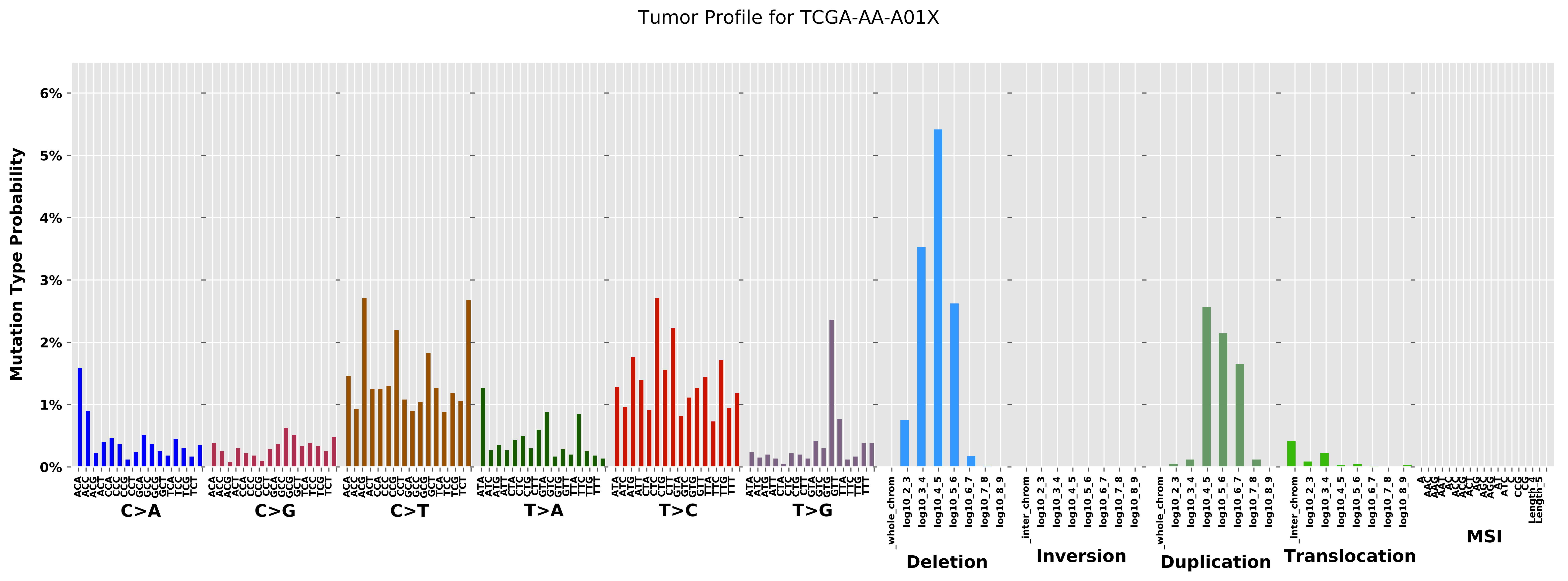
Tumor Profile for TCGA-AA-A01X
Mutation Type Probability
0%
1%
2%
3%
4%
5%
6%
C>A
C>G
C>T
T>A
T>C
T>G
Deletion
Inversion
Duplication
Translocation
MSI

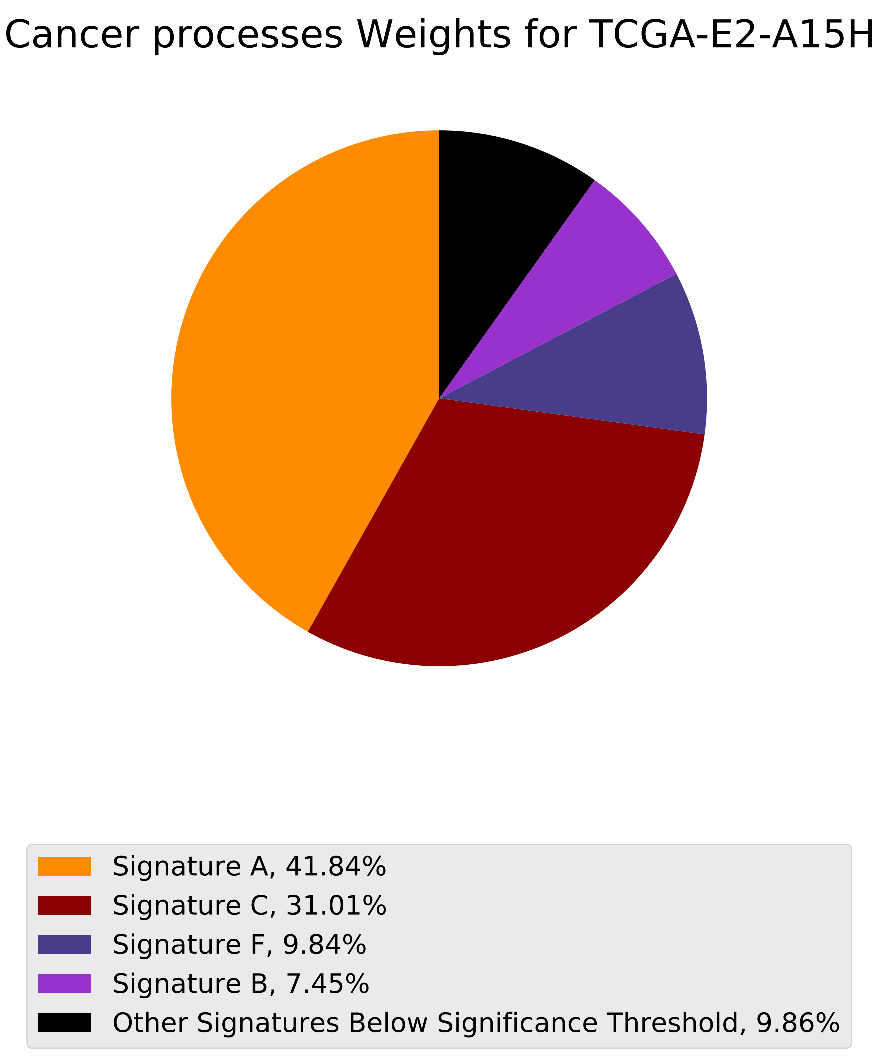
Cancer processes Weights for TCGA-E2-A15H
Signature A, 41.84%
Signature C, 31.01%
Signature F, 9.84%
Signature B, 7.45%
Other Signatures Below Significance Threshold, 9.86%

Tumor Profile for TCGA-E2-A15H

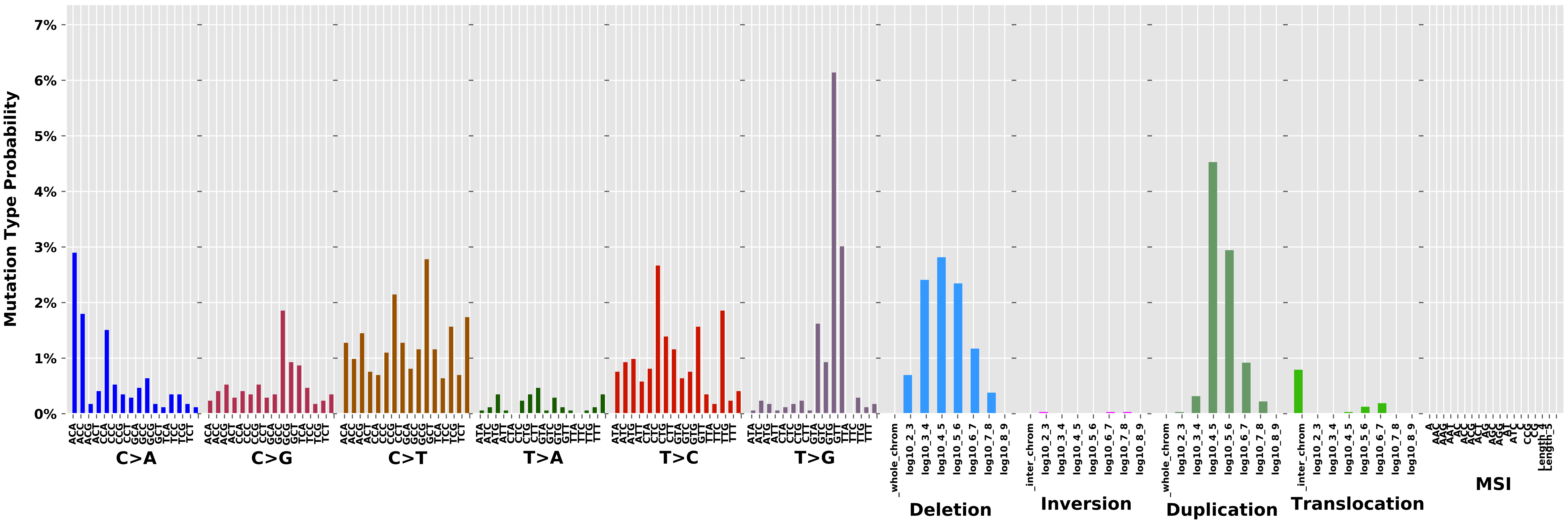

Cancer processes Weights for TCGA-E9-A1NH

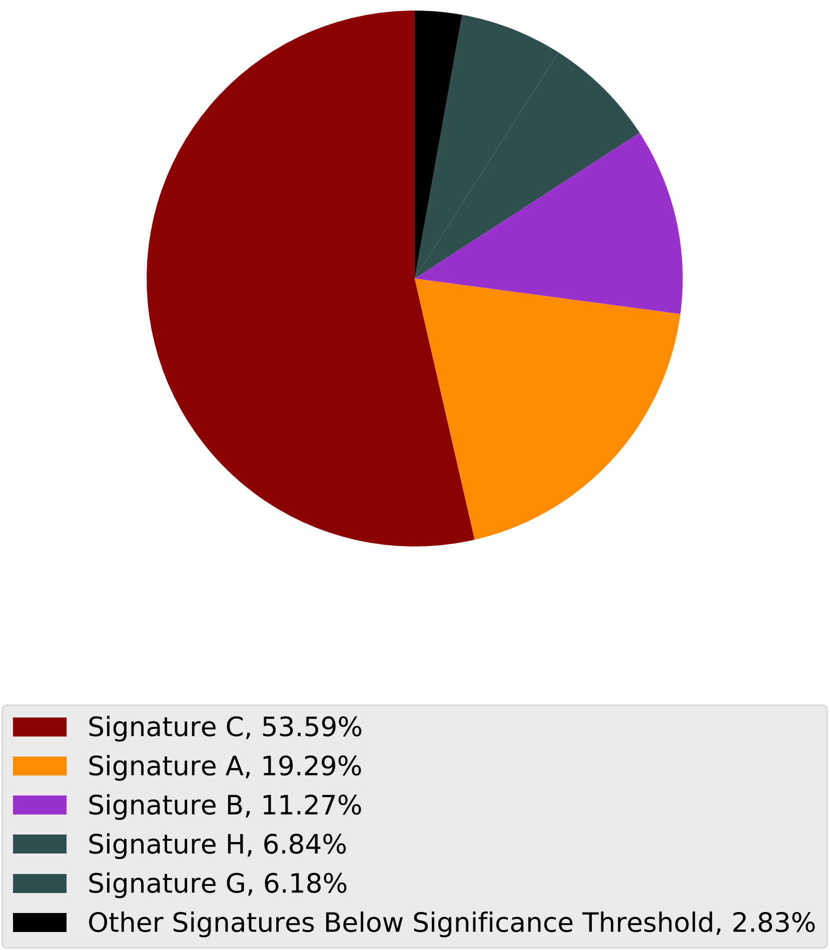

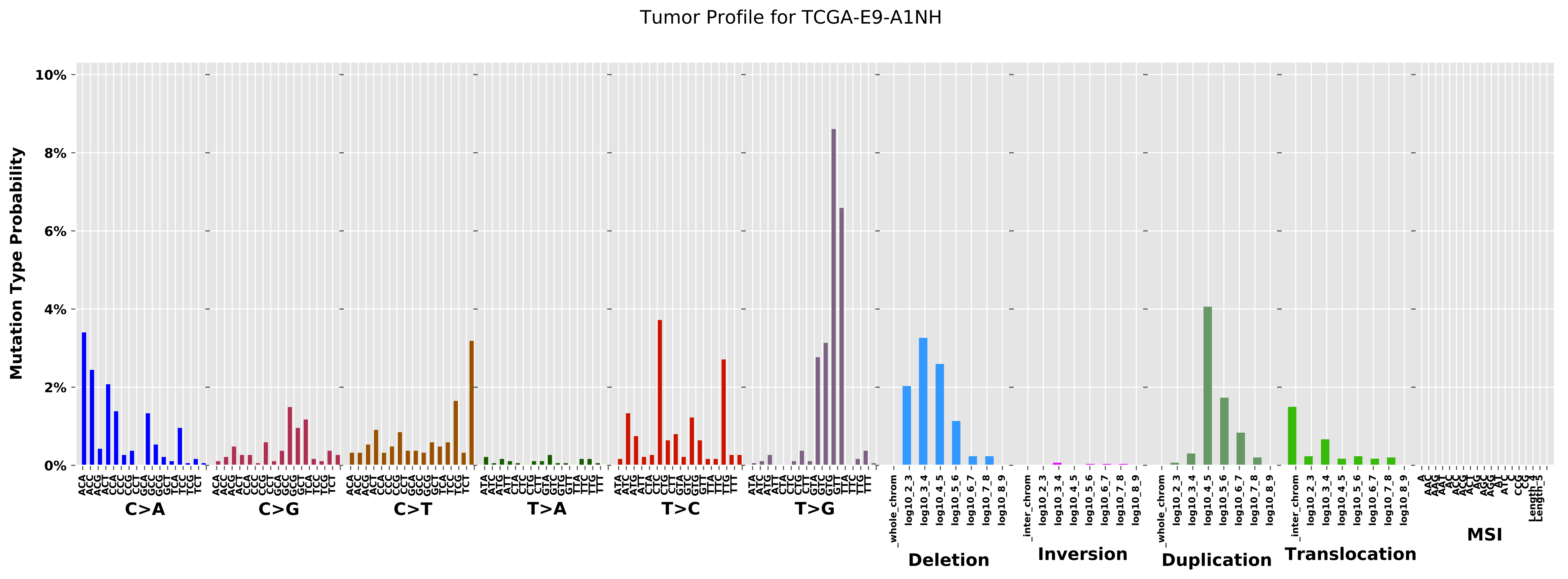

Tumor Profile for TCGA-E9-A1NH
Mutation Type Probability
0%
2%
4%
6%
8%
10%
C>A
C>G
C>T
T>A
T>C
T>G
Deletion
Inversion
Duplication
Translocation
MSI

Cancer processes Weights for TCGA-AA-A01R

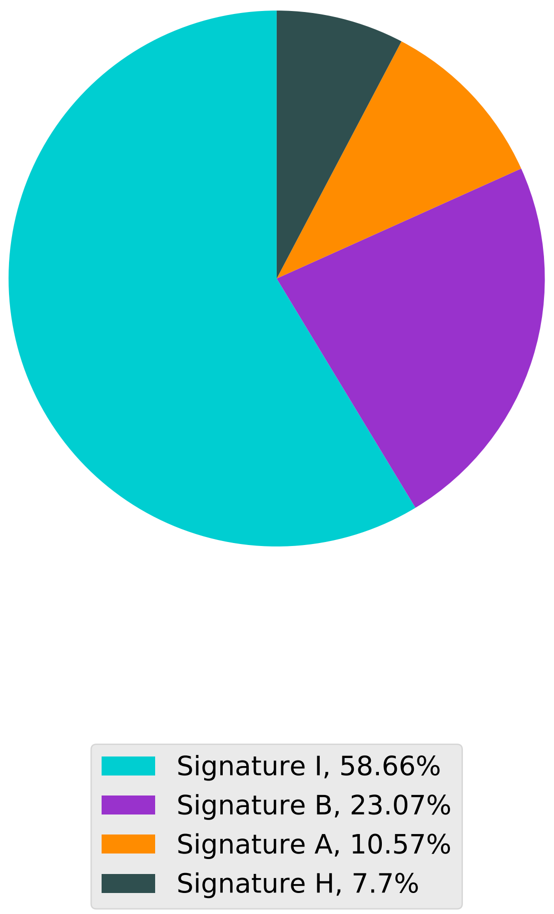

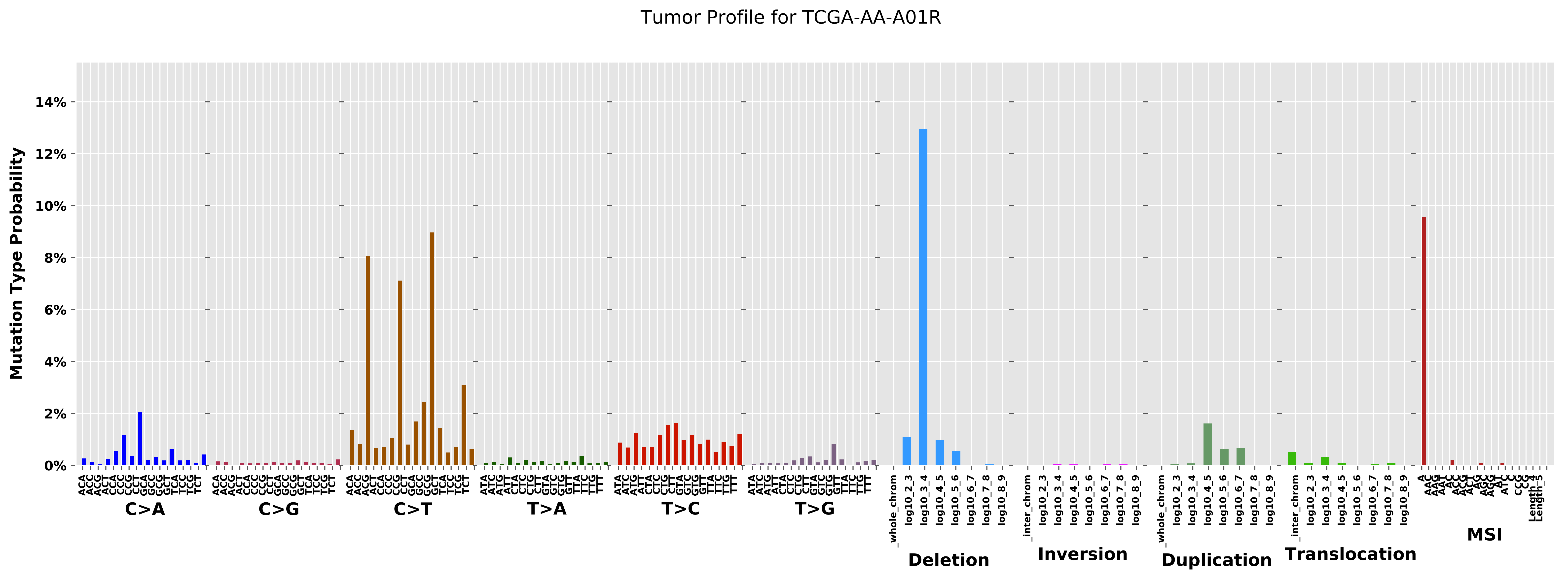

Tumor Profile for TCGA-AA-A01R
Mutation Type Probability
14%
12%
10%
8%
6%
4%
2%
0%
C>A
C>G
C>T
T>A
T>C
T>G
Deletion
Inversion
Duplication
Translocation
MSI

Cancer processes Weights for TCGA-AA-3666

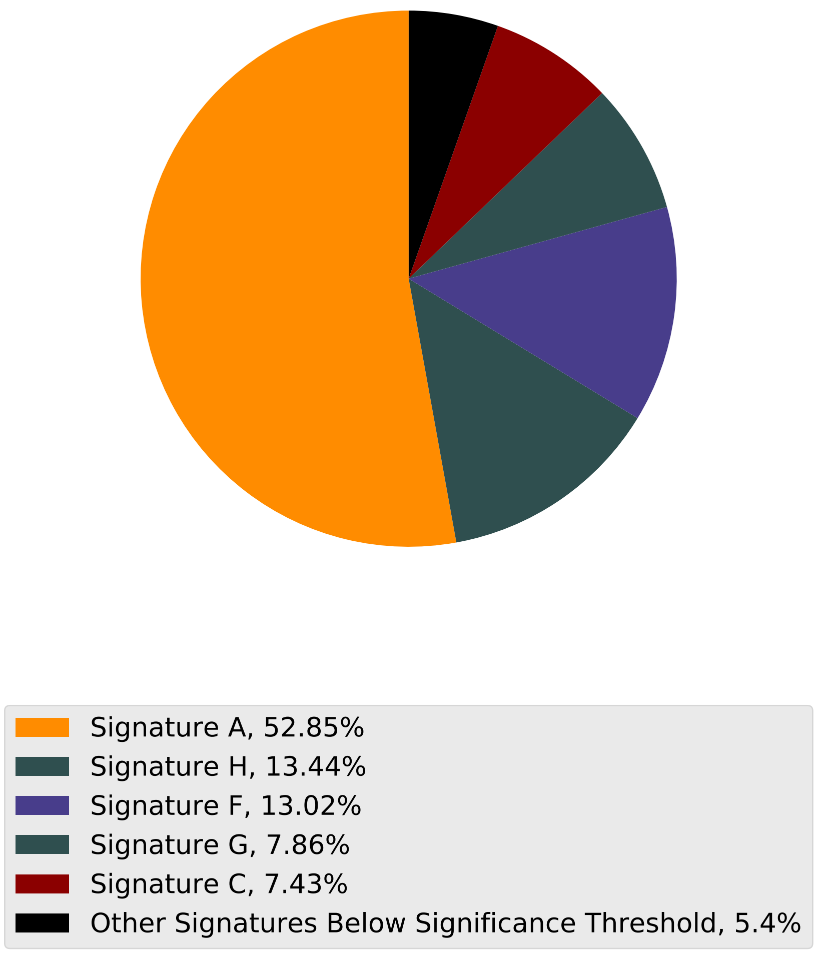

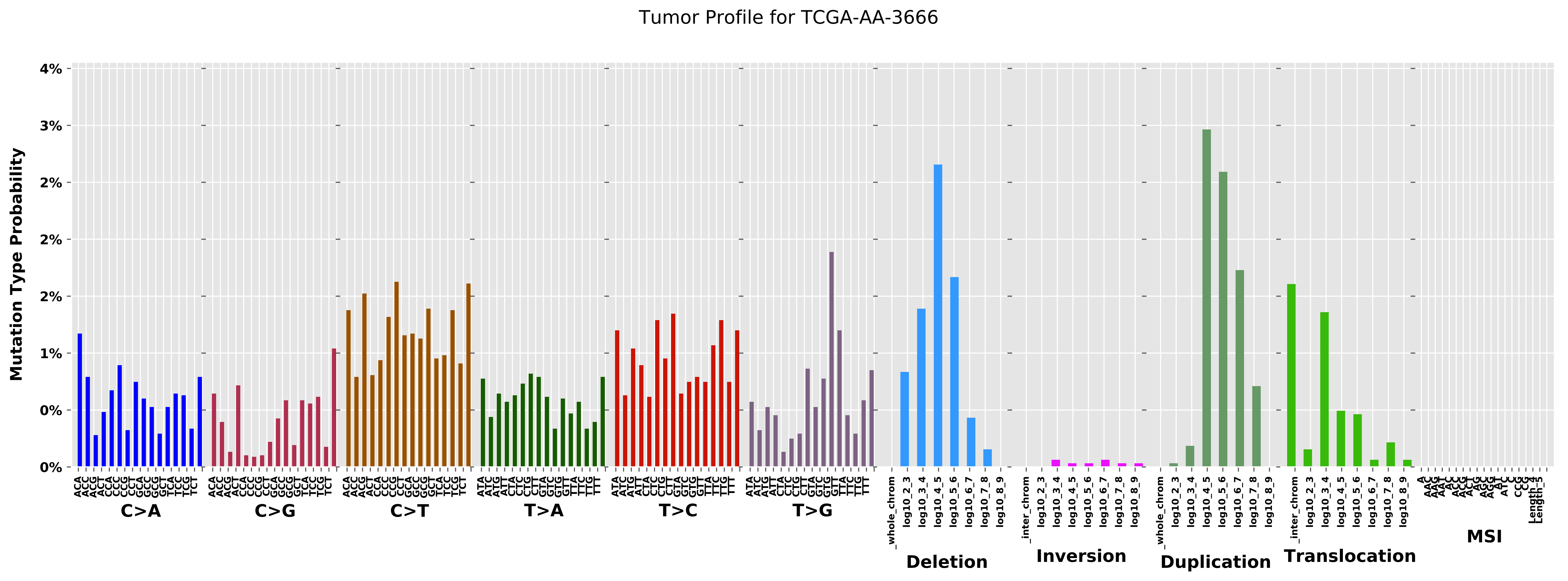
Tumor Profile for TCGA-AA-3666
Mutation Type Probability
4%
3%
2%
2%
2%
1%
0%
0%
C>A
C>G
C>T
T>A
T>C
T>G
Deletion
Inversion
Duplication
Translocation
MSI

Cancer processes Weights for TCGA-AR-A2LK

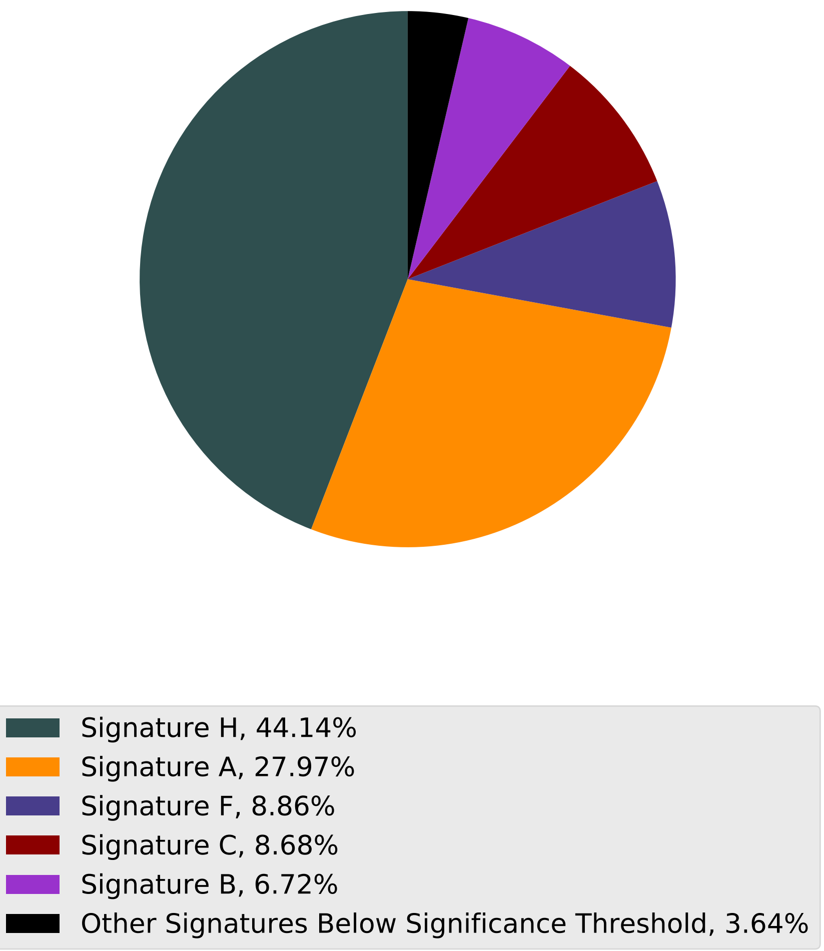

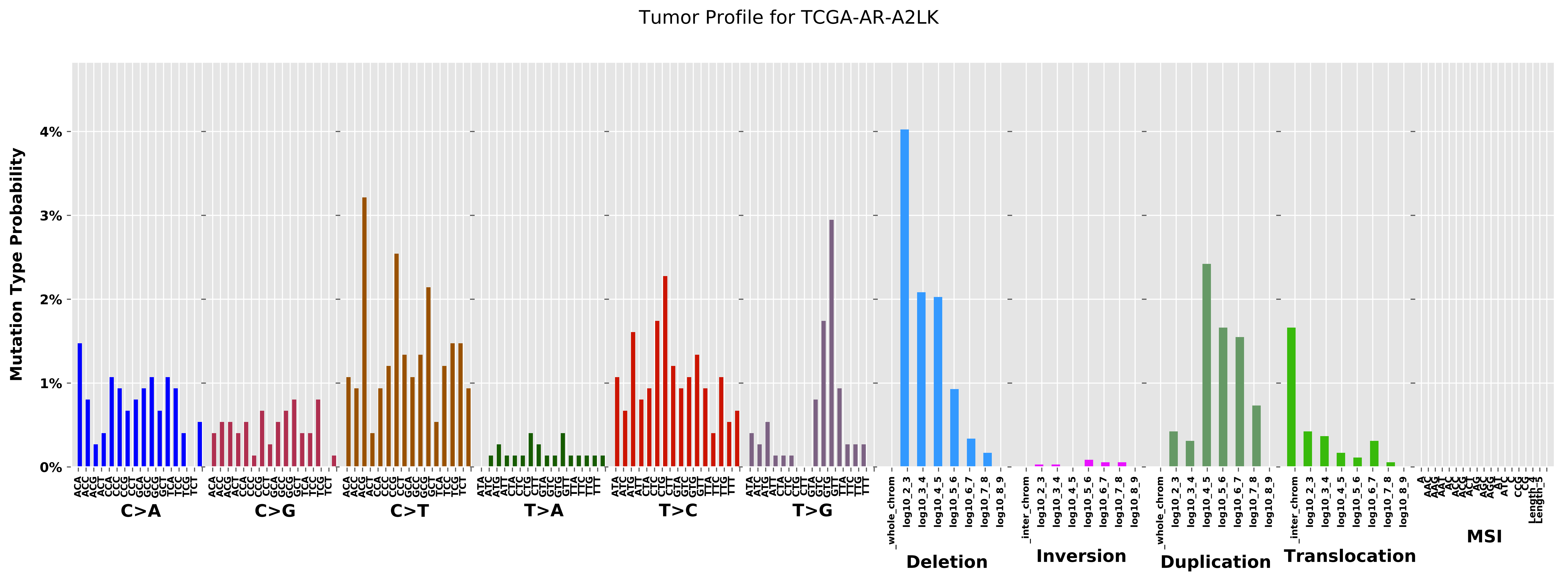

Tumor Profile for TCGA-AR-A2LK
Mutation Type Probability
4%
3%
2%
1%
0%
C>A
C>G
C>T
T>A
T>C
T>G
Deletion
Inversion
Duplication
Translocation
MSI

Cancer processes Weights for TCGA-AA-3534

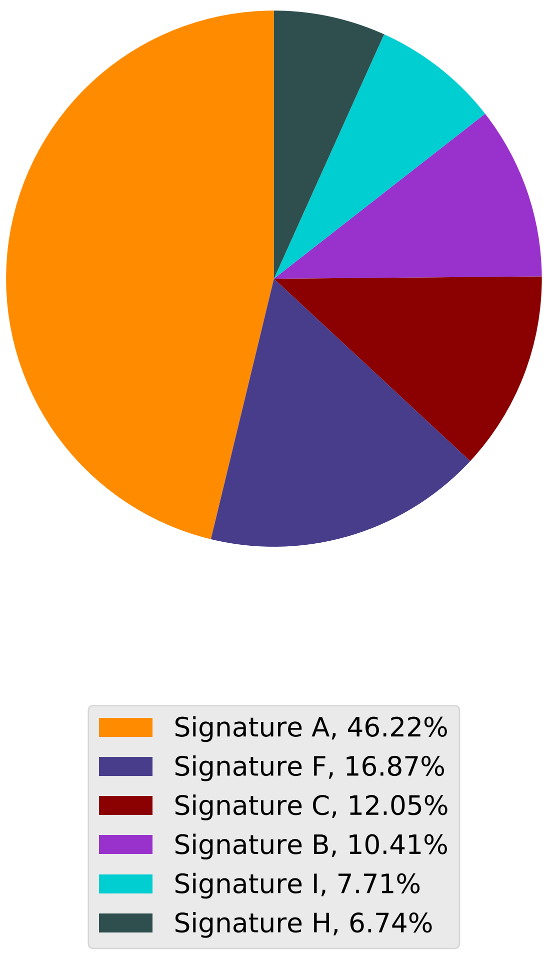

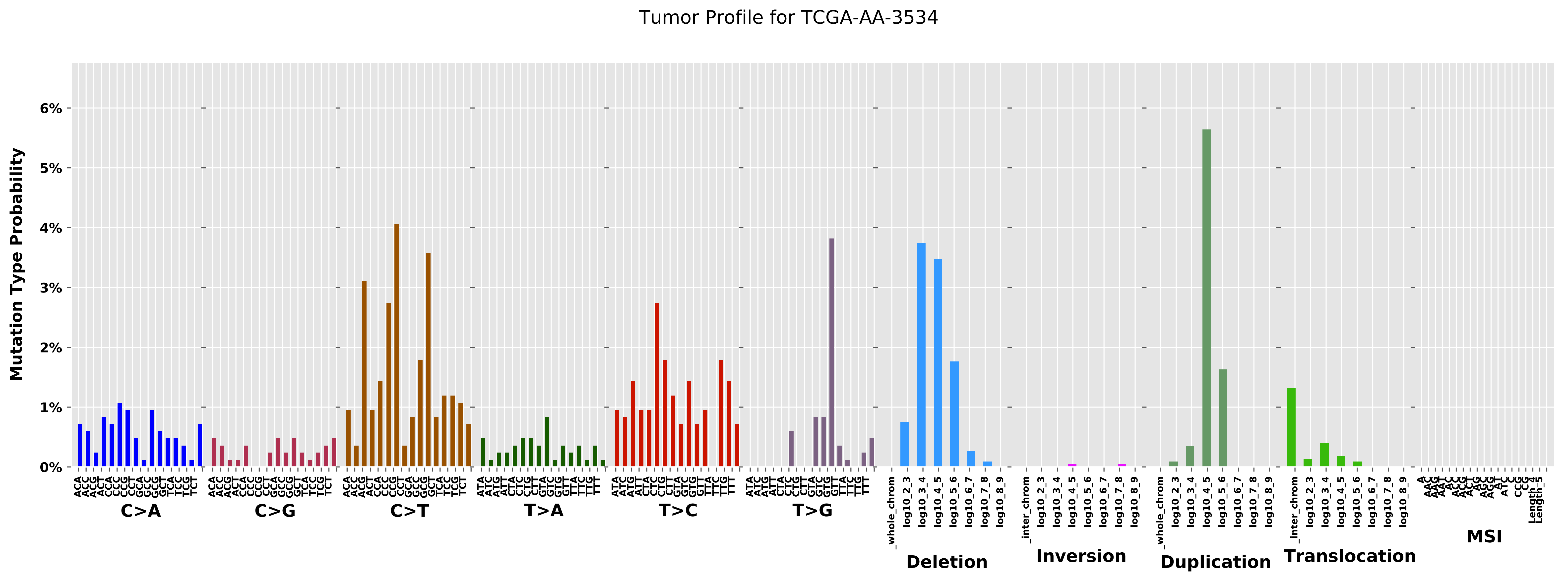

Tumor Profile for TCGA-AA-3534
Mutation Type Probability
0%
1%
2%
3%
4%
5%
6%
C>A
C>G
C>T
T>A
T>C
T>G
Deletion
Inversion
Duplication
Translocation
MSI

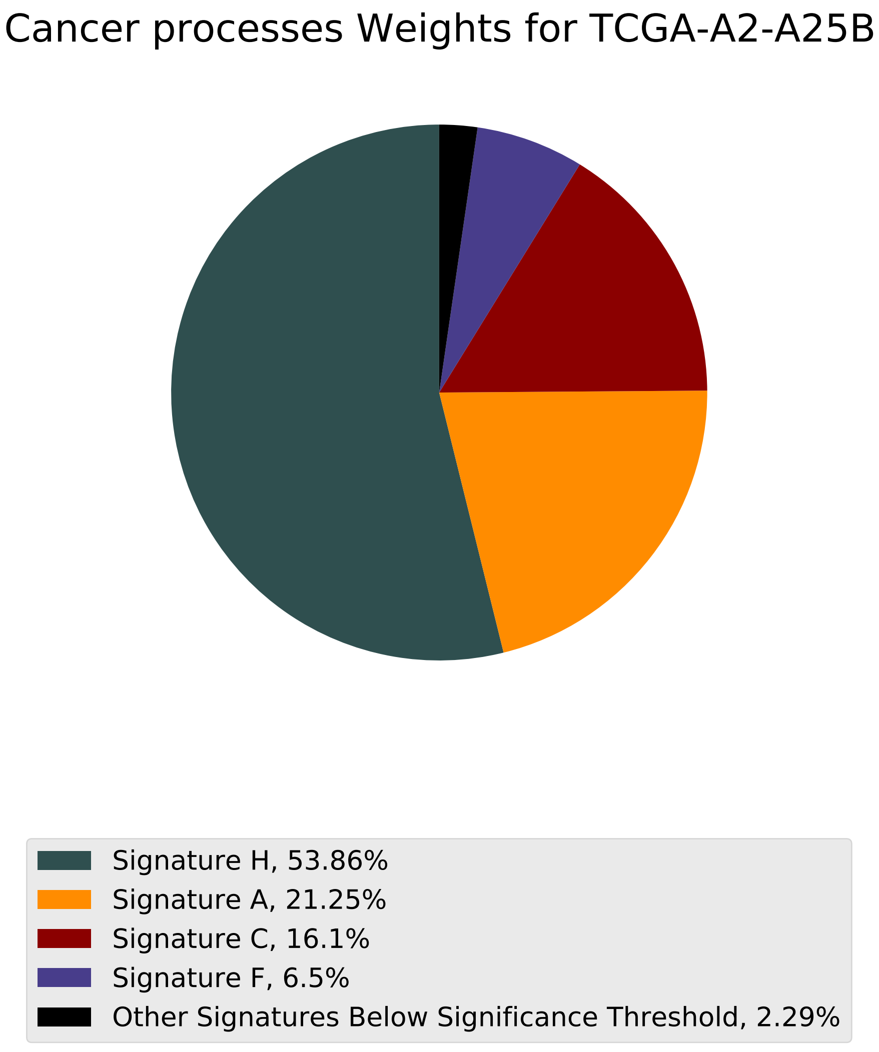
Cancer processes Weights for TCGA-A2-A25B
Signature H, 53.86%
Signature A, 21.25%
Signature C, 16.1%
Signature F, 6.5%
Other Signatures Below Significance Threshold, 2.29%

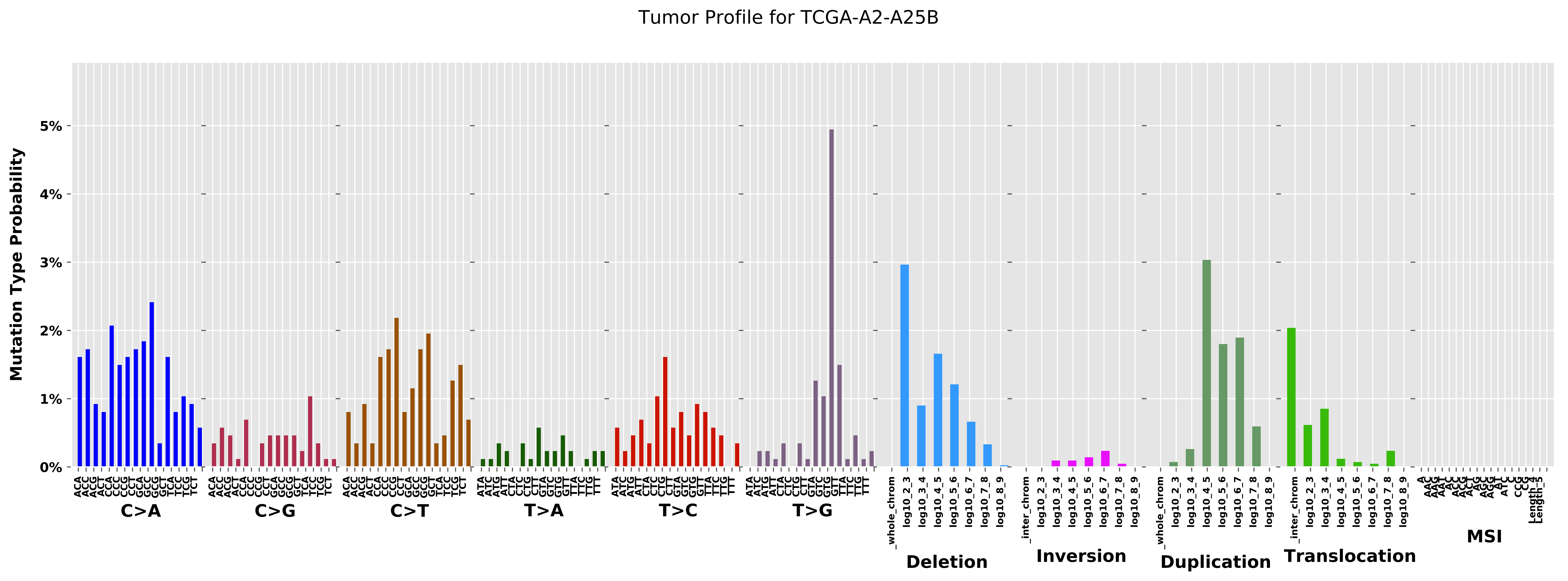

Tumor Profile for TCGA-A2-A25B
Mutation Type Probability
0%
1%
2%
3%
4%
5%
C>A
C>G
C>T
T>A
T>C
T>G
Deletion
Inversion
Duplication
Translocation
MSI

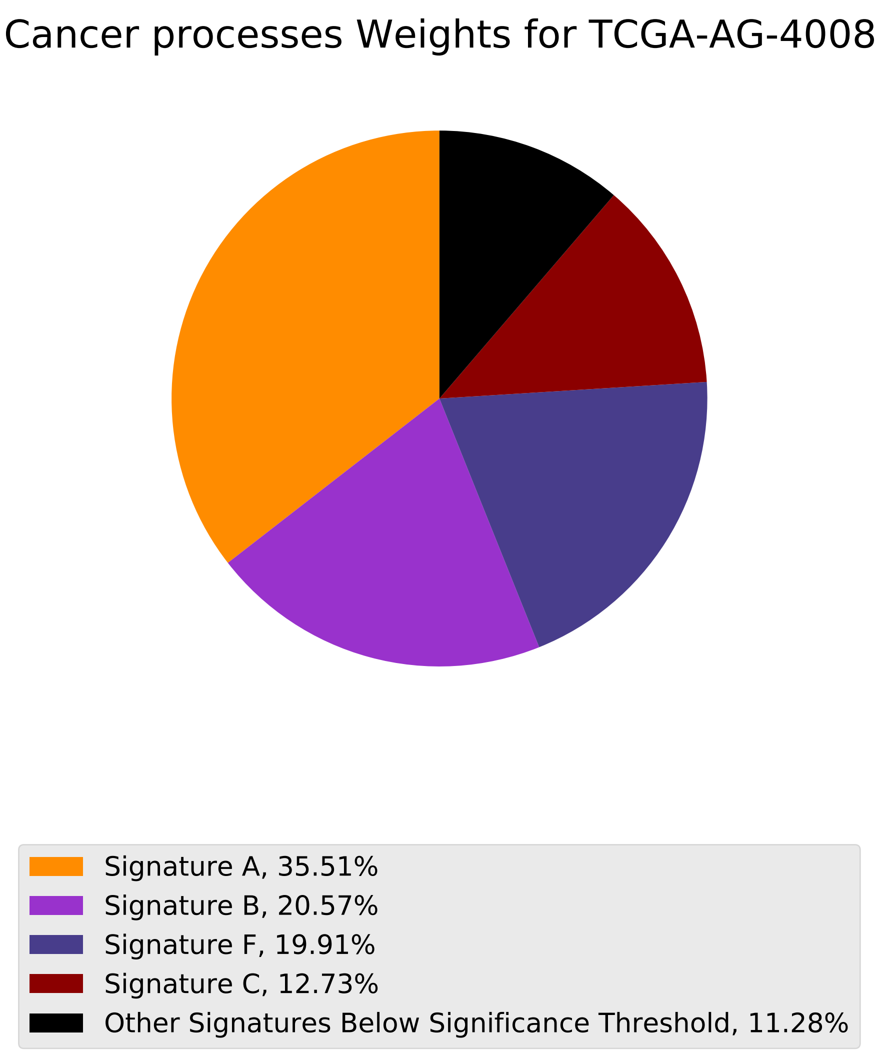
Cancer processes Weights for TCGA-AG-4008
Signature A, 35.51%
Signature B, 20.57%
Signature F, 19.91%
Signature C, 12.73%
Other Signatures Below Significance Threshold, 11.28%

Tumor Profile for TCGA-AG-4008

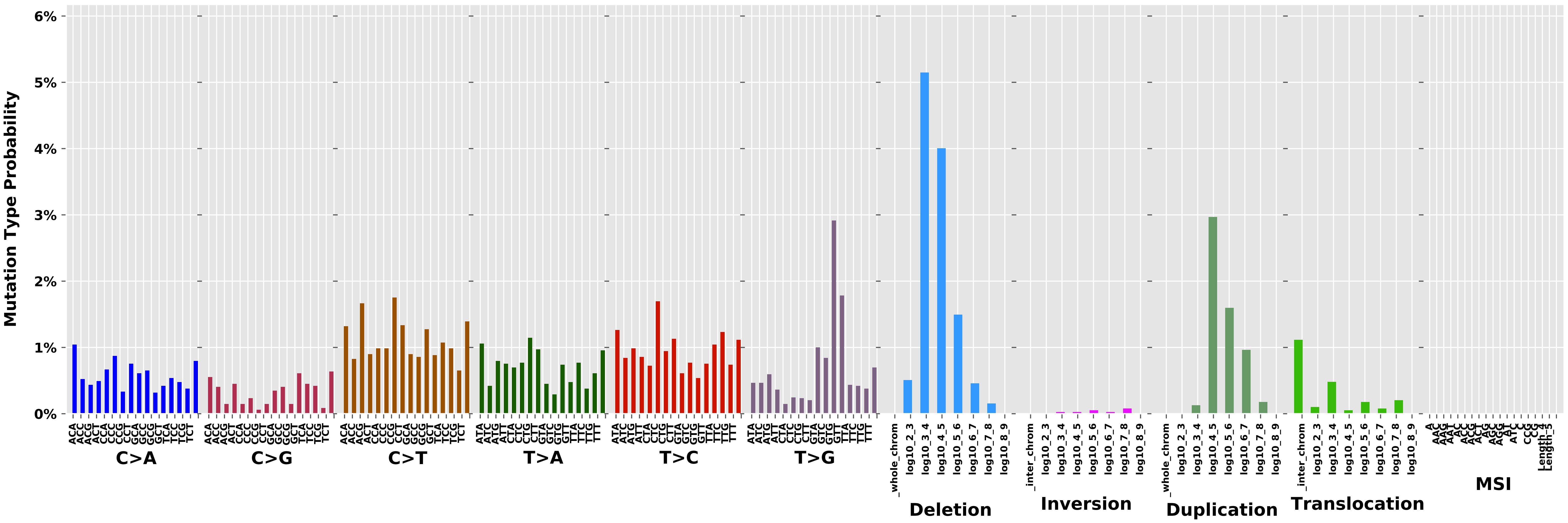

Cancer processes Weights for TCGA-A7-A26G

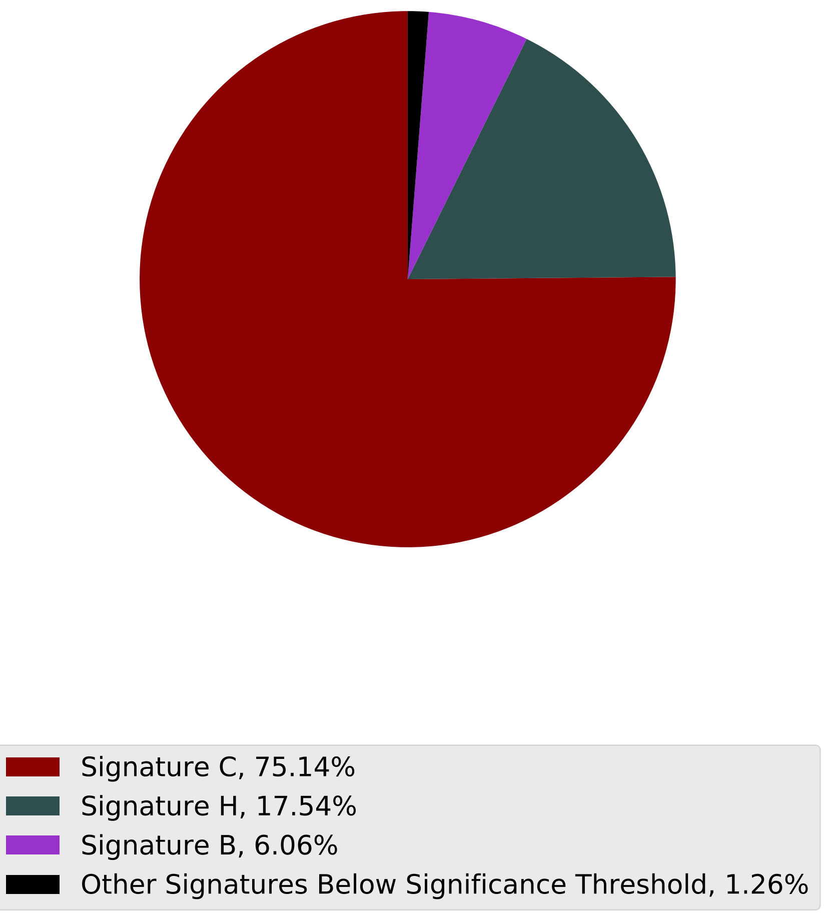

Tumor Profile for TCGA-A7-A26G

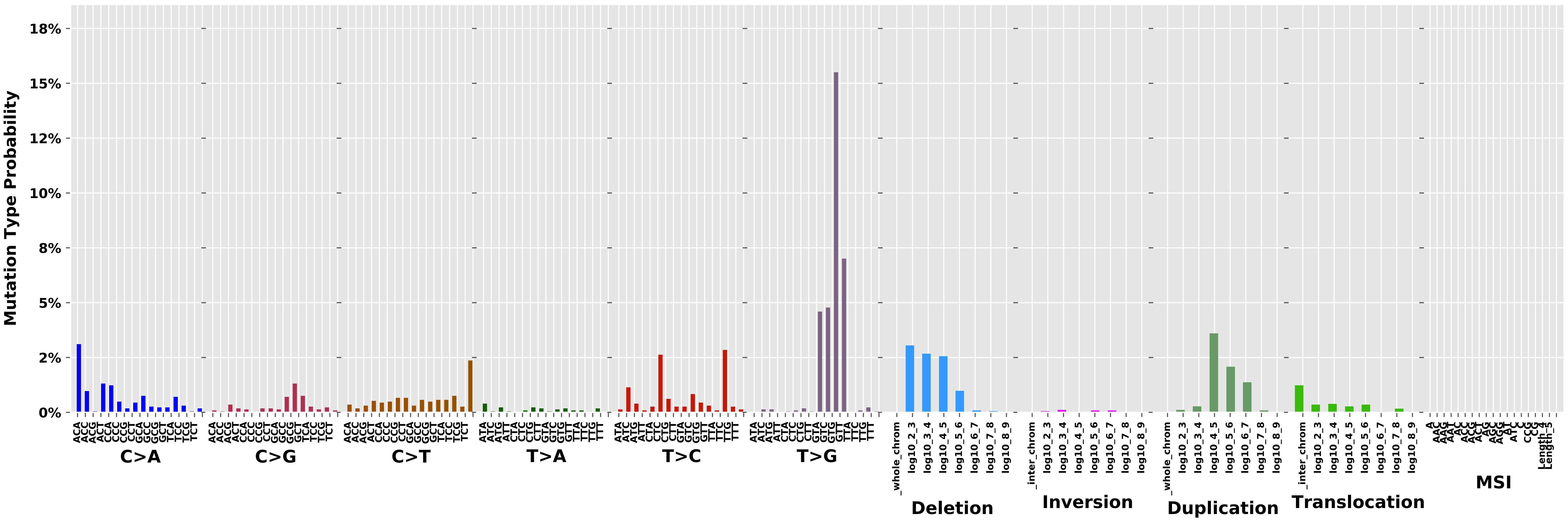

Cancer processes Weights for TCGA-AR-A24Z

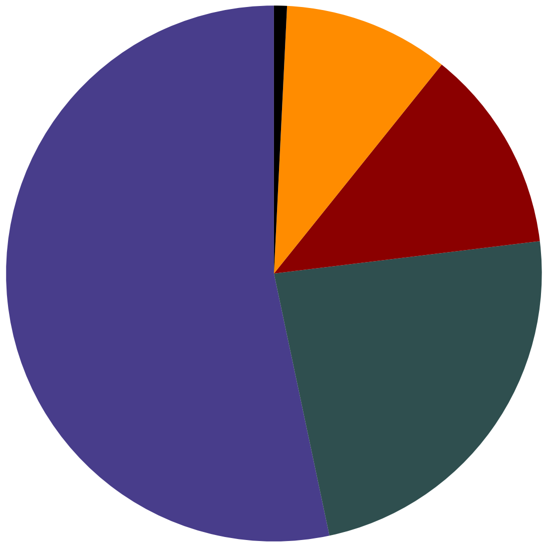

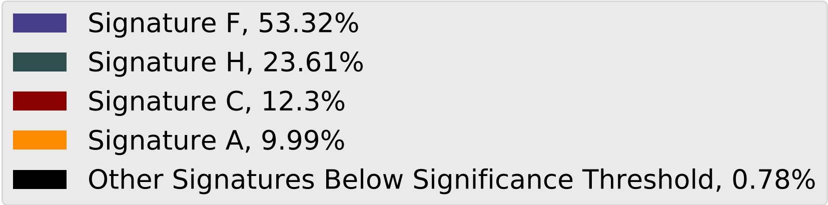

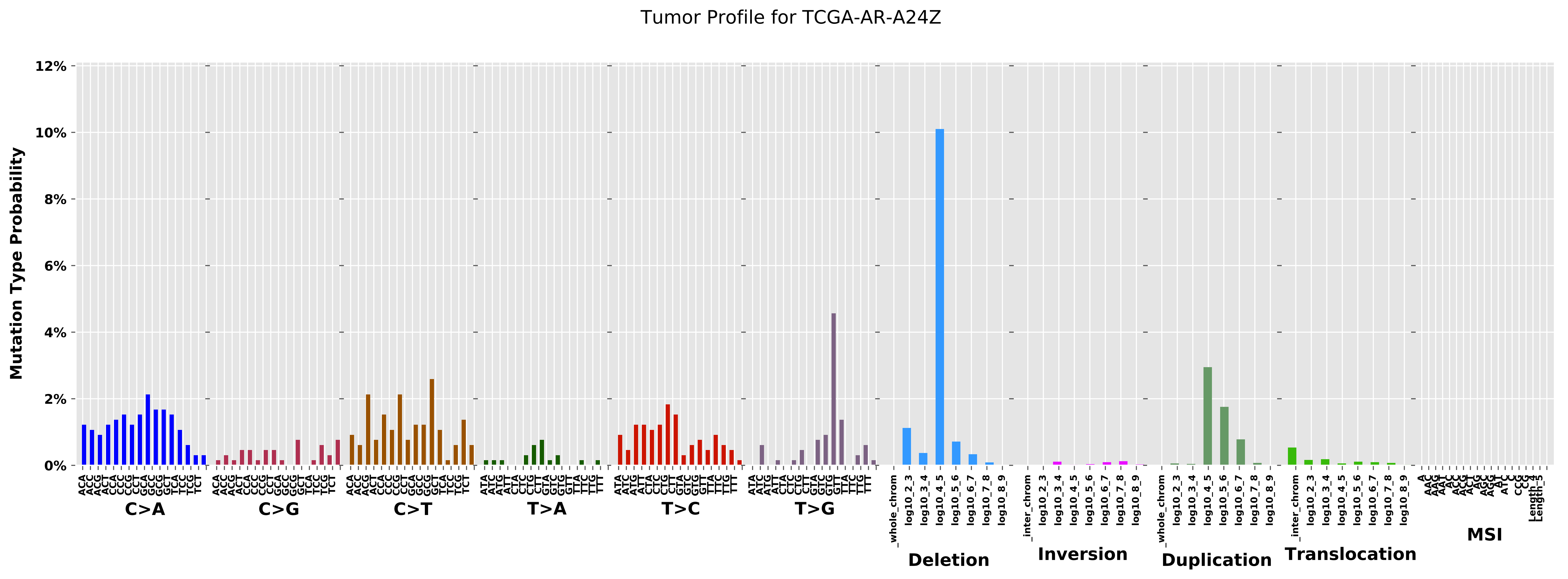

Tumor Profile for TCGA-AR-A24Z
Mutation Type Probability
12%
10%
8%
6%
4%
2%
0%
C>A
C>G
C>T
T>A
T>C
T>G
Deletion
Inversion
Duplication
Translocation
MSI
_whole_chrom
_inter_chrom
log10_2_3
log10_3_4
log10_4_5
log10_5_6
log10_6_7
log10_7_8
log10_8_9

Cancer processes Weights for TCGA-A2-A04P

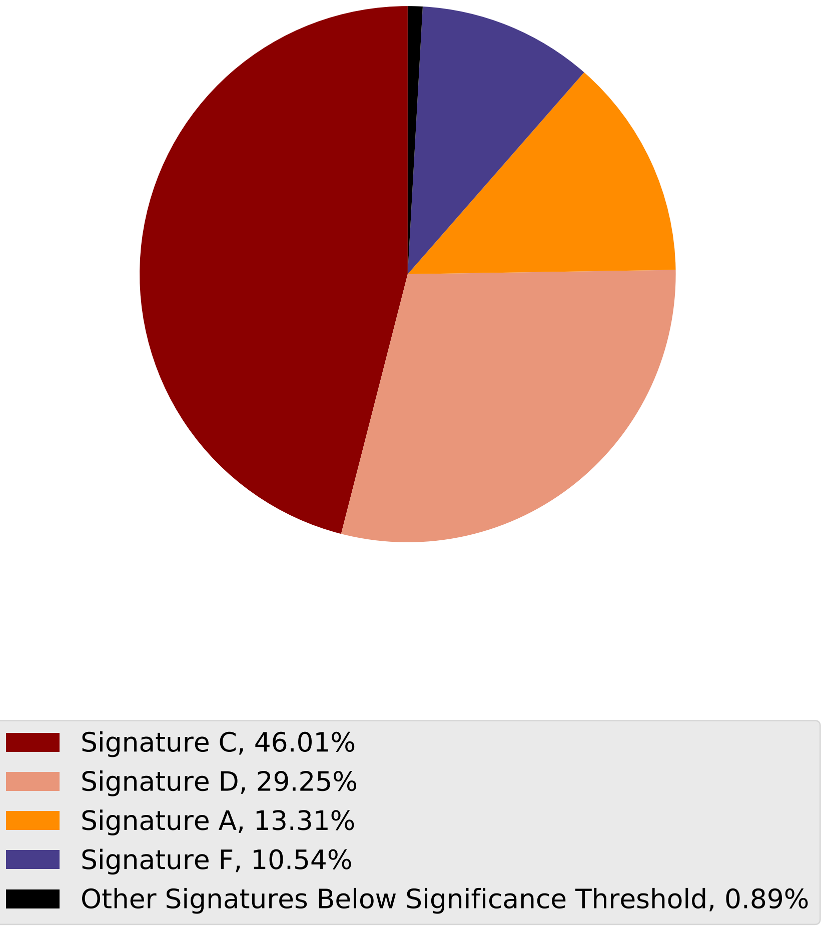

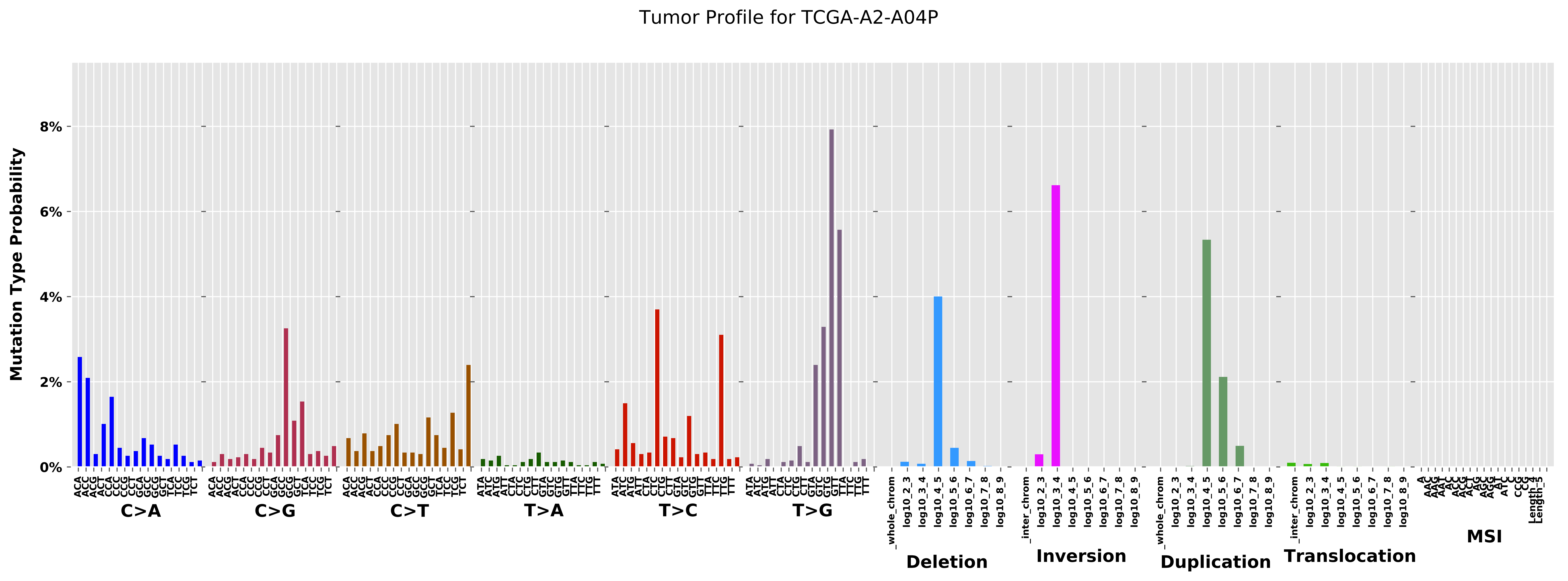
Tumor Profile for TCGA-A2-A04P
Mutation Type Probability
0%
2%
4%
6%
8%
C>A
C>G
C>T
T>A
T>C
T>G
Deletion
Inversion
Duplication
Translocation
MSI
_whole_chrom
_inter_chrom
log10_2_3
log10_3_4
log10_4_5
log10_5_6
log10_6_7
log10_7_8
log10_8_9

Cancer processes Weights for TCGA-CA-6718

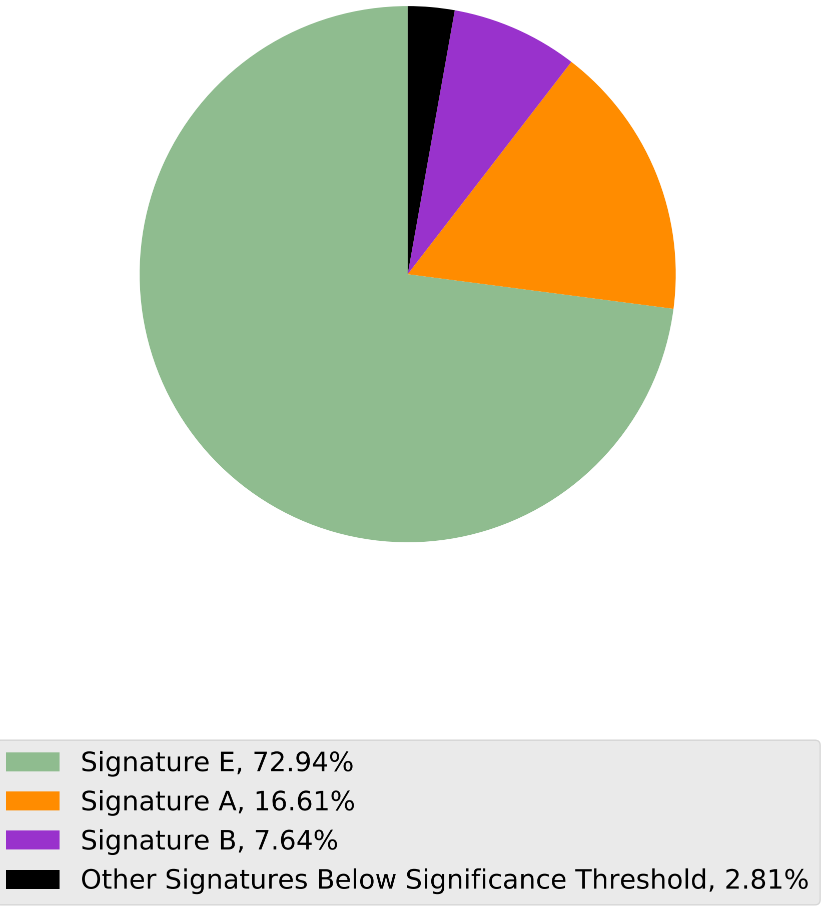

Tumor Profile for TCGA-CA-6718

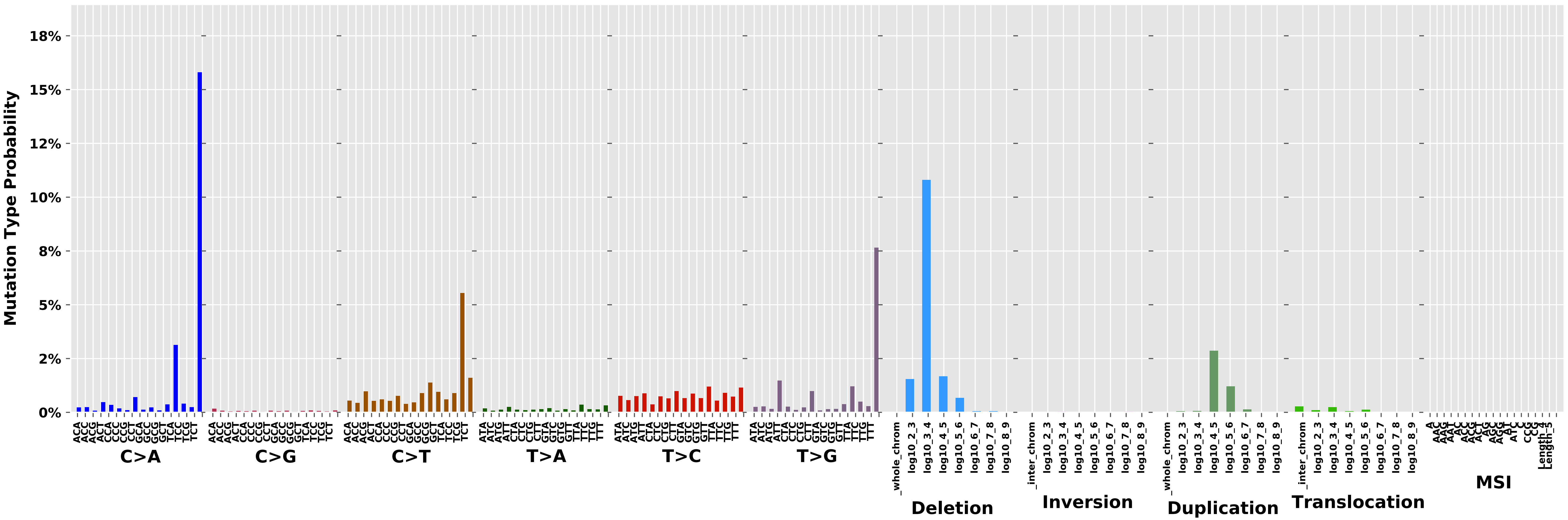

Cancer processes Weights for TCGA-BH-A0AV

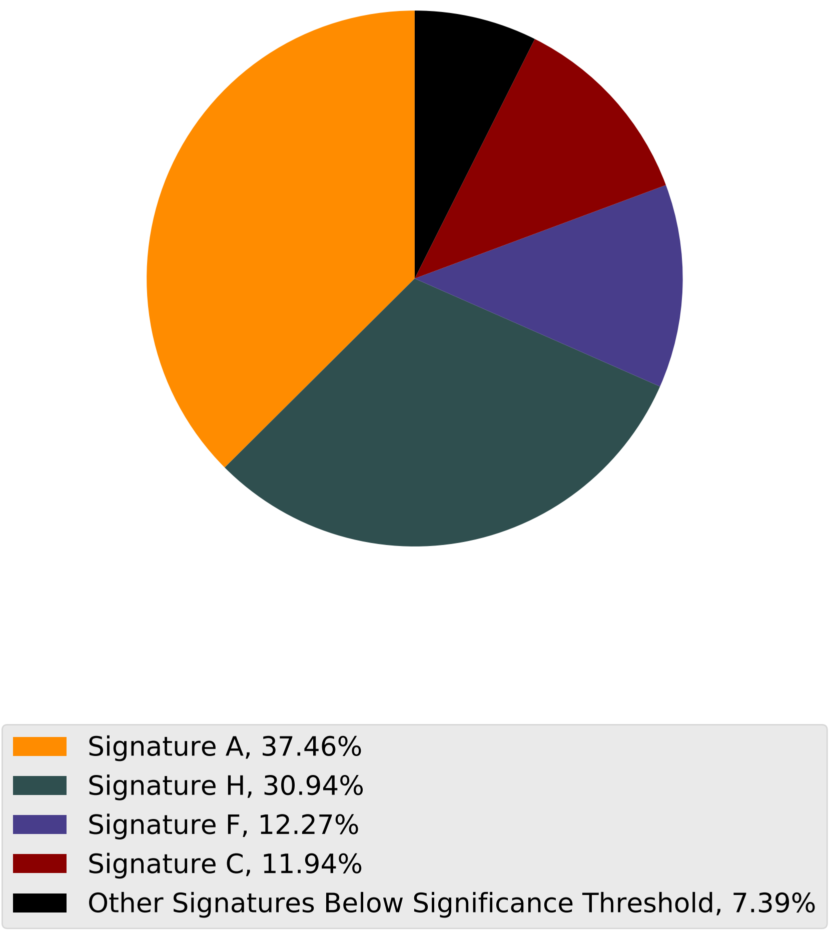

Tumor Profile for TCGA-BH-A0AV

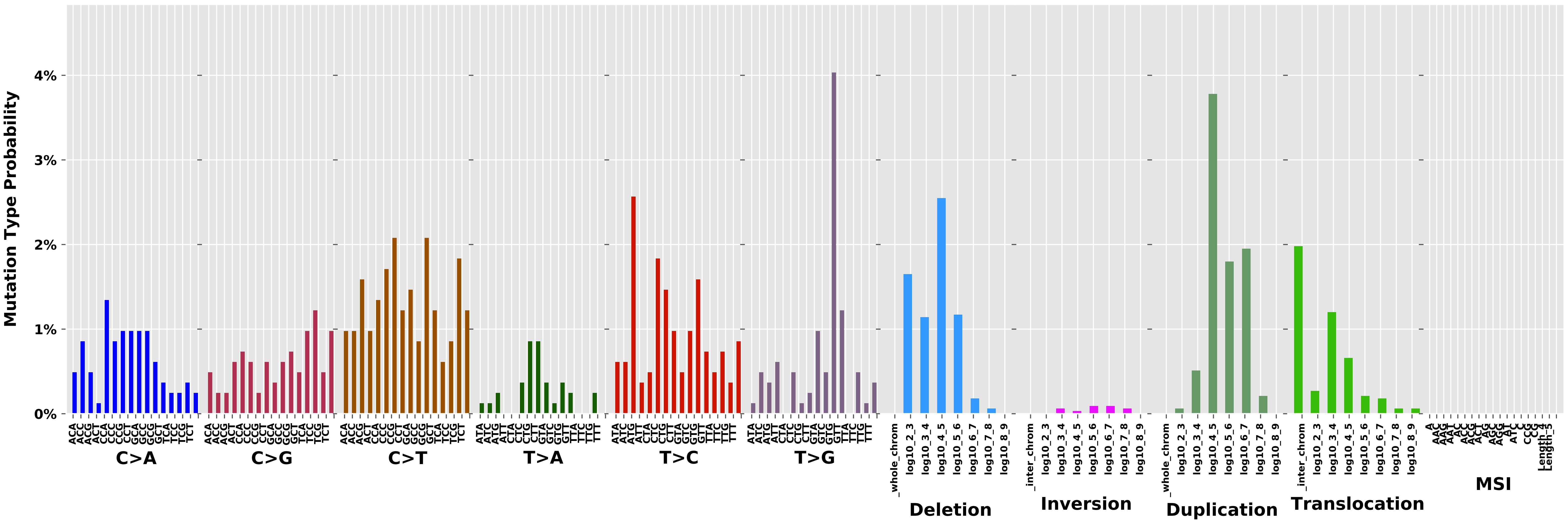

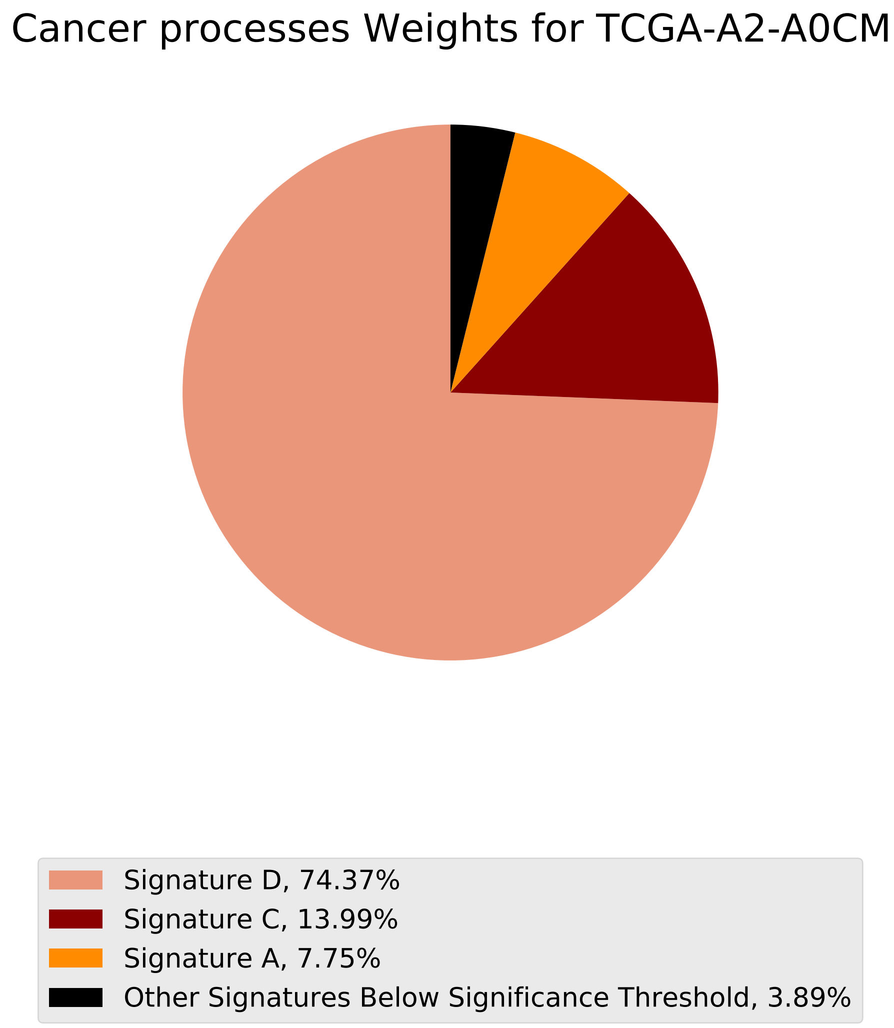
Cancer processes Weights for TCGA-A2-A0CM
Signature D, 74.37%
Signature C, 13.99%
Signature A, 7.75%
Other Signatures Below Significance Threshold, 3.89%

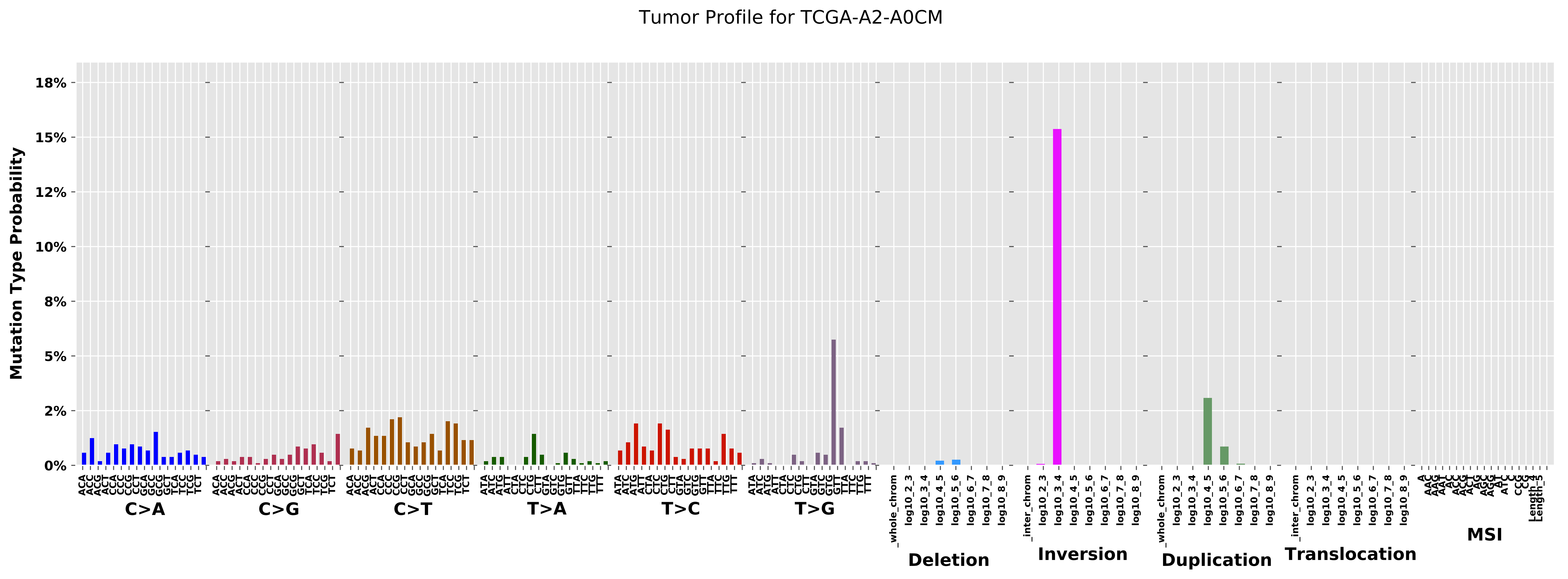

Tumor Profile for TCGA-A2-A0CM
Mutation Type Probability
0%
2%
5%
8%
10%
12%
15%
18%
C>A
C>G
C>T
T>A
T>C
T>G
Deletion
Inversion
Duplication
Translocation
MSI
_whole_chrom
_inter_chrom
log10_2_3
log10_3_4
log10_4_5
log10_5_6
log10_6_7
log10_7_8
log10_8_9

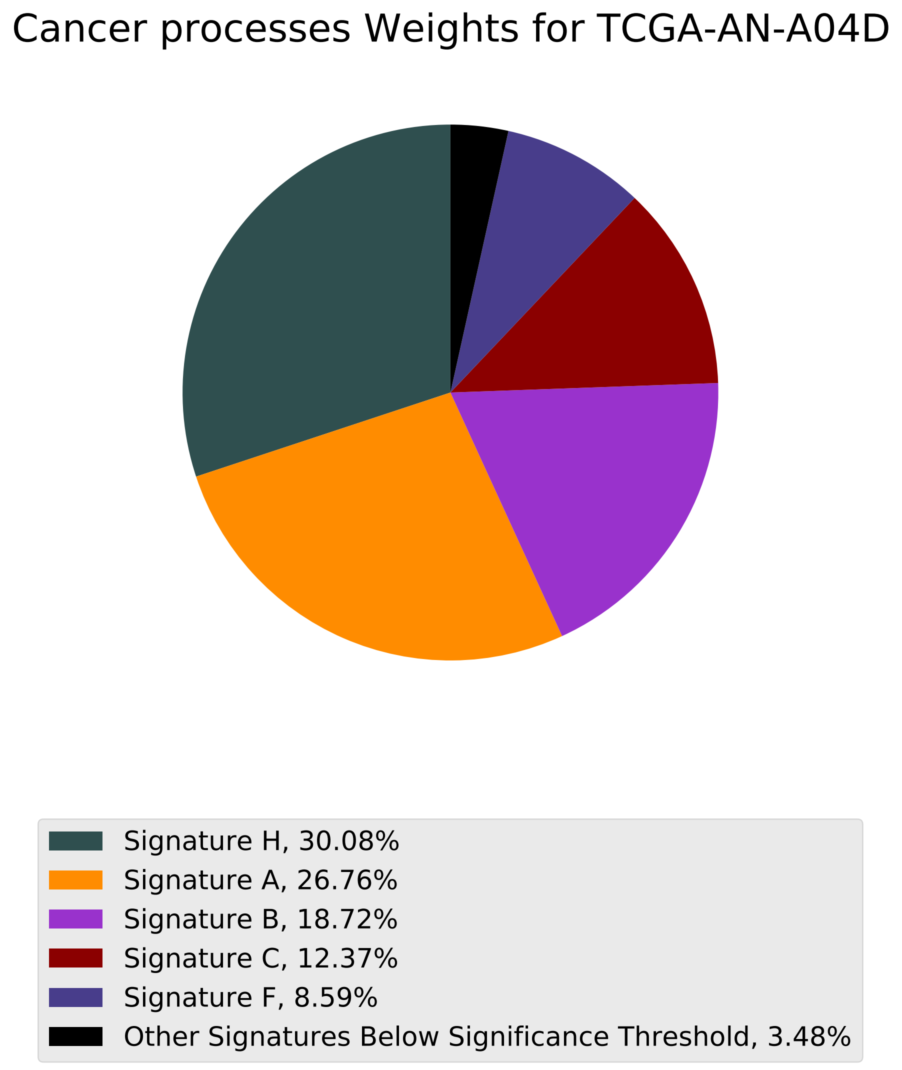
Cancer processes Weights for TCGA-AN-A04D
Signature H, 30.08%
Signature A, 26.76%
Signature B, 18.72%
Signature C, 12.37%
Signature F, 8.59%
Other Signatures Below Significance Threshold, 3.48%

Tumor Profile for TCGA-AN-A04D

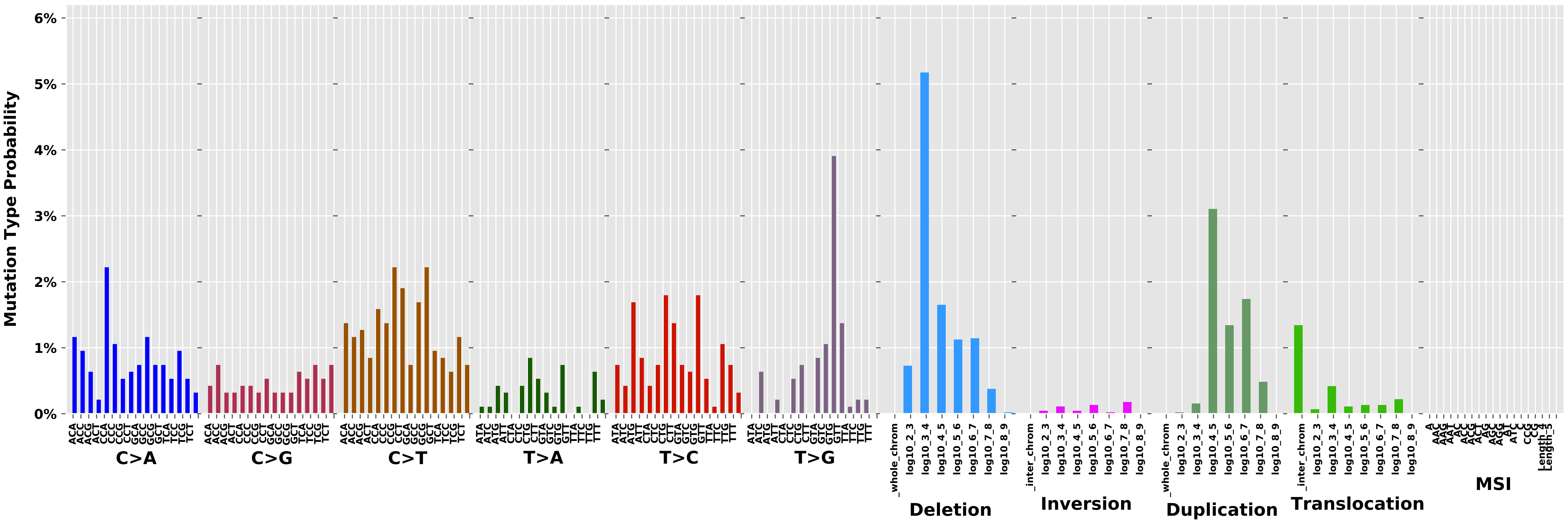

Cancer processes Weights for TCGA-A8-A092

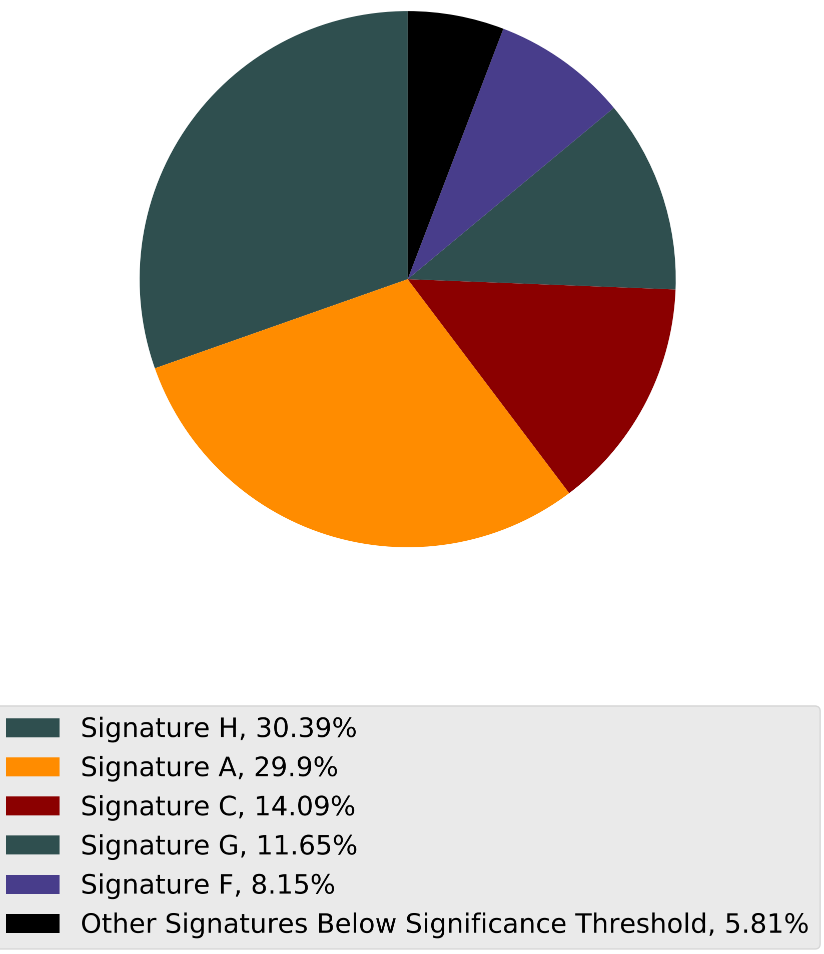

Tumor Profile for TCGA-A8-A092

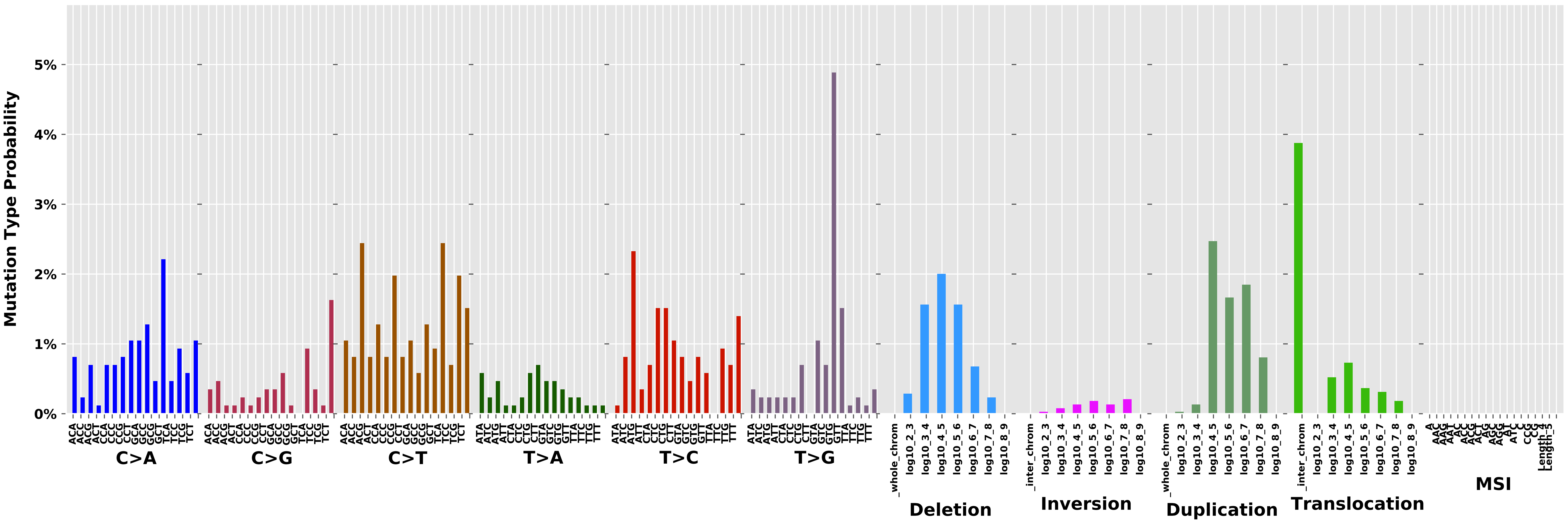

Cancer processes Weights for TCGA-A2-A04X

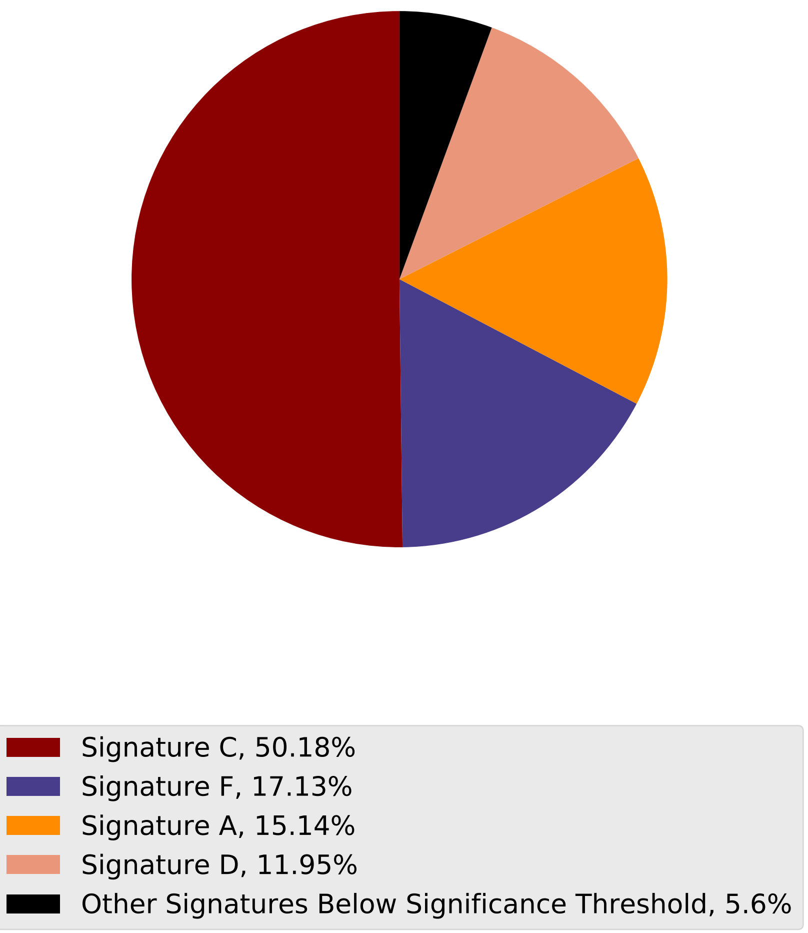

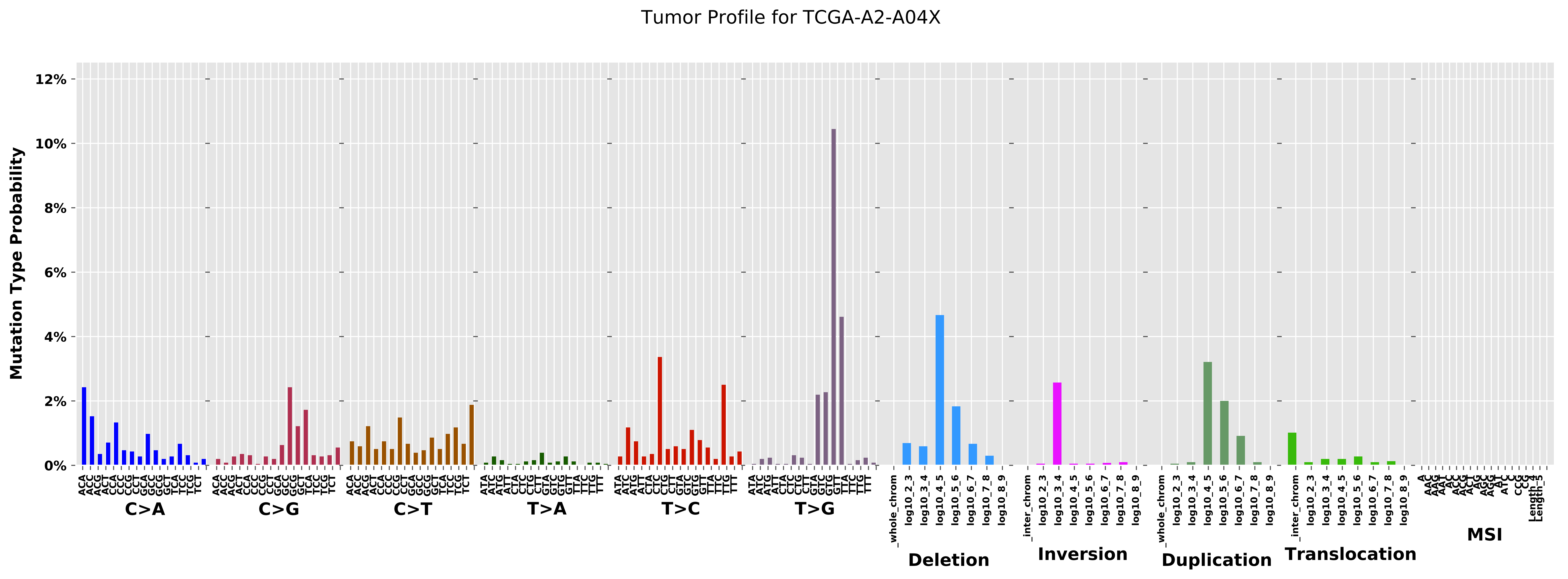

Tumor Profile for TCGA-A2-A04X
Mutation Type Probability
0%
2%
4%
6%
8%
10%
12%
C>A
C>G
C>T
T>A
T>C
T>G
Deletion
Inversion
Duplication
Translocation
MSI
_whole_chrom
_inter_chrom
log10_2_3
log10_3_4
log10_4_5
log10_5_6
log10_6_7
log10_7_8
log10_8_9

Cancer processes Weights for TCGA-A2-A0D2

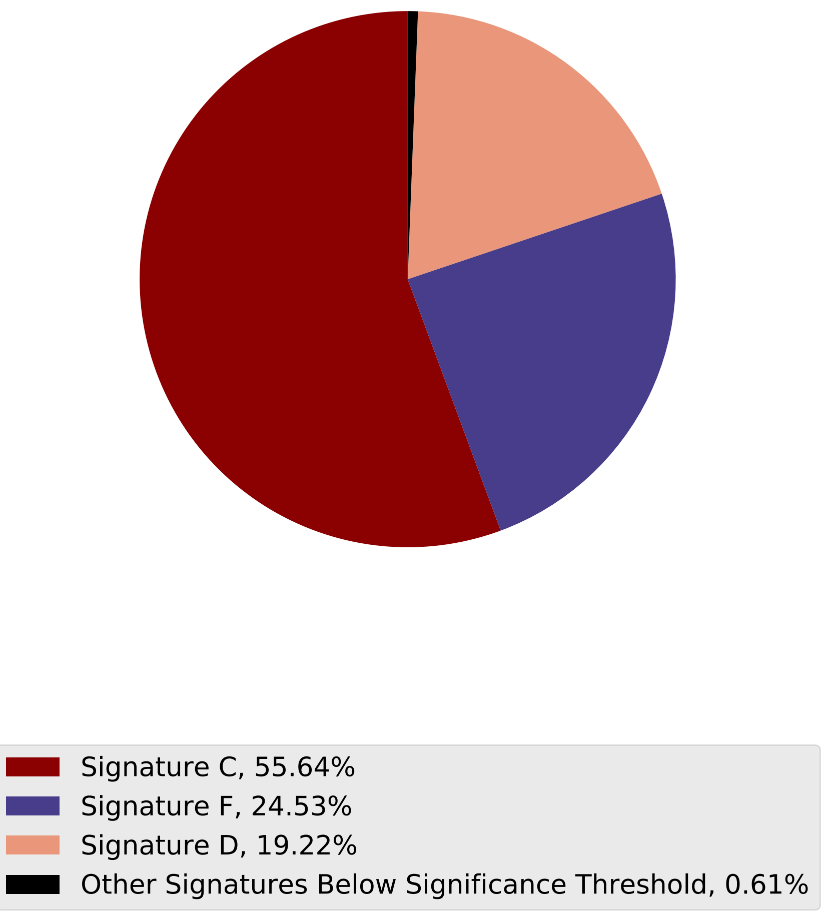

Tumor Profile for TCGA-A2-A0D2

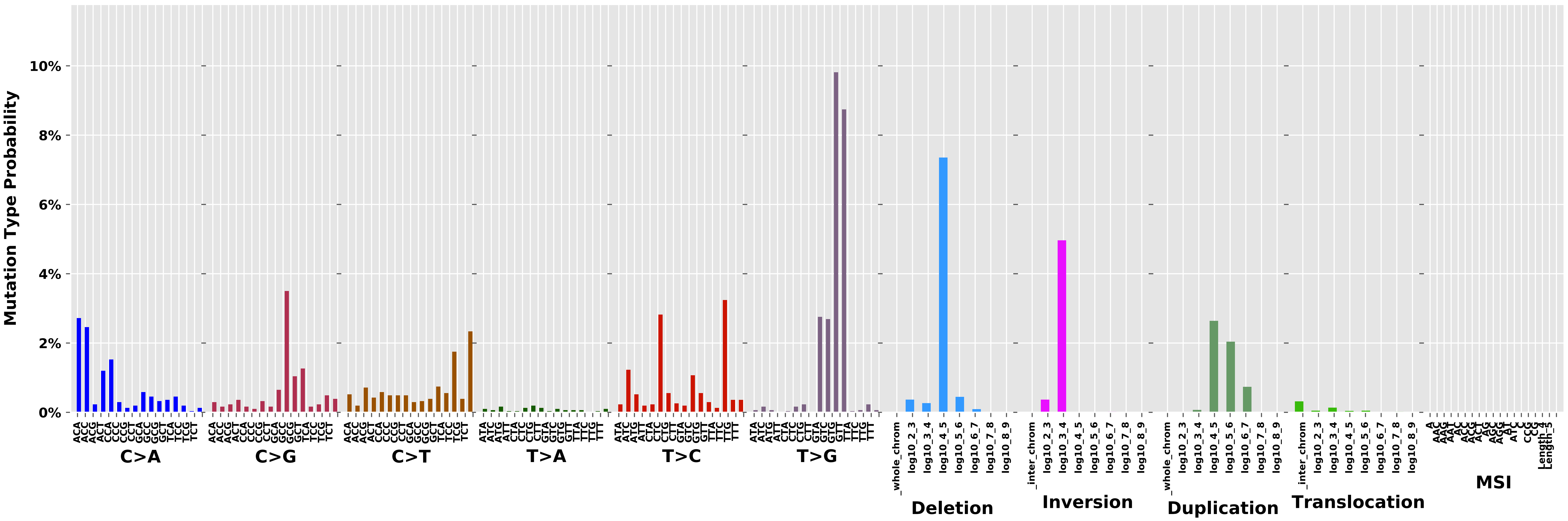

Cancer processes Weights for TCGA-B6-A0RU

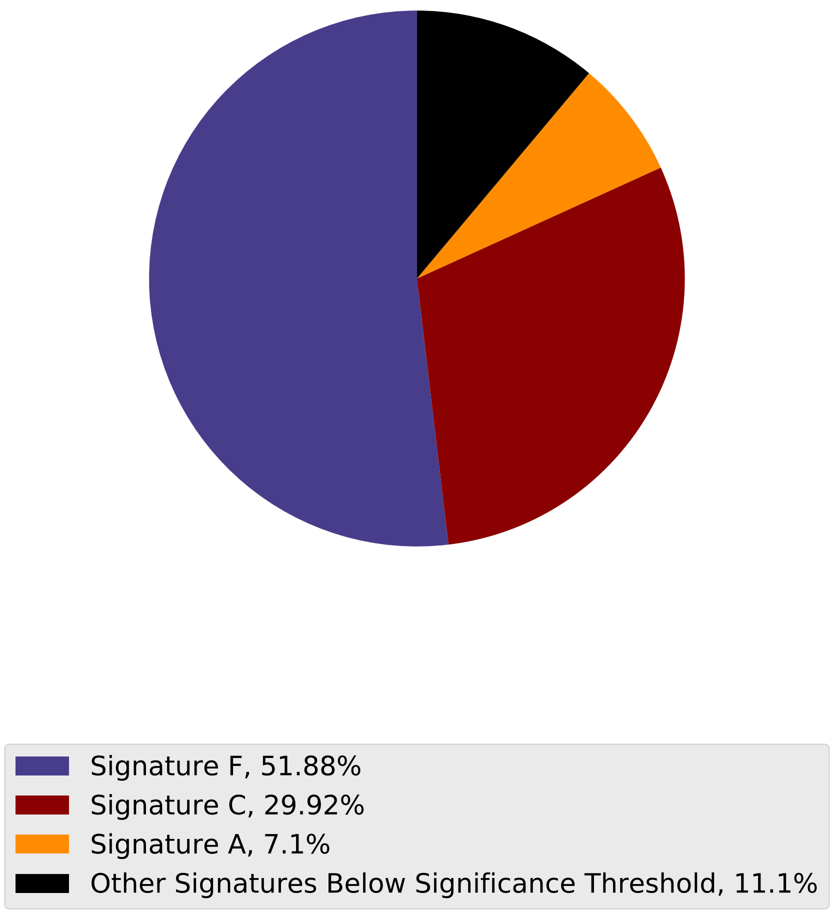

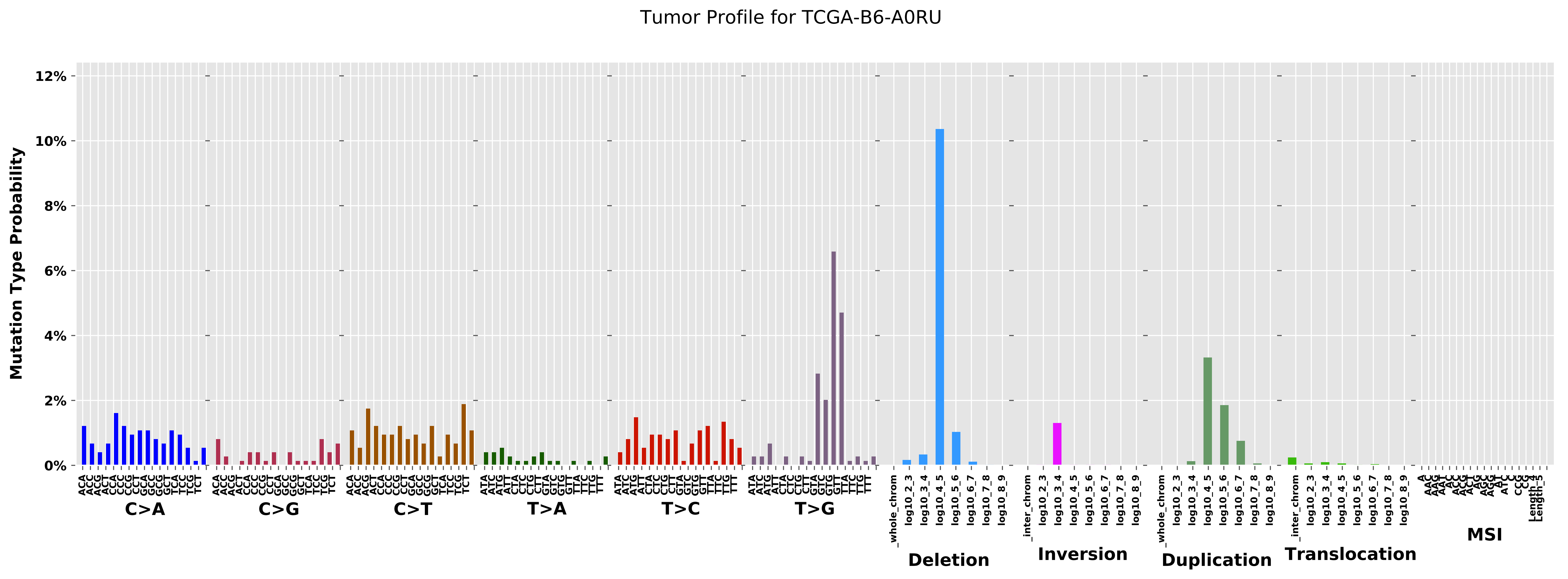
Tumor Profile for TCGA-B6-A0RU
Mutation Type Probability
12%
10%
8%
6%
4%
2%
0%
C>A
C>G
C>T
T>A
T>C
T>G
Deletion
Inversion
Duplication
Translocation
MSI
