# Supplementary material for: pyCancerSig: subclassifying human cancer with comprehensive single nucleotide, structural and microsatellite mutational signature deconstruction from whole genome sequencing
Source: BMC Bioinformatics. 2020 Apr 3;21:128. doi: 10.1186/s12859-020-3451-8 (PMC7118897; doi:10.1186/s12859-020-3451-8)
Supplement: Supplementary file 5 — Additional file 5. [file 12859_2020_3451_MOESM5_ESM.pdf]

Cancer processes Weights for TCGA-E2-A14X

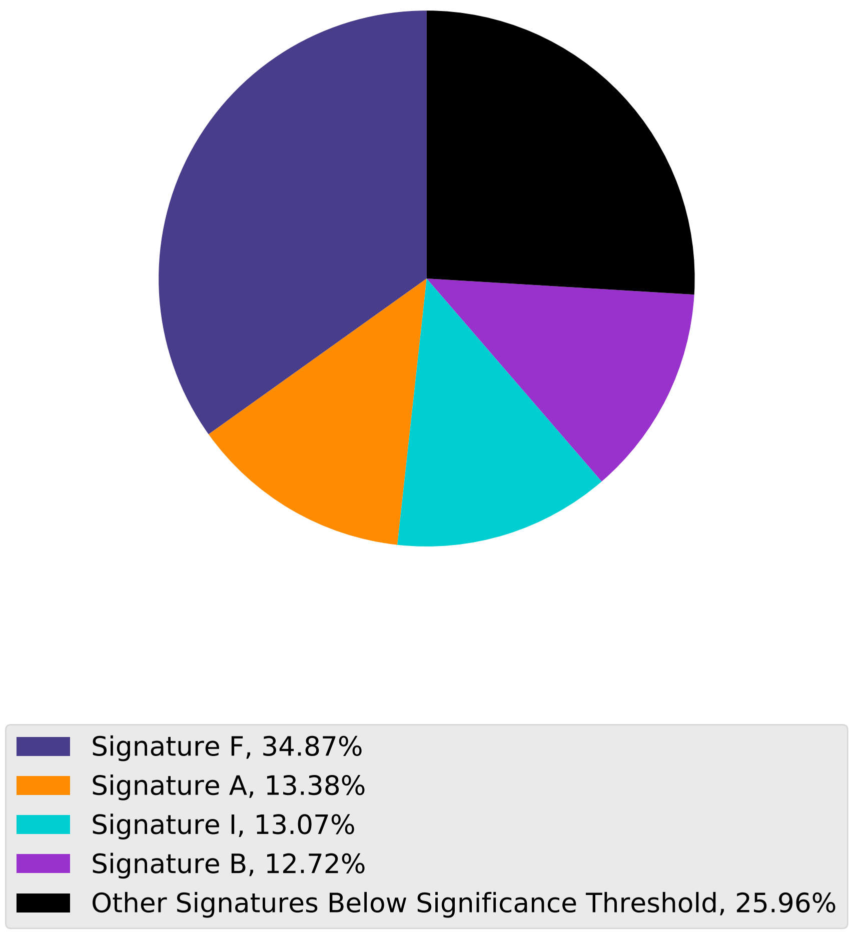

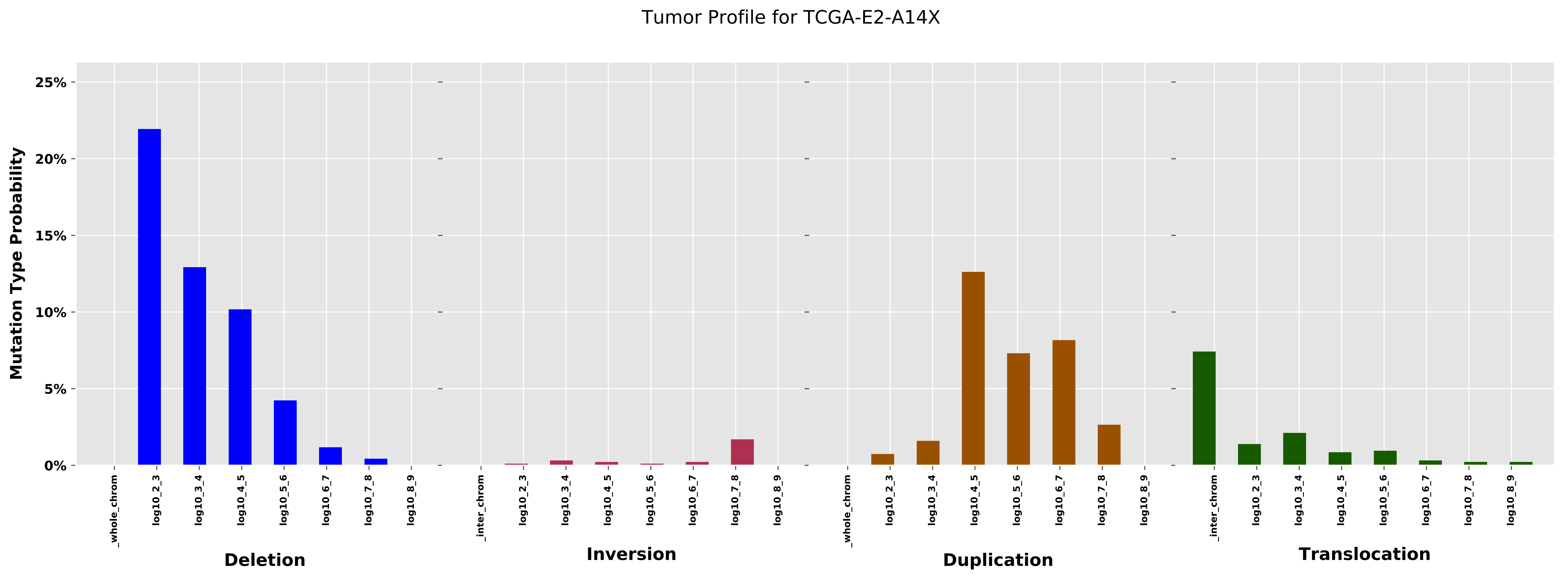

Tumor Profile for TCGA-E2-A14X
Mutation Type Probability
25%
20%
15%
10%
5%
0%
_whole_chrom
log10_2_3
log10_3_4
log10_4_5
log10_5_6
log10_6_7
log10_7_8
log10_8_9
Deletion
_inter_chrom
Inversion
Duplication
Translocation

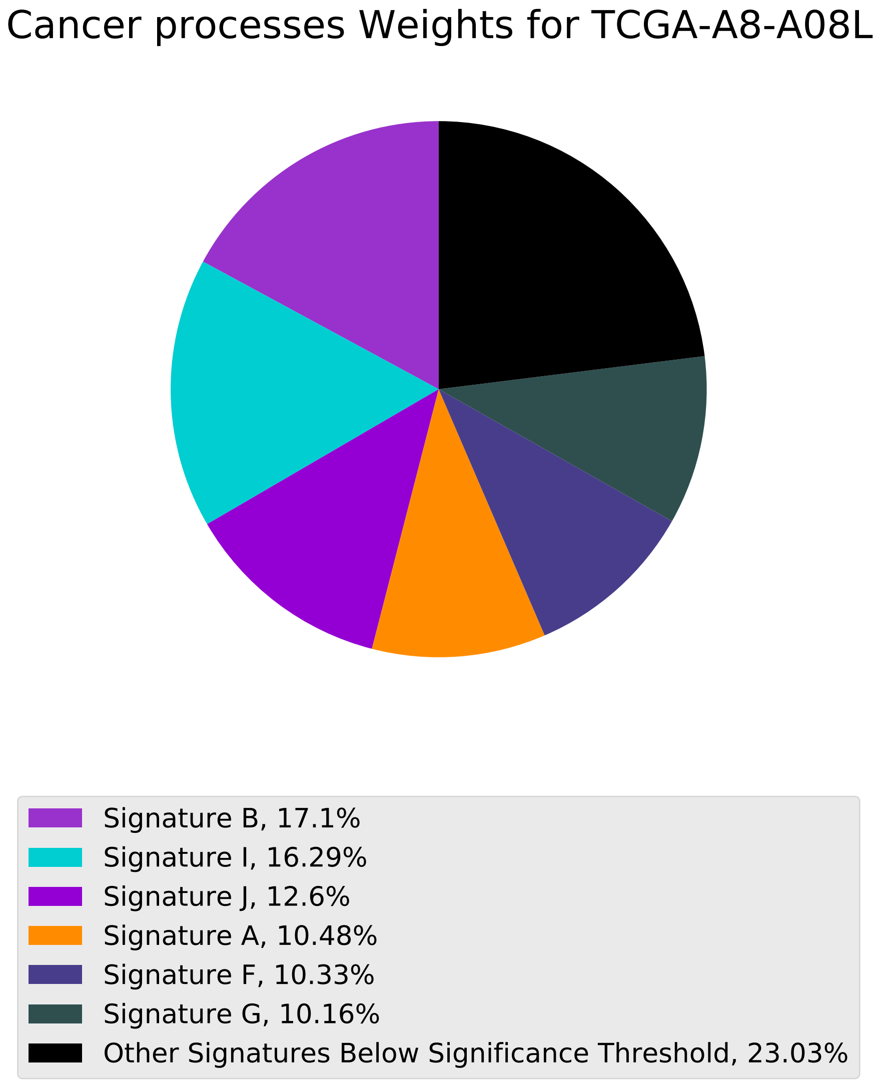
Cancer processes Weights for TCGA-A8-A08L
Signature B, 17.1%
Signature I, 16.29%
Signature J, 12.6%
Signature A, 10.48%
Signature F, 10.33%
Signature G, 10.16%
Other Signatures Below Significance Threshold, 23.03%

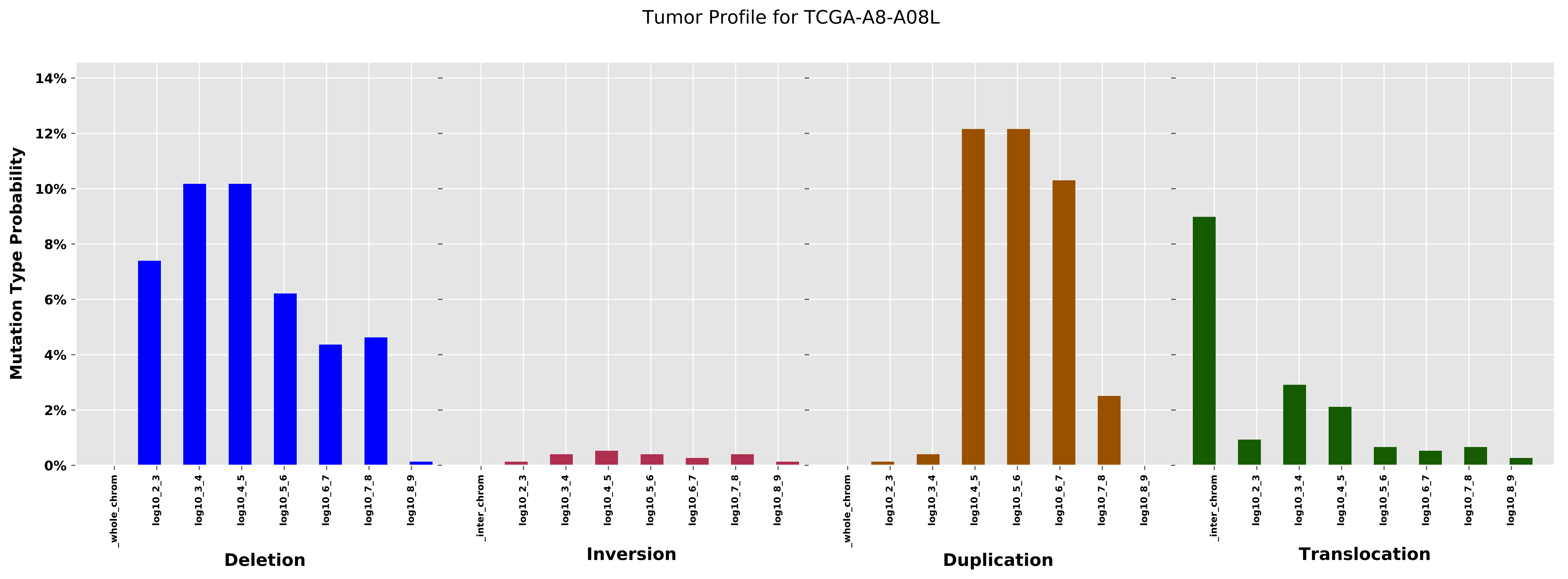

Tumor Profile for TCGA-A8-A08L
Mutation Type Probability
0%
2%
4%
6%
8%
10%
12%
14%
_whole_chrom
log10_2_3
log10_3_4
log10_4_5
log10_5_6
log10_6_7
log10_7_8
log10_8_9
_inter_chrom
Deletion
Inversion
Duplication
Translocation

Cancer processes Weights for TCGA-AO-A12H

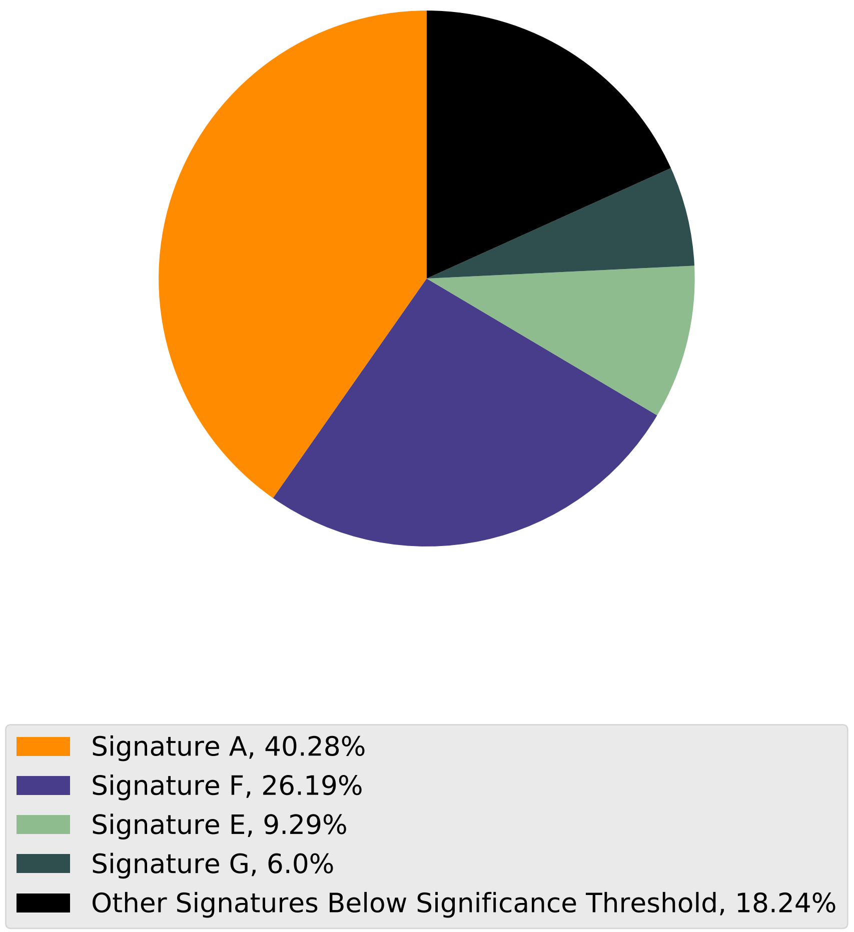

Tumor Profile for TCGA-AO-A12H

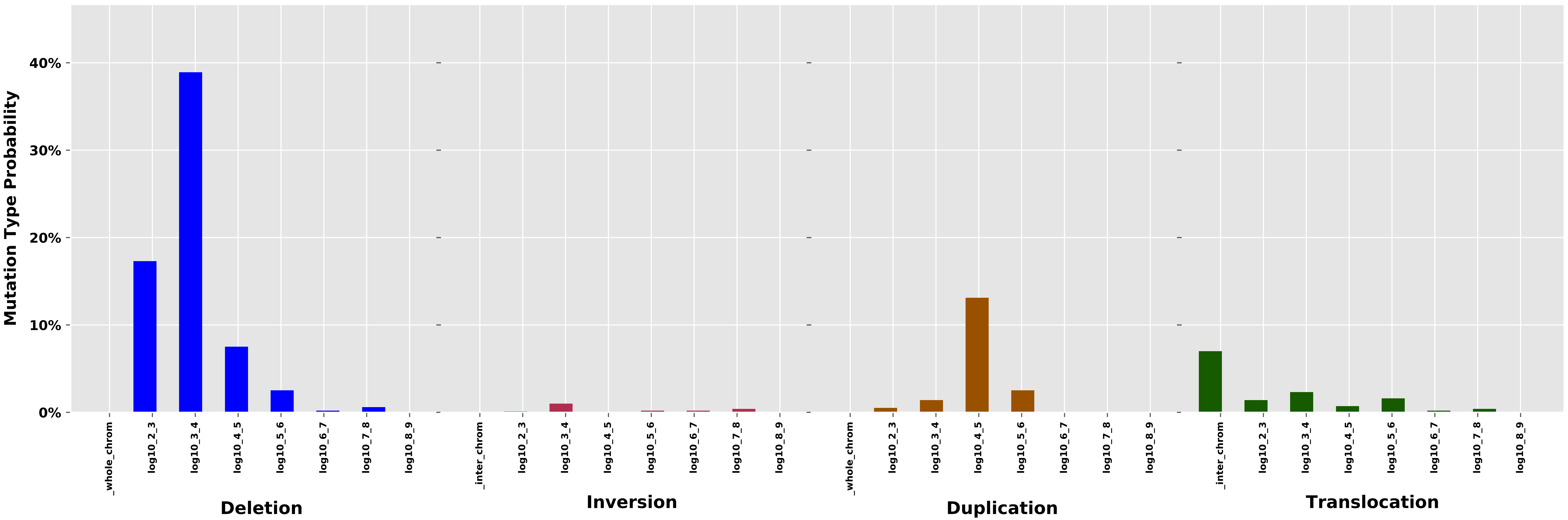

Cancer processes Weights for TCGA-EW-A3U0

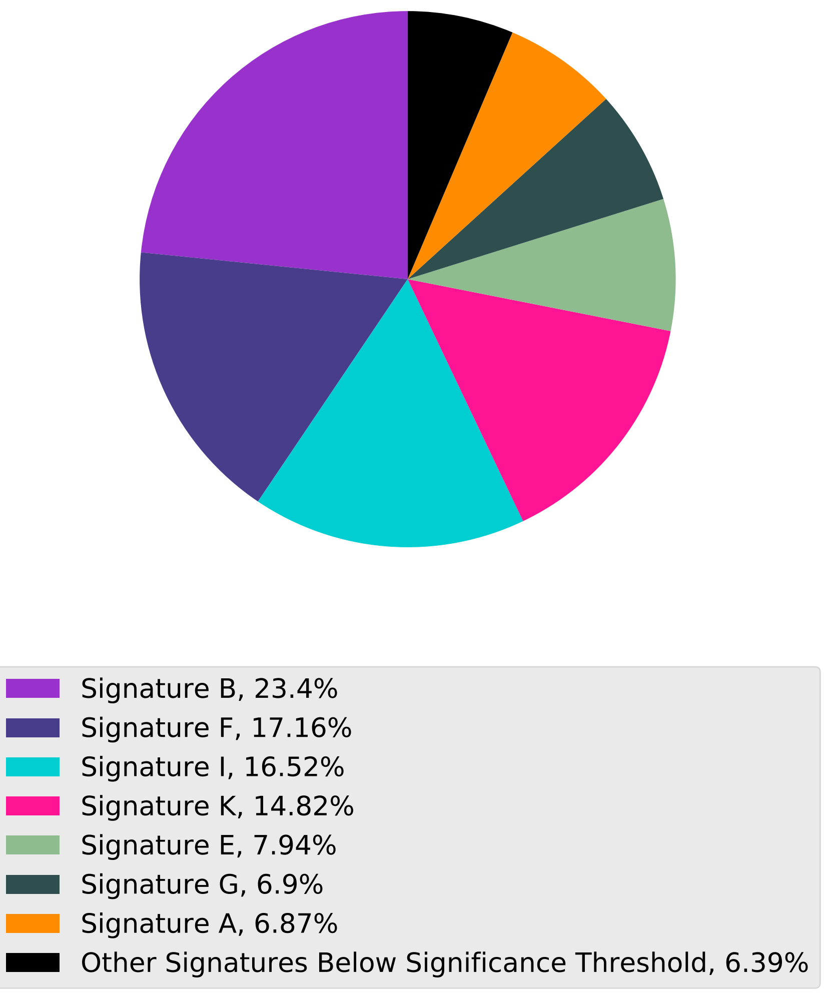

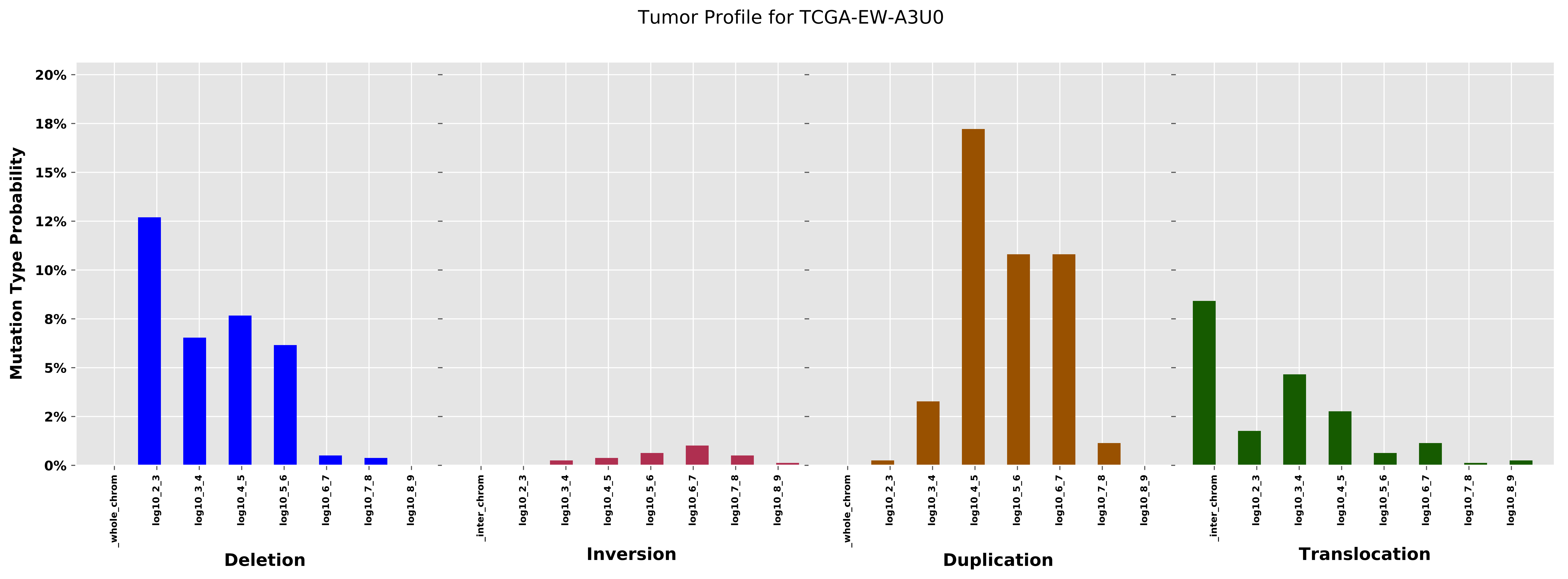

Tumor Profile for TCGA-EW-A3U0
Mutation Type Probability
20%
18%
15%
12%
10%
8%
5%
2%
0%
_whole_chrom
log10_2_3
log10_3_4
log10_4_5
log10_5_6
log10_6_7
log10_7_8
log10_8_9
_inter_chrom
Deletion
Inversion
Duplication
Translocation

Cancer processes Weights for TCGA-BH-A1FC

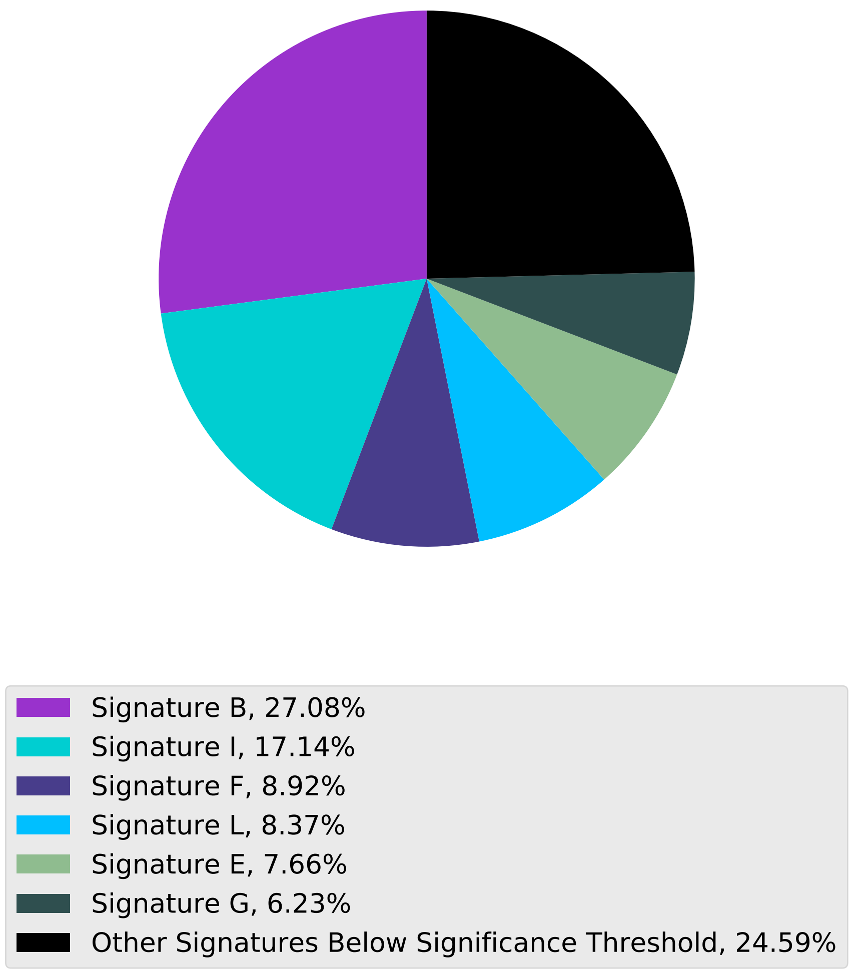

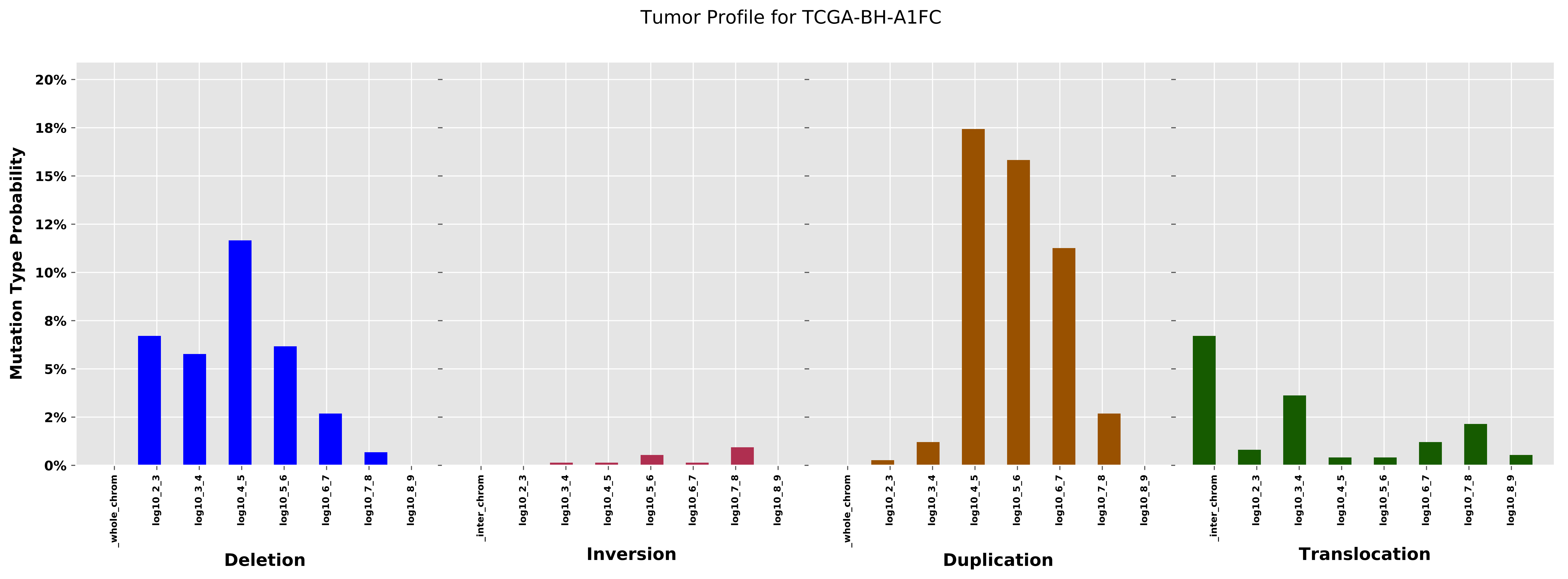
Tumor Profile for TCGA-BH-A1FC
Mutation Type Probability
20%
18%
15%
12%
10%
8%
5%
2%
0%
_whole_chrom
log10_2_3
log10_3_4
log10_4_5
log10_5_6
log10_6_7
log10_7_8
log10_8_9
_inter_chrom
Deletion
Inversion
Duplication
Translocation

Cancer processes Weights for TCGA-AF-2691

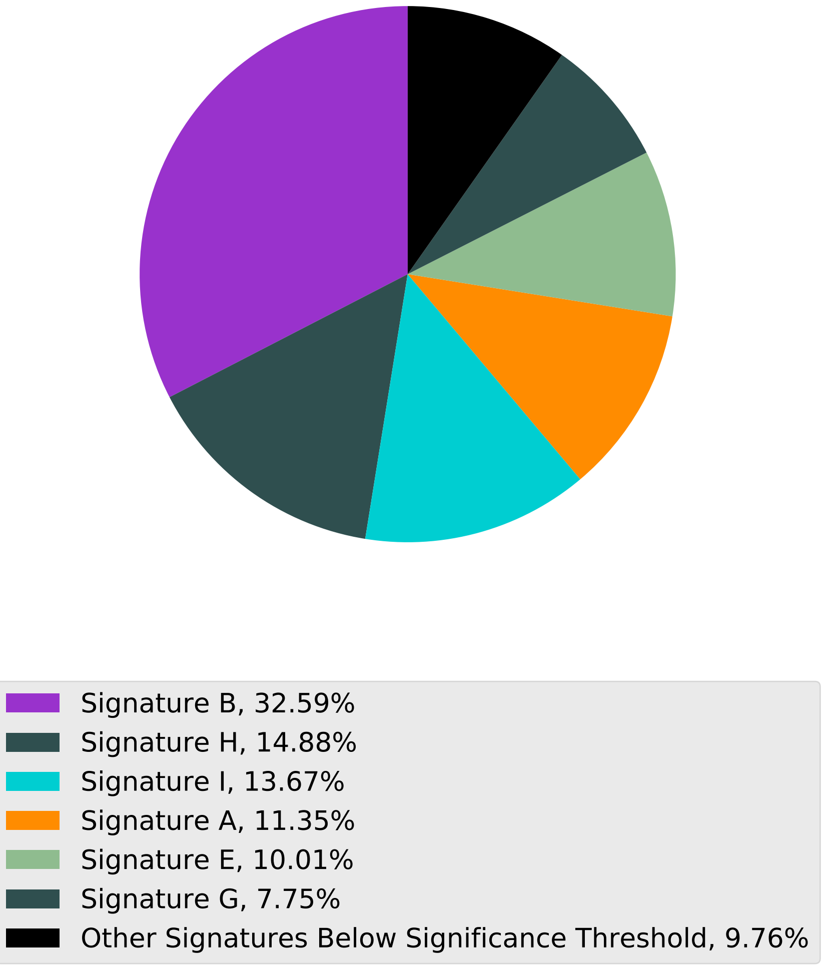

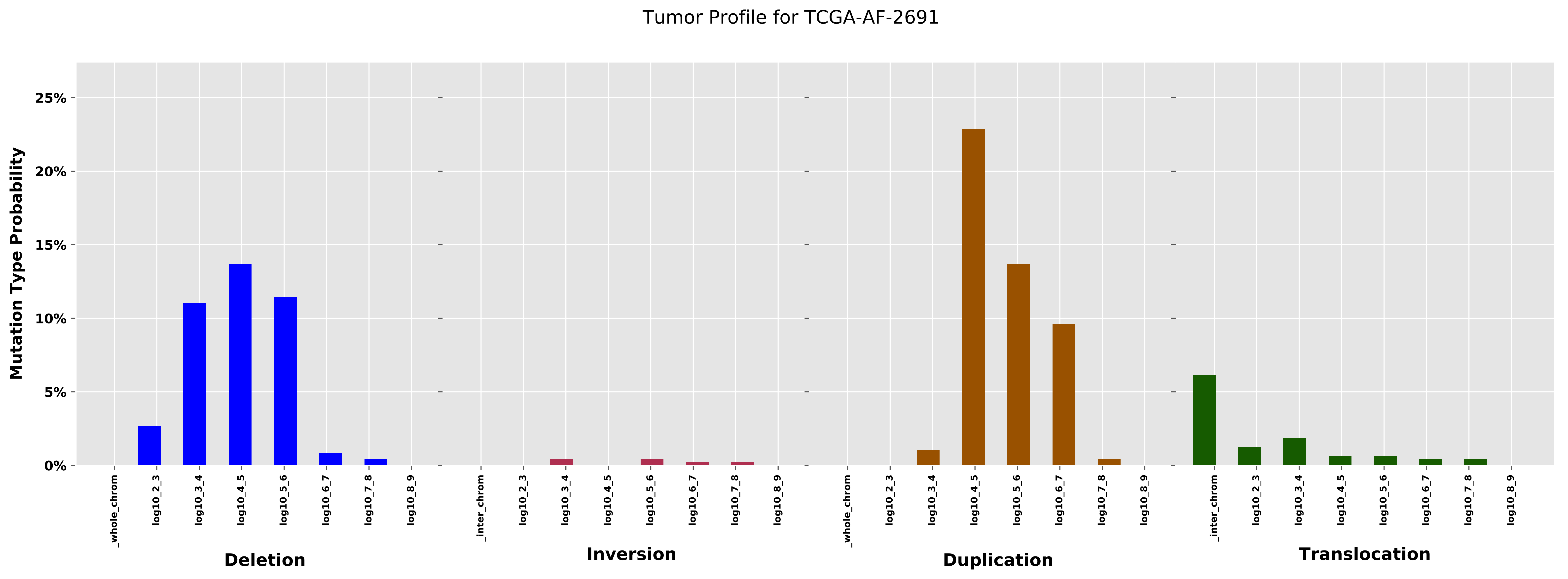

Tumor Profile for TCGA-AF-2691
Mutation Type Probability
25%
20%
15%
10%
5%
0%
_whole_chrom
log10_2_3
log10_3_4
log10_4_5
log10_5_6
log10_6_7
log10_7_8
log10_8_9
Deletion
_inter_chrom
Inversion
Duplication
Translocation

Cancer processes Weights for TCGA-AF-3913

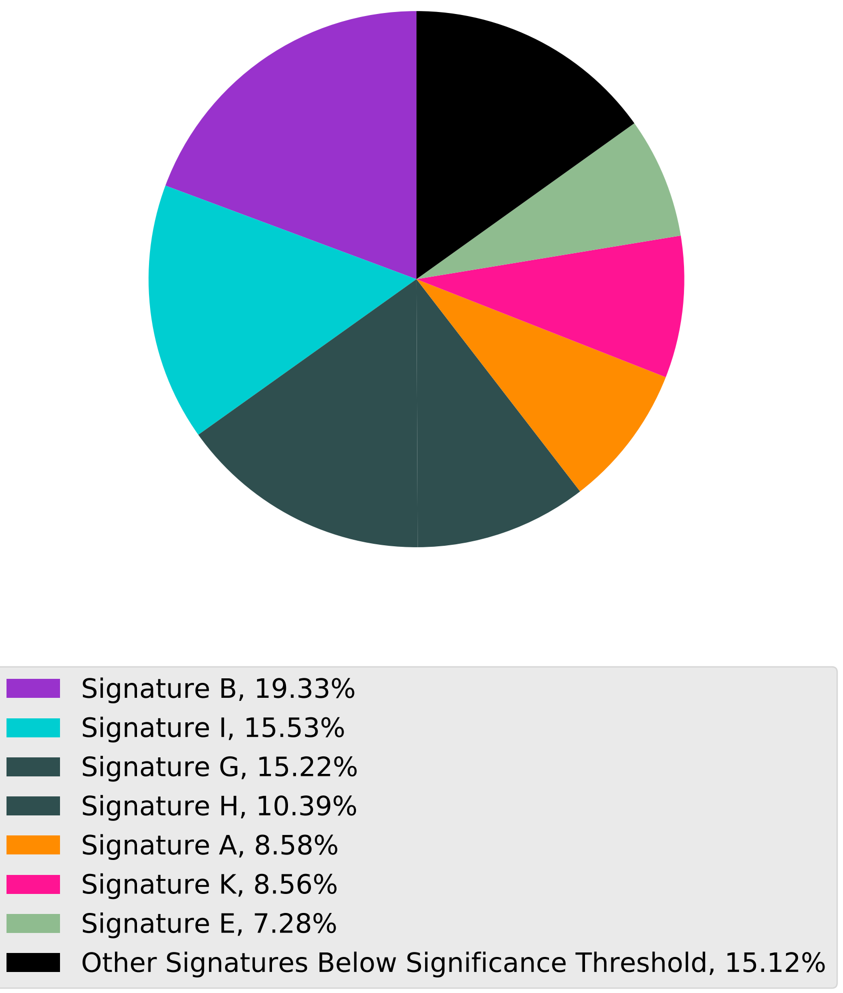

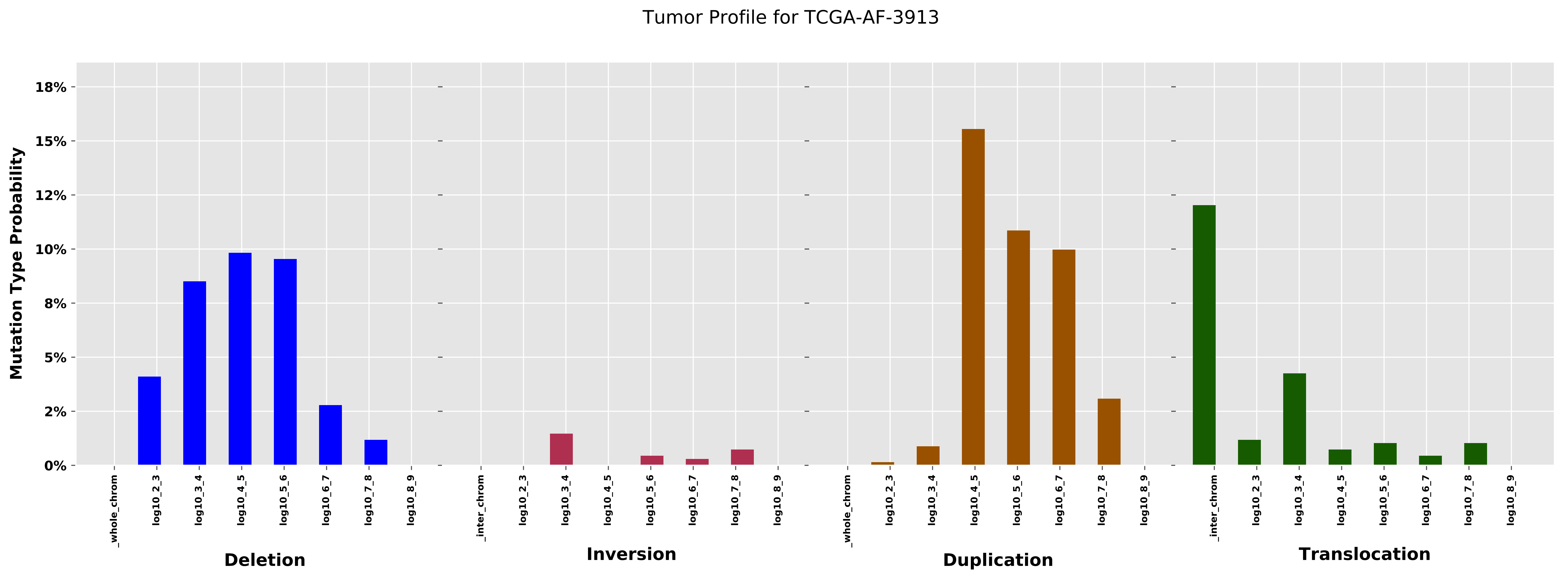

Tumor Profile for TCGA-AF-3913
Mutation Type Probability
18%
15%
12%
10%
8%
5%
2%
0%
_whole_chrom
log10_2_3
log10_3_4
log10_4_5
log10_5_6
log10_6_7
log10_7_8
log10_8_9
_inter_chrom
Deletion
Inversion
Duplication
Translocation

Cancer processes Weights for TCGA-AA-3977

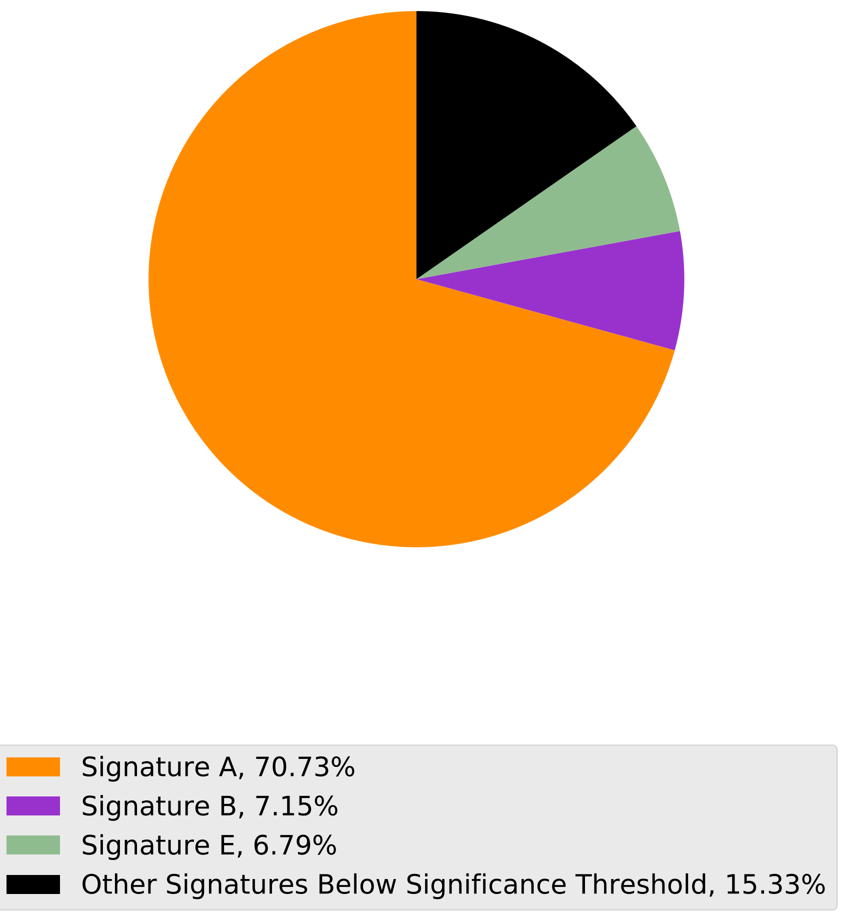

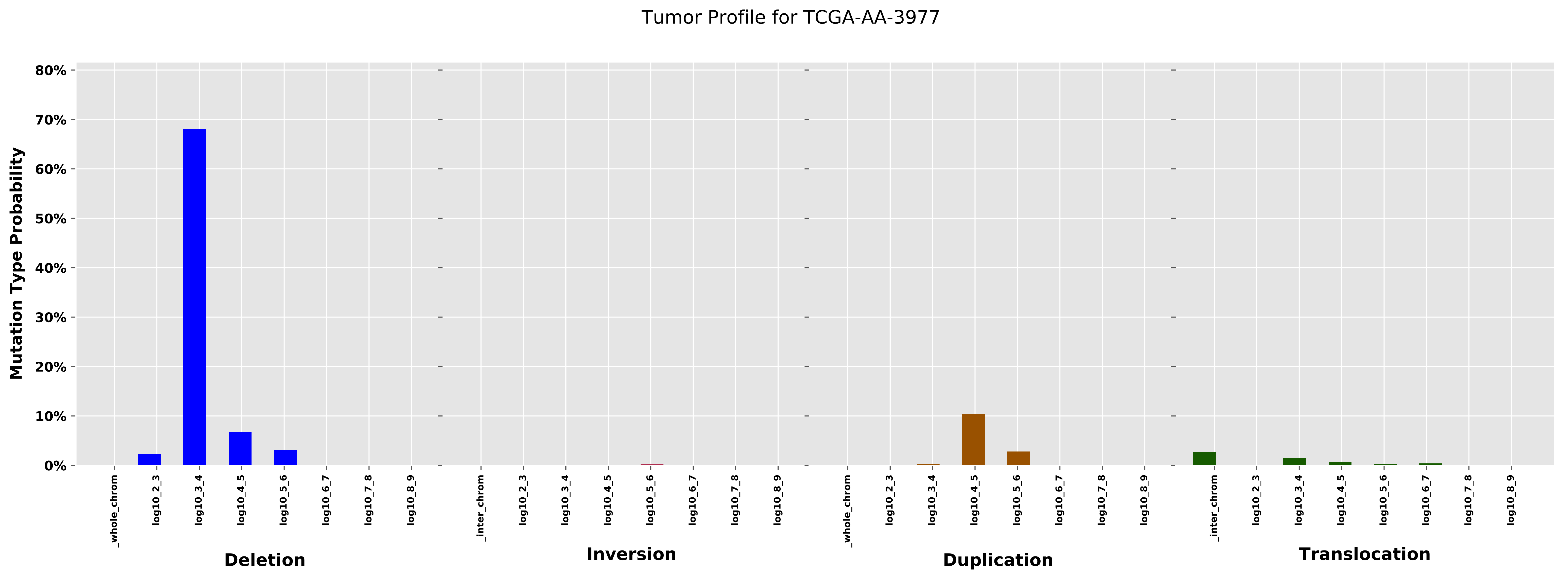

Tumor Profile for TCGA-AA-3977
Mutation Type Probability
0%
10%
20%
30%
40%
50%
60%
70%
80%
_whole_chrom
log10_2_3
log10_3_4
log10_4_5
log10_5_6
log10_6_7
log10_7_8
log10_8_9
_inter_chrom
Deletion
Inversion
Duplication
Translocation

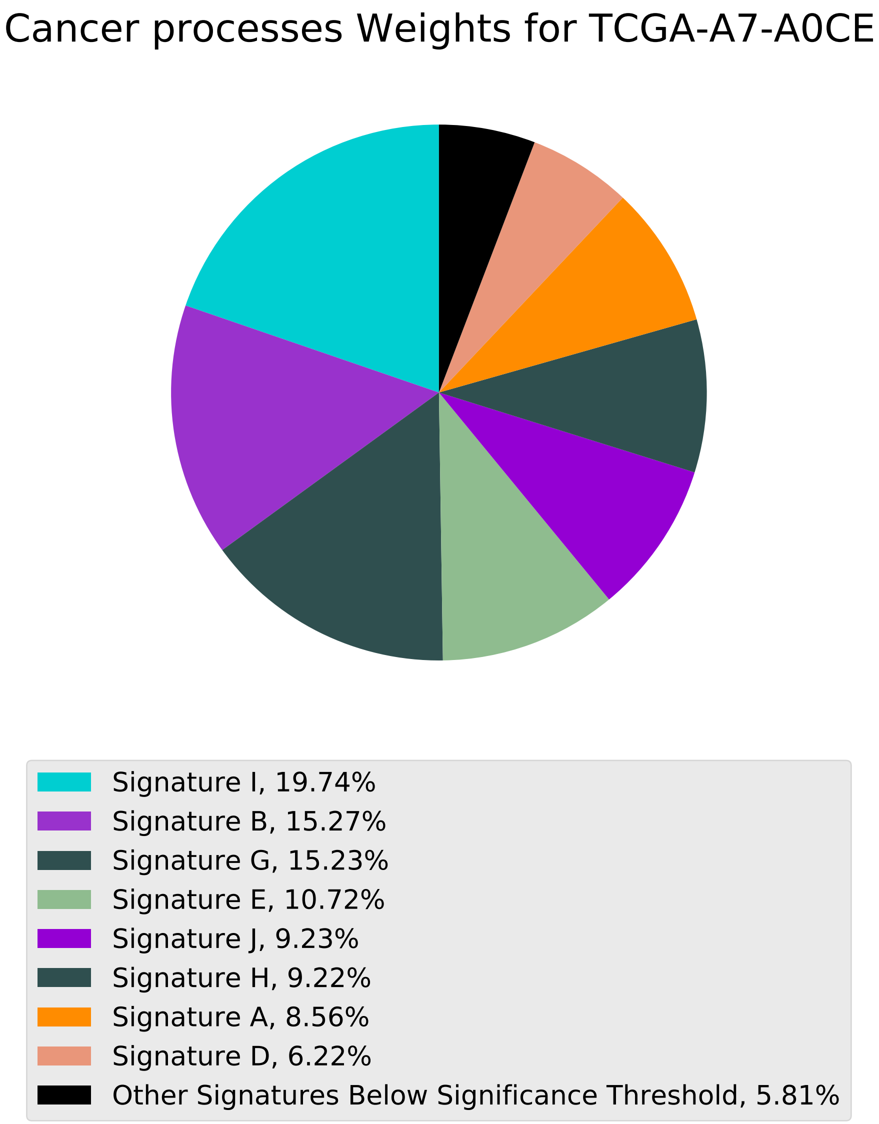
Cancer processes Weights for TCGA-A7-A0CE
Signature I, 19.74%
Signature B, 15.27%
Signature G, 15.23%
Signature E, 10.72%
Signature J, 9.23%
Signature H, 9.22%
Signature A, 8.56%
Signature D, 6.22%
Other Signatures Below Significance Threshold, 5.81%

Tumor Profile for TCGA-A7-A0CE

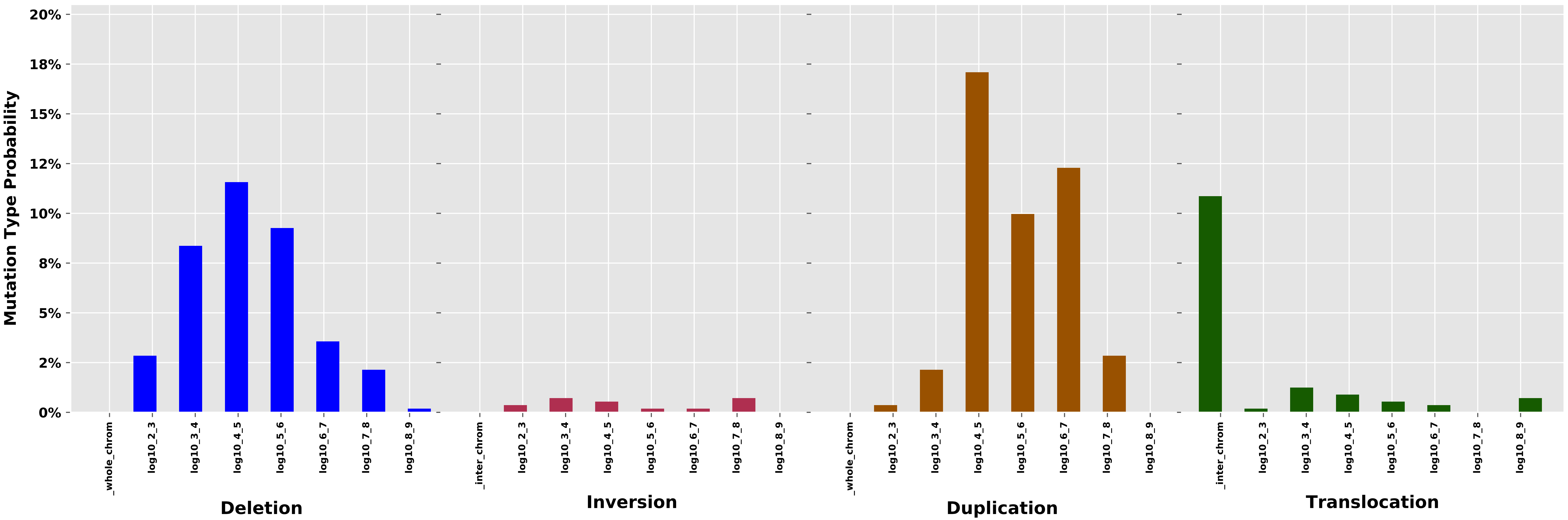

Cancer processes Weights for TCGA-AO-A0J4

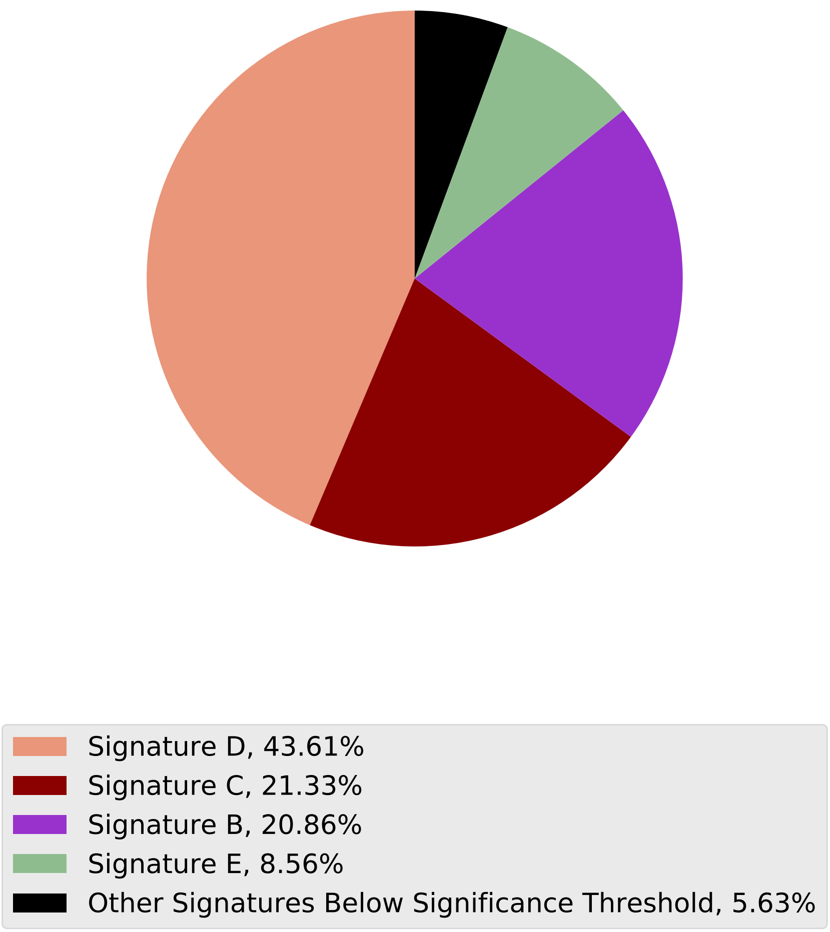

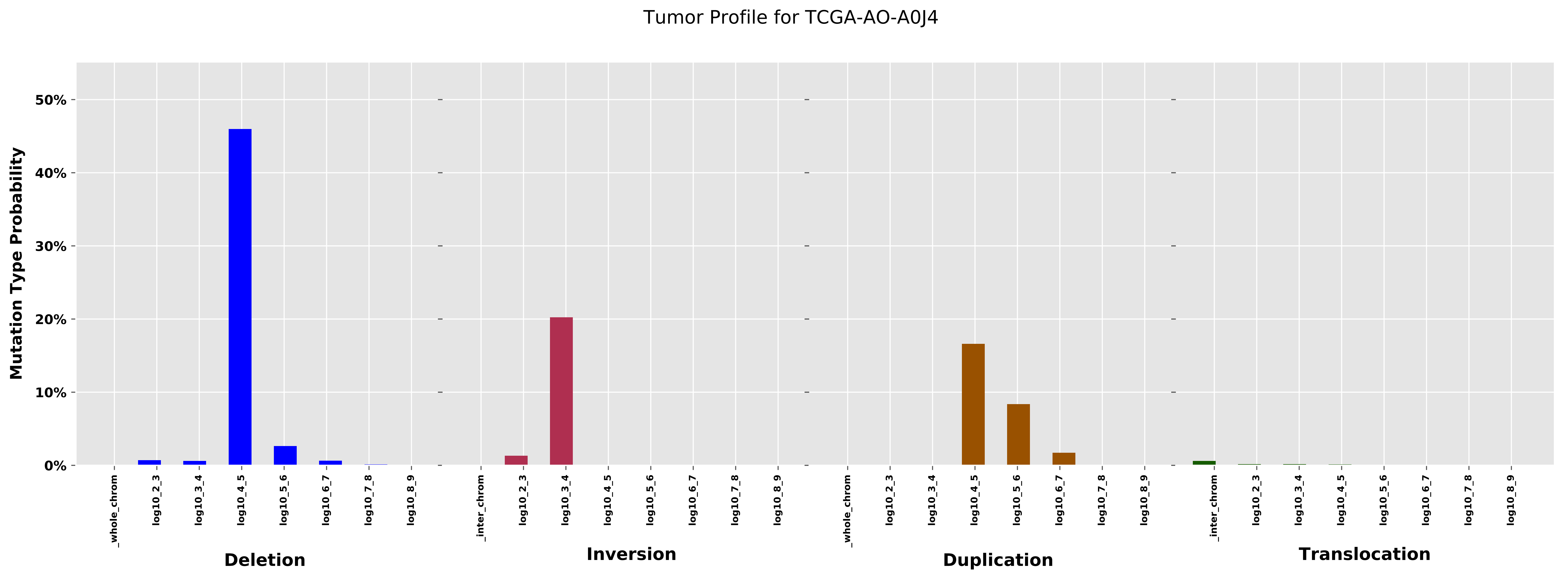
Tumor Profile for TCGA-AO-A0J4
Mutation Type Probability
50%
40%
30%
20%
10%
0%
_whole_chrom
log10_2_3
log10_3_4
log10_4_5
log10_5_6
log10_6_7
log10_7_8
log10_8_9
_inter_chrom
Deletion
Inversion
Duplication
Translocation

Cancer processes Weights for TCGA-AQ-A04J

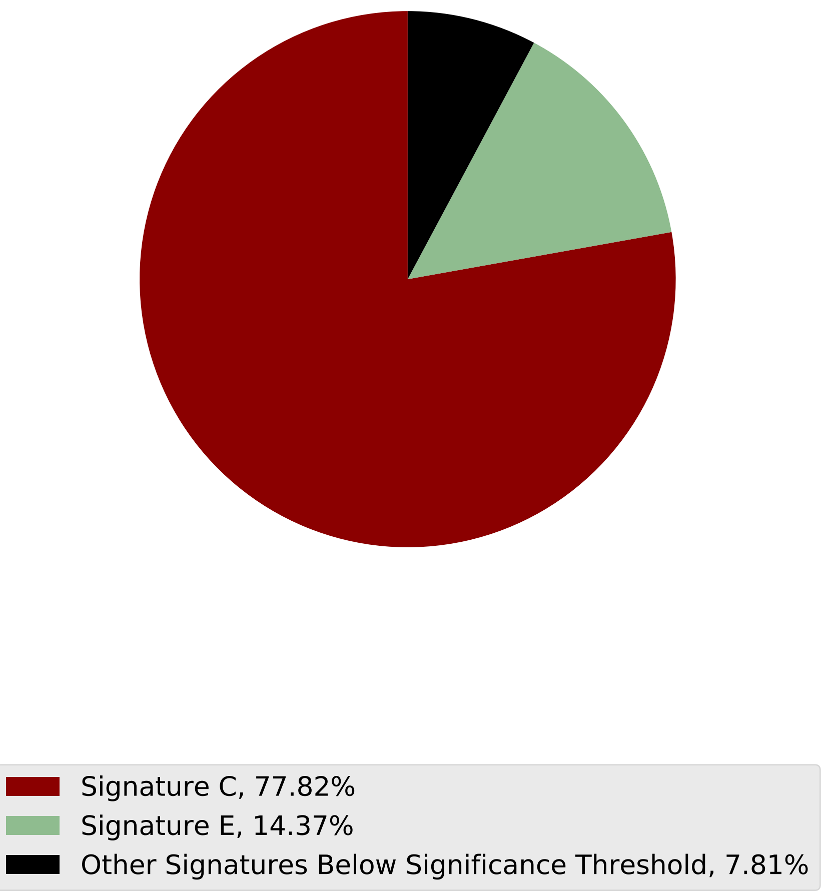

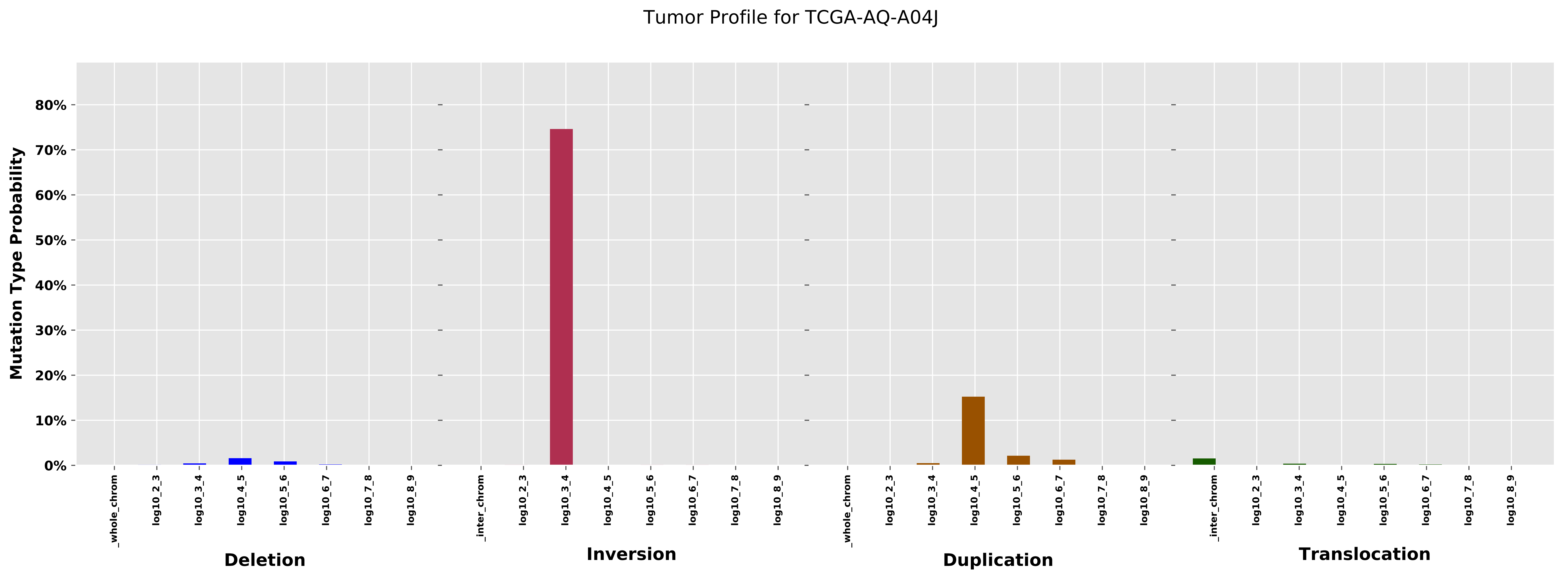

Tumor Profile for TCGA-AQ-A04J
Mutation Type Probability
80%
70%
60%
50%
40%
30%
20%
10%
0%
_whole_chrom
log10_2_3
log10_3_4
log10_4_5
log10_5_6
log10_6_7
log10_7_8
log10_8_9
Deletion
_inter_chrom
log10_2_3
log10_3_4
log10_4_5
log10_5_6
log10_6_7
log10_7_8
log10_8_9
Inversion
_whole_chrom
log10_2_3
log10_3_4
log10_4_5
log10_5_6
log10_6_7
log10_7_8
log10_8_9
Duplication
_inter_chrom
log10_2_3
log10_3_4
log10_4_5
log10_5_6
log10_6_7
log10_7_8
log10_8_9
Translocation

Cancer processes Weights for TCGA-AG-4007

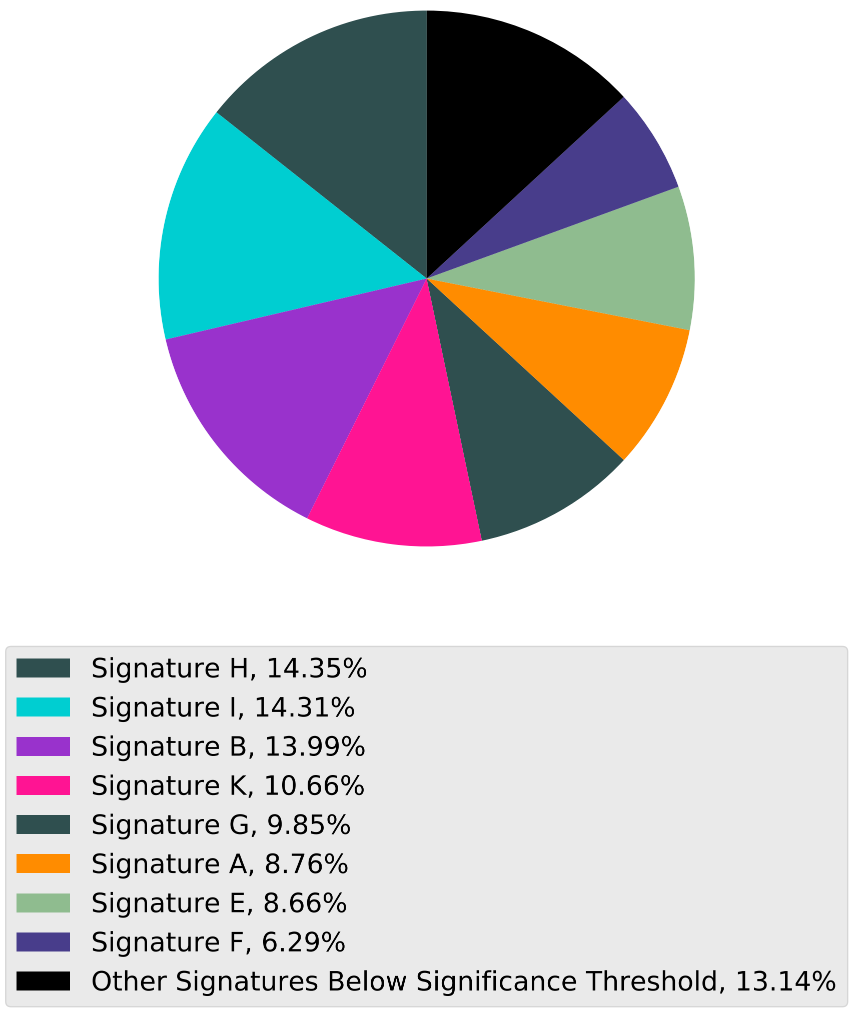

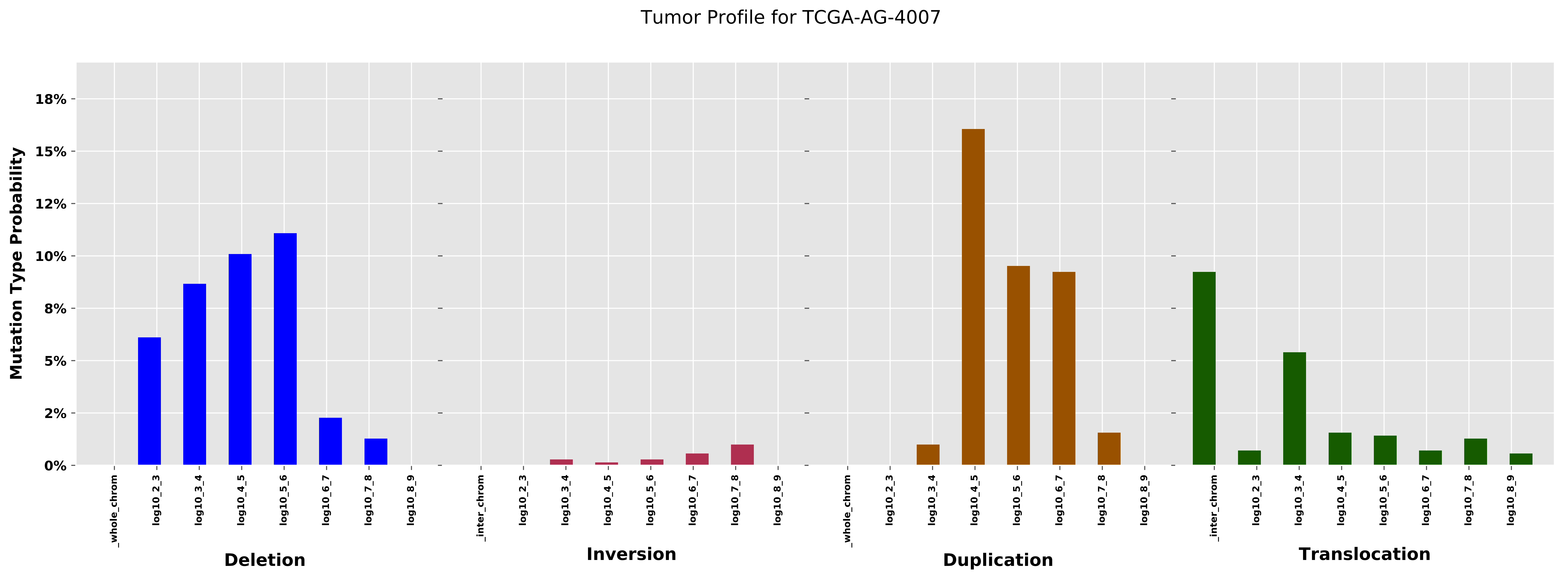

Tumor Profile for TCGA-AG-4007
Mutation Type Probability
18%
15%
12%
10%
8%
5%
2%
0%
_whole_chrom
log10_2_3
log10_3_4
log10_4_5
log10_5_6
log10_6_7
log10_7_8
log10_8_9
_inter_chrom
Deletion
Inversion
Duplication
Translocation

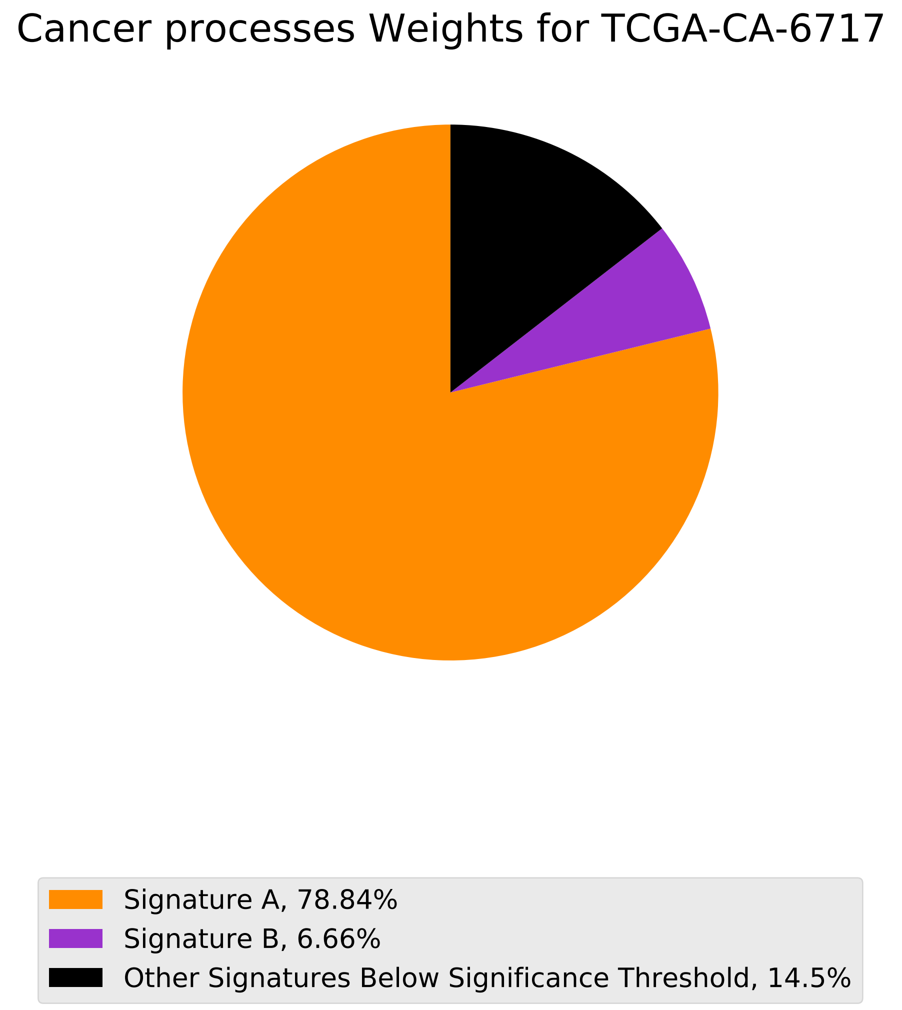
Cancer processes Weights for TCGA-CA-6717
Signature A, 78.84%
Signature B, 6.66%
Other Signatures Below Significance Threshold, 14.5%

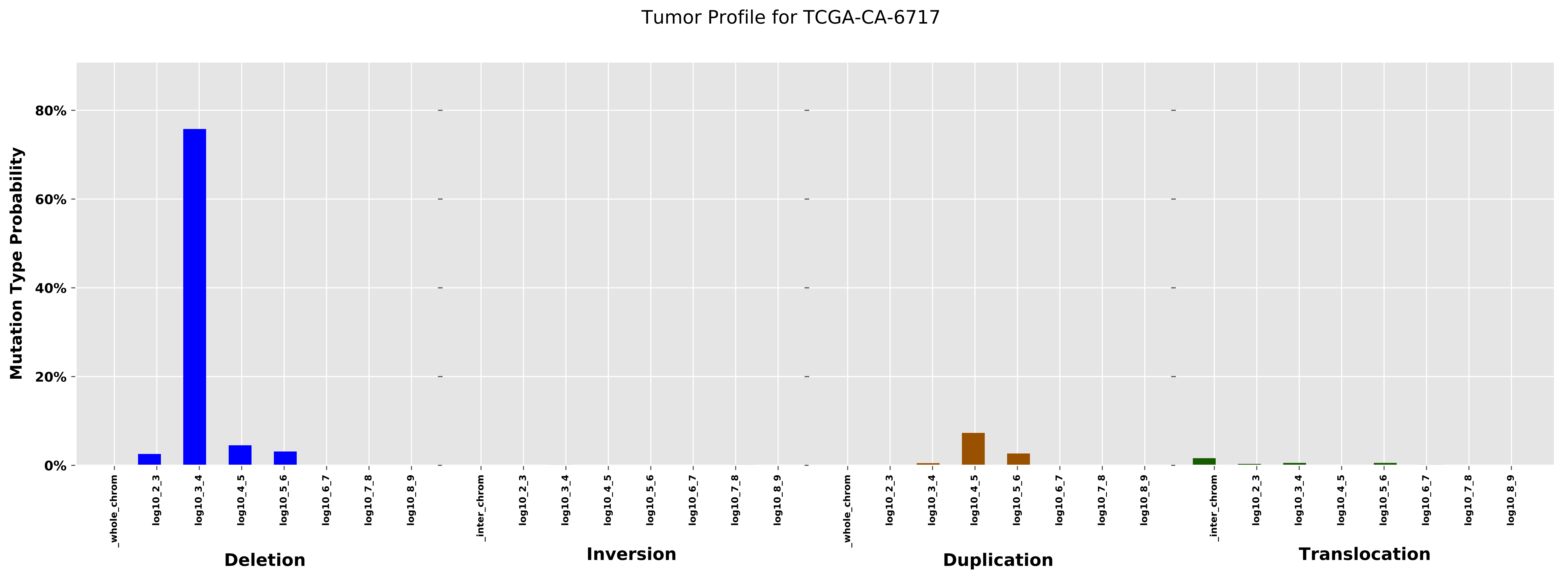

Tumor Profile for TCGA-CA-6717
Mutation Type Probability
80%
60%
40%
20%
0%
_whole_chrom
log10_2_3
log10_3_4
log10_4_5
log10_5_6
log10_6_7
log10_7_8
log10_8_9
_inter_chrom
Deletion
Inversion
Duplication
Translocation

Cancer processes Weights for TCGA-E2-A14P

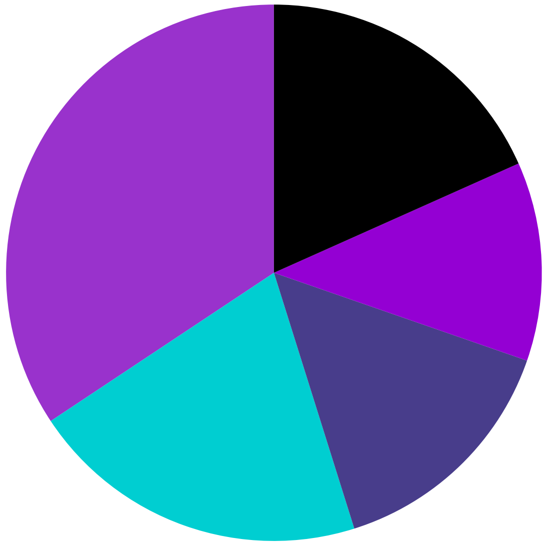

Signature B, 34.32%
Signature I, 20.53%
Signature F, 14.84%
Signature J, 11.99%
Other Signatures Below Significance Threshold, 18.32%

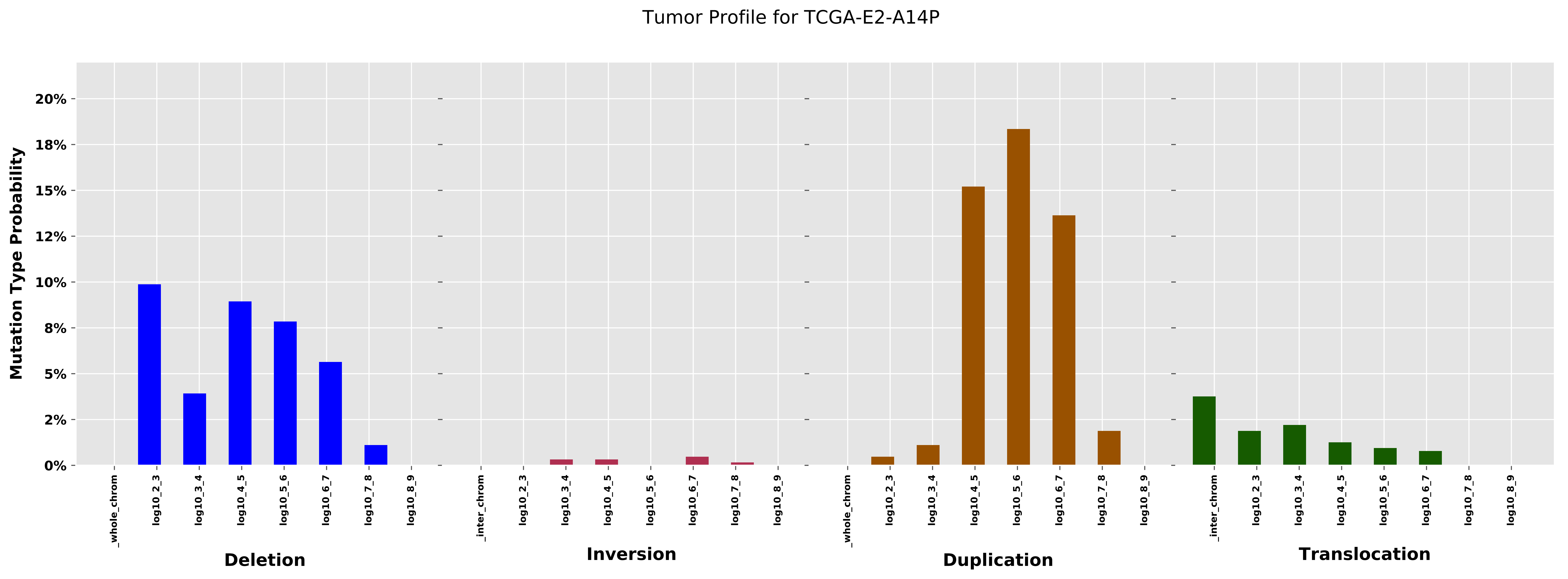

Tumor Profile for TCGA-E2-A14P
Mutation Type Probability
20%
18%
15%
12%
10%
8%
5%
2%
0%
_whole_chrom
log10_2_3
log10_3_4
log10_4_5
log10_5_6
log10_6_7
log10_7_8
log10_8_9
_inter_chrom
Deletion
Inversion
Duplication
Translocation

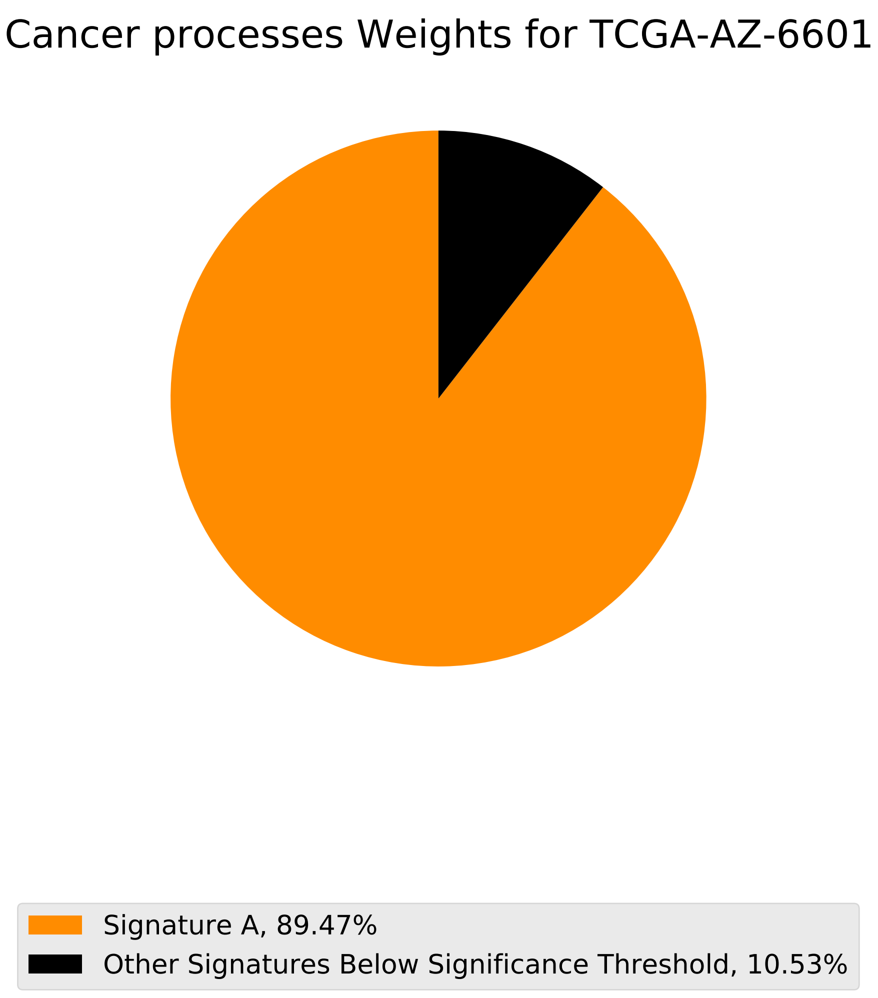
Cancer processes Weights for TCGA-AZ-6601
Signature A, 89.47%
Other Signatures Below Significance Threshold, 10.53%

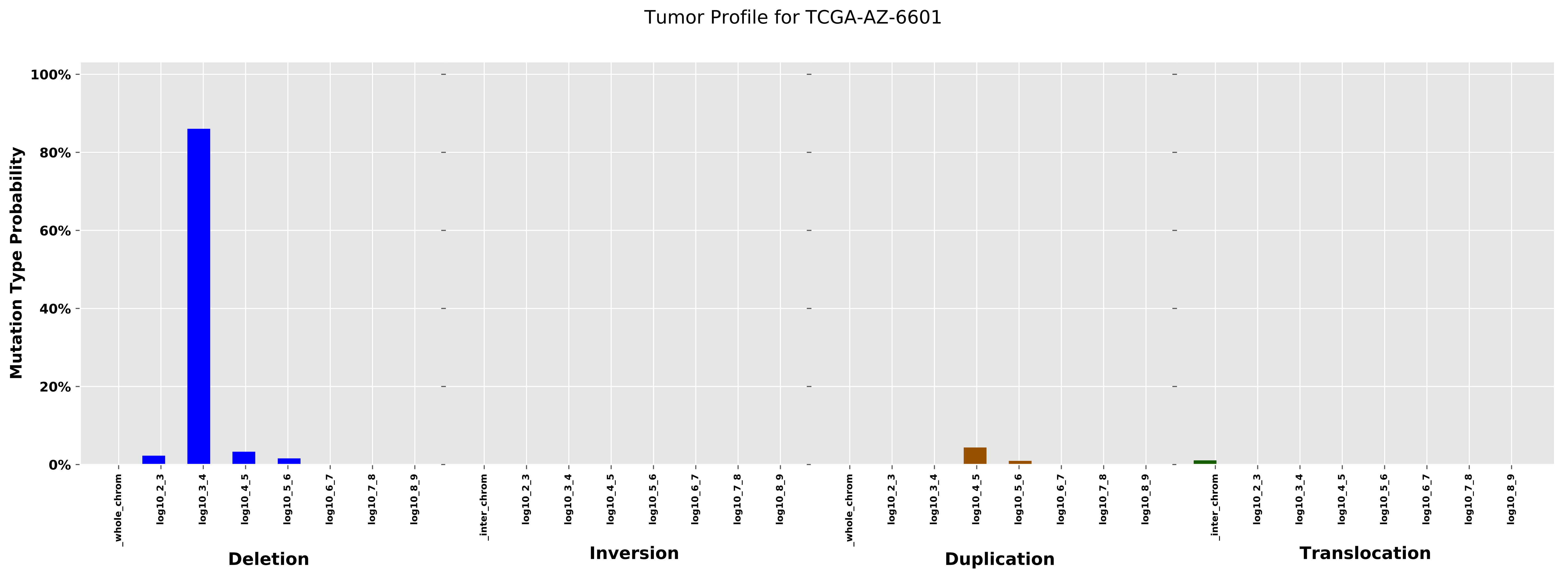

Tumor Profile for TCGA-AZ-6601
Mutation Type Probability
100%
80%
60%
40%
20%
0%
_whole_chrom
log10_2_3
log10_3_4
log10_4_5
log10_5_6
log10_6_7
log10_7_8
log10_8_9
Deletion
_inter_chrom
log10_2_3
log10_3_4
log10_4_5
log10_5_6
log10_6_7
log10_7_8
log10_8_9
Inversion
_whole_chrom
log10_2_3
log10_3_4
log10_4_5
log10_5_6
log10_6_7
log10_7_8
log10_8_9
Duplication
_inter_chrom
log10_2_3
log10_3_4
log10_4_5
log10_5_6
log10_6_7
log10_7_8
log10_8_9
Translocation

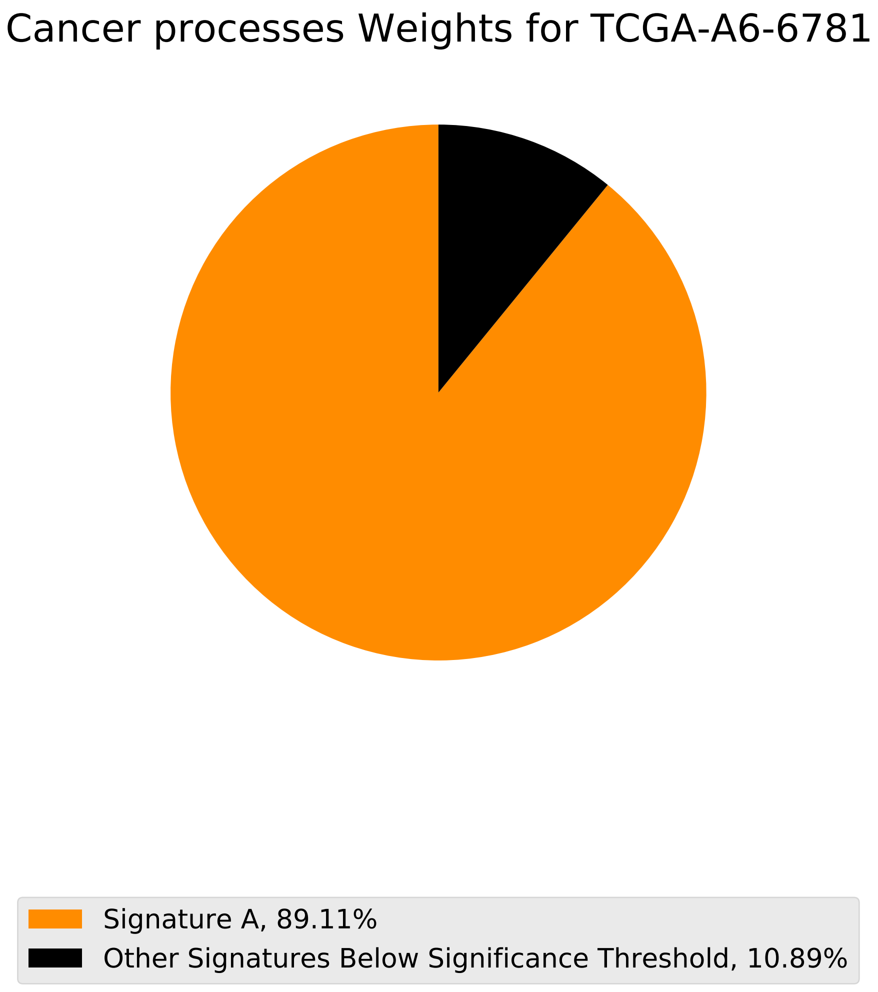
Cancer processes Weights for TCGA-A6-6781
Signature A, 89.11%
Other Signatures Below Significance Threshold, 10.89%

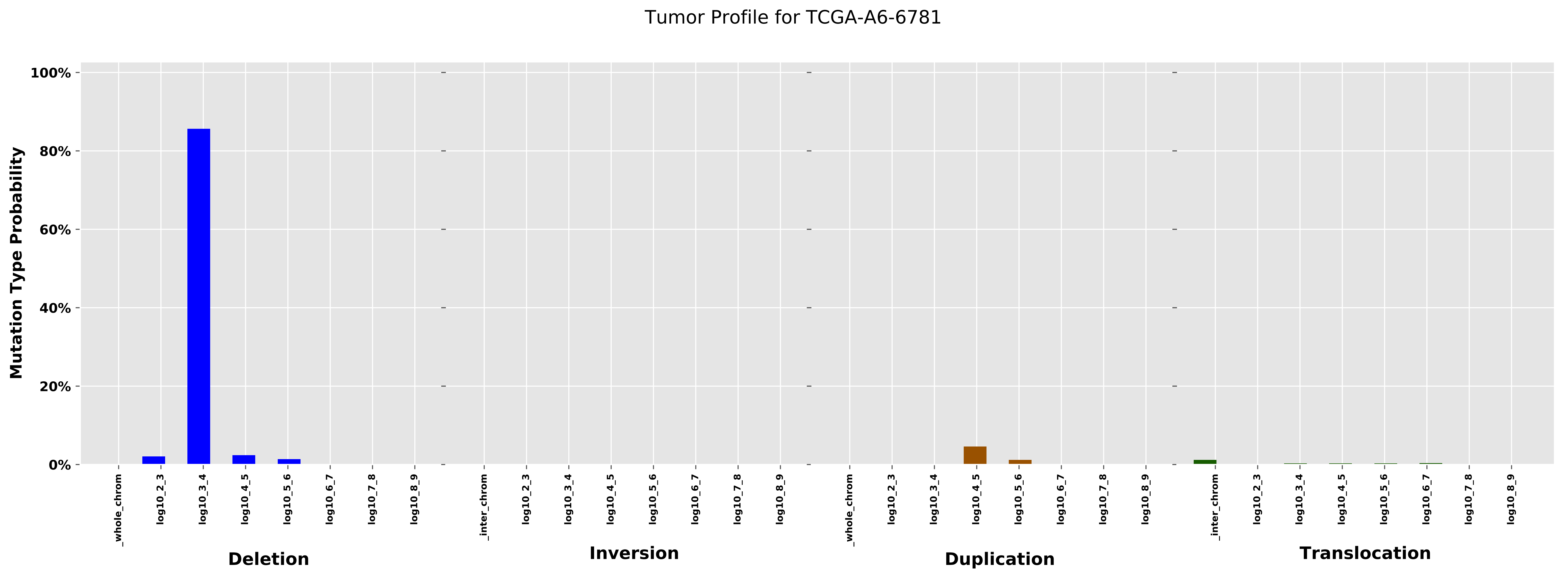

Tumor Profile for TCGA-A6-6781
Mutation Type Probability
100%
80%
60%
40%
20%
0%
_whole_chrom
log10_2_3
log10_3_4
log10_4_5
log10_5_6
log10_6_7
log10_7_8
log10_8_9
_inter_chrom
Deletion
Inversion
Duplication
Translocation

Cancer processes Weights for TCGA-GM-A2DF

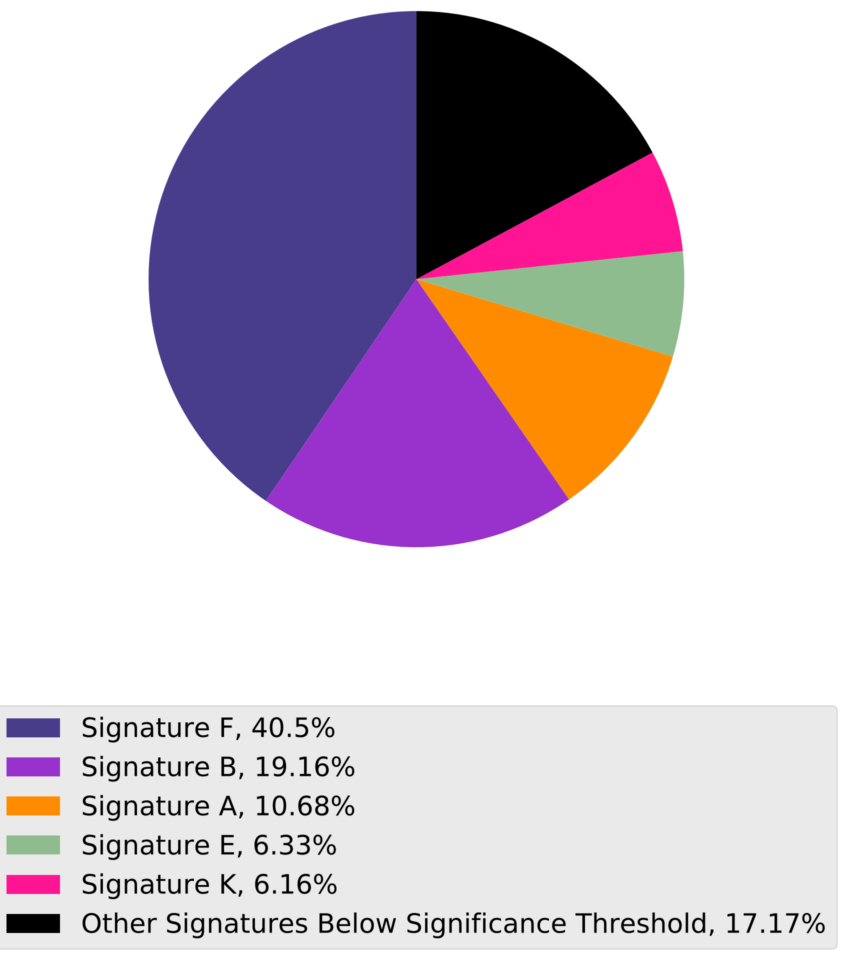

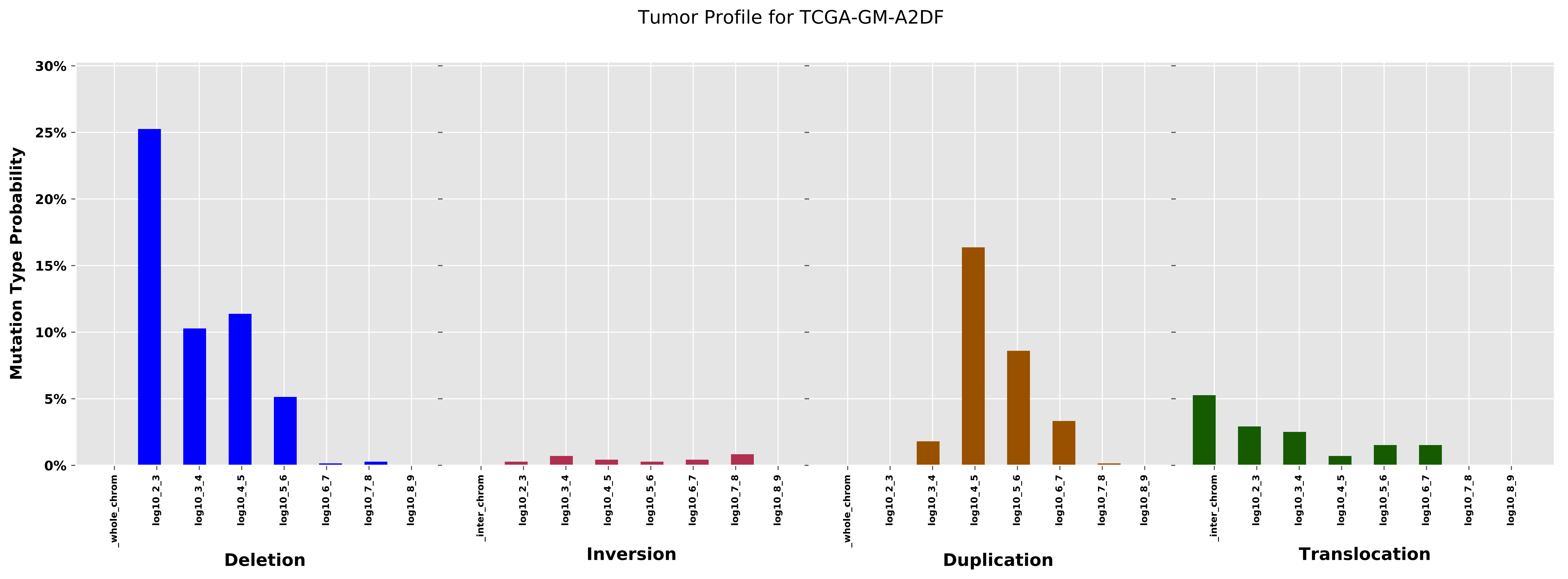

Tumor Profile for TCGA-GM-A2DF
Mutation Type Probability
30%
25%
20%
15%
10%
5%
0%
_whole_chrom
log10_2_3
log10_3_4
log10_4_5
log10_5_6
log10_6_7
log10_7_8
log10_8_9
_inter_chrom
Deletion
Inversion
Duplication
Translocation

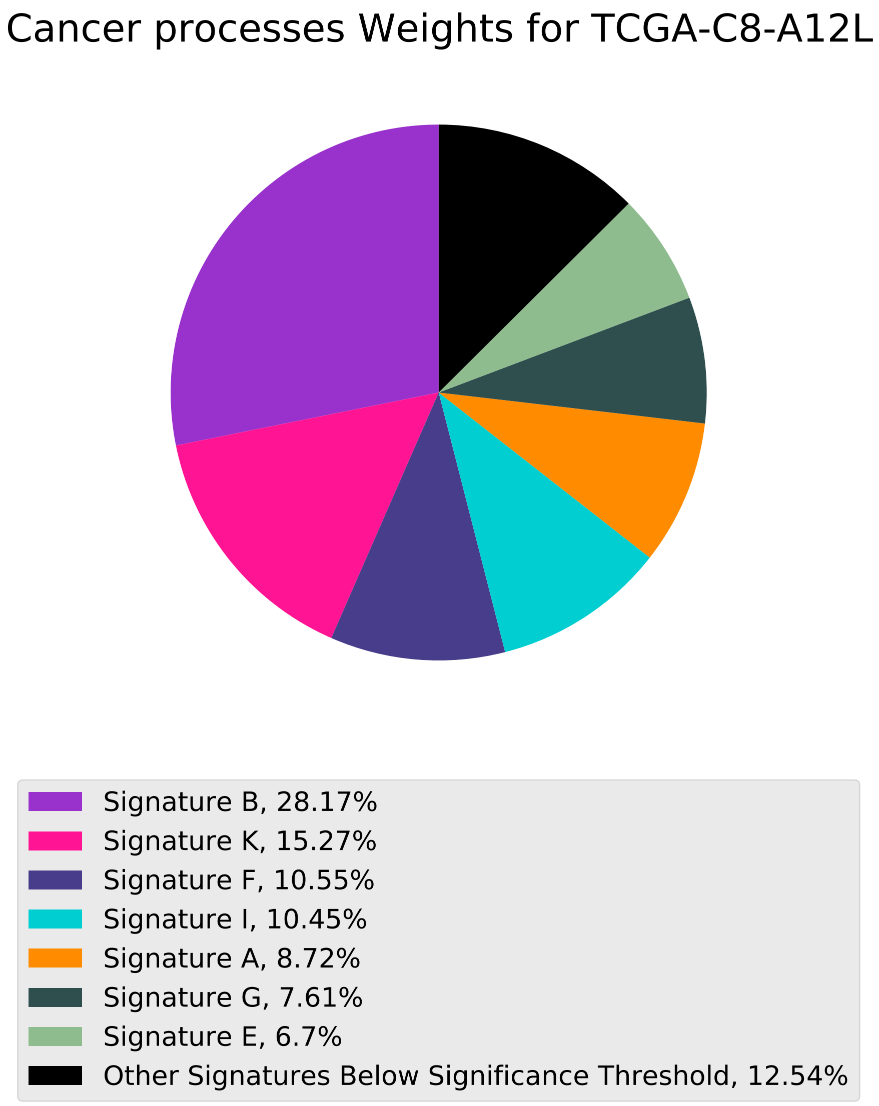
Cancer processes Weights for TCGA-C8-A12L
Signature B, 28.17%
Signature K, 15.27%
Signature F, 10.55%
Signature I, 10.45%
Signature A, 8.72%
Signature G, 7.61%
Signature E, 6.7%
Other Signatures Below Significance Threshold, 12.54%

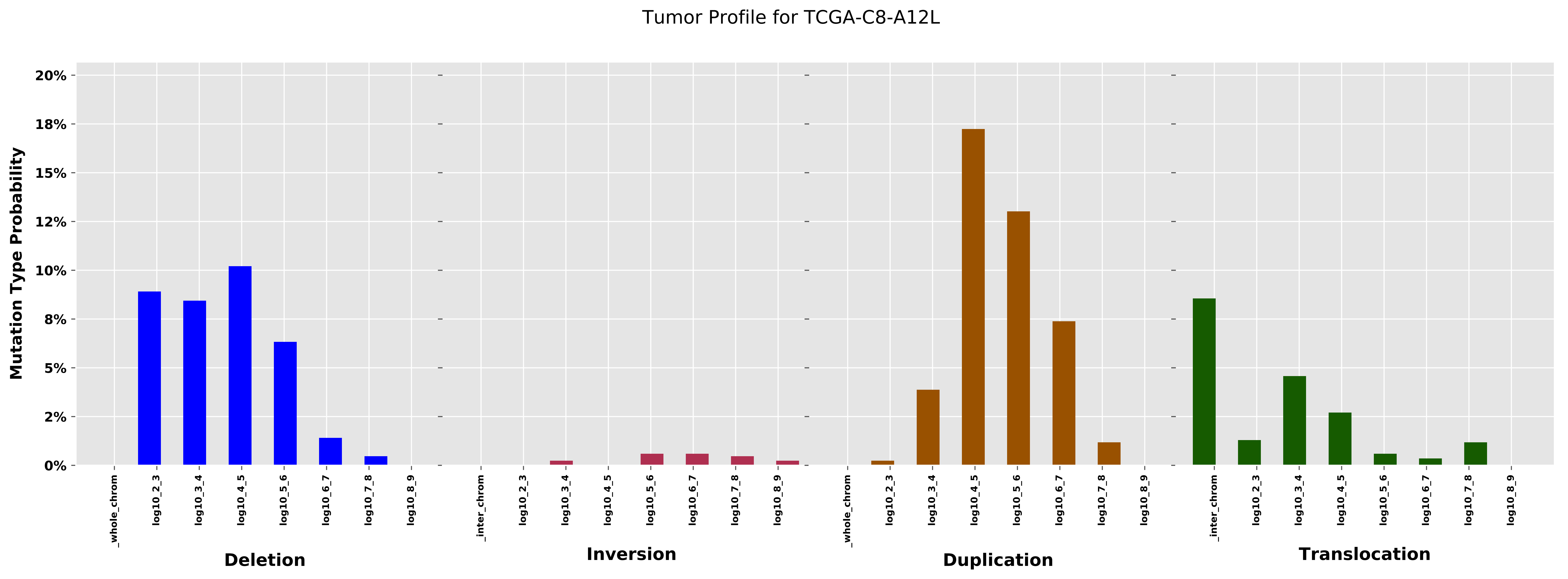

Tumor Profile for TCGA-C8-A12L
Mutation Type Probability
0%
2%
5%
8%
10%
12%
15%
18%
20%
_whole_chrom
log10_2_3
log10_3_4
log10_4_5
log10_5_6
log10_6_7
log10_7_8
log10_8_9
Deletion
_inter_chrom
Inversion
Duplication
Translocation

Cancer processes Weights for TCGA-AG-3890

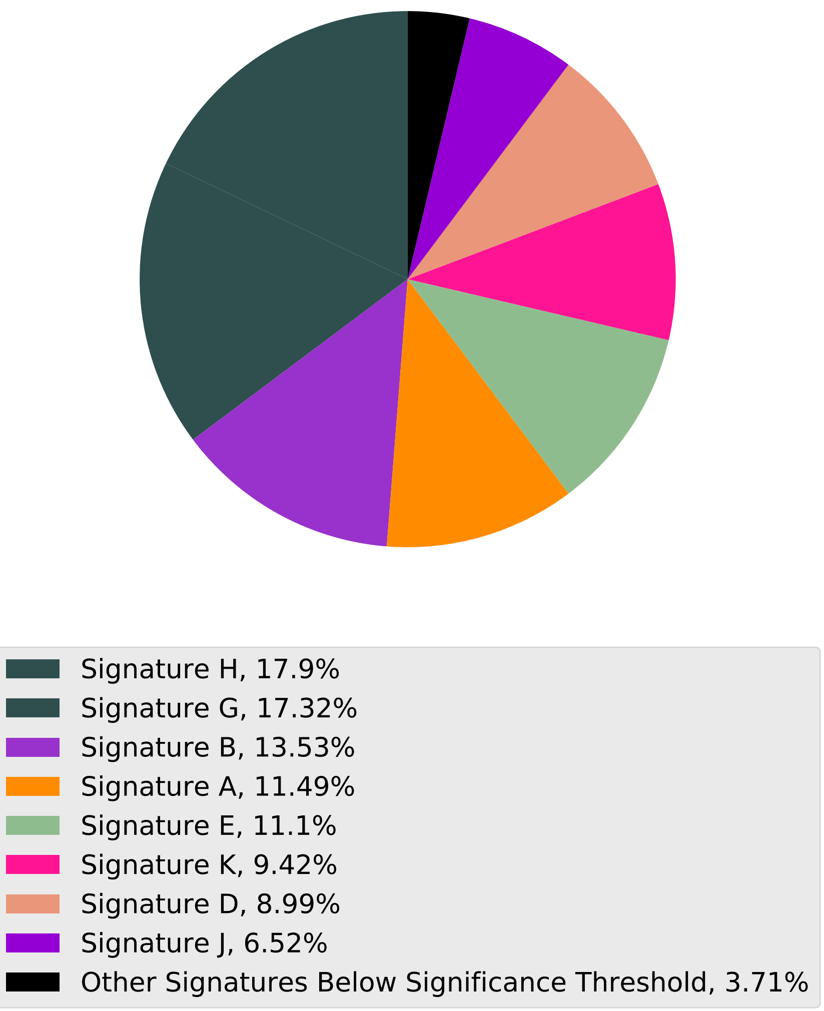

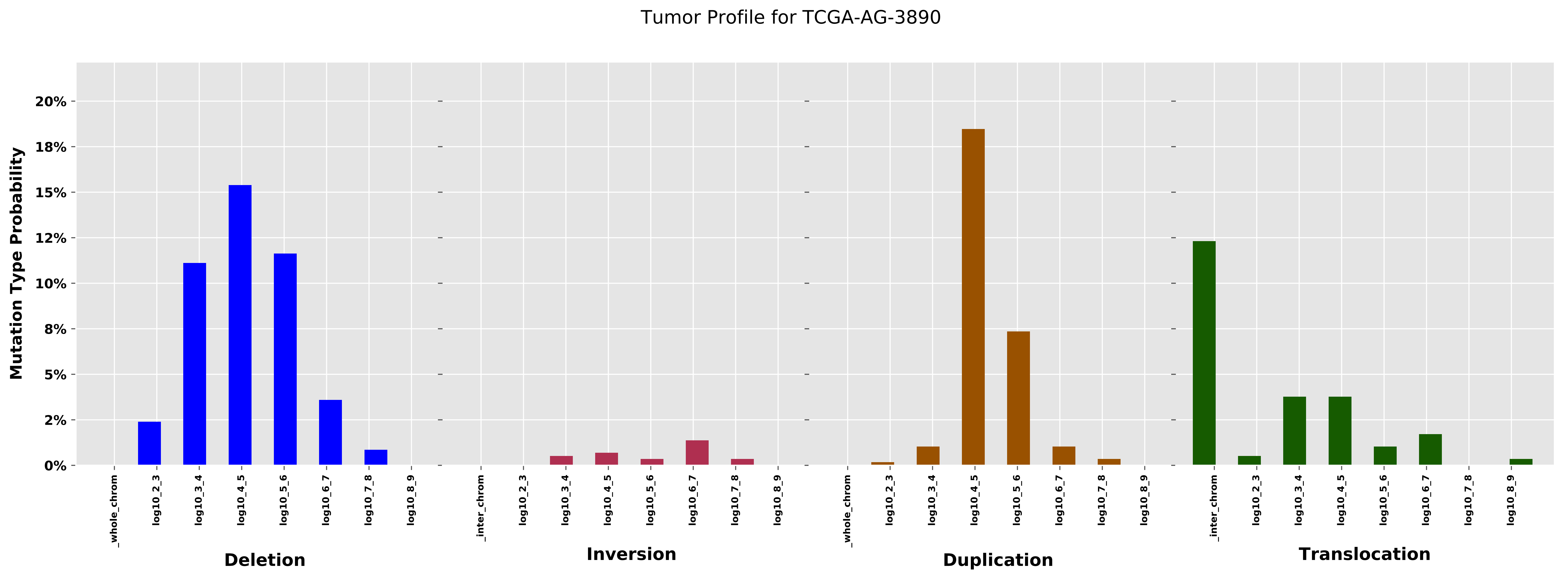
Tumor Profile for TCGA-AG-3890
Mutation Type Probability
0%
2%
5%
8%
10%
12%
15%
18%
20%
_whole_chrom
log10_2_3
log10_3_4
log10_4_5
log10_5_6
log10_6_7
log10_7_8
log10_8_9
_inter_chrom
Deletion
Inversion
Duplication
Translocation

Cancer processes Weights for TCGA-E2-A1LK

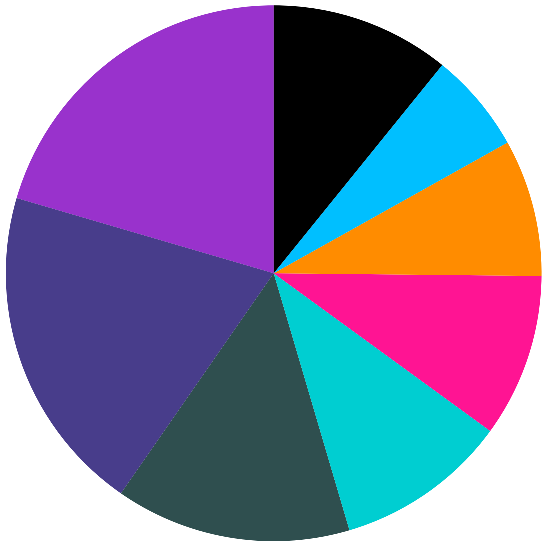

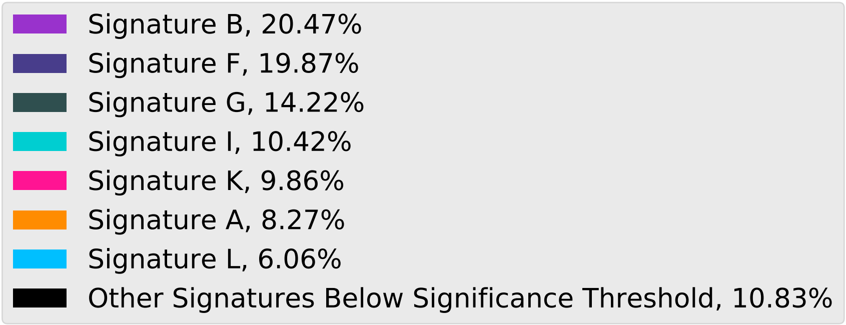

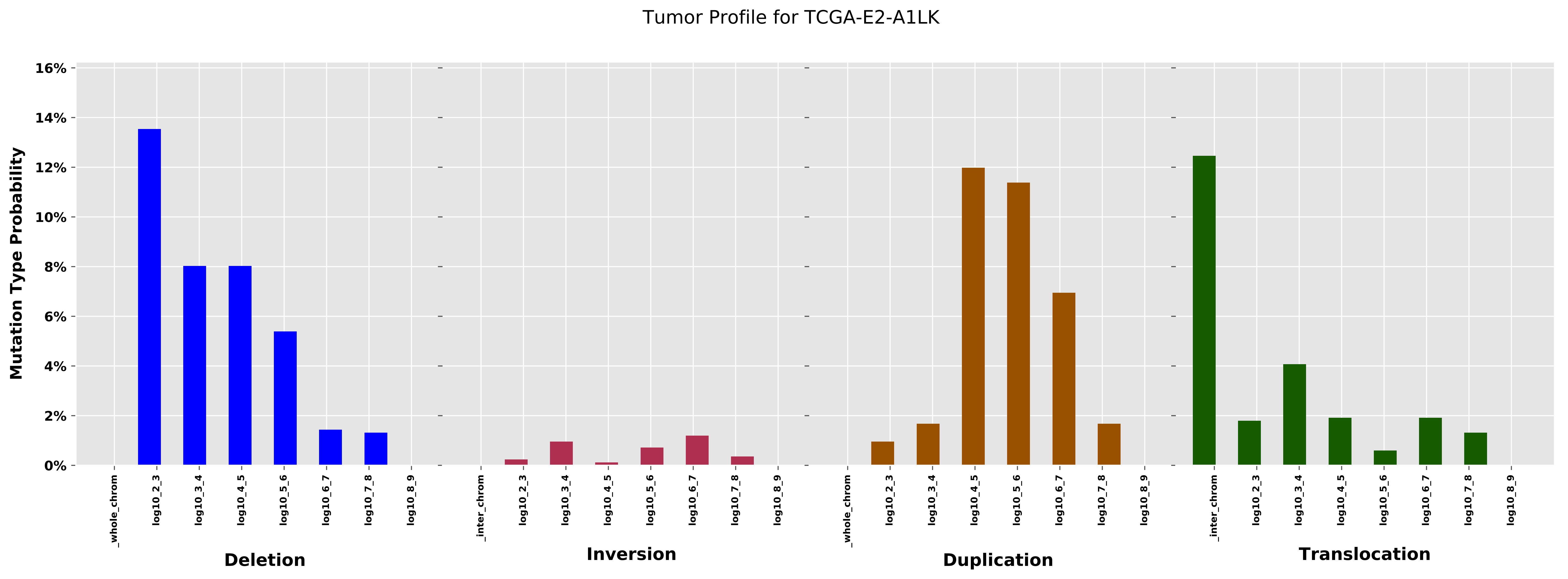
Tumor Profile for TCGA-E2-A1LK
Mutation Type Probability
16%
14%
12%
10%
8%
6%
4%
2%
0%
_whole_chrom
log10_2_3
log10_3_4
log10_4_5
log10_5_6
log10_6_7
log10_7_8
log10_8_9
Deletion
_inter_chrom
Inversion
Duplication
Translocation

Cancer processes Weights for TCGA-A2-A3Y0

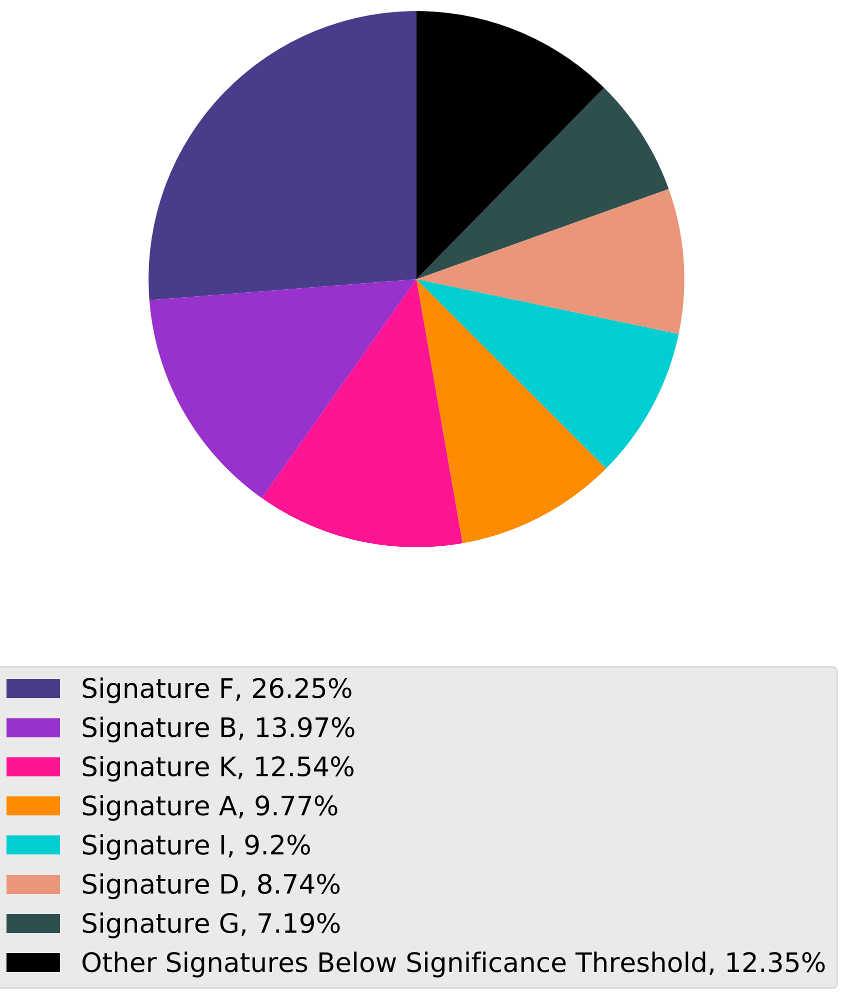

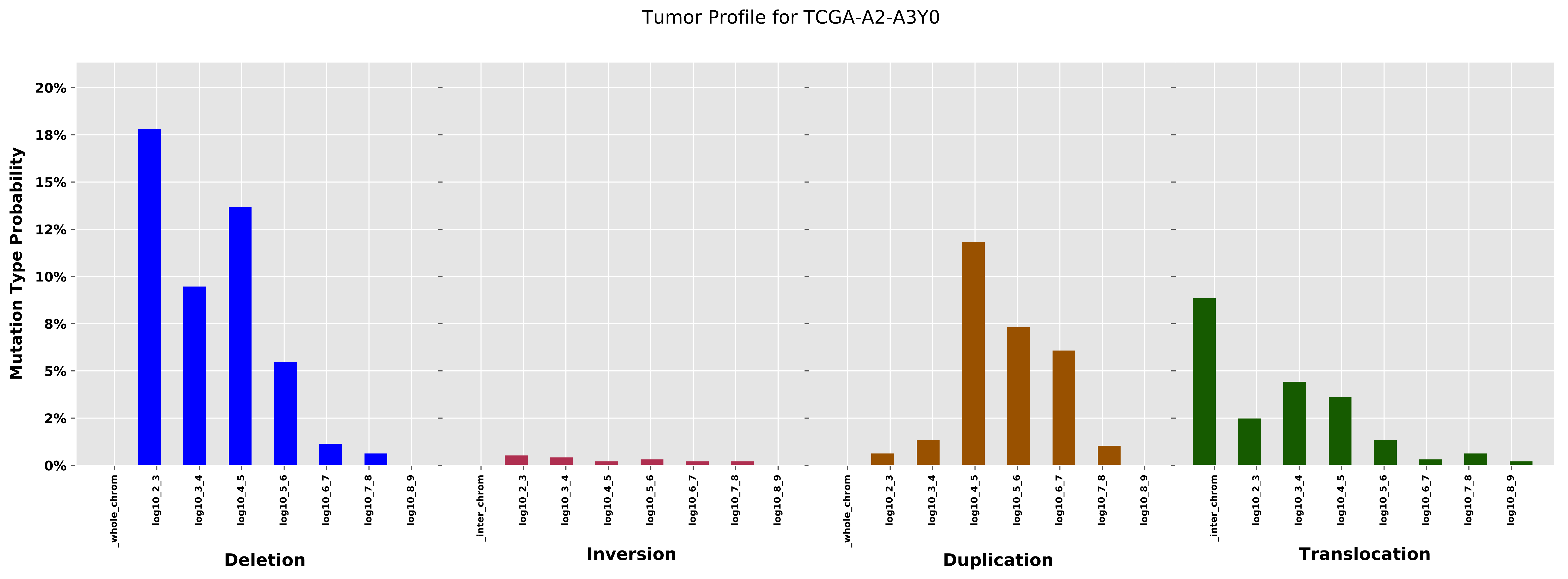

Tumor Profile for TCGA-A2-A3Y0
Mutation Type Probability
20%
18%
15%
12%
10%
8%
5%
2%
0%
_whole_chrom
log10_2_3
log10_3_4
log10_4_5
log10_5_6
log10_6_7
log10_7_8
log10_8_9
_inter_chrom
Deletion
Inversion
Duplication
Translocation

Cancer processes Weights for TCGA-B6-A0I1

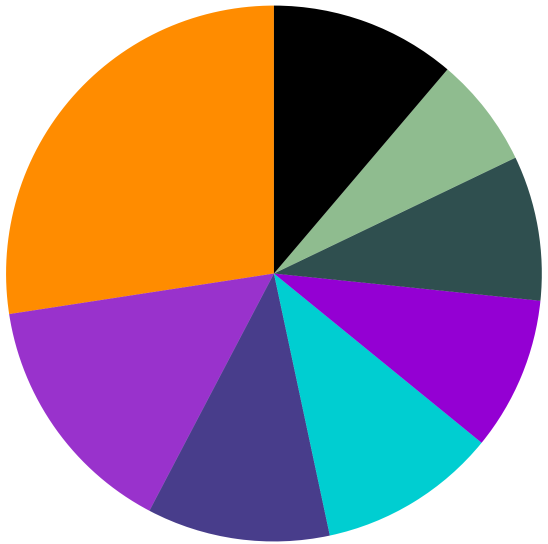

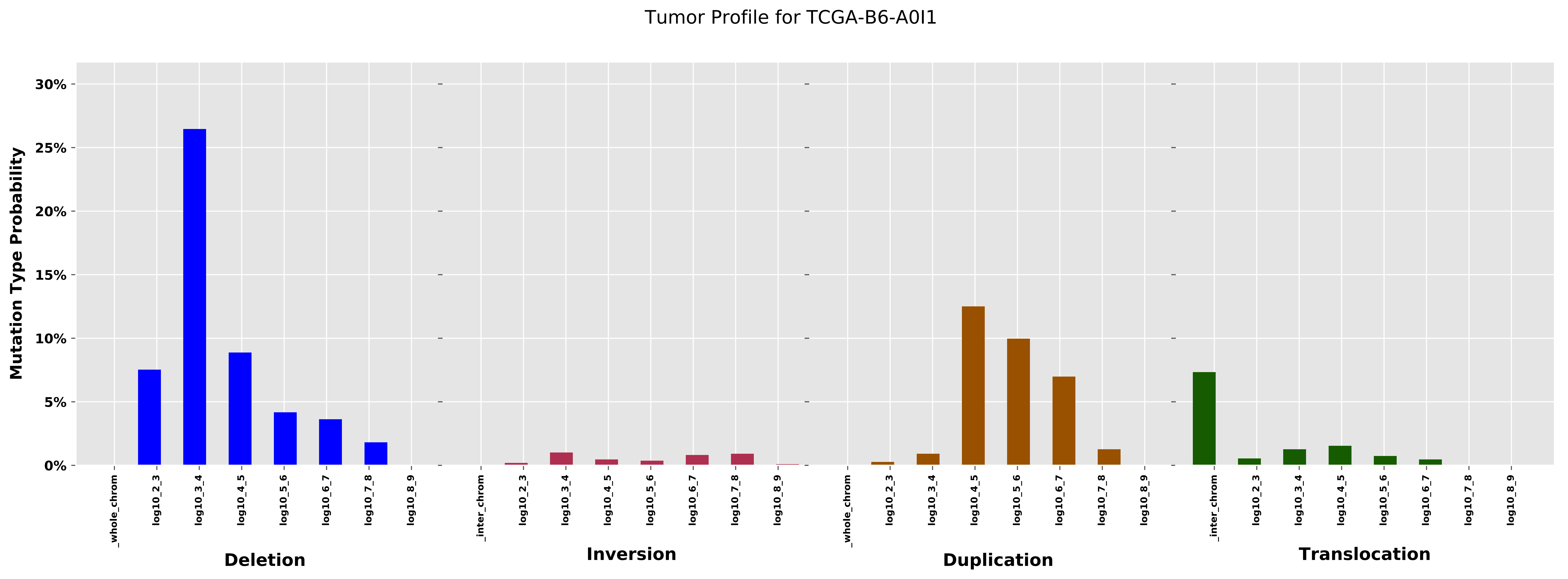

Tumor Profile for TCGA-B6-A0I1
Mutation Type Probability
30%
25%
20%
15%
10%
5%
0%
_whole_chrom
log10_2_3
log10_3_4
log10_4_5
log10_5_6
log10_6_7
log10_7_8
log10_8_9
_inter_chrom
Deletion
Inversion
Duplication
Translocation

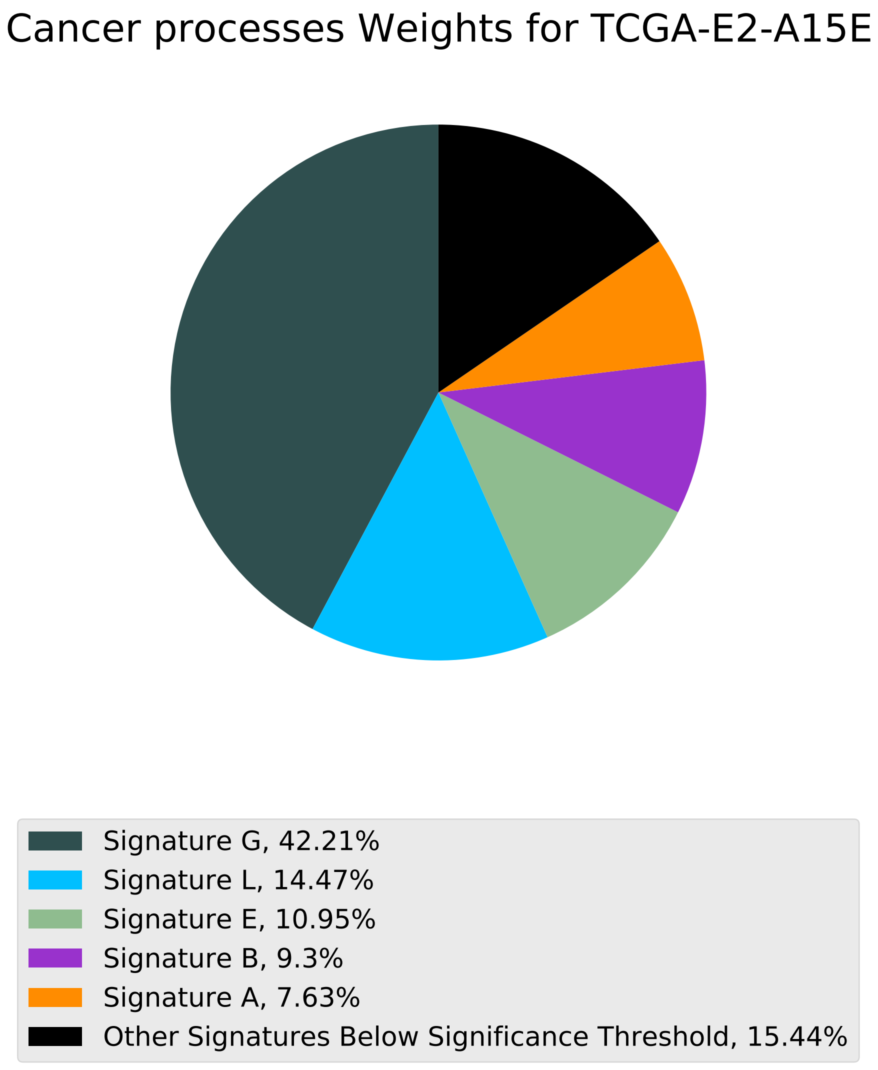
Cancer processes Weights for TCGA-E2-A15E
Signature G, 42.21%
Signature L, 14.47%
Signature E, 10.95%
Signature B, 9.3%
Signature A, 7.63%
Other Signatures Below Significance Threshold, 15.44%

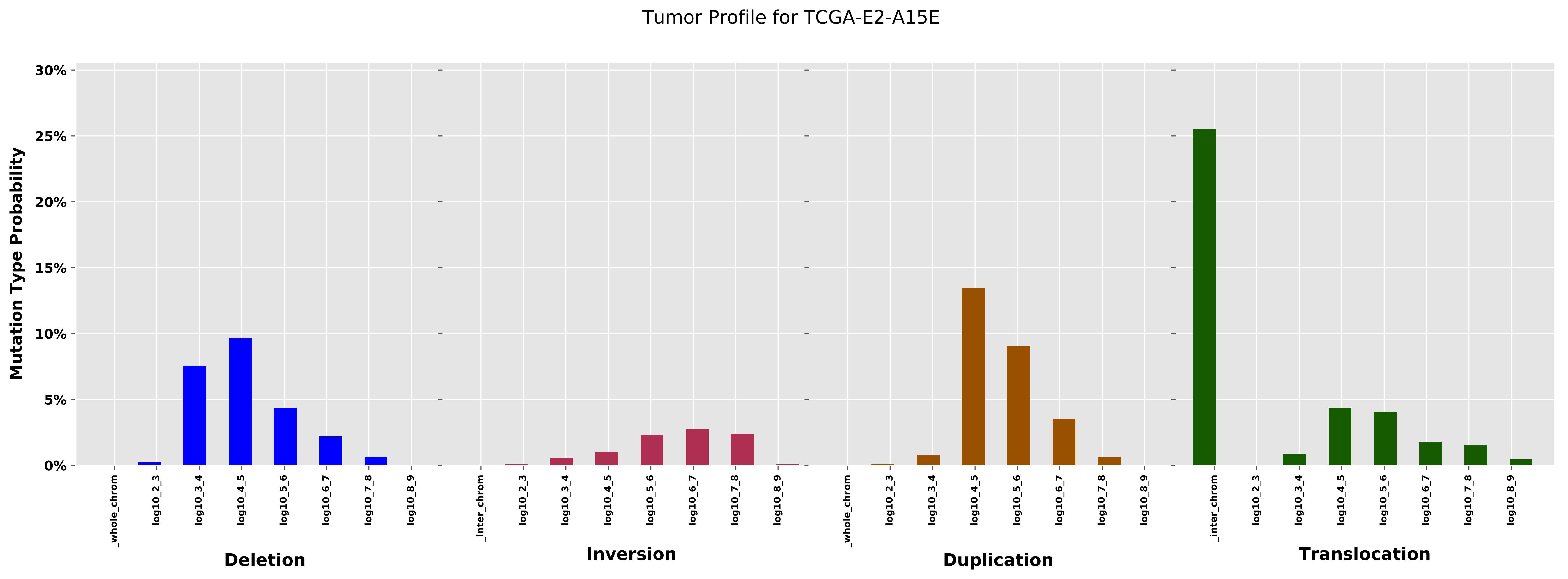

Tumor Profile for TCGA-E2-A15E
Mutation Type Probability
30%
25%
20%
15%
10%
5%
0%
_whole_chrom
log10_2_3
log10_3_4
log10_4_5
log10_5_6
log10_6_7
log10_7_8
log10_8_9
Deletion
_inter_chrom
Inversion
Duplication
Translocation

Cancer processes Weights for TCGA-A6-2680

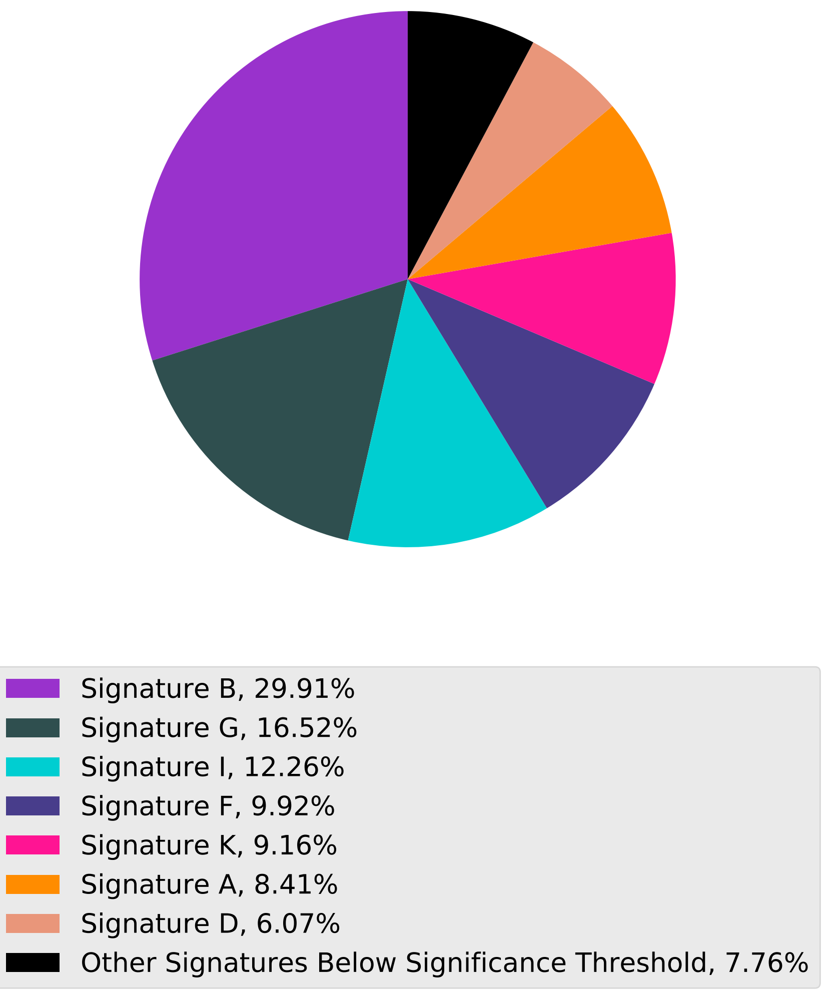

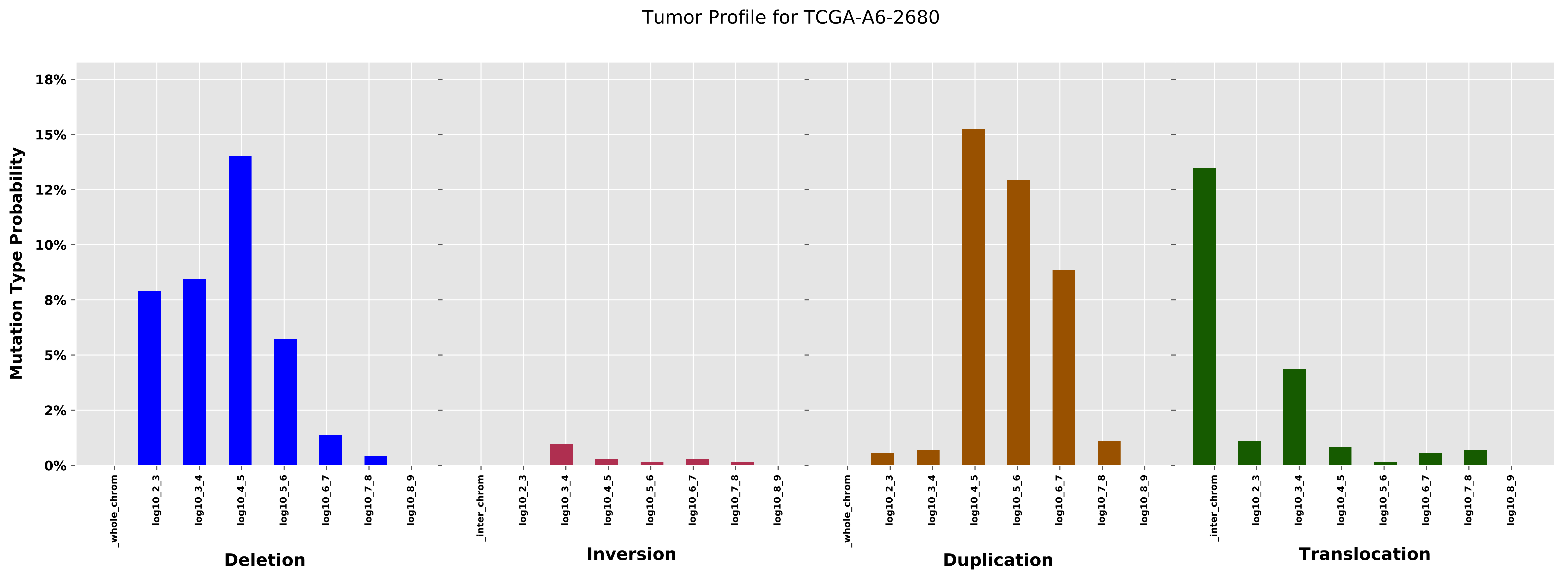

Tumor Profile for TCGA-A6-2680
Mutation Type Probability
18%
15%
12%
10%
8%
5%
2%
0%
_whole_chrom
log10_2_3
log10_3_4
log10_4_5
log10_5_6
log10_6_7
log10_7_8
log10_8_9
_inter_chrom
Deletion
Inversion
Duplication
Translocation

Cancer processes Weights for TCGA-A8-A07B

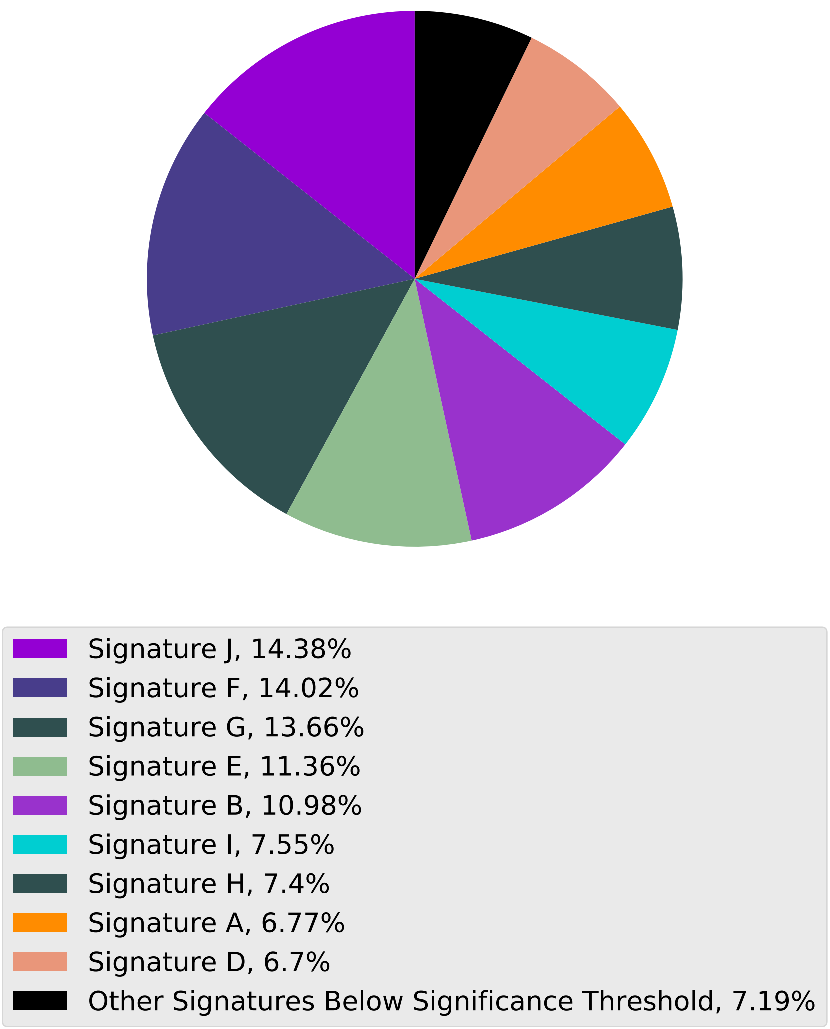

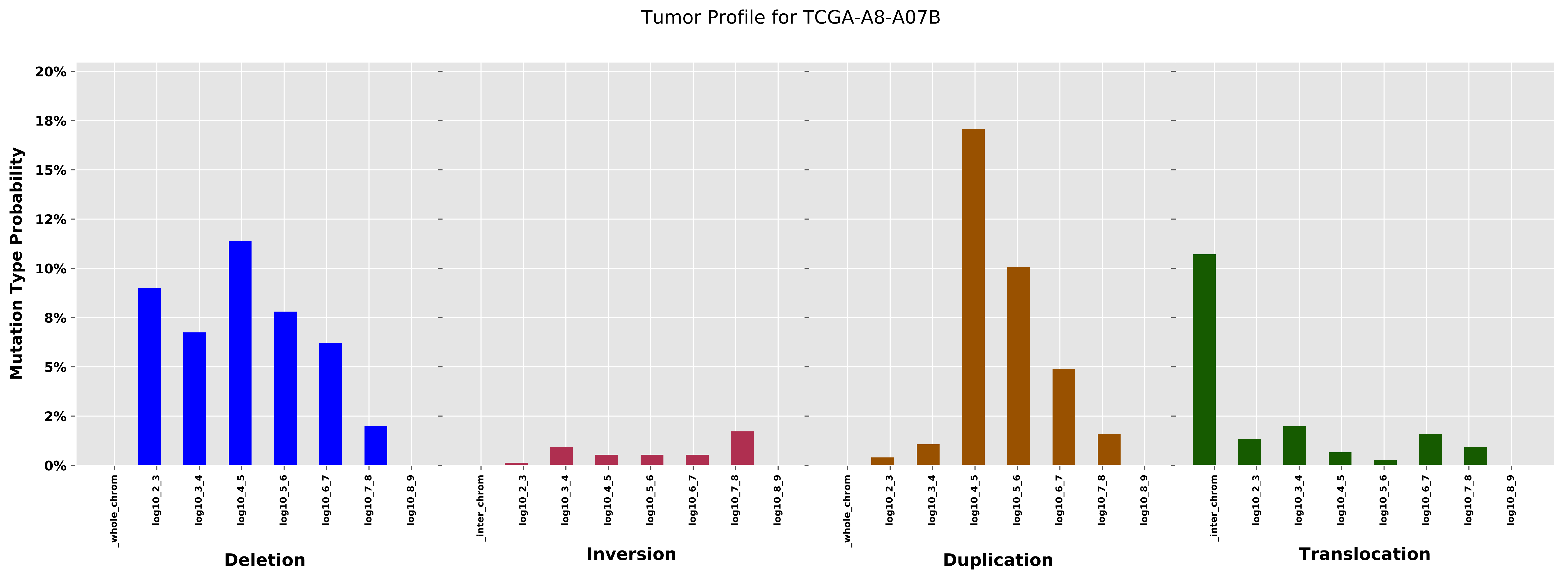

Tumor Profile for TCGA-A8-A07B
Mutation Type Probability
20%
18%
15%
12%
10%
8%
5%
2%
0%
_whole_chrom
log10_2_3
log10_3_4
log10_4_5
log10_5_6
log10_6_7
log10_7_8
log10_8_9
_inter_chrom
Deletion
Inversion
Duplication
Translocation

Cancer processes Weights for TCGA-AO-A0J6

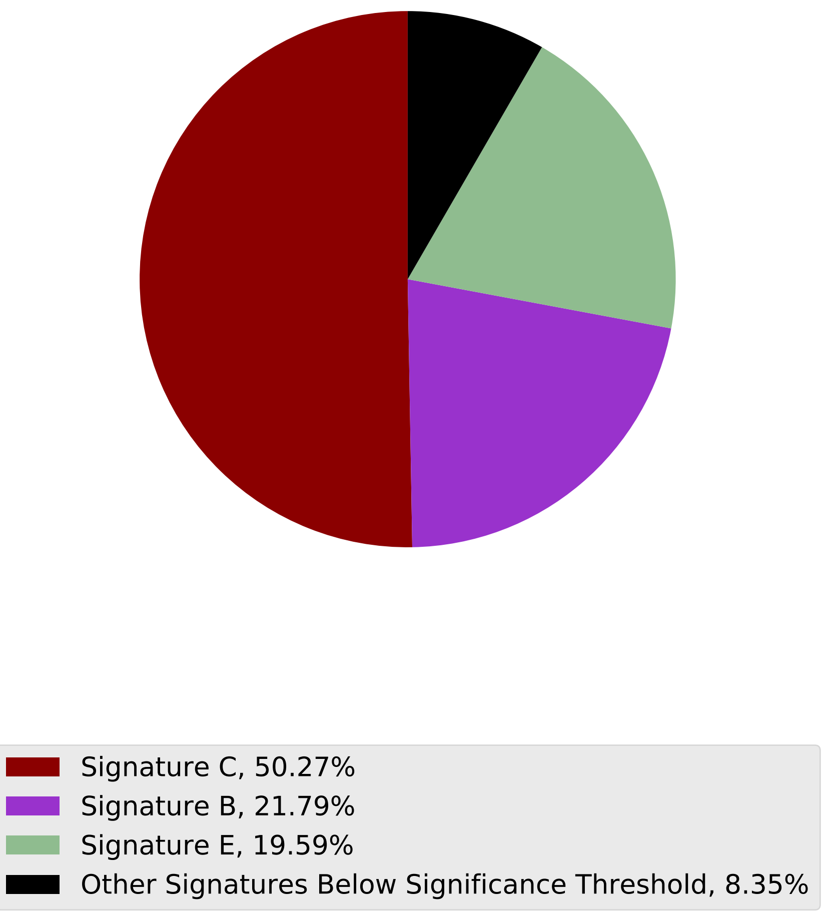

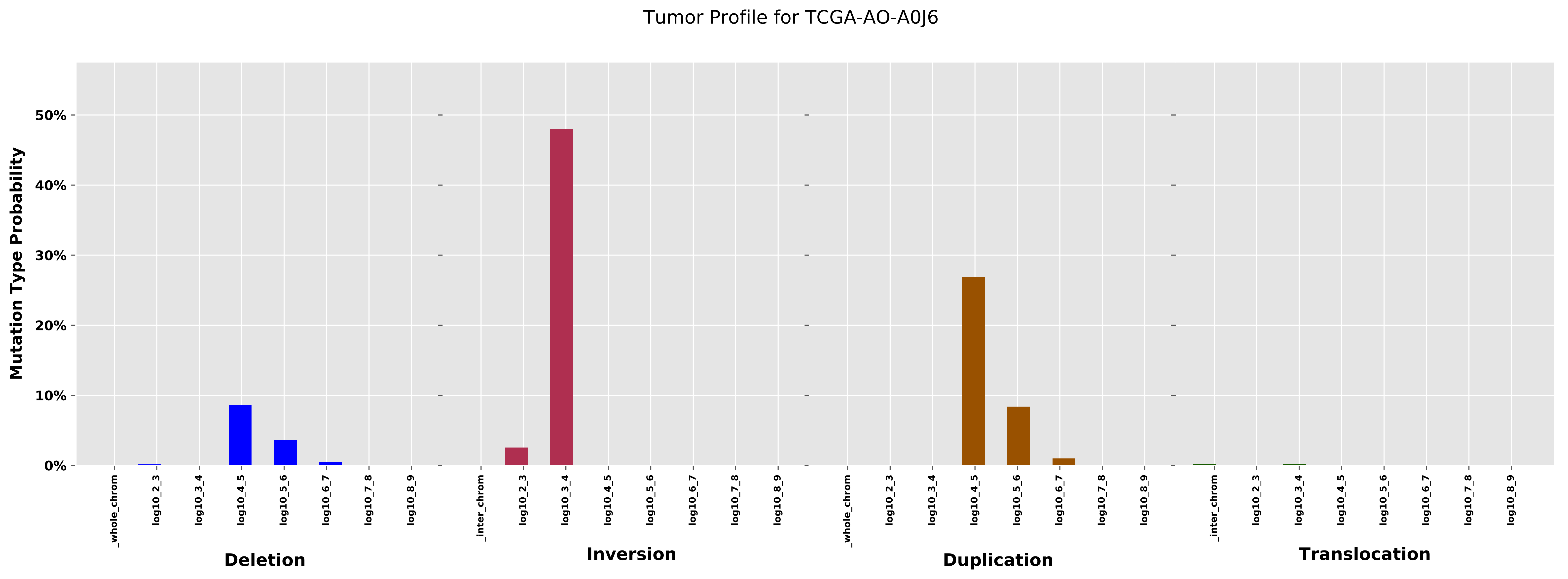
Tumor Profile for TCGA-AO-A0J6
Mutation Type Probability
50%
40%
30%
20%
10%
0%
_whole_chrom
log10_2_3
log10_3_4
log10_4_5
log10_5_6
log10_6_7
log10_7_8
log10_8_9
_inter_chrom
Deletion
Inversion
Duplication
Translocation

Cancer processes Weights for TCGA-A2-A3XX

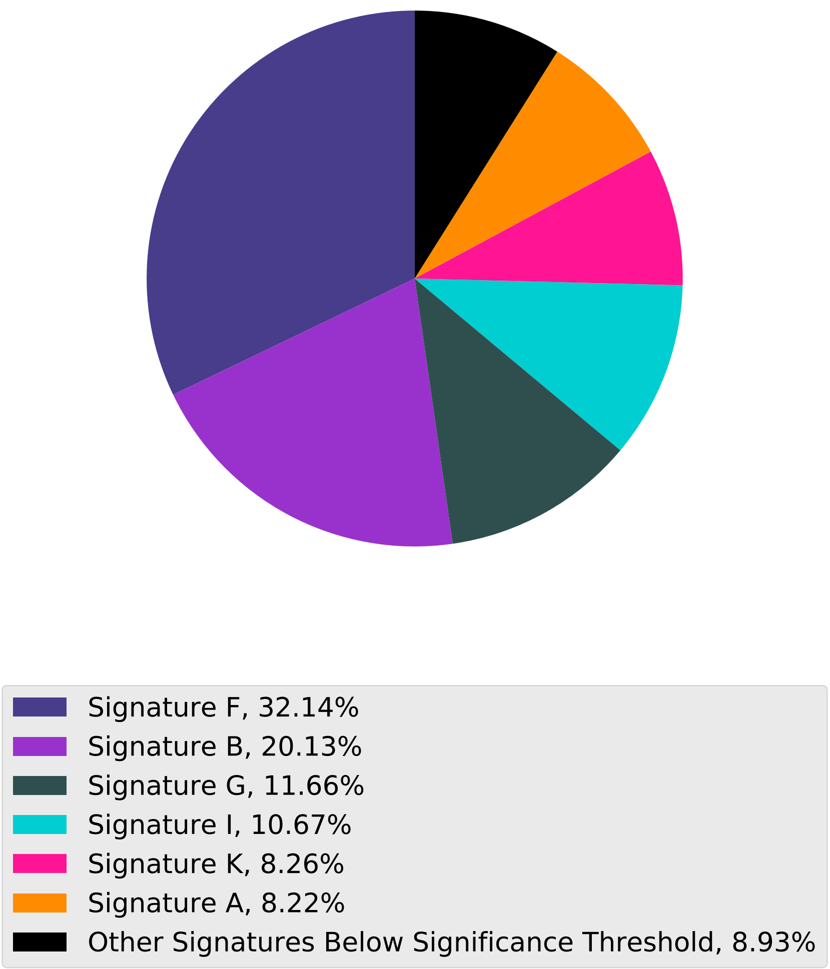

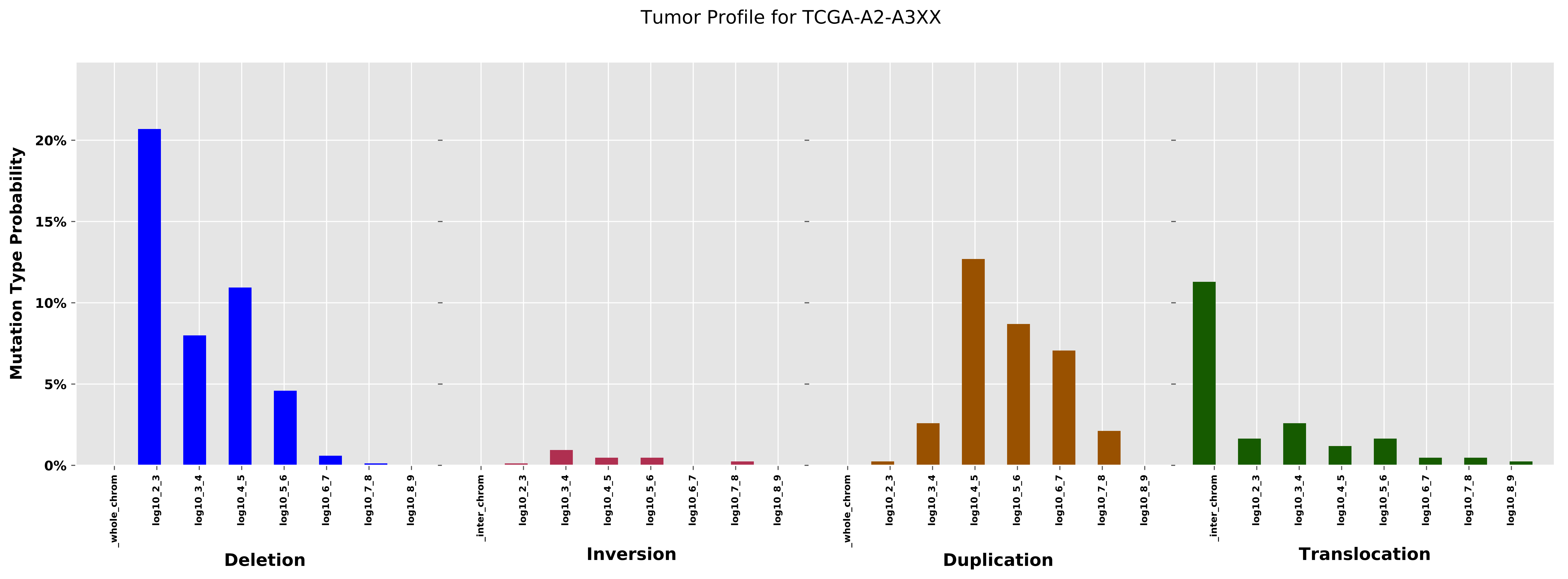

Tumor Profile for TCGA-A2-A3XX
Mutation Type Probability
0%
5%
10%
15%
20%
_whole_chrom
log10_2_3
log10_3_4
log10_4_5
log10_5_6
log10_6_7
log10_7_8
log10_8_9
Deletion
_inter_chrom
log10_2_3
log10_3_4
log10_4_5
log10_5_6
log10_6_7
log10_7_8
log10_8_9
Inversion
_whole_chrom
log10_2_3
log10_3_4
log10_4_5
log10_5_6
log10_6_7
log10_7_8
log10_8_9
Duplication
_inter_chrom
log10_2_3
log10_3_4
log10_4_5
log10_5_6
log10_6_7
log10_7_8
log10_8_9
Translocation

Cancer processes Weights for TCGA-A8-A075

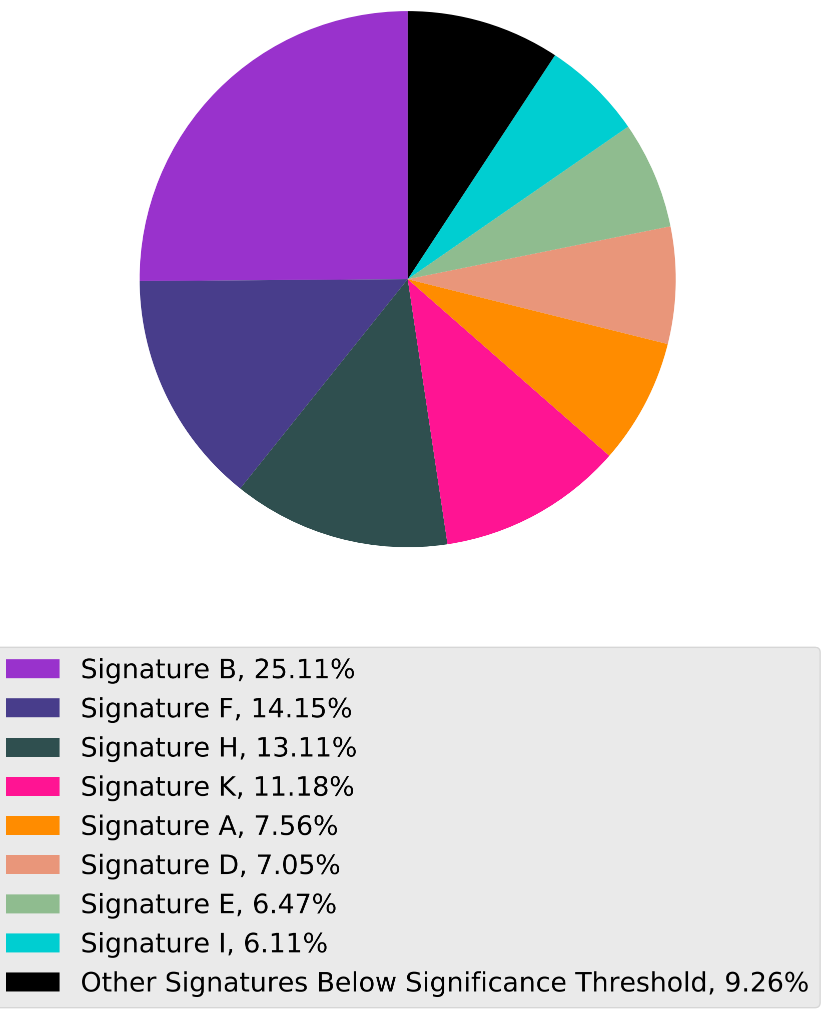

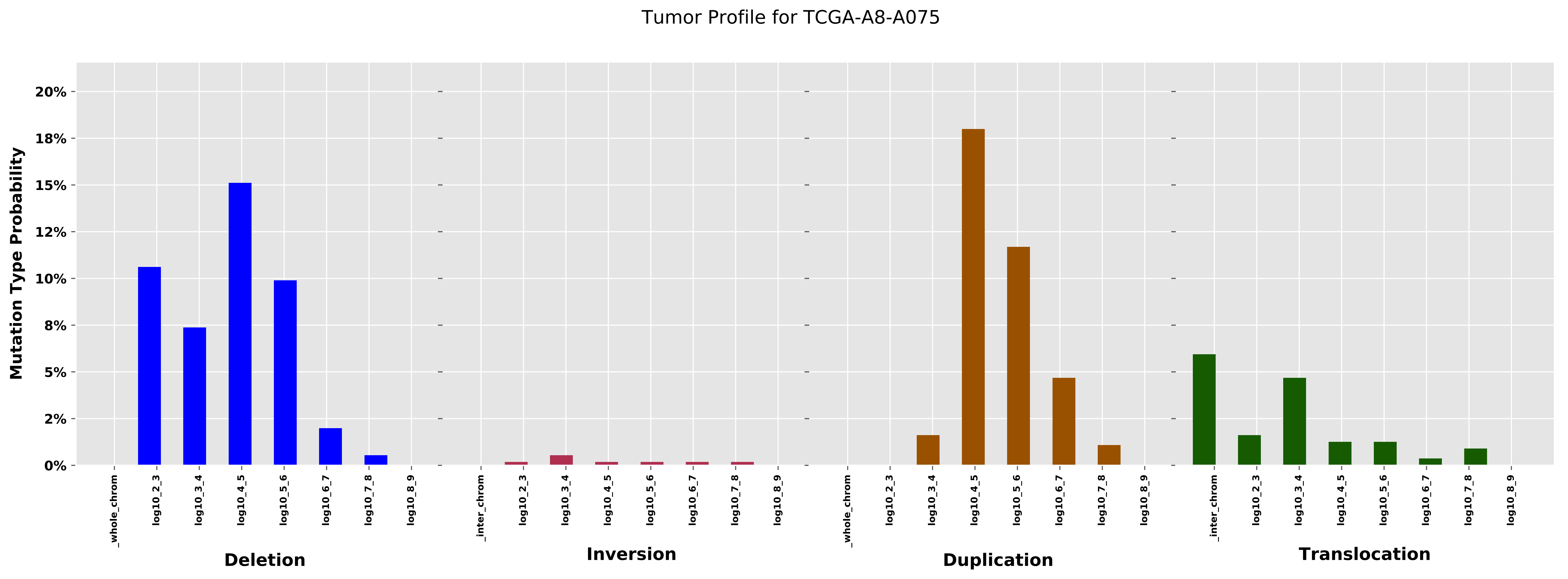

Tumor Profile for TCGA-A8-A075
Mutation Type Probability
0%
2%
5%
8%
10%
12%
15%
18%
20%
_whole_chrom
log10_2_3
log10_3_4
log10_4_5
log10_5_6
log10_6_7
log10_7_8
log10_8_9
_inter_chrom
Deletion
Inversion
Duplication
Translocation

Cancer processes Weights for TCGA-AR-A1AY

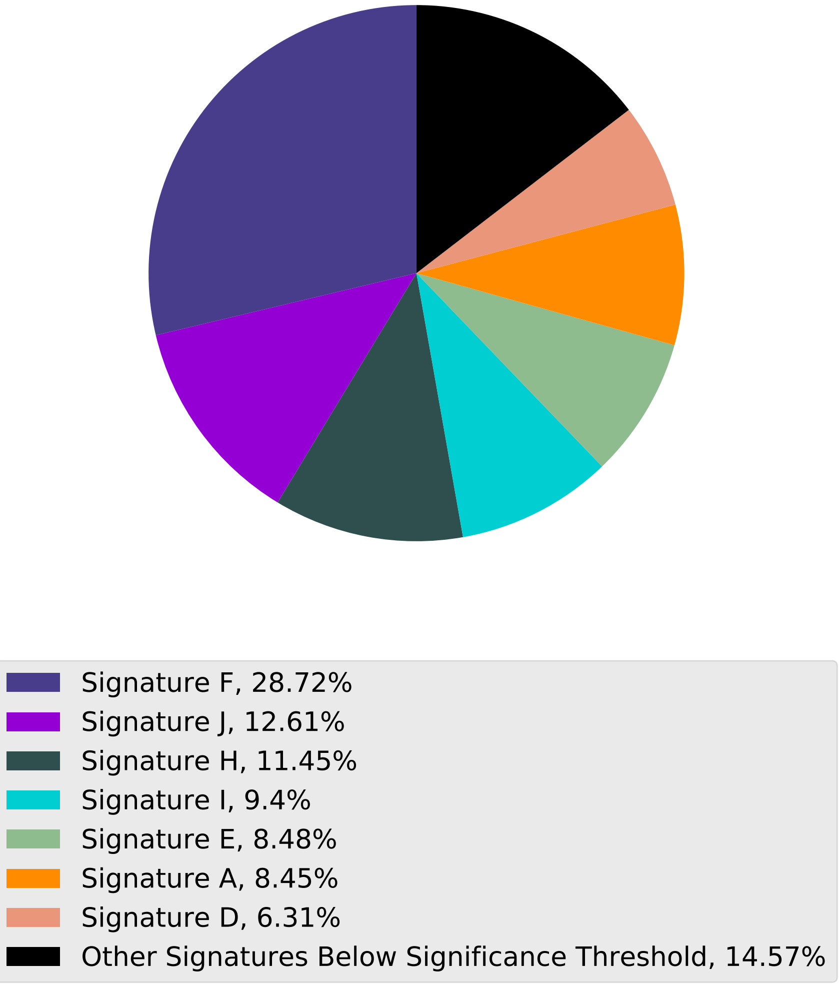

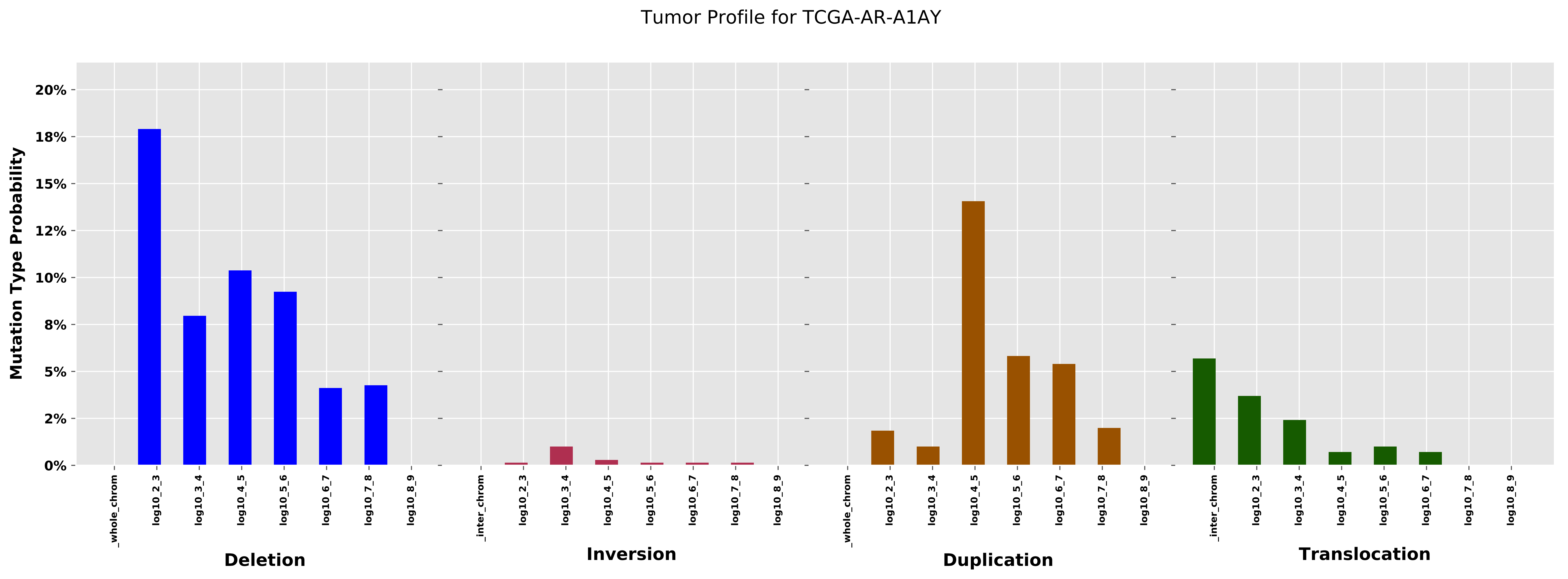

Tumor Profile for TCGA-AR-A1AY
Mutation Type Probability
20%
18%
15%
12%
10%
8%
5%
2%
0%
_whole_chrom
log10_2_3
log10_3_4
log10_4_5
log10_5_6
log10_6_7
log10_7_8
log10_8_9
Deletion
_inter_chrom
log10_2_3
log10_3_4
log10_4_5
log10_5_6
log10_6_7
log10_7_8
log10_8_9
Inversion
_whole_chrom
log10_2_3
log10_3_4
log10_4_5
log10_5_6
log10_6_7
log10_7_8
log10_8_9
Duplication
_inter_chrom
log10_2_3
log10_3_4
log10_4_5
log10_5_6
log10_6_7
log10_7_8
log10_8_9
Translocation

Cancer processes Weights for TCGA-AG-3901

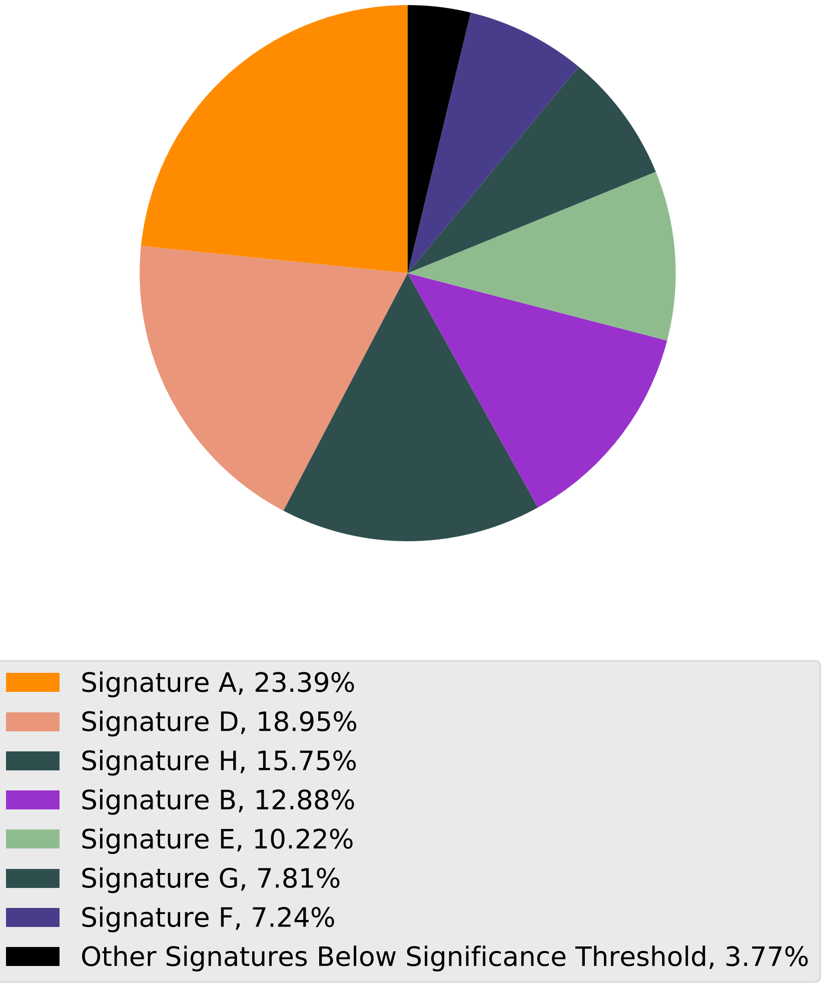

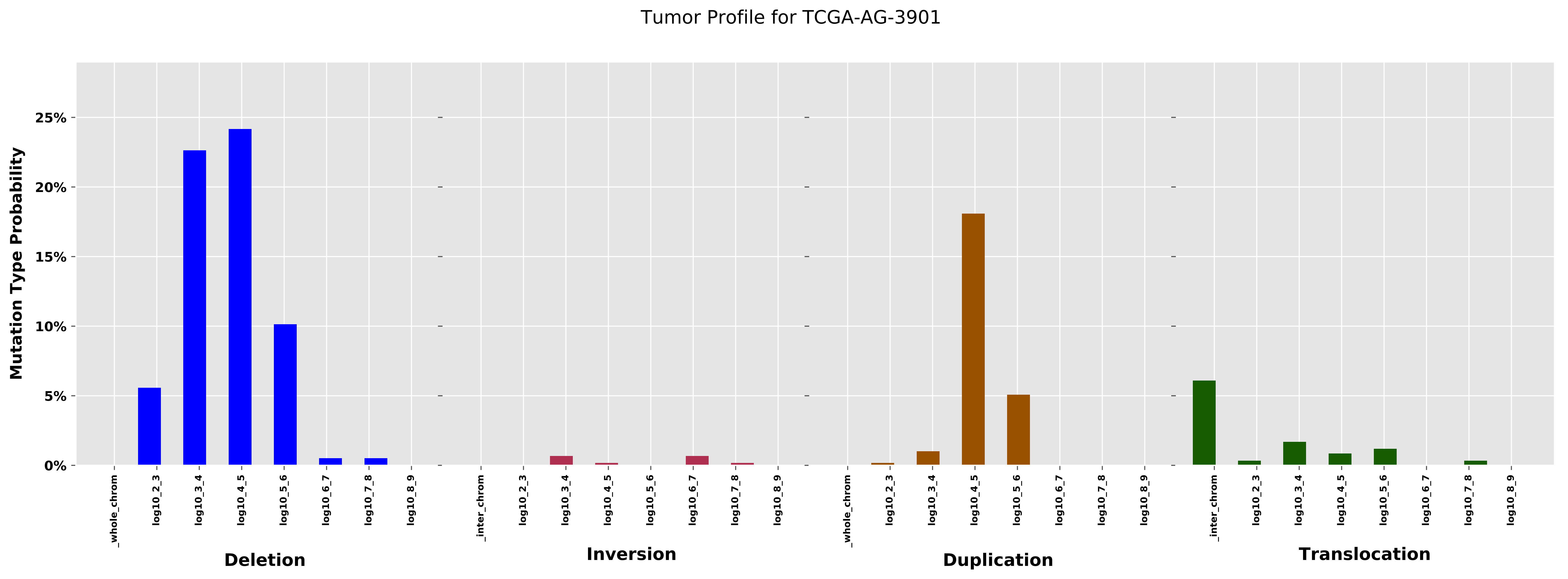
Tumor Profile for TCGA-AG-3901
Mutation Type Probability
0%
5%
10%
15%
20%
25%
_whole_chrom
log10_2_3
log10_3_4
log10_4_5
log10_5_6
log10_6_7
log10_7_8
log10_8_9
_inter_chrom
Deletion
Inversion
Duplication
Translocation

Cancer processes Weights for TCGA-E2-A152

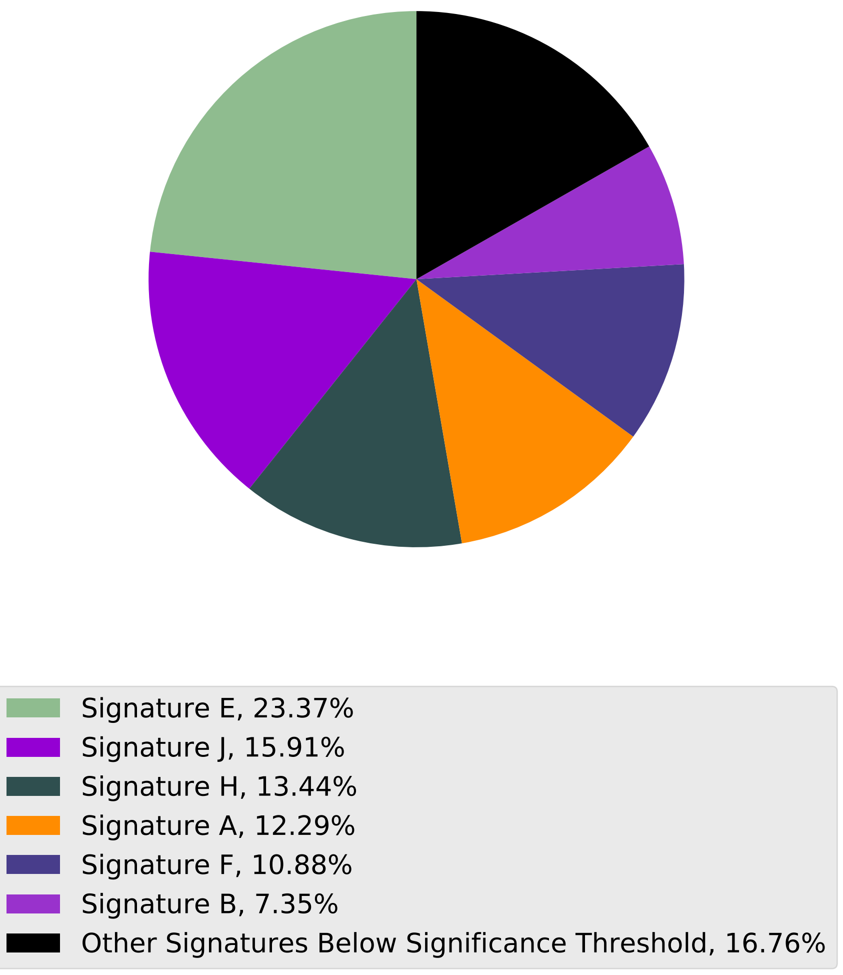

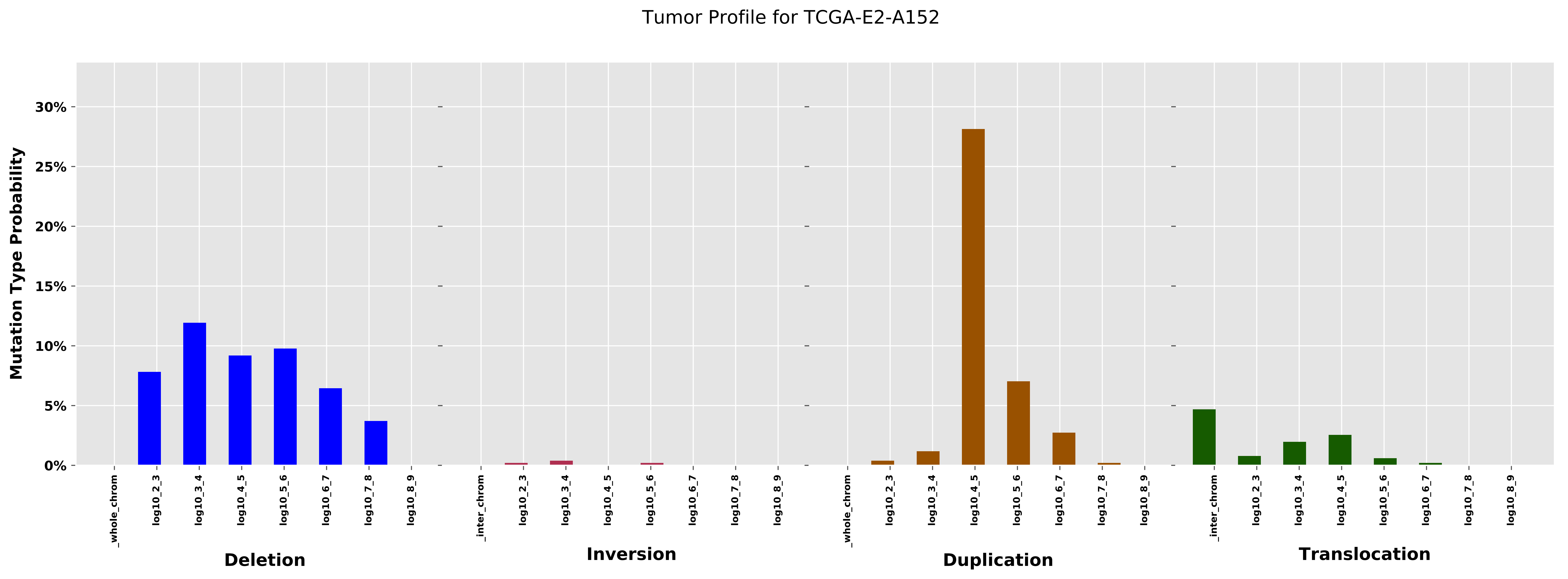

Tumor Profile for TCGA-E2-A152
Mutation Type Probability
30%
25%
20%
15%
10%
5%
0%
_whole_chrom
log10_2_3
log10_3_4
log10_4_5
log10_5_6
log10_6_7
log10_7_8
log10_8_9
Deletion
_inter_chrom
log10_2_3
log10_3_4
log10_4_5
log10_5_6
log10_6_7
log10_7_8
log10_8_9
Inversion
_whole_chrom
log10_2_3
log10_3_4
log10_4_5
log10_5_6
log10_6_7
log10_7_8
log10_8_9
Duplication
_inter_chrom
log10_2_3
log10_3_4
log10_4_5
log10_5_6
log10_6_7
log10_7_8
log10_8_9
Translocation

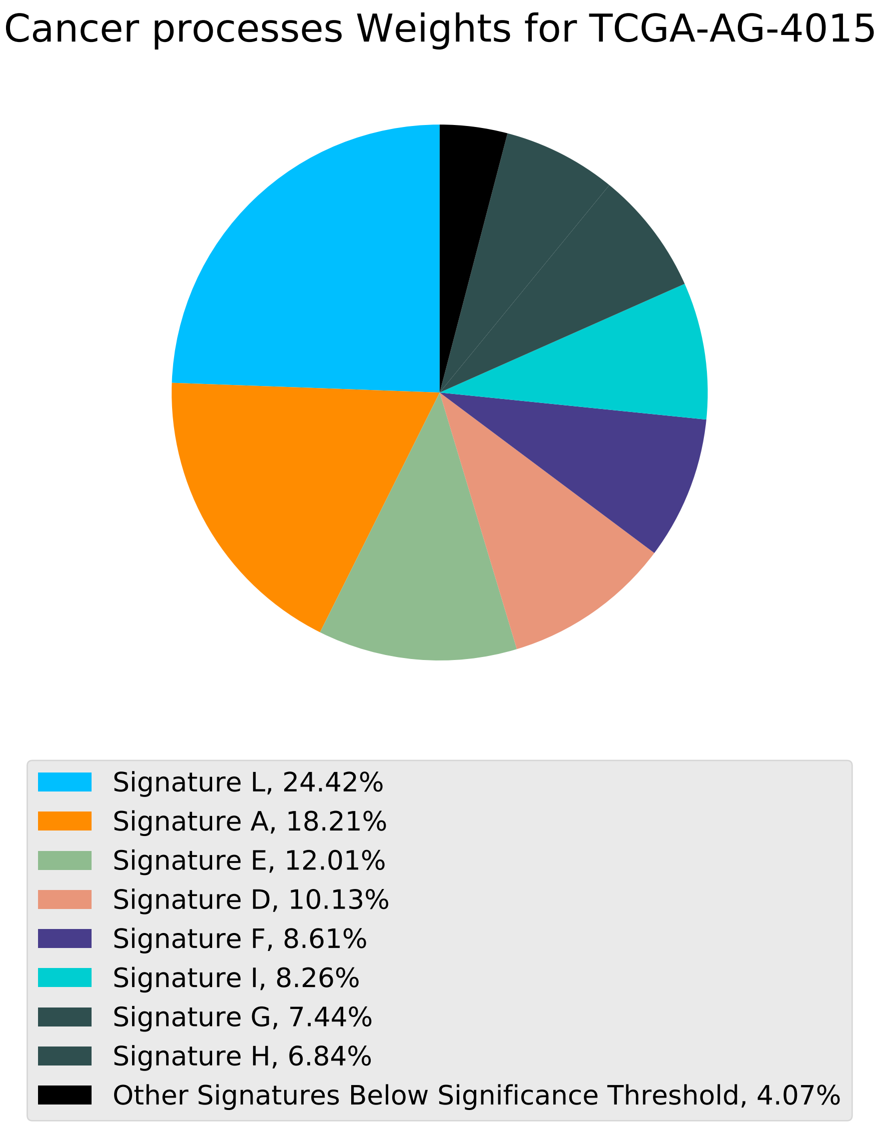
Cancer processes Weights for TCGA-AG-4015
Signature L, 24.42%
Signature A, 18.21%
Signature E, 12.01%
Signature D, 10.13%
Signature F, 8.61%
Signature I, 8.26%
Signature G, 7.44%
Signature H, 6.84%
Other Signatures Below Significance Threshold, 4.07%

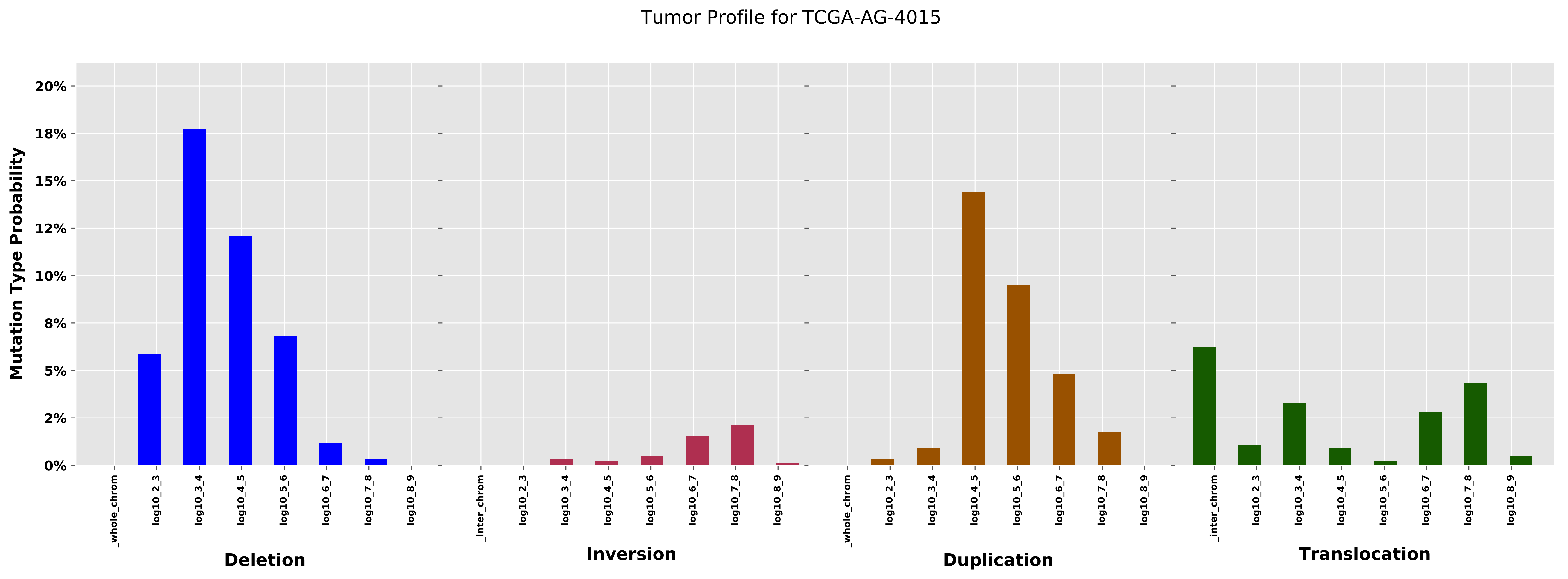
Tumor Profile for TCGA-AG-4015
Mutation Type Probability
20%
18%
15%
12%
10%
8%
5%
2%
0%
_whole_chrom
log10_2_3
log10_3_4
log10_4_5
log10_5_6
log10_6_7
log10_7_8
log10_8_9
_inter_chrom
Deletion
Inversion
Duplication
Translocation

Cancer processes Weights for TCGA-BH-A0B9

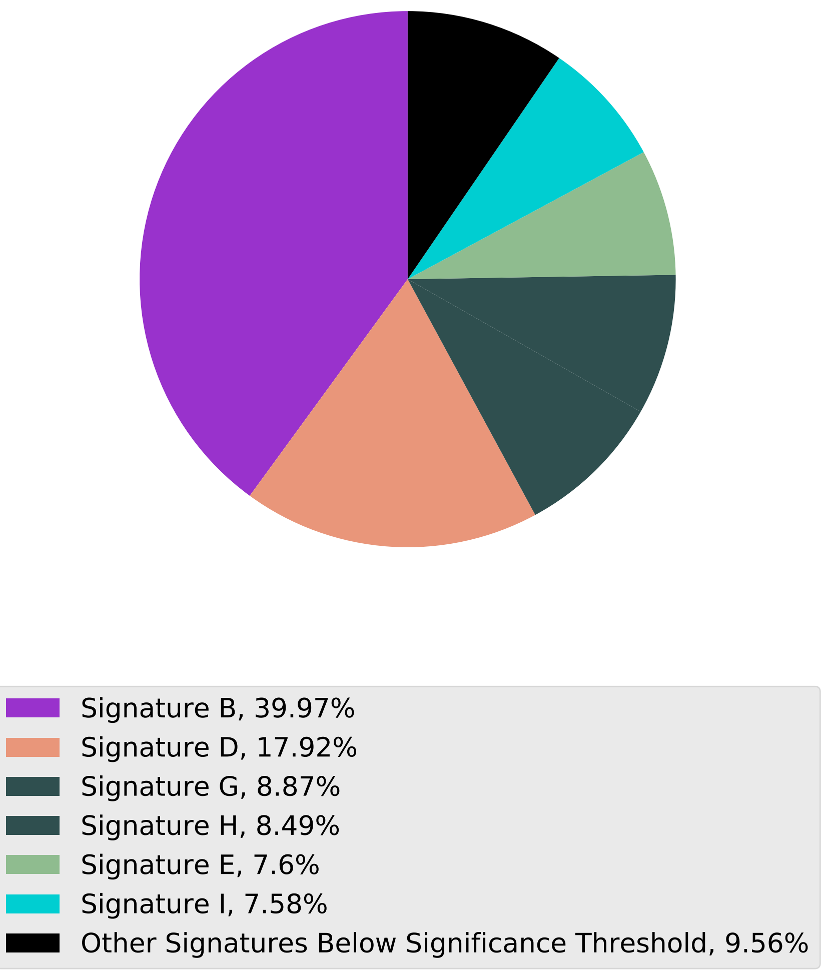

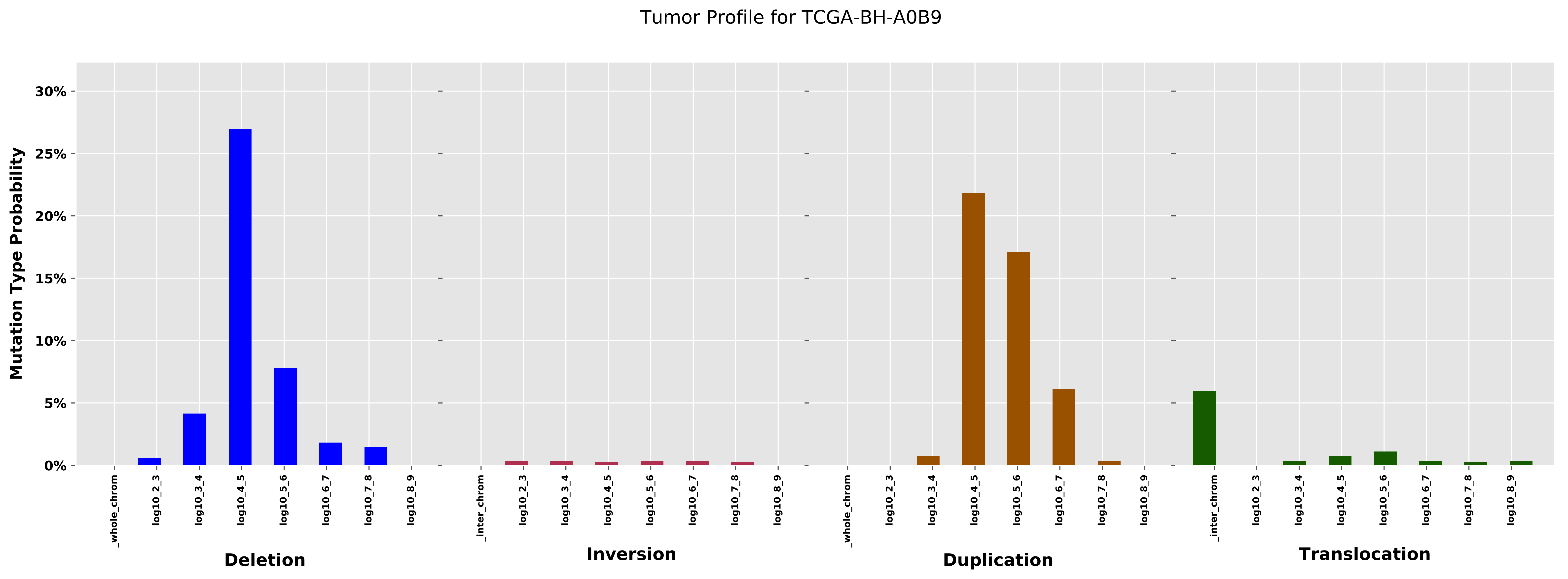

Tumor Profile for TCGA-BH-A0B9
Mutation Type Probability
30%
25%
20%
15%
10%
5%
0%
_whole_chrom
log10_2_3
log10_3_4
log10_4_5
log10_5_6
log10_6_7
log10_7_8
log10_8_9
_inter_chrom
Deletion
Inversion
Duplication
Translocation

Cancer processes Weights for TCGA-A2-A0D1

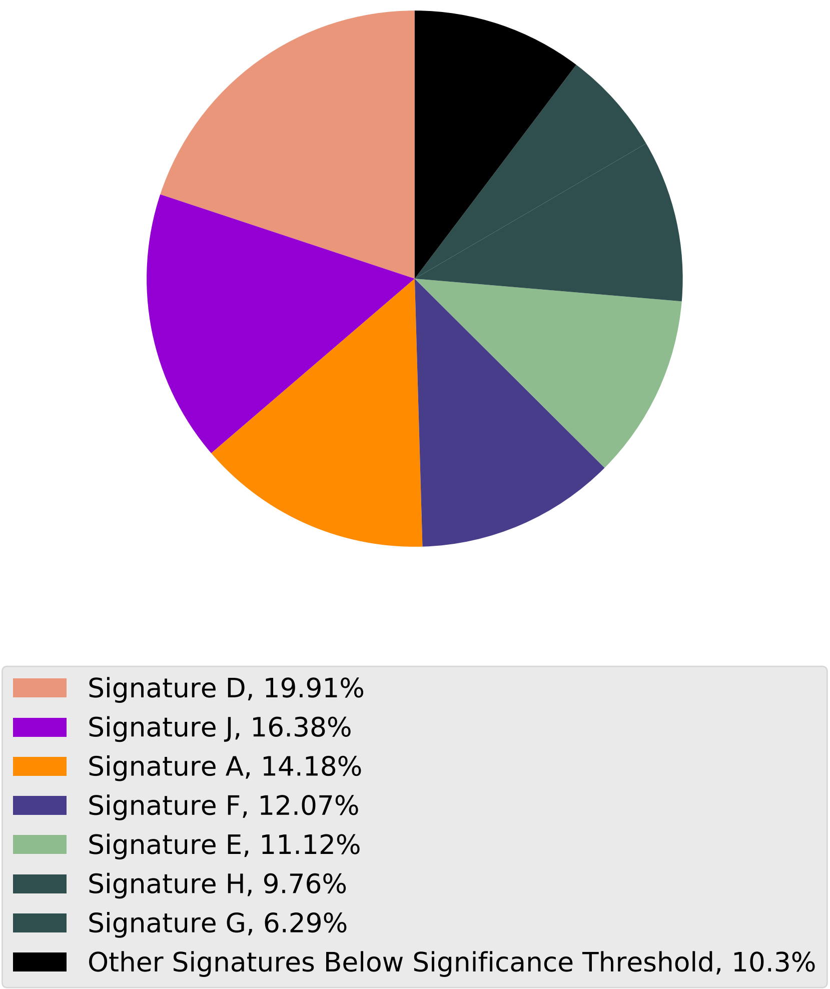

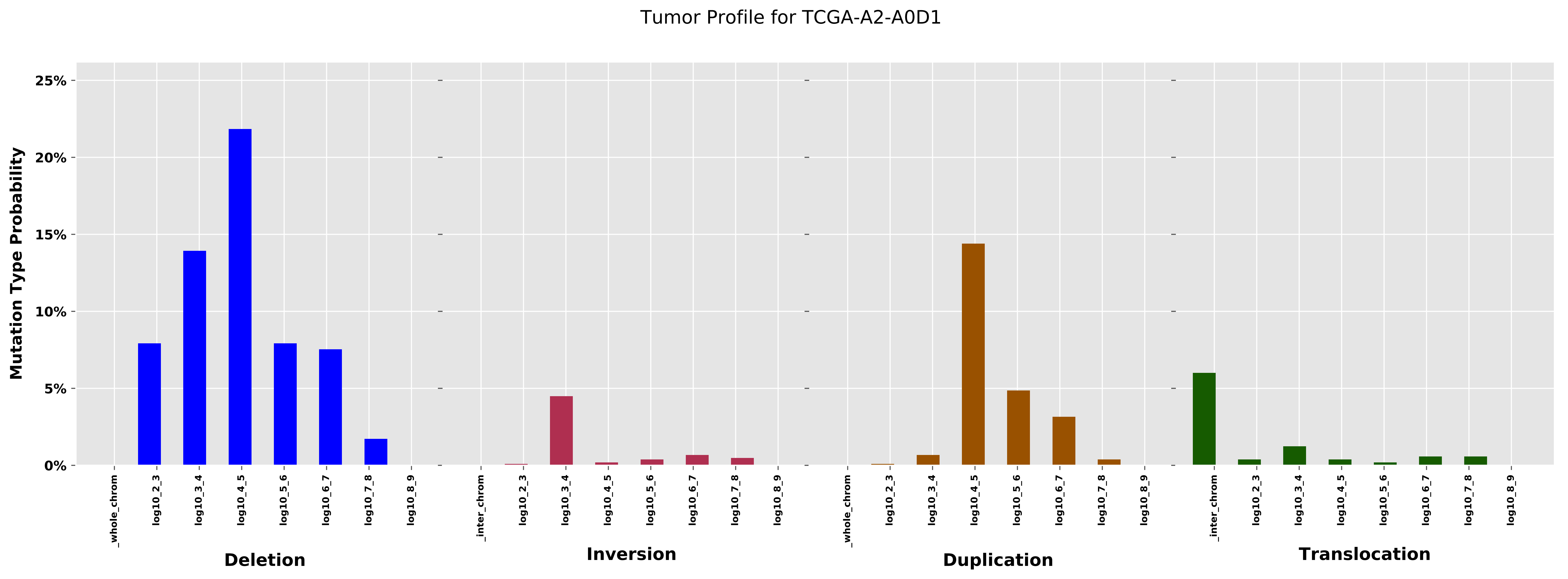

Tumor Profile for TCGA-A2-A0D1
Mutation Type Probability
25%
20%
15%
10%
5%
0%
_whole_chrom
log10_2_3
log10_3_4
log10_4_5
log10_5_6
log10_6_7
log10_7_8
log10_8_9
_inter_chrom
Deletion
Inversion
Duplication
Translocation

Cancer processes Weights for TCGA-AA-3529

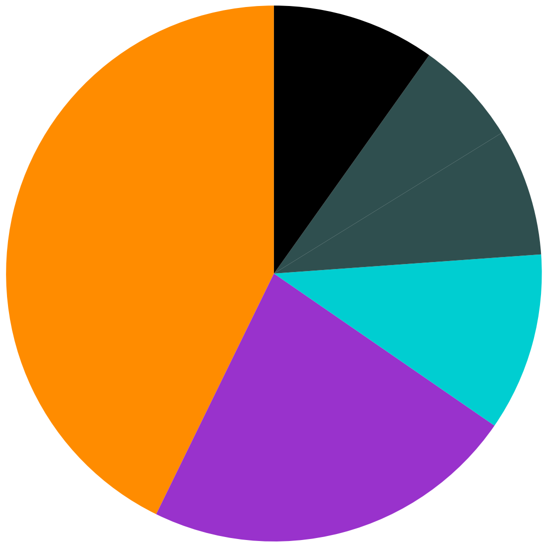

Signature A, 42.76%
Signature B, 22.64%
Signature I, 10.74%
Signature H, 7.66%
Signature G, 6.36%
Other Signatures Below Significance Threshold, 9.84%

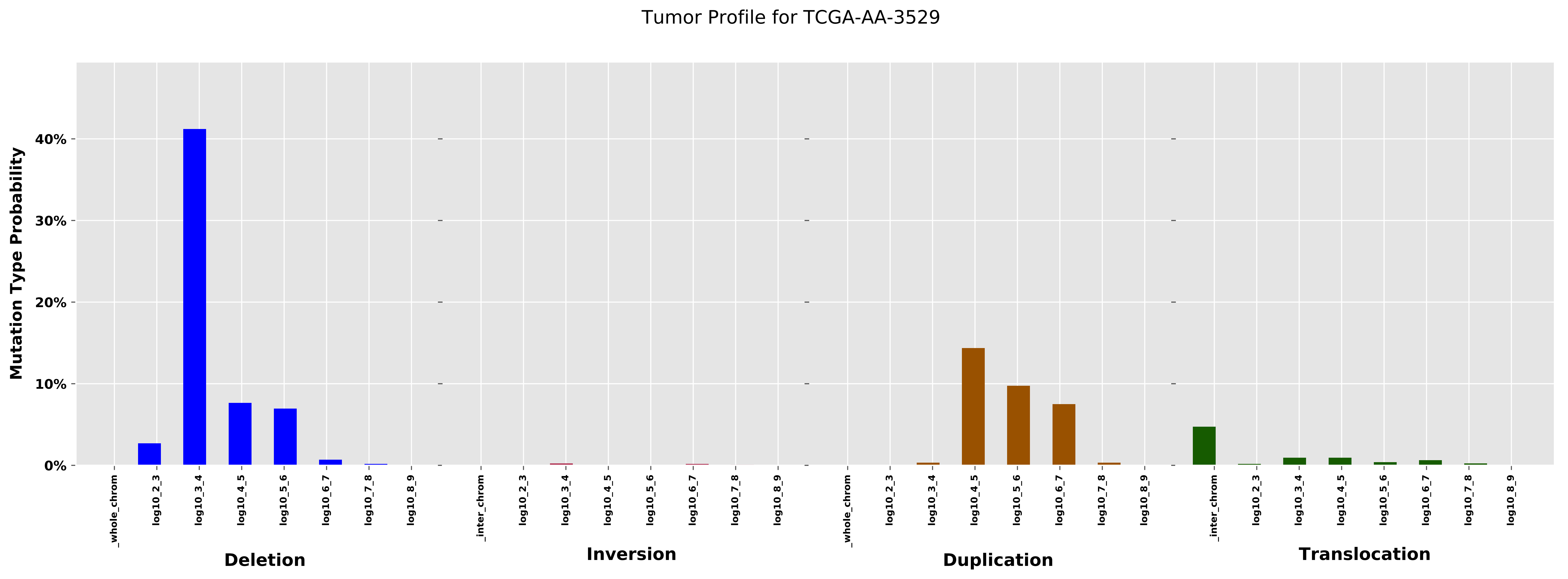

Tumor Profile for TCGA-AA-3529
Mutation Type Probability
0%
10%
20%
30%
40%
_whole_chrom
log10_2_3
log10_3_4
log10_4_5
log10_5_6
log10_6_7
log10_7_8
log10_8_9
_inter_chrom
Deletion
Inversion
Duplication
Translocation

Cancer processes Weights for TCGA-A6-3807

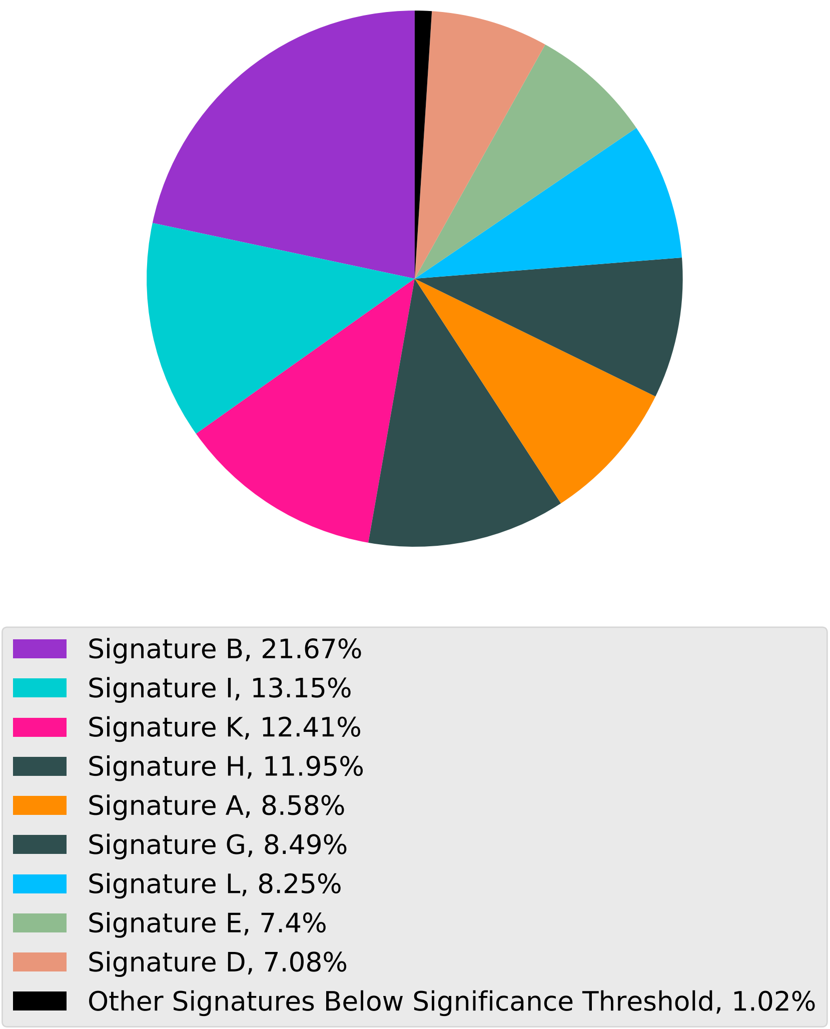

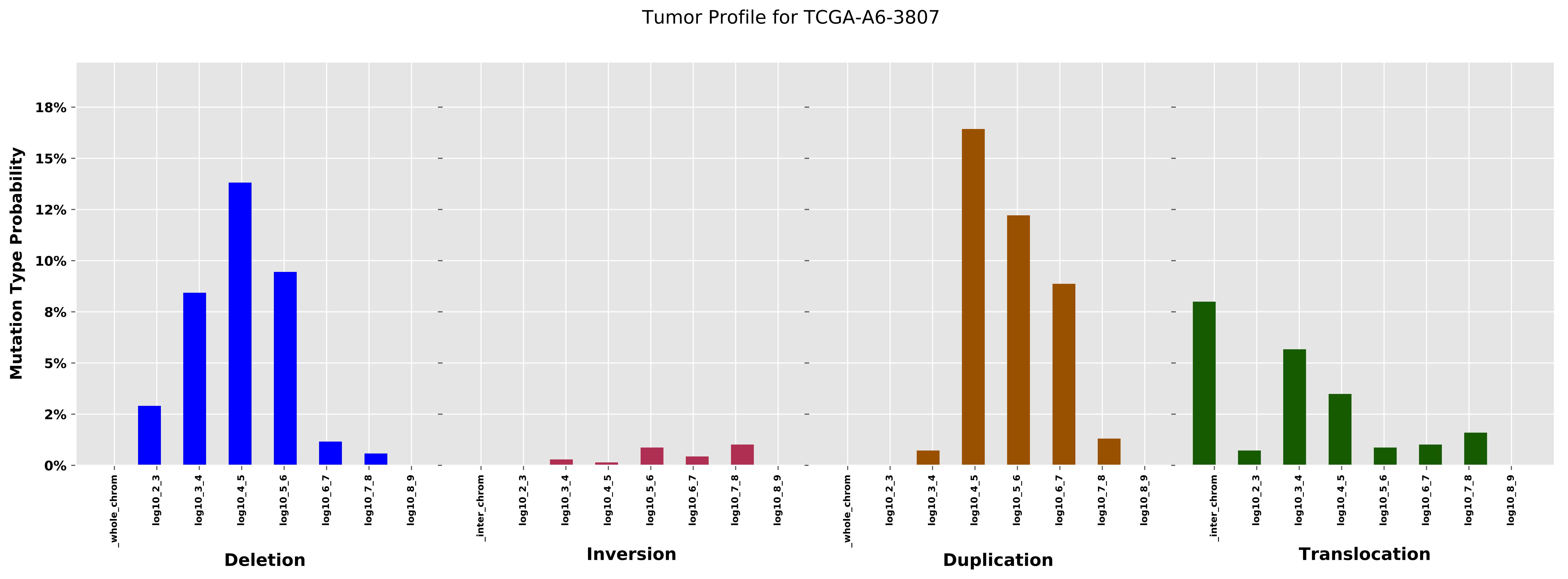

Tumor Profile for TCGA-A6-3807
Mutation Type Probability
0%
2%
5%
8%
10%
12%
15%
18%
_whole_chrom
log10_2_3
log10_3_4
log10_4_5
log10_5_6
log10_6_7
log10_7_8
log10_8_9
_inter_chrom
Deletion
Inversion
Duplication
Translocation

Cancer processes Weights for TCGA-B6-A0IQ

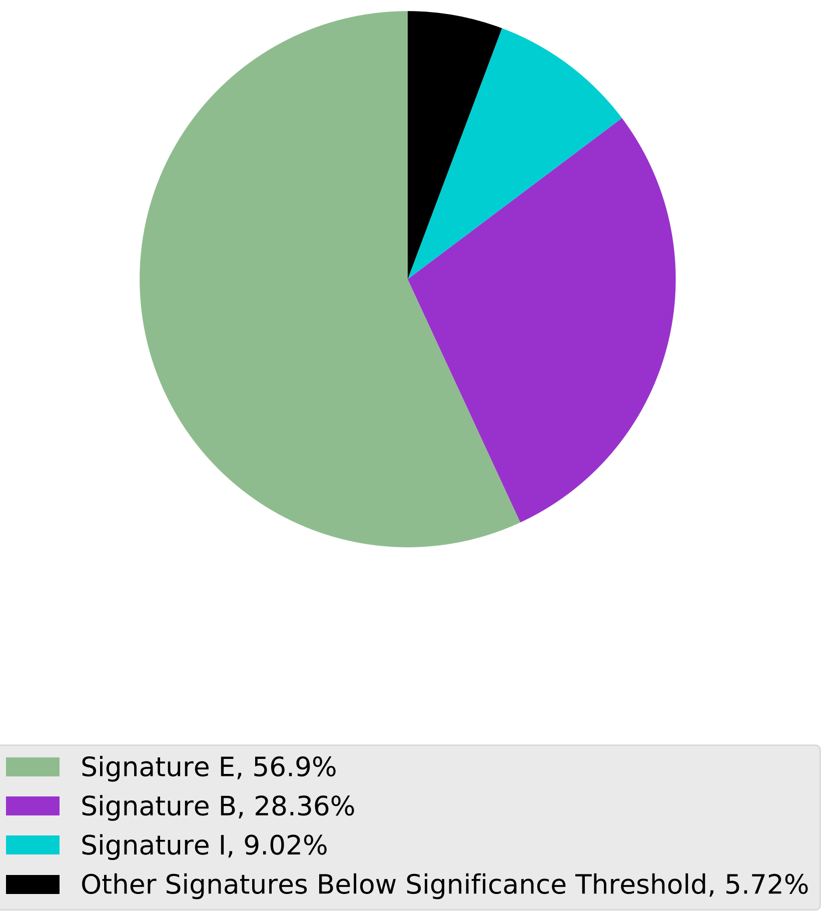

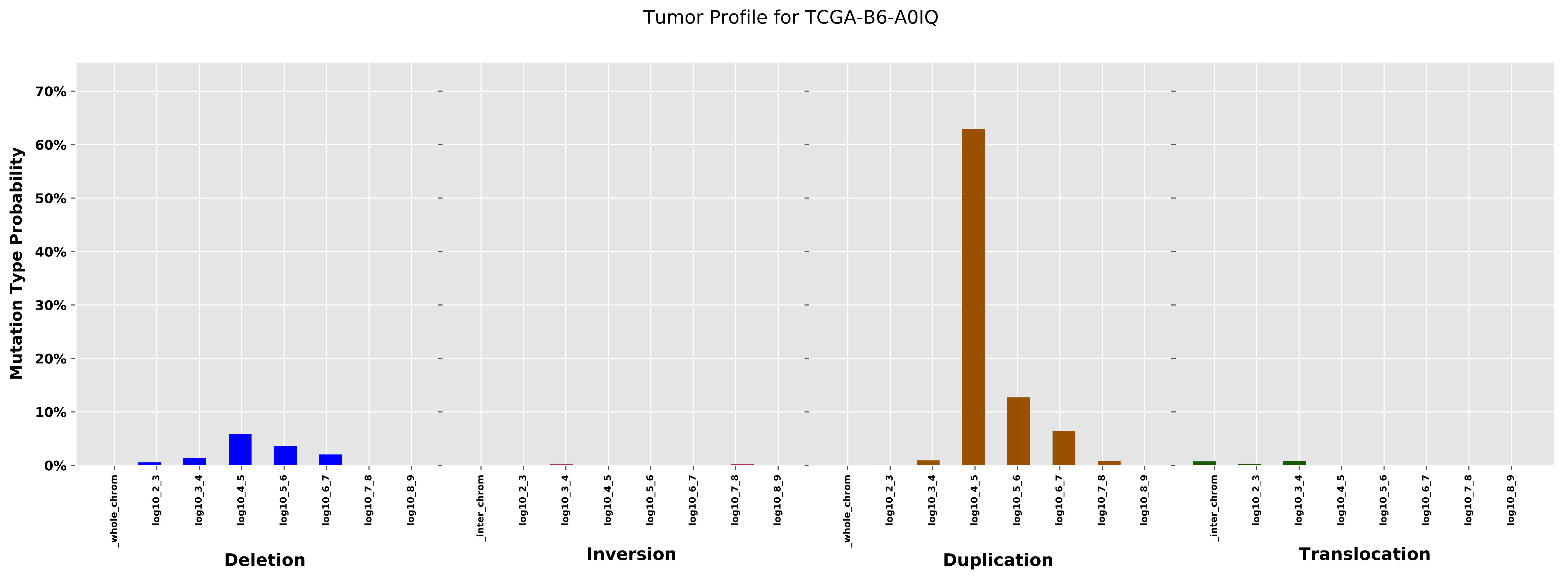

Tumor Profile for TCGA-B6-A0IQ
Mutation Type Probability
70%
60%
50%
40%
30%
20%
10%
0%
_whole_chrom
log10_2_3
log10_3_4
log10_4_5
log10_5_6
log10_6_7
log10_7_8
log10_8_9
_inter_chrom
Deletion
Inversion
Duplication
Translocation

Cancer processes Weights for TCGA-A2-A0YG

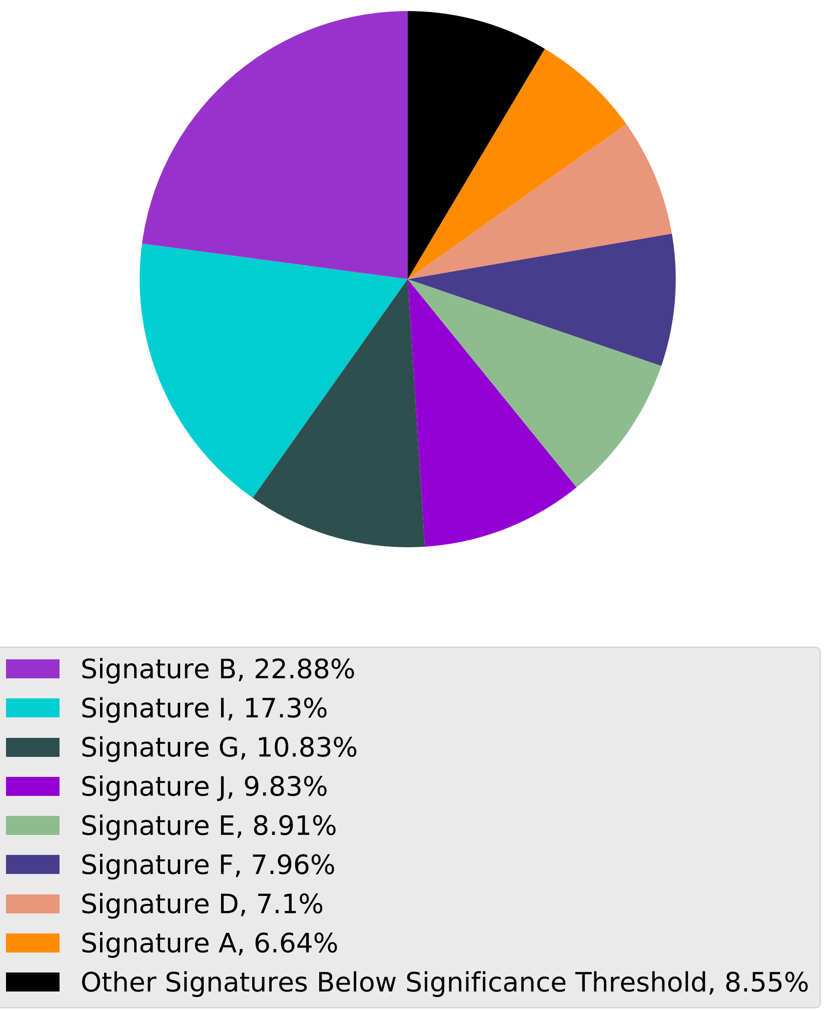

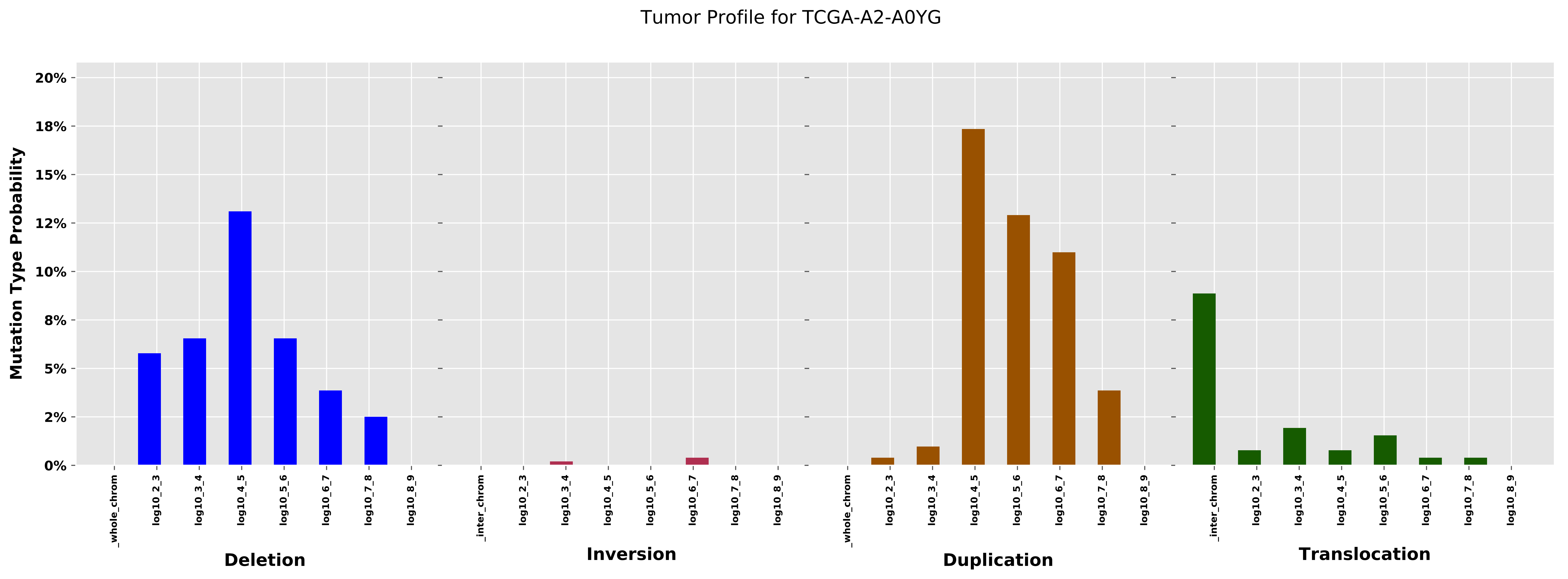

Tumor Profile for TCGA-A2-A0YG
Mutation Type Probability
20%
18%
15%
12%
10%
8%
5%
2%
0%
_whole_chrom
log10_2_3
log10_3_4
log10_4_5
log10_5_6
log10_6_7
log10_7_8
log10_8_9
_inter_chrom
Deletion
Inversion
Duplication
Translocation

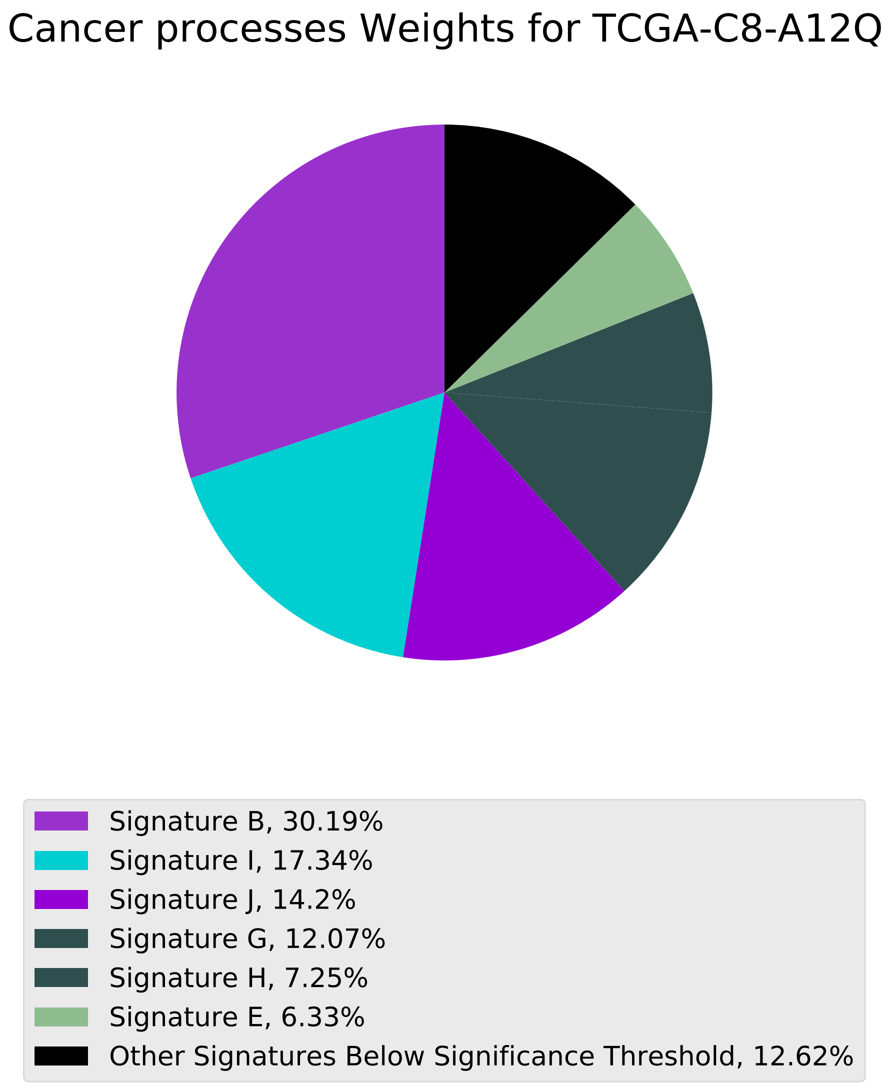
Cancer processes Weights for TCGA-C8-A12Q
Signature B, 30.19%
Signature I, 17.34%
Signature J, 14.2%
Signature G, 12.07%
Signature H, 7.25%
Signature E, 6.33%
Other Signatures Below Significance Threshold, 12.62%

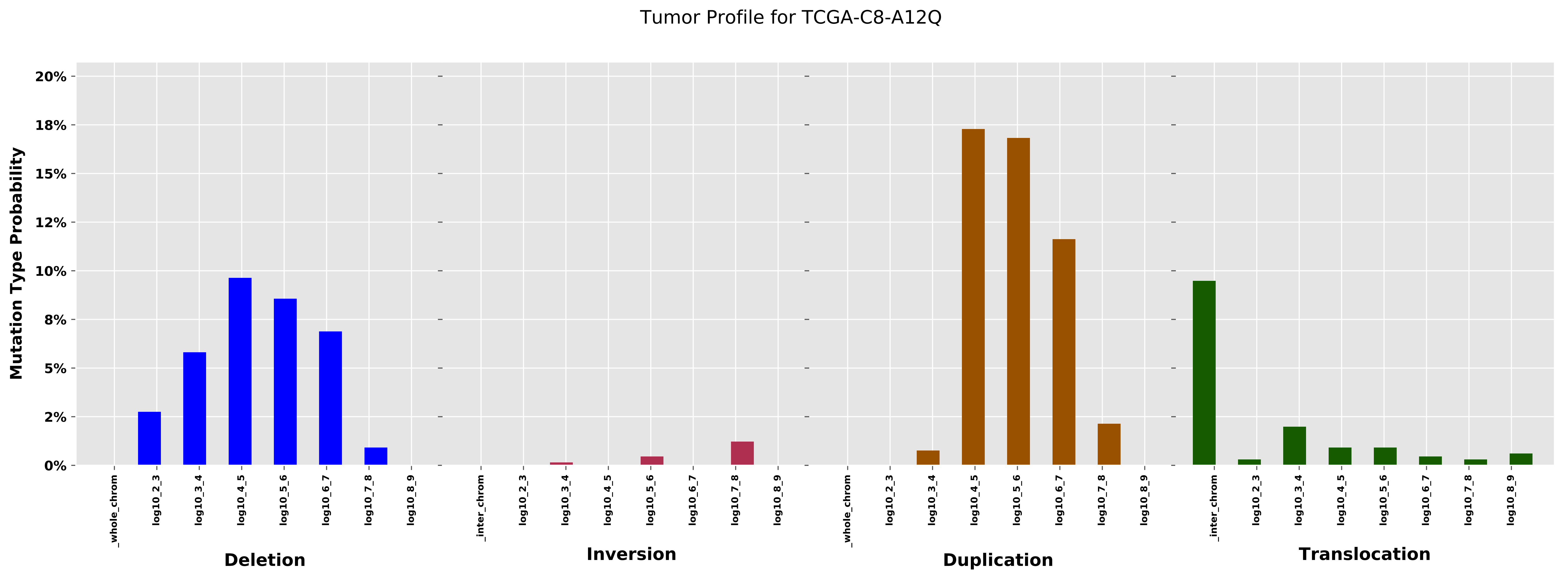

Tumor Profile for TCGA-C8-A12Q
Mutation Type Probability
0%
2%
5%
8%
10%
12%
15%
18%
20%
_whole_chrom
log10_2_3
log10_3_4
log10_4_5
log10_5_6
log10_6_7
log10_7_8
log10_8_9
_inter_chrom
Deletion
Inversion
Duplication
Translocation

Cancer processes Weights for TCGA-BH-A0BW

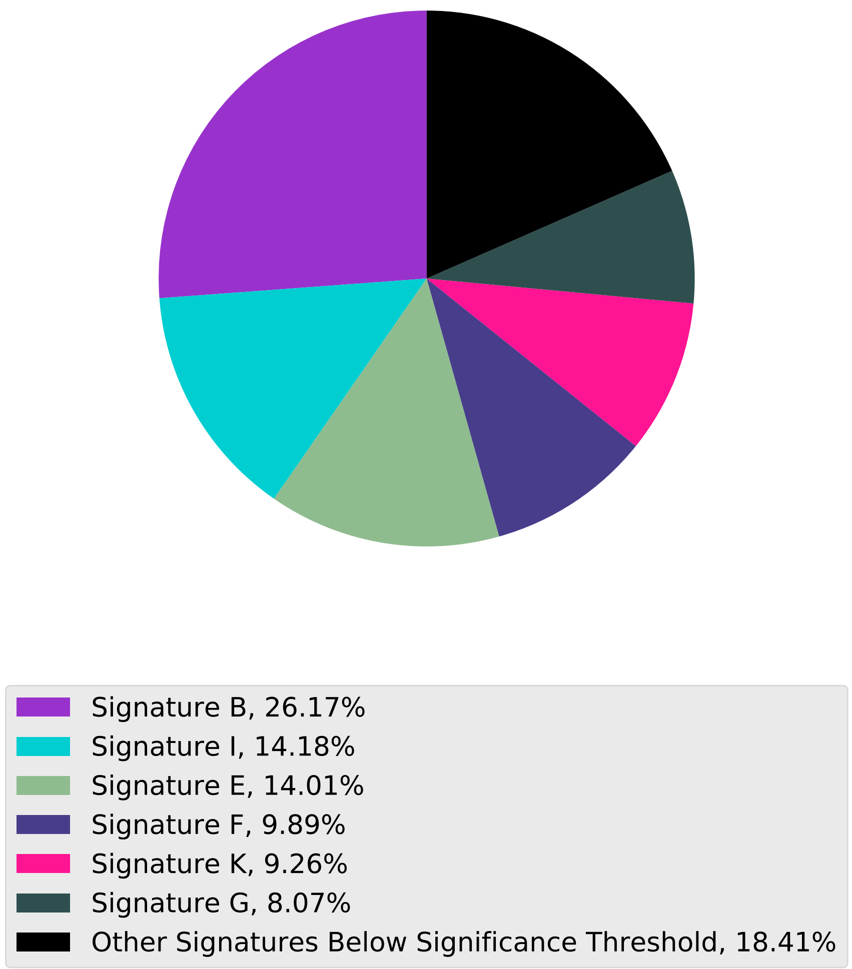

Tumor Profile for TCGA-BH-A0BW

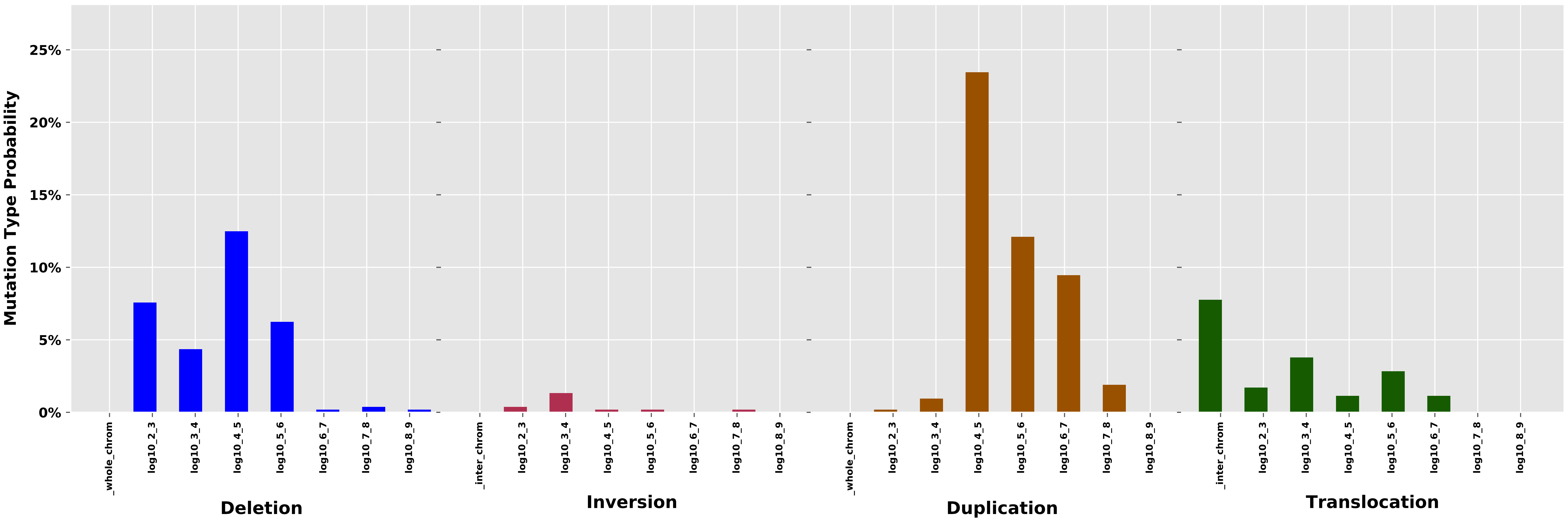

Cancer processes Weights for TCGA-A2-A0EY

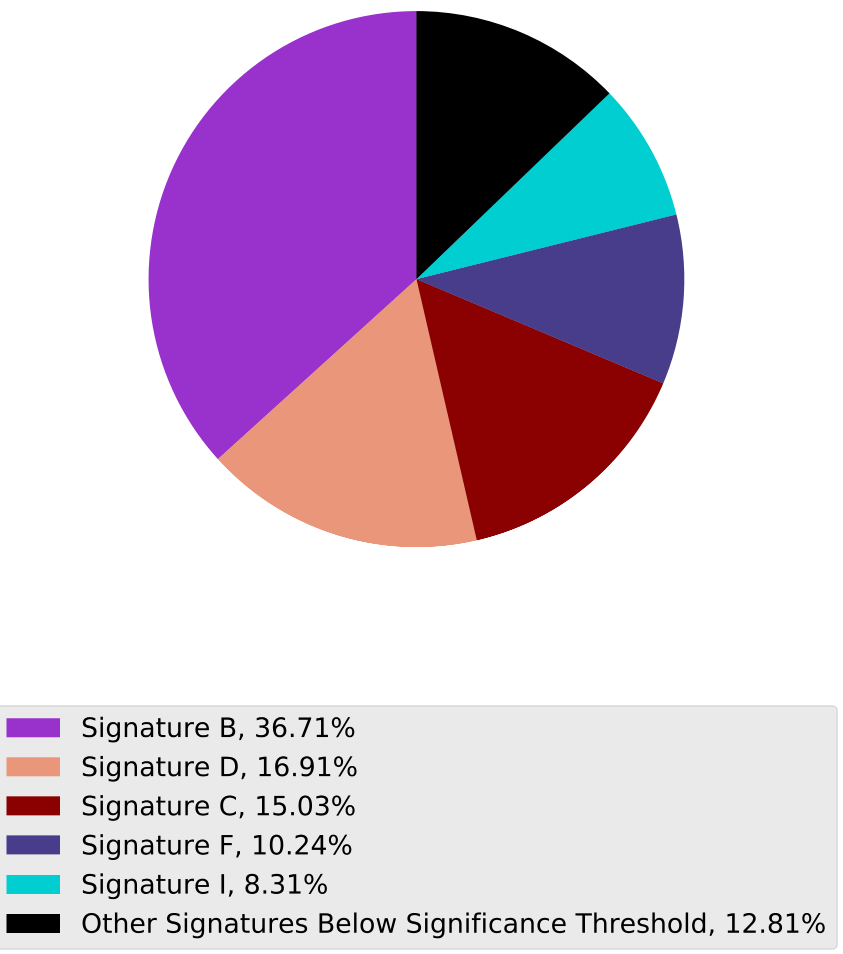

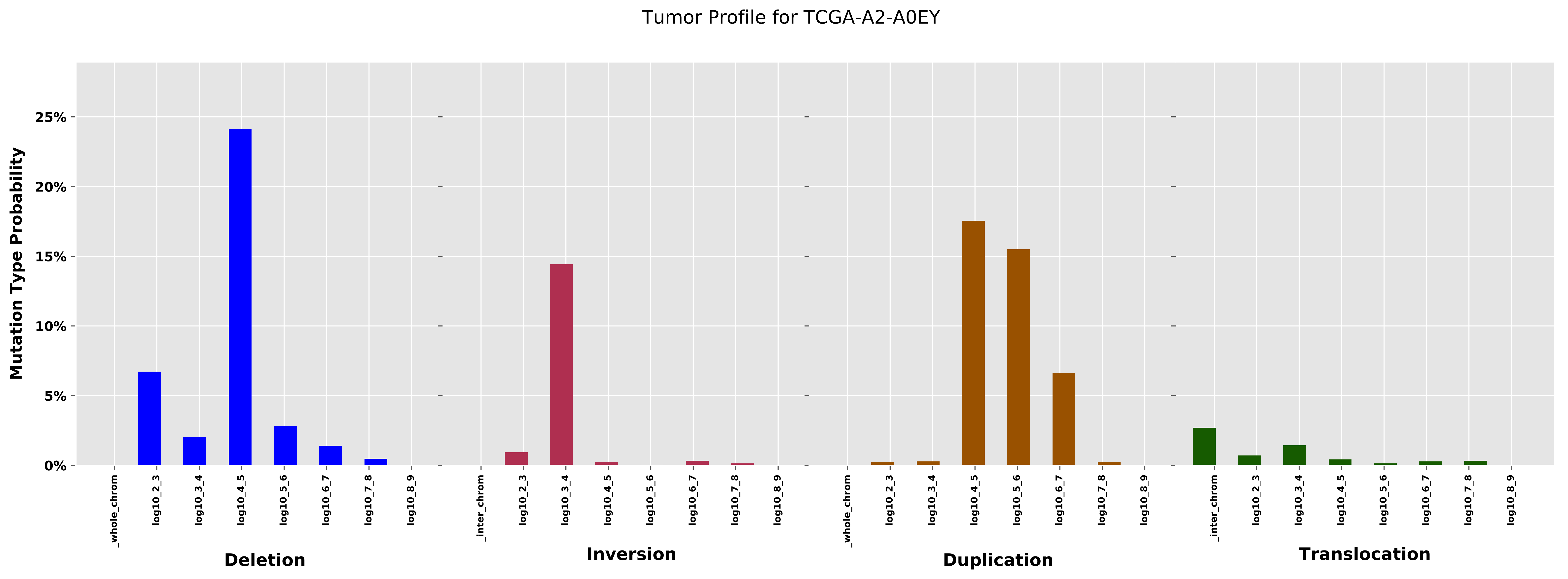

Tumor Profile for TCGA-A2-A0EY
Mutation Type Probability
25%
20%
15%
10%
5%
0%
_whole_chrom
log10_2_3
log10_3_4
log10_4_5
log10_5_6
log10_6_7
log10_7_8
log10_8_9
_inter_chrom
Deletion
Inversion
Duplication
Translocation

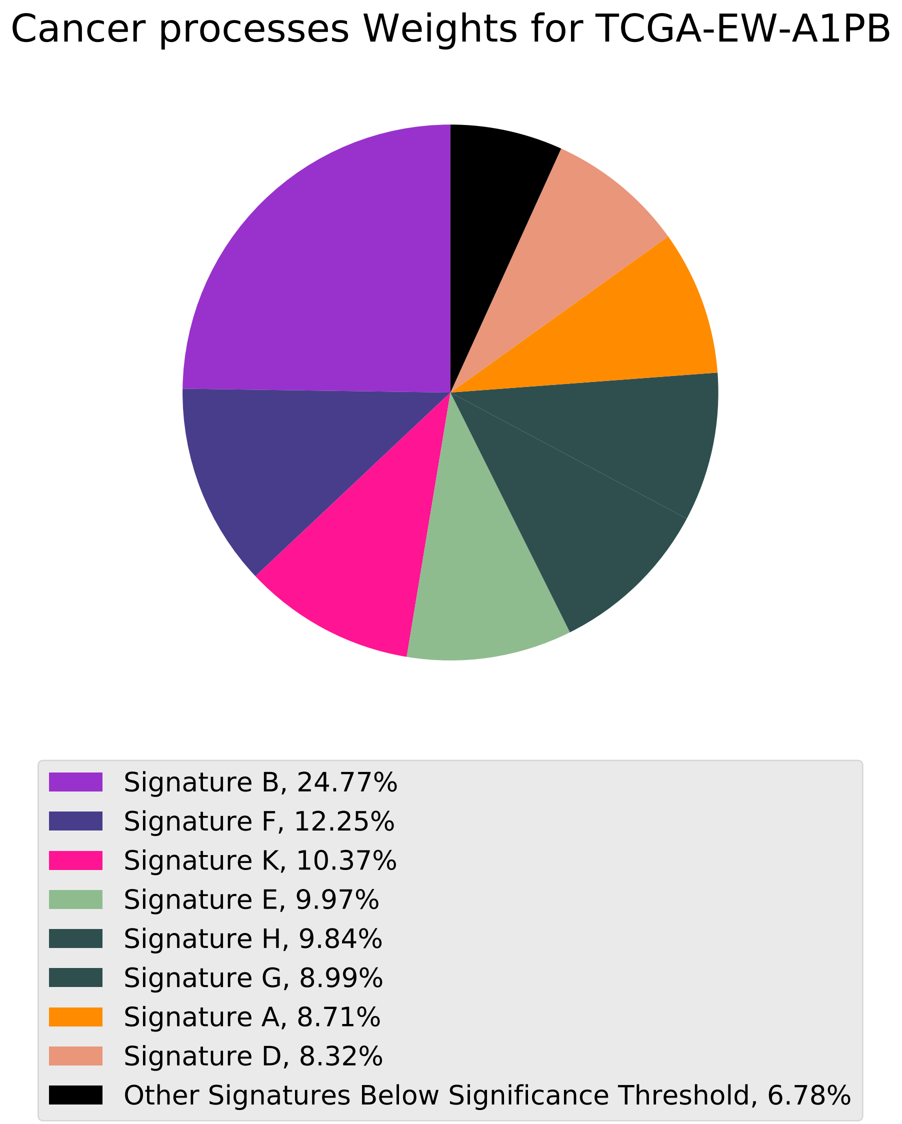
Cancer processes Weights for TCGA-EW-A1PB
Signature B, 24.77%
Signature F, 12.25%
Signature K, 10.37%
Signature E, 9.97%
Signature H, 9.84%
Signature G, 8.99%
Signature A, 8.71%
Signature D, 8.32%
Other Signatures Below Significance Threshold, 6.78%

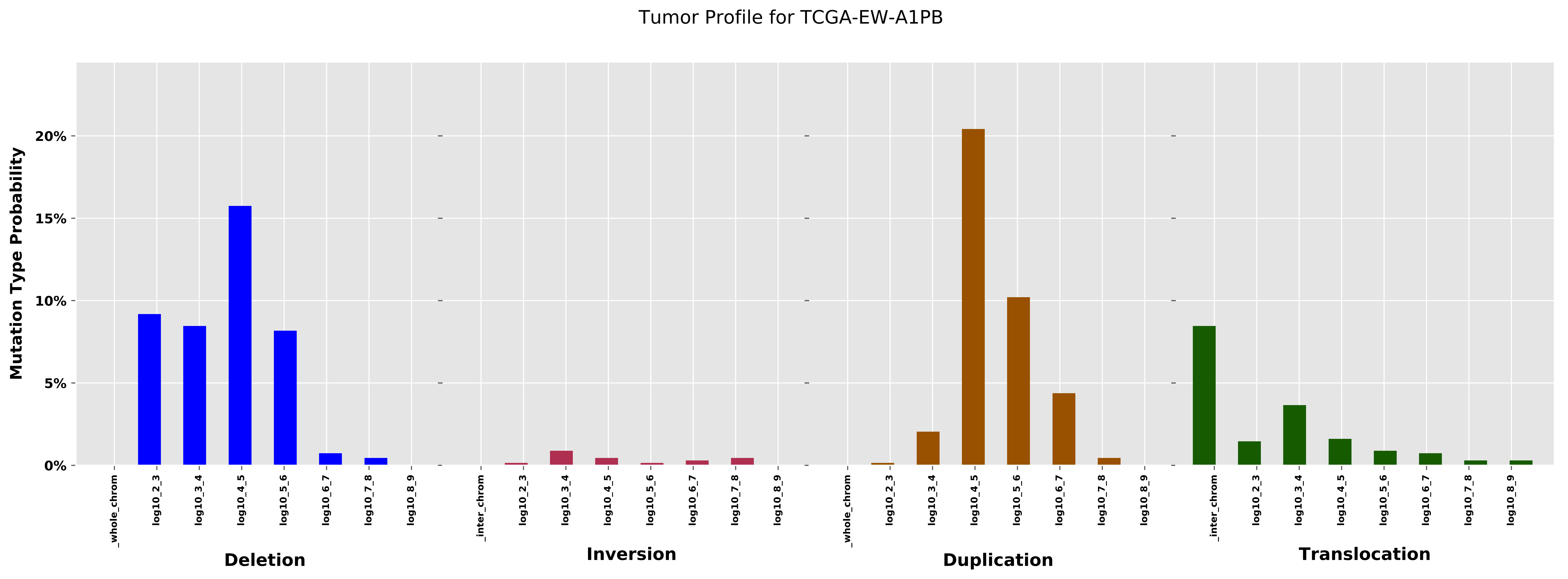
Tumor Profile for TCGA-EW-A1PB
Mutation Type Probability
20%
15%
10%
5%
0%
_whole_chrom
log10_2_3
log10_3_4
log10_4_5
log10_5_6
log10_6_7
log10_7_8
log10_8_9
_inter_chrom
Deletion
Inversion
Duplication
Translocation

Cancer processes Weights for TCGA-AD-6964

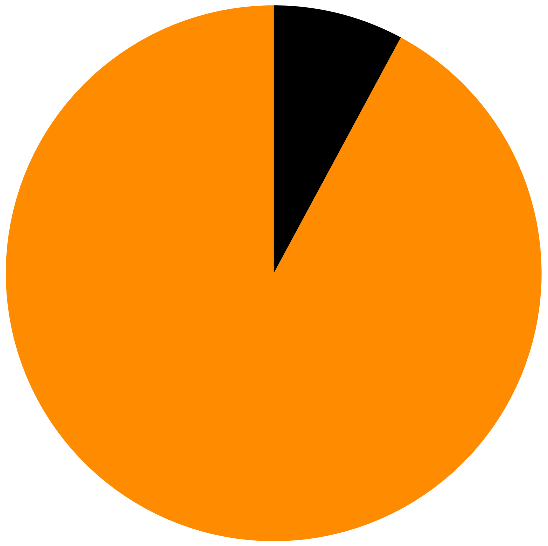

Signature A, 92.13%
Other Signatures Below Significance Threshold, 7.87%

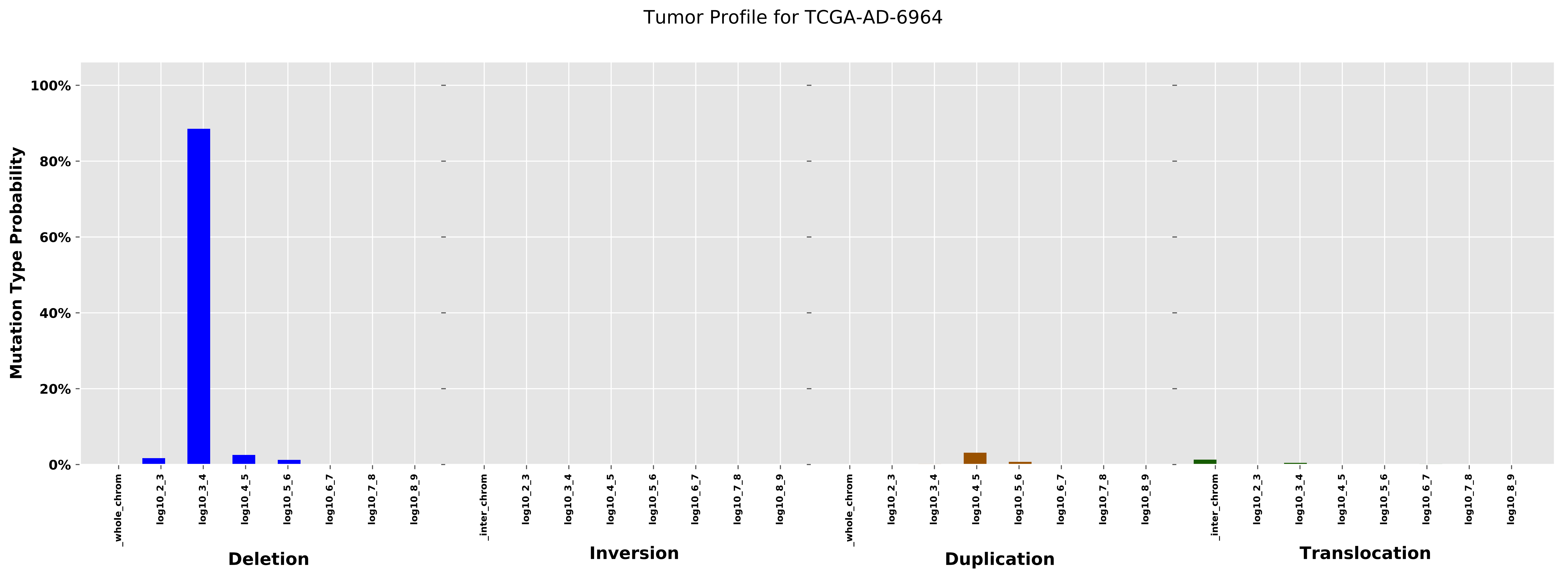

Tumor Profile for TCGA-AD-6964
Mutation Type Probability
100%
80%
60%
40%
20%
0%
_whole_chrom
log10_2_3
log10_3_4
log10_4_5
log10_5_6
log10_6_7
log10_7_8
log10_8_9
_inter_chrom
Deletion
Inversion
Duplication
Translocation

Cancer processes Weights for TCGA-AG-3896

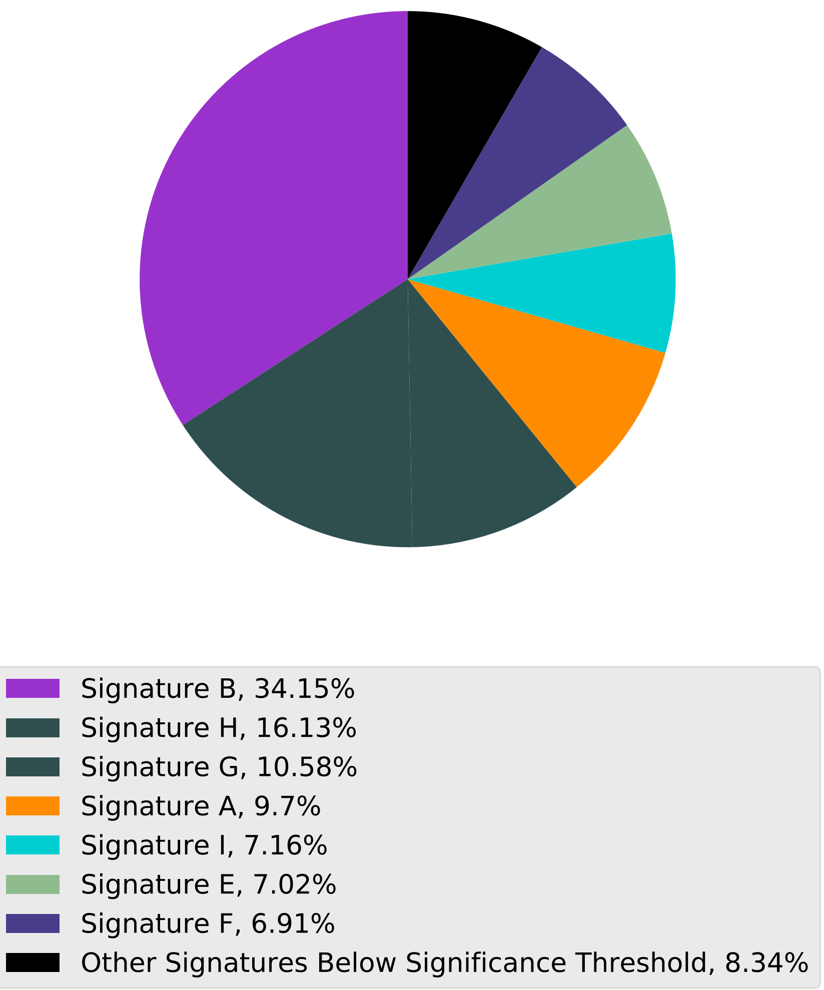

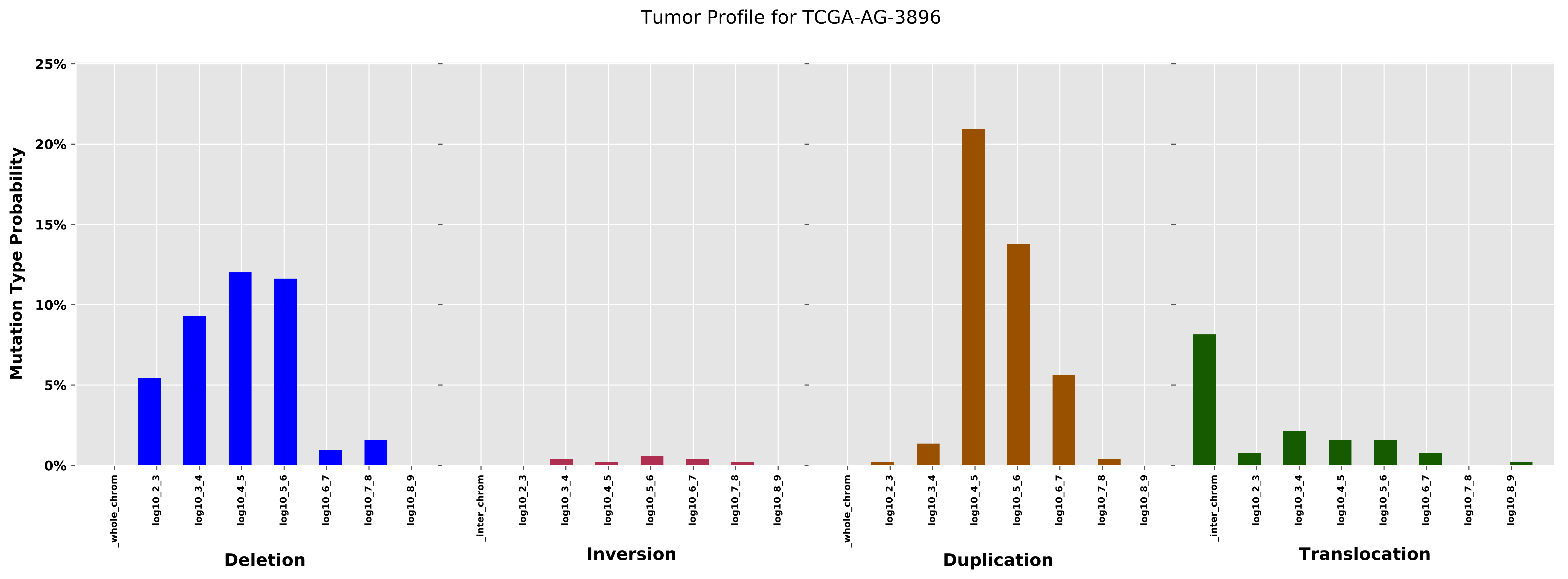
Tumor Profile for TCGA-AG-3896
Mutation Type Probability
25%
20%
15%
10%
5%
0%
_whole_chrom
log10_2_3
log10_3_4
log10_4_5
log10_5_6
log10_6_7
log10_7_8
log10_8_9
_inter_chrom
Deletion
Inversion
Duplication
Translocation

Cancer processes Weights for TCGA-E2-A15K

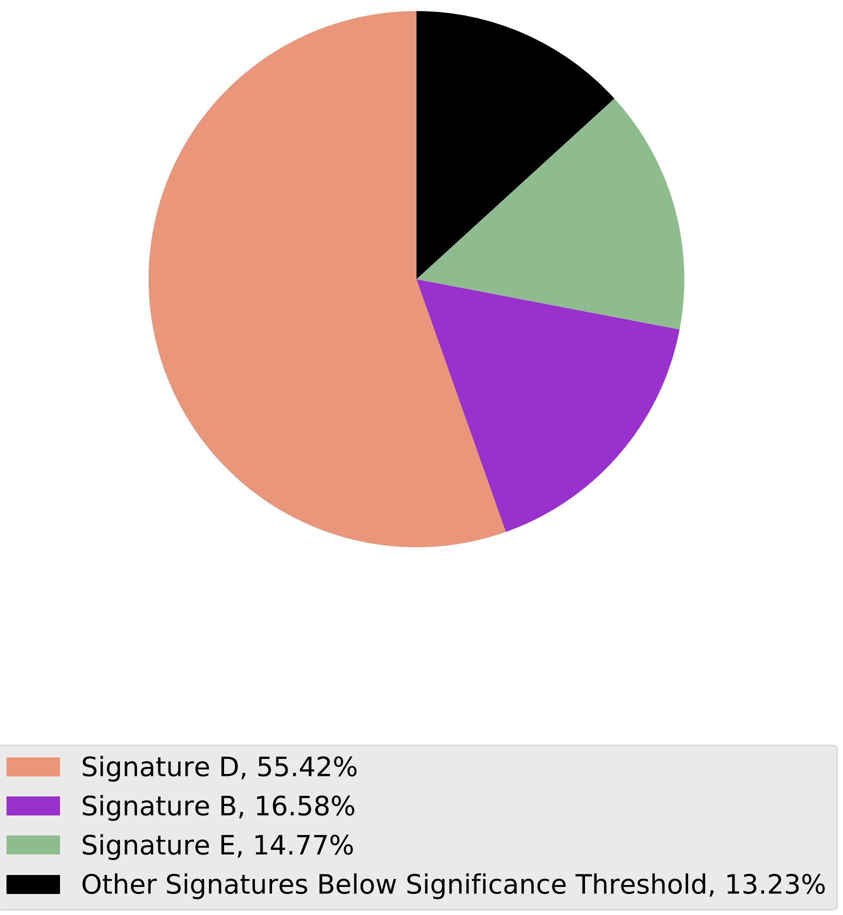

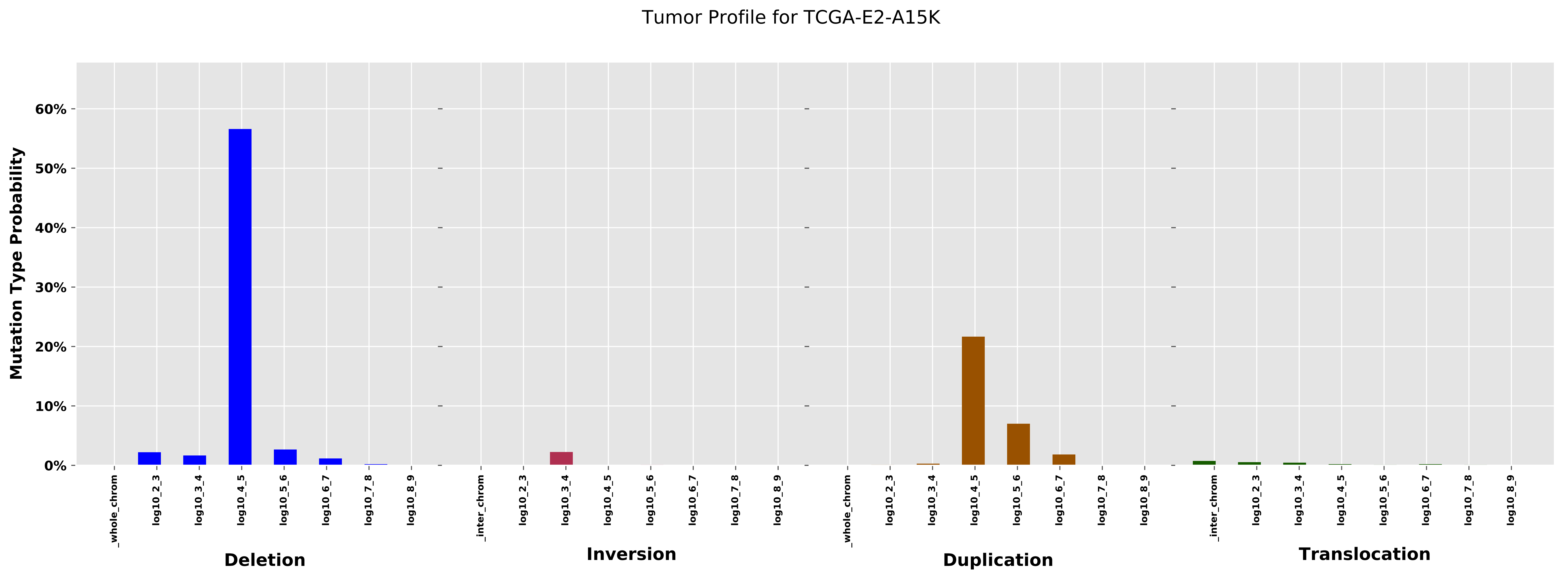

Tumor Profile for TCGA-E2-A15K
Mutation Type Probability
0%
10%
20%
30%
40%
50%
60%
_whole_chrom
log10_2_3
log10_3_4
log10_4_5
log10_5_6
log10_6_7
log10_7_8
log10_8_9
_inter_chrom
Deletion
Inversion
Duplication
Translocation

Cancer processes Weights for TCGA-B6-A0RE

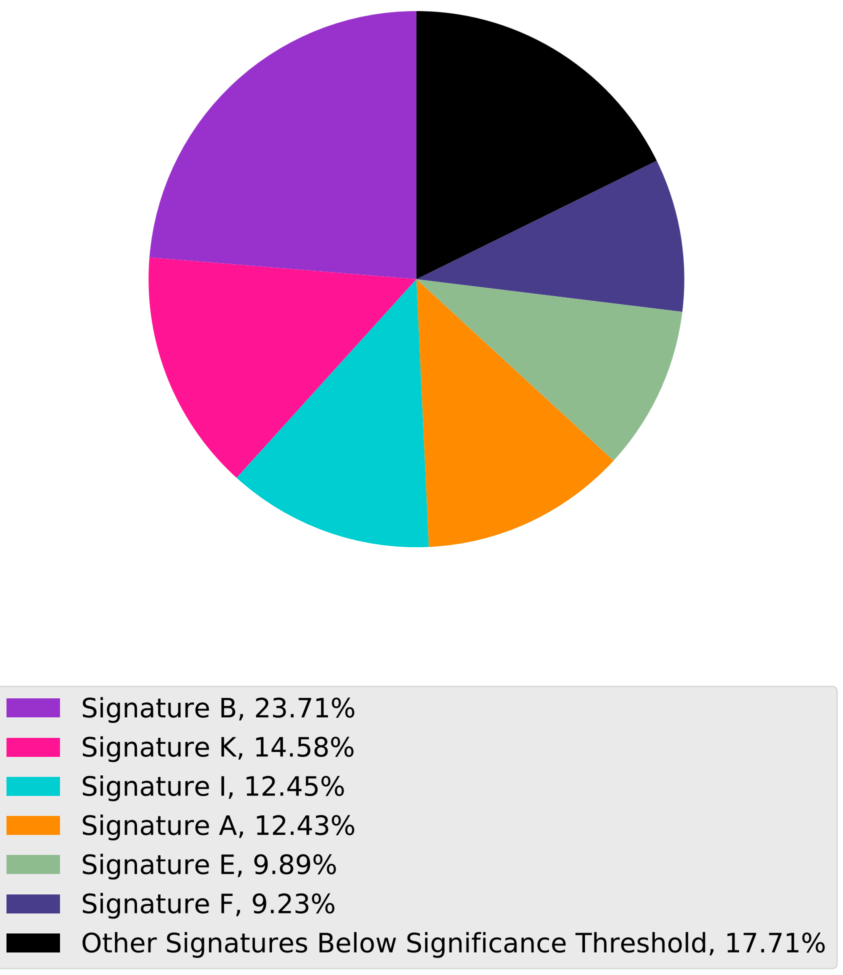

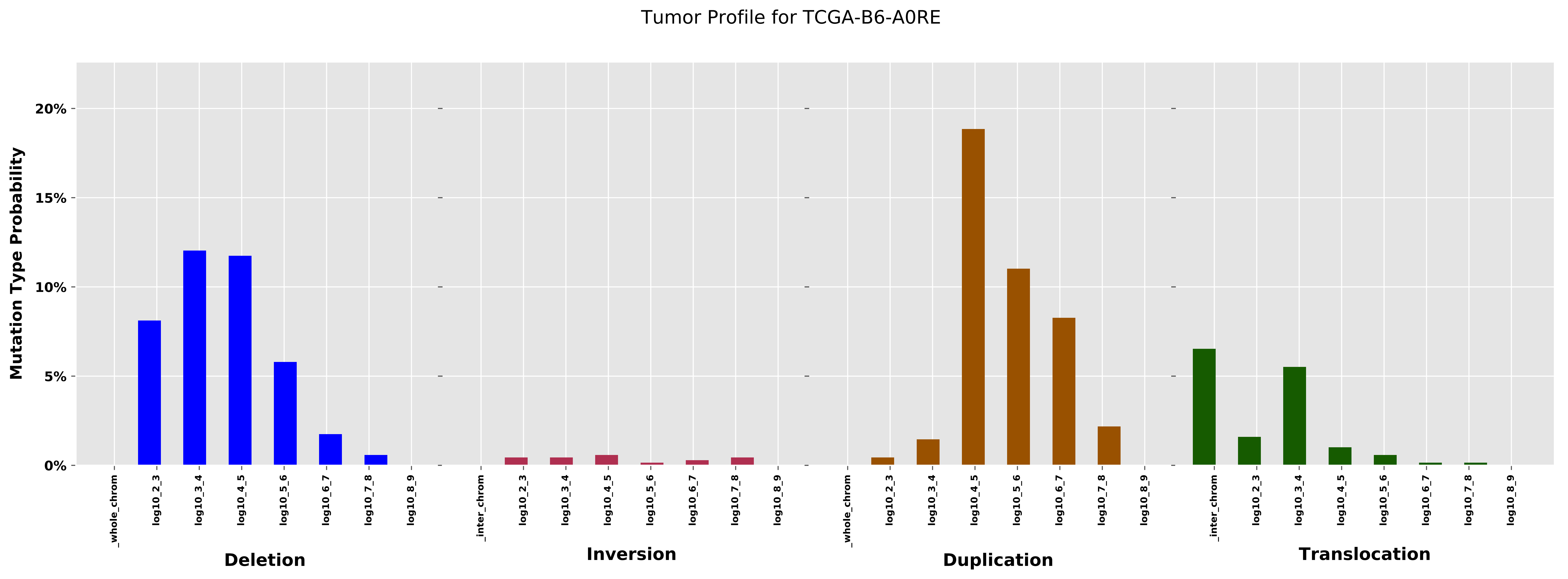

Tumor Profile for TCGA-B6-A0RE
Mutation Type Probability
20%
15%
10%
5%
0%
_whole_chrom
log10_2_3
log10_3_4
log10_4_5
log10_5_6
log10_6_7
log10_7_8
log10_8_9
Deletion
_inter_chrom
Inversion
Duplication
Translocation

Cancer processes Weights for TCGA-A8-A08S

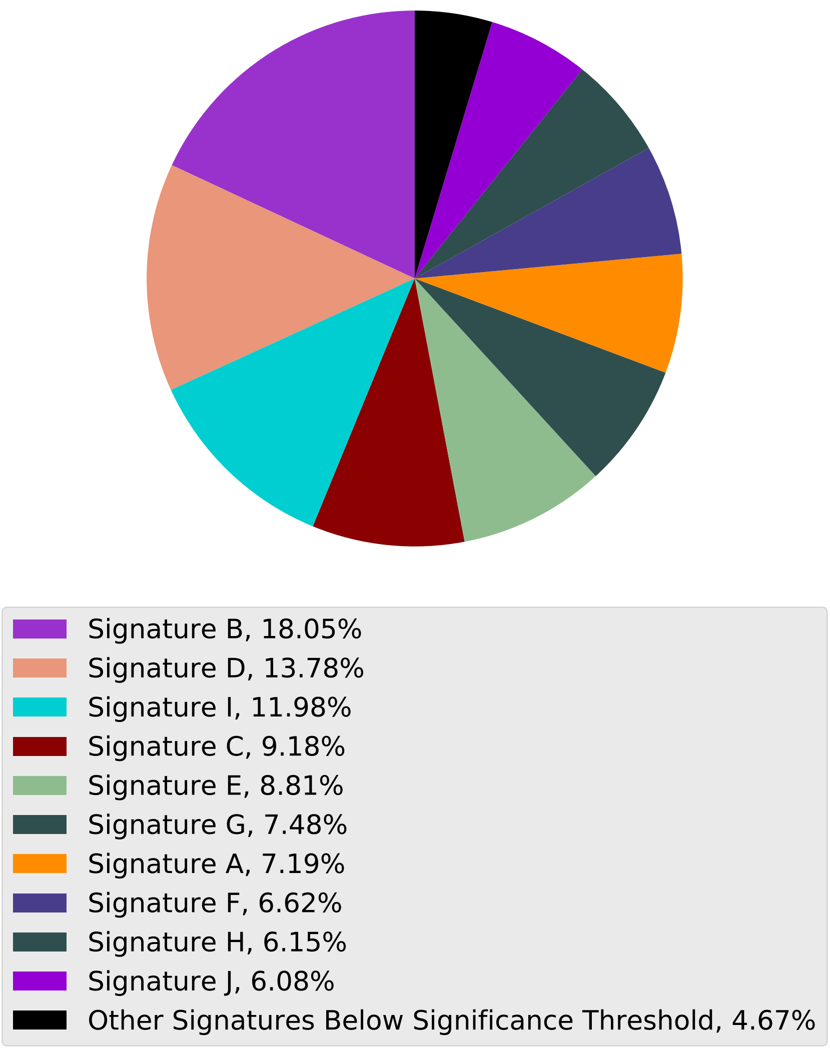

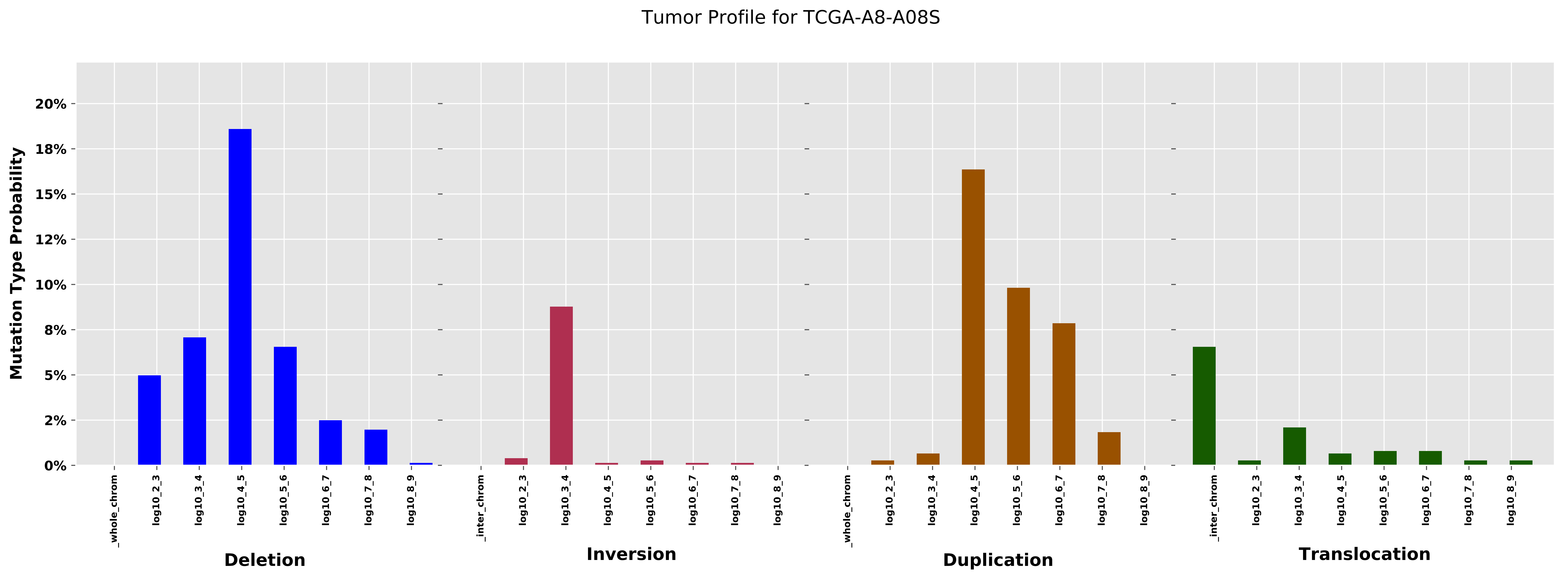

Tumor Profile for TCGA-A8-A08S
Mutation Type Probability
20%
18%
15%
12%
10%
8%
5%
2%
0%
_whole_chrom
log10_2_3
log10_3_4
log10_4_5
log10_5_6
log10_6_7
log10_7_8
log10_8_9
_inter_chrom
Deletion
Inversion
Duplication
Translocation

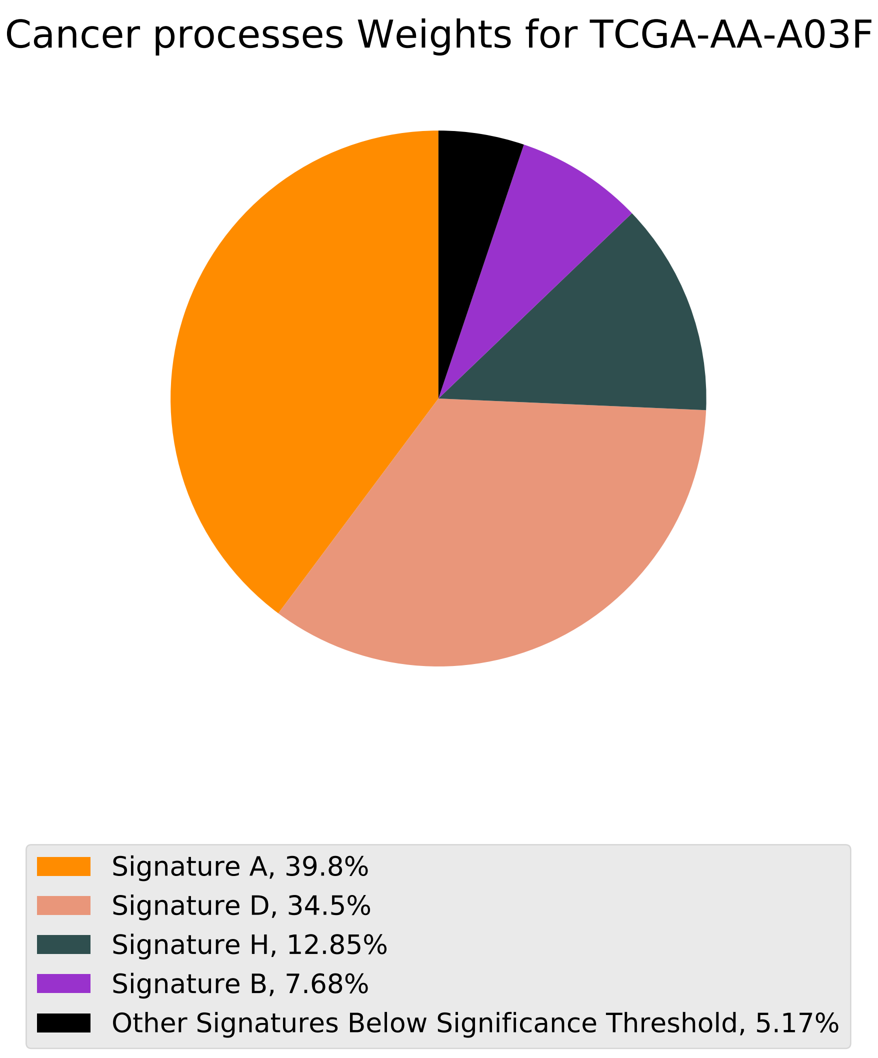
Cancer processes Weights for TCGA-AA-A03F
Signature A, 39.8%
Signature D, 34.5%
Signature H, 12.85%
Signature B, 7.68%
Other Signatures Below Significance Threshold, 5.17%

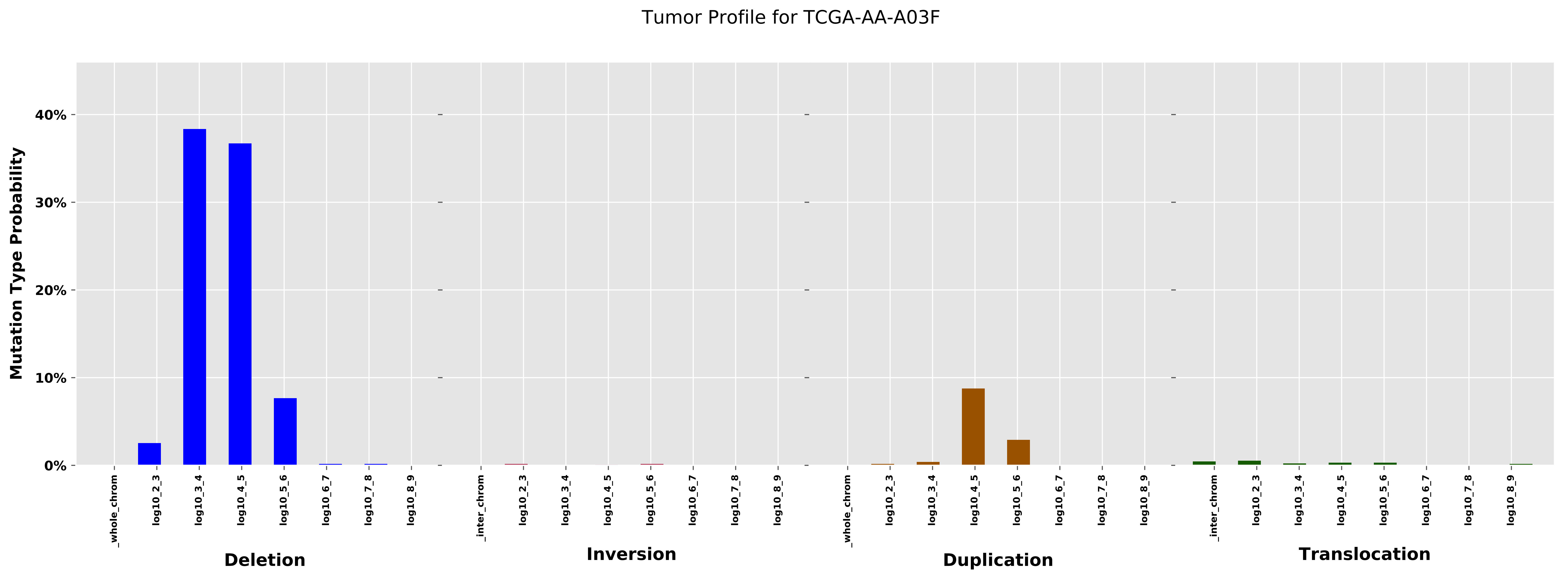

Tumor Profile for TCGA-AA-A03F
Mutation Type Probability
40%
30%
20%
10%
0%
_whole_chrom
log10_2_3
log10_3_4
log10_4_5
log10_5_6
log10_6_7
log10_7_8
log10_8_9
_inter_chrom
Deletion
Inversion
Duplication
Translocation

Cancer processes Weights for TCGA-EW-A1PH

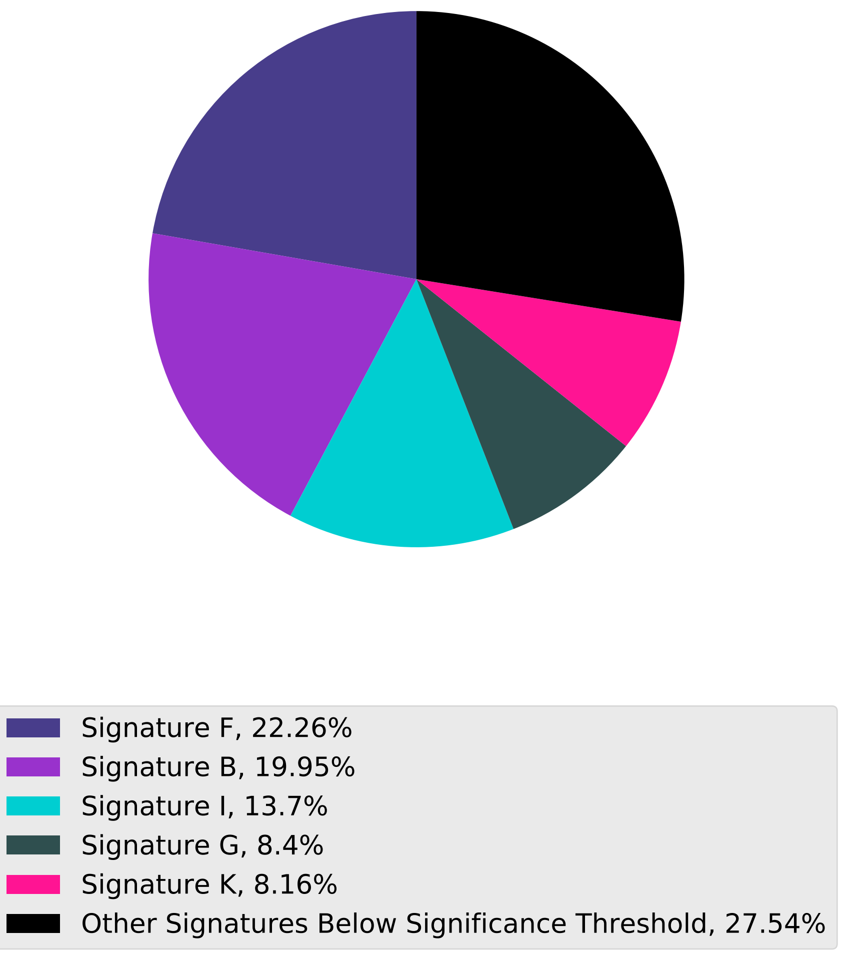

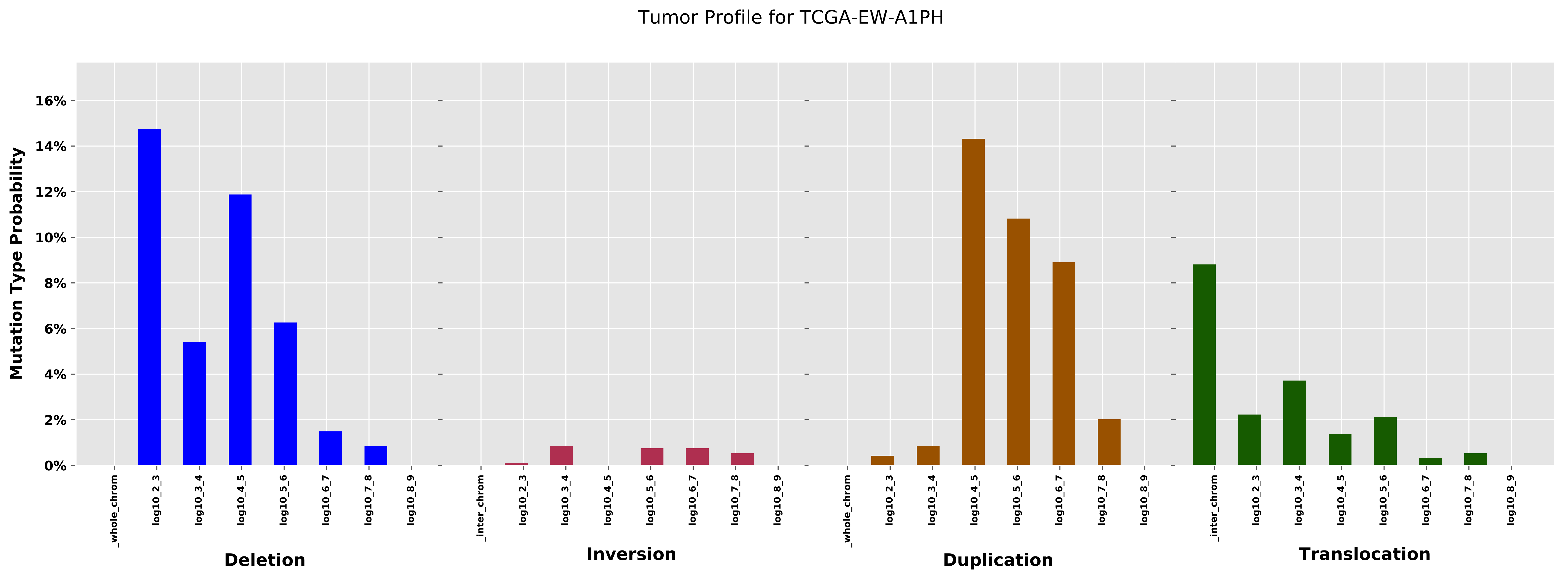
Tumor Profile for TCGA-EW-A1PH
Mutation Type Probability
16%
14%
12%
10%
8%
6%
4%
2%
0%
_whole_chrom
log10_2_3
log10_3_4
log10_4_5
log10_5_6
log10_6_7
log10_7_8
log10_8_9
_inter_chrom
Deletion
Inversion
Duplication
Translocation

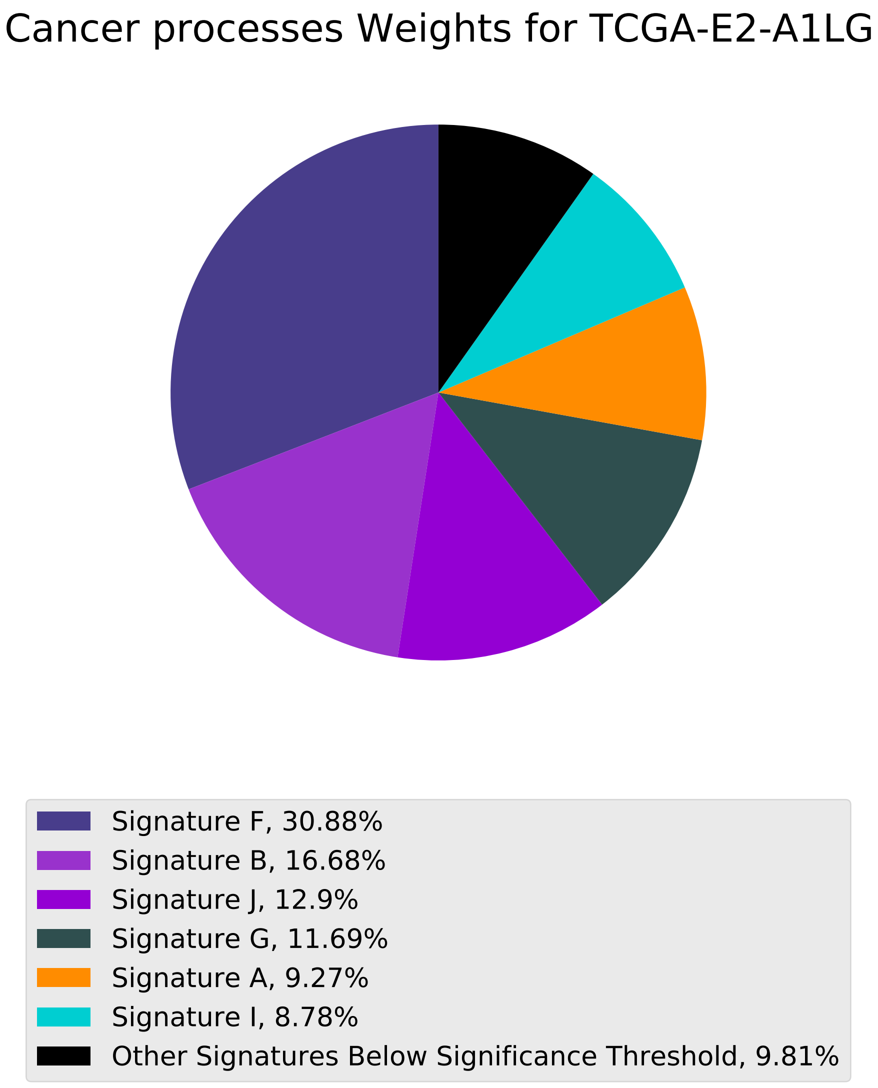
Cancer processes Weights for TCGA-E2-A1LG
Signature F, 30.88%
Signature B, 16.68%
Signature J, 12.9%
Signature G, 11.69%
Signature A, 9.27%
Signature I, 8.78%
Other Signatures Below Significance Threshold, 9.81%

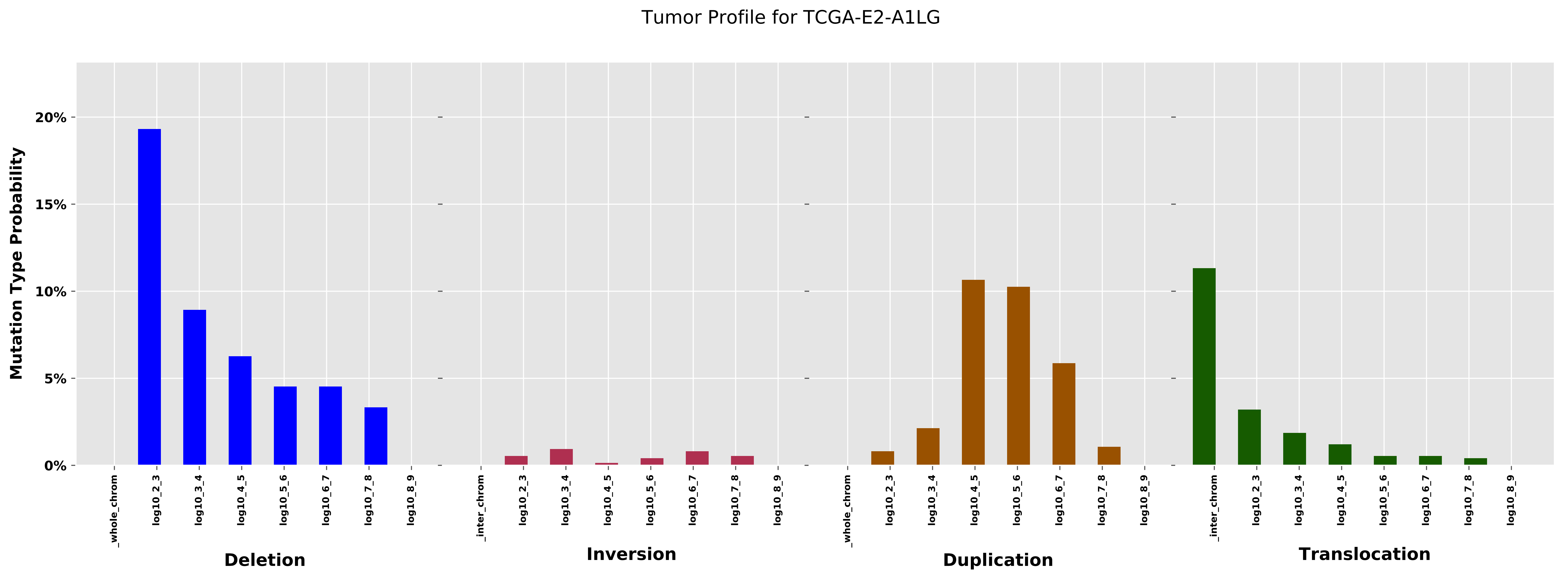

Tumor Profile for TCGA-E2-A1LG
Mutation Type Probability
20%
15%
10%
5%
0%
_whole_chrom
log10_2_3
log10_3_4
log10_4_5
log10_5_6
log10_6_7
log10_7_8
log10_8_9
_inter_chrom
Deletion
Inversion
Duplication
Translocation

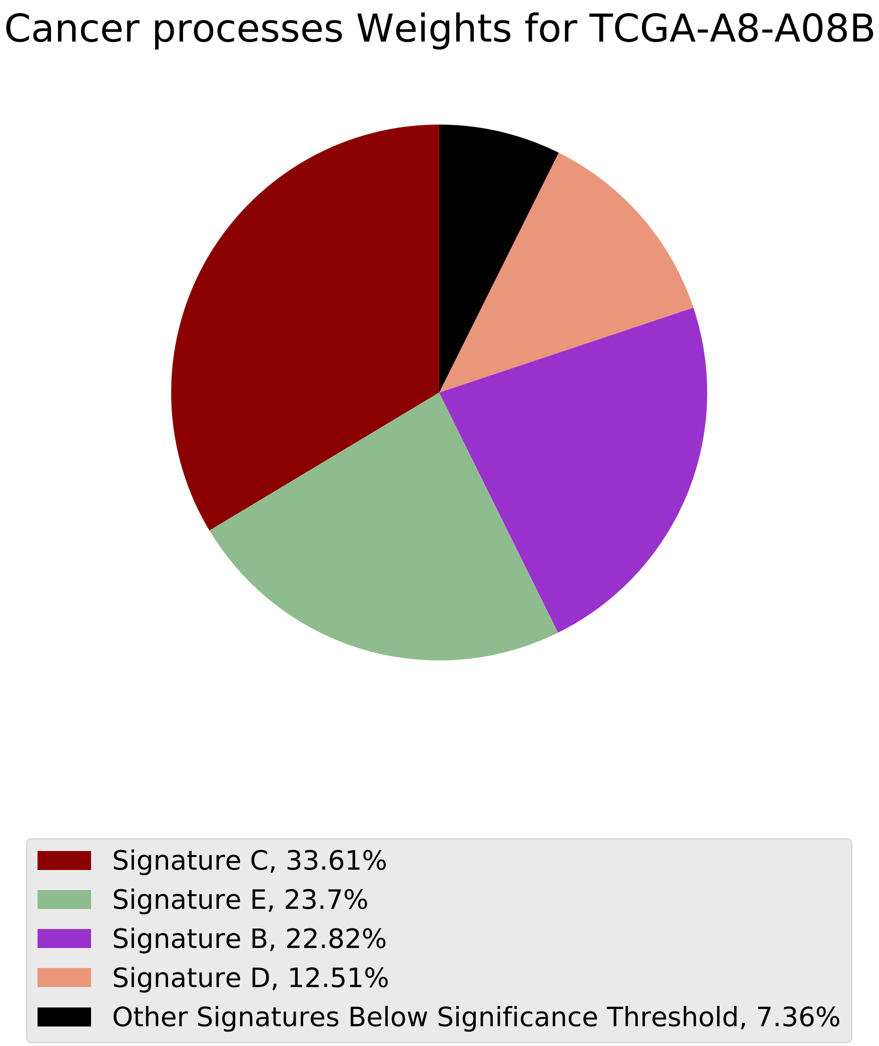
Cancer processes Weights for TCGA-A8-A08B
Signature C, 33.61%
Signature E, 23.7%
Signature B, 22.82%
Signature D, 12.51%
Other Signatures Below Significance Threshold, 7.36%

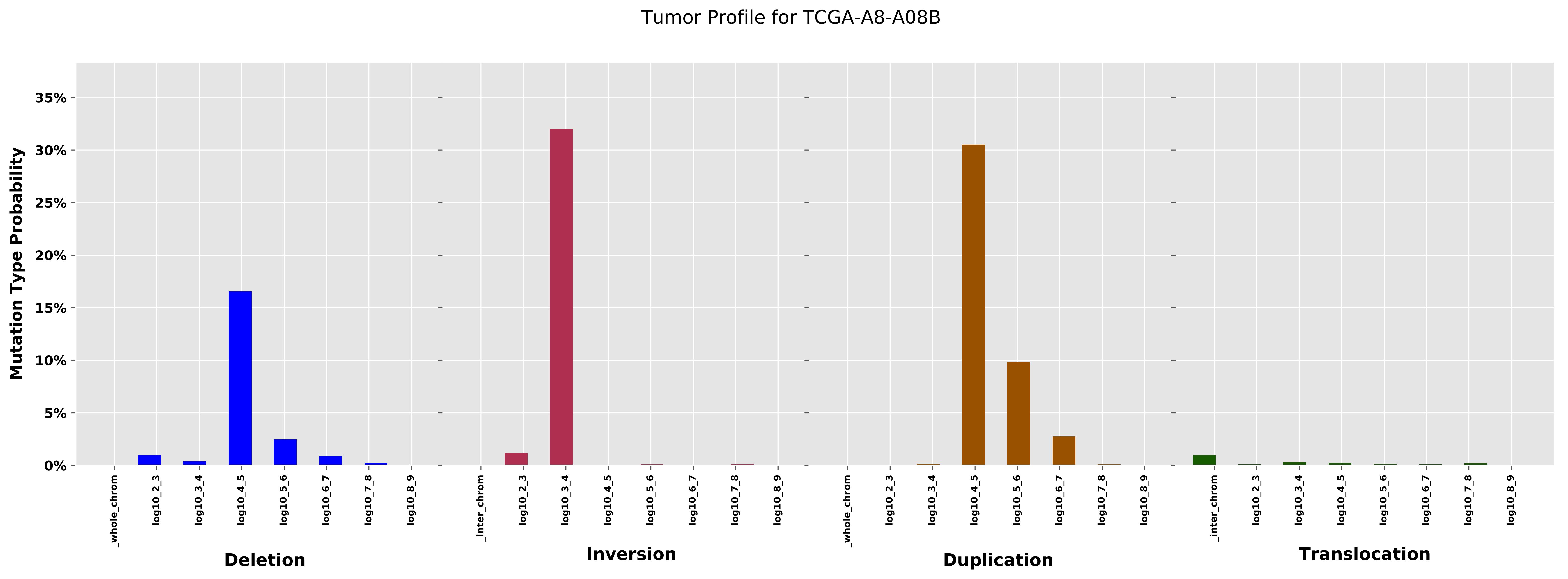

Tumor Profile for TCGA-A8-A08B
Mutation Type Probability
35%
30%
25%
20%
15%
10%
5%
0%
_whole_chrom
log10_2_3
log10_3_4
log10_4_5
log10_5_6
log10_6_7
log10_7_8
log10_8_9
_inter_chrom
Deletion
Inversion
Duplication
Translocation

Cancer processes Weights for TCGA-B6-A0RT

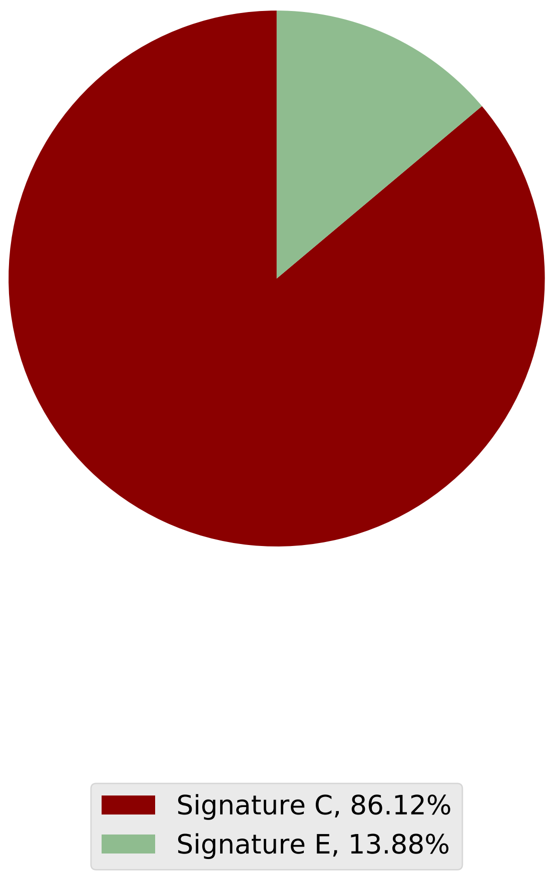

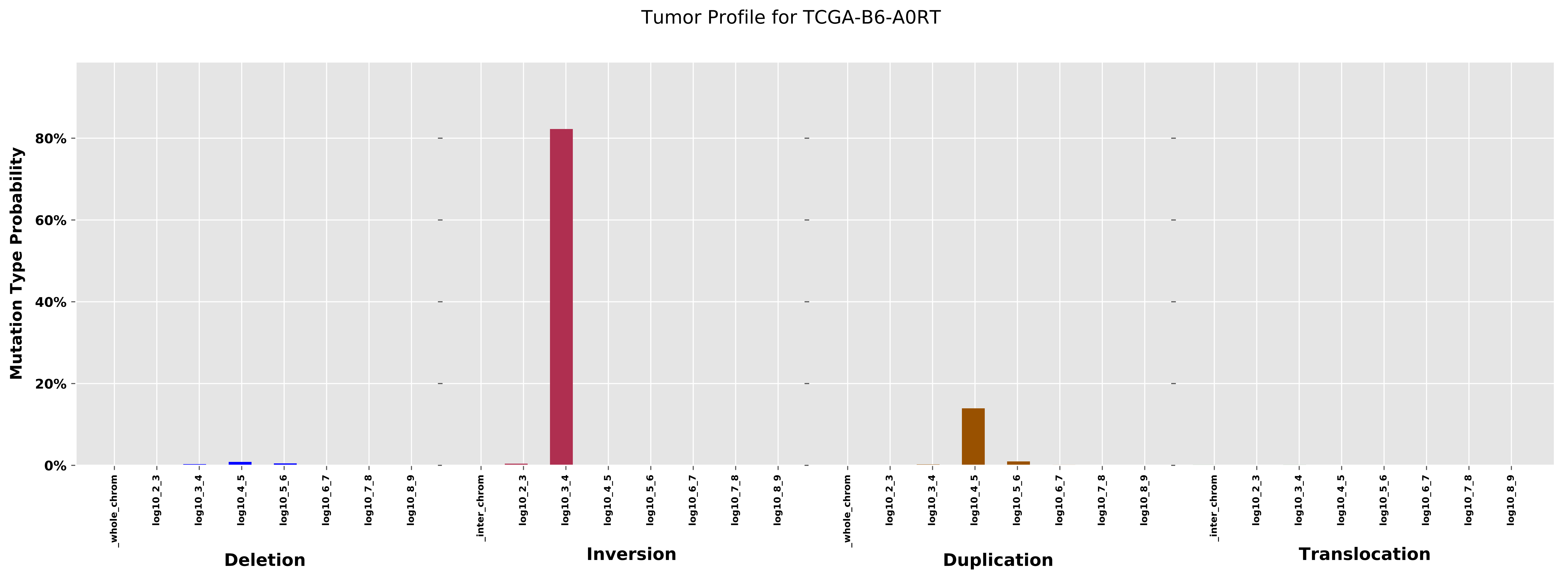

Tumor Profile for TCGA-B6-A0RT
Mutation Type Probability
0%
20%
40%
60%
80%
_whole_chrom
log10_2_3
log10_3_4
log10_4_5
log10_5_6
log10_6_7
log10_7_8
log10_8_9
_inter_chrom
Deletion
Inversion
Duplication
Translocation

Cancer processes Weights for TCGA-GM-A3XL

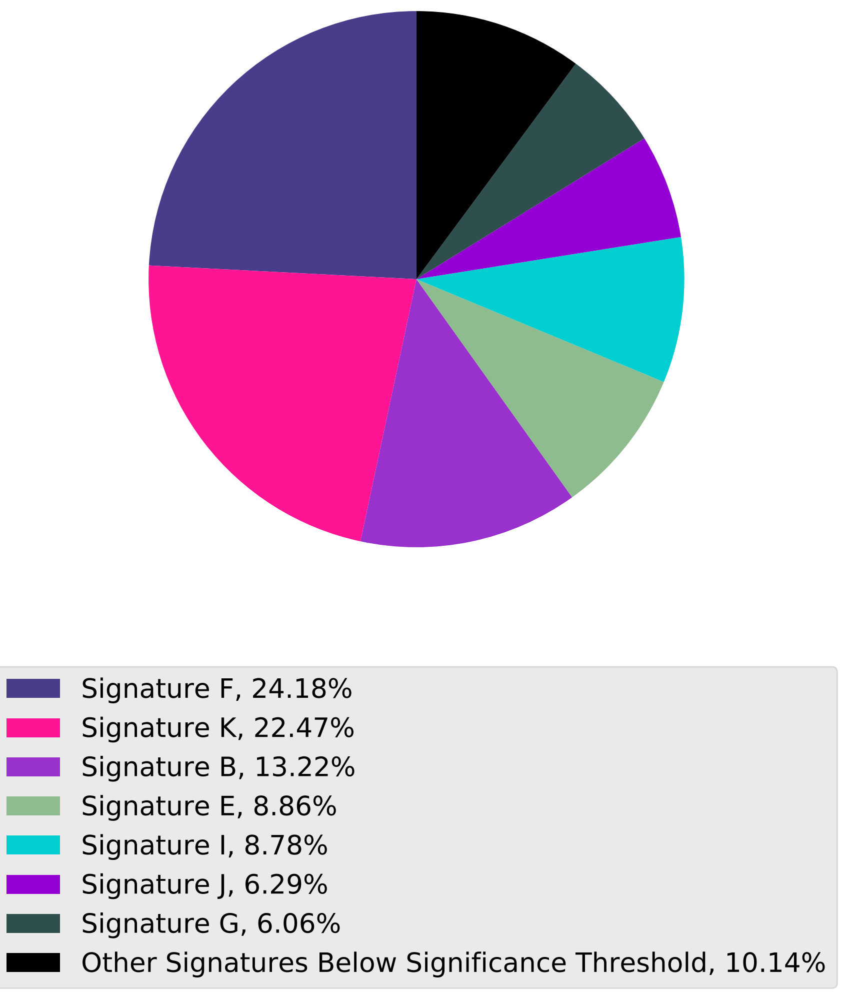

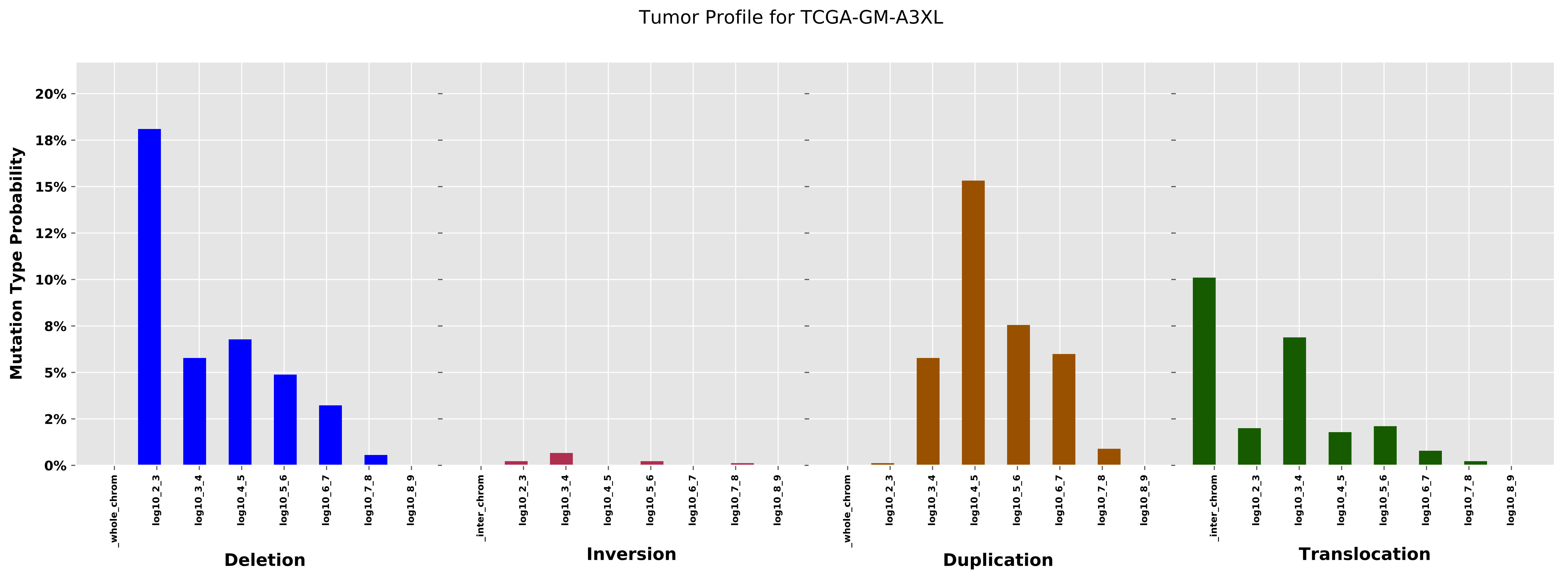

Tumor Profile for TCGA-GM-A3XL
Mutation Type Probability
20%
18%
15%
12%
10%
8%
5%
2%
0%
_whole_chrom
log10_2_3
log10_3_4
log10_4_5
log10_5_6
log10_6_7
log10_7_8
log10_8_9
Deletion
_inter_chrom
log10_2_3
log10_3_4
log10_4_5
log10_5_6
log10_6_7
log10_7_8
log10_8_9
Inversion
_whole_chrom
log10_2_3
log10_3_4
log10_4_5
log10_5_6
log10_6_7
log10_7_8
log10_8_9
Duplication
_inter_chrom
log10_2_3
log10_3_4
log10_4_5
log10_5_6
log10_6_7
log10_7_8
log10_8_9
Translocation

Cancer processes Weights for TCGA-AO-A0J2

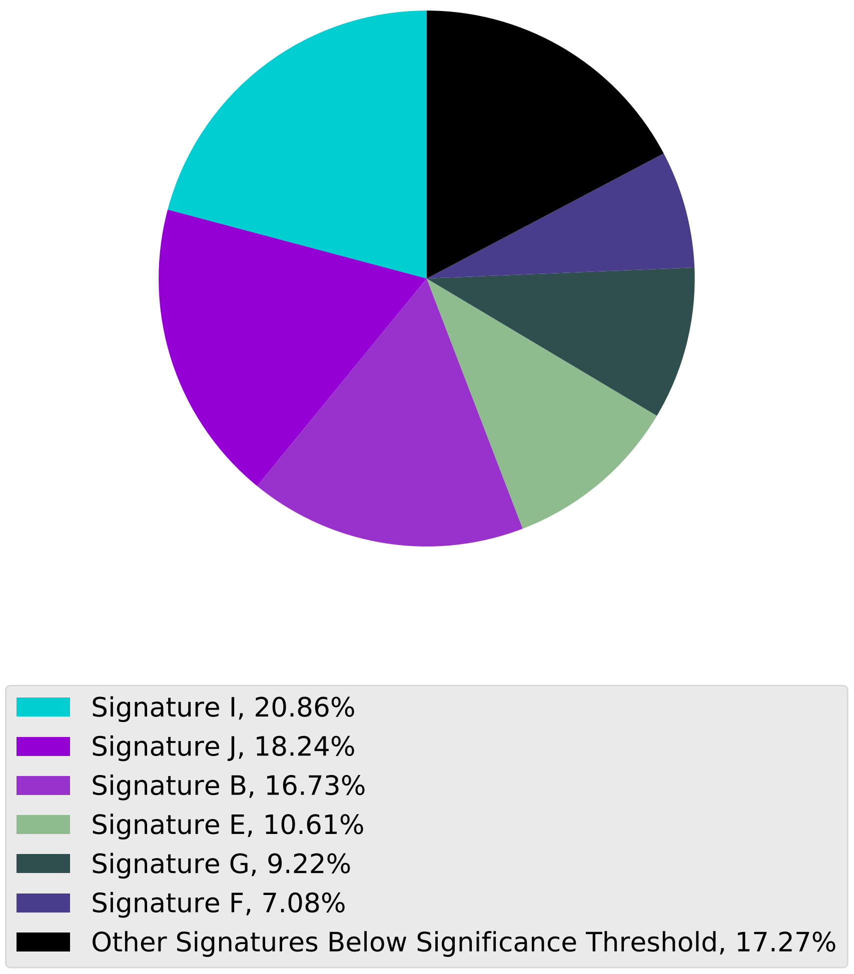

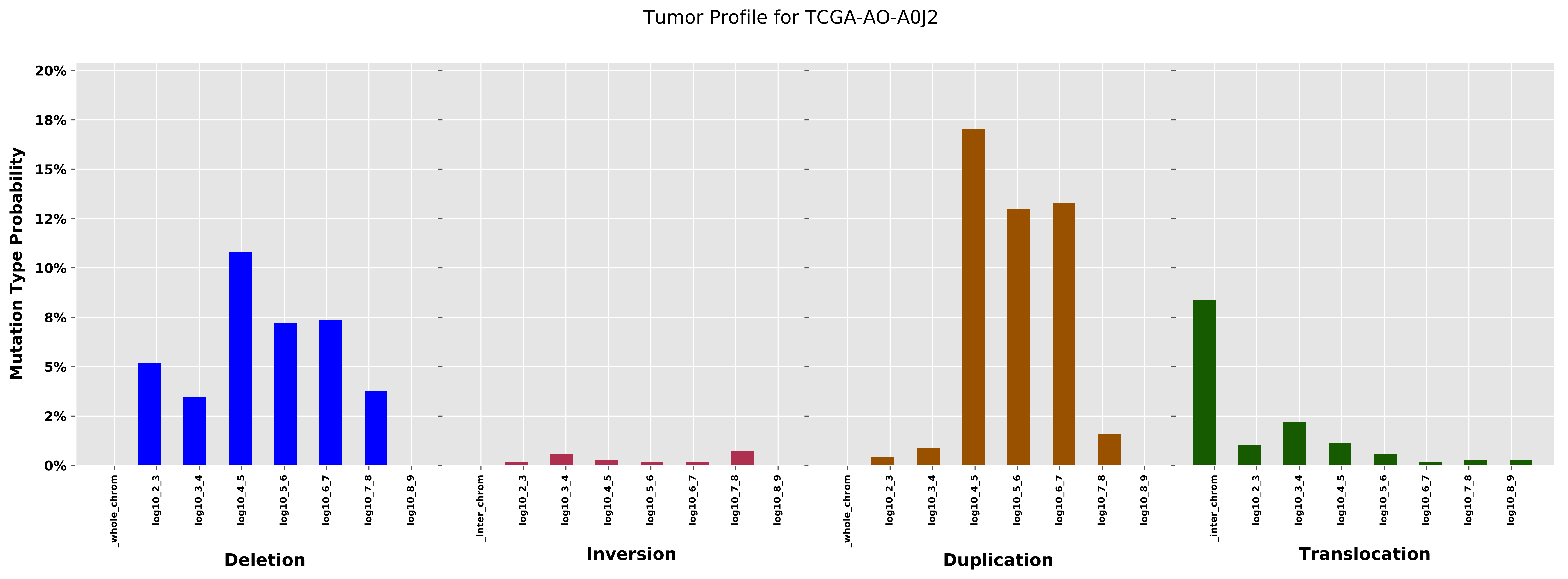

Tumor Profile for TCGA-AO-A0J2
Mutation Type Probability
20%
18%
15%
12%
10%
8%
5%
2%
0%
_whole_chrom
log10_2_3
log10_3_4
log10_4_5
log10_5_6
log10_6_7
log10_7_8
log10_8_9
Deletion
_inter_chrom
log10_2_3
log10_3_4
log10_4_5
log10_5_6
log10_6_7
log10_7_8
log10_8_9
Inversion
_whole_chrom
log10_2_3
log10_3_4
log10_4_5
log10_5_6
log10_6_7
log10_7_8
log10_8_9
Duplication
_inter_chrom
log10_2_3
log10_3_4
log10_4_5
log10_5_6
log10_6_7
log10_7_8
log10_8_9
Translocation

Cancer processes Weights for TCGA-E2-A156

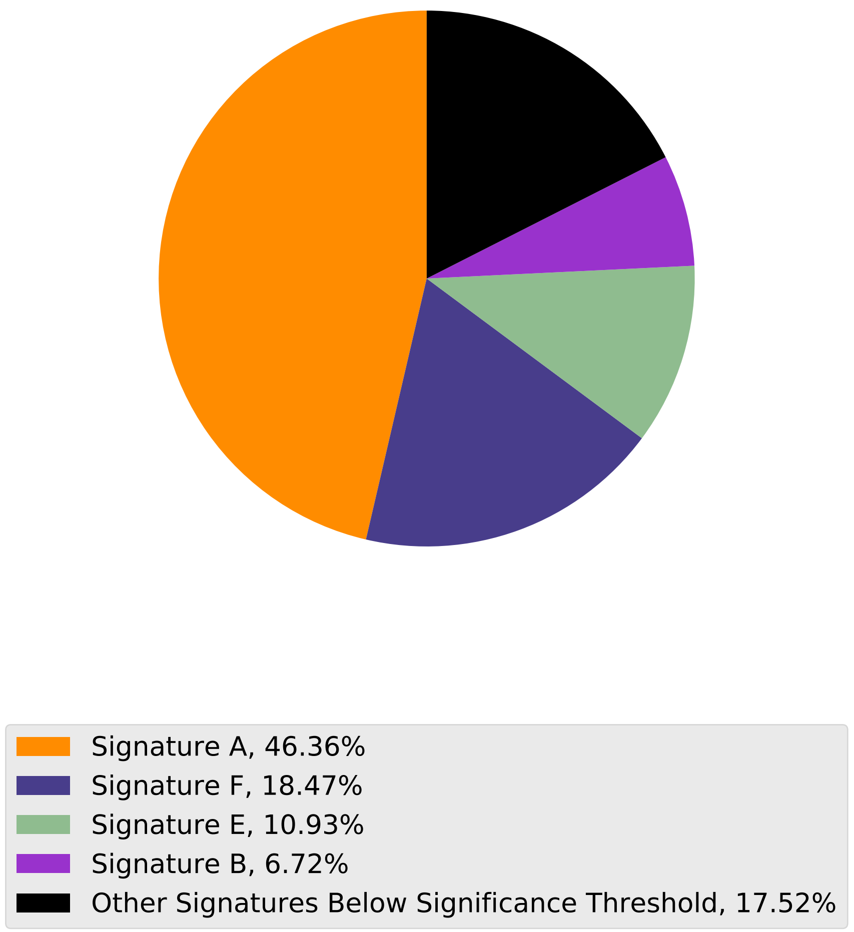

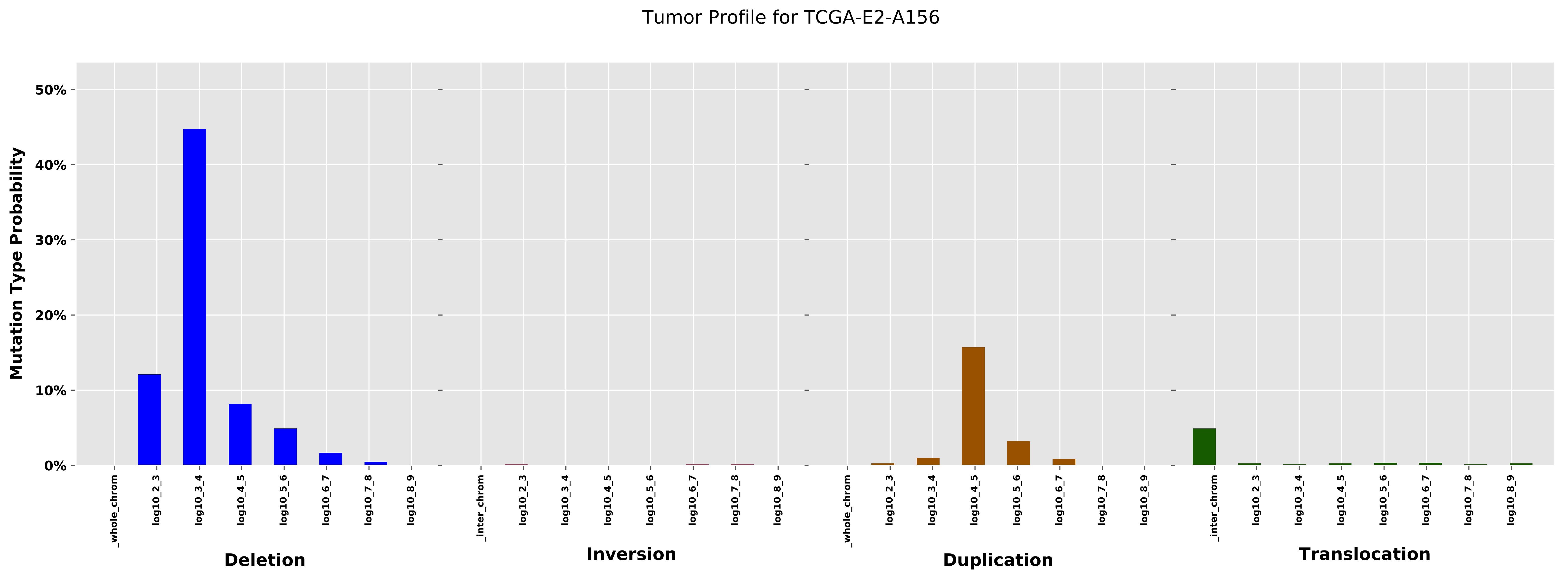
Tumor Profile for TCGA-E2-A156
Mutation Type Probability
50%
40%
30%
20%
10%
0%
_whole_chrom
log10_2_3
log10_3_4
log10_4_5
log10_5_6
log10_6_7
log10_7_8
log10_8_9
Deletion
_inter_chrom
log10_2_3
log10_3_4
log10_4_5
log10_5_6
log10_6_7
log10_7_8
log10_8_9
Inversion
_whole_chrom
log10_2_3
log10_3_4
log10_4_5
log10_5_6
log10_6_7
log10_7_8
log10_8_9
Duplication
_inter_chrom
log10_2_3
log10_3_4
log10_4_5
log10_5_6
log10_6_7
log10_7_8
log10_8_9
Translocation

Cancer processes Weights for TCGA-BH-A18R

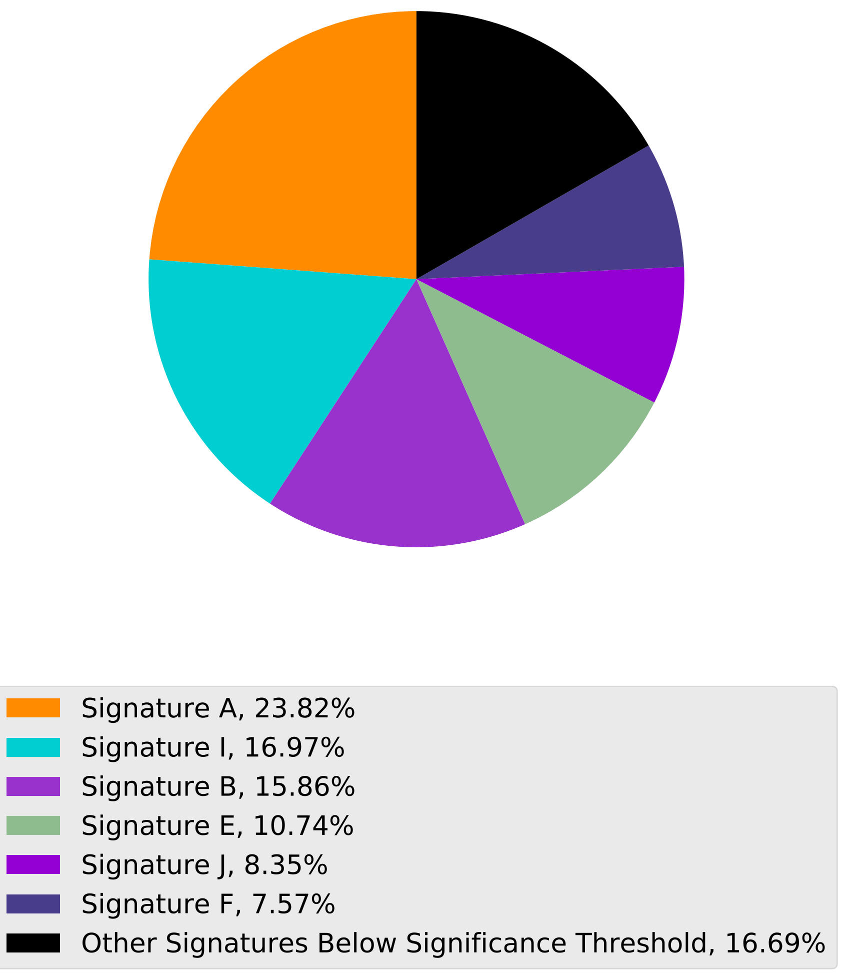

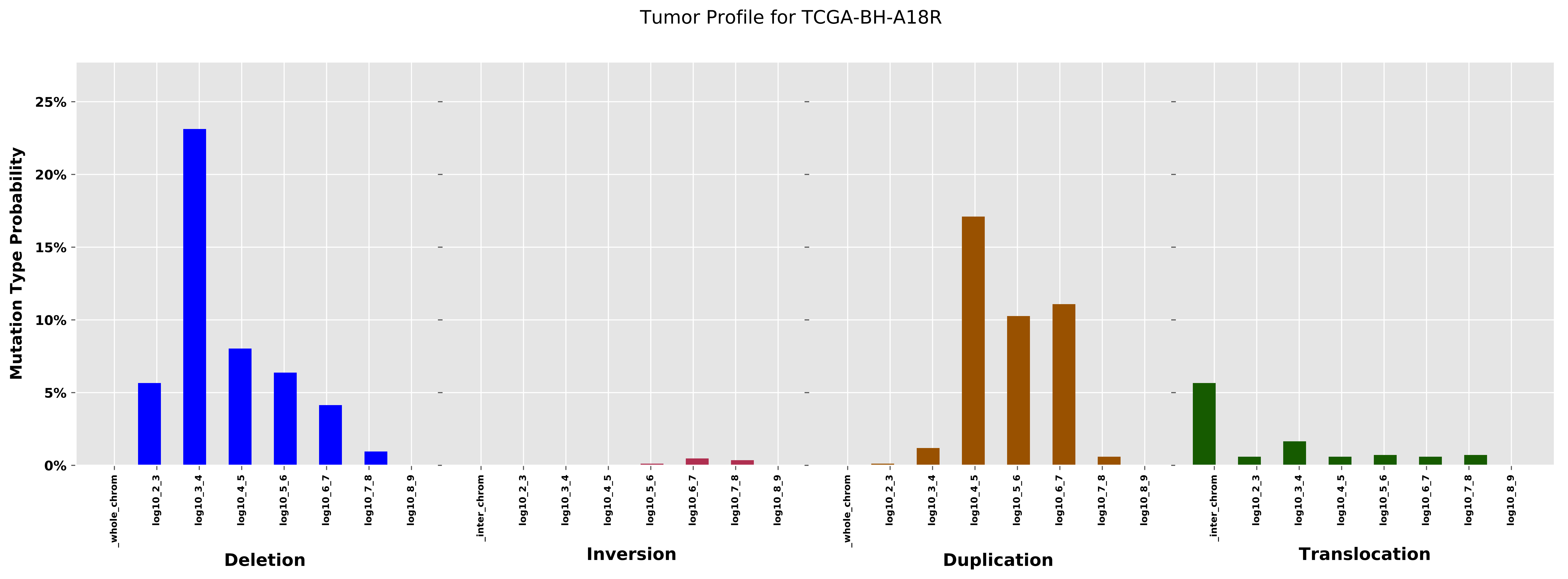

Tumor Profile for TCGA-BH-A18R
Mutation Type Probability
25%
20%
15%
10%
5%
0%
_whole_chrom
log10_2_3
log10_3_4
log10_4_5
log10_5_6
log10_6_7
log10_7_8
log10_8_9
_inter_chrom
Deletion
Inversion
Duplication
Translocation

Cancer processes Weights for TCGA-AO-A0JM

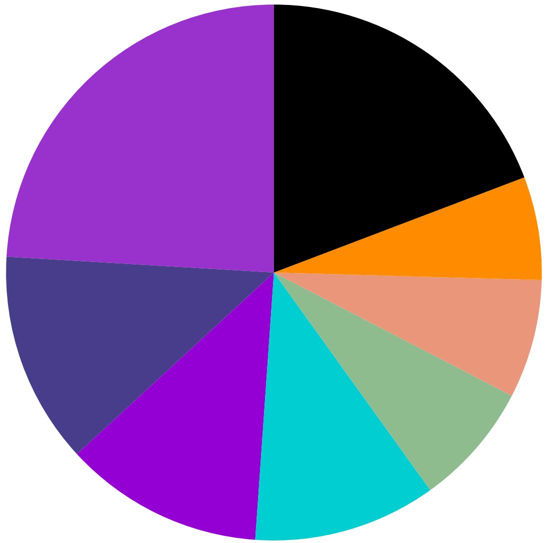

Signature B, 24.06%
Signature F, 12.79%
Signature J, 12.04%
Signature I, 11.03%
Signature E, 7.48%
Signature D, 7.16%
Signature A, 6.23%
Other Signatures Below Significance Threshold, 19.22%

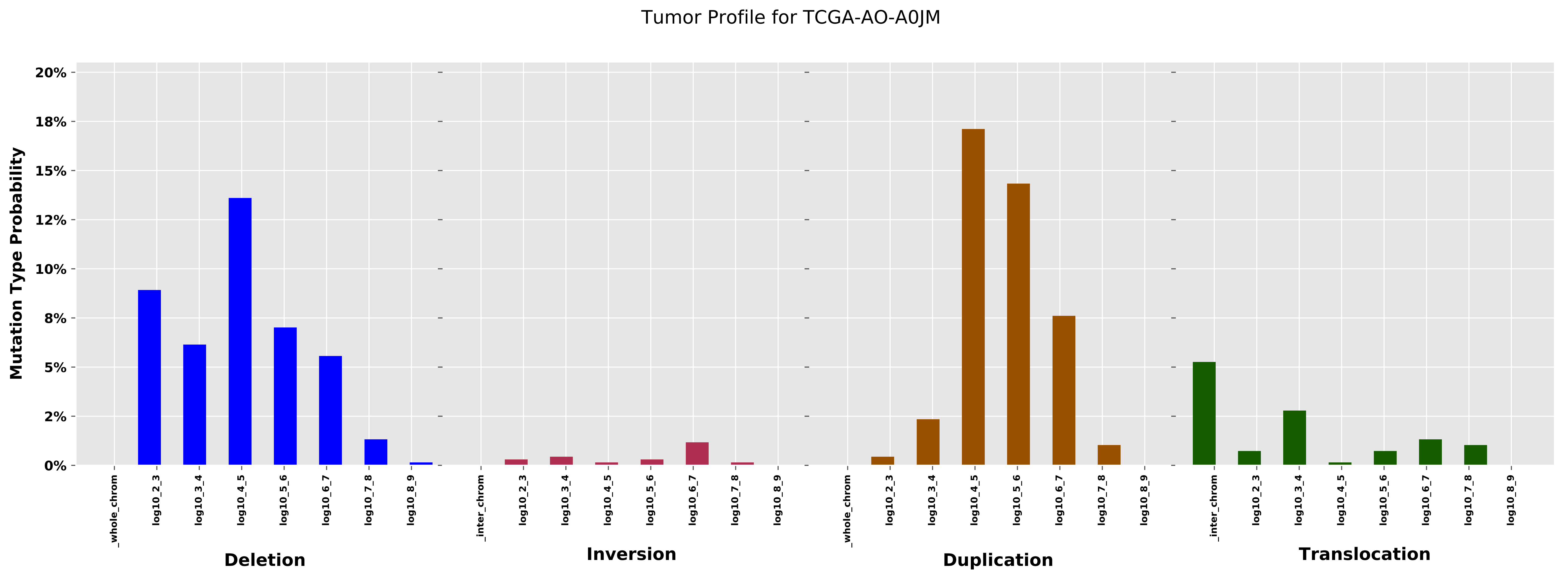

Tumor Profile for TCGA-AO-A0JM
Mutation Type Probability
20%
18%
15%
12%
10%
8%
5%
2%
0%
_whole_chrom
log10_2_3
log10_3_4
log10_4_5
log10_5_6
log10_6_7
log10_7_8
log10_8_9
_inter_chrom
Deletion
Inversion
Duplication
Translocation

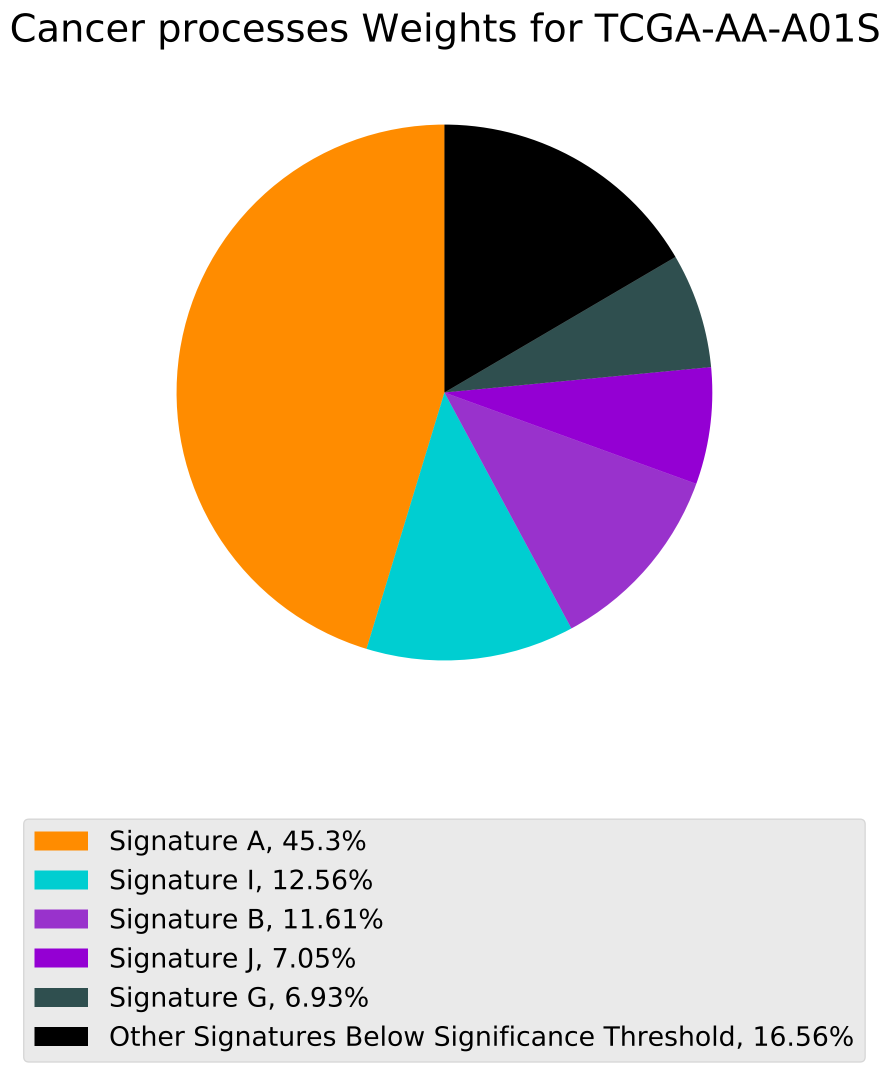
Cancer processes Weights for TCGA-AA-A01S
Signature A, 45.3%
Signature I, 12.56%
Signature B, 11.61%
Signature J, 7.05%
Signature G, 6.93%
Other Signatures Below Significance Threshold, 16.56%

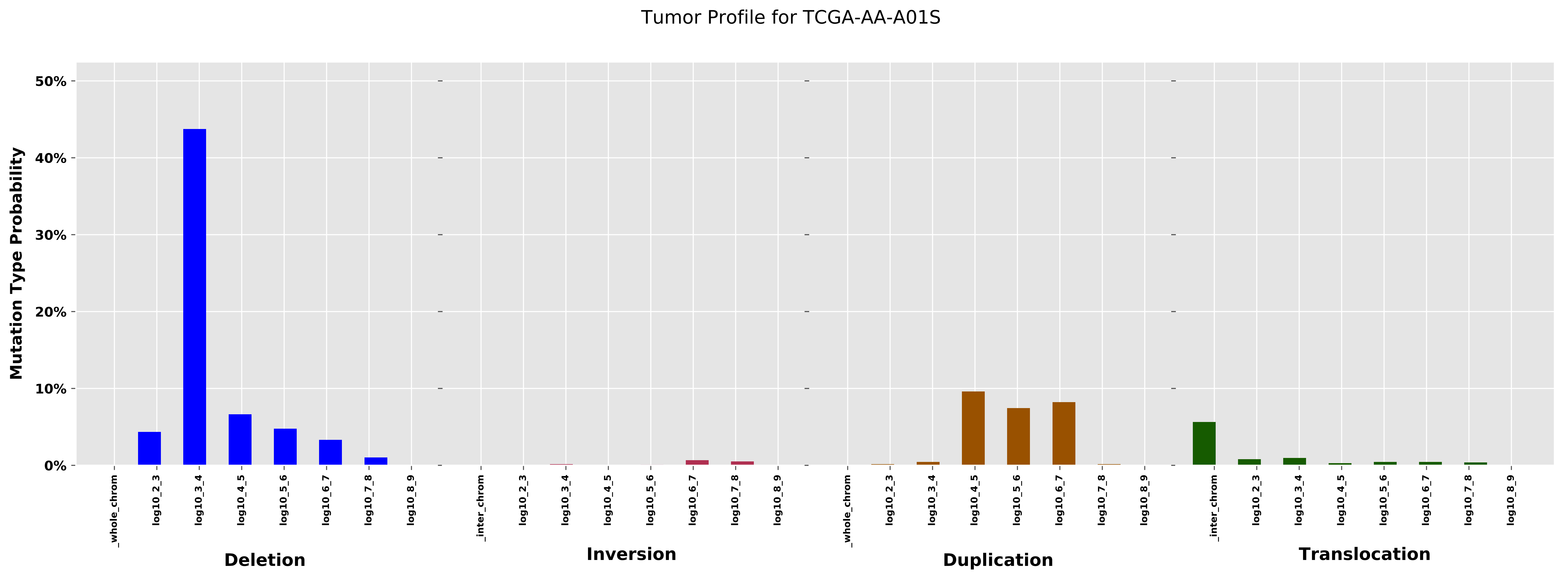

Tumor Profile for TCGA-AA-A01S
Mutation Type Probability
50%
40%
30%
20%
10%
0%
_whole_chrom
log10_2_3
log10_3_4
log10_4_5
log10_5_6
log10_6_7
log10_7_8
log10_8_9
_inter_chrom
Deletion
Inversion
Duplication
Translocation

Cancer processes Weights for TCGA-AN-A0AT

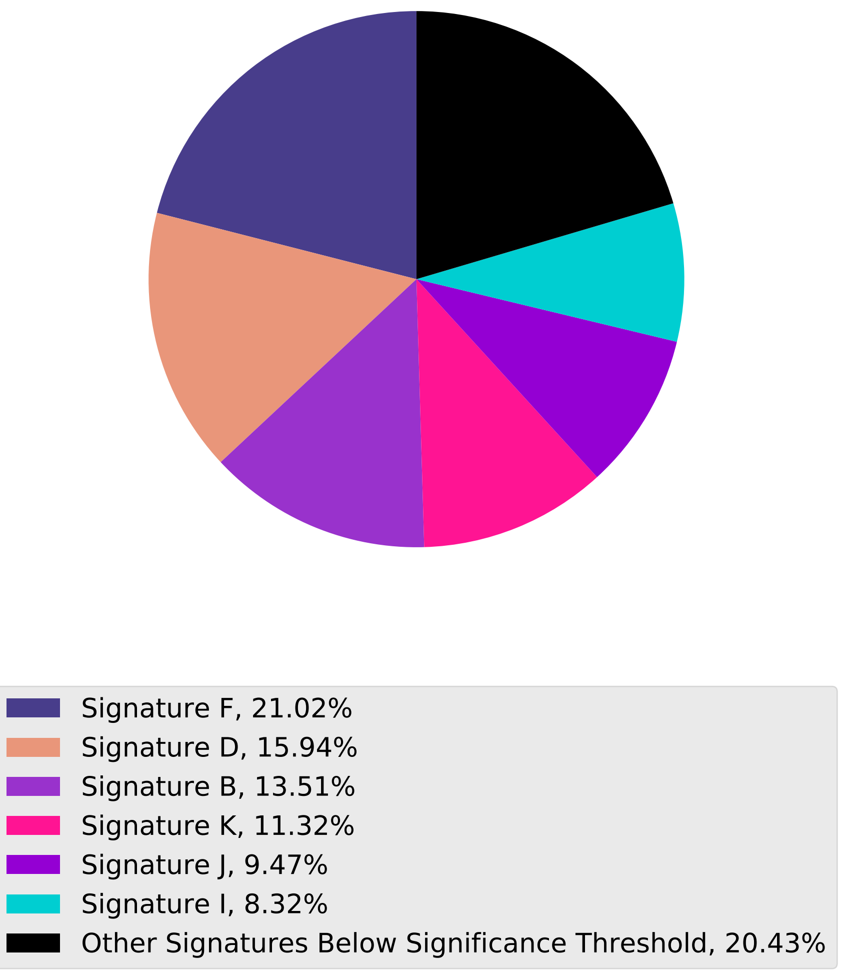

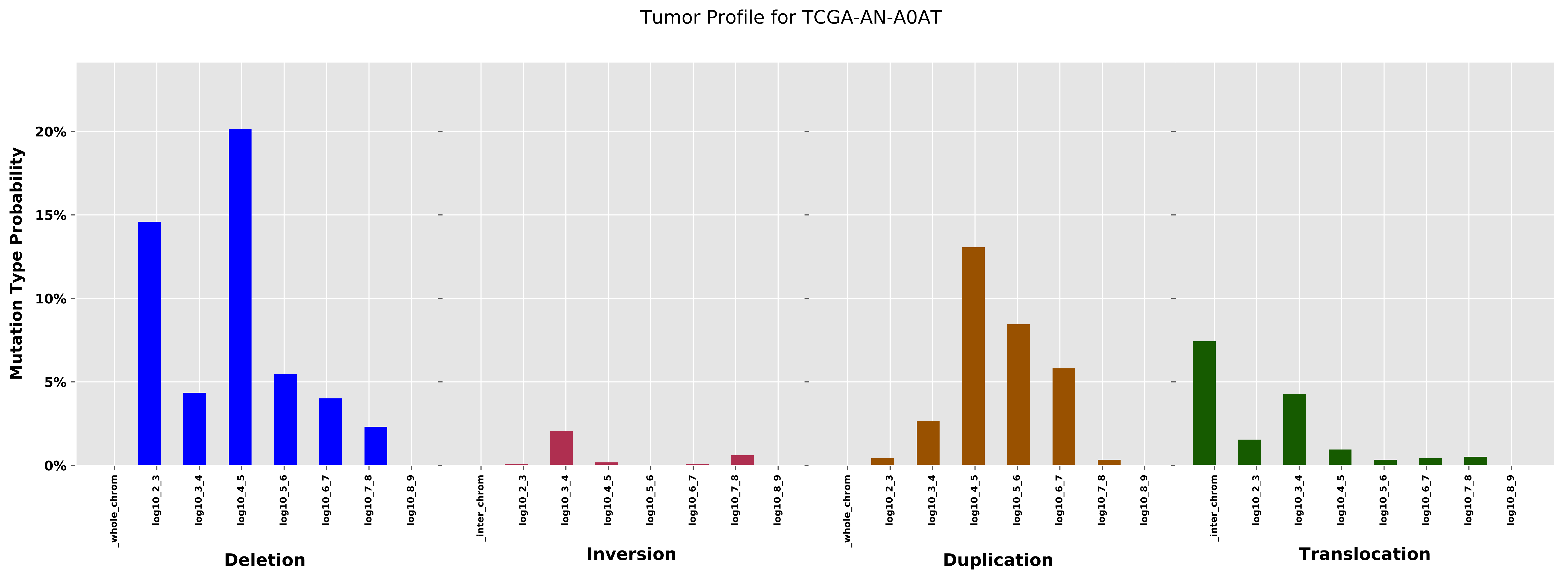

Tumor Profile for TCGA-AN-A0AT
Mutation Type Probability
20%
15%
10%
5%
0%
_whole_chrom
log10_2_3
log10_3_4
log10_4_5
log10_5_6
log10_6_7
log10_7_8
log10_8_9
_inter_chrom
Deletion
Inversion
Duplication
Translocation

Cancer processes Weights for TCGA-B6-A0WX

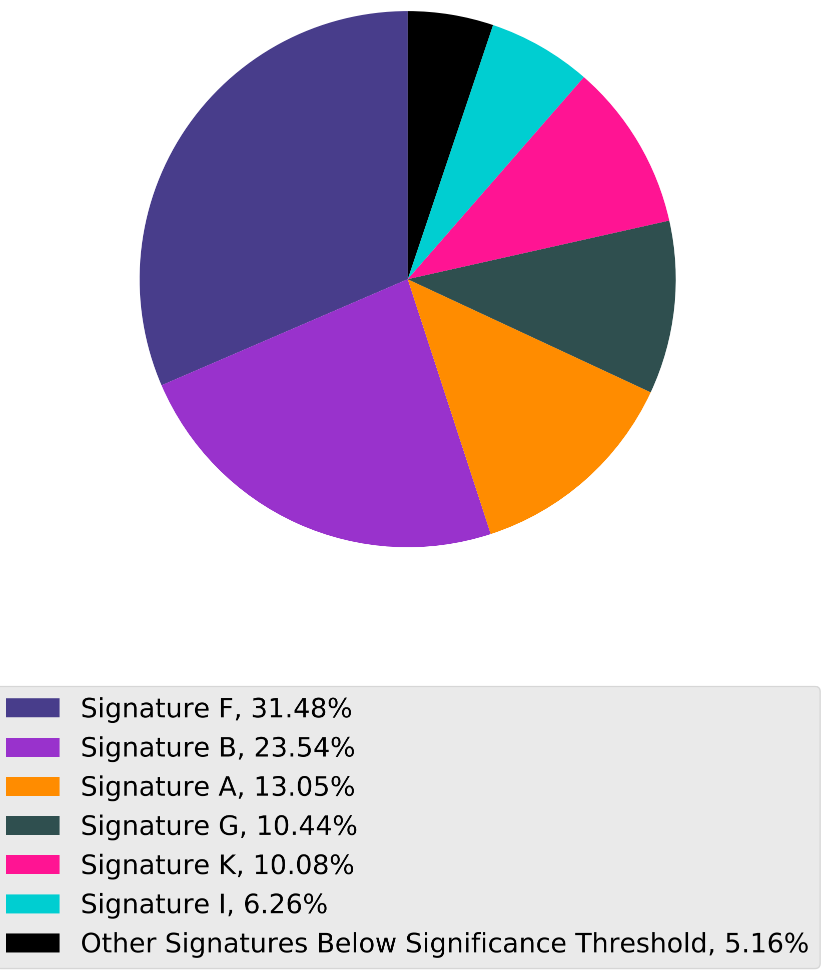

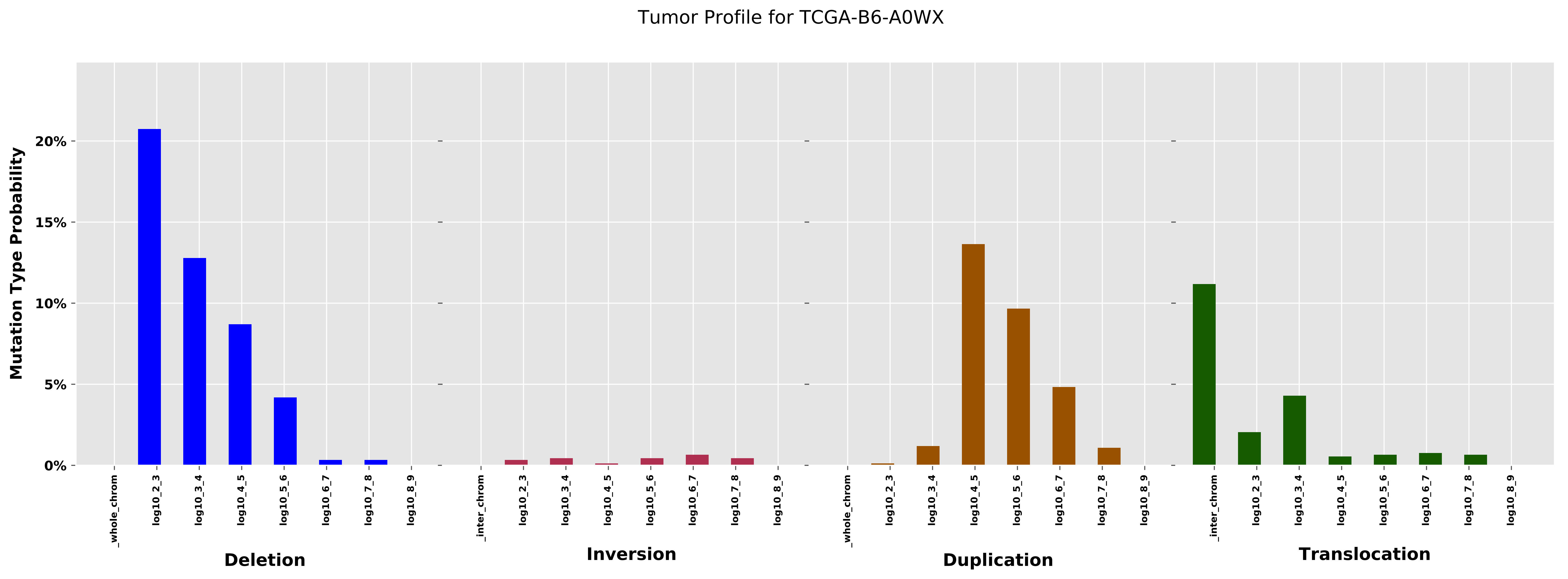

Tumor Profile for TCGA-B6-A0WX
Mutation Type Probability
20%
15%
10%
5%
0%
_whole_chrom
log10_2_3
log10_3_4
log10_4_5
log10_5_6
log10_6_7
log10_7_8
log10_8_9
_inter_chrom
Deletion
Inversion
Duplication
Translocation

Cancer processes Weights for TCGA-BH-A0WA

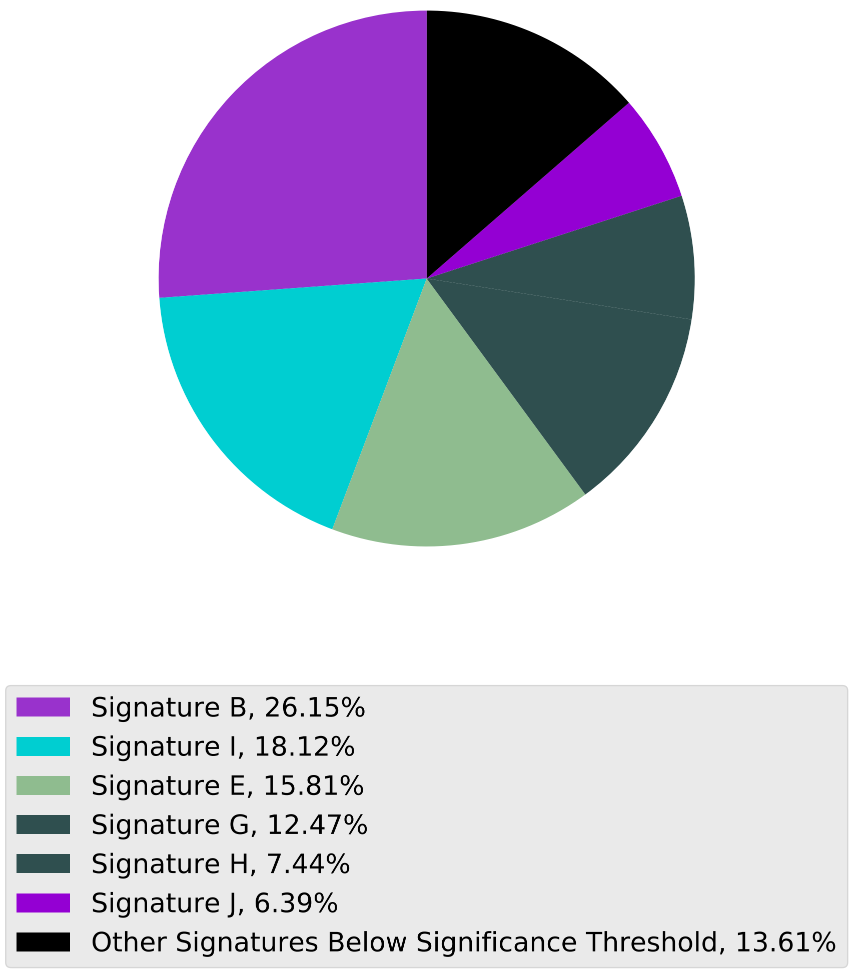

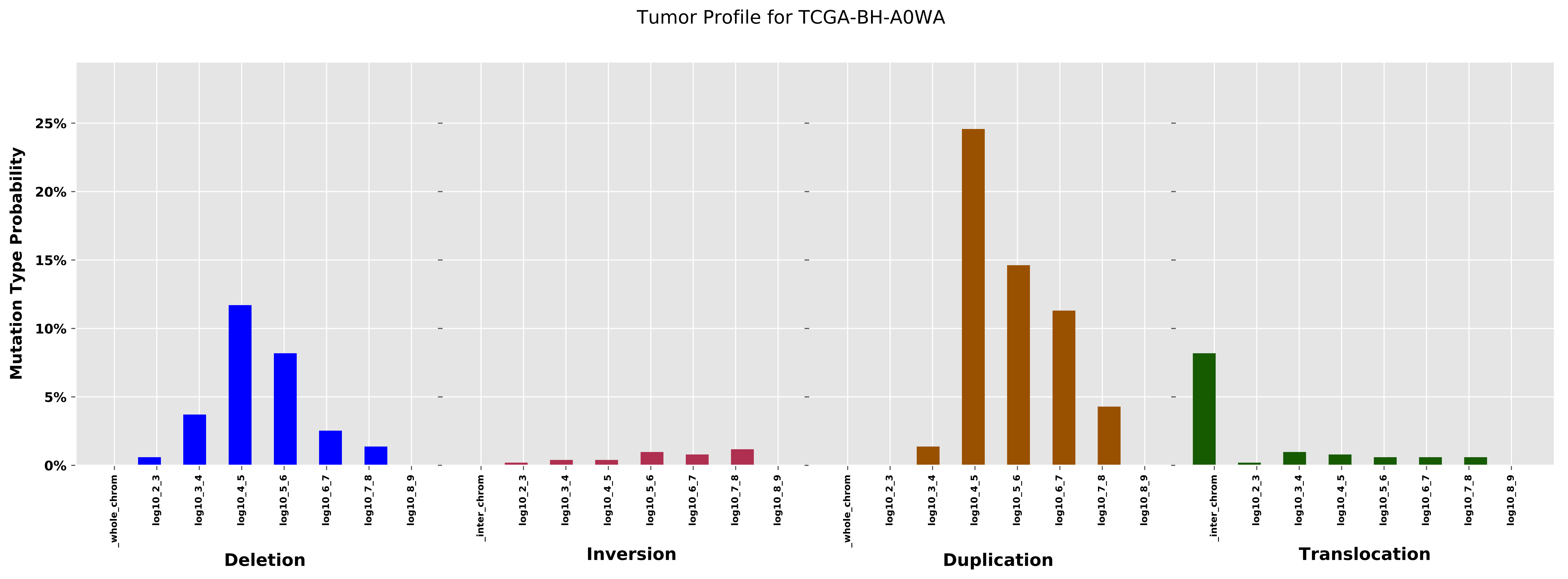
Tumor Profile for TCGA-BH-A0WA
Mutation Type Probability
25%
20%
15%
10%
5%
0%
_whole_chrom
log10_2_3
log10_3_4
log10_4_5
log10_5_6
log10_6_7
log10_7_8
log10_8_9
_inter_chrom
Deletion
Inversion
Duplication
Translocation

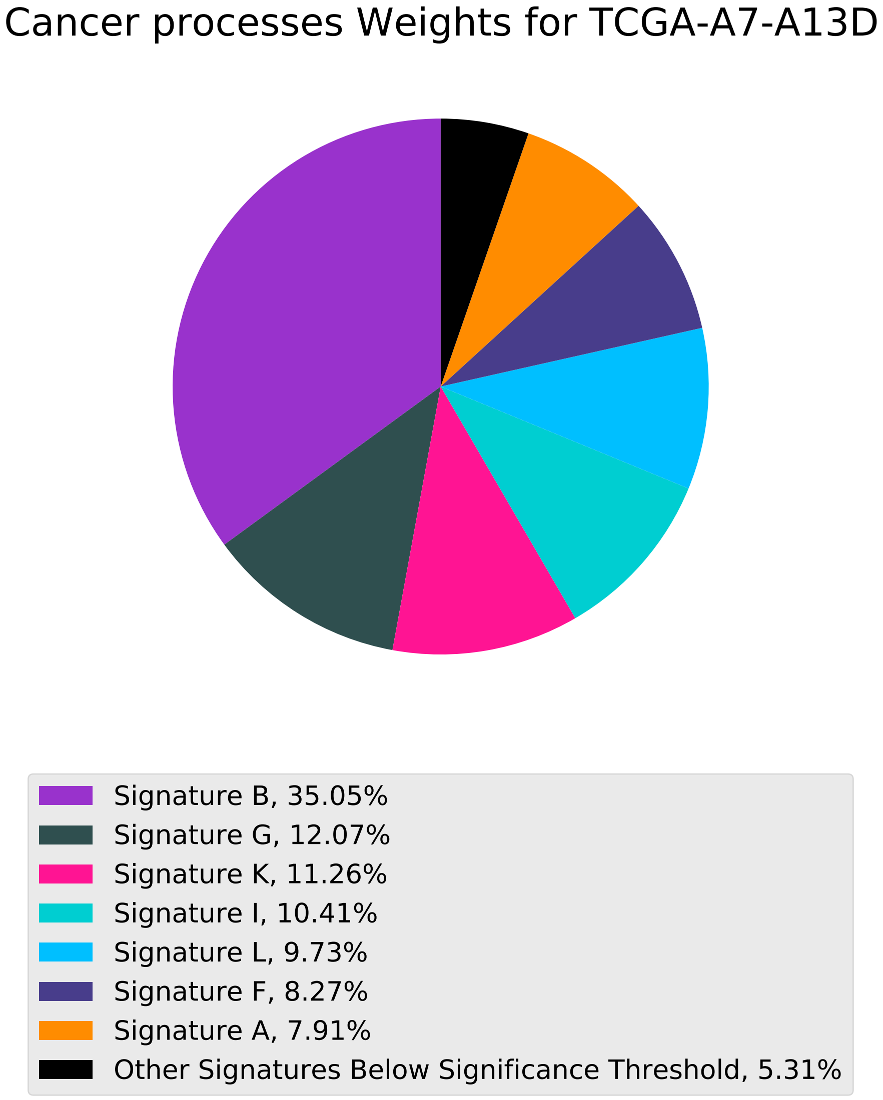
Cancer processes Weights for TCGA-A7-A13D
Signature B, 35.05%
Signature G, 12.07%
Signature K, 11.26%
Signature I, 10.41%
Signature L, 9.73%
Signature F, 8.27%
Signature A, 7.91%
Other Signatures Below Significance Threshold, 5.31%

Tumor Profile for TCGA-A7-A13D

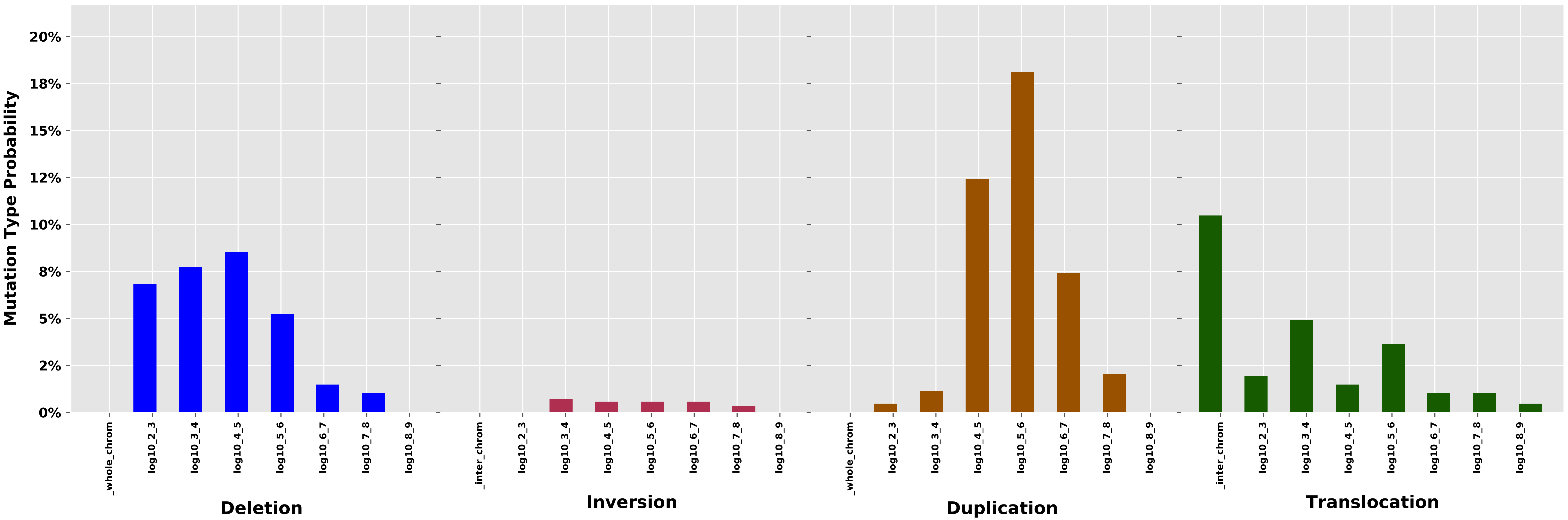

Cancer processes Weights for TCGA-B6-A0IJ

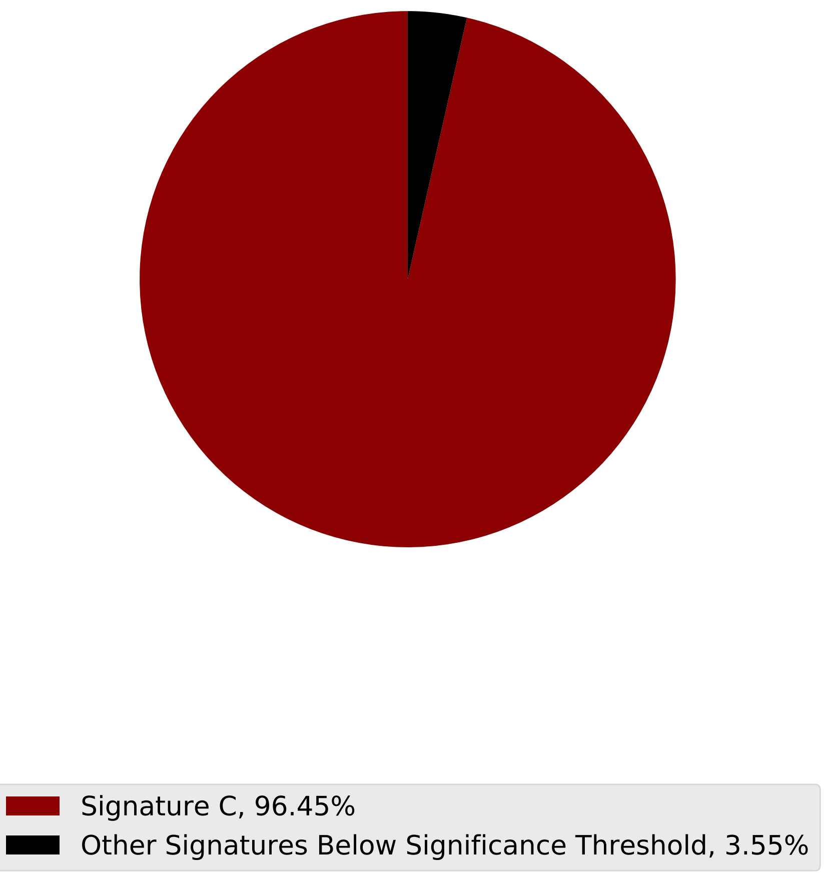

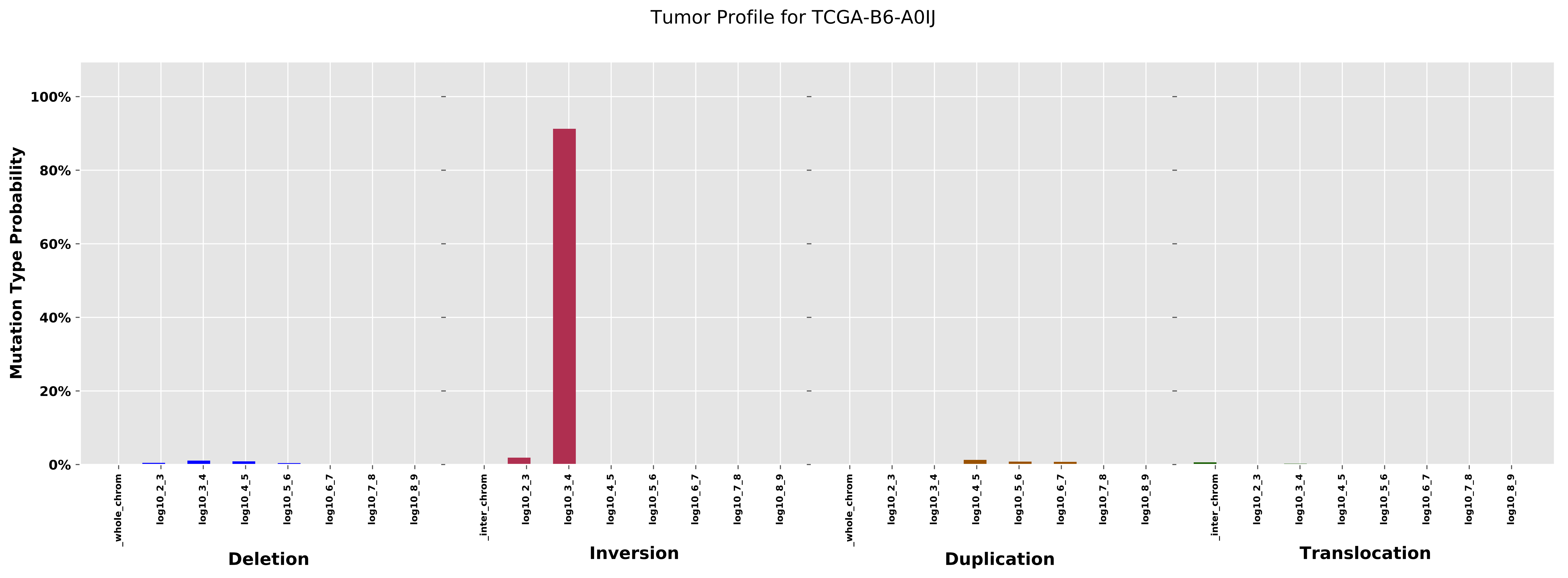

Tumor Profile for TCGA-B6-A0IJ
Mutation Type Probability
100%
80%
60%
40%
20%
0%
_whole_chrom
log10_2_3
log10_3_4
log10_4_5
log10_5_6
log10_6_7
log10_7_8
log10_8_9
_inter_chrom
Deletion
Inversion
Duplication
Translocation

Cancer processes Weights for TCGA-A2-A04Q

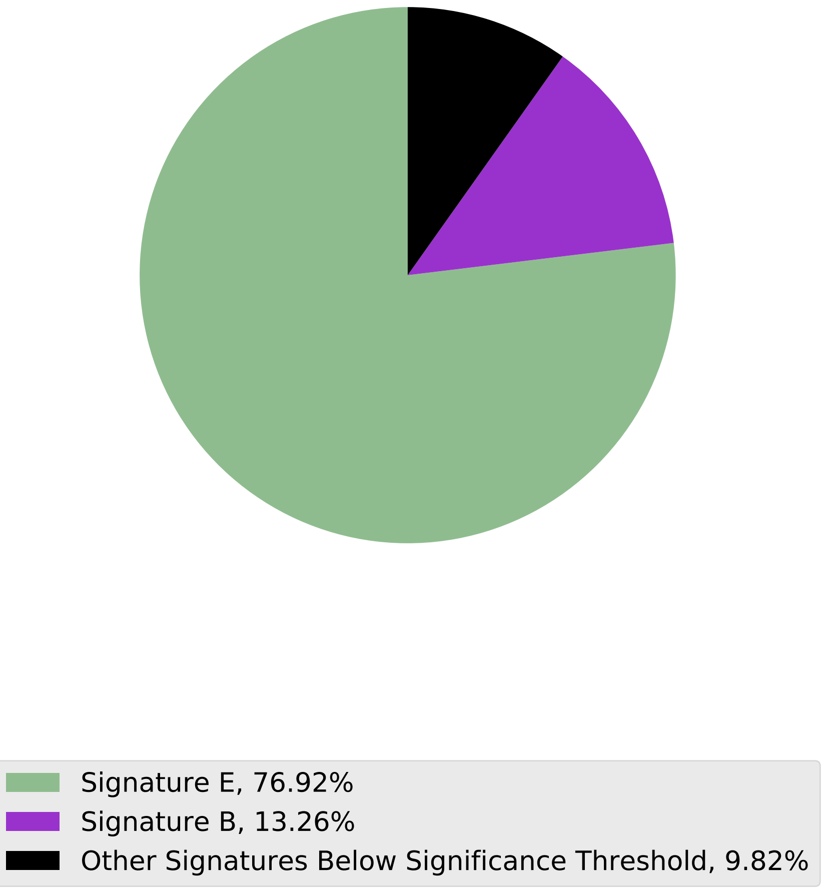

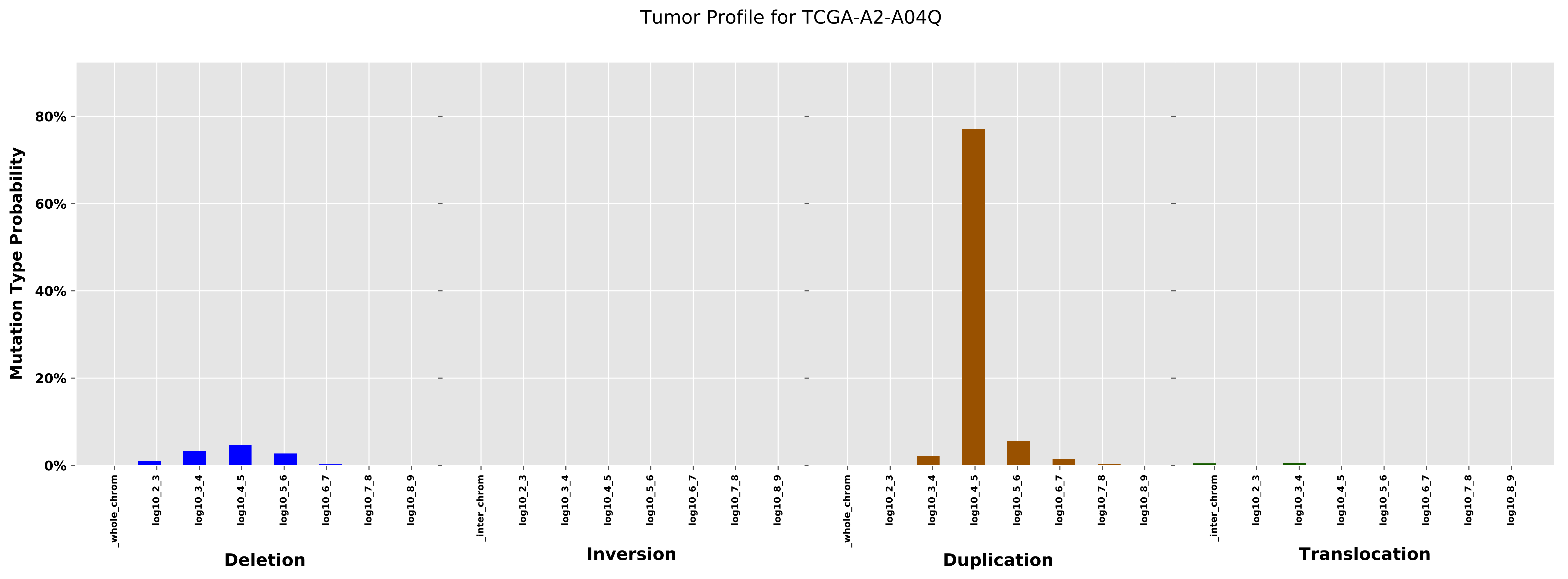

Tumor Profile for TCGA-A2-A04Q
Mutation Type Probability
80%
60%
40%
20%
0%
_whole_chrom
log10_2_3
log10_3_4
log10_4_5
log10_5_6
log10_6_7
log10_7_8
log10_8_9
_inter_chrom
Deletion
Inversion
Duplication
Translocation

Cancer processes Weights for TCGA-BH-A0B3

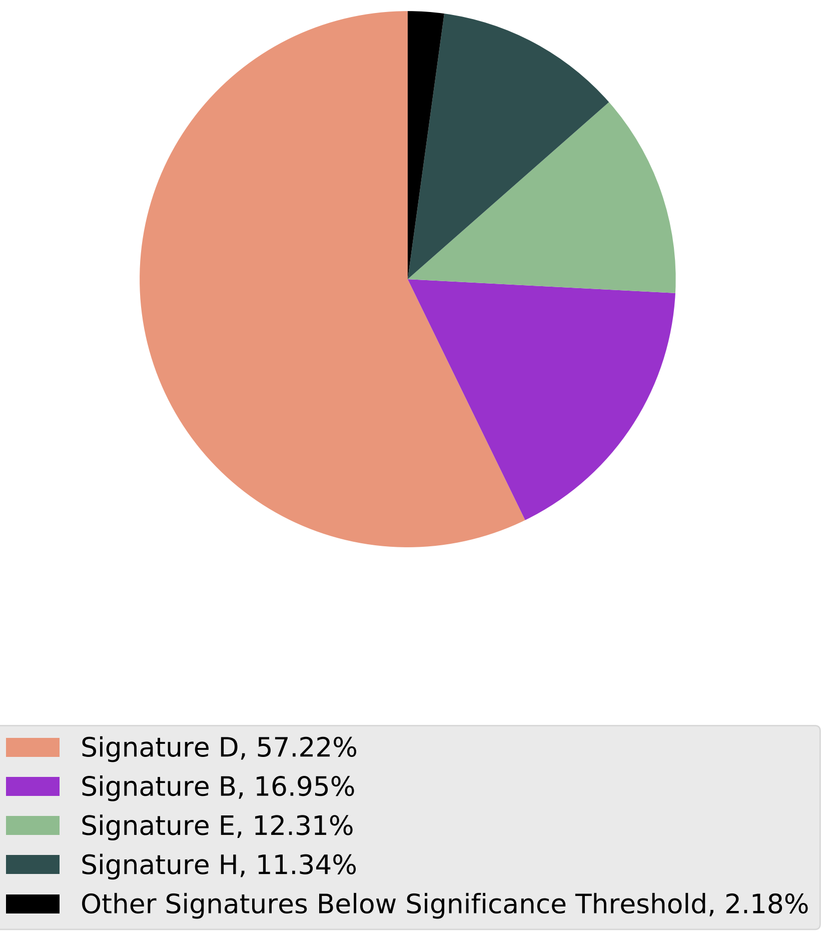

Tumor Profile for TCGA-BH-A0B3

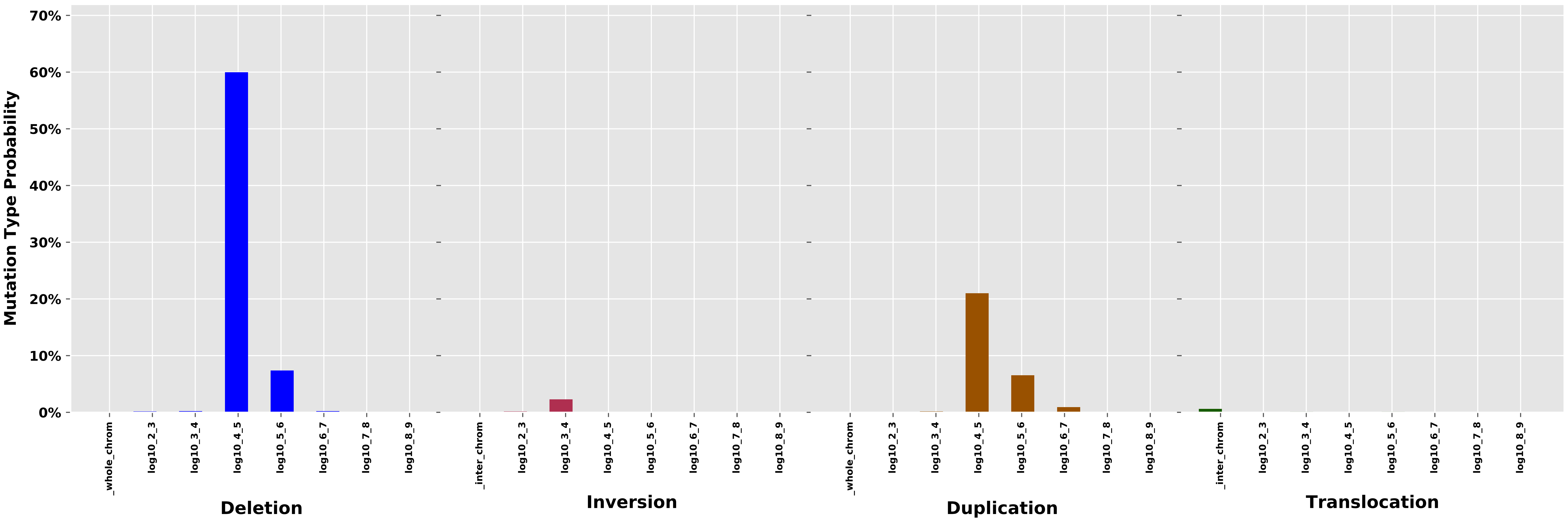

Cancer processes Weights for TCGA-BH-A18U

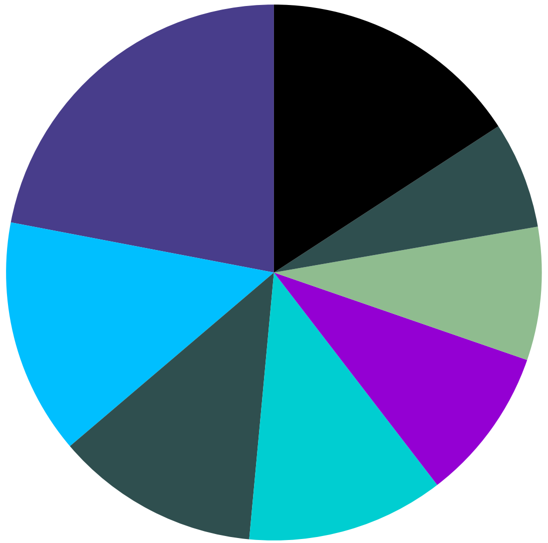

- Signature F, 22.0%
- Signature L, 14.22%
- Signature G, 12.31%
- Signature I, 11.91%
- Signature J, 9.27%
- Signature E, 8.04%
- Signature H, 6.45%
- Other Signatures Below Significance Threshold, 15.81%

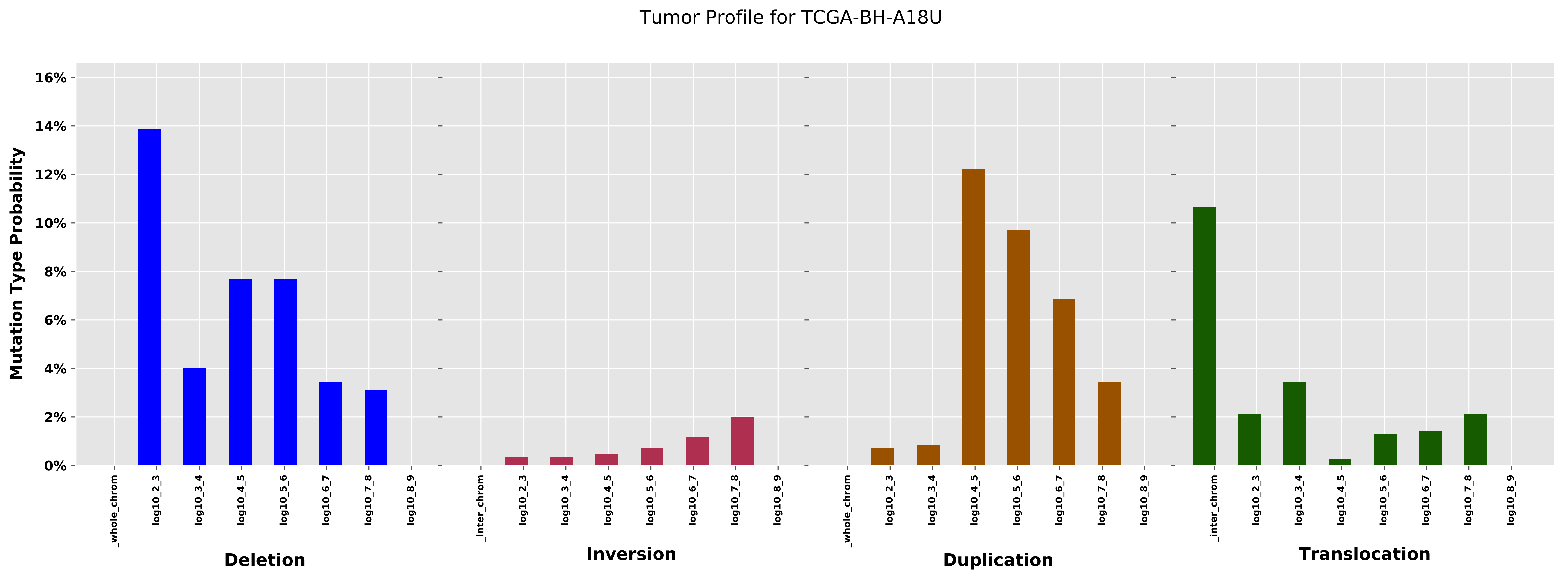

Tumor Profile for TCGA-BH-A18U
Mutation Type Probability
16%
14%
12%
10%
8%
6%
4%
2%
0%
_whole_chrom
log10_2_3
log10_3_4
log10_4_5
log10_5_6
log10_6_7
log10_7_8
log10_8_9
_inter_chrom
Deletion
Inversion
Duplication
Translocation

Cancer processes Weights for TCGA-GI-A2C9

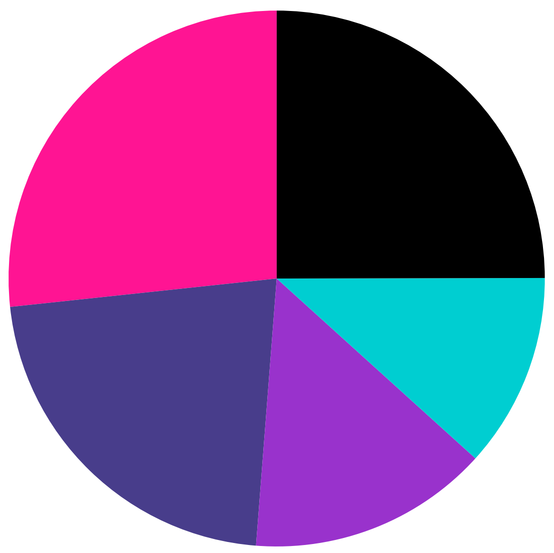

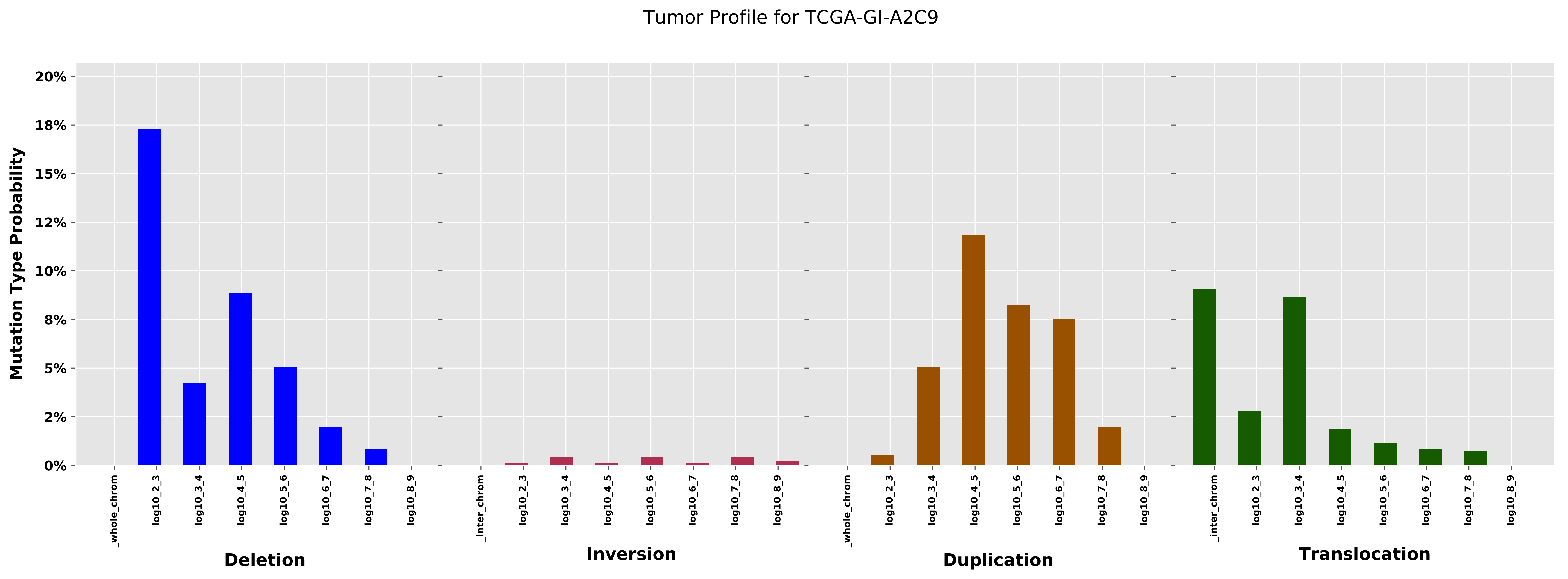

Tumor Profile for TCGA-GI-A2C9
Mutation Type Probability
20%
18%
15%
12%
10%
8%
5%
2%
0%
_whole_chrom
log10_2_3
log10_3_4
log10_4_5
log10_5_6
log10_6_7
log10_7_8
log10_8_9
_inter_chrom
Deletion
Inversion
Duplication
Translocation

Cancer processes Weights for TCGA-BH-A0DT

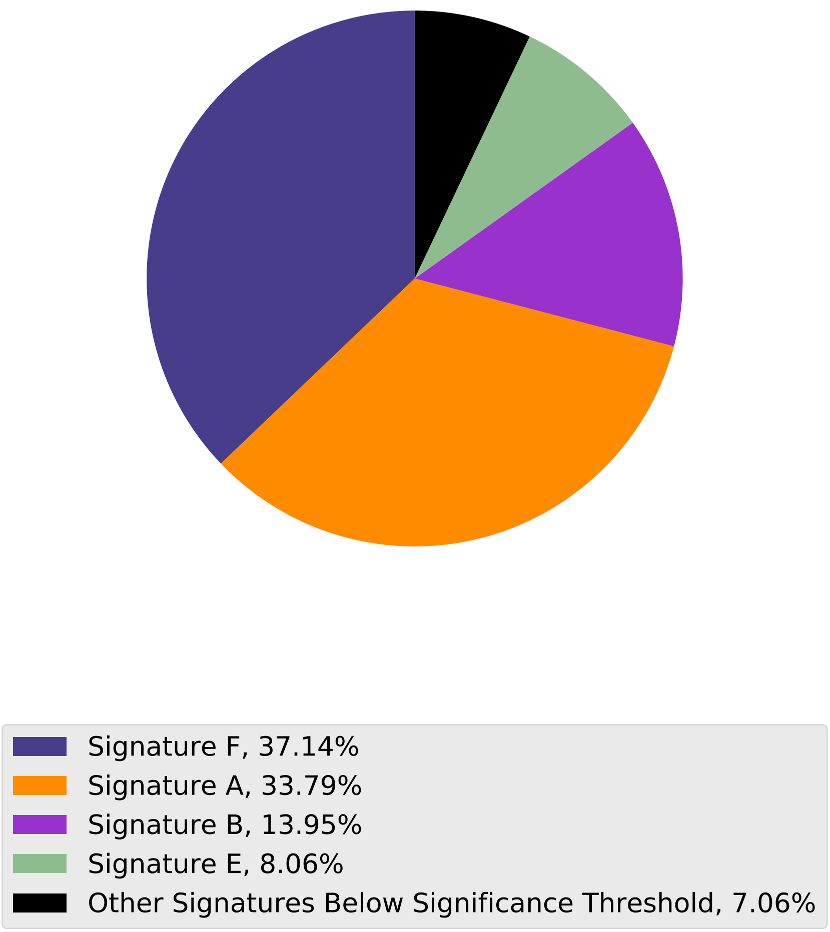

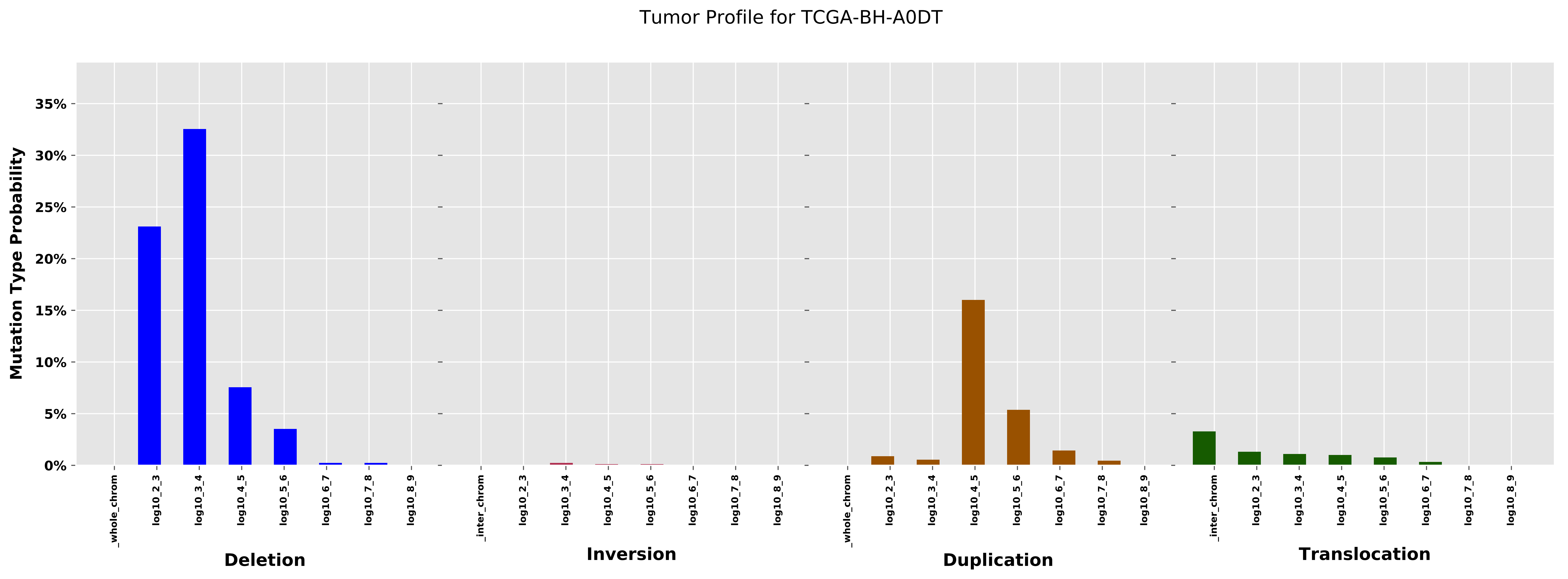

Tumor Profile for TCGA-BH-A0DT
Mutation Type Probability
35%
30%
25%
20%
15%
10%
5%
0%
_whole_chrom
log10_2_3
log10_3_4
log10_4_5
log10_5_6
log10_6_7
log10_7_8
log10_8_9
Deletion
_inter_chrom
log10_2_3
log10_3_4
log10_4_5
log10_5_6
log10_6_7
log10_7_8
log10_8_9
Inversion
_whole_chrom
log10_2_3
log10_3_4
log10_4_5
log10_5_6
log10_6_7
log10_7_8
log10_8_9
Duplication
_inter_chrom
log10_2_3
log10_3_4
log10_4_5
log10_5_6
log10_6_7
log10_7_8
log10_8_9
Translocation

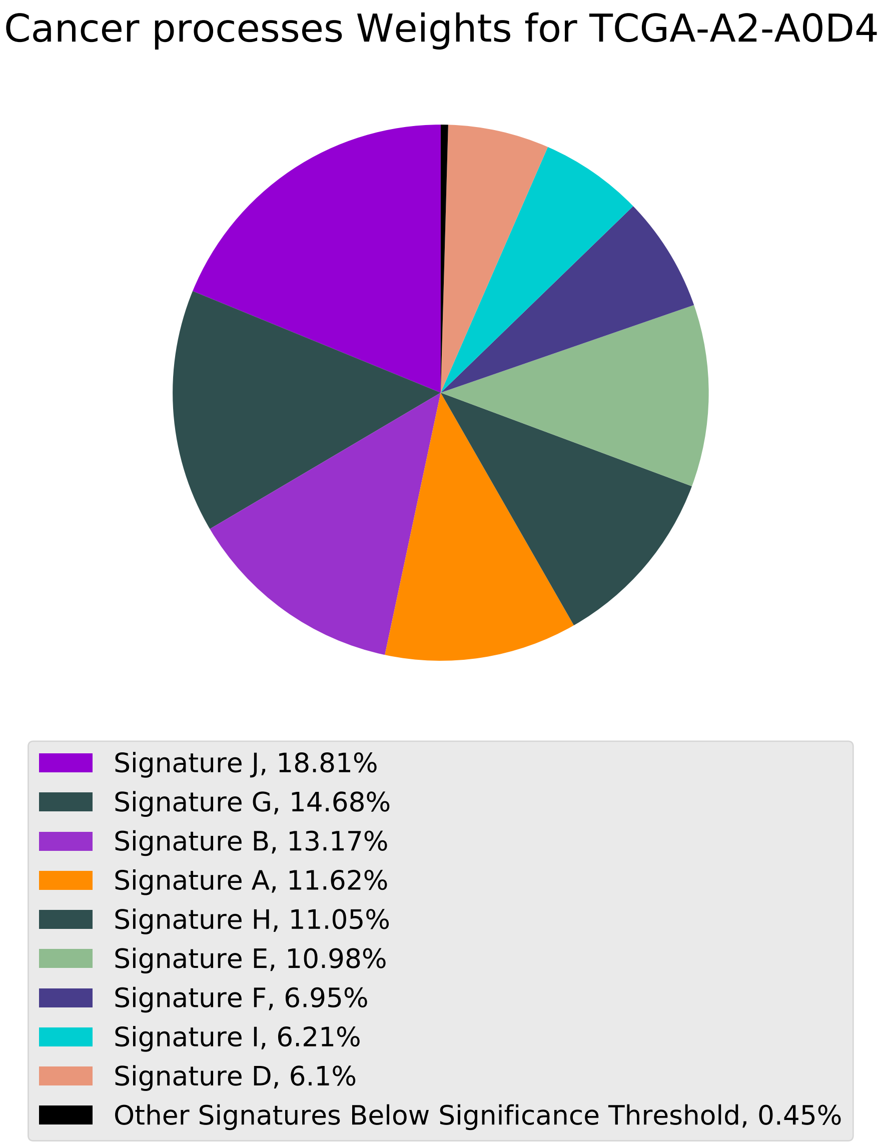
Cancer processes Weights for TCGA-A2-A0D4
Signature J, 18.81%
Signature G, 14.68%
Signature B, 13.17%
Signature A, 11.62%
Signature H, 11.05%
Signature E, 10.98%
Signature F, 6.95%
Signature I, 6.21%
Signature D, 6.1%
Other Signatures Below Significance Threshold, 0.45%

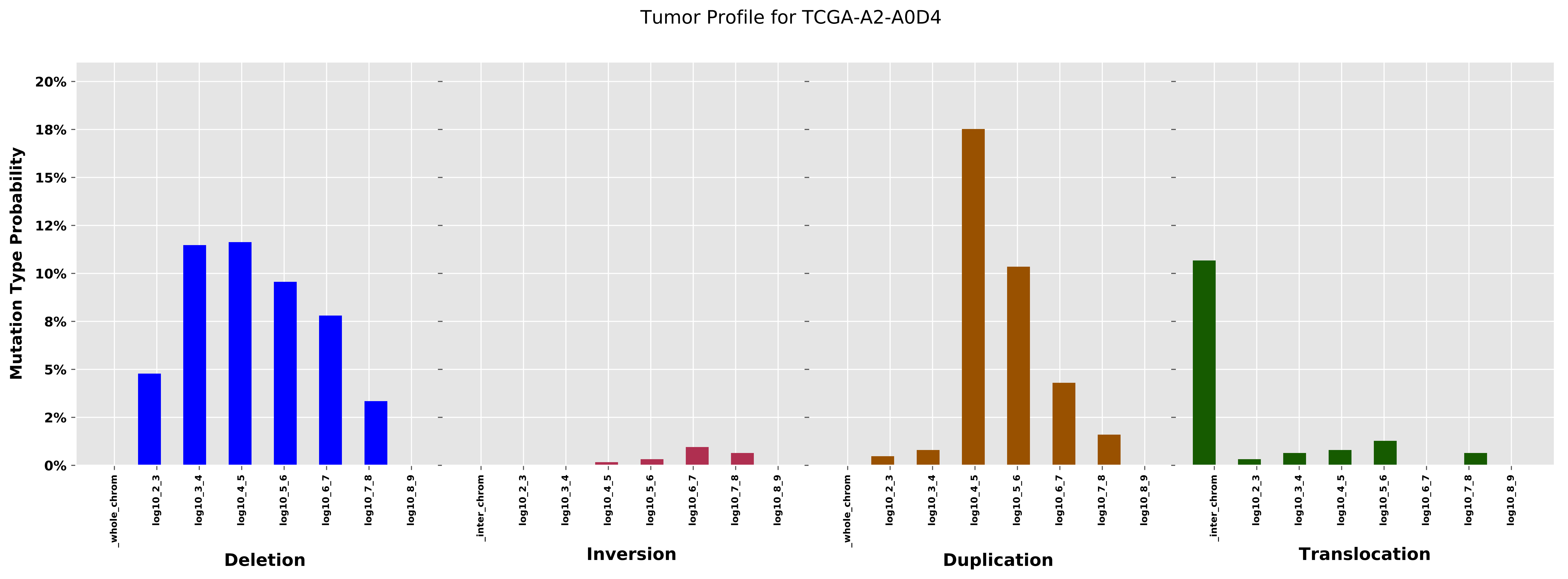
Tumor Profile for TCGA-A2-A0D4
Mutation Type Probability
20%
18%
15%
12%
10%
8%
5%
2%
0%
_whole_chrom
log10_2_3
log10_3_4
log10_4_5
log10_5_6
log10_6_7
log10_7_8
log10_8_9
_inter_chrom
Deletion
Inversion
Duplication
Translocation

Cancer processes Weights for TCGA-EW-A1P8

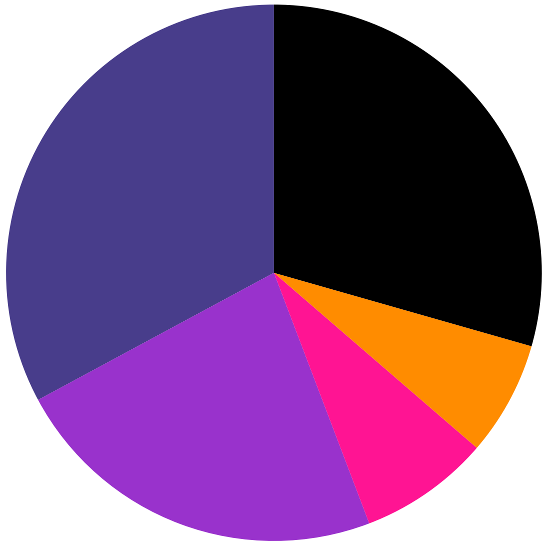

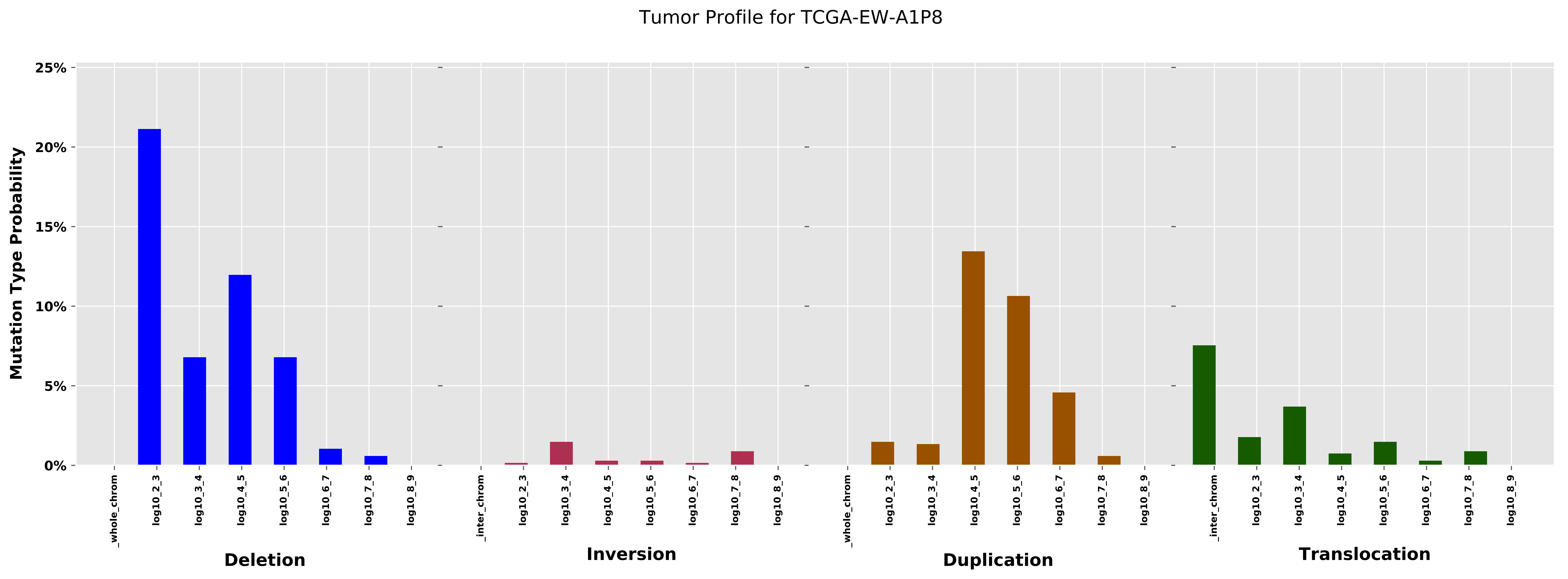

Tumor Profile for TCGA-EW-A1P8
Mutation Type Probability
25%
20%
15%
10%
5%
0%
_whole_chrom
log10_2_3
log10_3_4
log10_4_5
log10_5_6
log10_6_7
log10_7_8
log10_8_9
_inter_chrom
Deletion
Inversion
Duplication
Translocation

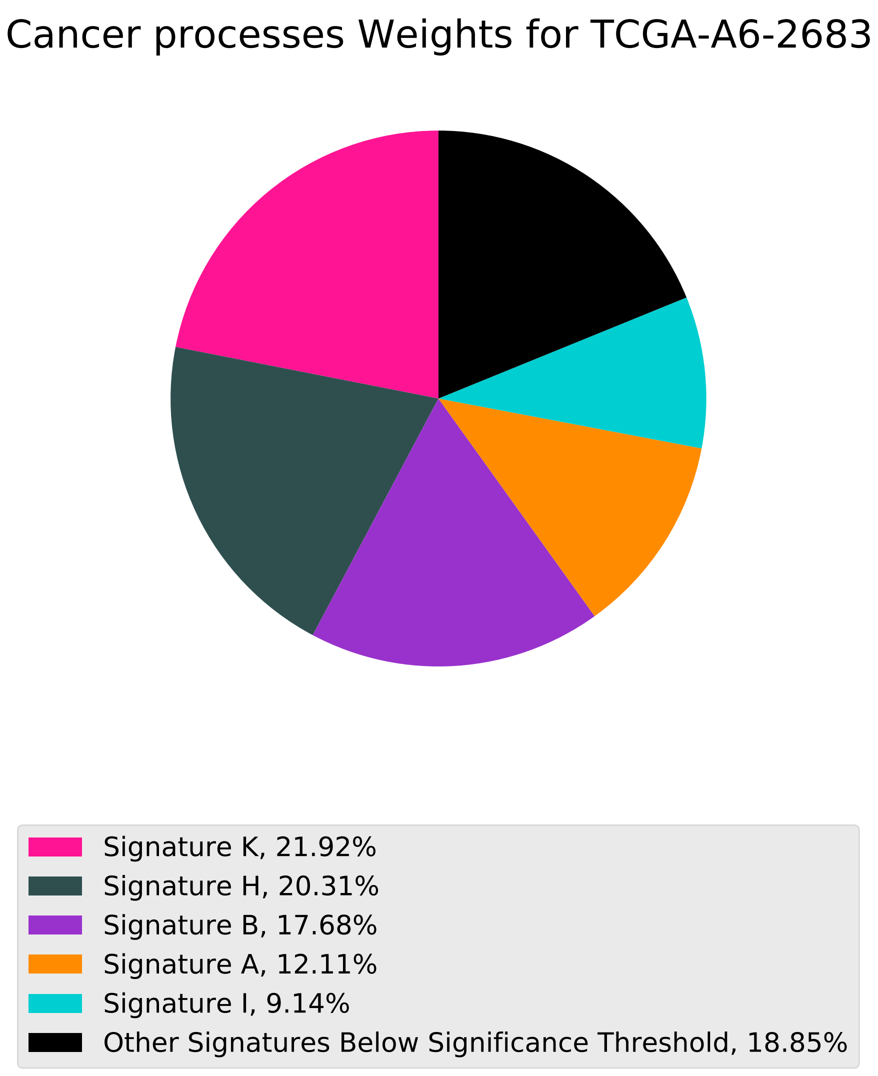
Cancer processes Weights for TCGA-A6-2683
Signature K, 21.92%
Signature H, 20.31%
Signature B, 17.68%
Signature A, 12.11%
Signature I, 9.14%
Other Signatures Below Significance Threshold, 18.85%

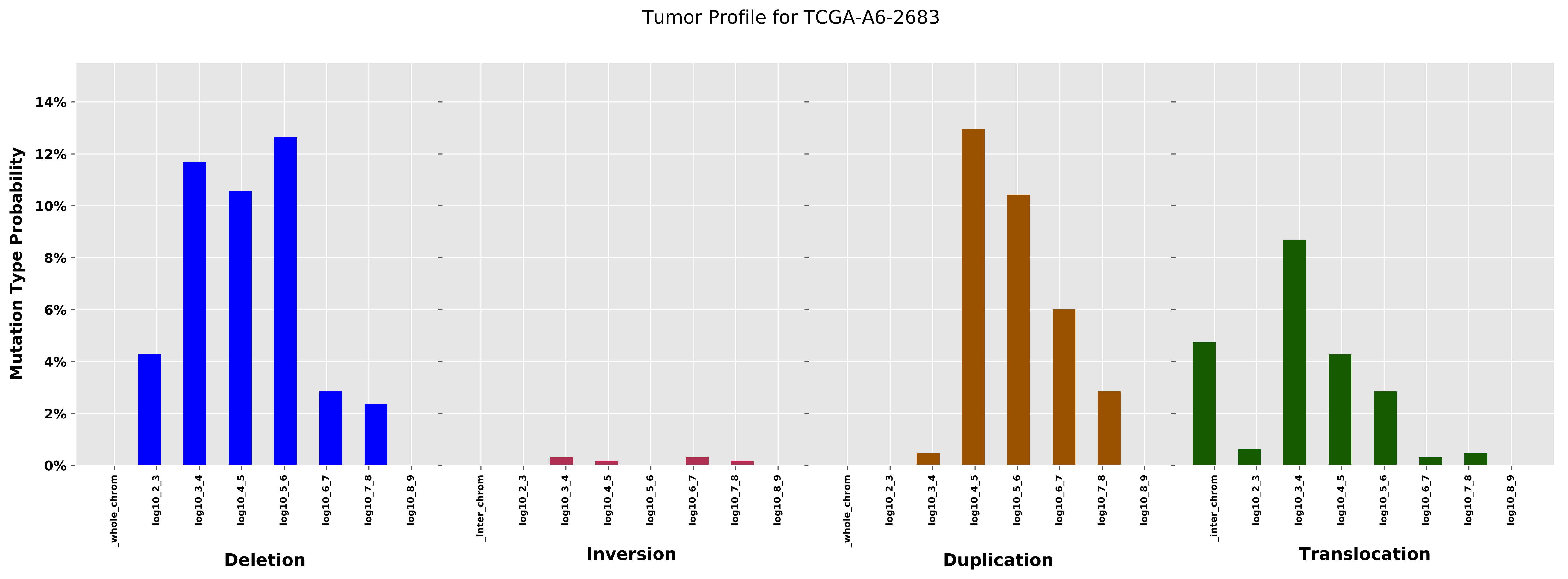

Tumor Profile for TCGA-A6-2683
Mutation Type Probability
0%
2%
4%
6%
8%
10%
12%
14%
_whole_chrom
log10_2_3
log10_3_4
log10_4_5
log10_5_6
log10_6_7
log10_7_8
log10_8_9
Deletion
_inter_chrom
log10_2_3
log10_3_4
log10_4_5
log10_5_6
log10_6_7
log10_7_8
log10_8_9
Inversion
_whole_chrom
log10_2_3
log10_3_4
log10_4_5
log10_5_6
log10_6_7
log10_7_8
log10_8_9
Duplication
_inter_chrom
log10_2_3
log10_3_4
log10_4_5
log10_5_6
log10_6_7
log10_7_8
log10_8_9
Translocation

Cancer processes Weights for TCGA-AC-A2BK

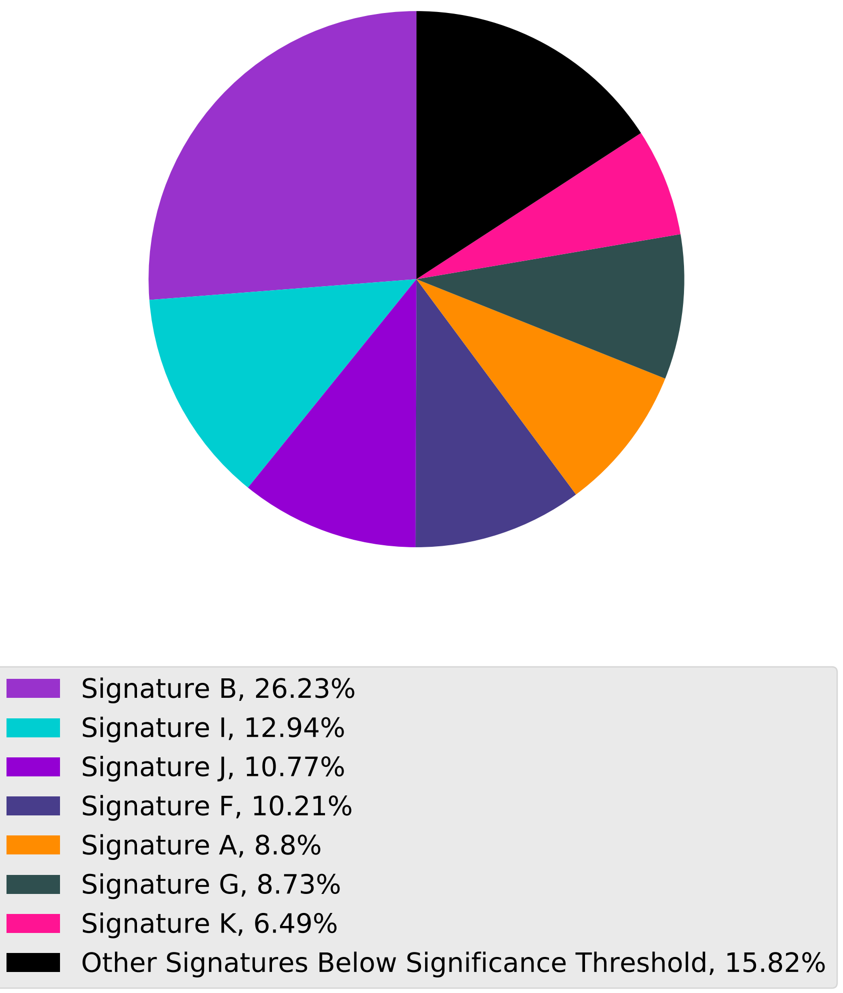

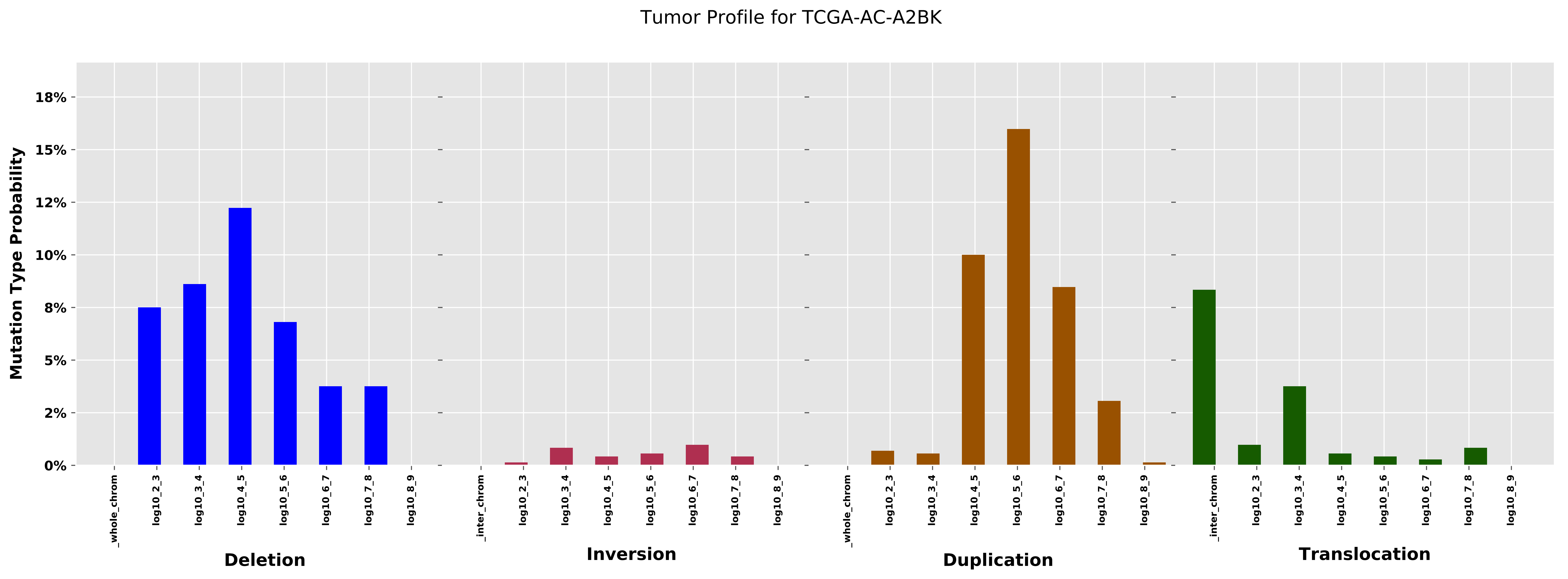

Tumor Profile for TCGA-AC-A2BK
Mutation Type Probability
18%
15%
12%
10%
8%
5%
2%
0%
_whole_chrom
log10_2_3
log10_3_4
log10_4_5
log10_5_6
log10_6_7
log10_7_8
log10_8_9
_inter_chrom
Deletion
Inversion
Duplication
Translocation

Cancer processes Weights for TCGA-AG-A032

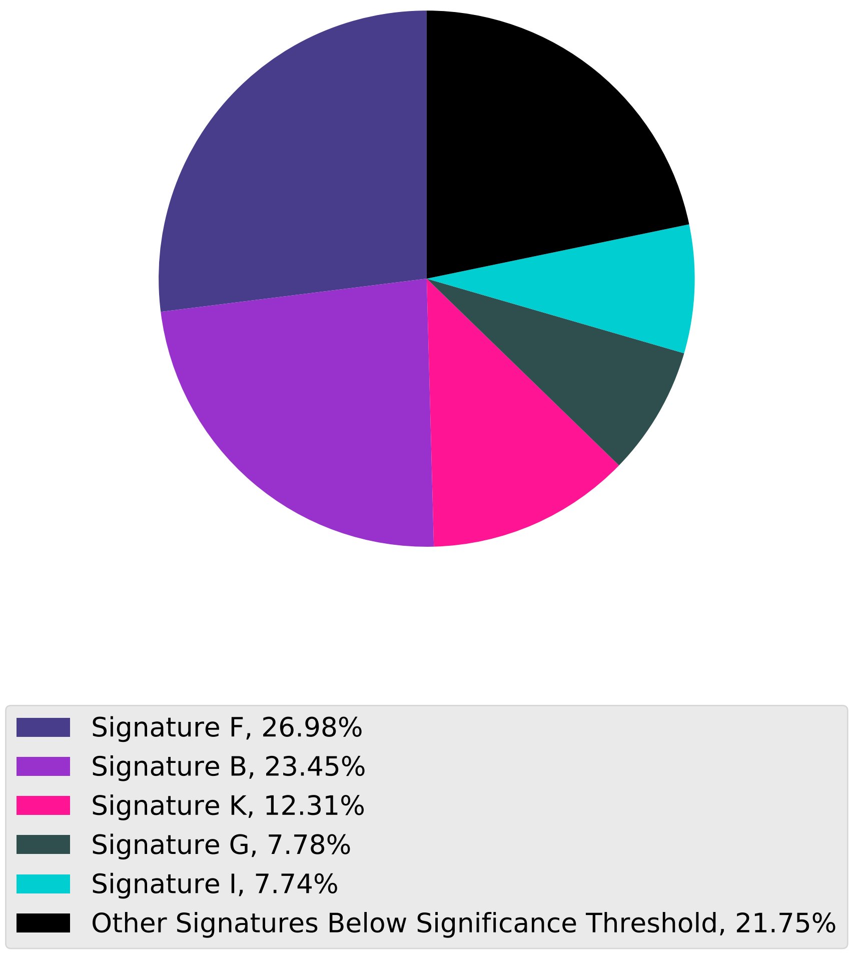

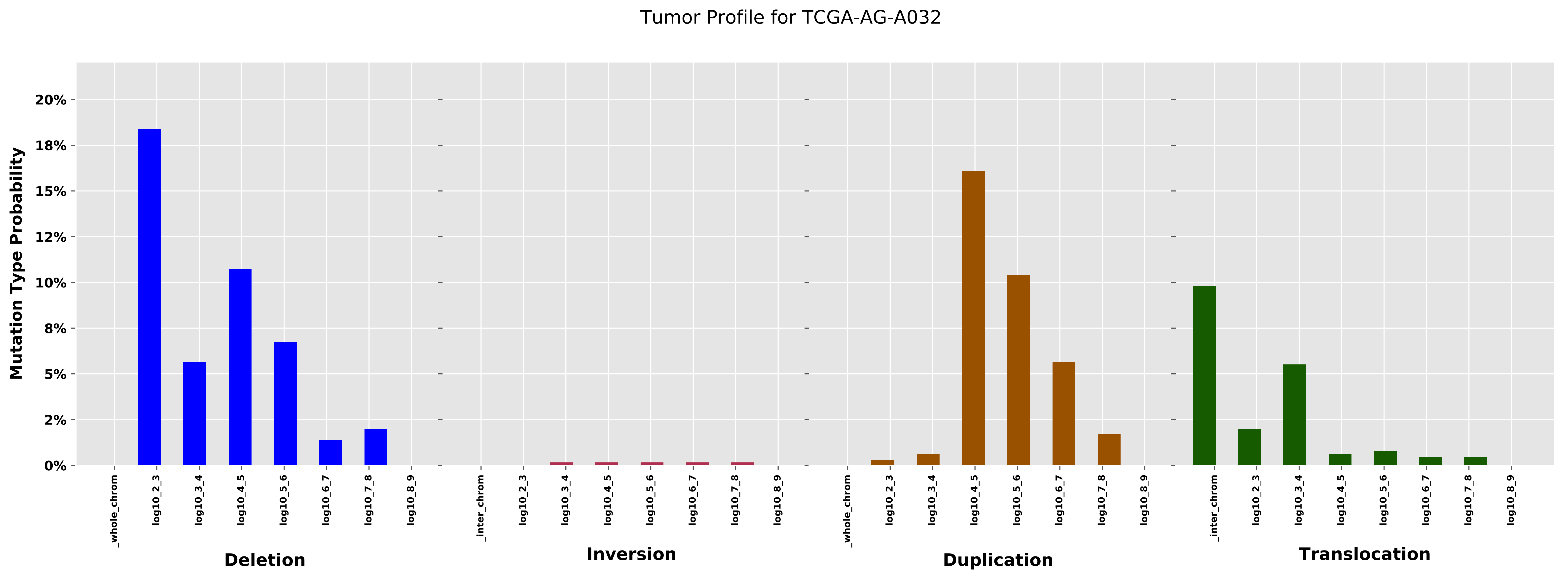

Tumor Profile for TCGA-AG-A032
Mutation Type Probability
20%
18%
15%
12%
10%
8%
5%
2%
0%
_whole_chrom
log10_2_3
log10_3_4
log10_4_5
log10_5_6
log10_6_7
log10_7_8
log10_8_9
_inter_chrom
Deletion
Inversion
Duplication
Translocation

Cancer processes Weights for TCGA-B6-A0I2

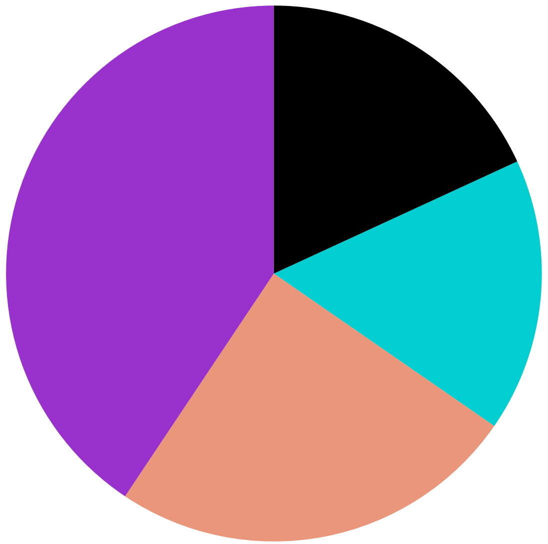

Signature B, 40.63%
Signature D, 24.74%
Signature I, 16.5%
Other Signatures Below Significance Threshold, 18.13%

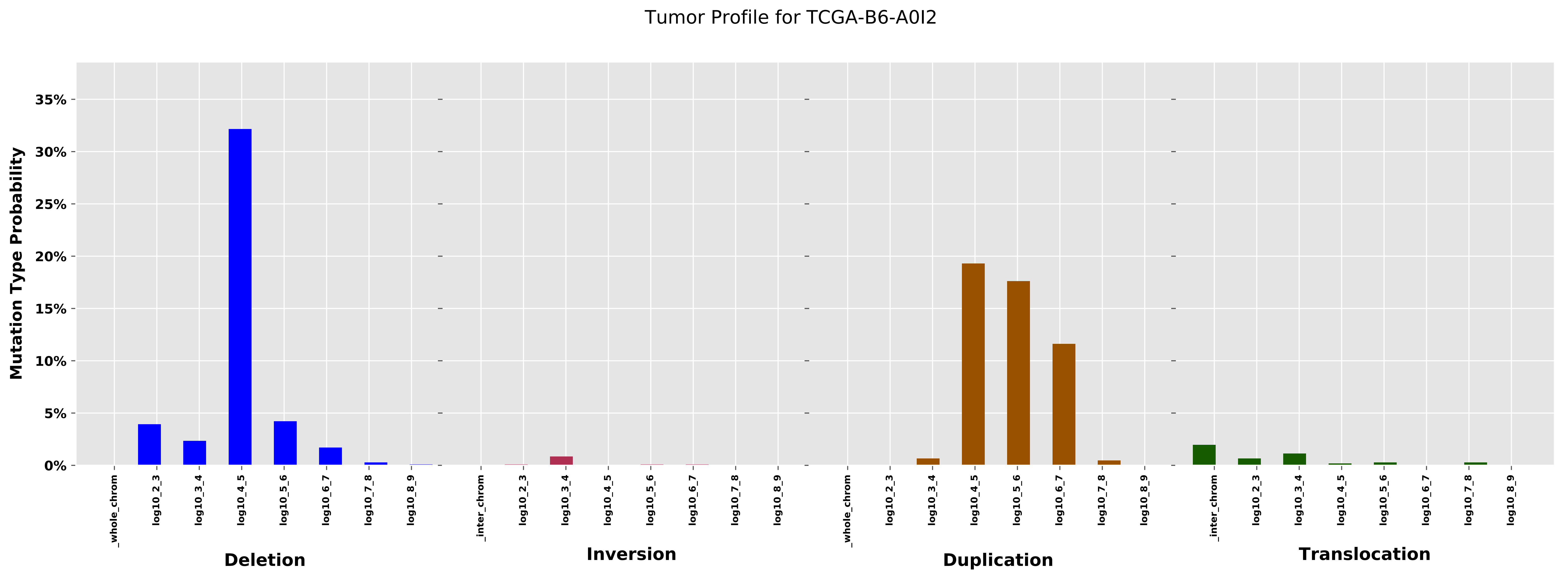

Tumor Profile for TCGA-B6-A0I2
Mutation Type Probability
35%
30%
25%
20%
15%
10%
5%
0%
_whole_chrom
log10_2_3
log10_3_4
log10_4_5
log10_5_6
log10_6_7
log10_7_8
log10_8_9
_inter_chrom
Deletion
Inversion
Duplication
Translocation

Cancer processes Weights for TCGA-A8-A094

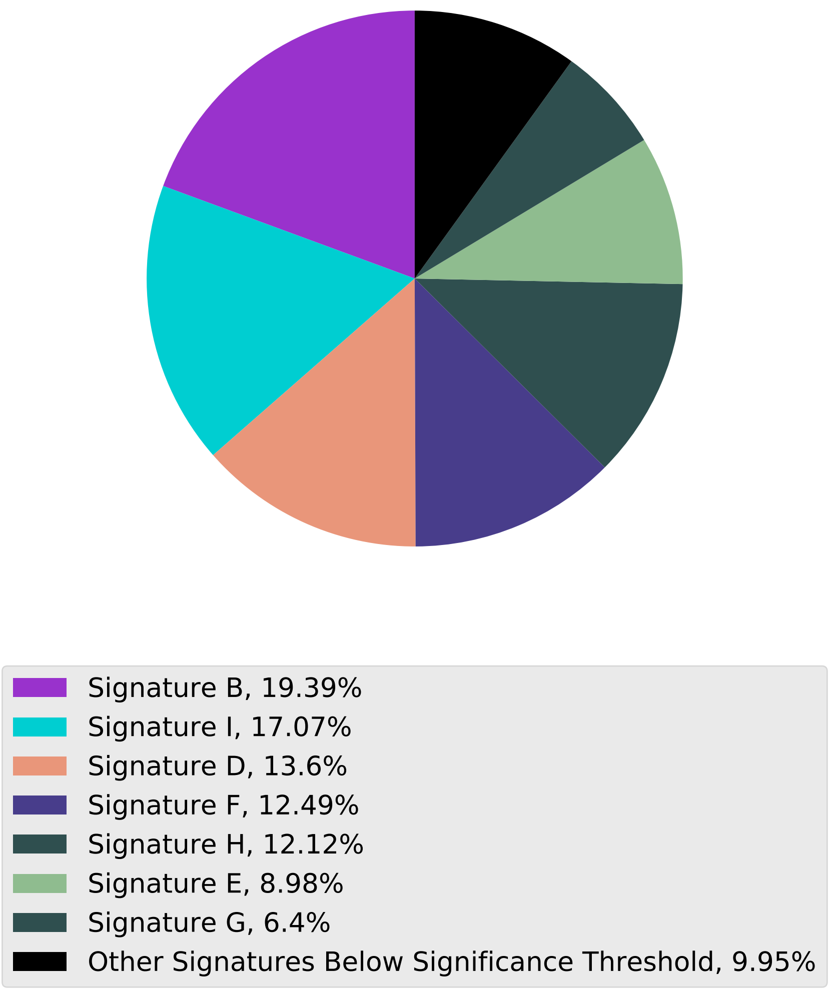

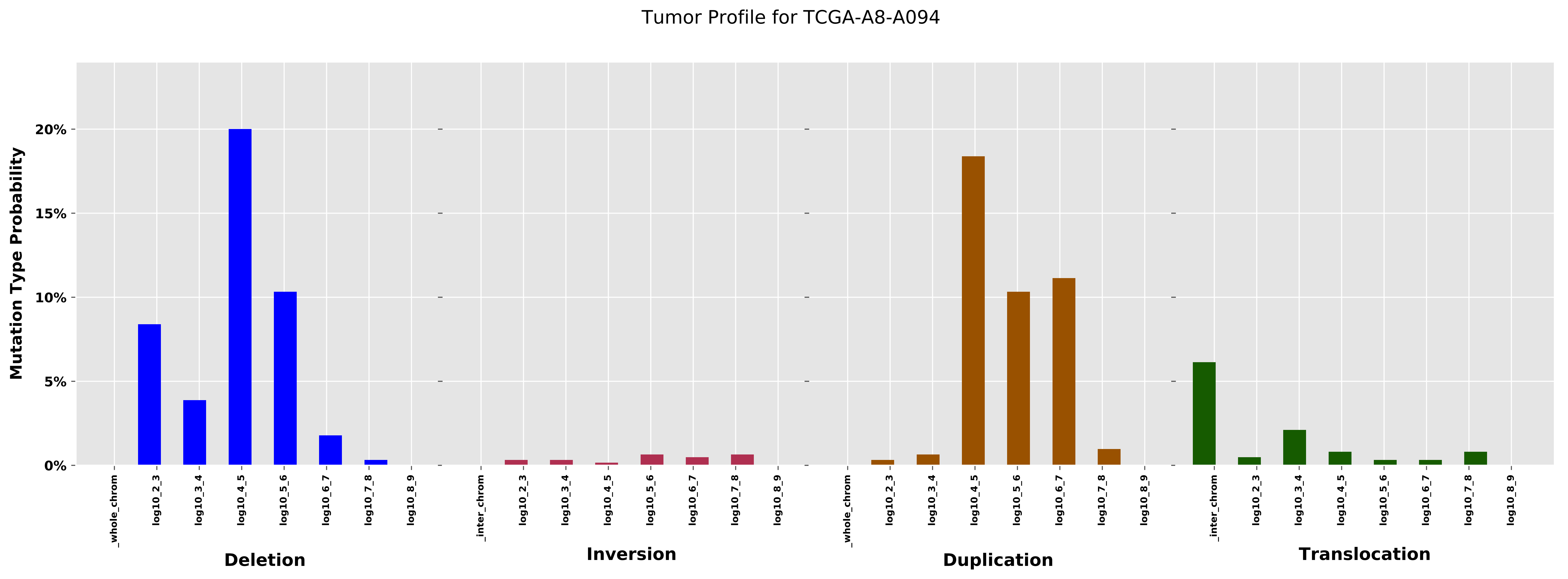
Tumor Profile for TCGA-A8-A094
Mutation Type Probability
20%
15%
10%
5%
0%
_whole_chrom
log10_2_3
log10_3_4
log10_4_5
log10_5_6
log10_6_7
log10_7_8
log10_8_9
_inter_chrom
Deletion
Inversion
Duplication
Translocation

Cancer processes Weights for TCGA-AA-3994

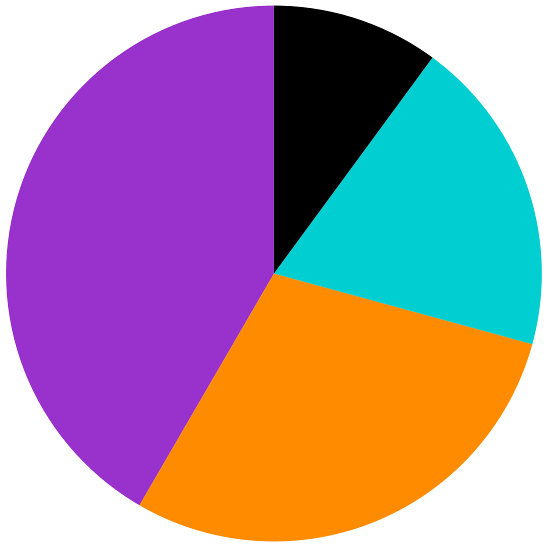

Signature B, 41.63%
Signature A, 29.13%
Signature I, 19.17%
Other Signatures Below Significance Threshold, 10.08%

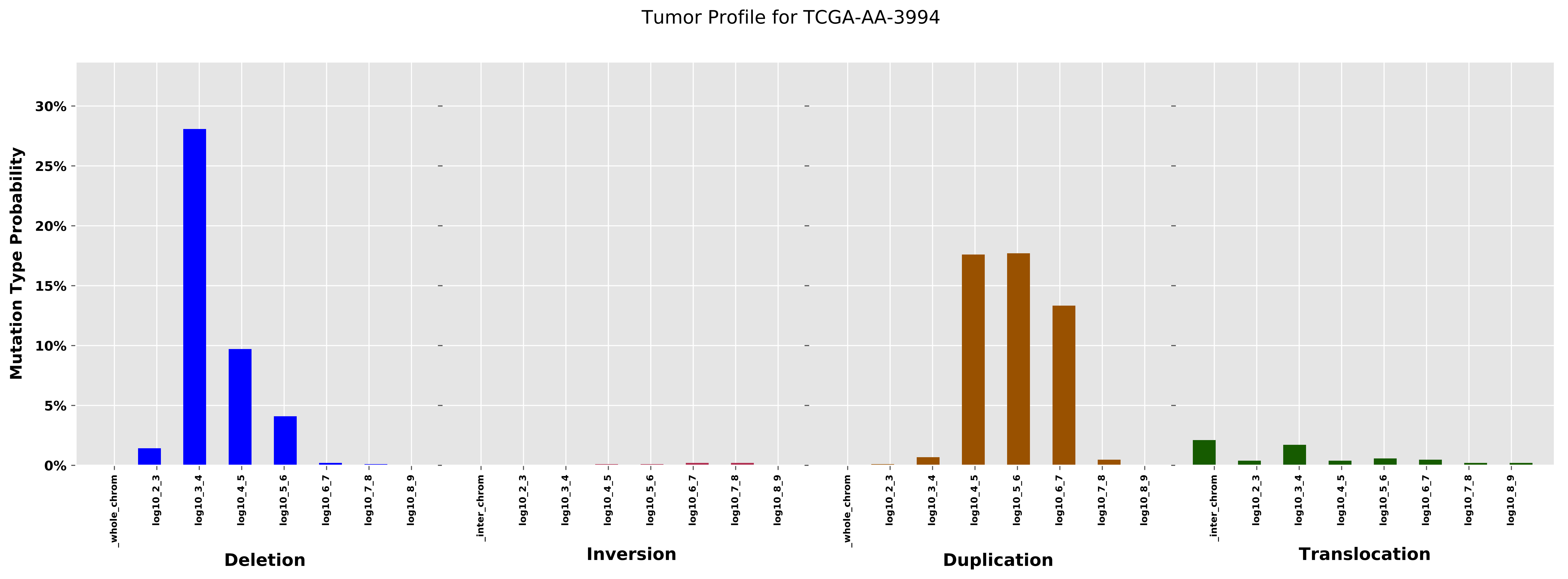
Tumor Profile for TCGA-AA-3994
Mutation Type Probability
30%
25%
20%
15%
10%
5%
0%
_whole_chrom
log10_2_3
log10_3_4
log10_4_5
log10_5_6
log10_6_7
log10_7_8
log10_8_9
Deletion
_inter_chrom
Inversion
Duplication
Translocation

Cancer processes Weights for TCGA-AG-3574

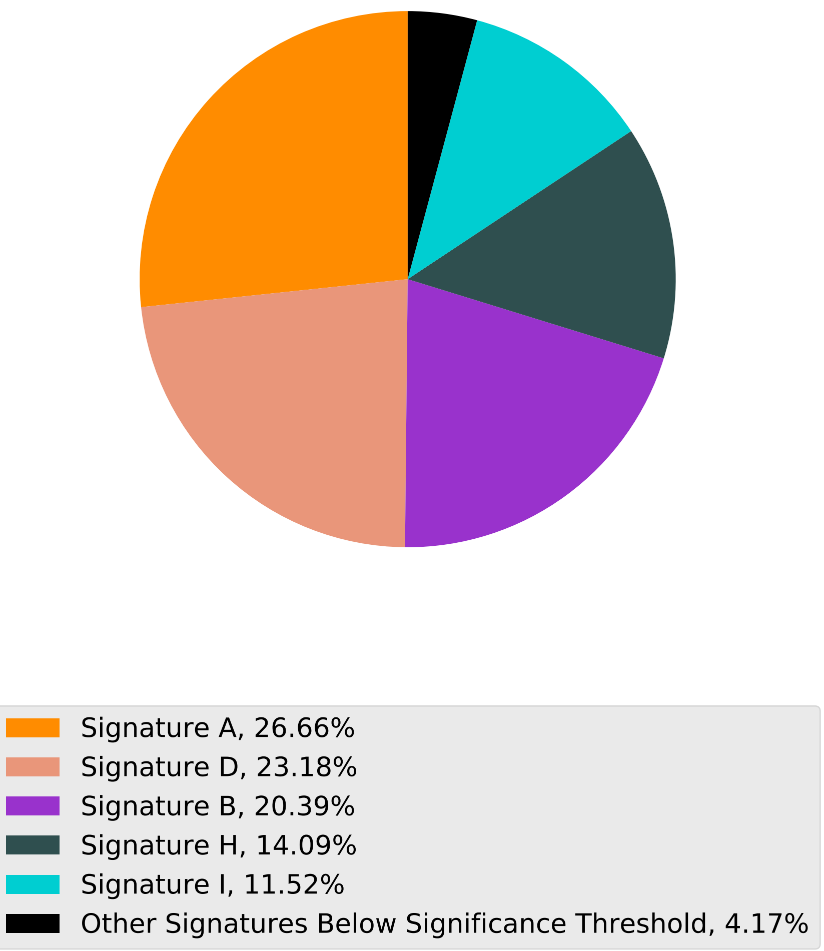

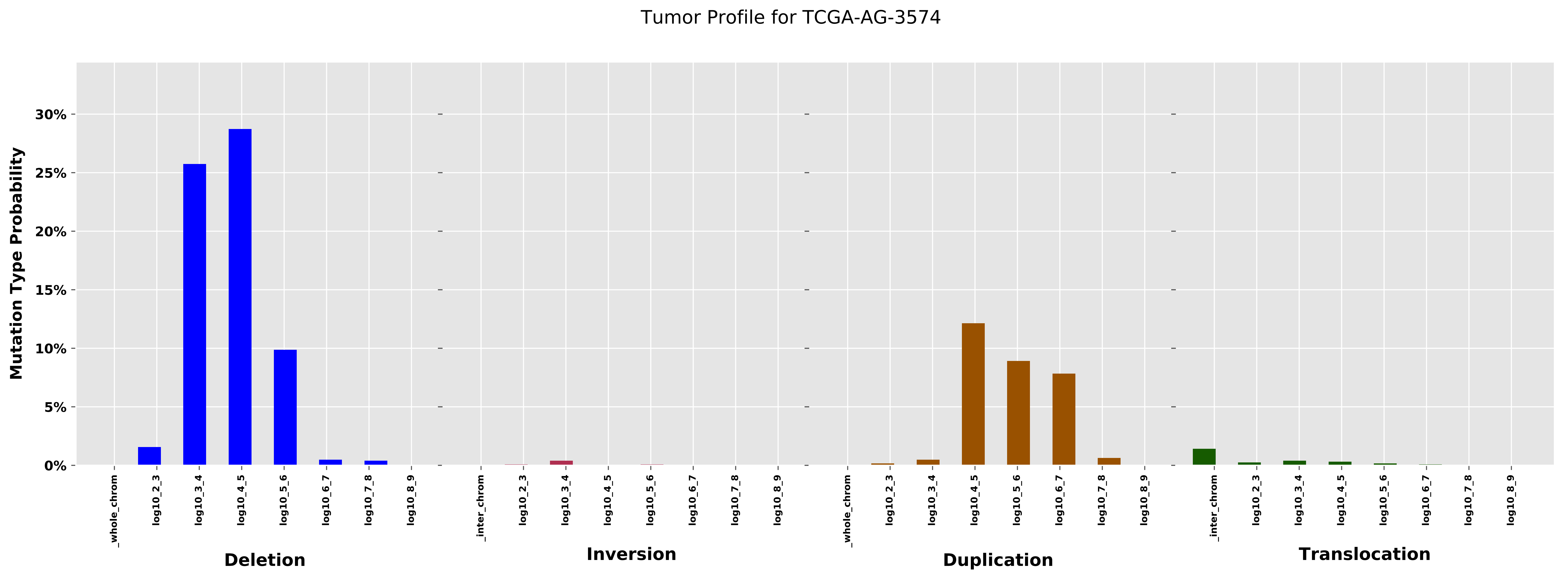
Tumor Profile for TCGA-AG-3574
Mutation Type Probability
30%
25%
20%
15%
10%
5%
0%
_whole_chrom
log10_2_3
log10_3_4
log10_4_5
log10_5_6
log10_6_7
log10_7_8
log10_8_9
Deletion
_inter_chrom
Inversion
Duplication
Translocation

Cancer processes Weights for TCGA-AO-A03L

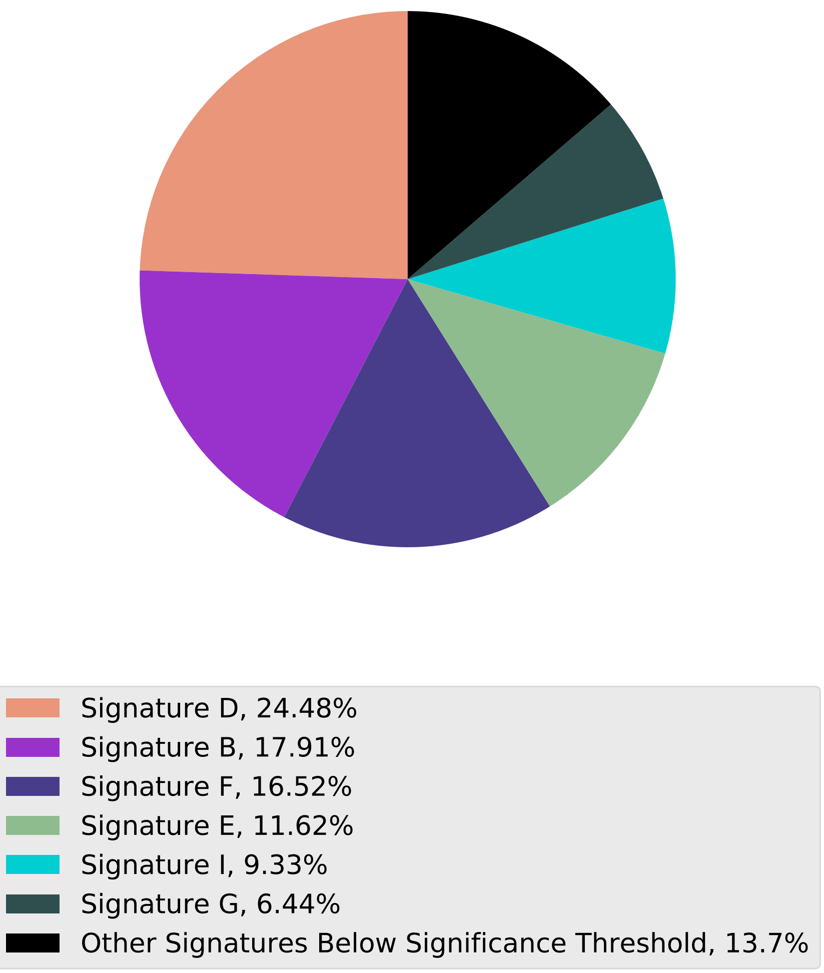

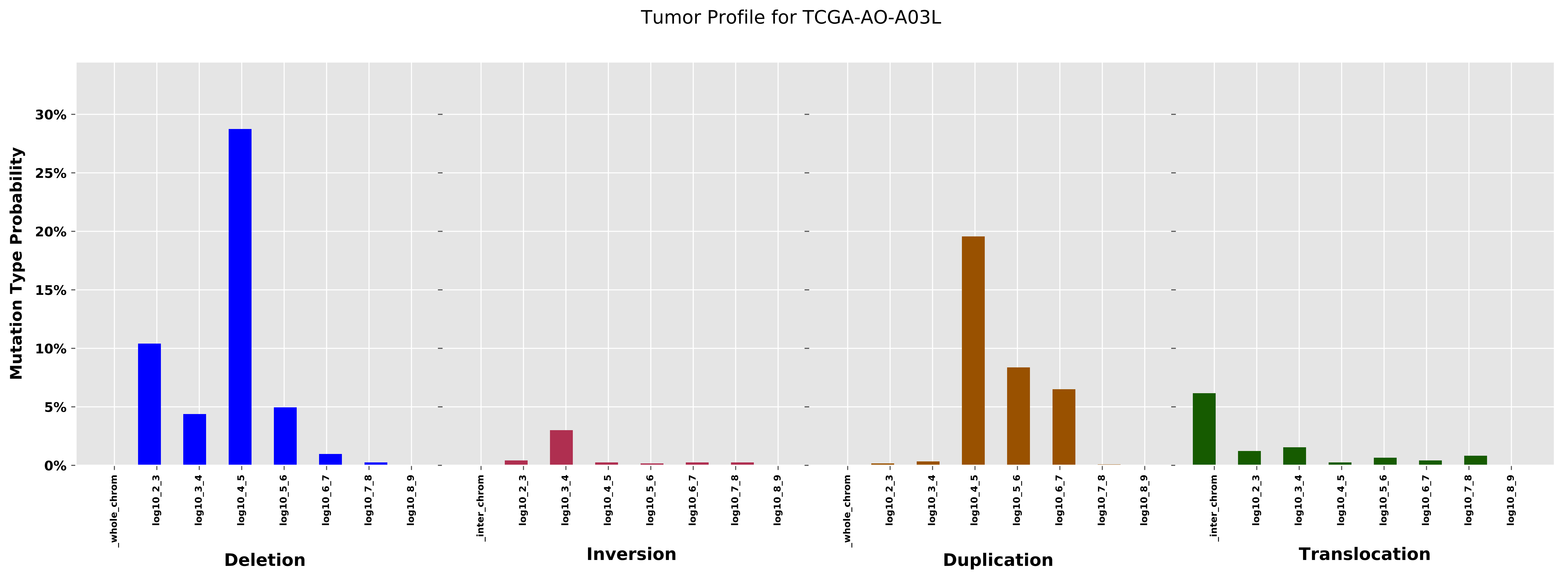
Tumor Profile for TCGA-AO-A03L
Mutation Type Probability
0%
5%
10%
15%
20%
25%
30%
_whole_chrom
log10_2_3
log10_3_4
log10_4_5
log10_5_6
log10_6_7
log10_7_8
log10_8_9
_inter_chrom
Deletion
Inversion
Duplication
Translocation

Cancer processes Weights for TCGA-A6-6141

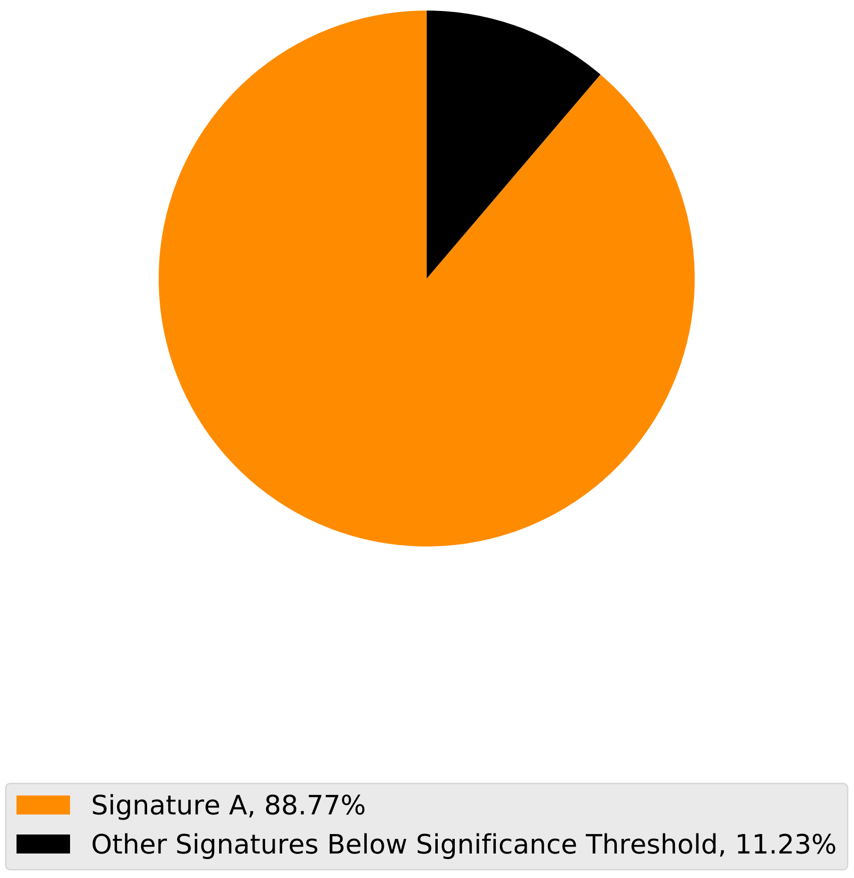

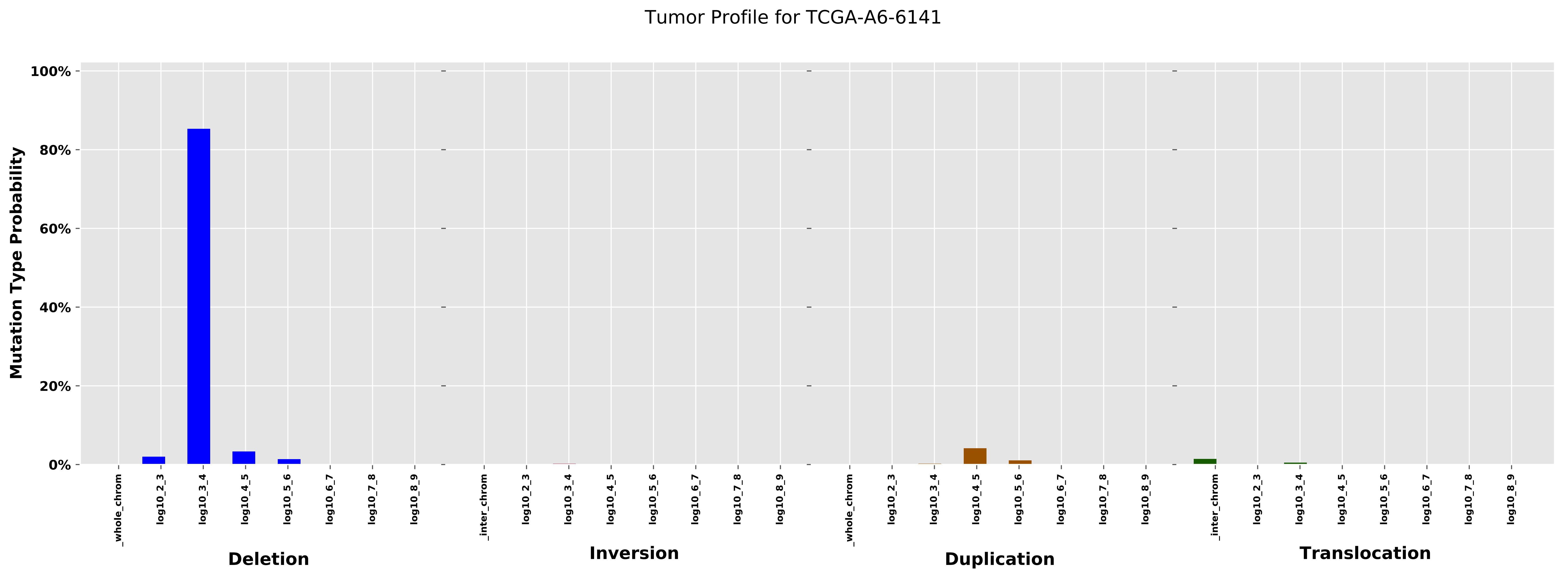

Tumor Profile for TCGA-A6-6141
Mutation Type Probability
100%
80%
60%
40%
20%
0%
_whole_chrom
log10_2_3
log10_3_4
log10_4_5
log10_5_6
log10_6_7
log10_7_8
log10_8_9
_inter_chrom
Deletion
Inversion
Duplication
Translocation

Cancer processes Weights for TCGA-AA-A02O

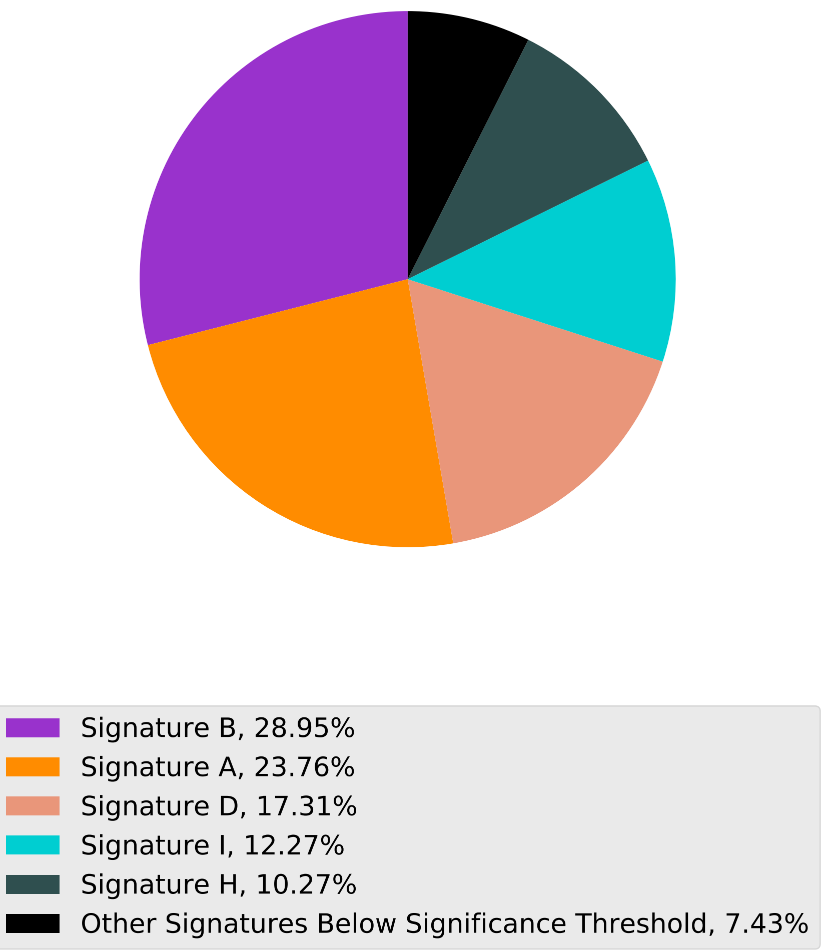

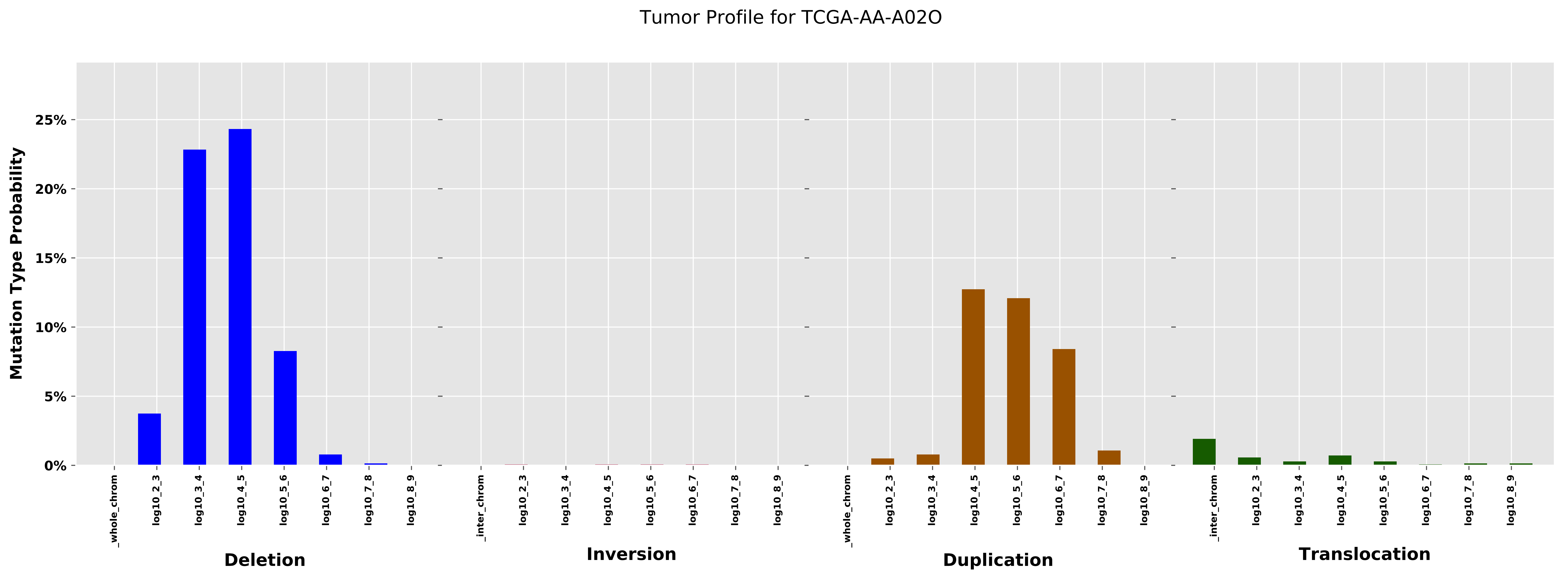
Tumor Profile for TCGA-AA-A02O
Mutation Type Probability
0%
5%
10%
15%
20%
25%
_whole_chrom
log10_2_3
log10_3_4
log10_4_5
log10_5_6
log10_6_7
log10_7_8
log10_8_9
_inter_chrom
Deletion
Inversion
Duplication
Translocation

Cancer processes Weights for TCGA-AG-3885

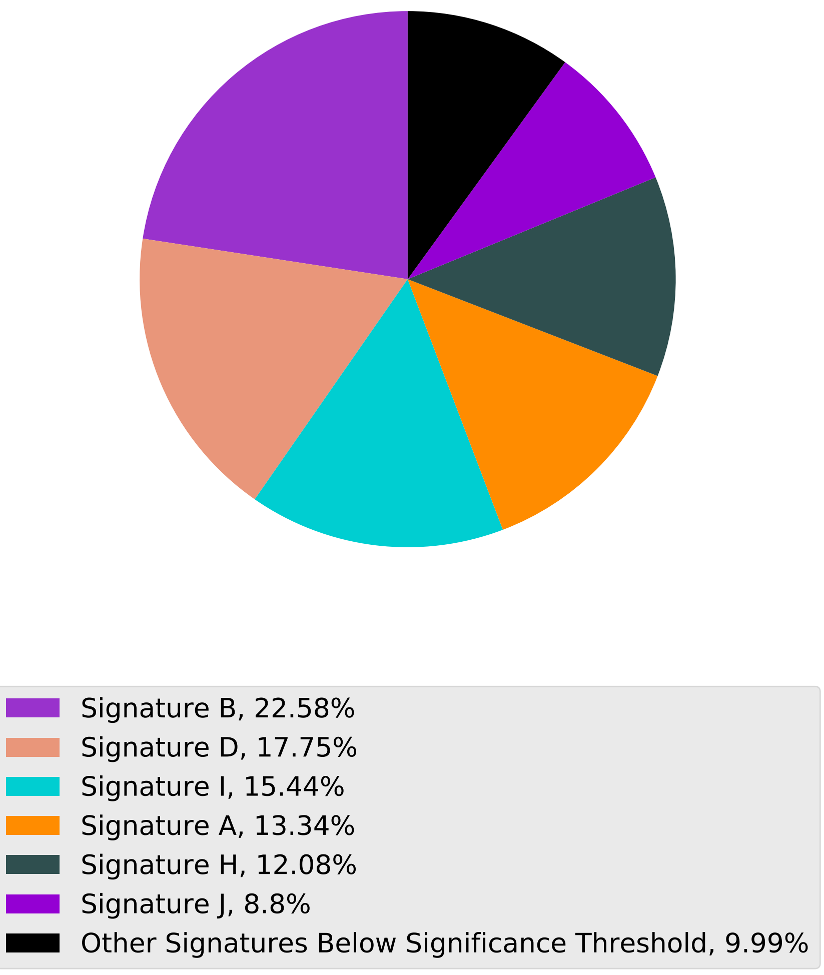

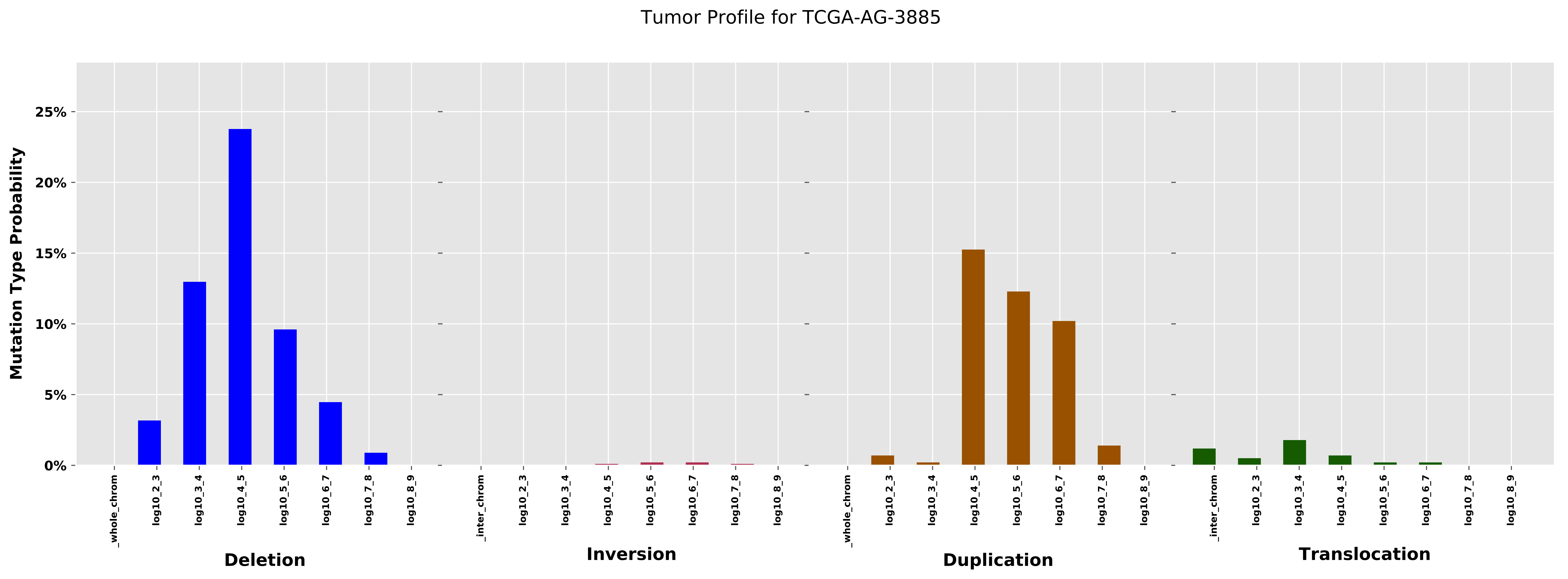

Tumor Profile for TCGA-AG-3885
Mutation Type Probability
25%
20%
15%
10%
5%
0%
_whole_chrom
log10_2_3
log10_3_4
log10_4_5
log10_5_6
log10_6_7
log10_7_8
log10_8_9
Deletion
_inter_chrom
Inversion
Duplication
Translocation

Cancer processes Weights for TCGA-AA-A01T

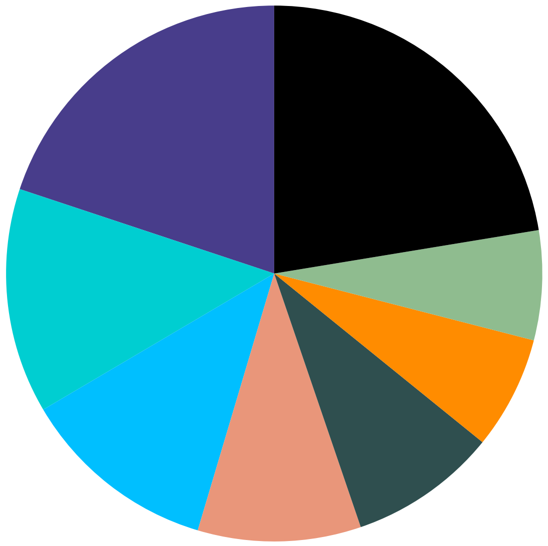

- Signature F, 19.9%
- Signature I, 13.58%
- Signature L, 11.92%
- Signature D, 9.84%
- Signature H, 8.93%
- Signature A, 6.82%
- Signature E, 6.61%
- Other Signatures Below Significance Threshold, 22.4%

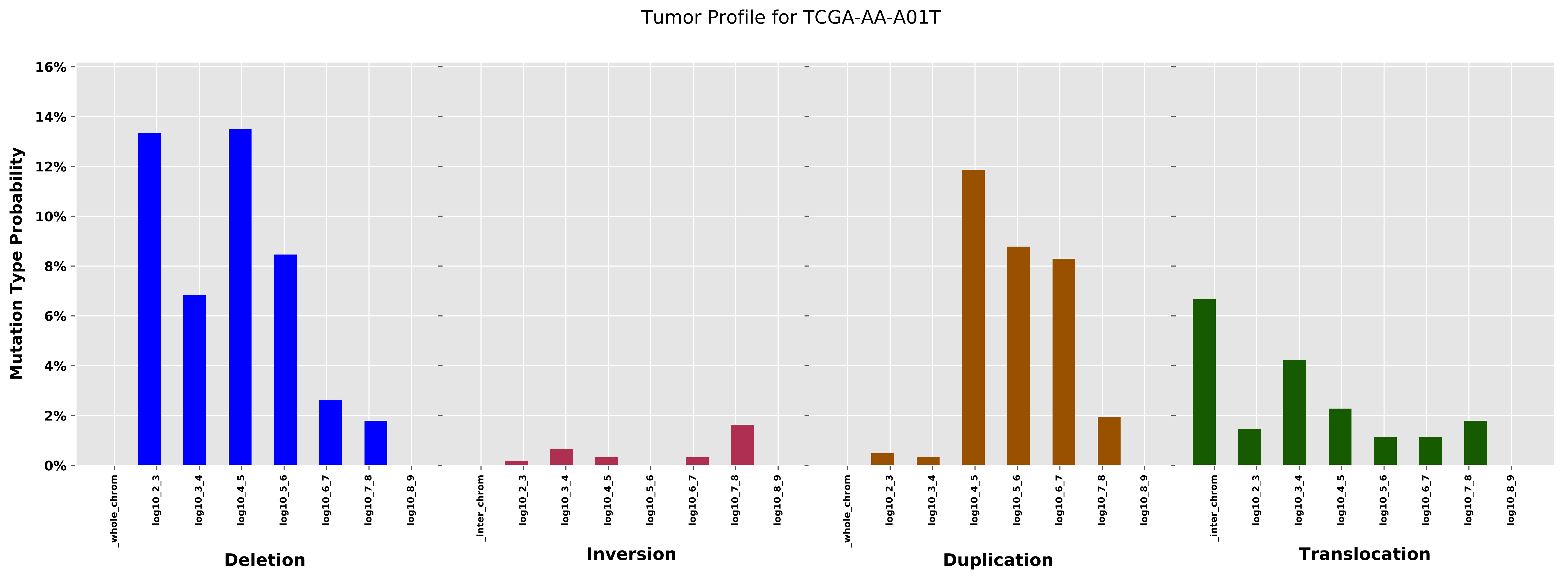

Tumor Profile for TCGA-AA-A01T
Mutation Type Probability
16%
14%
12%
10%
8%
6%
4%
2%
0%
_whole_chrom
log10_2_3
log10_3_4
log10_4_5
log10_5_6
log10_6_7
log10_7_8
log10_8_9
Deletion
_inter_chrom
log10_2_3
log10_3_4
log10_4_5
log10_5_6
log10_6_7
log10_7_8
log10_8_9
Inversion
_whole_chrom
log10_2_3
log10_3_4
log10_4_5
log10_5_6
log10_6_7
log10_7_8
log10_8_9
Duplication
_inter_chrom
log10_2_3
log10_3_4
log10_4_5
log10_5_6
log10_6_7
log10_7_8
log10_8_9
Translocation

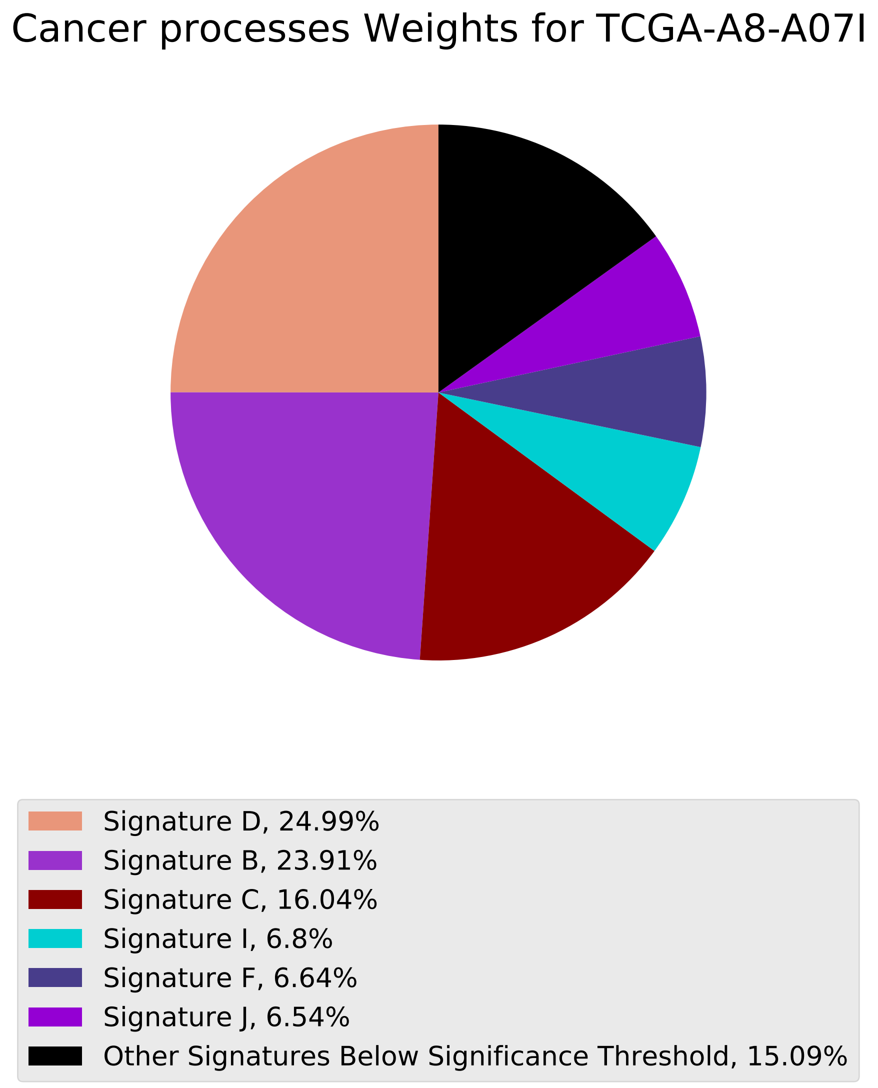
Cancer processes Weights for TCGA-A8-A07I
Signature D, 24.99%
Signature B, 23.91%
Signature C, 16.04%
Signature I, 6.8%
Signature F, 6.64%
Signature J, 6.54%
Other Signatures Below Significance Threshold, 15.09%

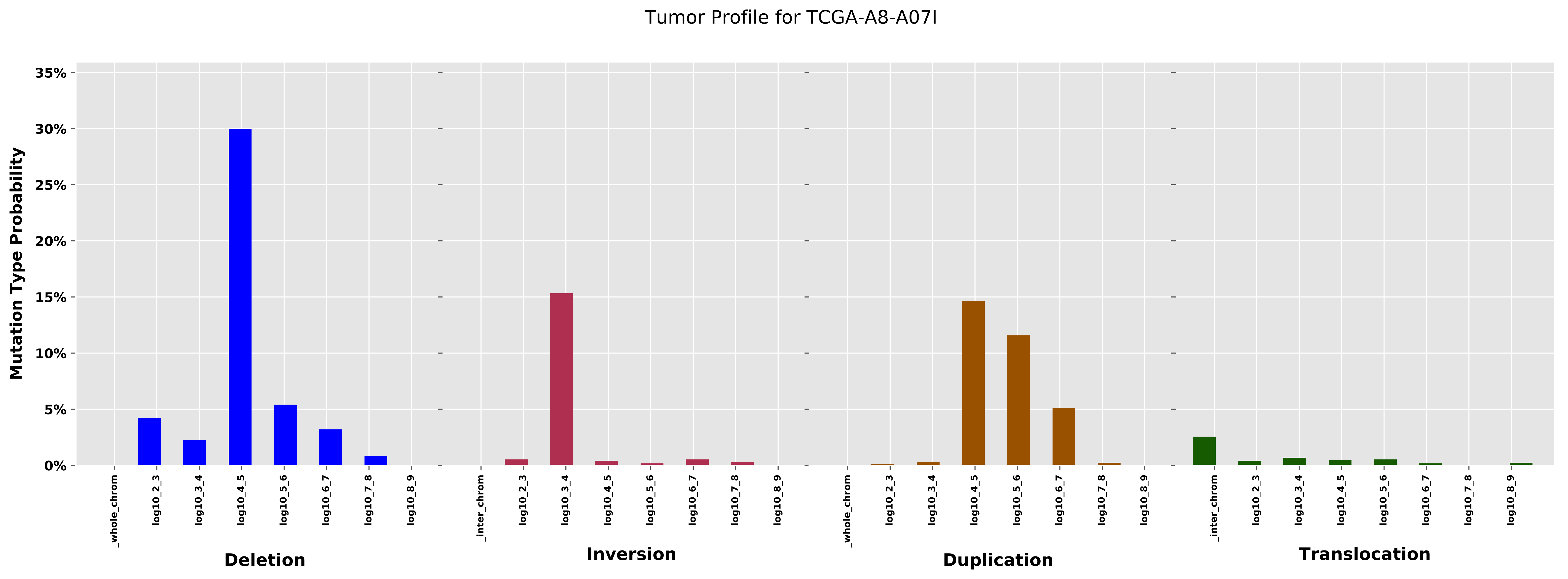
Tumor Profile for TCGA-A8-A07I
Mutation Type Probability
0%
5%
10%
15%
20%
25%
30%
35%
_whole_chrom
log10_2_3
log10_3_4
log10_4_5
log10_5_6
log10_6_7
log10_7_8
log10_8_9
_inter_chrom
Deletion
Inversion
Duplication
Translocation

Cancer processes Weights for TCGA-A6-2681

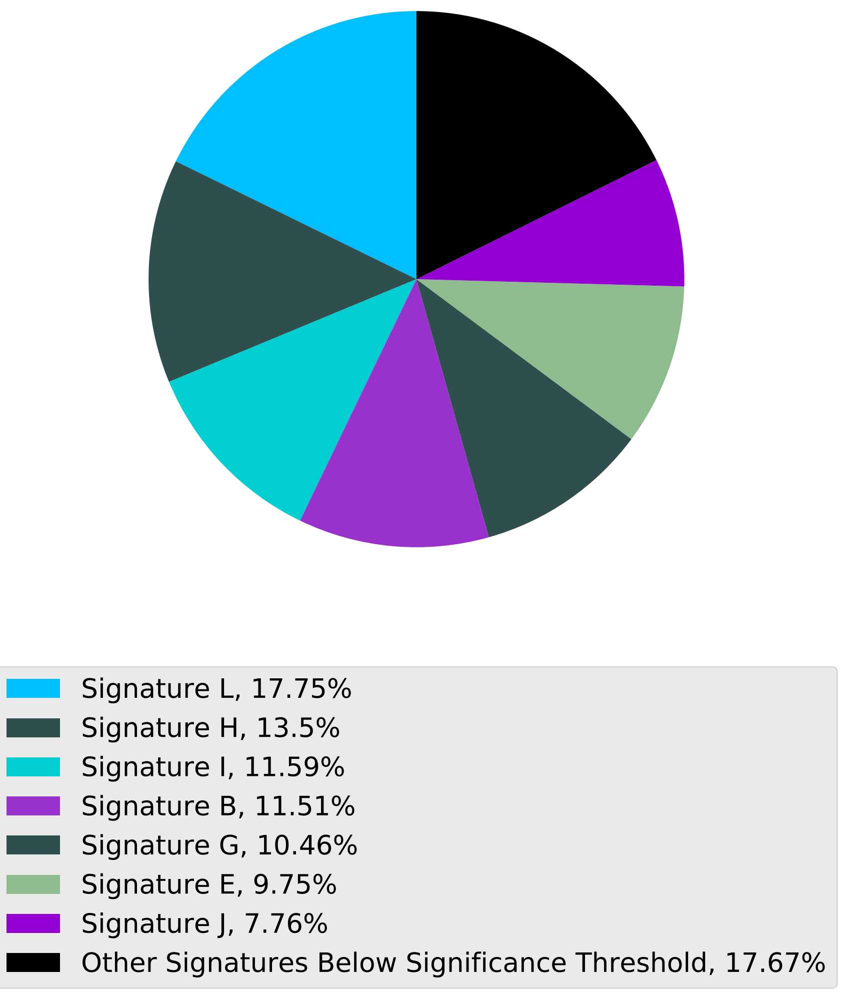

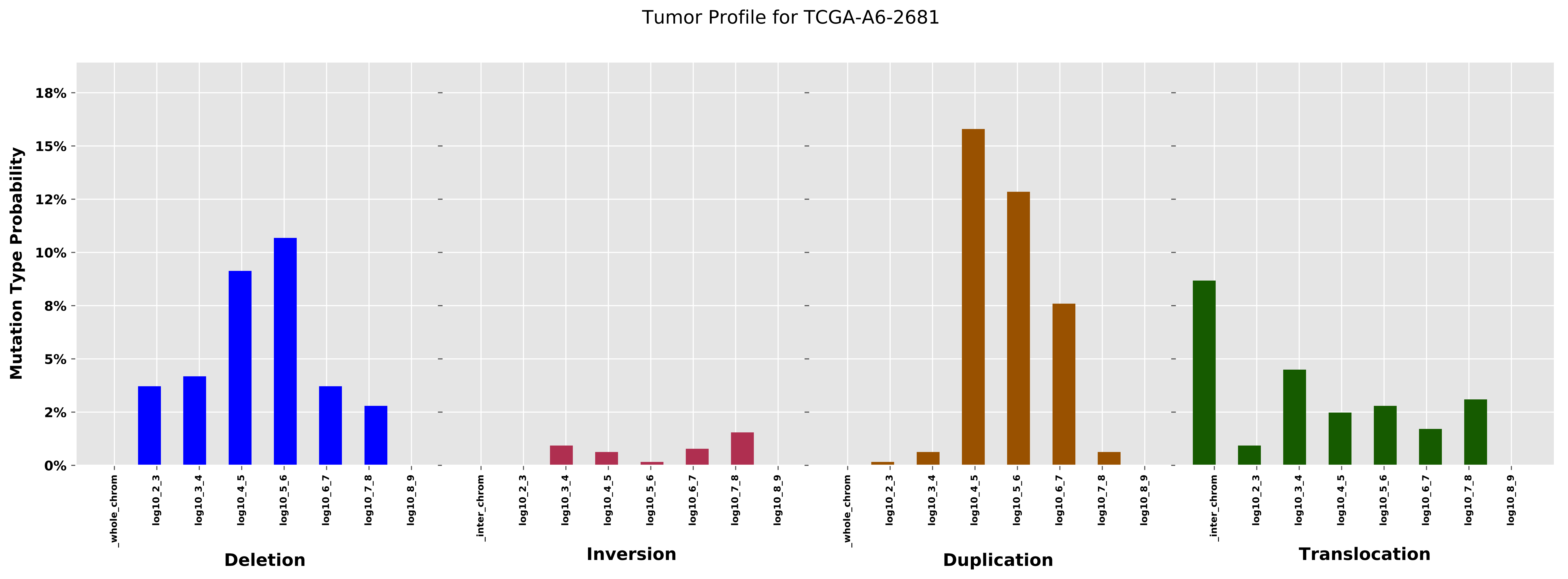

Tumor Profile for TCGA-A6-2681
Mutation Type Probability
0%
2%
5%
8%
10%
12%
15%
18%
_whole_chrom
log10_2_3
log10_3_4
log10_4_5
log10_5_6
log10_6_7
log10_7_8
log10_8_9
_inter_chrom
Deletion
Inversion
Duplication
Translocation

Cancer processes Weights for TCGA-AO-A124

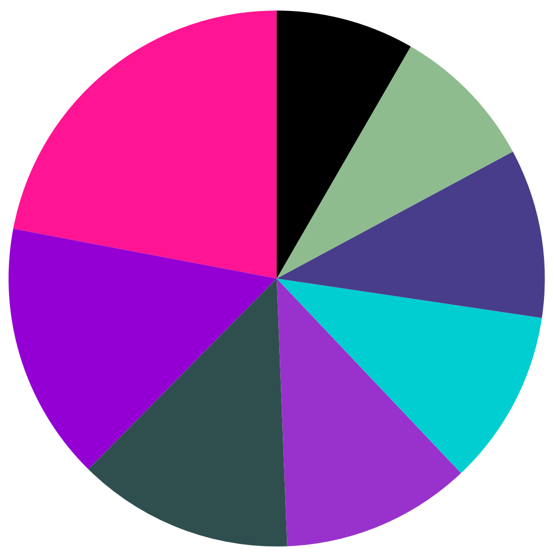

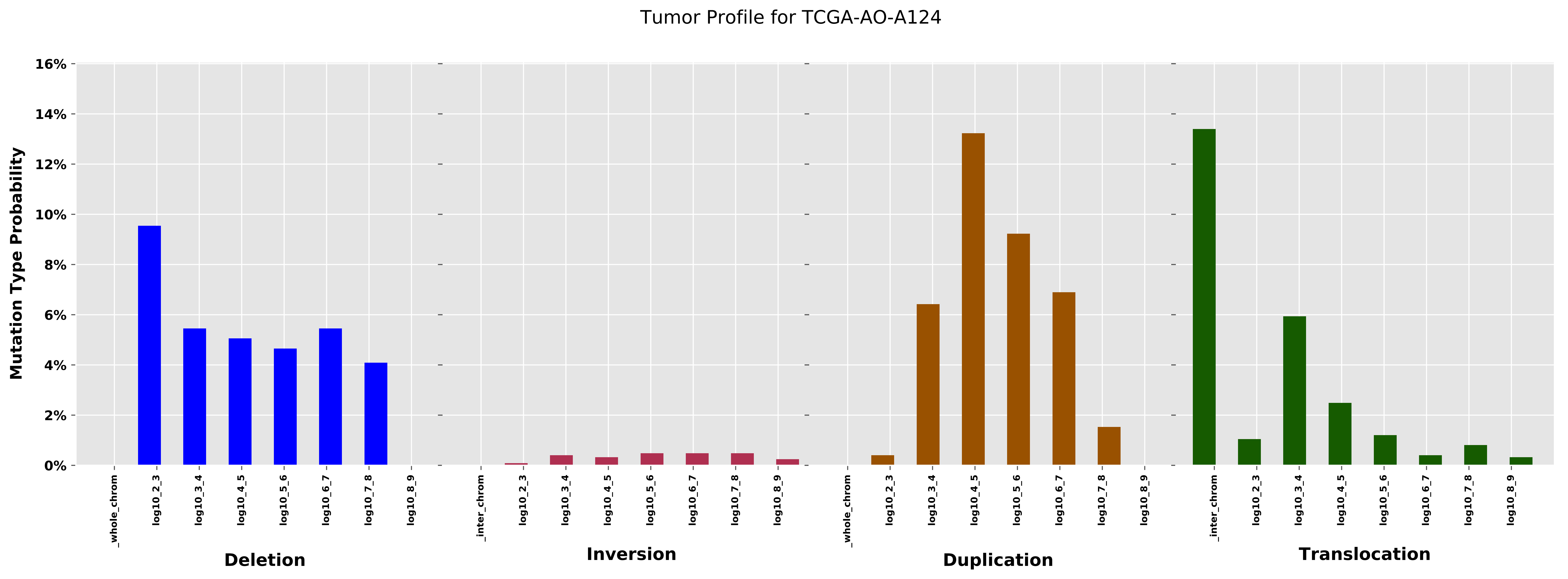

Tumor Profile for TCGA-AO-A124
Mutation Type Probability
16%
14%
12%
10%
8%
6%
4%
2%
0%
_whole_chrom
log10_2_3
log10_3_4
log10_4_5
log10_5_6
log10_6_7
log10_7_8
log10_8_9
_inter_chrom
Deletion
Inversion
Duplication
Translocation

Cancer processes Weights for TCGA-A2-A259

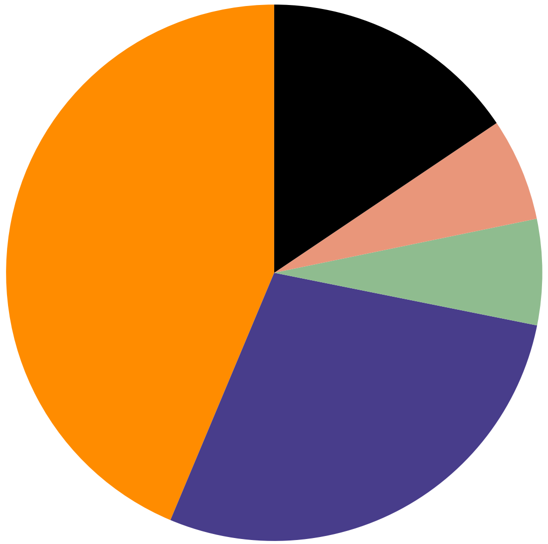

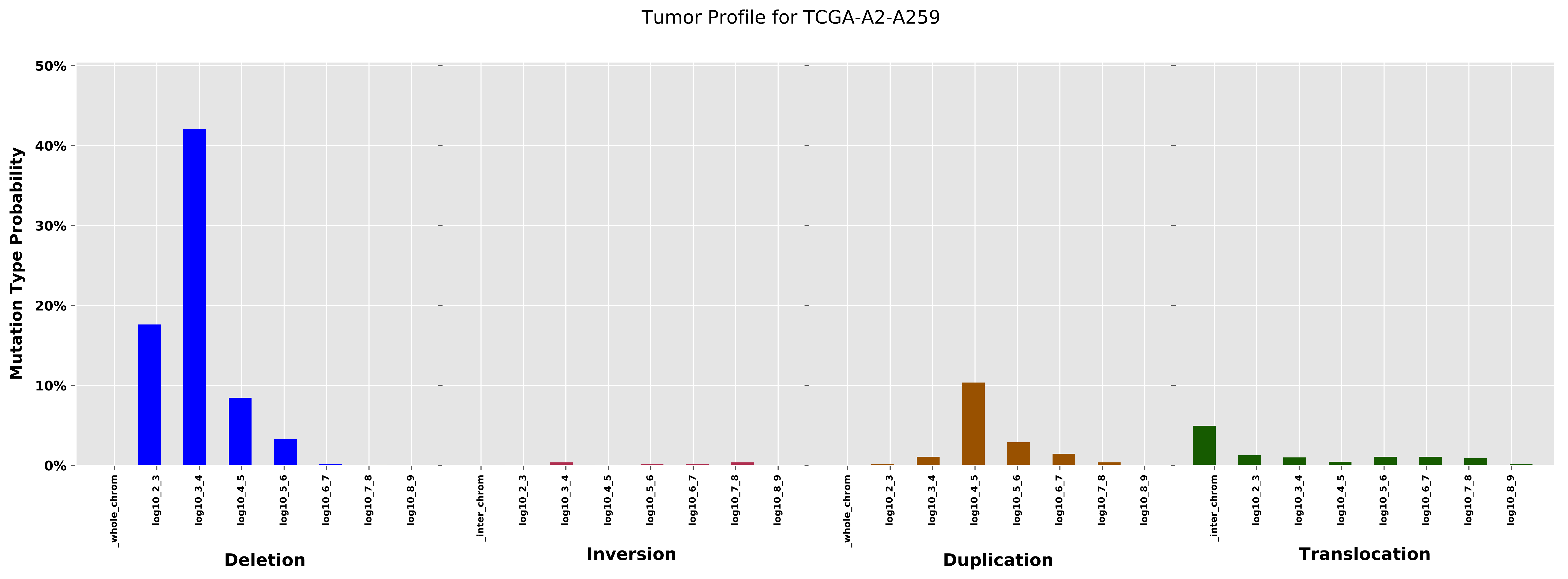

Tumor Profile for TCGA-A2-A259
Mutation Type Probability
50%
40%
30%
20%
10%
0%
_whole_chrom
log10_2_3
log10_3_4
log10_4_5
log10_5_6
log10_6_7
log10_7_8
log10_8_9
Deletion
_inter_chrom
log10_2_3
log10_3_4
log10_4_5
log10_5_6
log10_6_7
log10_7_8
log10_8_9
Inversion
_whole_chrom
log10_2_3
log10_3_4
log10_4_5
log10_5_6
log10_6_7
log10_7_8
log10_8_9
Duplication
_inter_chrom
log10_2_3
log10_3_4
log10_4_5
log10_5_6
log10_6_7
log10_7_8
log10_8_9
Translocation

Cancer processes Weights for TCGA-BH-A0DG

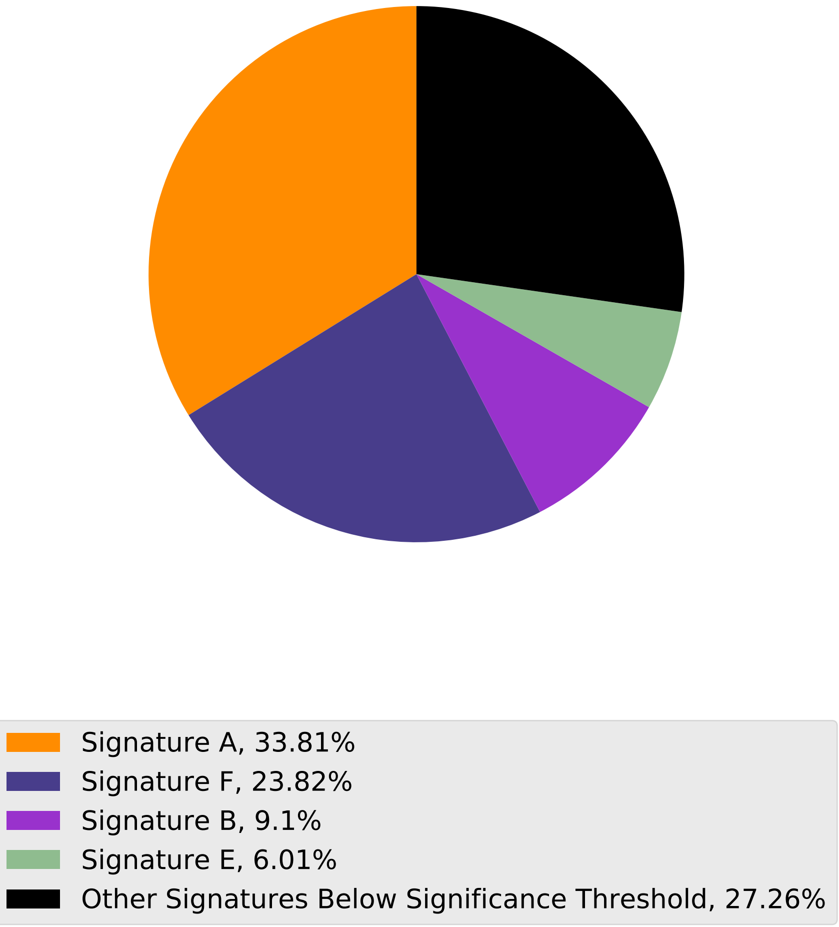

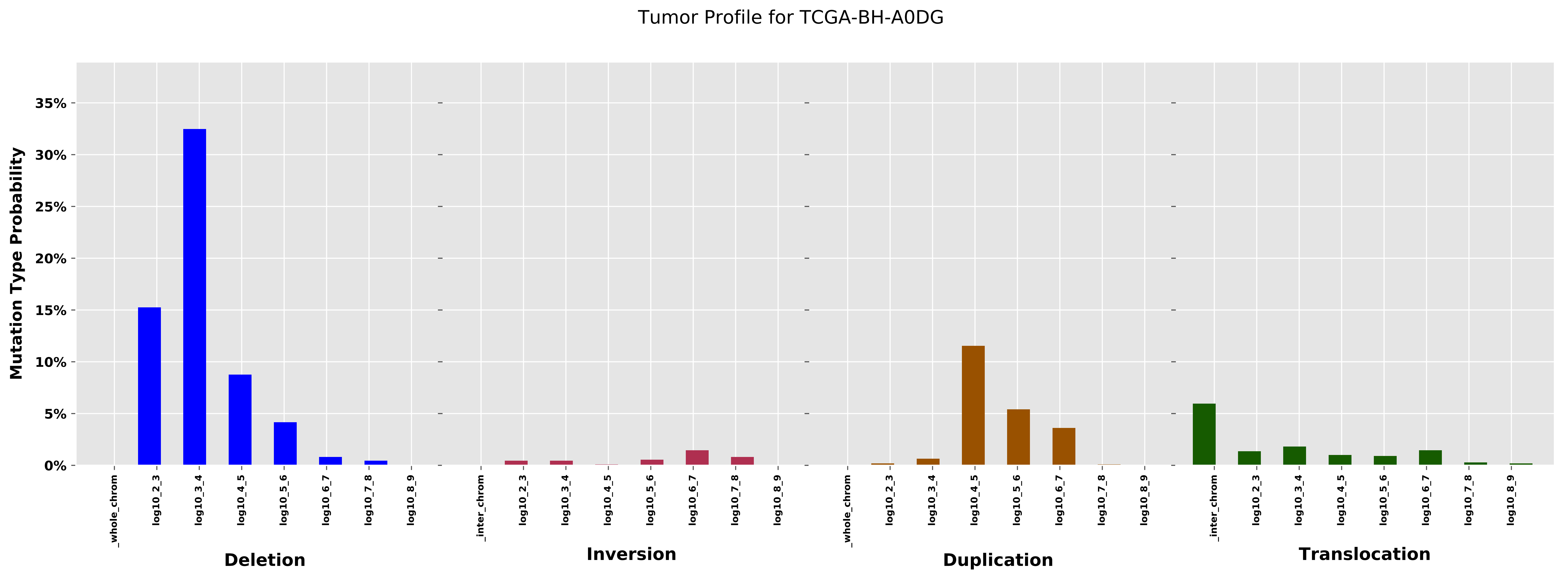

Tumor Profile for TCGA-BH-A0DG
Mutation Type Probability
0%
5%
10%
15%
20%
25%
30%
35%
_whole_chrom
log10_2_3
log10_3_4
log10_4_5
log10_5_6
log10_6_7
log10_7_8
log10_8_9
Deletion
_inter_chrom
Inversion
Duplication
Translocation

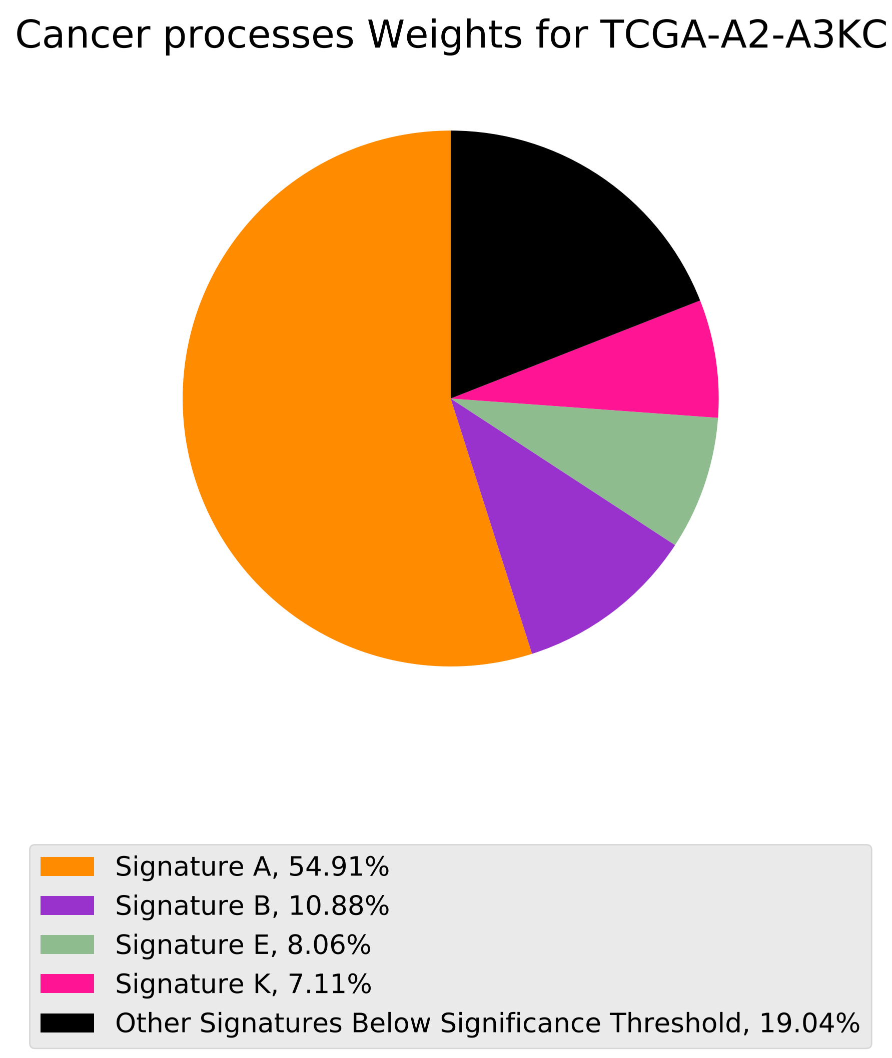
Cancer processes Weights for TCGA-A2-A3KC
Signature A, 54.91%
Signature B, 10.88%
Signature E, 8.06%
Signature K, 7.11%
Other Signatures Below Significance Threshold, 19.04%

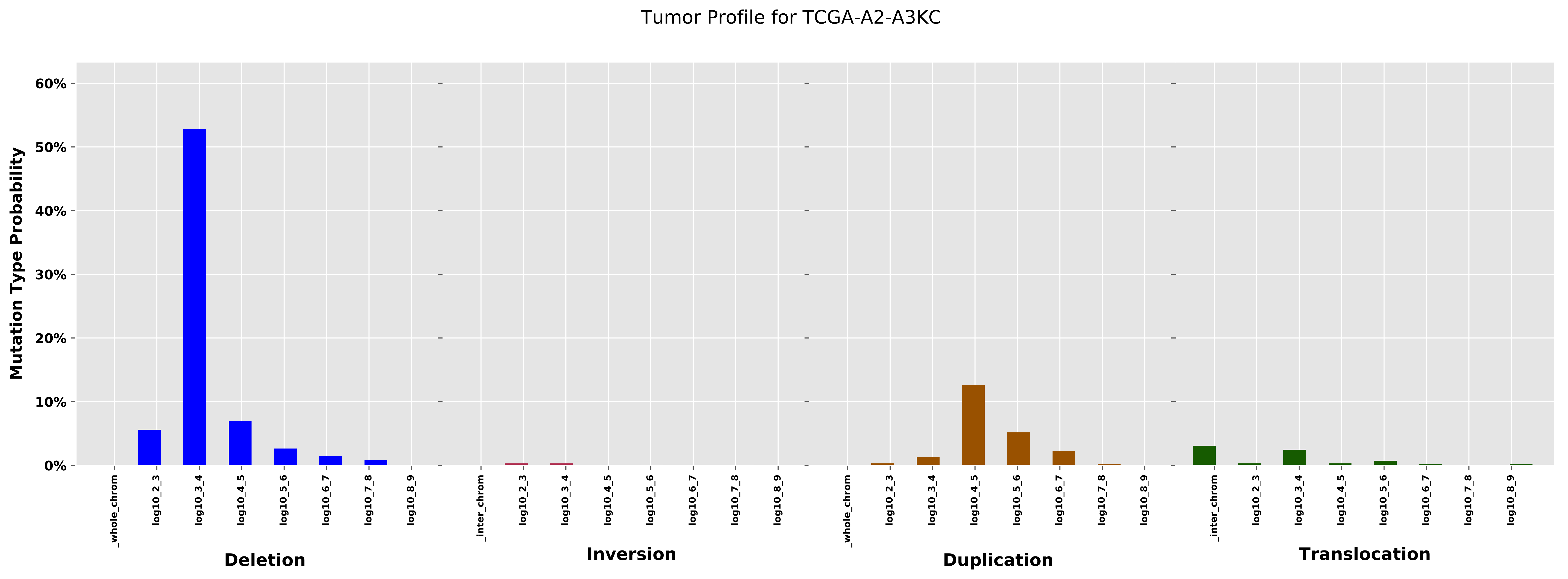

Tumor Profile for TCGA-A2-A3KC
Mutation Type Probability
60%
50%
40%
30%
20%
10%
0%
_whole_chrom
log10_2_3
log10_3_4
log10_4_5
log10_5_6
log10_6_7
log10_7_8
log10_8_9
_inter_chrom
Deletion
Inversion
Duplication
Translocation

Cancer processes Weights for TCGA-AA-3514

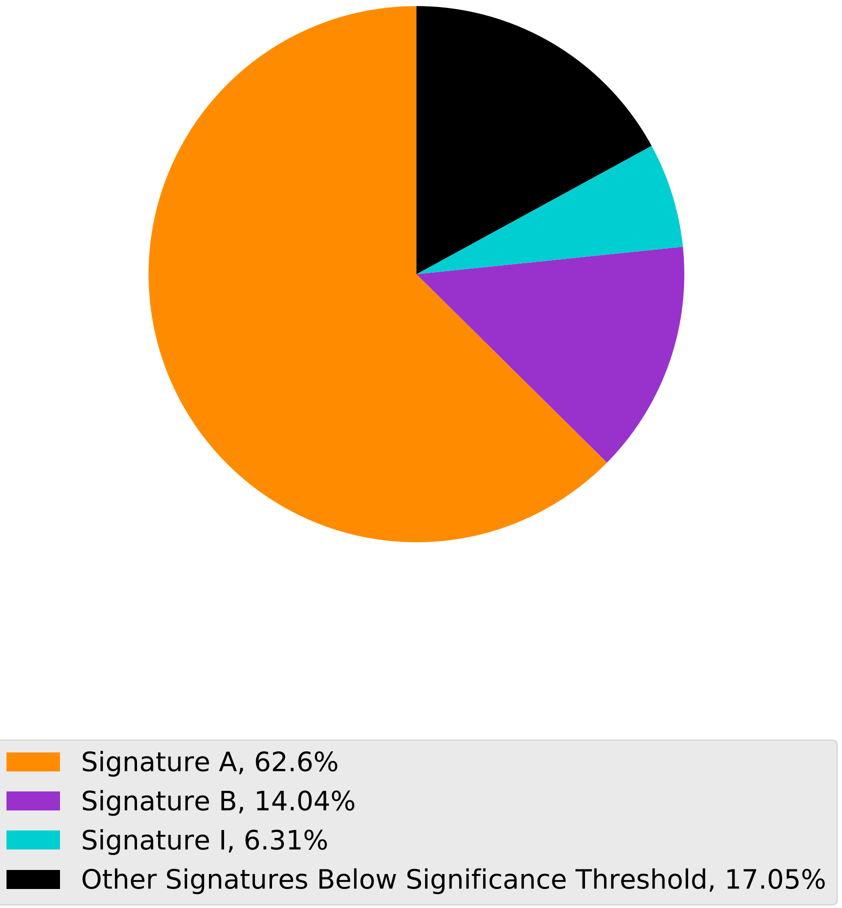

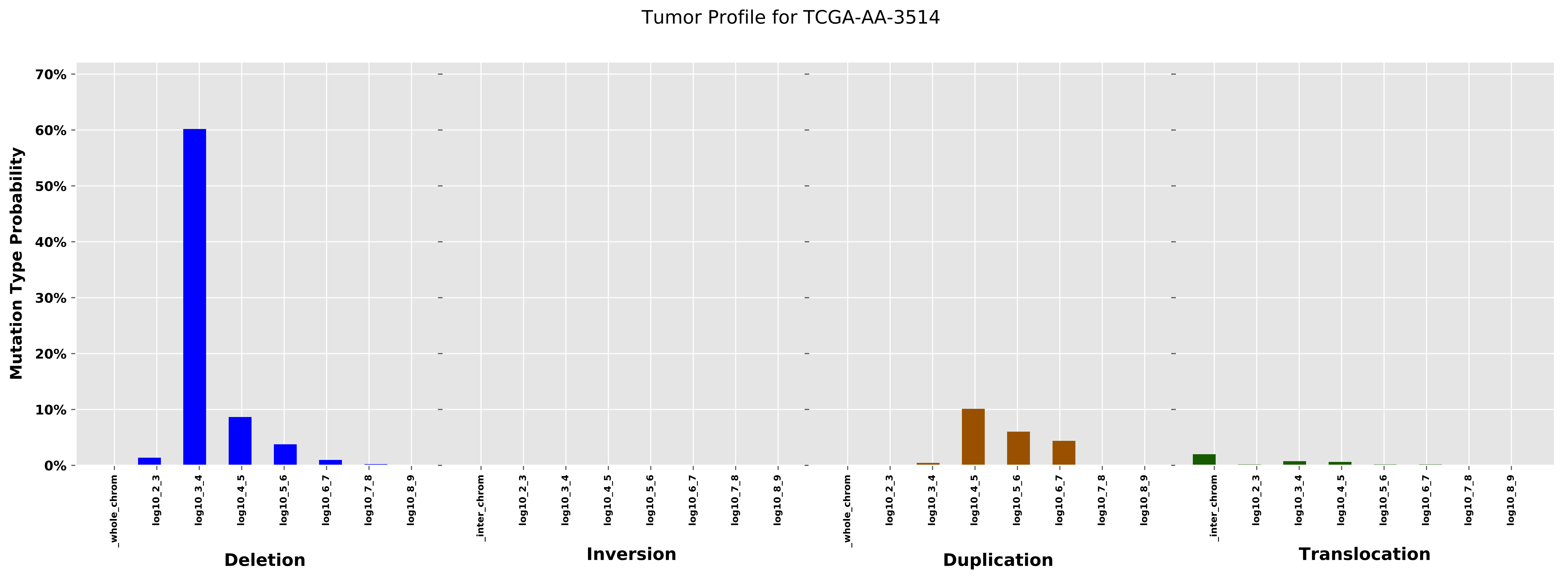

Tumor Profile for TCGA-AA-3514
Mutation Type Probability
70%
60%
50%
40%
30%
20%
10%
0%
_whole_chrom
log10_2_3
log10_3_4
log10_4_5
log10_5_6
log10_6_7
log10_7_8
log10_8_9
Deletion
_inter_chrom
log10_2_3
log10_3_4
log10_4_5
log10_5_6
log10_6_7
log10_7_8
log10_8_9
Inversion
_whole_chrom
log10_2_3
log10_3_4
log10_4_5
log10_5_6
log10_6_7
log10_7_8
log10_8_9
Duplication
_inter_chrom
log10_2_3
log10_3_4
log10_4_5
log10_5_6
log10_6_7
log10_7_8
log10_8_9
Translocation

Cancer processes Weights for TCGA-AN-A0XR

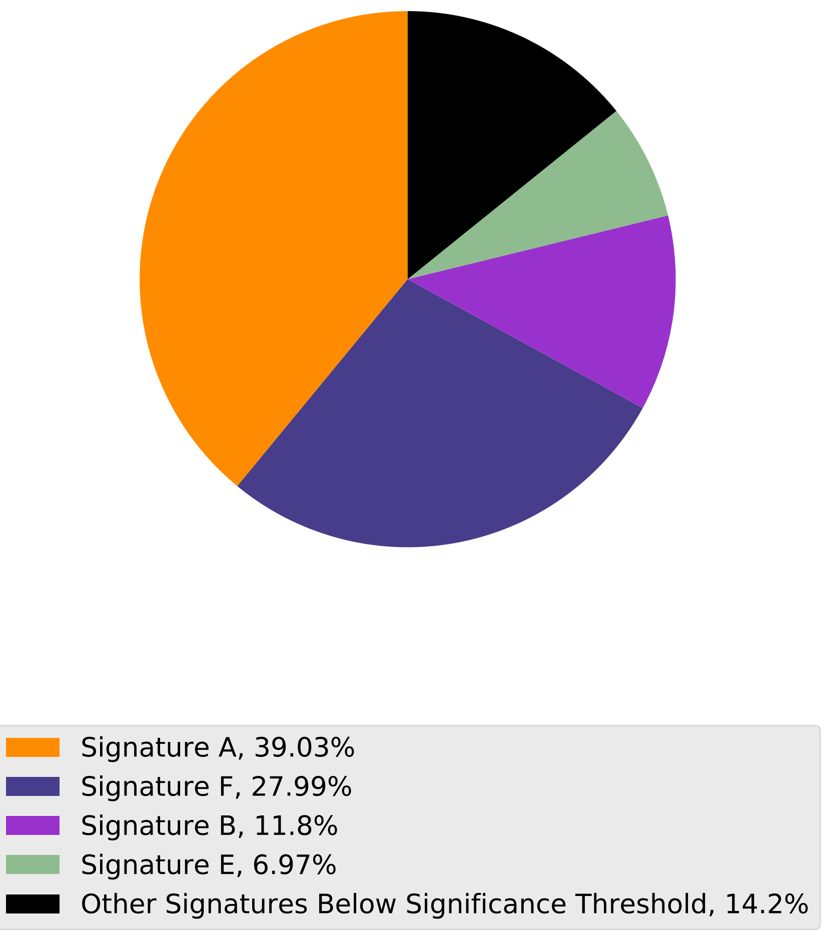

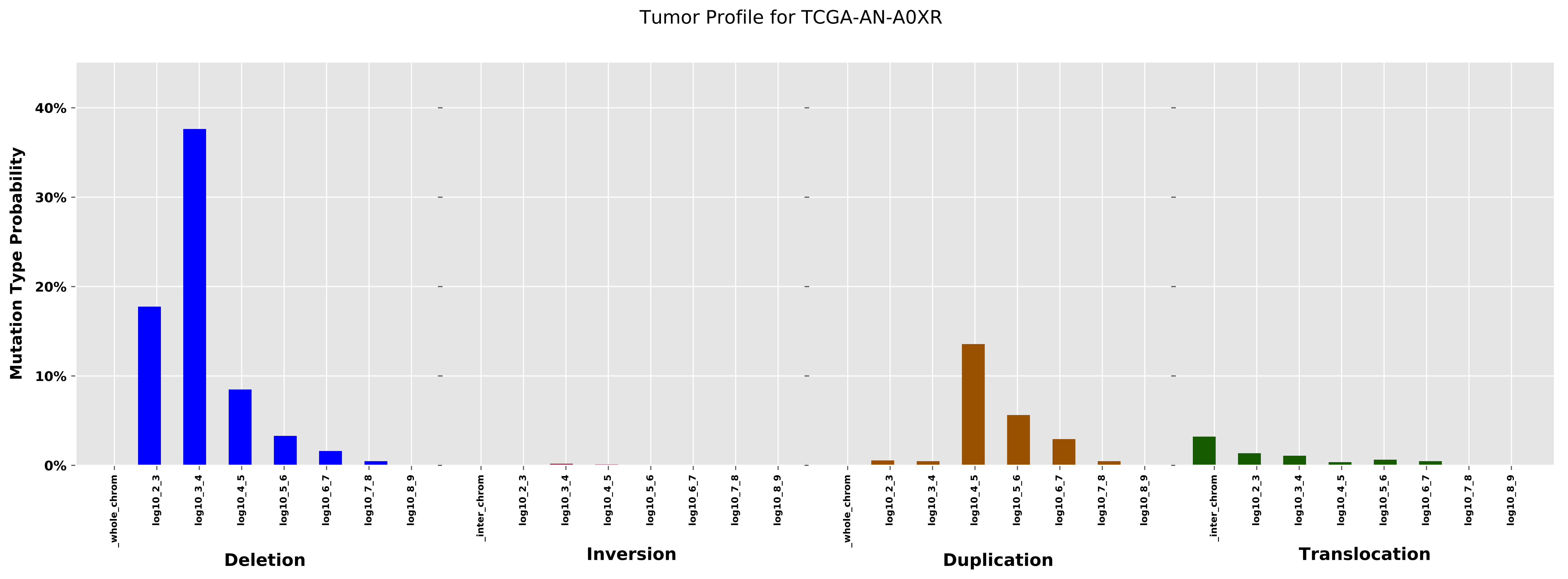

Tumor Profile for TCGA-AN-A0XR
Mutation Type Probability
40%
30%
20%
10%
0%
_whole_chrom
log10_2_3
log10_3_4
log10_4_5
log10_5_6
log10_6_7
log10_7_8
log10_8_9
_inter_chrom
Deletion
Inversion
Duplication
Translocation

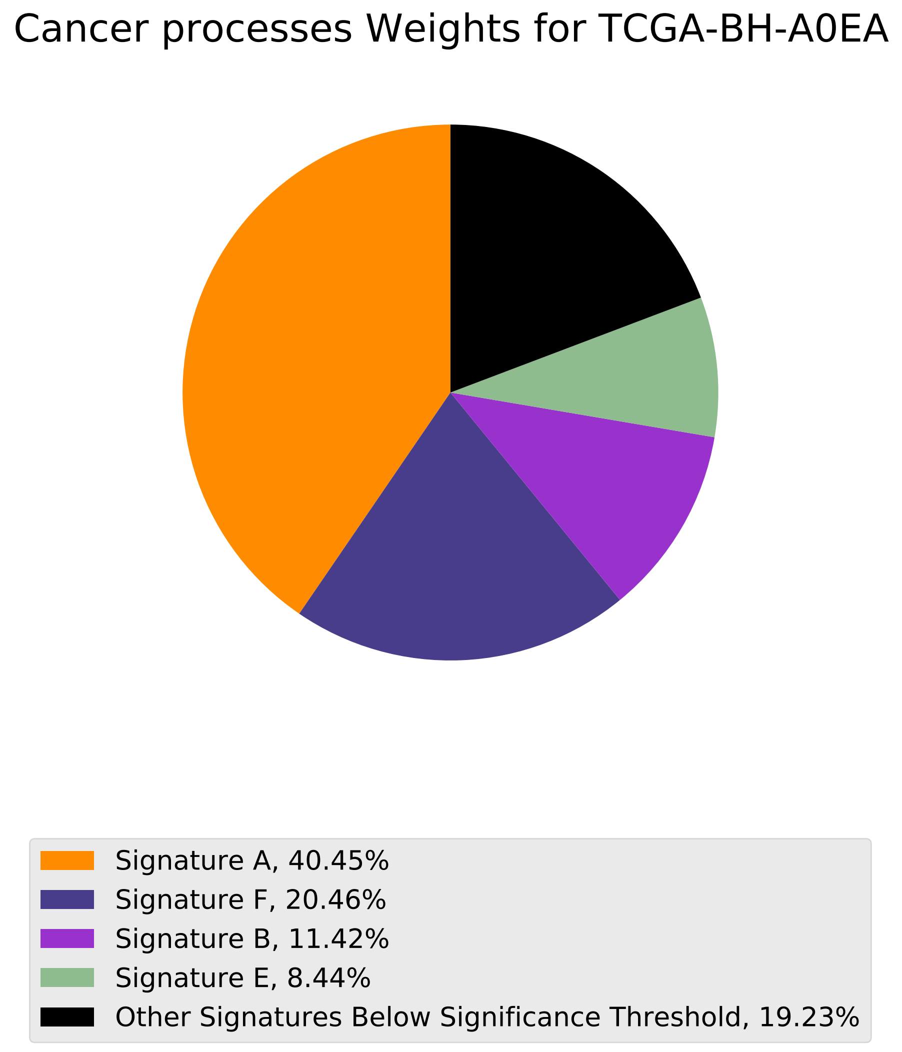
Cancer processes Weights for TCGA-BH-A0EA
Signature A, 40.45%
Signature F, 20.46%
Signature B, 11.42%
Signature E, 8.44%
Other Signatures Below Significance Threshold, 19.23%

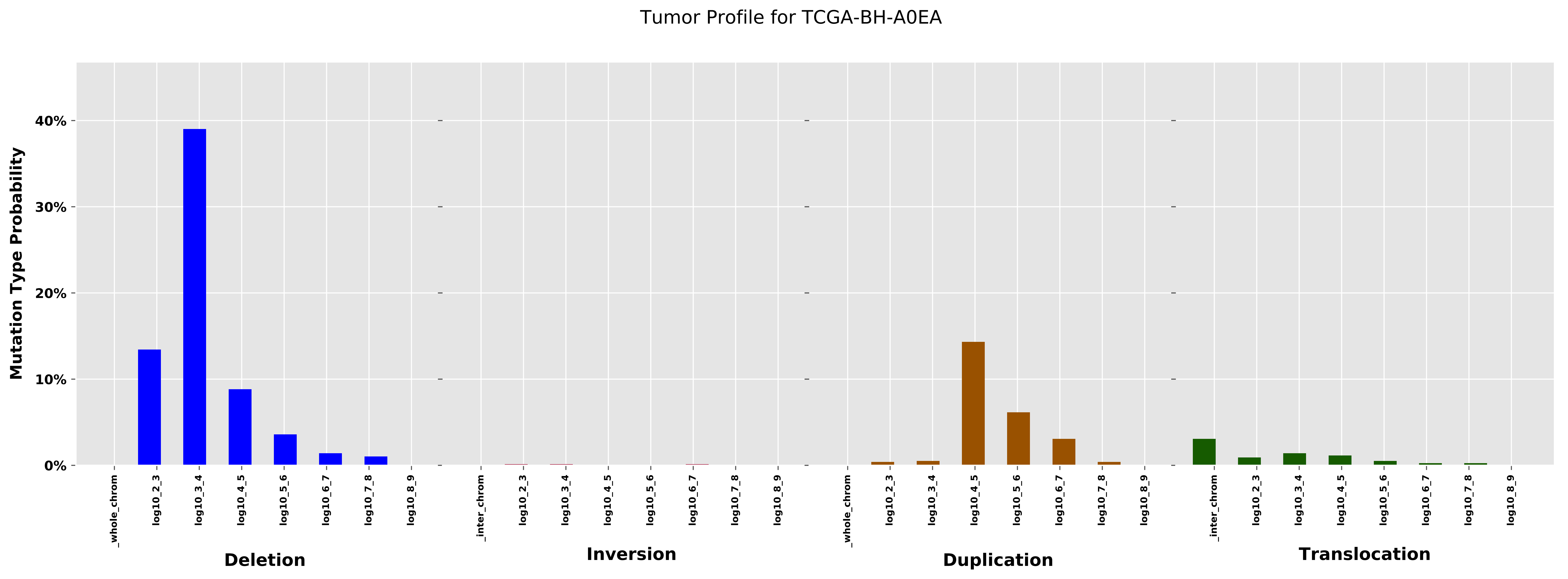

Tumor Profile for TCGA-BH-A0EA
Mutation Type Probability
40%
30%
20%
10%
0%
_whole_chrom
log10_2_3
log10_3_4
log10_4_5
log10_5_6
log10_6_7
log10_7_8
log10_8_9
_inter_chrom
Deletion
Inversion
Duplication
Translocation

Cancer processes Weights for TCGA-AR-A256

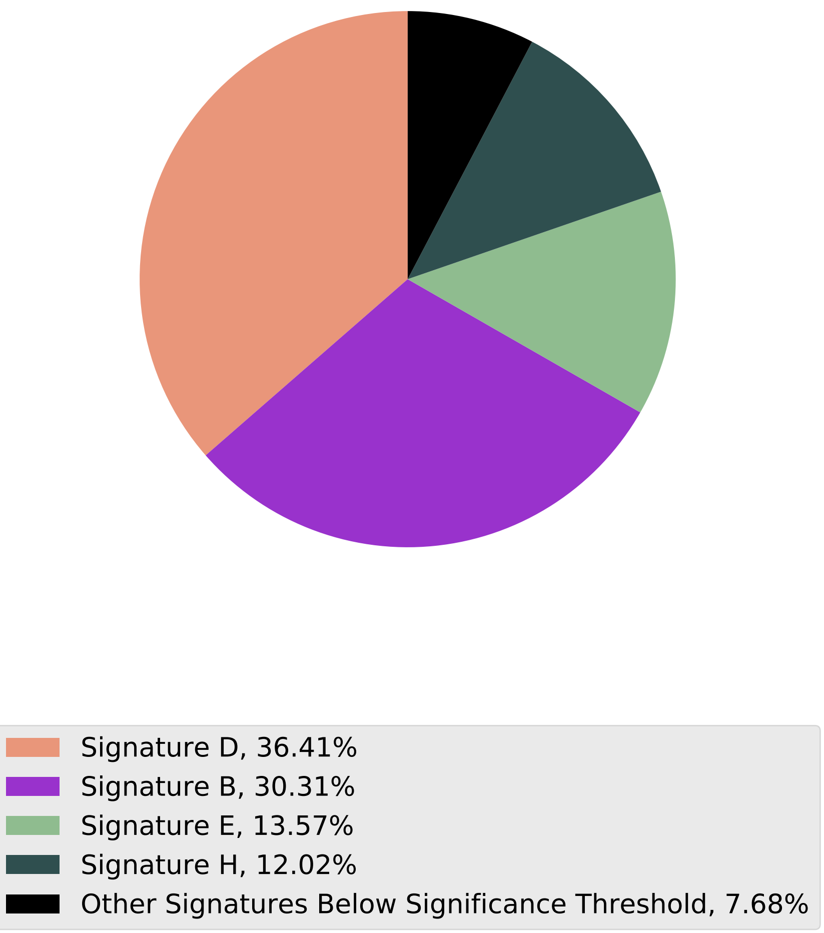

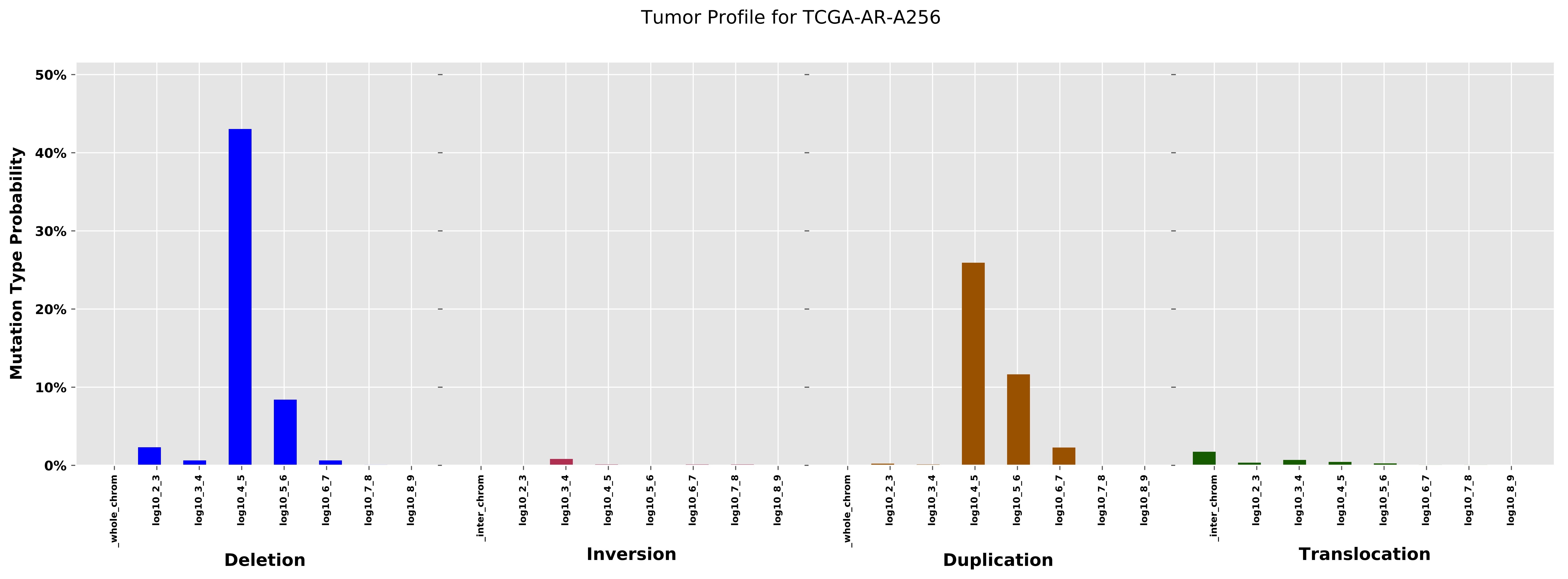
Tumor Profile for TCGA-AR-A256
Mutation Type Probability
50%
40%
30%
20%
10%
0%
_whole_chrom
log10_2_3
log10_3_4
log10_4_5
log10_5_6
log10_6_7
log10_7_8
log10_8_9
_inter_chrom
Deletion
Inversion
Duplication
Translocation

Cancer processes Weights for TCGA-A8-A09X

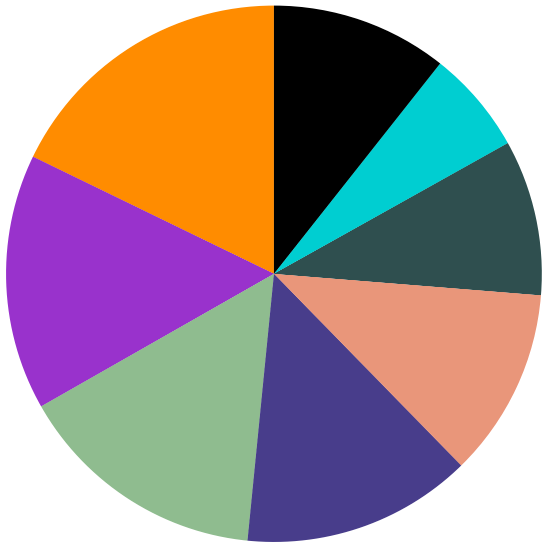

- Signature A, 17.81%
- Signature B, 15.43%
- Signature E, 15.19%
- Signature F, 13.9%
- Signature D, 11.4%
- Signature H, 9.38%
- Signature I, 6.24%
- Other Signatures Below Significance Threshold, 10.65%

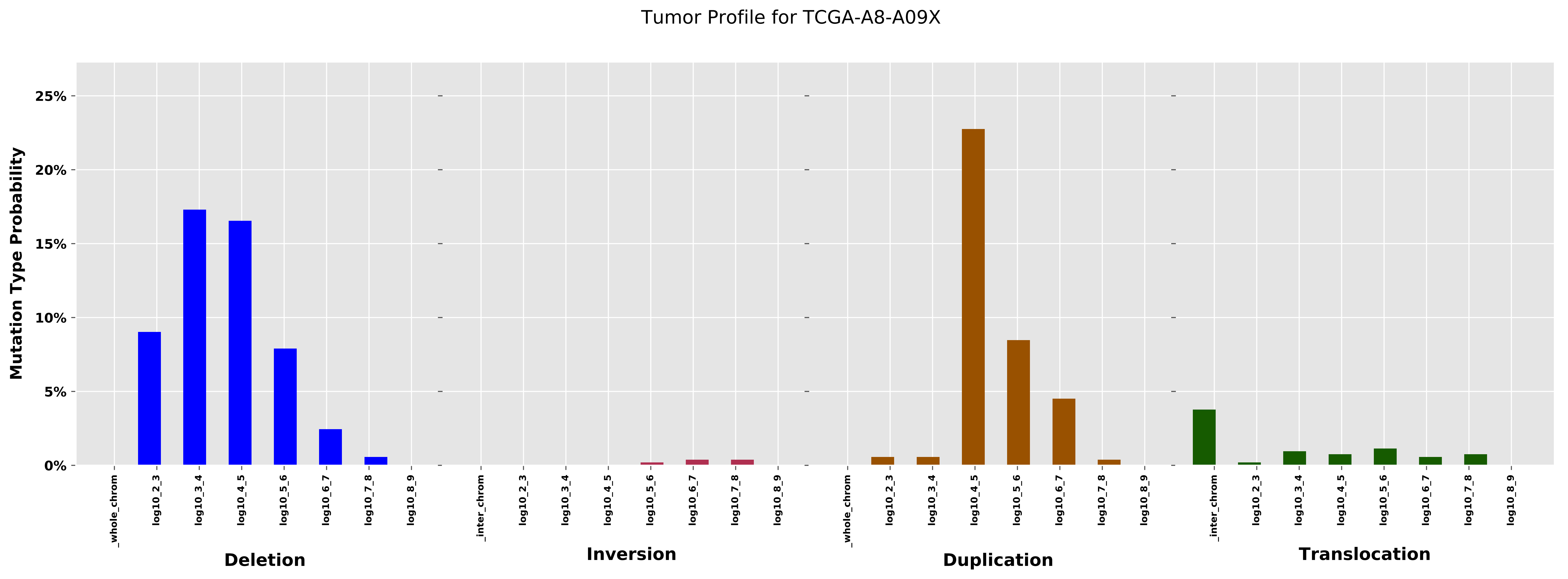

Tumor Profile for TCGA-A8-A09X
Mutation Type Probability
25%
20%
15%
10%
5%
0%
_whole_chrom
log10_2_3
log10_3_4
log10_4_5
log10_5_6
log10_6_7
log10_7_8
log10_8_9
_inter_chrom
Deletion
Inversion
Duplication
Translocation

Cancer processes Weights for TCGA-AA-A01V

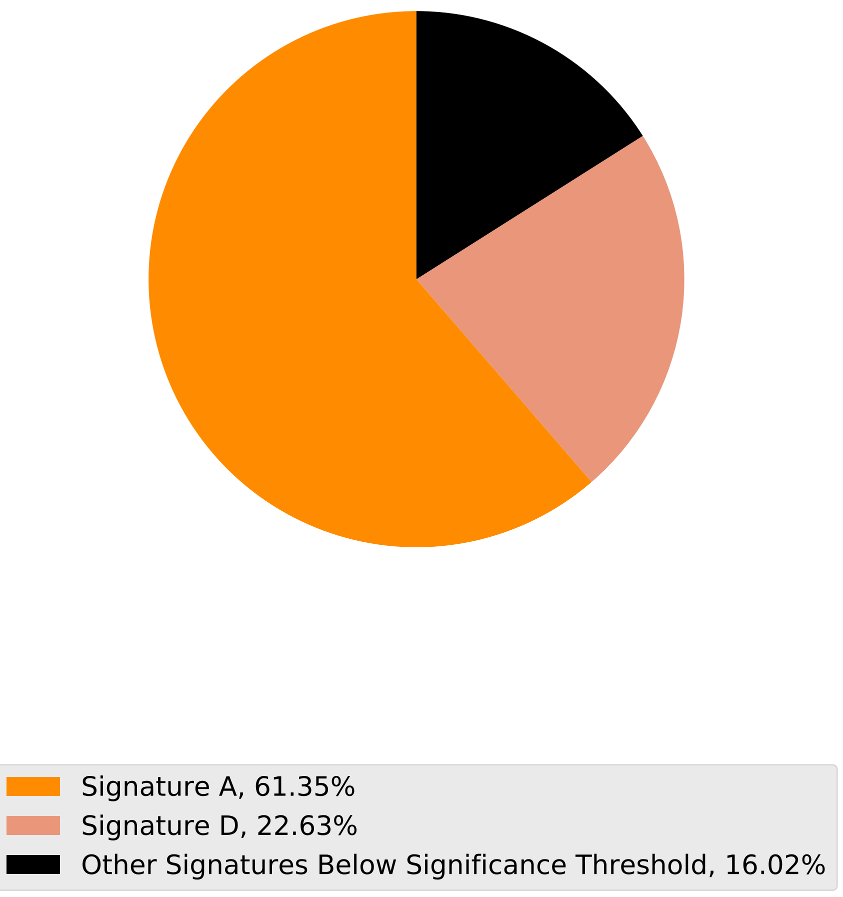

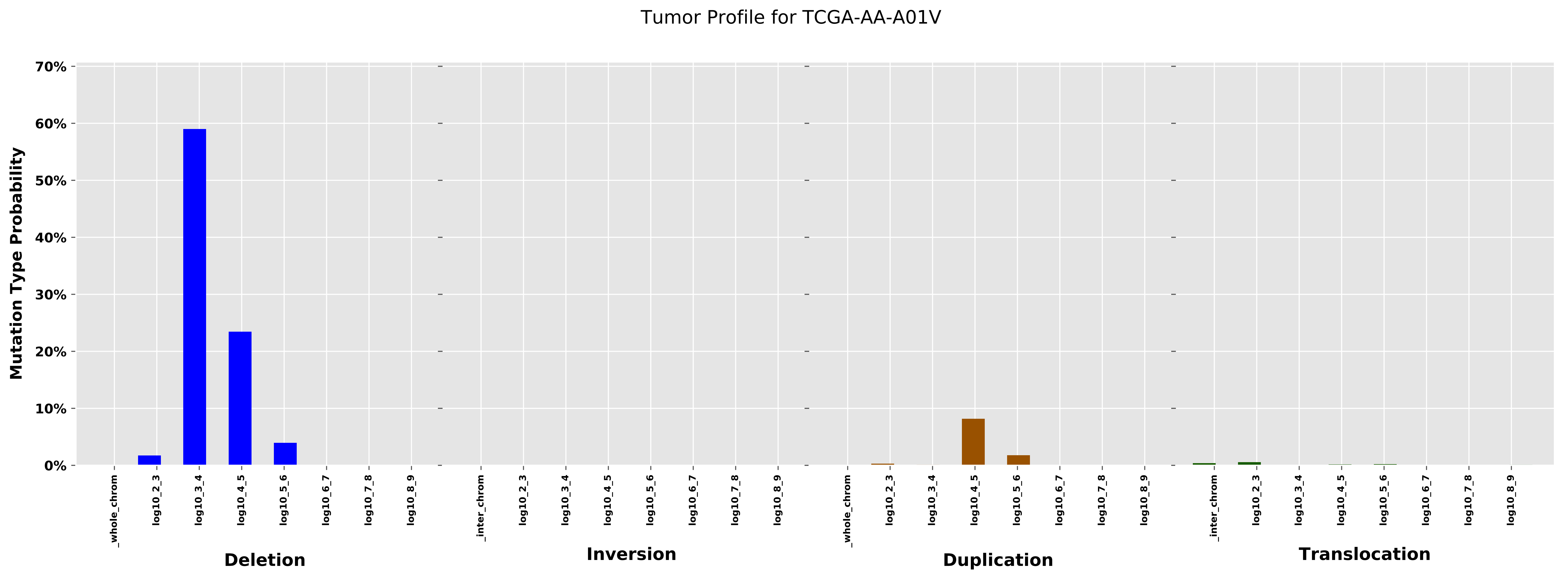
Tumor Profile for TCGA-AA-A01V
Mutation Type Probability
70%
60%
50%
40%
30%
20%
10%
0%
_whole_chrom
log10_2_3
log10_3_4
log10_4_5
log10_5_6
log10_6_7
log10_7_8
log10_8_9
_inter_chrom
Deletion
Inversion
Duplication
Translocation

Cancer processes Weights for TCGA-A2-A04T

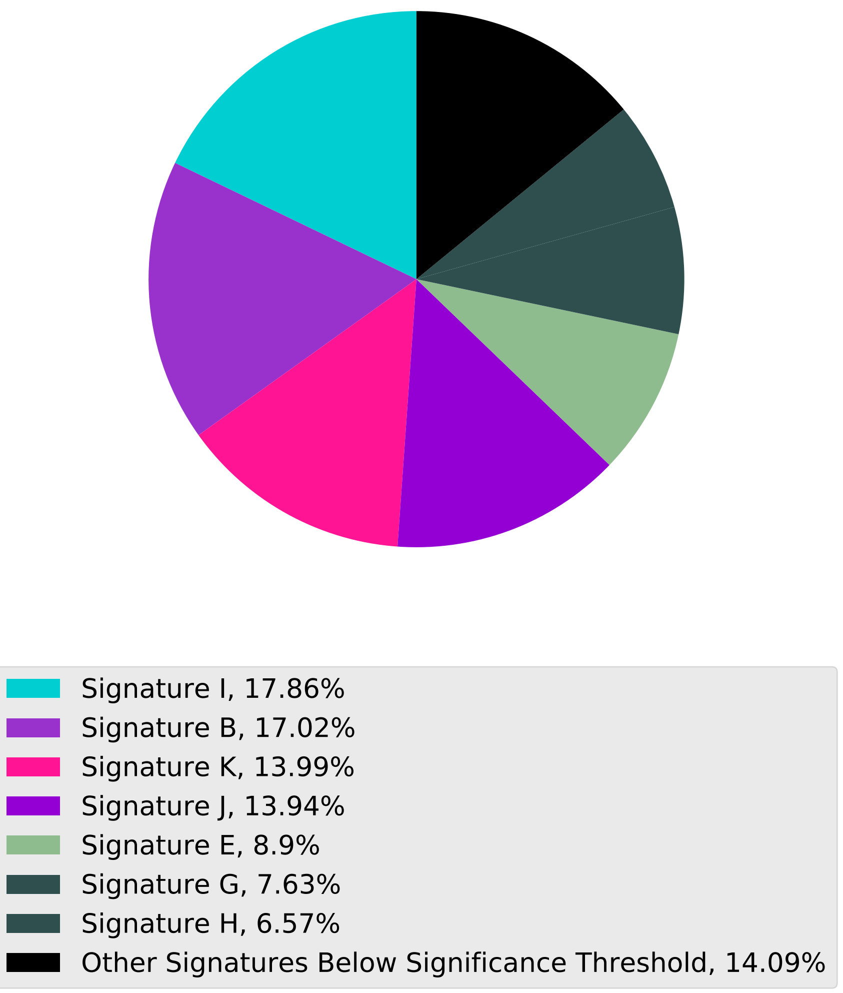

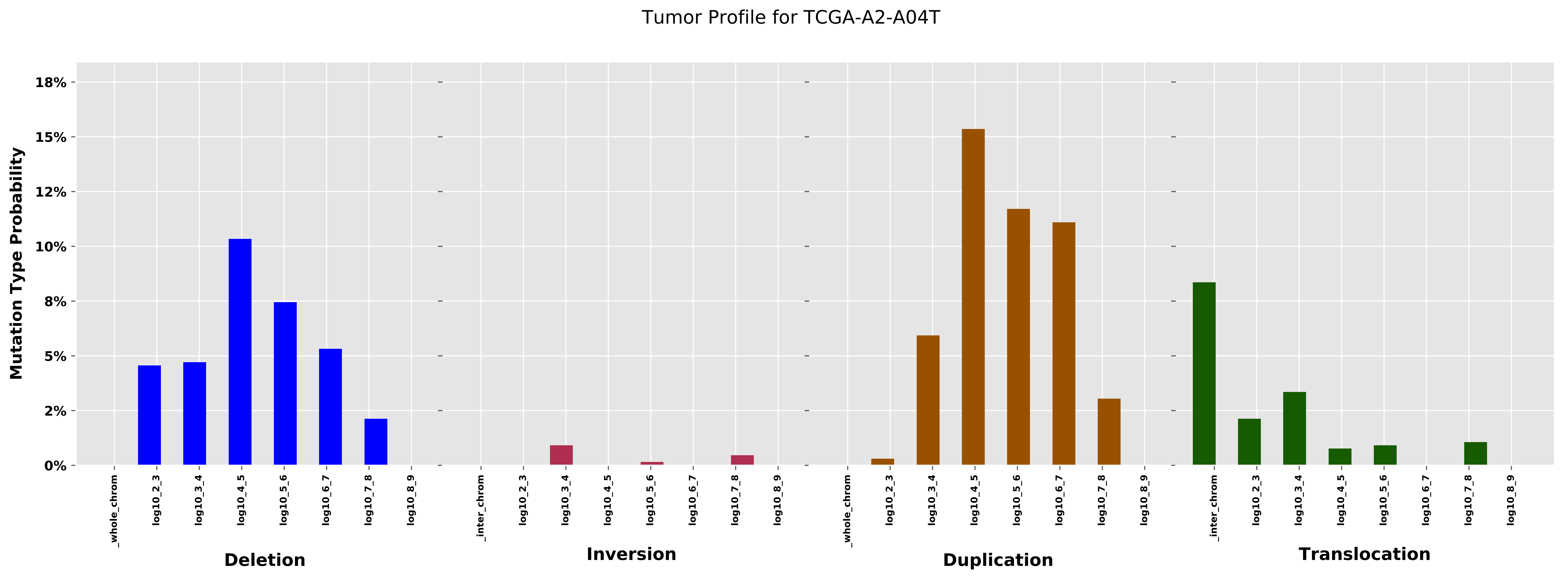

Tumor Profile for TCGA-A2-A04T
Mutation Type Probability
18%
15%
12%
10%
8%
5%
2%
0%
_whole_chrom
log10_2_3
log10_3_4
log10_4_5
log10_5_6
log10_6_7
log10_7_8
log10_8_9
_inter_chrom
Deletion
Inversion
Duplication
Translocation

Cancer processes Weights for TCGA-BH-A0H6

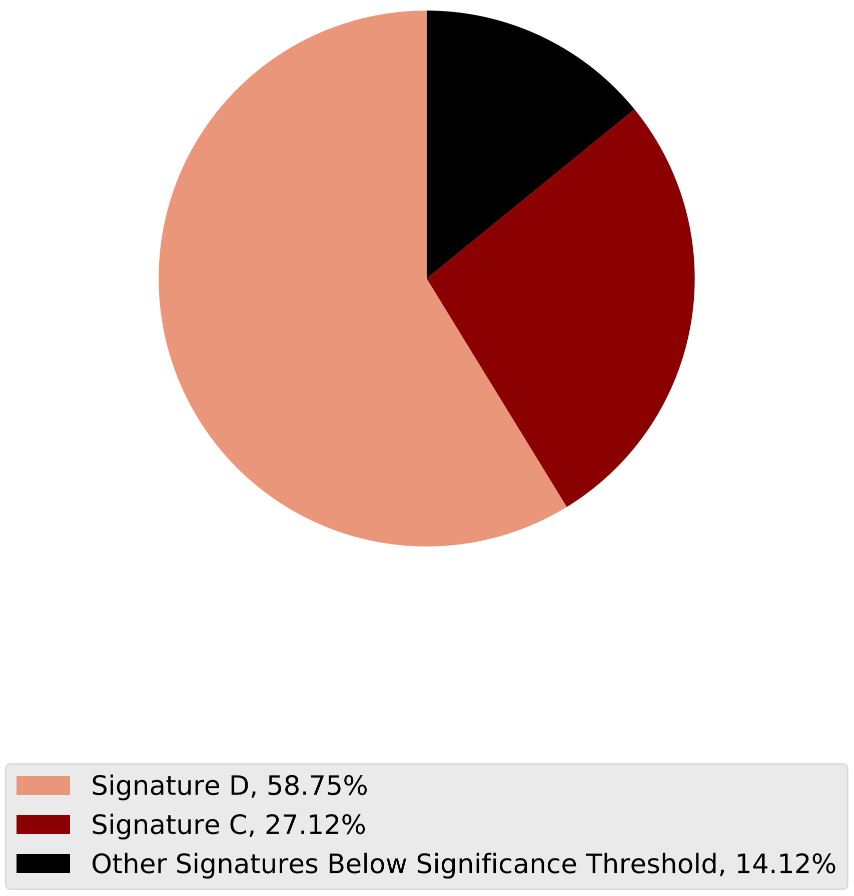

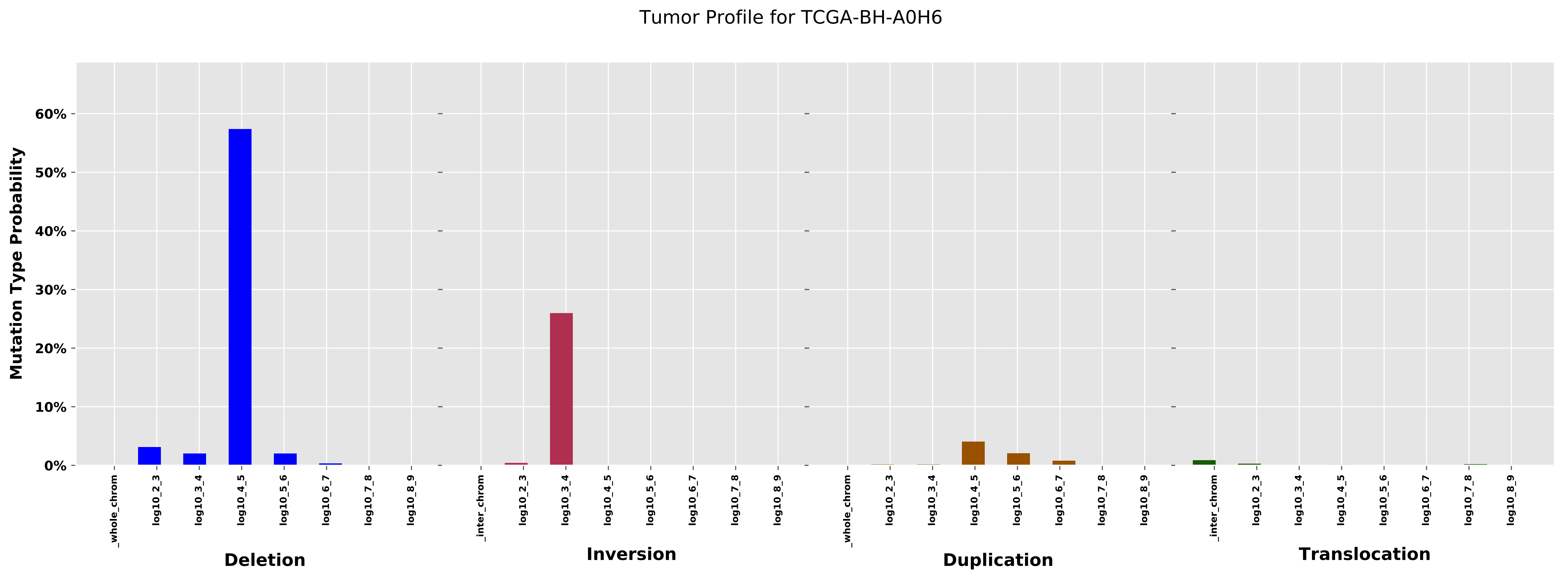

Tumor Profile for TCGA-BH-A0H6
Mutation Type Probability
60%
50%
40%
30%
20%
10%
0%
_whole_chrom
log10_2_3
log10_3_4
log10_4_5
log10_5_6
log10_6_7
log10_7_8
log10_8_9
Deletion
_inter_chrom
Inversion
Duplication
Translocation

Cancer processes Weights for TCGA-D8-A27F

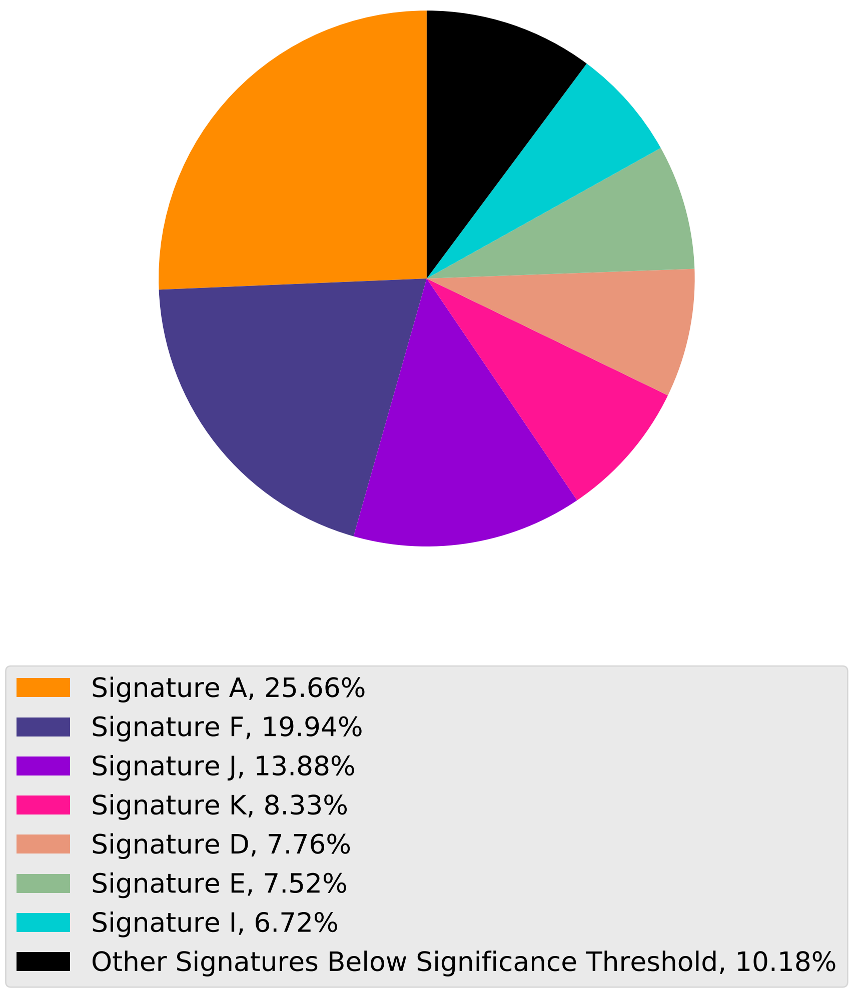

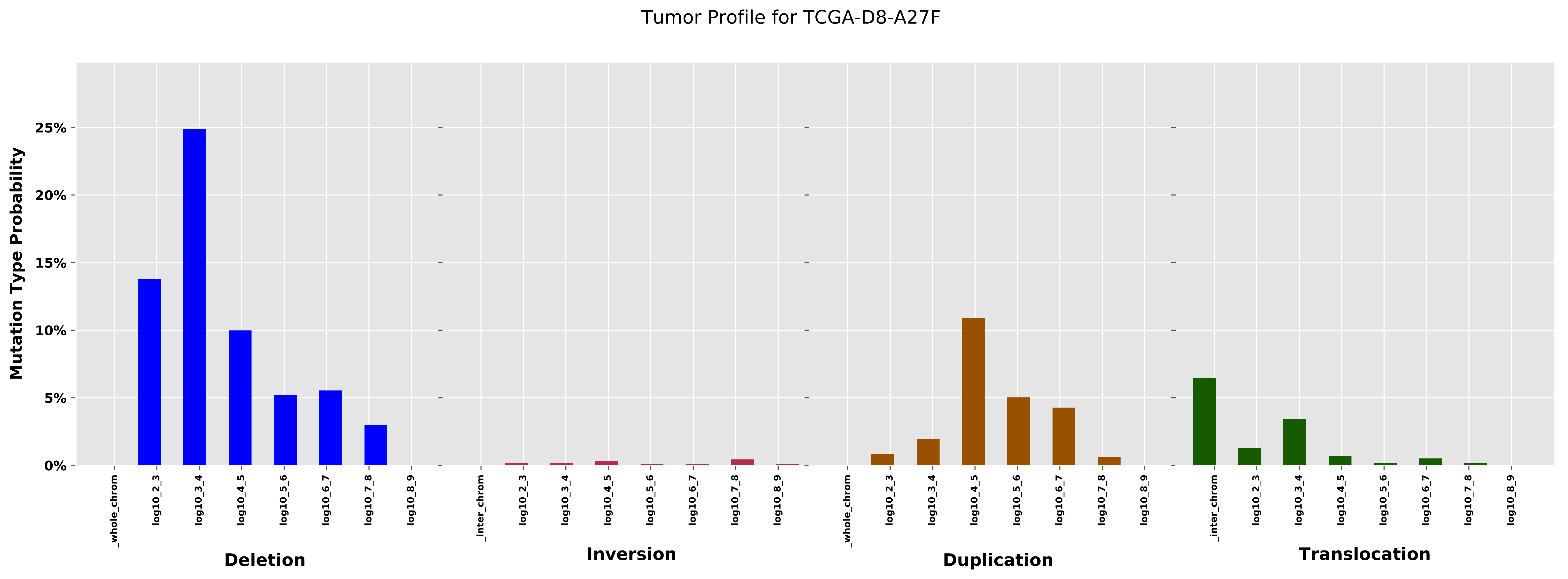

Tumor Profile for TCGA-D8-A27F
Mutation Type Probability
0%
5%
10%
15%
20%
25%
_whole_chrom
log10_2_3
log10_3_4
log10_4_5
log10_5_6
log10_6_7
log10_7_8
log10_8_9
_inter_chrom
Deletion
Inversion
Duplication
Translocation

Cancer processes Weights for TCGA-AG-3727

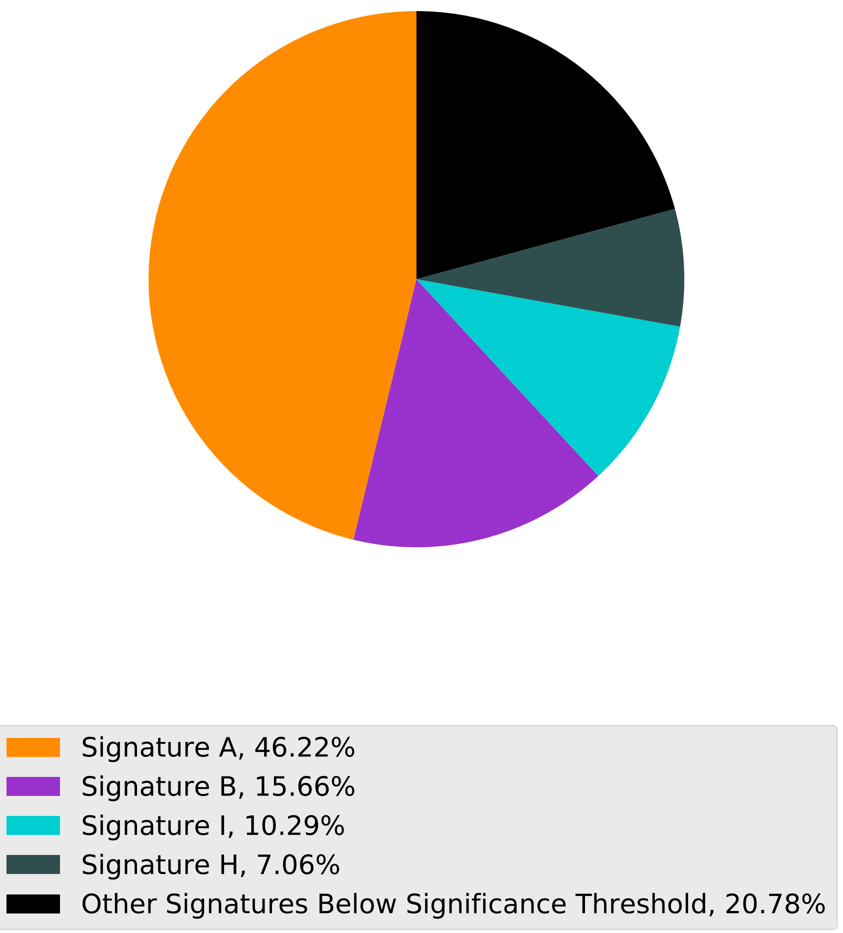

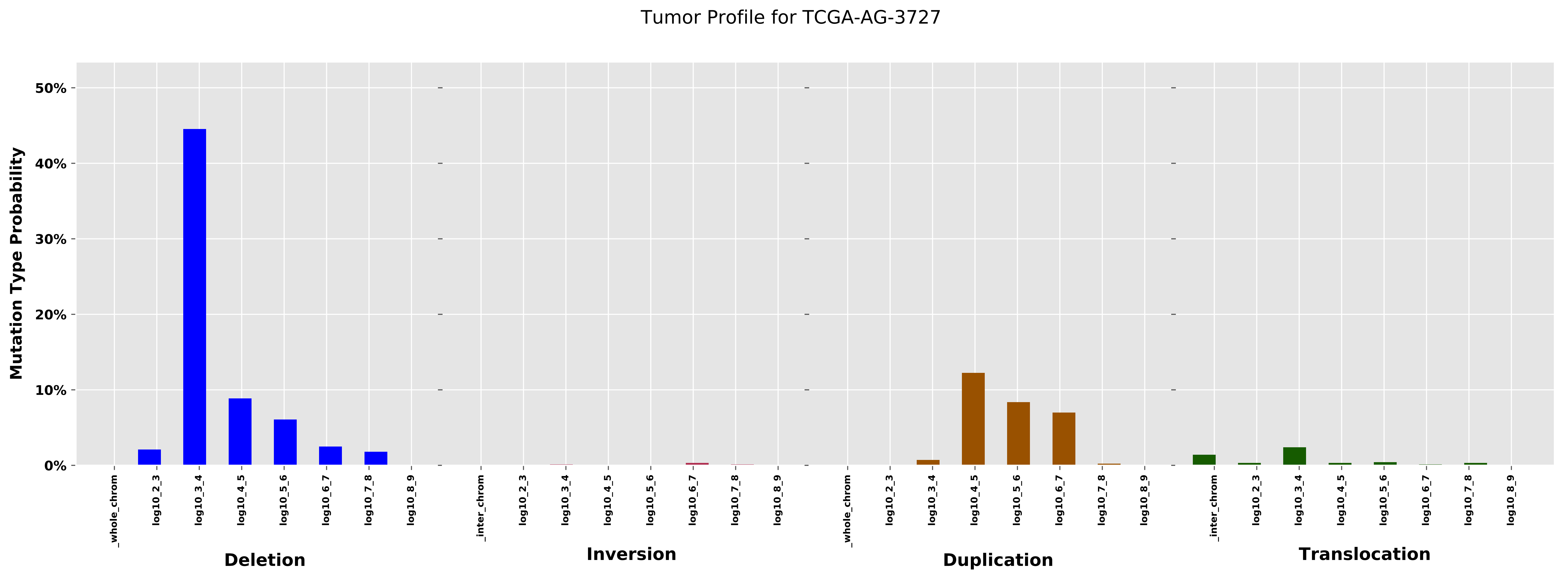
Tumor Profile for TCGA-AG-3727
Mutation Type Probability
50%
40%
30%
20%
10%
0%
_whole_chrom
log10_2_3
log10_3_4
log10_4_5
log10_5_6
log10_6_7
log10_7_8
log10_8_9
_inter_chrom
Deletion
Inversion
Duplication
Translocation

Cancer processes Weights for TCGA-A8-A09I

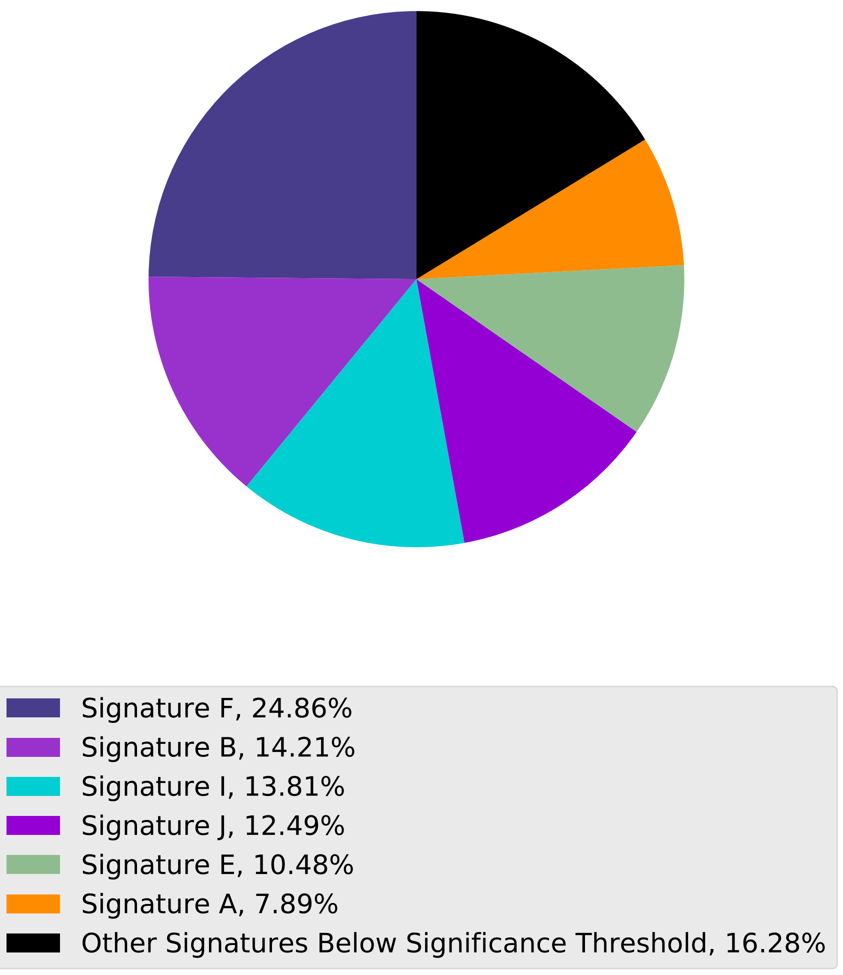

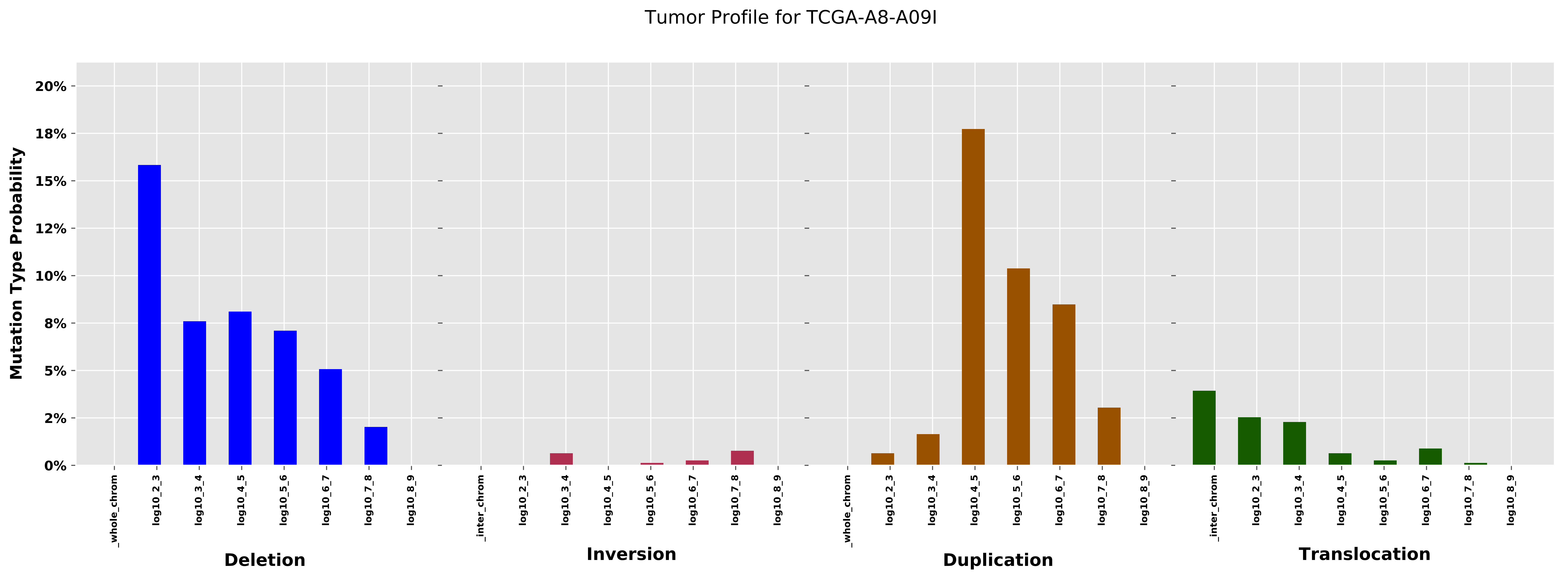

Tumor Profile for TCGA-A8-A09I
Mutation Type Probability
0%
2%
5%
8%
10%
12%
15%
18%
20%
_whole_chrom
log10_2_3
log10_3_4
log10_4_5
log10_5_6
log10_6_7
log10_7_8
log10_8_9
_inter_chrom
Deletion
Inversion
Duplication
Translocation

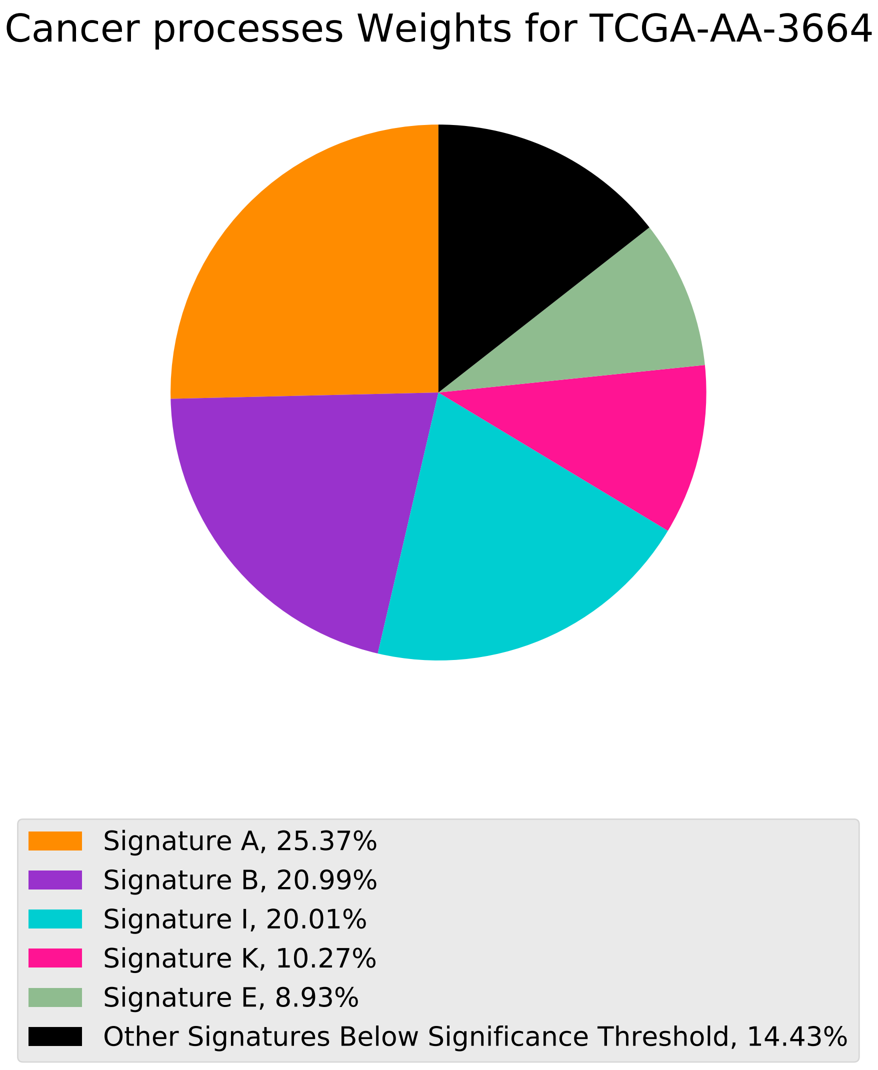
Cancer processes Weights for TCGA-AA-3664
Signature A, 25.37%
Signature B, 20.99%
Signature I, 20.01%
Signature K, 10.27%
Signature E, 8.93%
Other Signatures Below Significance Threshold, 14.43%

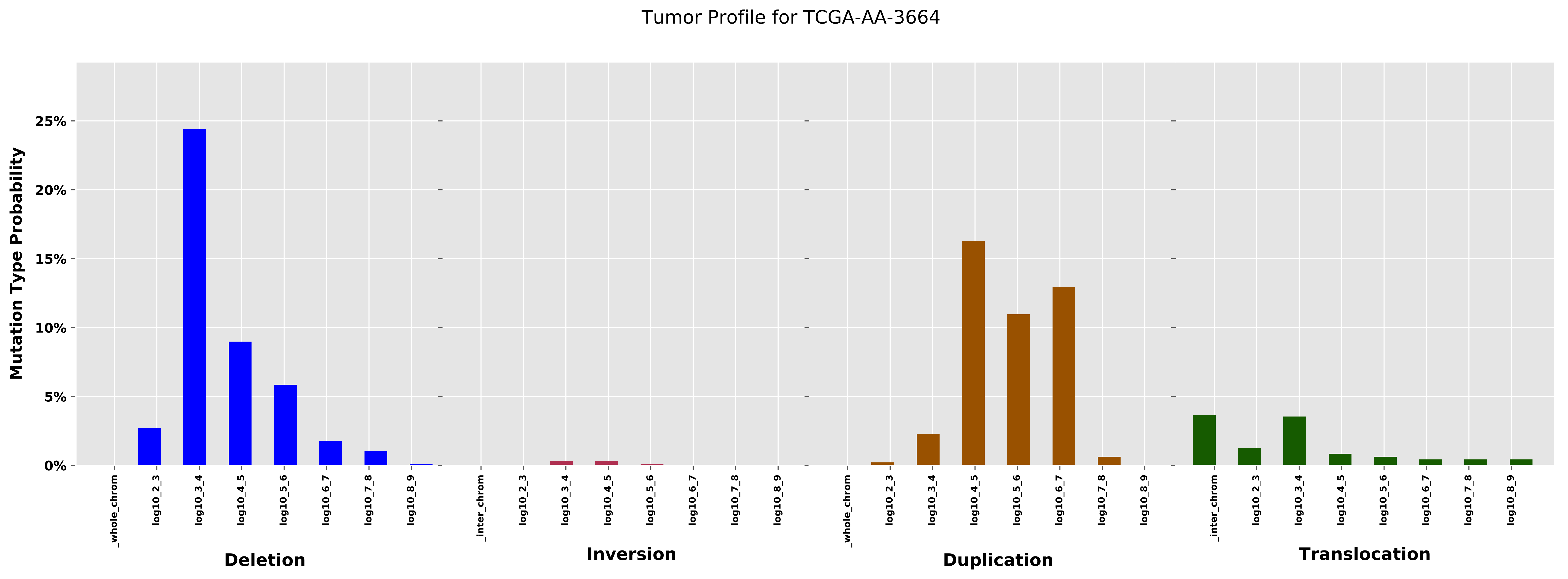

Tumor Profile for TCGA-AA-3664
Mutation Type Probability
25%
20%
15%
10%
5%
0%
_whole_chrom
log10_2_3
log10_3_4
log10_4_5
log10_5_6
log10_6_7
log10_7_8
log10_8_9
_inter_chrom
Deletion
Inversion
Duplication
Translocation

Cancer processes Weights for TCGA-E2-A1LL

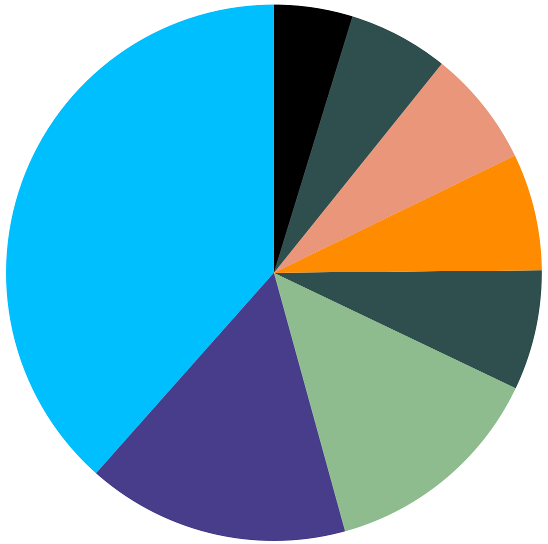

Signature L, 38.44%
Signature F, 15.84%
Signature E, 13.64%
Signature H, 7.23%
Signature A, 7.05%
Signature D, 7.02%
Signature G, 6.04%
Other Signatures Below Significance Threshold, 4.74%

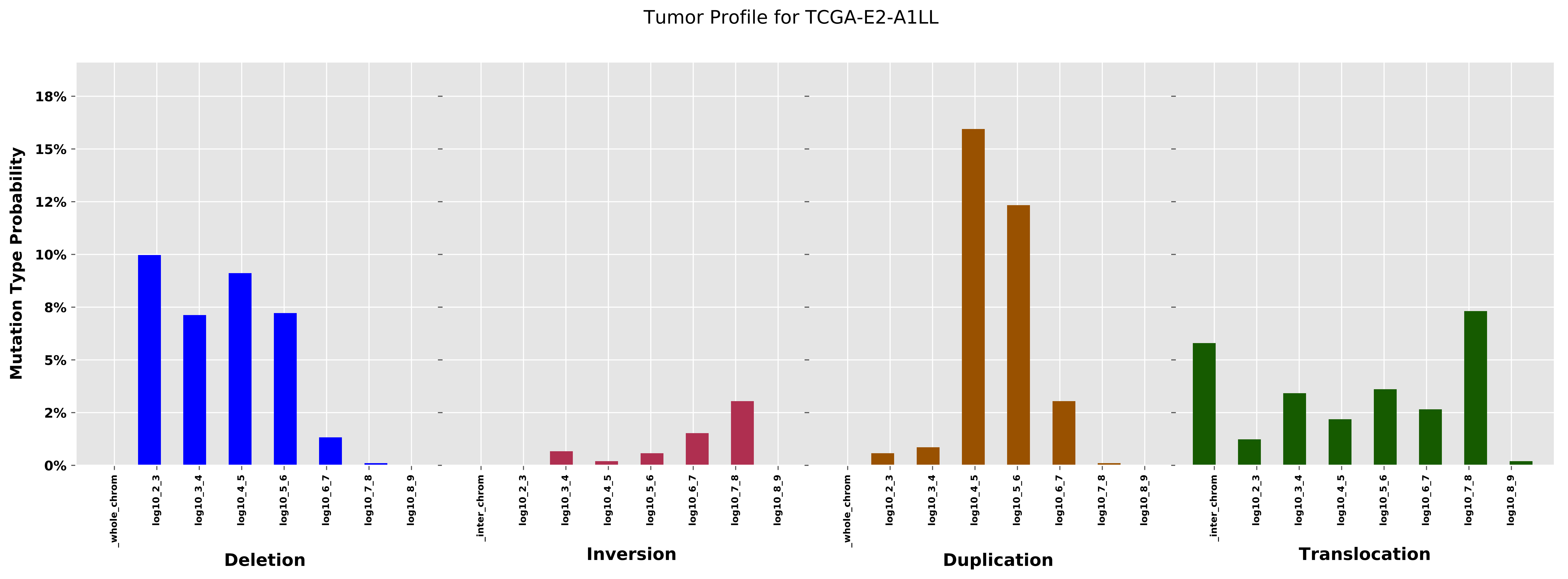

Tumor Profile for TCGA-E2-A1LL
Mutation Type Probability
18%
15%
12%
10%
8%
5%
2%
0%
_whole_chrom
log10_2_3
log10_3_4
log10_4_5
log10_5_6
log10_6_7
log10_7_8
log10_8_9
_inter_chrom
Deletion
Inversion
Duplication
Translocation

Cancer processes Weights for TCGA-A1-A0SM

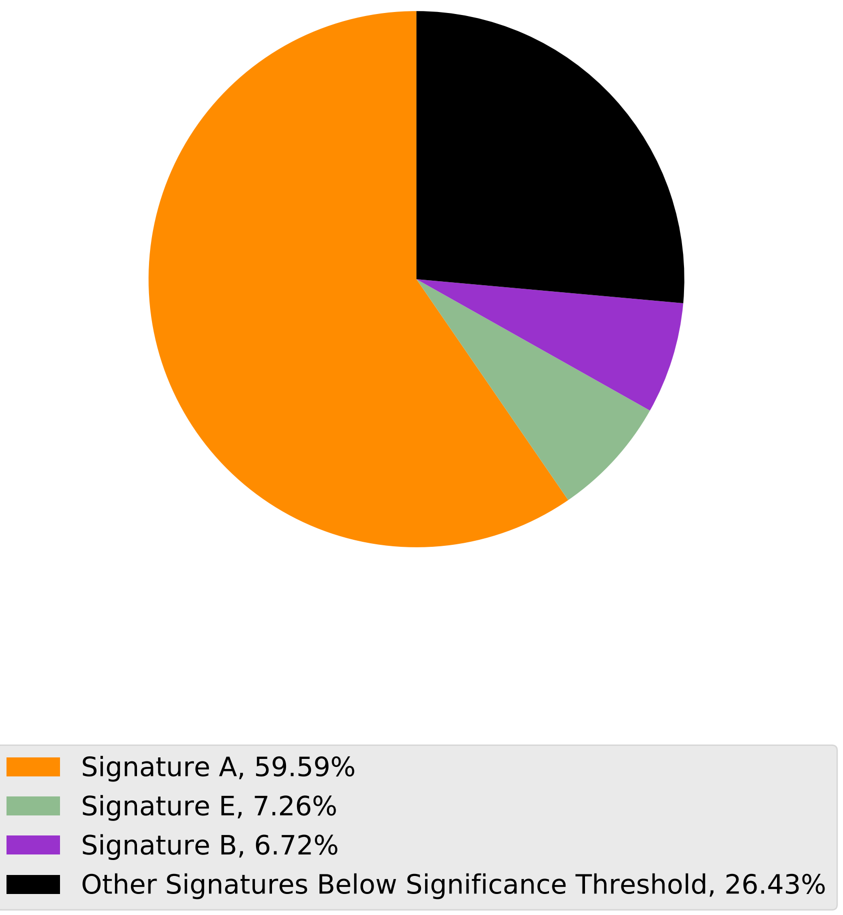

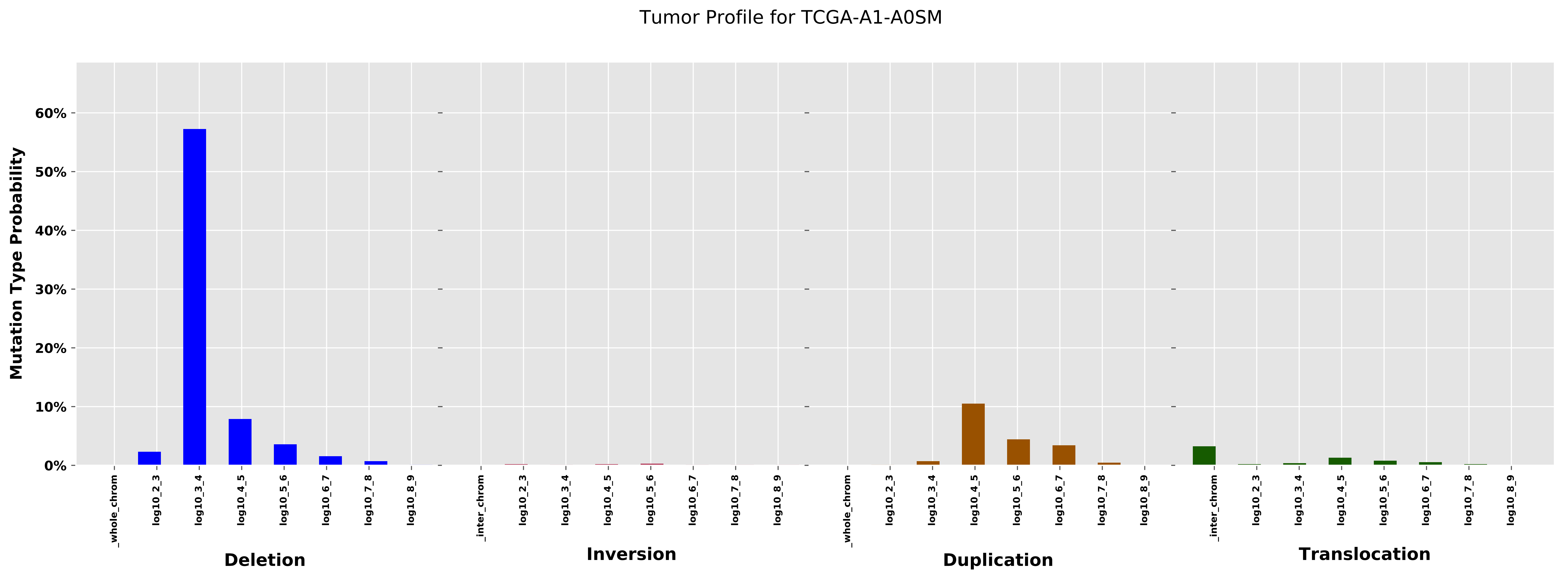

Tumor Profile for TCGA-A1-A0SM
Mutation Type Probability
60%
50%
40%
30%
20%
10%
0%
_whole_chrom
log10_2_3
log10_3_4
log10_4_5
log10_5_6
log10_6_7
log10_7_8
log10_8_9
_inter_chrom
Deletion
Inversion
Duplication
Translocation

Cancer processes Weights for TCGA-C8-A130

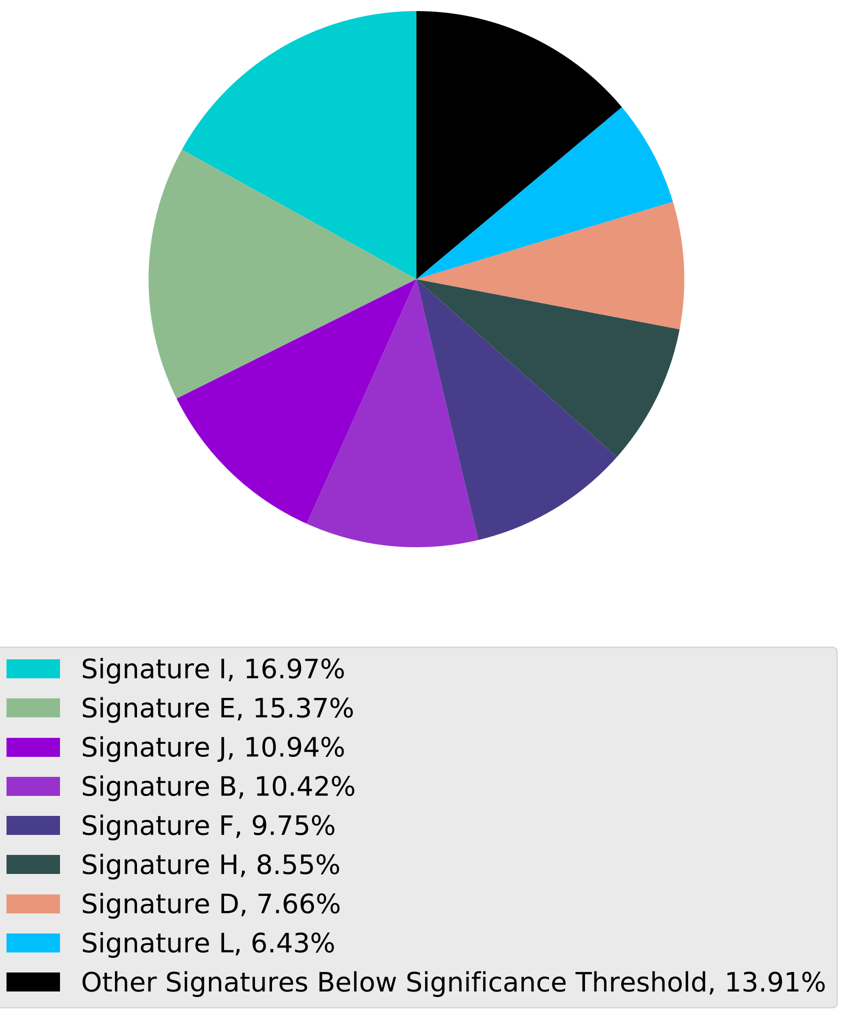

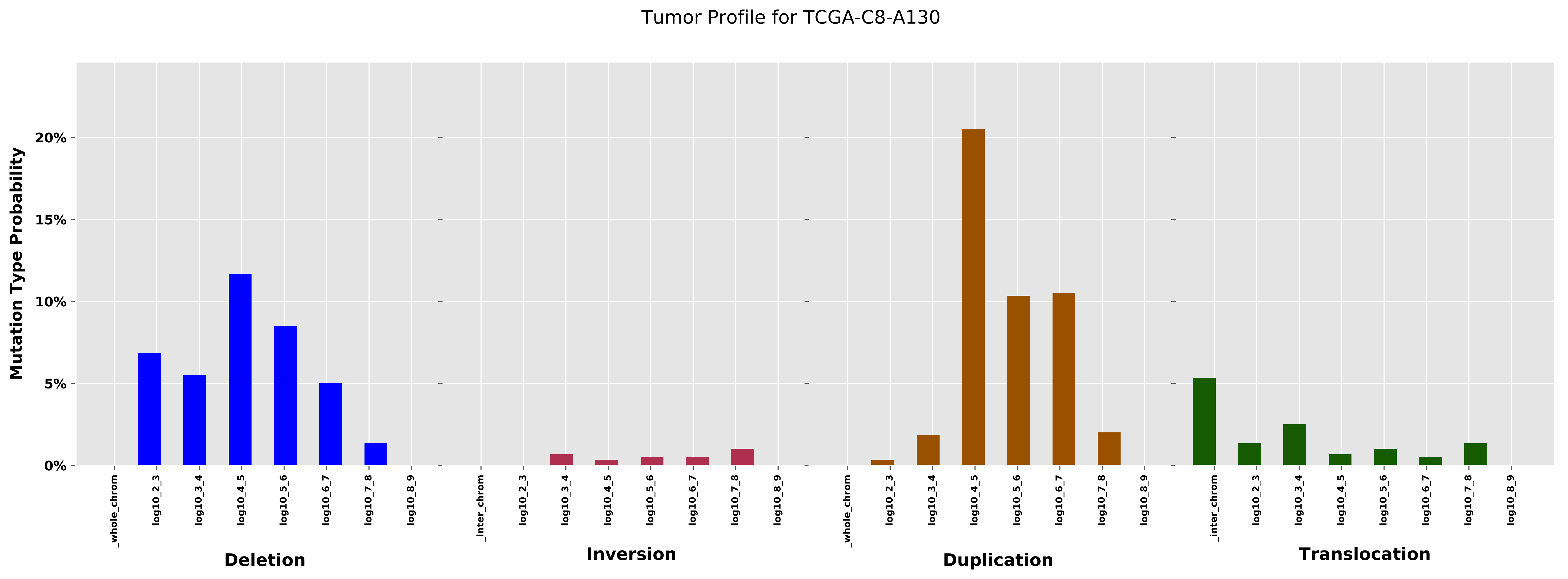

Tumor Profile for TCGA-C8-A130
Mutation Type Probability
20%
15%
10%
5%
0%
_whole_chrom
log10_2_3
log10_3_4
log10_4_5
log10_5_6
log10_6_7
log10_7_8
log10_8_9
_inter_chrom
Deletion
Inversion
Duplication
Translocation

Cancer processes Weights for TCGA-BH-A0H0

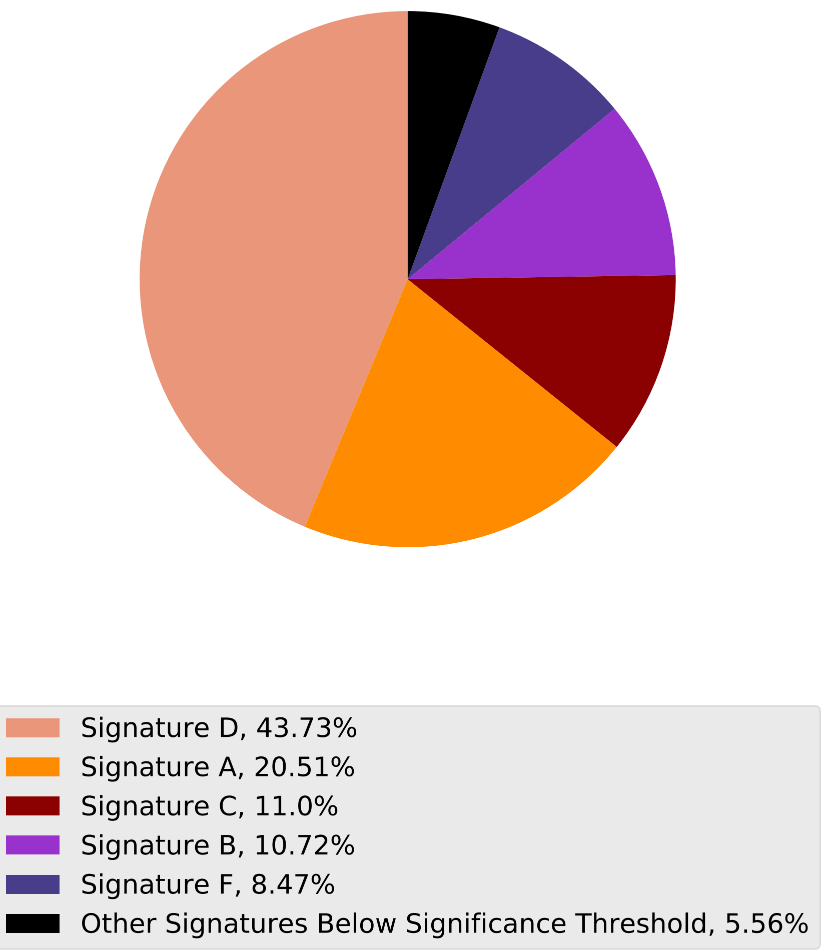

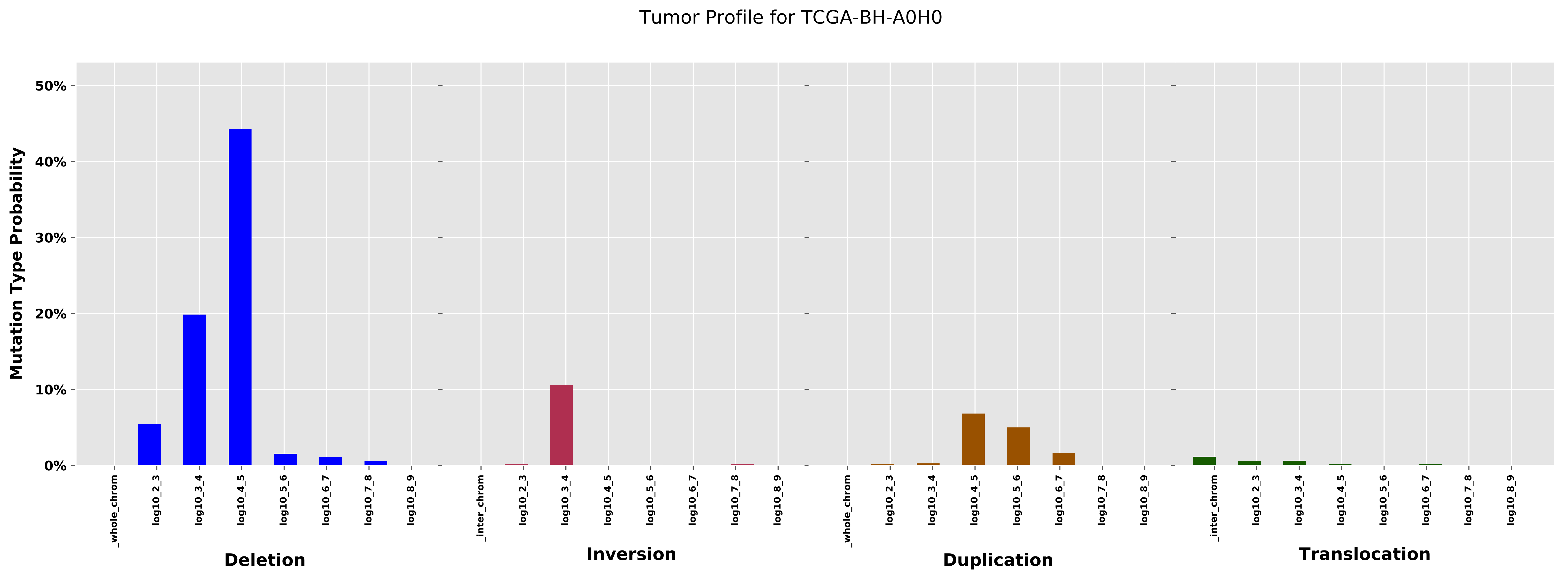

Tumor Profile for TCGA-BH-A0H0
Mutation Type Probability
0%
10%
20%
30%
40%
50%
_whole_chrom
log10_2_3
log10_3_4
log10_4_5
log10_5_6
log10_6_7
log10_7_8
log10_8_9
_inter_chrom
Deletion
Inversion
Duplication
Translocation

Cancer processes Weights for TCGA-EW-A1J5

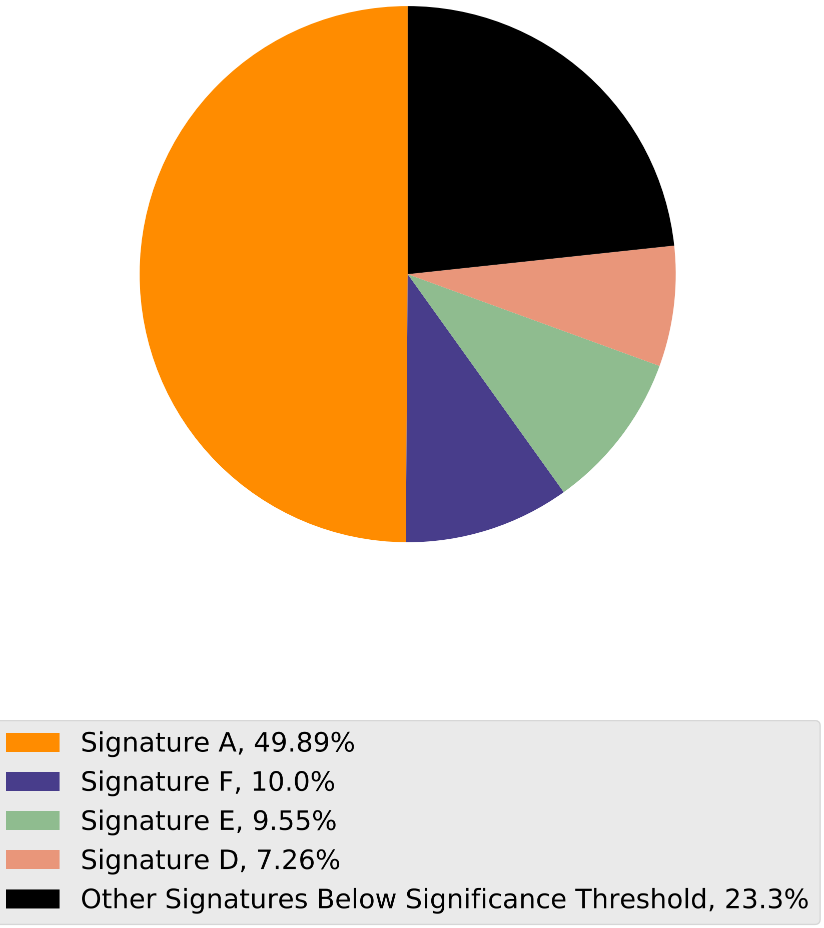

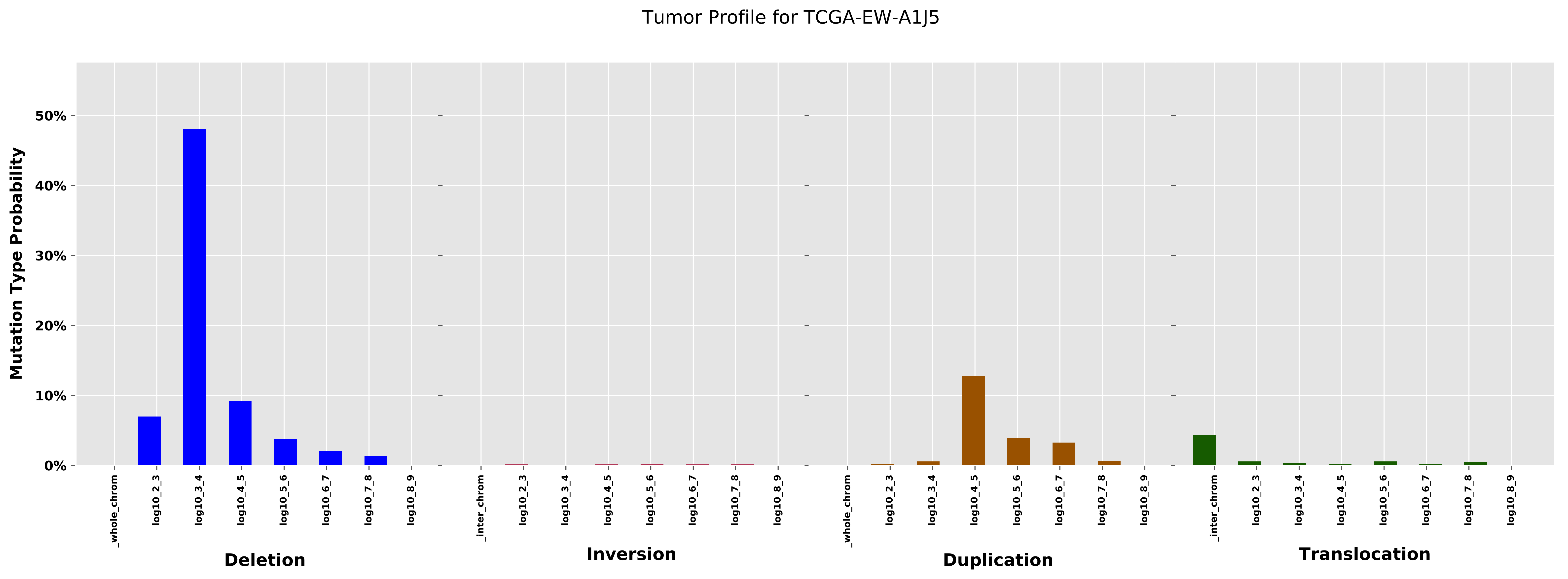

Tumor Profile for TCGA-EW-A1J5
Mutation Type Probability
0%
10%
20%
30%
40%
50%
_whole_chrom
log10_2_3
log10_3_4
log10_4_5
log10_5_6
log10_6_7
log10_7_8
log10_8_9
_inter_chrom
Deletion
Inversion
Duplication
Translocation

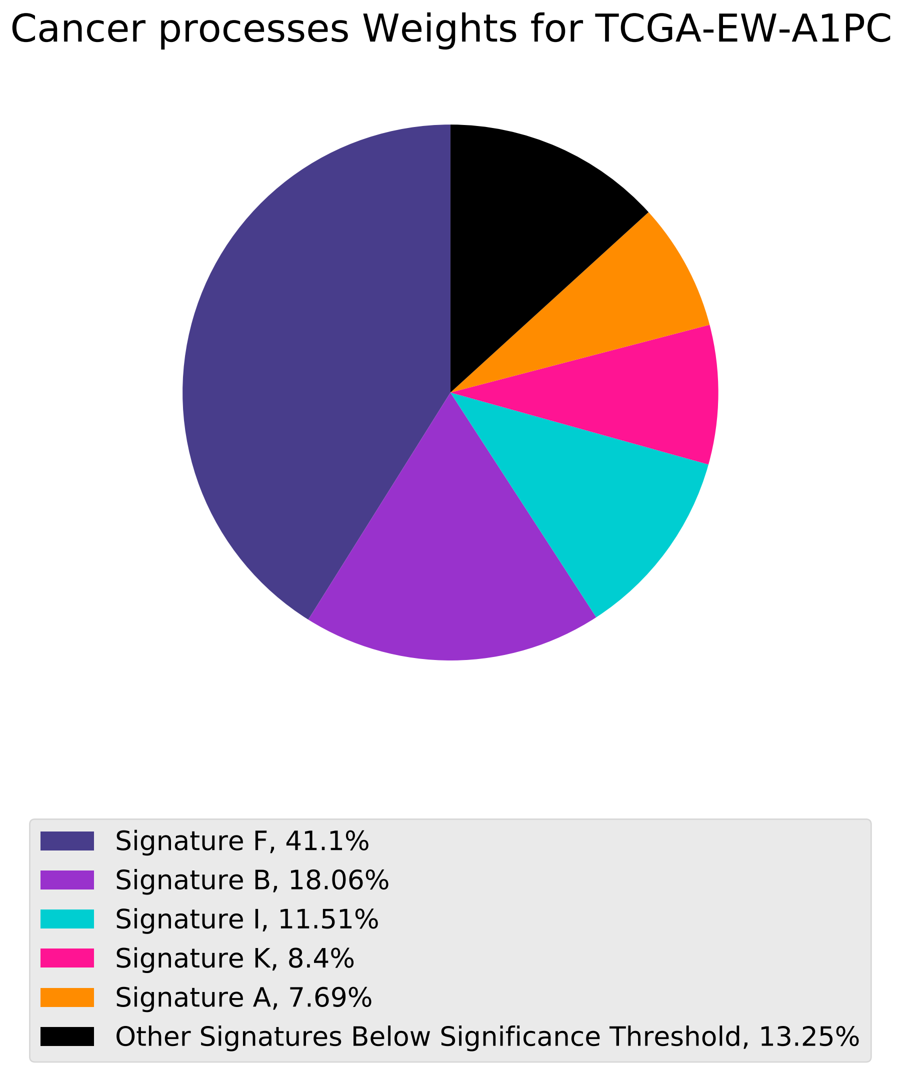
Cancer processes Weights for TCGA-EW-A1PC
Signature F, 41.1%
Signature B, 18.06%
Signature I, 11.51%
Signature K, 8.4%
Signature A, 7.69%
Other Signatures Below Significance Threshold, 13.25%

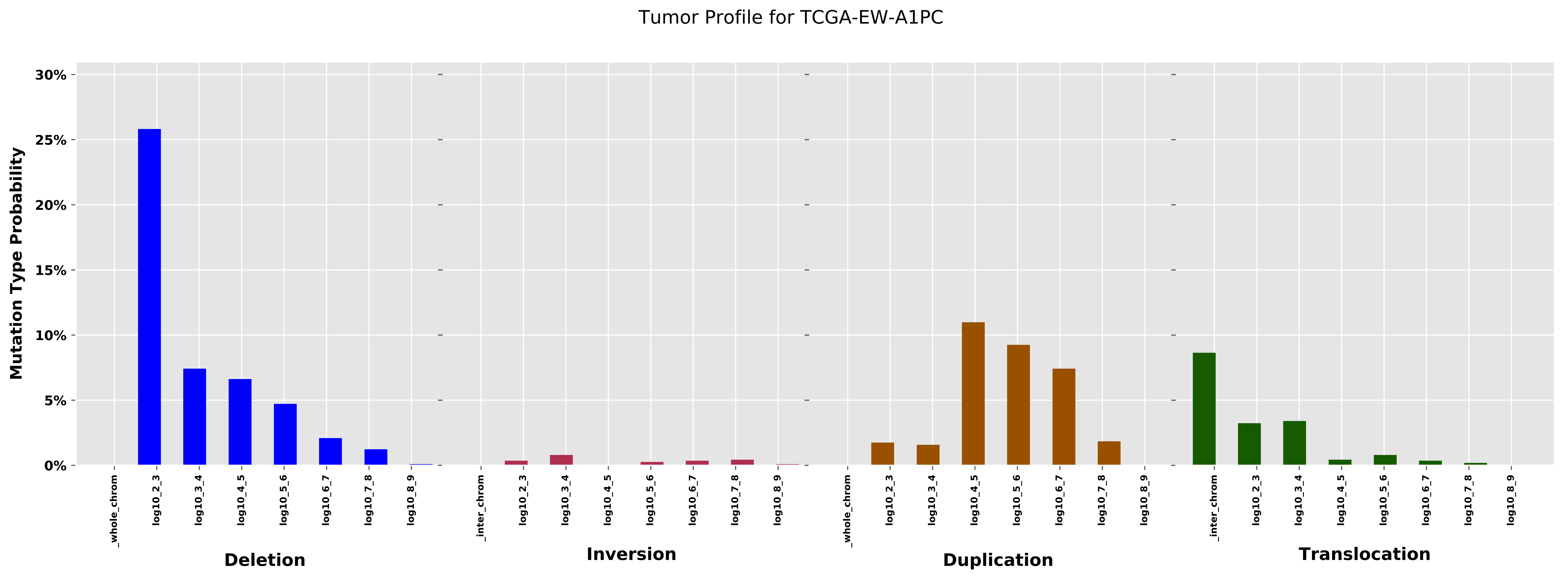

Tumor Profile for TCGA-EW-A1PC
Mutation Type Probability
30%
25%
20%
15%
10%
5%
0%
_whole_chrom
log10_2_3
log10_3_4
log10_4_5
log10_5_6
log10_6_7
log10_7_8
log10_8_9
_inter_chrom
Deletion
Inversion
Duplication
Translocation

Cancer processes Weights for TCGA-BH-A0E0

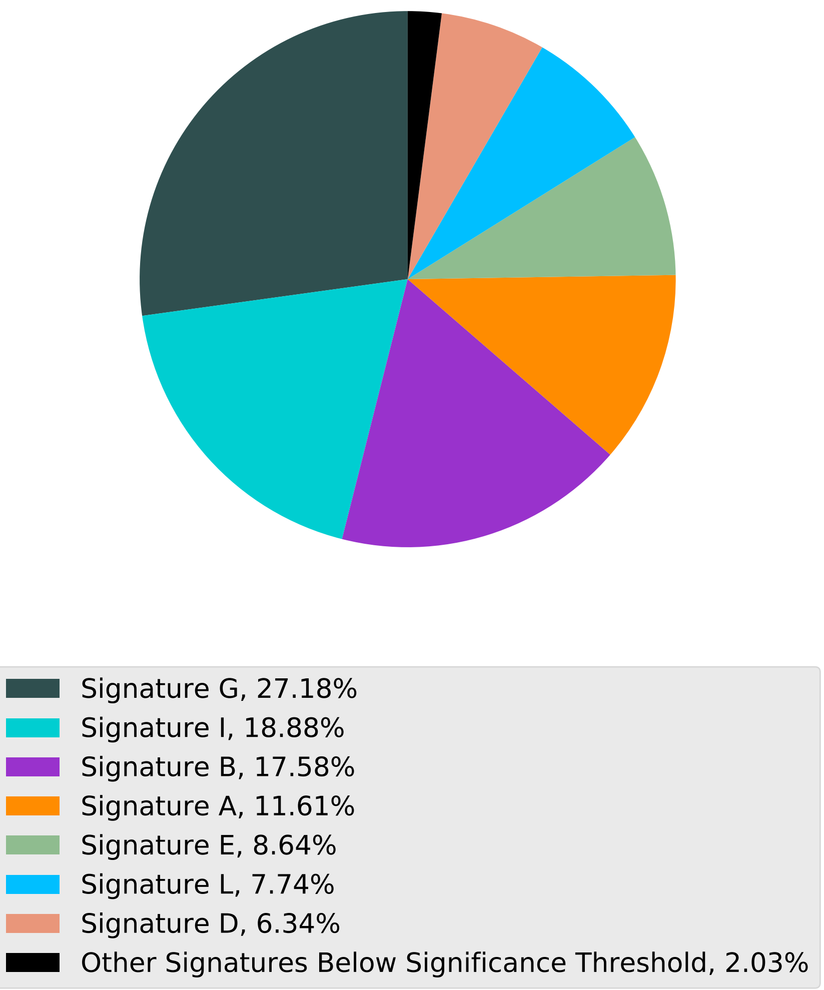

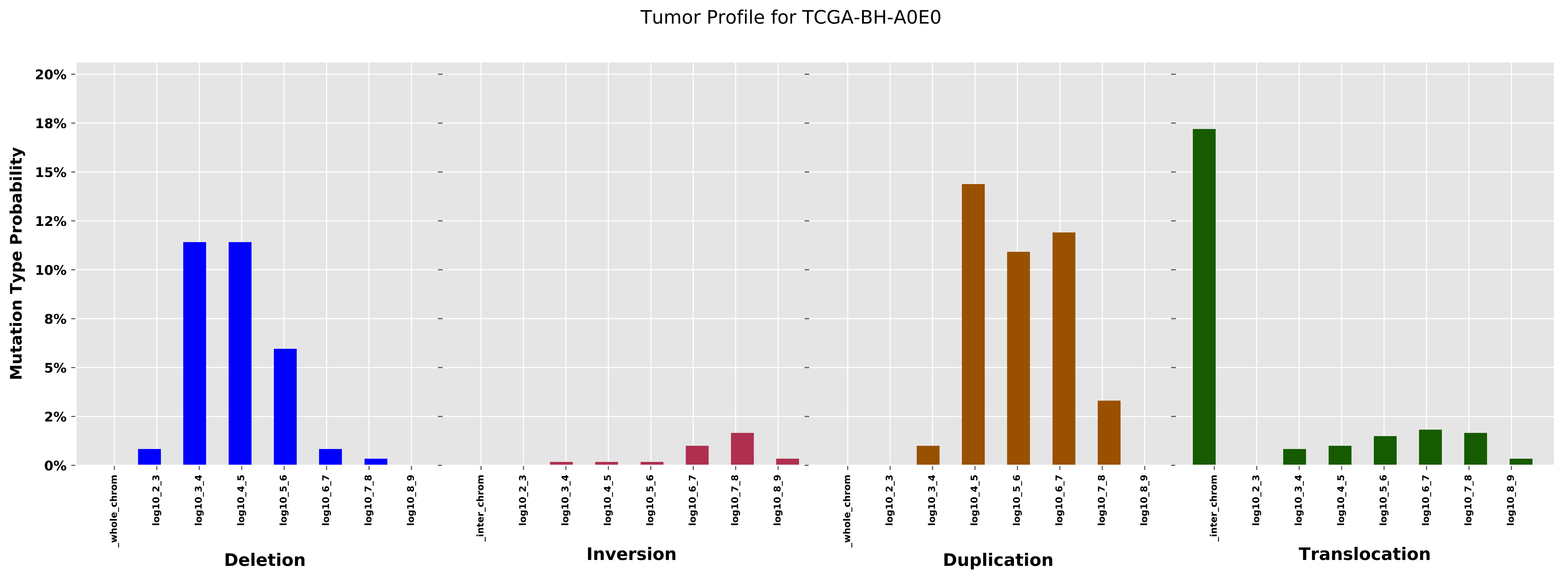

Tumor Profile for TCGA-BH-A0E0
Mutation Type Probability
20%
18%
15%
12%
10%
8%
5%
2%
0%
_whole_chrom
log10_2_3
log10_3_4
log10_4_5
log10_5_6
log10_6_7
log10_7_8
log10_8_9
_inter_chrom
Deletion
Inversion
Duplication
Translocation

Cancer processes Weights for TCGA-A2-A0D0

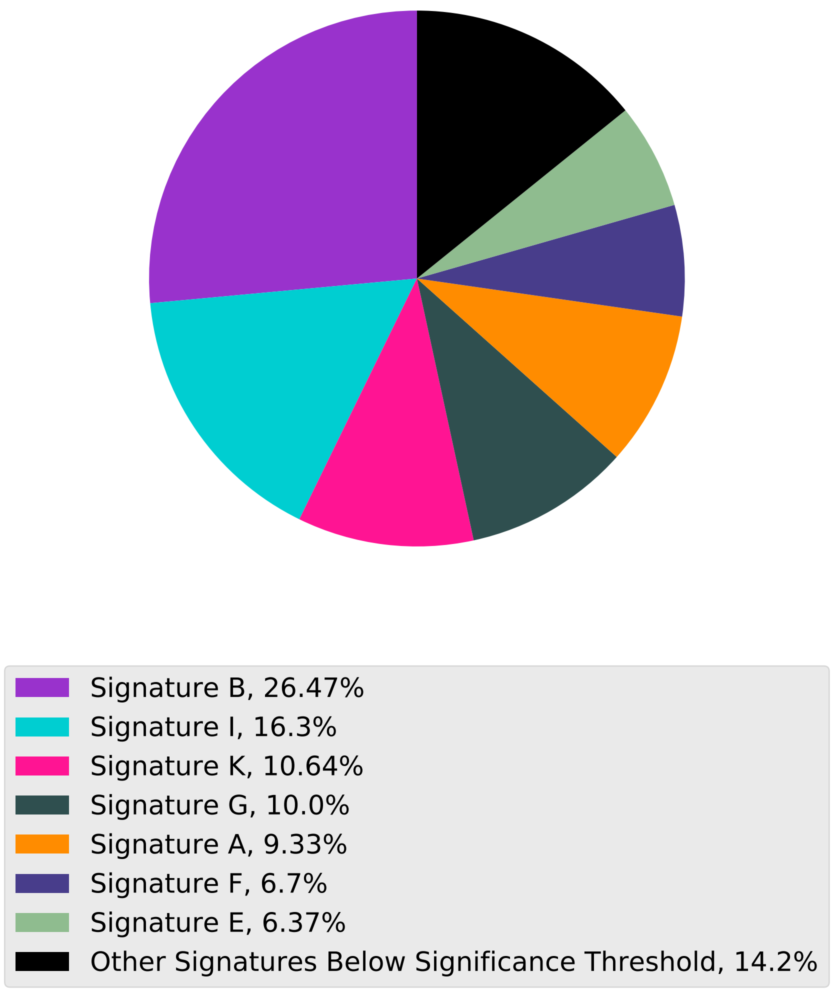

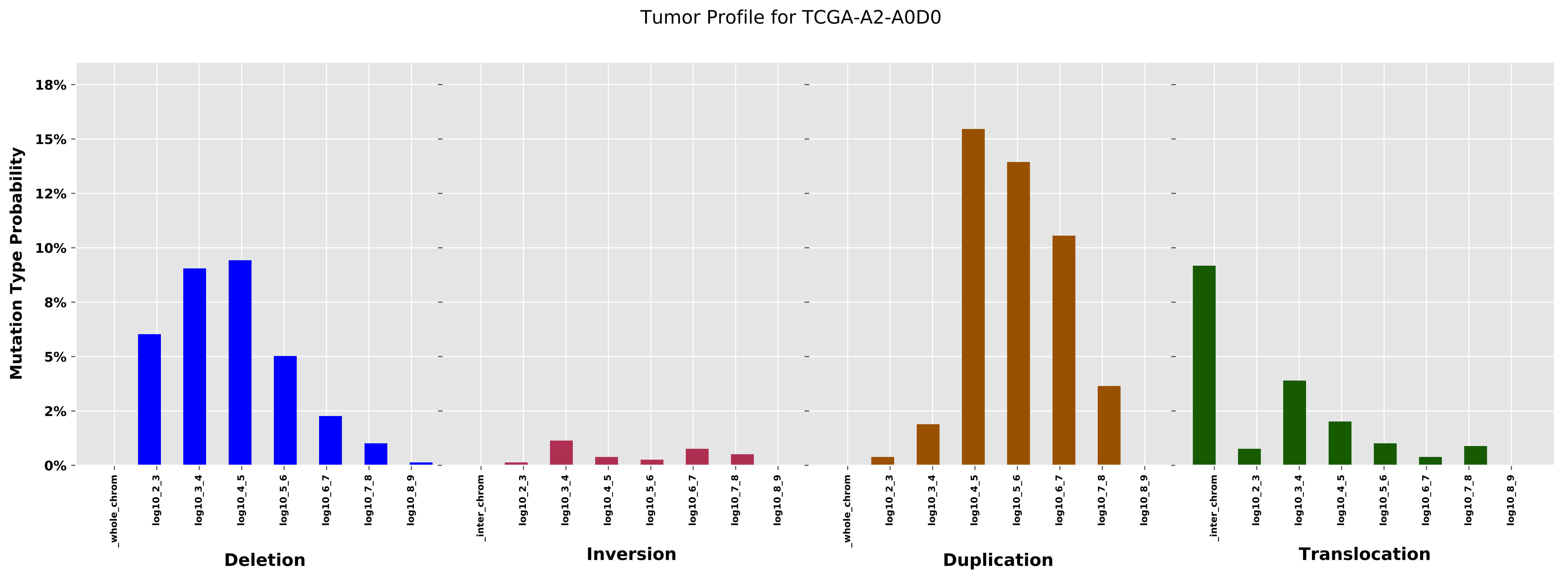

Tumor Profile for TCGA-A2-A0D0
Mutation Type Probability
18%
15%
12%
10%
8%
5%
2%
0%
_whole_chrom
log10_2_3
log10_3_4
log10_4_5
log10_5_6
log10_6_7
log10_7_8
log10_8_9
_inter_chrom
Deletion
Inversion
Duplication
Translocation

Cancer processes Weights for TCGA-AA-3956

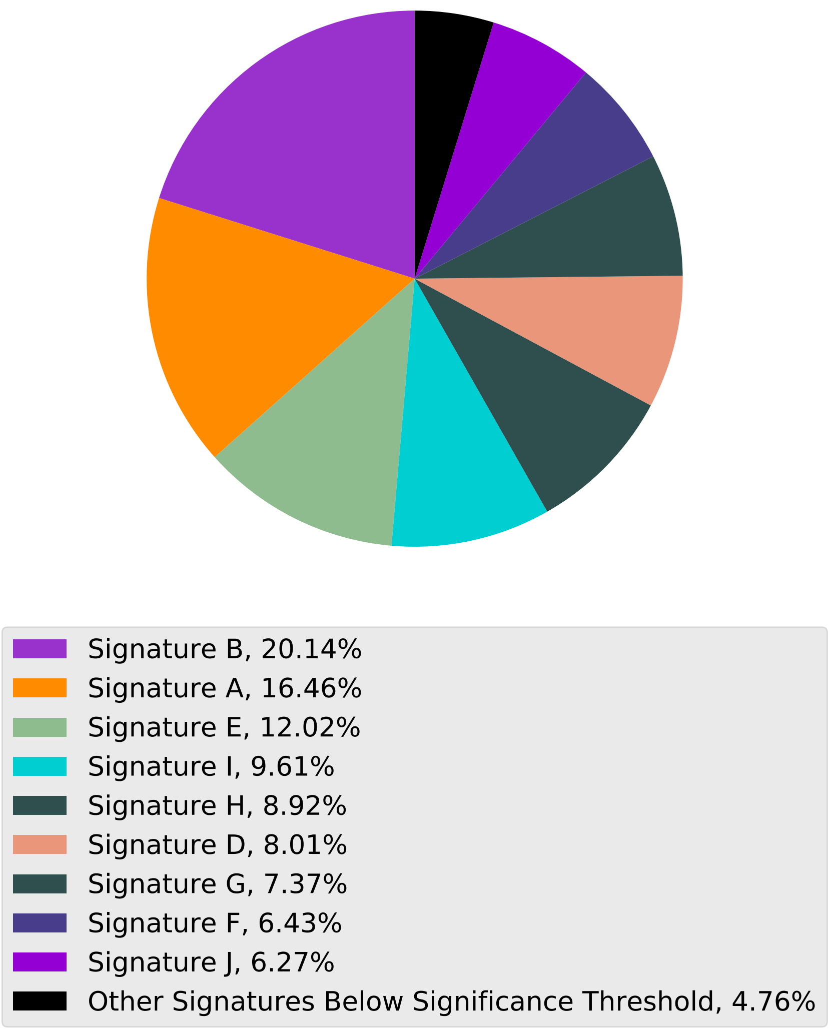

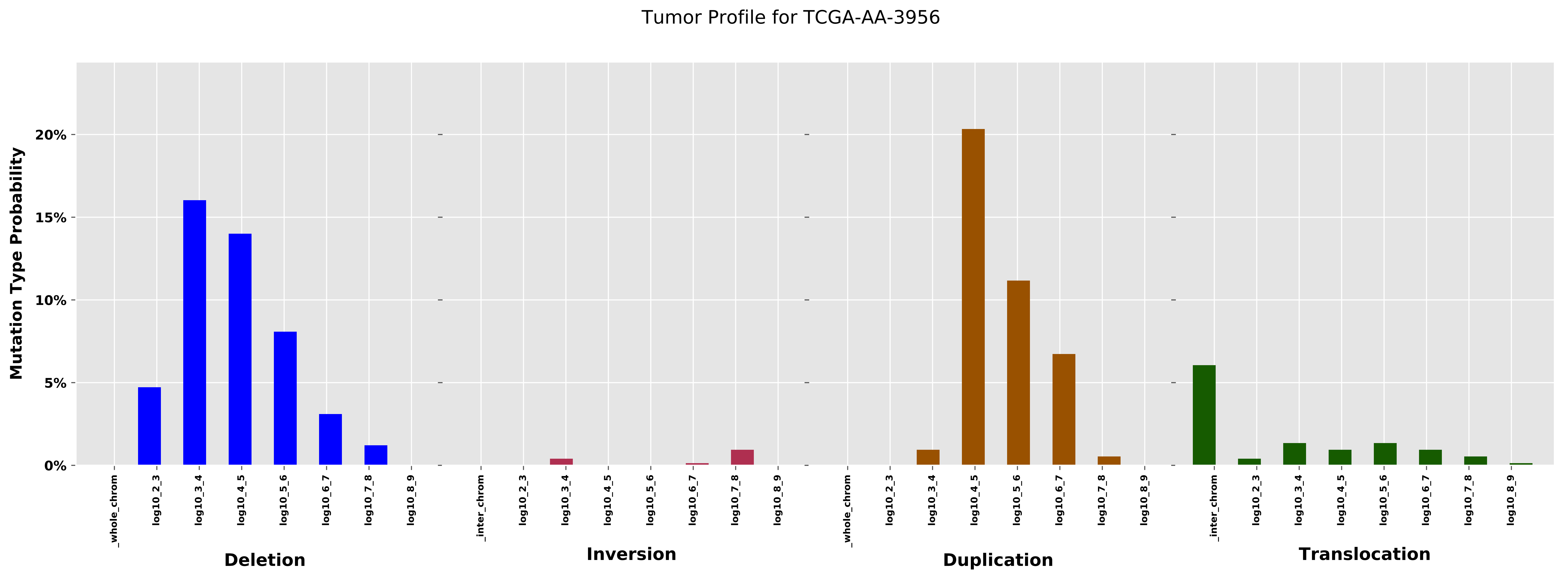

Tumor Profile for TCGA-AA-3956
Mutation Type Probability
20%
15%
10%
5%
0%
_whole_chrom
log10_2_3
log10_3_4
log10_4_5
log10_5_6
log10_6_7
log10_7_8
log10_8_9
_inter_chrom
log10_2_3
log10_3_4
log10_4_5
log10_5_6
log10_6_7
log10_7_8
log10_8_9
_whole_chrom
log10_2_3
log10_3_4
log10_4_5
log10_5_6
log10_6_7
log10_7_8
log10_8_9
_inter_chrom
log10_2_3
log10_3_4
log10_4_5
log10_5_6
log10_6_7
log10_7_8
log10_8_9
Deletion
Inversion
Duplication
Translocation

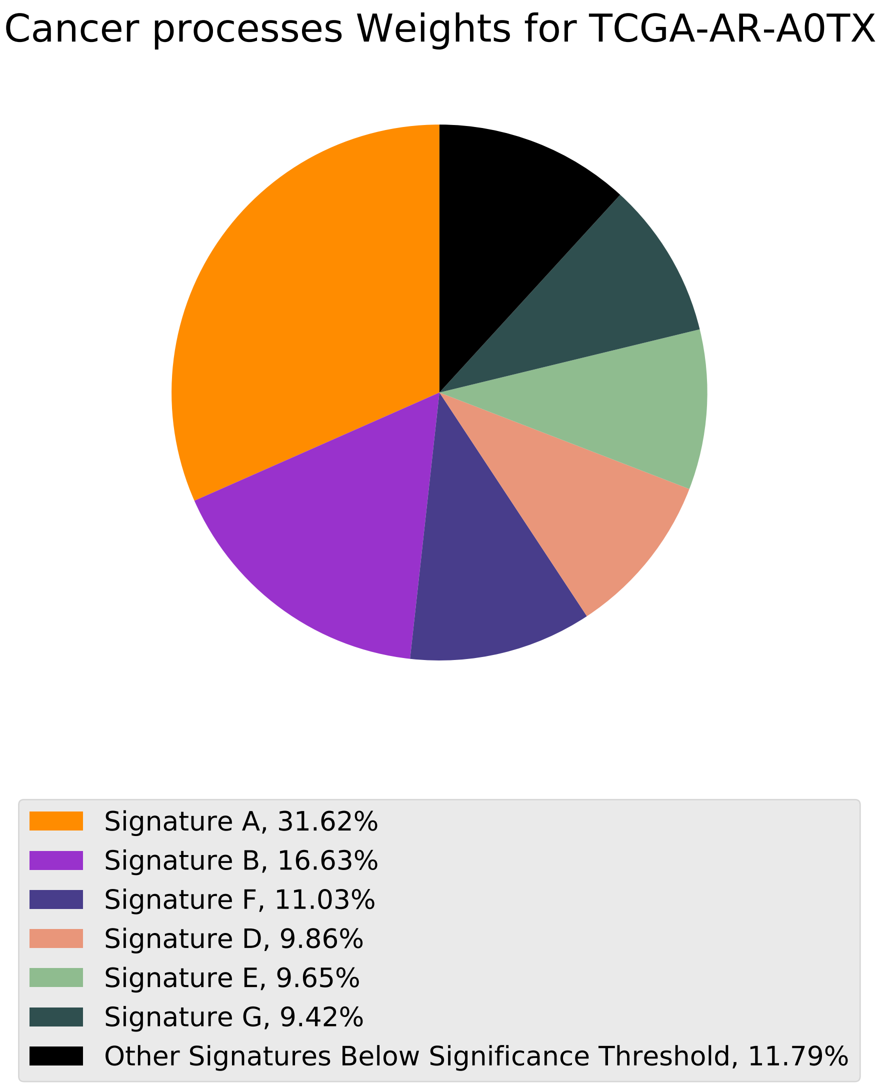
Cancer processes Weights for TCGA-AR-A0TX
Signature A, 31.62%
Signature B, 16.63%
Signature F, 11.03%
Signature D, 9.86%
Signature E, 9.65%
Signature G, 9.42%
Other Signatures Below Significance Threshold, 11.79%

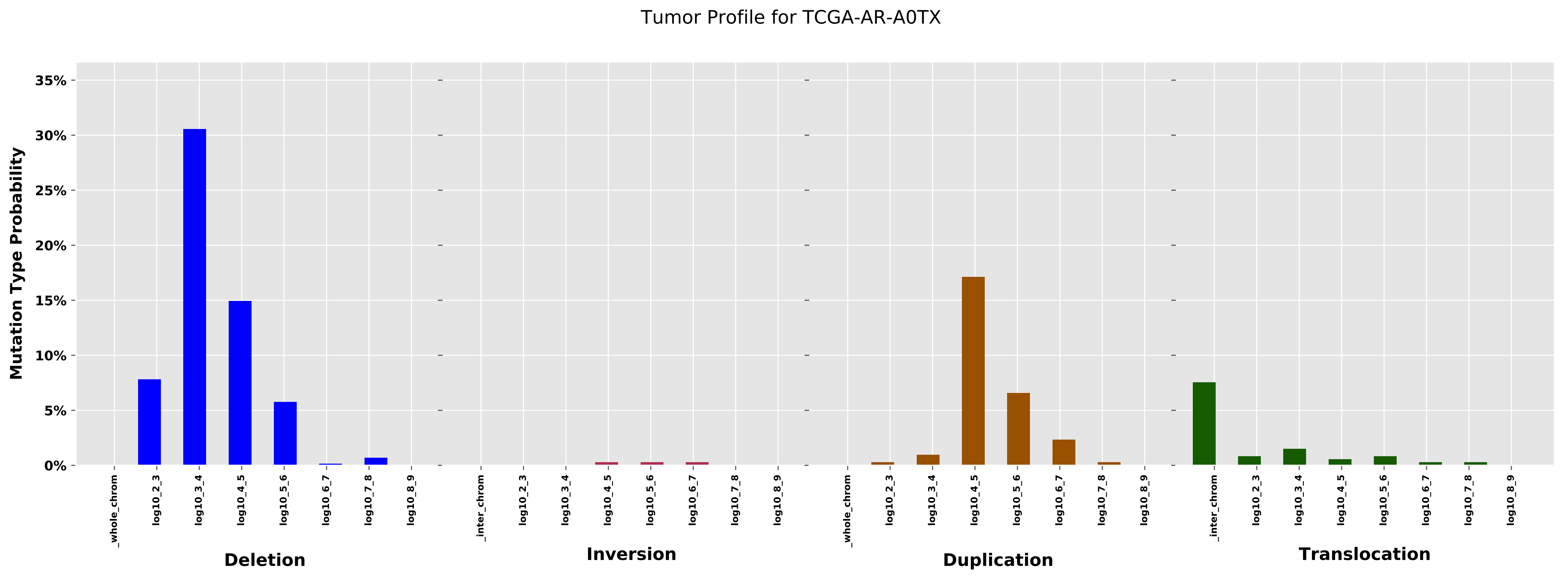

Tumor Profile for TCGA-AR-A0TX
Mutation Type Probability
35%
30%
25%
20%
15%
10%
5%
0%
_whole_chrom
log10_2_3
log10_3_4
log10_4_5
log10_5_6
log10_6_7
log10_7_8
log10_8_9
Deletion
_inter_chrom
log10_2_3
log10_3_4
log10_4_5
log10_5_6
log10_6_7
log10_7_8
log10_8_9
Inversion
_whole_chrom
log10_2_3
log10_3_4
log10_4_5
log10_5_6
log10_6_7
log10_7_8
log10_8_9
Duplication
_inter_chrom
log10_2_3
log10_3_4
log10_4_5
log10_5_6
log10_6_7
log10_7_8
log10_8_9
Translocation

Cancer processes Weights for TCGA-EI-6917

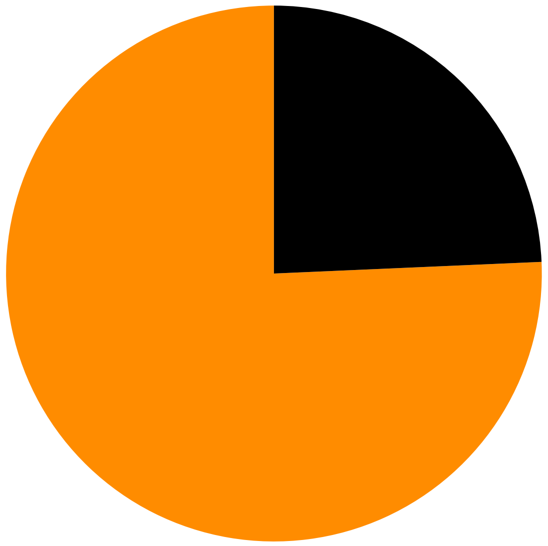

Signature A, 75.69%
Other Signatures Below Significance Threshold, 24.31%

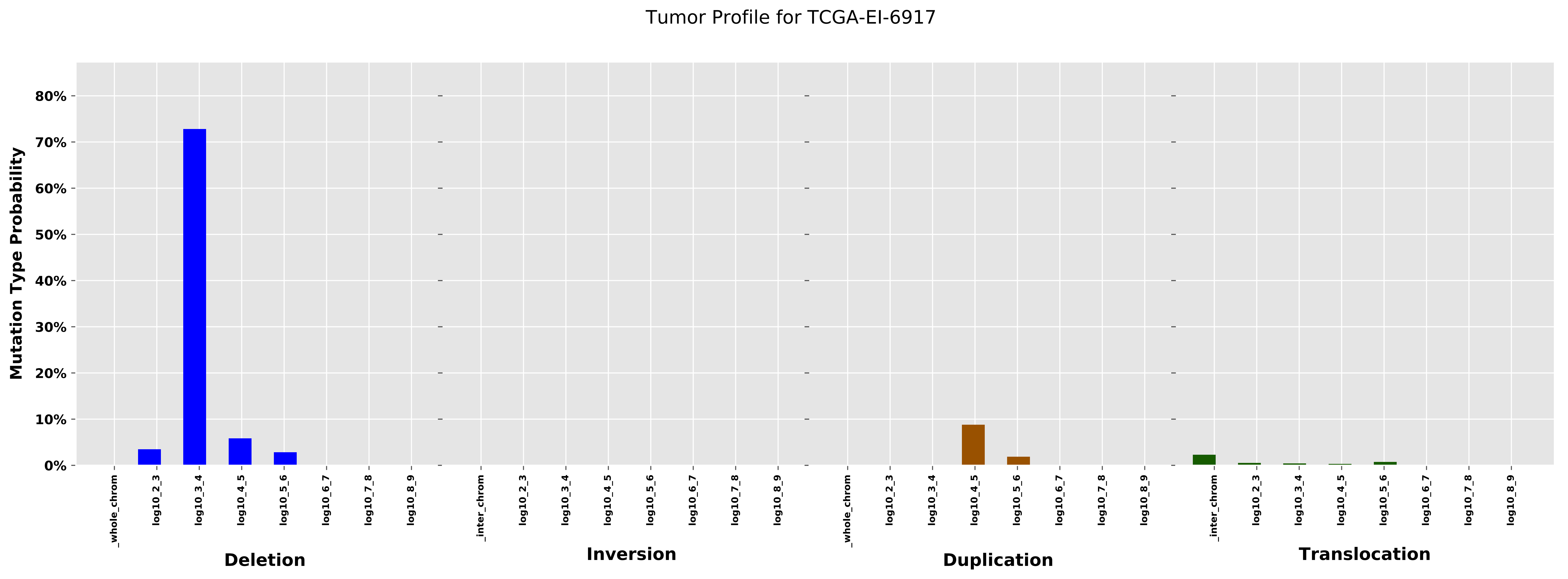

Tumor Profile for TCGA-EI-6917
Mutation Type Probability
80%
70%
60%
50%
40%
30%
20%
10%
0%
_whole_chrom
log10_2_3
log10_3_4
log10_4_5
log10_5_6
log10_6_7
log10_7_8
log10_8_9
_inter_chrom
Deletion
Inversion
Duplication
Translocation

Cancer processes Weights for TCGA-AA-A01X

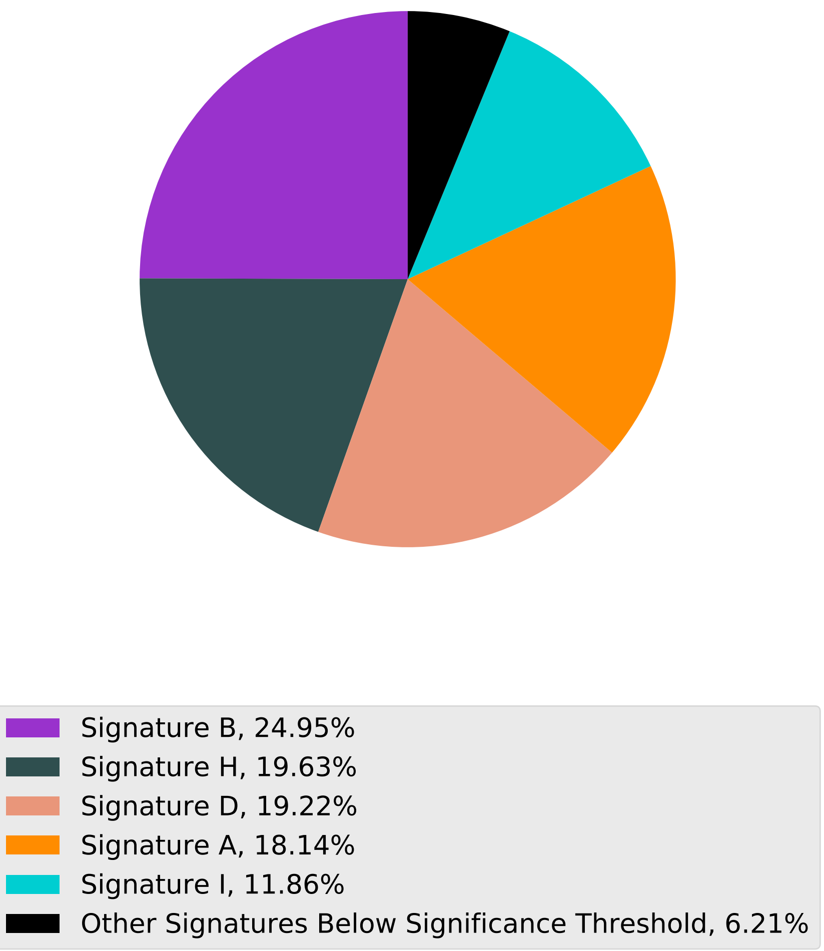

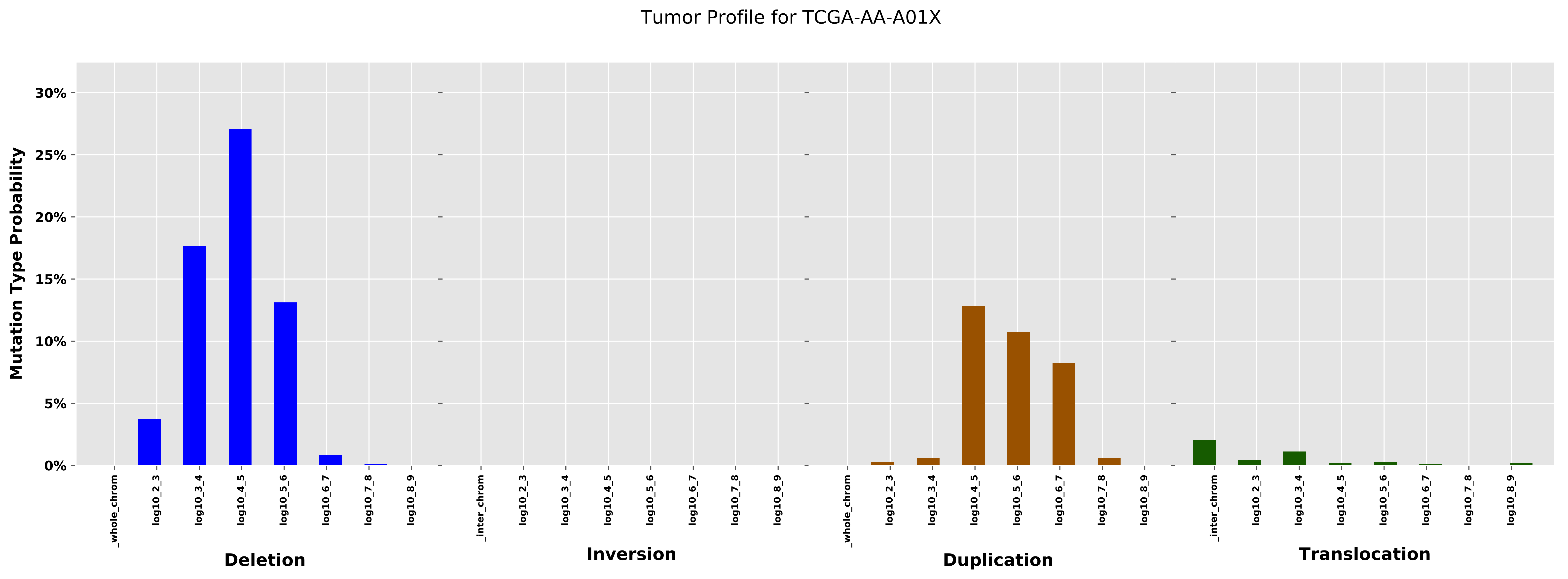

Tumor Profile for TCGA-AA-A01X
Mutation Type Probability
0%
5%
10%
15%
20%
25%
30%
_whole_chrom
log10_2_3
log10_3_4
log10_4_5
log10_5_6
log10_6_7
log10_7_8
log10_8_9
_inter_chrom
Deletion
Inversion
Duplication
Translocation

Cancer processes Weights for TCGA-E2-A15H

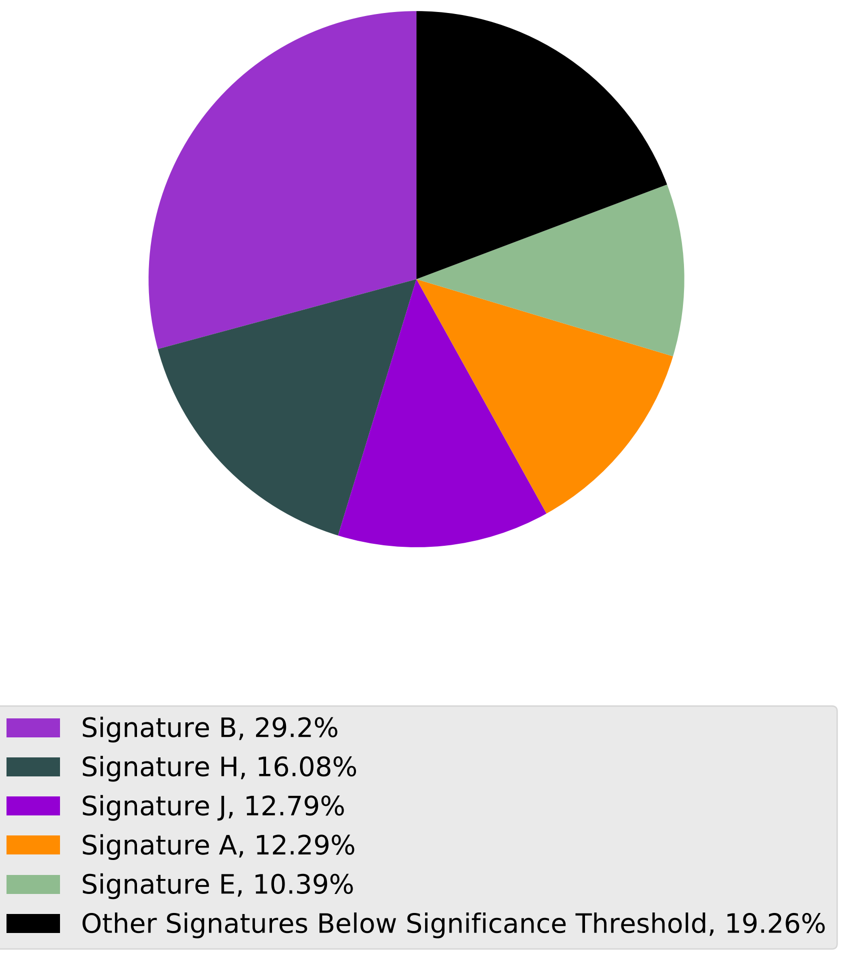

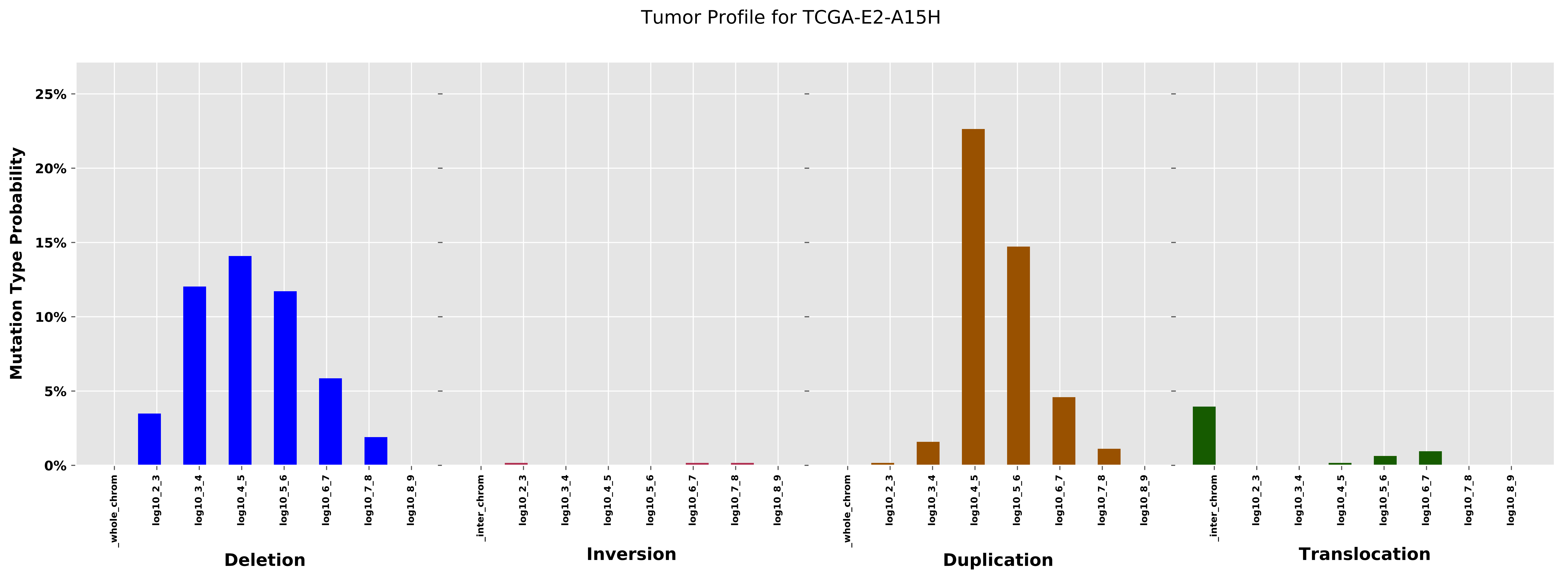
Tumor Profile for TCGA-E2-A15H
Mutation Type Probability
25%
20%
15%
10%
5%
0%
_whole_chrom
log10_2_3
log10_3_4
log10_4_5
log10_5_6
log10_6_7
log10_7_8
log10_8_9
Deletion
_inter_chrom
log10_2_3
log10_3_4
log10_4_5
log10_5_6
log10_6_7
log10_7_8
log10_8_9
Inversion
_whole_chrom
log10_2_3
log10_3_4
log10_4_5
log10_5_6
log10_6_7
log10_7_8
log10_8_9
Duplication
_inter_chrom
log10_2_3
log10_3_4
log10_4_5
log10_5_6
log10_6_7
log10_7_8
log10_8_9
Translocation

Cancer processes Weights for TCGA-E9-A1NH

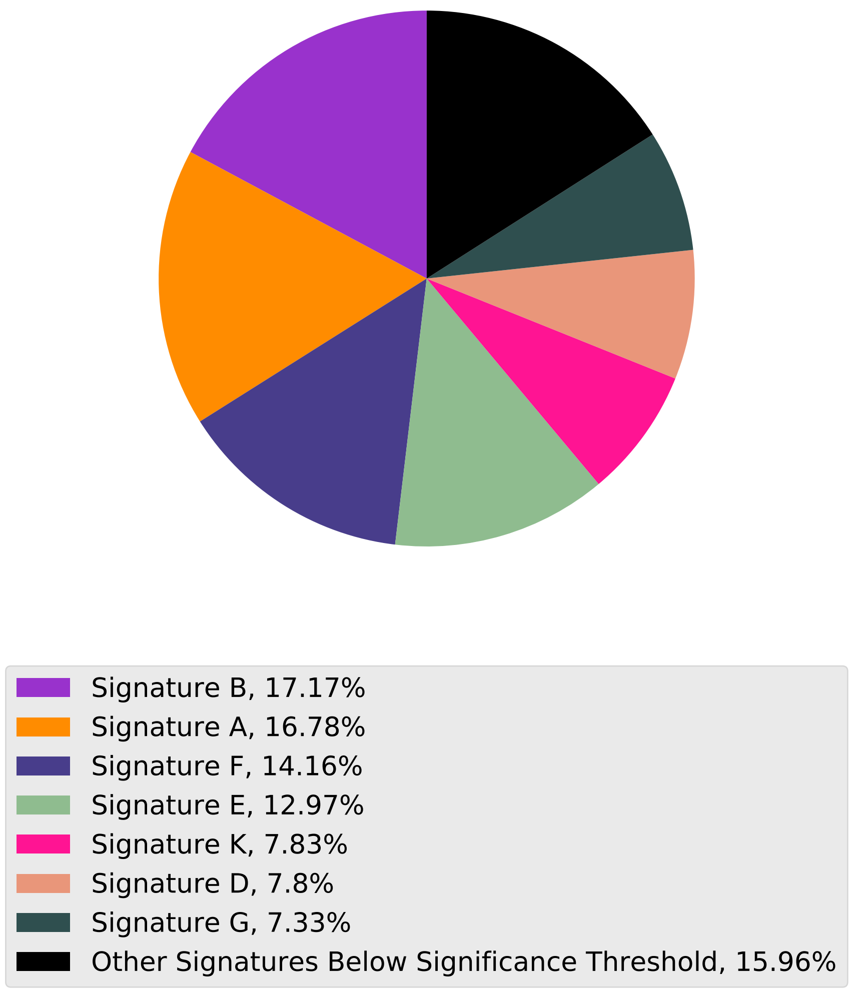

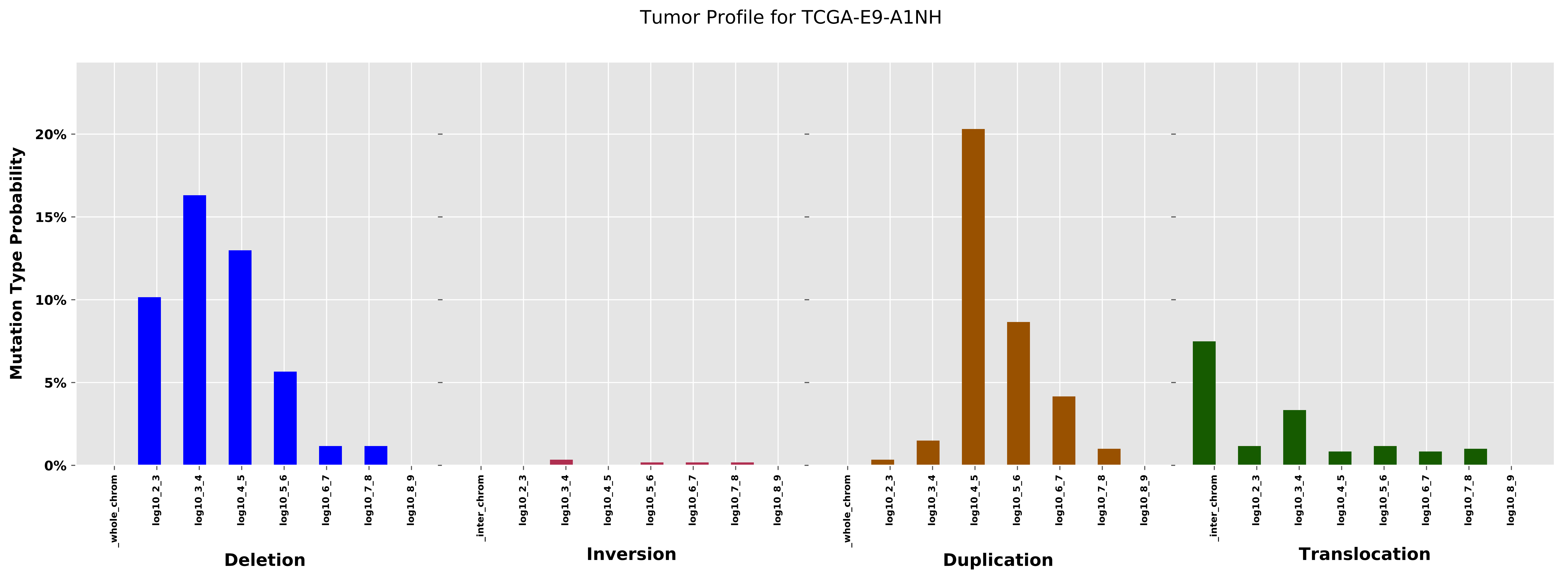
Tumor Profile for TCGA-E9-A1NH
Mutation Type Probability
0%
5%
10%
15%
20%
_whole_chrom
log10_2_3
log10_3_4
log10_4_5
log10_5_6
log10_6_7
log10_7_8
log10_8_9
_inter_chrom
Deletion
Inversion
Duplication
Translocation

Cancer processes Weights for TCGA-AA-A01R

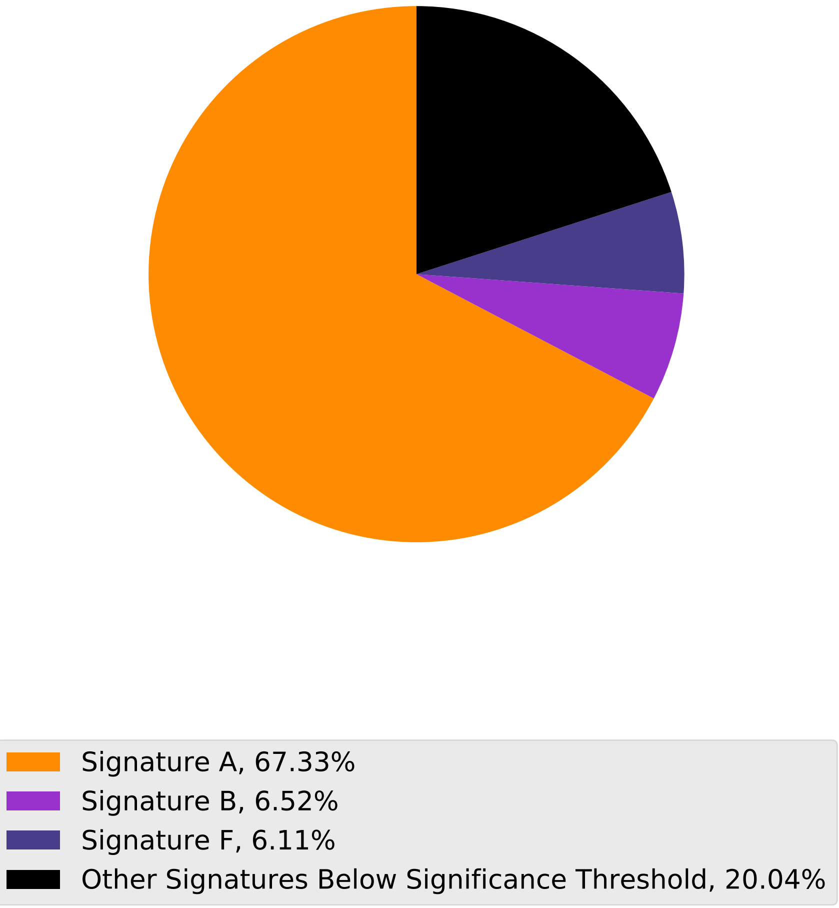

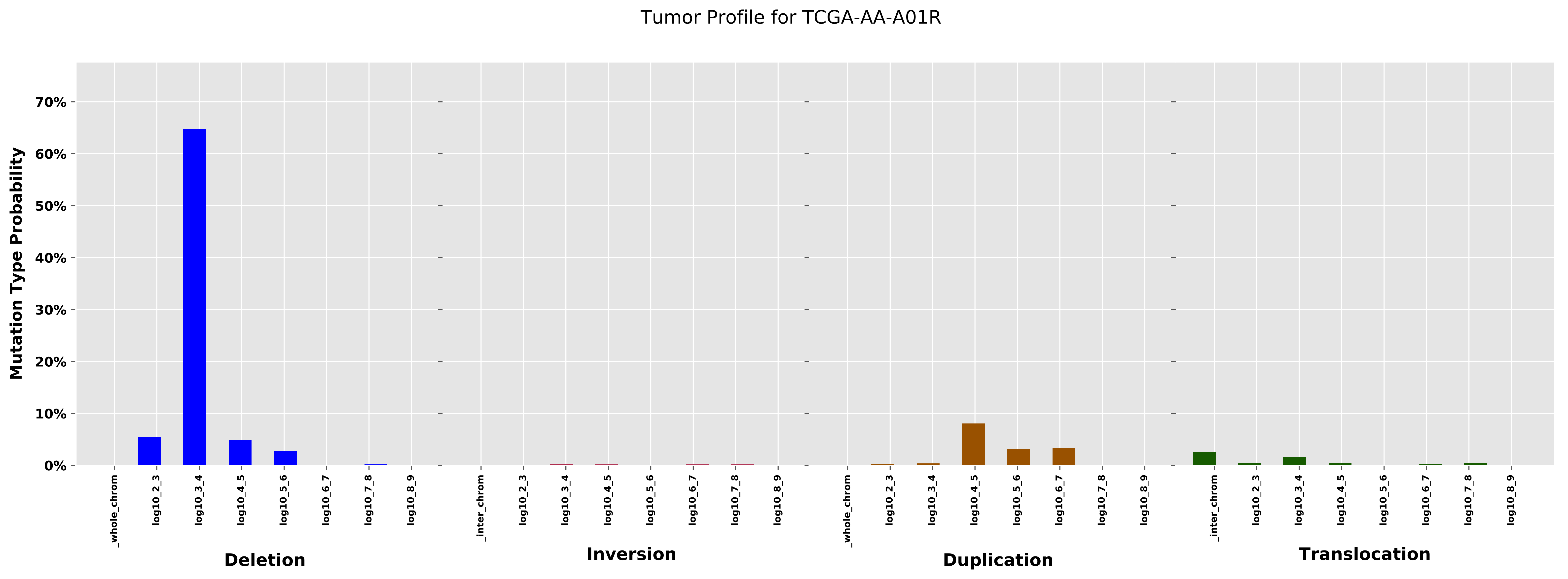

Tumor Profile for TCGA-AA-A01R
Mutation Type Probability
70%
60%
50%
40%
30%
20%
10%
0%
_whole_chrom
log10_2_3
log10_3_4
log10_4_5
log10_5_6
log10_6_7
log10_7_8
log10_8_9
_inter_chrom
Deletion
Inversion
Duplication
Translocation

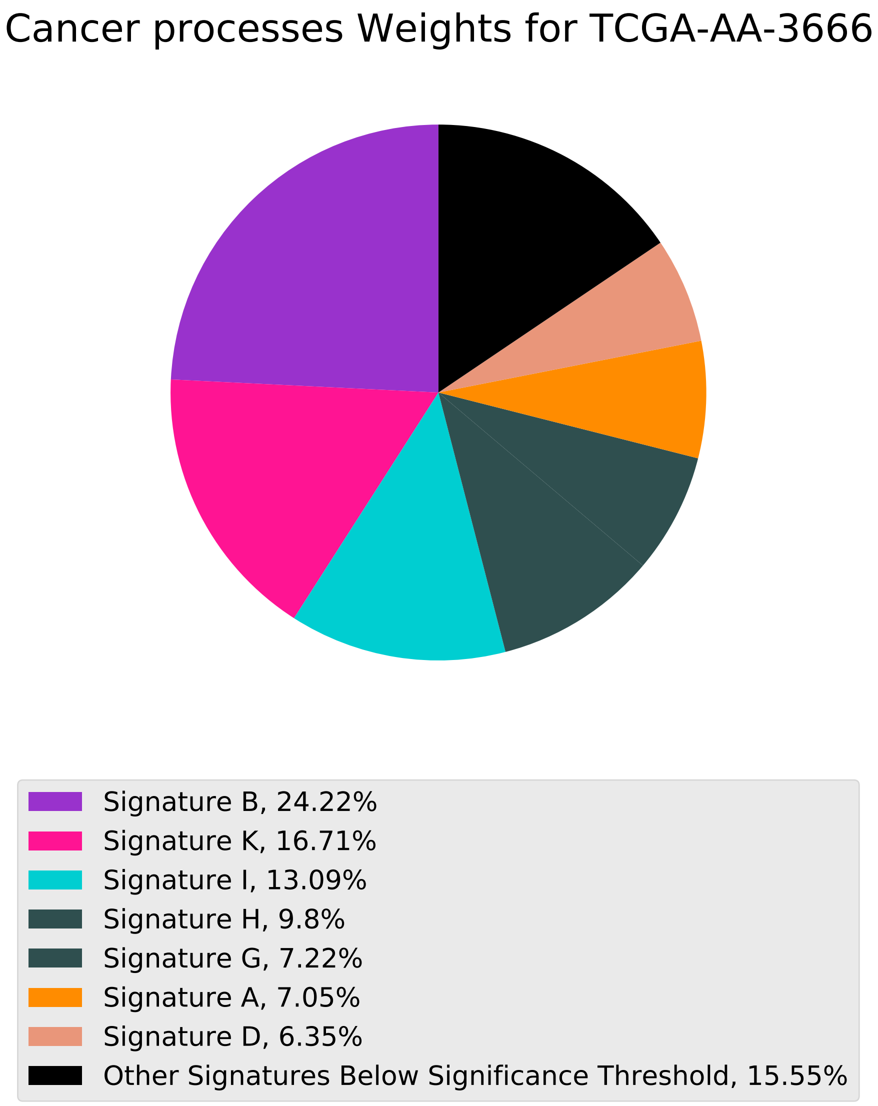
Cancer processes Weights for TCGA-AA-3666
Signature B, 24.22%
Signature K, 16.71%
Signature I, 13.09%
Signature H, 9.8%
Signature G, 7.22%
Signature A, 7.05%
Signature D, 6.35%
Other Signatures Below Significance Threshold, 15.55%

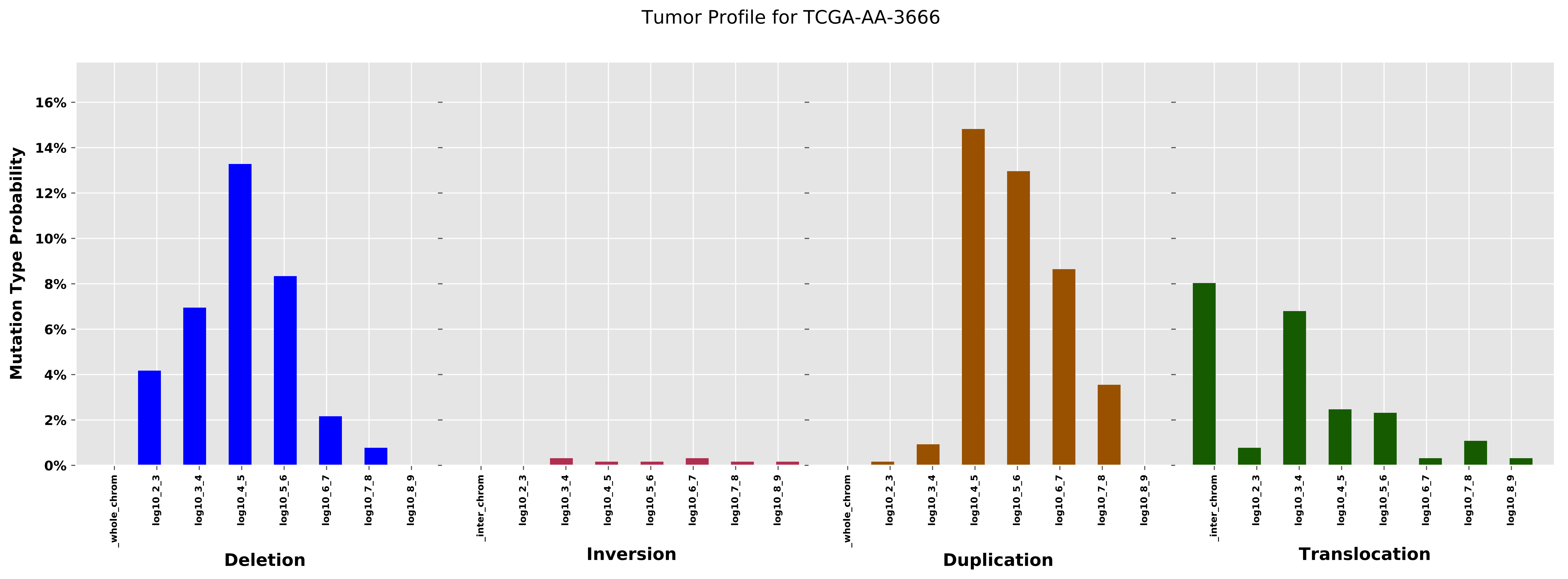

Tumor Profile for TCGA-AA-3666
Mutation Type Probability
16%
14%
12%
10%
8%
6%
4%
2%
0%
_whole_chrom
log10_2_3
log10_3_4
log10_4_5
log10_5_6
log10_6_7
log10_7_8
log10_8_9
_inter_chrom
Deletion
Inversion
Duplication
Translocation

Cancer processes Weights for TCGA-AR-A2LK

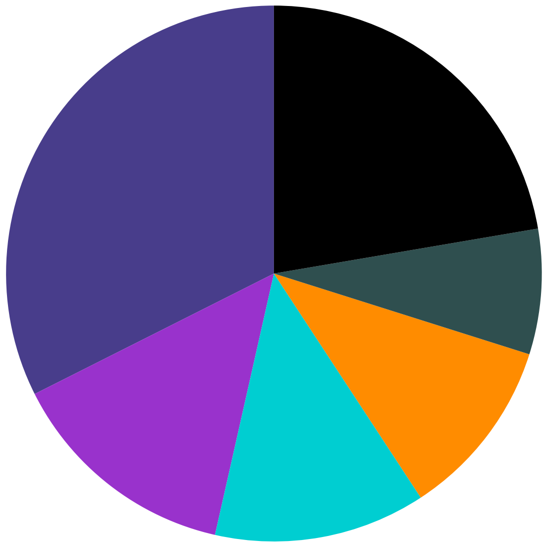

Signature F, 32.42%
Signature B, 14.05%
Signature I, 12.77%
Signature A, 10.89%
Signature G, 7.56%
Other Signatures Below Significance Threshold, 22.31%

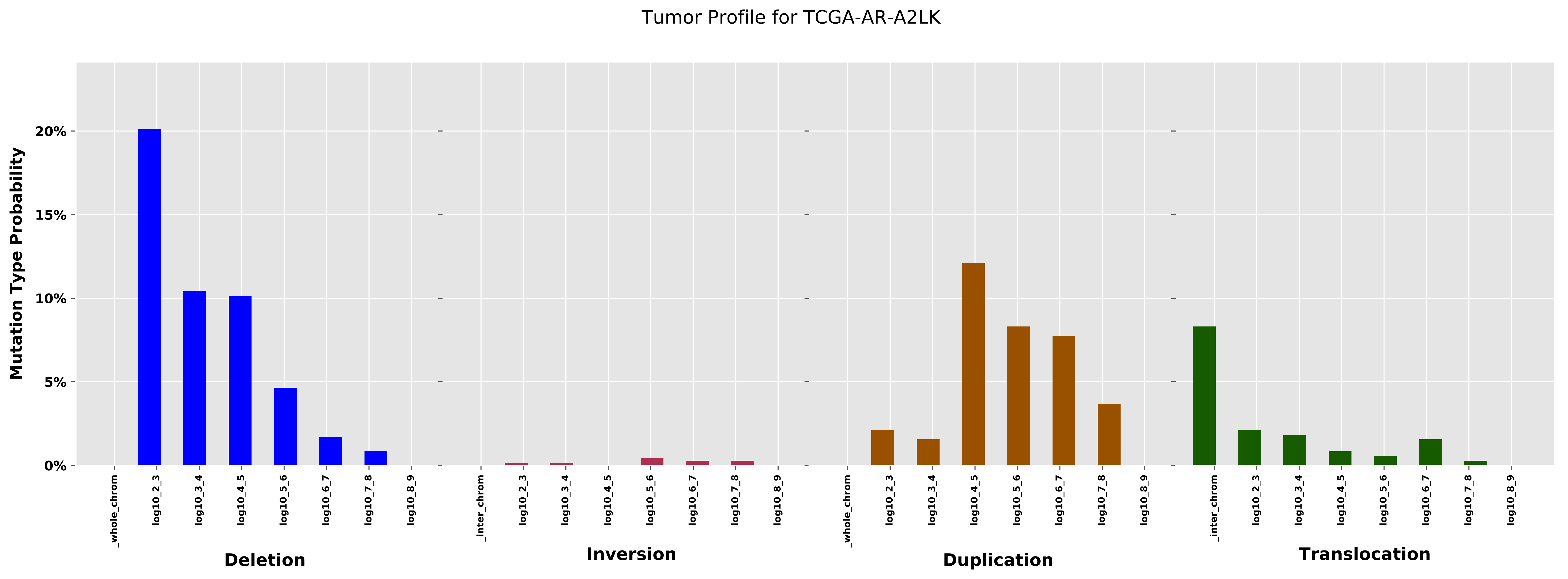
Tumor Profile for TCGA-AR-A2LK
Mutation Type Probability
20%
15%
10%
5%
0%
_whole_chrom
log10_2_3
log10_3_4
log10_4_5
log10_5_6
log10_6_7
log10_7_8
log10_8_9
_inter_chrom
Deletion
Inversion
Duplication
Translocation

Cancer processes Weights for TCGA-AA-3534

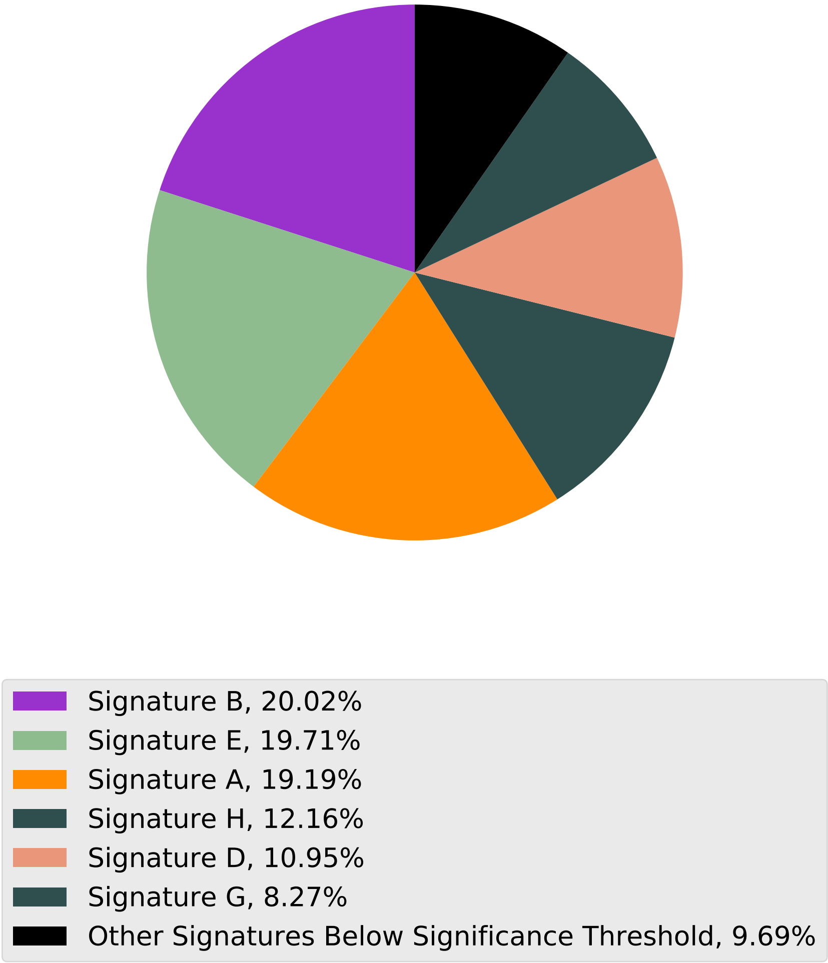

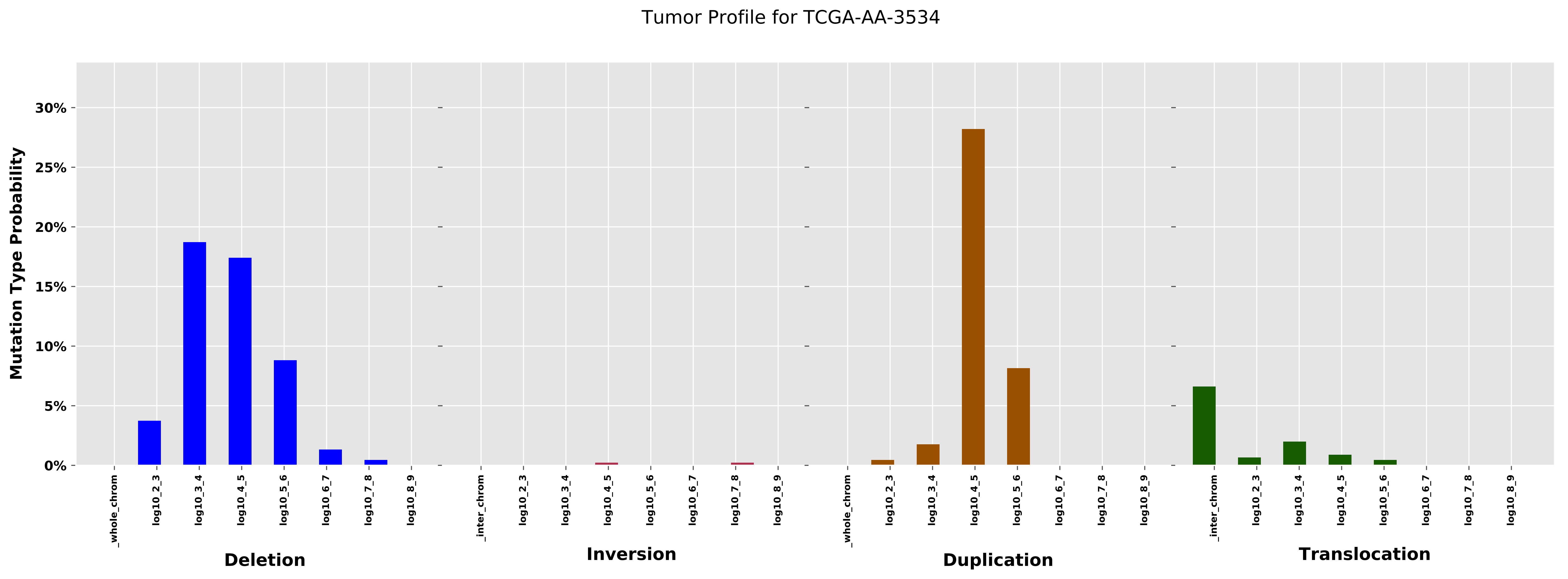
Tumor Profile for TCGA-AA-3534
Mutation Type Probability
0%
5%
10%
15%
20%
25%
30%
_whole_chrom
log10_2_3
log10_3_4
log10_4_5
log10_5_6
log10_6_7
log10_7_8
log10_8_9
_inter_chrom
Deletion
Inversion
Duplication
Translocation

Cancer processes Weights for TCGA-A2-A25B

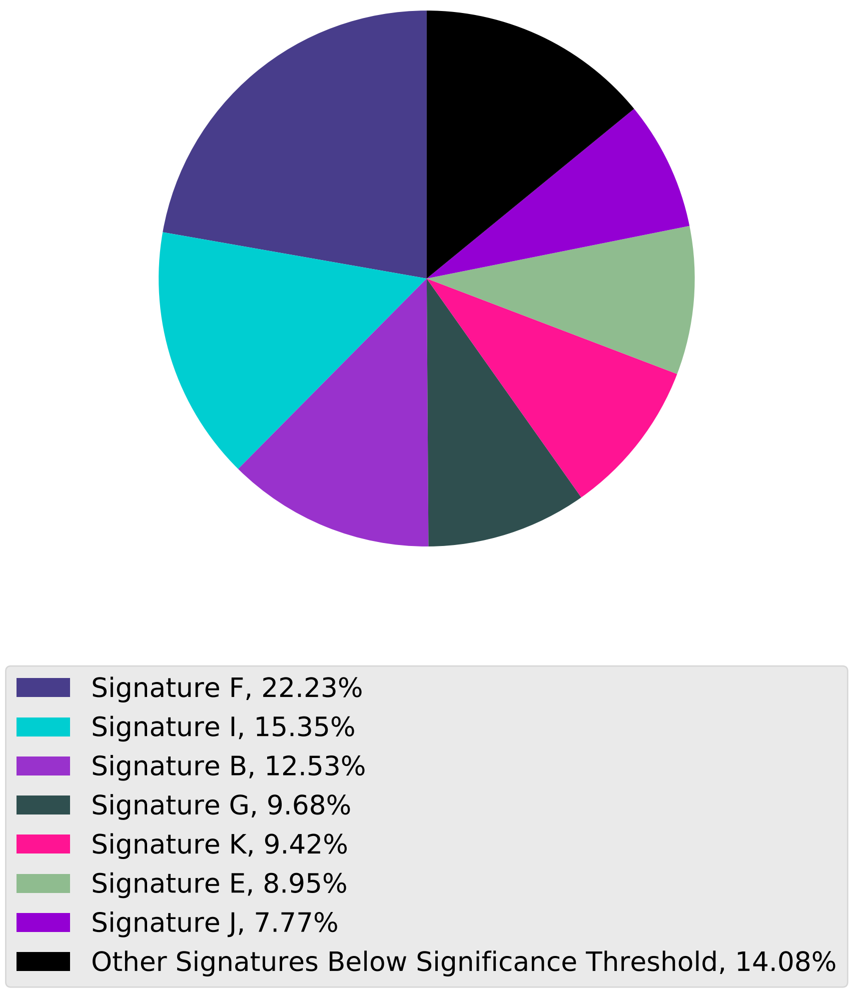

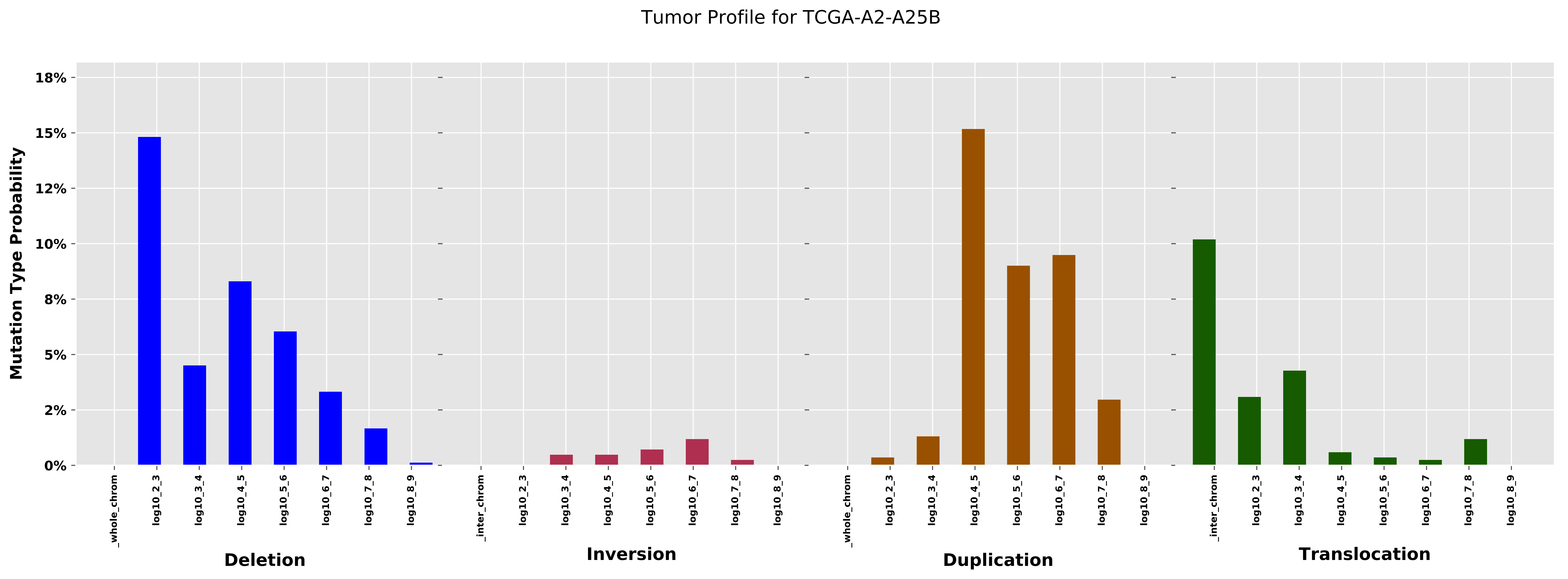
Tumor Profile for TCGA-A2-A25B
Mutation Type Probability
18%
15%
12%
10%
8%
5%
2%
0%
_whole_chrom
log10_2_3
log10_3_4
log10_4_5
log10_5_6
log10_6_7
log10_7_8
log10_8_9
_inter_chrom
Deletion
Inversion
Duplication
Translocation

Cancer processes Weights for TCGA-AG-4008

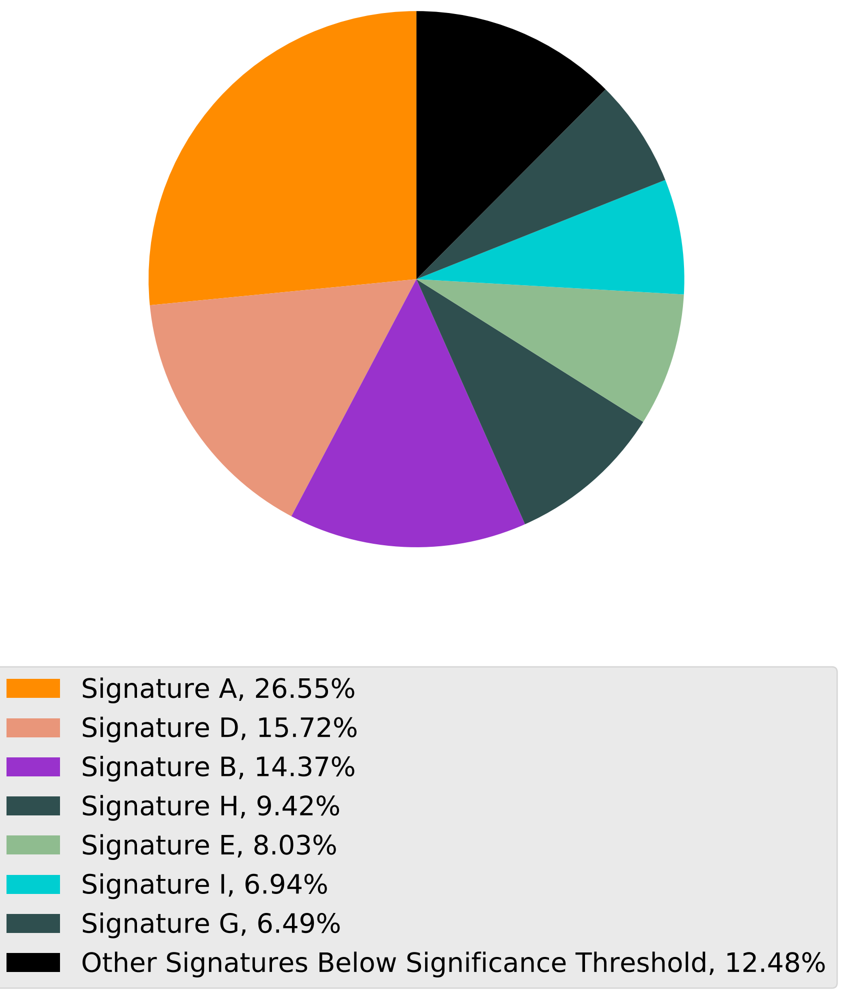

Tumor Profile for TCGA-AG-4008

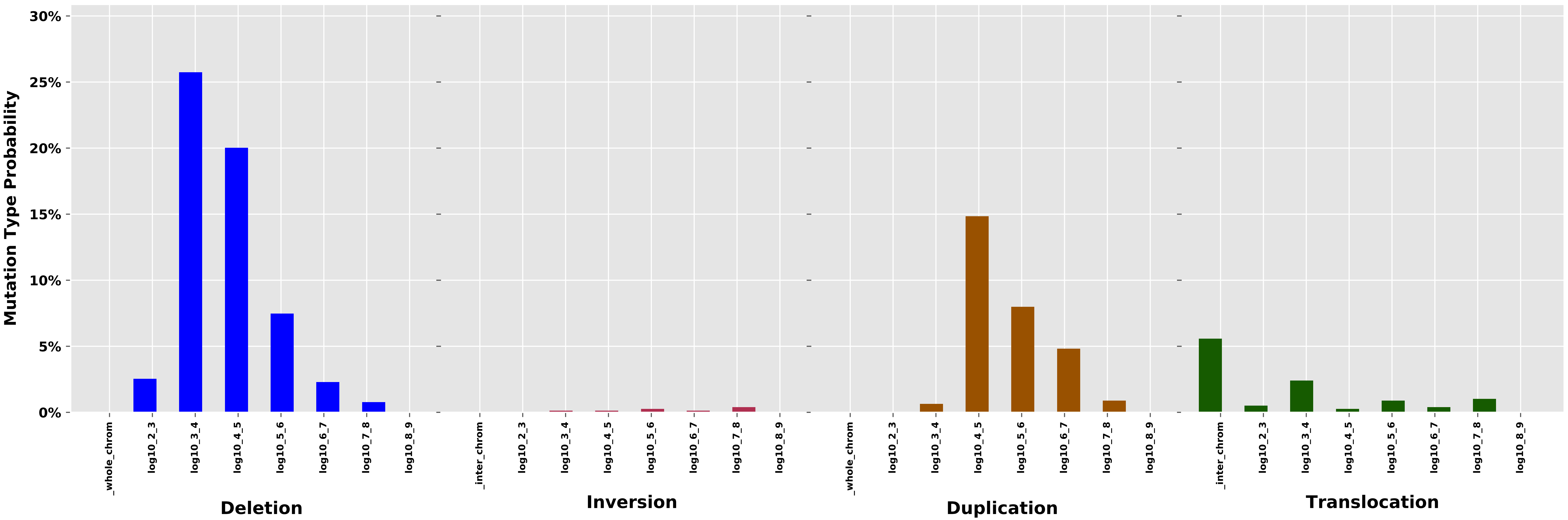

Cancer processes Weights for TCGA-A7-A26G

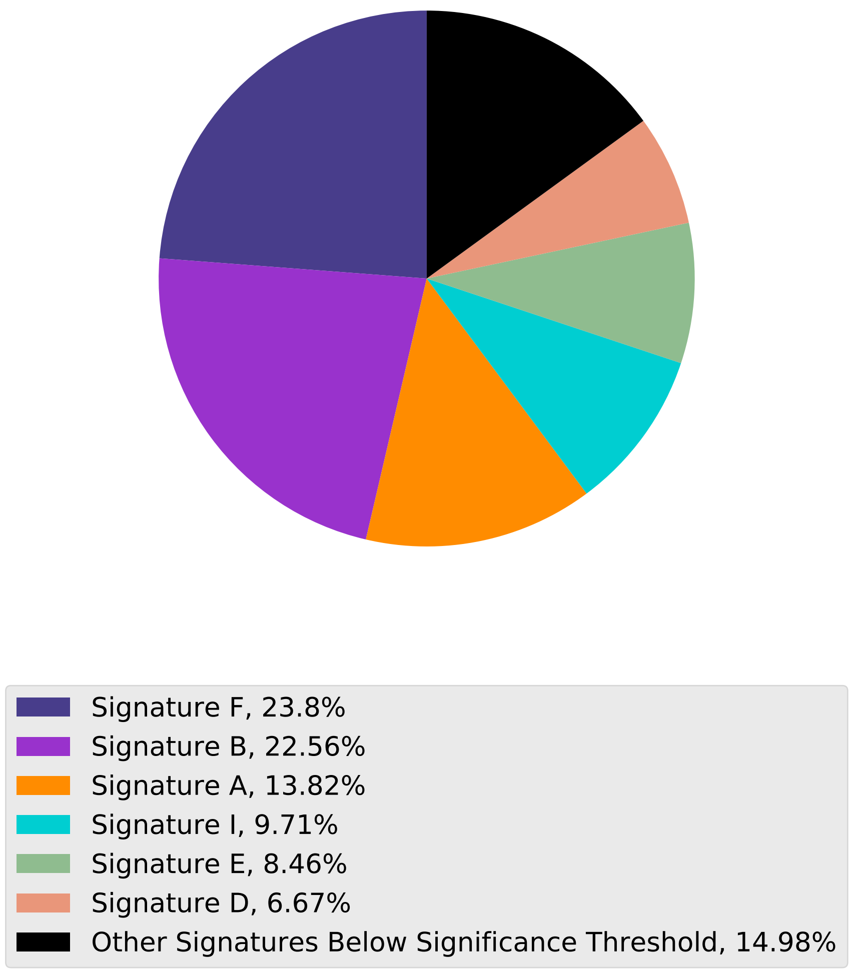

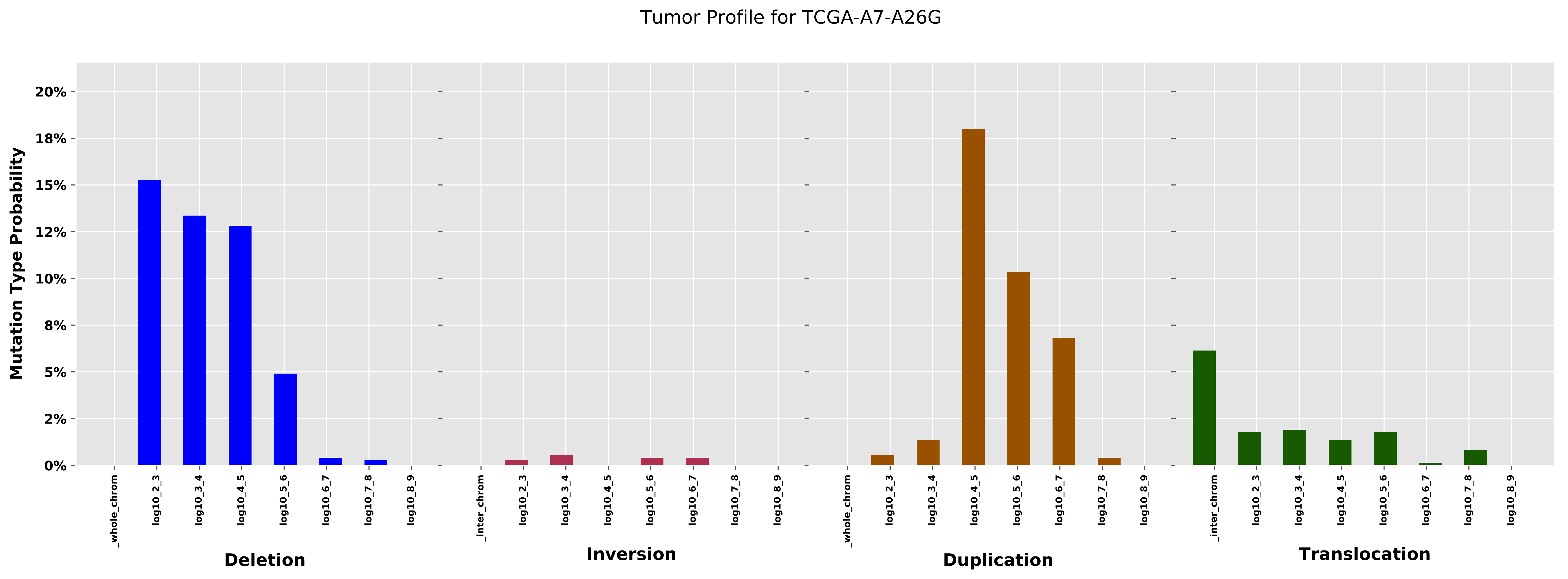

Tumor Profile for TCGA-A7-A26G
Mutation Type Probability
20%
18%
15%
12%
10%
8%
5%
2%
0%
_whole_chrom
log10_2_3
log10_3_4
log10_4_5
log10_5_6
log10_6_7
log10_7_8
log10_8_9
_inter_chrom
Deletion
Inversion
Duplication
Translocation

Cancer processes Weights for TCGA-AR-A24Z

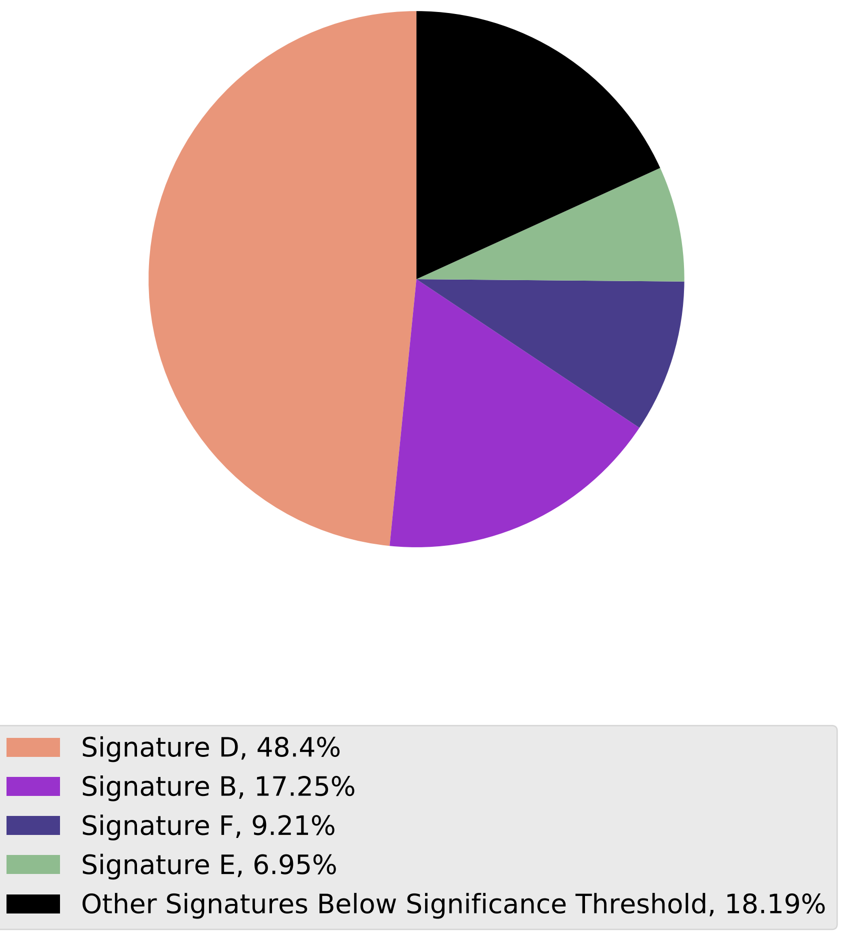

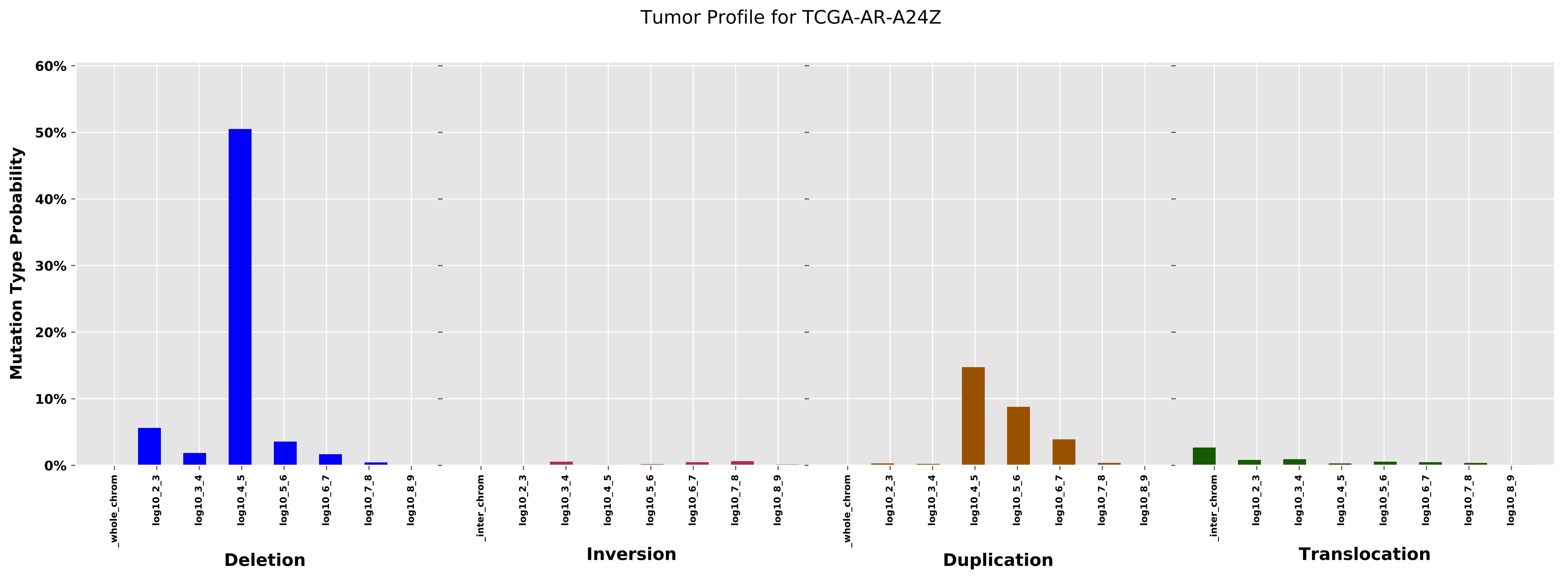

Tumor Profile for TCGA-AR-A24Z
Mutation Type Probability
60%
50%
40%
30%
20%
10%
0%
_whole_chrom
log10_2_3
log10_3_4
log10_4_5
log10_5_6
log10_6_7
log10_7_8
log10_8_9
Deletion
_inter_chrom
log10_2_3
log10_3_4
log10_4_5
log10_5_6
log10_6_7
log10_7_8
log10_8_9
Inversion
_whole_chrom
log10_2_3
log10_3_4
log10_4_5
log10_5_6
log10_6_7
log10_7_8
log10_8_9
Duplication
_inter_chrom
log10_2_3
log10_3_4
log10_4_5
log10_5_6
log10_6_7
log10_7_8
log10_8_9
Translocation

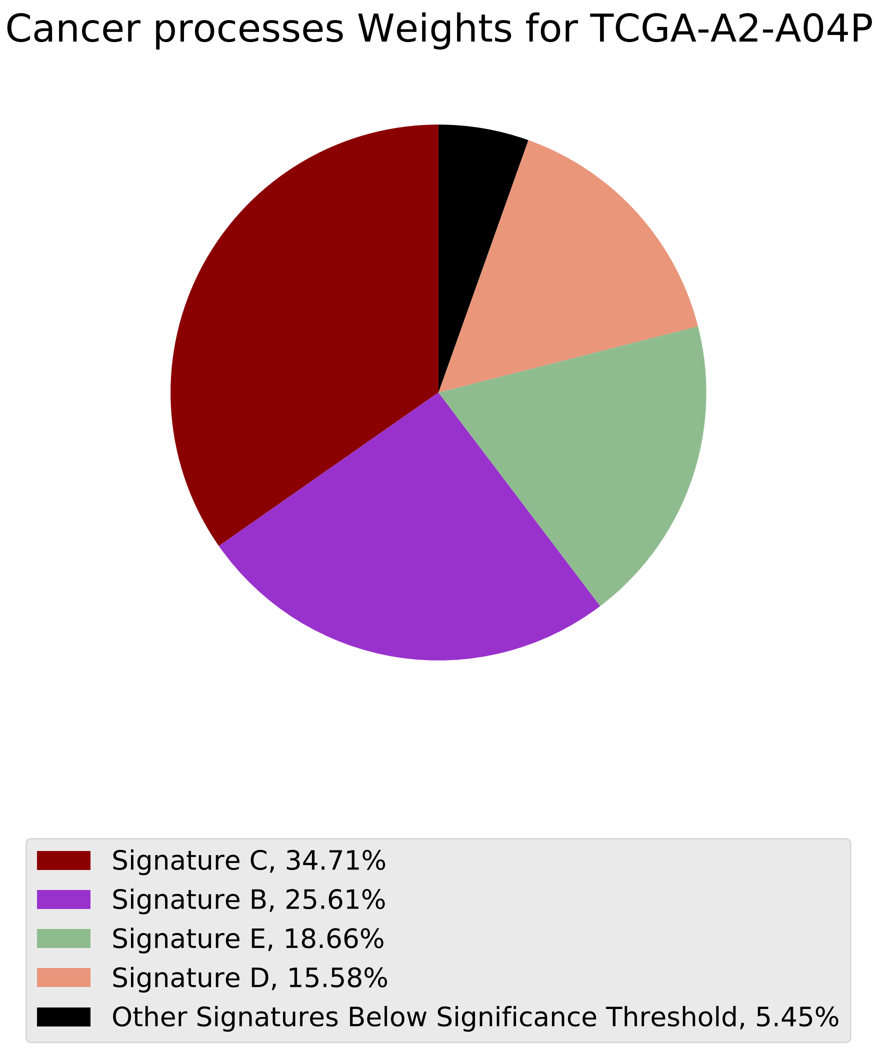
Cancer processes Weights for TCGA-A2-A04P
Signature C, 34.71%
Signature B, 25.61%
Signature E, 18.66%
Signature D, 15.58%
Other Signatures Below Significance Threshold, 5.45%

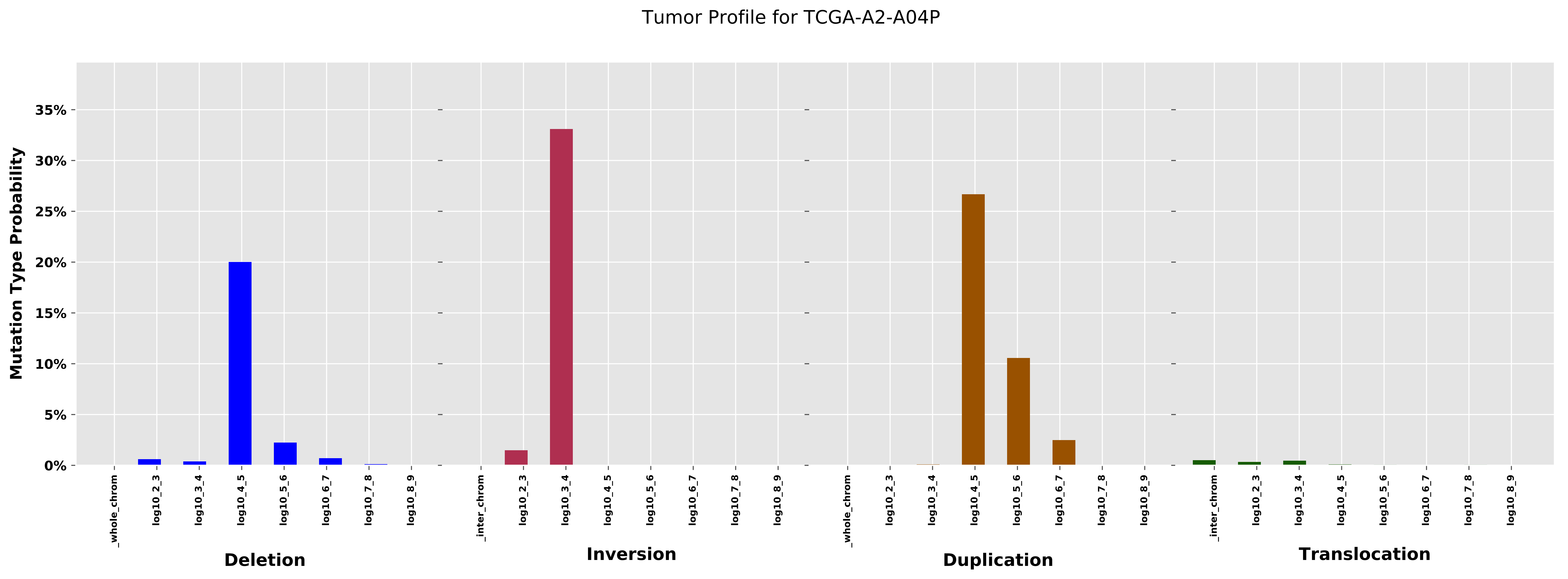

Tumor Profile for TCGA-A2-A04P
Mutation Type Probability
35%
30%
25%
20%
15%
10%
5%
0%
_whole_chrom
log10_2_3
log10_3_4
log10_4_5
log10_5_6
log10_6_7
log10_7_8
log10_8_9
Deletion
_inter_chrom
log10_2_3
log10_3_4
log10_4_5
log10_5_6
log10_6_7
log10_7_8
log10_8_9
Inversion
_whole_chrom
log10_2_3
log10_3_4
log10_4_5
log10_5_6
log10_6_7
log10_7_8
log10_8_9
Duplication
_inter_chrom
log10_2_3
log10_3_4
log10_4_5
log10_5_6
log10_6_7
log10_7_8
log10_8_9
Translocation

Cancer processes Weights for TCGA-CA-6718

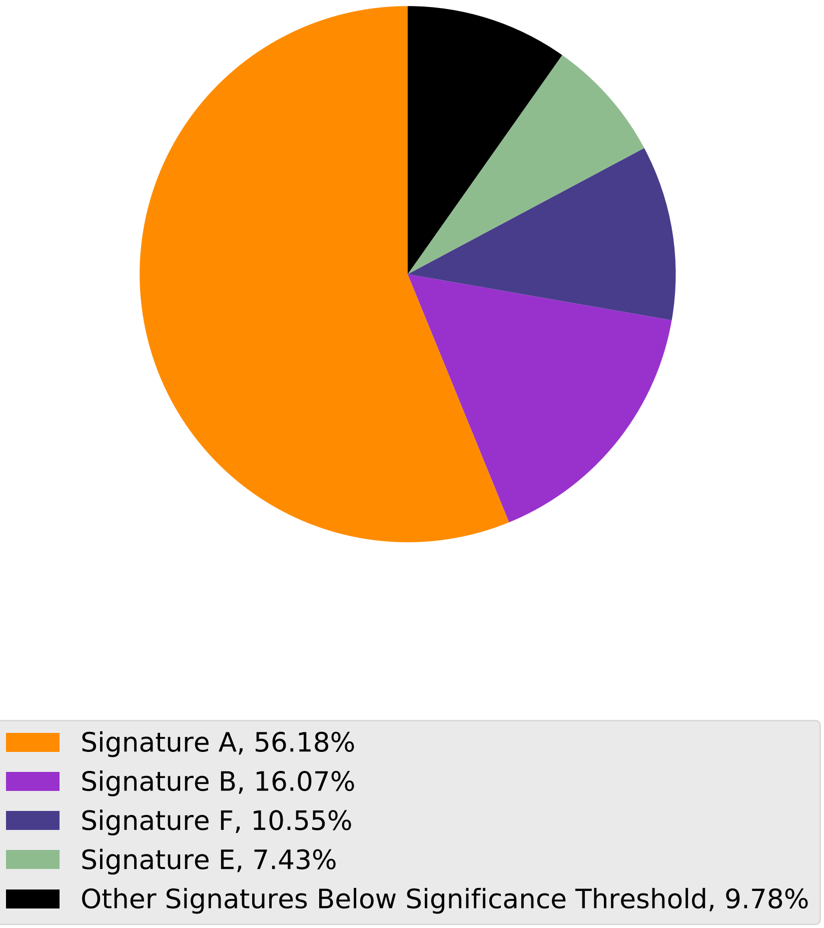

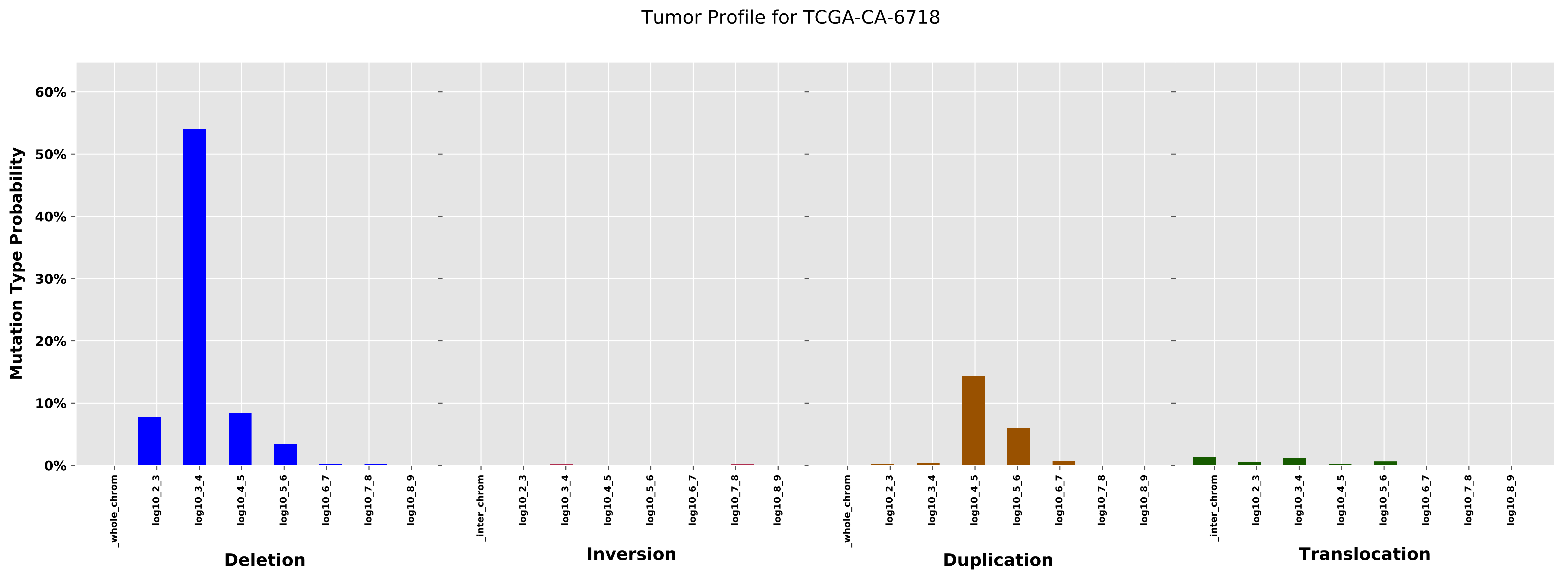

Tumor Profile for TCGA-CA-6718
Mutation Type Probability
60%
50%
40%
30%
20%
10%
0%
_whole_chrom
log10_2_3
log10_3_4
log10_4_5
log10_5_6
log10_6_7
log10_7_8
log10_8_9
_inter_chrom
Deletion
Inversion
Duplication
Translocation

Cancer processes Weights for TCGA-BH-A0AV

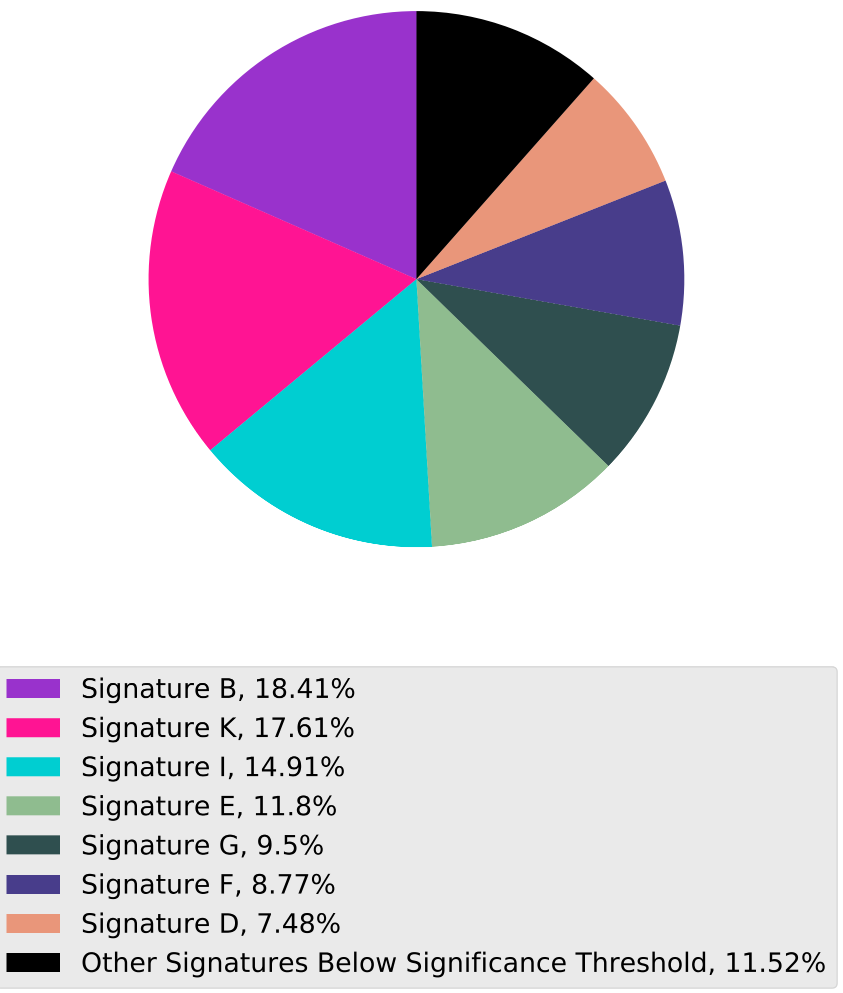

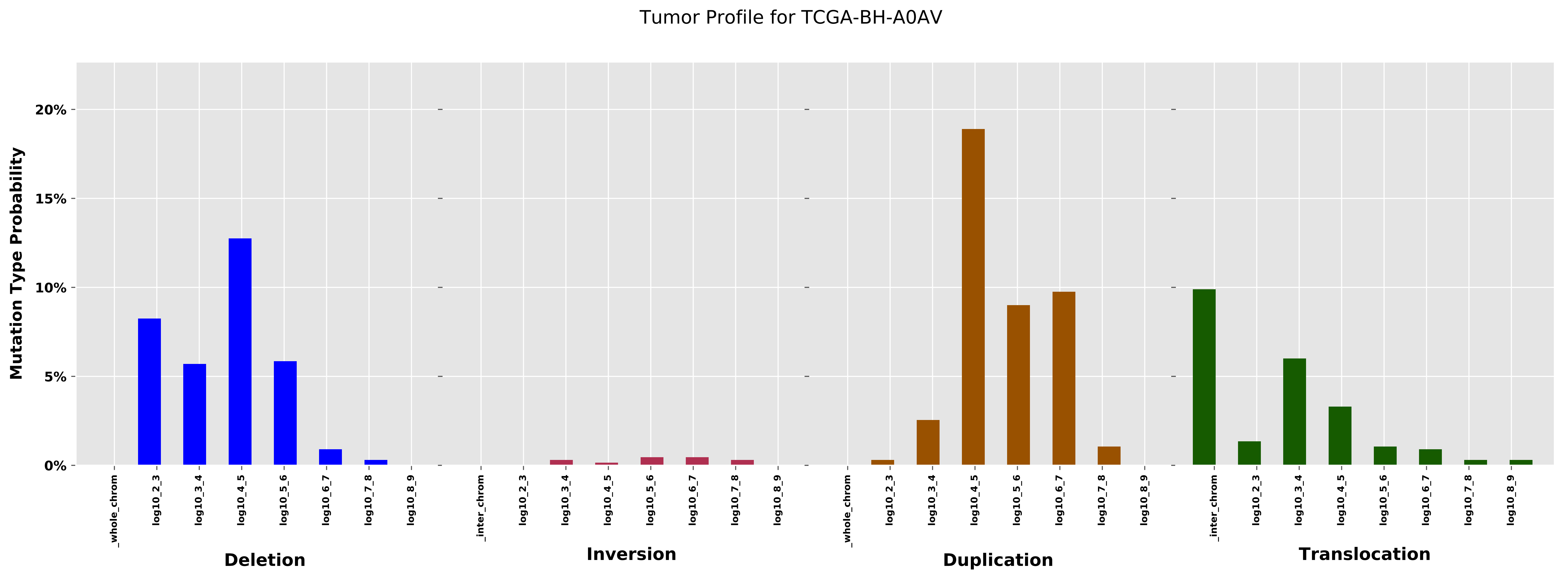

Tumor Profile for TCGA-BH-A0AV
Mutation Type Probability
20%
15%
10%
5%
0%
_whole_chrom
log10_2_3
log10_3_4
log10_4_5
log10_5_6
log10_6_7
log10_7_8
log10_8_9
_inter_chrom
Deletion
Inversion
Duplication
Translocation

Cancer processes Weights for TCGA-A2-A0CM

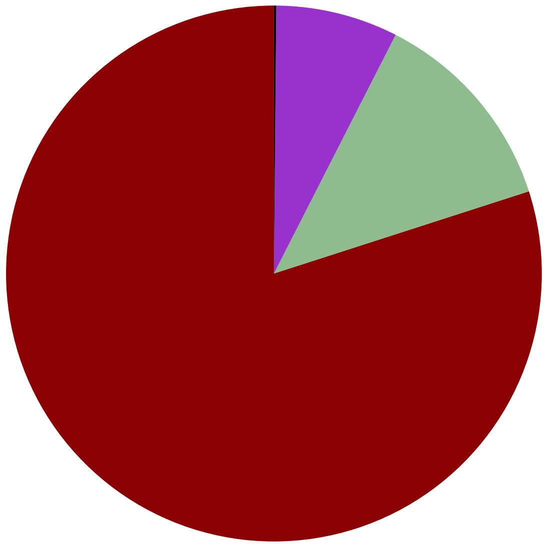

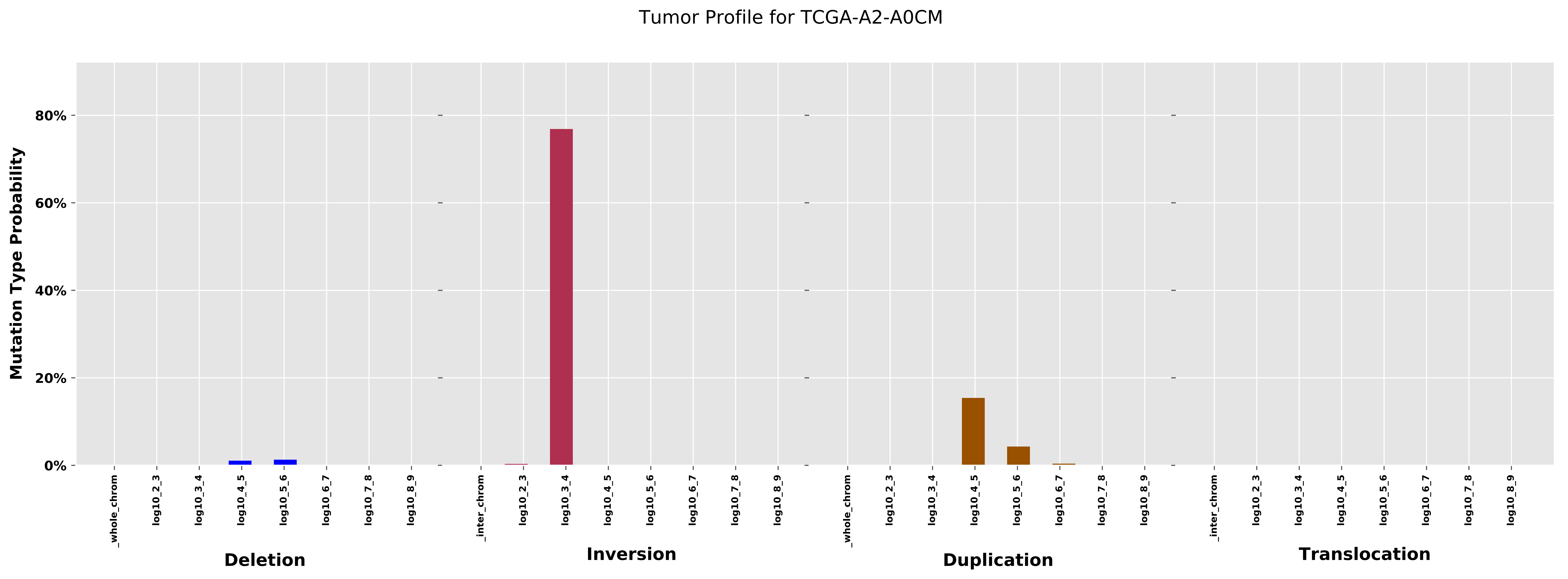

Tumor Profile for TCGA-A2-A0CM
Mutation Type Probability
80%
60%
40%
20%
0%
_whole_chrom
log10_2_3
log10_3_4
log10_4_5
log10_5_6
log10_6_7
log10_7_8
log10_8_9
_inter_chrom
Deletion
Inversion
Duplication
Translocation

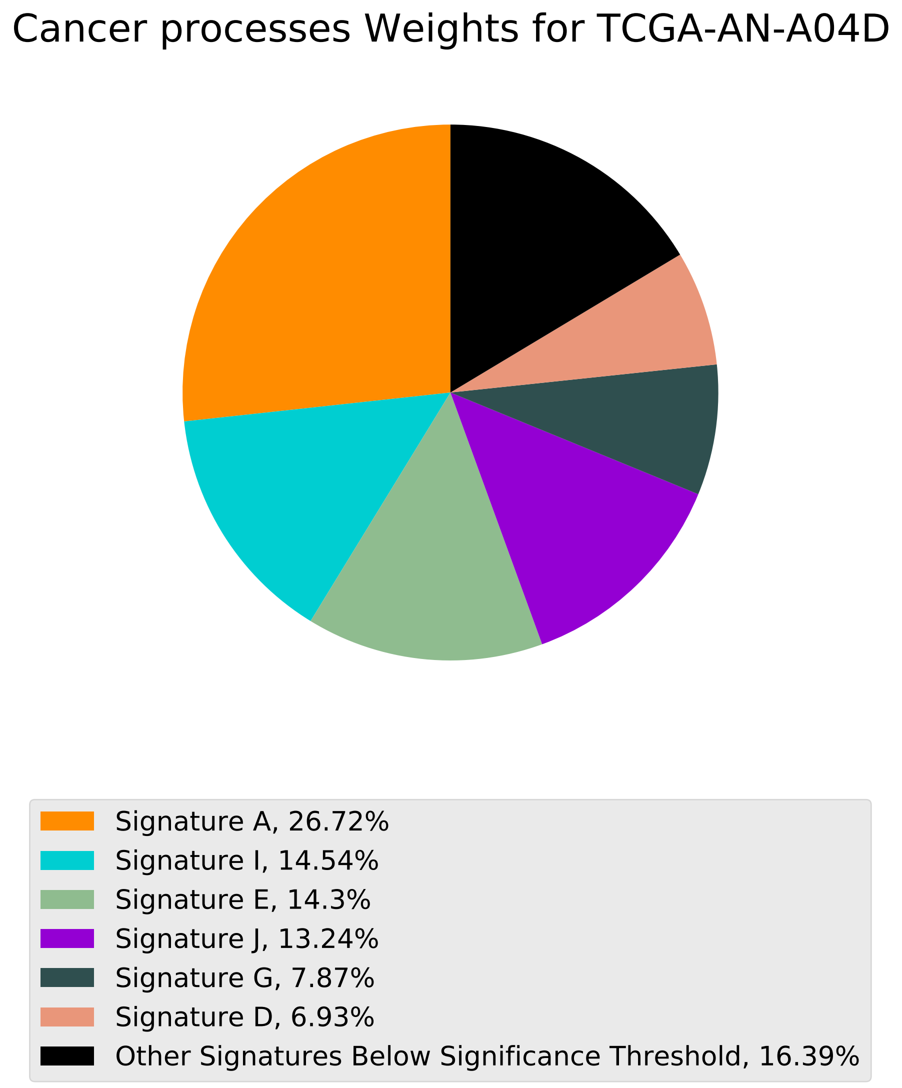
Cancer processes Weights for TCGA-AN-A04D
Signature A, 26.72%
Signature I, 14.54%
Signature E, 14.3%
Signature J, 13.24%
Signature G, 7.87%
Signature D, 6.93%
Other Signatures Below Significance Threshold, 16.39%

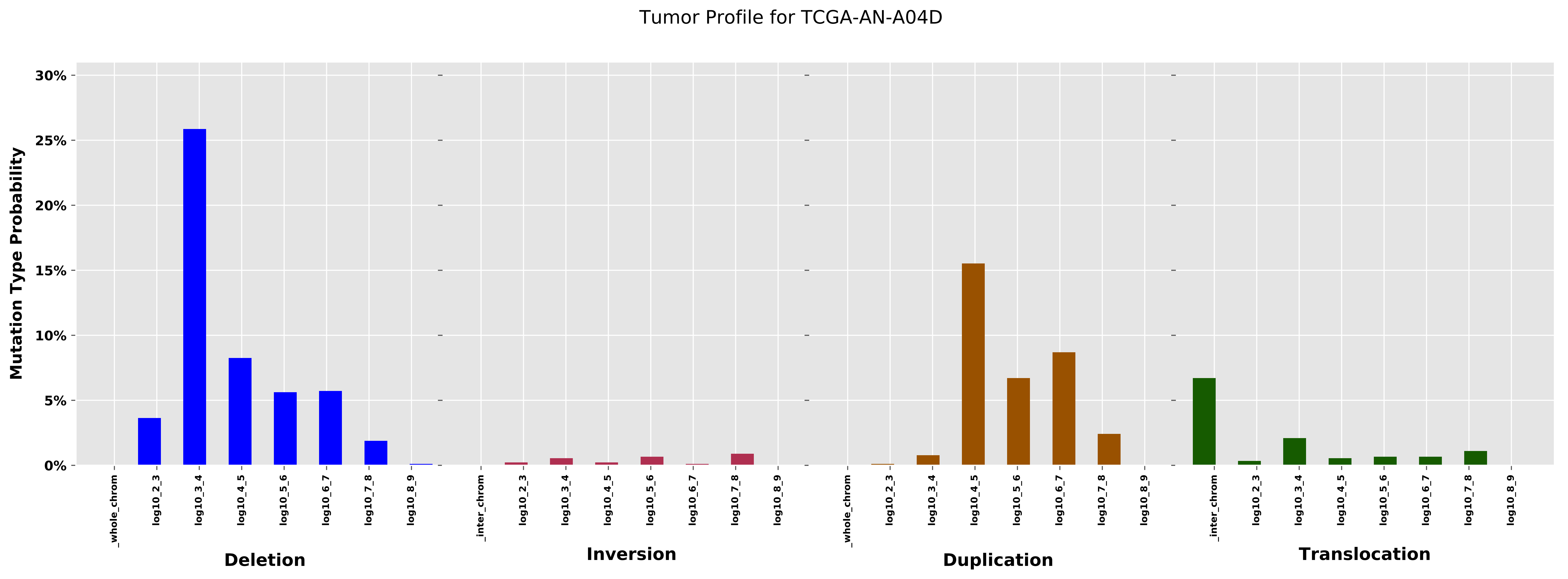

Tumor Profile for TCGA-AN-A04D
Mutation Type Probability
30%
25%
20%
15%
10%
5%
0%
_whole_chrom
log10_2_3
log10_3_4
log10_4_5
log10_5_6
log10_6_7
log10_7_8
log10_8_9
Deletion
_inter_chrom
Inversion
Duplication
Translocation

Cancer processes Weights for TCGA-A8-A092

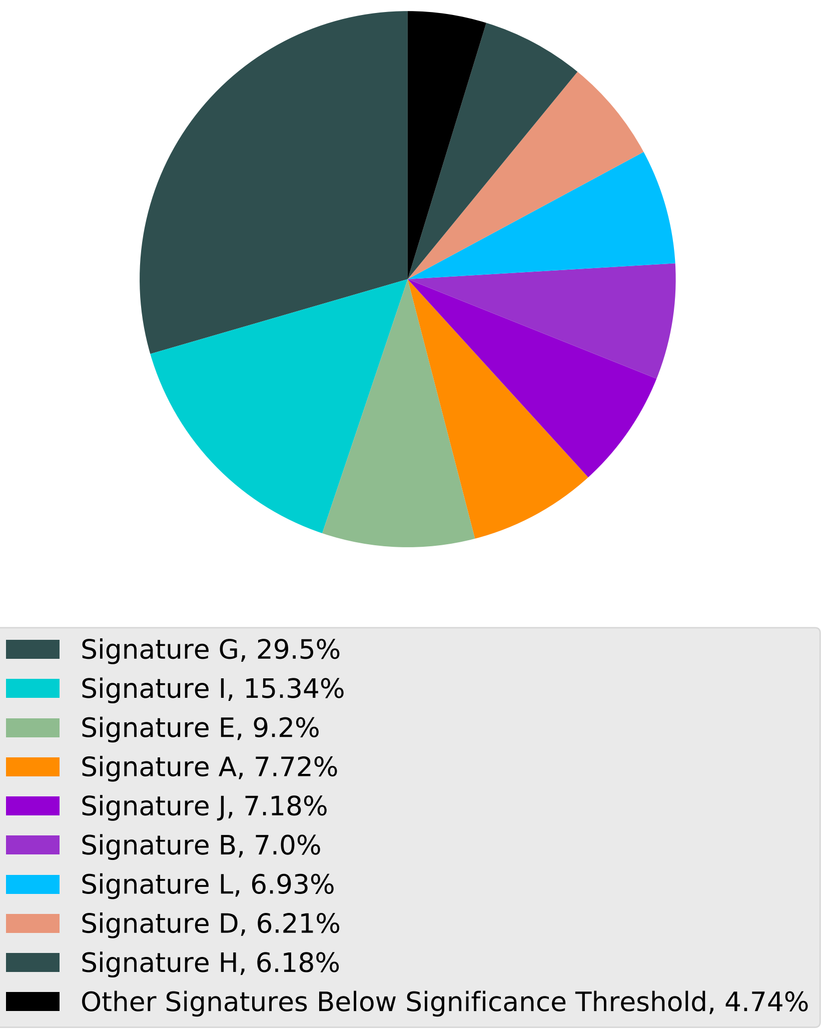

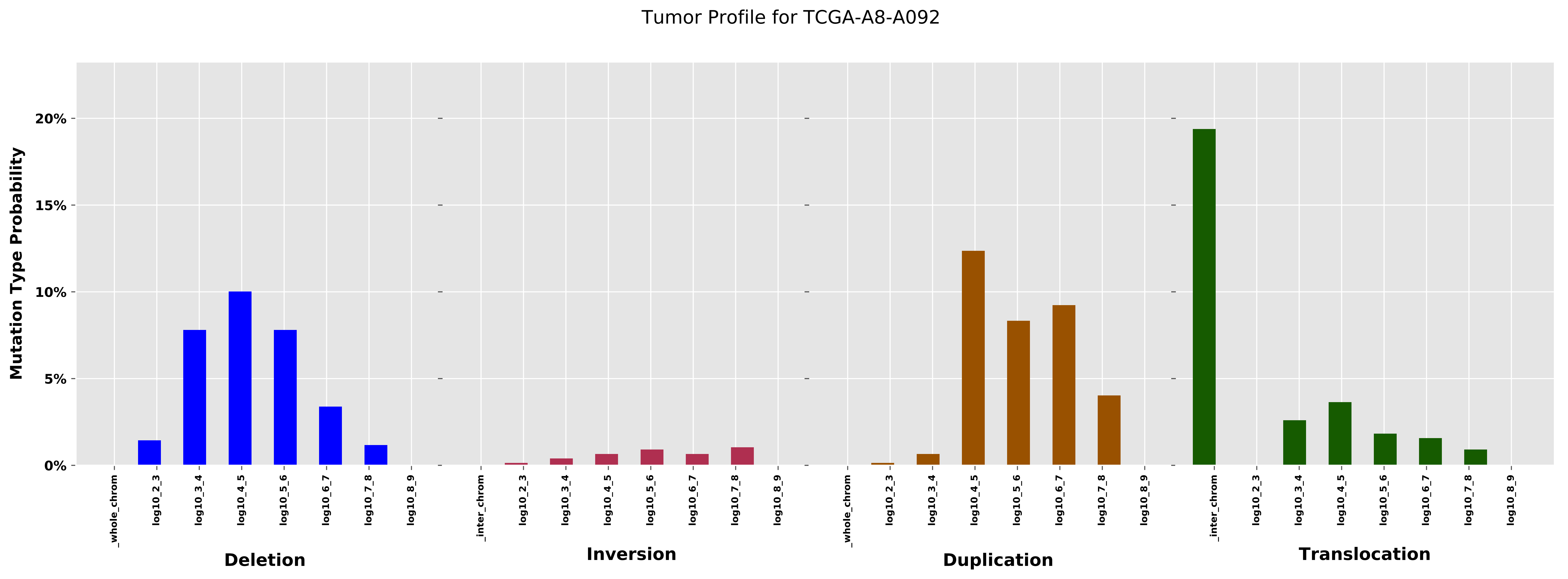

Tumor Profile for TCGA-A8-A092
Mutation Type Probability
0%
5%
10%
15%
20%
_whole_chrom
log10_2_3
log10_3_4
log10_4_5
log10_5_6
log10_6_7
log10_7_8
log10_8_9
_inter_chrom
Deletion
Inversion
Duplication
Translocation

Cancer processes Weights for TCGA-A2-A04X

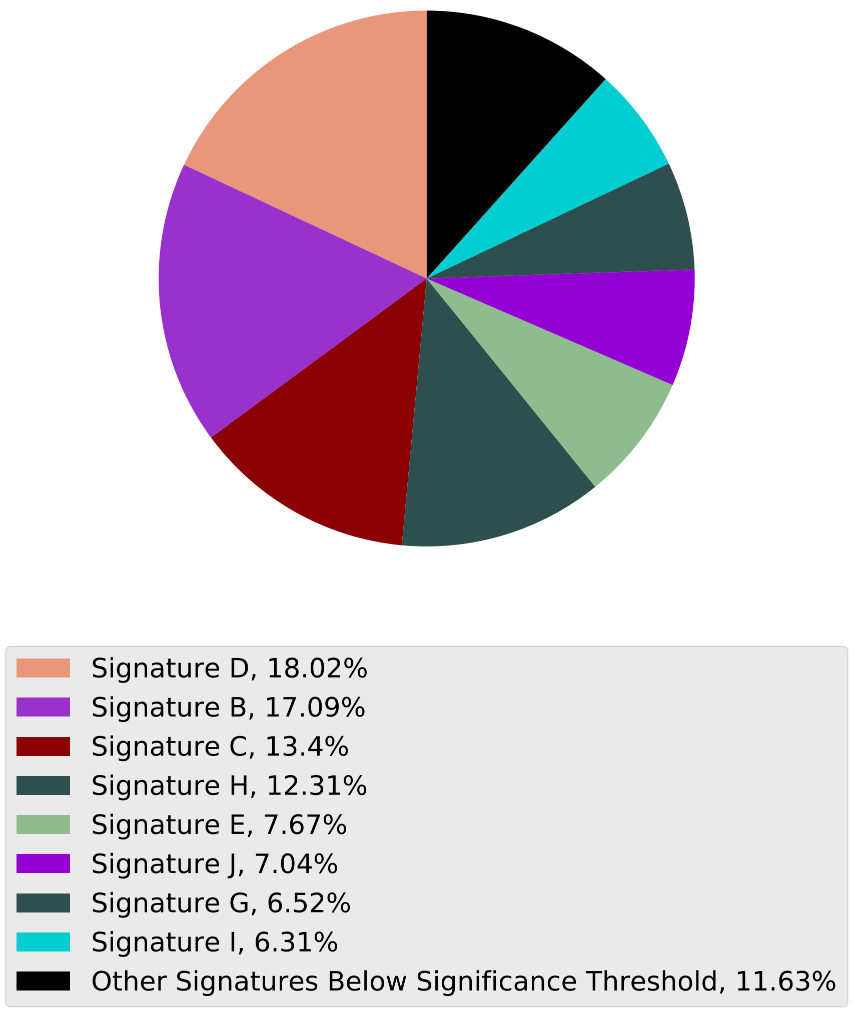

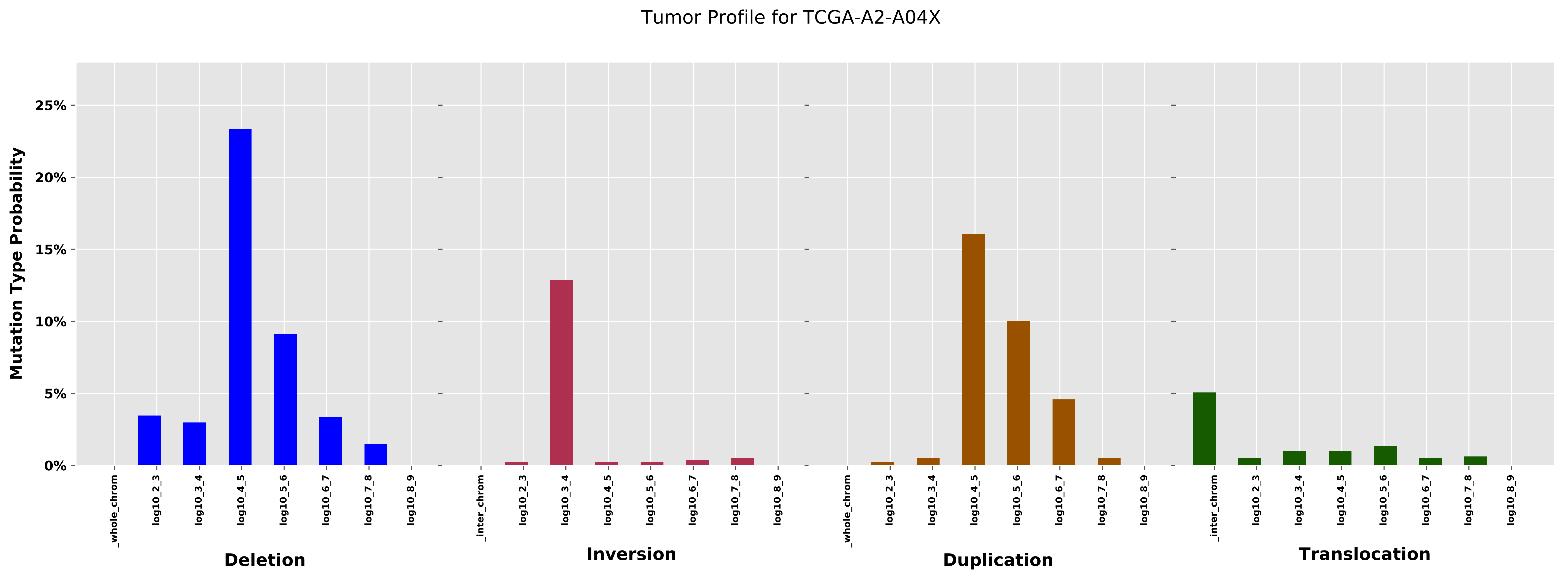
Tumor Profile for TCGA-A2-A04X
Mutation Type Probability
25%
20%
15%
10%
5%
0%
_whole_chrom
log10_2_3
log10_3_4
log10_4_5
log10_5_6
log10_6_7
log10_7_8
log10_8_9
Deletion
_inter_chrom
log10_2_3
log10_3_4
log10_4_5
log10_5_6
log10_6_7
log10_7_8
log10_8_9
Inversion
_whole_chrom
log10_2_3
log10_3_4
log10_4_5
log10_5_6
log10_6_7
log10_7_8
log10_8_9
Duplication
_inter_chrom
log10_2_3
log10_3_4
log10_4_5
log10_5_6
log10_6_7
log10_7_8
log10_8_9
Translocation

Cancer processes Weights for TCGA-A2-A0D2

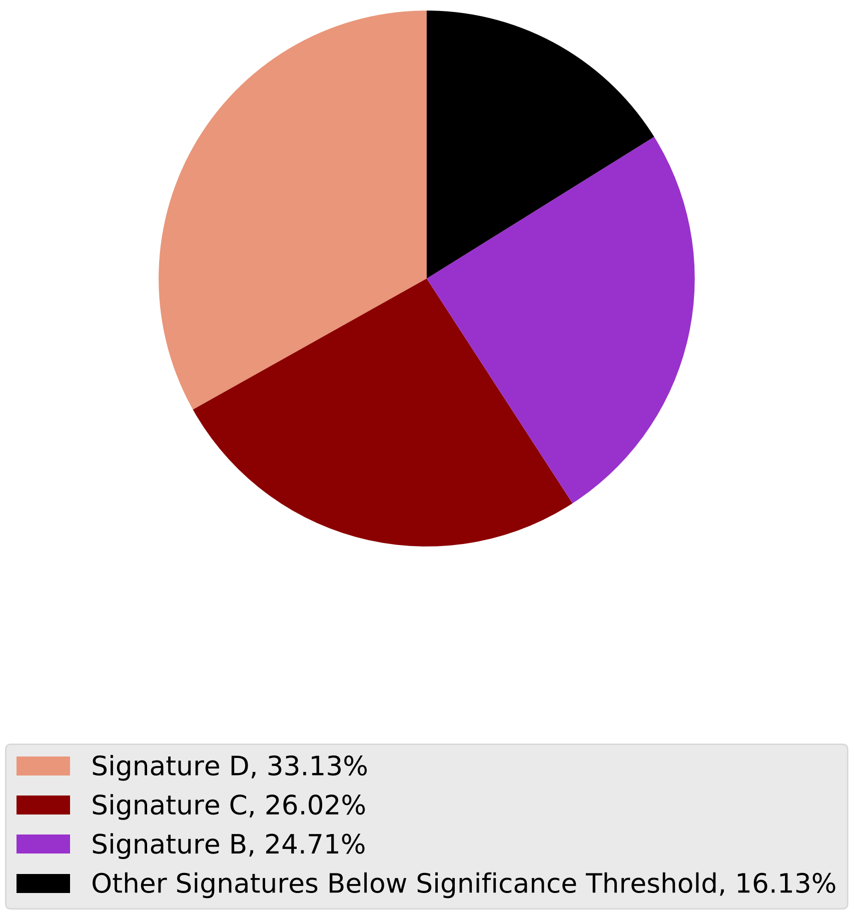

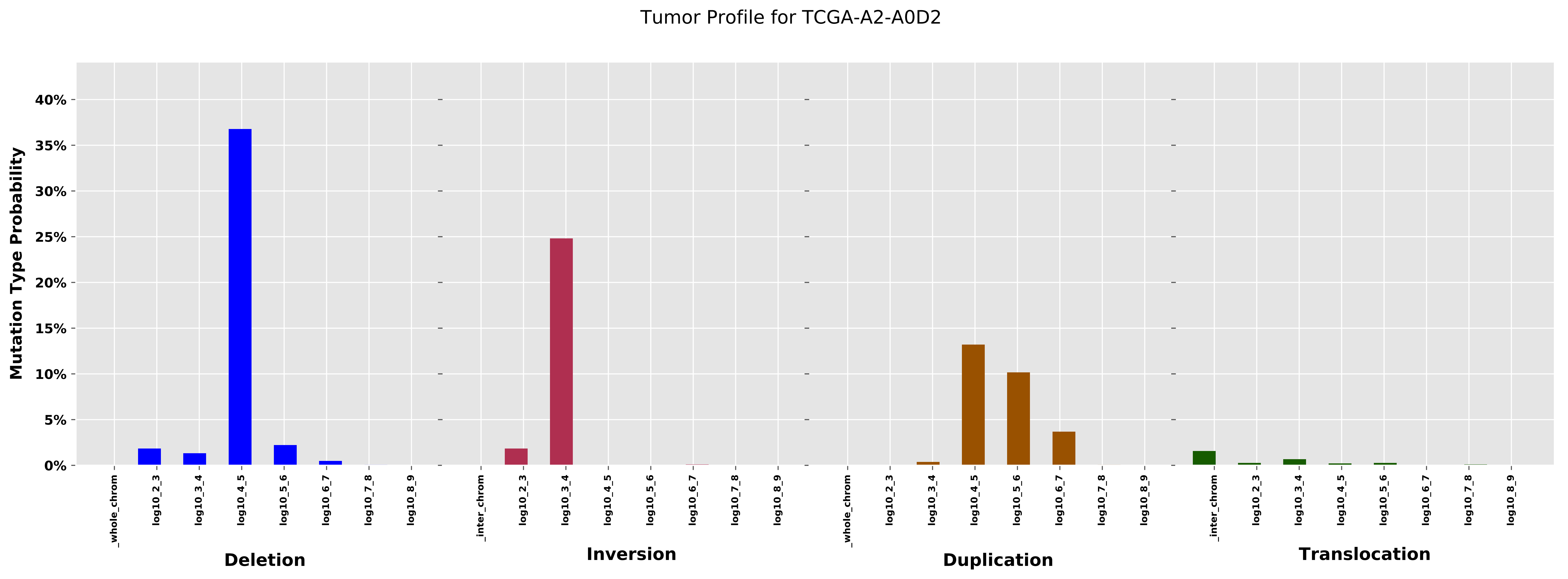

Tumor Profile for TCGA-A2-A0D2
Mutation Type Probability
0%
5%
10%
15%
20%
25%
30%
35%
40%
_whole_chrom
log10_2_3
log10_3_4
log10_4_5
log10_5_6
log10_6_7
log10_7_8
log10_8_9
_inter_chrom
Deletion
Inversion
Duplication
Translocation

Cancer processes Weights for TCGA-B6-A0RU

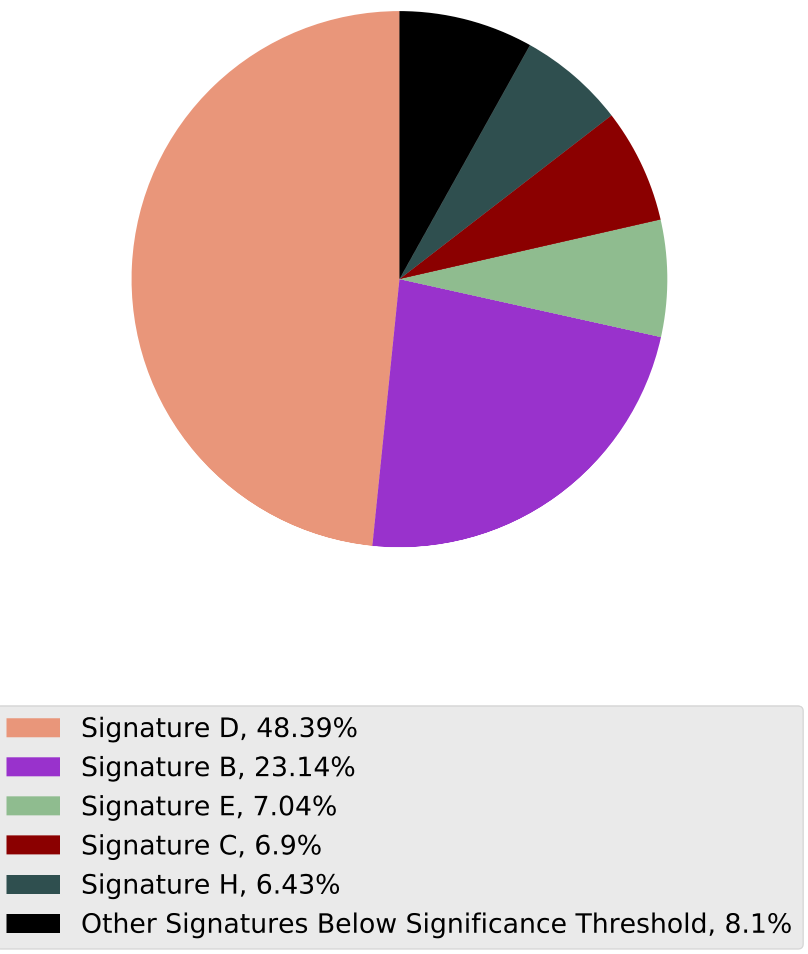

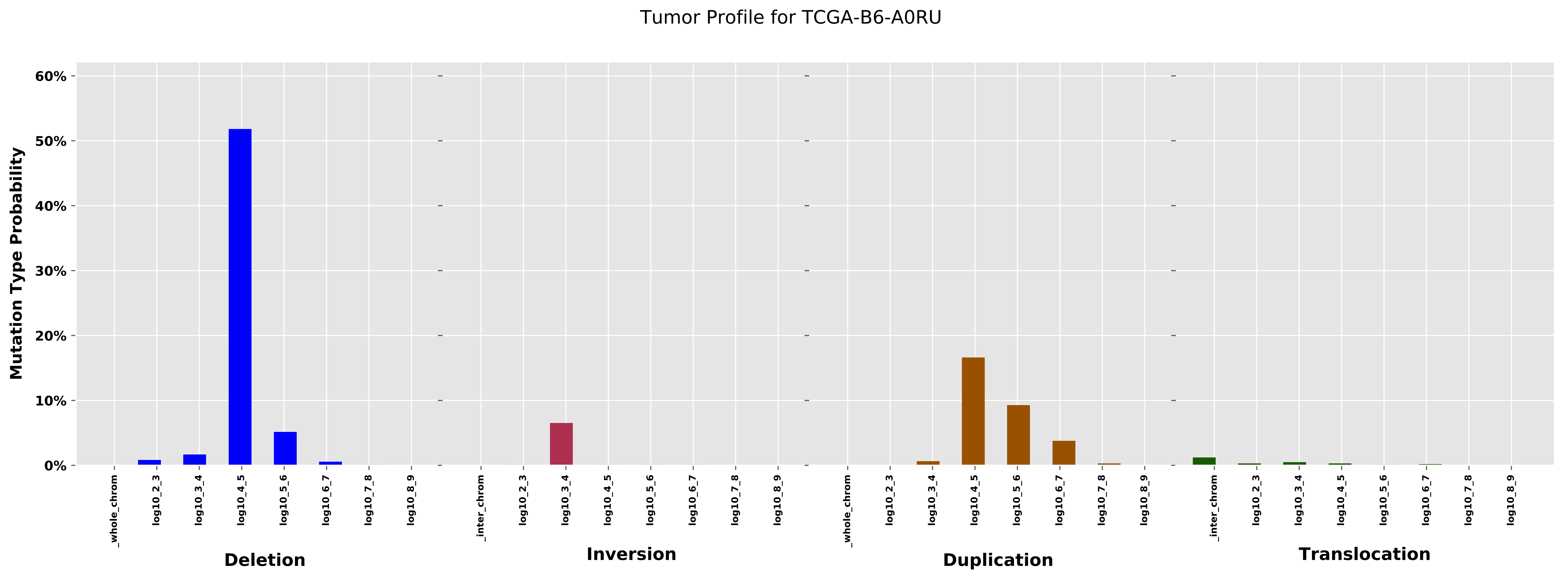

Tumor Profile for TCGA-B6-A0RU
Mutation Type Probability
60%
50%
40%
30%
20%
10%
0%
_whole_chrom
log10_2_3
log10_3_4
log10_4_5
log10_5_6
log10_6_7
log10_7_8
log10_8_9
_inter_chrom
log10_2_3
log10_3_4
log10_4_5
log10_5_6
log10_6_7
log10_7_8
log10_8_9
_whole_chrom
log10_2_3
log10_3_4
log10_4_5
log10_5_6
log10_6_7
log10_7_8
log10_8_9
_inter_chrom
log10_2_3
log10_3_4
log10_4_5
log10_5_6
log10_6_7
log10_7_8
log10_8_9
Deletion
Inversion
Duplication
Translocation
