# Supplementary material for: pyCancerSig: subclassifying human cancer with comprehensive single nucleotide, structural and microsatellite mutational signature deconstruction from whole genome sequencing
Source: BMC Bioinformatics. 2020 Apr 3;21:128. doi: 10.1186/s12859-020-3451-8 (PMC7118897; doi:10.1186/s12859-020-3451-8)
Supplement: Supplementary file 7 — Additional file 7. [file 12859_2020_3451_MOESM7_ESM.pdf]

Figure 1: Combined mutational profiles of samples with highest SVB versus samples with lowest SVB

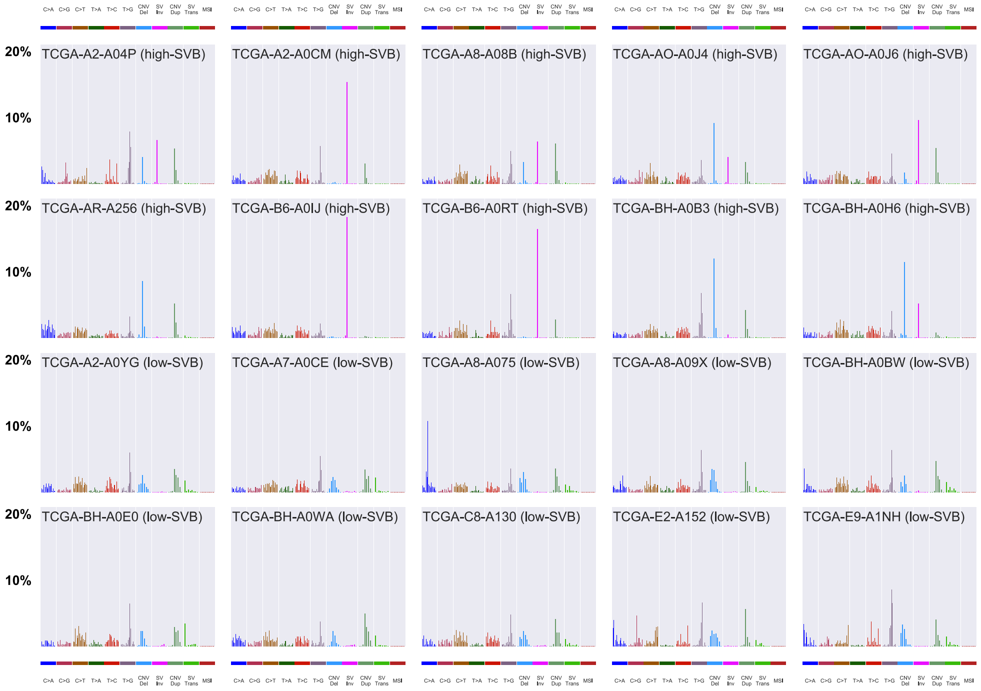

The samples in the first two rows have the highest SVB and the samples in the last two rows have the lowest SVB

Figure 2: SV-only mutational profiles of samples with highest SVB versus samples with lowest SVB

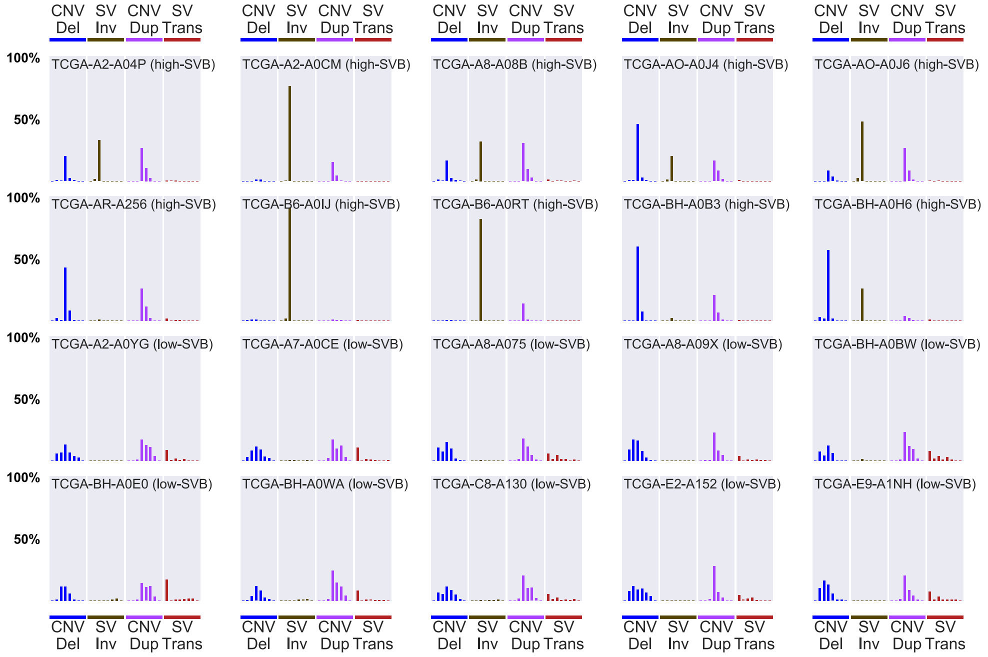

The samples in the first two rows have the highest SVB and the samples in the last two rows have the lowest SVB
